# A C–H activation-based enantioselective synthesis of lower carbo[*n*]helicenes

**DOI:** 10.1038/s41557-023-01174-5

**Published:** 2023-04-06

**Authors:** Shu-Min Guo, Soohee Huh, Max Coehlo, Li Shen, Grégory Pieters, Olivier Baudoin

**Affiliations:** 1 Department of Chemistry, University of Basel, Basel, Switzerland; 2 Département Médicaments et Technologies pour la Santé (DMTS), SCBM, Université Paris-Saclay, CEA, INRAE, Gif-sur-Yvette, France

**Keywords:** Asymmetric catalysis, Synthetic chemistry methodology, Single-molecule fluorescence

## Abstract

The three-dimensional structure of carbohelicenes has fascinated generations of molecular chemists and has been exploited in a wide range of applications. Their strong circularly polarized luminescence has attracted considerable attention in recent years due to promising applications in new optical materials. Although the enantioselective synthesis of fused carbo- and heterohelicenes has been achieved, a direct catalytic enantioselective method allowing the synthesis of lower, non-fused carbo[*n*]helicenes (*n* = 4–6) is still lacking. We report here that Pd-catalysed enantioselective C–H arylation in the presence of a unique bifunctional phosphine-carboxylate ligand provides a simple and general access to these lower carbo[*n*]helicenes. Computational mechanistic studies indicate that both the C–H activation and reductive elimination steps contribute to the overall enantioselectivity. The observed enantio-induction seems to arise from a combination of non-covalent interactions and steric repulsion between the substrate and ligand during the two key reductive elimination steps. The photophysical and chiroptical properties of the synthesized scalemic [*n*]helicenes have been systematically studied.

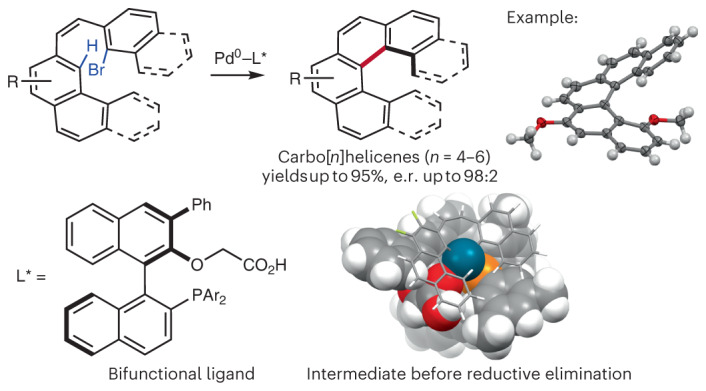

## Abstract (web summary)

A simple and general enantioselective method for the synthesis of non-fused lower carbo[*n*]helicenes (*n* = 4–6) is reported. The helicene scaffold is constructed with high enantioselectivity by Pd^0^-catalysed C–H arylation with aryl bromides. A bifunctional ligand provides a precise chiral environment that allows fine control of the enantioselectivity.

## Main

**Fig. 1:**
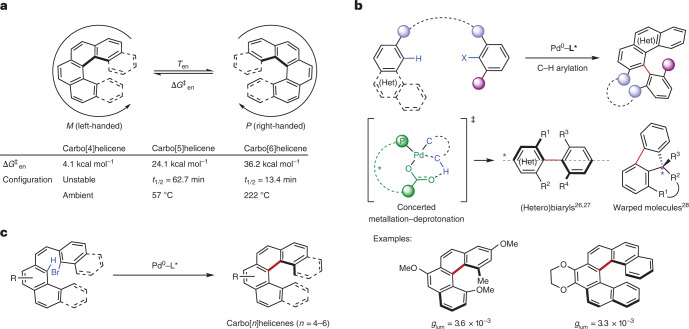
Design of the enantioselective synthesis of carbo[*n*]helicenes by Pd^0^-catalysed C–H arylation. **a**, Enantiomerization barriers for carbo[4]-, carbo[5]- and carbo[6]helicenes. *T*_en_, enantiomerization temperature. **b**, Pd^0^-catalysed enantioselective C–H arylation for the synthesis of axially chiral biaryl compounds and warped molecules showing the underlying concerted metallation–deprotonation process. L*, chiral ligand; *, stereogenic element. **c**, This report describes the synthesis of CPL-active lower carbo[*n*]helicenes by Pd^0^-catalysed C–H arylation.

Carbohelicenes are polycyclic aromatic compounds composed of consecutive *ortho*-fused benzene rings that adopt a non-planar, helical topology^1^. The large steric hindrance between the two terminal rings may result in a pair of configurationally stable *M* and *P* enantiomers and the presence of a stereogenic helix axis (Fig. 1a). This particular topology has been widely exploited^2^ in diverse fields ranging from asymmetric catalysis^3^ to optoelectronic devices^4^, stereochemical molecular recognition^5^ and photodynamic therapy^6^. In particular, the highly distorted, conjugated π-system of carbohelicenes and their heteroatomic analogues^7^, termed heterohelicenes, provides them with strong chiroptical properties, including circularly polarized luminescence (CPL)^8^, which have attracted considerable interest in recent years due to promising applications in new optical materials. The unique topology and properties of helicenes have fascinated synthetic chemists for decades^9,10^. Despite major achievements in enantioselective synthesis, the resolution of racemic mixtures is still the dominant method for obtaining optically pure helicenes and studying their chiroptical properties^11^. Enantioselective catalysis, which is ultimately the most desirable method for accessing a given non-racemic helicene, has developed considerably in recent years and important advances have been reported. In particular, both organocatalysis^12,13^ and transition metal catalysis^14–17^, proceeding by two main strategies, namely chirality transfer from non-helical enantioenriched precursors and stereoselective cycloaddition or annulation of achiral precursors, have enabled enantioselective access to certain types of carbo- and heterohelicenes^11^. In particular, 1,12-disubstituted carbo[4]helicenes have been accessed only by enantioselective Au-catalysed intramolecular alkyne hydroarylation (note, in this manuscript the term ‘[4]helicene’ is used as an extension of the IUPAC (International Union of Pure and Applied Chemistry)^18^ definition of [*n*]helicenes with *n* ≥ 5)^15^. In recent years, C–H bond functionalization has emerged as a powerful and step-economical approach to the synthesis of complex functional molecules, including polyaromatic systems of interest for organic materials^19–21^. Recently, a number of catalytic enantioselective C–H activation methods have been developed that have proven particularly efficient and versatile for the control of diverse stereogenic elements, including centres, planes and axes^22,23^. Despite intrinsic reactivity and selectivity issues, such approaches would be particularly appealing for the synthesis of non-racemic helicenes. Recently, You and co-workers reported the enantioselective synthesis of azoniahelicenes by Rh-catalysed C–H annulation of fused isoquinolines with alkynes^24^. In addition, Ackermann and co-workers reported an indirect access to enantioenriched carbohelicenes involving an initial Pd-catalysed atroposelective C–H alkenylation, followed by the conversion of the corresponding axially chiral biaryls to helicenes in three steps^25^. Despite all of these advances, a one-step enantioselective entry into lower, non-fused carbo[*n*]helicenes from achiral precursors is still lacking. Palladium(0)-catalysed C–H arylation could potentially contribute to filling this gap (Fig. 1b)^26^. Indeed, the control of axial chirality in (hetero)biaryls was recently reported by the groups of Cramer and Baudoin by such intramolecular^27^ and intermolecular^28^ reactions. Moreover, we have shown that enantioselective Pd^0^-catalysed C–H arylation allows the construction of warped polyaromatic systems^29^. Some of the obtained products adopted a helical shape reminiscent of helicenes that was induced by the generated stereogenic centre. Key to the success of this method was the design of a bifunctional chiral ligand incorporating both a phosphine and a carboxylate moiety^30^, the former enabling strong binding to the Pd centre and the latter performing the C–H bond cleavage by concerted metallation–deprotonation^31^. This bifunctional catalyst features a highly organized chiral pocket that seems to be well adapted to the enantioselective recognition of polyaromatic substrates through non-covalent interactions^29,30^. These features should therefore make this system also suitable for the enantioselective construction of helicenes. When considering this possibility, we were encouraged by precedents demonstrating the feasibility of racemic helicene synthesis^32,33^, despite limited efficiency and generality. Such limitations can be expected from the high steric repulsion and strain energy in the critical C–H activation and C–C coupling steps of the catalytic cycle. Another foreseeable problem in the development of such an enantioselective C–H arylation method is the low enantiomerization free-energy barriers (Δ*G*^‡^_en_) for lower carbo[4]helicenes (4.1 kcal mol^−1^) and carbo[5]helicenes (24.1 kcal mol^−1^)^34^. This problem can be circumvented through the design of suitable starting materials incorporating substituents on one of the peripheral rings, but potentially at the expense of reactivity.

We report here the development of an efficient asymmetric C–H arylation method that enables the synthesis of all lower carbo[*n*]helicenes (*n* = 4–6) from achiral precursors in a single step with generally excellent yields and enantioselectivities (enantiomeric ratio (e.r.) up to 98:2; Fig. 1c). Density functional theory (DFT) calculations have shed light on a complex mechanistic pathway in which both the C–H activation and reductive elimination steps have an impact on the enantioselectivity. Moreover, as this method has enabled the access to scarcely reported configurationally stable carbo[4]helicenes^15,35,36^, we have performed a comparative study of the photophysical and chiroptical properties of carbo[4]-, carbo[5]- and carbo[6]helicenes. The influence of the substitution pattern on the luminescence dissymmetry factors (*g*_lum_) of the smallest of these congeners was rationalized by calculation of the transition electric dipole moments (TEDMs) and transition magnetic dipole moments (TMDMs) in the excited states (S_1_–S_0_ transition).

## Results and discussion

### Method development

**Fig. 2:**
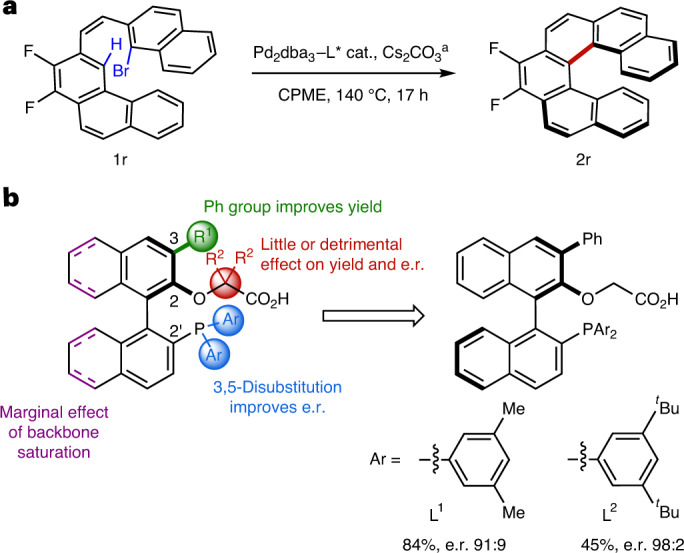
Ligand structure–activity relationship in the enantioselective synthesis of carbo[6]helicene 2r. **a**, Catalytic C–H arylation of **1r** leading to carbo[6]helicene **2r**. ^a^Optimized reagents and conditions: **1r** (0.1 mmol, 1.0 equiv.), Pd_2_dba_3_ (5 mol%), ligand (20 mol%), Cs_2_CO_3_ (0.5 equiv.), CPME (1 ml), 140 °C, 17 h. CPME, cyclopentyl methyl ether; cat., catalyst. **b**, Effect of ligand structure and substitution pattern on the yield and enantioselectivity of the C–H arylation of **1r** with the optimal ligands **L**^**1**^ and **L**^**2**^ selected in this study. The e.r. values were determined by HPLC on a chiral stationary phase.

**Fig. 3:**
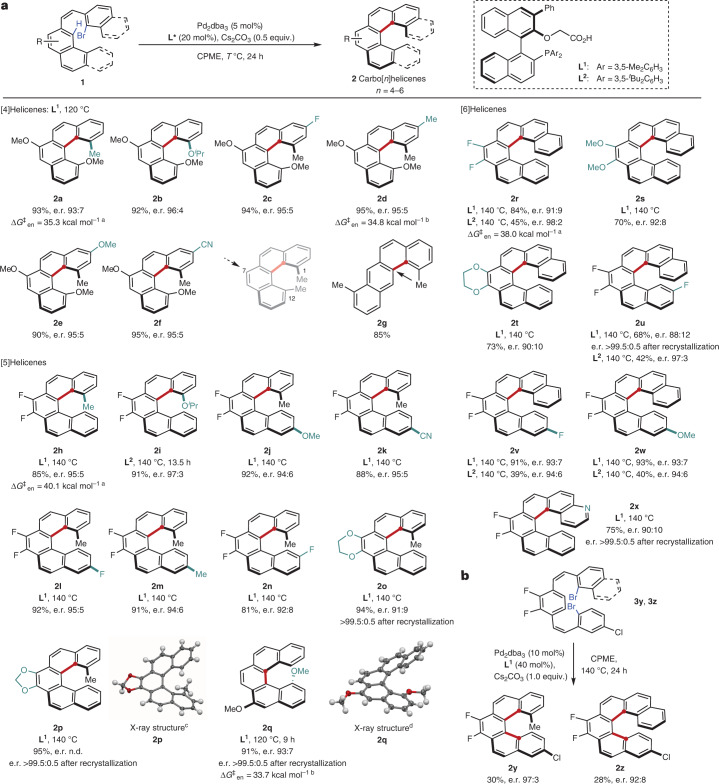
Scope of the enantioselective synthesis of carbo[*n*]helicenes. **a**, Scope of the monoarylation reaction. This method allows access to the three types of lower helicenes with various substitution patterns in high yields and enantioselectivities and is applicable to azahelicenes. Standard reagents and conditions: **1** (0.1 mmol, 1.0 equiv.), Pd_2_dba_3_ (5 mol%), ligand (20 mol%), Cs_2_CO_3_ (0.5 equiv.), CPME (1 ml), *T* °C, 24 h. The X-ray crystallographic structures of **2p** and **2q** are shown. ^a^Free energy of enantiomerization computed at the B3LYP-D3(BJ)/6-311G(d,p) level of theory. ^b^Experimental enantiomerization barrier measured at 120 °C. ^c^Thermal ellipsoids are shown at the 50% probability level. ^d^Thermal ellipsoids are shown at the 20% probability level. **b**, Synthesis of carbo[5]- and carbo[6]helicenes by double C–H arylation. This method allows a more direct access to these helicenes, albeit in lower yields. Reagents and conditions: **3** (0.1 mmol, 1.0 equiv.), Pd_2_dba_3_ (10 mol%), **L**^**1**^ (40 mol%), Cs_2_CO_3_ (1.0 equiv.), CPME (1 ml), 140 °C, 24 h. The e.r. values were determined by HPLC on a chiral stationary phase. The reference racemic products were synthesized using PCy_3_ instead of the chiral ligand. The absolute configurations were ascribed in analogy to **2p** and **2q**, and by comparing the calculated with the experimental ECD spectra for selected compounds. The red dots indicate the initial position of the bromide. **a**,**b**, Light blue highlights changes in substituents.

Based on the known stability of unsubstituted carbo[6]helicene (Fig. 1a), 3,4-difluorohexahelicene (**2r**) was deemed to possess sufficient stability to withstand prolonged periods of heating at 140 °C without substantial racemization (Fig. 2a). Hence, we initially studied the C–H arylation of substrate **1r**, in which the bromonaphthalene and phenanthrene rings are bridged by a *Z* olefin, and fluorine atoms are installed on the phenanthrene moiety to avoid undesired competitive C–H arylation at these sites leading to non-helicene products (vide infra). Bifunctional binaphthyl-based chiral phosphine-carboxylic acids quickly emerged as the most promising ligands for the reaction^29,30^, as compared with monofunctional ligands, including binaphthyl-based ones, in combination with an external carboxylic acid co-catalyst (Supplementary Fig. 1). Optimization studies on the ligand structure revealed a notable improvement in yield when a phenyl group was introduced at the 3-position of the binaphthyl core (Fig. 2b). In addition, 3,5-disubstitution of the aryl substituents on the phosphorus atom was found to be beneficial to the enantioselectivity. In particular, methyl substituents (**L**^**1**^) provided a higher yield (84%) and an e.r. of 91:9, whereas *tert*-butyl substituents (**L**^**2**^) provided a markedly enhanced e.r. of 98:2, albeit with a lower yield (45%). Substitution at the α-position of the carboxylic acid or partial saturation of the binaphthyl core led to detrimental or marginal effects on the yield and enantioselectivity. Further optimization of the base and solvent led to the standard conditions indicated in Fig. 2, and both ligands **L**^**1**^ and **L**^**2**^ were used in subsequent studies.

### Scope of the enantioselective synthesis of carbo[*n*]helicenes

Using the optimal conditions, we examined the versatility of the enantioselective C–H arylation reaction for the synthesis of lower carbo[*n*]helicenes (Fig. 3a). Due to the low enantiomerization barrier of unsubstituted carbo[4]- and carbo[5]helicenes, at least one of the terminal positions of the cove or fjord region was blocked with a substituent to generate configurationally stable products. The enantiomerization barriers for representative carbo[*n*]helicenes **2a**, **2h** and **2r** were calculated to be 35–40 kcal mol^−1^ (Supplementary Figs. 119–121), consistent with literature values^34^, which indicated that the various products should indeed possess sufficient configurational stability under the reaction conditions. The precursors **1** were synthesized by Wittig reaction to establish the required *Z* alkene linker between the two aryl fragments. In addition, as illustrated with **2g**, the current strategy requires the most reactive *ortho* position to the alkene on the naphthalene/phenanthrene (the 7-position on the helicene product) to be blocked to avoid cyclization on this less hindered position, which readily furnishes the corresponding non-helicene polyaromatic system. These precautions pending, the scope of the reaction was explored using **L**^**1**^ as the preferred ligand. In some cases, **L**^**2**^ was employed to achieve higher enantioselectivities.

First, a range of carbo[4]helicenes were successfully synthesized by intramolecular C–H arylation of substituted naphthalenes with *ortho*-substituted phenyl bromide precursors at 120 °C using **L**^**1**^ as the chiral ligand. Different terminal groups, including methyl, methoxy and isopropoxy groups, were found to be suitable (**2a**,**b**). Substrates containing electron-donating or -withdrawing groups on the bromobenzene ring also readily participated in this reaction to afford products **2c**–**f**. Remarkably, all carbo[4]helicene products were obtained in similar excellent yields (90–95%) and enantioselectivities (e.r. 93:7–96:4). The value of Δ*G*^‡^_en_ for **2d** was measured to be 34.8 kcal mol^−1^ in *m*-xylene at 120 °C, in agreement with the calculated barrier for **2a** of 35.3 kcal mol^−1^, hence confirming the configurational stability of these [4]helicenes.

The reaction was also found to be applicable to the synthesis of carbo[5]helicenes, for which only one blocking substituent on a terminal ring of the fjord region is in principle necessary to obtain configurationally stable products. Thus, the intramolecular C–H arylation of phenanthrenes with *ortho*-substituted phenyl bromides was first considered in analogy to carbo[4]helicenes. A methyl (**2h**) or isopropoxy (**2i**) group at the *ortho* position to the initial bromide provided excellent results. However, a methoxy substituent at this position of the fjord region led to a product (**2q**) that slowly racemized at 120 °C. The enantiomerization barrier of **2q** was measured to be 33.7 kcal mol^−1^ in *m*-xylene at 120 °C, which confirms the lower configurational stability of this particular product (compared with **2h**). In this case, decreasing the reaction time to 9 h was sufficient to limit this racemization, and **2q** was obtained with an e.r. of 93:7. The absolute configuration of **2q** was determined by X-ray crystallographic analysis to be *M*. Diverse substituents on the terminal (**2i**–**n**) and central (**2h** and **2o**,**p**) rings of the incipient carbo[5]helicene were very well tolerated, again affording excellent yields (81–95%) and e.r. values up to 97:3. In some instances, a very high optical purity (e.r. >99.5:0.5) was easily attained by simple recrystallization of the product (**2o**–**q**). Notably, product **2q** was obtained from a naphthalene-bromonaphthalene precursor instead of a phenanthrene-bromobenzene. This type of substrate allows access to a carbo[5]helicene with an unsubstituted central ring, which highlights the flexibility of the current method to access carbohelicenes with different substitution patterns.

Compared with carbo[4]- and carbo[5]helicenes, carbo[6]helicenes have larger extended π-surfaces, which makes the reaction more challenging. In this case, we investigated the C–H arylation of substituted phenanthrenes with *ortho*-substituted naphthyl bromides, and both **L**^**1**^ and **L**^**2**^ were tested to achieve optimal results. The ligand **L**^**1**^ consistently produced high yields of 68–93% and e.r. values of around 90:10 (**2r**–**x**), whereas with **L**^**2**^ the yields were lower, but the enantioselectivity was markedly improved (e.r. values of 94:6 to 98:2 for **2r** and **2u**–**w**). Similarly to the carbo[5]helicenes, the optical purity was further improved upon recrystallization (**2u**). Azahelicenes are important heteroanalogues of carbohelicenes with interesting chiroptical properties^7,37^. Therefore, in light of this interest, we prepared and tested a bromoquinoline (**1x**) to further probe the versatility of the developed catalytic enantioselective protocol beyond carbohelicenes. Gratifyingly, the corresponding aza[6]helicene **2x** was obtained in 75% yield with an e.r. of 90:10, and could be further enantioenriched upon recrystallization.

In addition, because the phenanthrene substrates for the synthesis of carbo[5]- and carbo[6]helicenes were themselves constructed through Pd^0^-catalysed C–H arylation with Pd(OAc)_2_–PCy_3_ as the catalyst, we considered the possibility of directly generating helicenes by double C–H arylation from a dibrominated precursor **3** using our chiral catalyst (Fig. 3b)^32^. Gratifyingly, using **L**^**1**^ as the ligand, both the carbo[5]helicene **2y** and carbo[6]helicene **2z** were isolated with levels of enantioselectivity comparable to those obtained in the monoarylation reactions (e.r. 97:3 and 92:8, respectively), albeit in low yields due to the formation of several byproducts. Note that the reaction is compatible with a chloride substituent, which provides further handles for derivatization. Investigations were conducted with an achiral catalyst to better understand the reduced efficiency of the double C–H arylation. By comparing the reaction of a naphthyl bromide and a phenyl bromide to generate the same carbo[6]helicene, we found that the former was much more efficient (76% yield) than the latter (0% yield), likely due to steric effects. This result explains why double C–H arylation, where the order of C–H arylation events cannot be easily controlled, is less efficient than the stepwise sequence.

## Mechanistic study

**Fig. 4:**
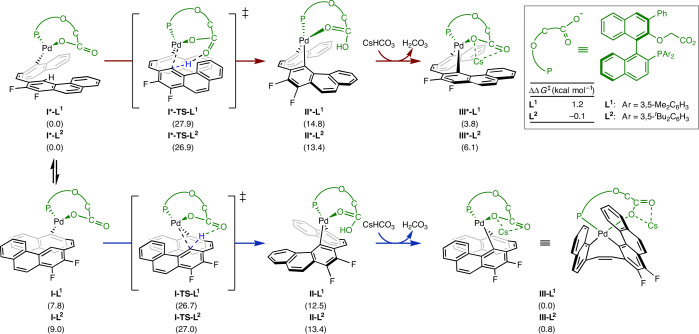
Computed C–H activation pathways in the synthesis of carbohelicenes. C–H activation pathways calculated at the PCM(toluene)-B3LYP(D3)/SDD+def2-TZVP//B3LYP(D3)/SDD+def2-SVP level of theory (values in parentheses are Δ*G* (in kcal mol^−1^)). Two C–H activation pathways (red and blue arrows) starting from intermediates **I-L**^**n**^ and I*-L^**n**^, that arise from oxidative addition and displacement of the bromide with the carboxylate group of the ligand, proceed with similar energy barriers and both contribute to the observed enantioselectivity. Subsequent deprotonation by caesium bicarbonate and coordination of Cs^+^ is exergonic and leads to intermediates **III-L**^**n**^ and III*-L^**n**^, respectively. The Cartesian coordinates for the optimized structures are provided in the Supplementary Information.

DFT calculations were performed to understand how the chiral bifunctional ligands induce enantioselectivity in the reaction. As the initial data were obtained with substrate **1r**, the corresponding oxidative addition complexes **I-L**^**n**^ (*n* = 1, 2) were chosen as models for these mechanistic studies (Fig. 4). Depending on how the substrate is positioned in the metal complexes, two isomers may be observed: **I-L**^**n**^ and I*-L^**n**^. Complex **I-L**^**1**^ undergoes C–H activation via **I-TS-L**^**1**^ with Δ*G*^‡^ = 26.7 kcal mol^−1^ to form **II-L**^**1**^ (bottom), whereas C–H activation in I*-L^**1**^ proceeds via I*-TS-L^**1**^ with a higher activation energy by ΔΔ*G*^‡^ = 1.2 kcal mol^−1^ than **I-TS-L**^**1**^ to form II*-L^**1**^ (top). This concerted metallation–deprotonation was computed to feature the highest activation barrier of the energy profile (see Supplementary Figs. 2 and 3 for the overall reaction profiles with **L**^**1**^ and **L**^**2**^). Meanwhile, the barrier for C–H activation in **I-L**^**2**^ is 27.0 kcal mol^−1^ via **I-TS-L**^**2**^, whereas the activation in I*-L^**2**^ has a lower barrier, albeit by only ΔΔ*G*^‡^ = −0.1 kcal mol^−1^, via I*-TS-L^**2**^. The small ΔΔ*G*^‡^ energy differences between these two C–H activation pathways imply that they both play a role in the stereochemical outcome of the reaction. The subsequent deprotonation of the complexes **II-L**^**n**^ and II*-L^**n**^ with caesium bicarbonate and Cs^+^ coordination is exergonic and provides the complexes **III-L**^**n**^ and III*-L^**n**^, respectively.

**Fig. 5:**
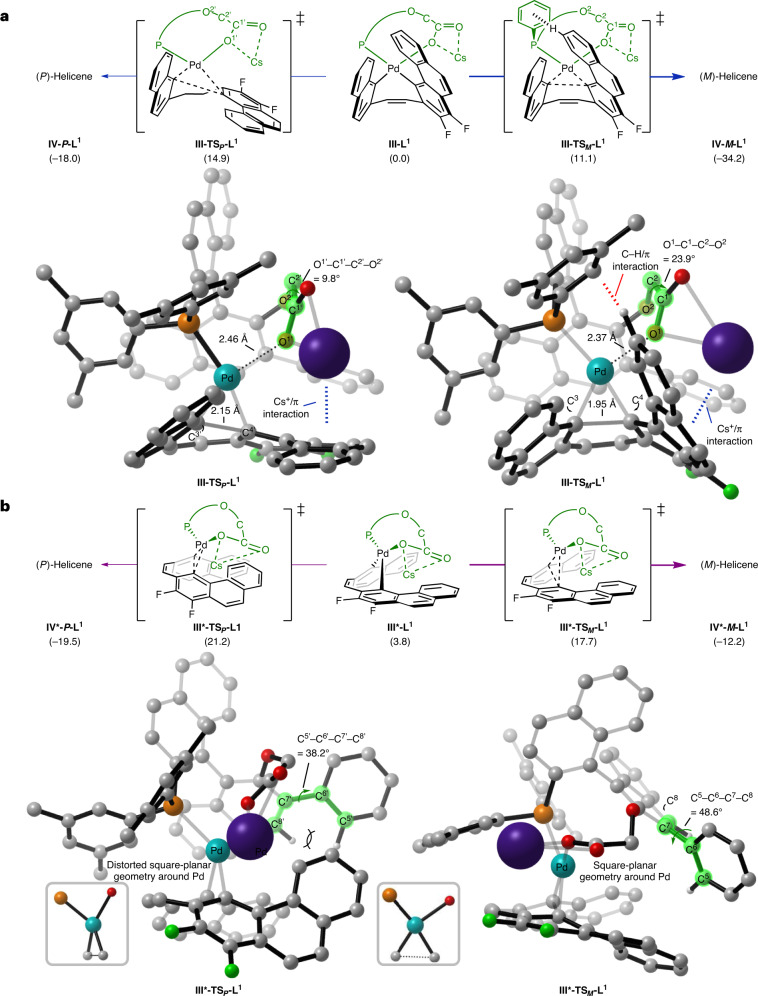
Computed reductive elimination pathways leading to carbo[6]helicene 2r. Reductive elimination pathways towards carbo[6]helicene **2r** with **L**^**1**^ as the ligand calculated at the PCM(toluene)-B3LYP(D3)/SDD+def2-TZVP//B3LYP(D3)/SDD+def2-SVP level of theory (the values in parentheses are Δ*G* (in kcal mol^−1^)). **a**,**b**, Reductive elimination from **III-L**^**1**^ (**a**) and III*-L^**1**^ (**b**). Bold arrows represent the energetically favoured pathways. Structure **IV** corresponds to helicene **2r** coordinated to Pd–**L**^**1**^. These reductive eliminations are enantiodetermining and the enantioselectivity is controlled by NCIs and steric repulsion between the substrate and ligand. For reductive elimination pathways with **L**^**2**^ and selected NCI plots, see Supplementary Figs. 6 and 7.

**Fig. 6:**
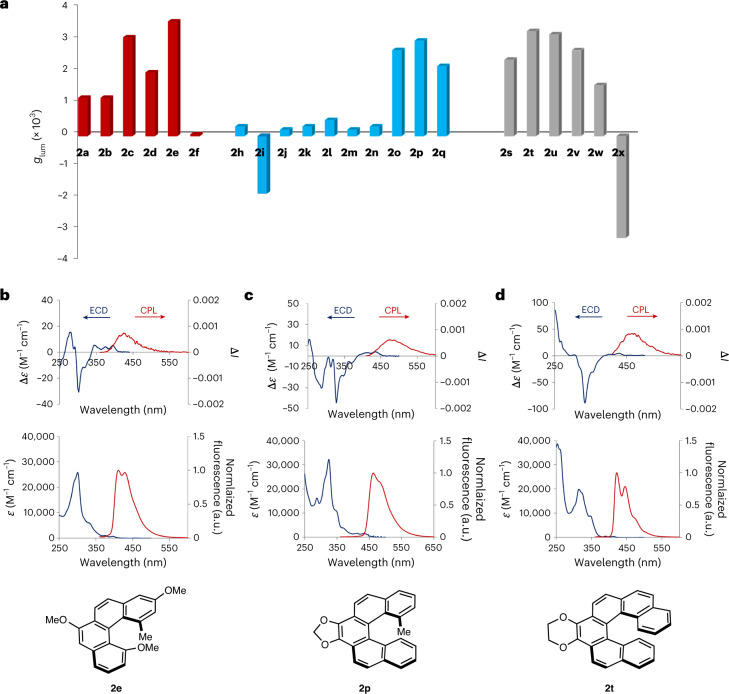
Circularly polarized absorption and luminescence of the synthesized [*n*]helicenes. **a**, Luminescence dissymmetry factors (at *λ*_max,em_) for [4]helicenes (red bars), [5]helicenes (blue bars) and [6]helicenes (grey bars) in dichloromethane (*c* = 10^–5^ M). **b**–**d**, ECD (blue traces) and CPL (red traces) spectra (top), and UV–visible (blue traces) and luminescence spectra (red traces) (bottom) of carbohelicenes **2e** (**b**), **2p** (**c**) and **2t** (**d**). Significant CPL was observed for the three types of helicenes, which was strongly impacted by the substitution in the [4]helicene and [5]helicene series.

Next, the reductive elimination from the complex **III-L**^**n**^ was computed to proceed via **III-TS**_***M***_**-L**^**n**^ or **III-TS**_***P***_**-L**^**n**^, producing the (*M*)- and (*P*)-carbo[6]helicene **IV**, respectively (Fig. 5a). The corresponding activation barriers were calculated to be Δ*G*^‡^ = 11.1 kcal mol^−1^ for **III-TS**_***M***_**-L**^**1**^ and Δ*G*^‡^ = 14.9 kcal mol^−1^ for **III-TS**_***P***_**-L**^**1**^, hence strongly favouring the experimentally observed major *M* enantiomer of **2r**. To determine the origins of the enantioselectivity, we carefully analysed the optimized transition-state (TS) structures of the reductive elimination step. We observed that non-covalent interactions (NCIs) between the catalyst and substrate strongly influence the enantiodetermining process (Fig. 6a)^38–41^. As shown in the NCI plots in Supplementary Fig. 6 (ref. ^42^), both **III-TS**_***M***_**-L**^**1**^ and **III-TS**_***P***_**-L**^**1**^ display cation/π interactions between Cs^+^ and the phenanthrene^43^, with a distance of 3.52(1) and 3.57(0) Å, respectively. The difference in the relative strengths of these Cs^+^/π interactions in these TSs is marginal based on the distances. However, there is a notable C−H/π interaction between the phenanthrene moiety of the substrate and one of the *m*-xylyl rings of the ligand in **III-TS**_***M***_**-L**^**1**^ that is absent in **III-TS**_***P***_**-L**^**1**^. This interaction might explain the experimentally observed effect of this aryl substituent of the ligand on the enantioselectivity (Fig. 2), and the decreased e.r. observed for compound **2u** bearing a fluorine atom at this position of the phenanthrene (Fig. 3a). In addition, the caesium carboxylate moiety in **III-TS**_***M***_**-L**^**1**^, in which the substrate phenanthrene moiety is tilted upwards, is positioned in a more staggered arrangement with a dihedral angle O^1^–C^1^–C^2^–O^2^ of 23.9°, providing the optimal Cs^+^/π interaction. In contrast, **III-TS**_***P***_**-L**^**1**^ has a dihedral angle O^1′^–C^1′^–C^2′^–O^2′^ of 9.8° to maintain the analogous Cs^+^/π interaction. The staggered conformation in **III-TS**_**M**_**-L**^**1**^ places O^1^ closer to Pd to give a shorter Pd···O^1^ distance of 2.37 Å, contributing to the stabilization of the TS, compared with in **III-TS**_***P***_**-L**^**1**^, which features a longer Pd···O^1′^ distance of 2.46 Å. Another stark difference was identified in the forming C^3(′)^–C^4(′)^ bond in **III-TS**_***M***_**-L**^**1**^ and **III-TS**_***P***_**-L**^**1**^, with a distance of 1.95 and 2.15 Å, respectively. This difference indicates that **III-TS**_***M***_**-L**^**1**^ is a later TS and takes advantage of the aromatization, which contributes to the facile formation of the (*M*)-carbohelicene.

Similarly, we located two diastereoisomeric TSs from intermediate III*-L^**1**^ through the second C–H activation pathway, namely III*-TS_***M***_**-L**^**1**^ and III*-TS_***P***_**-L**^**1**^, with the former being kinetically more accessible than the latter by 3.5 kcal mol^−1^, hence also favouring the (*M*)-carbohelicene product (Fig. 5b). The energy difference between these two TSs can be attributed to the different torsion angles C^5(′)^–C^6(′)^–C^7(′)^–C^8(′)^ of the phenyl-naphthyl moiety of the ligand^44,45^. Whereas III*-TS_***M***_**-L**^**1**^ has a favourable torsion angle C^5^–C^6^–C^7^–C^8^ of 48.6°, the phenyl-naphthyl system in III*-TS_***P***_**-L**^**1**^ displays a smaller torsion angle C^5′^–C^6′^–C^7′^–C^8′^ of 38.2° to avoid steric collision with the phenanthrene moiety of the substrate. In addition, III*-TS_***P***_**-L**^**1**^ deviates from the ideal square-planar geometry around the Pd to avoid the steric clash between the ligand and the phenanthrene of the substrate. In contrast, III*-TS_***M***_**-L**^**1**^ lacks this steric repulsion, with the phenanthrene positioned away from the phenyl-naphthyl moiety of the ligand. The TSs for the reductive elimination from intermediates **III-L**^**2**^ and III*-L^**2**^, which are qualitatively similar to those observed with **L**^**1**^, are fully illustrated in Supplementary Fig. 7.

As the diastereoisomeric TSs in each pathway display lower barriers than those of the C–H activation step, the current calculations indicate that the reductive elimination step is enantiodetermining. However, as the two C–H activation TSs have a comparable energy for both ligands **L**^**1**^ and **L**^**2**^, this step also has an impact on the quantitative product distribution, hence reflecting the complexity of the reaction dynamics.

## Study of photophysical and chiroptical properties

The photophysical and chiroptical properties of selected carbohelicenes were studied in dichloromethane by UV–visible, fluorescence (including prompt photoluminescence quantum yield (PLQY) and fluorescence lifetime measurements), electronic circular dichroism (ECD) and CPL spectroscopy. In addition, time-dependent DFT (TD-DFT) calculations were performed on selected compounds (**2a**, **2c–f**, **2p** and **2x**) in the excited state to understand the origin of the variation of the *g*_lum_ values with the substitution pattern. The dissymmetry factors in the excited state are reported in Fig. 6 and the PLQY values in Supplementary Fig. 118. For all compounds, the emission maxima (*λ*_max,em_), which range from 420 to 440 nm, were only slightly affected by the nature and position of the substituents, and mostly depended on the length of the helicene. The measured PLQYs (with an integrating sphere) are in line with those previously reported for carbohelicenes^46^, with values in the range of 2–13%. Notably, the carbo[5]- and carbo[6]helicenes incorporating a fused dioxolane or dioxane ring (**2o**, **2p** and **2t**) were among the compounds exhibiting the highest PLQY values (9.3–10%). Some substitution patterns positively affected the *g*_lum_ values. Indeed, in the carbo[4]helicene series, a substituent in the 3-position led to a notable increase in *g*_lum_ (**2a**, 1.2 × 10^–3^; **2d**, 1.8 × 10^–3^; **2c**, 3.1 × 10^–3^; **2e**, 3.6 × 10^–3^). TD-DFT calculations of the excited state (B3LYP-D3(BJ)/6-311+G(2d,2p)/IEF-PCM(DCM)) reproduced relatively well the experimental results, despite the calculated *g*_lum_ values being slightly overestimated compared with the experimental values^47,48^. Theoretically, the luminescence dissymmetry factors can be calculated using the formula *g*_lum_ = 4*µm*cos *θ*/(*µ*^2^ + *m*^2^), where *µ* is the TEDM, *m* is the TMDM and *θ* is the angle between these two transition moments relative to the S_1_–S_0_ transition. Analysis of the magnitude and relative orientation of the TEDM and TMDM suggests that the higher *g*_lum_ values measured for **2c** and **2e** in the [4]helicene series are mainly due to the more favourable orientation of the TEDM and TMDM, resulting in a higher value of cos *θ*. Although the presence of the nitrile group positively influenced the PLQY of **2f** (13% compared with 8% for **2a**), it was deleterious to the *g*_lum_ value, reaching only (0.1–0.2) × 10^–3^ at the emission maximum (bisignated CPL signal, Supplementary Fig. 35). Once again, the theoretical calculations of the excited state reproduced this experimental result well, with a computed *g*_lum_ value of 0.5 × 10^–3^ due to a smaller amplitude of the TMDM and an unfavourable orientation of the transition dipole moment vectors (leading to a low value of cos *θ* = 0.14) compared with the other [4]helicenes. Typically, the carbo[5]helicenes displayed the lowest *g*_lum_ values ((0.2–0.5) × 10^–3^), with the exceptions of **2o**–**q** ((2.2–3.0) × 10^–3^), suggesting that the fluorine atoms in the 7- and 8-positions have a detrimental effect on the chiroptical properties. Theoretical calculations on **2p** indicated a favourable relative orientation of the TEDM and TMDM for the S_1_–S_0_ transition, with a cos *θ* value of 0.77. The *g*_lum_ values of the carbo[6]helicene series were less affected by the substitution pattern. Interestingly, the aza[6]helicene **2x** displayed a sign inversion of the CPL as well as an enhanced dissymmetry factor (reaching –3.4 × 10^–3^ at *λ*_max,em_, Supplementary Fig. 51) compared with the other studied [6]helicenes. In this case also, the computational results are in line with the experimental data (both in terms of sign and amplitude), with a calculated *g*_lum_ value of –4.1 × 10^–3^.

## Conclusion

A general enantioselective access to lower carbo[*n*]helicenes has been developed through Pd^0^-catalysed C–H arylation in the presence of a chiral phosphine-carboxylate bifunctional ligand. This method provides good yields and high enantioselectivities (e.r. values up to 98:2) across a broad range of helicene products, and is also applicable to the synthesis of an aza[6]helicene. Carbo[5]- and carbo[6]helicenes can also be accessed by double C–H arylation of dibrominated precursors with similar levels of enantioselectivity, albeit in lower yields. Computational studies have shed light on the reaction mechanism, in particular, on the contribution of both the C–H activation and reductive elimination steps to the overall enantioselectivity. In addition, a combination of NCIs and steric repulsion between the substrate and ligand was shown to be responsible for the enantio-induction in the two key reductive elimination steps. A comparative study of the photophysical and chiroptical properties of selected helicene products showed a relatively high CPL response for the carbo[4]helicene congeners. TD-DFT calculations of the excited state provided insights into the origin of the variation of the *g*_lum_ values as a function of the substitution pattern. As the synthetic access to the carbo[4]helicenes is relatively straightforward, these results suggest new avenues for the optimization of chiroptical properties of helicene systems.

## Methods

### General procedure for the enantioselective C–H arylation

Aryl bromide **1** (0.1 mmol, 1 equiv.) and (*R*)-**L**^**1**^ (12.9 mg, 20 μmol, 20 mol%) or (*R*)-**L**^**2**^ (16.3 mg, 20 μmol, 20 mol%) were added to an oven-dried 5-ml microwave vial under ambient air. The vial was sealed with a septum, placed under vacuum and then flushed three times with Ar. The vial was then transferred to a glove box and Pd_2_dba_3_ (4.58 mg, 5 μmol, 5 mol%) and dry, ground Cs_2_CO_3_ (16.5 mg, 0.05 mmol, 0.5 equiv.) were added. CPME (1.0 ml, 0.1 M) was added to the mixture before sealing the vial and removing it from the glove box. The reaction mixture was heated at 120 or 140 °C for 24 h and then filtered through a plug of silica gel, eluted with ethyl acetate and concentrated under reduced pressure. The resulting crude product was purified by column chromatography on silica gel.

## Online content

Any methods, additional references, Nature Portfolio reporting summaries, source data, extended data, supplementary information, acknowledgements, peer review information; details of author contributions and competing interests; and statements of data and code availability are available at 10.1038/s41557-023-01174-5.

## Supplementary information

Supplementary Information Supplementary Figs. 1–125 and Table 1.

Supplementary Data 1 Cartesian coordinates of DFT-optimized structures.

Supplementary Data 2 Crystallographic data for compound **2p**; CCDC reference 2142962.

Supplementary Data 3 Crystallographic data for compound **2q**; CCDC reference 2142961.

## Data Availability

The data supporting the findings of this study are available in the Supplementary Information. The crystallographic data for the structures reported in this Article have been deposited at the Cambridge Crystallographic Data Centre, under deposition numbers CCDC 2142962 (**2p**) and 2142961 (**2q**). Copies of the data can be obtained free of charge via https://www.ccdc.cam.ac.uk/structures/.
