# Supplementary material for: A C–H activation-based enantioselective synthesis of lower carbo[n]helicenes
Source: Nat Chem. 2023 Apr 6;15(6):872–80. doi: 10.1038/s41557-023-01174-5 (PMC10239729; doi:10.1038/s41557-023-01174-5)
Supplement: Supplementary file 1 — Supplementary Figs. 1–125 and Table 1. [file 41557_2023_1174_MOESM1_ESM.pdf]

nature chemistry

Article

https://doi.org/10.1038/s41557-023-01174-5

A C–H activation-based enantioselective synthesis of lower carbo[*n*]helicenes

In the format provided by the authors and unedited

Table of Contents

General Methods 2

Experimental Sections 4

General procedure for ligand optimization: 4

General route for ligand synthesis 6

2-Bromobenzyl bromide phosphonium salt 13

General routes for C–H activation substrates synthesis: 18

General procedure for the enantioselective C–H arylation 46

Proof-of-concept double C–H arylation 82

Racemization Kinetics 90

X-ray Structures 95

Computational Mechanism Study 97

Photophysical and Chiroptical Properties Study 106

General information: 106

UV-Fluo spectra: 107

Circularly polarized luminescence spectra: 118

Circular dichroism spectra: 140

Lifetime measurement: 179

Quantum yield measurements: 201

Photophysical properties of helicenes: 223

DFT calculations: 225

DFT Comparison between the experimental and theoretical glum values (x 1000): 320

References 321

NMR Spectra 325

General Methods
Experimental procedures, reagents and glassware

All commercially available chemicals were used without additional purification for the synthesis of substrates. All reactions were carried out under an atmosphere of argon in oven-dried glassware with magnetic stirring, unless otherwise indicated. Cyclopentyl methyl ether (CPME) was dried over CaH_2 and $NaBH_4$, distilled and stored inside the Ar-filled glovebox. Toluene, tetrahydrofuran diethyl ether and dichloromethane were purified by the Innovative Technology Solvent Delivery System. Chemicals were used as obtained from the suppliers unless otherwise stated. Solvent compositions are given in (v/v).

Chromatography

Flash chromatography was performed with Silicycle silica gel 60 (0.040-0.063 μm grade). Analytical thin-layer chromatography was performed with commercial glass plates coated with 0.25 mm silica gel (E. Merck, Kieselgel 60 F254). Compounds were visualized under UV-light at 254 nm or 366 nm and oxidized in standard $KMnO_4$ dye followed by heating if necessary.

NMR spectroscopy

Nuclear magnetic resonance spectra were recorded on a Bruker Advance 400 (400 MHz), a Bruker Advance 500 (500 MHz) or a Bruker Advance 600 (600 MHz) in deuterated chloroform (residual peaks ^{1}H δ 7.26 ppm, ^{13}C δ 77.16 ppm) unless otherwise noted.

Chemical shifts (δ) are reported in parts per million (ppm) relative to residual chloroform (s, 7.26 ppm), DMSO (2.50 ppm) or TMS (0.00 ppm). Proton decoupled Carbon-13 nuclear magnetic resonance (13C{1H} NMR) were acquired at 126 MHz on a Bruker AV500 spectrometer. Proton decoupled Fluorine-19 nuclear magnetic resonance (19F{1H} NMR) were acquired at 376 MHz on a Bruker AV400 spectrometer. Proton decoupled Phosphorus-31 nuclear magnetic resonance (31P{1H} NMR) were acquired at 162 MHz on a Bruker AV400 spectrometer. Splitting patterns are designated as s, singlet; d, doublet; t, triplet; q, quartet; p, pentet; hept, heptet; dd, doublet of doublets; dt, doublet of triplets; ddd, doublet of doublets of doublets; tt, triplet of triplets; tq, triplet of quartets; qt, quartet of triplets; m, multiplet. All NMR data were recorded at 298 K.

Infrared spectroscopy

IR spectra were recorded on an ATR Varian Scimitar 800 and are reported in reciprocal centimeters (cm^{-1}).

Mass spectrometry

High resolution mass spectra were recorded by Dr. M. Pfeffer and S. Mittelheisser (Department of Chemistry, University of Basel) on a Bruker maXis 4G QTOF ESI mass spectrometer with addition of $AgNO_3$[1 mM] in difficult cases.

Melting points

Melting points were obtained on a Büchi melting point M-565, and are uncorrected.

Enantiomeric excesses

HPLC analyses were performed using a Shimadzu Prominence system with SIL-20A auto sample, CTO-20AC column oven, LC-20AD pump system, DGU20A3 degasser and SPD-M20A Diode Array or UV/VIS detector. The following chiral columns from Daicel Chemical Industries were used: OJ (Chiralcel®), IA (Chiralpak®), ODH (Chiralcel®) in 4.6 x 250 mm size.

X-Ray analyses

X-ray analyses were performed by Dr. A. Prescimone and Dr. F. Fadaei Tirani at the EPF Lausanne.

Optical properties

Optical rotations were measured on an Anton Paar MCP 100 Polarimeter in a 0.7 mL micro cuvette (cell length 100mm) with NaD-Line (λ = 589 nm). The concentration (c) was given in g/100 mL.

Experimental Sections

General procedure for ligand optimization:

To an oven-dried 2 ml microwave vial, under ambient air, was added (*Z*)-3-(2-(1-bromonaphthalen-2-yl)vinyl)-1,2-difluorophenanthrene **1r** (0.05 mmol, 1 equiv) and ligand (10 µmol, 20 mol%). The vial was sealed with a septum and placed under vacuum, followed by flushing with Ar gas for 3 times. The vial was then transferred to a glovebox where $Pd_2(dba)_3$ (4.58 mg, 5 µmol, 10 mol%), dry and ground Cs_2CO_3 (32.9 mg, 0.1 mmol, 2.0 equiv) were added. *p*-Xylene (0.5 ml) was added to the mixture before the vial was sealed and taken out of the glovebox. The reaction mixture was heated at 140 °C for 17 h. The reaction mixture was then filtered through a plug of silica gel (eluted with EtOAc) and concentrated under reduced pressure. The resulting crude product was purified by column chromatography on silica gel (cyclohexane/EtOAc).

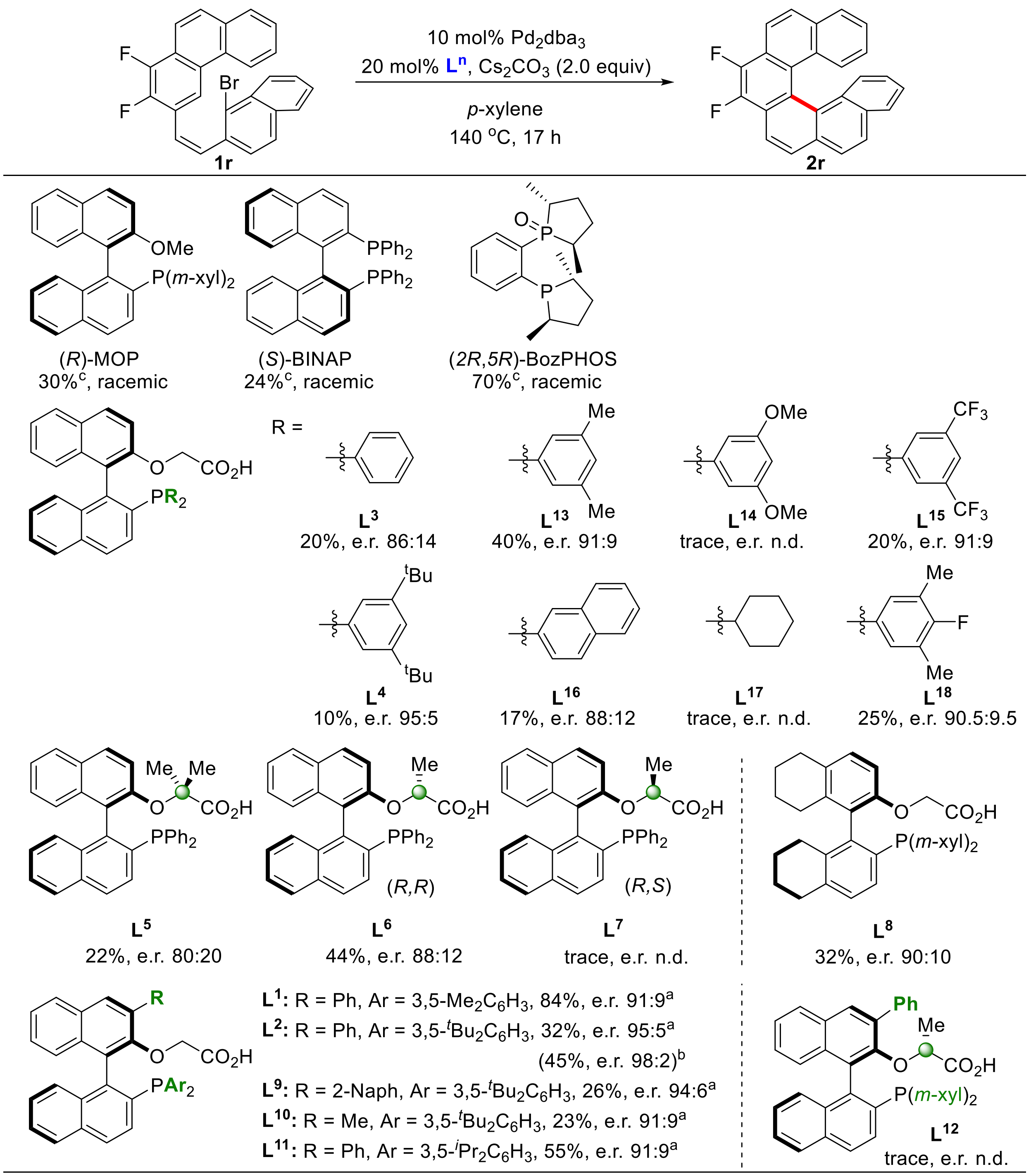

Fig. S1 Effect of the ligand structure on the enantioselective synthesis of carbo[6]helicene 2r. Standard conditions: **1a** (0.05 mmol, 1.0 equiv), Pd_2dba_3 (10 mol%), ligand (20 mol%), Cs_2CO_3 (2.0 equiv), *p*-xylene (0.5 mL), 140 °C, 17 h. [a]**1a** (0.1 mmol, 1.0 equiv), Pd_2dba_3 (5 mol%), ligand (20 mol%), Cs_2CO_3 (0.5 equiv), PhMe (1 mL), 140 °C, 17 h. [b]**1a** (0.1 mmol, 1.0 equiv), Pd_2dba_3 (5 mol%), ligand (20 mol%), Cs_2CO_3 (0.5 equiv), CPME (1 mL), 140 °C, 17 h. [c]CsOPiv (30 mol%) as additive. Yields were determined by ^{1}H-NMR with trichloroethene as internal standard. E.r. were determined by HPLC on a chiral stationary phase. CPME, cyclopentyl methyl ether; n.d., not determined.

General route for ligand synthesis:

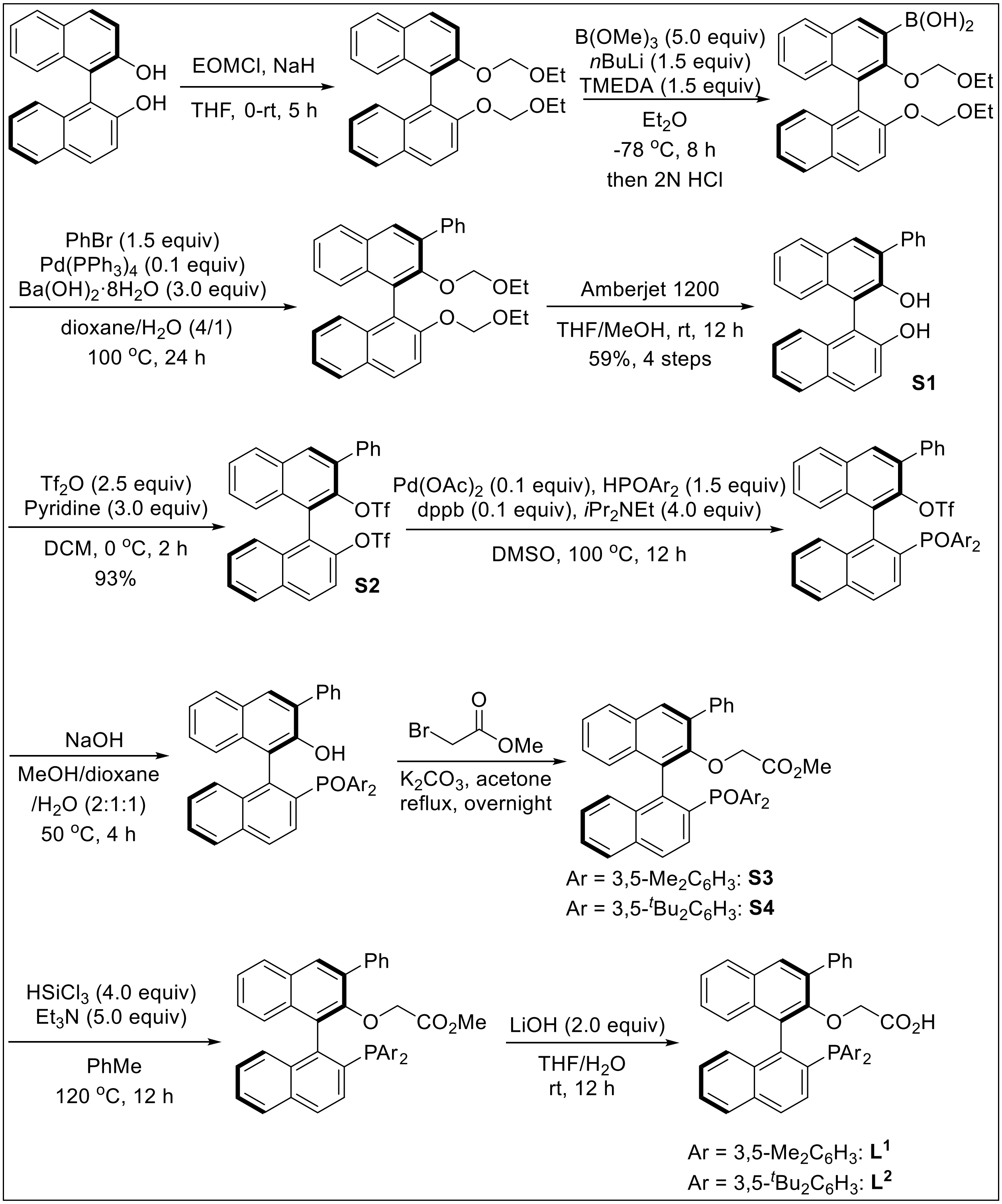

(*R*)-3-phenyl-[1,1'-binaphthalene]-2,2'-diol (S1): synthesized according to the following procedure.

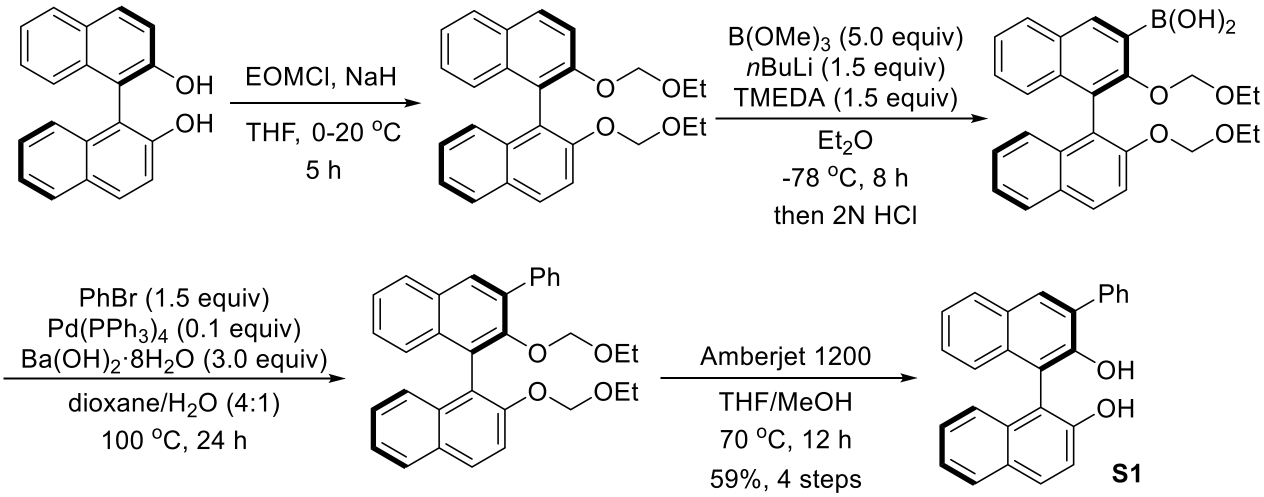

Protection of BINOL: NaH (0.624 g, 26.0 mmol) was mixed with dry THF (50 ml) in a 500 mL round bottom flask at 0 °C under argon atmosphere. To the mixture with stirring, was added a solution of (*R*)-BINOL (3.38 g, 11.8 mmol) in THF (15 mL). After the addition, the mixture was stirred at 0 °C for 1 h and then allowed to warm up to room temperature for 15 min. After the mixture was re-cooled to 0 °C, chloromethyl ethyl ether (2.41 mL, 26.0 mmol) was slowly added. After the addition, the reaction mixture was warmed to room temperature (around 20 °C) and stirred for 4.5 h. Saturated aqueous NH_4Cl (20 mL) was added to the flask and the product was extracted with ethyl acetate (3 × 100 mL). The organic layer was dried over anhydrous Na_2SO_4 and concentrated *in vacuo*. The obtained crude residue was used in the next step without further purification.

Borylation: In a 500 ml flask equipped with argon-inlet were placed Et_2O (150 ml) and TMEDA (2.86 ml, 19.0 mmol, 1.5 equiv). To this solution was added 2.5 M *n*BuLi in hexane (7.62 ml, 19.0 mmol, 1.5 equiv). The solution was stirred for 30 min at room temperature. The solution of crude (*R*)-2,2-bis(ethoxymethoxy)-1,1-binaphthyl from the last step in Et_2O (50 mL) was added, and the reaction was stirred for 3 h. The resulting light brown suspension was cooled to -78 °C, and $B(OMe)_3$ (4.25 mL, 38.1 mmol, 3.0 equiv) was added via syringe. The solution was allowed to warm to room temperature and stirred overnight. Water was added and the product was extracted with ethyl acetate (3 × 100 mL). The organic layer was dried over anhydrous Na_2SO_4 and concentrated *in vacuo*. The obtained crude product was used in the next step without further purification.

Suzuki coupling: A Schlenk tube was placed under argon atmosphere and charged with the crude product from the last step and $Ba(OH)_2•8H_2O$ (1.38 g, 8.07 mmol, 3.0 equiv). The flask was flushed with Ar gas for 3 times, introduced into a glovebox and charged with $Pd(PPh_3)_4$ (311 mg, 0.269 mmol, 10 mol%). Degassed 1,4-dioxane (40 mL) and water (10 mL) were added sequentially out

of the glovebox. Then bromobenzene (0.425 ml, 4.04 mmol, 1.5 equiv) was added. The tube was sealed and heated at 100 °C for 24 h. The reaction was cooled down to room temperature and filtered through a pad of celite. 1,4-Dioxane was then removed under reduced pressure and the residue was extracted with DCM. The combined organic phase was washed with brine and dried over anhydrous Na_2SO_4 and the solvent was removed under reduced pressure. The obtained crude product was used in the next step without further purification.

Deprotection: A 500 ml flask was charged with the crude (*R*)-2,2'-bis(ethoxymethoxy)-3-phenyl-1,1'-binaphthalene from the last step and Amberjet 1200 resin (1.269 g, 4.04 mmol, 1.5 equiv). THF (25 mL)/H_2O (25 mL) (1:1) was added under Ar gas. The reaction mixture was stirred and refluxed under Ar for 12 h. The reaction was cooled down to room temperature and filtered through a pad of celite. The filtrate was concentrated under reduced pressure and then extracted with ethyl acetate (3 × 100 ml). The crude was purified by column chromatography (cyclohexane/EtOAc 30/1) to afford (*R*)-3-phenyl-[1,1'-binaphthalene]-2,2'-diol (**S1**) (0.575 g, 59% for 4 steps).

White solid. $\boldsymbol{R_f}$ 0.35 (pentane/EtOAc 3/1); **^{1}H NMR** (500 MHz, Chloroform-*d*): δ 8.02 (s, 1H), 7.99 (d, *J* = 8.9 Hz, 1H), 7.92 (d, *J* = 8.1 Hz, 1H), 7.90 (d, *J* = 8.1 Hz, 1H), 7.77 – 7.71 (m, 2H), 7.49 (t, *J* = 7.6 Hz, 2H), 7.44 – 7.35 (m, 4H), 7.32 (tdd, *J* = 6.8, 3.8, 1.4 Hz, 2H), 7.23 (d, *J* = 8.4 Hz, 1H), 7.15 (d, *J* = 8.4 Hz, 1H), 5.28 (s, 1H), 5.11 (s, 1H). **^{13}C NMR** (126 MHz, Chloroform-*d*) δ 152.8, 150.4, 137.6, 133.6, 133.1, 131.7, 131.5, 130.9, 129.8, 129.7, 128.7, 128.6, 128.6, 128.0, 127.6, 124.6, 124.5, 124.4, 124.2, 117.9, 111.9, 111.6. **HRMS** (ESI): calcd. or $[C_{26}H_{18}O_2+Na]^+$, $[M+Na]^+$: 385.1199; found: 385.1204. **IR (ATR):** $\tilde{\nu}$ = 3502, 3058, 2923, 697, 1620, 1502, 1427, 1382, 1262, 1177, 1030, 960, 818, 750, 701 cm^{-1}. **$[\alpha]_D^{20}$**: +50.7 (c = 1.0, $CHCl_3$). **Mp**: 89.6 – 90.7 °C.

<u>(*R*)-3-phenyl-[1,1'-binaphthalene]-2,2'-diyl bis(trifluoromethanesulfonate) (S2):</u> synthesized according to the following procedure. Known compound.[1]

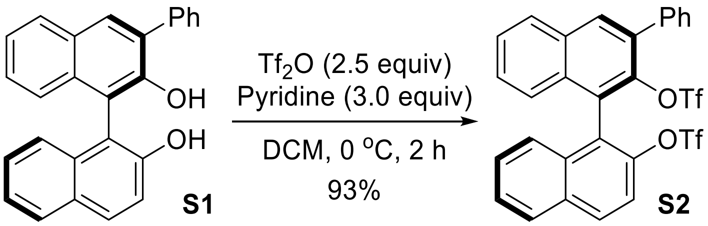

A 50 ml flask was charged with (*R*)-3-phenyl-[1,1'-binaphthalene]-2,2'-diol (**S1**) (0.575 g, 1.59 mmol, 1.0 equiv) and pyridine (0.385 ml, 4.77 mmol, 3.0 equiv) in CH_2Cl_2 (20 ml) under argon atmosphere. Trifluoromethanesulfonic acid anhydride (0.641 ml, 3.82 mmol, 2.4 equiv) was added at 0 °C. The reaction mixture was allowed to warm to room temperature and stirred for 2-5 h. The reaction was quenched with water, extracted with DCM (3 × 50 ml) and the combined organic layers were dried with anhydrous Na_2SO_4, filtered and concentrated under reduced pressure. The crude was purified by column chromatography (cyclohexane/EtOAc 20/1) to afford 3-phenyl-[1,1'-binaphthalene]-2,2'-diyl bis(trifluoromethanesulfonate) (**S2**) as white solid (0.925 g, 93%).

^{1}H NMR (400 MHz, Chloroform-*d*): δ 8.16 (d, *J* = 9.1 Hz, 1H), 8.11 (s, 1H), 8.02 (s, 1H), 8.00 (s, 1H), 7.68 – 7.55 (m, 5H), 7.53 – 7.48 (m, 2H), 7.48 – 7.35 (m, 4H), 7.22 (d, *J* = 8.6 Hz, 1H).

(*R*)-methyl 2-((2'-(bis(3,5-dimethylphenyl)phosphoryl)-3-phenyl-[1,1'-binaphthalen]-2-yl)-oxy)acetate (S3) and (*R*)-methyl 2-((2'-(bis(3,5-di-tert-butylphenyl)phosphoryl)-3-phenyl-[1,1'-binaphthalen]-2-yl)oxy)acetate (S4): synthesized according to the following procedure.

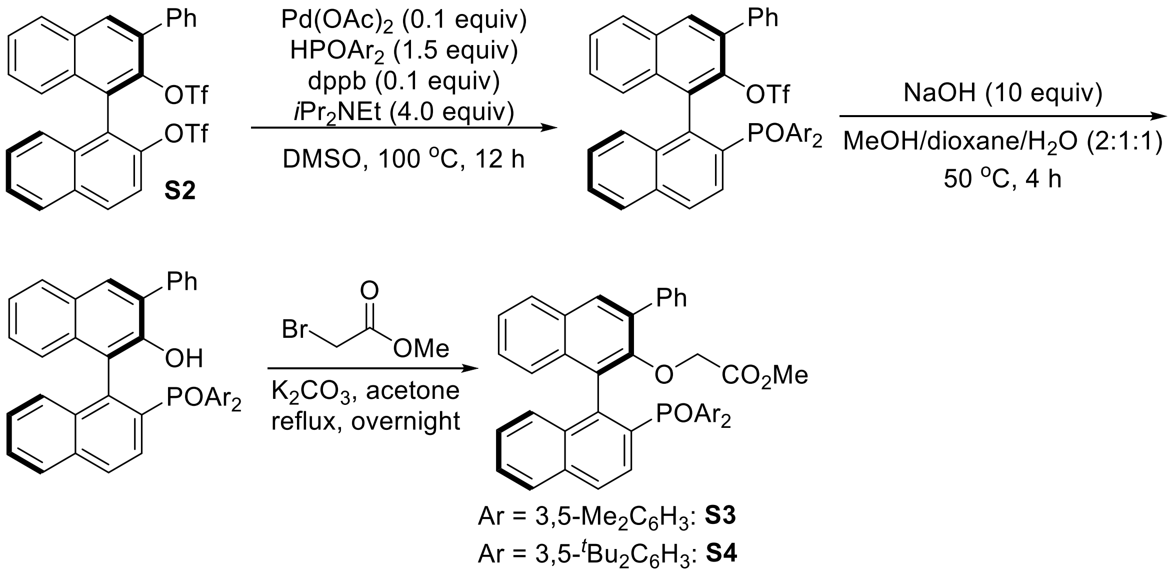

Synthesis of phosphine oxide: A Schlenk tube was charged with a mixture of (*R*)-3-phenyl-[1,1'-binaphthalene]-2,2'-diyl bis(trifluoromethanesulfonate) (**S2**) (1.0 equiv), diarylphosphine oxide (1.5 equiv), 1,4-bis (diphenylphosphino)propane (dppp) (0.1 equiv), palladium acetate (10 mol%) and diisopropylethylamine (4.0 equiv) in DMSO (0.1 M), and then heated at 100 °C under argon atmosphere. After 12 h, the reaction mixture cooled down to room temperature. The solvent was removed under reduced pressure. Water was added and extracted with ethyl acetate for three times. The combined organic layers were dried over anhydrous Na_2SO_4 and concentrated under reduced pressure. The obtained crude material was used in the next step without further purification.

Hydrolysis of triflate: A solution of phosphine oxide (1.0 equiv) obtained from the last step in 1,4-dioxane and MeOH was prepared and then water and NaOH (10 equiv) were added to the mixture (0.05 M, dioxane/MeOH/H_2O 2:1:1). The reaction mixture was stirred under 50 °C for 4 h. The reaction was monitored by TLC until no substrate was left. The mixture was cooled down to room temperature, concentrated under reduced pressure and extracted with ethyl acetate for three times. The combined organic layers were dried over anhydrous Na_2SO_4 and concentrated *in vacuo*. The obtained crude material was used in the next step without further purification.

Alkylation of hydroxyl group: A flask was charged with the compound (1.0 equiv) obtained from the last step and acetone (0.05 M). Potassium carbonate (5.0 equiv) and methyl bromoacetate (4.0 equiv) were subsequently added and the reaction mixture was refluxed overnight. The reaction

mixture was cooled down to room temperature and then concentrated reduced pressure. Water was added and extracted with ethyl acetate for three times. The combined organic layers were concentrated and the crude was purified by column chromatography (cyclohexane/EtOAc 1/1) to give the pure product.

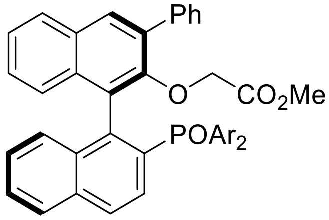

Ar = 3,5-$Me_2C_6H_3$: **S3**

515 mg, 77% (3 steps). White solid. ***R*f** 0.10 (pentane/EtOAc 1/1); **^{1}H NMR** (400 MHz, Chloroform-*d*): δ 8.12 – 8.00 (m, 2H), 7.93 (d, *J* = 8.2 Hz, 1H), 7.70 (s, 1H), 7.62 (dd, *J* = 9.0, 7.4 Hz, 3H), 7.51 (t, *J* = 7.4 Hz, 1H), 7.42 (t, *J* = 7.3 Hz, 2H), 7.39 – 7.28 (m, 3H), 7.25 – 7.18 (m, 2H), 7.13 (d, *J* = 8.6 Hz, 1H), 7.02 – 6.96 (m, 2H), 6.93 (d, *J* = 12.3 Hz, 2H), 6.80 (d, *J* = 8.4 Hz, 1H), 6.68 (s, 1H), 4.06 (d, *J* = 15.8 Hz, 1H), 3.63 (d, *J* = 15.8 Hz, 1H), 3.06 (s, 3H), 2.19 (s, 6H), 1.98 (s, 6H). **^{13}C NMR** (126 MHz, Chloroform-*d*) δ 169.0 (s), 153.2–125.1 (38 C, aromatic carbons, observed complexity due to C-P splitting), 69.9 (s), 51.3 (s), 21.4 (s), 21.3 (s). **^{31}P NMR** (162 MHz, Chloroform-*d*) δ 30.0. **HRMS** (ESI): calcd. for $[C_{45}H_{39}O_4P+Na]^+$, $[M+Na]^+$: 697.2478; found: 697.2485. **IR (ATR):** $\tilde{\nu}$ = 2951, 2208, 1766, 1600, 1557, 1457, 1377, 1291, 1128, 1078, 853, 732 cm^{-1}. **$[\alpha]_D^{20}$**: +140.3 (c = 1.0, $CHCl_3$). **Mp**: 77.0 – 77.5 °C.

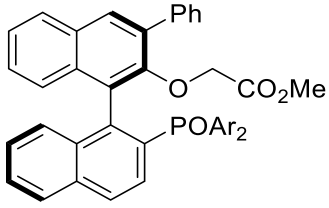

Ar = 3,5-^{t}$Bu_2C_6H_3$: **S4**

1.05 g, 98% (3 steps). White solid. ***R*f** 0.50 (pentane/EtOAc 3/1); **^{1}H NMR** (500 MHz, Chloroform-*d*): δ 7.97 – 7.91 (m, 2H), 7.88 (s, 1H), 7.75 – 7.67 (m, 3H), 7.59 – 7.50 (m, 3H), 7.49 (d, *J* = 1.8 Hz, 1H), 7.47 (d, *J* = 1.9 Hz, 1H), 7.45 – 7.38 (m, 3H), 7.38 – 7.30 (m, 3H), 7.17 (d, *J* = 1.8 Hz, 1H), 7.14 (d, *J* = 1.8 Hz, 1H), 7.08 (ddd, *J* = 8.1, 6.8, 1.2 Hz, 1H), 6.53 (ddd, *J* = 8.2, 6.7, 1.2 Hz, 1H), 6.39 (d, *J* = 8.5 Hz, 1H), 4.70 (d, *J* = 16.1 Hz, 1H), 4.02 (d, *J* = 16.1 Hz, 1H), 3.10 (s, 3H), 1.24 (s, 18H), 1.17 (s, 18H). **^{13}C NMR** (126 MHz, Chloroform-*d*) δ 169.8 (s), 153.1–124.6 (38 C, aromatic carbons, observed complexity due to C-P splitting), 69.9 (s), 51.3 (s), 35.2 (s), 35.0 (s), 31.5 (s), 31.5 (s). **^{31}P NMR** (202 MHz, Chloroform-*d*) δ 28.5. **HRMS** (ESI): calcd. for $[C_{57}H_{63}O_4P+Na]^+$, $[M+Na]^+$: 865.4356; found: 865.4358. **IR (ATR):** $\tilde{\nu}$ = 2962, 2215, 1738,

1593, 1459, 1364, 1249, 1196, 1078, 791, 733 cm^{-1}. **[α]$_D^{20}$**: +78.1 (c = 1.0, $CHCl_3$). **Mp**: 108.4 – 110.3 °C.

(*R*)-2-((2'-(bis(3,5-dimethylphenyl)phosphaneyl)-3-phenyl-[1,1'-binaphthalen]-2-yl)oxy)-acetic acid (L1) and (*R*)-2-((2'-(bis(3,5-di-tert-butylphenyl)phosphaneyl)-3-phenyl-[1,1'-bi-naphthalen]-2-yl)oxy)acetic acid (L2): synthesized according to the following procedure. **L1** is known.[1]

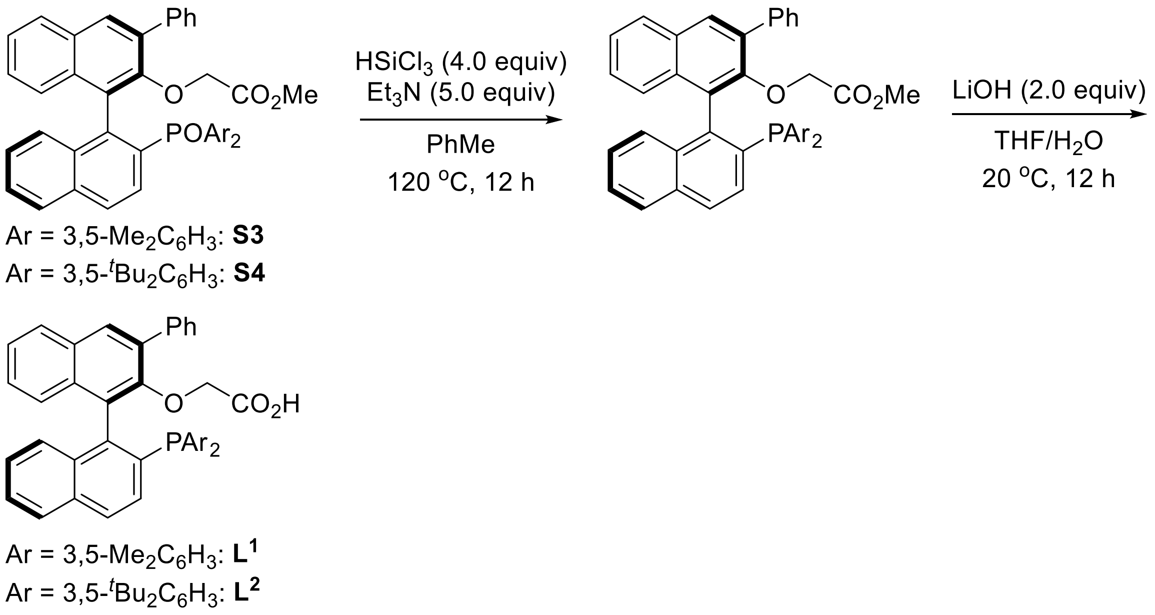

Reduction: To a flask charged with a solution of (*R*)-methyl 2-((2'-(bisaryl)-phosphoryl)-3-phenyl-[1,1'-binaphthalen]-2-yl)-oxy)acetate **S3** or **S4** (1.0 equiv) in toluene (0.1 M), triethylamine (5.0 equiv) and $HSiCl_3$ (4.0 equiv) were added under argon atmosphere. This reaction mixture was heated to 120 °C and refluxed for 12 h. Water was added to the reaction mixture after the mixture cooled down to room temperature. The mixture was extracted with ethyl acetate for three times. The combined organic layers were dried over anhydrous Na_2SO_4 and concentrated *in vacuo*. The obtained crude material was used in the next step without further purification.

Hydrolysis: LiOH (2.0 equiv) was added to a solution of compound (1.0 equiv) obtained from the last step in THF/H_2O (0.05 M, 1:1). The reaction mixture was degassed by Freeze-Pump-Thaw and stirred at room temperature. The reaction was monitored by TLC. When the reaction finished, the mixture was acidified by adding 2M HCl until the mixture reached pH = 2. The mixture was quickly extracted with ethyl acetate for three times. The combined organic phase was dried over anhydrous Na_2SO_4 and concentrated under reduced pressure. Column chromatography was used for the purification to give the pure product.

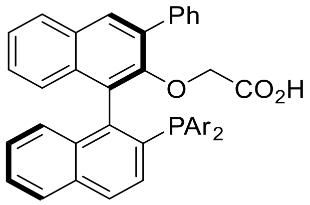

Ar = 3,5-$Me_2C_6H_3$: **L1**

430 mg, 88% (2 steps). **^{1}H NMR** (500 MHz, Chloroform-*d*): δ 7.97 (s, 1H), 7.95 (d, *J* = 8.7 Hz, 1H), 7.92 (d, *J* = 8.3 Hz, 2H), 7.55 (dd, *J* = 8.5, 2.8 Hz, 1H), 7.49 (ddt, *J* = 8.2, 3.3, 1.6 Hz, 3H), 7.47 – 7.39 (m, 3H), 7.42 – 7.35 (m, 1H), 7.29 (ddd, *J* = 8.1, 6.6, 1.3 Hz, 1H), 7.23 (d, *J* = 7.4 Hz, 1H), 7.19 (ddd, *J* = 8.3, 6.8, 1.3 Hz, 1H), 7.06 – 6.97 (m, 4H), 6.81 (s, 1H), 6.64 (d, *J* = 6.9 Hz, 2H), 3.69 (d, *J* = 15.7 Hz, 1H), 3.53 (d, *J* = 15.7 Hz, 1H), 2.28 (s, 6H), 2.15 (s, 6H).

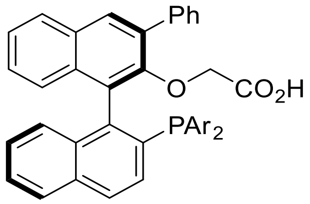

Ar = 3,5-$^t Bu_2C_6H_3$: **L2**

570 mg, 71% (2 steps). **^{1}H NMR** (500 MHz, Chloroform-*d*): δ 8.00 – 7.94 (m, 2H), 7.93 (d, *J* = 8.4 Hz, 1H), 7.87 (d, *J* = 7.7 Hz, 1H), 7.64 – 7.57 (m, 2H), 7.57 (dd, *J* = 8.6, 2.7 Hz, 1H), 7.50 (ddd, *J* = 8.1, 6.7, 1.3 Hz, 1H), 7.49 – 7.42 (m, 2H), 7.44 – 7.36 (m, 2H), 7.37 – 7.27 (m, 2H), 7.27 – 7.21 (m, 3H), 7.20 (t, *J* = 1.8 Hz, 1H), 7.00 (ddd, *J* = 8.2, 6.8, 1.2 Hz, 1H), 6.84 (d, *J* = 8.5 Hz, 1H), 6.82 (dd, *J* = 8.2, 1.9 Hz, 2H), 3.88 (d, *J* = 15.8 Hz, 1H), 3.63 (d, *J* = 15.8 Hz, 1H), 1.24 (s, 18H), 1.14 (s, 18H). **^{13}C NMR** (126 MHz, Chloroform-*d*) δ 168.1 (s), 151.1–122.2 (38 C, aromatic carbons, observed complexity due to C-P splitting), 69.1 (s), 35.1 (s), 34.9 (s), 31.5 (s), 31.5 (s). **^{31}P NMR** (202 MHz, Chloroform-*d*) δ -11.2. **HRMS** (ESI): calcd. for $[C_{56}H_{61}O_3P+Na]^+$, $[M+Na]^+$: 835.4251; found: 835.4245. **IR (ATR):** $\tilde{\nu}$ = 2961, 1786, 1579, 1459, 1362, 1248, 1196, 1077, 733 cm^{-1}. **$[\alpha]_D^{20}$**: -72.0 (c = 1.0, $CHCl_3$). **Mp**: 117.8 – 118.4 °C.

2-Bromobenzyl bromide phosphonium salt:

General Procedure 1 (GP1):[2]

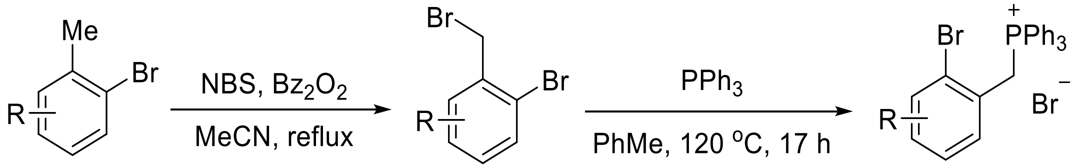

Under argon atmosphere, 2-bromotoluene (1.0 equiv), *N*-bromosuccinimide (1.1 equiv), benzoyl peroxide (0.05 equiv) and acetonitrile (0.33 M) were added to a glass flask. The mixture was heated at 80 °C and the reaction was monitored by TLC. The reaction mixture was cooled down to room temperature, filtered, and concentrated *in vacuo*. The crude residues were purified by silica gel flash column chromatography to afford the corresponding 2-bromobenzyl bromide.

A solution of 2-bromobenzyl bromide (1.0 equiv) and PPh_3 (1.01 equiv) in dry toluene (0.50 M) was stirred at 120 °C for 17 h until appearance of a white precipitate. The suspension was filtered and the solid was washed with toluene (4 x 5 mL) and hexane (3 x 5 mL) to afford 2-bromobenzyl bromide phosphonium as white powder. The obtained salt was used without further purification in the Wittig reaction.

General Procedure 2 (GP2):[3]

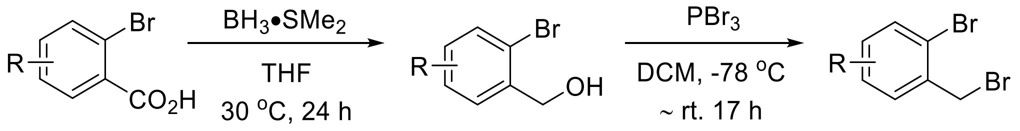

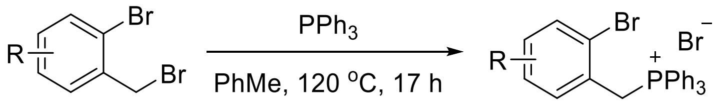

$BH_3 \cdot Me_2S$ (1.2 equiv) was slowly added to a solution of 2-bromo-3-methylbenzoic acid (1.0 equiv) in THF (0.50 M). The reaction mixture was heated at room temperature for 24 h. Then, 2 M solution of NaOH was added slowly and the resulting mixture was stirred for 30 min. The aqueous layer was extracted three times with ether. The combined organic layers were washed with water and brine, and dried over anhydrous Na_2SO_4, filtered, and concentrated *in vacuo*. The concentrated 2-bromobenzyl alcohol was directly used in the next step without purification.

To a solution of 2-bromobenzyl alcohol (1.0 equiv) in dry CH_2Cl_2 (0.10 M) at -78 °C, PBr_3 (0.5 equiv) was added in a drop wise manner. The mixture was then allowed to warm to room temperature and stirred overnight. The reaction was quenched in water and extracted with CH_2Cl_2. The combined organic layers were washed with $NaHCO_3$ (aq.), dried over anhydrous Na_2SO_4 and

evaporated under reduced pressure. 2-Bromobenzyl bromide was obtained after column chromatography.

A solution of 2-bromobenzyl bromide (1.0 equiv) and PPh_3 (1.0 equiv) in dry toluene (0.40 M) was stirred at 120 °C for 17 h until a white precipitate forms. The suspension was filtered and the solid was washed with toluene (4 x 25 mL) and hexane (3 x 55 mL) to afford 2-bromobenzyl bromide phosphonium salt as white powder. The obtained salt was used without further purification in the Wittig reaction.

1-bromo-2-(bromomethyl)-4-methoxybenzene (S5): synthesized according to the **GP1** from corresponding 2-bromotoluene (2.0 g, 9.95 mmol). Known compound.[4]

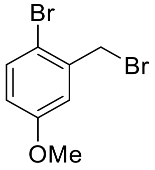

Yield: 96% (2.69 g). Yellow crystalline solid. **^{1}H NMR** (400 MHz, Chloroform-*d*) δ 7.43 (d, *J* = 8.8 Hz, 1H), 6.98 (d, *J* = 3.0 Hz, 1H), 6.72 (dd, *J* = 8.8, 3.0 Hz, 1H), 4.54 (s, 2H), 3.78 (s, 3H).

4-bromo-3-(bromomethyl)benzonitrile (S6): synthesized according to the **GP1** from corresponding 2-bromotoluene (2.02 g, 10.0 mmol). Known compound.[5]

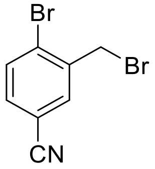

Yield: 98% (2.70 g). White flocculent solid. **^{1}H NMR** (400 MHz, Chloroform-*d*) δ 7.75 (d, *J* = 2.0 Hz, 1H), 7.72 (d, *J* = 8.3 Hz, 1H), 7.45 (dd, *J* = 8.3, 2.0 Hz, 1H), 4.57 (s, 2H).

1-bromo-2-(bromomethyl)-4-methylbenzene (S7): synthesized according to the **GP2** from corresponding commercial avalible 2-bromo-phenyl methanol (1.14 g, 5.67 mmol). Known compound.[6]

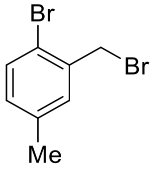

Yield: 90% (1.35 g). Colourless liquid. **^{1}H NMR** (400 MHz, Chloroform-*d*) δ 7.44 (dd, *J* = 8.1, 2.1 Hz, 1H), 7.26 (d, *J* = 2.1 Hz, 1H), 6.97 (d, *J* = 8.3 Hz, 1H), 4.57 (s, 2H), 2.30 (s, 3H).

2-bromo-1-(bromomethyl)-3-methylbenzene (S8): synthesized according to the **GP2** from corresponding 2-bromo-3-methylbenzoic acid (1.0 g, 4.65 mmol). Known compound.[7]

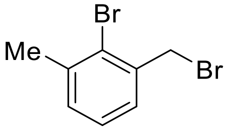

Yield: 92% (1.21 g, 2 steps). Yellow liquid. **^{1}H NMR** (400 MHz, Chloroform-*d*) δ 7.32 – 7.27 (m, 1H), 7.20 – 7.16 (m, 2H), 4.65 (s, 2H), 2.44 (s, 3H).

2-bromo-1-(bromomethyl)-3-isopropoxybenzene (S9): synthesized according to the **GP1** from corresponding 2-bromotoluene (1.77 g, 7.73 mmol).

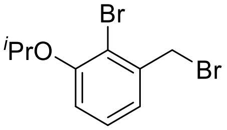

Yield: 50% (1.20 g). Colourless thick liquid. ***R*f** 0.25 (cyclohexane/EtOAc 20/1); **^{1}H NMR** (500 MHz, Chloroform-*d*): δ 7.21 (t, *J* = 7.9 Hz, 1H), 7.04 (dd, *J* = 7.7, 1.4 Hz, 1H), 6.85 (dd, *J* = 8.2, 1.4 Hz, 1H), 4.64 (s, 2H), 4.56 (hept, *J* = 6.1 Hz, 1H), 1.39 (d, *J* = 6.1 Hz, 6H). **^{13}C NMR** (126 MHz, Chloroform-*d*) δ 155.3, 138.9, 128.2, 123.3, 116.2, 115.4, 72.5, 34.3, 22.2. **HRMS** (APPI/LTQ-Orbitrap): calcd. for $[C_{10}H_{12}Br_2O]^+$, $[M]^+$: 305.9249; found: 305.9249. **IR (ATR):** $\tilde{\nu}$ = 2974, 2361, 2331, 1570, 1462, 1300, 1270, 1113, 1025, 910, 783, 641 cm^{-1}.

2-bromo-1-(bromomethyl)-3,5-dimethylbenzene (S10): synthesized according to the **GP2** from corresponding 2-bromo-3-methylbenzoic acid (8.72 g, 38.1 mmol), 8.05 g (from 8.2 g of crude phenyl methanol) was engaged in the following bromination. Known compound.[8]

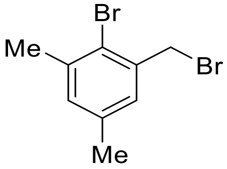

Yield: 85% (8.88 g, 2 steps). Colourless liquid. **^{1}H NMR** (400 MHz, Chloroform-*d*) δ 7.09 (s, 1H), 7.00 (s, 1H), 4.60 (s, 2H), 2.38 (s, 3H), 2.26 (s, 3H).

2-bromo-1-(bromomethyl)-5-methoxy-3-methylbenzene (S11): synthesized according to the **GP1** from corresponding 2-bromotoluene (4.30 g, 20.0 mmol). Known compound.[9]

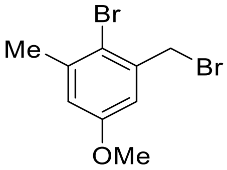

Yield: 60% (3.55 g). White solid. **^{1}H NMR** (400 MHz, Chloroform-*d*) δ 6.84 (d, *J* = 3.0 Hz, 1H), 6.76 (d, *J* = 3.1 Hz, 1H), 4.60 (s, 2H), 3.79 (s, 3H), 2.40 (s, 3H).

4-bromo-3-(bromomethyl)-5-methylbenzonitrile (S12): synthesized according to the **GP1** from corresponding 2-bromotoluene (4.20 g, 20.0 mmol).

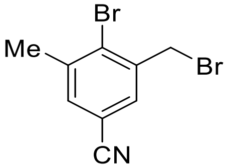

Yield: 52% (2.97 g). White solid. ***R*f** 0.20 (cyclohexane/EtOAc 10/1); **^{1}H NMR** (400 MHz, Chloroform-*d*): δ 7.58 (d, *J* = 1.9 Hz, 1H), 7.46 (d, *J* = 1.9 Hz, 1H), 4.61 (s, 2H), 2.49 (s, 3H). **^{13}C NMR** (126 MHz, Chloroform-*d*) δ 141.5, 139.3, 133.6, 132.7, 131.8, 117.8, 111.7, 32.8, 24.0. **HRMS** (APPI/LTQ-Orbitrap): calcd. for $[C_9H_7Br_2N+H]^+$, $[M+H]^+$: 287.9018; found: 287.9020. **IR (ATR):** $\tilde{\nu}$ = 2986, 2361, 2226, 1764, 1459, 1217, 1031, 884, 734 cm^{-1}. **Mp**: 107.7 – 109.4 °C.

2-bromo-1-(bromomethyl)-5-fluoro-3-methylbenzene (S13): synthesized according to the **GP1** from corresponding 2-bromotoluene (1.95 g, 9.60 mmol).

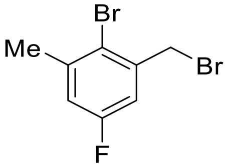

Yield: 85% (2.30 g). Colourless liquid. ***R*f** 0.20 (cyclohexane/EtOAc 20/1); **^{1}H NMR** (400 MHz, Chloroform-*d*): δ 7.04 (dd, *J* = 8.6, 3.1 Hz, 1H), 6.94 (dd, *J* = 8.8, 3.1 Hz, 1H), 4.59 (s, 2H), 2.43 (s, 3H). **^{13}C NMR** (126 MHz, Chloroform-*d*) δ 161.1 (d, $^{1}J_{C\text{-}F}$ = 247.0 Hz), 141.4 (d, $^{3}J_{C\text{-}F}$ = 7.9 Hz), 138.8 (d, $^{3}J_{C\text{-}F}$ = 8.0 Hz), 121.1 (d, $^{4}J_{C\text{-}F}$ = 3.2 Hz), 117.8 (d, $^{2}J_{C\text{-}F}$ = 22.2 Hz), 115.4 (d, $^{2}J_{C\text{-}F}$ = 23.1 Hz), 33.6, 23.9. **^{19}F NMR** (376 MHz, Chloroform-*d*) δ -115.3. **HRMS** (APPI/LTQ-Orbitrap): calcd. for $[C_8H_7Br_2F]^+$, $[M]^+$: 279.8893; found: 279.8897. **IR (ATR):** $\tilde{\nu}$ = 2980, 2362, 1599, 1460, 1312, 1142, 1029, 864, 789, 634 cm^{-1}.

1-bromo-2-(bromomethyl)naphthalene (S14): synthesized according to the **GP1** from corresponding 2-bromotoluene (11.1 g, 50.0 mmol). Known compound.[10]

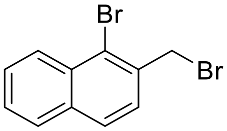

Yield: 93% (14.0 g). Colourless crystalline solid **^{1}H NMR** (400 MHz, Chloroform-*d*) δ 8.30 (dd, *J* = 8.5, 1.1 Hz, 1H), 7.78 (d, *J* = 7.8 Hz, 1H), 7.75 (d, *J* = 8.5 Hz, 1H), 7.58 (ddd, *J* = 8.5, 6.9, 1.4 Hz, 1H), 7.55 – 7.50 (m, 1H), 7.47 (d, *J* = 8.5 Hz, 1H), 4.83 (s, 2H).

5-bromo-6-(bromomethyl)quinoline (S15): synthesized according to the following procedure. Known compound.[11]

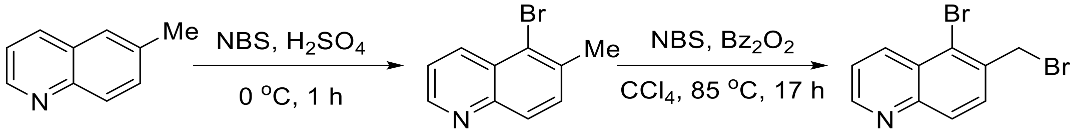

N-bromosuccinimide (2.55 g, 14.3 mmol) was added to a solution of 6-methylquinoline (2.05 g, 14.3 mmol) in 18 mL of *conc*. sulfuric acid, and the mixture was stirred at 0 °C for 1 h. It was then poured into 150 mL of ice water, and the aqueous mixture was adjusted to pH = 10 with 45% KOH solution. The precipitated salts were removed by filtration and water phase was extracted with EtOAc (3 x 100 mL). The combined organic layer was dried over anhydrous Na_2SO_4 and concentrated *in vacuo*. The crude residues were used in the next step without further purification.

Under argon atmosphere, 5-bromo-6-methylquinoline (1.30 g, 5.85 mmol), *N*-bromosuccinimide (1.15 g, 6.44 mmol), benzoyl peroxide (113 mg, 0.468 mmol) and CCl_4 (60 mL) were added to a glass flask (250 mL). The mixture was heated at 85 °C for 17 h. The reaction mixture was cooled to room temperature, filtered, and concentrated *in vacuo*. The crude residues were purified by silica gel flash column chromatography affording pure 5-bromo-6-(bromomethyl)-quinoline (**S15**) (1.35 g, 41% in 2 steps).

^{1}H NMR (400 MHz, Chloroform-*d*) δ 8.94 (dd, *J* = 4.3, 1.6 Hz, 1H), 8.65 (ddd, *J* = 8.6, 1.7, 0.9 Hz, 1H), 8.08 (dd, *J* = 8.6, 0.8 Hz, 1H), 7.78 (d, *J* = 8.7 Hz, 1H), 7.54 (dd, *J* = 8.6, 4.2 Hz, 1H), 4.85 (s, 2H).

General routes for C–H activation substrates synthesis:

General Procedure 3 (GP3): Substrates synthesis for [4]helicene and [5]helicene

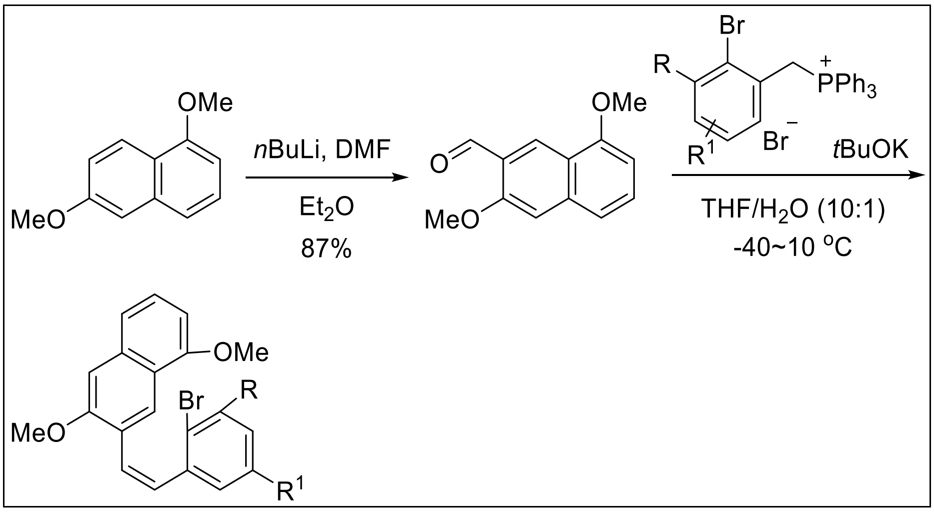

3,8-dimethoxy-2-naphthaldehyde (S16)

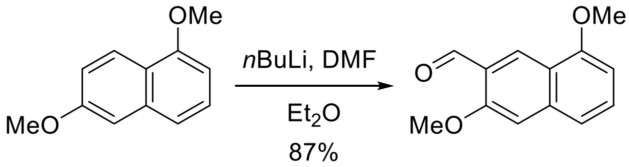

A solution of *n*BuLi in hexane (19.3 mL, 48.4 mmol) was added to a stirred solution of 1,6-dimethoxynaphthalene (7.0 g, 37.2 mmol) in dry Et_2O kept under Ar at -65 °C. The reaction mixture was then warmed up to room temperature slowly overnight. Then DMF (5.75 mL, 74.4 mmol) was added in one portion at -65 °C. The reaction was stirred at room temperature for 5 h. Quenched with excess of NH_4C1 (aq.), then extracted with EtOAc (3×100 mL). The combined organic layers were washed with brine and dried over anhydrous Na_2SO_4. Volatile material was removed under reduced pressure and the crude residue was purified by column chromatography (cyclohexane/EtOAc 2/1) to afford 3,8-dimethoxy-2-naphthaldehyde **(S16)** (7.0 g, 87%).

Yellow crystalline solid. $\boldsymbol{R_f}$ 0.15 (cyclohexane/EtOAc 10/1); **^{1}H NMR** (500 MHz, Chloroform-*d*): δ 10.54 (s, 1H), 8.80 (s, 1H), 7.48 – 7.42 (m, 1H), 7.30 (d, *J* = 8.3 Hz, 1H), 7.13 (s, 1H), 6.69 (dd, *J* = 7.7, 0.9 Hz, 1H), 4.02 (s, 3H), 3.99 (s, 3H). **^{13}C NMR** (126 MHz, Chloroform-*d*) δ 190.4, 158.3, 157.5, 139.0, 130.2, 126.7, 124.9, 120.2, 109.0, 106.2, 102.8, 55.8, 55.7. **HRMS** (ESI): calcd. for $[C_{13}H_{12}O_3+Na]^+$, $[M+Na]^+$: 239.0679; found: 239.0683. **IR (ATR):** $\tilde{\nu}$ = 2970, 2361, 1683, 1625, 1456, 1362, 1253, 1146, 1023, 787, 673 cm^{-1}. **Mp**: 137.1 – 138.4 °C.

Wittig reaction:[12]

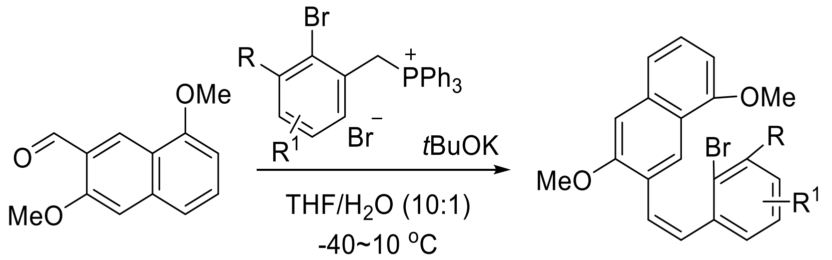

2-Bromobenzyl bromide phosphonium salt (1.1 equiv) was treated with potassium *tert*-butoxide (1.2 equiv) in THF (0.25 M)/H_2O (2.5 M) at -40 °C and stirred for 15 min. 2,3-Dimethoxy-1,4-dicarbaldehyde (1.0 equiv) was added. The mixture was stirred and warmed to 10 °C in 3 h. The resulting mixture was extracted with EtOAc and washed with brine. The crude product was purified by column chromatography (cyclohexane/EtOAc 50/1), re-precipitation or recrystallization.

(*Z*)-7-(2-bromo-3-methylstyryl)-1,6-dimethoxynaphthalene (1a): synthesized according to the **GP3** from **S16** (0.398 g, 1.80 mmol).

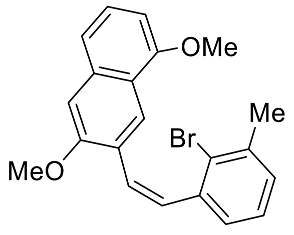

Recrystallized in Et_2O/DCM. **Yield**: 58% (397 mg). White crystal. ***R*f** 0.30 (cyclohexane/EtOAc 20/1); **^{1}H NMR** (500 MHz, Chloroform-*d*): δ 7.93 (s, 1H), 7.32 – 7.22 (m, 2H), 7.04 (d, *J* = 7.4 Hz, 1H), 7.02 (s, 1H), 6.96 – 6.90 (m, 2H), 6.89 – 6.82 (m, 2H), 6.59 (dd, *J* = 7.0, 1.6 Hz, 1H), 3.82 (s, 3H), 3.81 (s, 3H), 2.48 (s, 3H). **^{13}C NMR** (126 MHz, Chloroform-*d*) δ 156.3, 155.9, 138.9, 138.3, 135.4, 131.6, 129.2, 128.0, 126.7, 126.6, 126.6, 126.5, 126.2, 124.1, 120.5, 118.9, 105.1, 102.4, 55.6, 55.4, 23.9. **HRMS** (ESI): calcd. for $[C_{21}H_{19}BrO_2]^+$, $[M]^+$: 382.0563; found: 382.0556. **IR (ATR):** $\tilde{\nu}$ = 2955, 1629, 1575, 1459, 1368, 1231, 1138, 1024, 837, 791, 738 cm^{-1}. **Mp**: 152.3 – 154.3 °C.

(*Z*)-7-(2-bromo-3-isopropoxystyryl)-1,6-dimethoxynaphthalene (1b): synthesized according to the **GP3** from **S16** (0.50 g, 2.31 mmol).

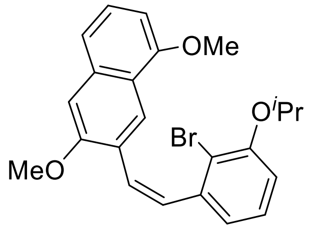

Recrystallized in cyclohexane/Et_2O. **Yield**: 60% (590 mg). White crystalline solid. ***R*f** 0.25 (cyclohexane/EtOAc 20/1); **^{1}H NMR** (500 MHz, Chloroform-*d*): δ 7.91 (s, 1H), 7.33 – 7.21 (m, 2H), 7.02 (s, 1H), 6.91 (d, *J* = 12.1 Hz, 1H), 6.87 (t, J = 7.9 Hz, 1H), 6.81 (d, *J* = 12.1 Hz,

1H), 6.73 (dd, J = 8.2, 1.5 Hz, 1H), 6.70 (dd, J = 7.6, 1.5 Hz, 1H), 6.58 (dd, J = 7.0, 1.6 Hz, 1H), 4.56 (hept, J = 6.1 Hz, 1H), 3.82 (s, 3H), 3.81 (s, 3H), 1.41 (d, J = 6.1 Hz, 6H). **^{13}C NMR** (126 MHz, Chloroform-*d*) δ 156.3, 155.9, 154.9, 140.3, 135.4, 131.1, 127.4, 126.9, 126.7, 126.3, 124.2, 123.2, 120.5, 119.0, 115.9, 114.7, 105.1, 102.4, 72.7, 55.6, 55.5, 22.3. **HRMS** (ESI): calcd. for $[C_{23}H_{23}BrO_3+Na]^+$, $[M+Na]^+$: 449.0723; found: 449.0722. **IR (ATR):** $\tilde{\nu}$ = 2978, 1628, 1576, 1459, 1368, 1265, 1138, 1025, 838, 789, 740 cm^{-1}. **Mp**: 143.6 – 145.8 °C.

(*Z*)-7-(2-bromo-5-fluoro-3-methylstyryl)-1,6-dimethoxynaphthalene (1c): synthesized according to the **GP3** from **S16** (0.50 g, 2.31 mmol).

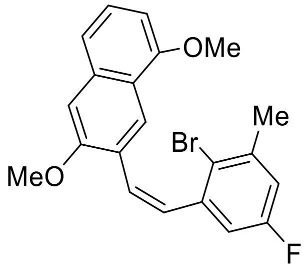

Recrystallized in Et_2O/DCM. **Yield**: 73% (675 mg). Light green crystal. ***R*f** 0.28 (cyclohexane/EtOAc 20/1); **^{1}H NMR** (400 MHz, Chloroform-*d*): δ 7.90 (s, 1H), 7.32 – 7.21 (m, 2H), 7.02 (s, 1H), 6.93 (d, J = 12.1 Hz, 1H), 6.80 – 6.77 (m, 2H), 6.75 (d, J = 12.0 Hz, 1H), 6.66 (dd, J = 9.3, 3.1 Hz, 1H), 6.59 (dd, J = 7.1, 1.5 Hz, 1H), 3.82 (s, 3H), 3.81 (s, 3H), 2.44 (s, 3H). **^{13}C NMR** (126 MHz, Chloroform-*d*) δ 161.2 (d, $^1J_{C\text{-F}}$ = 245.1 Hz), 156.1, 155.9, 140.5 (d, $^3J_{C\text{-F}}$ = 8.4 Hz), 140.2 (d, $^3J_{C\text{-F}}$ = 8.2 Hz), 135.5, 130.5 (d, $^4J_{C\text{-F}}$ = 2.0 Hz), 127.7, 126.9, 125.6, 124.1, 120.8 (d, $^4J_{C\text{-F}}$ = 3.0 Hz), 120.5, 119.0, 116.2 (d, $^2J_{C\text{-F}}$ = 22.8 Hz), 114.6 (d, $^2J_{C\text{-F}}$ = 22.9 Hz), 105.3, 102.5, 55.6, 55.4, 24.0 (d, $^4J_{C\text{-F}}$ = 1.6 Hz). **^{19}F NMR** (376 MHz, Chloroform-*d*) δ -116.8. **HRMS** (ESI): calcd. for $[C_{21}H_{18}BrFO_2]^+$, $[M]^+$: 400.0469; found: 400.0466. **IR (ATR):** $\tilde{\nu}$ = 2958, 2361, 1629, 1577, 1459, 1368, 1231, 1140, 1025, 866, 838, 783, 675 cm^{-1}. **Mp**: 150.5 – 152.5 °C.

(*Z*)-7-(2-bromo-3,5-dimethylstyryl)-1,6-dimethoxynaphthalene (1d): synthesized according to the **GP3** from **S16** (0.50 g, 2.31 mmol).

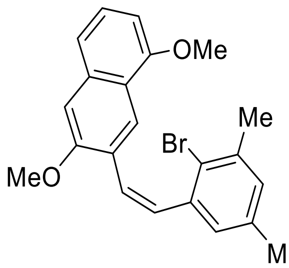

Recrystallized in Et_2O/DCM. **Yield**: 62% (570 mg). White crystal. ***R*f** 0.30 (cyclohexane/EtOAc 20/1); **^{1}H NMR** (500 MHz, Chloroform-*d*): δ 7.97 (s, 1H), 7.31 – 7.22 (m, 2H), 7.02 (s, 1H), 6.90 (d, J = 12.3 Hz, 1H), 6.88 (d, J = 2.2 Hz, 1H), 6.79 (d, *j* = 11.9 Hz, 2H), 6.59 (dd, J = 6.9, 1.6 Hz, 1H), 3.82 (s, 6H), 2.42 (s, 3H), 1.98 (s, 3H). **^{13}C NMR** (126 MHz, Chloroform-*d*) δ 156.3, 155.9, 138.7, 138.0, 136.3, 135.4, 131.5, 130.2, 128.5, 126.6, 126.2, 124.0, 123.1, 120.5, 119.0, 105.0, 102.4, 55.7, 55.4, 23.7, 20.8. **HRMS** (ESI/QTOF):

calcd. for $[C_{22}H_{21}BrO_2]^+$, $[M]^+$: 396.0719; found: 396.0713. **IR (ATR):** $\tilde{\nu}$ = 2955, 2361, 1628, 1576, 1459, 1368, 1270, 1231, 1138, 1024, 838, 744, 678 cm^{-1}. **Mp**: 134.7 – 136.8 °C.

(*Z*)-7-(2-bromo-5-methoxy-3-methylstyryl)-1,6-dimethoxynaphthalene (1e): synthesized according to the **GP3** from **S16** (0.50 g, 2.31 mmol).

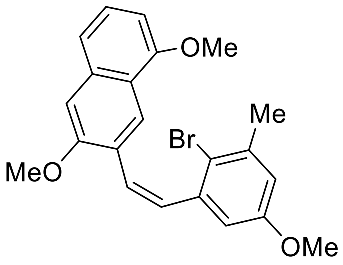

Recrystallized in Et_2O/DCM. **Yield**: 56% (533 mg). White crystal. ***R*f** 0.15 (cyclohexane/EtOAc 20/1); **^{1}H NMR** (500 MHz, Chloroform-*d*): δ 8.00 (s, 1H), 7.31 – 7.22 (m, 2H), 7.02 (s, 1H), 6.92 (d, *J* = 12.1 Hz, 1H), 6.83 (d, *J* = 12.2 Hz, 1H), 6.64 (d, *J* = 3.1 Hz, 1H), 6.59 (dd, *J* = 7.2, 1.4 Hz, 1H), 6.54 (d, *J* = 3.0 Hz, 1H), 3.83 (s, 3H), 3.83 (s, 3H), 3.34 (s, 3H), 2.43 (s, 3H). **^{13}C NMR** (126 MHz, Chloroform-*d*) δ 157.9, 156.3, 155.9, 139.3, 139.2, 135.4, 131.5, 126.8, 126.7, 126.1, 124.1, 120.5, 119.0, 117.4, 116.3, 112.5, 105.1, 102.4, 55.6, 55.4, 55.3, 24.0. **HRMS** (ESI): calcd. for $[C_{22}H_{21}BrO_3]^+$, $[M]^+$: 412.0669; found: 412.0663. **IR (ATR):** $\tilde{\nu}$ = 2957, 2362, 1628, 1577, 1459, 1367, 1310, 1231, 1134, 1023, 838, 783, 679 cm^{-1}. **Mp**: 137.7 – 139.3 °C.

(*Z*)-4-bromo-3-(2-(3,8-dimethoxynaphthalen-2-yl)vinyl)-5-methylbenzonitrile (1f): synthesized according to the **GP3** from **S16** (0.50 g, 2.31 mmol).

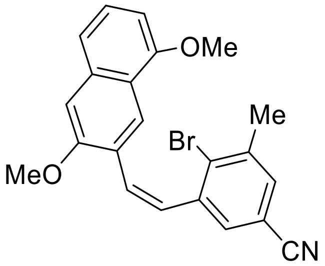

Recrystallized in Et_2O/DCM. **Yield**: 67% (630 mg). White crystal. ***R*f** 0.10 (cyclohexane/EtOAc 20/1); **^{1}H NMR** (500 MHz, Chloroform-*d*): δ 7.83 (s, 1H), 7.32 – 7.28 (m, 2H), 7.27 – 7.21 (m, 2H), 7.03 (s, 1H), 7.01 (dd, *J* = 12.1, 0.9 Hz, 1H), 6.71 (d, *J* = 12.0 Hz, 1H), 6.61 (dd, *J* = 7.4, 1.1 Hz, 1H), 3.84 (s, 3H), 3.83 (s, 3H), 2.49 (s, 3H). **^{13}C NMR** (101 MHz, Chloroform-*d*) δ 155.9, 155.8, 140.8, 140.2, 135.7, 132.2, 131.6, 131.3, 129.3, 128.9, 127.2, 125.0, 124.1, 120.4, 119.1, 118.4, 110.7, 105.5, 102.6, 55.6, 55.4, 23.9. **HRMS** (ESI): calcd. for $[C_{22}H_{18}BrNO_2+Na]^+$, $[M+Na]^+$: 430.0413; found: 430.0410. **IR (ATR):** $\tilde{\nu}$ = 2960, 2362, 2228, 1628, 1576, 1459, 1368, 1233, 1138, 1024, 910, 840, 682 cm^{-1}. **Mp**: 177.9 – 179.9 °C.

(*Z*)-4-bromo-3-(2-(3,8-dimethoxynaphthalen-2-yl)vinyl)-5-methylbenzonitrile (1g): synthesized according to literature reported procedure followed by Wittig reaction (corresponding aldehyde: 0.50 g, 2.94 mmol).[13,14]

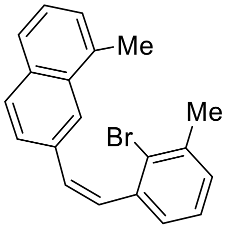

Yield: 50% (493 mg). White solid. ***R*f** 0.10 (cyclohexane); **^{1}H NMR** (600 MHz, Chloroform-d) δ 7.81 (s, 1H), 7.61 (d, *J* = 8.4 Hz, 2H), 7.32 (t, *J* = 7.6 Hz, 1H), 7.24 (dd, *J* = 10.6, 3.6 Hz, 2H), 7.14 (d, *J* = 7.4 Hz, 1H), 7.05 (d, *J* = 7.7 Hz, 1H), 6.99 (q, *J* = 7.5, 6.5 Hz, 1H), 6.87 (d, *J* = 12.0 Hz, 1H), 6.76 (d, *J* = 12.0 Hz, 1H), 2.50 (s, 6H). **^{13}C NMR** (151 MHz, Chloroform-*d*) δ 138.9, 138.8, 134.5, 133.9, 132.8, 132.6, 131.3, 130.9, 129.7, 128.6, 128.2, 126.9, 126.9, 126.8, 126.5, 126.2, 125.9, 125.0, 23.9, 19.3. **HRMS** (ESI) (0.2 mM $AgNO_3$): calcd. for $[C_{20}H_{17}Br+Ag]^+$, $[M+Ag]^+$: 442.9559; found: 442.9552. **IR (ATR):** $\tilde{\nu}$ = 2967, 1578, 1446, 1382, 1025, 886, 836, 746, 647 cm^{-1}. **Mp**: 104.8 – 106.5 °C.

(*Z*)-7-(2-(1-bromonaphthalen-2-yl)vinyl)-1,6-dimethoxynaphthalene (1q): synthesized according to the **GP3** from **S16** (0.50 g, 2.31 mmol).

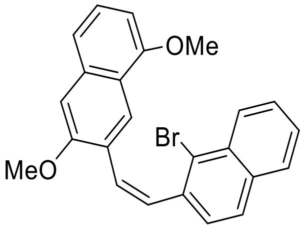

Recrystallized in Et_2O/DCM. **Yield**: 49% (470 mg). Yellow crystal. ***R*f** 0.28 (cyclohexane/EtOAc 20/1); **^{1}H NMR** (500 MHz, Chloroform-*d*): δ 8.39 (d, *J* = 8.8 Hz, 1H), 7.91 (s, 1H), 7.69 (d, *J* = 8.0 Hz, 1H), 7.58 (ddd, *J* = 8.4, 6.8, 1.3 Hz, 1H), 7.47 (ddd, *J* = 8.1, 6.9, 1.2 Hz, 1H), 7.42 (d, *J* = 8.5 Hz, 1H), 7.31 – 7.23 (m, 2H), 7.18 (d, *J* = 8.5 Hz, 1H), 7.09 – 7.00 (m, 3H), 6.54 (dd, *J* = 6.6, 2.1 Hz, 1H), 3.82 (s, 3H), 3.62 (s, 3H). **^{13}C NMR** (126 MHz, Chloroform-*d*) δ 156.3, 155.9, 136.7, 135.5, 133.7, 132.7, 131.4, 128.1, 127.9, 127.4, 127.3, 127.2, 127.1, 126.9, 126.4, 126.2, 124.5, 124.0, 120.5, 118.9, 105.1, 102.4, 55.5, 55.5. **HRMS** (ESI): calcd. for $[C_{24}H_{19}BrO_2+Na]^+$, $[M+Na]^+$: 441.0461; found: 441.0460. **IR (ATR):** $\tilde{\nu}$ = 2958, 2361, 1628, 1575, 1458, 1368, 1232, 1137, 1024, 970, 910, 813, 744, 651 cm^{-1}. **Mp**: 180.7 – 182.5 °C.

General Procedure 4 (GP4): Substrates synthesis for [5]helicene and [6]helicene

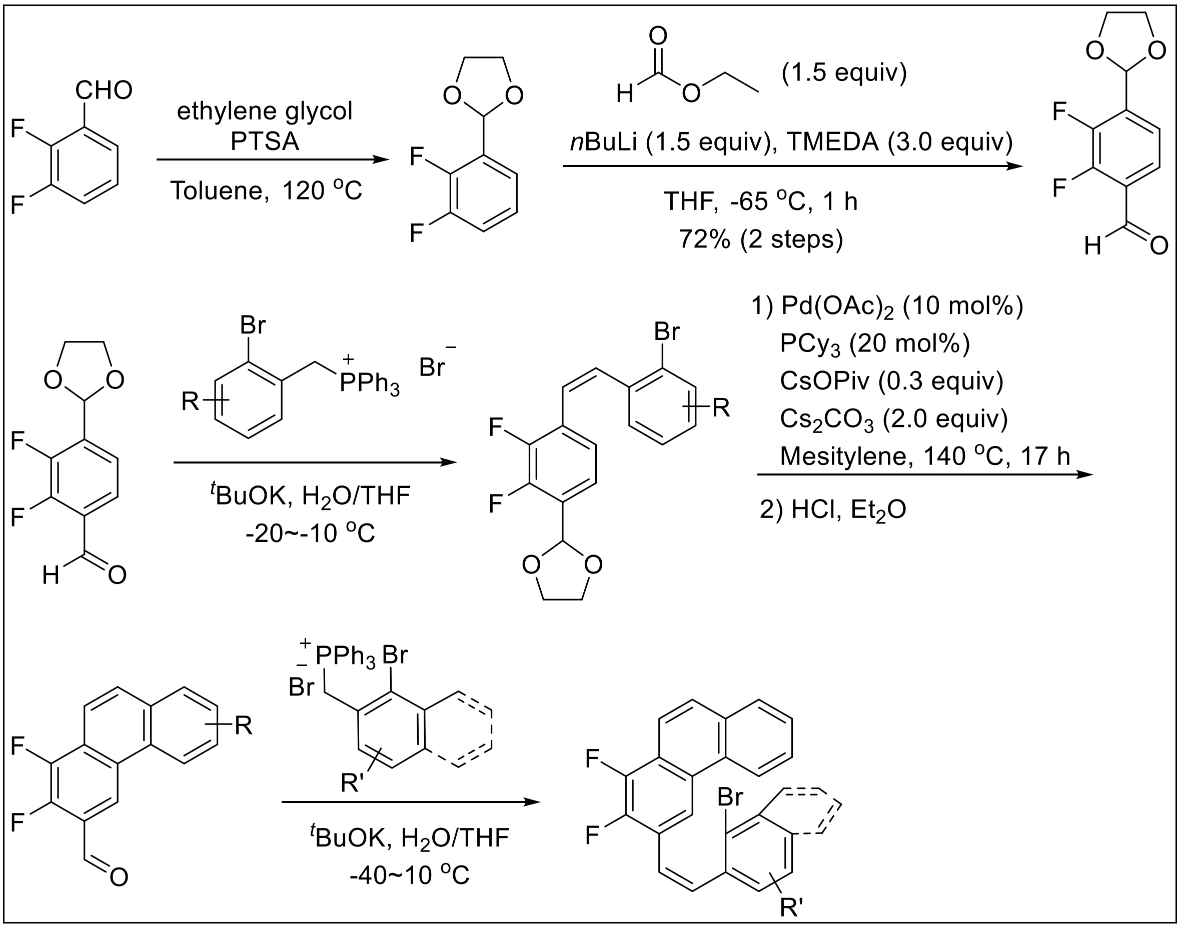

4-(1,3-dioxolan-2-yl)-2,3-difluorobenzaldehyde (S17): synthesized according to the following procedure.

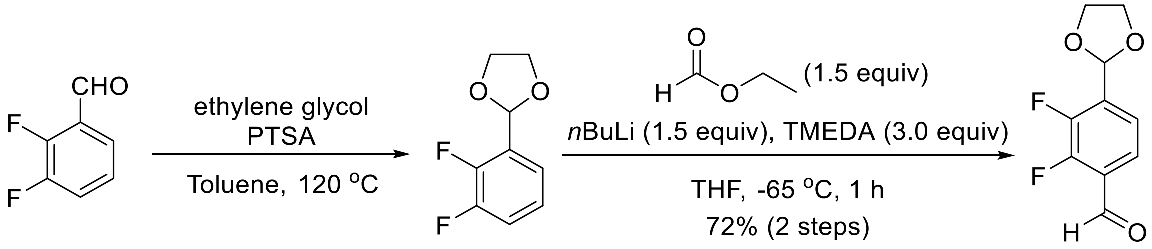

To 2,3-difluorobenzaldehyde (20.0 g, 141 mmol) in 300 mL of anhydrous toluene was added *p*-toluenesulfonic acid (2.43 g, 14.1 mmol) and ethylene glycol (43.8 g, 705 mmol). The reaction mixture was refluxed for 24 h using Dean–Stark apparatus. Upon completion, the reaction mixture was cooled to room temperature and was washed three times with saturated $NaHCO_3$ solution (300 mL) and brine (100 mL), dried over anhydrous Na_2SO_4, filtered, and evaporated to provide crude products. The obtained colorless liquid 2-(2,3-difluorophenyl)-1,3-dioxolane (equiv.) was used in the next step without purification.

The solution of 2-(2,3-difluorophenyl)-1,3-dioxolane (12.0 g, 64.5 mmol) in diethyl ether (80 mL) was cooled under -65 °C. *n*BuLi 2.5 M in hexane (38.7 mL, 96.8 mmol) was added slowly over 20 min. The lithiation mixture was stirred under -65 °C for 2 h. Then ethyl formate (7.78

mL, 96.8 mmol) was added to the mixture and the mixture was kept under -65 °C for at most 1 h, followed by quenching with water. The organic layer was separated and the aqueous layer was extracted with 3 × 50 mL diethyl ether. The extract was dried over anhydrous Na_2SO_4, volatile material was removed under reduced pressure and the crude residue was purified by column chromatography (cyclohexane/EtOAc 10/1) to afford 10 g 4-(1,3-dioxolan-2-yl)-2,3-difluorobenzaldehyde (**S17**) with 72% yield in 2 steps.

White crystalline solid. ***R*f** 0.30 (cyclohexane/EtOAc 5/1); **^{1}H NMR** (400 MHz, Chloroform-*d*): δ 10.34 (s, 1H), 7.64 (ddd, *J* = 7.9, 5.8, 1.8 Hz, 1H), 7.41 (ddd, *J* = 7.8, 5.6, 1.7 Hz, 1H), 6.12 (s, 1H), 4.18 – 4.12 (m, 2H), 4.12 – 4.05 (m, 2H). **^{13}C NMR** (126 MHz, Chloroform-*d*) δ 186.0 (dd, ^3J = 6.2 Hz, ^4J = 3.0 Hz), 152.7 (dd, ^1J = 261.5 Hz, ^2J = 13.2 Hz), 149.3 (dd, ^1J = 255.6 Hz, ^2J = 11.9 Hz), 134.0 (d, ^3J = 9.5 Hz), 126.2 (d, ^3J = 5.6 Hz), 123.0 (d, ^4J = 4.1 Hz), 122.6 (dd, ^3J = 4.4, ^4J = 2.6 Hz), 98.3 (t, ^3J = 3.0 Hz), 65.8, 65.8. **^{19}F NMR** (376 MHz, Chloroform-*d*) δ -142.5 (d, *J* = 20.1 Hz), -147.0 (d, *J* = 20.2 Hz). **HRMS** (ESI): calcd. for $[C_{10}H_8F_2O_3+Na]^+$, $[M+Na]^+$: 237.0334; found: 237.0331. **IR (ATR):** $\tilde{\nu}$ = 2963, 1698, 1469, 1401, 1256, 1114, 1017, 971, 837, 774 cm^{-1}. **Mp**: 79.4 – 80.6 °C.

Wittig reaction:[12]

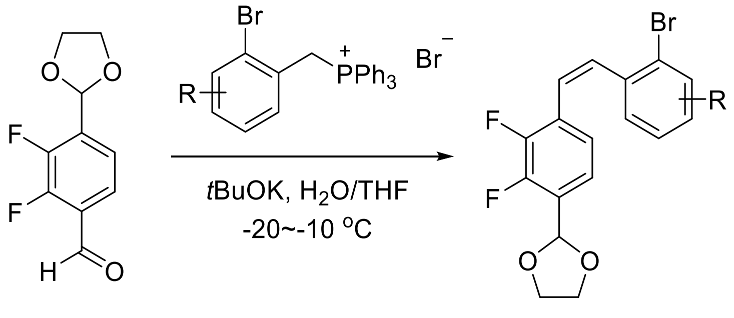

2-Bromobenzyl bromide phosphonium salt (1.1 equiv) was treated with potassium *tert*-butoxide (1.2 equiv) in THF (0.25 M)/H_2O (2.5 M) at -20 °C and stirred for 15 min. 4-(1,3-dioxolan-2-yl)-2,3-difluorobenzaldehyde (**S17**) (1.0 equiv) was added. The mixture was then warmed slowly to -10 °C in 1 h, and kept at -10 °C. The reaction was monitored by TLC. The resulting mixture was quenched with water and extracted with EtOAc. The extract was dried over Na_2SO_4, volatile material was removed under reduced pressure and the crude residue was purified by column chromatography (cyclohexane/EtOAc).

(*Z*)-2-(4-(2-bromostyryl)-2,3-difluorophenyl)-1,3-dioxolane (S18): synthesized from **S17** (2.92 g, 13.6 mmol).

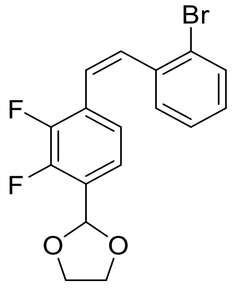

Yield: 92% (4.60 g, only *Z*). White solid. ***R*f** 0.28 (cyclohexane/EtOAc 5/1); **^{1}H NMR** (400 MHz, Chloroform-*d*) δ 7.64 – 7.55 (m, 1H), 7.14 – 7.05 (m, 3H), 6.99 (ddd, *J* = 8.1, 6.2, 1.8 Hz, 1H), 6.83 (d, *J* = 12.1 Hz, 1H), 6.76 (ddd, *J* = 8.1, 6.2, 1.8 Hz, 1H), 6.74 – 6.68 (m, 1H), 6.01 (s, 1H), 4.14 – 4.07 (m, 2H), 4.07 – 4.00 (m, 2H). **^{13}C NMR** (126 MHz, Chloroform-*d*) δ 149.4 (dd, ^1J = 251.8 Hz, $^2J_{C\text{-F}}$ = 13.8 Hz), 148.5 (dd, ^1J = 250.6 Hz, $^2J_{C\text{-F}}$ = 12.8 Hz), 137.2, 133.2 (d, $^4J_{C\text{-F}}$ = 1.7 Hz), 132.9, 130.6, 129.4, 127.4, 127.3 (d, $^2J_{C\text{-F}}$ = 10.9 Hz), 126.3 (d, $^2J_{C\text{-F}}$ = 9.3 Hz), 124.7 (dd, $^3J_{C\text{-F}}$ = 4.2 Hz, $^4J_{C\text{-F}}$ = 2.4 Hz), 123.9, 122.7 (t, $^4J_{C\text{-F}}$ = 3.2 Hz), 121.5 (t, $^3J_{C\text{-F}}$ = 3.5 Hz), 98.7 (t, $^4J_{C\text{-F}}$ = 3.2 Hz), 65.7. **^{19}F NMR** (376 MHz, Chloroform-*d*) δ -140.5 (d, *J* = 20.1 Hz), -144.2 (d, *J* = 20.3 Hz). **HRMS** (ESI): calcd. for $[C_{17}H_{13}BrF_2O_2+Na]^+$, $[M+Na]^+$: 388.9959; found: 388.9964. **IR (ATR):** $\tilde{\nu}$ = 2994, 2878, 1636, 1451, 13677, 1299, 1260, 1111, 1011, 942, 853, 754, 726 cm^{-1}. **Mp**: 77.4 – 79.4 °C.

<u>(*Z*)-2-(4-(2-bromo-5-methoxystyryl)-2,3-difluorophenyl)-1,3-dioxolane (S19):</u> synthesized from **S17** (0.50 g, 2.33 mmol).

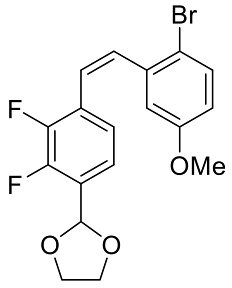

Yield: 93% (860 mg, *Z/E* 10:1). Yellow solid. ***R*f** 0.10 (cyclohexane/EtOAc 10/1); analysis for *Z*-(**S19**): **^{1}H NMR** (400 MHz, Chloroform-*d*): δ 7.46 (d, *J* = 8.8 Hz, 1H), 7.02 (ddd, *J* = 8.2, 6.3, 1.8 Hz, 1H), 6.87 – 6.78 (m, 2H), 6.75 – 6.65 (m, 2H), 6.62 (d, *J* = 3.1 Hz, 1H), 6.02 (s, 1H), 4.17 – 4.07 (m, 2H), 4.07 – 3.95 (m, 2H), 3.56 (s, 3H). **^{19}F NMR** (376 MHz, Chloroform-*d*) δ -140.5 (d, *J* = 20.1 Hz), -144.2 (d, *J* = 20.3 Hz).

<u>(*Z*)-3-(4-(1,3-dioxolan-2-yl)-2,3-difluorostyryl)-4-bromobenzonitrile (S20):</u> synthesized from **S17** (1.0 g, 4.67 mmol).

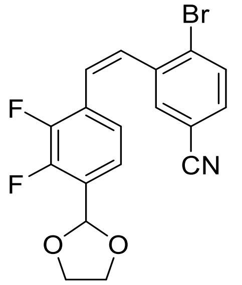

Yield: 97% (1.8 g, *Z/E* 5:1). White crystalline solid. ***R*f** 0.30 (cyclohexane/EtOAc 5/1); analysis for *Z*-(**S20**): **^{1}H NMR** (400 MHz, Chloroform-*d*): δ 7.73 (d, *J* = 8.3 Hz, 1H), 7.42 – 7.32 (m, 2H), 7.06 (ddd, *J* = 8.1, 6.1, 1.8 Hz, 1H), 6.82 (d, *J* = 12.1 Hz, 1H), 6.74 (d, *J* = 12.1 Hz, 1H), 6.69 (ddd, *J* = 8.2, 6.2, 1.8 Hz, 1H), 6.05 (s, 1H), 4.15 – 4.06 (m, 2H), 4.08 – 3.91 (m, 2H). **^{19}F NMR** (376 MHz, Chloroform-*d*) δ -139.6 (d, *J* = 20.3 Hz), -143.0 (d, *J* = 20.3 Hz).

(*Z*)-2-(4-(2-bromo-5-fluorostyryl)-2,3-difluorophenyl)-1,3-dioxolane (S21): synthesized from **S17** (1.0 g, 4.67 mmol).

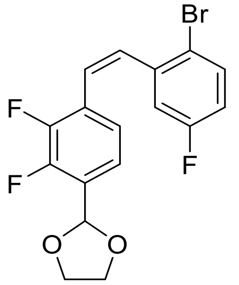

Yield: 98% (1.78 g, *Z/E* 10:1). White solid. ***R*f** 0.15 (cyclohexane/EtOAc 10/1); analysis for *Z*-(**S21**): **^{1}H NMR** (500 MHz, Chloroform-*d*): δ 7.54 (dd, *J* = 8.7, 5.3 Hz, 1H), 7.04 (ddd, *J* = 8.2, 6.2, 1.8 Hz, 1H), 6.88 – 6.70 (m, 5H), 6.03 (s, 1H), 4.17 – 4.06 (m, 2H), 4.09 – 3.97 (m, 2H). **^{13}C NMR** (126 MHz, Chloroform-*d*) δ 161.7 (d, $^{1}J_{C\text{-F}}$ = 247.4 Hz), 149.5 (dd, $^{1}J_{C\text{-F}}$ = 252.1 Hz, $^{2}J_{C\text{-F}}$ = 13.8 Hz), 148.5 (dd, $^{1}J_{C\text{-F}}$ = 251.1 Hz, $^{2}J_{C\text{-F}}$ = 12.8 Hz), 139.0 (d, $^{3}J_{C\text{-F}}$ = 8.1 Hz), 134.2 (d, $^{3}J_{C\text{-F}}$ = 8.1 Hz), 132.2 (t, $^{4}J_{C\text{-F}}$ = 1.8 Hz), 126.9 (d, $^{3}J_{C\text{-F}}$ = 9.5 Hz), 126.7 (d, $^{3}J_{C\text{-F}}$ = 10.9 Hz), 124.5 (dd, $^{4}J_{C\text{-F}}$ = 4.2, 2.3 Hz), 123.7 (t, $^{4}J_{C\text{-F}}$ = 3.1 Hz), 121.7 (t, $^{4}J_{C\text{-F}}$ = 3.7 Hz), 118.2 (d, $^{4}J_{C\text{-F}}$ = 3.2 Hz), 117.4 (d, $^{2}J_{C\text{-F}}$ = 23.3 Hz), 116.7 (d, $^{2}J_{C\text{-F}}$ = 22.7 Hz), 98.7 (t, $^{4}J_{C\text{-F}}$ = 3.1 Hz), 65.67. **^{19}F NMR** (376 MHz, Chloroform-*d*) δ -114.4, -140.1 (d, *J* = 20.3 Hz), -143.7 (d, *J* = 20.2 Hz). **HRMS** (APPI/LTQ-Orbitrap): calcd. for $[C_{17}H_{12}BrF_3O_2]^+$, $[M]^+$: 383.9973; found: 383.9977. **IR (ATR):** $\tilde{\nu}$ = 2891, 2362, 1575, 1459, 1398, 1272, 1221, 1108, 1030, 945, 883, 813, 633 cm^{-1}. **Mp**: 67.0 – 69.0 °C.

(*Z*)-2-(4-(2-bromo-5-methylstyryl)-2,3-difluorophenyl)-1,3-dioxolane (S22): synthesized from **S17** (1.0 g, 4.67 mmol).

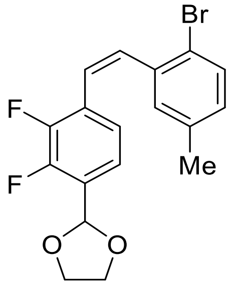

Yield: 97% (1.72 g, *Z/E* 8:1). White crystalline solid. ***R*f** 0.10 (cyclohexane/EtOAc 10/1); analysis for *Z*-(**S22**): **^{1}H NMR** (400 MHz, Chloroform-*d*): δ 7.46 (d, *J* = 8.9 Hz, 1H), 6.98 (ddd, *J* = 8.2, 6.3, 1.8 Hz, 1H), 6.92 (d, *J* = 6.7 Hz, 2H), 6.79 (d, *J* = 12.3 Hz, 1H), 6.79 – 6.72 (m, 1H), 6.70 (d, *J* = 12.1 Hz, 1H), 6.02 (s, 1H), 4.24 – 4.05 (m, 2H), 4.07 – 3.92 (m, 2H), 2.13 (s, 3H). **^{13}C NMR** (126 MHz, Chloroform-*d*) δ 149.2 (dd, $^{1}J_{C\text{-F}}$ = 251.6 Hz, $^{2}J_{C\text{-F}}$ = 13.9 Hz), 148.3 (dd, $^{1}J_{C\text{-F}}$ = 250.5 Hz, $^{2}J_{C\text{-F}}$ = 12.7 Hz), 137.1, 136.7, 133.0 (d, $^{4}J_{C\text{-F}}$ = 1.8 Hz), 132.4, 130.8, 130.0, 127.1 (d, $^{3}J_{C\text{-F}}$ = 10.7 Hz), 126.0 (d, $^{3}J_{C\text{-F}}$ = 9.3 Hz), 124.3 (dd, $^{4}J_{C\text{-F}}$ = 4.1, 2.3 Hz), 122.1 (t, $^{4}J_{C\text{-F}}$ = 3.4 Hz), 121.1 (t, $^{4}J_{C\text{-F}}$ = 3.9 Hz), 120.2, 98.6 (t, $^{4}J_{C\text{-F}}$ = 3.2 Hz), 65.4, 20.7. **^{19}F NMR** (376 MHz, Chloroform-*d*) δ -140.8 (d, *J* = 20.2 Hz), -144.3 (d, *J* = 20.2 Hz). **HRMS** (APPI/LTQ-Orbitrap): calcd. for $[C_{18}H_{15}BrF_2O_2]^+$, $[M]^+$: 380.0218; found: 380.0221. **IR**

(ATR): $\tilde{\nu}$ = 2889, 2361, 1635, 1457, 1397, 1270, 1220, 1107, 1025, 972, 945, 807, 629 cm^{-1}. **Mp**: 76.5 – 78.3 °C.

(*Z*)-2-(4-(2-bromo-4-fluorostyryl)-2,3-difluorophenyl)-1,3-dioxolane (S23): synthesized from **S17** (1.0 g, 4.67 mmol).

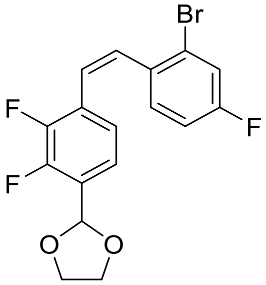

Yield: 98% (1.76 g, only *Z*). White crystalline solid. ***R*f** 0.15 (cyclohexane/EtOAc 10/1); **^{1}H NMR** (500 MHz, Chloroform-*d*): δ 7.35 (dd, *J* = 8.2, 2.6 Hz, 1H), 7.09 – 6.98 (m, 2H), 6.82 (td, *J* = 8.3, 2.5 Hz, 1H), 6.77 (d, *J* = 12.0 Hz, 1H), 6.79 – 6.71 (m, 1H), 6.70 (d, *J* = 12.0 Hz, 1H), 6.02 (s, 1H), 4.17 – 4.07 (m, 2H), 4.09 – 3.98 (m, 2H). **^{13}C NMR** (126 MHz, Chloroform-*d*) δ 161.9 (d, $^{1}J_{C\text{-F}}$ = 251.8 Hz), 149.5 (dd, $^{1}J_{C\text{-F}}$ = 252.0 Hz, $^{2}J_{C\text{-F}}$ = 13.5 Hz), 148.5 (dd, $^{1}J_{C\text{-F}}$ = 250.8 Hz, $^{2}J_{C\text{-F}}$ = 12.7 Hz), 133.3 (d, $^{3}J_{C\text{-F}}$ = 3.6 Hz), 132.2 (d, $^{4}J_{C\text{-F}}$ = 1.8 Hz), 131.6 (d, $^{3}J_{C\text{-F}}$ = 8.4 Hz), 127.1 (d, $^{3}J_{C\text{-F}}$ = 10.9 Hz), 126.5 (d, $^{3}J_{C\text{-F}}$ = 9.4 Hz), 124.6 (dd, $^{4}J_{C\text{-F}}$ = 4.2, 2.3 Hz), 124.1 (dd, $^{3}J_{C\text{-F}}$ = 9.5 Hz), 123.0 (t, $^{4}J_{C\text{-F}}$ = 2.7 Hz), 121.6 (t, $^{4}J_{C\text{-F}}$ = 2.7 Hz), 120.2 (d, $^{2}J_{C\text{-F}}$ = 24.3 Hz), 114.9 (d, $^{2}J_{C\text{-F}}$ = 21.2 Hz), 98.7 (t, $^{4}J_{C\text{-F}}$ = 3.2 Hz), 65.7. **^{19}F NMR** (376 MHz, Chloroform-*d*) δ -111.8, -140.2 (d, *J* = 20.3 Hz), -144.0 (d, *J* = 20.2 Hz). **HRMS** (APPI/LTQ-Orbitrap): calcd. for $[C_{17}H_{12}BrF_3O_2]^+$, $[M]^+$: 383.9967; found: 383.9976. **IR (ATR):** $\tilde{\nu}$ = 2894, 2362, 1596, 1461, 1397, 1241, 1109, 1030, 973, 861, 787, 631 cm^{-1}. **Mp**: 82.5 – 84.5 °C.

C-H activation reaction and deprotection:

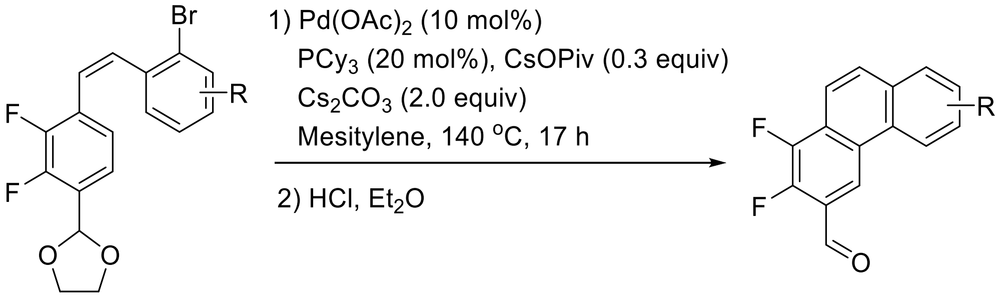

To an oven-dried 250 mL flask, under ambient air, was added (*Z*)-2-(4-(2-bromostyryl)-2,3-difluorophenyl)-1,3-dioxolane (1.0 equiv) and Cs_2CO_3 (2.0 equiv). The flask was flushed with Ar gas 3 times and then transferred to a glovebox, where Pd_2dba_3 (10 mol%), PCy_3 (20 mol%) and CsOPiv (0.3 equiv) were added. Mesitylene (0.1 M) was added to the mixture out of the glovebox, followed by flushing with Ar gas 3 times. The reaction mixture was then heated at 140 °C for 17 h. The reaction mixture was filtered through a plug of silica gel (eluted with EtOAc) and concentrated under reduced pressure. The resulting crude product was purified by column chromatography on silica gel (cyclohexane/EtOAc) to afford 2-(1,2-difluorophenanthren-3-yl)-1,3-dioxolane.

To a solution of 2-(1,2-difluorophenanthren-3-yl)-1,3-dioxolane (1 equiv) in Et_2O (0.05 M) was added 11M HCl (5 mL per mmol substrate) and the reaction was stirred at room temperature for 1 h. The mixture was extracted with Et_2O, dried over anhydrous $MgSO_4$, filtered and concentrated under reduced pressure to afford 1,2-difluorophenanthrene-3-carbaldehyde.

1,2-difluorophenanthrene-3-carbaldehyde (S24): synthesized from **S18** (4.3 g, 11.7 mmol), 2.8 g (from 3.0 g crude 2-(1,2-difluorophenanthren-3-yl)-1,3-dioxolane) was hydrolyzed to aldehyde.

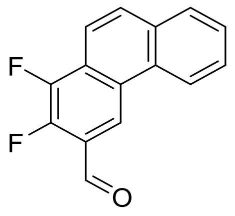

Yield: 90% (2.37 g, 2steps). Light yellow crystalline solid. ***R*f** 0.4 (cyclohexane/EtOAc 5/1); **^{1}H NMR** (400 MHz, Chloroform-*d*) δ 10.49 (s, 1H), 8.93 (dd, *J* = 5.6, 1.8 Hz, 1H), 8.67 (d, *J* = 8.1 Hz, 1H), 7.98 (d, *J* = 9.2 Hz, 1H), 7.95 (d, *J* = 4.9 Hz, 1H), 7.93 – 7.91 (m, 1H), 7.75 (ddd, *J* = 8.3, 7.0, 1.5 Hz, 1H), 7.68 (ddd, *J* = 8.2, 7.1, 1.3 Hz, 1H). **^{13}C NMR** (126 MHz, Chloroform-*d*) δ 186.7 (dd, ^{3}J = 5.5, ^{4}J = 3.3 Hz), 148.5 (dd, $^{1}J_{C\text{-F}}$ = 257.0 Hz, $^{2}J_{C\text{-F}}$ = 12.8 Hz), 145.9 (dd, $^{1}J_{C\text{-F}}$ = 252.5 Hz, $^{2}J_{C\text{-F}}$ = 11.6 Hz), 131.9 (d, ^{4}J = 2.4 Hz), 131.8, 129.4, 128.6, 128.1, 127.2, 127, 127.1 (d, ^{4}J = 3.0 Hz), 123.6 (d, ^{3}J = 8.2 Hz), 123.1, 119.5 (dd, ^{3}J = 4.2 Hz, $^{4}J_{C\text{-F}}$ = 1.7 Hz), 117.8 (dd, ^{3}J = 6.3, 4.9 Hz).**^{19}F NMR** (376 MHz, Chloroform-*d*) δ -146.6 (d, *J* = 19.3 Hz), -151.9 (d, *J* = 19.5 Hz). **HRMS** (ESI): calcd. for $[C_{15}H_8F_2O+Na]^+$, $[M+Na]^+$: 265.0435; found: 265.0435. **IR (ATR):** $\tilde{\nu}$ = 3064, 2856, 2778, 1694, 1636, 1570, 1467, 1365, 1311, 1228, 1199, 887, 752 cm^{-1}. **Mp**: 155.0 – 157.0 °C.

1,2-difluoro-7-methoxyphenanthrene-3-carbaldehyde (S25): synthesized from **S19** (1.24 g, 3.12 mmol). 0.7 g (from 0.74 g crude 2-(1,2-difluorophenanthren-3-yl)-1,3-dioxolane) was hydrolyzed to aldehyde.

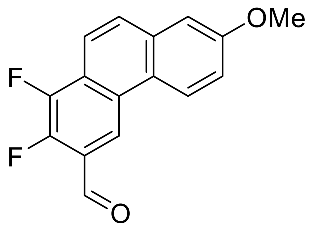

Yield: 75% (650 mg, 2 steps). Light yellow crystalline solid. ***R*f** 0.10 (cyclohexane/EtOAc 10/1); **^{1}H NMR** (400 MHz, Chloroform-*d*): δ 10.51 (s, 1H), 8.87 (dd, *J* = 5.7, 1.9 Hz, 1H), 8.60 (d, *J* = 9.1 Hz, 1H), 7.99 (d, *J* = 9.1 Hz, 1H), 7.89 (d, *J* = 9.1 Hz, 1H), 7.38 (dd, *J* = 9.1, 2.7 Hz, 1H), 7.30 (d, *J* = 2.7 Hz, 1H), 3.98 (s, 3H). **^{13}C NMR** (151 MHz, Chloroform-*d*) δ 186.7, 159.1, 147.7 (dd, $^{1}J_{C\text{-F}}$ = 255.9 Hz, $^{2}J_{C\text{-F}}$ = 12.8 Hz), 145.8 (dd, $^{1}J_{C\text{-F}}$ = 252.3 Hz, $^{2}J_{C\text{-F}}$ = 11.5 Hz), 133.2, 131.3 (d, $^{4}J_{C\text{-F}}$ = 2.3 Hz), 127.1 (t, $^{4}J_{C\text{-F}}$ = 3.0 Hz), 125.8 (d, $^{2}J_{C\text{-F}}$ = 12.6 Hz), 124.5, 124.2, 123.6 (d, $^{3}J_{C\text{-F}}$ = 8.0 Hz), 118.9, 118.8 (d, $^{4}J_{C\text{-F}}$ = 4.1 Hz), 118.3 (t, $^{4}J_{C\text{-F}}$ = 5.5 Hz), 109.1, 55.5. **^{19}F NMR** (376 MHz, Chloroform-*d*) δ -146.8 (d, *J* = 19.5 Hz), -153.6 (d, *J* = 19.5 Hz).

HRMS (ESI): calcd. for $[C_{16}H_{10}F_2O_2+Na]^+$, $[M+Na]^+$: 295.0541; found: 295.0539. **IR (ATR):** $\tilde{\nu}$ = 2971, 2362, 1693, 1633, 1477, 1361, 1262, 1210, 1041, 872, 817, 671 cm^{-1}. **Mp**: 164.3 – 166.0 °C.

<u>7,8-difluoro-6-formylphenanthrene-2-carbonitrile (S26):</u> synthesized from **S20** (1.5 g, 3.82 mmol).

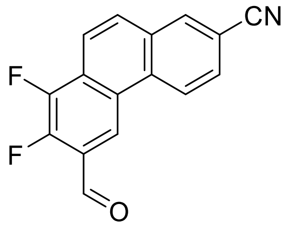

Yield: 84% (800 mg, 2 steps). Yellow solid. $\boldsymbol{R_f}$ 0.35 (cyclohexane/EtOAc 5/1); **^{1}H NMR** (400 MHz, Chloroform-*d*): δ 10.55 (s, 1H), 9.00 (d, *J* = 4.8 Hz, 1H), 8.80 (d, *J* = 8.6 Hz, 1H), 8.30 (d, *J* = 1.6 Hz, 1H), 8.15 (d, *J* = 9.1 Hz, 1H), 7.98 (d, *J* = 9.2 Hz, 1H), 7.95 (dd, *J* = 8.6, 1.6 Hz, 1H). **^{13}C NMR** (151 MHz, Chloroform-*d*) δ 186.2 (ddd, $^3J_{C\text{-F}}$ = 9.2 Hz, $^4J_{C\text{-F}}$ = 5.3, 3.0 Hz), 149.6 (dd, $^1J_{C\text{-F}}$ = 259.9 Hz, $^2J_{C\text{-F}}$ = 12.7 Hz), 146.2 (dd, $^1J_{C\text{-F}}$ = 254.4 Hz, $^2J_{C\text{-F}}$ = 11.6 Hz), 134.3, 132.6 (t, $^4J_{C\text{-F}}$ = 2.0 Hz), 131.4, 130.8 (d, $^4J_{C\text{-F}}$ = 2.5 Hz), 129.9, 128.0 (dd, $^2J_{C\text{-F}}$ = 12.4 Hz, $^4J_{C\text{-F}}$ = 2.0 Hz), 126.2 (t, $^4J_{C\text{-F}}$ = 2.9 Hz), 124.4, 124.4 (d, $^3J_{C\text{-F}}$ = 10.8 Hz), 120.0 (dd, $^4J_{C\text{-F}}$ = 5.2, 1.0 Hz), 120.0 (dd, $^4J_{C\text{-F}}$ = 2.8, 1.9 Hz), 118.6, 111.7. **^{19}F NMR** (376 MHz, Chloroform-*d*) δ -145.0 (d, *J* = 19.4 Hz), -148.8 (d, *J* = 19.4 Hz). **HRMS** (APPI/LTQ-Orbitrap): calcd. for $[C_{16}H_8F_2NO+H]^+$, $[M+H]^+$: 268.0568; found: 268.0570. **IR (ATR):** $\tilde{\nu}$ = 2986, 2362, 2230, 1702, 1636, 1471, 1315, 1036, 902, 832, 789, 670 cm^{-1}. **Mp**: 261.9 – 263.9 °C.

<u>1,2,7-trifluorophenanthrene-3-carbaldehyde (S27):</u> synthesized from **S21** (1.5 g, 3.89 mmol). 1.0 g (from 1.1 g crude 2-(1,2-difluorophenanthren-3-yl)-1,3-dioxolane) was hydrolyzed to aldehyde.

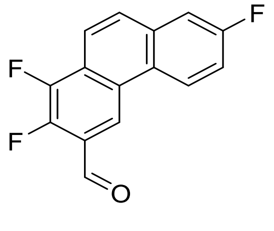

Yield: 93% (880 mg, 2 steps). White solid. $\boldsymbol{R_f}$ 0.25 (cyclohexane/EtOAc 10/1); **^{1}H NMR** (400 MHz, Chloroform-*d*): δ 10.51 (s, 1H), 8.87 (dd, *J* = 5.6, 1.8 Hz, 1H), 8.66 (dd, *J* = 9.1, 5.2 Hz, 1H), 8.02 (d, *J* = 9.1 Hz, 1H), 7.88 (d, *J* = 9.1 Hz, 1H), 7.57 (dd, *J* = 8.9, 2.7 Hz, 1H), 7.49 (td, *J* = 8.6, 2.7 Hz, 1H). **^{13}C NMR** (126 MHz, Chloroform-*d*) δ 186.5 (dd, $^4J_{C\text{-F}}$ = 5.7, 3.3 Hz), 162.0 (d, $^1J_{C\text{-F}}$ = 249.3 Hz), 148.4 (dd, $^1J_{C\text{-F}}$ = 257.3 Hz, $^2J_{C\text{-F}}$ = 12.6 Hz), 146.0 (dd, $^1J_{C\text{-F}}$ = 253.2 Hz, $^2J_{C\text{-F}}$ = 11.6 Hz), 133.3 (d, $^3J_{C\text{-F}}$ = 8.6 Hz), 131.0 (d, $^4J_{C\text{-F}}$ = 2.5 Hz), 126.9 (t, $^4J_{C\text{-F}}$ = 2.5 Hz), 126.7 (t, $^4J_{C\text{-F}}$ = 2.1 Hz), 126.6 (d, $^2J_{C\text{-F}}$ = 12.2 Hz), 125.6 (d, $^3J_{C\text{-F}}$ = 8.9 Hz), 124.0 (d, $^3J_{C\text{-F}}$ = 8.4 Hz), 119.2 (t, $^4J_{C\text{-F}}$ = 5.5 Hz), 119.1 (d, $^4J_{C\text{-F}}$ = 4.3 Hz), 117.6 (d, $^2J_{C\text{-F}}$ = 23.8 Hz), 113.6 (d, $^2J_{C\text{-F}}$ = 20.9 Hz). **^{19}F NMR** (376 MHz, Chloroform-*d*) δ -112.5 (d, *J* = 2.4 Hz), -146.0 (d, *J* = 19.4 Hz), -152.1 (dd, *J* = 19.4, 2.2 Hz). **HRMS** (APPI/LTQ-Orbitrap): calcd. for

$[C_{15}H_7F_3O]^+$, $[M]^+$: 260.0444; found: 260.0444. **IR (ATR):** $\tilde{\nu}$ = 2975, 2362, 1704, 1638, 1528, 1476, 1393, 1316, 1257, 1146, 1065, 961, 861, 820, 650 cm^{-1}. **Mp**: 161.9 – 163.8 °C.

1,2-difluoro-7-methylphenanthrene-3-carbaldehyde (S28): synthesized from **S22** (1.5 g, 3.93 mmol). 0.77 g (from 1.0 g crude 2-(1,2-difluorophenanthren-3-yl)-1,3-dioxolane) was hydrolyzed to aldehyde.

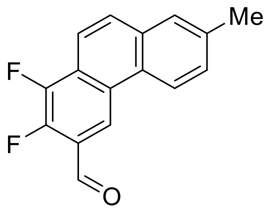

Yield: 85% (770 mg, 2 steps). Yellow solid. ***R*f** 0.30 (cyclohexane/EtOAc 10/1); **^{1}H NMR** (400 MHz, Chloroform-*d*): δ 10.51 (s, 1H), 8.93 (d, *J* = 5.5 Hz, 1H), 8.58 (d, *J* = 8.4 Hz, 1H), 7.97 (d, *J* = 9.1 Hz, 1H), 7.89 (d, *J* = 9.1 Hz, 1H), 7.72 (s, 1H), 7.58 (dd, *J* = 8.5, 1.9 Hz, 1H), 2.59 (s, 3H). **^{13}C NMR** (151 MHz, Chloroform-*d*) δ 186.9 (dd, $^3J_{C\text{-F}}$ = 5.4 Hz), 148.3 (dd, $^1J_{C\text{-F}}$ = 256.5 Hz, $^2J_{C\text{-F}}$ = 12.8 Hz), 145.9 (dd, $^1J_{C\text{-F}}$ = 252.2 Hz, $^2J_{C\text{-F}}$ = 11.5 Hz), 138.1, 132.0, 131.8 (d, $^4J_{C\text{-F}}$ = 2.2 Hz), 130.3, 128.9, 128.0 (t, $^4J_{C\text{-F}}$ = 1.9 Hz), 127.2 (t, $^4J_{C\text{-F}}$ = 3.0 Hz), 126.8 (dd, $^2J_{C\text{-F}}$ = 12.4 Hz, $^4J_{C\text{-F}}$ = 1.6 Hz), 123.6 (d, $^3J_{C\text{-F}}$ = 8.0 Hz), 123.0, 119.3 (dd, $^4J_{C\text{-F}}$ = 4.2, 1.4 Hz), 117.8 (t, $^4J_{C\text{-F}}$ = 5.3 Hz), 21.7. **^{19}F NMR** (376 MHz, Chloroform-*d*) δ -146.8 (d, *J* = 19.3 Hz), -152.7 (d, *J* = 19.5 Hz). **HRMS** (ESI): calcd. for $[C_{16}H_{10}F_2O+Na]^+$, $[M+Na]^+$: 279.0592; found: 279.0592. **IR (ATR):** $\tilde{\nu}$ = 2852, 2361, 1701, 1635, 1474, 1314, 1203, 1034, 821, 790, 736, 667 cm^{-1}. **Mp**: 128.7 – 130.6 °C.

1,2,6-trifluorophenanthrene-3-carbaldehyde (S29): synthesized from **S23** (1.5 g, 3.89 mmol). 1.0 g (from 1.06 g crude 2-(1,2-difluorophenanthren-3-yl)-1,3-dioxolane) was hydrolyzed to aldehyde.

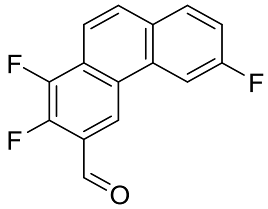

Yield: 90% (900 mg, 2 steps). White solid. ***R*f** 0.30 (cyclohexane/EtOAc 10/1); **^{1}H NMR** (400 MHz, Chloroform-*d*): δ 10.52 (s, 1H), 8.85 (dd, *J* = 5.7, 1.8 Hz, 1H), 8.31 (dd, *J* = 10.6, 2.5 Hz, 1H), 8.08 – 7.91 (m, 3H), 7.44 (td, *J* = 8.4, 2.5 Hz, 1H). **^{13}C NMR** (151 MHz, Chloroform-*d*) δ 186.5 (ddd, $^3J_{C\text{-F}}$ = 8.9 Hz, $^4J_{C\text{-F}}$ = 5.5, 3.1 Hz), 162.6 (d, $^1J_{C\text{-F}}$ = 249.2 Hz), 148.9 (dd, $^1J_{C\text{-F}}$ = 258.1 Hz, $^2J_{C\text{-F}}$ = 12.8 Hz), 146.0 (dd, $^1J_{C\text{-F}}$ = 252.9 Hz, $^2J_{C\text{-F}}$ = 11.5 Hz), 131.8 (d, $^3J_{C\text{-F}}$ = 9.1 Hz), 131.7 (d, $^3J_{C\text{-F}}$ = 9.1 Hz), 131.3, 128.5, 127.5 (dd, $^2J_{C\text{-F}}$ = 12.5 Hz, $^4J_{C\text{-F}}$ = 1.8 Hz), 126.5 (q, $^4J_{C\text{-F}}$ = 3.4 Hz), 123.7 (d, $^3J_{C\text{-F}}$ = 8.3 Hz), 119.7 (d, $^4J_{C\text{-F}}$ = 4.4 Hz), 117.3 (d, $^2J_{C\text{-F}}$ = 23.9 Hz), 117.2 (dd, $^4J_{C\text{-F}}$ = 5.9, 2.2 Hz), 108.6 (d, $^2J_{C\text{-F}}$ = 23.0 Hz).**^{19}F NMR** (376 MHz, Chloroform-*d*) δ -109.8 (d, *J* = 1.5 Hz), -146.0 (dd, *J* = 19.3, 1.5 Hz), -150.9 (d, *J* = 19.5 Hz). **HRMS** (ESI/QTOF): calcd. for $[C_{15}H_7F_3O+Na]^+$, $[M+Na]^+$: 283.0341; found: 283.0337. **IR**

(ATR): $\tilde{\nu}$ = 2975, 2361, 1699, 1627, 1468, 1398, 1300, 1228, 1098, 1034, 836, 727, 668 cm^{-1}. **Mp**: 152.9 – 154.5 °C.

Wittig reaction:[5]

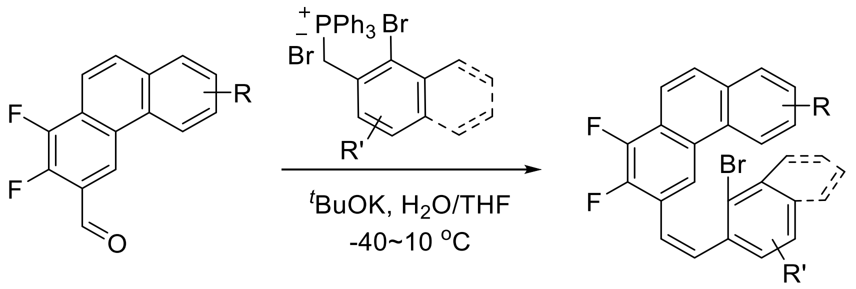

2-Bromobenzyl bromide phosphonium salt (1.1 equiv) was treated with potassium *tert*-butoxide (1.2 equiv) in THF (0.25 M)/H_2O (2.5 M) at -40 °C and stirred for 15 min. 1,2-difluorophenanthrene-3-carbaldehyde (5.0 mmol) was added. The mixture was stirred and warmed slowly to 10 °C in 5 h, and kept under 10 °C. The reaction was monitored by TLC. The resulting mixture was quenched with water and extracted with EtOAc, washed with brine and dried over anhydrous Na_2SO_4. The crude product was purified by column chromatography (cyclohexane/EtOAc 99/1), re-precipitation or recrystallization.

(*Z*)-3-(2-bromo-3-methylstyryl)-1,2-difluorophenanthrene (1h): synthesized from **S24** (0.363 g, 1.50 mmol).

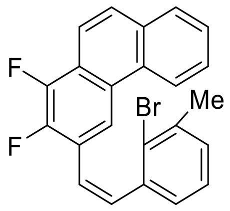

Re-precipitated in Et_2O. **Yield**: 50% (310 mg). White solid. ***R*f** 0.35 (cyclohexane/EtOAc 20/1); **^{1}H NMR** (400 MHz, Chloroform-*d*): δ 8.11 (dd, *J* = 6.3, 1.8 Hz, 1H), 7.90 (d, *J* = 9.0 Hz, 1H), 7.93 – 7.85 (m, 1H), 7.84 (dd, *J* = 7.5, 1.8 Hz, 1H), 7.74 (d, *J* = 9.1 Hz, 1H), 7.53 (pd, *J* = 7.1, 1.5 Hz, 2H), 7.16 (dd, *J* = 7.7, 1.4 Hz, 1H), 7.07 – 6.90 (m, 4H), 2.53 (s, 3H). **^{13}C NMR** (126 MHz, Chloroform-*d*) δ 146.1 (dd, $^1J_{C\text{-}F}$ = 249.4, $^2J_{C\text{-}F}$ = 19.6 Hz), 146.0 (dd, $^1J_{C\text{-}F}$ = 249.3, $^2J_{C\text{-}F}$ = 21.0 Hz), 139.0, 138.4, 134.1 (d, $^4J_{C\text{-}F}$ = 1.5 Hz), 131.7, 130.1, 129.5, 129.0, 128.5 (d, $^4J_{C\text{-}F}$ = 2.2 Hz), 127.9, 127.5, 127.3, 127.1, 126.5, 126.4, 125.0 (d, $^2J_{C\text{-}F}$ = 12.7 Hz), 122.8 (t, $^3J_{C\text{-}F}$ = 4.1 Hz), 122.5, 122.3 (d, $^2J_{C\text{-}F}$ = 11.2 Hz), 119.3 (dd, $^4J_{C\text{-}F}$ = 3.7, 2.5 Hz), 118.0 (t, $^3J_{C\text{-}F}$ = 5.5 Hz), 23.9. **^{19}F NMR** (376 MHz, Chloroform-*d*) δ -144.0 (d, *J* = 19.3 Hz), -148.5 (d, *J* = 19.2 Hz). **HRMS** (ESI): calcd. for $[C_{23}H_{15}BrF_2]^+$, $[M]^+$: 408.0320; found: 408.0316. **IR (ATR):** $\tilde{\nu}$ = 3058, 2361, 1643, 1460, 1298, 1101, 971, 882, 816, 748, 669 cm^{-1}. **Mp**: 142.9 – 145.0 °C.

(Z)-3-(2-bromo-3-isopropoxystyryl)-1,2-difluorophenanthrene (1i): synthesized from **S24** (0.363 g, 1.50 mmol).

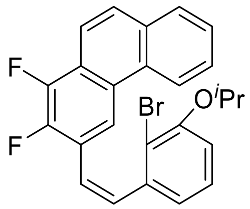

Re-precipitated in Et_2O. **Yield**: 27% (180 mg). Light yellow solid. ***R*f** 0.30 (cyclohexane/EtOAc 20/1); **^{1}H NMR** (400 MHz, Chloroform-*d*): δ 8.09 (dd, *J* = 6.3, 1.8 Hz, 1H), 7.89 (dd, *J* = 8.7, 2.8 Hz, 2H), 7.83 (dd, *J* = 7.8, 1.4 Hz, 1H), 7.74 (d, *J* = 9.0 Hz, 1H), 7.54 (ddd, *J* = 8.0, 7.0, 1.3 Hz, 1H), 7.48 (ddd, *J* = 8.4, 7.0, 1.5 Hz, 1H), 7.03 – 6.91 (m, 3H), 6.83 (dd, *J* = 8.2, 1.4 Hz, 1H), 6.79 (dd, *J* = 7.6, 1.4 Hz, 1H), 4.63 (hept, *J* = 6.1 Hz, 1H), 1.46 (d, *J* = 6.1 Hz, 6H). **^{13}C NMR** (151 MHz, Chloroform-*d*) δ 155.2, 146.4 (dd, $^1J_{C\text{-}F}$ = 249.8, $^2J_{C\text{-}F}$ = 12.0 Hz), 145.9 (dd, $^1J_{C\text{-}F}$ = 248.8, $^2J_{C\text{-}F}$ = 12.3 Hz), 139.8, 133.6, 131.7, 129.5, 129.0, 128.5 (d, $^4J_{C\text{-}F}$ = 2.1 Hz), 128.1, 127.4, 127.0, 126.4 (d, $^4J_{C\text{-}F}$ = 3.3 Hz), 124.9 (d, $^2J_{C\text{-}F}$ = 12.6 Hz), 123.0 (t, $^4J_{C\text{-}F}$ = 3.5 Hz), 122.6, 122.6, 122.2 (d, $^2J_{C\text{-}F}$ = 12.7 Hz), 119.4 (dd, $^3J_{C\text{-}F}$ = 4.2, $^4J_{C\text{-}F}$ = 2.6 Hz), 118.0 (t, $^3J_{C\text{-}F}$ = 5.4 Hz), 115.3, 114.7, 72.5, 22.3. **^{19}F NMR** (376 MHz, Chloroform-*d*) δ -144.1 (d, *J* = 19.3 Hz), -148.6 (d, *J* = 19.3 Hz). **HRMS** (ESI) (0.2 mM $AgNO_3$): calcd. for $[C_{25}H_{19}BrF_2O+Ag]^+$, $[M+Ag]^+$: 558.9633; found: 558.9626. **IR (ATR):** $\tilde{\nu}$ = 2979, 2362, 1643, 1563, 1459, 1385, 1287, 1114, 1031, 968, 817, 749 cm^{-1}. **Mp**: 85.9 – 87.9 °C.

(Z)-3-(2-bromo-3-methylstyryl)-1,2-difluoro-7-methoxyphenanthrene (1j): synthesized from **S25** (0.50 g, 1.84 mmol).

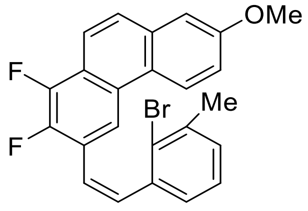

Recrystallized in cyclohexane/Et_2O. **Yield**: 57% (460 mg). White crystal. ***R*f** 0.23 (cyclohexane/EtOAc 20/1); **^{1}H NMR** (400 MHz, Chloroform-*d*): δ 7.99 (dd, *J* = 6.3, 1.8 Hz, 1H), 7.88 (d, *J* = 9.1 Hz, 1H), 7.79 (d, *J* = 9.0 Hz, 1H), 7.67 (d, *J* = 9.1 Hz, 1H), 7.19 (d, *J* = 2.7 Hz, 1H), 7.16 – 7.09 (m, 2H), 7.05 – 6.88 (m, 4H), 3.93 (s, 3H), 2.52 (s, 3H). **^{13}C NMR** (126 MHz, Chloroform-*d*) δ 158.6, 146.0 (dd, $^1J_{C\text{-}F}$ = 249.0, $^2J_{C\text{-}F}$ = 12.8 Hz), 145.5 (dd, $^1J_{C\text{-}F}$ = 248.4, $^2J_{C\text{-}F}$ = 12.2 Hz), 139.0, 138.4, 133.9 (d, $^4J_{C\text{-}F}$ = 1.4 Hz), 133.2, 130.0, 128.1 (d, $^4J_{C\text{-}F}$ = 2.2 Hz), 127.9, 127.2, 126.7 (t, $^4J_{C\text{-}F}$ = 3.0 Hz), 126.4, 125.0 (d, $^2J_{C\text{-}F}$ = 12.6 Hz), 124.1, 123.8, 122.9 (t, $^4J_{C\text{-}F}$ = 3.4 Hz), 121.2 (d, $^2J_{C\text{-}F}$ = 12.6 Hz), 118.7 (t, $^4J_{C\text{-}F}$ = 2.1 Hz), 118.6 (d, $^3J_{C\text{-}F}$ = 5.8 Hz), 118.0, 109.0, 55.6, 23.9. **^{19}F NMR** (376 MHz, Chloroform-*d*) δ -145.6 (d, *J* = 19.3 Hz), -148.7 (d, *J* = 19.3 Hz). **HRMS** (ESI): calcd. for $[C_{24}H_{17}BrF_2O+Na]^+$, $[M+Na]^+$: 461.0323; found: 461.0316. **IR (ATR):** $\tilde{\nu}$ = 2957, 2361, 1613, 1522, 1473, 1306, 1259, 1210, 1170, 1040, 854, 821, 770, 657 cm^{-1}. **Mp**: 139.0 – 140.7 °C.

(*Z*)-6-(2-bromo-3-methylstyryl)-7,8-difluorophenanthrene-2-carbonitrile (1k): synthesized from **S26** (0.50 g, 1.87 mmol).

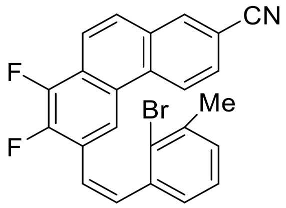

Re-precipitated in Et_2O. **Yield**: 52% (425 mg). Yellow solid. $\boldsymbol{R_f}$ 0.20 (cyclohexane/EtOAc 20/1); **^{1}H NMR** (500 MHz, Chloroform-*d*): δ 8.18 (d, *J* = 1.7 Hz, 1H), 8.09 (dd, *J* = 6.1, 2.1 Hz, 1H), 8.02 (d, *J* = 9.0 Hz, 1H), 7.90 (d, *J* = 8.7 Hz, 1H), 7.74 (d, *J* = 9.1 Hz, 1H), 7.68 (dd, *J* = 8.6, 1.8 Hz, 1H), 7.18 (dd, *J* = 7.0, 2.2 Hz, 1H), 7.04 (d, *J* = 12.0 Hz, 1H), 7.02 – 6.97 (m, 2H), 6.95 (d, *J* = 12.5 Hz, 1H), 2.53 (s, 3H). **^{13}C NMR** (151 MHz, Chloroform-*d*) δ 147.1 (dd, $^1J_{C\text{-F}}$ = 252.6, $^2J_{C\text{-F}}$ = 12.2 Hz), 146.0 (dd, $^1J_{C\text{-F}}$ = 251.3, $^2J_{C\text{-F}}$ = 12.8 Hz), 139.2, 138.2, 134.9, 134.0, 132.0 (t, $^4J_{C\text{-F}}$ = 2.1 Hz), 131.3, 130.2, 128.9, 127.8, 127.5 (d, $^4J_{C\text{-F}}$ = 2.2 Hz), 127.3, 126.3, 126.1 (d, $^2J_{C\text{-F}}$ = 12.7 Hz), 125.4 (t, $^4J_{C\text{-F}}$ = 3.1 Hz), 123.7, 123.1 (d, $^2J_{C\text{-F}}$ = 12.7 Hz), 122.2 (t, $^4J_{C\text{-F}}$ = 3.4 Hz), 120.1 (t, $^4J_{C\text{-F}}$ = 5.4 Hz), 120.0 (dd, $^4J_{C\text{-F}}$ = 3.1, 0.9 Hz), 118.9, 110.6, 23.9. **^{19}F NMR** (376 MHz, Chloroform-*d*) δ -140.8 (d, *J* = 19.2 Hz), -147.1 (d, *J* = 19.2 Hz). **HRMS** (ESI) (0.2 mM $AgNO_3$): calcd. for $[C_{24}H_{14}BrF_2N+Ag]^+$, $[M+Ag]^+$: 539.9323; found: 539.9316. **IR (ATR):** $\tilde{\nu}$ = 2983, 2361, 2230, 1640, 1472, 1392, 1301, 1031, 902, 826, 796, 769, 733, 678 cm^{-1}. **Mp**: 185.9 – 187.0 °C.

(*Z*)-3-(2-bromo-3-methylstyryl)-1,2,7-trifluorophenanthrene (1l): synthesized from **S27** (0.50 g, 1.92 mmol).

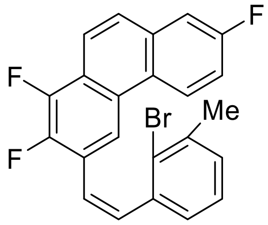

Re-precipitated in Et_2O. **Yield**: 55% (447 mg). White solid. $\boldsymbol{R_f}$ 0.35 (cyclohexane/EtOAc 20/1); **^{1}H NMR** (500 MHz, Chloroform-*d*): δ 8.02 (dd, *J* = 6.2, 2.3 Hz, 1H), 7.93 (d, *J* = 9.0 Hz, 1H), 7.84 (dd, *J* = 9.0, 5.4 Hz, 1H), 7.67 (d, *J* = 9.1 Hz, 1H), 7.46 (dd, *J* = 9.2, 2.7 Hz, 1H), 7.25 (ddd, *J* = 9.1, 8.1, 2.7 Hz, 1H), 7.16 (dd, *J* = 7.3, 1.8 Hz, 1H), 7.05 – 6.91 (m, 4H), 2.53 (s, 3H). **^{13}C NMR** (151 MHz, Chloroform-*d*) δ 161.5 (d, $^1J_{C\text{-F}}$ = 247.4 Hz), 146.0 (dd, $^1J_{C\text{-F}}$ = 249.7 Hz, $^2J_{C\text{-F}}$ = 13.2 Hz), 146.0 (dd, $^1J_{C\text{-F}}$ = 249.8 Hz, $^2J_{C\text{-F}}$ = 12.2 Hz), 139.1, 138.3, 134.3 (d, $^4J_{C\text{-F}}$ = 1.4 Hz), 133.1 (d, $^3J_{C\text{-F}}$ = 8.9 Hz), 130.1, 127.9, 127.7 (t, $^4J_{C\text{-F}}$ = 2.1, 1.4 Hz), 127.3, 126.4, 126.3 (t, $^4J_{C\text{-F}}$ = 2.1, 1.4 Hz), 126.1 (d, $^4J_{C\text{-F}}$ = 2.0 Hz), 125.4 (d, $^2J_{C\text{-F}}$ = 12.6 Hz), 124.9 (d, $^3J_{C\text{-F}}$ = 8.7 Hz), 122.7 (t, $^4J_{C\text{-F}}$ = 3.4 Hz), 121.7 (d, $^2J_{C\text{-F}}$ = 12.4 Hz), 119.4 (t, $^4J_{C\text{-F}}$ = 5.4 Hz), 119.0 (t, $^4J_{C\text{-F}}$ = 3.4 Hz), 116.5 (d, $^2J_{C\text{-F}}$ = 23.7 Hz), 113.1 (d, $^2J_{C\text{-F}}$ = 20.6 Hz), 23.9. **^{19}F NMR** (376 MHz, Chloroform-*d*) δ -114.4 (d, *J* = 2.0 Hz), -144.1 (dd, *J* = 19.3, 2.2 Hz), -148.0 (d, *J* = 19.3 Hz). **HRMS** (APPI/LTQ-Orbitrap): calcd. for $[C_{23}H_{14}BrF_3]^+$, $[M]^+$: 426.0225; found: 426.0230. **IR (ATR):** $\tilde{\nu}$ = 2982, 2362, 1638, 1470, 1255, 1145, 1030, 959, 871, 791, 623 cm^{-1}. **Mp**: 127.7 – 129.7 °C.

(Z)-3-(2-bromo-3-methylstyryl)-1,2-difluoro-7-methylphenanthrene (1m): synthesized from **S28** (0.50 g, 1.95 mmol).

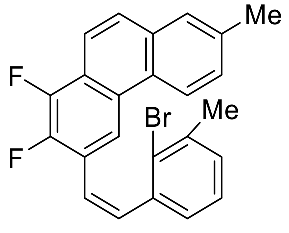

Recrystallized in Et_2O. **Yield**: 48% (396 mg). White crystalline solid. ***R*f** 0.35 (cyclohexane/EtOAc 20/1); **^{1}H NMR** (500 MHz, Chloroform-*d*): δ 8.05 (dd, *J* = 6.4, 2.4 Hz, 1H), 7.86 (d, *J* = 9.0 Hz, 1H), 7.77 (d, *J* = 8.4 Hz, 1H), 7.67 (d, *J* = 9.1 Hz, 1H), 7.61 (s, 1H), δ 7.35 (dd, *J* = 8.5, 1.8 Hz, 1H), 7.15 (ddt, *J* = 7.4, 1.5, 0.7 Hz, 1H), 7.03 (ddt, *J* = 7.7, 1.8, 0.7 Hz, 1H), 7.01 – 6.90 (m, 3H), 2.53 (s, 3H), 2.51 (s, 3H). **^{13}C NMR** (126 MHz, Chloroform-*d*) δ 146.0 (dd, $^1J_{C\text{-F}}$ = 249.1 Hz, $^2J_{C\text{-F}}$ = 14.3 Hz), 145.9 (dd, $^1J_{C\text{-F}}$ = 248.9 Hz, $^2J_{C\text{-F}}$ = 13.7 Hz), 139.0, 138.5, 136.9, 133.9 (d, $^4J_{C\text{-F}}$ = 1.5 Hz), 131.9, 130.0, 129.3, 128.5, 128.3 (d, $^4J_{C\text{-F}}$ = 2.2 Hz), 127.9, 127.3, 127.3, 126.6 (t, $^4J_{C\text{-F}}$ = 3.9 Hz), 126.4, 124.8 (d, $^2J_{C\text{-F}}$ = 12.6 Hz), 122.9 (t, $^4J_{C\text{-F}}$ = 3.5 Hz), 122.4, 121.9 (d, $^2J_{C\text{-F}}$ = 12.4 Hz), 119.0 (dd, $^4J_{C\text{-F}}$ = 4.3, 2.6 Hz), 118.0 (t, $^4J_{C\text{-F}}$ = 5.4 Hz), 23.9, 21.5. **^{19}F NMR** (376 MHz, Chloroform-*d*) δ -144.8 (d, *J* = 19.2 Hz), -148.7 (d, *J* = 19.3 Hz). **HRMS** (ESI) (0.2 mM $AgNO_3$): calcd. for $[C_{24}H_{17}BrF_2+Ag]^+$, $[M+Ag]^+$: 528.9527; found: 528.9519. **IR (ATR):** $\tilde{\nu}$ = 2980, 2362, 1638, 1473, 1293, 1242, 1030, 882, 821, 771, 673 cm^{-1}. **Mp**: 144.6 – 146.6 °C.

(Z)-3-(2-bromo-3-methylstyryl)-1,2,6-trifluorophenanthrene (1n): synthesized from **S29** (0.50 g, 1.92 mmol).

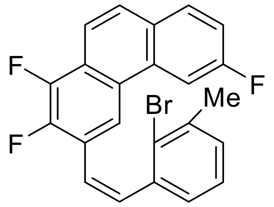

Recrystallized in Et_2O/DCM. **Yield**: 45% (368 mg). Colourless crystal. ***R*f** 0.35 (cyclohexane/EtOAc 20/1); **^{1}H NMR** (500 MHz, Chloroform-*d*): δ 7.93 (dd, *J* = 6.4, 1.6 Hz, 1H), 7.87 – 7.82 (m, 1H), 7.80 (dd, *J* = 8.7, 5.9 Hz, 1H), 7.70 (d, *J* = 9.1 Hz, 1H), 7.41 (dd, *J* = 11.0, 1.9 Hz, 1H), 7.29 (ddd, *J* = 8.8, 8.1, 2.5 Hz, 1H), 7.24 – 7.16 (m, 1H), 7.04 – 6.97 (m, 3H), 6.94 (d, *J* = 12.1 Hz, 1H), 2.55 (s, 3H). **^{13}C NMR** (126 MHz, Chloroform-*d*) δ 162.0 (d, $^1J_{C\text{-F}}$ = 246.9 Hz), 146.5 (dd, $^1J_{C\text{-F}}$ = 250.2 Hz, $^2J_{C\text{-F}}$ = 12.2 Hz), 146.0 (dd, $^1J_{C\text{-F}}$ = 250.4 Hz, $^2J_{C\text{-F}}$ = 12.6 Hz), 139.3, 138.6, 134.3 (d, $^4J_{C\text{-F}}$ = 1.5 Hz), 131.1, 131.0, 130.3, 128.4, 127.9, 127.7, 127.4, 126.3, 125.8 (dd, $^4J_{C\text{-F}}$ = 4.0, 2.8 Hz), 125.0 (d, $^2J_{C\text{-F}}$ = 12.5 Hz), 122.5 (d, $^4J_{C\text{-F}}$ = 3.7 Hz), 122.4 (dd, $^4J_{C\text{-F}}$ = 4.6, 1.4 Hz), 119.5 (t, $^4J_{C\text{-F}}$ = 3.3 Hz), 117.3 (td, $^4J_{C\text{-F}}$ = 5.7, 2.6 Hz), 116.2 (d, $^2J_{C\text{-F}}$ = 23.7 Hz), 107.8 (d, $^2J_{C\text{-F}}$ = 22.8 Hz), 23.8. **^{19}F NMR** (376 MHz, Chloroform-*d*) δ -112.2, -143.2 (d, *J* = 19.1 Hz), -148.0 (d, *J* = 19.0 Hz). **HRMS** (ESI) (0.2 mM $AgNO_3$): calcd. for $[C_{23}H_{14}BrF_3+Ag]^+$, $[M+Ag]^+$: 532.9276; found: 532.9272. **IR (ATR):** $\tilde{\nu}$ = 3057,

2362, 1610, 1516, 1470, 1401, 1297, 1229, 1091, 1030, 861, 834, 761, 740, 670 cm^{-1}. **Mp**: 149.3 – 151.3 °C.

(*Z*)-3-(2-(1-bromonaphthalen-2-yl)vinyl)-1,2-difluorophenanthrene (1r): synthesized from **S24** (1.46 g, 6.03 mmol).

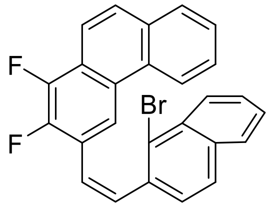

Re-precipitated in Et_2O. **Yield**: 47% (1.27 g). Light yellow powder. ***R*f** 0.33 (cyclohexane/EtOAc 20/1); **^{1}H NMR** (500 MHz, Chloroform-*d*): δ 8.44 (d, *J* = 8.5 Hz, 1H), 8.09 (d, *J* = 5.0 Hz, 1H), 7.90 (d, *J* = 9.0 Hz, 1H), 7.79 (d, *J* = 7.3 Hz, 1H), 7.76 – 7.70 (m, 2H), 7.65 (ddd, *J* = 8.4, 6.9, 1.3 Hz, 1H), 7.59 (d, *J* = 8.4 Hz, 1H), 7.57 – 7.50 (m, 2H), 7.46 (ddd, *J* = 8.0, 7.0, 1.1 Hz, 1H), 7.28 (d, *J* = 8.5 Hz, 1H), 7.24 (ddd, *J* = 8.4, 7.0, 1.3 Hz, 1H), 7.22 (d, *J* = 12.0 Hz, 1H), 7.06 (d, *J* = 12.2 Hz, 1H). **^{13}C NMR** (126 MHz, Chloroform-*d*) δ 146.1 (dd, $^{1}J_{C\text{-F}}$ = 249.1 Hz, $^{2}J_{C\text{-F}}$ = 17.3 Hz), 146.0 (dd, $^{1}J_{C\text{-F}}$ = 249.7 Hz, $^{2}J_{C\text{-F}}$ = 18.4 Hz), 135.9, 134.0, 134.0, 134.0, 132.8, 131.7, 129.4, 128.9, 128.7 (d, $^{4}J_{C\text{-F}}$ = 2.2 Hz), 128.4, 127.8, 127.4, 127.3, 127.0, 127.0, 126.6 (t, $^{4}J_{C\text{-F}}$ = 3.1 Hz), 124.9 (t, $^{3}J_{C\text{-F}}$ = 12.6 Hz), 124.3, 123.3 (t, $^{4}J_{C\text{-F}}$ = 3.1 Hz), 122.5, 122.5 (d, $^{4}J_{C\text{-F}}$ = 1.2 Hz), 119.5 (dd, $^{4}J_{C\text{-F}}$ = 4.0, 2.7 Hz), 117.9 (t, $^{4}J_{C\text{-F}}$ = 5.6 Hz) . **^{19}F NMR** (376 MHz, Chloroform-*d*) δ -143.9 (d, *J* = 19.3 Hz), -148.2 (d, *J* = 19.2 Hz). **HRMS** (ESI) (0.2 mM $AgNO_3$): calcd. for $[C_{26}H_{15}BrF_2+Ag]^+$, $[M+Ag]^+$: 550.9371; found: 550.9365. **IR (ATR):** $\tilde{\nu}$ = 3059, 2362, 1642, 1470, 1294, 1029, 788 cm^{-1}. **Mp**: 142.1 – 144.3 °C.

(*Z*)-3-(2-(1-bromonaphthalen-2-yl)vinyl)-1,2,6-trifluorophenanthrene (1u): synthesized from **S29** (0.35 g, 1.35 mmol).

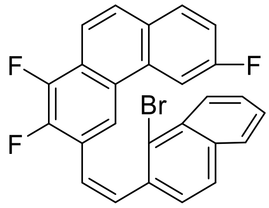

Re-precipitated in Et_2O. **Yield**: 43% (270 mg). White powder. ***R*f** 0.32 (cyclohexane/EtOAc 20/1); **^{1}H NMR** (400 MHz, Chloroform-*d*): δ 8.43 (d, *J* = 8.5 Hz, 1H), 7.93 (d, *J* = 5.4 Hz, 1H), 7.85 (d, *J* = 9.1 Hz, 1H), 7.80 – 7.73 (m, 2H), 7.70 (d, *J* = 9.1 Hz, 1H), 7.65 (ddd, *J* = 8.4, 6.8, 1.3 Hz, 1H), 7.59 – 7.50 (m, 2H), 7.34 – 7.11 (m, 4H), 7.05 (d, *J* = 12.0 Hz, 1H). **^{13}C NMR** (151 MHz, Chloroform-*d*) δ 161.7 (d, $^{1}J_{C\text{-F}}$ = 247.6 Hz), 146.3 (dd, $^{1}J_{C\text{-F}}$ = 250.7 Hz, $^{2}J_{C\text{-F}}$ = 12.1 Hz), 145.8 (dd, $^{1}J_{C\text{-F}}$ = 250.1 Hz, $^{2}J_{C\text{-F}}$ = 12.7 Hz), 135.6, 134.1 (d, $^{4}J_{C\text{-F}}$ = 1.4 Hz), 133.8, 132.5, 130.9 (d, $^{3}J_{C\text{-F}}$ = 9.1 Hz), 130.8 (d, $^{3}J_{C\text{-F}}$ = 8.7 Hz), 128.2, 128.2, 127.8, 127.8 (d, $^{4}J_{C\text{-F}}$ = 3.9 Hz), 127.2, 127.0, 126.9, 126.9 125.7 (q, $^{4}J_{C\text{-F}}$ = 3.5 Hz), 124.8 (d, $^{2}J_{C\text{-F}}$ = 12.6 Hz), 124.0, 122.8 (t, $^{4}J_{C\text{-F}}$ = 3.4 Hz), 122.5 (d, $^{2}J_{C\text{-F}}$ = 12.6 Hz), 119.5 (t, $^{4}J_{C\text{-F}}$ = 2.8 Hz), 117.0 (td, $^{4}J_{C\text{-F}}$ = 5.4, 2.6 Hz), 116.0 (t, $^{2}J_{C\text{-F}}$ = 23.8 Hz), 107.5 (d, $^{2}J_{C\text{-F}}$ = 23.0 Hz), **^{19}F NMR** (376 MHz, Chloroform-*d*) δ -111.7 , -142.8 (d, *J* = 19.0 Hz), -147.7 (d, *J* = 19.2 Hz). **HRMS** (ESI) (0.2 mM $AgNO_3$): calcd. for $[C_{26}H_{14}BrF_3+Ag]^+$, $[M+Ag]^+$: 568.9276; found: 568.9261. **IR (ATR):**

$\tilde{\nu}$ = 3057, 2362, 1610, 1516, 1470, 1297, 1229, 1174, 1091, 1030, 861, 834, 761, 741, 670 cm^{-1}. **Mp**: 173.3 – 175.3 °C.

<u>(*Z*)-3-(2-(1-bromonaphthalen-2-yl)vinyl)-1,2,7-trifluorophenanthrene (1v):</u> synthesized from **S27** (0.30 g, 1.15 mmol).

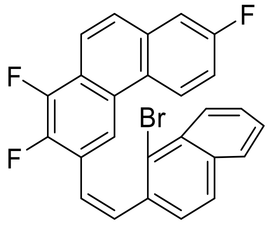

Re-precipitated in Et_2O. **Yield**: 49% (260 mg). White powder. ***R*f** 0.35 (cyclohexane/EtOAc 20/1); **^{1}H NMR** (400 MHz, Chloroform-*d*): δ 8.43 (d, *J* = 8.5 Hz, 1H), 7.96 (d, *J* = 4.4 Hz, 1H), 7.91 (d, *J* = 9.1 Hz, 1H), 7.74 (d, *J* = 8.1 Hz, 1H), 7.70 – 7.60 (m, 2H), 7.59 – 7.45 (m, 3H), 7.39 (dd, *J* = 9.2, 2.7 Hz, 1H), 7.25 (d, *J* = 5.4 Hz, 1H), 7.21 (d, *J* = 12.1 Hz, 1H), 7.04 (d, *J* = 12.0 Hz, 1H), 6.94 (td, *J* = 8.7, 2.7 Hz, 1H). **^{13}C NMR** (126 MHz, Chloroform-*d*) δ 161.4 (d, $^{1}J_{C\text{-}F}$ = 247.5 Hz), 146.0 (dd, $^{1}J_{C\text{-}F}$ = 250.4 Hz, $^{2}J_{C\text{-}F}$ = 12.7 Hz), 146.0 (dd, $^{1}J_{C\text{-}F}$ = 250.5 Hz, $^{2}J_{C\text{-}F}$ = 12.0 Hz), 135.9, 134.2 (d, $^{4}J_{C\text{-}F}$ = 1.4 Hz), 134.0, 133.1 (d, $^{3}J_{C\text{-}F}$ = 9.0 Hz), 132.7, 128.3, 127.9, 127.9, 127.8 (t, $^{4}J_{C\text{-}F}$ = 1.8 Hz), 127.4, 127.3, 127.1, 126.3 (t, $^{4}J_{C\text{-}F}$ = 3.1 Hz), 126.0 (d, $^{4}J_{C\text{-}F}$ = 1.3 Hz), 125.3 (d, $^{2}J_{C\text{-}F}$ = 12.5 Hz), 124.8 (d, $^{3}J_{C\text{-}F}$ = 8.8 Hz), 124.3, 123.1 (t, $^{4}J_{C\text{-}F}$ = 3.6 Hz), 121.9 (d, $^{2}J_{C\text{-}F}$ = 12.8 Hz), 119.3 (t, $^{3}J_{C\text{-}F}$ = 5.6 Hz), 119.2 (t, $^{4}J_{C\text{-}F}$ = 2.9 Hz), 116.4 (d, $^{2}J_{C\text{-}F}$ = 23.7 Hz), 113.0 (d, $^{2}J_{C\text{-}F}$ = 20.6 Hz). **^{19}F NMR** (376 MHz, Chloroform-*d*) δ -114.3 (d, *J* = 1.9 Hz), -144.1 (dd, *J* = 19.3, 2.2 Hz), -147.8 (d, *J* = 19.1 Hz). **HRMS** (ESI) (0.2 mM $AgNO_3$): calcd. for $[C_{26}H_{14}BrF_3+Ag]^+$, $[M+Ag]^+$: 568.9276; found: 568.9265. **IR (ATR):** $\tilde{\nu}$ = 3062, 2363, 1622, 1472, 1302, 1254, 1145, 1030, 959, 871, 781, 632 cm^{-1}. **Mp**: 169.3 – 171.1 °C.

<u>(*Z*)-3-(2-(1-bromonaphthalen-2-yl)vinyl)-1,2-difluoro-7-methoxyphenanthrene (1w):</u> synthesized from **S25** (0.30 g, 1.1 mmol).

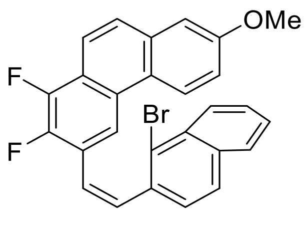

Re-precipitated in Et_2O. **Yield**: 40% (210 mg). White powder. ***R*f** 0.25 (cyclohexane/EtOAc 20/1); **^{1}H NMR** (500 MHz, Chloroform-*d*): δ 8.43 (d, *J* = 8.6 Hz, 1H), 7.96 (d, *J* = 5.9 Hz, 1H), 7.88 (d, *J* = 9.0 Hz, 1H), 7.73 (d, *J* = 8.1 Hz, 1H), 7.68 – 7.61 (m, 2H), 7.57 – 7.49 (m, 2H), 7.47 (d, *J* = 9.0 Hz, 1H), 7.27 (d, *J* = 8.5 Hz, 1H), 7.20 (d, *J* = 12.1 Hz, 1H), 7.14 (d, *J* = 2.7 Hz, 1H), 7.05 (d, *J* = 12.0 Hz, 1H), 6.86 (dd, *J* = 9.1, 2.7 Hz, 1H), 3.88 (s, 3H). **^{13}C NMR** (151 MHz, Chloroform-*d*) δ 158.5, 146.0 (dd, $^{1}J_{C\text{-}F}$ = 249.6 Hz, $^{2}J_{C\text{-}F}$ = 12.7 Hz), 145.6 (dd, $^{1}J_{C\text{-}F}$ = 248.4 Hz, $^{2}J_{C\text{-}F}$ = 12.2 Hz), 135.9, 134.0, 133.8 (d, $^{4}J_{C\text{-}F}$ = 1.3 Hz), 133.1, 132.7, 128.3, 128.2 (d, $^{4}J_{C\text{-}F}$ = 2.1 Hz), 127.8, 127.8, 127.4, 127.4, 127.0, 126.7 (t, $^{4}J_{C\text{-}F}$ = 3.0 Hz), 124.9 (d, $^{2}J_{C\text{-}F}$ = 12.5 Hz), 124.2, 124.0, 123.6 (t, $^{4}J_{C\text{-}F}$ = 1.9 Hz), 123.4 (t, $^{4}J_{C\text{-}F}$ = 3.5 Hz), 121.3 (d, $^{2}J_{C\text{-}F}$ = 12.7 Hz), 118.9 (dd, $^{4}J_{C\text{-}F}$ = 4.2, 2.4 Hz) 118.6 (t, $^{4}J_{C\text{-}F}$ = 5.4 Hz), 118.0, 108.8, 55.6. **^{19}F NMR**

(376 MHz, Chloroform-*d*) δ -145.5 (d, *J* = 19.3 Hz), -148.4 (d, *J* = 19.3 Hz). **HRMS** (ESI): calcd. for $[C_{27}H_{17}BrF_2O]^+$, $[M]^+$: 474.0425; found: 474.0426. **IR (ATR):** $\widetilde{\nu}$ = 2959, 2362, 1613, 1522, 1473, 1259, 1211, 1171, 1041, 855, 819, 740, 674 cm^{-1}. **Mp**: 149.1 – 151.1 °C.

(*Z*)-5-bromo-6-(2-(1,2-difluorophenanthren-3-yl)vinyl)quinoline (1x): synthesized from **S24** (0.50 g, 2.06 mmol).

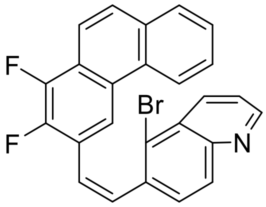

Yield: 92% (843 mg). Yellow solid. ***R*f** 0.25 (cyclohexane/EtOAc 5/1); **^{1}H NMR** (400 MHz, DMSO-d_6) δ 8.96 (dd, *J* = 4.2, 1.6 Hz, 1H), 8.68 (d, *J* = 8.4 Hz, 1H), 8.37 (d, *J* = 6.2 Hz, 1H), 8.07 (d, *J* = 8.4 Hz, 1H), 8.02 – 7.95 (m, 2H), 7.91 (d, *J* = 9.1 Hz, 1H), 7.81 (d, *J* = 8.8 Hz, 1H), 7.74 (dd, *J* = 8.6, 4.2 Hz, 1H), 7.62 (t, *J* = 7.5 Hz, 1H), 7.54 – 7.41 (m, 2H), 7.27 (d, *J* = 12.0 Hz, 1H), 7.19 (d, *J* = 12.0 Hz, 1H). **^{13}C NMR** (151 MHz, DMSO-d_6) δ 151.5, 147.6, 145.4 (dd, $^1J_{C\text{-}F}$ = 248.4 Hz, $^2J_{C\text{-}F}$ = 12.6 Hz), 144.9 (dd, $^1J_{C\text{-}F}$ = 247.7 Hz, $^2J_{C\text{-}F}$ = 12.4 Hz), 136.2, 134.9, 133.5, 131.2, 130.3, 129.2, 129.1, 128.8, 128.4, 127.9, 127.6, 127.4, 126.2, 124.5, 124.5 (d, $^2J_{C\text{-}F}$ = 13.1 Hz), 123.3, 122.3, 122.2, 121.5 (d, $^2J_{C\text{-}F}$ = 12.5 Hz), 119.9, 117.2. **^{19}F NMR** (376 MHz, DMSO-d_6) δ -142.6 (d, *J* = 20.4 Hz), -148.2 (d, *J* = 20.5 Hz). **HRMS** (APPI/LTQ-Orbitrap): calcd. for $[C_{25}H_{15}BrF_2N+H]^+$, $[M+H]^+$: 446.0350; found: 446.0359. **IR (ATR):** $\widetilde{\nu}$ = 3033, 2359, 2253, 1976, 1642, 1551, 1464, 1333, 1292,1027, 975,880, 837, 808, 756 cm^{-1}. **Mp**: 177.3 – 178.7°C.

General Procedure 5 (GP5):

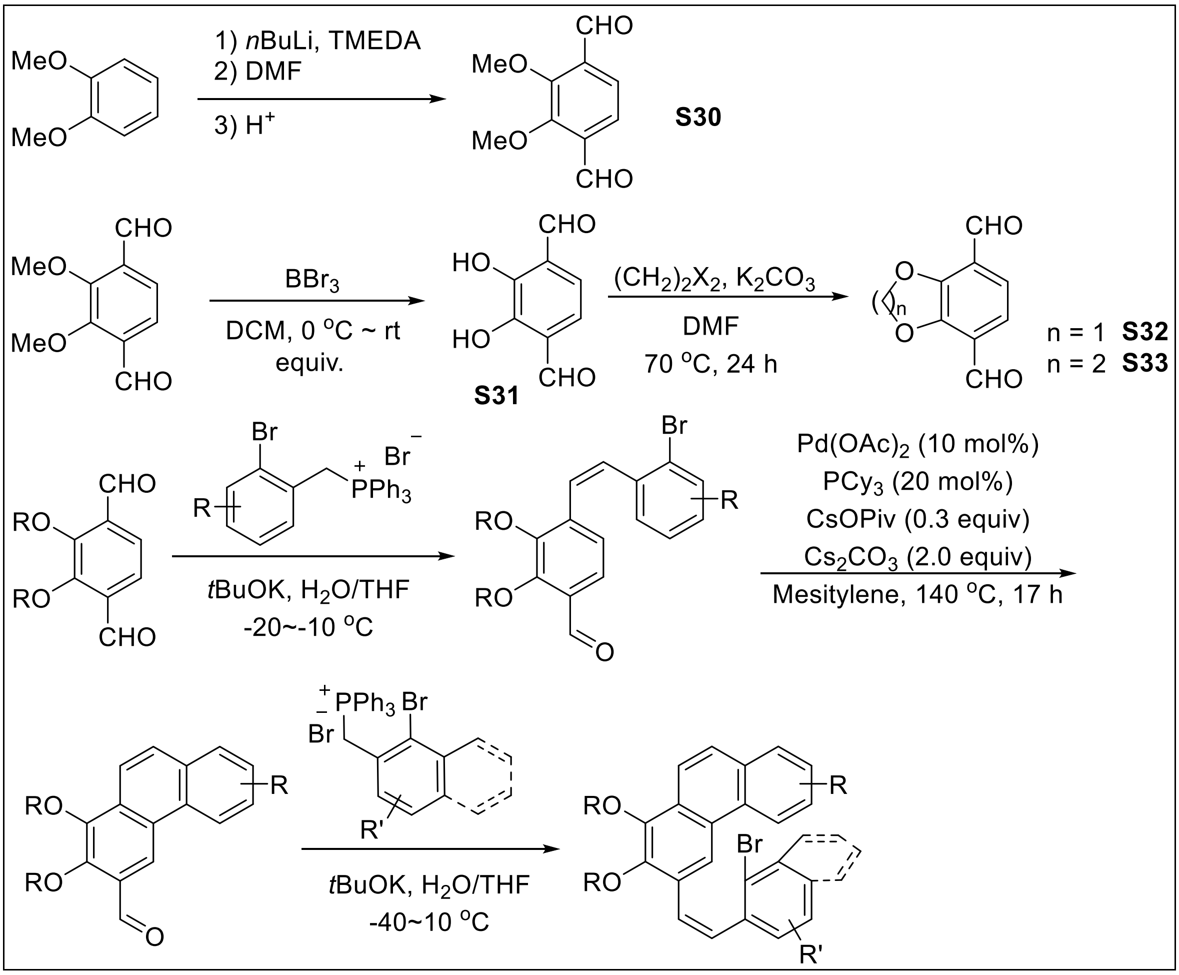

2,3-dimethoxyterephthalaldehyde (S30): synthesized according to the following procedure. Known conpound.[13]

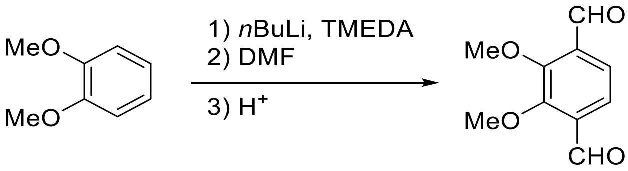

A round-bottom flask was charged with 1,2-dimethoxybenzene (6.91 g, 50.0 mmol), anhydrous Et_2O (100 mL), and tetramethylethylenediamine (TMEDA, 37.5 mL, 250 mmol), followed by dropwise addition of *n*BuLi (100 mL, 250 mmol, 2.5 M solution in hexane) at 0 °C. The mixture turned yellow, and white precipitate was formed. The mixture was warmed to room temperature and further refluxed for 20 h, cooled with ice-water bath, and DMF (30 mL, 388 mmol) was added dropwise. The reaction mixture was warmed to room temperature for 15 min, then poured into ice-water in one portion. The mixture was extracted with Et_2O (3 × 200 mL), and the combined organic phases were dried over anhydrous Na_2SO_4 and finally evaporated and purified by column chromatography (cyclohexane/EtOAc 5:1) to afford 2,3-dimethoxyterephthal-aldehyde as a yellow solid (4.1 g, 21.1 mmol, 42%).

^{1}H NMR (400 MHz, Chloroform-*d*): δ 10.44 (s, 2H), 7.63 (s, 2H), 4,02 (s, 6H).

2,3-dihydroxyterephthalaldehyde (S31): synthesized according to literature reported procedure from **S30** (6.5 g, 33.5 mmol). Known compound.[15]

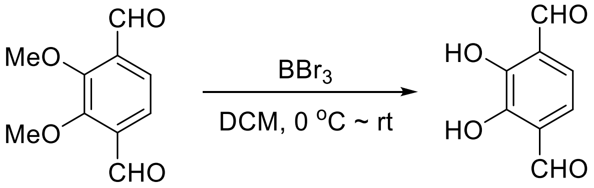

Yield: >99% (5.68 g). Brown crystalline solid. **^{1}H NMR** (400 MHz, Chloroform-*d*) δ 10.94 (s, 2H), 10.03 (s, 2H), 7.28 (s, 2H).

benzo[*d*][1,3]dioxole-4,7-dicarbaldehyde (S32): synthesized according to literature reported procedure from **S31** (1.0 g, 6.02 mmol). Known compound.[15]

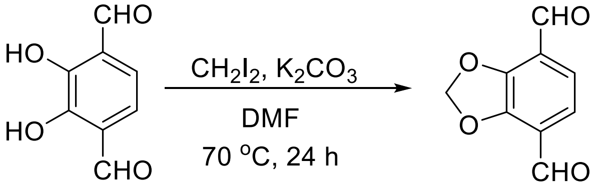

Yield: 73% (778 mg). Yellow solid. **^{1}H NMR** (400 MHz, Chloroform-*d*) δ 10.20 (s, 2H), 7.38 (s, 2H), 6.34 (s, 2H).

2,3-dihydrobenzo[*b*][1,4]dioxine-5,8-dicarbaldehyde (S33): synthesized according to literature reported procedure from **S31** (1.0 g, 6.02 mmol). Known compound.[15]

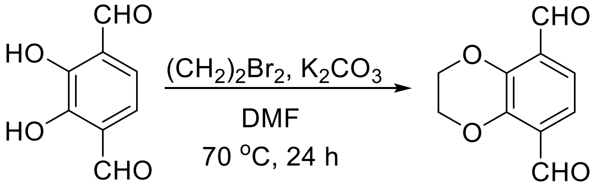

Yield: 94% (1.08 g). Yellow crystalline solid. **^{1}H NMR** (400 MHz, Chloroform-*d*) δ 10.42 (s, 2H), 7.41 (s, 2H), 4.49 (s, 4H).

Wittig reaction:[12]

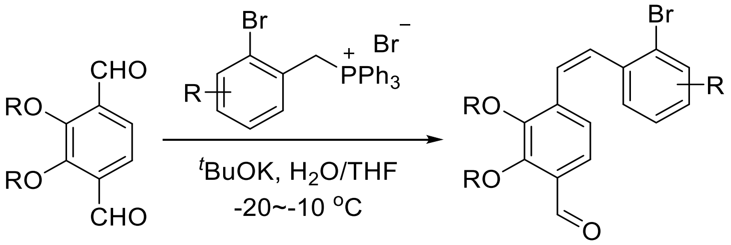

2-Bromobenzyl bromide phosphonium salt (1.1 equiv) was treated with potassium *tert*-butoxide (1.2 equiv) in THF (0.25 M)/H_2O (2.5 M) at -20 °C and stirred for 15 min. Dicarbaldehyde (5.0 mmol) was added. The mixture was stirred and warmed slowly to -10 °C in 1 h, and kept at -10 °C. The reaction was monitored by TLC. The resulting mixture was quenched with water and extracted with EtOAc. The extract was dried over anhydrous Na_2SO_4,

volatile material was removed under reduced pressure and the crude residue was purified by column chromatography.

(*Z*)-4-(2-bromostyryl)-2,3-dimethoxybenzaldehyde (S34): synthesized from **S30** (1.20 g, 6.18 mmol).

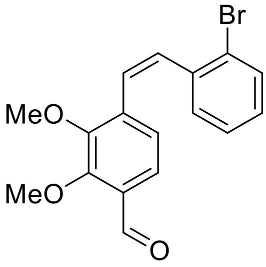

Yield: 87% (2.1 g, *Z/E* 15:1). Light yellow crystalline solid. R_f 0.35 (cyclohexane/EtOAc 10/1); analysis for *Z*-(**S33**): **^{1}H NMR** (500 MHz, Chloroform-*d*): δ 10.32 (d, *J* = 0.8 Hz, 1H), 7.62 – 7.57 (m, 1H), 7.28 (d, *J* = 8.2 Hz, 1H), 7.13 – 7.04 (m, 3H), 6.84 (s, 2H), 6.79 (dd, *J* = 8.2, 0.8 Hz, 1H), 3.99 (s, 3H), 3.90 (s, 3H). **^{13}C NMR** (101 MHz, Chloroform-*d*) δ 189.6, 156.6, 151.6, 138.2, 137.4, 133.0, 132.8, 130.7, 129.4, 129.3, 127.2, 125.8, 125.4, 124.0, 122.4, 62.4, 61.1. **HRMS** (ESI): calcd. for $[C_{17}H_{15}BrO_3+Na]^+$, $[M+Na]^+$: 369.0097; found: 369.0095. **IR (ATR):** $\tilde{\nu}$ = 2937, 2361, 1686, 1594, 1560, 1457, 1410, 1377, 1253, 1048, 1020, 765, 631 cm^{-1}. **Mp**: 56.9 – 58.9 °C.

(*Z*)-7-(2-bromostyryl)benzo[*d*][1,3]dioxole-4-carbaldehyde (S35): synthesized from **S32** (1.35 g, 7.58 mmol).

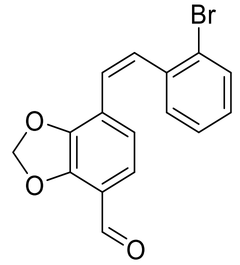

Yield: 78% (1.95 g, *Z/E* 5:1). Yellow powder. R_f 0.25 (cyclohexane/EtOAc 10/1); analysis for *Z*-(**S34**): **^{1}H NMR** (500 MHz, Chloroform-*d*): δ 10.03 (s, 1H), 7.65 – 7.55 (m, 1H), 7.20 – 7.09 (m, 3H), 7.05 (d, *J* = 8.4 Hz, 1H), 6.85 (d, *J* = 12.0 Hz, 1H), 6.64 (d, *J* = 12.0 Hz, 1H), 6.63 (d, *J* = 8.4 Hz, 1H), 6.01 (s, 2H).

(*Z*)-8-(2-bromostyryl)-2,3-dihydrobenzo[*b*][1,4]dioxine-5-carbaldehyde (S36): synthesized from **S33** (1.92 g, 10.0 mmol),

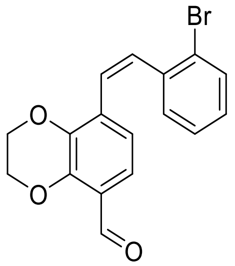

Yield: 83% (2.87 g, only *Z*). Yellow crystalline solid. ***R*f** 0.15 (cyclohexane/EtOAc 10/1); **^{1}H NMR** (500 MHz, Chloroform-*d*): δ 10.29 (s, 1H), 7.63 – 7.55 (m, 1H), 7.13 (d, *J* = 8.2 Hz, 1H), 7.12 – 7.04 (m, 3H), 6.81 (d, *J* = 12.1 Hz, 1H), 6.75 (d, *J* = 12.1 Hz, 1H), 6.62 (d, *J* = 8.2 Hz, 1H), 4.39 – 4.32 (m, 2H), 4.28 – 4.21 (m, 2H). **^{13}C NMR** (126 MHz, Chloroform-*d*) δ 188.9, 146.6, 141.7, 137.7, 132.8, 132.7, 131.9, 130.7, 129.2, 127.2, 125.3, 124.1, 123.9, 122.0, 120.0, 64.5, 64.1. **HRMS** (ESI): calcd. for $[C_{17}H_{13}BrO_3+Na]^+$, $[M+Na]^+$: 366.9940; found: 366.9939. **IR (ATR):** $\tilde{\nu}$ = 2986, 2362, 1681, 1602, 1438, 1374, 1256, 1088, 780, 655 cm^{-1}. **Mp**: 127.8 – 129.8 °C.

C–H activation reaction:

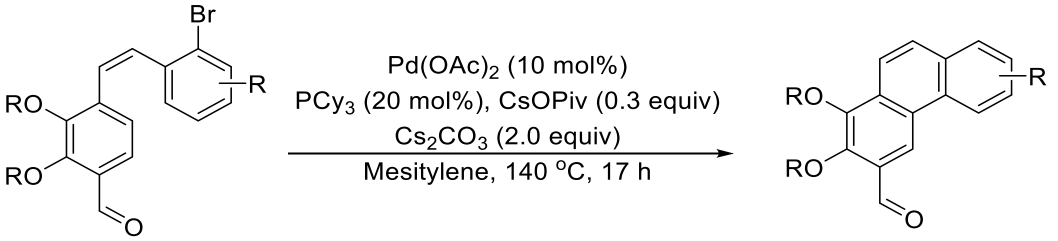

To an oven-dried 250 mL flask, under ambient air, was added (*Z*)-(2-bromostyryl)benzodioxole-carbaldehyde (1 equiv) and Cs_2CO_3 (2.0 equiv). The flask was flushed with Ar gas 3 times and then taken to a glovebox, where Pd_2dba_3 (10 mol%), PCy_3 (20 mol%) and CsOPiv (3.51 mmol, 0.3 equiv) were added. Mesitylene (80 mL) was added to the mixture out of the glovebox, followed by flushing with Ar gas 3 times. The reaction mixture was then heated at 140 °C for 17 h. The reaction mixture was filtered through a plug of silica gel (eluted with EtOAc) and concentrated under reduced pressure. The resulting crude product was purified by column chromatography on silica gel (cyclohexane/EtOAc).

<u>1,2-dimethoxyphenanthrene-3-carbaldehyde (S37)</u>: known compound, synthesized from **S34** (2.0 g, 5.76 mmol).[12]

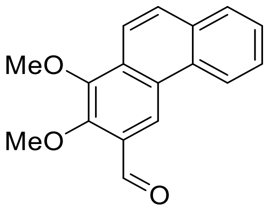

Yield: 65% (1.0 g). Yellow solid. ***R*f** 0.30 (cyclohexane/EtOAc 10/1); **^{1}H NMR** (400 MHz, Chloroform-*d*): δ 10.58 (s, 1H), 8.93 (s, 1H), 8.66 (d, *J* = 8.2 Hz, 1H), 8.04 (d, *J* = 9.1 Hz, 1H), 7.88 – 7.76 (m, 2H), 7.69 – 7.60 (m, 1H), 7.63 – 7.52 (m, 1H), 4.13 (s, 3H), 4.05 (s, 3H).

<u>phenanthro[1,2-*d*][1,3]dioxole-11-carbaldehyde (S38):</u> synthesized from **S35** (1.95 g, 5.88 mmol).

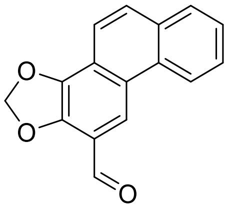

Yield: 60% (882 mg). Yellow crystalline solid. ***R*f** 0.20 (cyclohexane/EtOAc 10/1); **^{1}H NMR** (500 MHz, Chloroform-*d*): δ 10.33 (s, 1H), 8.66 (s, 1H), 8.62 (d, *J* = 8.1 Hz, 1H), 7.85 (d, *J* = 7.1 Hz, 1H), 7.79 (d, *J* = 9.0 Hz, 1H), 7.72 (d, *J* = 8.6 Hz, 1H), 7.69 (ddd, *J* = 8.4, 7.1, 1.4 Hz, 1H), 7.60 (ddd, *J* = 8.1, 7.0, 1.1 Hz, 1H), 6.36 (s, 2H). **^{13}C NMR** (126 MHz, Chloroform-*d*) δ 188.9, 144.7, 144.4, 131.4, 130.9, 130.9, 129.4, 128.2, 127.1, 126.0, 122.9, 121.1, 119.2, 118.5, 118.2, 103.5. **HRMS** (ESI): calcd. for $[C_{16}H_{10}O_3+Na]^+$, $[M+Na]^+$: 273.0522; found: 273.0525. **IR (ATR):** $\tilde{\nu}$ = 2904, 1746, 1690, 1642, 1455, 1320, 1274, 1127, 1073, 818, 752 cm^{-1}. **Mp**: 207.7 – 209.7 °C.

2,3-dihydrophenanthro[1,2-*b*][1,4]dioxine-12-carbaldehyde (S39): synthesized from **S36** (2.87 g, 8.31 mmol).

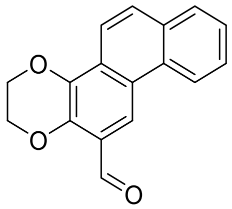

Yield: 63% (1.38 g). Yellow powder. ***R*f** 0.15 (cyclohexane/EtOAc 10/1); **^{1}H NMR** (500 MHz, Chloroform-*d*): δ 10.56 (s, 1H), 8.77 (s, 1H), 8.67 (d, *J* = 8.3 Hz, 1H), 8.02 (d, *J* = 9.0 Hz, 1H), 7.87 (d, *J* = 7.9 Hz, 1H), 7.82 (d, *J* = 9.1 Hz, 1H), 7.67 (ddd, *J* = 8.4, 7.0, 1.4 Hz, 1H), 7.59 (ddd, *J* = 8.0, 7.1, 1.2 Hz, 1H), 4.57 – 4.53 (m, 2H), 4.53 – 4.49 (m, 2H). **^{13}C NMR** (126 MHz, Chloroform-*d*) δ 189.8, 141.8, 138.6, 131.4, 131.0, 130.1, 129.0, 127.7, 127.5, 126.8, 124.5, 124.2, 122.9, 119.1, 116.9, 64.7, 64.6. **HRMS** (ESI): calcd. for $[C_{17}H_{12}O_3+Na]^+$, $[M+Na]^+$: 287.0679; found: 287.0680. **IR (ATR):** $\tilde{\nu}$ = 2987, 2361, 1680, 1613, 1460, 1350, 1283, 1112, 1088, 820, 743 cm^{-1}. **Mp**: 201.1 – 203.1 °C.

Wittig reaction:[12]

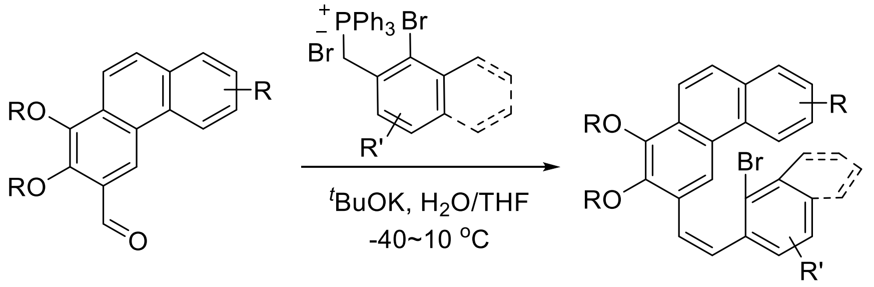

2-Bromobenzyl bromide phosphonium salt (1.1 equiv) was treated with potassium *tert*-butoxide (1.2 equiv) in THF (0.25 M)/H_2O (2.5 M) at -40 ^{o}C and stirred for 15 min. phenanthrodioxole-carbaldehyde (5.0 mmol) was added. The mixture was stirred and warmed slowly to 10 ^{o}C in 5 h, and kept under 10 ^{o}C. The reaction was monitored by TLC. The resulting mixture was quenched with water and extracted with EtOAc, washed with brine and dried over anhydrous Na_2SO_4. The crude product was purified by column chromatography (cyclohexane/EtOAc 99/1), re-precipitation or recrystallization.

(*Z*)-12-(2-bromo-3-methylstyryl)-2,3-dihydrophenanthro[1,2-*b*][1,4]dioxine (1o): synthesized from **S39** (0.80 g, 3.03 mmol).

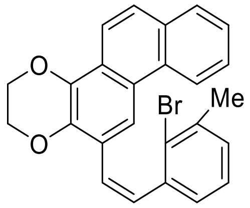

Yield: 77% (1.0 g). White crystalline solid. ***R*f** 0.23 (cyclohexane/EtOAc 20/1); **^{1}H NMR** (500 MHz, Chloroform-*d*): δ 8.00 – 7.90 (m, 3H), 7.85 – 7.75 (m, 1H), 7.64 (d, *J* = 9.0 Hz, 1H), 7.55 – 7.41 (m, 2H), 7.10 (d, *J* = 7.2 Hz, 1H), 7.05 (d, *J* = 7.1 Hz, 1H), 6.99 (d, *J* = 12.1 Hz, 1H), 6.93 – 6.83 (m, 2H), 4.48 – 4.40 (m, 2H), 4.37 – 4.25 (m, 2H), 2.52 (s, 3H). **^{13}C NMR** (126 MHz, Chloroform-*d*) δ 139.2, 138.8, 138.6, 138.0, 132.1, 131.3, 130.3, 129.5, 128.6, 128.1, 127.0, 126.7, 126.7, 126.4, 125.9, 125.6, 125.5, 124.3, 122.9, 122.4, 119.2, 116.4, 64.5, 64.5, 23.9. **HRMS** (ESI): calcd. for $[C_{25}H_{19}BrO_2]^+$, $[M]^+$: 430.0563; found: 430.0556. **IR (ATR):** $\tilde{\nu}$ = 2978, 1616, 1461, 1354, 1288, 1096, 1024, 968, 818, 752, 663 cm^{-1}. **Mp**: 126.3 – 128.3 °C.

<u>(*Z*)-11-(2-bromo-3-methylstyryl)phenanthro[1,2-*d*][1,3]dioxole (1p):</u> synthesized from **S38** (0.476 g, 1.90 mmol).

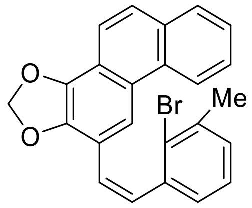

Yield: 62% (490 mg). Light yellow crystalline solid. ***R*f** 0.30 (cyclohexane/EtOAc 20/1); **^{1}H NMR** (500 MHz, Chloroform-*d*): δ 7.96 (d, *J* = 0.7 Hz, 1H), 7.96 – 7.89 (m, 1H), 7.79 – 7.73 (m, 1H), 7.64 (dd, *J* = 8.9, 0.8 Hz, 1H), 7.59 (dd, *J* = 8.9, 0.6 Hz, 1H), 7.50 – 7.44 (m, 2H), 7.17 (ddt, *J* = 7.4, 1.5, 0.7 Hz, 1H), 7.12 (ddt, *J* = 7.7, 1.7, 0.7 Hz, 1H), 6.99 (t, *J* = 7.6 Hz, 1H), 6.91 (d, *J* = 11.9 Hz, 1H), 6.87 (dd, *J* = 12.0, 0.6 Hz, 1H), 6.05 (s, 2H), 2.53 (s, 3H). **^{13}C NMR** (126 MHz, Chloroform-*d*) δ 143.0, 142.6, 139.0, 138.8, 132.7, 131.3, 130.6, 129.8, 129.0, 128.0, 127.5, 127.1, 127.0, 126.4, 126.1, 125.6, 124.0, 122.5, 119.0, 118.7, 116.9, 116.5, 102.5, 23.9. **HRMS** (ESI): calcd. for $[C_{24}H_{17}BrO_2]^+$, $[M]^+$: 416.0406; found: 416.0407. **IR (ATR):** $\tilde{\nu}$ = 2884, 2362, 1647, 1436, 1345, 1104, 1070, 1025, 930, 814, 750, 655 cm^{-1}. **Mp**: 159.0 – 161.0 °C.

<u>(*Z*)-3-(2-(1-bromonaphthalen-2-yl)vinyl)-1,2-dimethoxyphenanthrene (1s):</u> synthesized from **S37** (0.10 g, 0.376 mmol).

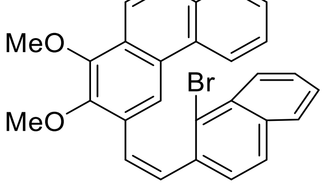

Yield: 37% (65 mg). Thick yellow liquid. ***R*f** 0.10 (cyclohexane/EtOAc 10/1); **^{1}H NMR** (400 MHz, Chloroform-d) δ 8.44 (d, *J* = 8.5 Hz, 1H), 8.11 (s, 1H), 8.01 (d, *J* = 9.1 Hz, 1H), 7.75

(dd, J = 7.9, 1.3 Hz, 1H), 7.71 (d, J = 8.1 Hz, 1H), 7.68 – 7.59 (m, 3H), 7.54 – 7.44 (m, 2H), 7.40 (ddd, J = 8.0, 7.0, 1.1 Hz, 1H), 7.31 (d, J = 8.5 Hz, 1H), 7.23 – 7.09 (m, 3H), 4.04 (s, 3H), 4.03 (s, 3H). **^{13}C NMR** (151 MHz, Chloroform-*d*) δ 148.9, 148.2, 136.5, 133.8, 132.8, 131.8, 131.6, 130.1, 129.9, 128.5, 128.3, 127.8, 127.6, 127.5, 127.4, 127.4, 127.3, 126.9, 126.8, 126.8, 126.8, 126.3, 124.2, 122.5, 120.1, 120.1, 61.6, 61.3. **HRMS** (APPI/LTQ-Orbitrap): calcd. for $[C_{28}H_{21}BrO_2]^+$, $[M]^+$: 468.0719; found: 468.0727. **IR (ATR):** $\tilde{\nu}$ = 2931, 1597, 1462, 1419, 1357, 1279, 1230, 1063, 1025, 970, 886, 824, 789, 755, 636 cm^{-1}.

(*Z*)-12-(2-(1-bromonaphthalen-2-yl)vinyl)-2,3-dihydrophenanthro[1,2-*b*][1,4]dioxine (1t): synthesized from **S39** (0.125 g, 0.473 mmol).

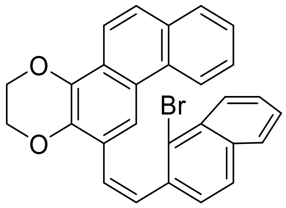

Yield: 70% (155 mg). Yellow crystalline solid. ***R*f** 0.20 (cyclohexane/EtOAc 20/1); **^{1}H NMR** (500 MHz, Chloroform-*d*): δ 8.43 (dd, J = 8.5, 1.1 Hz, 1H), 8.05 – 7.92 (m, 2H), 7.75 (ddd, J = 7.9, 1.4, 0.7 Hz, 1H), 7.71 (tdd, J = 8.0, 1.2, 0.5 Hz, 2H), 7.65 – 7.59 (m, 2H), 7.50 (ddd, J = 8.0, 6.9, 1.2 Hz, 1H), 7.45 (dd, J = 8.5, 0.7 Hz, 1H), 7.39 (ddd, J = 8.0, 7.0, 1.1 Hz, 1H), 7.31 (d, J = 8.5 Hz, 1H), 7.22 (ddd, J = 8.4, 7.0, 1.4 Hz, 1H), 7.15 – 7.07 (m, 2H), 4.46 – 4.39 (m, 2H), 4.35 – 4.25 (m, 2H). **^{13}C NMR** (126 MHz, Chloroform-*d*) δ 138.8, 138.1, 136.7, 133.8, 132.7, 131.9, 131.3, 130.2, 128.6, 128.3, 127.8, 127.5, 127.3, 127.3, 126.9, 126.7, 126.7, 126.3, 125.9, 125.5, 124.4, 124.0, 123.1, 122.4, 119.1, 116.7, 64.6, 64.5. **HRMS** (ESI): calcd. for $[C_{28}H_{19}BrO_2+Na]^+$, $[M+Na]^+$: 489.0461; found: 489.0455. **IR (ATR):** $\tilde{\nu}$ = 2978, 2361, 1616, 1464, 1354, 1288, 1095, 973, 906, 819, 751, 643 cm^{-1}. **Mp**: 85.8 – 87.8 °C.

General procedure for the enantioselective C–H arylation (GP6):

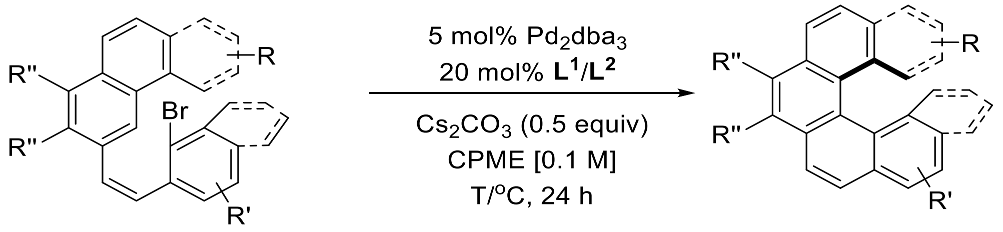

To an oven-dried 5 mL microwave vial under ambient air was added (*Z*)-2-bromostyrene (0.1 mmol, 1 equiv) (**1**) and (*R*)-$\mathbf{L^1}$ (12.9 mg, 20 µmol, 20 mol%) or (*R*)-$\mathbf{L^2}$ (16.3 mg, 20 µmol, 20 mol%). The vial was sealed with a septum and put under vacuum, followed by flushing 3 times with Ar gas. The vial was then transferred to a glovebox, and Pd_2dba_3 (4.58 mg, 5 µmol, 5 mol%), dry and ground Cs_2CO_3 (16.5 mg, 0.05 mmol, 0.5 equiv) was added. Cyclopentylmethylether (1.0 mL, 0.1 M) was added to the mixture before the vial was sealed and taken out of the glovebox. The reaction mixture was heated at 120 °C or 140 °C for 24 hours. The reaction mixture was then filtered through a plug of silica gel (eluted with EtOAc) and concentrated under reduced pressure. The resulting crude product was purified by column chromatography on silica gel.

1,6-dimethoxy-12-methylbenzo[*c*]phenanthrene (2a): synthesized according to **GP6**, run for 24 h at 120 °C, with L^1 as the ligand.

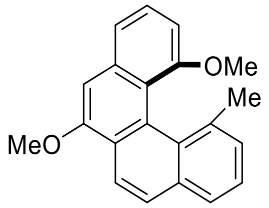

Yield: 93% (28 mg). Thick green liquid. ***R*f** 0.30 (pentane/EtOAc 20/1); **^{1}H NMR** (500 MHz, Chloroform-*d*): δ 8.23 (d, *J* = 8.6 Hz, 1H), 7.87 (d, *J* = 8.6 Hz, 1H), 7.82 – 7.76 (m, 1H), 7.55 – 7.46 (m, 3H), 7.38 – 7.29 (m, 1H), 7.15 (s, 1H), 6.82 (dd, *J* = 6.1, 2.8 Hz, 1H), 4.12 (s, 3H), 3.61 (s, 3H), 2.14 (s, 3H). **^{13}C NMR** (126 MHz, Chloroform-*d*) δ 156.7, 154.0, 138.2, 134.3, 132.9, 131.3, 127.7, 127.0, 126.7, 126.5, 125.8, 125.6, 124.7, 119.3, 118.6, 118.3, 103.8, 103.1, 56.0, 54.6, 22.5. **HRMS** (APPI/LTQ-Orbitrap): calcd. for $[C_{21}H_{18}O_2]^+$, $[M]^+$: 302.1301; found: 302.1302. **IR (ATR):** $\tilde{\nu}$ = 2930, 2362, 1616, 1516, 1459, 1346, 1256, 1154, 1047, 1019, 824, 780, 694 cm^{-1}. **$[\alpha]_D^{20}$**: –685.1 (c = 0.067, $CHCl_3$).

Chiral HPLC: (Chiralpak IA, 4.6 x 250 mm; n-heptane/i-PrOH 97/3, 0.5 mL/min, 293 nm; t_R(minor) = 10.0 min, t_R(major) = 11.3 min, 6.8:93.2 *er*.

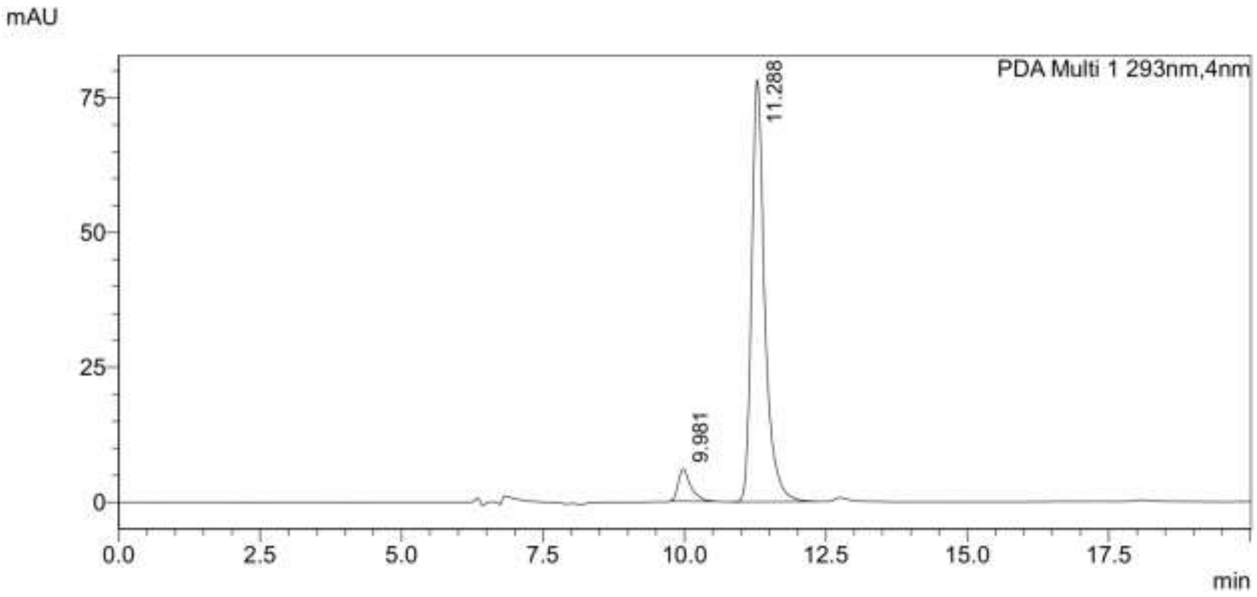

<Peak Table>

PDA Ch1 293nm

Peak#	Ret. Time	Area	Height	Area%
1	9.981	93031	6019	6.838
2	11.288	1267506	78286	93.162
Total		1360537	84305	100.000

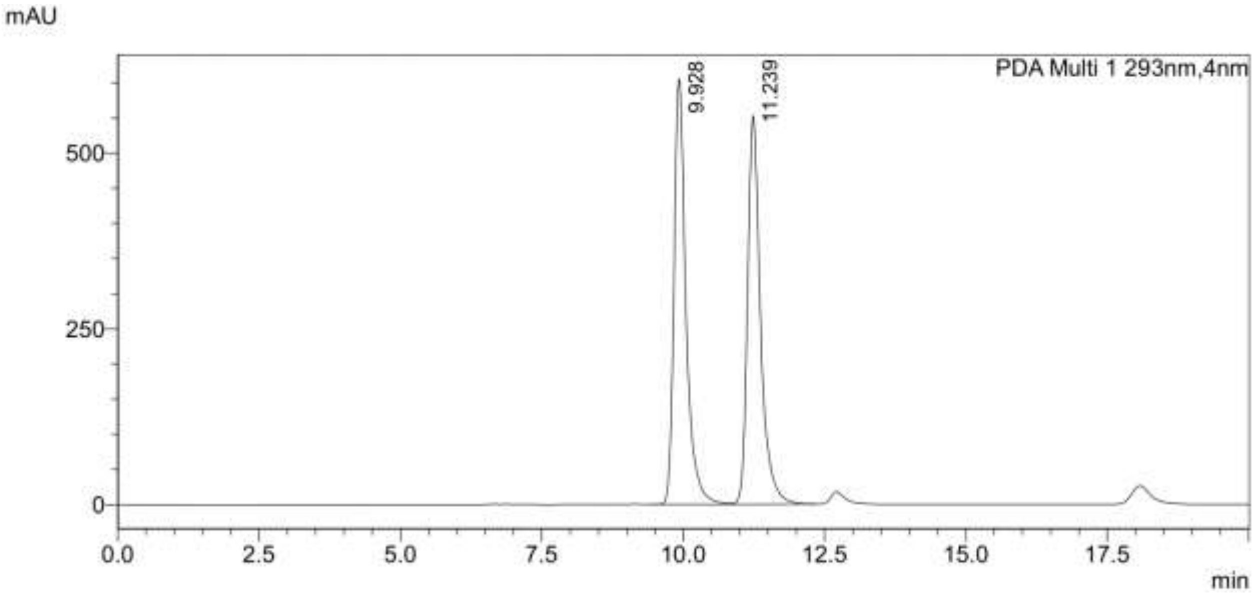

<Peak Table>

PDA Ch1 293nm

Peak#	Ret. Time	Area	Height	Area%
1	9.928	8882277	605292	50.147
2	11.239	8830377	552897	49.853
Total		17712654	1158189	100.000

12-isopropoxy-1,6-dimethoxybenzo[*c*]phenanthrene (2b): synthesized according to **GP6**, run for 24 h at 120 °C, with L^1 as the ligand.

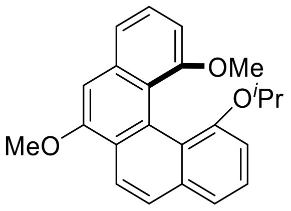

Yield: 92% (32 mg). White foam. R_f 0.28 (pentane/EtOAc 20/1); **^{1}H NMR** (500 MHz, Chloroform-*d*): δ 8.24 (dd, *J* = 8.7, 0.8 Hz, 1H), 7.85 (d, *J* = 8.6 Hz, 1H), 7.62 (dd, *J* = 8.0, 1.1 Hz, 1H), 7.54 – 7.40 (m, 3H), 7.10 (s, 1H), 7.02 (dd, *J* = 7.6, 0.9 Hz, 1H), 6.81 (dd, *J* = 7.7, 1.3 Hz, 1H), 4.11 (s, 3H), 3.70 (s, 3H), 3.61 (p, *J* = 6.0 Hz, 1H), 0.90 (d, *J* = 5.9 Hz, 3H), 0.40 (d, *J* = 6.1 Hz, 3H). **^{13}C NMR** (126 MHz, Chloroform-*d*) δ 158.8, 156.8, 153.5, 134.2, 134.1, 126.9, 126.8, 126.3, 125.6, 124.6, 124.0, 121.0, 119.4, 118.6, 118.3, 113.5, 104.1, 103.0, 72.9, 55.9, 54.7, 22.4, 21.5. **HRMS** (ESI): calcd. for $[C_{23}H_{22}O_3+Na]^+$, $[M+Na]^+$: 369.1461; found: 369.1462. **IR (ATR):** $\tilde{\nu}$ = 2972, 2362, 1617, 1563, 1514, 1459, 1326, 1258, 1154, 1130, 1019, 908, 822, 756, 624 cm^{-1}. **$[\alpha]_D^{20}$**: –854.0 (c = 1.0, $CHCl_3$).

Chiral HPLC: (Chiralpak IA, 4.6 x 250 mm; n-heptane/i-PrOH 97/3, 0.5 mL/min, 293 nm; t_R(minor) = 9.1 min, t_R(major) = 10.3 min, 4.0:96.0 *er*.

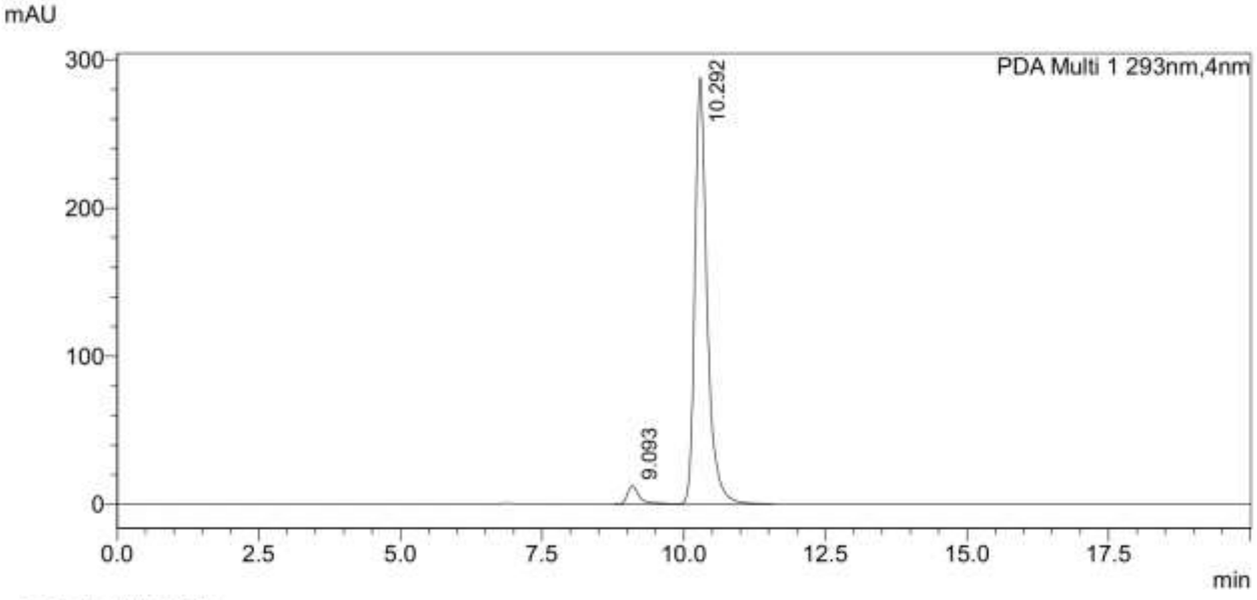

<Peak Table>

PDA Ch1 293nm

Peak#	Ret. Time	Area	Height	Area%
1	9.093	181516	12338	4.011
2	10.292	4344122	288071	95.989
Total		4525638	300410	100.000

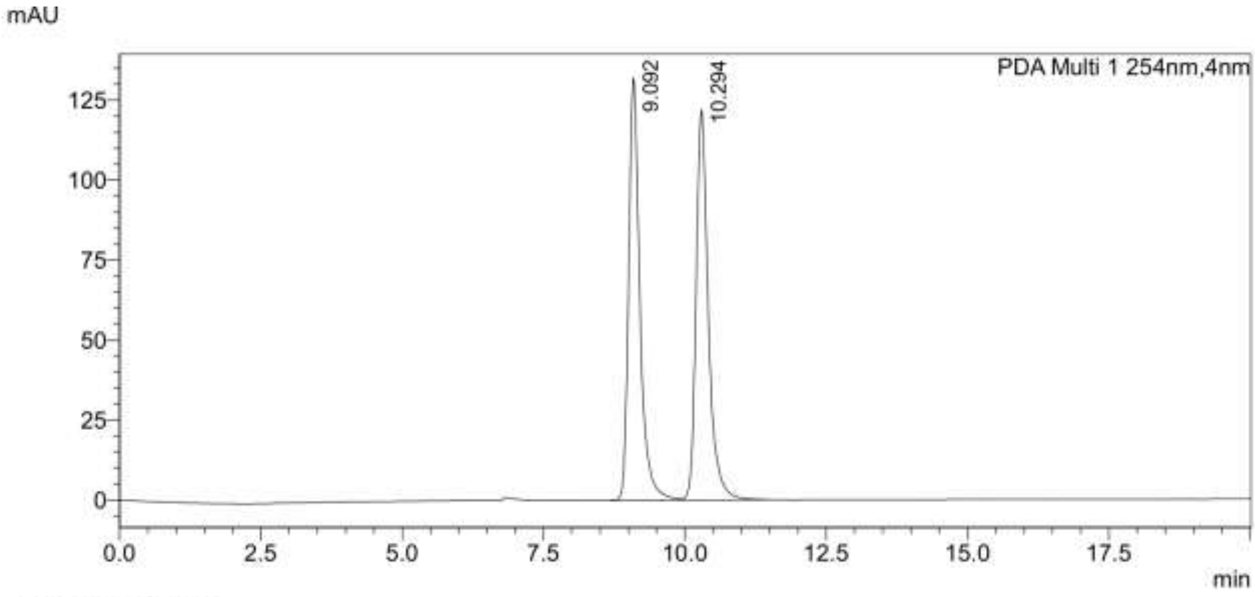

<Peak Table>

PDA Ch1 254nm

Peak#	Ret. Time	Area	Height	Area%
1	9.092	1829437	132004	49.954
2	10.294	1832823	121967	50.046
Total		3662260	253971	100.000

3-fluoro-7,12-dimethoxy-1-methylbenzo[*c*]phenanthrene (2c): synthesized according to **GP6**, run for 24 h at 120 °C, with L^1 as the ligand.

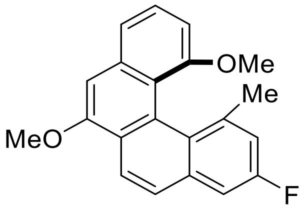

Yield: 94% (30 mg). Thick brown liquid. ***R*f** 0.25 (pentane/EtOAc 20/1); **^{1}H NMR** (500 MHz, Chloroform-*d*): δ 8.26 (dd, *J* = 8.6, 0.8 Hz, 1H), 7.80 (d, *J* = 8.7 Hz, 1H), 7.57 – 7.48 (m, 2H), 7.42 (dd, *J* = 9.0, 2.8 Hz, 1H), 7.14 (s, 1H), 7.09 (dd, *J* = 9.4, 2.5 Hz, 1H), 6.81 (dd, *J* = 4.9, 4.0 Hz, 1H), 4.12 (s, 3H), 3.63 (s, 3H), 2.15 (s, 3H). **^{13}C NMR** (126 MHz, Chloroform-*d*) δ 160.5 (d, $^1J_{C\text{-F}}$ = 245.7 Hz), 156.6, 154.0, 141.3 (d, $^3J_{C\text{-F}}$ = 7.9 Hz), 134.5, 133.9 (d, $^3J_{C\text{-F}}$ = 9.1 Hz), 128.3 (d, $^4J_{C\text{-F}}$ = 1.8 Hz), 127.2, 127.1 (d, $^3J_{C\text{-F}}$ = 4.3 Hz), 126.1 (d, $^4J_{C\text{-F}}$ = 1.8 Hz), 125.8, 119.9, 119.5, 118.0, 115.4 (d, $^2J_{C\text{-F}}$ = 23.3 Hz), 108.0 (d, $^2J_{C\text{-F}}$ = 20.0 Hz), 103.8, 103.0, 56.00, 54.6, 22.5 (d, $^1J_{C\text{-F}}$ = 1.8 Hz). **^{19}F NMR** (376 MHz, Chloroform-*d*) δ -117.3. **HRMS** (ESI): calcd. for $[C_{21}H_{17}FO_2]^+$, $[M]^+$: 320.1207; found: 320.1204. **IR (ATR):** $\tilde{\nu}$ = 2932, 2362, 1613, 1519, 1458, 1346, 1277, 1220, 1155, 1118, 1020, 973, 908, 856, 732, 638 cm^{-1}. **$[\alpha]_D^{20}$**: –530.1 (c = 1.0, $CHCl_3$).

Chiral HPLC: (Chiralpak IA, 4.6 x 250 mm; n-heptane/i-PrOH 97/3, 0.5 mL/min, 293 nm; t_R(minor) = 17.0 min, t_R(major) = 20.5 min, 4.7:95.3 *er*.

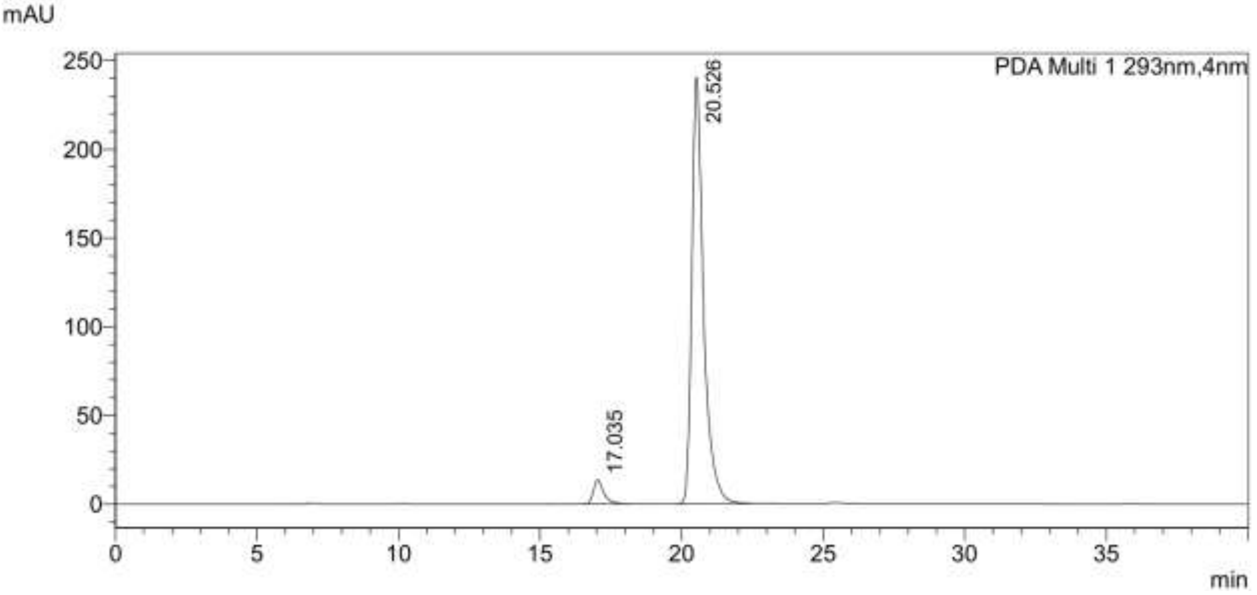

<Peak Table>

PDA Ch1 293nm

Peak#	Ret. Time	Area	Height	Area%
1	17.035	342606	13643	4.703
2	20.526	6942720	240487	95.297
Total		7285327	254130	100.000

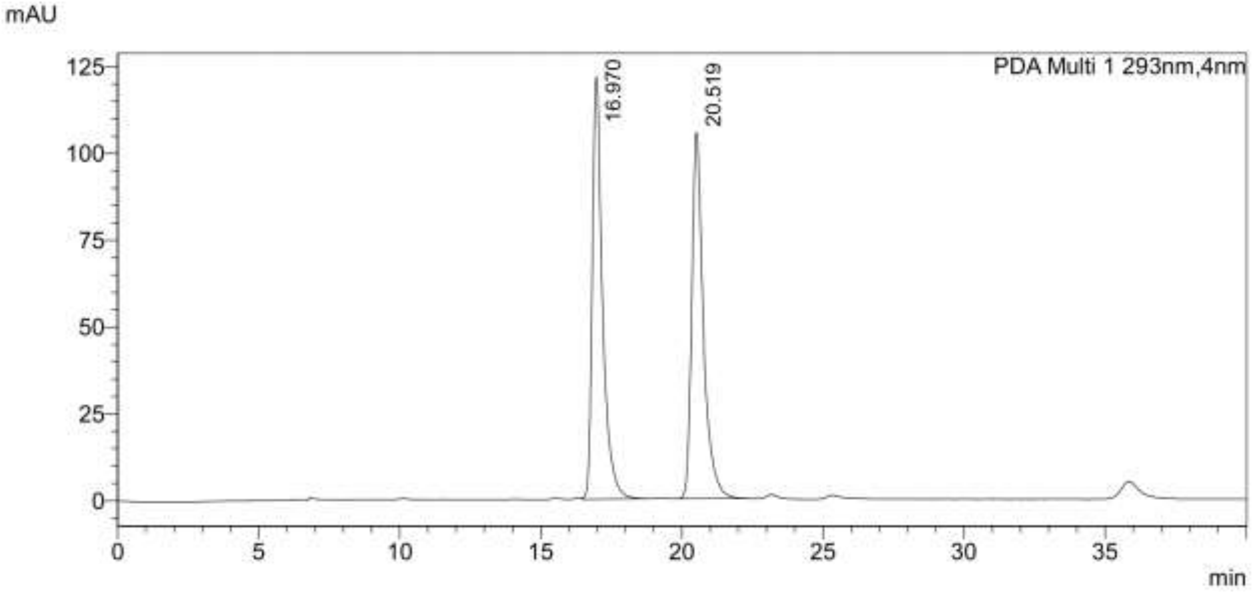

<Peak Table>

PDA Ch1 293nm

Peak#	Ret. Time	Area	Height	Area%
1	16.970	3057676	121508	50.159
2	20.519	3038289	105427	49.841
Total		6095965	226935	100.000

7,12-dimethoxy-1,3-dimethylbenzo[*c*]phenanthrene (2d): synthesized according to **GP6**, run for 24 h at 120 °C, with L^1 as the ligand.

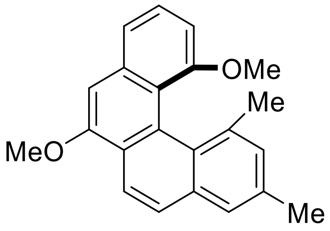

Yield: 95% (30 mg). White foam. $\boldsymbol{R_f}$ 0.30 (pentane/EtOAc 20/1); **^{1}H NMR** (400 MHz, Chloroform-*d*): δ 8.21 (d, *J* = 8.6 Hz, 1H), 7.81 (d, *J* = 8.6 Hz, 1H), 7.58 (s, 1H), 7.54 – 7.45 (m, 2H), 7.19 – 7.13 (m, 1H), 7.13 (s, 1H), 6.81 (dd, *J* = 5.8, 3.1 Hz, 1H), 4.12 (s, 3H), 3.62 (s, 3H), 2.57 (s, 3H), 2.12 (s, 3H). **^{13}C NMR** (151 MHz, Chloroform-*d*) δ 156.7, 154.1, 138.0, 135.3, 134.3, 133.2, 129.5, 128.7, 127.4, 126.9, 126.2, 125.7, 124.1, 119.3, 118.6, 118.3, 103.7, 102.7, 56.0, 54.6, 22.3, 21.5. **HRMS** (ESI): calcd. for $[C_{22}H_{20}O_2]^+$, $[M]^+$: 316.1458; found: 316.1457. **IR (ATR):** $\tilde{\nu}$ = 2931, 2362, 1611, 1515, 1458, 1346, 1280, 1221, 1152, 1116, 1020, 908, 856, 733, 697 cm^{-1}. **$[\alpha]_D^{20}$**: –370.7 (c = 1.0, $CHCl_3$).

Chiral HPLC: (Chiralpak IA, 4.6 x 250 mm; n-heptane/i-PrOH 97/3, 0.3 mL/min, 293 nm; t_R(minor) = 16.5 min, t_R(major) = 20.4 min, 5.2:94.8 *er*.

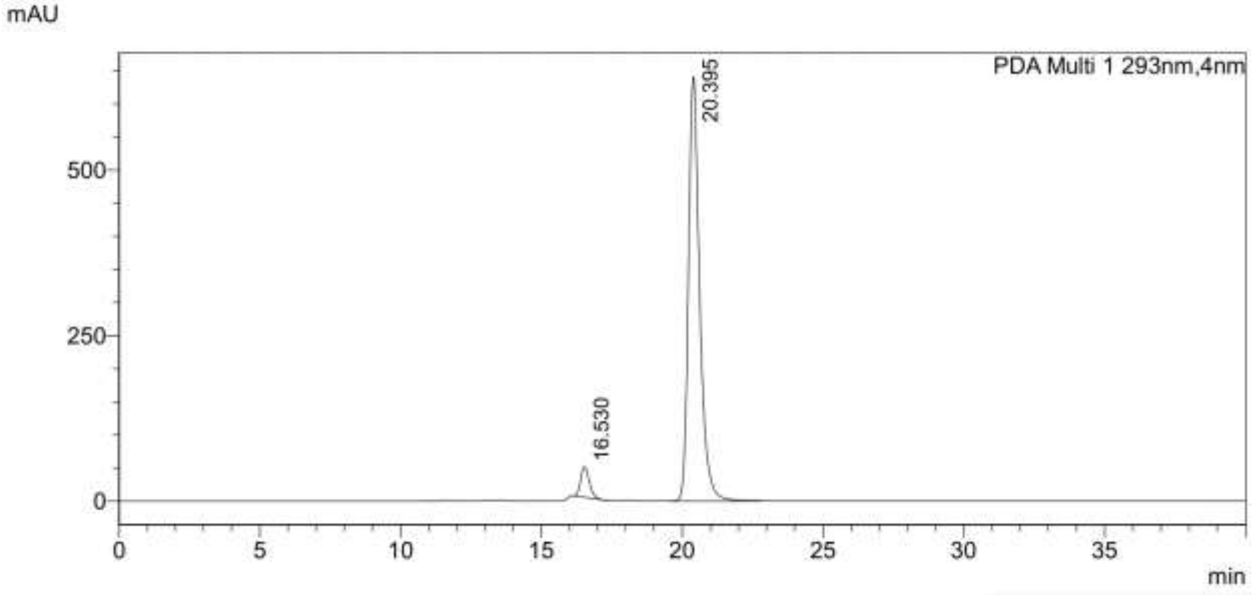

<Peak Table>

PDA Ch1 293nm

Peak#	Ret. Time	Area	Height	Area%
1	16.530	974664	45929	5.198
2	20.395	17777837	641206	94.802
Total		18752501	687135	100.000

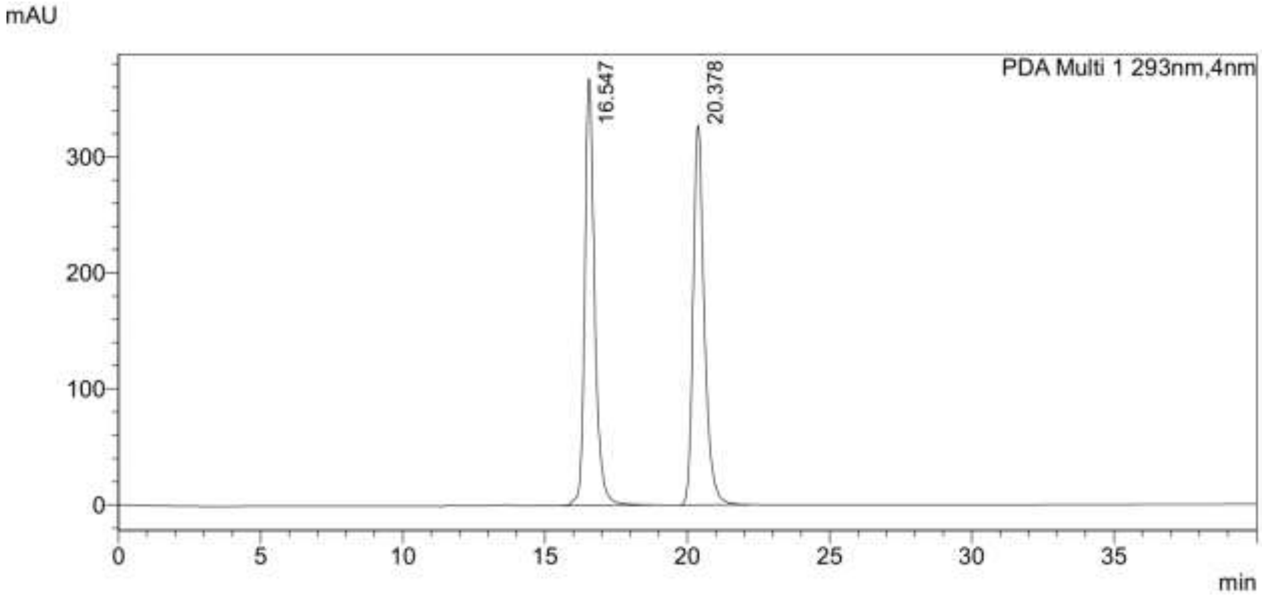

<Peak Table>

PDA Ch1 293nm

Peak#	Ret. Time	Area	Height	Area%
1	16.547	8907534	368335	49.847
2	20.378	8962201	327458	50.153
Total		17869735	695793	100.000

<u>3,7,12-trimethoxy-1-methylbenzo[*c*]phenanthrene (2e):</u> synthesized according to **GP6**, run for 24 h at 120 °C, with L^1 as the ligand.

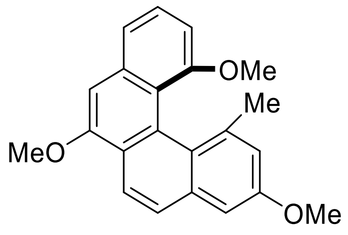

Yield: 90% (30 mg). White foam. R_f 0.20 (pentane/EtOAc 20/1); **^{1}H NMR** (400 MHz, Chloroform-*d*): δ 8.22 (d, *J* = 8.7 Hz, 1H), 7.80 (d, *J* = 8.7 Hz, 1H), 7.58 – 7.45 (m, 2H), 7.15 (d, *J* = 2.7 Hz, 1H), 7.09 (s, 1H), 7.00 – 6.95 (m, 1H), 6.80 (dd, *J* = 5.1, 3.8 Hz, 1H), 4.11 (s, 3H), 4.00 (s, 3H), 3.64 (s, 3H), 2.12 (s, 3H). **^{13}C NMR** (101 MHz, Chloroform-*d*) δ 157.4, 156.8, 154.1, 140.2, 134.5, 134.3, 127.2, 126.9, 126.6, 125.9, 125.4, 119.3, 119.3, 118.1, 117.5, 103.7, 103.6, 102.3, 56.0, 55.4, 54.6, 22.4. **HRMS** (ESI): calcd. for $[C_{22}H_{20}O_3]^+$, $[M]^+$: 332.1407; found: 332.1407. **IR (ATR):** $\tilde{\nu}$ = 2934, 1604, 1516, 1460, 1368, 1271, 1196, 1163, 1123, 1020, 908, 843, 733 cm^{-1}. **$[\alpha]_D^{20}$**: –351.0 (c = 1.0, $CHCl_3$).

Chiral HPLC: (Chiralpak OD-H, 4.6 x 250 mm; n-heptane/i-PrOH 90/10, 1.0 mL/min, 293 nm; t_R(minor) = 7.0 min, t_R(major) = 13.4 min, 5.4:94.6 *er*.

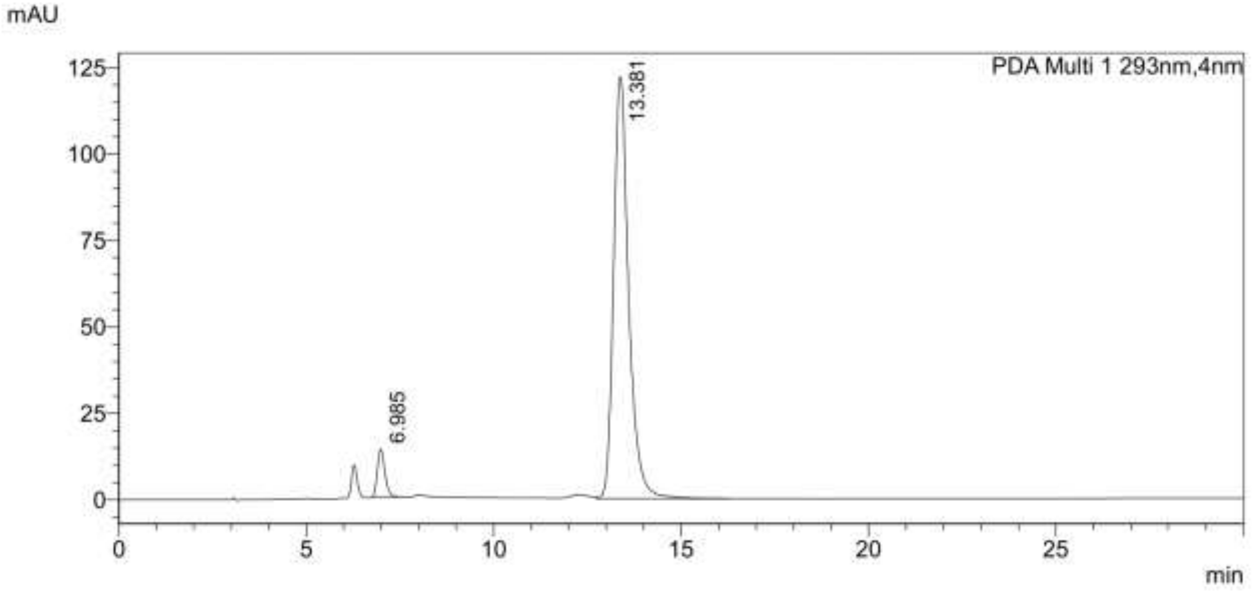

<Peak Table>

PDA Ch1 293nm

Peak#	Ret. Time	Area	Height	Area%
1	6.985	194845	13861	5.381
2	13.381	3425965	121965	94.619
Total		3620810	135826	100.000

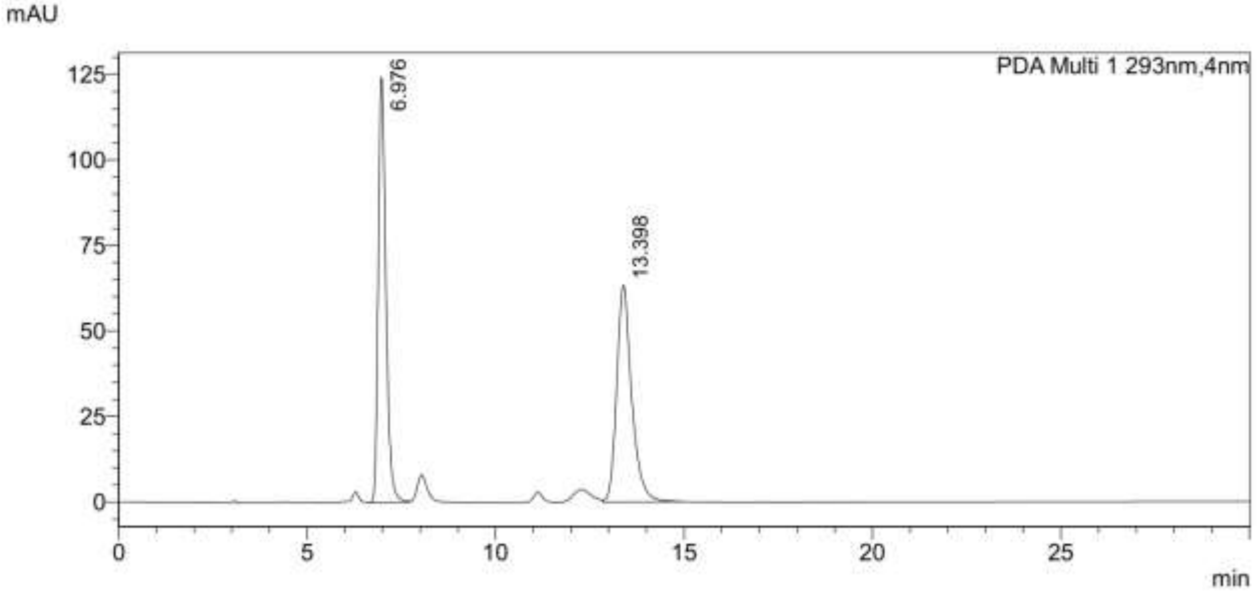

<Peak Table>

PDA Ch1 293nm

Peak#	Ret. Time	Area	Height	Area%
1	6.976	1762919	124515	50.080
2	13.398	1757315	63414	49.920
Total		3520235	187929	100.000

7,12-dimethoxy-1-methylbenzo[*c*]phenanthrene-3-carbonitrile (2f): synthesized according to **GP6**, run for 24 h at 120 °C, with L^1 as the ligand.

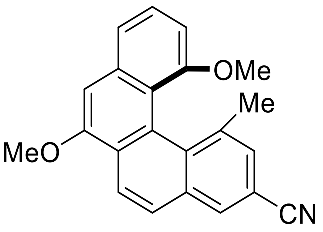

Yield: 95% (30 mg). Thick brown liquid. R_f 0.25 (pentane/EtOAc 10/1); **^{1}H NMR** (500 MHz, Chloroform-*d*): δ 8.34 (d, *J* = 8.6 Hz, 1H), 8.16 (d, *J* = 1.7 Hz, 1H), 7.85 (d, *J* = 8.7 Hz, 1H), 7.61 – 7.50 (m, 2H), 7.46 (dd, *J* = 1.7, 0.9 Hz, 1H), 7.22 (s, 1H), 6.85 (dd, *J* = 6.7, 2.2 Hz, 1H), 4.13 (s, 3H), 3.63 (s, 3H), 2.14 (s, 3H). **^{13}C NMR** (151 MHz, Chloroform-*d*) δ 156.4, 153.6, 139.6, 134.5, 133.6, 131.9, 130.3, 128.2, 127.8, 127.1, 126.7, 125.1, 120.6, 119.9, 119.5, 117.9, 108.7, 104.6, 104.2, 56.1, 54.5, 22.3. **HRMS** (ESI): calcd. for $[C_{22}H_{17}NO_2+Na]^+$, $[M+Na]^+$: 350.1151; found: 350.1150. **IR (ATR):** $\tilde{\nu}$ = 2969, 2362, 2227, 1617, 1513, 1459, 1349, 1281, 1222, 1152, 1054, 1020, 881, 781, 735, 697 cm^{-1}. **$[\alpha]_D^{20}$**: –542.9 (c = 0.28, $CHCl_3$).

Chiral HPLC: (Chiralpak IA, 4.6 x 250 mm; n-heptane/i-PrOH 97/3, 0.5 mL/min, 293 nm; t_R(minor) = 17.0 min, t_R(major) = 20.5 min, 4.7:95.3 *er*.

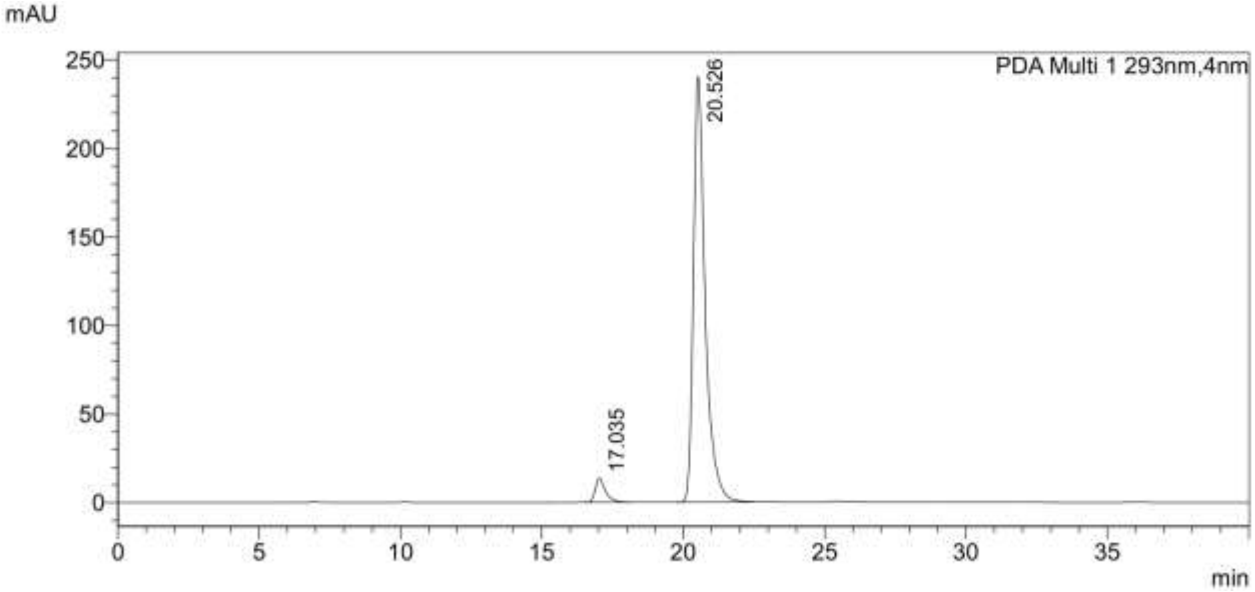

<Peak Table>

PDA Ch1 293nm

Peak#	Ret. Time	Area	Height	Area%
1	17.035	342606	13643	4.703
2	20.526	6942720	240487	95.297
Total		7285327	254130	100.000

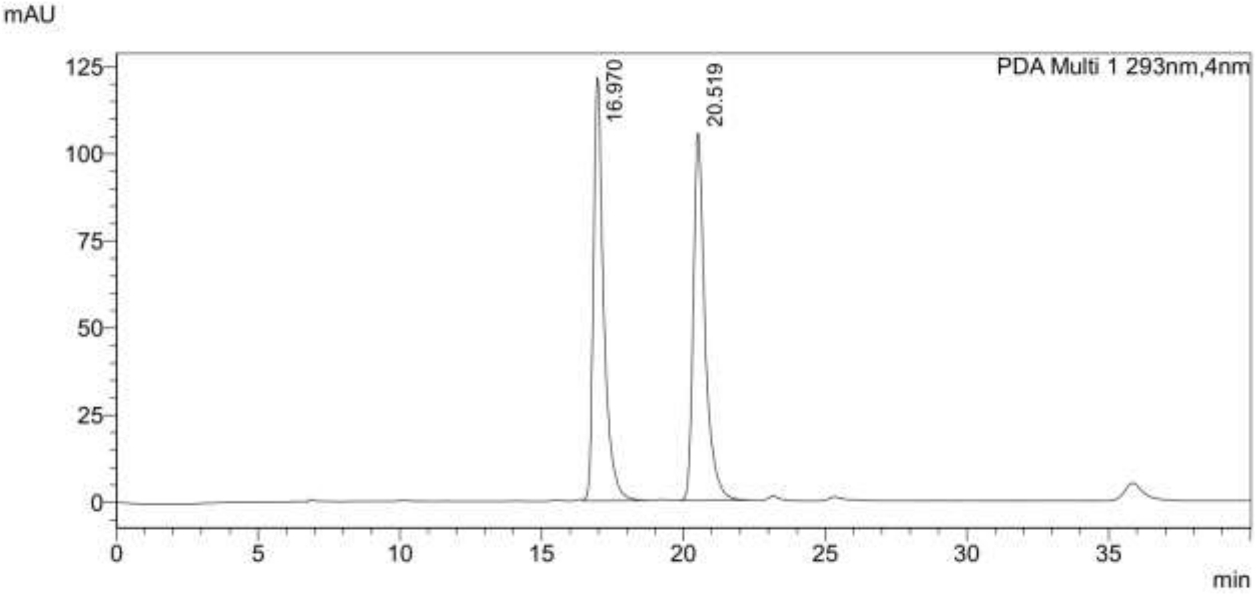

<Peak Table>

PDA Ch1 293nm

Peak#	Ret. Time	Area	Height	Area%
1	16.970	3057676	121508	50.159
2	20.519	3038289	105427	49.841
Total		6095965	226935	100.000

1,8-dimethyltetraphene (2g): synthesized according to **GP6**, run for 24 h at 120 °C. Known compound, with L^1 as the ligand.[16]

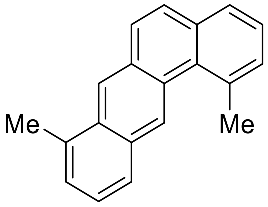

Yield: 85% (22 mg). Light yellow soild. ***R*f** 0.3 (cyclohexane); **^{1}H NMR** (600 MHz, Chloroform-*d*): δ 9.36 (s, 1H), 8.48 (s, 1H), 8.00 (d, *J* = 8.3 Hz, 1H), 7.81 (d, *J* = 8.9 Hz, 1H), 7.75 (dd, *J* = 6.9, 2.2 Hz, 1H), 7.62 (d, *J* = 8.9 Hz, 1H), 7.58 – 7.50 (m, 2H), 7.47 (dd, *J* = 8.3, 6.7 Hz, 1H), 7.42 (dt, *J* = 6.8, 1.2 Hz, 1H), 3.27 (s, 3H), 2.86 (s, 3H). **^{13}C NMR** (151 MHz, Chloroform-*d*) δ 136.2, 133.9, 133.9, 132.1, 131.7, 131.6, 130.7, 130.2, 130.0, 128.1, 128.0, 127.9, 127.5, 126.5, 126.5, 125.4, 123.3, 27.5, 19.6. **HRMS** (ESI) (0.2 mM $AgNO_3$): calcd. for $[C_{40}H_{32}+Ag]^+$, $[2M+Ag]^+$: 619.1549; found: 619.1549. **IR (ATR):** $\tilde{\nu}$ = 2962, 2368, 1452, 1377, 1070, 879, 815, 759, 687 cm^{-1}.

3,4-difluoro-10-methyldibenzo[*c,g*]phenanthrene (2h): synthesized according to **GP6**, run for 24 h at 140 °C, with L^1 as the ligand.

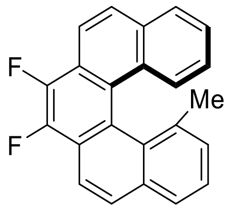

Yield: 85% (28 mg). Light yellow solid. R_f 0.35 (pentane/EtOAc 20/1); **^{1}H NMR** (500 MHz, Chloroform-*d*): δ 8.19 (d, *J* = 8.8 Hz, 1H), 8.10 (d, *J* = 8.6 Hz, 1H), 8.03 – 7.93 (m, 3H), 7.90 (dd, *J* = 7.9, 1.3 Hz, 1H), 7.82 – 7.76 (m, 1H), 7.58 (t, *J* = 7.5 Hz, 1H), 7.50 (ddd, *J* = 8.0, 6.8, 1.1 Hz, 1H), 7.23 (dd, *J* = 7.1, 1.1 Hz, 1H), 7.16 (ddd, *J* = 8.4, 6.9, 1.4 Hz, 1H), 1.51 (s, 3H). **^{13}C NMR** (151 MHz, Chloroform-*d*) δ 143.2 (dd, $^1J_{C\text{-F}}$ = 250.1 Hz, $^2J_{C\text{-F}}$ = 20.6 Hz), 143.1 (dd, $^1J_{C\text{-F}}$ = 249.6 Hz, $^2J_{C\text{-F}}$ = 20.9 Hz), 136.1, 133.0, 131.5 (d, $^4J_{C\text{-F}}$ = 1.9 Hz), 131.3, 131.2, 129.6, 129.3 (d, $^4J_{C\text{-F}}$ = 1.9 Hz), 128.5 (d, $^4J_{C\text{-F}}$ = 1.7 Hz), 128.3, 126.9, 126.6, 126.0, 125.8 (t, $^4J_{C\text{-F}}$ = 2.7 Hz), 125.7, 125.6, 124.2 (d, $^3J_{C\text{-F}}$ = 13.8 Hz), 122.4 (t, $^4J_{C\text{-F}}$ = 2.7 Hz), 121.6 (d, $^3J_{C\text{-F}}$ = 13.6 Hz), 117.8 (dd, $^3J_{C\text{-F}}$ = 6.9 Hz, $^4J_{C\text{-F}}$ = 5.2 Hz), 117.3 (dd, $^3J_{C\text{-F}}$ = 7.0 Hz, $^4J_{C\text{-F}}$ = 5.3 Hz), 23.4. **^{19}F NMR** (376 MHz, Chloroform-*d*) δ -151.3 (d, *J* = 18.3 Hz), -152.7 (d, *J* = 18.5 Hz). **HRMS** (ESI): calcd. for $[C_{23}H_{14}F_2]^+$, $[M]^+$: 328.1058; found: 328.1042. **IR (ATR):** $\tilde{\nu}$ = 2926, 2362, 1643, 1493, 1428, 1401, 1279, 1054, 821, 757, 659 cm^{-1}. **$[\alpha]_D^{20}$**: –1457 (c = 1.0, $CHCl_3$). **Mp**: 178.7 – 180.5°C.

Chiral HPLC: (Chiralpak OJ-H, 4.6 x 250 mm; n-heptane/i-PrOH 97/3, 0.5 mL/min, 254 nm; t_R(minor) = 9.3 min, t_R(major) = 12.7 min, 5.4:94.6 *er*.

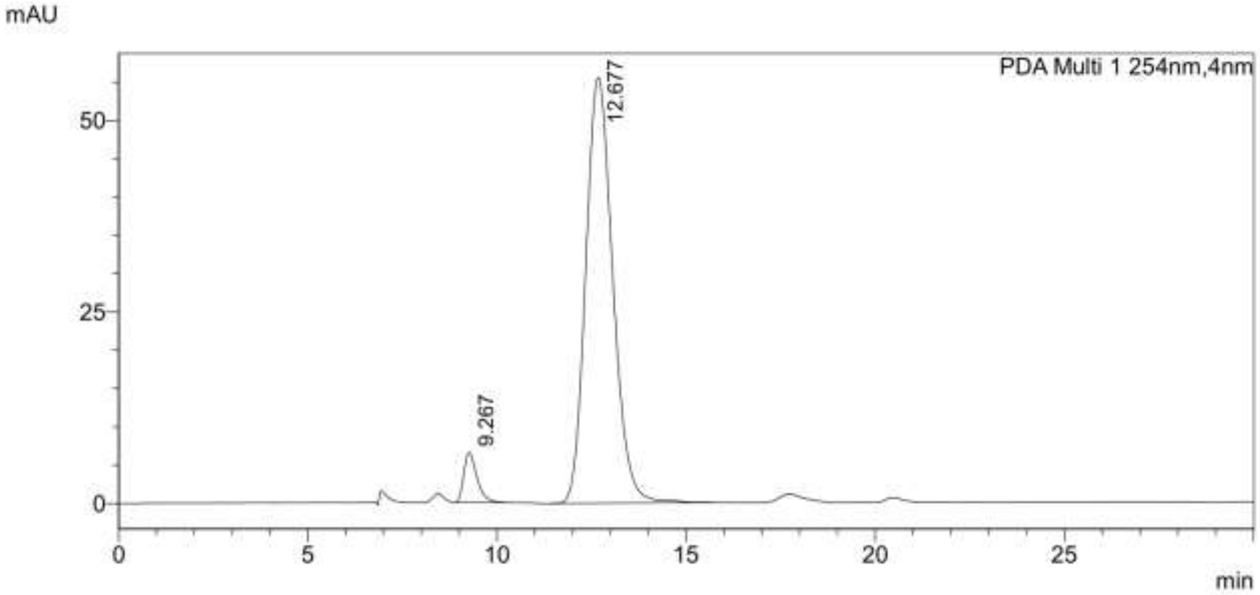

<Peak Table>

PDA Ch1 254nm

Peak#	Ret. Time	Area	Height	Area%
1	9.267	157509	6511	5.394
2	12.677	2762805	55590	94.606
Total		2920313	62100	100.000

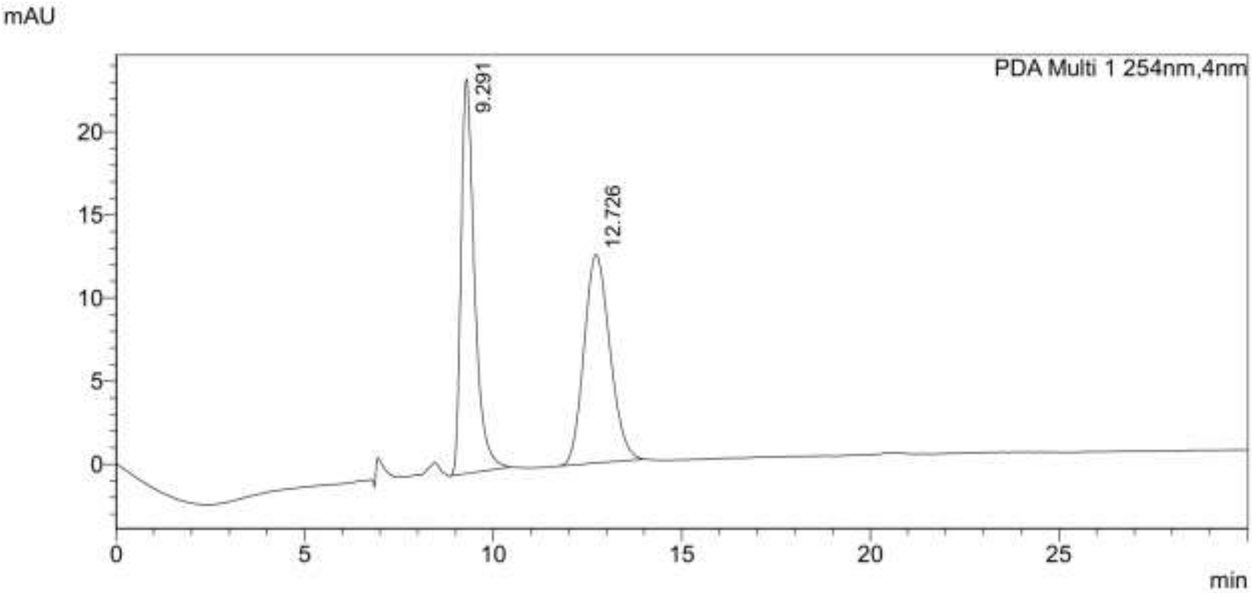

<Peak Table>

PDA Ch1 254nm

Peak#	Ret. Time	Area	Height	Area%
1	9.291	591827	23756	49.707
2	12.726	598804	12554	50.293
Total		1190630	36310	100.000

<u>3,4-difluoro-10-isopropoxydibenzo[*c*,*g*]phenanthrene (2i):</u> synthesized according to **GP6**, run for 13.5 h at 140 °C, with L^2 as the ligand.

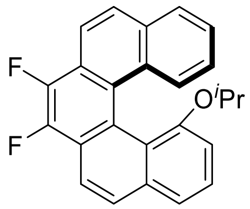

Yield: 91% (34 mg). Light green foam. ***R*f** 0.35 (pentane/EtOAc 20/1); **^{1}H NMR** (500 MHz, Chloroform-*d*): δ 8.14 (dd, *J* = 14.1, 8.7 Hz, 3H), 7.97 (dd, *J* = 8.7, 7.3 Hz, 2H), 7.93 (dd, *J* = 7.9, 1.3 Hz, 1H), 7.60 (dd, *J* = 7.9, 1.3 Hz, 1H), 7.55 (t, *J* = 7.8 Hz, 1H), 7.48 (ddd, *J* = 8.0, 6.9, 1.1 Hz, 1H), 7.19 (ddd, *J* = 8.4, 6.8, 1.4 Hz, 1H), 6.85 – 6.78 (m, 1H), 4.09 – 3.89 (m, 1H), 0.52 (d, J = 6.0 Hz, 3H), 0.32 (d, J = 6.0 Hz, 3H). **^{13}C NMR** (151 MHz, Chloroform-*d*) δ 154.9, 143.1 (dd, $^1J_{C\text{-}F}$ = 249.5 Hz, $^2J_{C\text{-}F}$ = 11.8 Hz), 142.8 (dd, $^1J_{C\text{-}F}$ = 248.7 Hz, $^2J_{C\text{-}F}$ = 12.0 Hz), 134.3, 133.3, 130.1, 128.6 (d, $^4J_{C\text{-}F}$ = 1.9 Hz), 128.3 (d, $^4J_{C\text{-}F}$ = 1.8 Hz), 127.9, 127.3, 126.9, 126.3, 125.8, 125.6, 123.6 (d, $^2J_{C\text{-}F}$ = 13.7 Hz), 122.6, 121.6 (d, $^2J_{C\text{-}F}$ = 13.3 Hz), 120.7 (d, $^4J_{C\text{-}F}$ = 2.7 Hz), 119.5, 118.2 (dd, $^3J_{C\text{-}F}$ = 7.4 Hz, $^4J_{C\text{-}F}$ = 5.3 Hz), 117.4 (dd, $^3J_{C\text{-}F}$ = 7.4 Hz, $^4J_{C\text{-}F}$ = 5.2 Hz), 108.8, 69.6, 21.2, 20.5. **^{19}F NMR** (376 MHz, Chloroform-*d*) δ -151.9 (d, *J* = 18.6 Hz), -153.9 (d, *J* = 18.5 Hz). **HRMS** (ESI): calcd. for $[C_{25}H_{18}F_2O]^+$, $[M]^+$: 372.1320; found: 372.1321. **IR (ATR):** $\tilde{\nu}$ = 2978, 2362, 1650, 1558, 1403, 1258, 1118, 1043, 817, 749, 668 cm^{-1}. **$[\alpha]_D^{20}$**: –2012 (c = 0.3, $CHCl_3$).

Chiral HPLC: (Chiralpak OJ-H, 4.6 x 250 mm; n-heptane/i-PrOH 97/3, 0.3 mL/min, 254 nm; t_R(minor) = 16.1 min, t_R(major) = 19.3 min, 2.8:97.2 *er*.

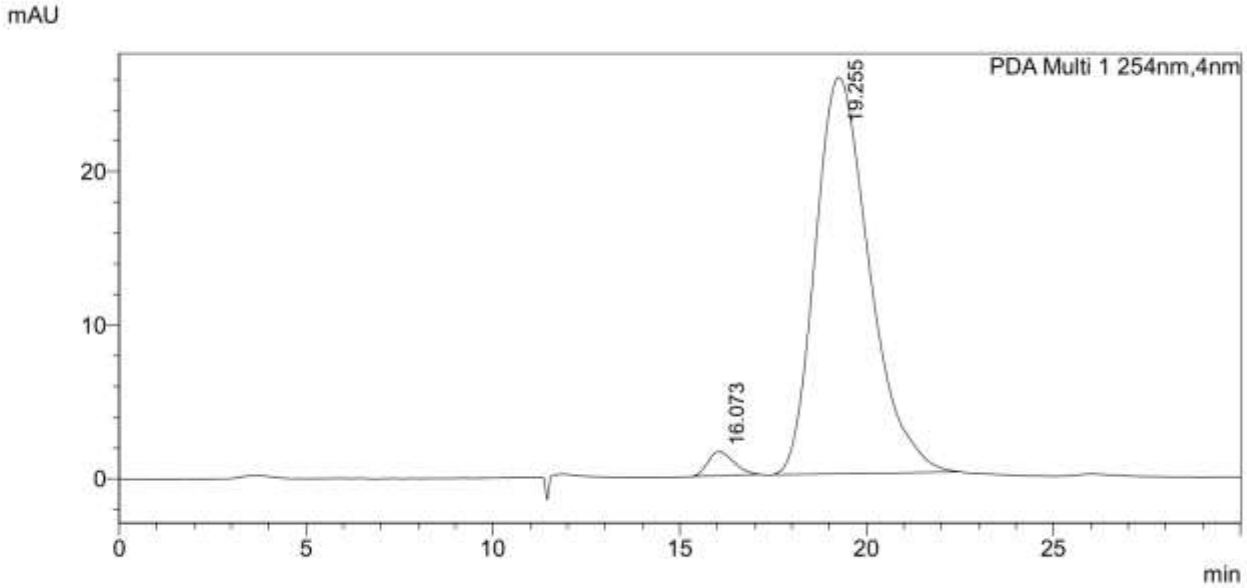

<Peak Table>

PDA Ch1 254nm

Peak#	Ret. Time	Area	Height	Area%
1	16.073	75098	1572	2.811
2	19.255	2596118	25824	97.189
Total		2671216	27396	100.000

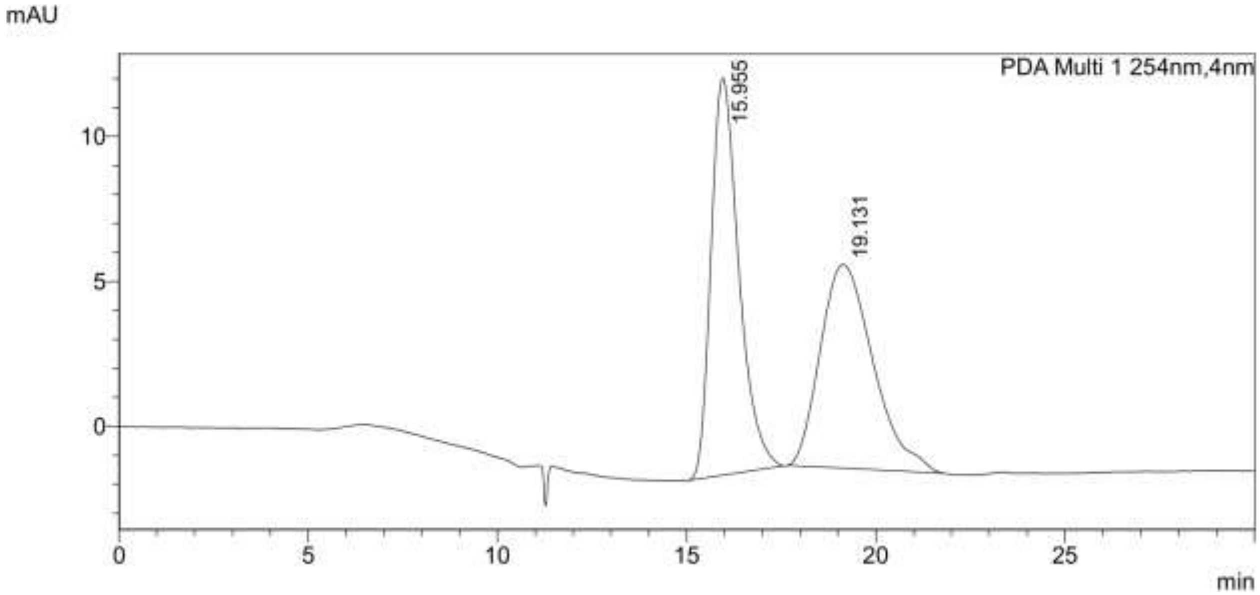

<Peak Table>

PDA Ch1 254nm

Peak#	Ret. Time	Area	Height	Area%
1	15.955	699938	13709	50.642
2	19.131	682190	7028	49.358
Total		1382128	20736	100.000

3,4-difluoro-8-methoxy-11-methyldibenzo[*c,g*]phenanthrene (2j): synthesized according to **GP6**, run for 24 h at 140 °C, with L^1 as the ligand.

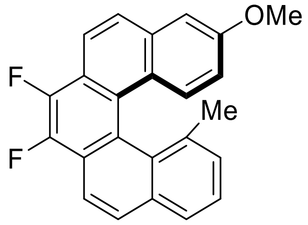

Yield: 92% (33 mg). Yellow foam. $\boldsymbol{R_f}$ 0.30 (pentane/EtOAc 20/1); **^{1}H NMR** (500 MHz, Chloroform-*d*): δ 8.17 (d, *J* = 8.8 Hz, 1H), 8.08 (d, *J* = 8.6 Hz, 1H), 7.97 (d, *J* = 8.7 Hz, 1H), 7.93 (d, *J* = 8.8 Hz, 1H), 7.88 (dd, *J* = 8.0, 1.2 Hz, 1H), 7.68 (d, *J* = 9.2 Hz, 1H), 7.57 (t, *J* = 7.5 Hz, 1H), 7.30 (d, *J* = 2.7 Hz, 1H), 7.23 (dd, *J* = 7.1, 1.1 Hz, 1H), 6.82 (dd, *J* = 9.3, 2.8 Hz, 1H), 3.96 (s, 3H), 1.55 (s, 3H). **^{13}C NMR** (151 MHz, Chloroform-*d*) δ 158.1, 143.4 (dd, $^1J_{C\text{-F}}$ = 250.3 Hz, $^2J_{C\text{-F}}$ = 12 Hz), 142.5 (dd, $^1J_{C\text{-F}}$ = 248.5 Hz, $^2J_{C\text{-F}}$ = 12.0 Hz), 136.0, 132.9, 131.3, 129.6, 129.2 (d, $^4J_{C\text{-F}}$ = 1.6 Hz), 128.0 (d, $^4J_{C\text{-F}}$ = 1.8 Hz), 127.3, 126.7, 126.3, 126.2 (d, $^4J_{C\text{-F}}$ = 2.5 Hz), 125.7, 124.3 (d, $^2J_{C\text{-F}}$ = 14.0 Hz), 122.0 (d, $^4J_{C\text{-F}}$ = 2.7 Hz), 120.5 (d, $^2J_{C\text{-F}}$ = 13.7 Hz), 118.5 (dd, $^3J_{C\text{-F}}$ = 6.6 Hz, $^4J_{C\text{-F}}$ = 5.8 Hz), 117.3 (dd, $^3J_{C\text{-F}}$ = 7.0 Hz, $^4J_{C\text{-F}}$ = 5.3 Hz), 117.0, 107.4, 55.5, 23.5. **^{19}F NMR** (376 MHz, Chloroform-*d*) δ -151.5 (d, *J* = 18.5 Hz), -154.2 (d, *J* = 18.4 Hz). **HRMS** (ESI): calcd. for $[C_{24}H_{16}F_2O]^+$, $[M]^+$: 358.1164; found: 358.1166. **IR (ATR):** $\tilde{\nu}$ = 2929, 2362, 1645, 1613, 1410, 1285, 1239, 1053, 850, 819, 733, 666 cm^{-1}. $\mathbf{[\alpha]_D^{20}}$: –1540 (c = 0.4, $CHCl_3$).

Chiral HPLC: (Chiralpak OJ-H, 4.6 x 250 mm; n-heptane/i-PrOH 90/10, 1.0 mL/min, 293 nm; t_R(minor) = 5.2 min, t_R(major) = 8.8 min, 6.0:94.0 *er*.

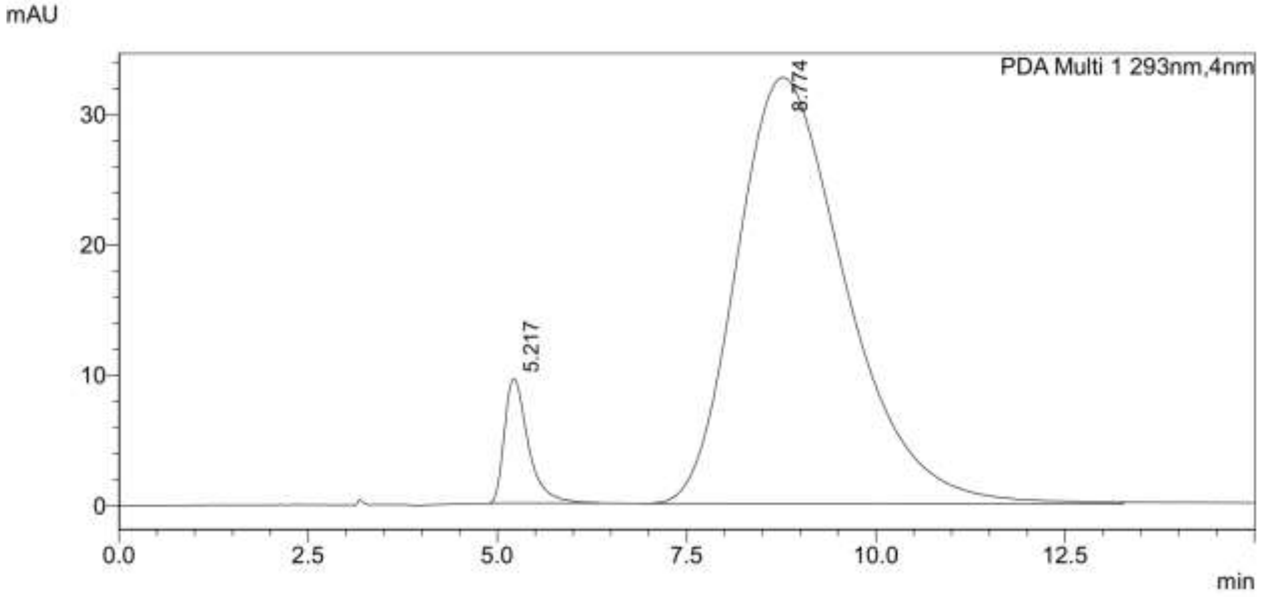

<Peak Table>

PDA Ch1 293nm

Peak#	Ret. Time	Area	Height	Area%
1	5.217	208863	9544	5.988
2	8.774	3279315	32696	94.012
Total		3488178	42240	100.000

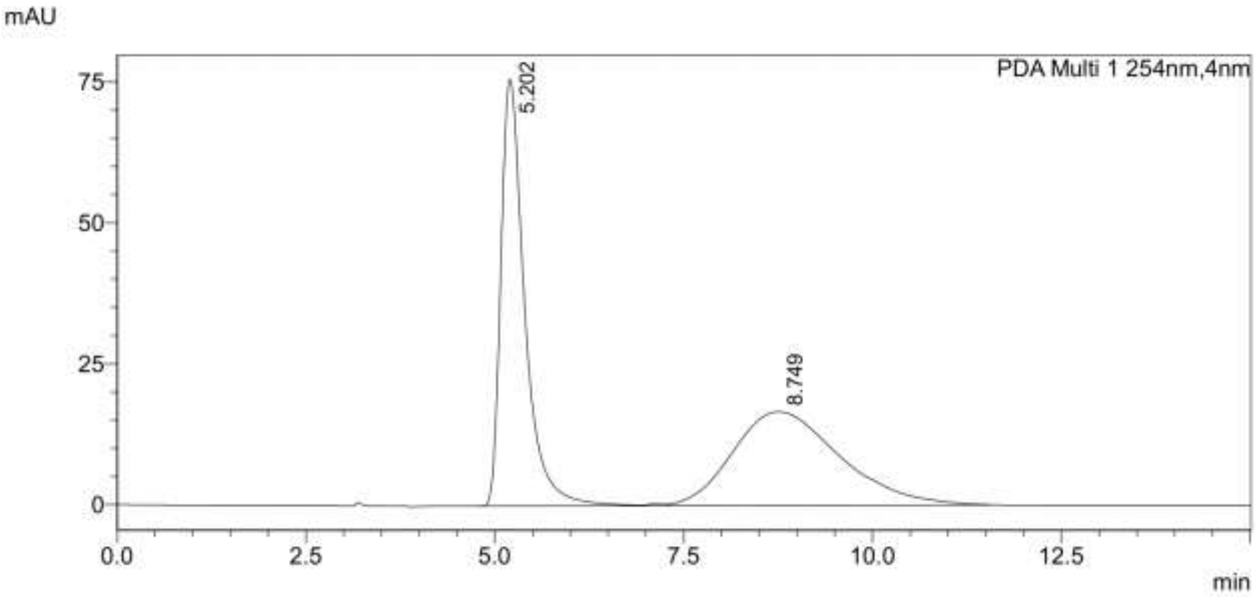

<Peak Table>

PDA Ch1 254nm

Peak#	Ret. Time	Area	Height	Area%
1	5.202	1665018	75753	50.473
2	8.749	1633802	16630	49.527
Total		3298820	92383	100.000

3,4-difluoro-11-methyldibenzo[*c,g*]phenanthrene-8-carbonitrile (2k): synthesized according to **GP6**, run for 24 h at 140 °C, with L^1 as the ligand.

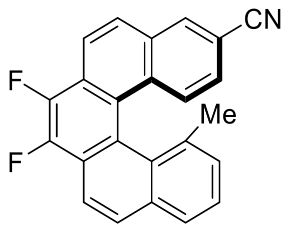

Yield: 88% (31 mg). Light yellow solid. ***R*f** 0.10 (cyclohexane/EtOAc 50/1); **^{1}H NMR** (500 MHz, Chloroform-*d*): δ 8.33 (d, *J* = 1.7 Hz, 1H), 8.31 (d, *J* = 8.8 Hz, 1H), 8.12 (d, *J* = 8.6 Hz, 1H), 8.04 (d, *J* = 8.6 Hz, 1H), 8.01 (d, *J* = 8.8 Hz, 1H), 7.93 (d, *J* = 7.7 Hz, 1H), 7.86 (d, *J* = 8.8 Hz, 1H), 7.66 – 7.58 (m, 1H), 7.31 (dd, *J* = 8.8, 1.8 Hz, 1H), 7.27 (d, *J* = 6.8 Hz, 1H), 1.50 (s, 3H). **^{13}C NMR** (151 MHz, Chloroform-*d*) δ 144.2 (dd, $^1J_{C\text{-F}}$ = 252.9 Hz, $^2J_{C\text{-F}}$ = 11.8 Hz), 143.1 (dd, $^1J_{C\text{-F}}$ = 251.5 Hz, $^2J_{C\text{-F}}$ = 12.2 Hz), 135.5, 133.8, 133.5, 133.3, 130.7, 130.5, 130.1, 130.1 (d, $^4J_{C\text{-F}}$ = 1.6 Hz), 127.8 (d, $^4J_{C\text{-F}}$ = 1.9 Hz), 127.5, 127.0, 126.7, 126.1, 124.9 (t, $^4J_{C\text{-F}}$ = 2.3 Hz), 124.7 (d, $^2J_{C\text{-F}}$ = 13.9 Hz), 122.8 (d, $^2J_{C\text{-F}}$ = 13.6 Hz), 122.5 (t, $^4J_{C\text{-F}}$ = 2.9 Hz), 120.0 (dd, $^3J_{C\text{-F}}$ = 6.4 Hz, $^4J_{C\text{-F}}$ = 5.6 Hz), 119.1, 117.3 (dd, $^3J_{C\text{-F}}$ = 7.0 Hz, $^4J_{C\text{-F}}$ = 5.2 Hz), 109.8,

23.4. **^{19}F NMR** (376 MHz, Chloroform-*d*) δ -149.3 (d, *J* = 18.3 Hz), -150.6 (d, *J* = 18.2 Hz). **HRMS** (ESI) (0.2 mM $AgNO_3$): calcd. for $[C_{24}H_{13}F_2N+Ag]^+$, $[M+Ag]^+$: 460.0062; found: 460.0062. **IR (ATR):** $\widetilde{\nu}$ = 2927, 2362, 2229, 1625, 1409, 1441, 1409, 1284, 1053, 906, 821, 732, 666 cm^{-1}. **$[\alpha]_D^{20}$**: –1115 (c = 1.0, $CHCl_3$). **Mp**: 123.5 – 125.0°C.

Chiral HPLC: (Chiralpak OD-H, 4.6 x 250 mm; n-heptane/i-PrOH 90/10, 1.0 mL/min, 254 nm; t_R(minor) = 6.5 min, t_R(major) = 7.0 min, 4.9:95.1 *er*.

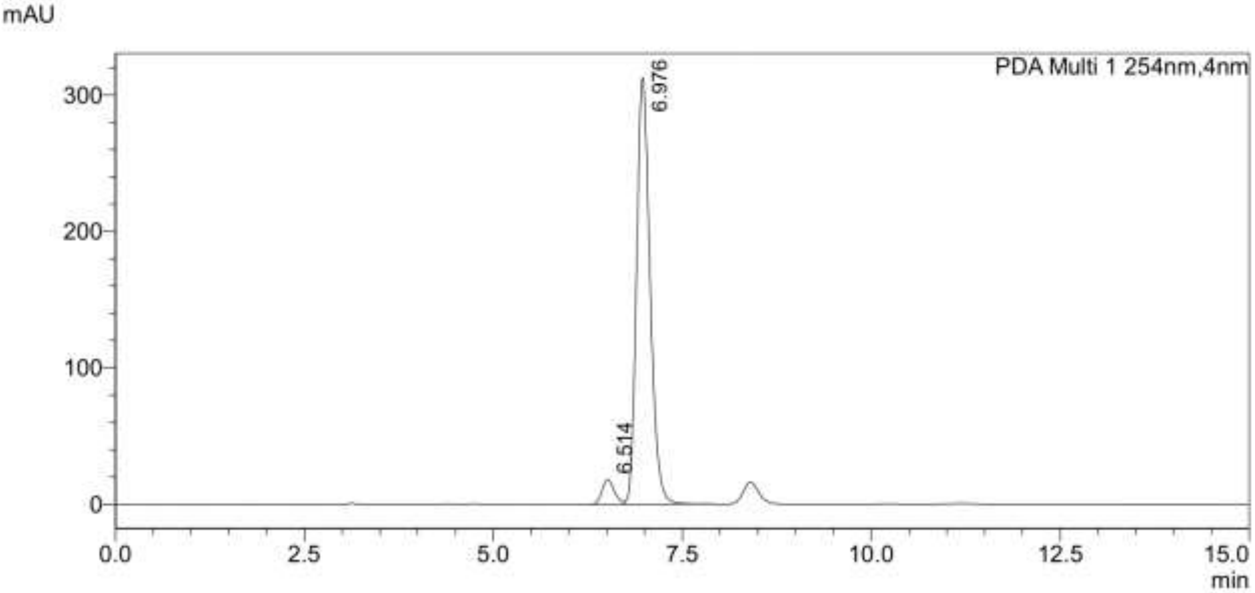

<Peak Table>

PDA Ch1 254nm

Peak#	Ret. Time	Area	Height	Area%
1	6.514	201664	17903	4.938
2	6.976	3882542	312900	95.062
Total		4084206	330803	100.000

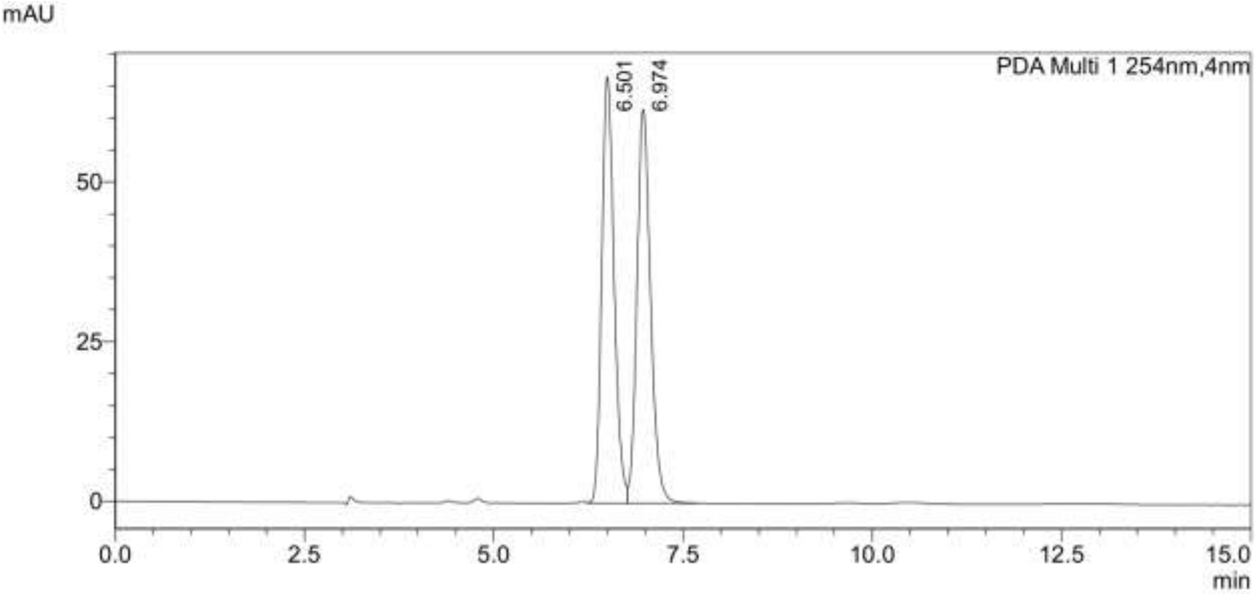

<Peak Table>

PDA Ch1 254nm

Peak#	Ret. Time	Area	Height	Area%
1	6.501	759457	66800	49.545
2	6.974	773411	61722	50.455
Total		1532868	128522	100.000

3,4,8-trifluoro-11-methyldibenzo[*c,g*]phenanthrene (2l): synthesized according to **GP6**, run for 24 h at 140 °C, with L^1 as the ligand.

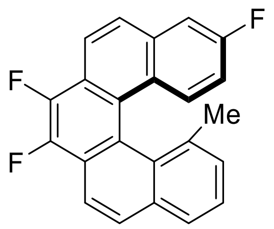

Yield: 92% (32 mg). White solid. R_f 0.40 (cyclohexane); **^{1}H NMR** (500 MHz, Chloroform-*d*): δ 8.22 (d, *J* = 8.8 Hz, 1H), 8.10 (d, *J* = 8.6 Hz, 1H), 7.99 (d, *J* = 8.6 Hz, 1H), 7.94 (d, *J* = 8.8 Hz, 1H), 7.90 (dd, *J* = 7.9, 1.3 Hz, 1H), 7.78 (dd, *J* = 9.3, 5.6 Hz, 1H), 7.65 – 7.55 (m, 2H), 7.24 (d, *J* = 7.0 Hz, 1H), 6.92 (ddd, *J* = 9.3, 8.0, 2.8 Hz, 1H), 1.53 (s, 3H). **^{13}C NMR** (151 MHz, Chloroform-*d*) δ 160.9 (d, $^1J_{C\text{-}F}$ = 248.1 Hz), 143.3 (dd, $^1J_{C\text{-}F}$ = 250.8 Hz, $^2J_{C\text{-}F}$ = 11.8 Hz), 143.1 (dd, $^1J_{C\text{-}F}$ = 250.0 Hz, $^2J_{C\text{-}F}$ = 12.0 Hz), 135.8, 133.1, 132.7 (d, $^3J_{C\text{-}F}$ = 8.6 Hz), 131.1, 129.8, 129.5 (d, $^4J_{C\text{-}F}$ = 1.7 Hz), 128.3, 128.1 (d, $^3J_{C\text{-}F}$ = 8.3 Hz), 127.8 (dd, $^3J_{C\text{-}F}$ = 4.0 Hz, $^4J_{C\text{-}F}$ = 1.7 Hz), 127.0, 125.9, 125.8, 124.4 (d, $^2J_{C\text{-}F}$ = 13.7 Hz), 122.1, 121.1 (d, $^2J_{C\text{-}F}$ = 13.7 Hz), 119.2 (t, $^3J_{C\text{-}F}$ = 6.0 Hz), 117.3 (dd, $^3J_{C\text{-}F}$ = 7.1 Hz, $^4J_{C\text{-}F}$ = 5.3 Hz), 115.3 (d, $^2J_{C\text{-}F}$ = 23.6 Hz), 112.0 (d, $^2J_{C\text{-}F}$ = 20.5 Hz), 23.4. **^{19}F NMR** (376 MHz, Chloroform-*d*) δ -114.4 (d, *J* = 1.9 Hz), -151.1 (d, *J* = 18.3 Hz), -152.7 (dd, *J* = 18.5, 1.8 Hz). **HRMS** (ESI) (0.2 mM $AgNO_3$): calcd. for $[C_{23}H_{13}F_3]^+$, $[M]^+$: 346.0964; found: 346.0962. **IR (ATR):** $\tilde{\nu}$ = 2968, 2362, 1646, 1526, 1441, 1410, 1282, 1235, 1150, 1053, 868, 819, 663 cm^{-1}. **$[\alpha]_D^{20}$**: –1972 (c = 0.5, $CHCl_3$). **Mp**: 116.7 – 118.8°C.

Chiral HPLC: (Chiralpak OJ-H, 4.6 x 250 mm; n-heptane/i-PrOH 97/3, 0.3 mL/min, 254 nm; t_R(minor) = 16.7 min, t_R(major) = 19.4 min, 4.7:95.3 *er*.

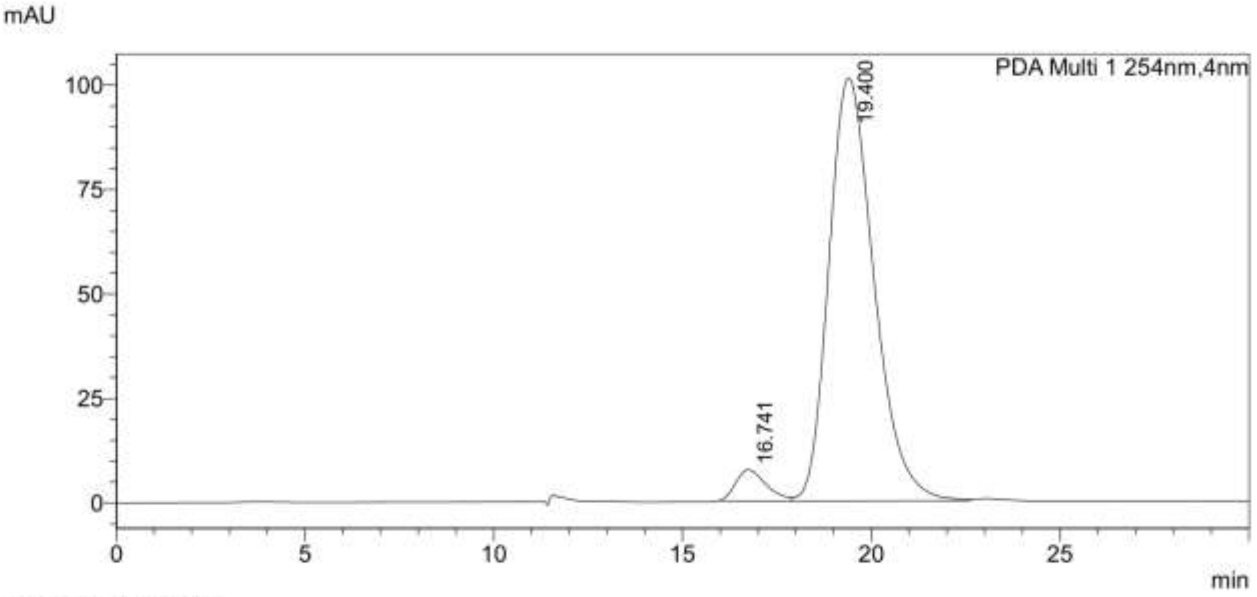

<Peak Table>

PDA Ch1 254nm

Peak#	Ret. Time	Area	Height	Area%
1	16.741	410275	7383	4.652
2	19.400	8409504	101127	95.348
Total		8819779	108510	100.000

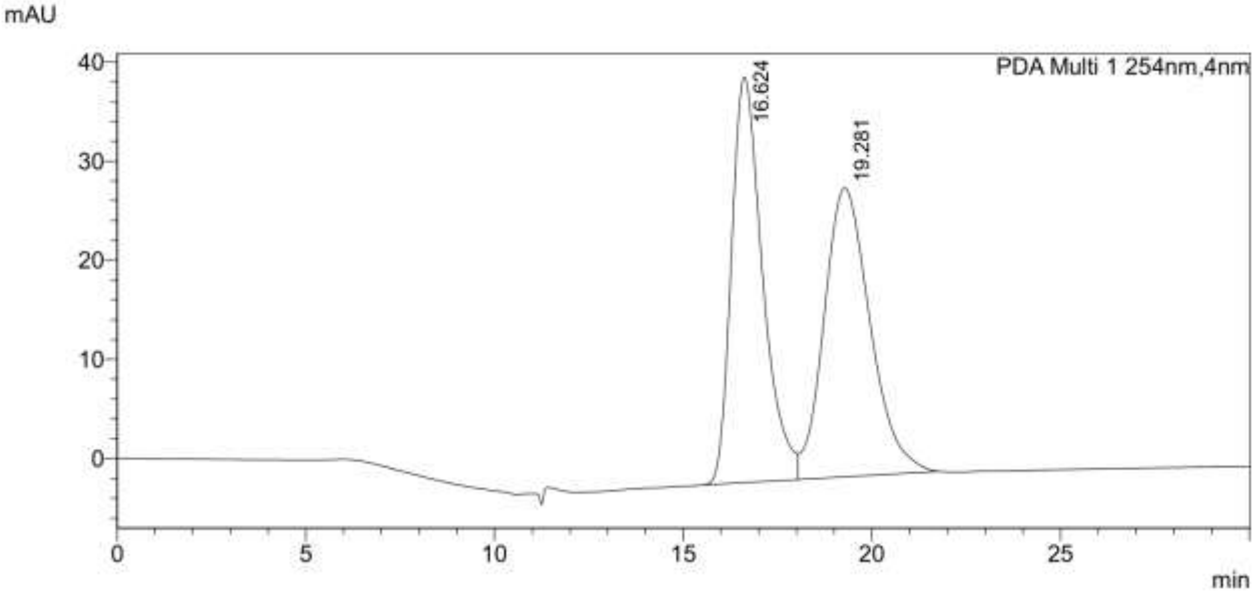

<Peak Table>

PDA Ch1 254nm

Peak#	Ret. Time	Area	Height	Area%
1	16.624	2352753	40874	48.765
2	19.281	2471960	29124	51.235
Total		4824713	69999	100.000

3,4-difluoro-8,11-dimethyldibenzo[*c,g*]phenanthrene (2m): synthesized according to **GP6**, run for 24 h at 140 °C, with L^1 as the ligand.

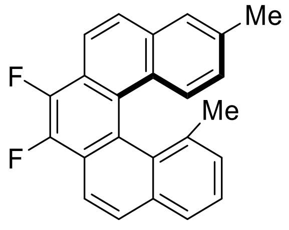

Yield: 91% (31 mg). White solid. R_f 0.30 (cyclohexane); ^{1}**H NMR** (500 MHz, Chloroform-*d*): δ 8.16 (d, *J* = 8.8 Hz, 1H), 8.09 (d, *J* = 8.6 Hz, 1H), 7.98 (d, *J* = 8.6 Hz, 1H), 7.93 (d, *J* = 8.8 Hz, 1H), 7.89 (dd, *J* = 7.9, 1.2 Hz, 1H), 7.73 (s, 1H), 7.67 (d, *J* = 8.7 Hz, 1H), 7.57 (t, *J* = 7.5 Hz, 1H), 7.23 (d, *J* = 7.1 Hz, 1H), 6.99 (dd, *J* = 8.7, 1.9 Hz, 1H), 2.51 (s, 3H), 1.53 (s, 3H). ^{13}**C NMR** (151 MHz, Chloroform-*d*) 143.3 (dd, $^1J_{C\text{-}F}$ = 250.3 Hz, $^2J_{C\text{-}F}$ = 11.8 Hz), 142.9 (dd, $^1J_{C\text{-}F}$ = 249.1 Hz, $^2J_{C\text{-}F}$ = 12.1 Hz), 136.5, 136.2, 133.0, 131.6, 131.3, 129.6 (t, $^4J_{C\text{-}F}$ = 1.9 Hz) 129.5, 129.2 (d, $^4J_{C\text{-}F}$ = 1.7 Hz), 128.3 (d, $^4J_{C\text{-}F}$ = 1.7 Hz), 127.9, 127.7, 126.7, 126.0 (d, $^4J_{C\text{-}F}$ = 2.5 Hz), 125.7, 125.5, 124.1 (d, $^2J_{C\text{-}F}$ = 13.8 Hz), 122.3 (t, $^4J_{C\text{-}F}$ = 2.7 Hz), 121.2 (d, $^2J_{C\text{-}F}$ = 13.6 Hz), 117.8 (dd, $^3J_{C\text{-}F}$ = 6.9 Hz, $^4J_{C\text{-}F}$ = 5.3 Hz), 117.3 (dd, $^3J_{C\text{-}F}$ = 7.1 Hz, $^4J_{C\text{-}F}$ = 5.2 Hz), 23.5, 21.6. ^{19}**F NMR** (376 MHz, Chloroform-*d*) δ -151.4 (d, *J* = 18.5 Hz), -153.5 (d, *J* = 18.4 Hz). **HRMS** (ESI) (0.2 mM $AgNO_3$): calcd. for $[C_{24}H_{16}F_2+Ag]^+$, $[M+Ag]^+$: 449.0266; found: 449.0260. **IR (ATR):** $\tilde{\nu}$ = 2969, 2362, 1645, 1442, 1408, 1284, 1095, 1052, 884, 819, 792, 733, 661 cm^{-1}. **$[\alpha]_D^{20}$**: –2030 (c = 0.6, $CHCl_3$). **Mp**: 152.0 – 153.7 °C.

Chiral HPLC: (Chiralpak OJ-H, 4.6 x 250 mm; n-heptane/i-PrOH 97/3, 0.3 mL/min, 254 nm; t_R(minor) = 23.3 min, t_R(major) = 26.4 min, 5.8:94.2 *er*.

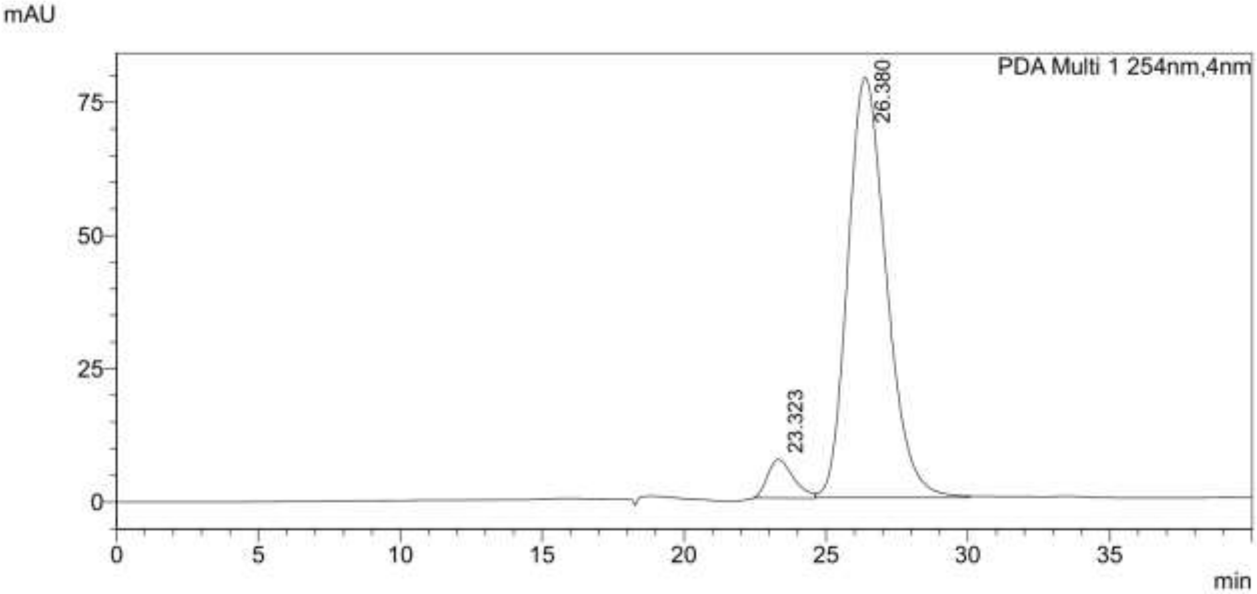

<Peak Table>

PDA Ch1 254nm

Peak#	Ret. Time	Area	Height	Area%
1	23.323	462653	7254	5.834
2	26.380	7467853	78840	94.166
Total		7930506	86093	100.000

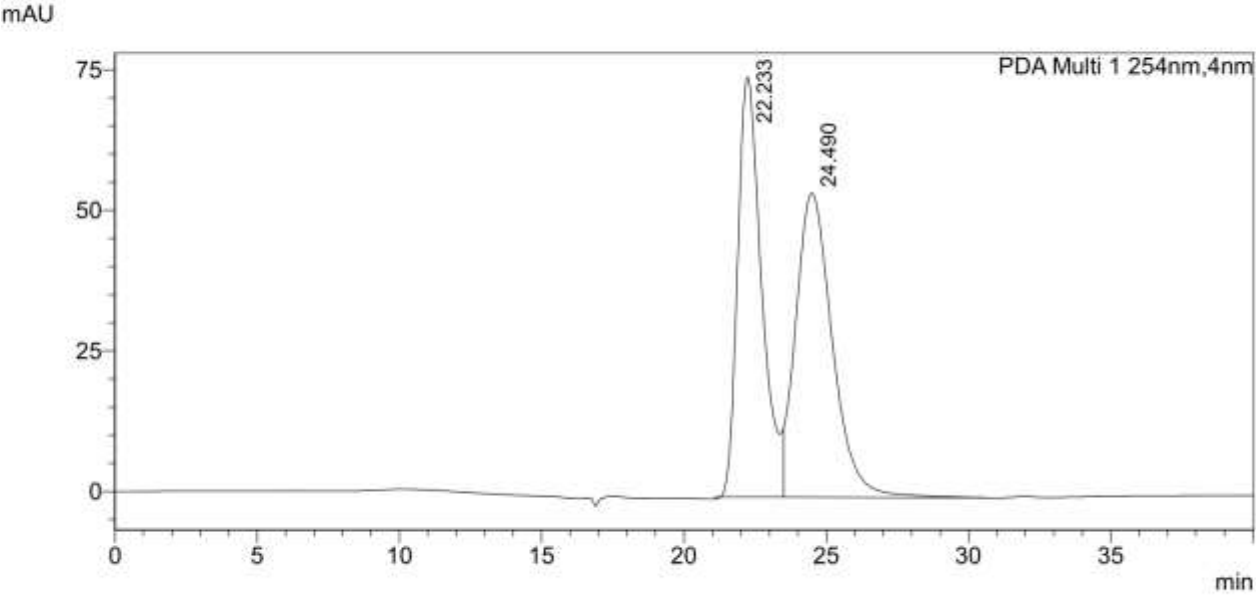

<Peak Table>

PDA Ch1 254nm

Peak#	Ret. Time	Area	Height	Area%
1	22.233	4377087	74708	47.489
2	24.490	4839952	54099	52.511
Total		9217039	128808	100.000

3,4,9-trifluoro-11-methyldibenzo[*c,g*]phenanthrene (2n): synthesized according to **GP6**, run for 24 h at 140 °C, with L^1 as the ligand.

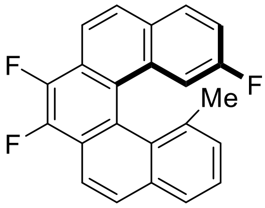

Yield: 81% (28 mg). White crystal. $\boldsymbol{R_f}$ 0.15 (cyclohexane); **^{1}H NMR** (500 MHz, Chloroform-*d*): δ 8.16 (d, *J* = 8.8 Hz, 1H), 8.10 (d, *J* = 8.6 Hz, 1H), 8.03 – 7.92 (m, 3H), 7.91 (d, *J* = 8.3 Hz, 1H), 7.61 (t, *J* = 7.5 Hz, 1H), 7.48 (dd, *J* = 12.1, 2.6 Hz, 1H), 7.31 – 7.23 (m, 2H), 1.61 (s, 3H). **^{13}C NMR** (151 MHz, Chloroform-*d*) 161.0 (d, $^1J_{C\text{-F}}$ = 245.3 Hz), 143.5 (dd, $^1J_{C\text{-F}}$ = 250.8 Hz, $^2J_{C\text{-F}}$ = 13.1 Hz), 143.2 (dd, $^1J_{C\text{-F}}$ = 250.0 Hz, $^2J_{C\text{-F}}$ = 12.8 Hz), 135.8, 133.2, 132.8, 130.6 (d, $^3J_{C\text{-F}}$ = 8.9 Hz), 130.5, 129.8, 129.5, 128.2, 128.1, 127.2, 125.9, 125.3, 124.2 (d, $^2J_{C\text{-F}}$ = 14.0 Hz), 122.5, 122.2 (d, $^2J_{C\text{-F}}$ = 13.5 Hz), 117.3 (d, $^3J_{C\text{-F}}$ = 5.9 Hz), 117.2 (d, $^3J_{C\text{-F}}$ = 6.1 Hz), 116.1 (d, $^2J_{C\text{-F}}$ = 24.4 Hz), 110.2 (d, $^2J_{C\text{-F}}$ = 23.8 Hz), 23.2. **^{19}F NMR** (376 MHz, Chloroform-*d*) δ -113.7, -151.1 (d, *J* = 18.2 Hz), -151.7 (d, *J* = 18.3 Hz). **HRMS** (ESI): calcd. for $[C_{23}H_{13}F_3]^+$, $[M]^+$: 346.0964; found: 346.0958. **IR (ATR):** $\tilde{\nu}$ = 2926, 2362, 1649, 1623, 1523, 1436, 1409, 1315, 1276, 1215, 1173, 1100, 1054, 828, 819, 762 cm^{-1}. **$[\alpha]_D^{20}$**: –1722 (c = 0.04, $CHCl_3$). **Mp**: 157.4 – 159.4 °C.

Chiral HPLC: (Chiralpak OJ-H, 4.6 x 250 mm; n-heptane/i-PrOH 97/3, 0.5 mL/min, 254 nm; t_R(minor) = 9.7 min, t_R(major) = 12.6 min, 7.7:92.3 *er*.

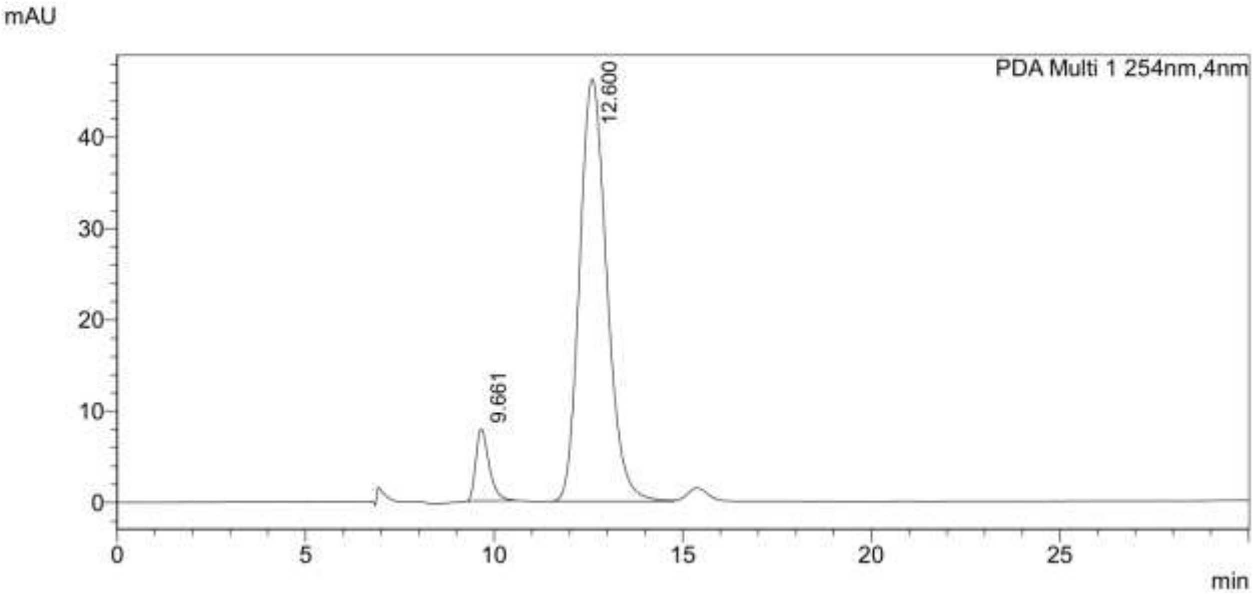

<Peak Table>

PDA Ch1 254nm

Peak#	Ret. Time	Area	Height	Area%
1	9.661	192593	7879	7.669
2	12.600	2318768	46269	92.331
Total		2511362	54148	100.000

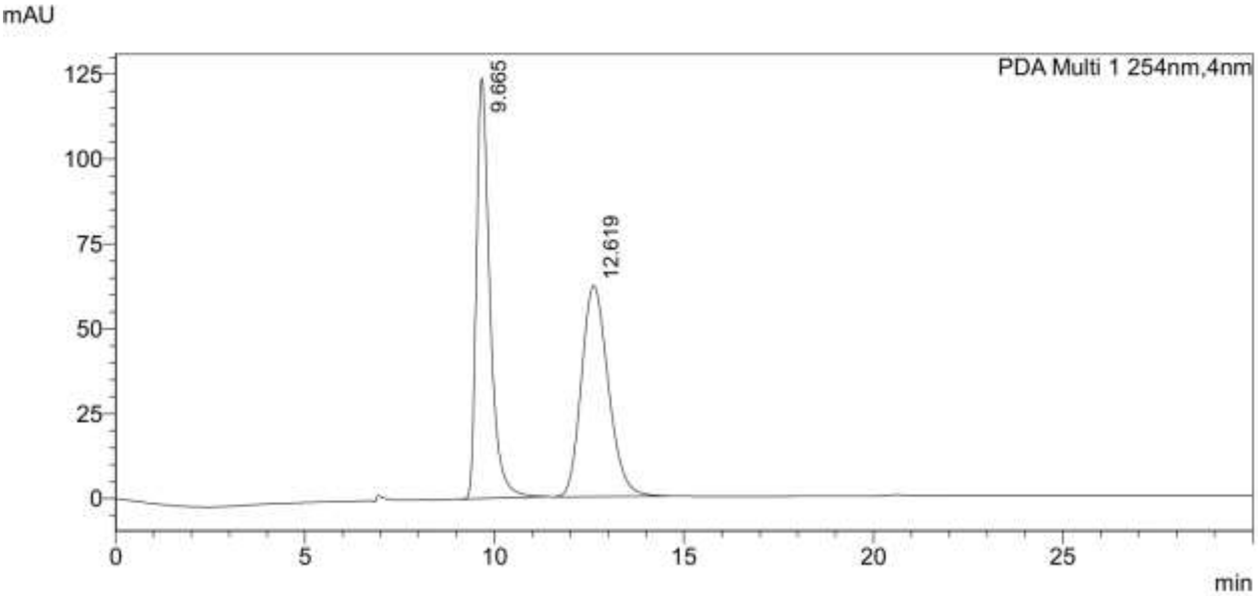

<Peak Table>

PDA Ch1 254nm

Peak#	Ret. Time	Area	Height	Area%
1	9.665	3061128	123864	49.942
2	12.619	3068223	62178	50.058
Total		6129351	186042	100.000

10-methyl-2,3-dihydronaphtho[2',1':3,4]phenanthro[1,2-*b*][1,4]dioxine (2o): synthesized according to **GP6**, run for 24 h at 140 °C, with L^1 as the ligand.

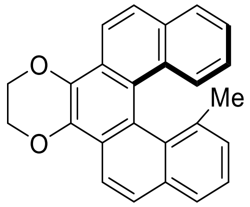

Yield: 94% (33 mg). Yellow solid. R_f 0.20 (pentane/EtOAc 20/1); ^{1}**H NMR** (500 MHz, Chloroform-*d*): δ 8.25 (d, *J* = 8.8 Hz, 1H), 8.16 (d, *J* = 8.6 Hz, 1H), 7.93 – 7.88 (m, 3H), 7.85 (d, *J* = 7.6 Hz, 1H), 7.76 (dd, *J* = 8.6, 1.0 Hz, 1H), 7.56 – 7.48 (m, 1H), 7.41 (ddd, *J* = 8.0, 6.8, 1.2 Hz, 1H), 7.17 (d, *J* = 6.8 Hz, 1H), 7.10 (ddd, *J* = 8.4, 6.8, 1.4 Hz, 1H), 4.76 – 4.60 (m, 2H), 4.60 – 4.43 (m, 2H), 1.52 (s, 3H). ^{13}**C NMR** (151 MHz, Chloroform-*d*) δ 135.8, 135.4, 135.0, 132.4, 131.9, 131.7, 130.7, 128.9, 128.0, 128.0, 127.2, 125.9, 125.7, 125.7, 125.4, 125.4, 125.3, 123.9, 123.0, 120.1, 118.6, 118.1, 65.2, 65.0, 23.5. **HRMS** (ESI): calcd. for $[C_{25}H_{18}O_2]^+$, $[M]^+$: 350.1301; found: 350.1305. **IR (ATR):** $\tilde{\nu}$ = 2923, 2362, 1753, 1619, 1459, 1401, 1371, 1280, 1114, 788 cm^{-1}. $[\alpha]_D^{20}$: –685 (c = 0.08, $CHCl_3$). **Mp**: 226.5 – 228.4 °C.

Chiral HPLC: (Chiralpak IA, 4.6 x 250 mm; n-heptane/i-PrOH 97/3, 0.5 mL/min, 254 nm; t_R(minor) = 11.0 min, t_R(major) = 12.2 min, >0.5:99.5 *er* (after crystallization).

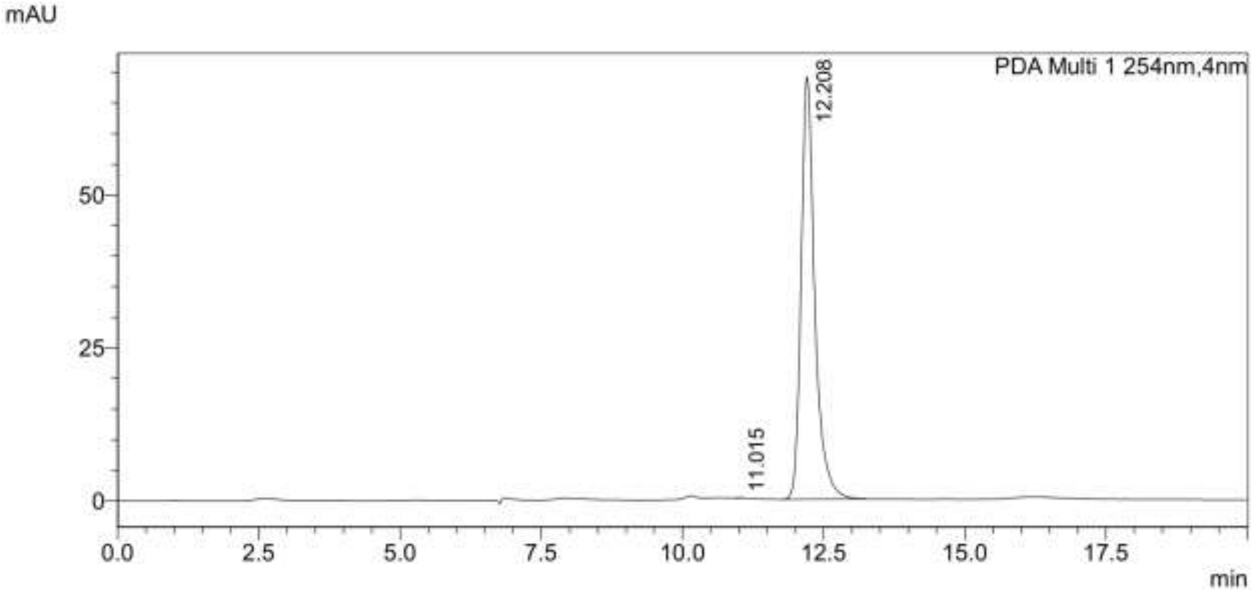

<Peak Table>

PDA Ch1 254nm

Peak#	Ret. Time	Area	Height	Area%
1	11.015	130	31	0.011
2	12.208	1166277	68980	99.989
Total		1166406	69010	100.000

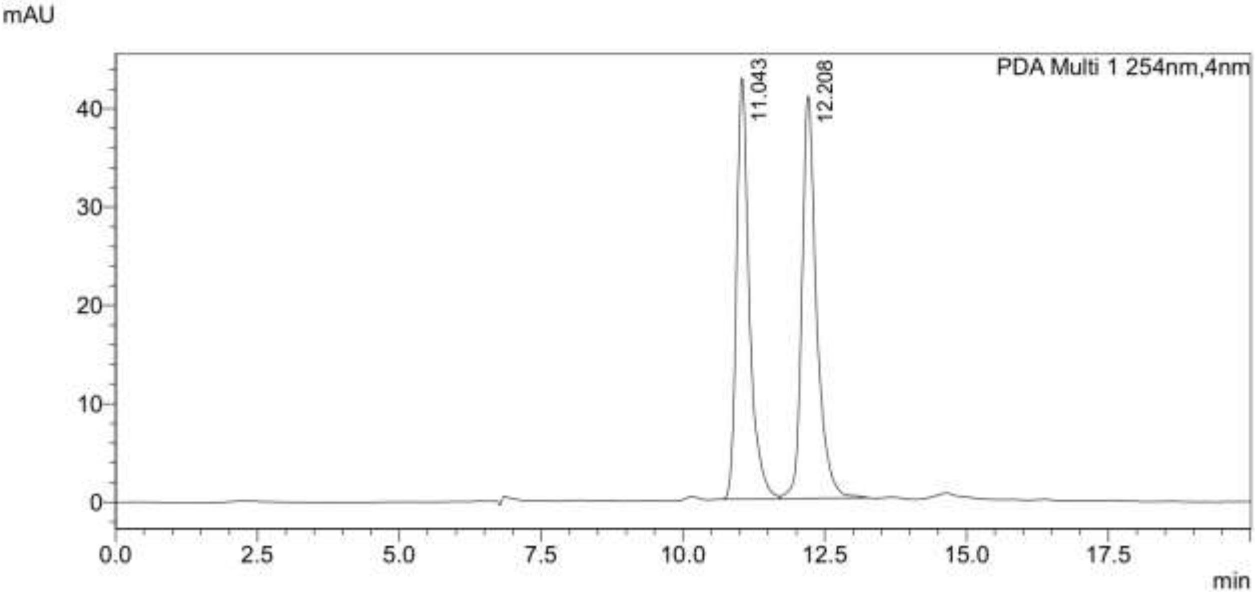

<Peak Table>

PDA Ch1 254nm

Peak#	Ret. Time	Area	Height	Area%
1	11.043	674010	42855	47.785
2	12.208	736499	40892	52.215
Total		1410510	83747	100.000

9-methylnaphtho[2',1':3,4]phenanthro[1,2-*d*][1,3]dioxole (2p): synthesized according to **GP6**, run for 24 h at 140 °C, with L^1 as the ligand.

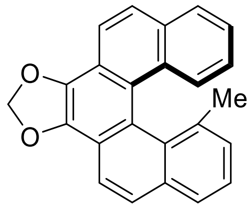

Yield: 95% (32 mg). Yellow crystal. ***R*f** 0.28 (pentane/EtOAc 20/1); **^{1}H NMR** (500 MHz, Chloroform-*d*): δ 7.96 (d, *J* = 8.7 Hz, 1H), 7.94 – 7.86 (m, 4H), 7.85 (d, *J* = 7.8 Hz, 1H), 7.81 (d, *J* = 8.5 Hz, 1H), 7.51 (t, *J* = 7.5 Hz, 1H), 7.43 (ddd, *J* = 8.0, 6.9, 1.2 Hz, 1H), 7.18 (dd, *J* = 7.2, 0.7 Hz, 1H), 7.11 (ddd, *J* = 8.4, 6.8, 1.4 Hz, 1H), 6.40 (d, *J* = 1.7 Hz, 1H), 6.35 (d, *J* = 1.7 Hz, 1H), 1.56 (s, 3H). **^{13}C NMR** (151 MHz, Chloroform-*d*) δ 140.2, 139.8, 135.9, 132.5, 132.3, 130.8, 129.2, 128.7, 128.4, 128.0, 125.9, 125.7, 125.7, 125.7, 125.6, 125.0, 120.9, 120.1, 118.4, 117.9, 117.9, 102.5, 23.6. **HRMS** (ESI): calcd. for $[C_{24}H_{16}O_2]^+$, $[M]^+$: 336.1145; found: 336.1147. **IR (ATR):** $\tilde{\nu}$ = 2926, 2362, 1764, 1722, 1643, 1441, 1404, 1097, 1072, 816, 733 cm^{-1}. **$[\alpha]_D^{20}$**: –998 (c = 0.1, $CHCl_3$). **Mp**: 219.2 – 221.2 °C.

Chiral HPLC: (Chiralpak IA, 4.6 x 250 mm; n-heptane/i-PrOH 97/3, 0.5 mL/min, 254 nm; t_R(minor) = 9.8 min, t_R(major) = 10.2 min, >0.5:99.5 *er* (after crystallization).

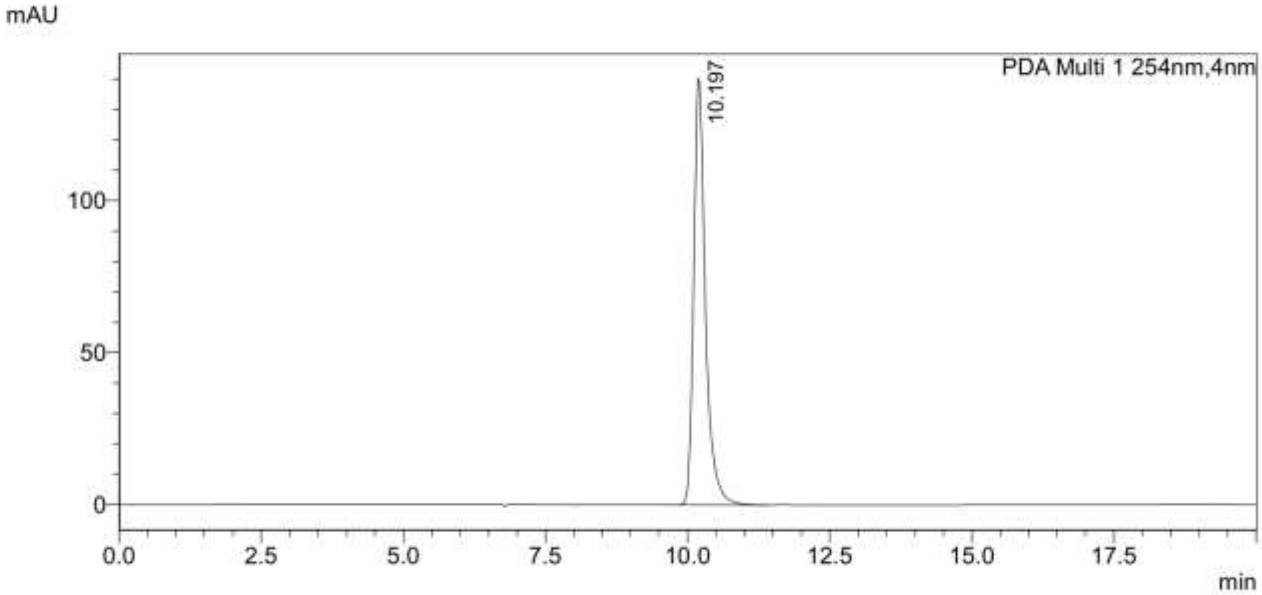

<Peak Table>

PDA Ch1 254nm

Peak#	Ret. Time	Area	Height	Area%
1	10.197	2017764	140466	100.000
Total		2017764	140466	100.000

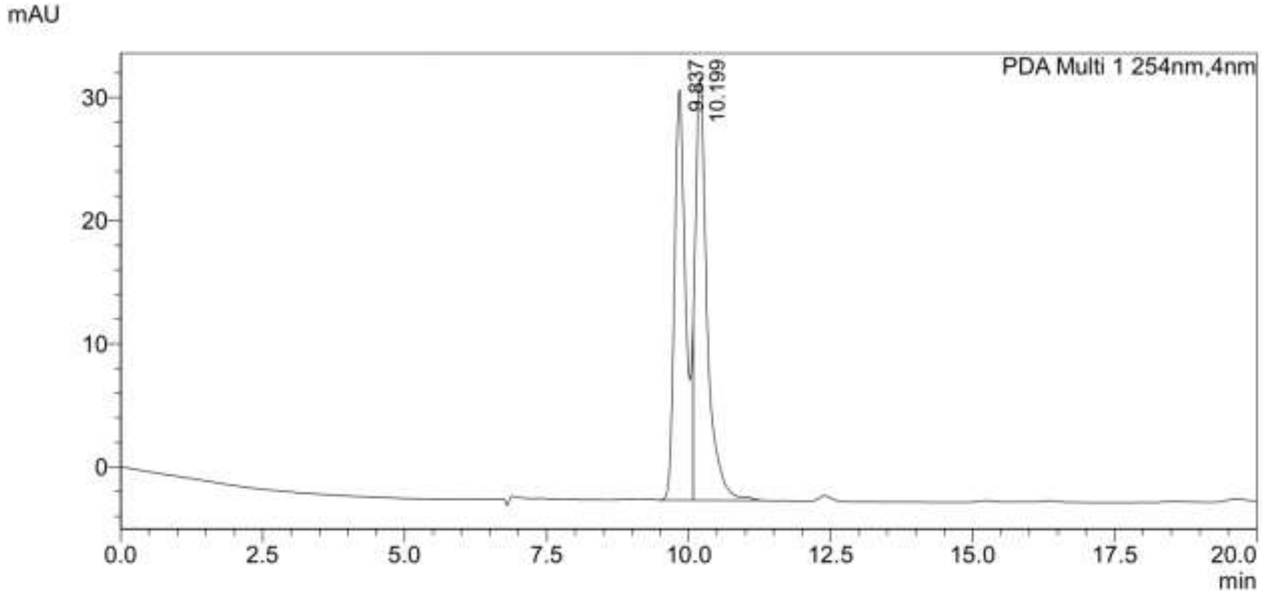

<Peak Table>

PDA Ch1 254nm

Peak#	Ret. Time	Area	Height	Area%
1	9.837	467470	33310	48.791
2	10.199	490638	34335	51.209
Total		958108	67645	100.000

2,11-dimethoxydibenzo[*c,g*]phenanthrene (2q): synthesized according to **GP6**, run for 9 h at 120 °C, with L^1 as the ligand.

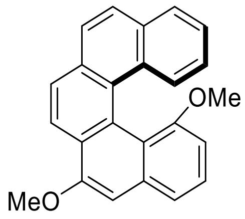

Yield: 91% (31 mg). Light brown crystal. $\boldsymbol{R_f}$ 0.25 (pentane/EtOAc 20/1); **^{1}H NMR** (500 MHz, Chloroform-*d*): δ 8.41 (d, *J* = 8.4 Hz, 1H), 8.09 (dd, *J* = 8.0, 0.8 Hz, 1H), 7.94 (d, *J* = 8.4 Hz, 1H), 7.92 (dd, *J* = 7.9, 1.4 Hz, 1H), 7.88 (d, *J* = 1.5 Hz, 2H), 7.55 – 7.47 (m, 2H), 7.45 (ddd, *J* = 8.0, 6.8, 1.2 Hz, 1H), 7.19 (ddd, *J* = 8.3, 6.8, 1.5 Hz, 1H), 7.16 (s, 1H), 6.65 (dd, *J* = 7.2, 1.8 Hz, 1H), 4.16 (s, 3H), 2.95 (s, 3H). **^{13}C NMR** (151 MHz, Chloroform-*d*) δ 156.6, 154.0, 135.0, 133.5, 130.9, 130.6, 128.6, 127.6, 127.4, 127.3, 127.3, 127.1, 126.1, 125.7, 125.5, 124.8, 124.6, 120.0, 119.2, 118.2, 104.5, 102.9, 56.0, 53.9. **HRMS** (ESI): calcd. for $[C_{24}H_{18}O_2+K]^+$, $[M+K]^+$: 377.0938; found: 377.0936. **IR (ATR):** $\tilde{\nu}$ = 2959, 2362, 1614, 1558, 1522, 1460, 1342, 1239, 1156, 1044, 908, 835, 759, 732, 678 cm^{-1}. **$[\alpha]_D^{20}$**: –3432 (c = 0.1, $CHCl_3$). **Mp**: 209.2 – 211.2 °C.

Chiral HPLC: (Chiralpak IA, 4.6 x 250 mm; n-heptane/i-PrOH 97/3, 0.5 mL/min, 254 nm; t_R(minor) = 10.2 min, t_R(major) = 12.9 min, 7.0:93.0 *er*.

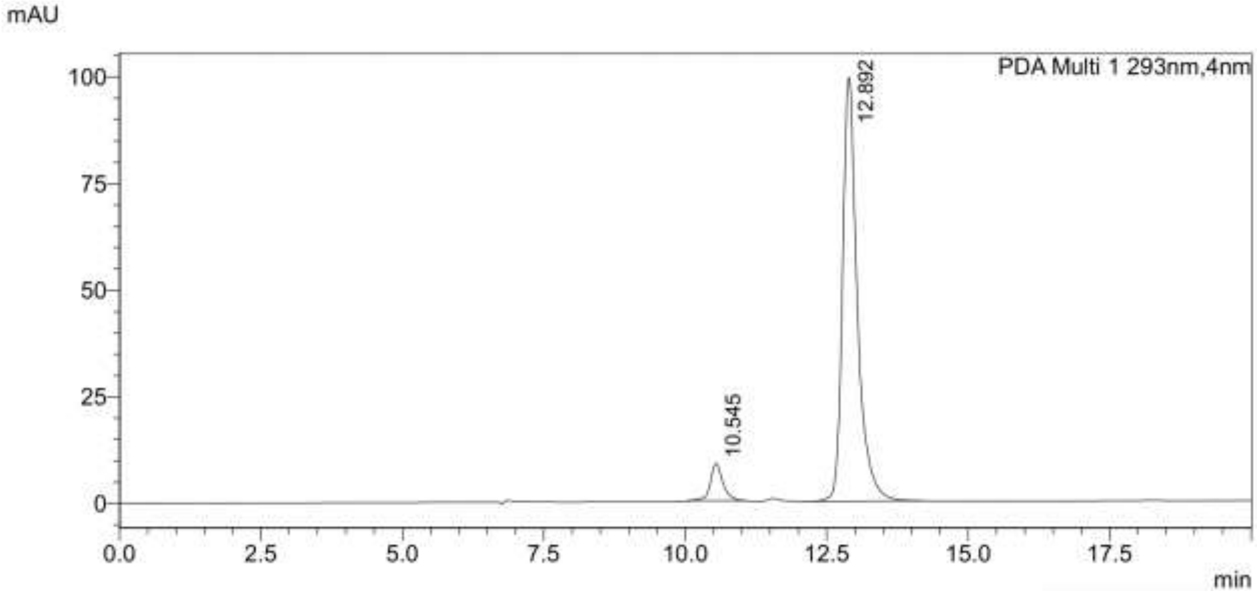

<Peak Table>

PDA Ch1 293nm

Peak#	Ret. Time	Area	Height	Area%
1	10.545	135103	8695	6.965
2	12.892	1804663	99360	93.035
Total		1939766	108055	100.000

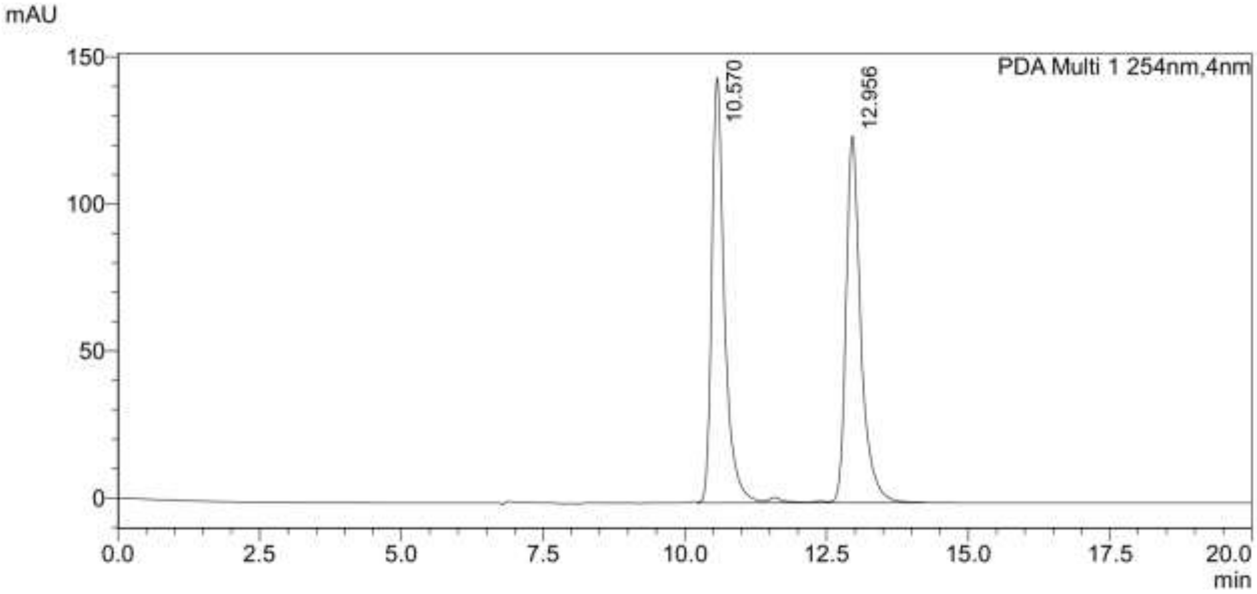

<Peak Table>

PDA Ch1 254nm

Peak#	Ret. Time	Area	Height	Area%
1	10.570	2306525	144839	50.455
2	12.956	2264888	124797	49.545
Total		4571413	269636	100.000

<u>3,4-difluorohexahelicene (2r):</u> synthesized according to **GP6**, run for 24 h at 140 °C, with L^2 as the ligand.

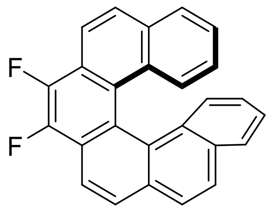

Yield: 45% (17 mg). White crystalline solid. ***R*f** 0.28 (pentane/EtOAc 20/1); **^{1}H NMR** (600 MHz, Chloroform-*d*): δ 8.30 (d, *J* = 8.3 Hz, 1H), 8.22 (d, *J* = 8.7 Hz, 1H), 8.08 (d, *J* = 8.3 Hz, 1H), 8.02 (d, *J* = 8.7 Hz, 1H), 7.94 (s, 2 H), 7.84 (d, *J* = 8.0 Hz, 2H), 7.51 (dd, *J* = 8.6, 3.4 Hz, 2H), 7.26 – 7.21 (m, 2H), 6.78 – 6.63 (m, 2H). **^{13}C NMR** (151 MHz, Chloroform-*d*) δ 143.5 (dd, $^{1}J_{C\text{-}F}$ = 251.3 Hz, $^{2}J_{C\text{-}F}$ = 11.7 Hz), 143.9 (dd, $^{1}J_{C\text{-}F}$ = 248.4 Hz, $^{2}J_{C\text{-}F}$ = 12.0 Hz), 132.2, 131.5, 131.2, 129.8, 129.5, 129.3 (d, $^{4}J_{C\text{-}F}$ = 1.7 Hz), 128.6 (d, $^{4}J_{C\text{-}F}$ = 1.3 Hz), 128.5, 128.0, 127.9, 127.8, 127.8, 127.8, 126.2, 126.1, 126.1, 125.6 (d, $^{4}J_{C\text{-}F}$ = 2.5 Hz), 125.5, 125.1, 124.5 (d, $^{2}J_{C\text{-}F}$ = 14.0 Hz), 122.8 (d, $^{2}J_{C\text{-}F}$ = 14.0 Hz), 121.6 (dd, $^{3}J_{C\text{-}F}$ = 4.6 Hz, $^{4}J_{C\text{-}F}$ = 2.4 Hz), 118.8 (dd, $^{3}J_{C\text{-}F}$ = 6.9 Hz, $^{3}J_{C\text{-}F}$ = 6.2 Hz), 117.8 (dd, J dd, $^{3}J_{C\text{-}F}$ = 7.2 Hz, $^{3}J_{C\text{-}F}$ = 5.4 Hz). **^{19}F NMR** (376 MHz, Chloroform-*d*) δ -151.3 (d, *J* = 18.2 Hz), -152.4 (d, *J* = 18.2 Hz). **HRMS** (ESI) (0.2 mM $AgNO_3$): calcd. for $[C_{26}H_{14}F_2]^+$, $[M]^+$: 364.1058; found: 364.1055. **IR (ATR):** $\tilde{\nu}$ = 3394, 1657, 1044, 1019, 995, 825, 762, 611 cm^{-1}. **$[\alpha]_D^{20}$**: –2172 (c = 1.0, $CHCl_3$). **Mp**: decomposed at 190 °C.

Chiral HPLC: (Chiralpak OJ-H, 4.6 x 250 mm; n-heptane/i-PrOH 90/10, 1.0 mL/min, 254 nm; t_R(minor) = 4.9 min, t_R(major) = 7.6 min, 2.2:97.8 *er*.

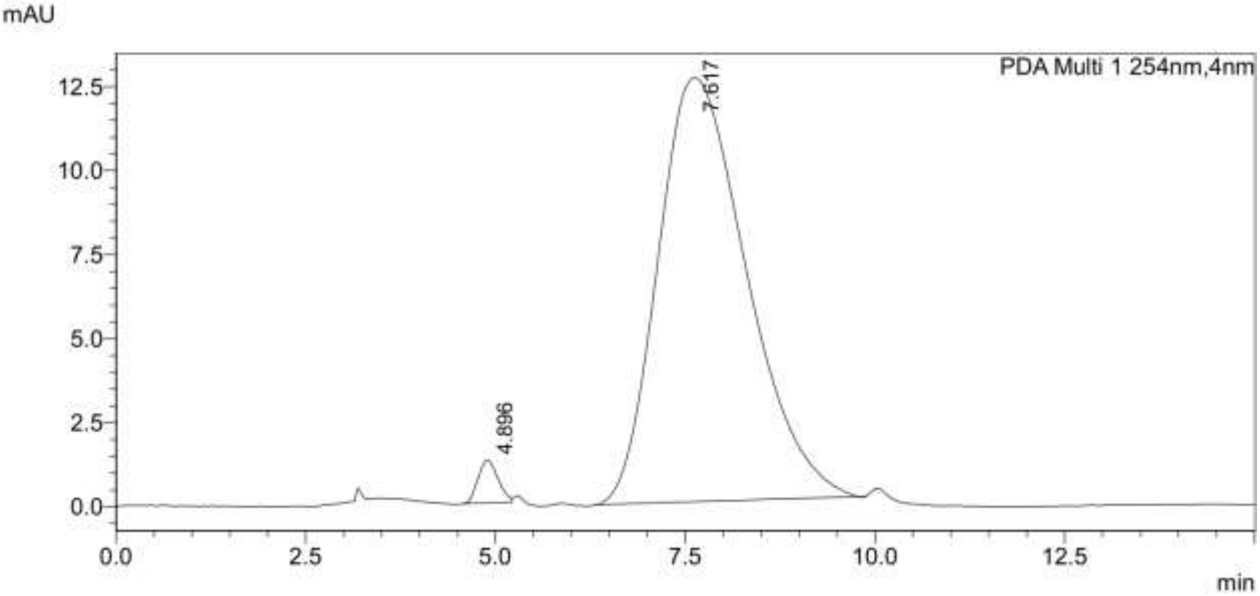

<Peak Table>

PDA Ch1 254nm

Peak#	Ret. Time	Area	Height	Area%
1	4.896	23179	1265	2.211
2	7.617	1025035	12626	97.789
Total		1048214	13890	100.000

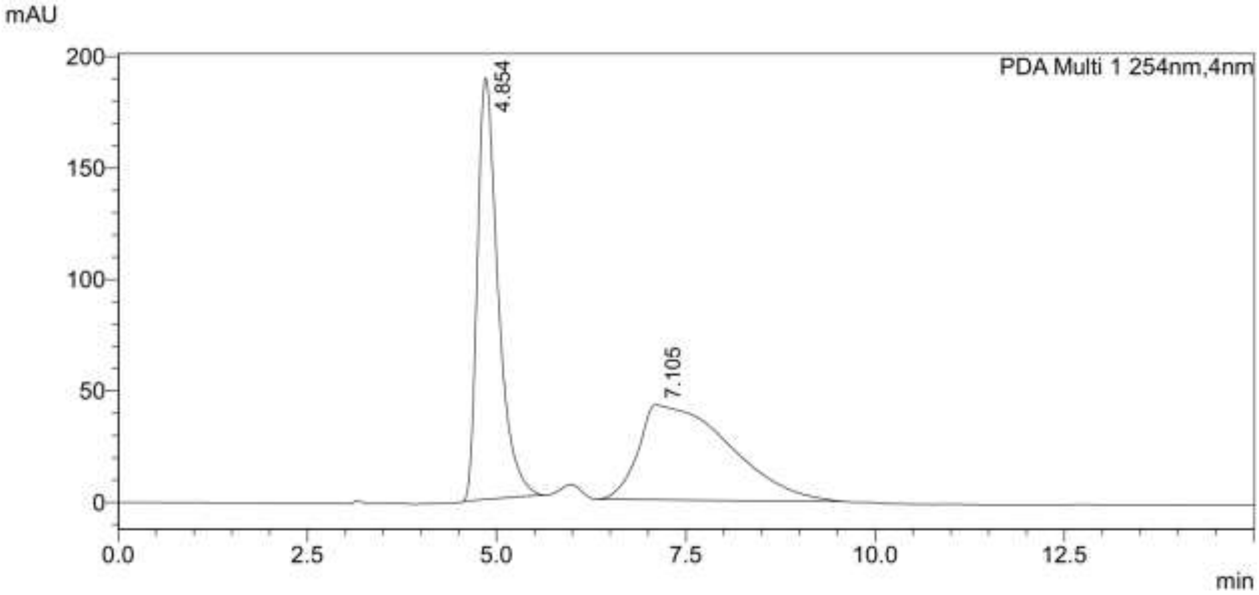

<Peak Table>

PDA Ch1 254nm

Peak#	Ret. Time	Area	Height	Area%
1	4.854	3596812	189276	50.313
2	7.105	3552037	42574	49.687
Total		7148849	231850	100.000

3,4-dimethoxyhexahelicene (2s): synthesized according to **GP6**, run for 24 h at 140 °C, with L^1 as the ligand.

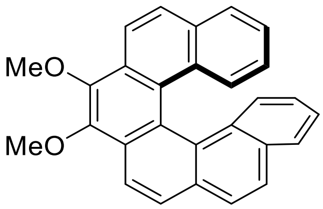

Yield: 70% (27 mg). Light yellow foam. ***R*f** 0.40 (cyclohexane/EtOAc 50/1); **^{1}H NMR** (400 MHz, Chloroform-*d*): δ 8.39 (d, *J* = 8.5 Hz, 1H), 8.34 (d, *J* = 8.8 Hz, 1H), 8.00 (d, *J* = 8.4 Hz, 1H), 7.98 – 7.85 (m, 3H), 7.81 (dt, *J* = 8.0, 1.9 Hz, 2H), 7.53 (dd, *J* = 13.9, 8.5 Hz, 2H), 7.19 (dddd, *J* = 8.1, 6.9, 5.6, 1.2 Hz, 2H), 6.66 (dddd, *J* = 8.5, 6.9, 2.7, 1.5 Hz, 2H), 4.23 (s, 3H), 4.18 (s, 3H). **^{13}C NMR** (151 MHz, Chloroform-*d*) δ 145.1, 144.8, 132.0, 131.4, 130.8, 130.0, 129.9, 129.6, 128.2, 128.2, 128.0, 127.8, 127.8, 127.7, 127.7, 127.6, 127.4, 126.3, 125.9, 125.7, 125.4, 124.9, 124.7, 122.0, 120.5, 119.8, 61.5, 61.4. **HRMS** (ESI): calcd. for $[C_{28}H_{20}O_2]^+$, $[M]^+$: 388.1458; found: 388.1462. **IR (ATR):** $\tilde{\nu}$ = 2935, 1601, 1461, 1382, 1277, 1096, 1065, 829, 751 cm^{-1}. **$[\alpha]_D^{20}$**: –2217 (c = 0.1, $CHCl_3$).

Chiral HPLC: (Chiralpak OJ, 4.6 x 250 mm; n-heptane/i-PrOH 99/1, 0.3 mL/min, 254 nm; t_R(minor) = 27.4 min, t_R(major) = 38.6 min, 7.8:92.2 *er*.

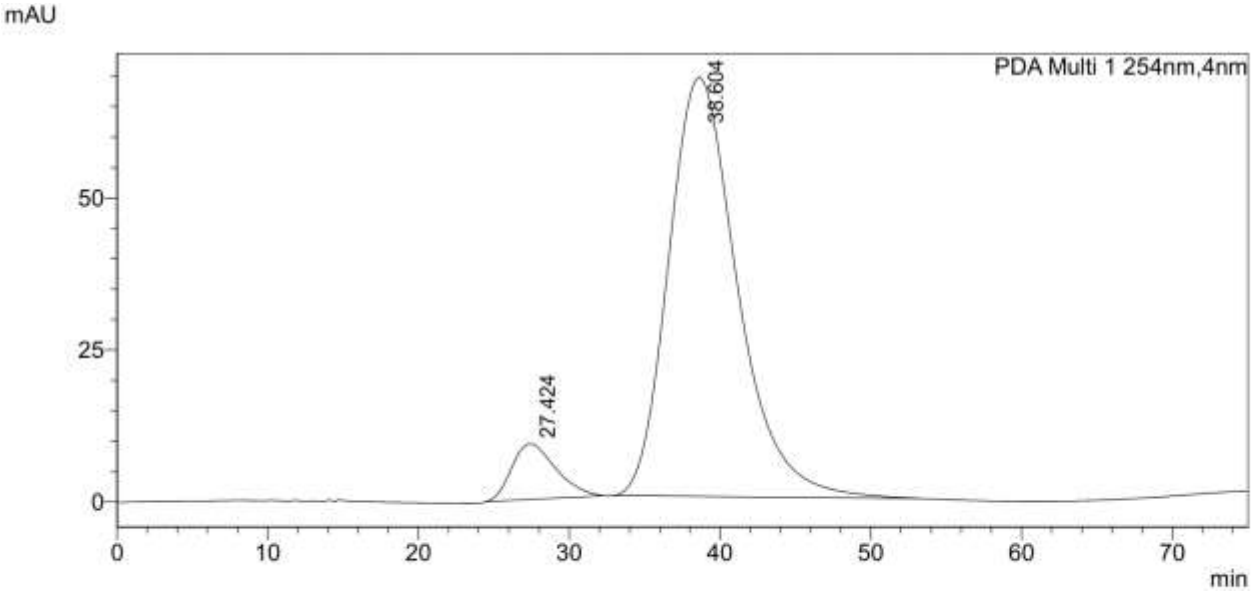

<Peak Table>

PDA Ch1 254nm

Peak#	Ret. Time	Area	Height	Area%
1	27.424	1850700	9140	7.811
2	38.604	21843916	68847	92.189
Total		23694616	77986	100.000

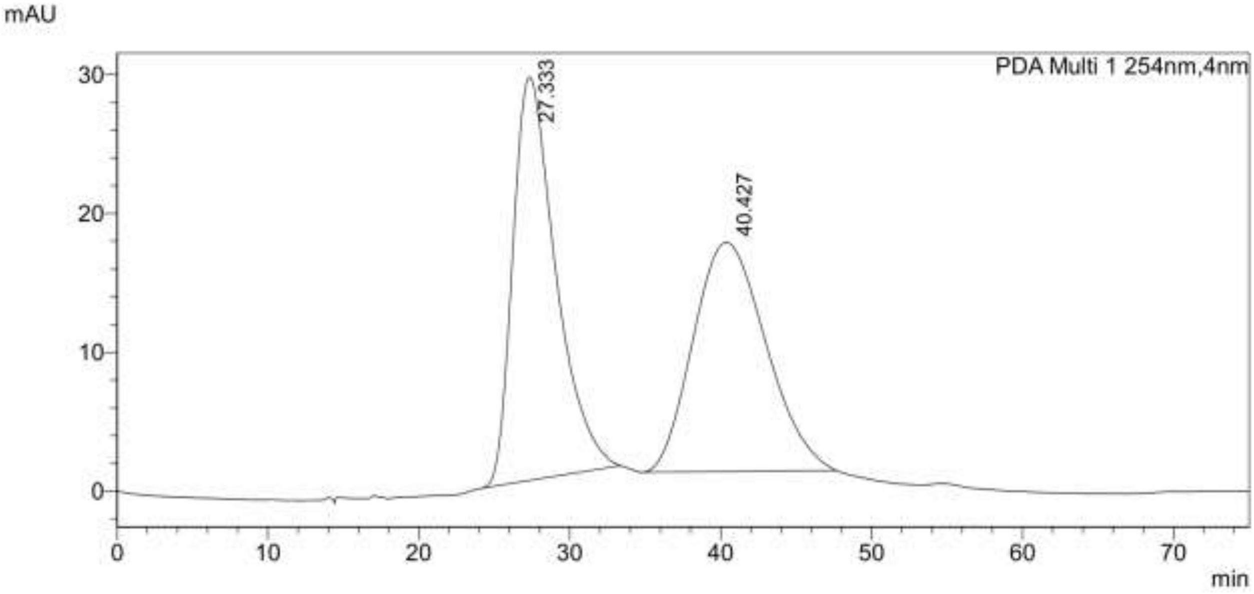

<Peak Table>

PDA Ch1 254nm

Peak#	Ret. Time	Area	Height	Area%
1	27.333	5736767	29064	50.905
2	40.427	5532891	16510	49.095
Total		11269658	45574	100.000

2,3-dihydrophenanthro[3',4':3,4]phenanthro[1,2-*b*][1,4]dioxine (2t): synthesized according to **GP6**, run for 24 h at 140 °C, with L^1 as the ligand.

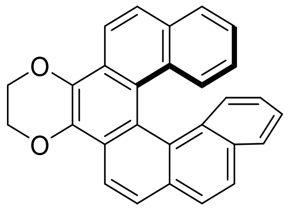

Yield: 73% (28 mg). Yellow foam. ***R*f** 0.30 (cyclohexane/EtOAc 50/1); **^{1}H NMR** (500 MHz, Chloroform-*d*): δ 8.34 (d, *J* = 8.4 Hz, 1H), 8.27 (d, *J* = 8.8 Hz, 1H), 7.97 (d, *J* = 8.4 Hz, 1H), 7.95 – 7.88 (m, 2H), 7.86 (d, *J* = 8.6 Hz, 1H), 7.79 (dd, *J* = 8.0, 1.3 Hz, 2H), 7.57 (d, *J* = 8.5 Hz, 1H), 7.50 (d, *J* = 8.5 Hz, 1H), 7.17 (dddd, *J* = 14.8, 8.0, 6.8, 1.2 Hz, 2H), 6.72 – 6.59 (m, 2H), 4.73 – 4.67 (m, 2H), 4.63 – 4.50 (m, 2H). **^{13}C NMR** (151 MHz, Chloroform-*d*) δ 135.3, 135.0, 132.0, 131.0, 130.2, 130.2, 129.9, 128.3, 128.2, 128.1, 127.9, 127.7, 127.6, 127.4, 127.3, 126.6, 126.4, 125.6, 125.0, 124.9, 124.6, 124.4, 123.5, 119.8, 119.3, 118.7, 65.3, 65.1. **HRMS** (ESI): calcd. for $[C_{28}H_{18}O_2]^+$, $[M]^+$: 386.1301; found: 386.1304. **IR (ATR):** $\tilde{\nu}$ = 2984, 2362, 1752, 1607, 1396, 1363, 1282, 1108, 1057, 907, 834, 748, 680 cm^{-1}. **$[\alpha]_D^{20}$**: –1521 (c = 0.1, $CHCl_3$).

Chiral HPLC: (Chiralpak IA, 4.6 x 250 mm; n-heptane/i-PrOH 90/10, 1.0 mL/min, 254 nm; t_R(minor) = 5.1 min, t_R(major) = 5.6 min, 10.1:89.9 *er*.

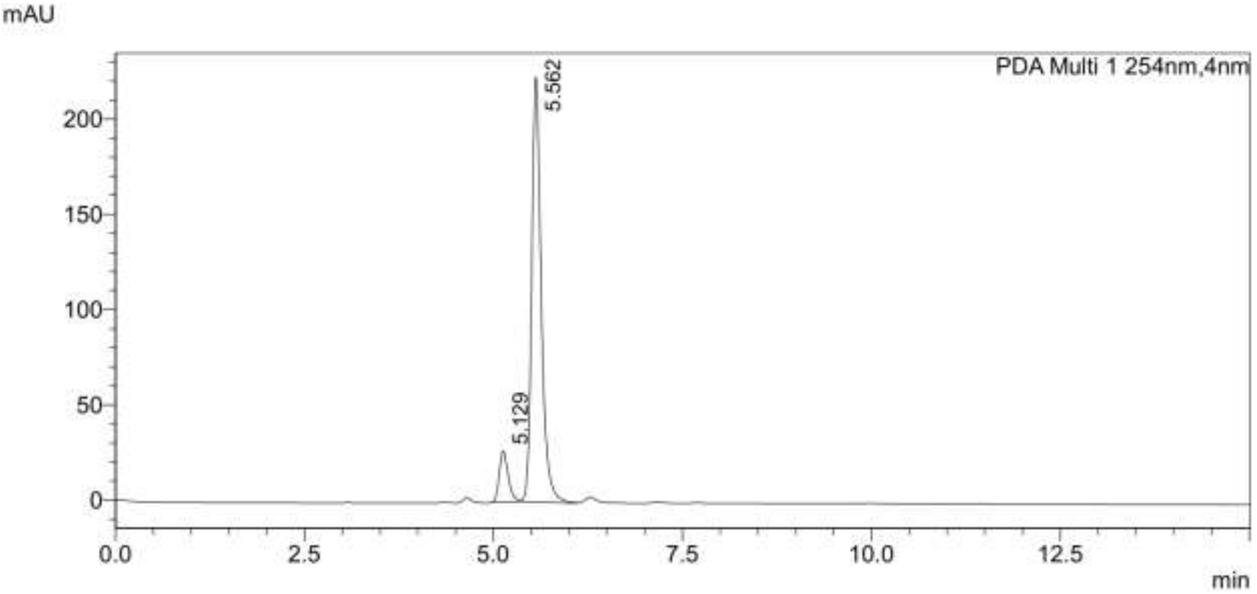

<Peak Table>

PDA Ch1 254nm

Peak#	Ret. Time	Area	Height	Area%
1	5.129	221446	26987	10.138
2	5.562	1962915	222930	89.862
Total		2184361	249917	100.000

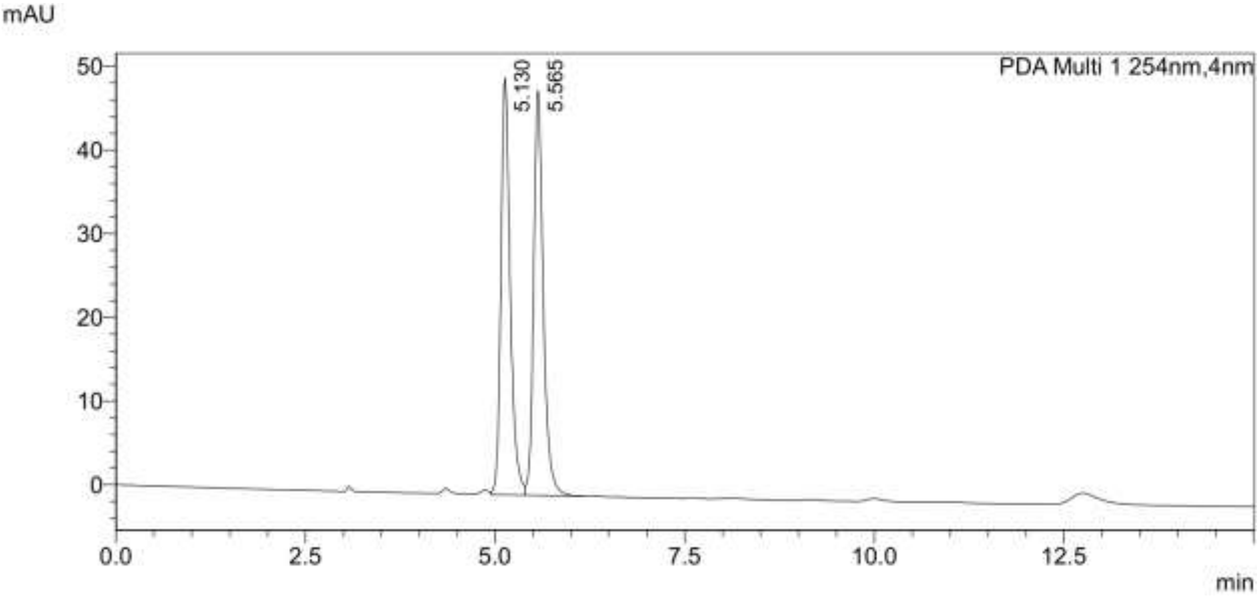

<Peak Table>

PDA Ch1 254nm

Peak#	Ret. Time	Area	Height	Area%
1	5.130	423159	49862	49.731
2	5.565	427742	48442	50.269
Total		850901	98304	100.000

3,4,14-trifluorohexahelicene (2u): synthesized according to **GP6**, run for 24 h at 140 °C, with L^2 as the ligand.

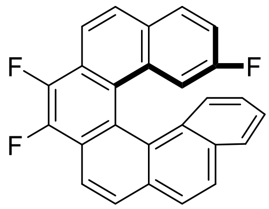

Yield: 42% (16 mg). White crystal. ***R*f** 0.28 (cyclohexane/EtOAc 20/1); **^{1}H NMR** (400 MHz, Chloroform-*d*): δ 8.31 (dd, *J* = 8.4, 0.6 Hz, 1H), 8.19 (d, *J* = 8.8 Hz, 1H), 8.09 (d, *J* = 8.5 Hz, 1H), 8.04 – 7.92 (m, 3H), 7.87 (dd, *J* = 8.0, 1.3 Hz, 1H), 7.83 (dd, *J* = 8.8, 6.1 Hz, 1H), 7.50 (d, *J* = 1.0 Hz, 1H), 7.30 (ddd, *J* = 8.0, 6.9, 1.1 Hz, 1H), 7.13 (dd, *J* = 12.2, 2.6 Hz, 1H), 7.02 (ddd, *J* = 8.9, 7.8, 2.6 Hz, 1H), 6.74 (ddd, *J* = 8.5, 6.9, 1.4 Hz, 1H). **^{13}C NMR** (151 MHz, Chloroform-*d*) δ 160.8 (d, $^1J_{C\text{-F}}$ = 245.4 Hz), 143.7 (dd, $^1J_{C\text{-F}}$ = 251.5 Hz, $^2J_{C\text{-F}}$ = 10.2 Hz), 143.0 (dd, $^1J_{C\text{-F}}$ = 247.0 Hz, $^2J_{C\text{-F}}$ = 7.9 Hz), 132.3, 131.5, 131.0 (d, $^3J_{C\text{-F}}$ = 10.0 Hz), 130.2, 130.1, 129.7, 128.9, 128.8, 128.4, 128.1, 127.3, 127.2, 126.4, 126.2, 125.2, 124.9, 124.5 (d, $^2J_{C\text{-F}}$ = 14.0 Hz), 123.4 (d, $^2J_{C\text{-F}}$ = 14.4 Hz), 121.7, 118.8 (t, $^3J_{C\text{-F}}$ = 6.5 Hz), 117.3 (t, $^3J_{C\text{-F}}$ = 5.1 Hz), 115.6 (d, $^2J_{C\text{-F}}$ = 24.4 Hz), 112.6 (d, $^2J_{C\text{-F}}$ = 24.1 Hz). **^{19}F NMR** (376 MHz, Chloroform-*d*) δ -115.0, -151.0 (d, *J* = 18.1 Hz), -151.3 (d, *J* = 18.1 Hz). **HRMS** (ESI) (0.2 mM $AgNO_3$): calcd. for $[C_{26}H_{13}F_3+Ag]^+$, $[M+Ag]^+$: 489.0015; found: 489.0010. **IR (ATR):** $\tilde{\nu}$ = 2934, 1604, 1516, 1460, 1368, 1271, 1196, 1163, 1123, 1062, 1020, 908, 843, 733, 697 cm^{-1}. **$[\alpha]_D^{20}$**: –2060 (c = 0.04, $CHCl_3$). **Mp**: 235.2 – 237.2 °C.

Chiral HPLC: (Chiralpak IA, 4.6 x 250 mm; n-heptane/i-PrOH 97/3, 0.5 mL/min, 254 nm; t_R(minor) = 9.1 min, t_R(major) = 9.8 min, 3.3:96.7 *er*.

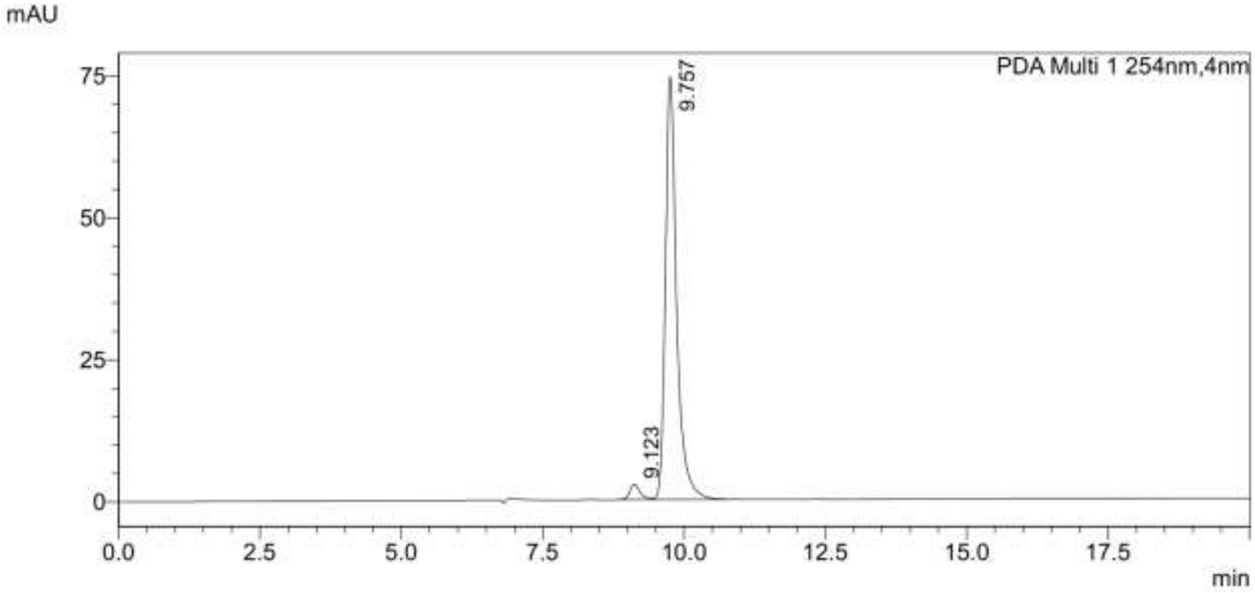

<Peak Table>

PDA Ch1 254nm

Peak#	Ret. Time	Area	Height	Area%
1	9.123	34715	2704	3.268
2	9.757	1027398	74435	96.732
Total		1062112	77139	100.000

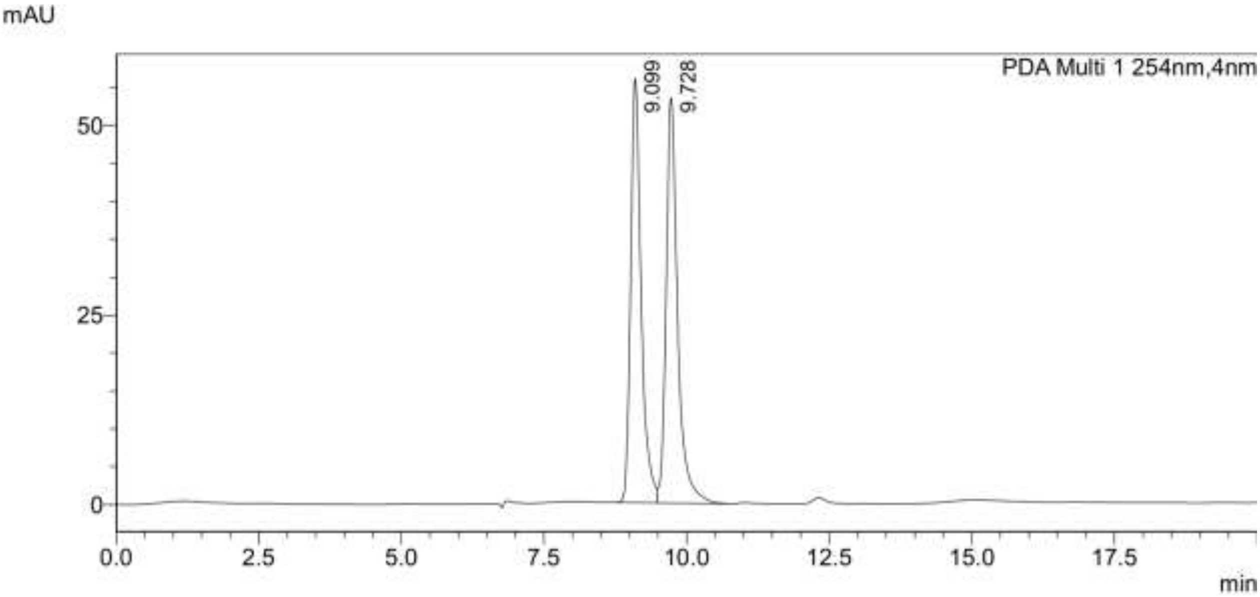

<Peak Table>

PDA Ch1 254nm

Peak#	Ret. Time	Area	Height	Area%
1	9.099	717718	55988	48.871
2	9.728	750869	53533	51.129
Total		1468587	109521	100.000

<u>3,4,15-trifluorohexahelicene (2v):</u> synthesized according to **GP6**, run for 24 h at 140 °C, with L^1 as the ligand.

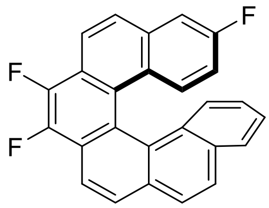

Yield: 91% (35 mg). Light yellow solid. $\boldsymbol{R}_\mathbf{f}$ 0.10 (cyclohexane/EtOAc 50/1); **^{1}H NMR** (400 MHz, Chloroform-*d*): δ 8.27 (d, *J* = 8.4 Hz, 1H), 8.23 (d, *J* = 8.8 Hz, 1H), 8.05 (d, *J* = 8.4 Hz, 1H), 7.98 – 7.89 (m, 3H), 7.84 (dd, *J* = 8.0, 1.3 Hz, 1H), 7.55 – 7.40 (m, 3H), 7.35 – 7.18 (m, 1H), 6.72 (ddd, *J* = 8.5, 6.9, 1.4 Hz, 1H), 6.44 (ddd, *J* = 9.3, 8.0, 2.8 Hz, 1H). **^{13}C NMR** (151 MHz, Chloroform-*d*) δ 160.4 (d, $^{1}J_{C\text{-}F}$ = 247.8 Hz), 143.0 (dd, $^{1}J_{C\text{-}F}$ = 250.1 Hz, $^{2}J_{C\text{-}F}$ = 12.1 Hz), 143.0 (dd, $^{1}J_{C\text{-}F}$ = 250.9 Hz, $^{2}J_{C\text{-}F}$ = 11.9 Hz), 132.9 (d, $^{3}J_{C\text{-}F}$ = 8.8 Hz), 132.2, 131.3 (d, $^{4}J_{C\text{-}F}$ = 1.5 Hz), 130.3 (d, $^{3}J_{C\text{-}F}$ = 8.6 Hz), 129.5, 128.8 (d, $^{4}J_{C\text{-}F}$ = 1.7 Hz), 128.6, 128.5 (dd, $^{4}J_{C\text{-}F}$ = 4.1, 1.6 Hz), 127.9, 127.8, 127.7 (d, $^{4}J_{C\text{-}F}$ = 1.9 Hz), 126.3, 126.2, 125.5 (d, $^{4}J_{C\text{-}F}$ = 2.7 Hz), 125.3, 124.7 (d, $^{2}J_{C\text{-}F}$ = 14.2 Hz), 122.3 (d, $^{2}J_{C\text{-}F}$ = 14.1 Hz), 121.3 (t, $^{4}J_{C\text{-}F}$ = 2.4 Hz), 119.2 (dd, $^{3}J_{C\text{-}F}$ = 7.2, 5.2 Hz), 118.8 (t, $^{3}J_{C\text{-}F}$ = 6.5 Hz), 114.7 (d, $^{2}J_{C\text{-}F}$ = 23.8 Hz), 111.6 (d, $^{2}J_{C\text{-}F}$ = 20.6 Hz). **^{19}F NMR** (376 MHz, Chloroform-*d*) δ -114.9 (d, *J* = 2.5 Hz), -151.1 (d, *J* = 18.1 Hz), -152.4 (dd, *J* = 18.1, 2.0 Hz). **HRMS** (ESI) (0.2 mM $AgNO_3$): calcd. for $[C_{26}H_{13}F_3]^+$, $[M]^+$: 382.0964; found: 382.0960. **IR (ATR):** $\tilde{\nu}$ = 2923, 2362, 1650, 1494, 1403, 1283, 1249, 1150, 1054, 958, 796, 672 cm^{-1}. **$[\alpha]_D^{20}$**: –1678 (c = 1.0, $CHCl_3$). **Mp**: 167.5 – 169.3 °C.

Chiral HPLC: (Chiralpak IA, 4.6 x 250 mm; n-heptane/i-PrOH 97/3, 0.5 mL/min, 254 nm; t_R(minor) = 8.8 min, t_R(major) = 9.5 min, 7.4:92.6 *er*.

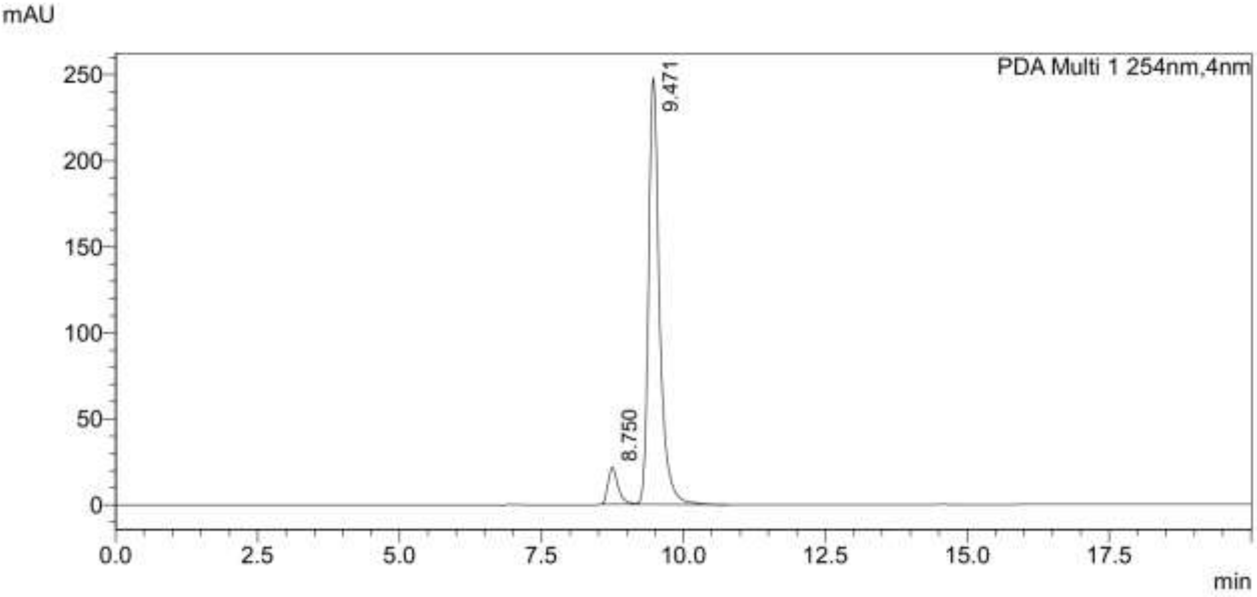

<Peak Table>

PDA Ch1 254nm

Peak#	Ret. Time	Area	Height	Area%
1	8.750	266219	21553	7.374
2	9.471	3343840	247999	92.626
Total		3610059	269552	100.000

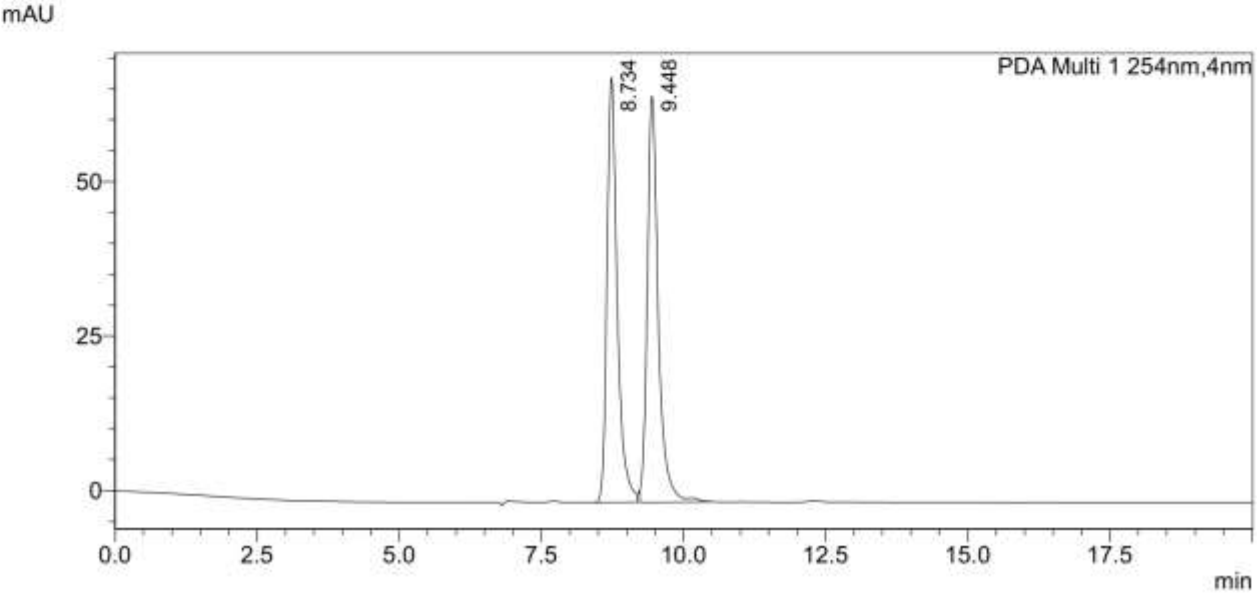

<Peak Table>

PDA Ch1 254nm

Peak#	Ret. Time	Area	Height	Area%
1	8.734	865514	68787	49.442
2	9.448	885053	65728	50.558
Total		1750566	134514	100.000

3,4-difluoro-15-methoxyhexahelicene (2w): synthesized according to **GP6**, run for 24 h at 140 °C, with L^1 as the ligand.

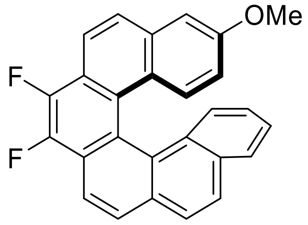

Yield: 93% (37 mg). Yellow solid. R_f 0.20 (cyclohexane/EtOAc 50/1); **^{1}H NMR** (500 MHz, Chloroform-*d*): δ 8.28 (d, *J* = 8.3 Hz, 1H), 8.20 (d, *J* = 8.7 Hz, 1H), 8.05 (d, *J* = 8.5 Hz, 1H), 7.94 (d, J = 9.0 Hz, 3H), 7.87 – 7.80 (m, 1H), 7.58 – 7.53 (m, 1H), 7.41 (d, J = 9.3 Hz, 1H), 7.26 – 7.21 (m, 1H), 7.18 (d, J = 2.7 Hz, 1H), 6.73 (ddd, *J* = 8.4, 6.8, 1.4 Hz, 1H), 6.34 (dd, *J* = 9.3, 2.7 Hz, 1H), 3.84 (s, 3H). **^{13}C NMR** (151 MHz, Chloroform-*d*) δ 157.5, 143.2 (dd, $^1J_{C\text{-}F}$ = 250.7 Hz, $^2J_{C\text{-}F}$ = 12.6 Hz), 142.6 (dd, $^1J_{C\text{-}F}$ = 249.7 Hz, $^2J_{C\text{-}F}$ = 12.1 Hz), 133.1, 132.1, 131.2, 129.7, 129.4, 128.7 (d, $^4J_{C\text{-}F}$ = 1.7 Hz), 128.5, 128.4, 128.0, 127.9, 127.8, 126.2, 126.2, 125.9 (d, $^4J_{C\text{-}F}$ = 2.7 Hz), 125.2, 124.6 (d, $^2J_{C\text{-}F}$ = 14.2 Hz), 124.3, 121.5 (d, $^2J_{C\text{-}F}$ = 13.8 Hz), 121.3, 118.8 (t, $^3J_{C\text{-}F}$ = 6.5 Hz), 118.4 (dd, $^3J_{C\text{-}F}$ = 6.9, 5.5 Hz), 116.5, 107.2, 55.4. **^{19}F NMR** (376 MHz, Chloroform-*d*) δ -151.4 (d, *J* = 18.3 Hz), -153.9 (d, *J* = 18.3 Hz). **HRMS** (ESI): calcd. for $[C_{27}H_{16}F_2O]^+$, $[M]^+$: 394.1164; found: 394.1161. **IR (ATR):** $\tilde{\nu}$ = 2987, 2336, 1651, 1616, 1494, 1403, 1253, 1047, 832, 797 cm^{-1}. **$[\alpha]_D^{20}$**: –2760 (c = 1.0, $CHCl_3$). **Mp**: 153.2 – 155.2 °C.

Chiral HPLC: (Chiralpak IA, 4.6 x 250 mm; n-heptane/i-PrOH 97/3, 0.5 mL/min, 254 nm; t_R(minor) = 9.8 min, t_R(major) = 10.8 min, 7.2:92.8 *er*.

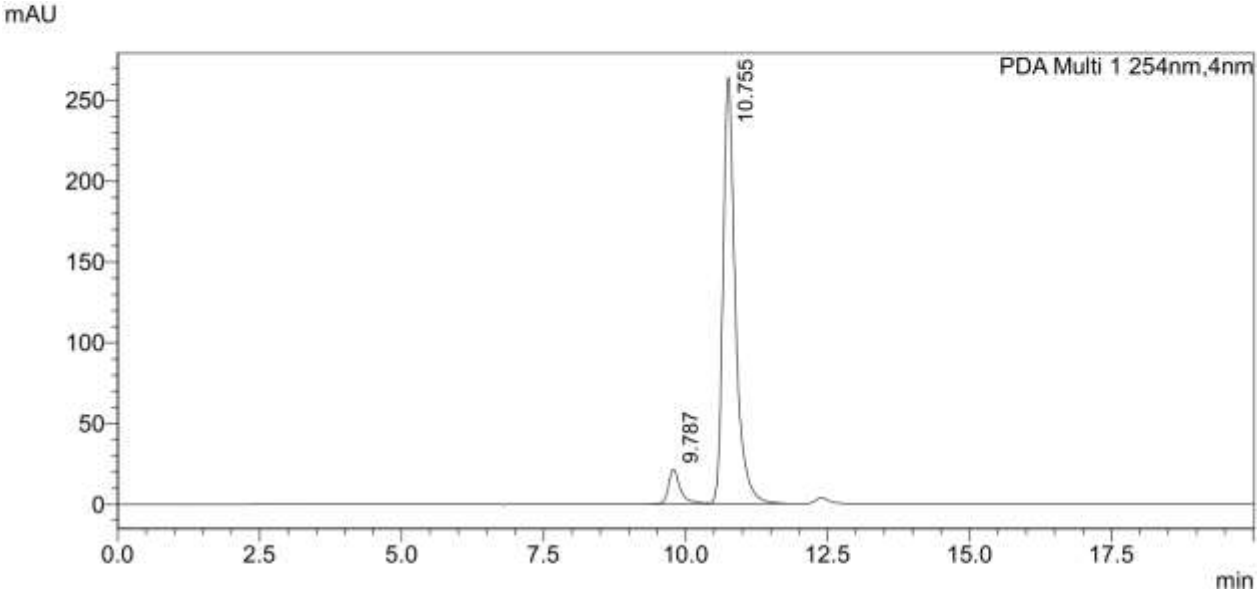

<Peak Table>

PDA Ch1 254nm

Peak#	Ret. Time	Area	Height	Area%
1	9.787	309823	21388	7.212
2	10.755	3986356	264030	92.788
Total		4296179	285418	100.000

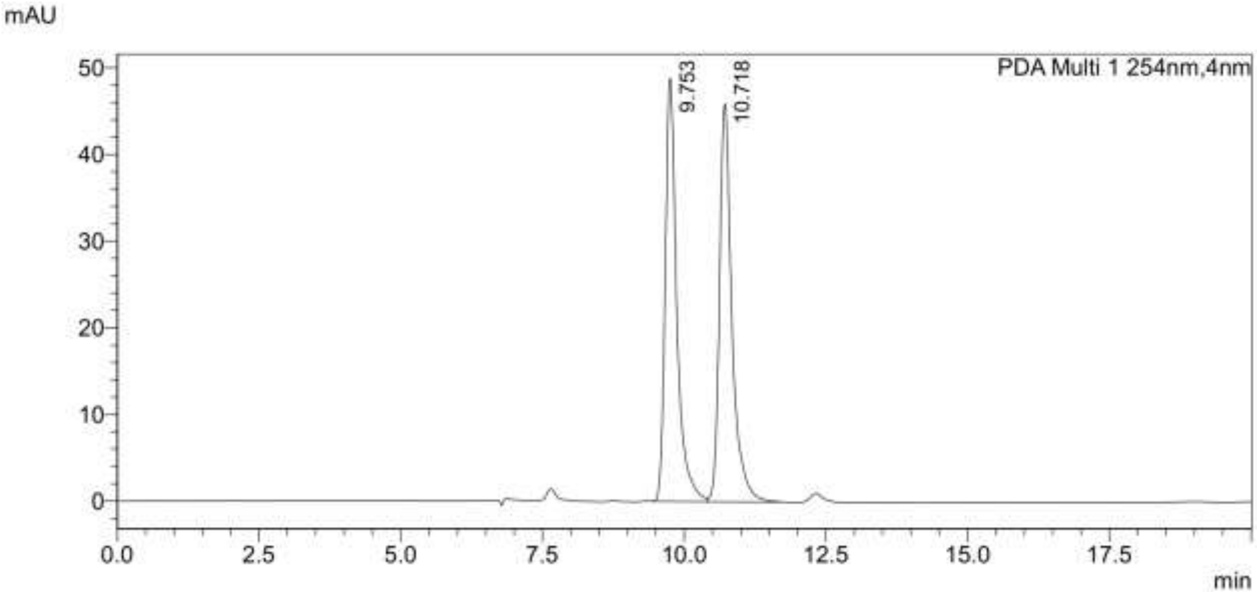

<Peak Table>

PDA Ch1 254nm

Peak#	Ret. Time	Area	Height	Area%
1	9.753	691136	48839	49.646
2	10.718	700985	45916	50.354
Total		1392121	94755	100.000

9,10-difluorobenzo[5,6]phenanthro[4,3-*f*]quinoline (2x): synthesized according to **GP6**, run for 24 h at 140 °C, with L^1 as the ligand.

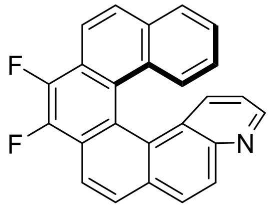

Yield: 75% (27 mg). Yellow solid. ***R*f** 0.3 (cyclohexane/EtOAc 5/1); **^{1}H NMR** (400 MHz, Chloroform-*d*): δ 8.63 (dd, *J* = 4.3, 1.6 Hz, 1H), 8.36 (d, *J* = 8.4 Hz, 1H), 8.22 (d, *J* = 8.8 Hz, 1H), 8.19 (s, 2H), 8.12 (d, *J* = 8.4 Hz, 1H), 8.03 (d, *J* = 8.8 Hz, 1H), 7.87 (dd, *J* = 8.1, 1.3 Hz, 1H), 7.81 (dd, *J* = 8.5, 1.6 Hz, 1H), 7.50 (d, *J* = 8.5 Hz, 1H), 7.35 – 7.27 (m, 1H), 6.76 (ddd, *J* = 8.4, 6.9, 1.4 Hz, 1H), 6.62 (dd, *J* = 8.6, 4.2 Hz, 1H). **^{13}C NMR** (151 MHz, Chloroform-*d*) δ 149.6, 147.7, 143.4 (dd, $^1J_{C\text{-}F}$ = 251.5 Hz, $^2J_{C\text{-}F}$ = 11.9 Hz), 143.3 (dd, $^1J_{C\text{-}F}$ = 251.1 Hz, $^2J_{C\text{-}F}$ = 12.1 Hz), 135.1, 131.8, 131.1, 130.2, 129.8, 129.6 (d, $^4J_{C\text{-}F}$ = 1.7 Hz), 129.0, 128.6, 128.3, 128.0, 127.4, 126.5, 126.1, 125.3, 125.1, 124.7 (d, $^2J_{C\text{-}F}$ = 14.3 Hz), 123.2 (d, $^2J_{C\text{-}F}$ = 14.2 Hz), 121.4, 119.7 (t, $^3J_{C\text{-}F}$ = 6.5 Hz), 119.5, 117.9 (t, $^3J_{C\text{-}F}$ = 6.9, 5.5 Hz). **^{19}F NMR** (376 MHz, Chloroform-*d*) δ -155.0 (d, *J* = 18.1 Hz), -156.4 (d, *J* = 18.0 Hz). **HRMS** (ESI): calcd. for $[C_{25}H_{14}F_2N+H]^+$, $[M+H]^+$: 366.1089; found: 366.1086. **IR (ATR):** $\tilde{\nu}$ = 2987, 2336, 1651, 1616, 1494, 1403, 1253, 1094, 723, 650 cm^{-1}. **$[\alpha]_D^{20}$**: –2760 (c = 1.0, $CHCl_3$). **Mp**: 165.1 – 166.8 °C.

Chiral HPLC: (Chiralpak OD-H, 4.6 x 250 mm; n-heptane/i-PrOH 97/3, 0.3 mL/min, 254 nm; t_R(major) = 31.7 min, t_R(minor) = 34.4 min, 90.4:9.6 *er*.

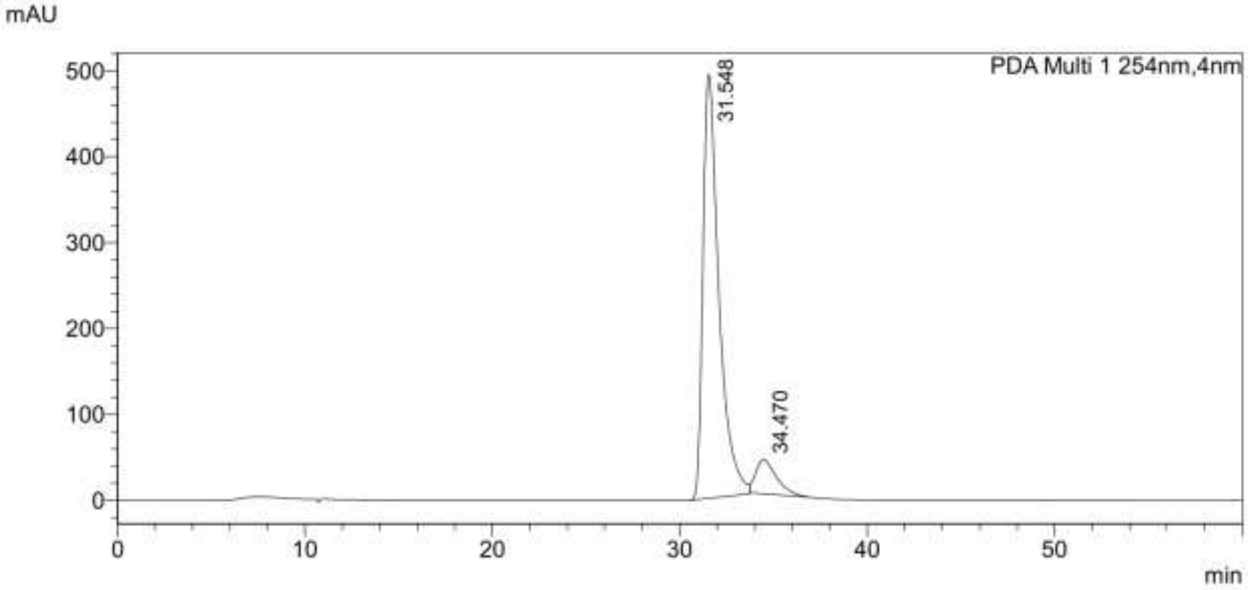

<Peak Table>

PDA Ch1 254nm

Peak#	Ret. Time	Area	Height	Area%
1	31.548	29232880	493275	90.366
2	34.470	3116563	39977	9.634
Total		32349443	533252	100.000

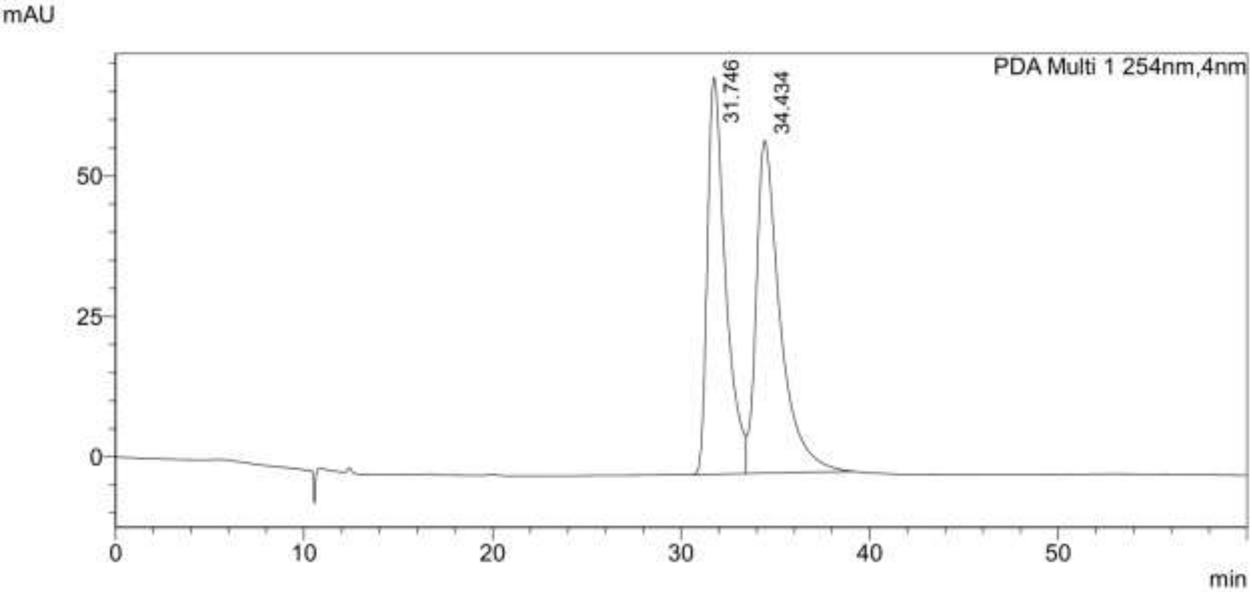

<Peak Table>

PDA Ch1 254nm

Peak#	Ret. Time	Area	Height	Area%
1	31.746	4730879	70552	47.571
2	34.434	5214007	59158	52.429
Total		9944887	129709	100.000

Proof-of-concept double C–H arylation:

Substrate synthesis for double C–H arylation:

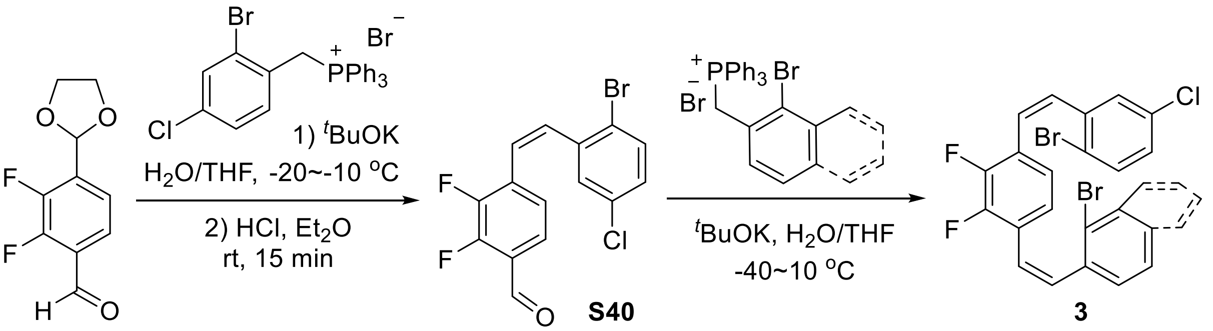

(Z)-4-(2-bromo-5-chlorostyryl)-2,3-difluorobenzaldehyde (S40): synthesized according to the **GP4**, wittig reaction and deprotection (2 steps).[12]

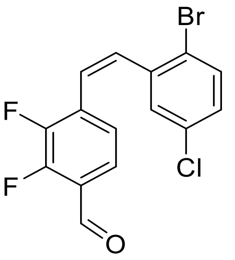

Yield: 78.6% (2 steps, 700 mg, *Z/E* 9:1). Analysis for *Z*-(**S39**): Cololess film. ***R*f** 0.20 (cyclohexane/EtOAc 10/1); **^{1}H NMR** (500 MHz, Chloroform-*d*): δ 10.29 (s, 1H), 7.55 (d, *J* = 8.6 Hz, 1H), 7.39 (ddd, *J* = 8.1, 6.1, 1.7 Hz, 1H), 7.13 (dd, *J* = 8.6, 2.6 Hz, 1H), 7.04 (d, *J* = 2.6 Hz, 1H), 6.94 – 6.83 (m, 2H), 6.79 (dd, *J* = 12.1, 1.4 Hz, 1H). **^{13}C NMR** (126 MHz, Chloroform-*d*) δ 185.8 (dd, $^{3}J_{C\text{-F}}$ = 6.1 Hz, $^{4}J_{C\text{-F}}$ = 3.0 Hz), 153.0 (dd, $^{1}J_{C\text{-F}}$ = 261.4 Hz, $^{2}J_{C\text{-F}}$ = 13.6 Hz), 148.5 (dd, $^{1}J_{C\text{-F}}$ = 253.4 Hz, $^{2}J_{C\text{-F}}$ = 12.3 Hz), 138.4, 134.3, 133.9 (d, $^{4}J_{C\text{-F}}$ = 1.9 Hz), 133.5, 132.2 (d, $^{2}J_{C\text{-F}}$ = 10.4 Hz), 130.1, 129.9, 125.0 (dd, $^{3}J_{C\text{-F}}$ = 4.3 Hz, $^{4}J_{C\text{-F}}$ = 2.0 Hz), 124.8 (d, $^{3}J_{C\text{-F}}$ = 5.5 Hz), 123.2 (t, $^{4}J_{C\text{-F}}$ = 3.1 Hz), 122.5 (dd, $^{3}J_{C\text{-F}}$ = 4.2 Hz, $^{4}J_{C\text{-F}}$ = 1.4 Hz), 121.7. **^{19}F NMR** (376 MHz, Chloroform-*d*) δ -138.9 (d, *J* = 20.1 Hz), -146.4 (d, *J* = 20.2 Hz). **HRMS** (APPI/LTQ-Orbitrap): calcd. for $[C_{15}H_{8}BrClF_{2}O]^{+}$, $[M]^{+}$: 355.9410; found: 355.9442. **IR (ATR):** $\tilde{\nu}$ = 2857, 2362, 1690, 1573, 1451, 1255, 1095, 1003, 904, 838, 767, 629 cm^{-1}.

1-((Z)-2-bromo-3-methylstyryl)-4-((Z)-2-bromo-5-chlorostyryl)-2,3-difluorobenzene (3y): synthesized according to the **GP4**.[12]

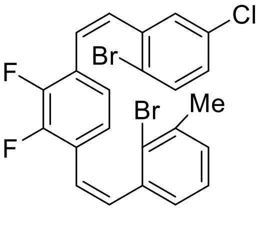

Yield: 47.7% (350 mg). White solid. ***R*f** 0.15 (cyclohexane); **^{1}H NMR** (500 MHz, Chloroform-*d*): δ 7.47 (d, *J* = 9.2 Hz, 1H), 7.13 – 7.01 (m, 3H), 6.99 (t, *J* = 7.6 Hz, 1H), 6.91 (dd, *J* = 7.7, 1.7 Hz, 1H), 6.82 (d, *J* = 12.0 Hz, 1H), 6.76 – 6.60 (m, 3H), 6.51 (dd, *J* = 8.5, 5.9 Hz, 1H), 6.44

(dd, J = 8.5, 5.9 Hz, 1H), 2.42 (s, 3H). **^{13}C NMR** (126 MHz, Chloroform-*d*) δ 148.8 (dd, $^{1}J_{C\text{-F}}$ = 253.1 Hz, $^{2}J_{C\text{-F}}$ = 15.4 Hz), 148.7 (dd, $^{1}J_{C\text{-F}}$ = 251.8 Hz, $^{2}J_{C\text{-F}}$ = 14.7 Hz), 138.9, 137.7, 134.0, 133.9 (d, $^{4}J_{C\text{-F}}$ = 1.5 Hz), 133.2, 131.3 (d, $^{4}J_{C\text{-F}}$ = 1.5 Hz), 130.3, 130.1, 129.3, 128.2, 127.0, 126.3, 126.0 (d, $^{2}J_{C\text{-F}}$ = 10.5 Hz), 124.5 (d, $^{2}J_{C\text{-F}}$ = 10.4 Hz), 124.4 (t, $^{3}J_{C\text{-F}}$ = 3.3 Hz), 123.9 (t, $^{3}J_{C\text{-F}}$ = 3.2 Hz), 123.7 (dd, $^{3}J_{C\text{-F}}$ = 4.3 Hz, $^{4}J_{C\text{-F}}$ = 2.5 Hz), 122.0 (t, $^{3}J_{C\text{-F}}$ = 3.2 Hz), 121.8, 23.8. **^{19}F NMR** (565 MHz, Chloroform-*d*) δ -140.4 (dd, J = 20.2, 6.3 Hz), -140.5 (dd, J = 20.2, 6.3 Hz). **HRMS** (APPI/LTQ-Orbitrap): calcd. for $[C_{23}H_{15}Br_2ClF_2]^+$, $[M]^+$: 521.9192; found: 521.9210. **IR (ATR):** $\tilde{\nu}$ = 2361, 1450, 1301, 1215, 1095, 1026, 902, 846, 777, 733, 624 cm^{-1}. **Mp**: 70.9 – 72.3 °C.

<u>**1-bromo-2-((*Z*)-4-((*Z*)-2-bromo-5-chlorostyryl)-2,3-difluorostyryl)naphthalene (3z):**</u> synthesized according to the **GP4**.[12]

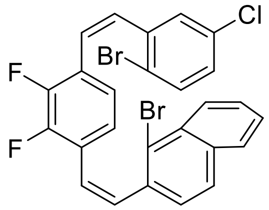

Yield: 40.6% (159 mg). Yellow solid. ***R*f** 0.20 (cyclohexane); **^{1}H NMR** (500 MHz, Chloroform-*d*): δ 8.33 (d, J = 8.2 Hz, 1H), 7.76 (d, J = 8.0 Hz, 1H), 7.59 (ddd, J = 9.7, 6.1, 1.8 Hz, 2H), 7.52 (ddd, J = 8.1, 7.0, 1.2 Hz, 1H), 7.43 (d, J = 8.5 Hz, 1H), 7.15 (d, J = 8.5 Hz, 1H), 7.09 – 6.95 (m, 3H), 6.80 (d, J = 12.0 Hz, 1H), 6.72 (d, J = 12.1 Hz, 1H), 6.67 (d, J = 12.1 Hz, 1H), 6.53 – 6.46 (m, 1H), 6.43 – 6.34 (m, 1H). **^{13}C NMR** (126 MHz, Chloroform-*d*) δ 148.9 (dd, $^{1}J_{C\text{-F}}$ = 252.1 Hz, $^{2}J_{C\text{-F}}$ = 14.8 Hz), 148.7 (dd, $^{1}J_{C\text{-F}}$ = 252.2 Hz, $^{2}J_{C\text{-F}}$ = 14.8 Hz), 138.8, 135.4, 134.0, 134.0, 133.9, 133.1, 132.6, 131.4, 130.2, 129.3, 128.3, 127.7, 127.7, 127.6, 127.4, 127.0, 126.0 (dd, $^{2}J_{C\text{-F}}$ = 8.3 Hz, $^{3}J_{C\text{-F}}$ 2.9 Hz), 124.8 (dd, $^{2}J_{C\text{-F}}$ = 6.5 Hz, $^{3}J_{C\text{-F}}$ 4.8 Hz), 124.7 (t, $^{3}J_{C\text{-F}}$ = 3.2 Hz), 124.1, 123.9 (d, $^{3}J_{C\text{-F}}$ = 3.1 Hz), 123.8 (d, $^{3}J_{C\text{-F}}$ = 3.1 Hz), 122.6 (t, $^{3}J_{C\text{-F}}$ = 3.3 Hz), 121.8. **^{19}F NMR** (376 MHz, Chloroform-*d*) δ -140.3. **HRMS** (APPI/LTQ-Orbitrap): calcd. for $[C_{26}H_{15}Br_2ClF_2]^+$, $[M]^+$: 557.9201; found: 557.9197. **IR (ATR):** $\tilde{\nu}$ = 3729, 2361, 1456, 1465, 1301, 1029, 807, 773 cm^{-1}. **Mp**: 126.5 – 128.5 °C.

Double C–H arylation:

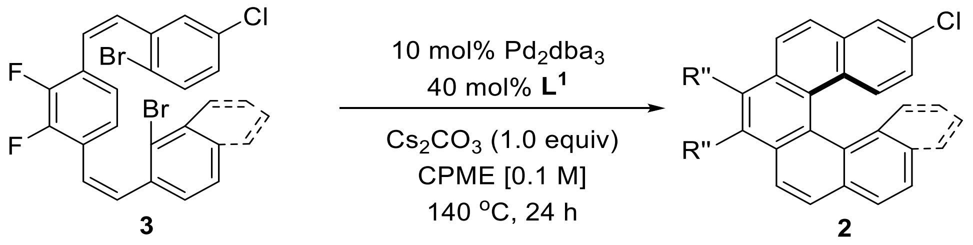

To an oven-dried 5 mL microwave vial, under ambient air, was added (*Z*)-2-bromostyrene (0.1 mmol, 1 equiv) (**3**) and (*R*)-**L^{1}** (25.8 mg, 40 µmol, 40 mol%). The vial was sealed with a septum and put under vacuum, followed by flushing with Ar gas 3 times. The vial was then transferred to a glovebox where Pd_2dba_3 (9.16 mg, 10 µmol, 10 mol%), dry and ground Cs_2CO_3 (32.9 mg, 0.1 mmol, 1.0 equiv) was added. Cyclopentylmethylether (1.0 mL, 0.1 M) was added to the

mixture before the vial was sealed and taken out of the glovebox. The reaction mixture was heated at 140 ^{o}C for 24 hours. The reaction mixture was then filtered through a plug of silica gel (eluted with EtOAc) and concentrated under reduced pressure. The resulting crude product was purified by column chromatography on silica gel.

8-chloro-3,4-difluoro-11-methyldibenzo[c,g]phenanthrene (2y):

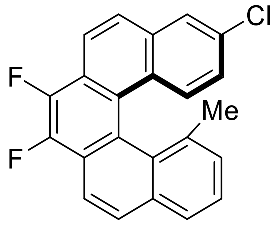

Yield: 30% (11 mg). light yellow foam. $\boldsymbol{R_f}$ 0.30 (cyclohexane/EtOAc 50/1); **^{1}H NMR** (500 MHz, Chloroform-*d*): δ 8.22 (d, *J* = 8.8 Hz, 1H), 8.10 (d, *J* = 8.6 Hz, 1H), 8.00 (d, *J* = 8.6 Hz, 1H), 7.95 – 7.88 (m, 3H), 7.72 (d, *J* = 9.1 Hz, 1H), 7.59 (t, *J* = 7.6 Hz, 1H), 7.25 (d, *J* = 7.2 Hz, 1H), 7.10 (dd, *J* = 9.2, 2.2 Hz, 1H), 1.54 (s, 3H). **^{13}C NMR** (126 MHz, Chloroform-*d*) δ 145.1 (dd, $^{1}J_{C\text{-F}}$ = 250.5 Hz, $^{2}J_{C\text{-F}}$ = 11.8 Hz), 145.0 (dd, $^{1}J_{C\text{-F}}$ = 250.7 Hz, $^{2}J_{C\text{-F}}$ = 11.8 Hz), 135.8, 133.1, 132.3, 131.0, 129.8, 129.6 (d, $^{4}J_{C\text{-F}}$ = 1.6 Hz), 127.5 (d, $^{4}J_{C\text{-F}}$ = 1.8 Hz), 127.3, 127.2, 127.1, 126.5, 125.9, 125.6 (d, $^{4}J_{C\text{-F}}$ = 2.5 Hz), 124.5 (d, $^{2}J_{C\text{-F}}$ = 14.1 Hz), 122.2 (d, $^{4}J_{C\text{-F}}$ = 2.9 Hz), 121.6 (d, $^{2}J_{C\text{-F}}$ = 13.2 Hz), 119.3 (d, $^{3}J_{C\text{-F}}$ = 5.9 Hz), 119.2 (d, $^{3}J_{C\text{-F}}$ = 6.2 Hz), 117.3 (d, $^{3}J_{C\text{-F}}$ = 5.9 Hz), 117.2 (d, $^{3}J_{C\text{-F}}$ = 6.4 Hz), 23.5. **^{19}F NMR** (376 MHz, Chloroform-*d*) δ -151.0 (d, *J* = 18.4 Hz), -151.9 (d, *J* = 18.2 Hz). **HRMS** (APPI/LTQ-Orbitrap): calcd. for $[C_{23}H_{13}ClF_2]^+$, $[M]^+$: 362.0674; found: 362.0668. **IR (ATR):** $\tilde{\nu}$ = 2925, 2361, 1646, 1408, 1281, 1051, 869, 794, 659 cm^{-1}. **$[\alpha]_D^{20}$**: –2049 (c = 0.1, $CHCl_3$).

Chiral HPLC: (Chiralpak OJ-H, 4.6 x 250 mm; n-heptane/i-PrOH 97/3, 0.5 mL/min, 254 nm; t_R(minor) = 10.4min, t_R(major) = 14.1 min, 3.0:97.0 *er*.

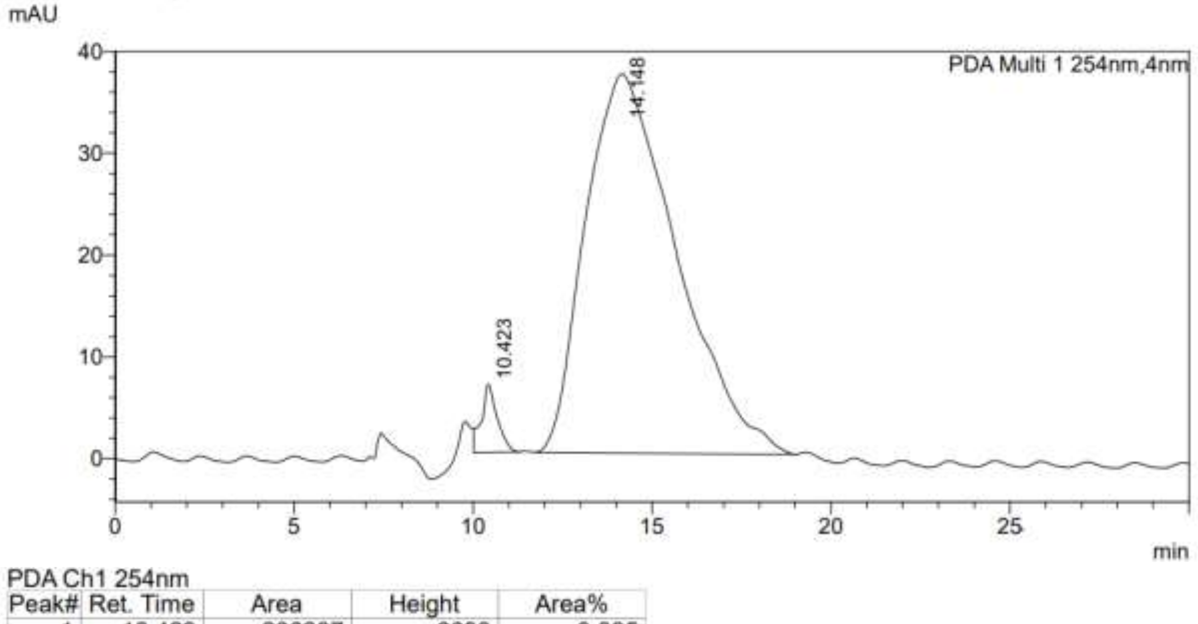

PDA Ch1 254nm

Peak#	Ret. Time	Area	Height	Area%
1	10.423	206287	6693	3.035
2	14.148	6589988	37239	96.965
Total		6796275	43931	100.000

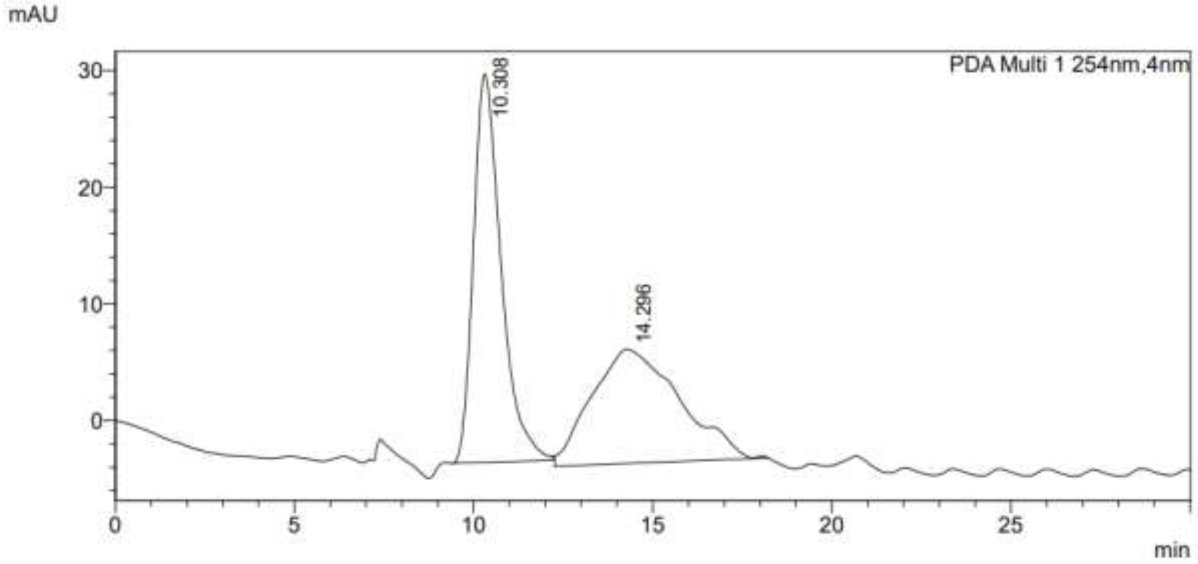

PDA Ch1 254nm

Peak#	Ret. Time	Area	Height	Area%
1	10.308	1791481	33289	51.782
2	14.296	1668156	9778	48.218
Total		3459638	43067	100.000

15-chloro-3,4-difluorohexahelicene (2z):

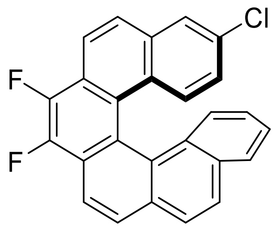

Yield: 28% (11 mg). Yellow foam. ***R*f** 0.40 (cyclohexane/EtOAc 50/1); **^{1}H NMR** (600 MHz, Chloroform-*d*): δ 8.30 (d, *J* = 8.4 Hz, 1H), 8.25 (d, *J* = 8.8 Hz, 1H), 8.09 (d, *J* = 8.4 Hz, 1H), 8.02 – 7.90 (m, 3H), 7.85 (d, *J* = 8.0 Hz, 1H), 7.82 (d, *J* = 2.2 Hz, 1H), 7.48 (d, *J* = 8.5 Hz, 1H), 7.44 (d, *J* = 9.2 Hz, 1H), 7.36 – 7.26 (m, 1H), 6.80 – 6.72 (m, 1H), 6.63 (dd, *J* = 9.0, 2.2 Hz, 1H). **^{13}C NMR** (151 MHz, Chloroform-*d*) δ 143.3 (dd, $^1J_{C\text{-F}}$ = 250.8 Hz, $^2J_{C\text{-F}}$ = 11.7 Hz), 143.0 (dd, $^1J_{C\text{-F}}$ = 250.7 Hz, $^2J_{C\text{-F}}$ = 11.6 Hz), 132.5, 132.2, 131.8, 131.4, 129.5, 129.3, 128.9 (d, $^4J_{C\text{-F}}$ = 1.6 Hz), 128.7, 128.2 (d, $^4J_{C\text{-F}}$ = 1.6 Hz), 128.0, 127.9, 127.8, 127.7, 126.8, 126.4, 126.2, 125.9, 125.4, 125.3 (t, $^4J_{C\text{-F}}$ = 2.4 Hz), 124.7 (d, $^2J_{C\text{-F}}$ = 14.1 Hz), 122.7 (d, $^2J_{C\text{-F}}$ = 14.1 Hz), 121.4 (d, $^4J_{C\text{-F}}$ = 2.9 Hz), 119.2 (dd, $^3J_{C\text{-F}}$ = 7.3 Hz, $^4J_{C\text{-F}}$ = 5.2 Hz), 118.81 (t, $^3J_{C\text{-F}}$ = 6.5 Hz). **^{19}F NMR** (565 MHz, Chloroform-*d*) δ -151.0 (d, *J* = 17.8 Hz), -151.6 (d, *J* = 17.9 Hz). **HRMS** (APPI/LTQ-Orbitrap): calcd. for $[C_{26}H_{13}ClF_2]^+$, $[M]^+$: 398.0677; found: 398.0668. **IR (ATR):** $\widetilde{\nu}$ = 2921, 2361, 1651, 1401, 1282, 1091, 875, 792, 652 cm^{-1}. **$[\alpha]_D^{20}$**: –1996 (c = 0.08, $CHCl_3$).

Chiral HPLC: (Chiralpak IA, 4.6 x 250 mm; n-heptane/i-PrOH 97/3, 0.5 mL/min, 254 nm; t_R(minor) = 9.5 min, t_R(major) = 10.2 min, 7.6:92.4 *er*.

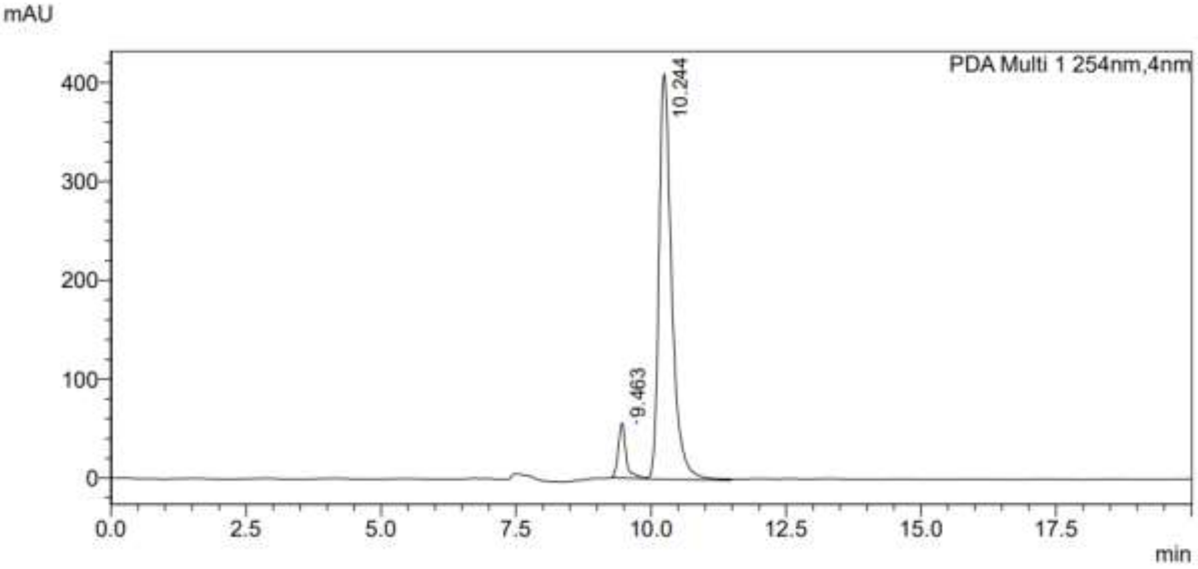

PDA Ch1 254nm

Peak#	Ret. Time	Area	Height	Area%
1	9.463	554566	55637	7.612
2	10.244	6731244	409527	92.388
Total		7285810	465164	100.000

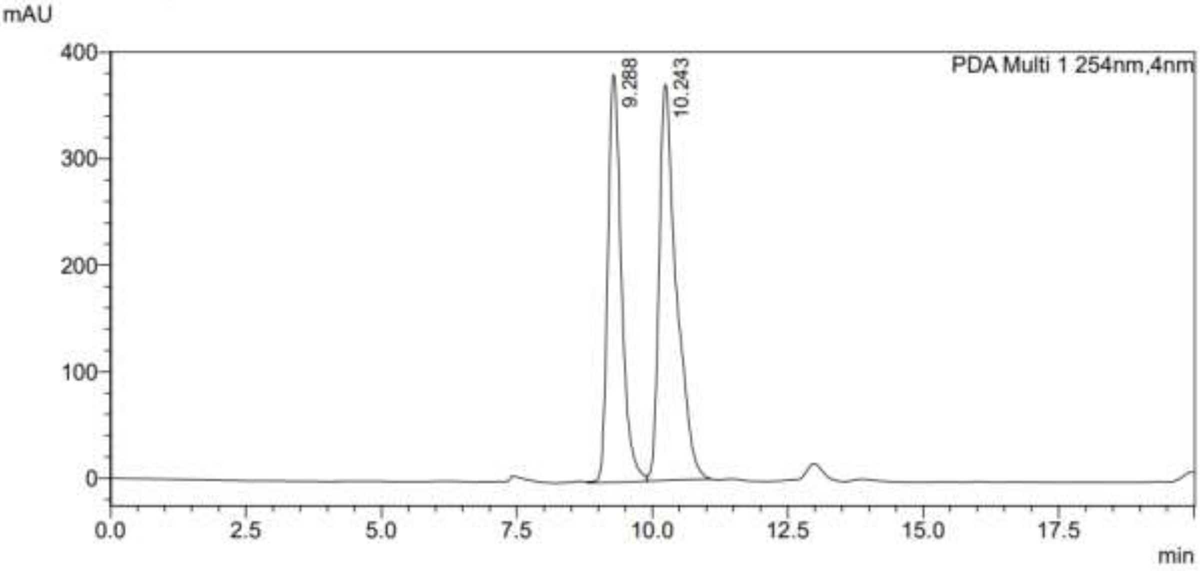

PDA Ch1 254nm

Peak#	Ret. Time	Area	Height	Area%
1	9.288	6714332	382804	44.894
2	10.243	8241564	372038	55.106
Total		14955896	754842	100.000

The second C–H arylation from naphthyl bromide *vs* phenyl bromide:

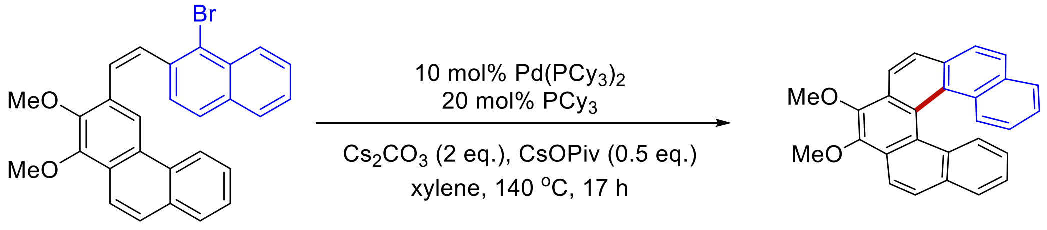

1s **2s** 76%

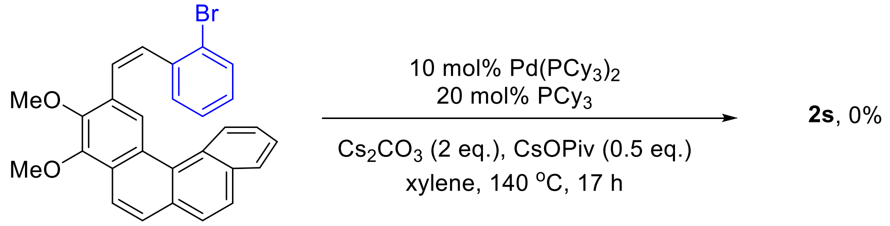

1s'

To an oven-dried 5 mL microwave vial under ambient air, was added (*Z*)-2-bromostyrene (0.05 mmol, 1 equiv) (**1s** or **1s'**). The vial was sealed with a septum and put under vacuum, followed by flushing 3 times with Ar gas. The vial was then transferred to a glovebox where Pd(PCy)$_3$ (3.34 mg, 5 µmol, 10 mol%), and PCy$_3$ (2.8 mg, 10 µmol, 20 mol%), dry and ground CsOPiv (3.51 mg, 15 µmol, 0.3 equiv), Cs_2CO_3 (32.6 mg, 0.1 mmol, 2.0 equiv) was added. *p*-Xylene (0.5 mL, 0.1 M) was added to the mixture before the vial was sealed and taken out of the glovebox. The reaction mixture was heated at 140 °C for 17 hours. The reaction mixture was then filtered through a plug of silica gel (eluted with EtOAc) and concentrated under reduced pressure. The yields of resulting crudes were determined by ^{1}H NMR with trichloroethylene as internal standard.

The crude ^{1}H NMR were shown as follows:

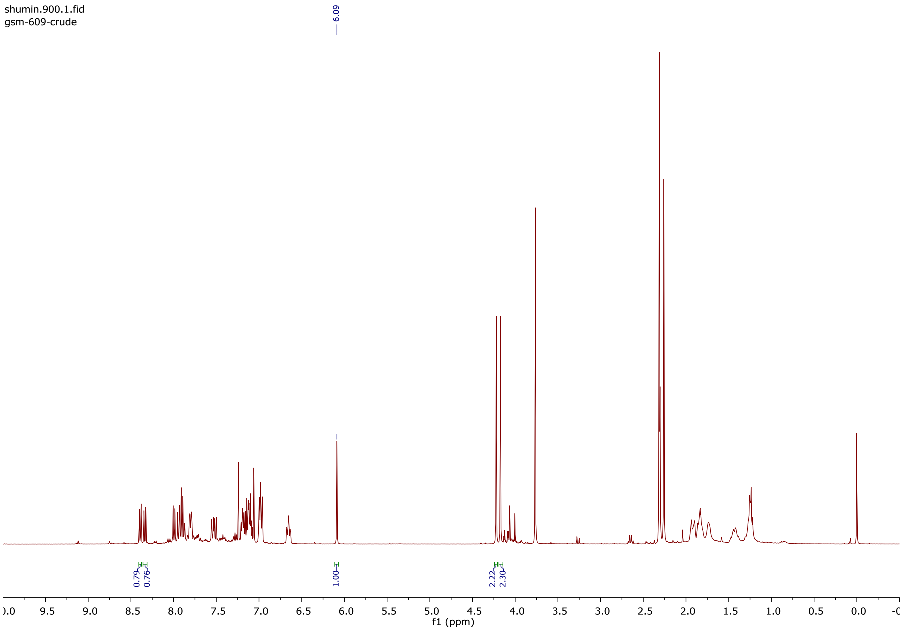

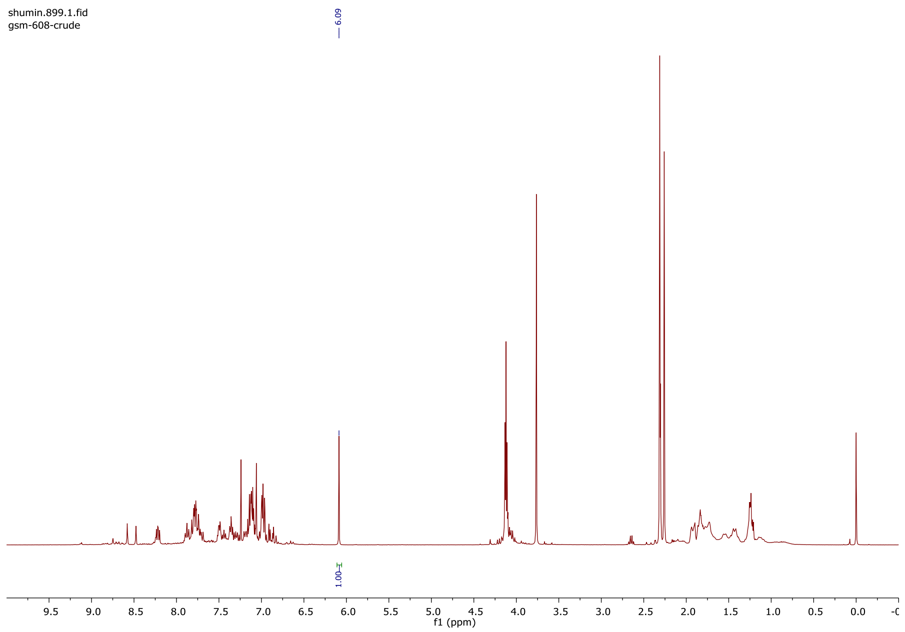

3,4-dimethoxybenzo[c]phenanthrene-2-carbaldehyde (S41): synthesized according to the **GP5** from **S30** (1.5 g, 7.72 mmol), 0.245 g (from 2.4 g crude *cis*-alkene) was engaged in the following step.[12]

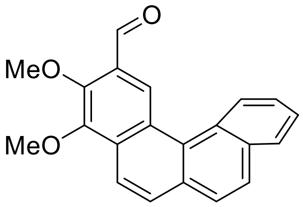

Yield: 78.3% (2 steps, 195 mg). Yellow foam. ***R*f** 0.20 (cyclohexane/EtOAc 10/1); **^{1}H NMR** (400 MHz, Chloroform-*d*): δ 10.62 (s, 1H), 9.38 (t, *J* = 0.9 Hz, 1H), 9.01 (dt, *J* = 8.6, 1.0 Hz, 1H), 8.25 (dd, *J* = 8.8, 0.8 Hz, 1H), 8.03 (dd, *J* = 7.9, 1.5 Hz, 1H), 7.97 – 7.89 (m, 2H), 7.82 (d, *J* = 8.5 Hz, 1H), 7.79 – 7.72 (m, 1H), 7.67 (ddd, *J* = 8.0, 6.9, 1.1 Hz, 1H), 4.19 (s, 3H), 4.13 (s, 3H). **^{13}C NMR** (126 MHz, Chloroform-*d*) δ 190.5, 150.4, 148.0, 134.0, 133.5, 130.9, 130.5, 130.1, 129.2, 128.8, 128.6, 128.3, 128.1, 127.3, 127.3, 126.7, 126.7, 125.6, 120.5, 62.5, 61.7. **HRMS** (ESI-MS): calcd. for $[C_{21}H_{16}NaO_3]^+$, $[M]^+$: 339.0992; found: 339.0993. **IR (ATR):** $\tilde{\nu}$ = 2937, 2856, 2361, 1687, 1594, 1460, 1391, 1263, 1066, 1018, 842, 768 cm^{-1}.

(Z)-2-(2-bromostyryl)-3,4-dimethoxybenzo[c]phenanthrene (1s'): synthesized according to the **GP5** from S40 (0.12 g, 0.379 mmol).[12]

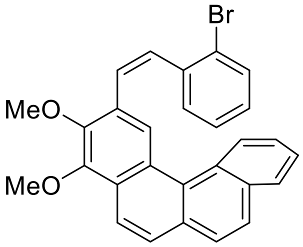

Yield: 97.2% (173 mg). Yellow solid. ***R*f** 0.20 (cyclohexane); analysis for *Z*-(**S39**): **^{1}H NMR** (400 MHz, Chloroform-*d*): δ 8.49 (s, 1H), 8.22 (dd, *J* = 8.8, 0.8 Hz, 1H), 7.90 (dd, *J* = 8.0, 1.4 Hz, 1H), 7.85 – 7.70 (m, 5H), 7.51 (ddd, *J* = 8.0, 6.9, 1.1 Hz, 1H), 7.37 (dd, *J* = 7.6, 2.0 Hz, 1H), 7.20 (ddd, *J* = 8.5, 6.9, 1.5 Hz, 1H), 7.16 – 7.03 (m, 3H), 6.90 (d, J = 11.9 Hz, 1H), 4.13 (s, 3H), 4.12 (s, 3H). **^{13}C NMR** (101 MHz, Chloroform-*d*) δ 148.0, 147.6, 138.2, 133.5, 133.2, 131.6, 131.2, 130.7, 130.5, 130.1, 129.1, 128.9, 128.4, 128.3, 127.5, 127.5, 127.5, 127.4, 127.4, 127.3, 126.9, 126.3, 125.9, 125.5, 124.7, 120.7, 61.7, 61.3. **HRMS** (ESI-MS): calcd. for $[C_{28}H_{21}BrNaO_2]^+$, $[M]^+$: 491.0617; found: 491.0609. **IR (ATR):** $\tilde{\nu}$ = 2934, 2362, 1601, 1459, 1415, 1360, 1237, 1065, 921, 838, 762 cm^{-1}. **Mp**: 116.3 – 118.3 °C.

Racemization Kinetics

To an oven-dried 2-mL microwave tube, approximately 1 mg of **2d** or **2q** was added and dissolved in 1 mL degassed *m*-xylene. The sealed vial was covered by aluminum foil, then heated at the specified temperature.

The change in enantiomeric excess (*ee*) over time was obtained by chiral HPLC (Chiralpak IA, 4.6 x 250 mm; n-heptane/i-PrOH 97/3, 0.3~0.5 mL/min, 293 nm) and plotted as ln(ee_0/*ee*) versus time, where ee_0 is the initial enantiomeric excess at t = 0. The gradient of the obtained graph corresponds to the rate constant of racemization, $k_{racemization}$, at the specific temperature.

The rate constant for enantiomerisation, $k_{enantiomerization}$, is related to the racemization rate constant according to the following equation:

$$k_{enantiomerization} = \frac{k_{racemization}}{2}$$

The half-life of racemization at that temperature can be calculated as:

$$t_{1/2\ racemization} = \frac{\ln 2}{k_{racemization}}$$

The barrier to rotation, $\Delta G^{\ddagger}_{enantiomerization}$, was subsequently calculated using the following form of the Eyring equation:

$$\Delta G^{\ddagger}_{enantiomerization} = RT \cdot \mathrm{In} \frac{k_B T}{\mathrm{h} k_{enantiomerization}}$$

Where: R = Gas constant = 8.314462 $J{\cdot}K^{-1}{\cdot}mol^{-1}$, h = Planck constant = $6.62608{\times}10^{-34}$ J·s, k_B = Boltzmann constant = $1.38066{\times}10^{-23}$ $J{\cdot}K^{-1}$, and T = temperature in Kelvin.

7,12-dimethoxy-1,3-dimethylbenzo[*c*]phenanthrene (2d):

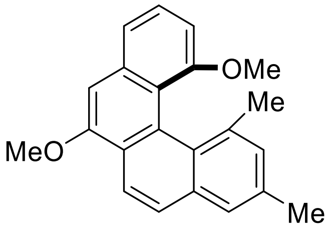

Solvent: *m*-xylene, **Temperature:** 120 °C

Chiral HPLC: (Chiralpak IA, 4.6 x 250 mm; n-heptane/i-PrOH 97/3, 0.3 mL/min, 293 nm)

Time (*seconds*)	Enantiomeric Excess (*ee*)	In[ee_0/ee]
0	89.1	0
43200	85.8	0.038
86400	82.4	0.078
129600	80.3	0.104
172800	78.8	0.122
216000	76.4	0.153
259200	73.4	0.194

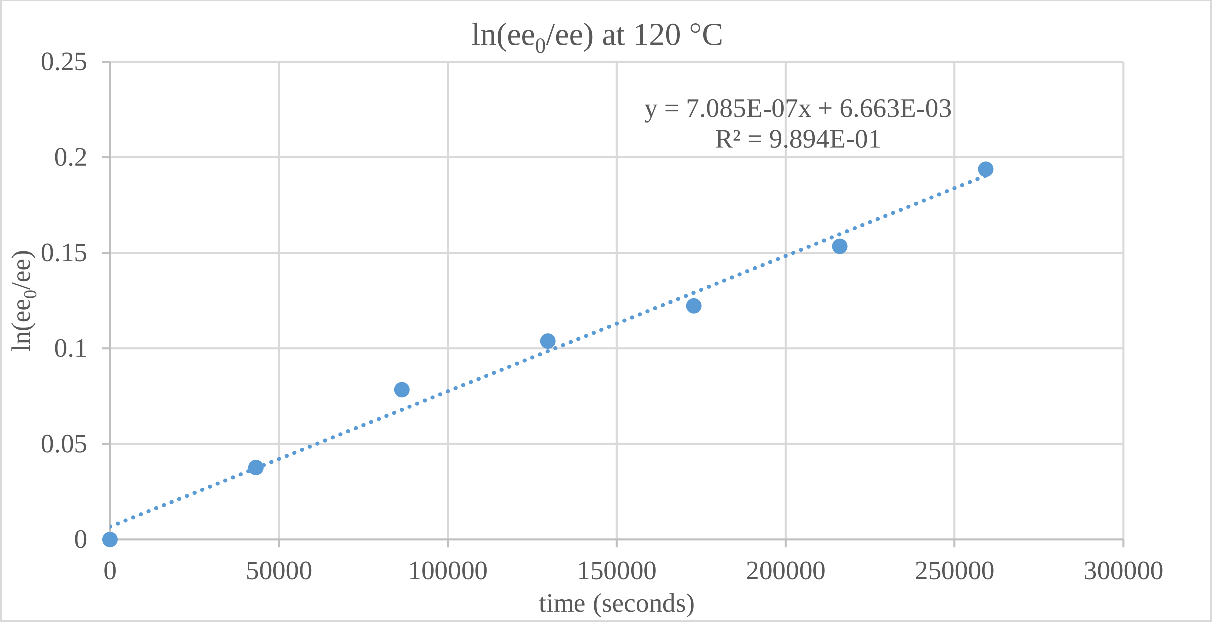

$k_{racemization}$ (120 °C) = 7.09×10^{-7} s^{-1}

$k_{enantiomerization}$ (120 °C) = 3.54×10^{-7} s^{-1}

$\Delta G^{\ddagger}_{enantiomerization}$ (120 °C) = 34.8 kcal·mol^{-1}

Solvent: *m*-xylene, **Temperature:** 140 °C

Chiral HPLC: (Chiralpak IA, 4.6 x 250 mm; n-heptane/i-PrOH 97/3, 0.3 mL/min, 293 nm)

Time (seconds)	Enantiomeric Excess (*ee*)	In[ee_0/ee]
0	89.1	0
14400	79.3	0.116
28800	74.2	0.183
43200	66.5	0.293
57600	59.0	0.412
72000	53.2	0.516
86400	49.1	0.595

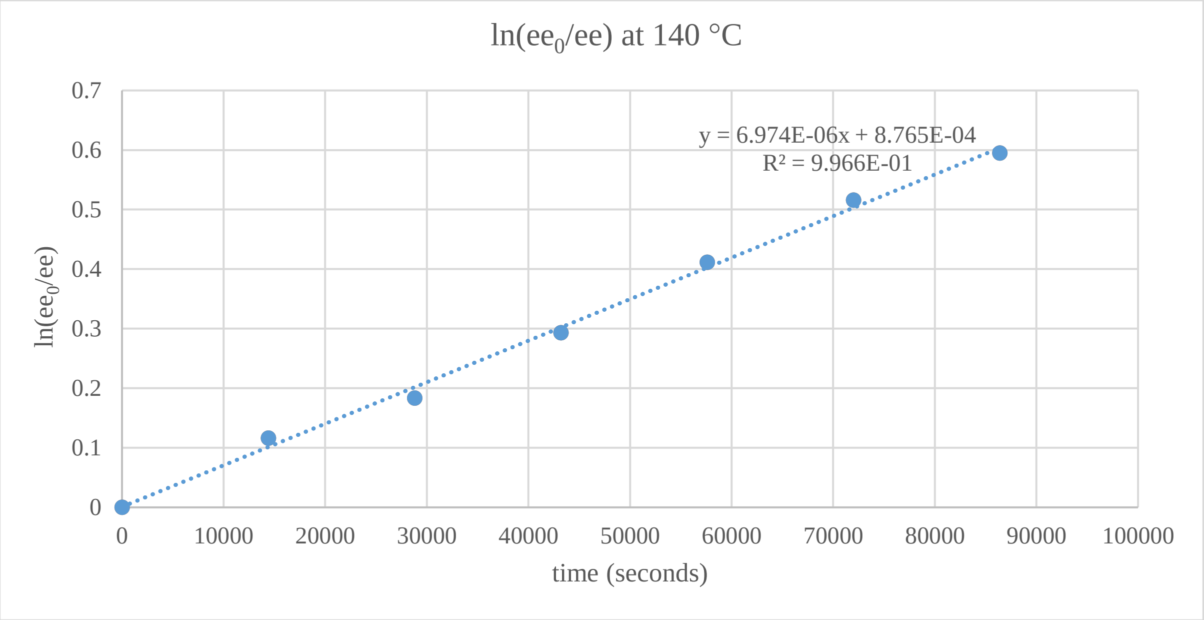

$k_{racemization}$ (140 °C) = 6.97×10^{-6} s^{-1}

$k_{enantiomerization}$ (140 °C) = 3.49×10^{-6} s^{-1}

$\Delta G^{\ddagger}_{enantiomerization}$ (140 °C) = 34.8 kcal·mol^{-1}

2,11-dimethoxydibenzo[*c*,*g*]phenanthrene (2q):

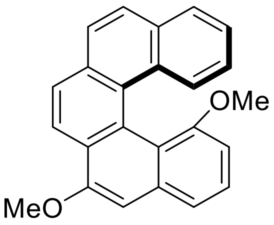

Solvent: *m*-xylene, **Temperature:** 120 °C

Chiral HPLC: (Chiralpak IA, 4.6 x 250 mm; n-heptane/i-PrOH 97/3, 0.5 mL/min, 293 nm)

Time (seconds)	Enantiomeric Excess (*ee*)	In[ee_0/ee]
0	86.1	0
43,200	72.0	0.179
86,400	68.1	0.234
129,600	58.9	0.379
172,800	52.4	0.497
216,000	46.5	0.616

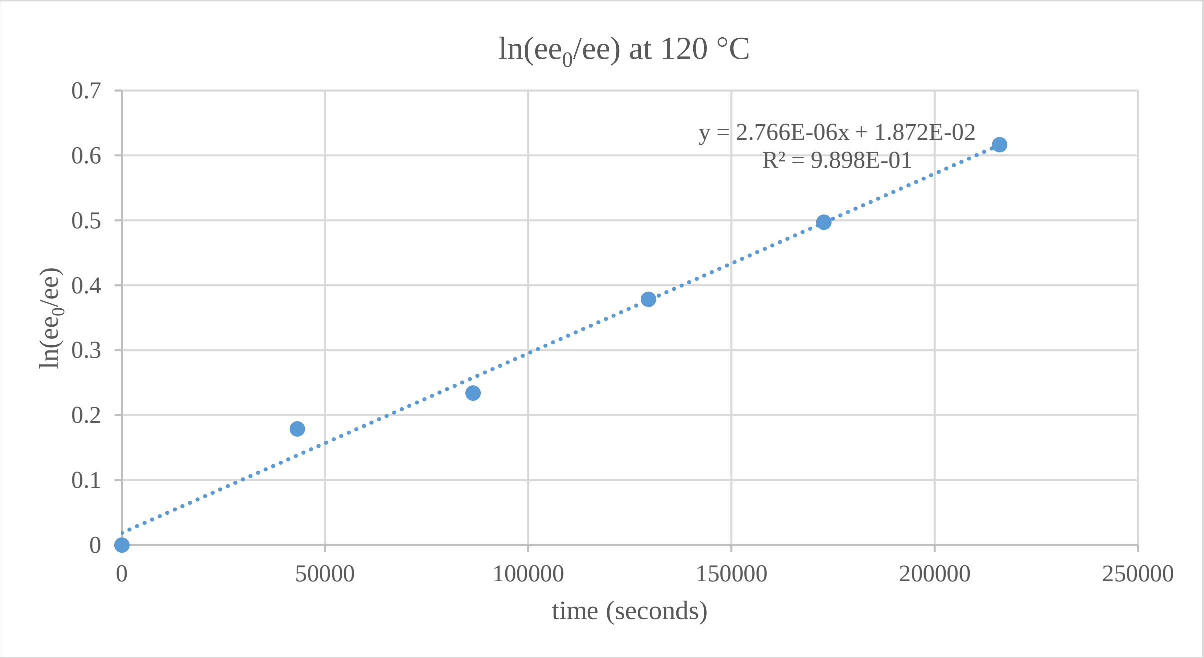

$k_{racemization}$ (120 °C) = 2.77×10^{-6} s^{-1}

$k_{enantiomerization}$ (120 °C) = 1.38×10^{-6} s^{-1}

$\Delta G^{\ddagger}_{enantiomerization}$ (120 °C) = 33.7 kcal·mol^{-1}

Solvent: *m*-xylene, **Temperature:** 140 °C

Chiral HPLC: (Chiralpak IA, 4.6 x 250 mm; n-heptane/i-PrOH 97/3, 0.5 mL/min, 293 nm)

Time (seconds)	Enantiomeric Excess (*ee*)	In[ee_0/ee]
0	86.1	0
43200	45.7	0.633
57600	33.6	0.940
72000	24.2	1.270
86400	15.7	1.699

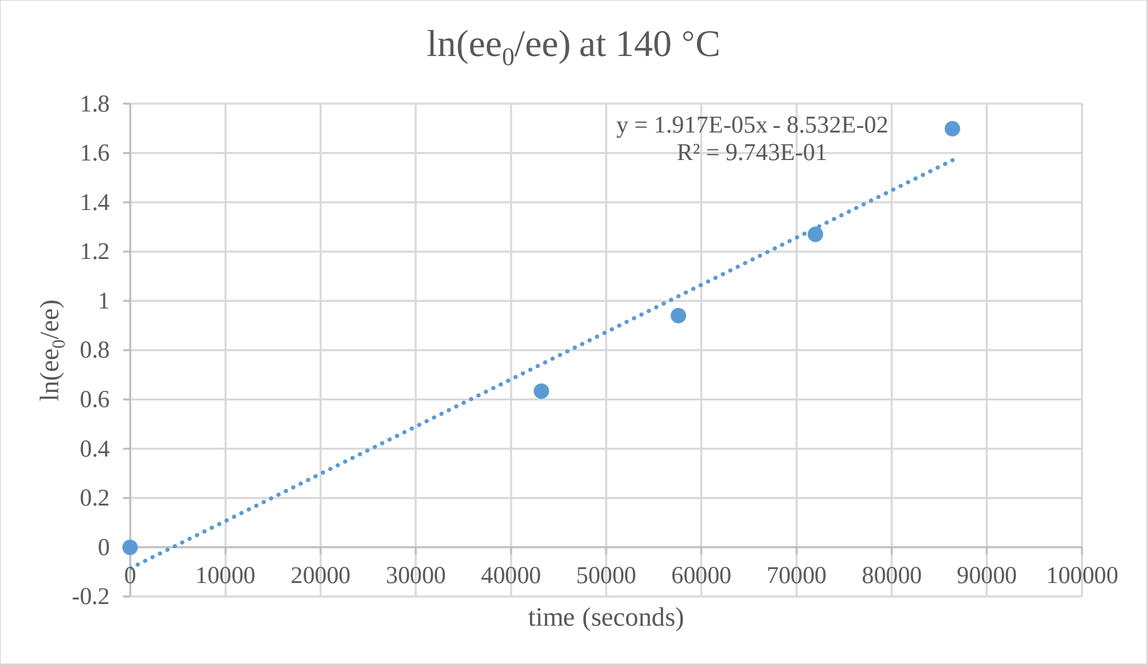

$k_{racemization}$ (140 °C) = 1.92×10^{-5} s^{-1}

$k_{enantiomerization}$ (140 °C) =9.59×10^{-6} s^{-1}

$\Delta G^{\ddagger}_{enantiomerization}$ (140 °C) = 33.9 kcal·mol^{-1}

X-ray Structures

X-ray structure of 2p (CCDC 2142962)

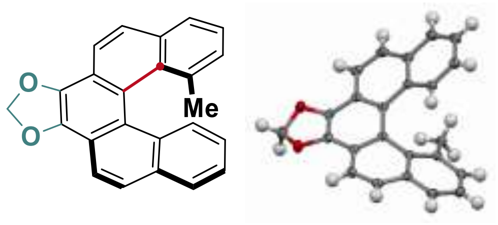

Formula	$C_{24}H_{16}O_2$
$D_{calc.}$/ g cm^{-3}	1.389
μ/mm^{-1}	0.690
Formula Weight	336.37
Colour	clear intense yellow
Shape	plate-shaped
Size/mm^3	0.37×0.23×0.05
T/K	139.99(10)
Crystal System	orthorhombic
Flack Parameter	-0.03(3)
Space Group	$P2_12_12_1$
a/Å	6.85871(3)
b/Å	12.62934(6)
c/Å	18.57324(8)
α/°	90
β/°	90
γ/°	90
V/Å^3	1608.832(12)
Z	4
Z'	1
Wavelength/Å	1.54184
Radiation type	Cu$K\alpha$
Θ_{min}/°	4.233
Θ_{max}/°	75.082
Measured Refl's.	57680
Indep't Refl's	3259
Refl's I≥2σ(I)	3238
R_{int}	0.0207
Parameters	237
Restraints	0
Largest Peak/e Å^{-3}	0.177
Deepest Hole/e Å^{-3}	-0.122
GooF	1.038
wR_2 (all data)	0.0645
wR_2	0.0644
R_1 (all data)	0.0245
R_1	0.0244

X-ray structure of 2q (CCDC 2142961)

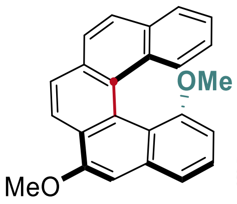

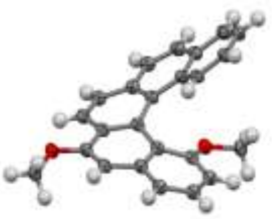

Formula	$C_{24}H_{18}O_2$
$D_{calc.}$/ g cm^{-3}	1.338
μ/mm^{-1}	0.661
Formula Weight	338.38
Colour	colourless
Shape	block-shaped
Size/mm^3	0.20×0.16×0.11
T/K	150
Crystal System	monoclinic
Flack Parameter	-0.6(6)
Hooft Parameter	0.08(8)
Space Group	$P2_1$
a/Å	10.1597(4)
b/Å	6.9772(2)
c/Å	11.9252(5)
α/°	90
β/°	96.441(3)
γ/°	90
V/Å^3	840.00(5)
Z	2
Z'	1
Wavelength/Å	1.54186
Radiation type	Cu K_α
Θ_{min}/°	3.730
Θ_{max}/°	70.097
Measured Refl's.	19833
Indep't Refl's	3035
Refl's I≥2 σ(I)	2689
R_{int}	0.0574
Parameters	238
Restraints	1
Largest Peak	0.227
Deepest Hole	-0.223
GooF	1.155
wR_2 (all data)	0.2516
wR_2	0.2302
R_1 (all data)	0.0871
R_1	0.0792

Computational Mechanism Study

Computational details

DFT calculations were performed with Gaussian 09 program.[17] For the geometry optimization, DFT calculations were carried out with Gaussian 09 program, with B3LYP functionals[18,19] and Grimme's D3 correction[20] with a mixed basis sets of SDD basis set[21] for Pd and Cs atoms and def2-SVP basis sets for the remaining atoms.[22] Transition states were realized by the presence of single negative frequency, and no imaginary frequencies were found for all optimized intermediates. Intrinsic reaction coordinate (IRC) calculations were carried out to confirm that the computed transition states properly connect the reactant and the product structures along the reaction trajectory. Electronic energies of optimized structures were further corrected by single point calculations with SDD basis sets for Pd and Cs atoms and def2-TZVP basis sets for other atoms. Solvation of toluene was considered by single point calculation with integral equation formalism PCM model[23] to evaluate solution phase electronic energies (E_{Sol}). Thermodynamic parameters including Gibbs free energies were obtained by frequency calculations, wherein thermochemistry correction energy (G – E) was acquired.

Final solution phase Gibbs free energies (G_{Sol}) were calculated as follows:

$$G_{Sol} = E_{Sol} + (G - E) \quad (1)$$

$$\Delta G_{Sol} = \Sigma G_{Sol} \text{ for products} - \Sigma G_{Sol} \text{ for reactants} \quad (2)$$

The noncovalent interactions (NCI) were determined using Multiwfn software.[24] Figures of three-dimensional molecular structures were prepared using VMD and CYLview.[25,26]

The global energy profile for the reaction with L[1] employed as ligand

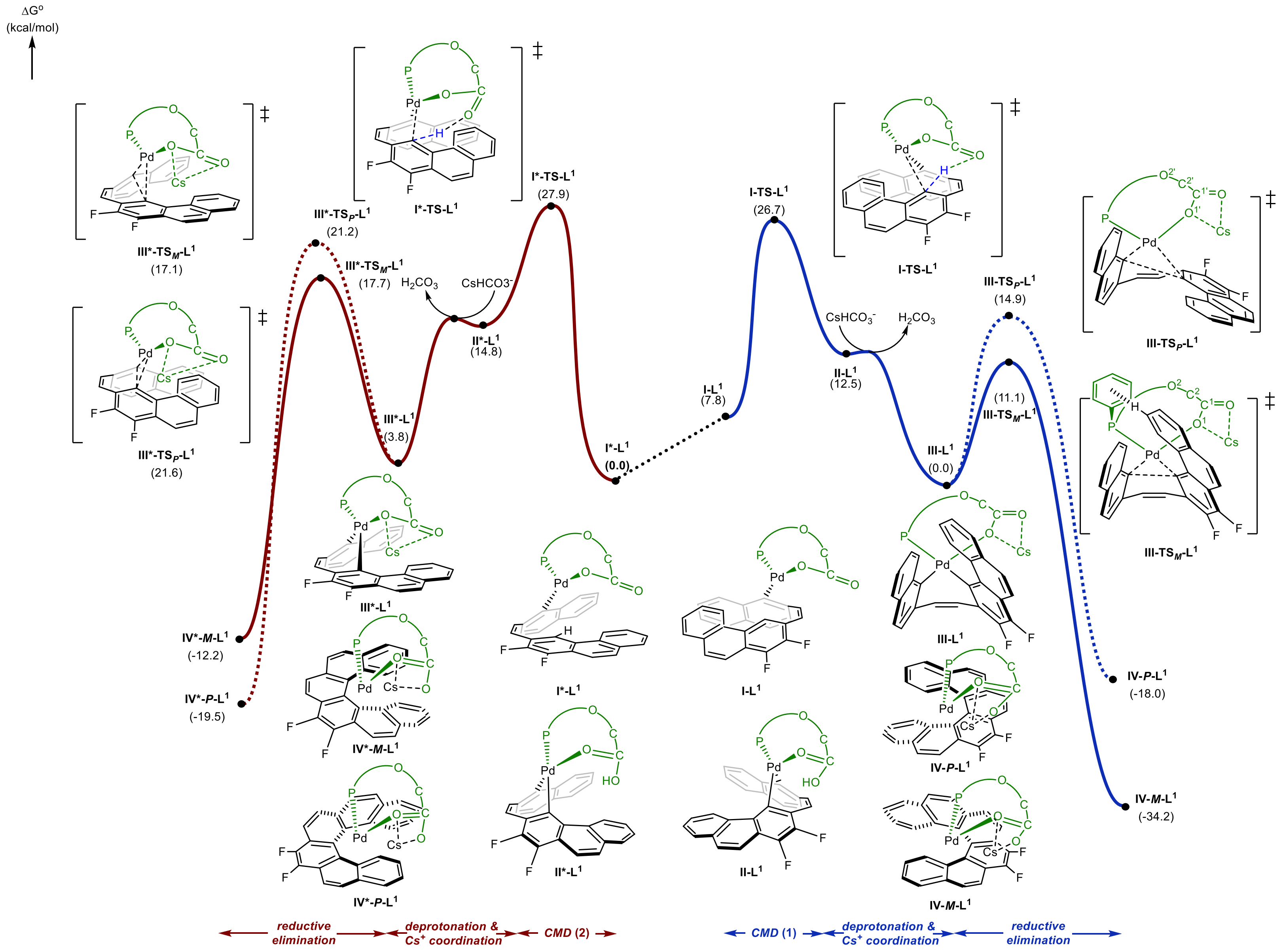

Fig. S2. Energy diagram of the reaction pathway with **L[1]**.

The global energy profile for the reaction with L^2 employed as ligand

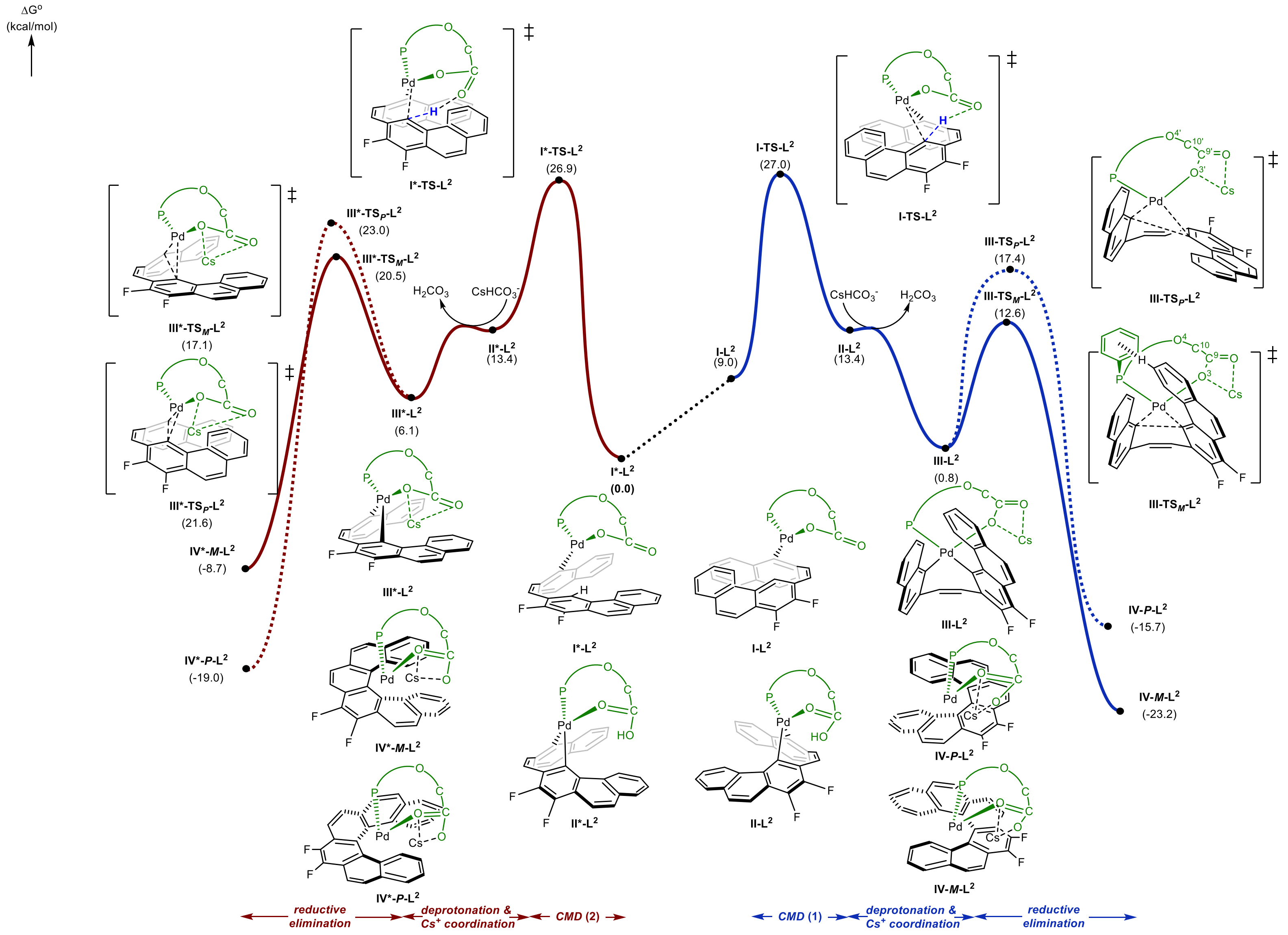

Fig. S3. Energy diagram of the reaction pathway with **L^2**.

C–H activation

To analyze the origin of the energetic difference between two TSs in C–H activation steps (Fig. S. 3.), the optimized geometries of **I-TS-L^{n}** and **I*-TS-L^{n}** are shown in Fig. S. 4. By comparing the atomic distances near the reaction sites, different structural features between transition states were identified. The atomic distances of Pd···$C^{1(')}$ are 2.25 Å for **I-TS-L^{1}** and 2.36 Å for **I*-TS-L^{1}**. With **L^{2}**, the atomic distance of Pd–$C^{2(')}$ was found to be 2.27 Å for **I-TS-L^{2}** and 2.37 Å for **I*-TS-L^{2}**. Indeed, the space-filling models suggest that in **I*-TS-L^{1}**, there might be a steric factor from the phenyl group of phenyl-naphthyl moiety of the ligand that hinders the phenanthrene ring of the substrate from getting closer to Pd during CMD processes, hence resulting in a longer Pd···C^{1} distance in **I*-TS-L^{1}** than Pd···$C^{1'}$ distance in **I-TS-L^{1}**. Therefore, the short distance of Pd···$C^{1'}$ in **I-TS-L^{1}** might translate into a more stable bond formation during C–H activation step, rendering **I-TS-L^{1}** lower in energy than **I*-TS-L^{1}**. However, this stabilization factor observed in **I-TS-L^{1}** is no longer the most crucial component when **L^{2}** is employed as ligand. Indeed, the geometry of **I-TS-L^{2}** shows two short H···H contacts between C–H of the phenanthrene ring of the substrate and hydrogen of the *t*Bu substituent in the aryl ring of **L^{2}**, with H···H = 2.18 Å, much shorter than the van der Waals distance, 2.4 Å. In sharp contrast, **I-TS-L^{1}** contains no such short H···H contacts, hence resulting in a bigger ΔΔG‡ value with **L^{1}** than that with **L^{2}**.

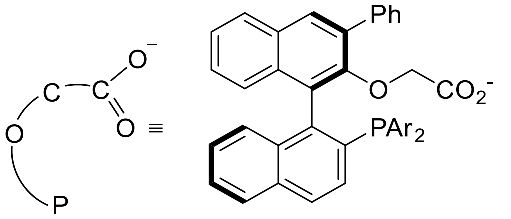

L^{1}: Ar = 3,5-$Me_2C_6H_3$
L^{2}: Ar = 3,5-*t*$Bu_2C_6H_3$

ΔΔG‡ (kcal mol^{-1})	
**L^{1}**	1.2
**L^{2}**	-0.1

Fig. S4. ΔΔG‡ values of transition states for C–H activation.

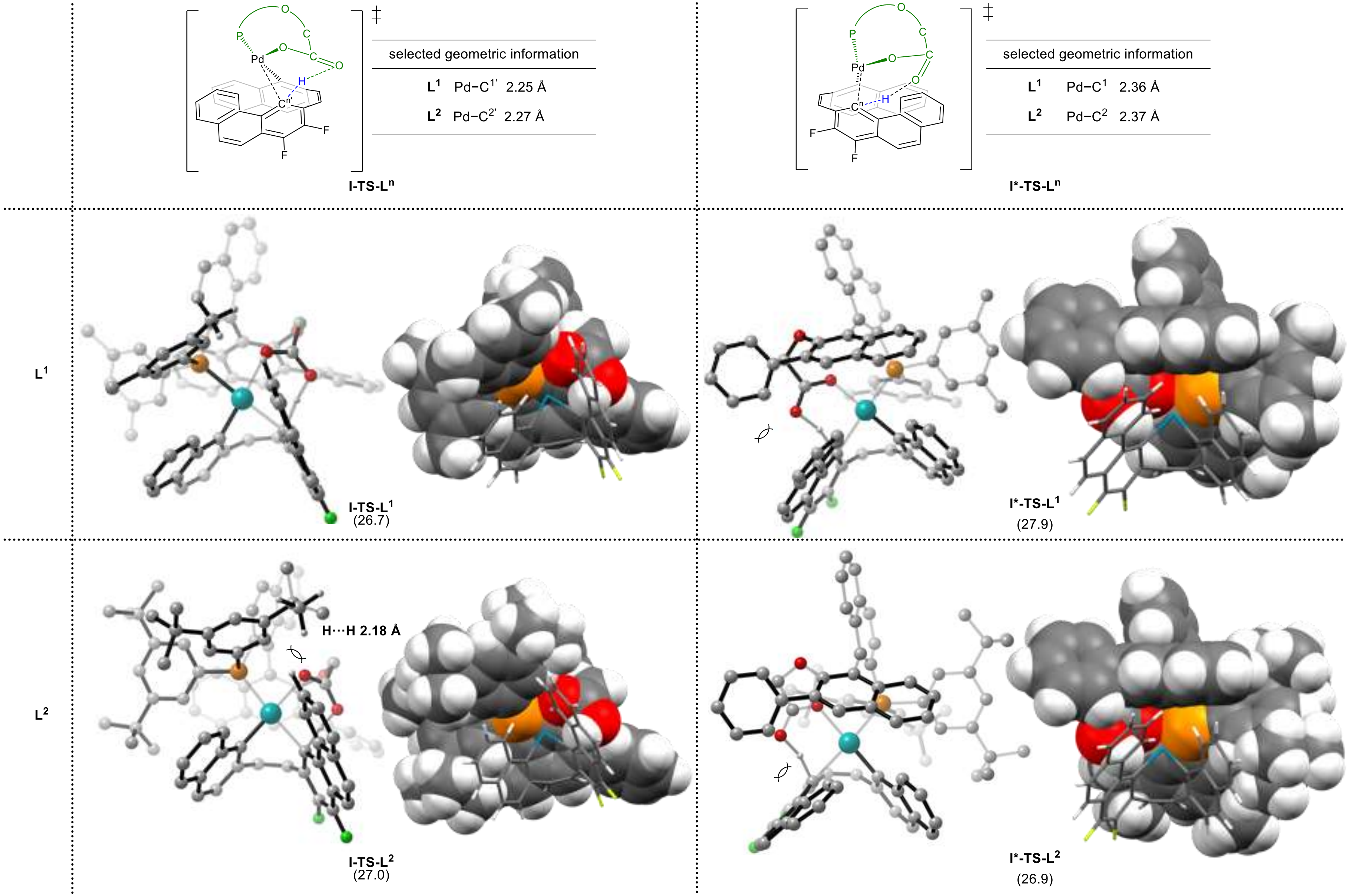

Fig. S5. Optimized geometries of transition states for C–H activation.

Reductive elimination with L^1 employed as ligand

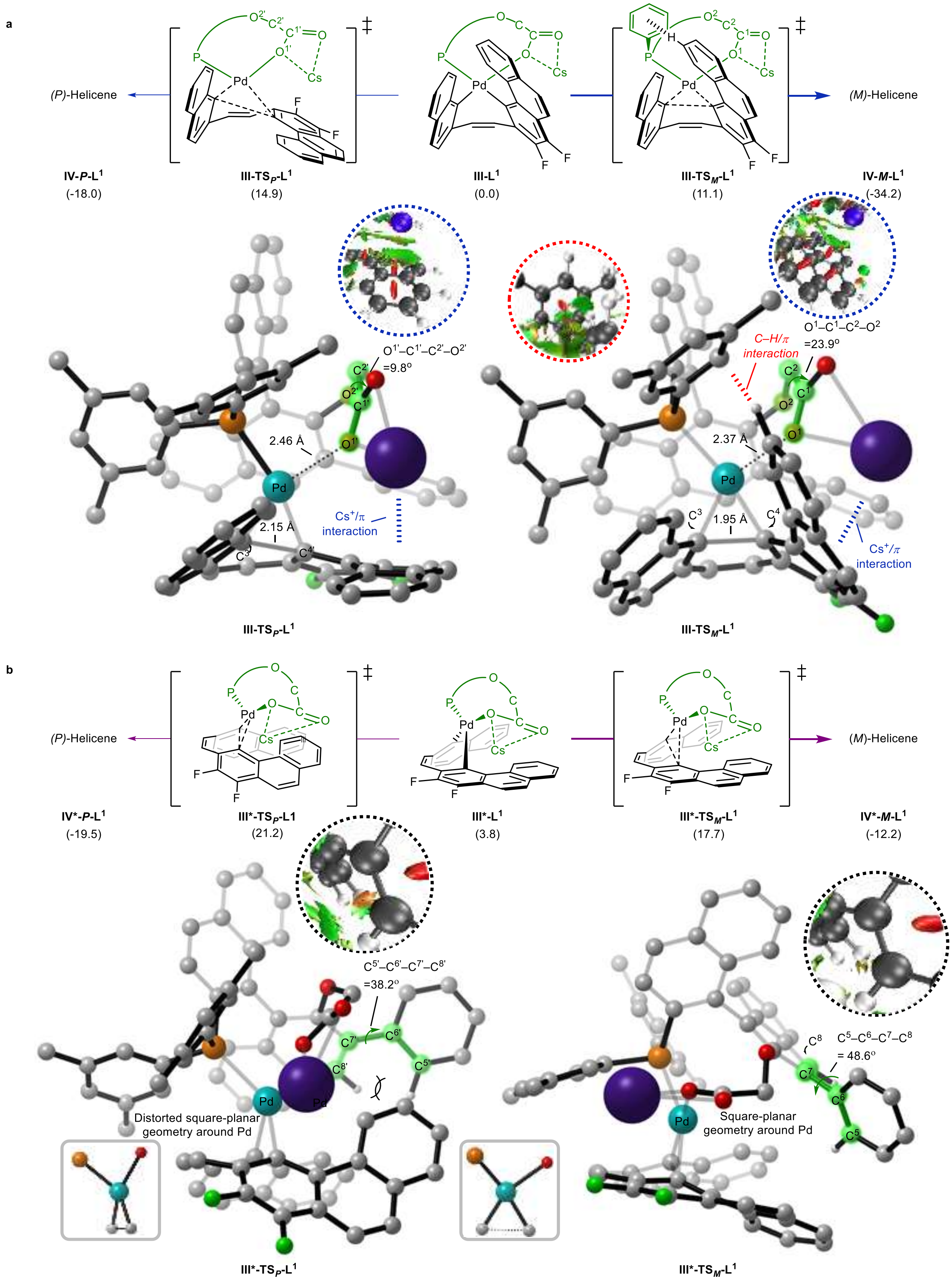

Fig. S6. Reductive elimination pathways with **L^1** including selected NCI plots.

Reductive elimination with L^2 employed as ligand

The reductive elimination TSs from intermediates **III-L^2** and **III*-L^2**, are qualitatively similar to those observed with **L^1**. The activation barriers were calculated to be $\Delta G^{\ddagger}$ = 11.8 kcal mol^{-1} for **III-TS$_M$-L^2** and $\Delta G^{\ddagger}$ = 16.6 kcal mol^{-1} for **III-TS$_P$-L^2**, hence favoring the experimentally observed major (*M*) enantiomer of carbo[6]helicene **2r**. Similar to the reaction employing **L^1** as ligands, noncovalent interactions played an important role in determining the stereochemical outcomes of the reaction.

In addition, two diastereomeric TSs from the intermediate **III*-L^2** were located, which were obtained through the second C–H activation pathway, **III*-TS$_M$-L^2** and **III*-TS$_P$-L^2**. The activation barriers were calculated to be $\Delta G^{\ddagger}$ = kcal mol^{-1} for **III*-TS$_M$-L^2**, whereas higher energy of $\Delta G^{\ddagger}$ = 16.9 kcal mol^{-1} was calculated for **III*-TS$_P$-L^2**, hence also favouring the (*M*) carbohelicene product **2r**. The difference in torsion angles $C^{13(')}$–$C^{14(')}$–$C^{15(')}$–$C^{16(')}$ of the phenyl-naphthyl moiety of the ligand and the deviation from the ideal square-planar geometry were observed, similar to the reaction using **L^1** as ligands.

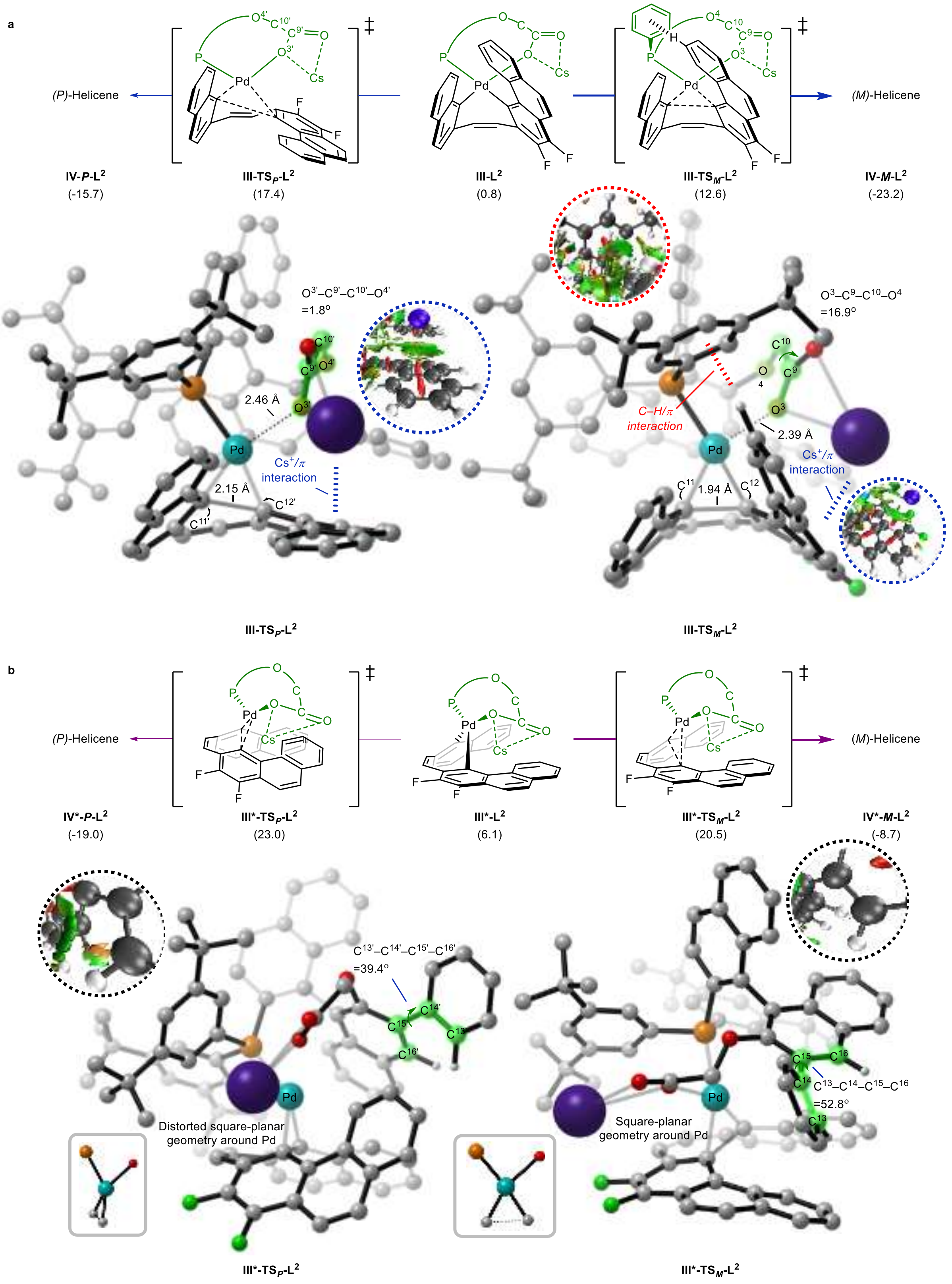

Fig. S7. Reductive elimination pathways with $\mathbf{L^2}$ including selected NCI plots.

Photophysical and Chiroptical Properties Study

General information:

UV−visible spectra were recorded on a JASCO V-750 spectrometer using a 10 mm*10 mm quartz cell. Circular dichroism (CD) spectra were recorded on a Jasco (model J-815) spectropolarimeter equipped with a Peltier thermostated cell holder and Xe laser. Data were recorded at 20°C using a 1 mm*10 mm quartz cell. The obtained signals were processed by subtracting solvent and cell contribution.

Emission spectra and Time Correlated Single Photon Counts (TCSPC) experiments were performed on an Edinburgh FS-5 spectrofluorometer with SC-20 module coupled with a 365 nm Picosecond pulsed diode lasers. A 10 mm*10 mm quartz cell was used. The absolute fluorescence quantum yields for solutions were determined using an SC-30 integrating sphere.

The circularly polarized luminescence (CPL) measurements were performed using a commercialized instrument JASCO CPL-300 at room temperature in 10 mm*10 mm cell. Excitation wavelength and instrument parameters were adapted for every sample. Data pitch was set at 1 nm and spectra displayed are mean values of a minimum of 10 accumulations.

UV-Fluo spectra:

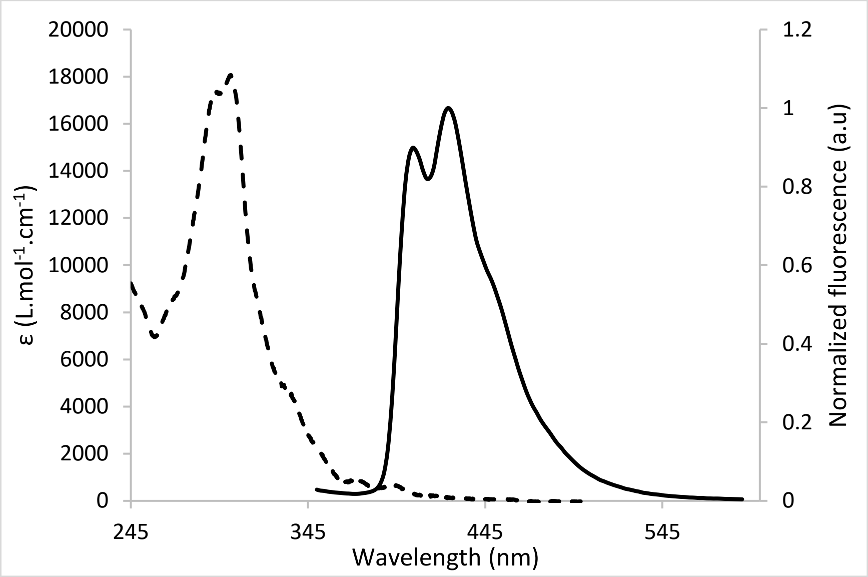

Fig. S8. ε = f (λ) (dashed line) and fluorescence (black line) spectra in dichloromethane for **2a**. [c] = 1 x 10^{-5} M

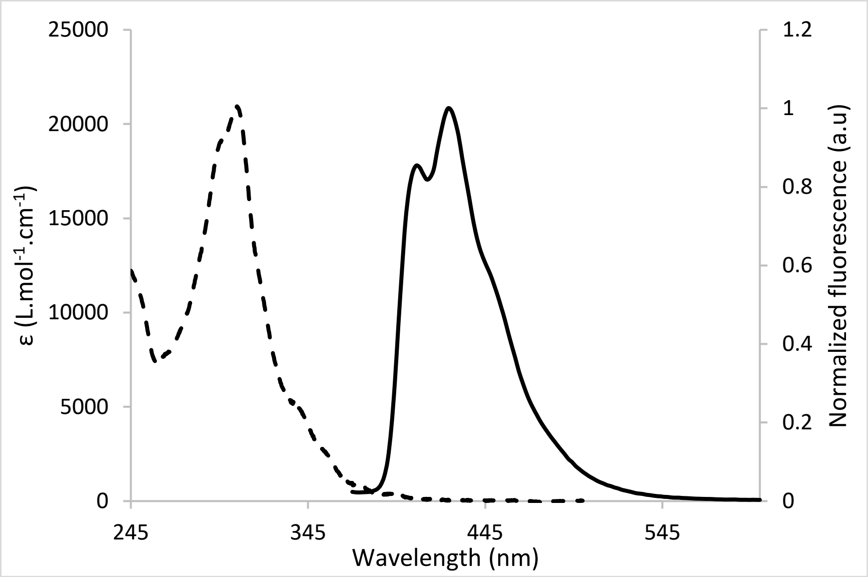

Fig. S9. ε = f (λ) (dashed line) and fluorescence (black line) spectra in dichloromethane for **2b**. [c] = 1 x 10^{-5} M

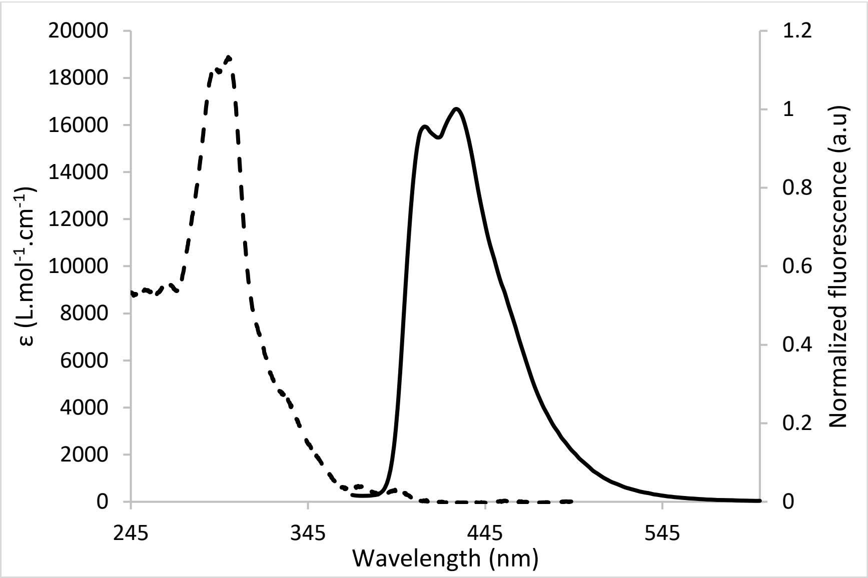

Fig. S10. ε = f (λ) (dashed line) and fluorescence (black line) spectra in dichloromethane for **2c**. [c] = 1 x 10^{-5} M

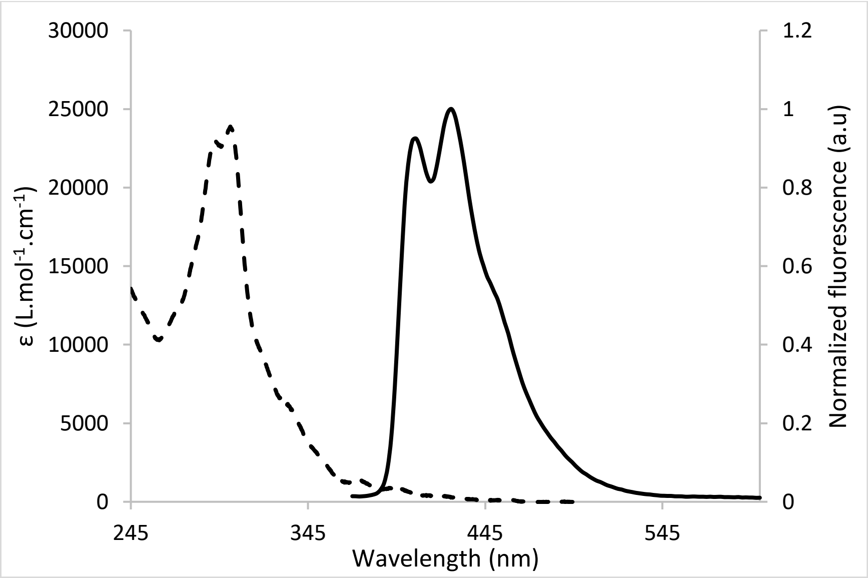

Fig. S11. ε = f (λ) (dashed line) and fluorescence (black line) spectra in dichloromethane for **2d**. [c] = 1 x 10^{-5} M

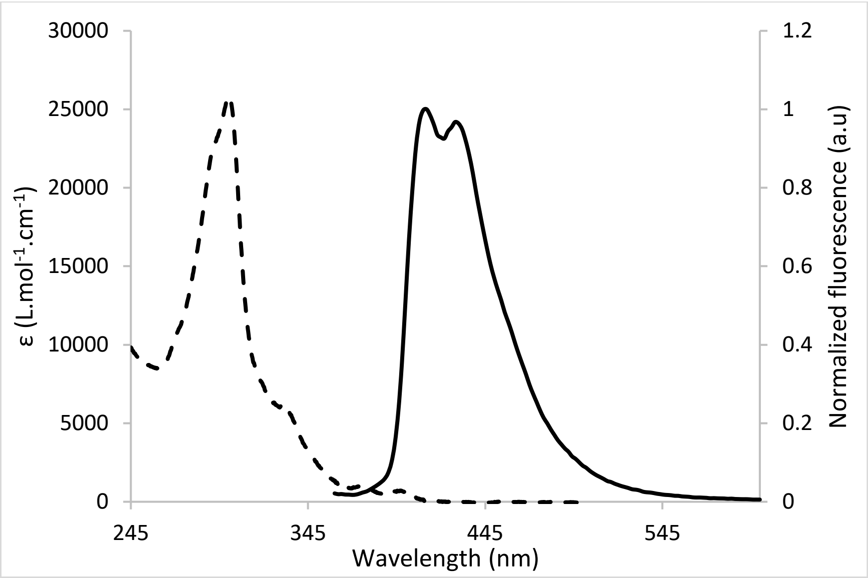

Fig. S12. ε = f (λ) (dashed line) and fluorescence (black line) spectra in dichloromethane for **2e**. [c] = 1 x 10^{-5} M

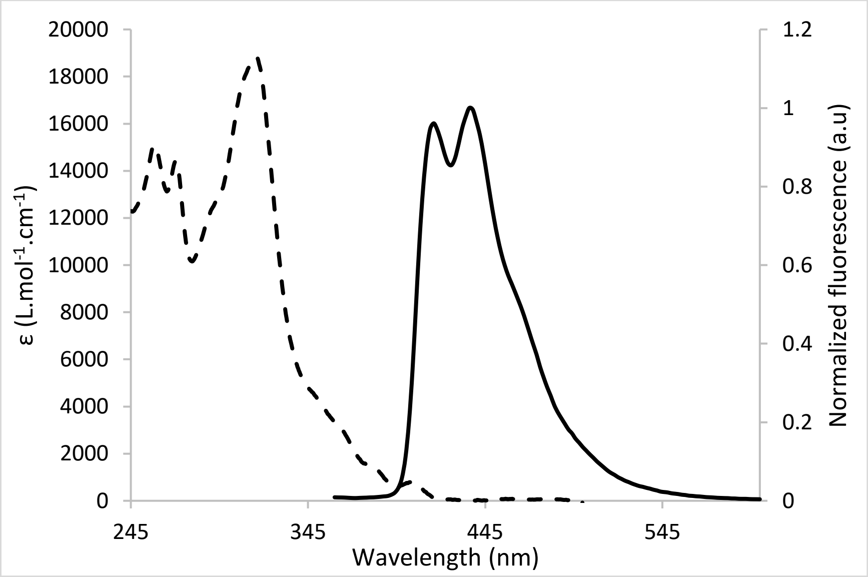

Fig. S13. ε = f (λ) (dashed line) and fluorescence (black line) spectra in dichloromethane for **2f**. [c] = 1 x 10^{-5} M

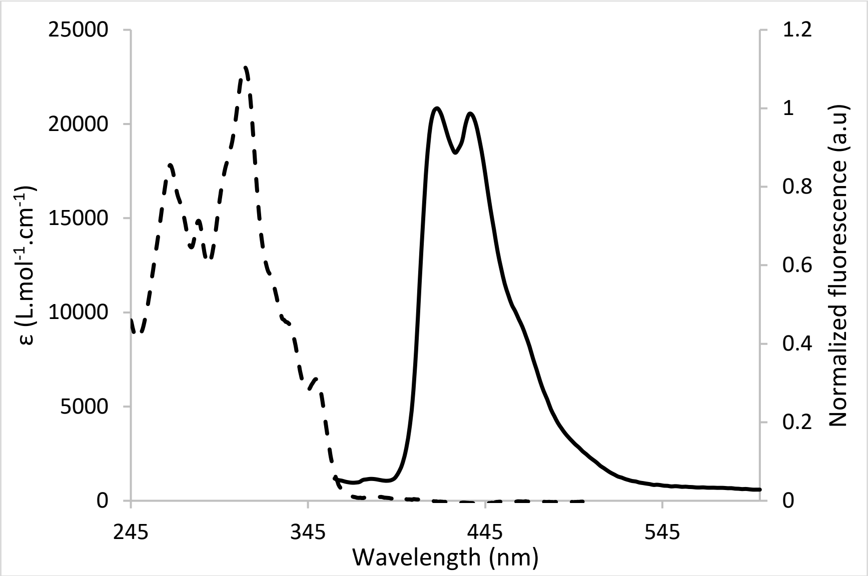

Fig. S14. ε = f (λ) (dashed line) and fluorescence (black line) spectra in dichloromethane for **2h**. [c] = 1 x 10^{-5} M

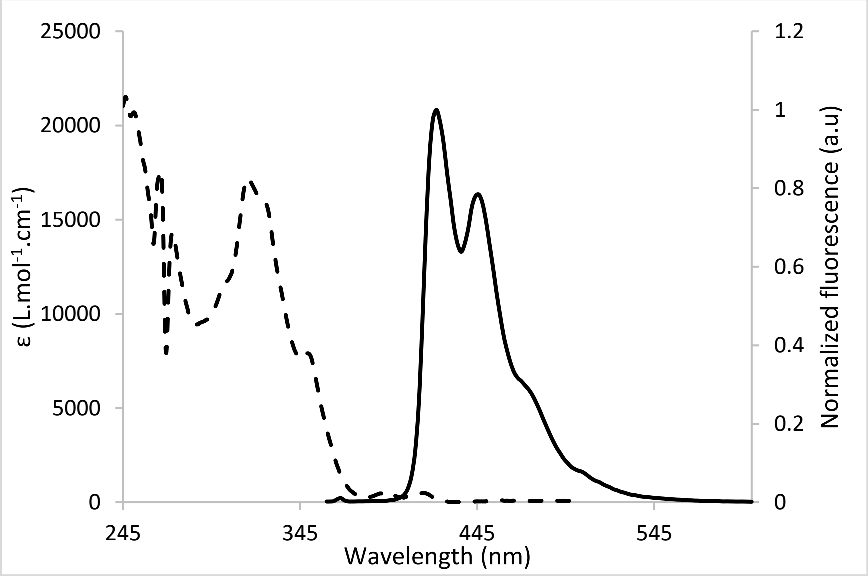

Fig. S15. ε = f (λ) (dashed line) and fluorescence (black line) spectra in dichloromethane for **2i**. [c] = 1 x 10^{-5} M

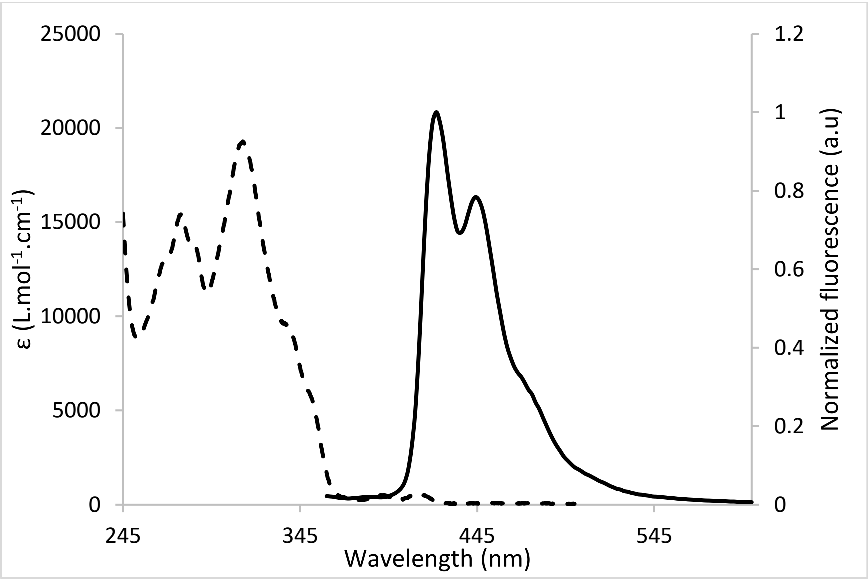

Fig. S16. ε = f (λ) (dashed line) and fluorescence (black line) spectra in dichloromethane for **2j**. [c] = 1 x 10^{-5} M

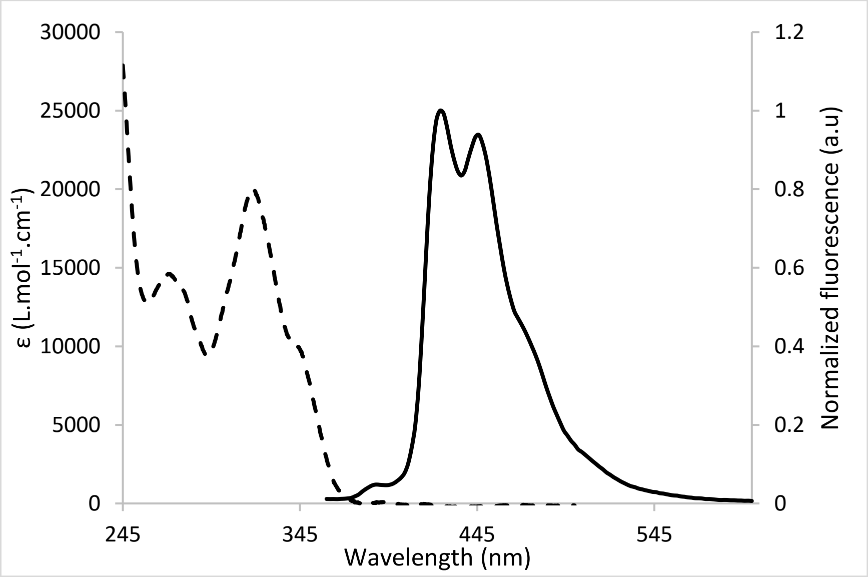

Fig. S17. ε = f (λ) (dashed line) and fluorescence (black line) spectra in dichloromethane for **2k**. [c] = 1 x 10^{-5} M

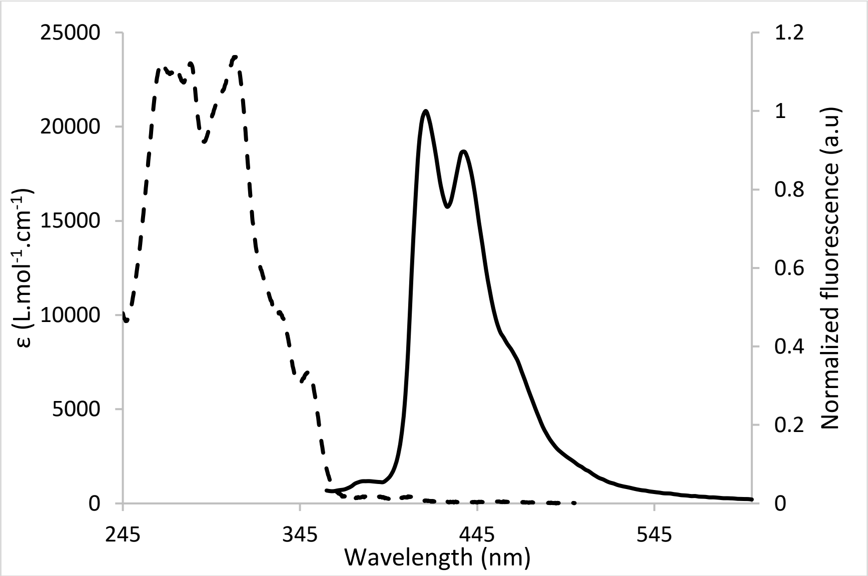

Fig. S18. ε = f (λ) (dashed line) and fluorescence (black line) spectra in dichloromethane for **2l**. [c] = 1 x 10^{-5} M

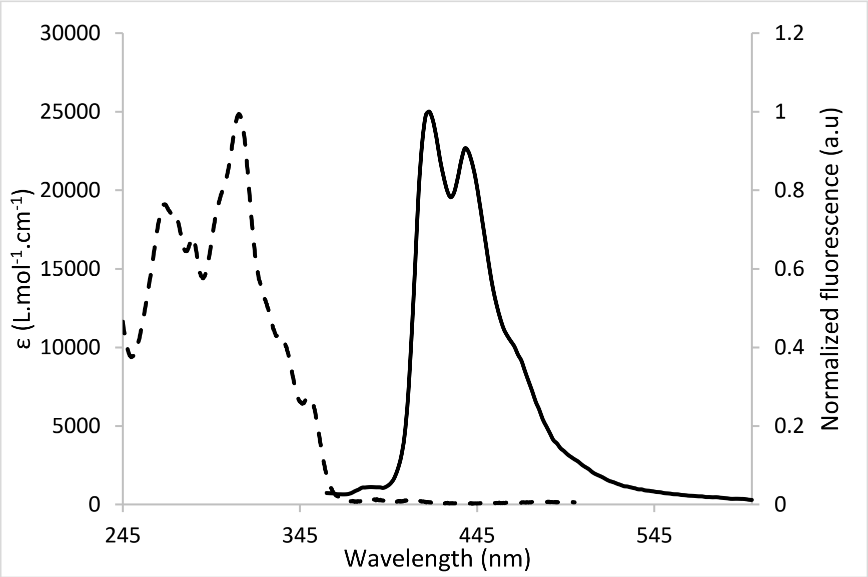

Fig. S19. ε = f (λ) (dashed line) and fluorescence (black line) spectra in dichloromethane for **2m**. [c] = 1 x 10^{-5} M

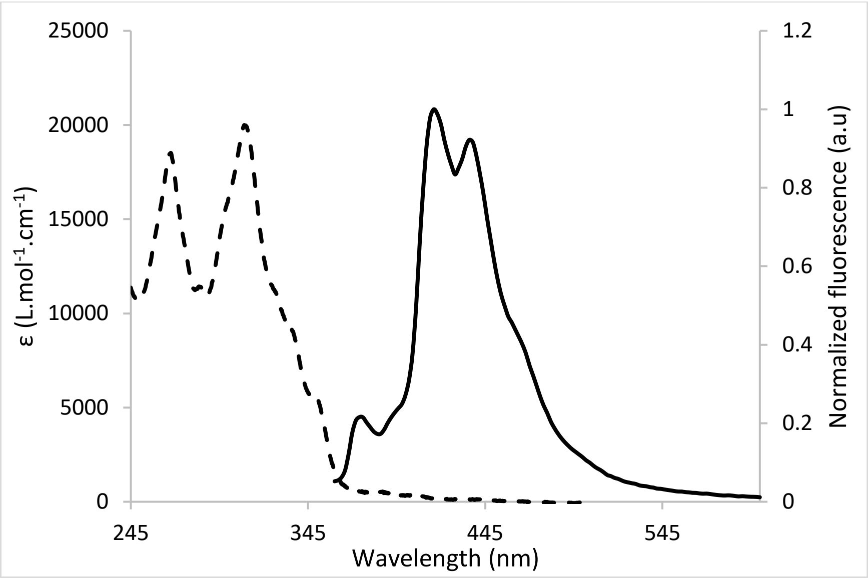

Fig. S20. ε = f (λ) (dashed line) and fluorescence (black line) spectra in dichloromethane for **2n**. [c] = 1 x 10^{-5} M

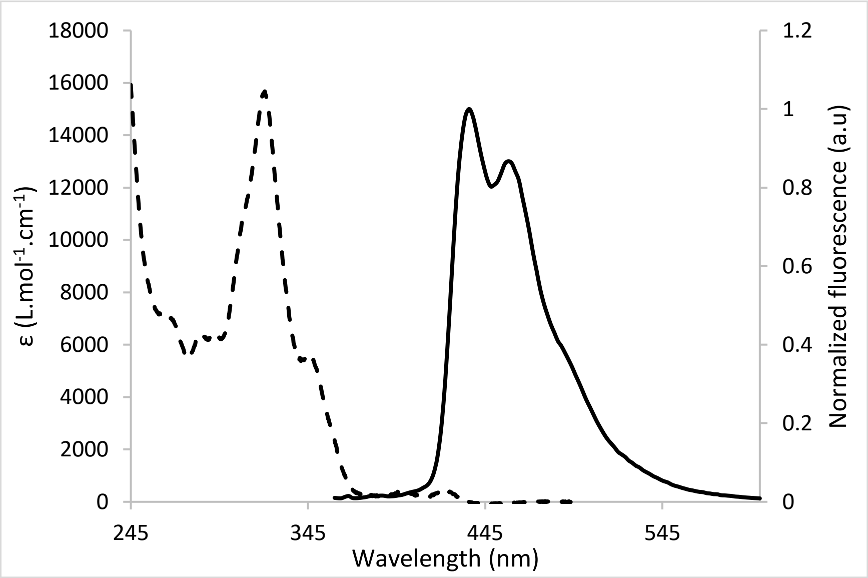

Fig. S21. ε = f (λ) (dashed line) and fluorescence (black line) spectra in dichloromethane for **2o**. [c] = 1 x 10^{-5} M

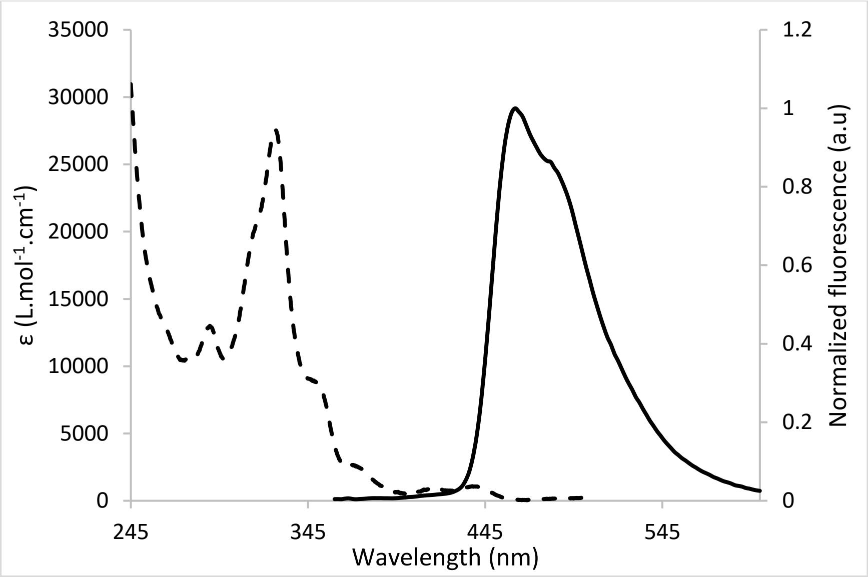

Fig. S22. ε = f (λ) (dashed line) and fluorescence (black line) spectra in dichloromethane for **2p**. [c] = 1 x 10^{-5} M

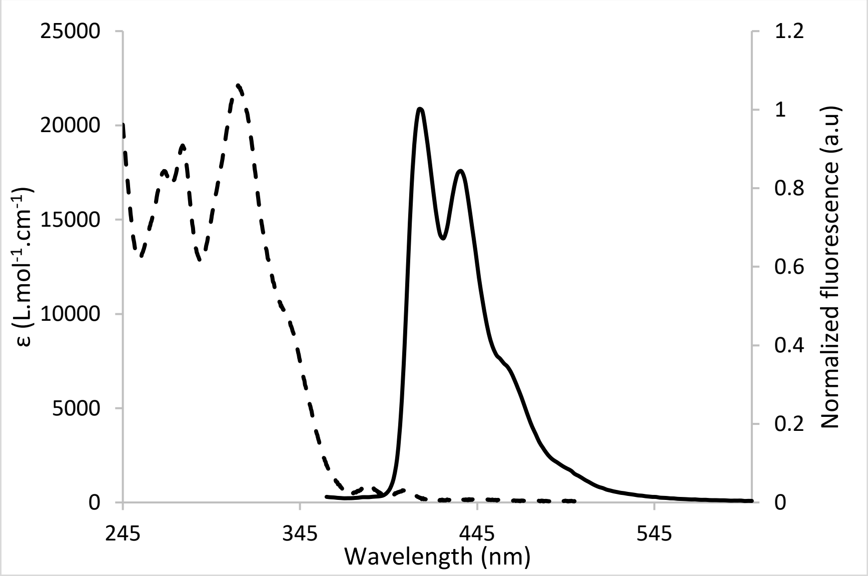

Fig. S23. ε = f (λ) (dashed line) and fluorescence (black line) spectra in dichloromethane for **2q**. [c] = 1 x 10^{-5} M

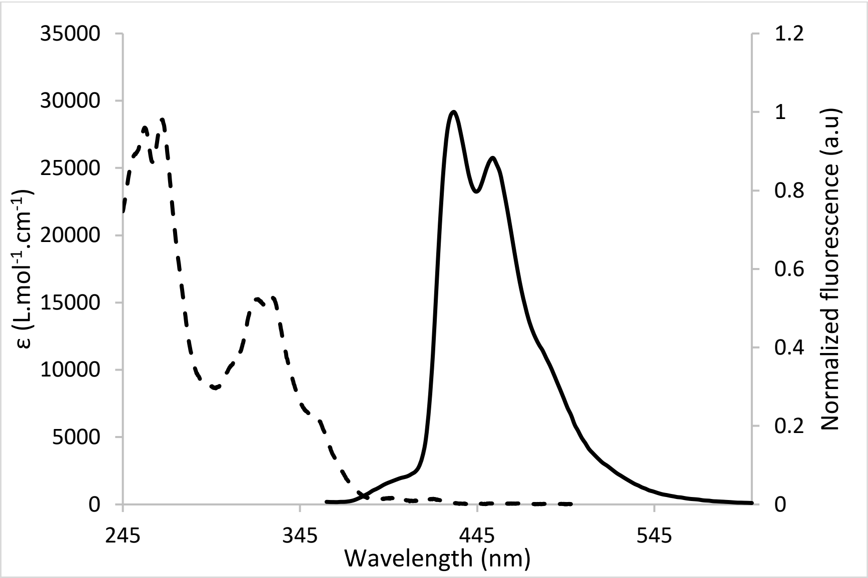

Fig. S24. ε = f (λ) (dashed line) and fluorescence (black line) spectra in dichloromethane for **2s**. [c] = 1 x 10^{-5} M

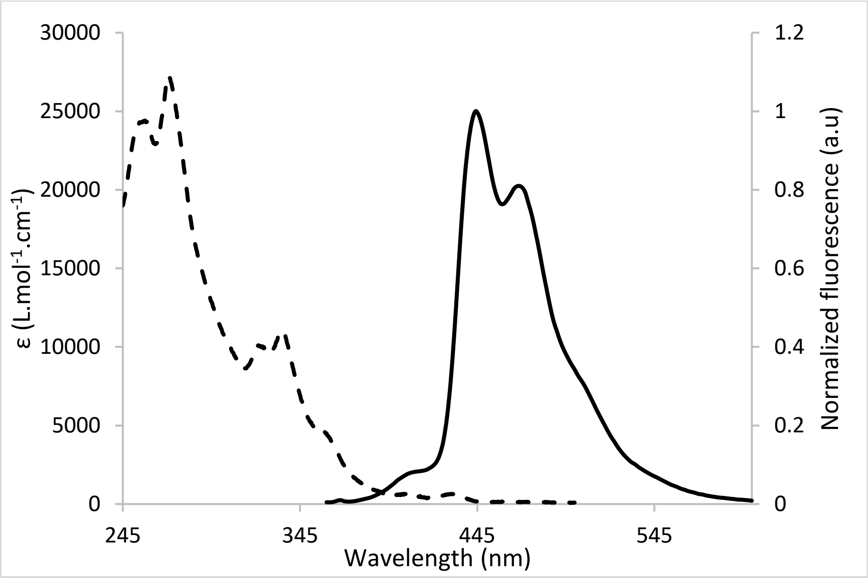

Fig. S25. ε = f (λ) (dashed line) and fluorescence (black line) spectra in dichloromethane for **2t**. [c] = 1 x 10^{-5} M

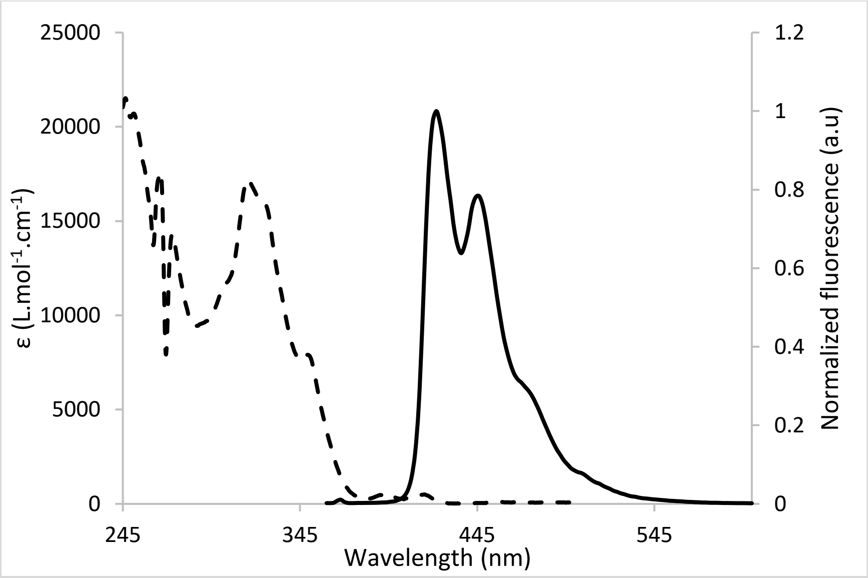

Fig. S26. ε = f (λ) (dashed line) and fluorescence (black line) spectra in dichloromethane for **2u**. [c] = 1 x 10^{-5} M

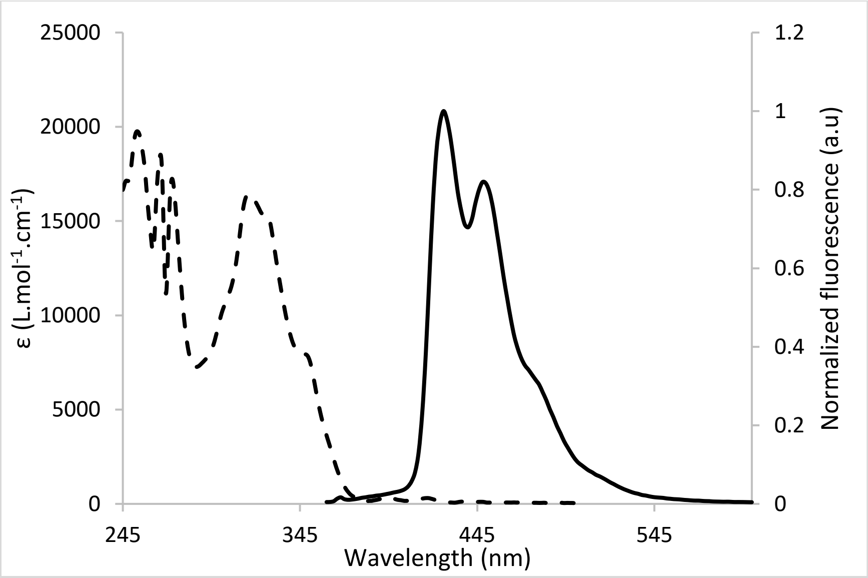

Fig. S27. ε = f (λ) (dashed line) and fluorescence (black line) spectra in dichloromethane for **2v**. [c] = 1 x 10^{-5} M

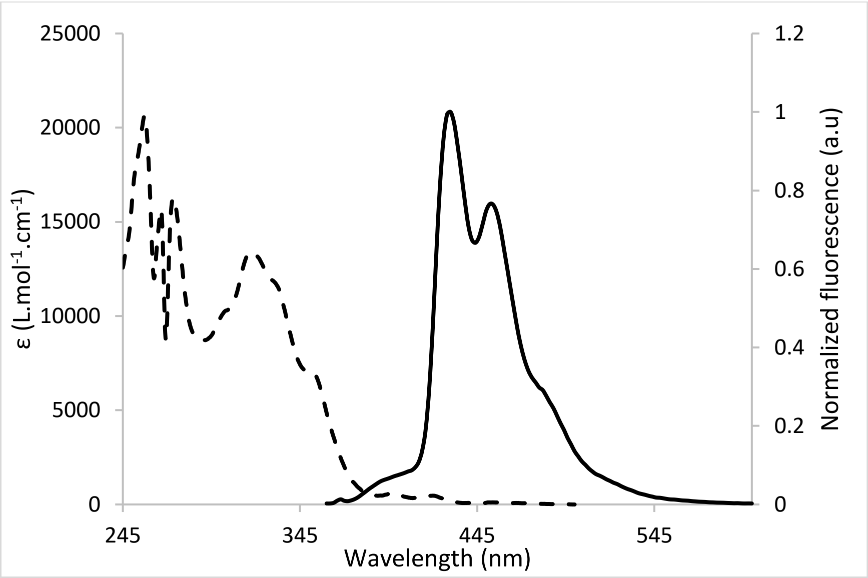

Fig. S28. ε = f (λ) (dashed line) and fluorescence (black line) spectra in dichloromethane for **2w**. [c] = 1 x 10^{-5} M

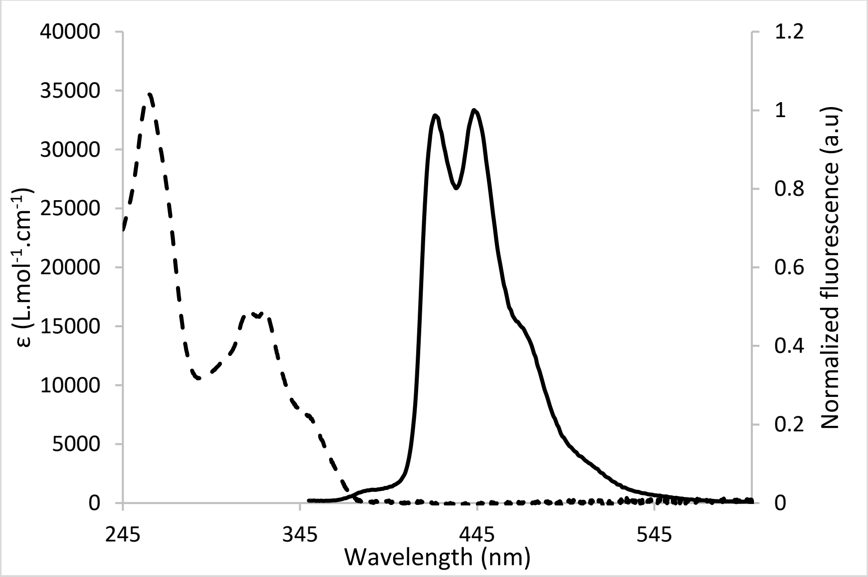

Fig. S29. ε = f (λ) (dashed line) and fluorescence (black line) spectra in dichloromethane for **2x**. [c] = 1 x 10^{-5} M

Circularly polarized luminescence spectra:

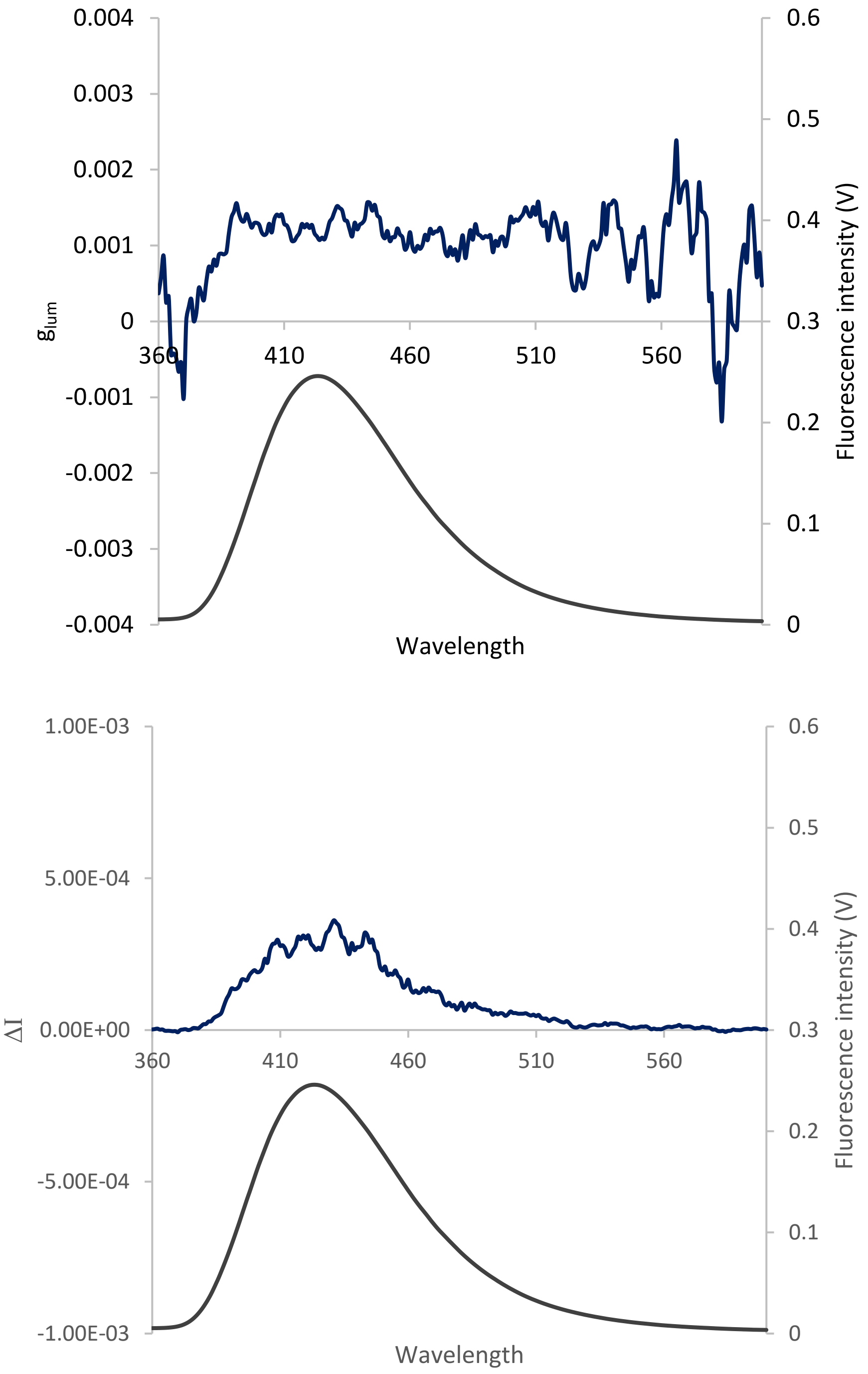

Fig. S30. g_{lum} = f (λ) (top) and CPL spectra (bottom) in dichloromethane for **2a**. [c] = 1 x 10^{-5} M. λ_{exc} = 290 nm.

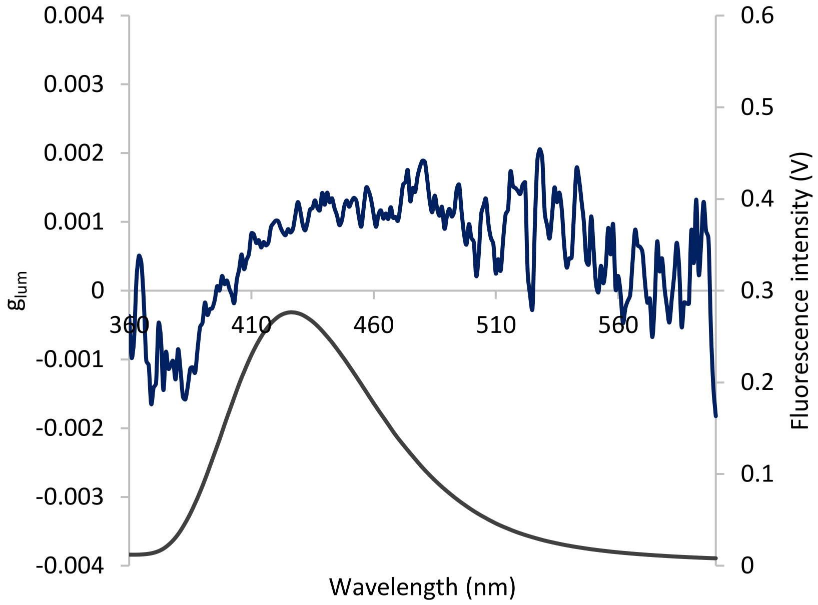

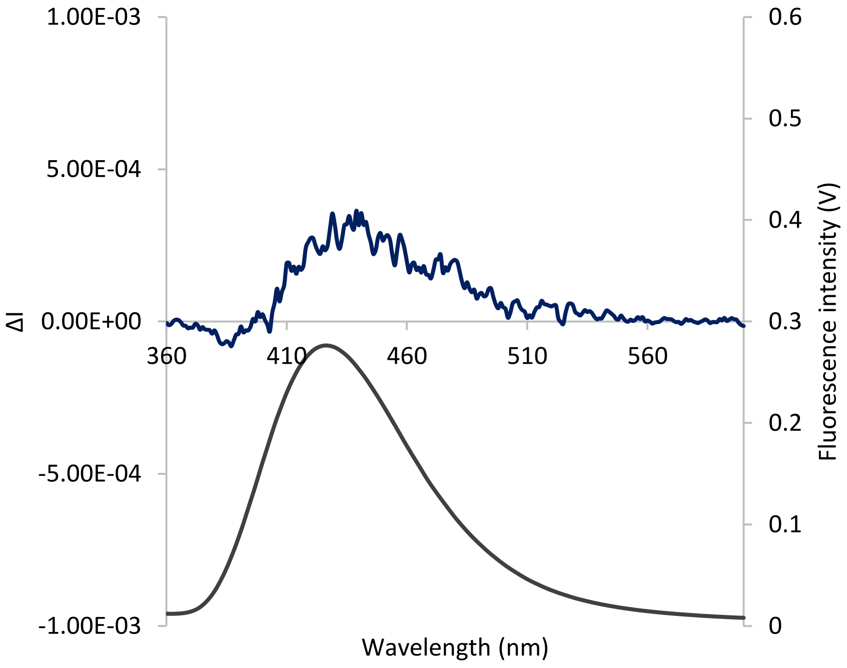

Fig. S31. g_{lum} = f (λ) (top) and CPL spectra (bottom) in dichloromethane for **2b**. [c] = 1 x 10^{-5} M. λ_{exc} = 290 nm.

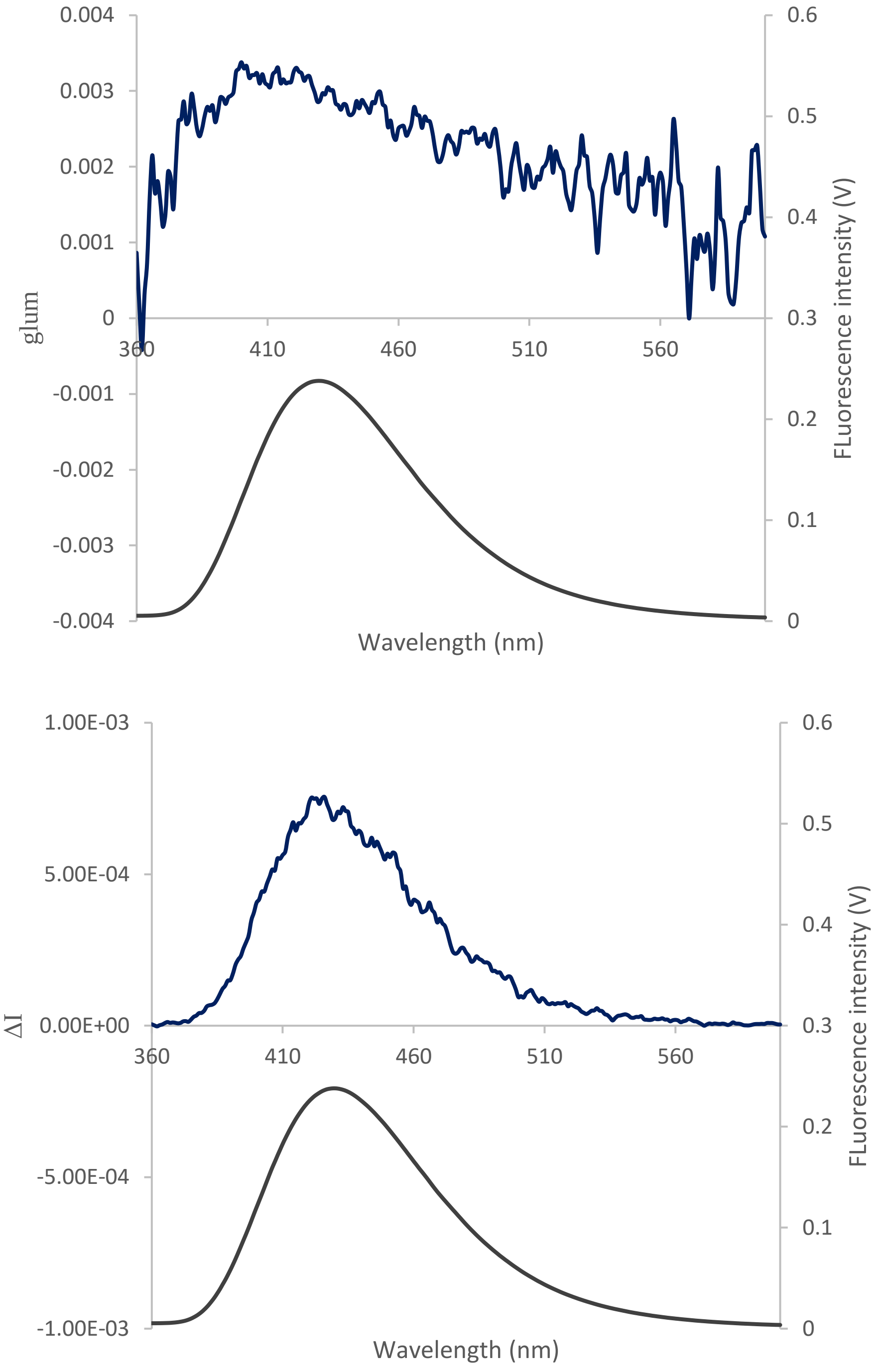

Fig. S32. g_{lum} = f (λ) (top) and CPL spectra (bottom) in dichloromethane for **2c**. [c] = 1 x 10^{-5} M. λ_{exc} = 290 nm.

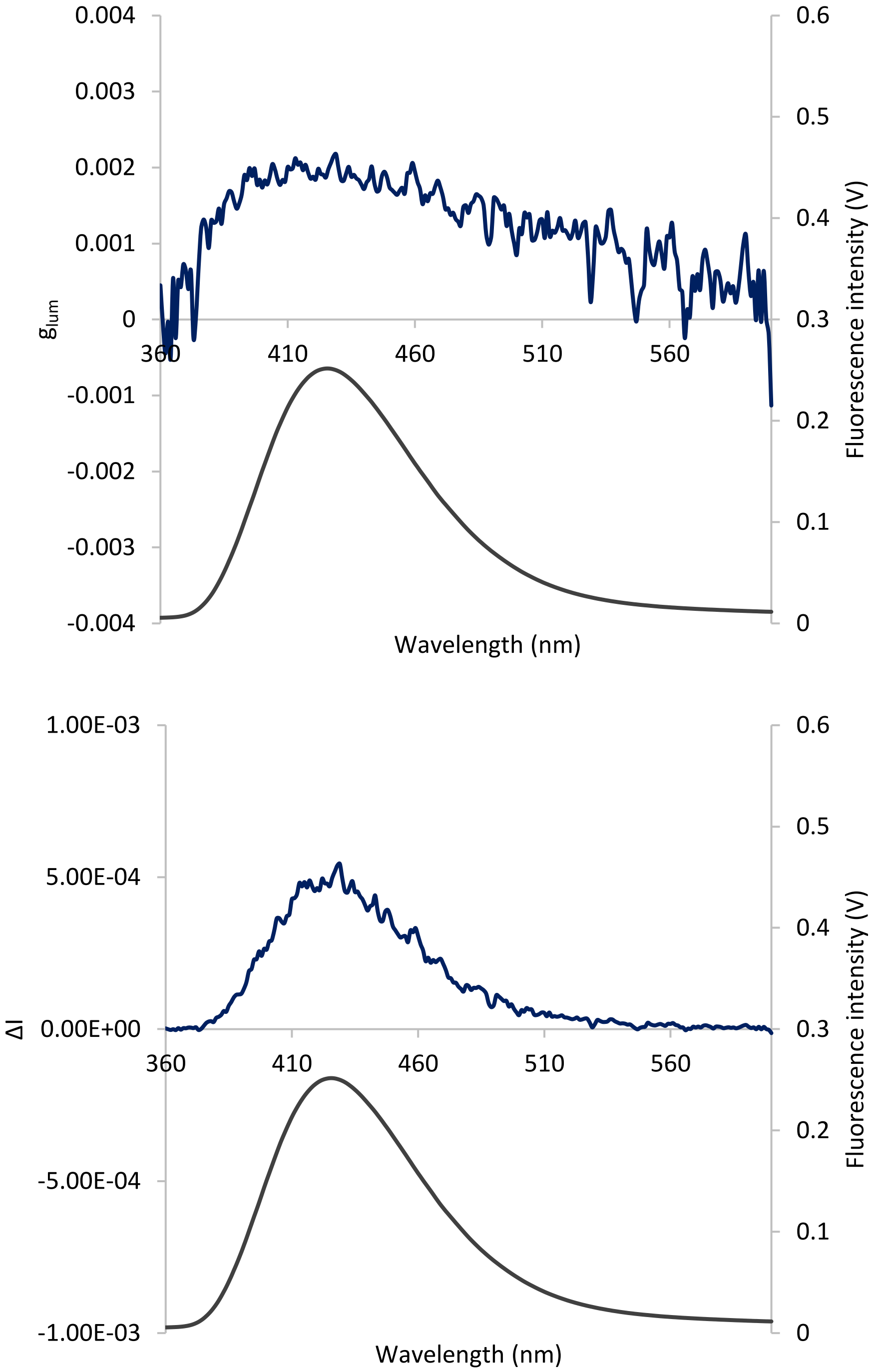

Fig. S33. g_{lum} = f (λ) (top) and CPL spectra (bottom) in dichloromethane for **2d**. [c] = 1 x 10^{-5} M

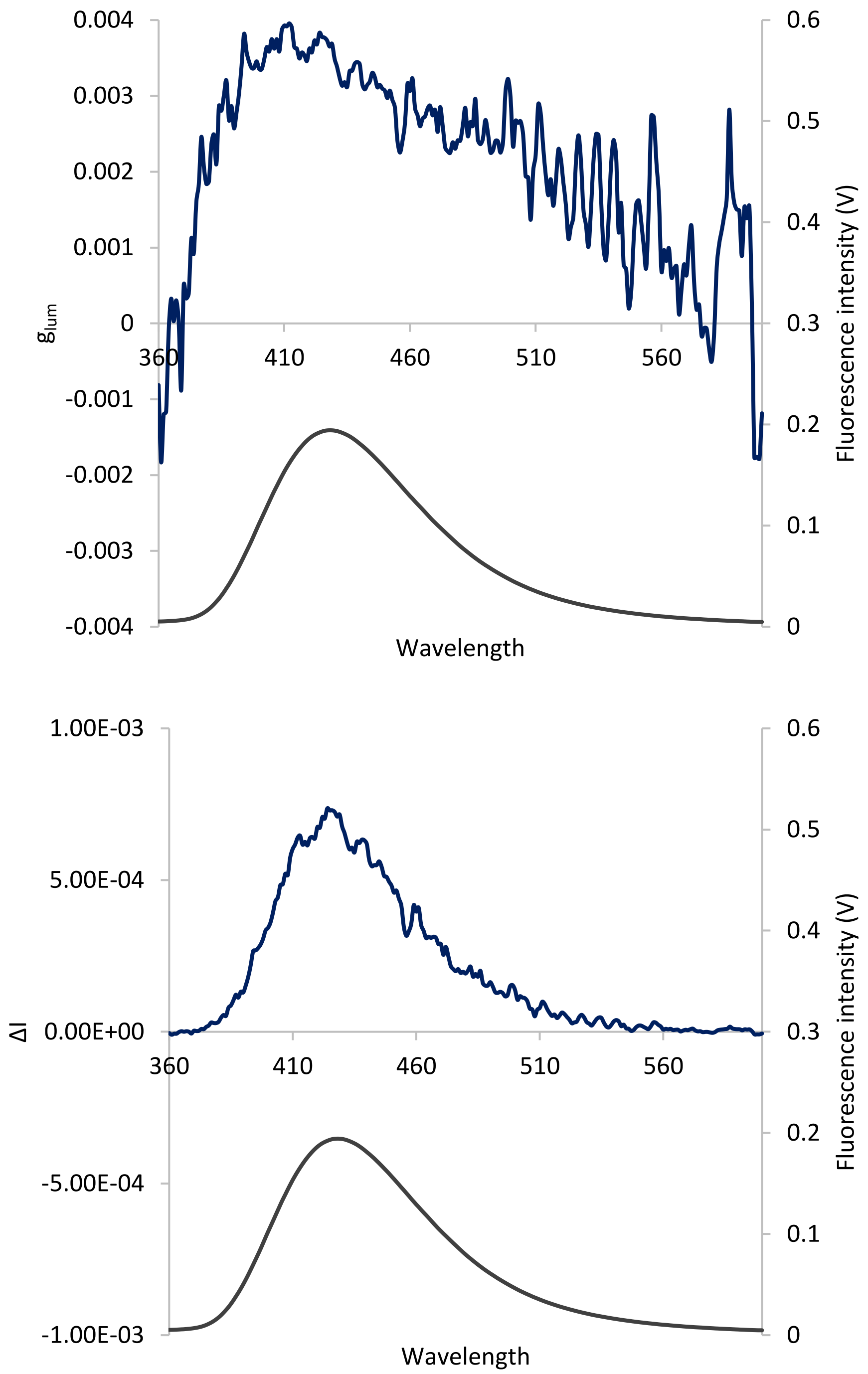

Fig. S34. g_{lum} = f (λ) (top) and CPL spectra (bottom) in dichloromethane for **2e**. [c] = 1 x 10^{-5} M. λ_{exc} = 290 nm.

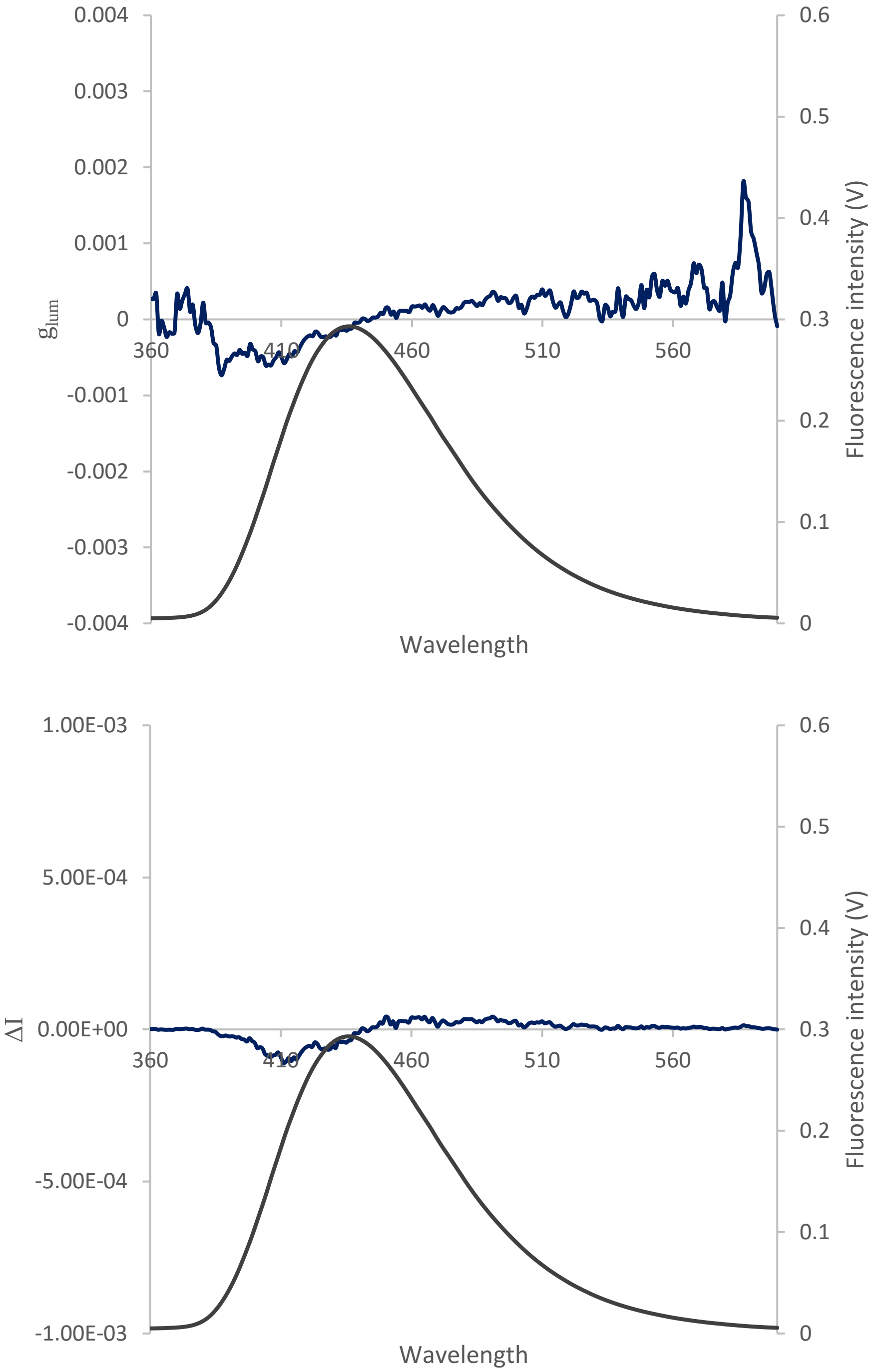

Fig. S35. g_{lum} = f (λ) (top) and CPL spectra (bottom) in dichloromethane for **2f**. [c] = 1 x 10^{-5} M. λ_{exc} = 290 nm.

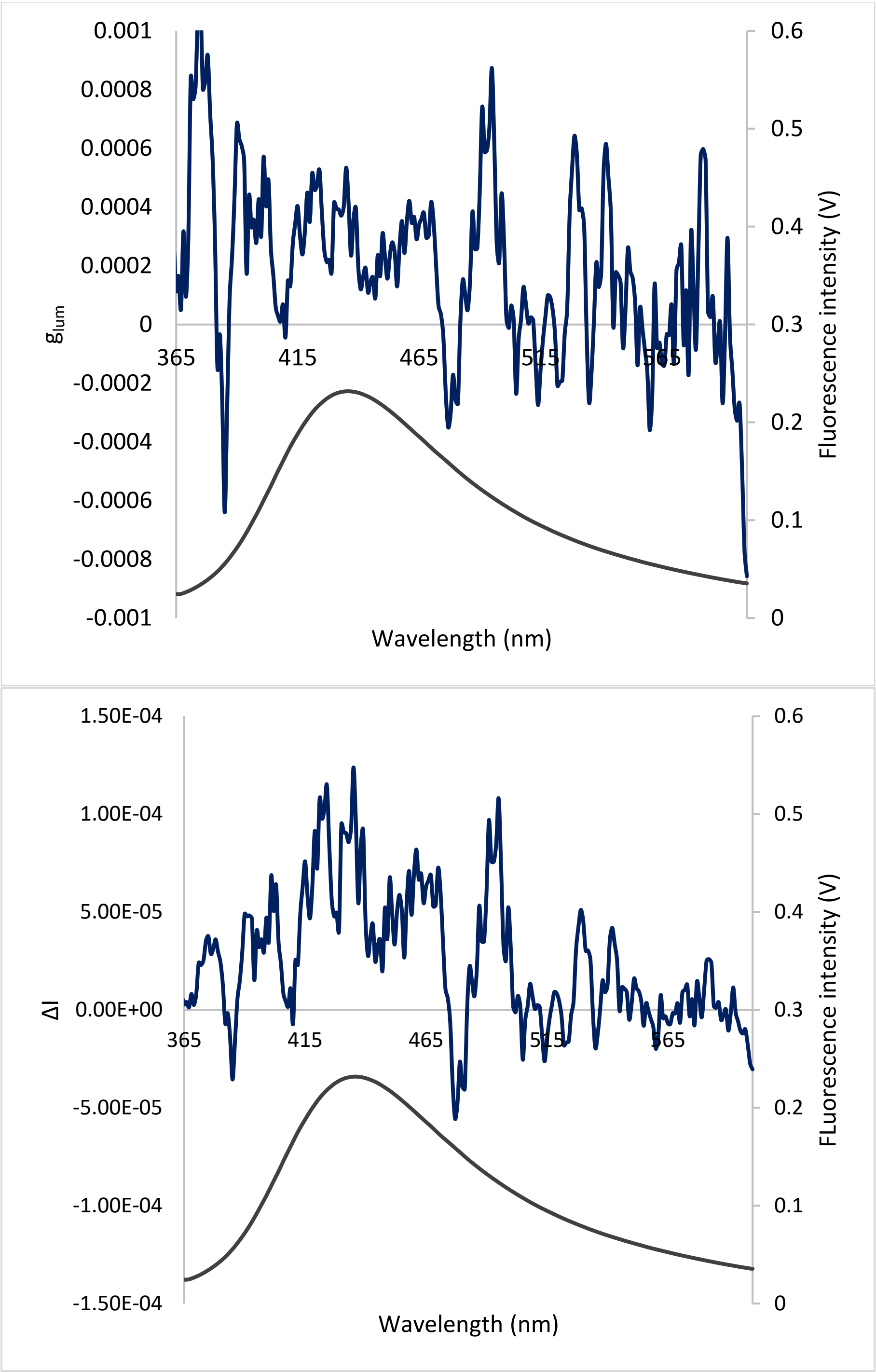

Fig. S36. g_{lum} = f (λ) (top) and CPL spectra (bottom) in dichloromethane for **2h**. [c] = 1 x 10^{-5} M. λ_{exc} = 310 nm.

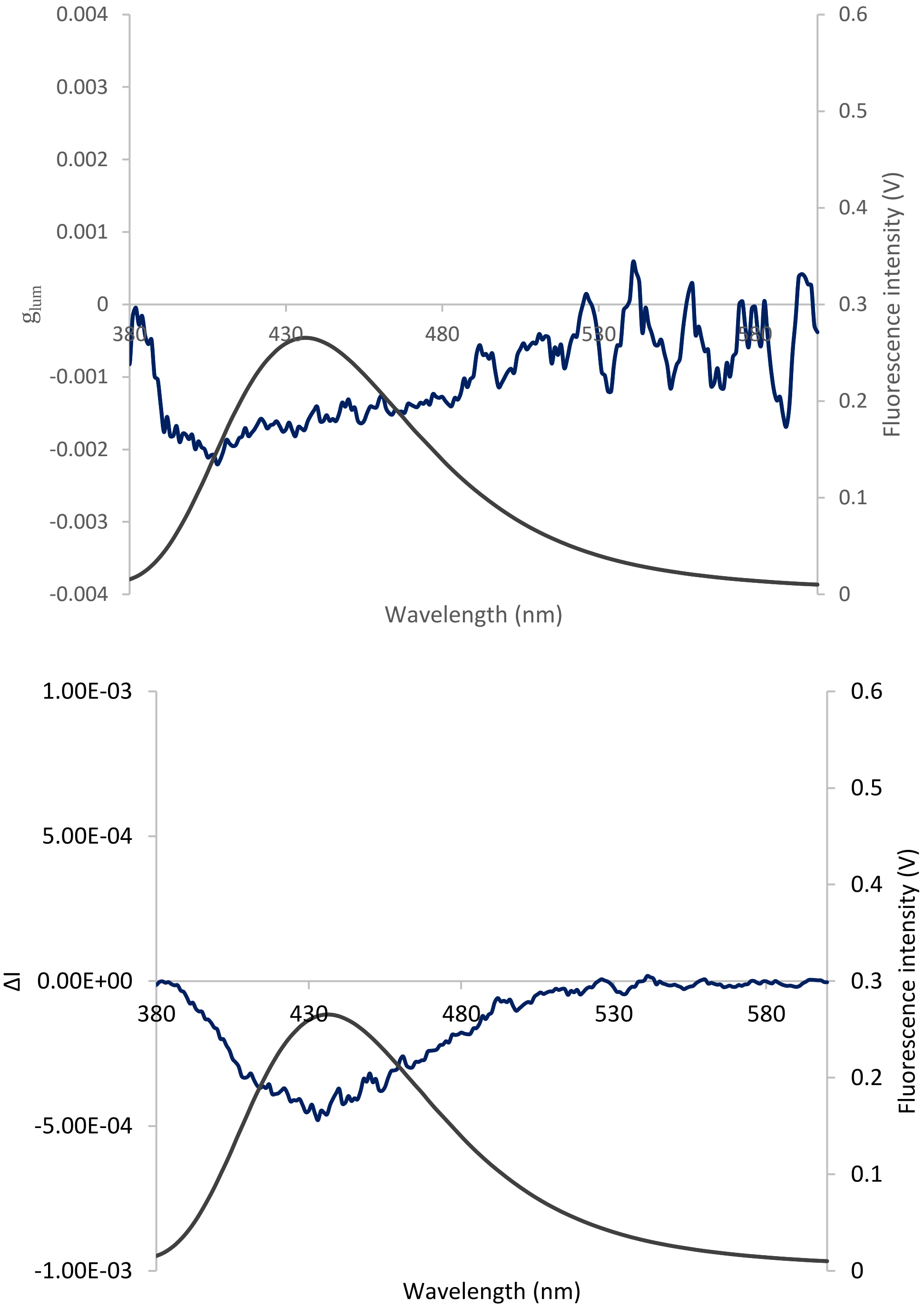

Fig. S37. g_{lum} = f (λ) (top) and CPL spectra (bottom) in dichloromethane for **2i**. [c] = 1 x 10^{-5} M. λ_{exc} = 310 nm.

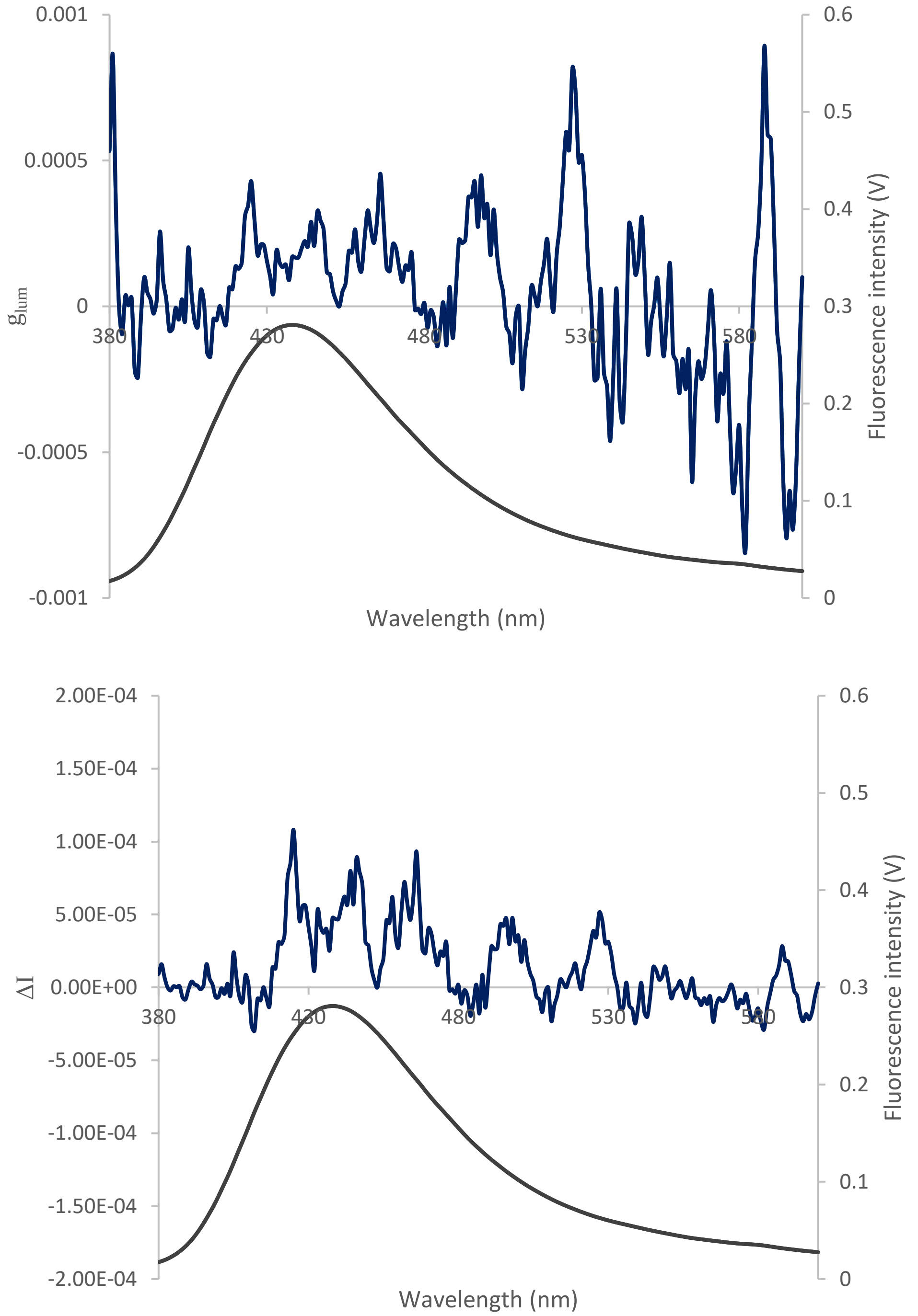

Fig. S38. g_{lum} = f (λ) (top) and CPL spectra (bottom) in dichloromethane for **2j**. [c] = 1 x 10^{-5} M. λ_{exc} = 310 nm.

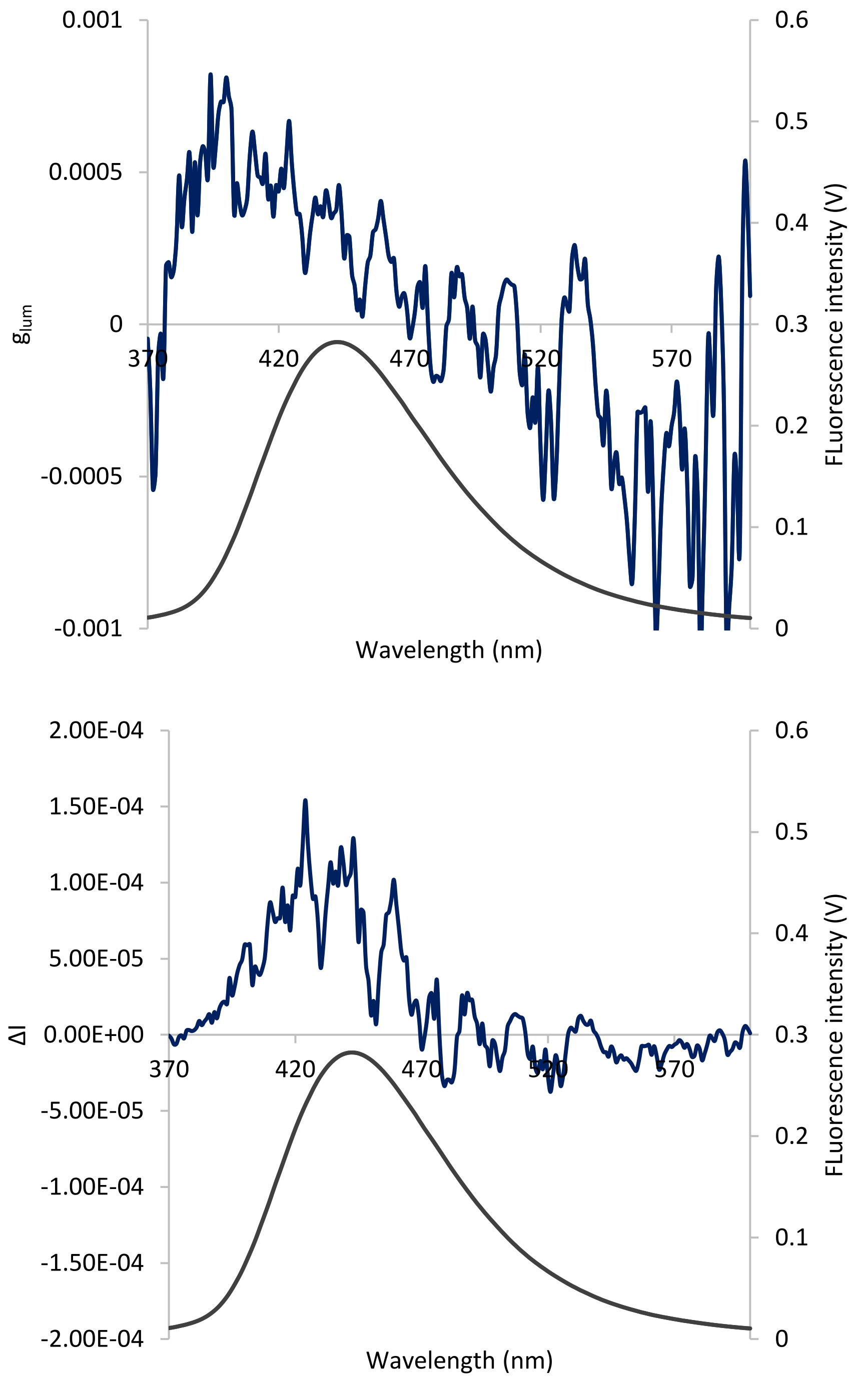

Fig. S39. $g_{lum} = f(\lambda)$ (top) and CPL spectra (bottom) in dichloromethane for **2k**. [c] = 1 x 10^{-5} M. λ_{exc} = 310 nm.

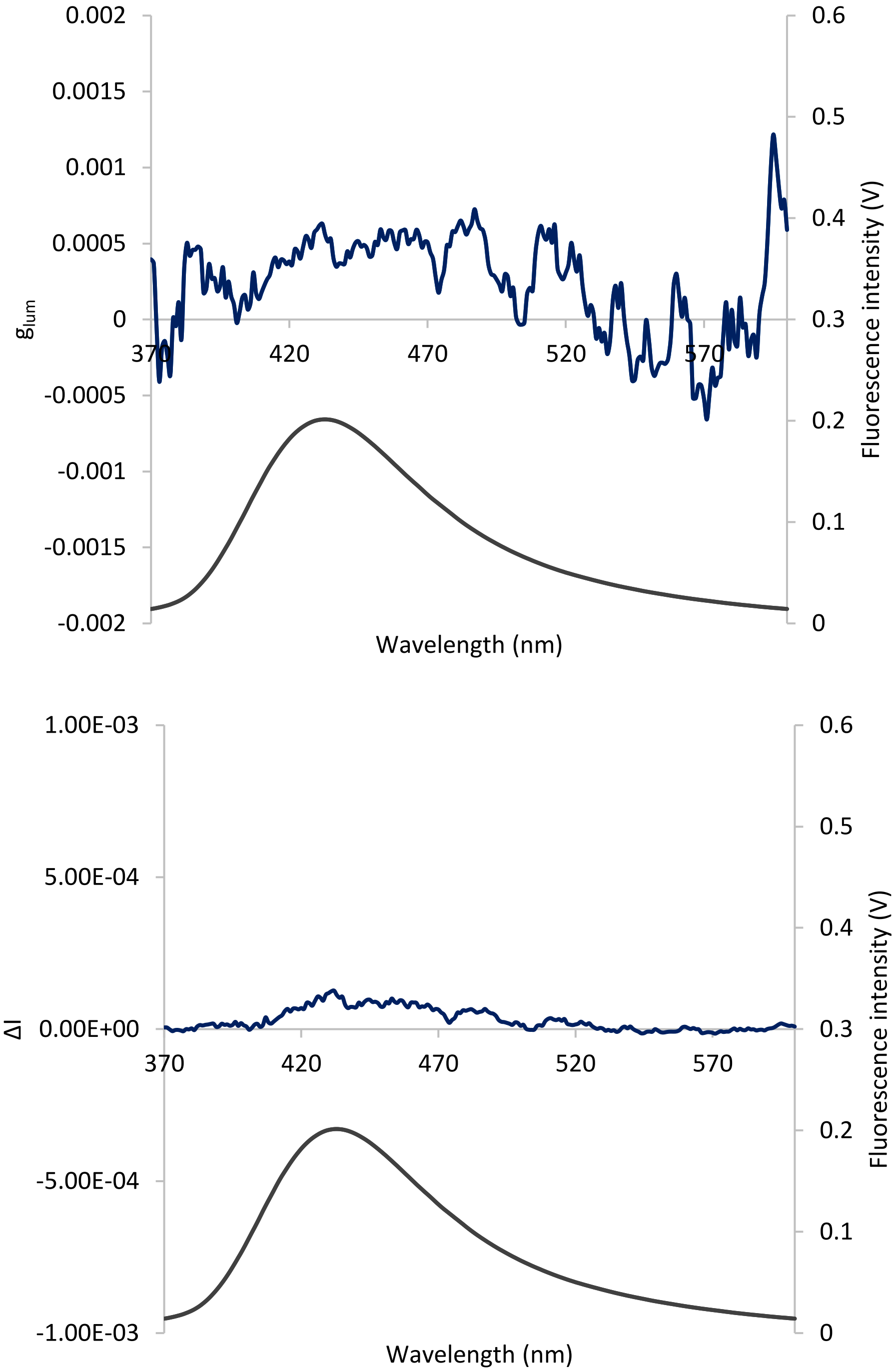

Fig. S40. g_{lum} = f (λ) (top) and CPL spectra (bottom) in dichloromethane for **2l**. [c] = 1 x 10^{-5} M. λ_{exc} = 310 nm.

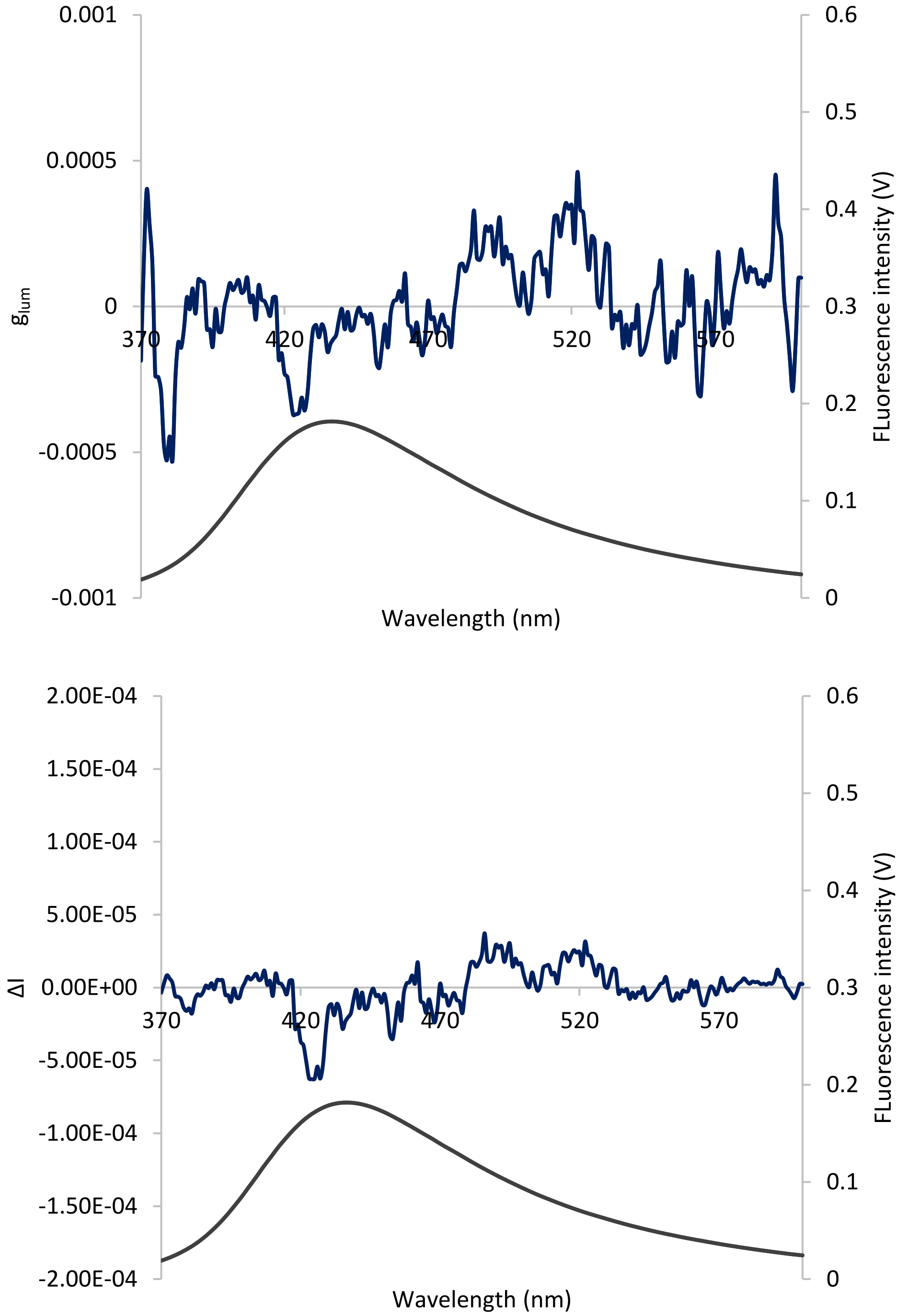

Fig. S41. g_{lum} = f (λ) (top) and CPL spectra (bottom) in dichloromethane for **2m**. [c] = 1 x 10^{-5} M. λ_{exc} = 310 nm.

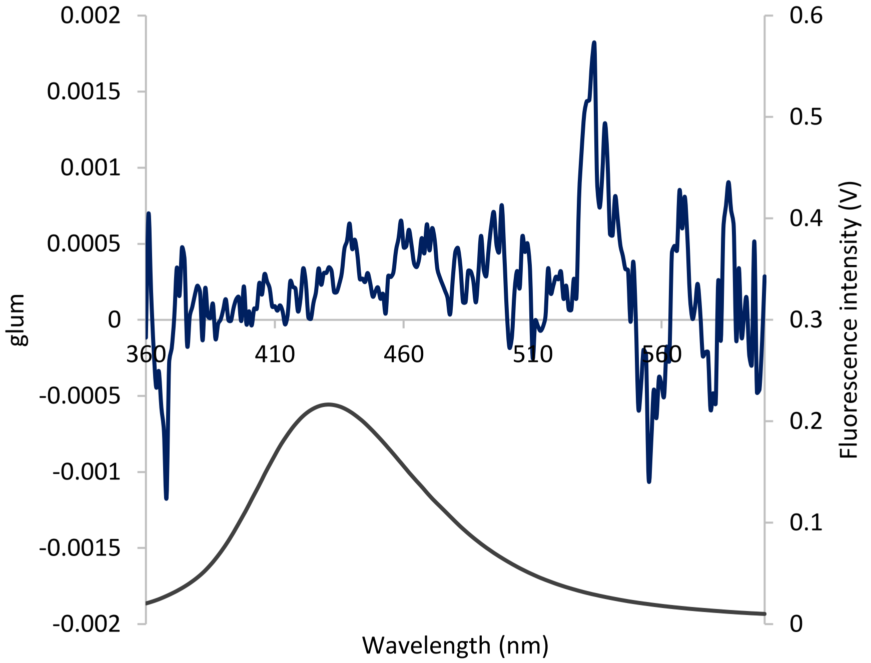

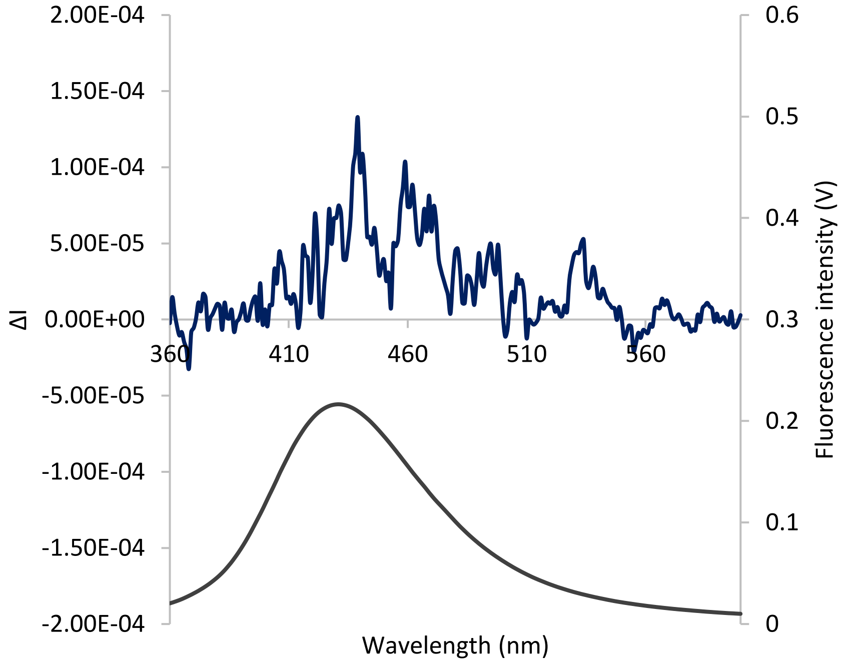

Fig. S42. g_{lum} = f (λ) (top) and CPL spectra (bottom) in dichloromethane for **2n**. [c] = 1 x 10^{-5} M. λ_{exc} = 310 nm.

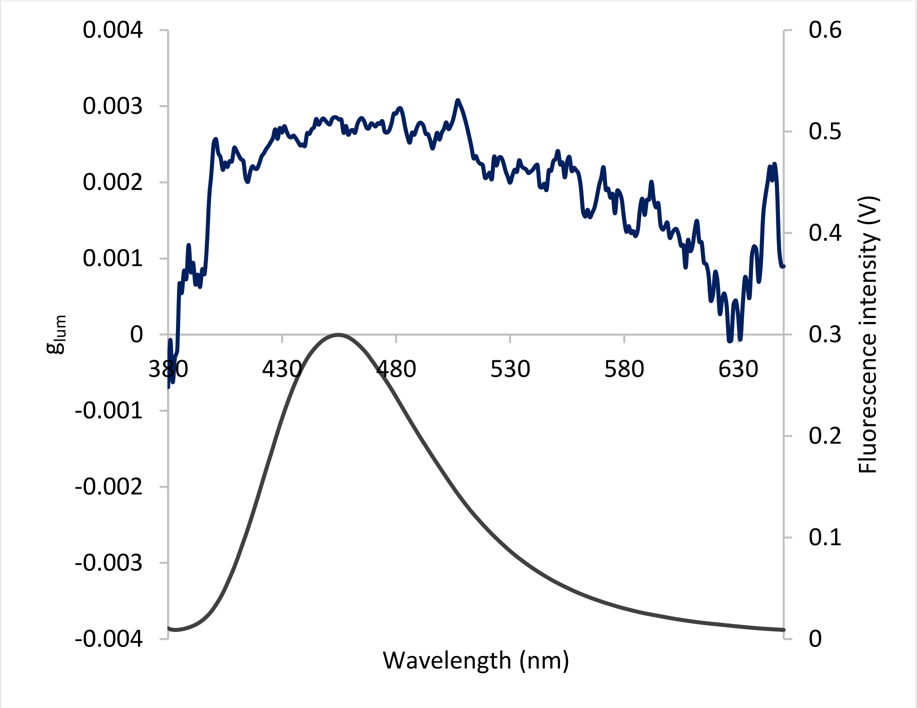

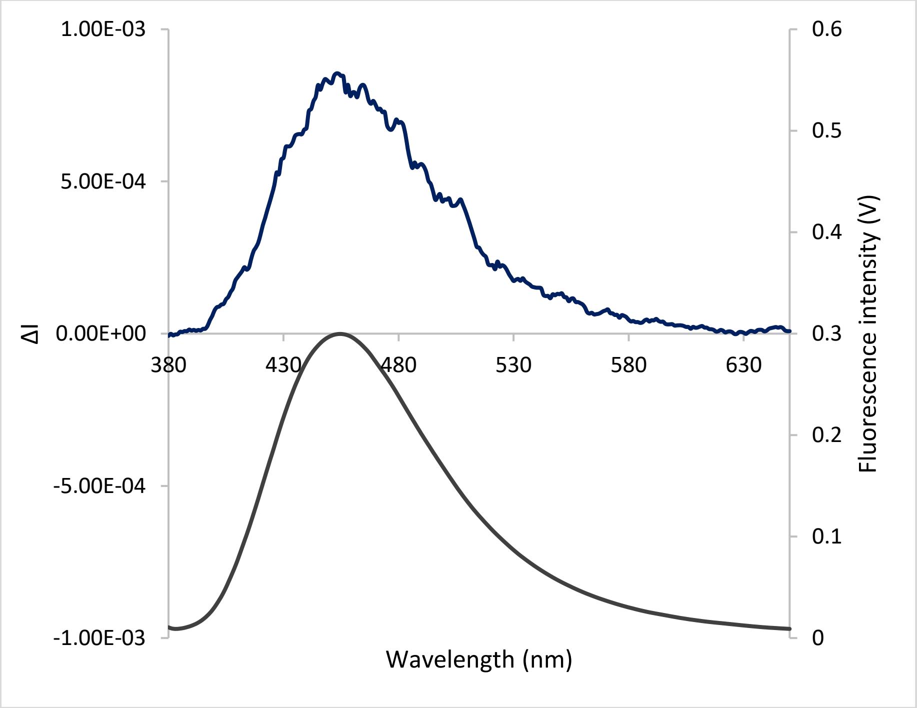

Fig. S43. g_{lum} = f (λ) (top) and CPL spectra (bottom) in dichloromethane for **2o**. [c] = 1 x 10^{-5} M. λ_{exc} = 320 nm.

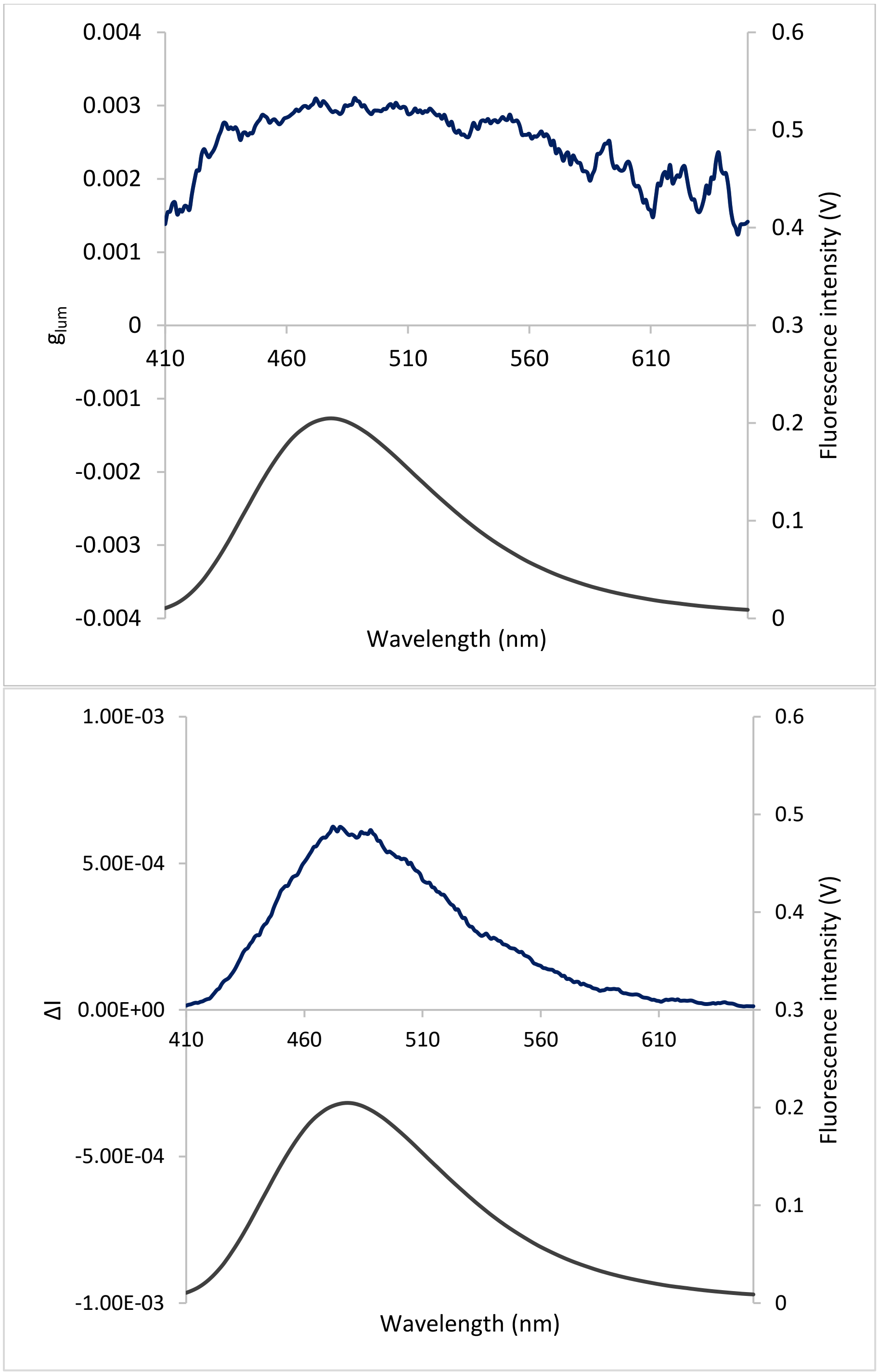

Fig. S44. g_{lum} = f (λ) (top) and CPL spectra (bottom) in dichloromethane for **2p**. [c] = 1 x 10^{-5} M. λ_{exc} = 330 nm.

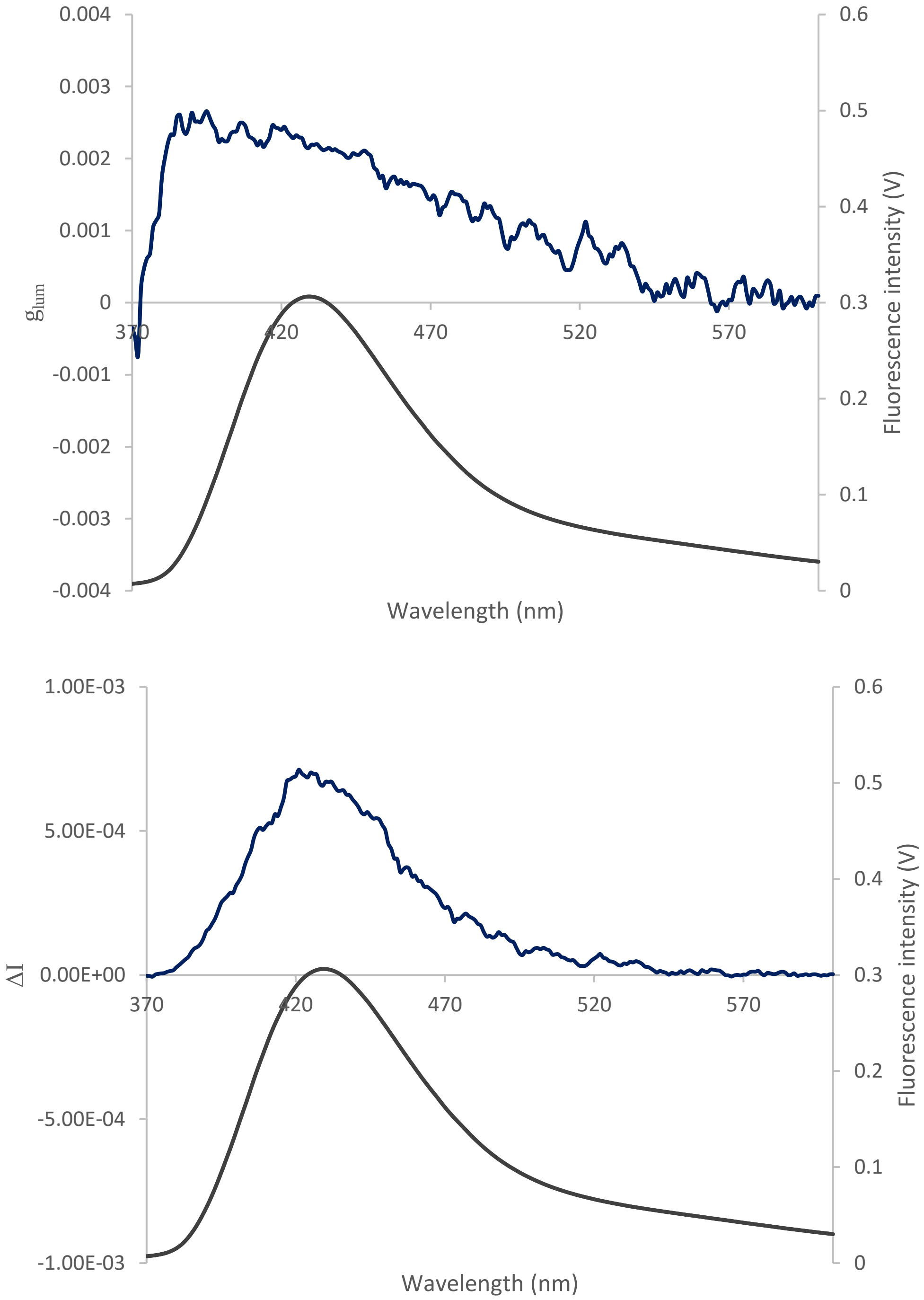

Fig. S45. g_{lum} = f (λ) (top) and CPL spectra (bottom) in dichloromethane for **2q**. [c] = 1 x 10^{-5} M. λ_{exc} = 310 nm.

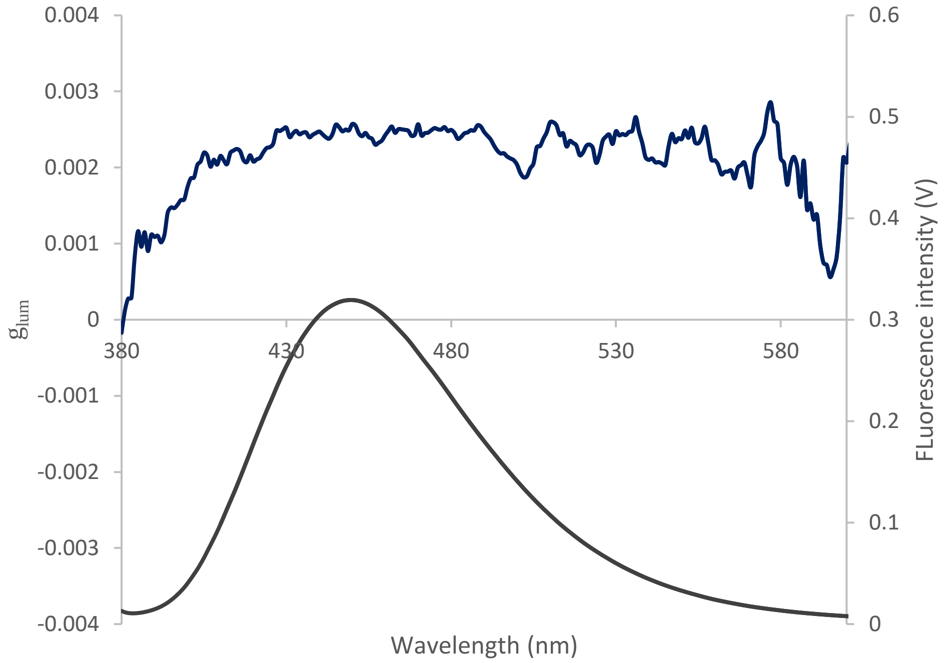

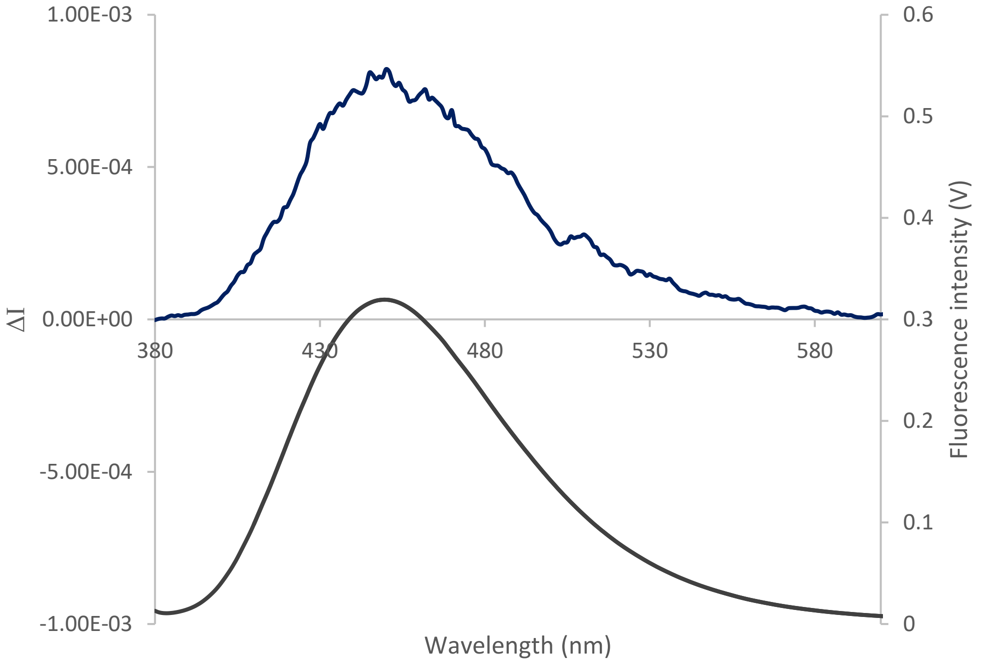

Fig. S46. g_{lum} = f (λ) (top) and CPL spectra (bottom) in dichloromethane for **2s**. [c] = 1 x 10^{-5} M. λ_{exc} = 320 nm.

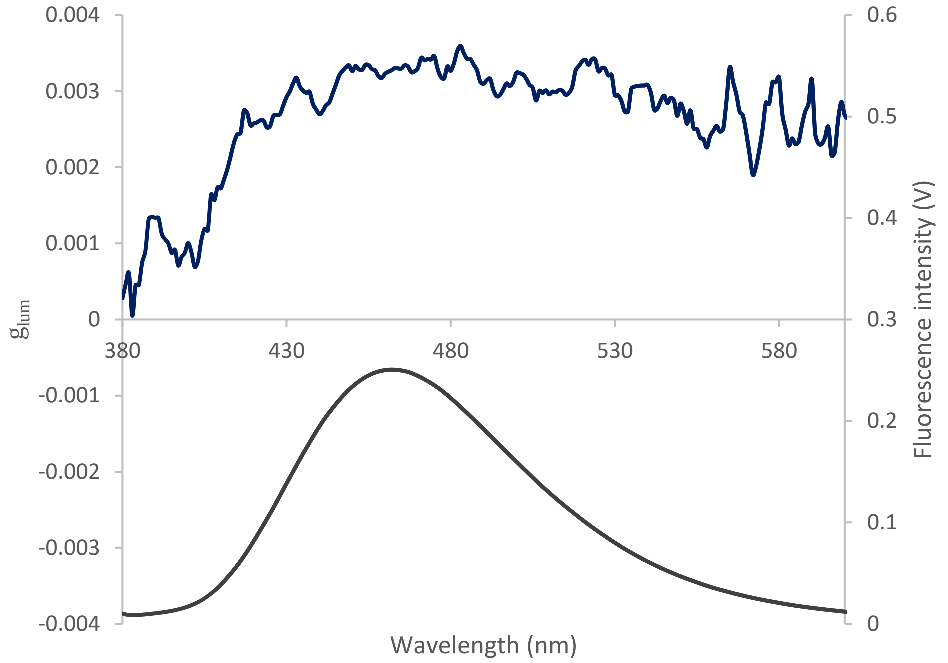

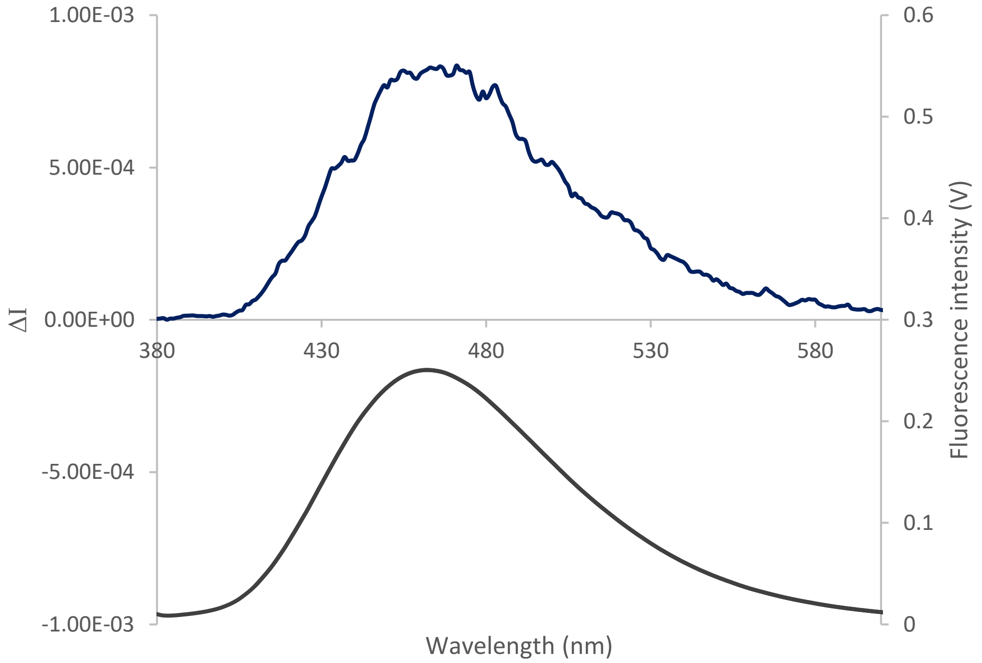

Fig. S47. g_{lum} = f (λ) (top) and CPL spectra (bottom) in dichloromethane for **2t**. [c] = 1 x 10^{-5} M. λ_{exc} = 320 nm.

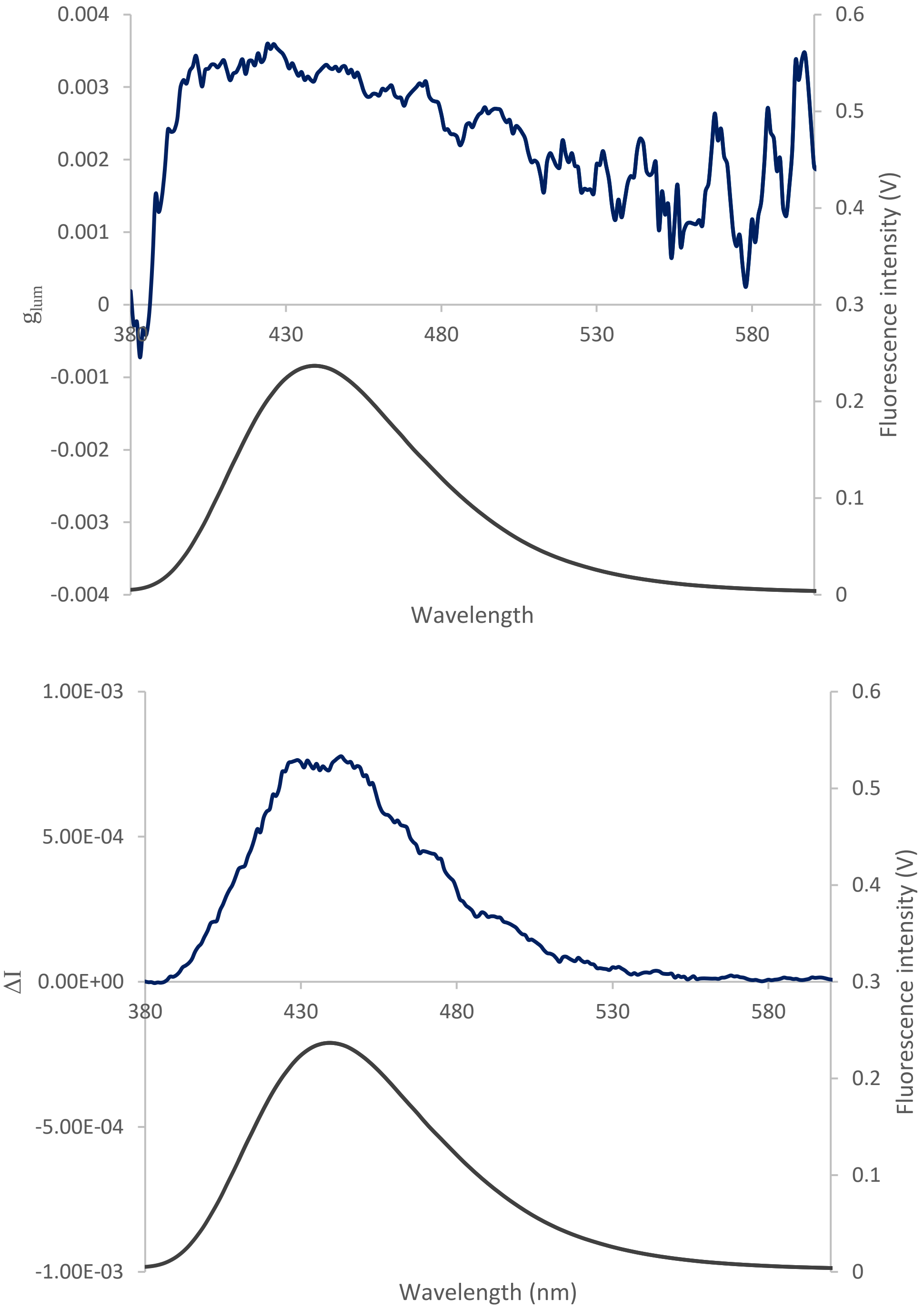

Fig. S48. g_{lum} = f (λ) (top) and CPL spectra (bottom) in dichloromethane for **2u**. [c] = 1 x 10^{-5} M. λ_{exc} = 310 nm.

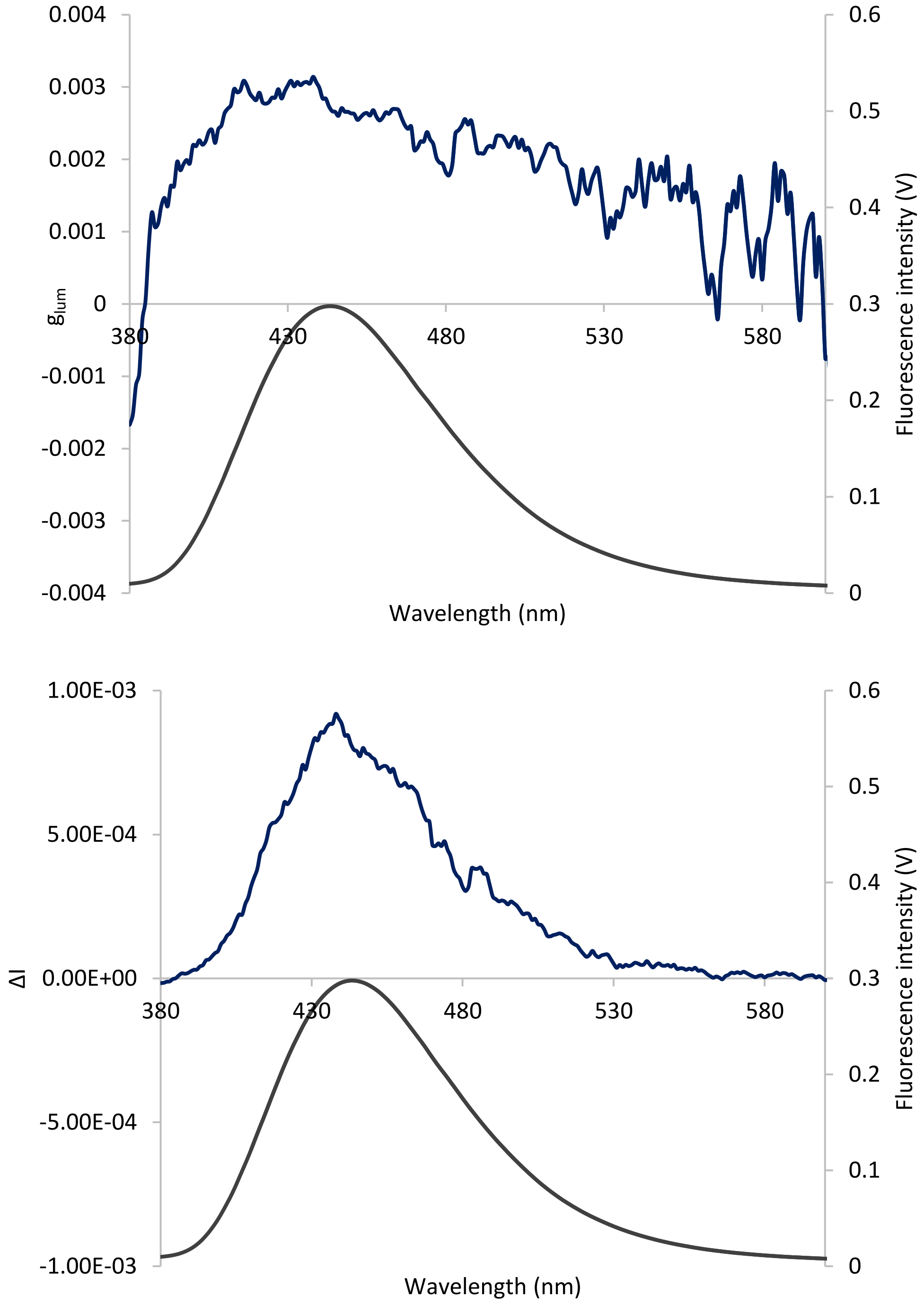

Fig. S49. g_{lum} = f (λ) (top) and CPL spectra (bottom) in dichloromethane for **2v**. [c] = 1 x 10^{-5} M. λ_{exc} = 320 nm.

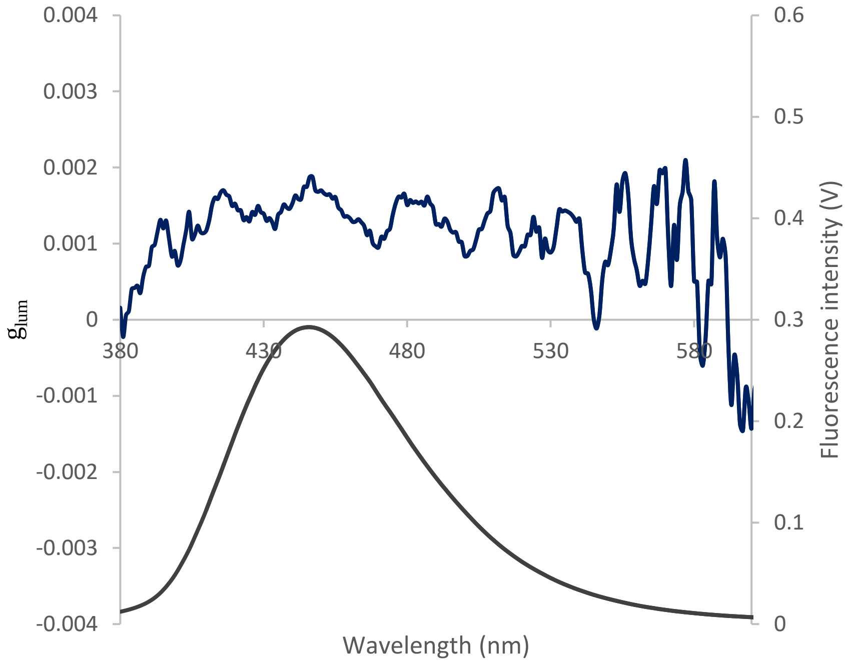

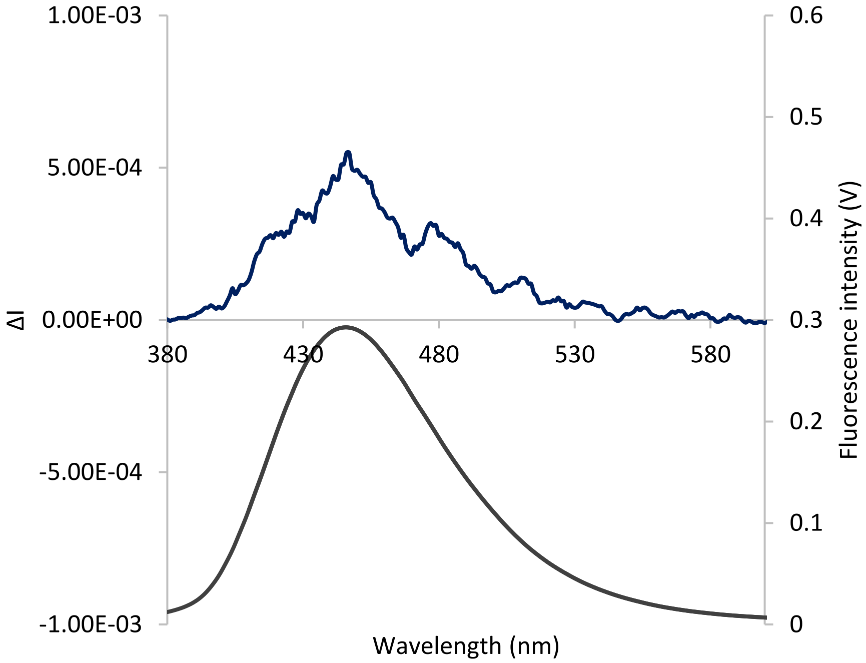

Fig. S50. g_{lum} = f (λ) (top) and CPL spectra (bottom) in dichloromethane for **2w**. [c] = 1 x 10^{-5} M. λ_{exc} = 320 nm.

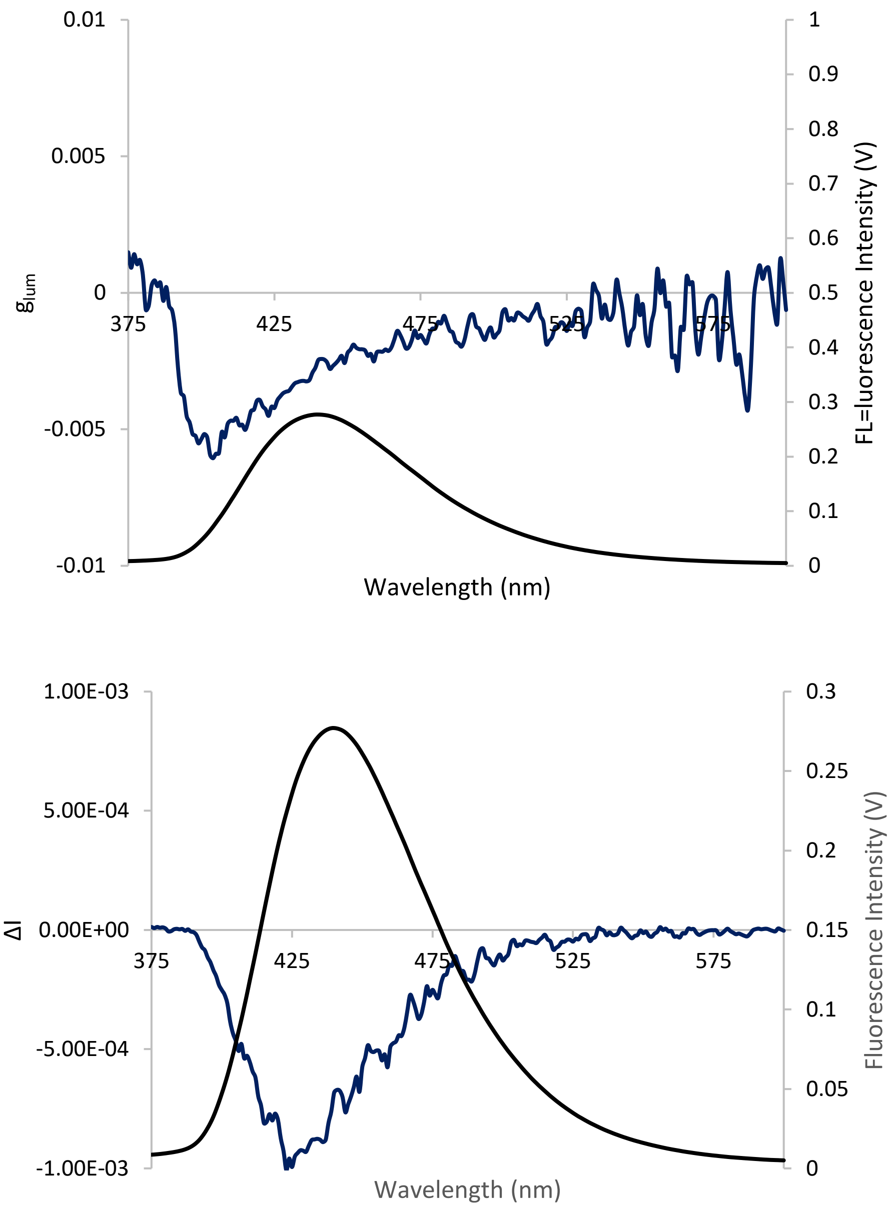

Fig. S51. g_{lum} = f (λ) (top) and CPL spectra (bottom) in dichloromethane for **2x**. [c] = 1 x 10^{-5} M. λ_{exc} = 310 nm.

Circular dichroism spectra:

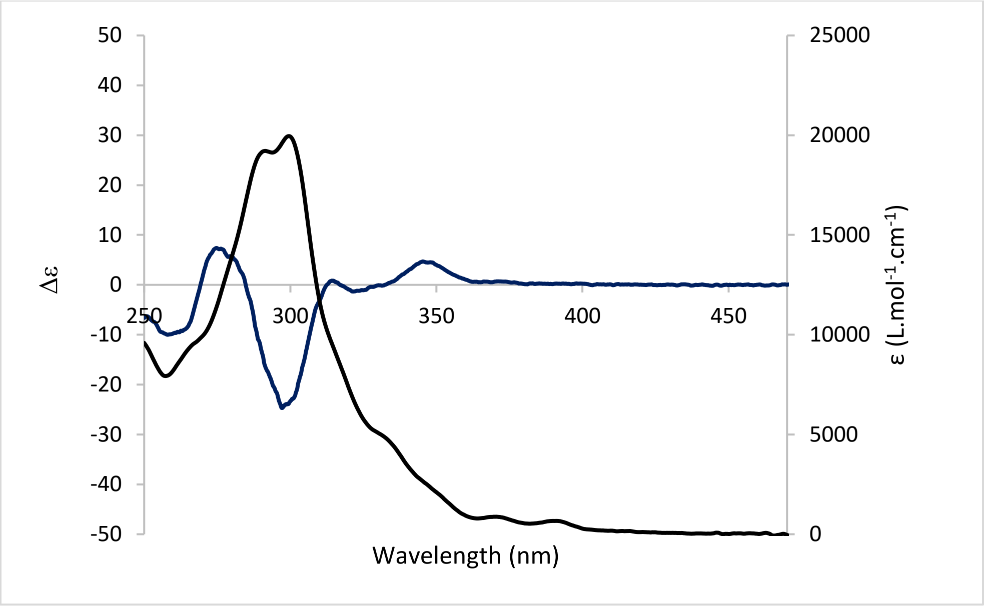

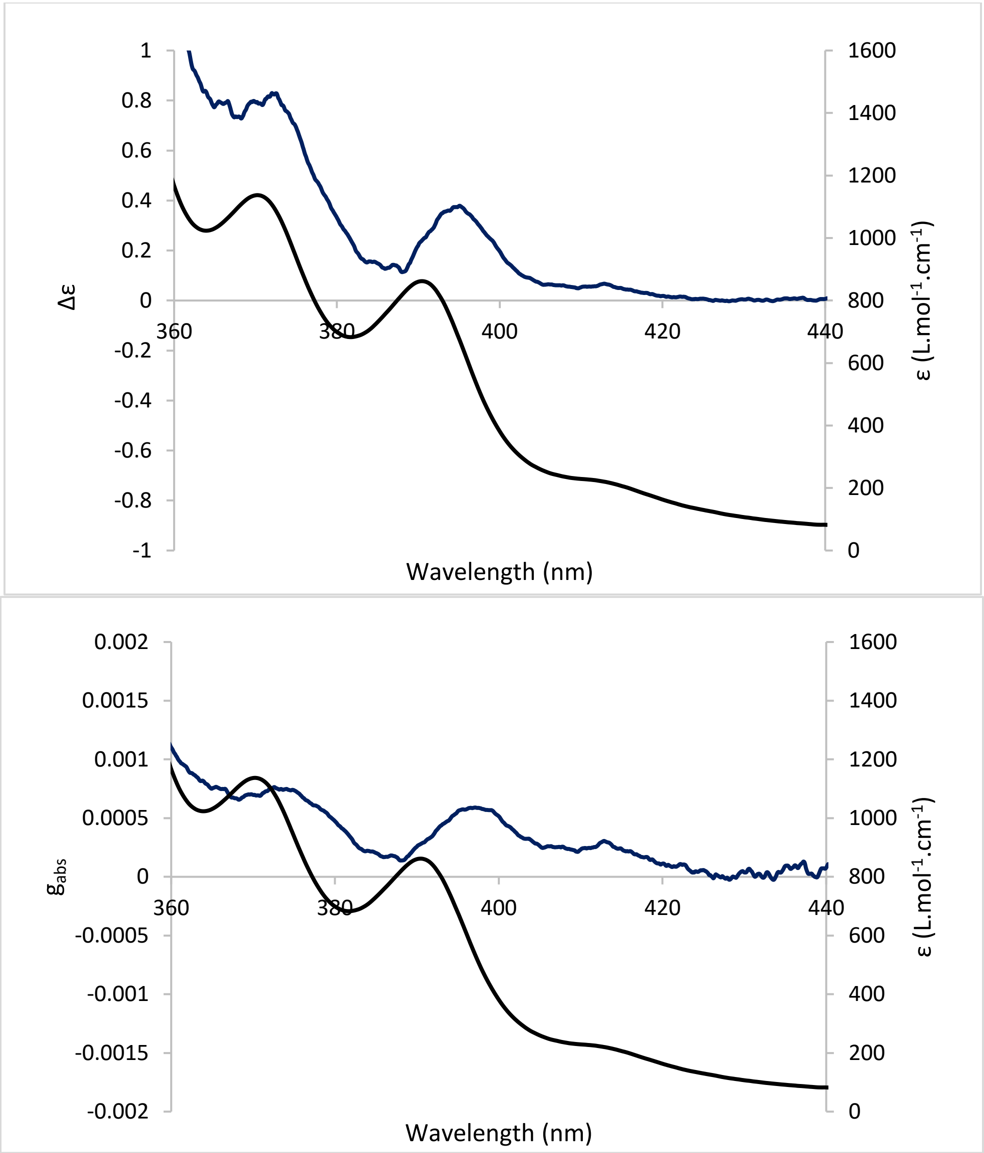

Fig. S52. $\Delta\varepsilon = f(\lambda)$ (top) and $g_{abs} = f(\lambda)$ (bottom) in dichloromethane for **2a**. 1 mm cell was used for the 250-500 nm region and 1 cm cell for the 360-440 nm region.

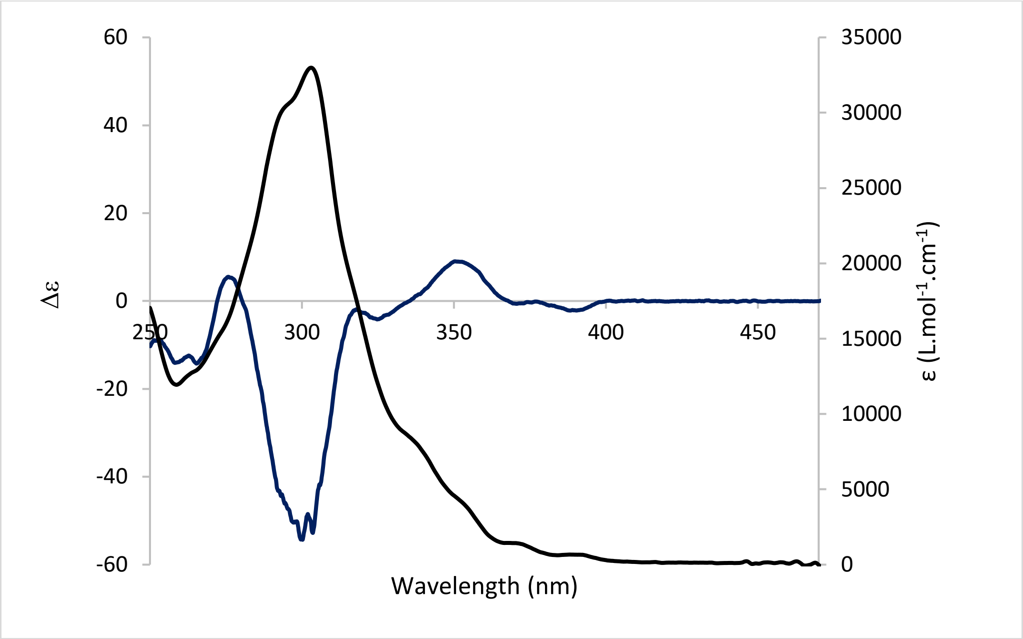

60
40
20
0
-20
-40
-60
Δε
250
300
350
400
450
35000
30000
25000
20000
15000
10000
5000
0
ε (L.mol-1.cm-1)
Wavelength (nm)

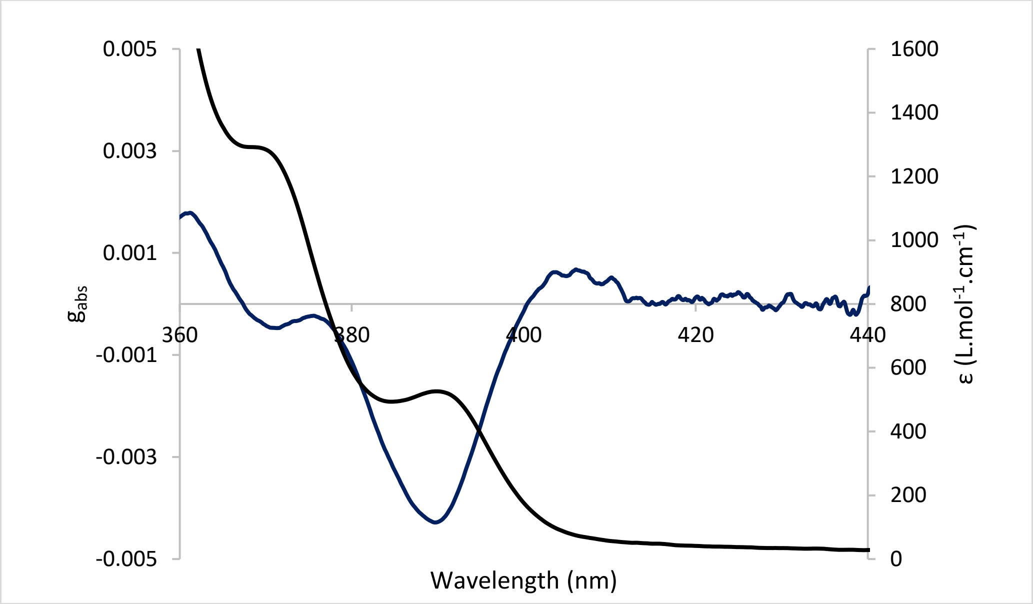

0.005
0.003
0.001
-0.001
-0.003
-0.005
gabs
360
380
400
420
440
1600
1400
1200
1000
800
600
400
200
0
ε (L.mol-1.cm-1)
Wavelength (nm)

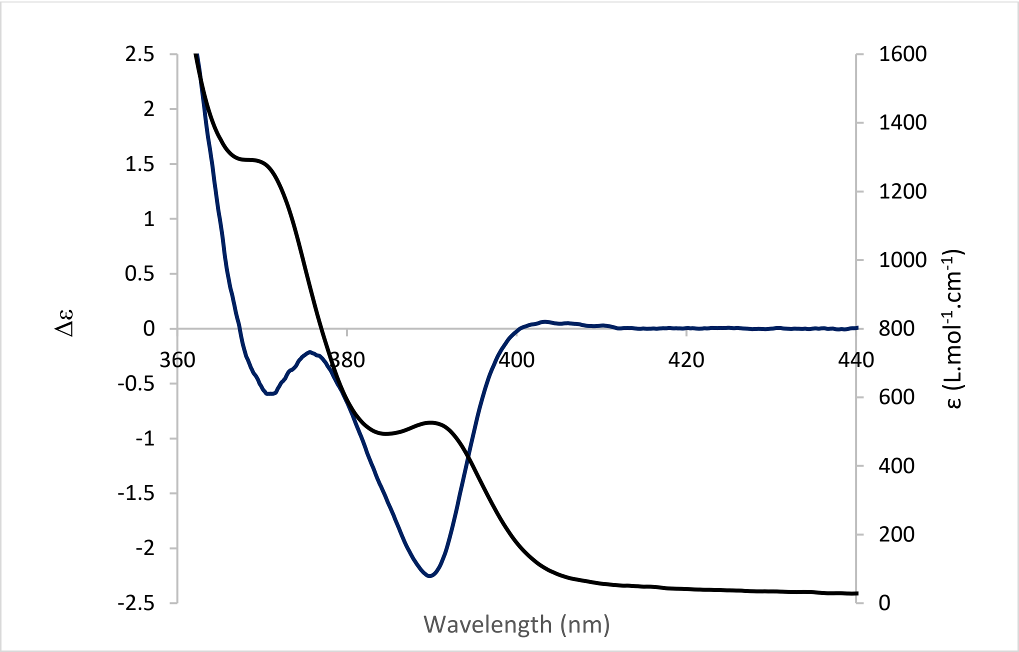

Fig. S53. Δε = f(λ) (top) and g_{abs} = f (λ) (bottom) in dichloromethane for **2b**. [c] = 1 x 10^{-3} M. 1 mm cell was used for the 250-500 nm region and 1 cm cell for the 360-440 nm region.

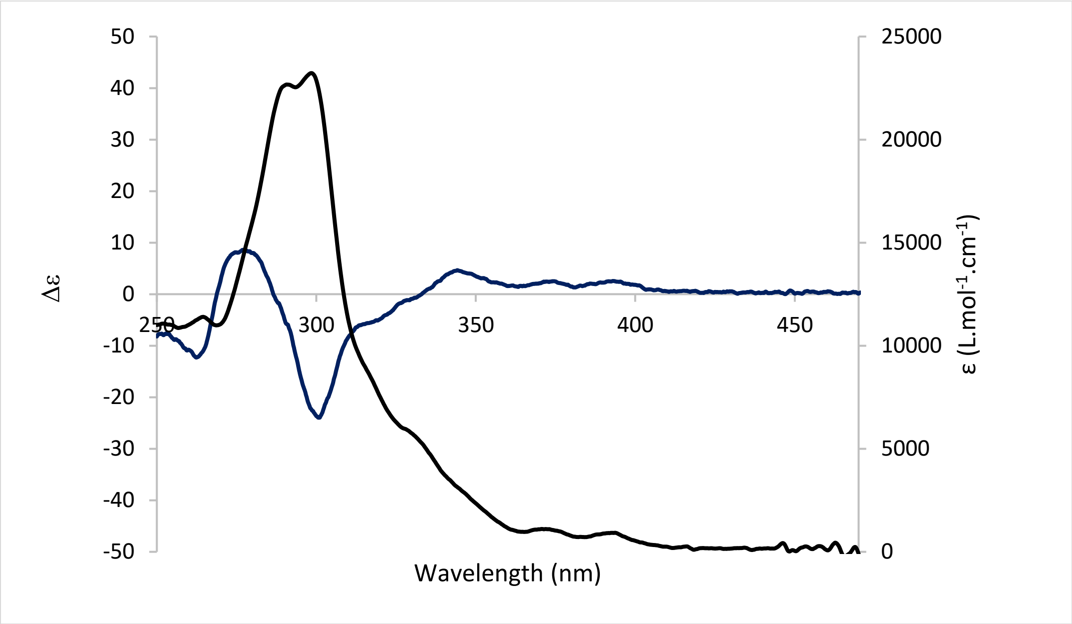

Δε
ε (L.mol-1.cm-1)
Wavelength (nm)
50
40
30
20
10
0
-10
-20
-30
-40
-50
250
300
350
400
450
25000
20000
15000
10000
5000
0

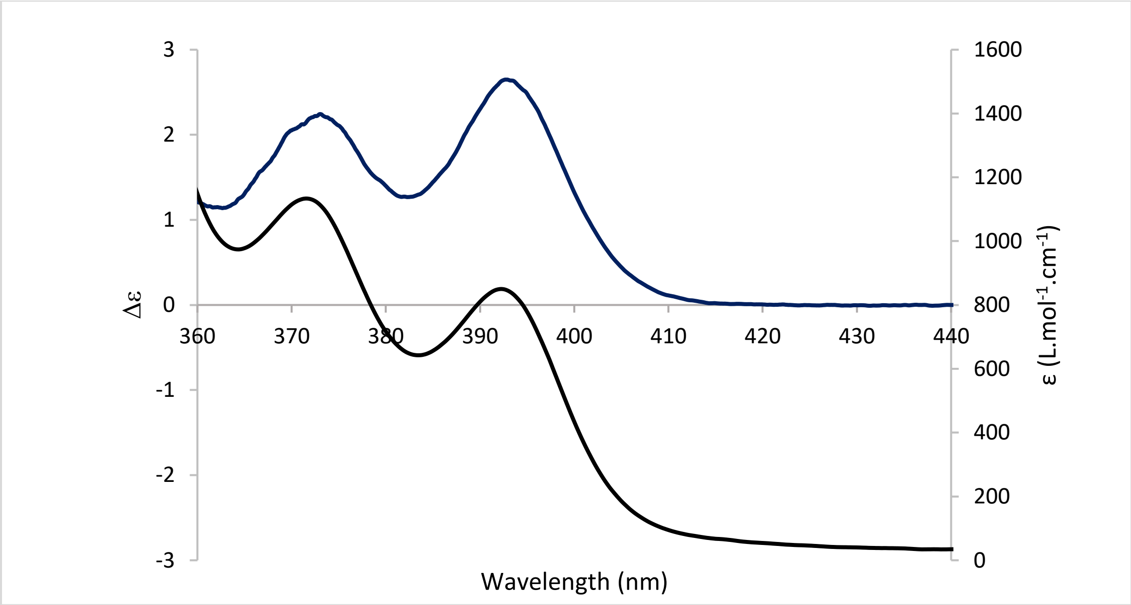

Δε
ε (L.mol-1.cm-1)
Wavelength (nm)
3
2
1
0
-1
-2
-3
360
370
380
390
400
410
420
430
440
1600
1400
1200
1000
800
600
400
200
0

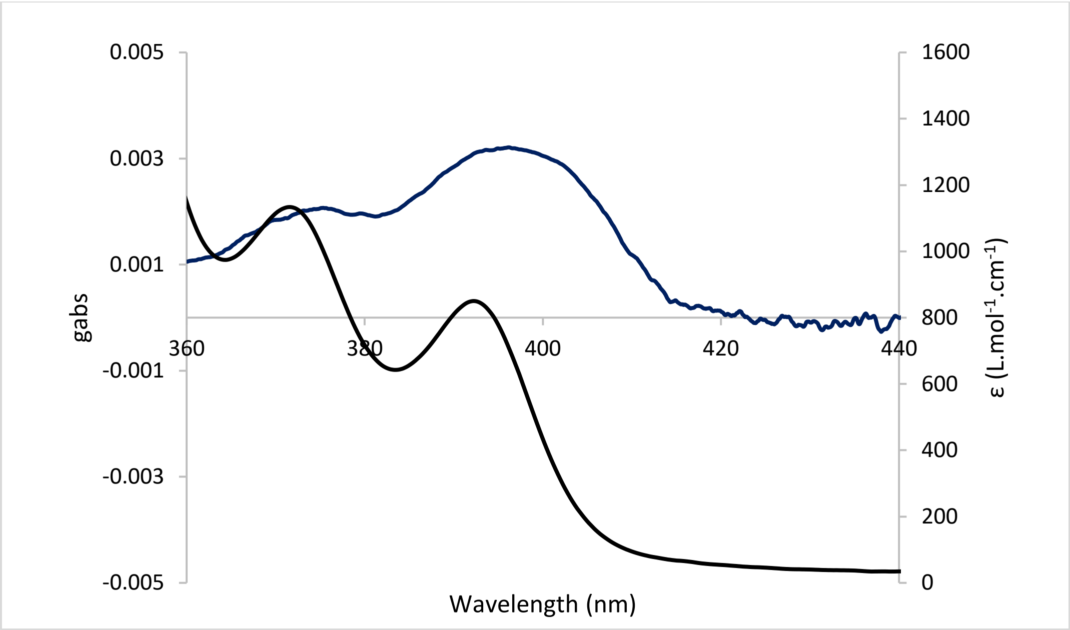

Fig. S54. Δε = f(λ) (top) and g_{abs} = f (λ) (bottom) in dichloromethane for **2c**. [c] = 1 x 10^{-3} M.. 1 mm cell was used for the 250-500 nm region and 1 cm cell for the 360-440 nm region.

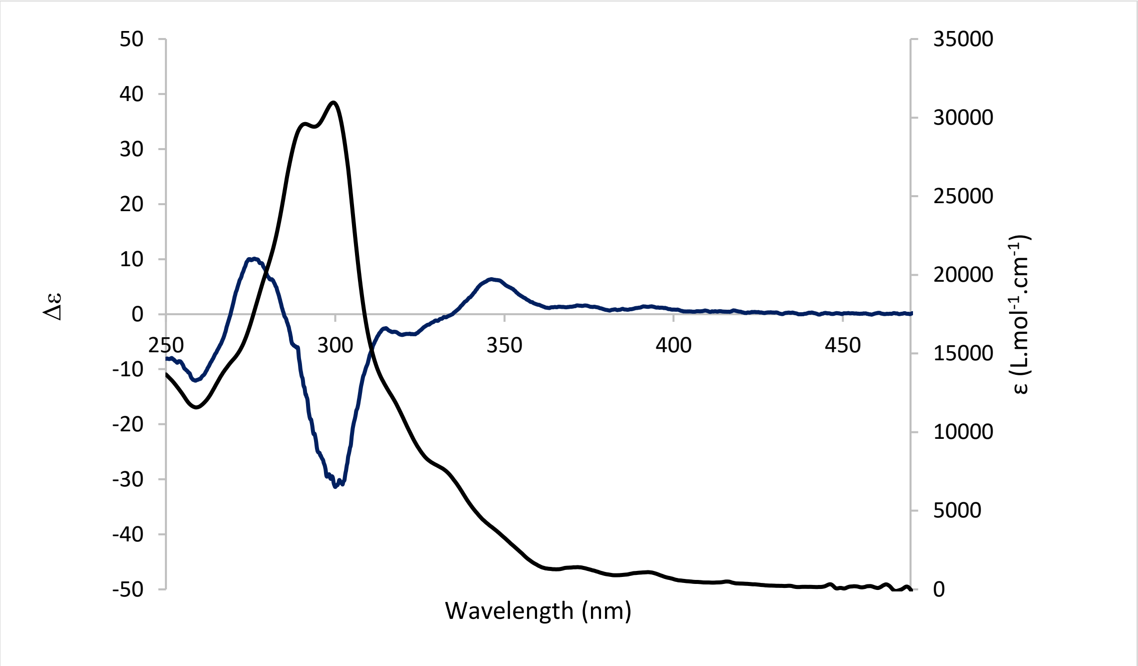
50
40
30
20
10
0
-10
-20
-30
-40
-50
Δε
250
300
350
400
450
35000
30000
25000
20000
15000
10000
5000
0
ε (L.mol-1.cm-1)
Wavelength (nm)

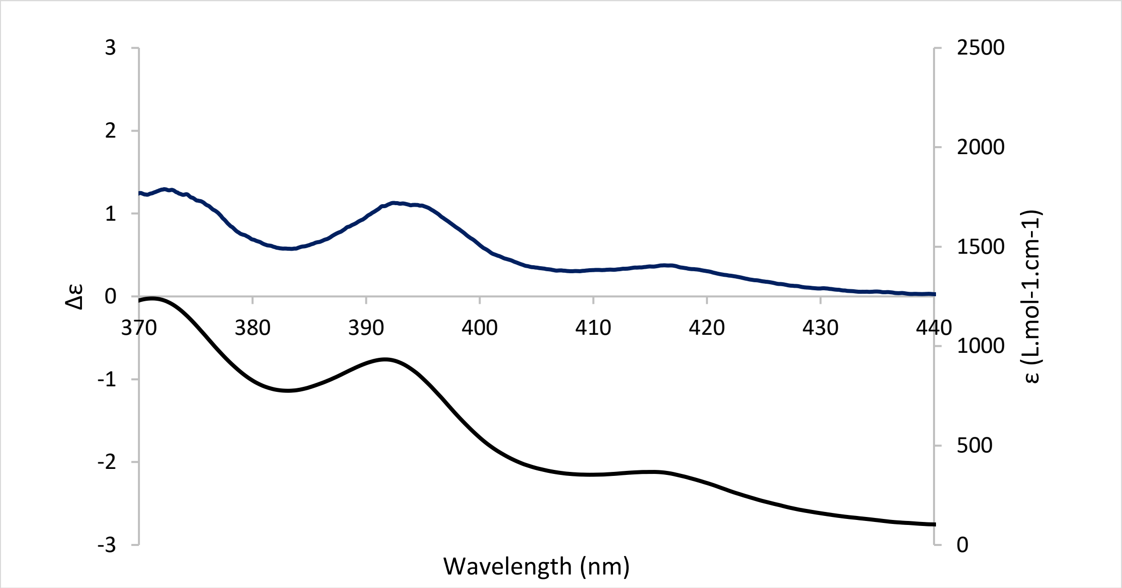
3
2
1
0
-1
-2
-3
Δε
370
380
390
400
410
420
430
440
2500
2000
1500
1000
500
0
ε (L.mol-1.cm-1)
Wavelength (nm)

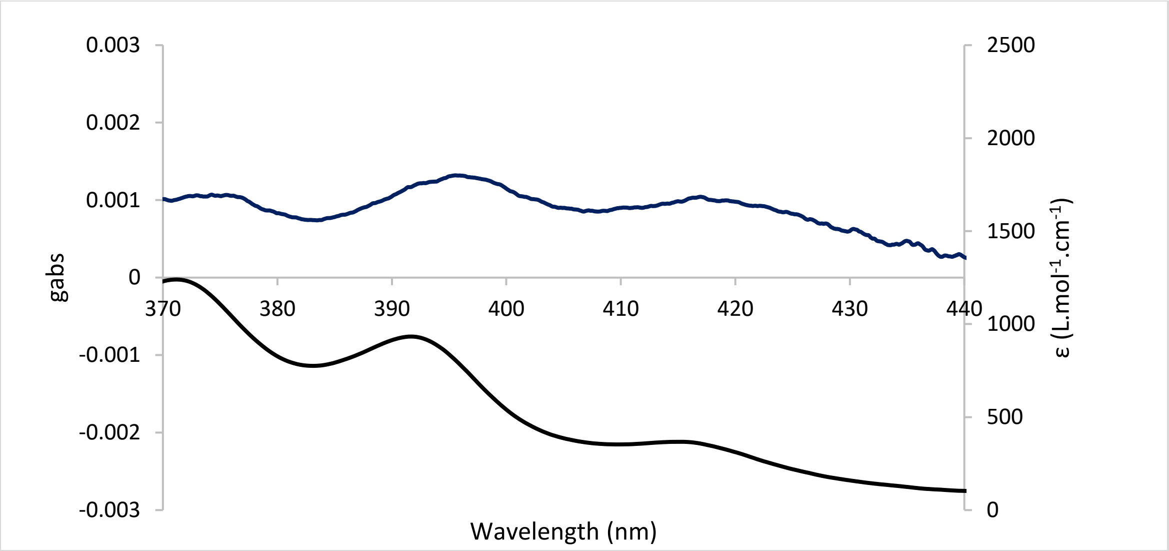

Fig. S55. Δε = f(λ) (top) and g_{abs} = f (λ) (bottom) in dichloromethane for **2d**. [c] = 1 x 10^{-3} M. 1 mm cell was used for the 250-500 nm region and 1 cm cell for the 360-440 nm region.

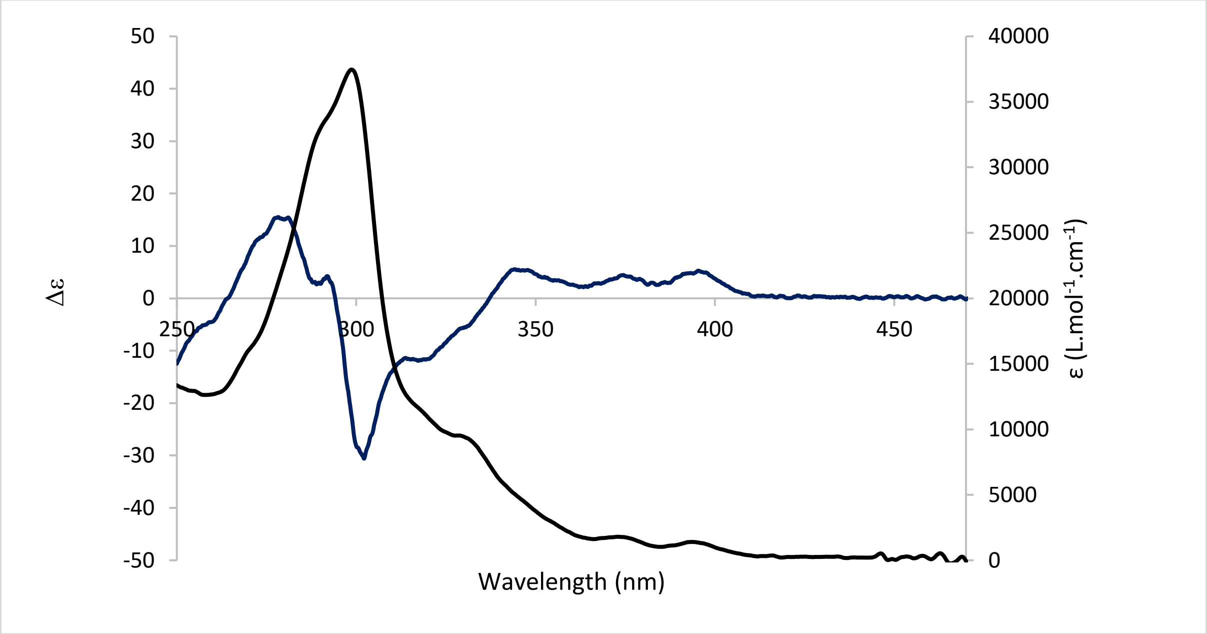

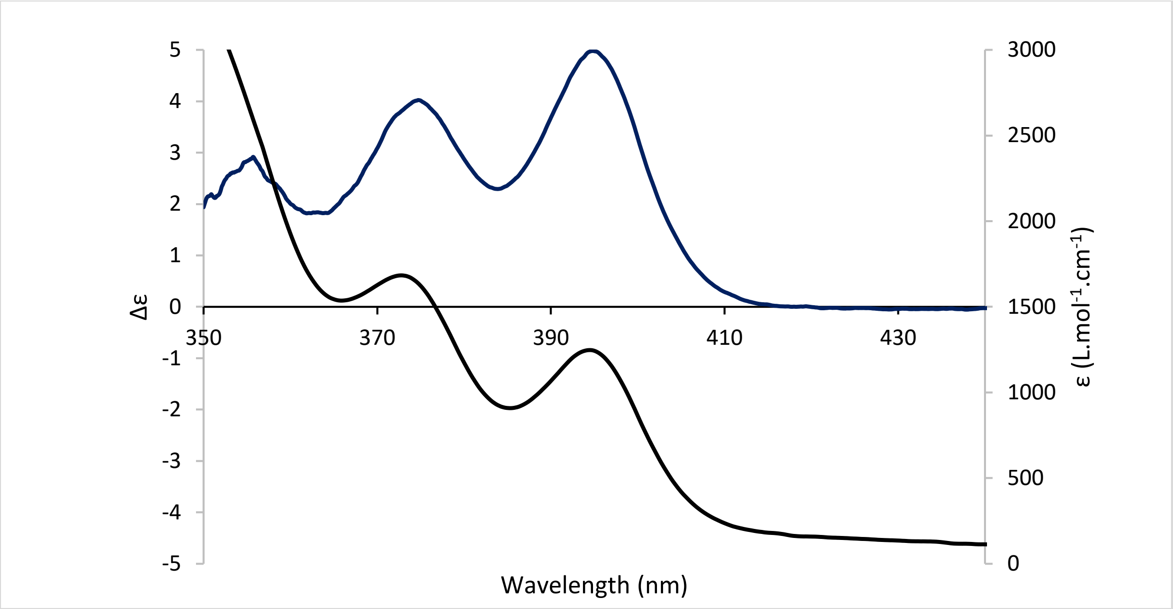

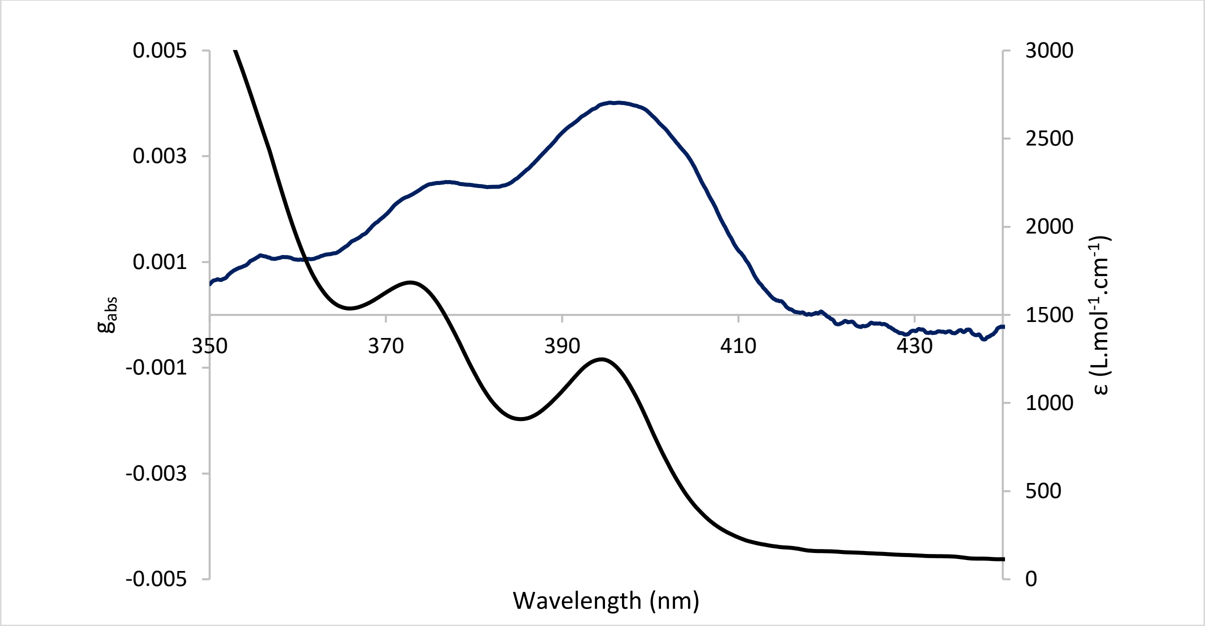

Fig. S56. $\Delta\varepsilon = f(\lambda)$ (top) and $g_{abs} = f(\lambda)$ (bottom) in dichloromethane for **2e**. [c] = 1 x 10^{-3} M. 1 mm cell were used for the 250-500 nm spectrum and 1 cm cuve for the 360-440 nm spectrum.

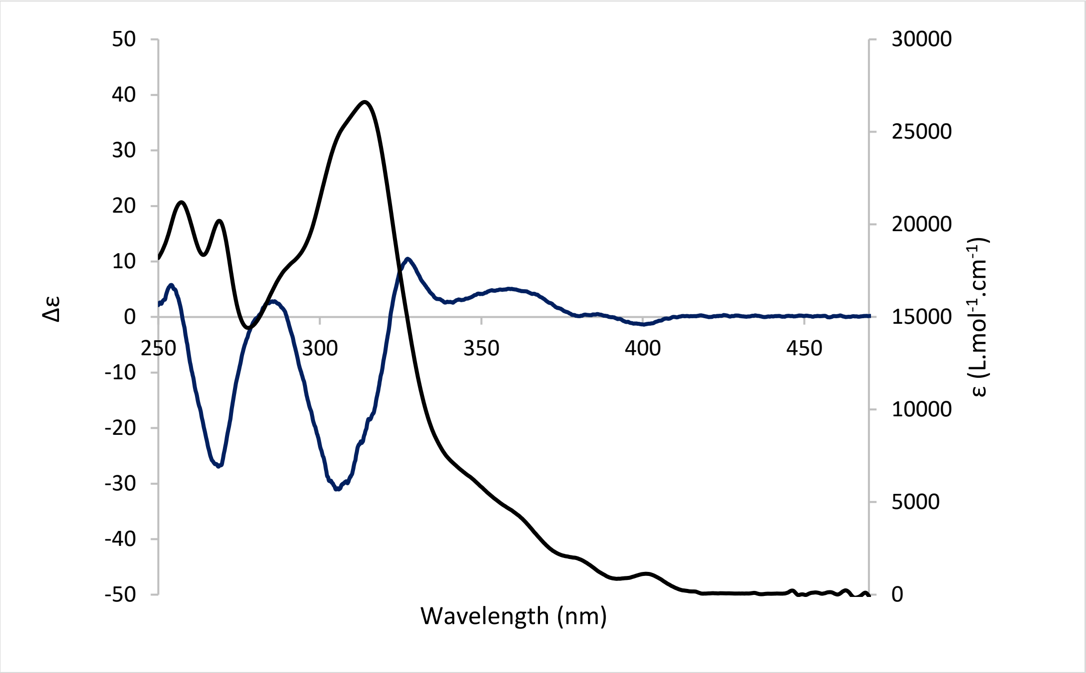
50
40
30
20
10
0
-10
-20
-30
-40
-50
Δε
250
300
350
400
450
30000
25000
20000
15000
10000
5000
0
ε (L.mol-1.cm-1)
Wavelength (nm)

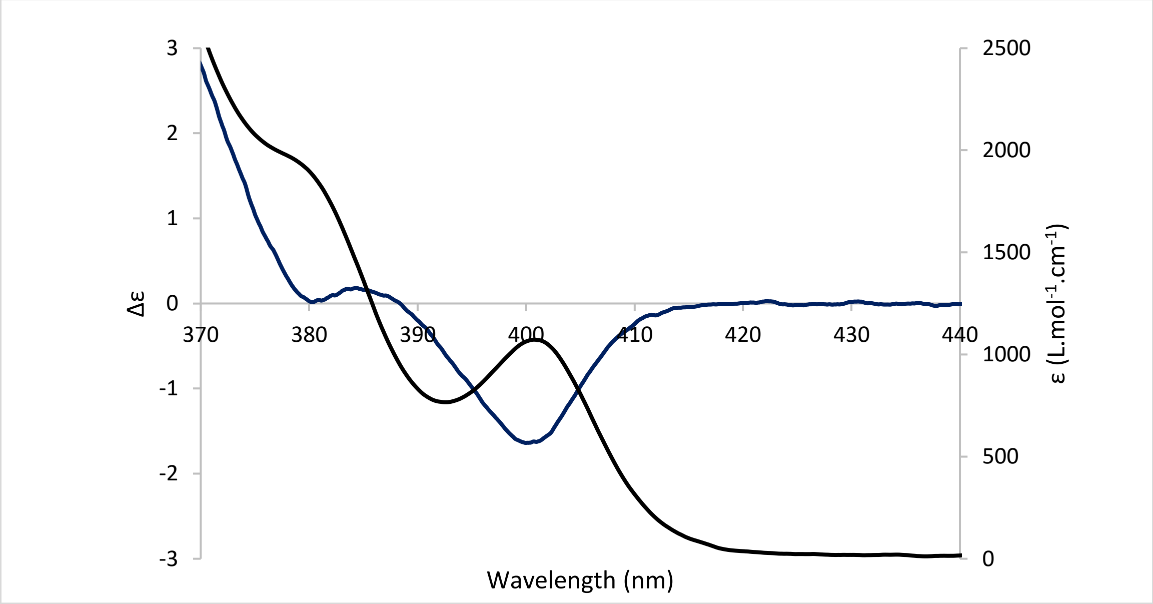
3
2
1
0
-1
-2
-3
Δε
370
380
390
400
410
420
430
440
2500
2000
1500
1000
500
0
ε (L.mol-1.cm-1)
Wavelength (nm)

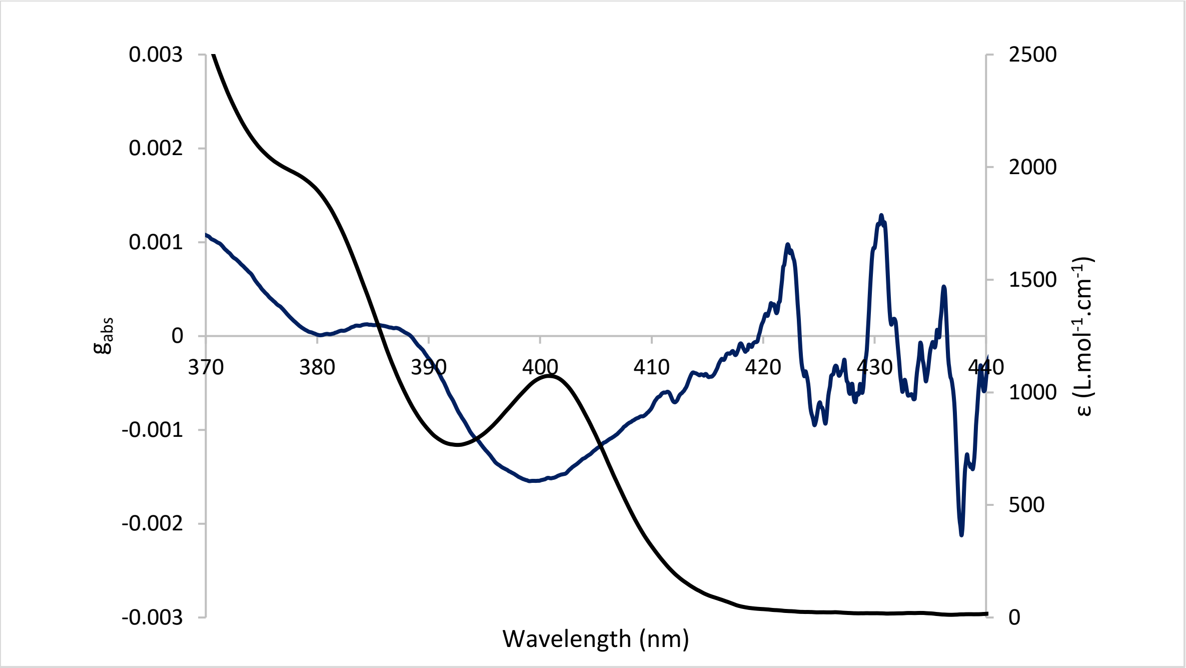

Fig. S57. Δε = f(λ) (top) and g_{abs} = f (λ) (bottom) in dichloromethane for **2f**. [c] = 1 x 10^{-3} M. 1 mm cell was used for the 250-500 nm region and 1 cm cell for the 360-440 nm region.

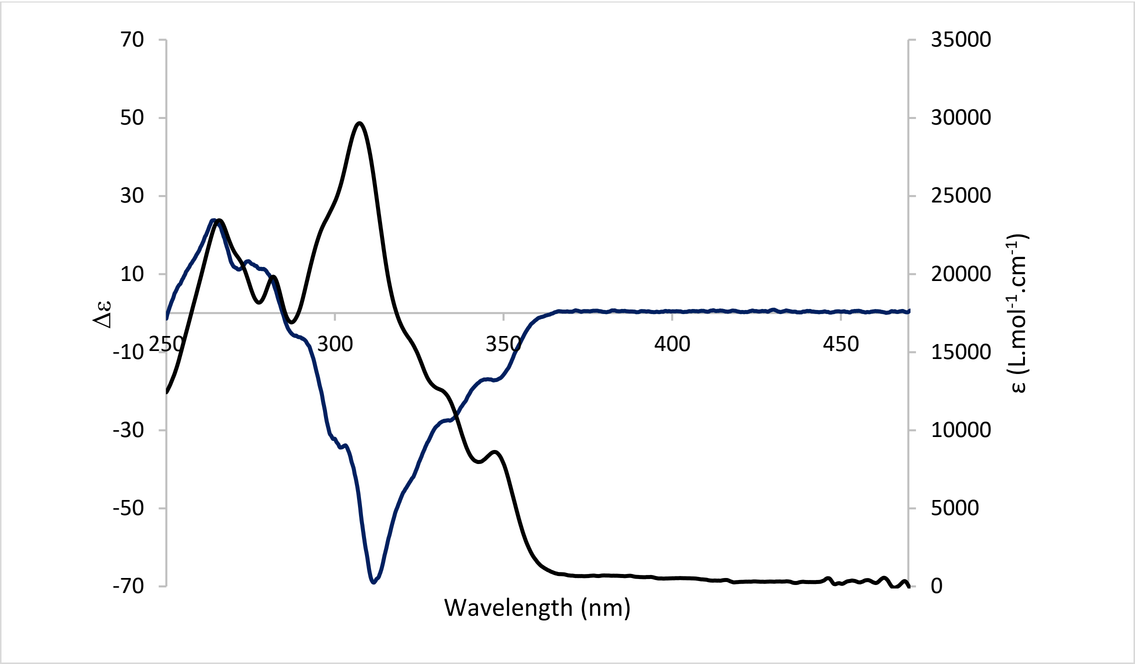

70
50
30
10
-10
-30
-50
-70
Δε
35000
30000
25000
20000
15000
10000
5000
0
ε (L.mol-1.cm-1)
250
300
350
400
450
Wavelength (nm)

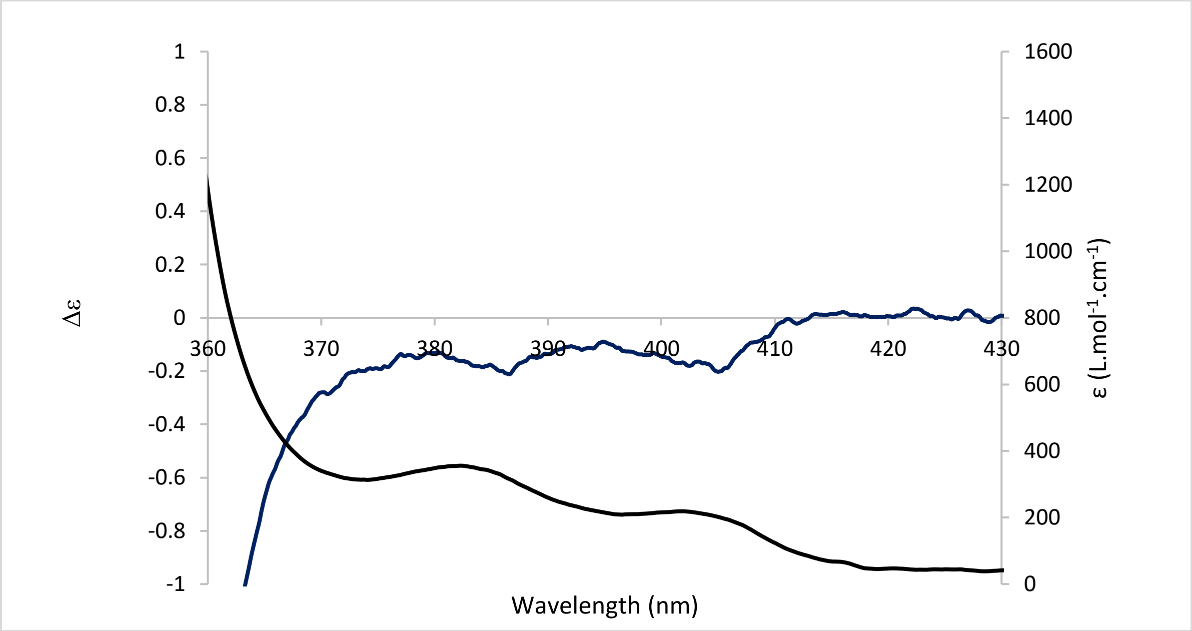

1
0.8
0.6
0.4
0.2
0
-0.2
-0.4
-0.6
-0.8
-1
Δε
1600
1400
1200
1000
800
600
400
200
0
ε (L.mol-1.cm-1)
360
370
380
390
400
410
420
430
Wavelength (nm)

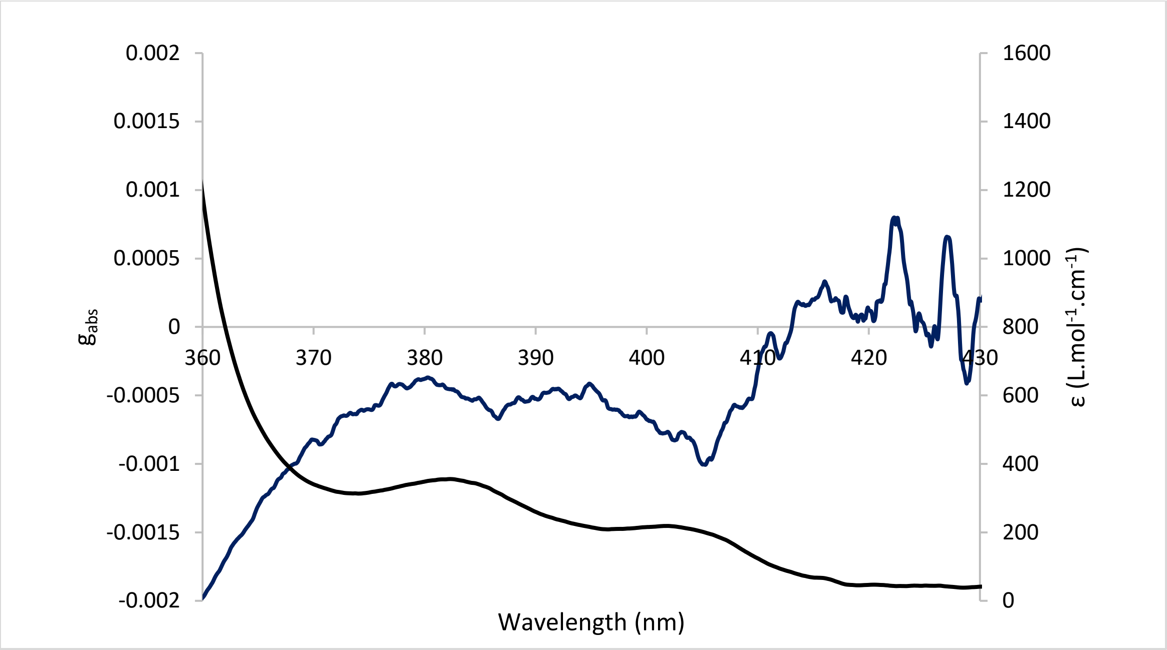

Fig. S58. $\Delta\varepsilon = f(\lambda)$ (top) and $g_{abs} = f(\lambda)$ (bottom) in dichloromethane for **2h**. [c] = 1 x 10^{-3} M. 1 mm cell was used for the 250-500 nm region and 1 cm cell for the 360-440 nm region.

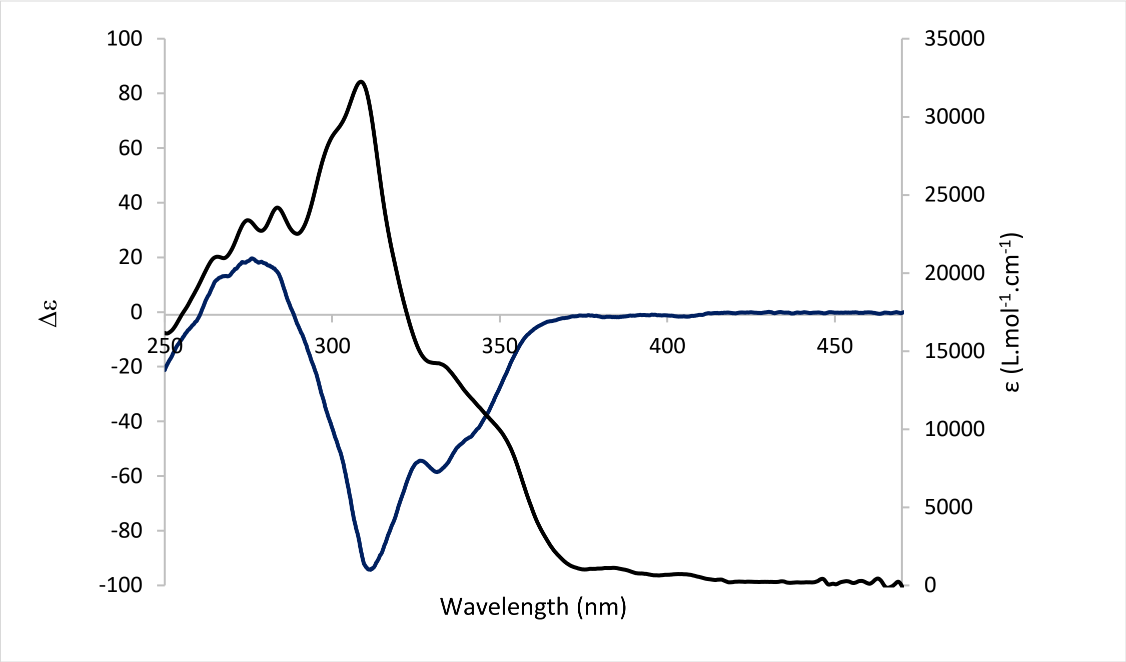

100
80
60
40
20
0
-20
-40
-60
-80
-100
Δε
250
300
350
400
450
35000
30000
25000
20000
15000
10000
5000
0
ε (L.mol-1.cm-1)
Wavelength (nm)

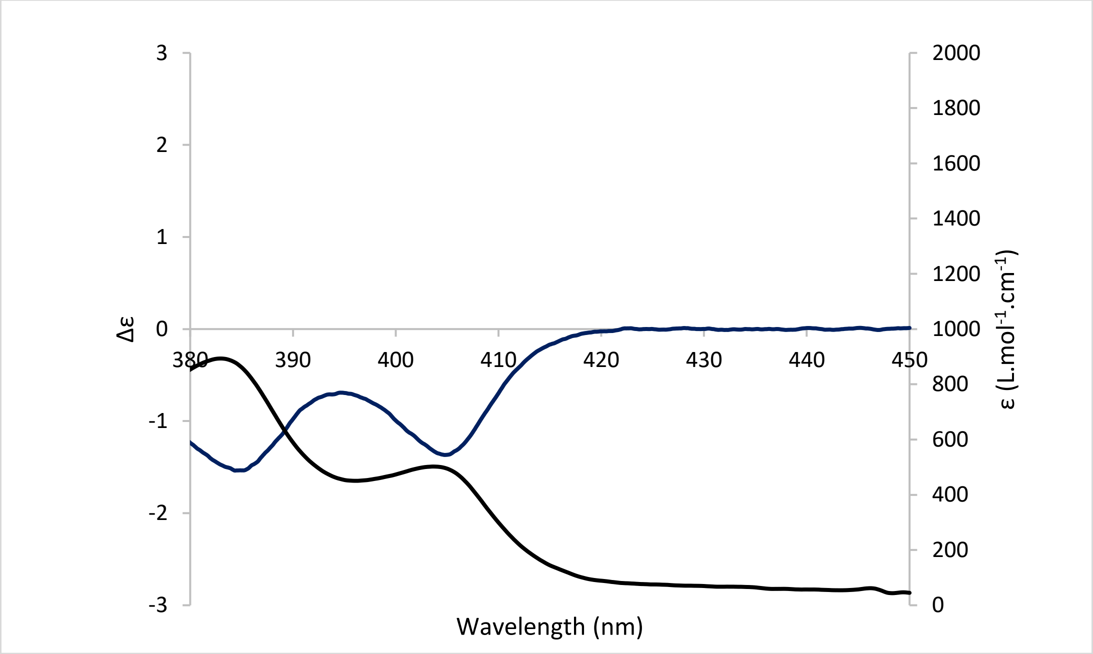

3
2
1
0
-1
-2
-3
Δε
380
390
400
410
420
430
440
450
2000
1800
1600
1400
1200
1000
800
600
400
200
0
ε (L.mol-1.cm-1)
Wavelength (nm)

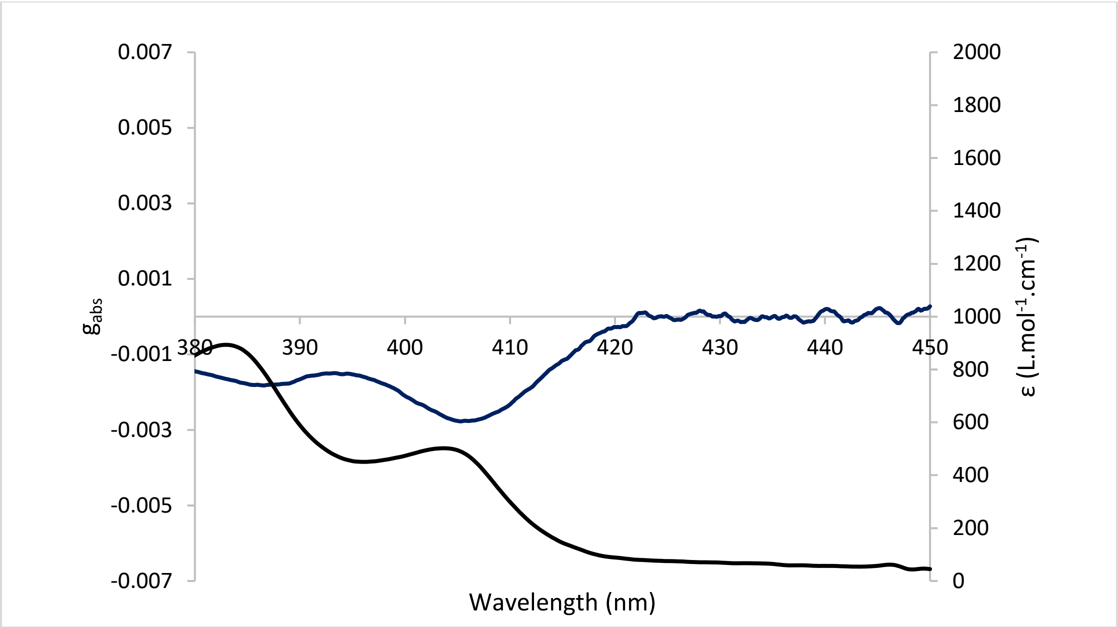

Fig. S59. Δε = f(λ) (top) and g_{abs} = f (λ) (bottom) in dichloromethane for **2i**. [c] = 1 x 10^{-3} M. 1 mm cell was used for the 250-500 nm region and 1 cm cell for the 360-440 nm region.

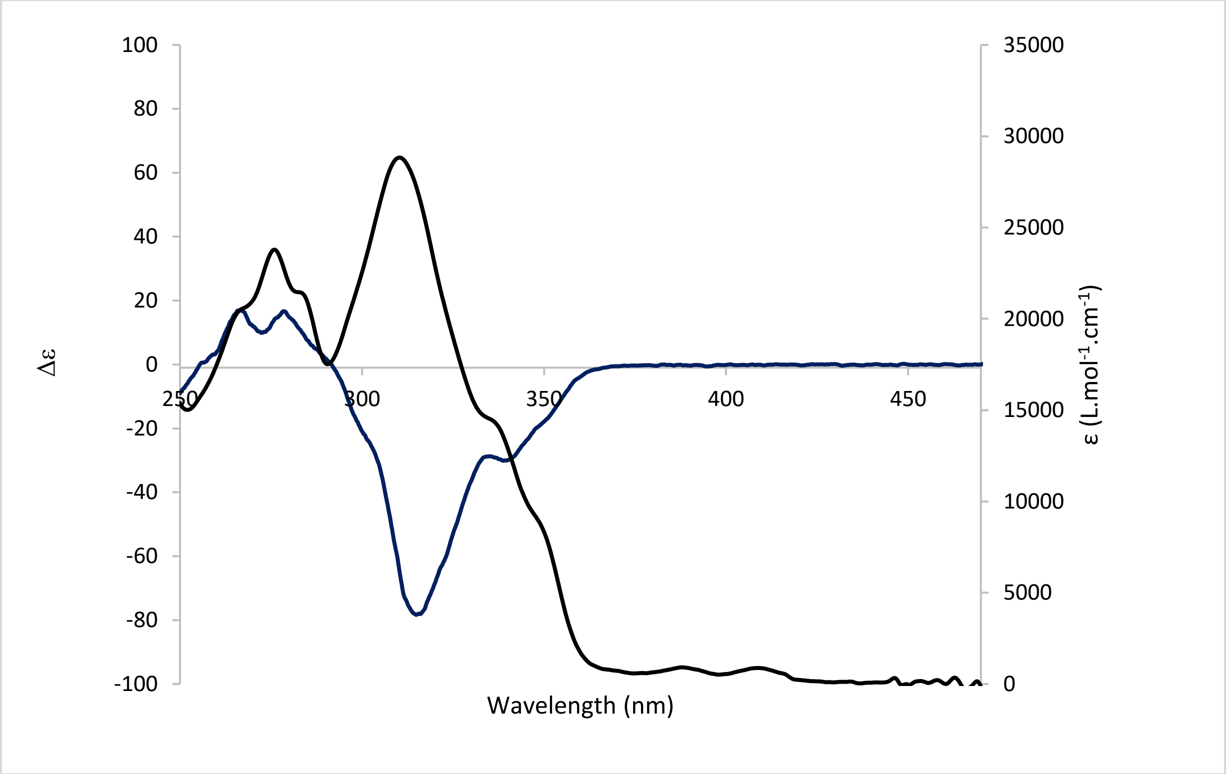

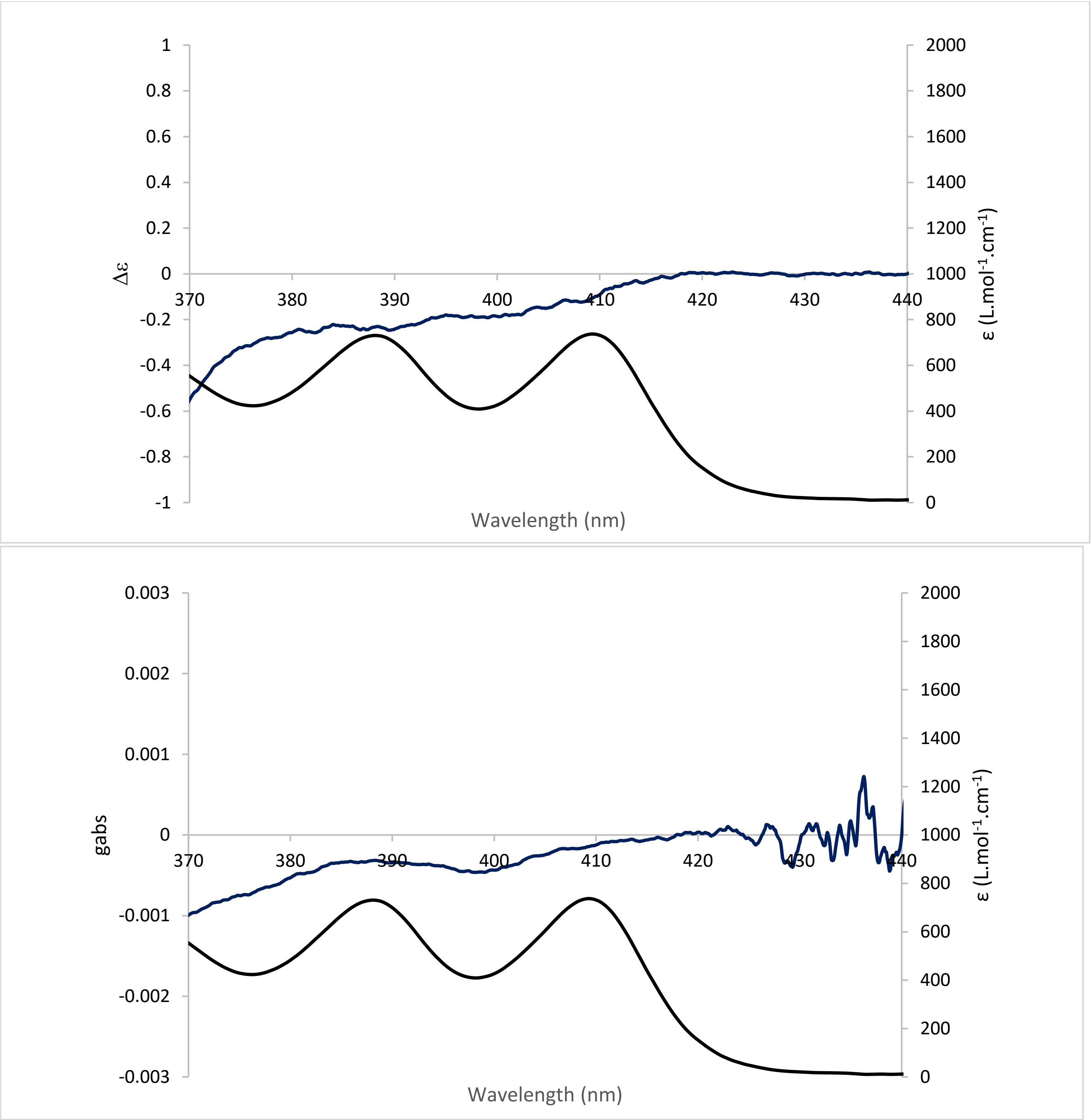

Fig. S60. $\Delta\varepsilon = f(\lambda)$ (top) and $g_{abs} = f(\lambda)$ (bottom) in dichloromethane for **2j**. [c] = 1 x 10^{-3} M. 1 mm cell was used for the 250-500 nm region and 1 cm cell for the 360-440 nm region.

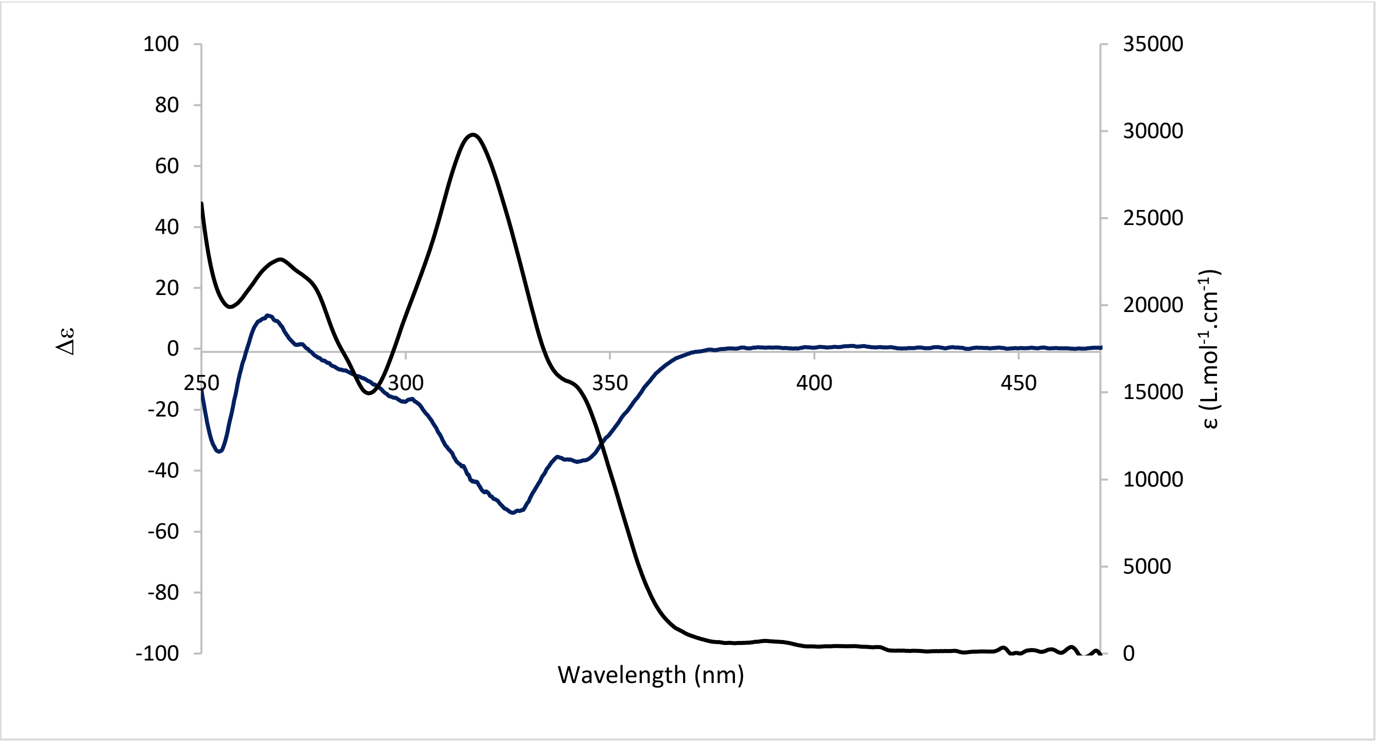

100
80
60
40
20
0
-20
-40
-60
-80
-100
Δε
250
300
350
400
450
Wavelength (nm)
35000
30000
25000
20000
15000
10000
5000
0
ε (L.mol-1.cm-1)

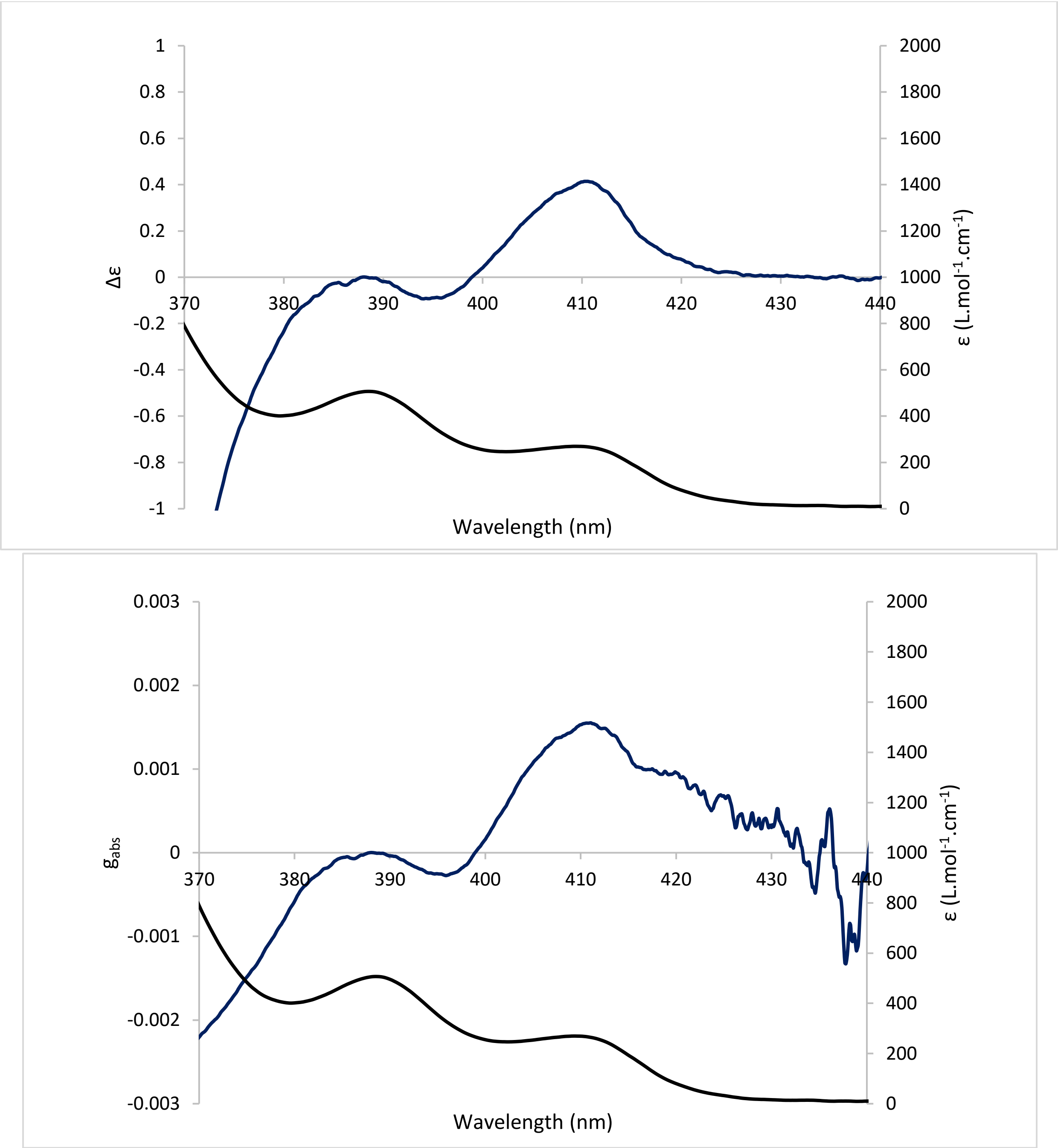

Fig. S61. $\Delta\varepsilon = f(\lambda)$ (top) and $g_{abs} = f(\lambda)$ (bottom) in dichloromethane for **2k**. [c] = 1 x 10^{-3} M. 1 mm cell was used for the 250-500 nm region and 1 cm cell for the 360-440 nm region.

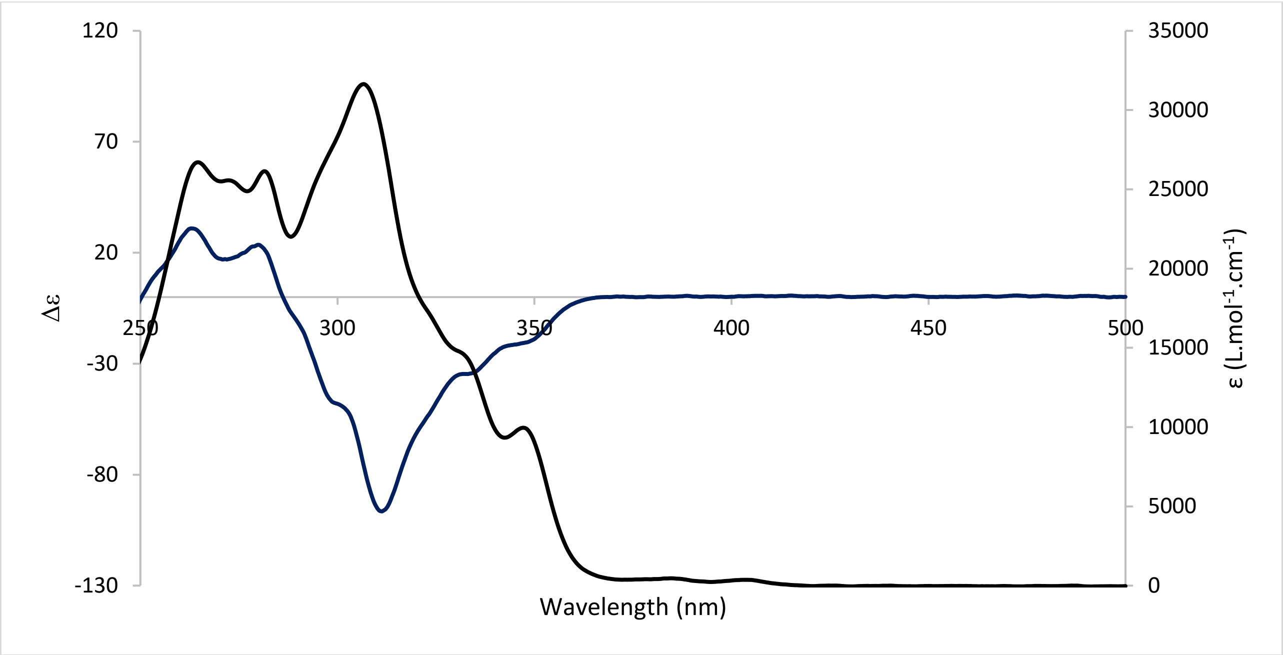
120
70
20
-30
-80
-130
Δε
250
300
350
400
450
500
Wavelength (nm)
35000
30000
25000
20000
15000
10000
5000
0
ε (L.mol-1.cm-1)

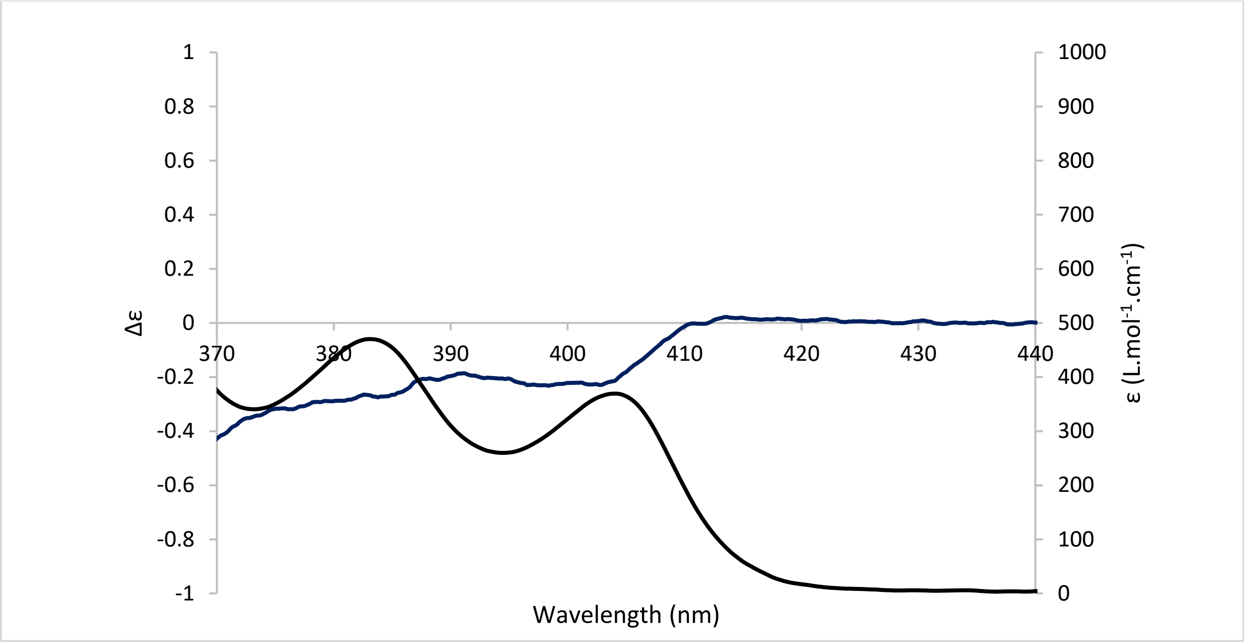

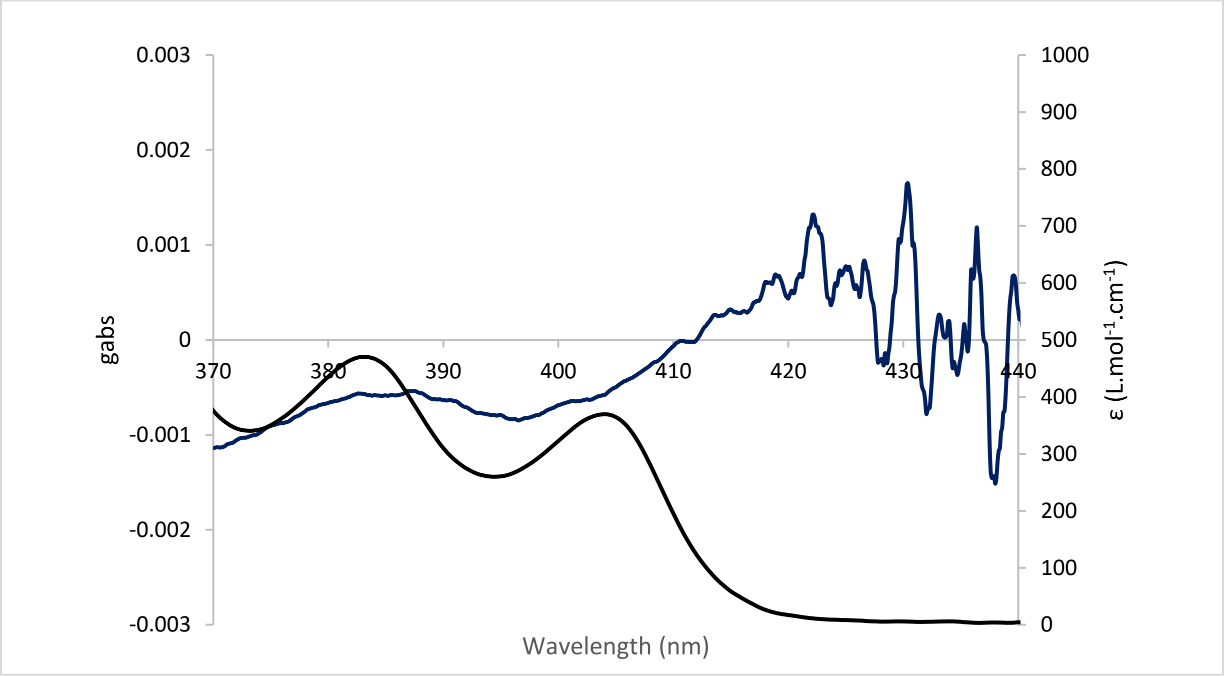

Fig. S62. Δε = f(λ) (top) and g_{abs} = f (λ) (bottom) in dichloromethane for **2l**. [c] = 1 x 10^{-3} M. 1 mm cell was used for the 250-500 nm region and 1 cm cell for the 360-440 nm region.

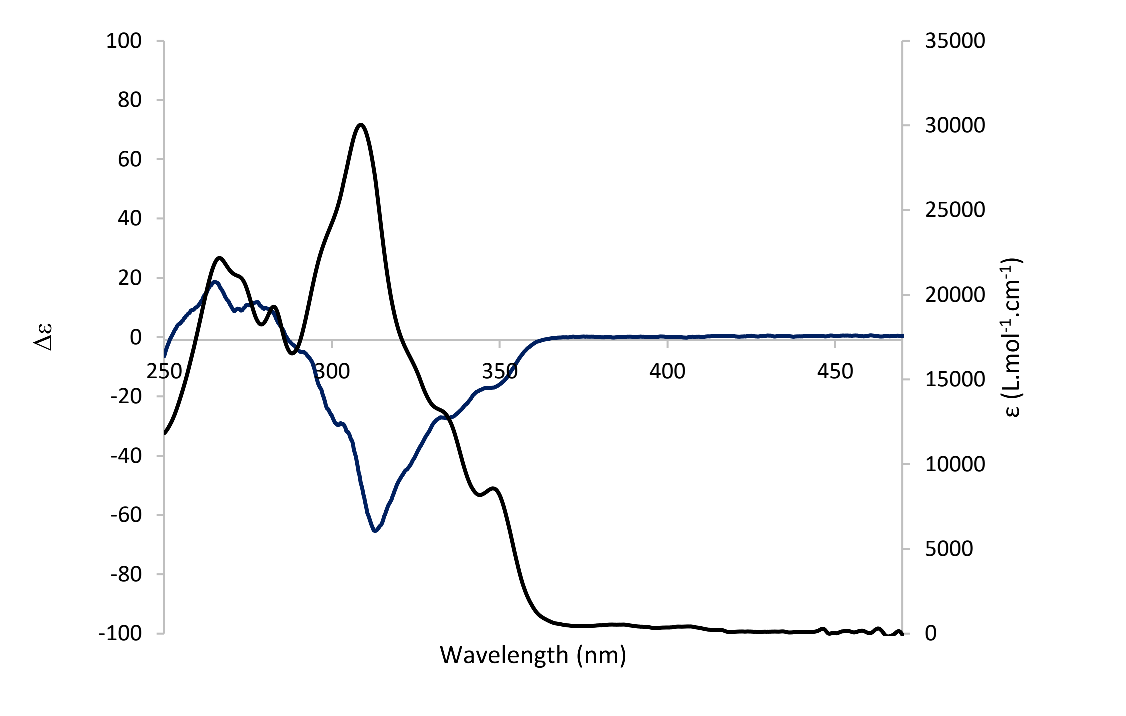

100
80
60
40
20
0
-20
-40
-60
-80
-100
Δε
250
300
350
400
450
35000
30000
25000
20000
15000
10000
5000
0
ε (L.mol-1.cm-1)
Wavelength (nm)

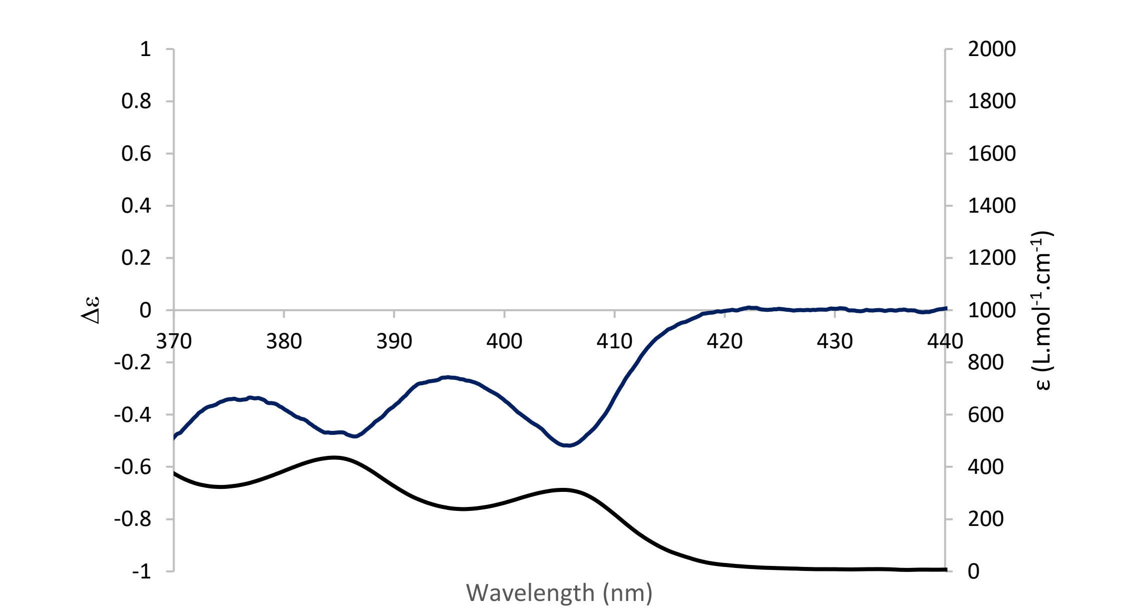

1
0.8
0.6
0.4
0.2
0
-0.2
-0.4
-0.6
-0.8
-1
Δε
370
380
390
400
410
420
430
440
2000
1800
1600
1400
1200
1000
800
600
400
200
0
ε (L.mol-1.cm-1)
Wavelength (nm)

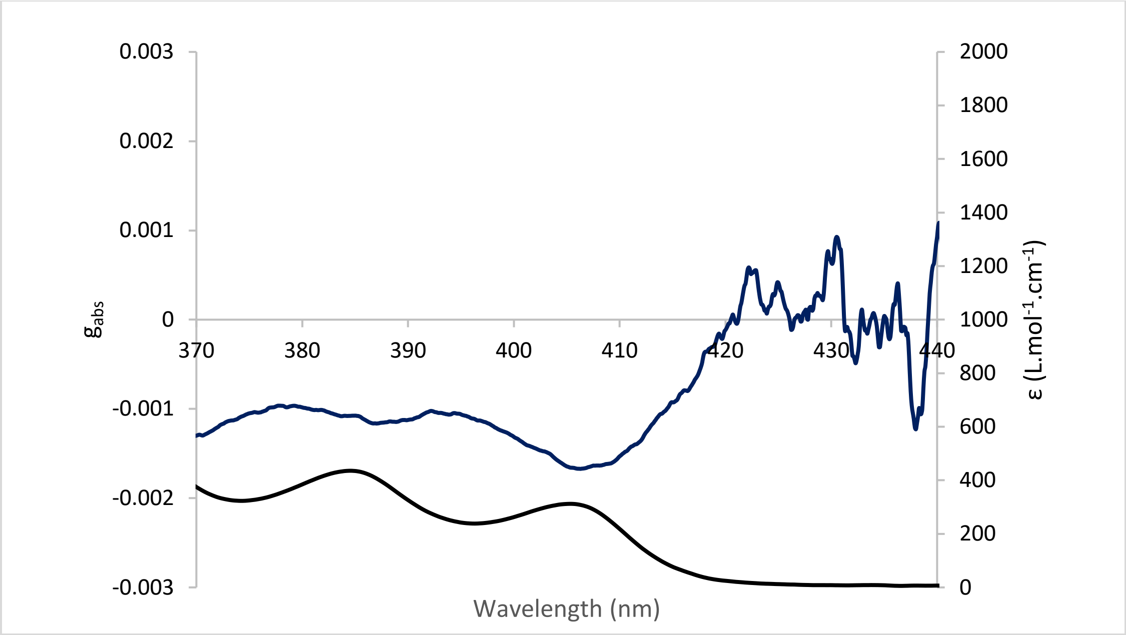

Fig. S63. $\Delta\varepsilon = f(\lambda)$ (top) and $g_{abs} = f(\lambda)$ (bottom) in dichloromethane for **2m**. [c] = 1 x 10^{-3} M. 1 mm cell was used for the 250-500 nm region and 1 cm cell for the 360-440 nm region.

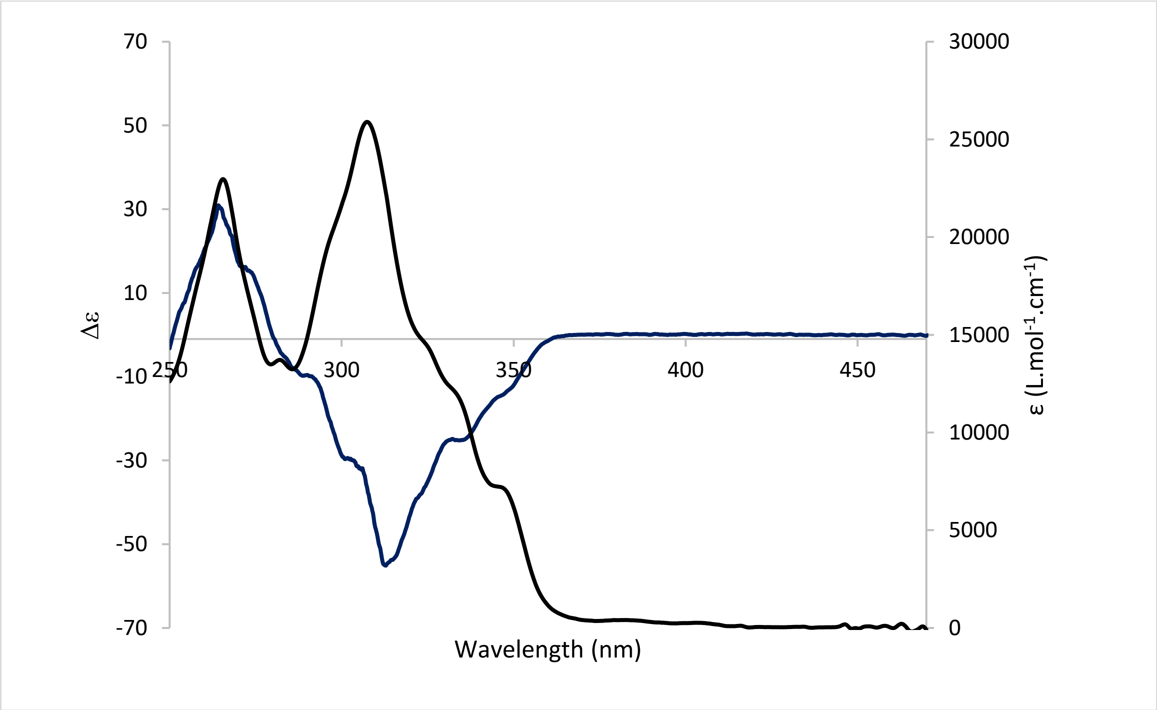

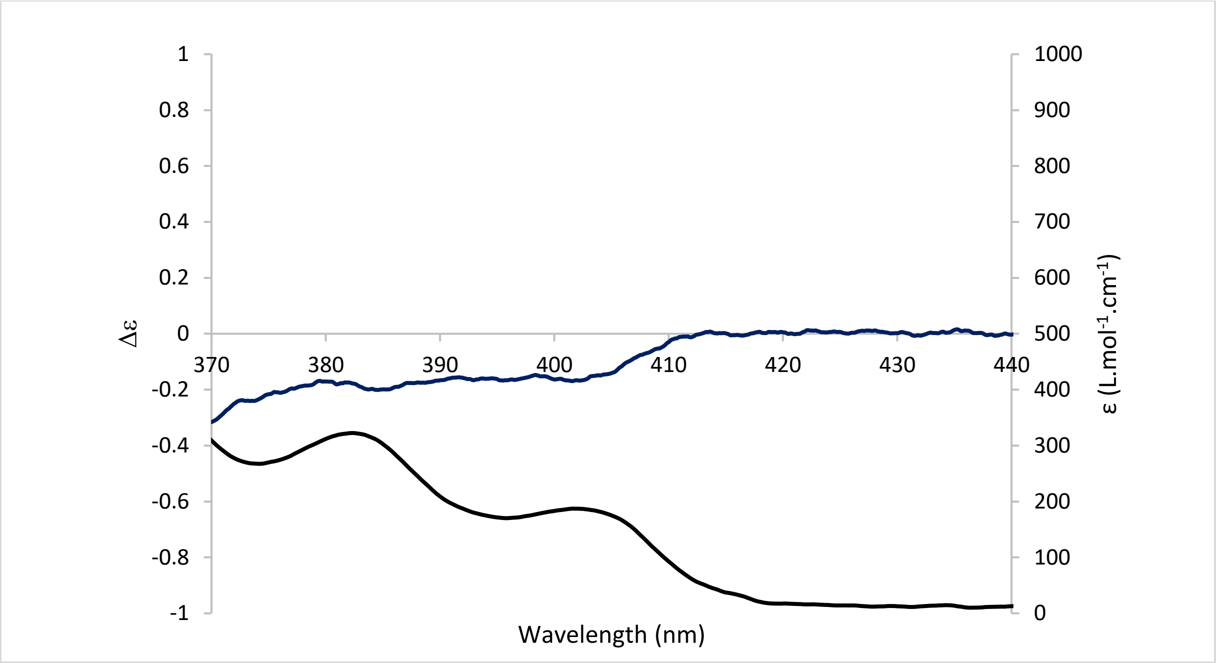

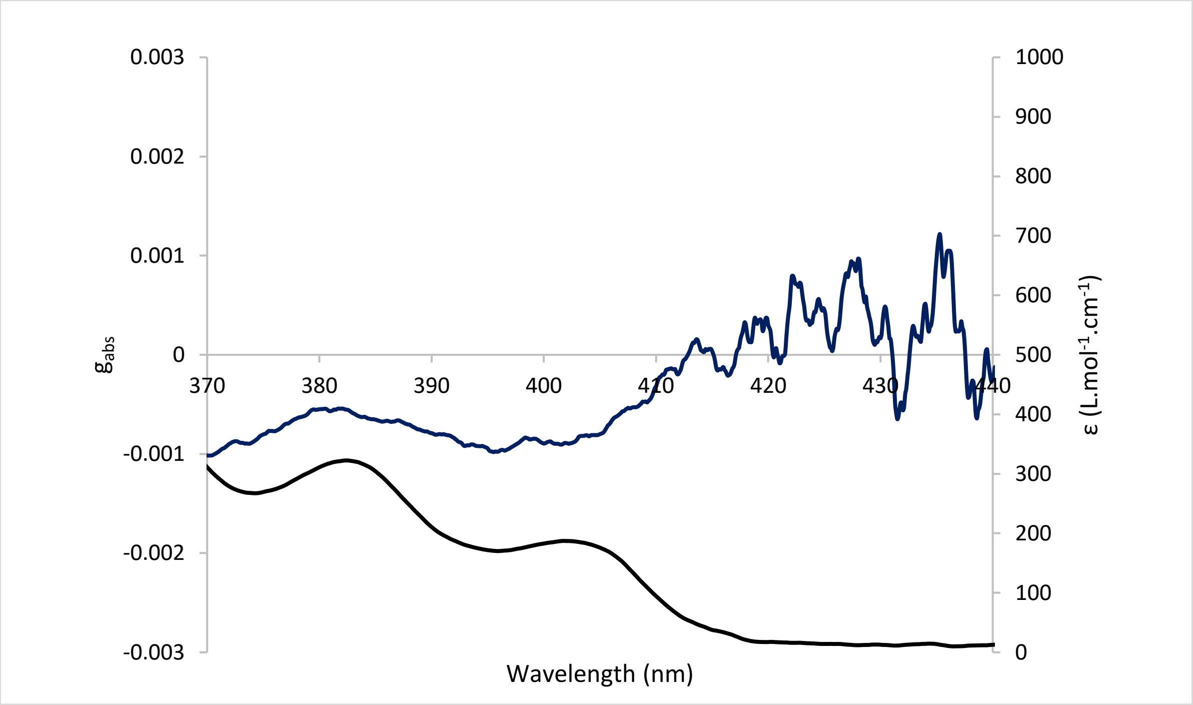

Fig. S64. $\Delta\varepsilon = f(\lambda)$ (top) and $g_{abs} = f(\lambda)$ (bottom) in dichloromethane for **2n**. [c] = 1 x 10^{-3} M. 1 mm cell was used for the 250-500 nm region and 1 cm cell for the 360-440 nm region.

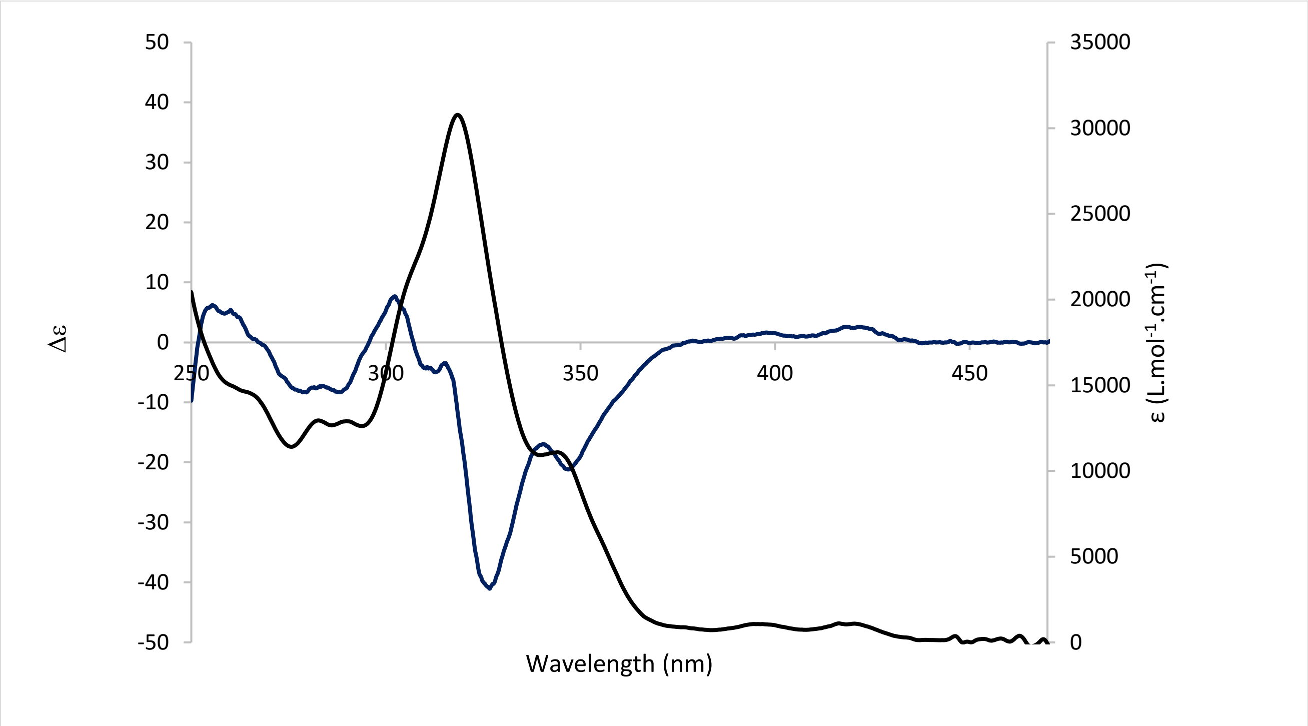

50
40
30
20
10
0
-10
-20
-30
-40
-50
Δε
250
300
350
400
450
35000
30000
25000
20000
15000
10000
5000
0
ε (L.mol-1.cm-1)
Wavelength (nm)

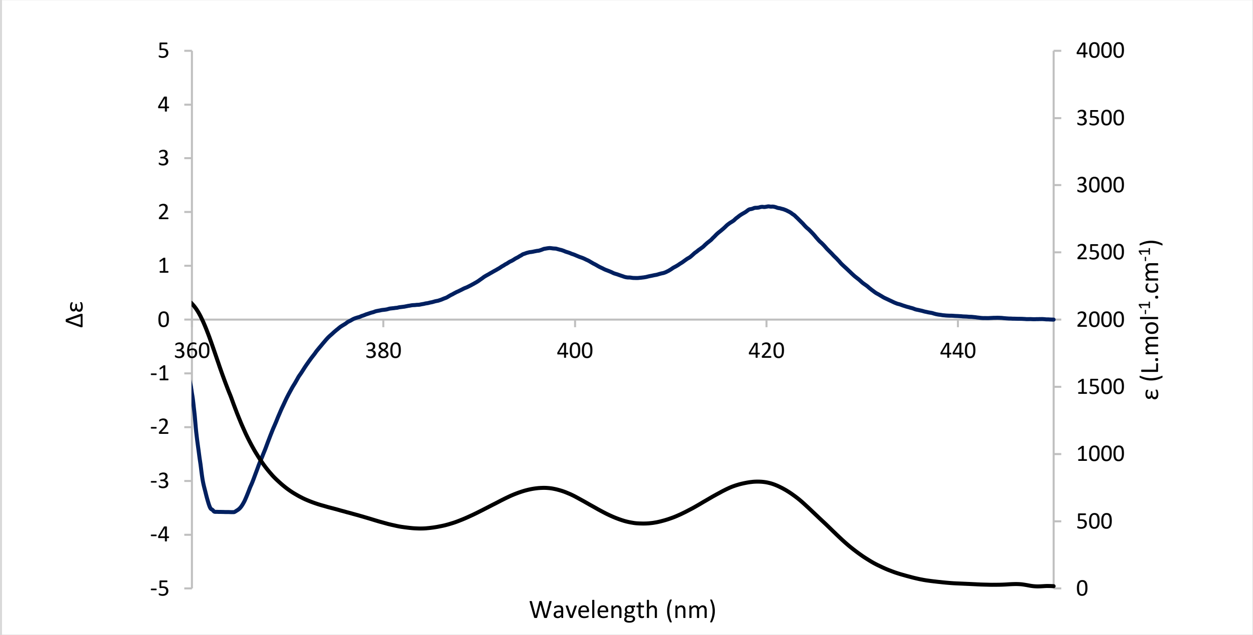

5
4
3
2
1
0
-1
-2
-3
-4
-5
Δε
360
380
400
420
440
4000
3500
3000
2500
2000
1500
1000
500
0
ε (L.mol-1.cm-1)
Wavelength (nm)

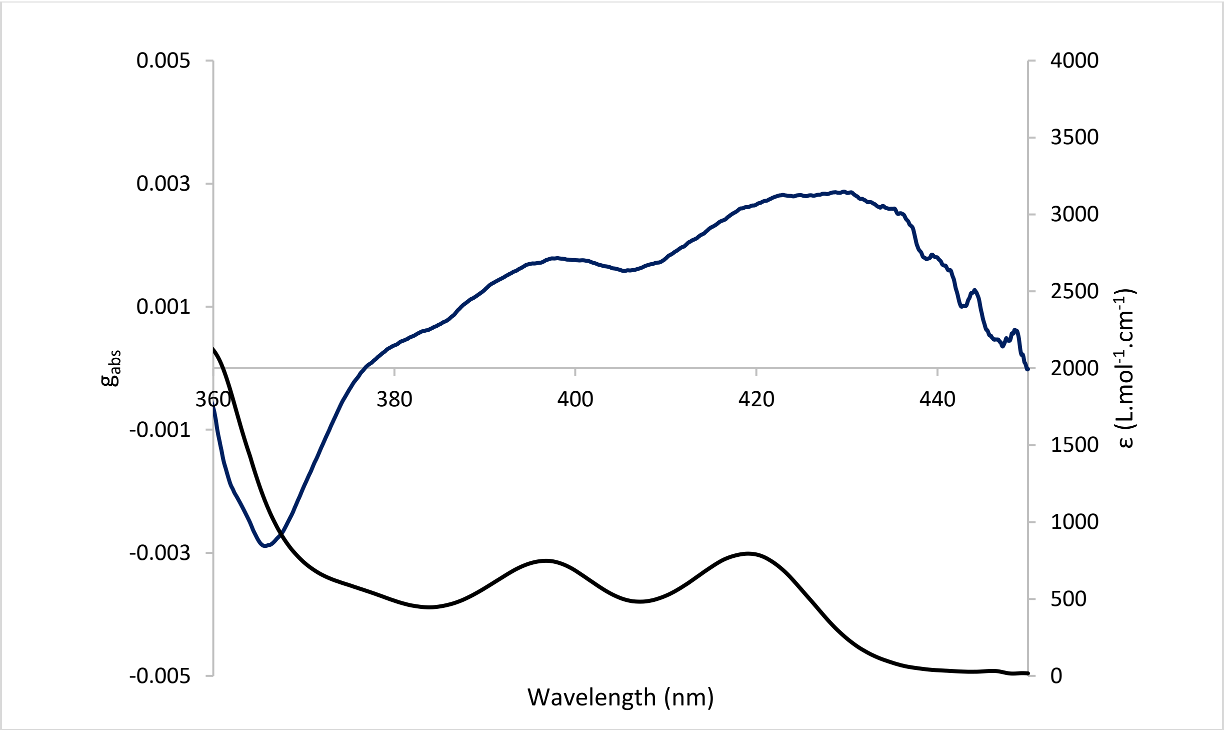

Fig. S65. Δε = f(λ) (top) and g_{abs} = f (λ) (bottom) in dichloromethane for **2o**. [c] = 1 x 10^{-3} M. 1 mm cell was used for the 250-500 nm region and 1 cm cell for the 360-450 nm region.

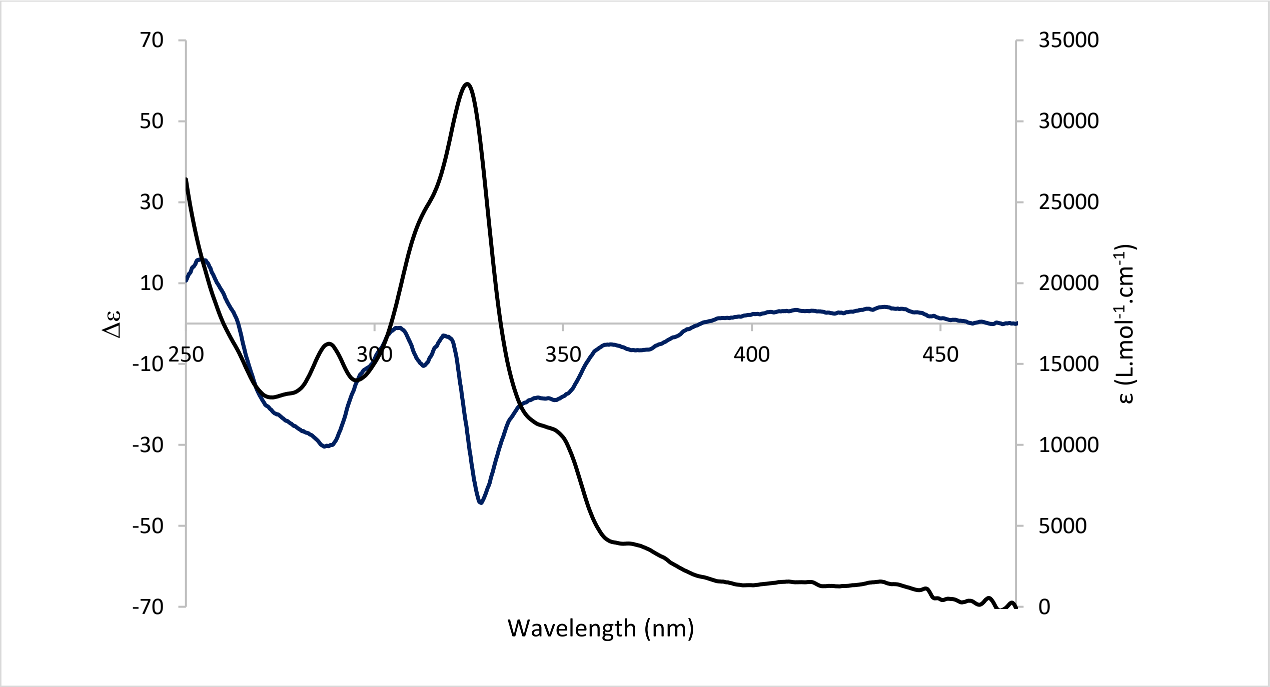

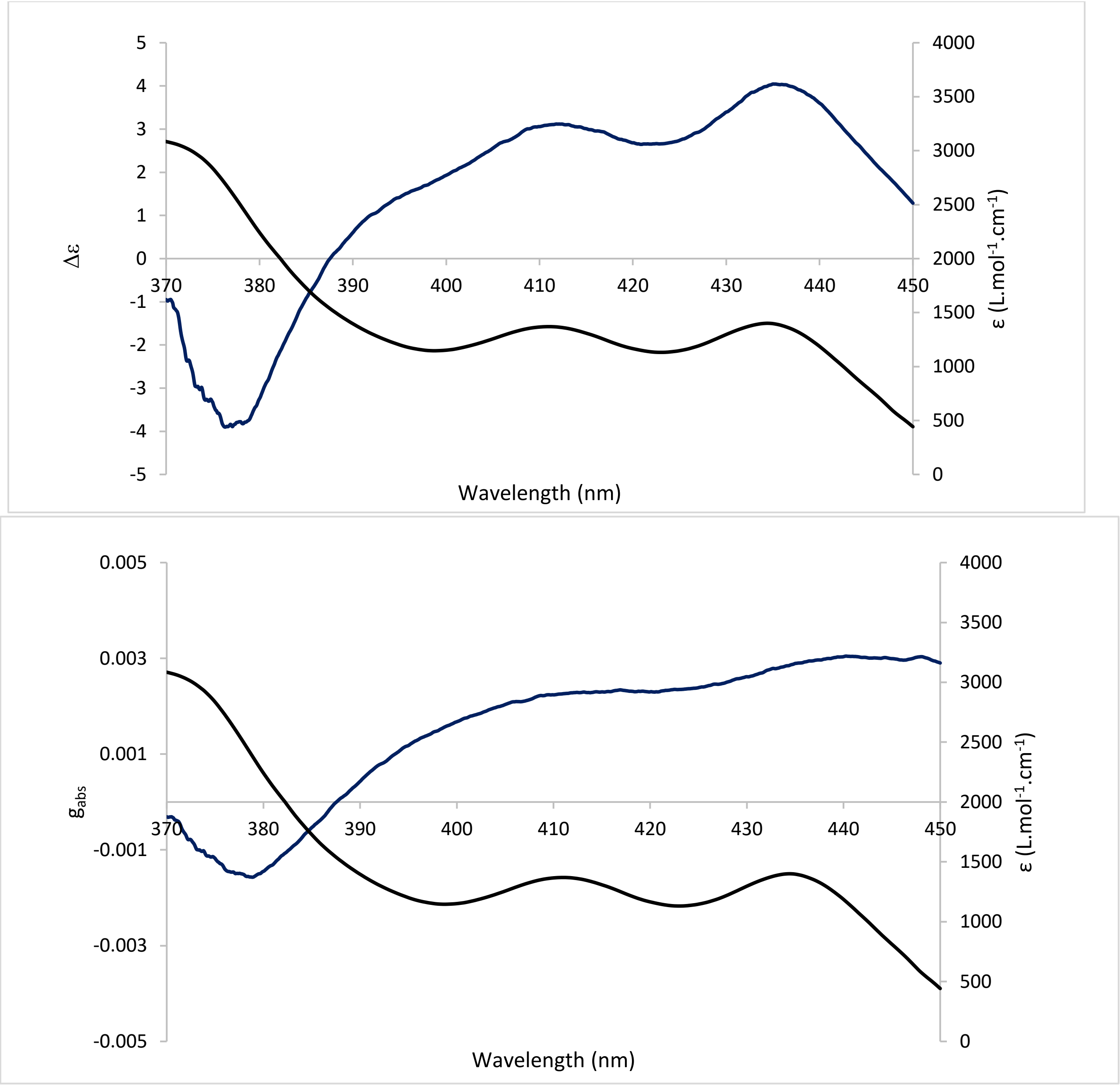

Fig. S66. $\Delta\varepsilon = f(\lambda)$ (top) and $g_{abs} = f(\lambda)$ (bottom) in dichloromethane for **2p**. [c] = 1 x 10^{-3} M. 1 mm cell was used for the 250-500 nm region and 1 cm cell for the 370-450 nm region.

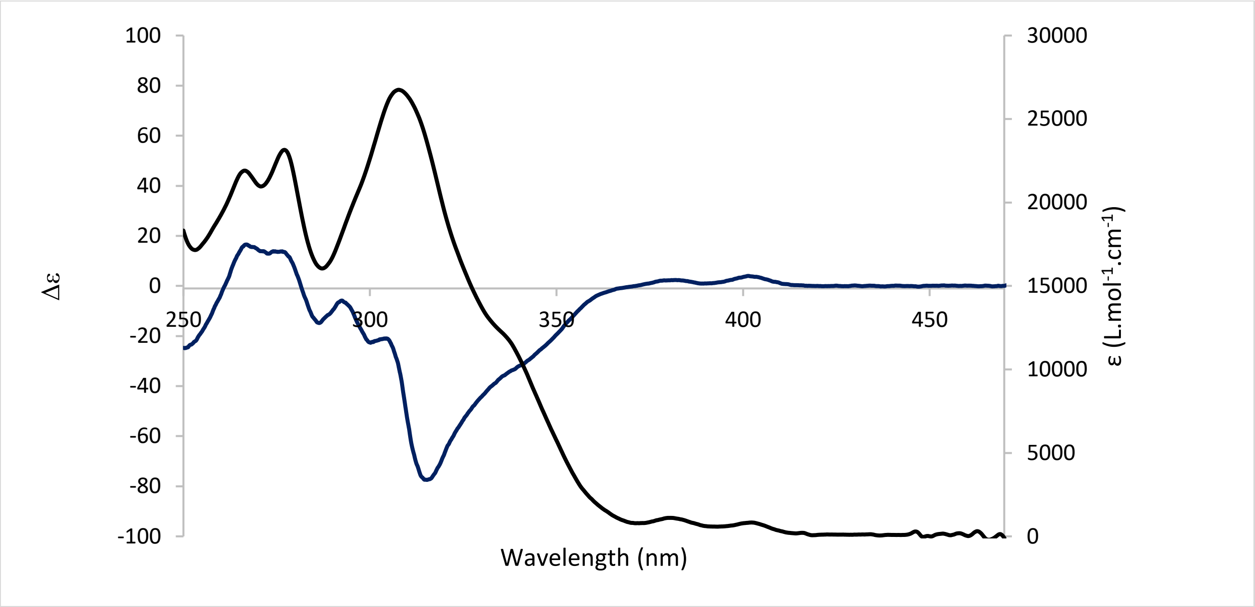
100
80
60
40
20
0
-20
-40
-60
-80
-100
Δε
250
300
350
400
450
Wavelength (nm)
30000
25000
20000
15000
10000
5000
0
ε (L.mol-1.cm-1)

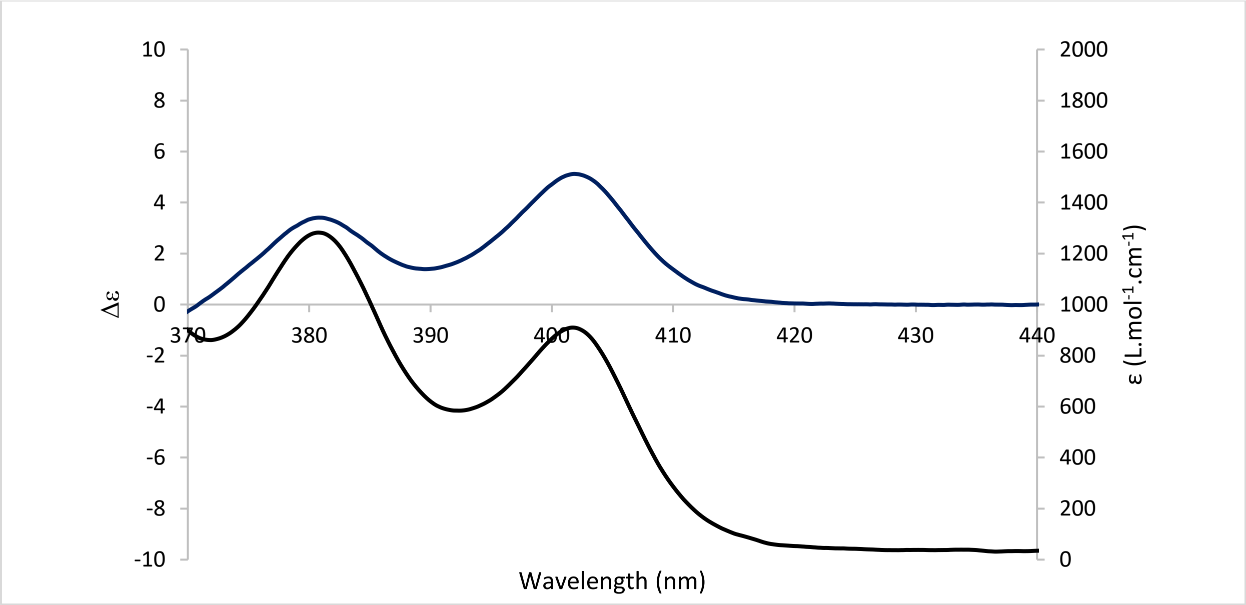

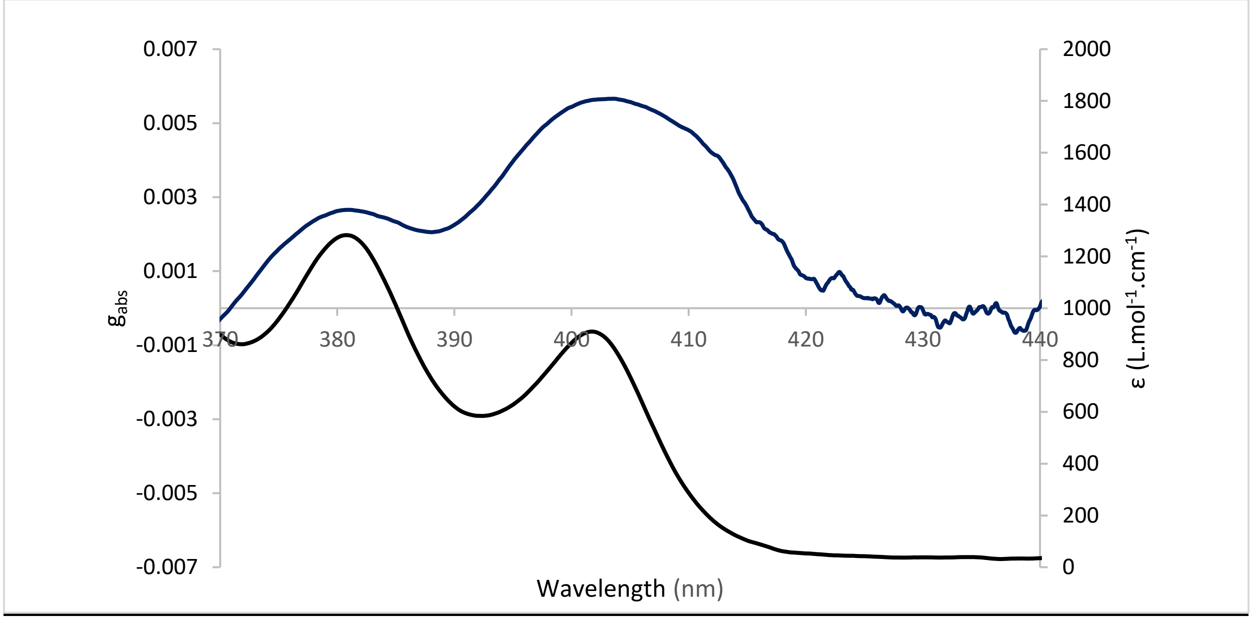

Fig. S67. Δε = f(λ) (top) and g_{abs} = f (λ) (bottom) in dichloromethane for **2q**. [c] = 1 x 10^{-3} M. 1 mm cell was used for the 250-500 nm region and 1 cm cell for the 360-440 nm region.

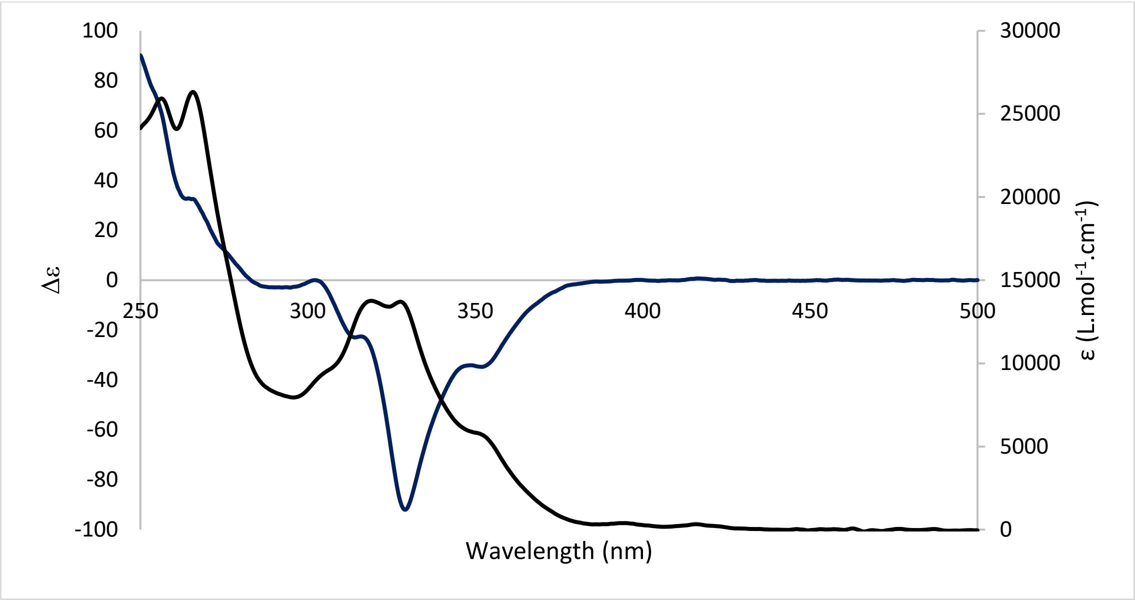

100
80
60
40
20
0
-20
-40
-60
-80
-100
Δε
250
300
350
400
450
500
Wavelength (nm)
30000
25000
20000
15000
10000
5000
0
ε (L.mol-1.cm-1)

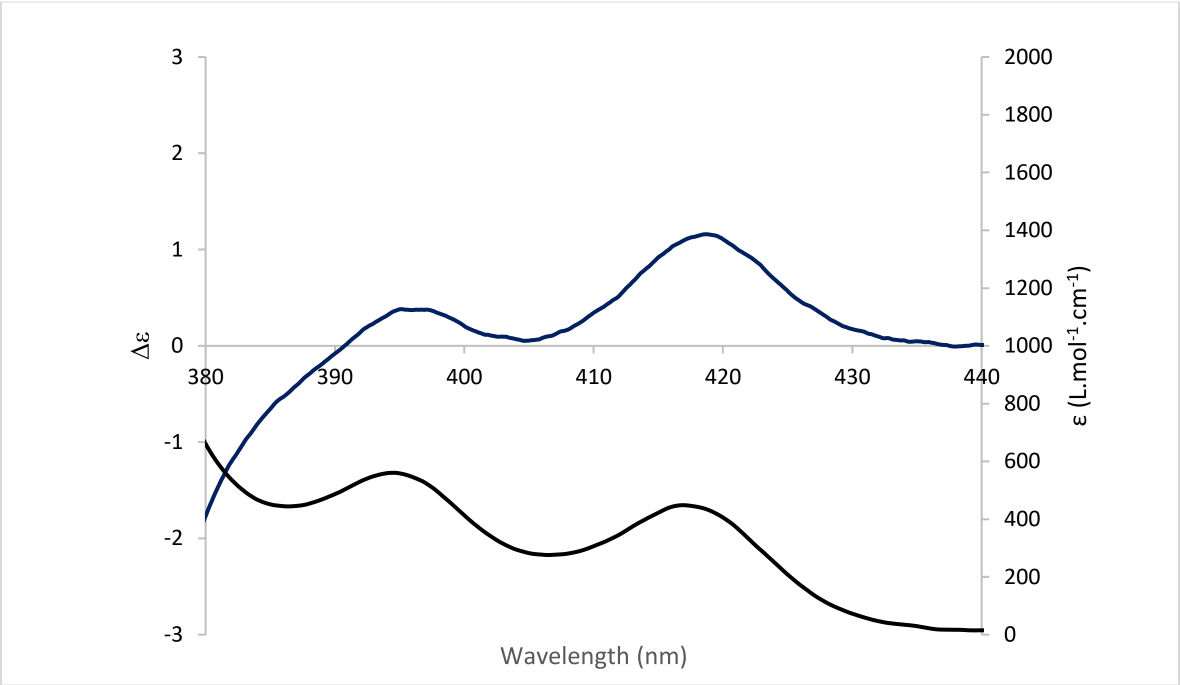

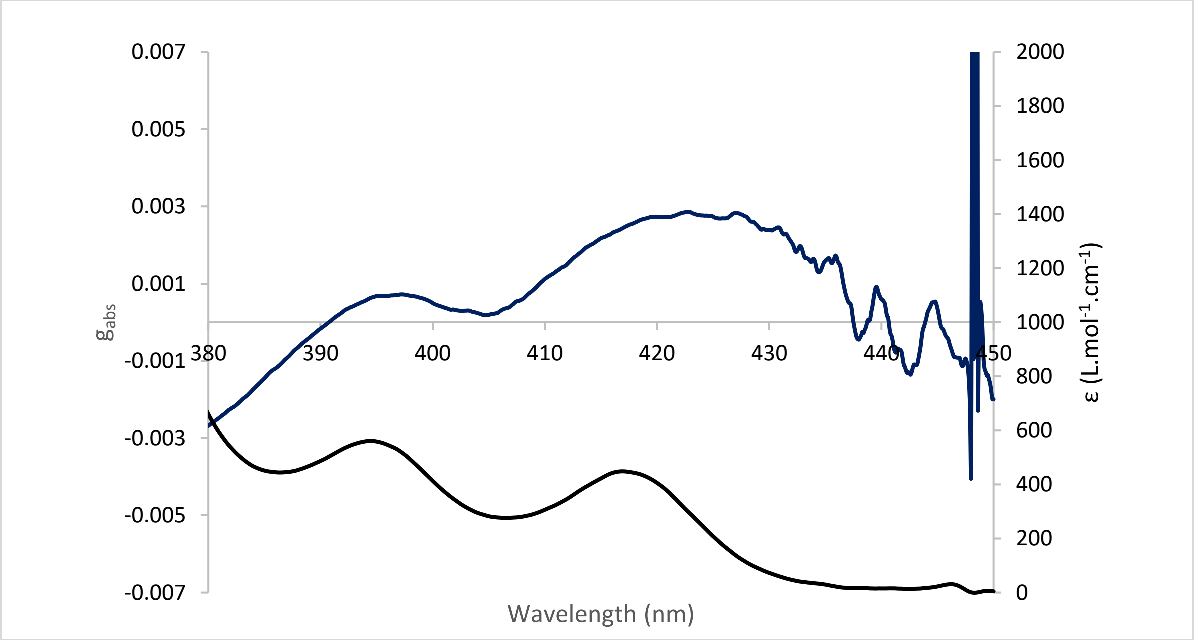

Fig. S68. $\Delta\varepsilon = f(\lambda)$ (top) and $g_{abs} = f(\lambda)$ (bottom) in dichloromethane for **2s**. [c] = 1 x 10^{-3} M. 1 mm cell was used for the 250-500 nm region and 1 cm cell for the 380-450 nm region.

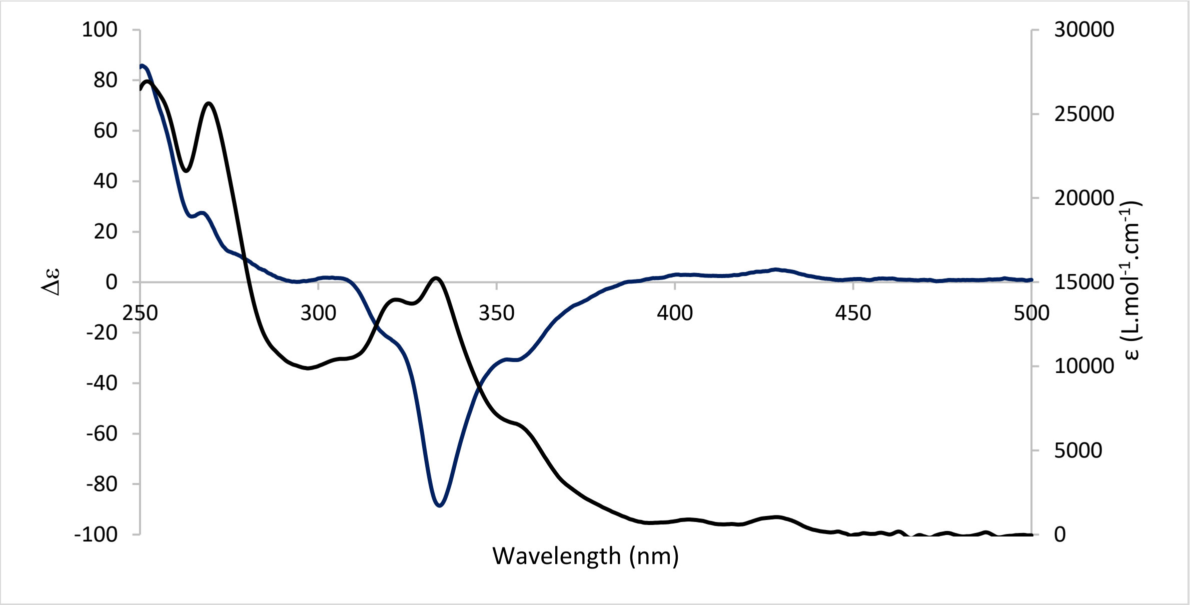
Δε
ε (L.mol-1.cm-1)
Wavelength (nm)
100
80
60
40
20
0
-20
-40
-60
-80
-100
250
300
350
400
450
500
30000
25000
20000
15000
10000
5000
0

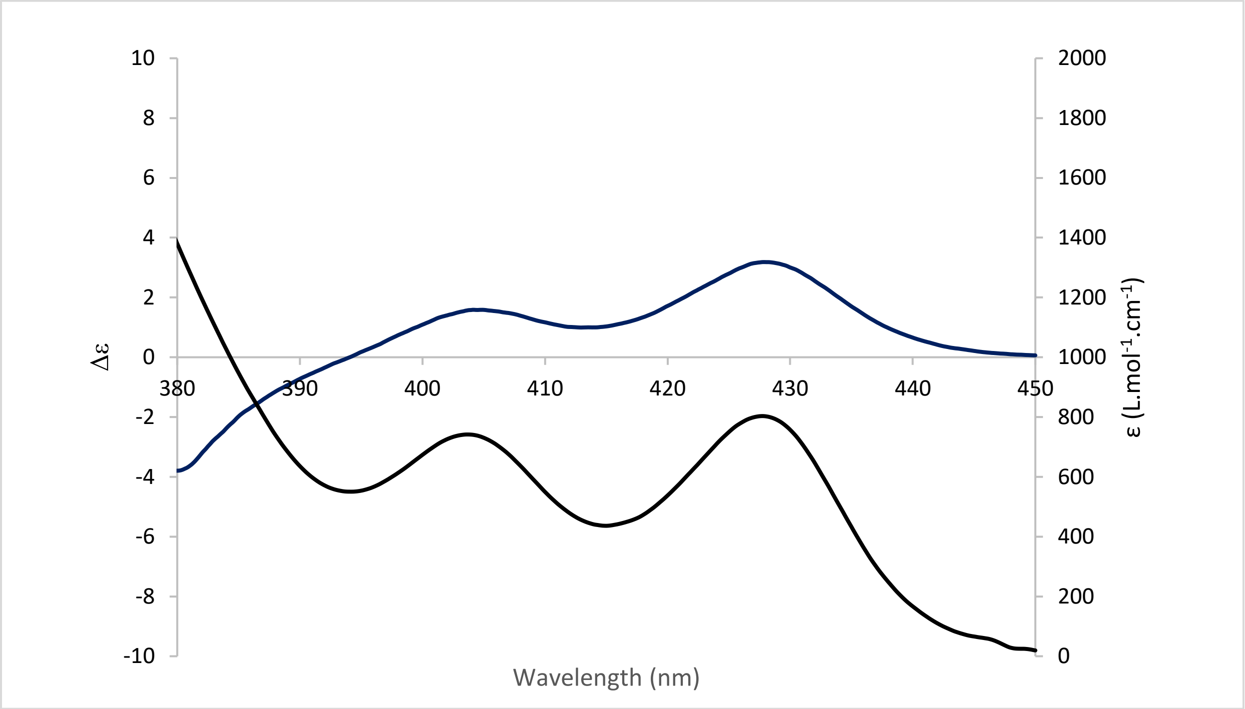
Δε
ε (L.mol-1.cm-1)
Wavelength (nm)
10
8
6
4
2
0
-2
-4
-6
-8
-10
380
390
400
410
420
430
440
450
2000
1800
1600
1400
1200
1000
800
600
400
200
0

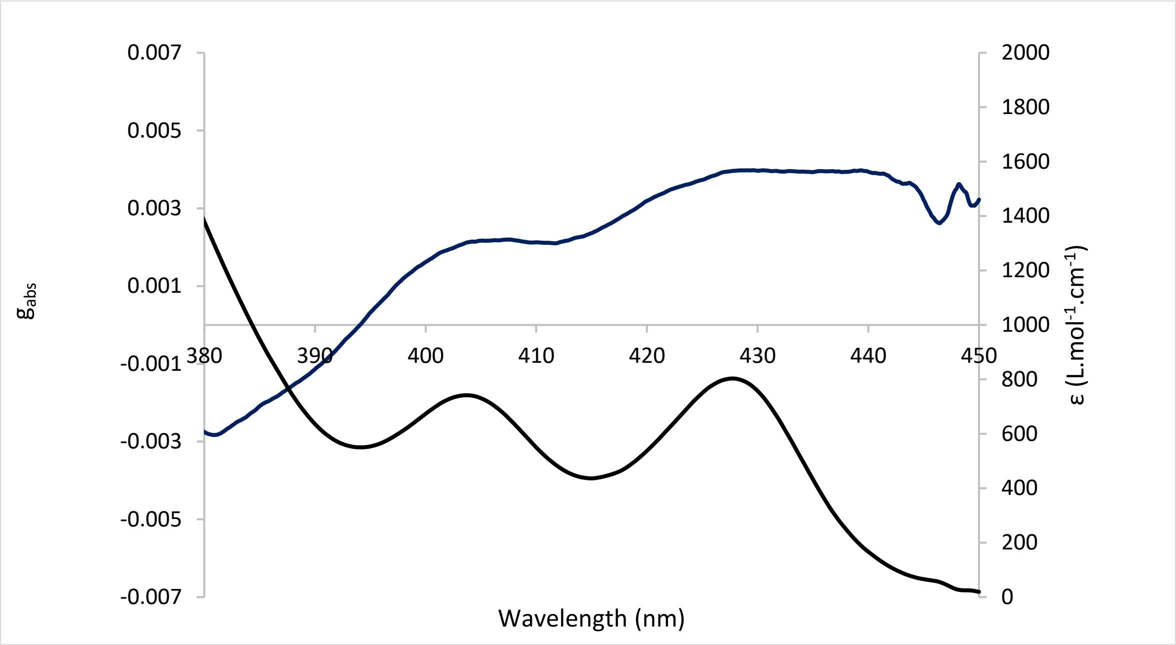

Fig. S69. $\Delta\varepsilon = f(\lambda)$ (top) and $g_{abs} = f(\lambda)$ (bottom) in dichloromethane for **2t**. [c] = 1 x 10^{-3} M. 1 mm cell was used for the 250-500 nm region and 1 cm cell for the 360-440 nm region.

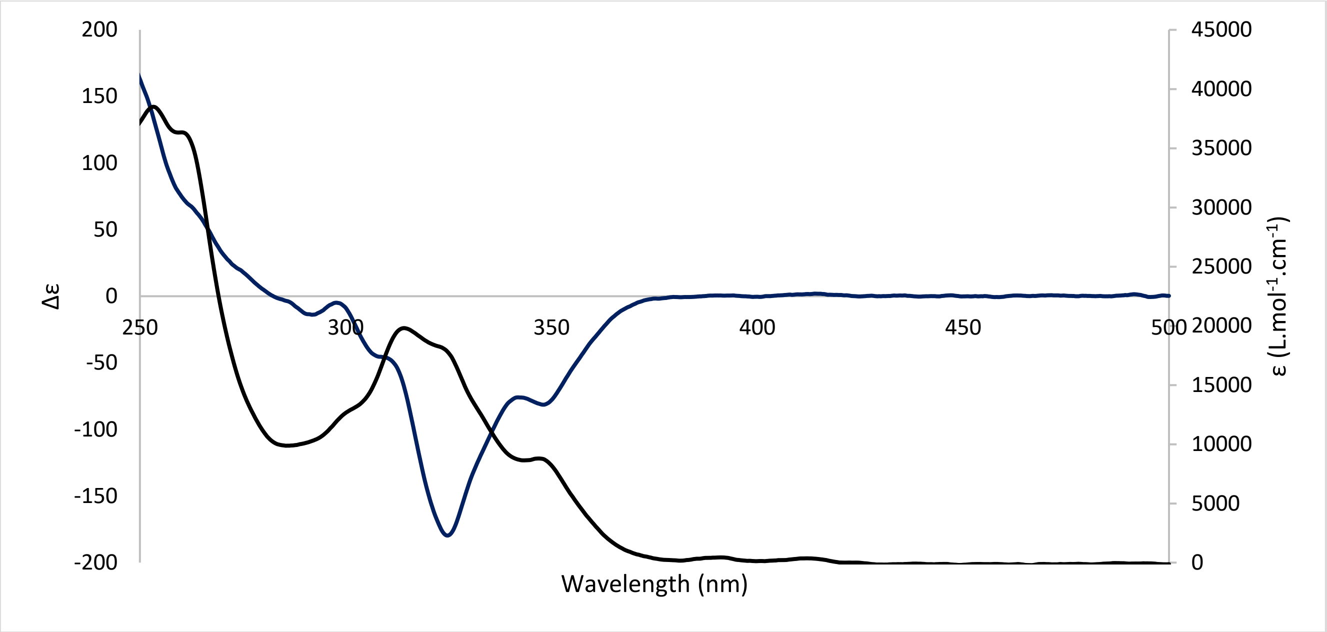
200
150
100
50
0
-50
-100
-150
-200
Δε
250
300
350
400
450
500
45000
40000
35000
30000
25000
20000
15000
10000
5000
0
ε (L.mol-1.cm-1)
Wavelength (nm)

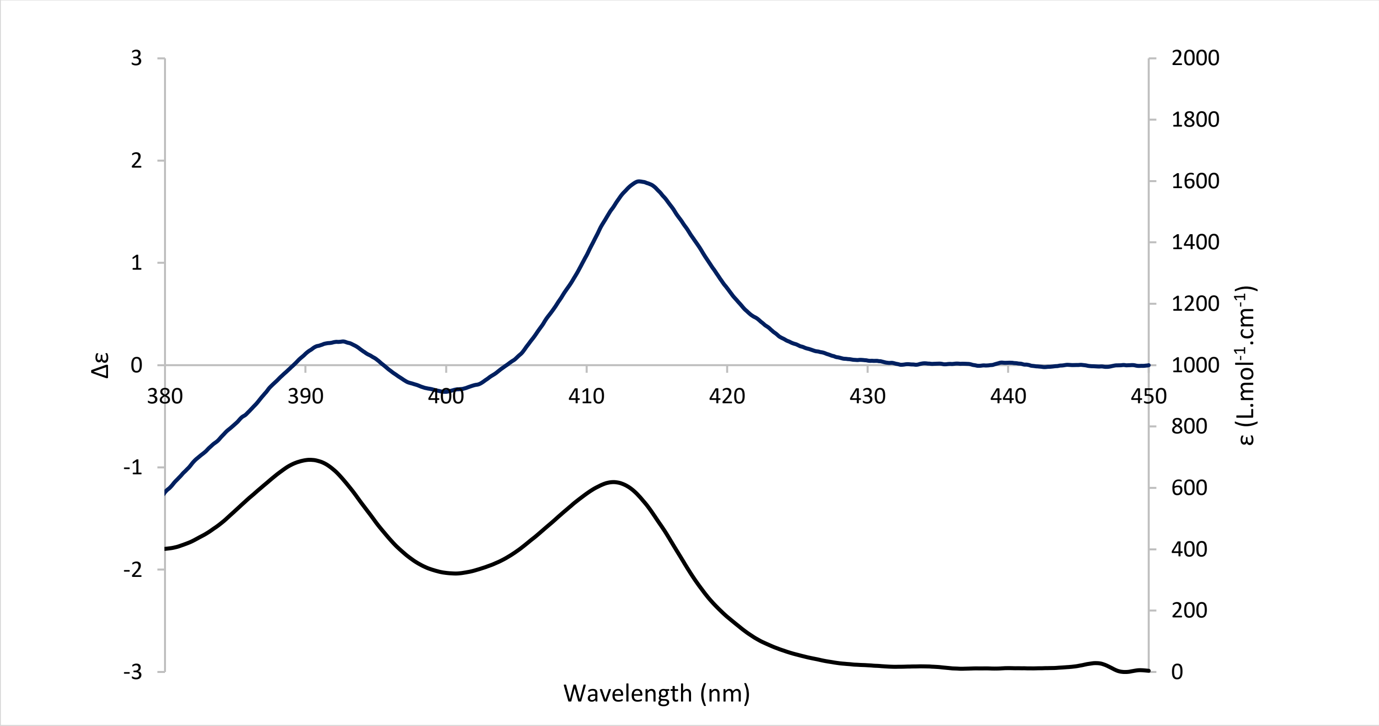
3
2
1
0
-1
-2
-3
Δε
380
390
400
410
420
430
440
450
2000
1800
1600
1400
1200
1000
800
600
400
200
0
ε (L.mol-1.cm-1)
Wavelength (nm)

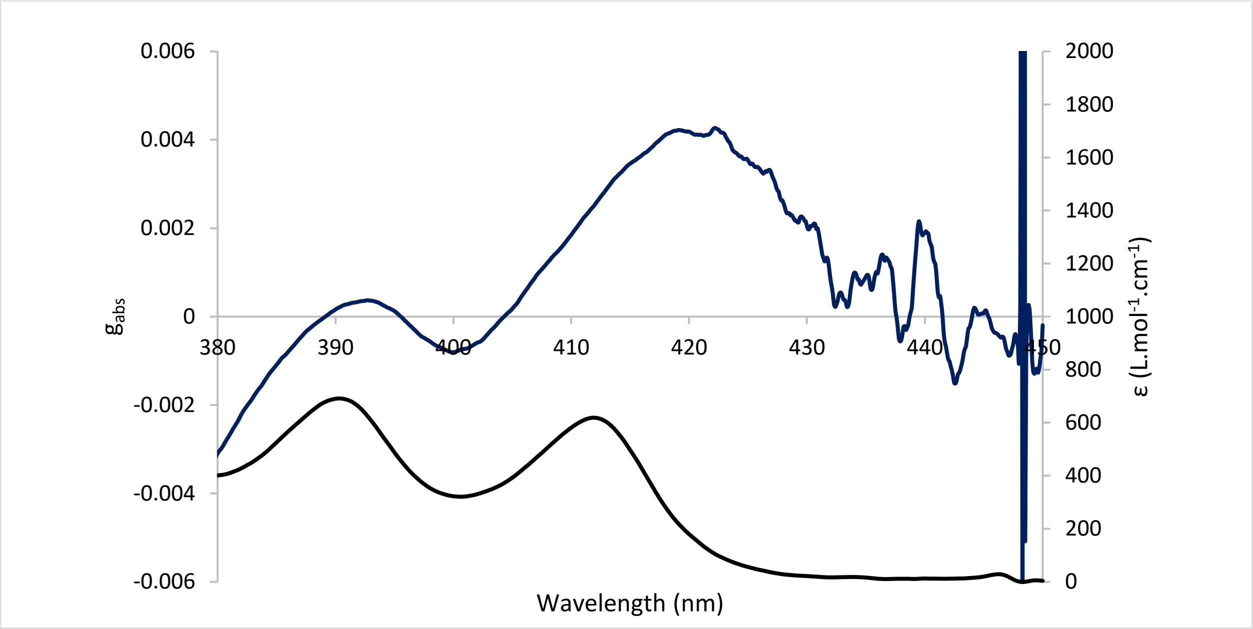

Fig. S70. $\Delta\varepsilon = f(\lambda)$ (top) and $g_{abs} = f(\lambda)$ (bottom) in dichloromethane for **2u**. [c] = 1 x 10^{-3} M. 1 mm cell was used for the 250-500 nm region and 1 cm cell for the 380-450 nm region.

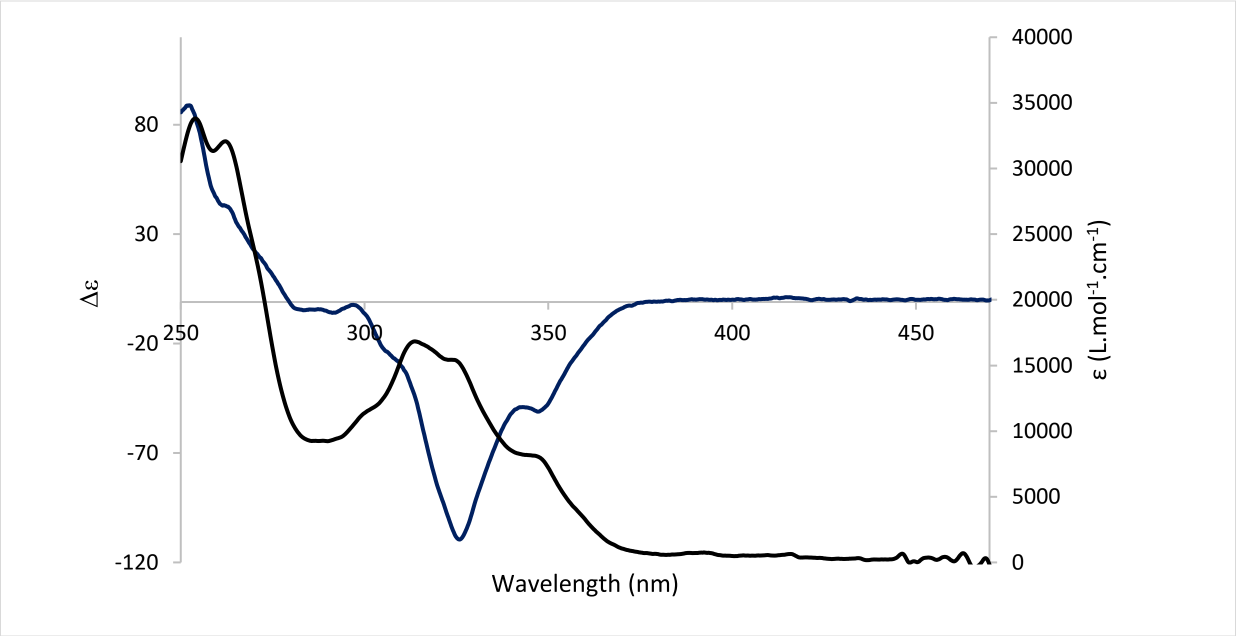
Δε
ε (L.mol-1.cm-1)
Wavelength (nm)
80
30
-20
-70
-120
250
300
350
400
450
40000
35000
30000
25000
20000
15000
10000
5000
0

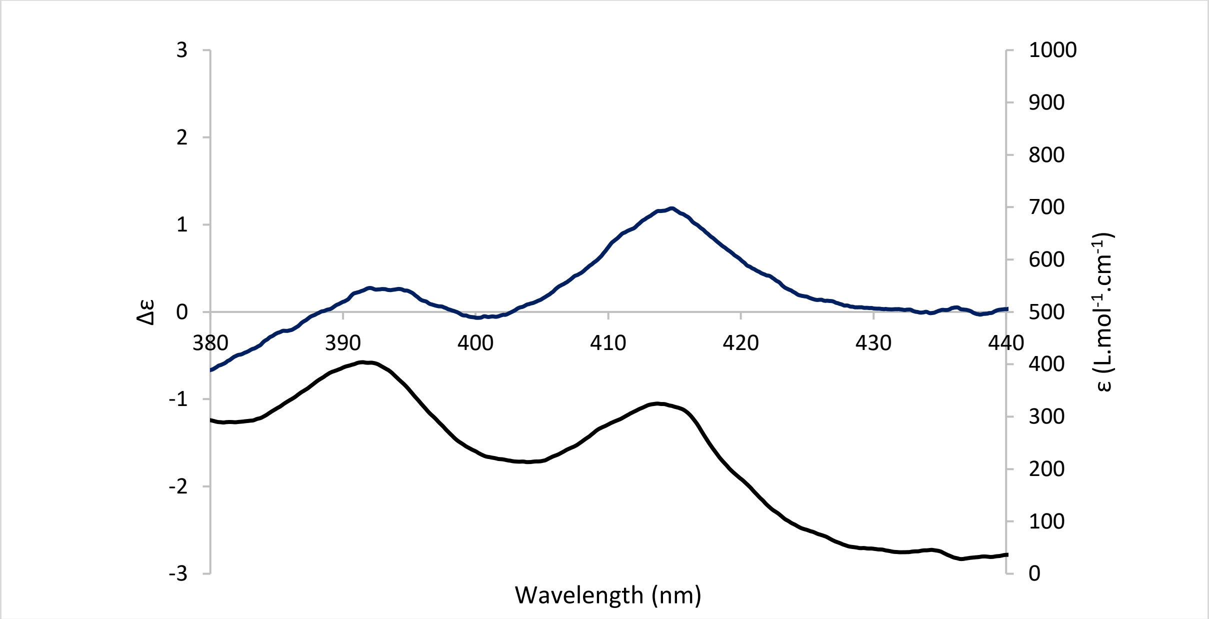
Δε
ε (L.mol-1.cm-1)
Wavelength (nm)
3
2
1
0
-1
-2
-3
380
390
400
410
420
430
440
1000
900
800
700
600
500
400
300
200
100
0

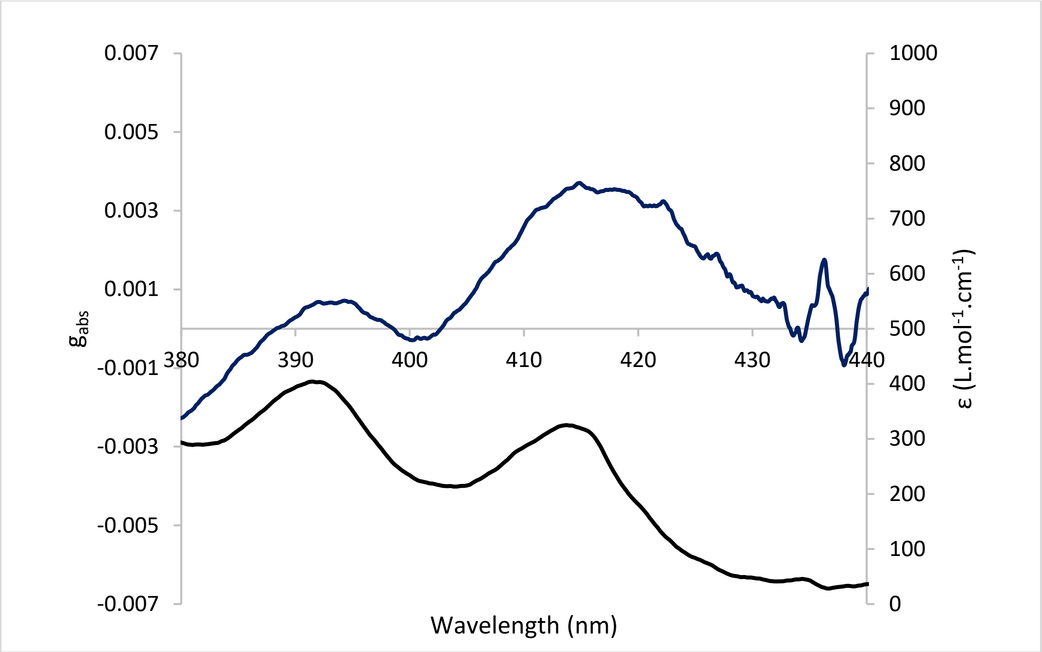

Fig. S71. Δε = f(λ) (top) and g_{abs} = f (λ) (bottom) in dichloromethane for **2v**. [c] = 1 x 10^{-3} M. 1 mm cell was used for the 250-500 nm region and 1 cm cell for the 360-440 nm region.

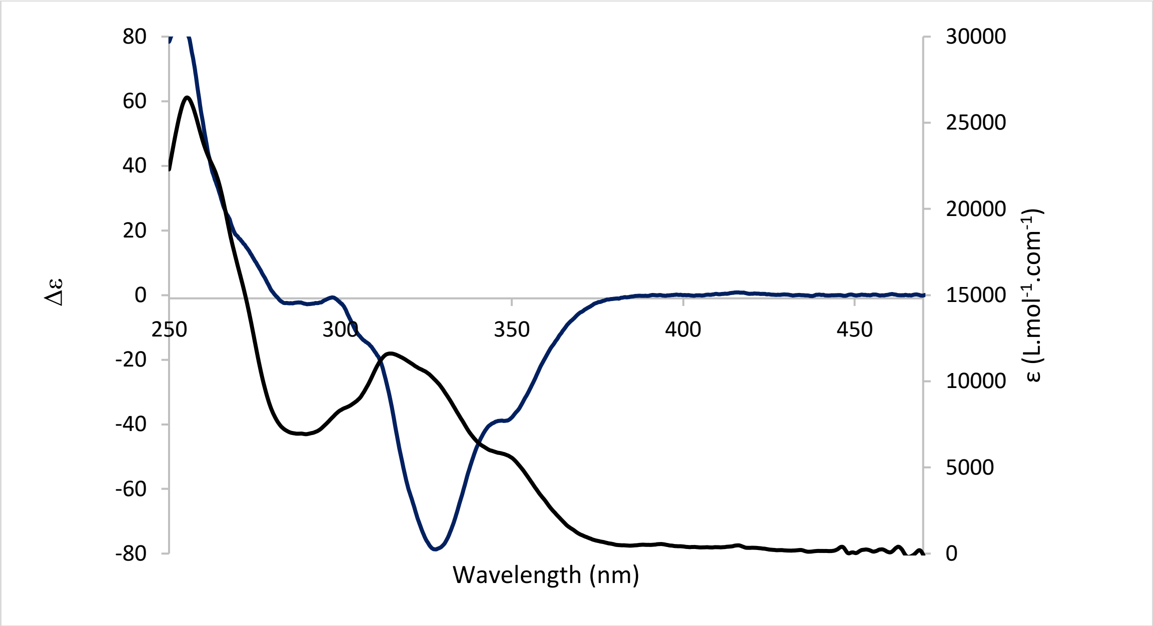
80
60
40
20
0
-20
-40
-60
-80
Δε
250
300
350
400
450
Wavelength (nm)
30000
25000
20000
15000
10000
5000
0
ε (L.mol-1.com-1)

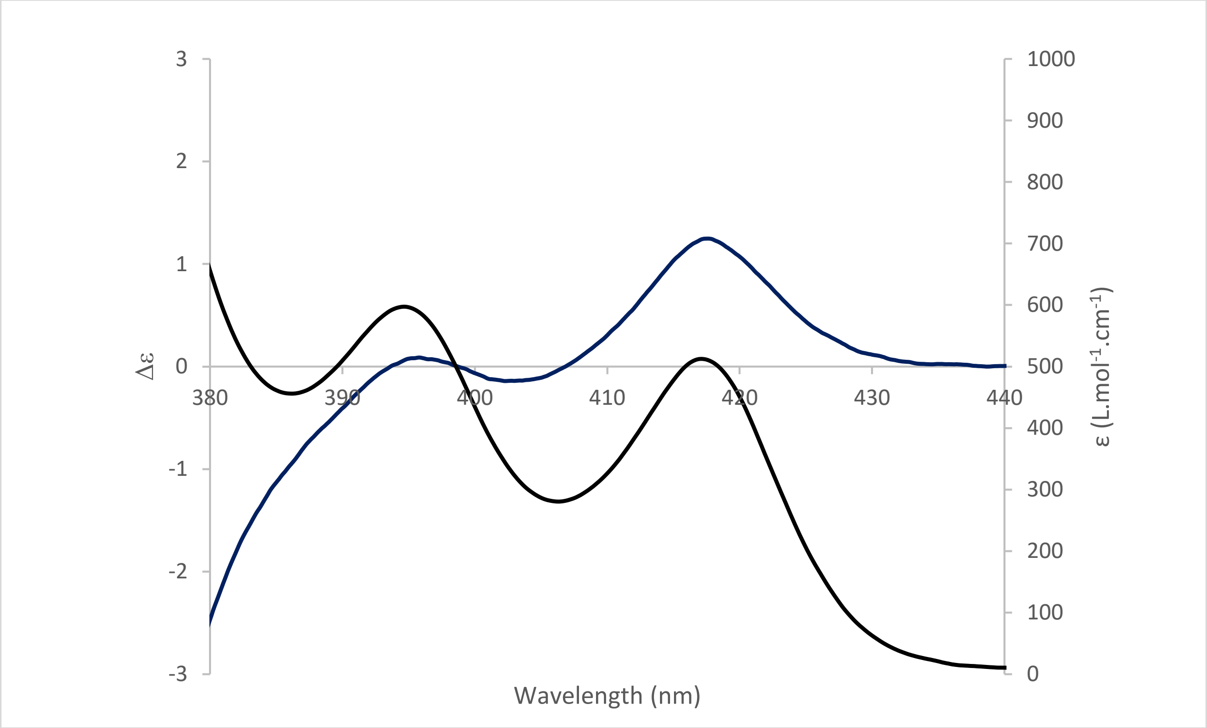
3
2
1
0
-1
-2
-3
Δε
380
390
400
410
420
430
440
Wavelength (nm)
1000
900
800
700
600
500
400
300
200
100
0
ε (L.mol-1.cm-1)

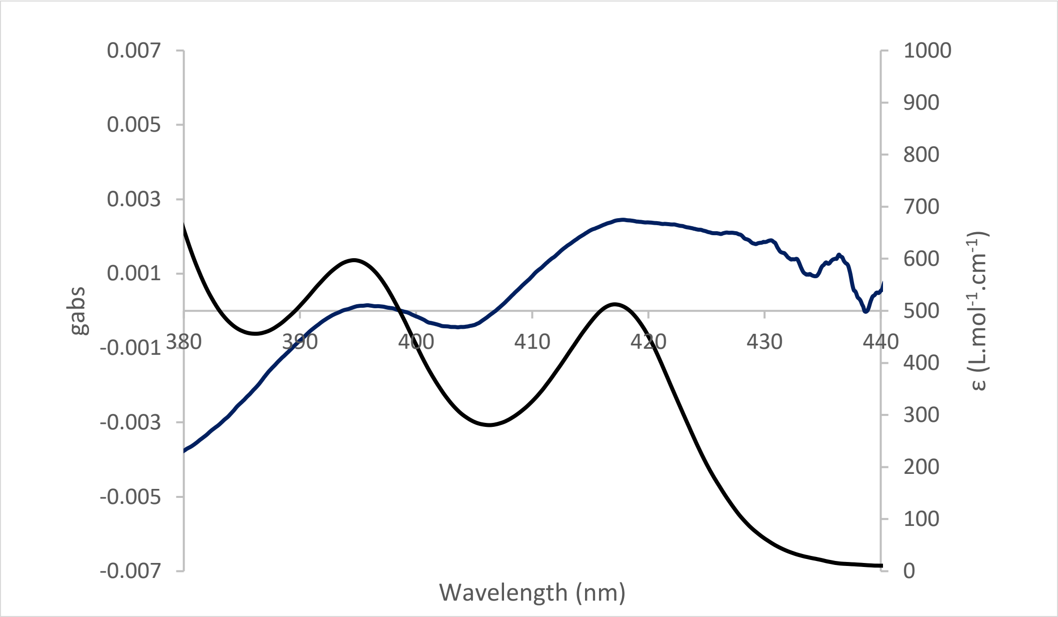

Fig. S72. Δε = f(λ) (top) and g_{abs} = f (λ) (bottom) in dichloromethane for **2w**. [c] = 1 x 10^{-3} M. 1 mm cell was used for the 250-500 nm region and 1 cm cell for the 360-440 nm region.

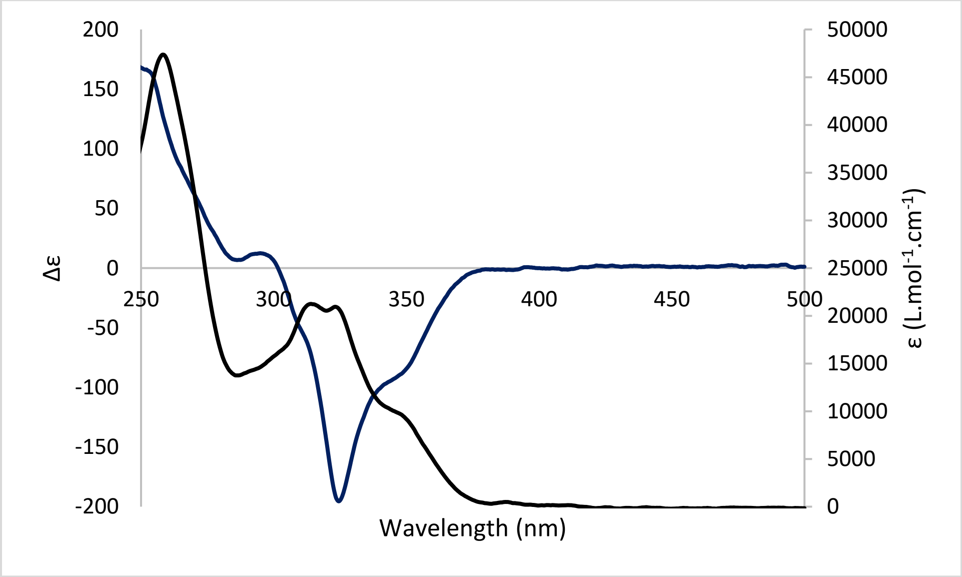

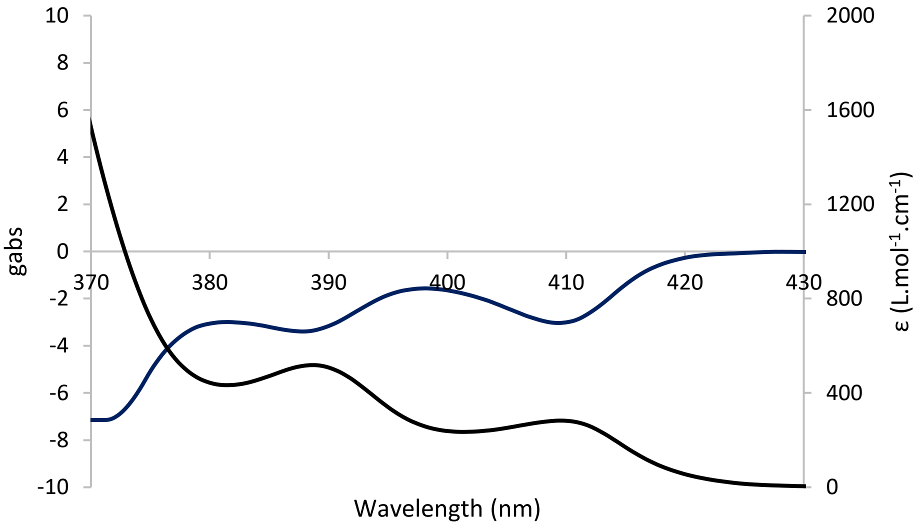

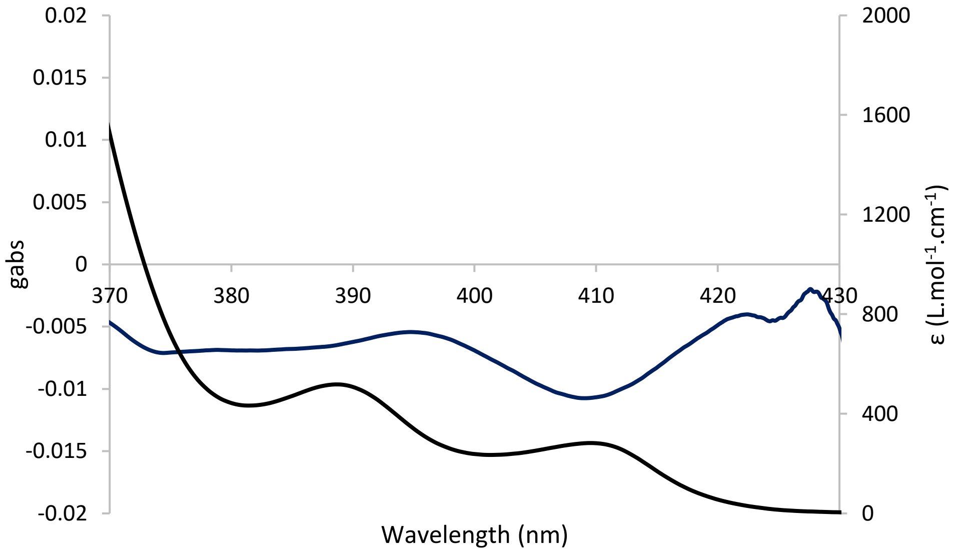

Fig. S73. $\Delta\varepsilon = f(\lambda)$ (top) and $g_{abs} = f(\lambda)$ (bottom) in dichloromethane for **2x**. [c] = 1 x 10^{-3} M. 1 mm cell was used for the 250-500 nm region and 1 cm cell for the 360-440 nm region.

Lifetime measurement:

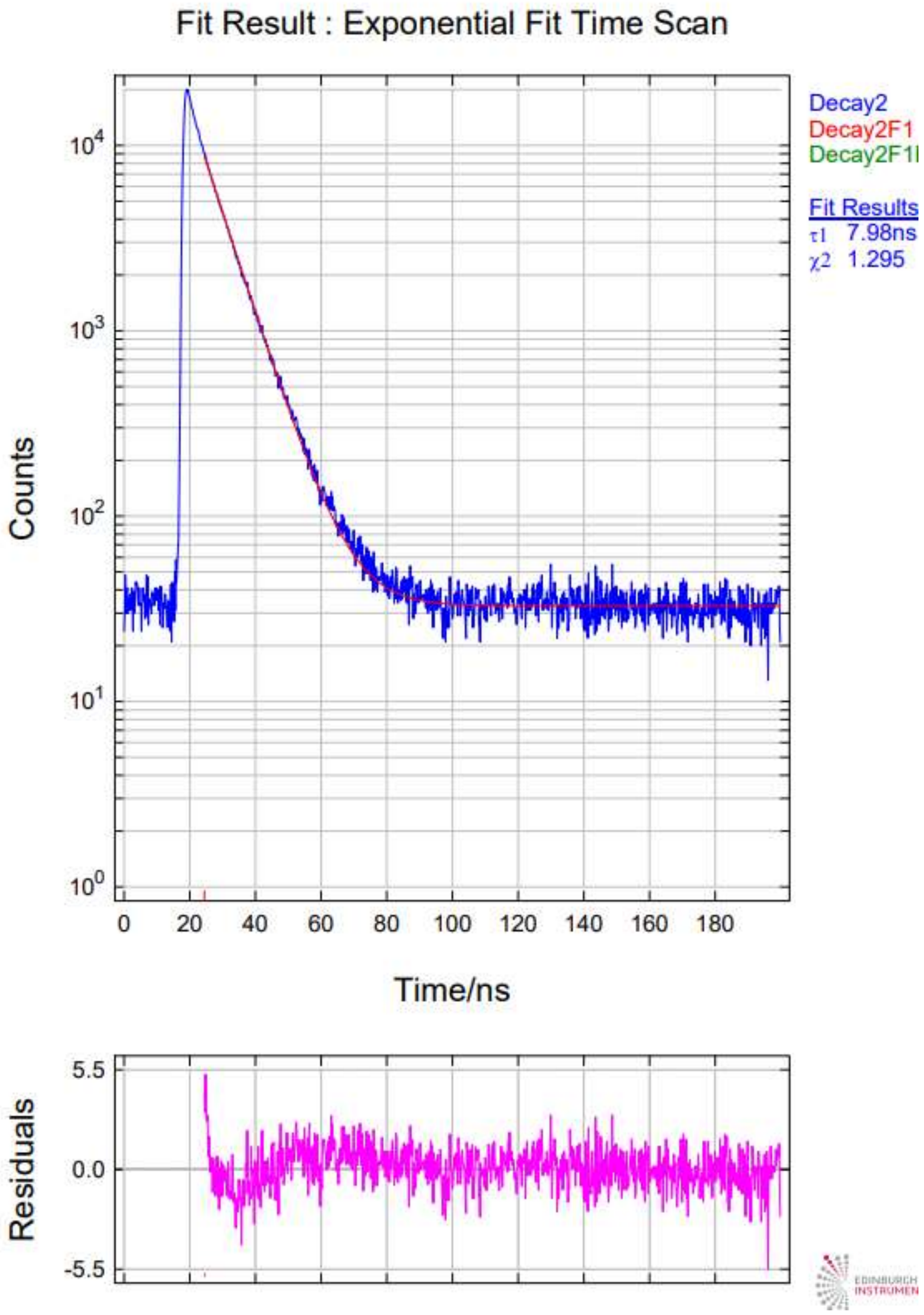

Fig. S74. TCSPC experiments in dichloromethane solution for **2a**. [c] = 1 x 10^{-5} M. λ_{exc} = 365 nm.

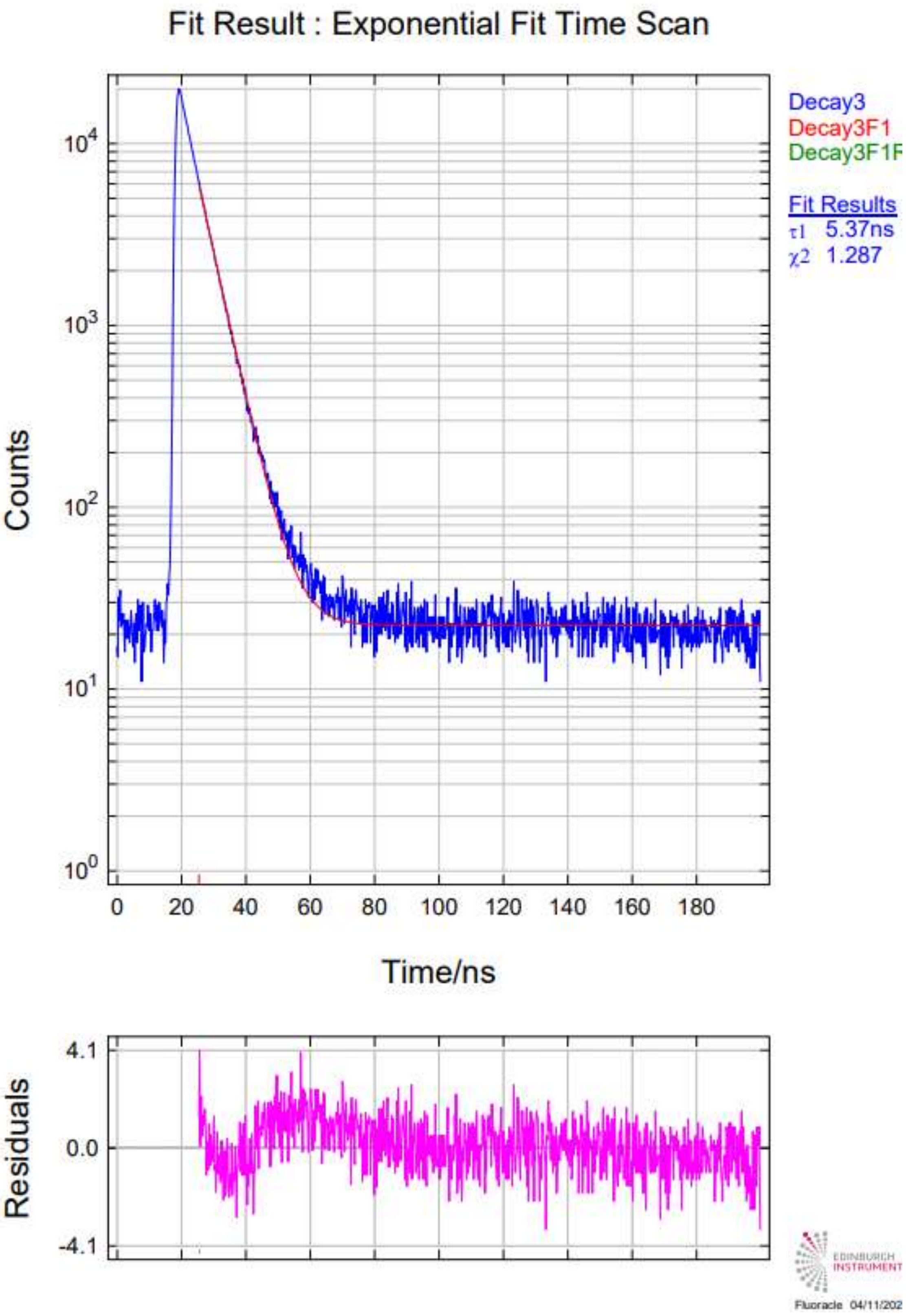

Fig. S75. TCSPC experiments in dichloromethane solution for **2b**. [c] = 1 x 10^{-5} M. λ_{exc} = 365 nm.

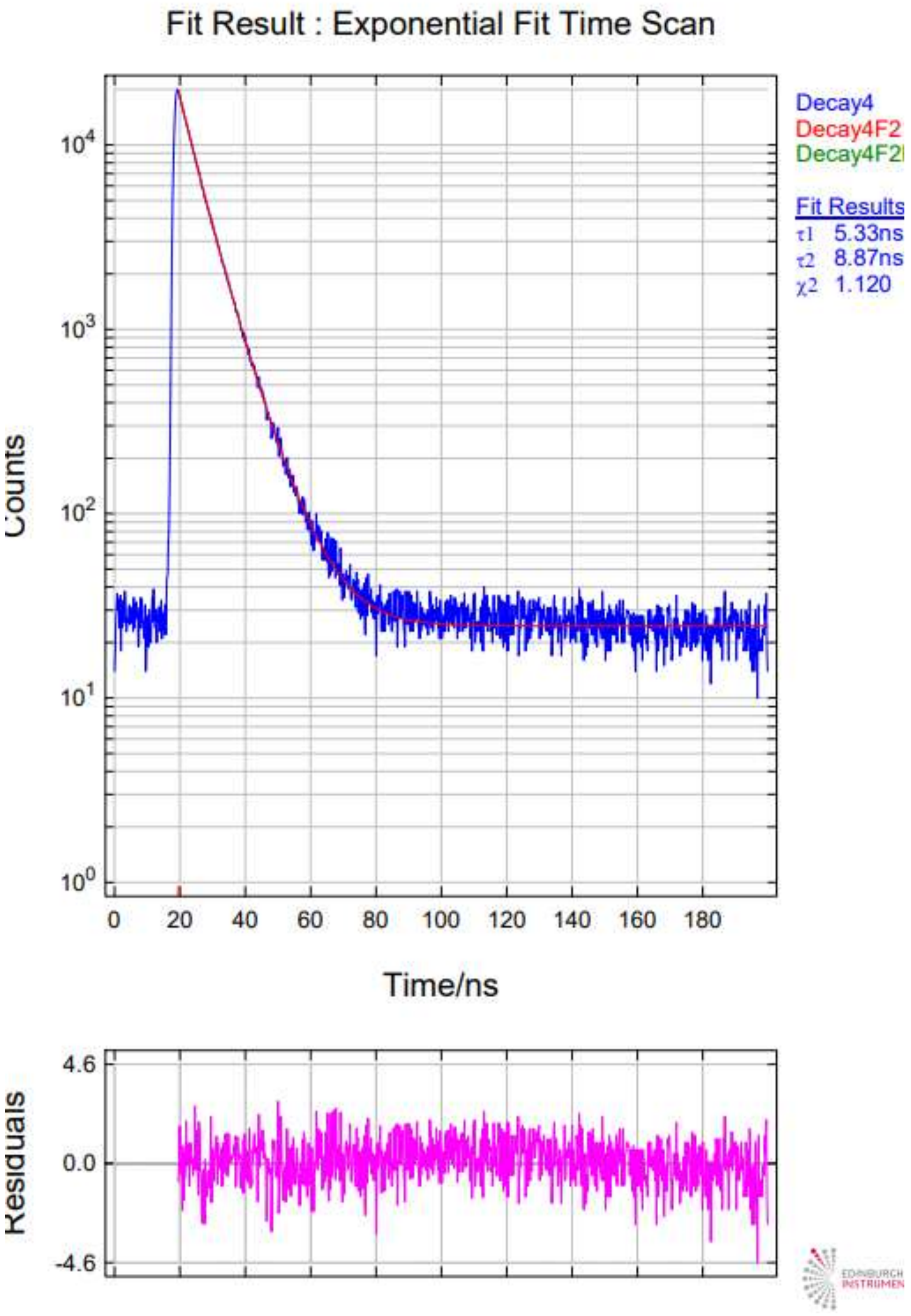

Fig. S76. TCSPC experiments in dichloromethane solution for **2c**. [c] = 1 x 10^{-5} M. λ_{exc} = 365 nm.

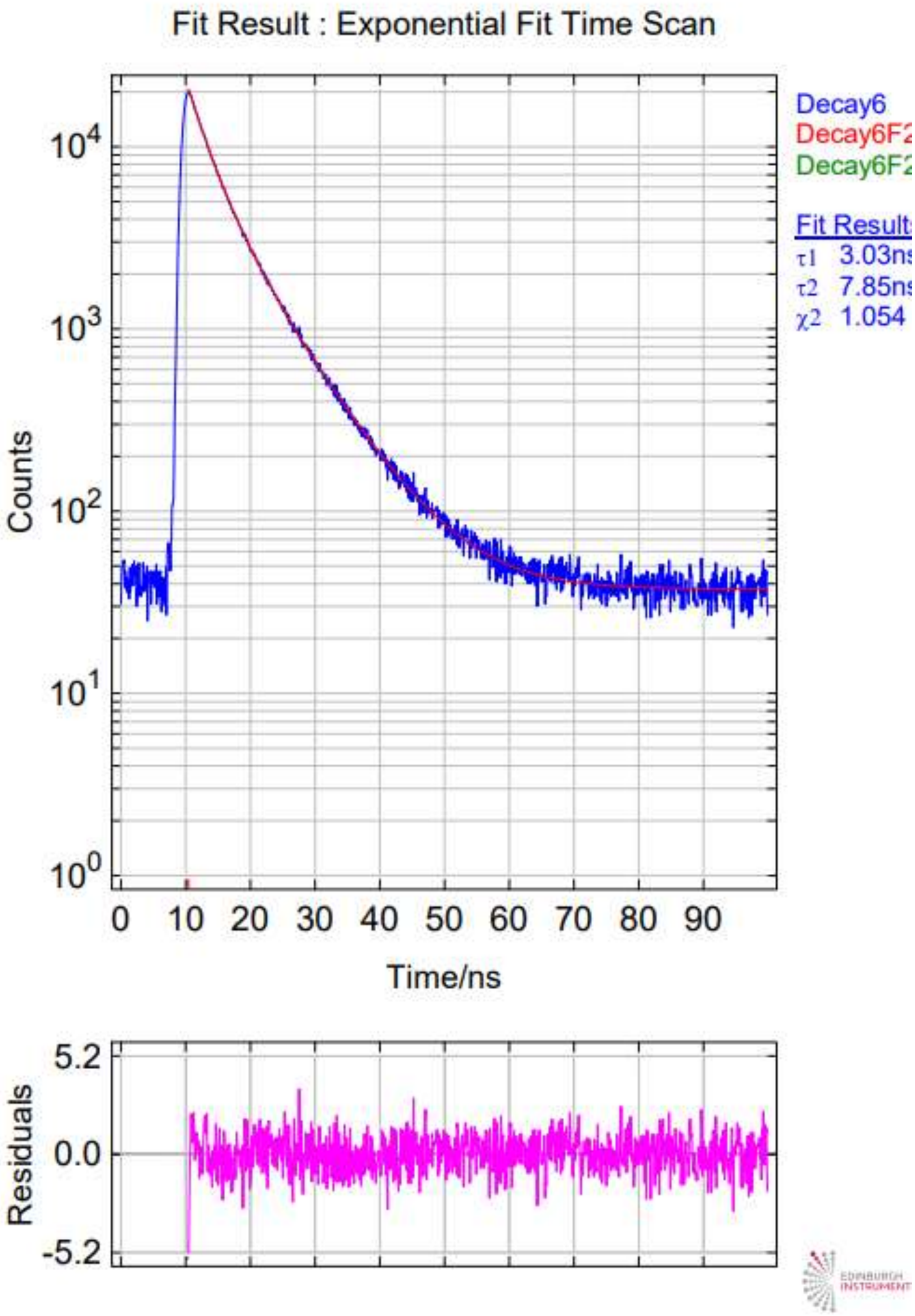

Fig. S77. TCSPC experiments in dichloromethane solution for **2d**. [c] = 1 x 10^{-5} M. λ_{exc} = 365 nm.

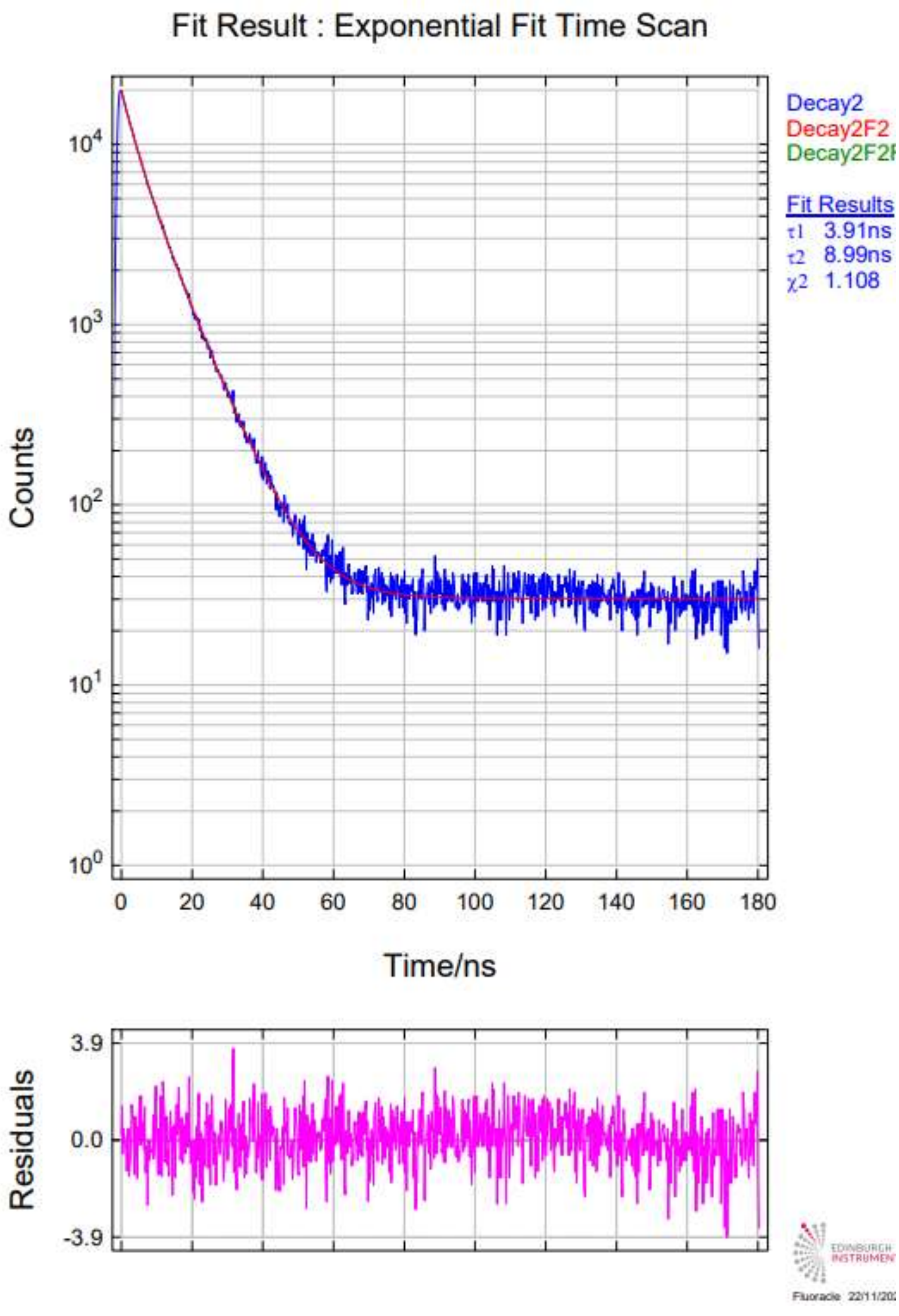

Fig. S78. TCSPC experiments in dichloromethane solution for **2e**. [c] = 1 x 10^{-5} M. λ_{exc} = 365 nm.

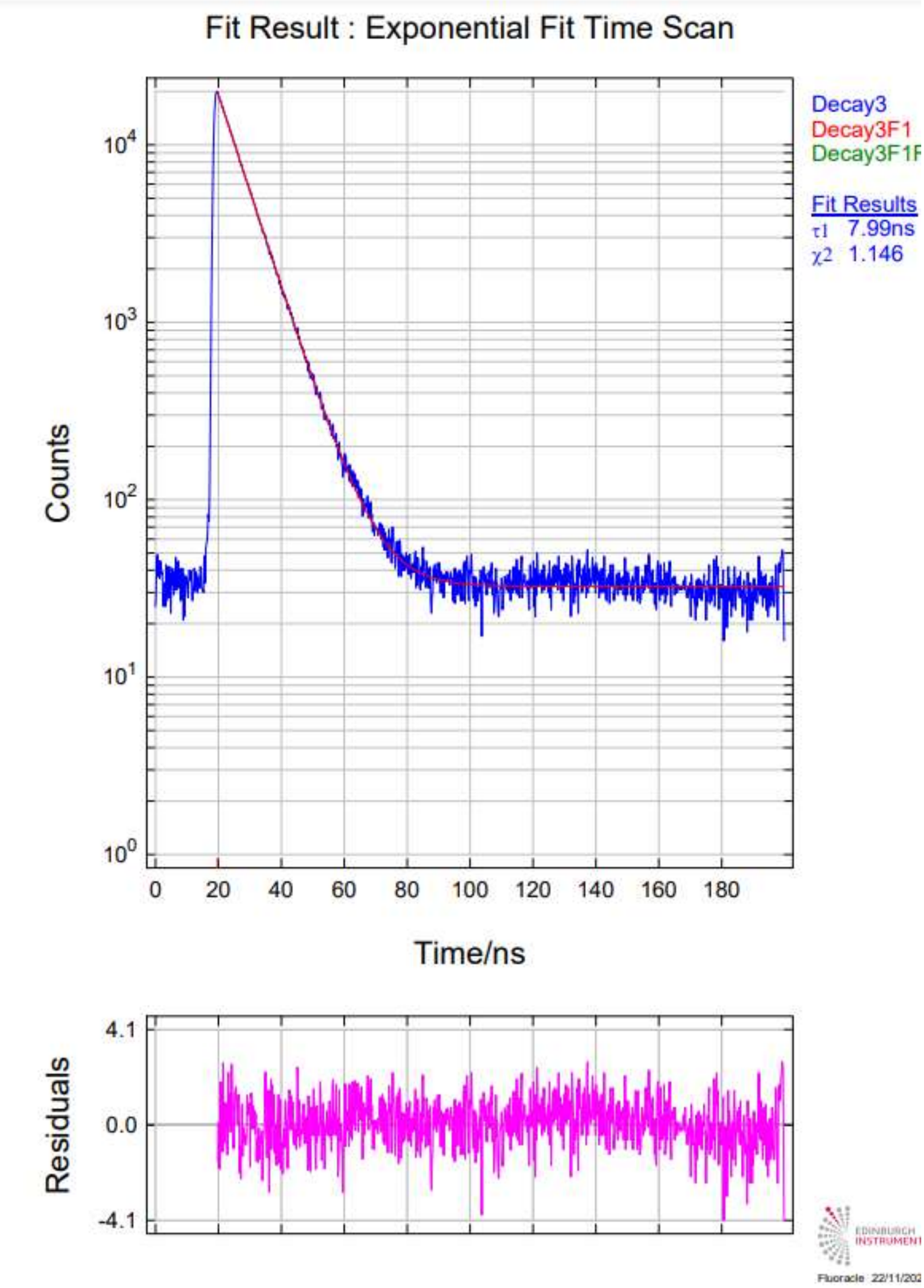

Fig. S79. TCSPC experiments in dichloromethane solution for **2f**. [c] = 1 x 10^{-5} M. λ_{exc} = 365 nm.

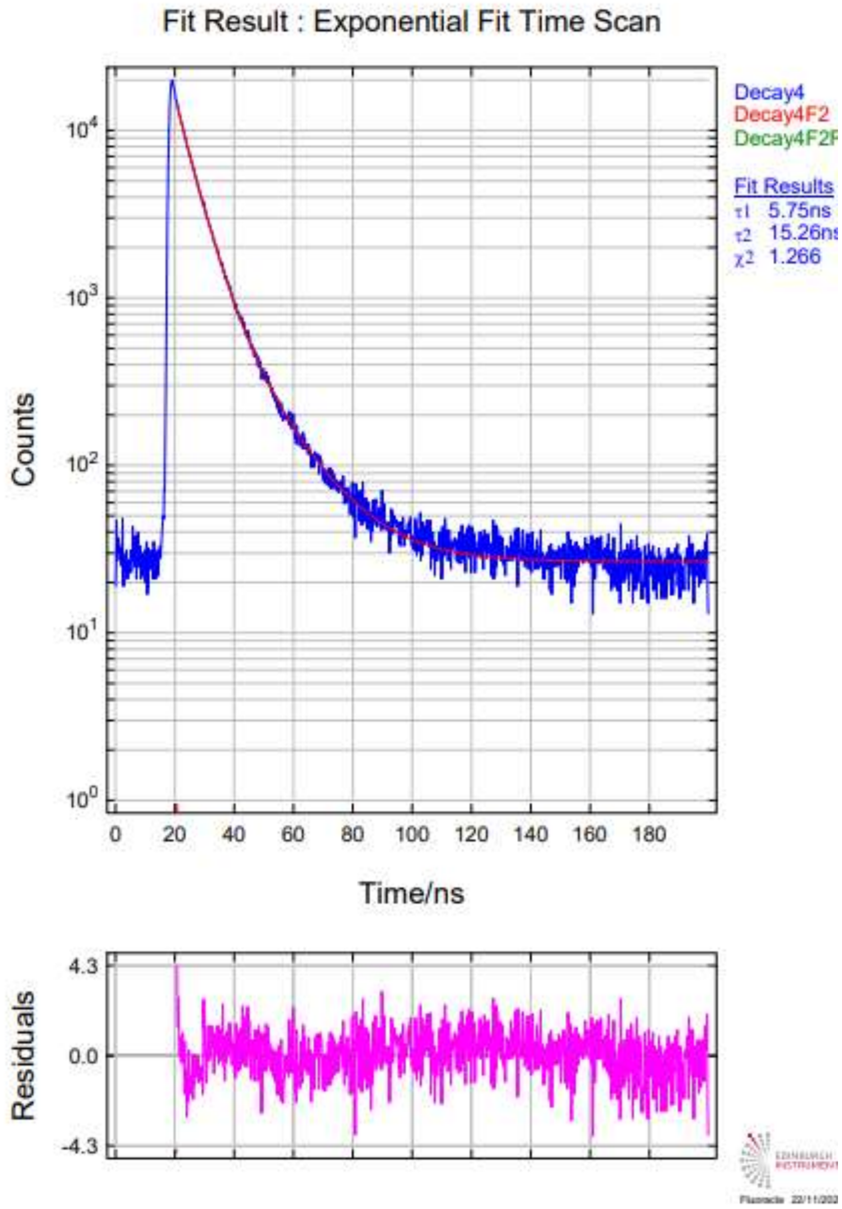

Fig. S80. TCSPC experiments in dichloromethane solution for **2h**. [c] = 1 x 10^{-5} M. λ_{exc} = 365 nm.

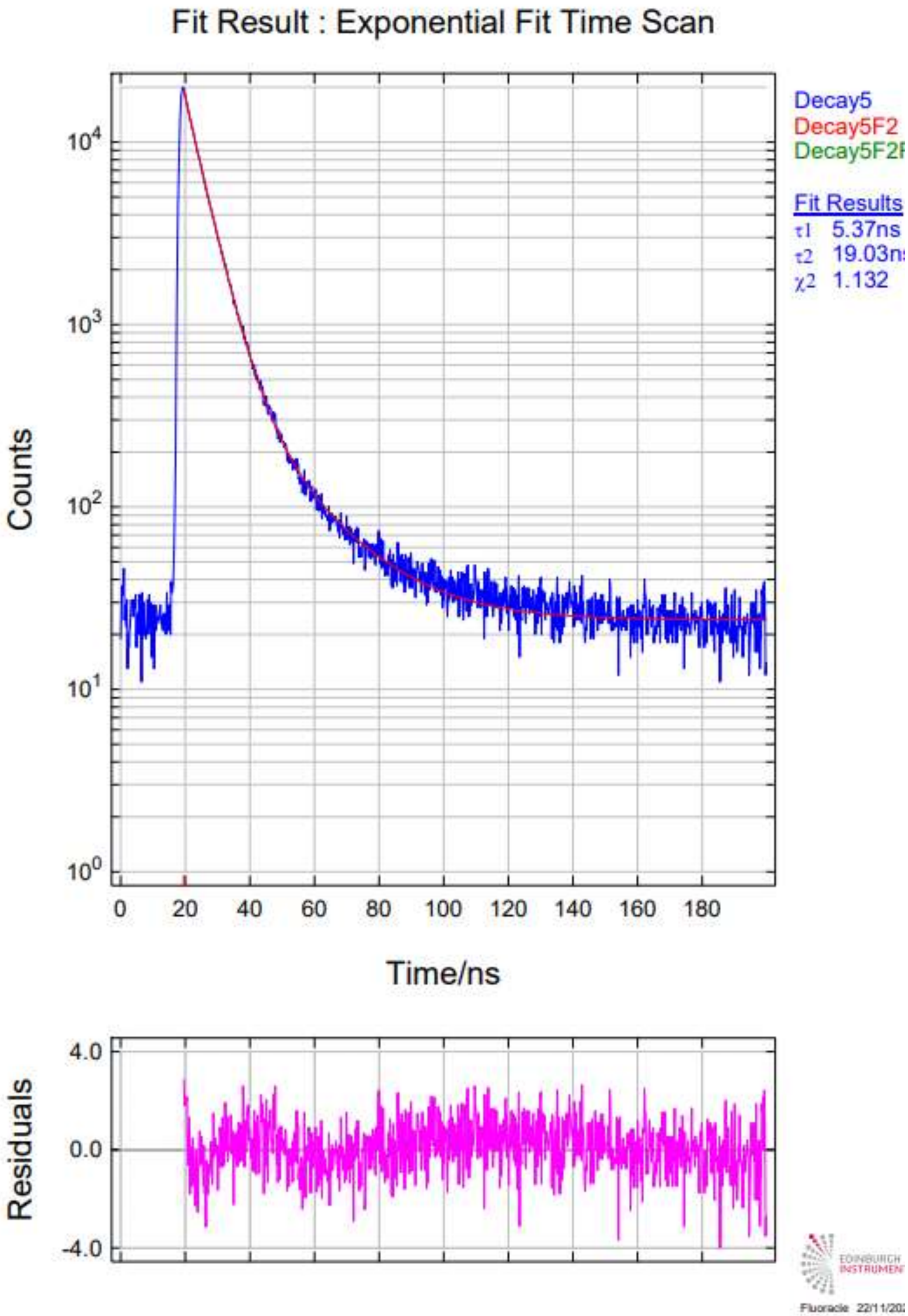

Fig. S81. TCSPC experiments in dichloromethane solution for **2i**. [c] = 1 x 10^{-5} M. λ_{exc} = 365 nm.

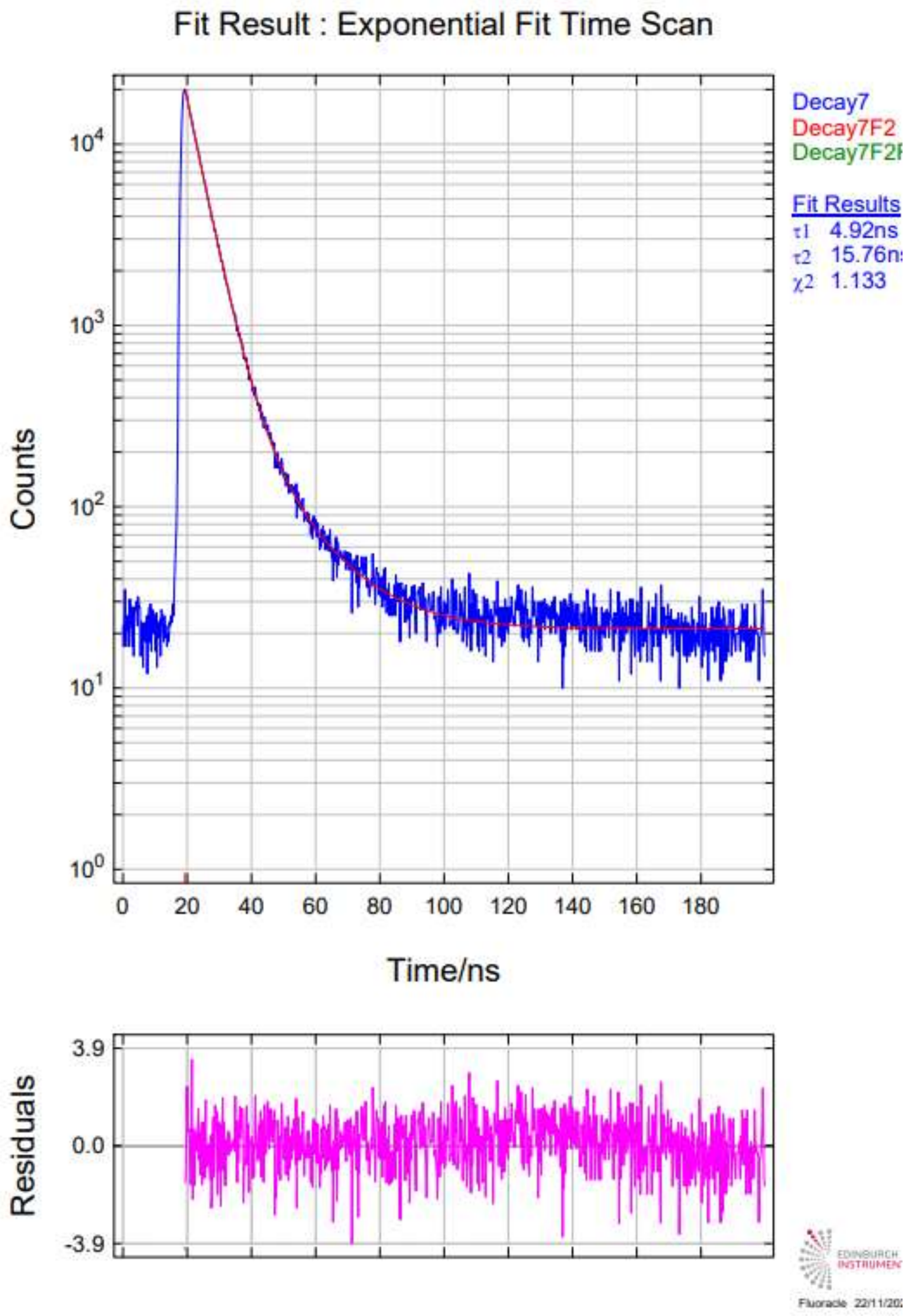

Fig. S82. TCSPC experiments in dichloromethane solution for **2j**. [c] = 1 x 10^{-5} M. λ_{exc} = 365 nm.

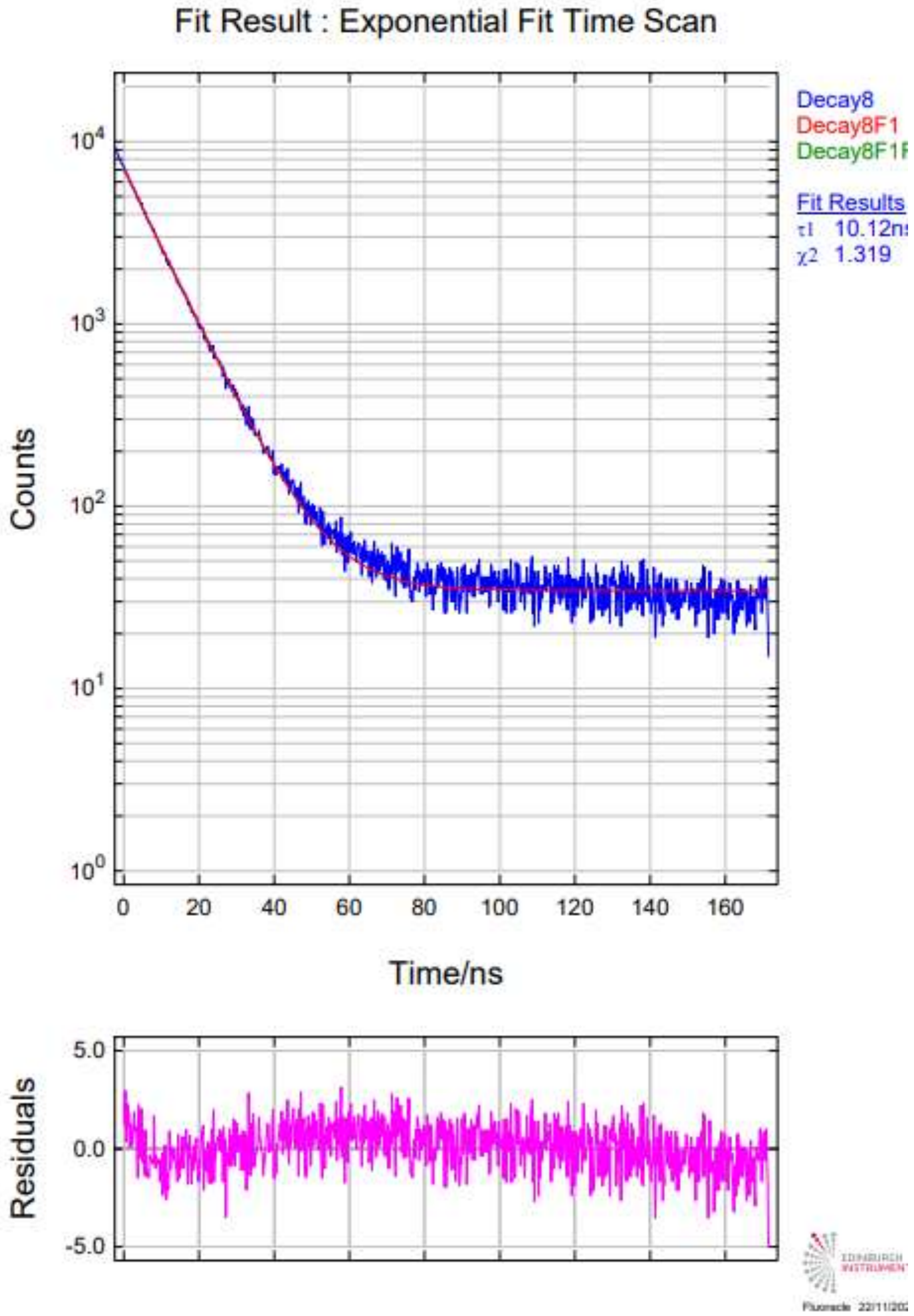

Fig. S83. TCSPC experiments in dichloromethane solution for **2k**. [c] = 1 x 10^{-5} M. λ_{exc} = 365 nm.

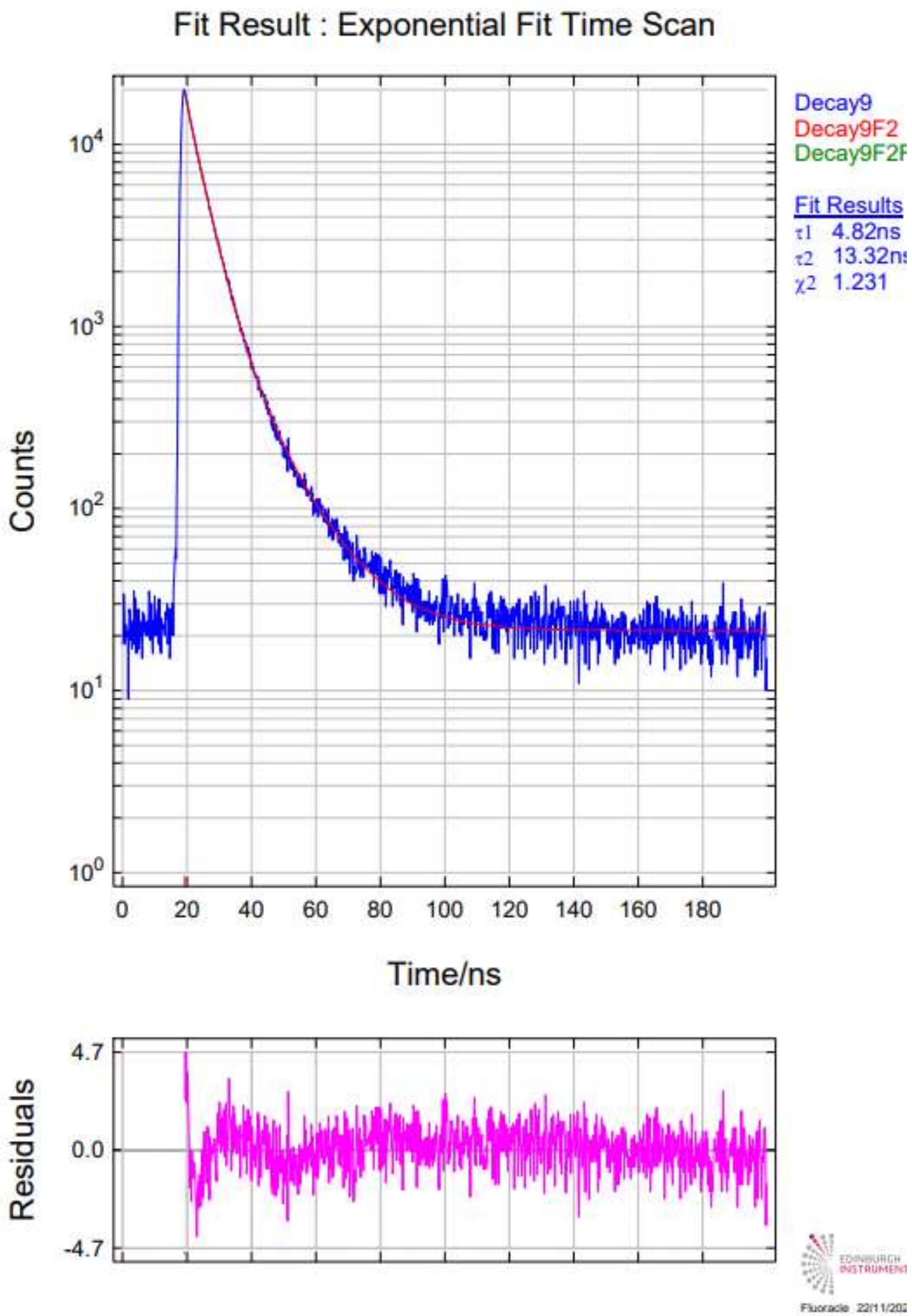

Fig. S84. TCSPC experiments in dichloromethane solution for **2l**. [c] = 1 x 10^{-5} M. λ_{exc} = 365 nm.

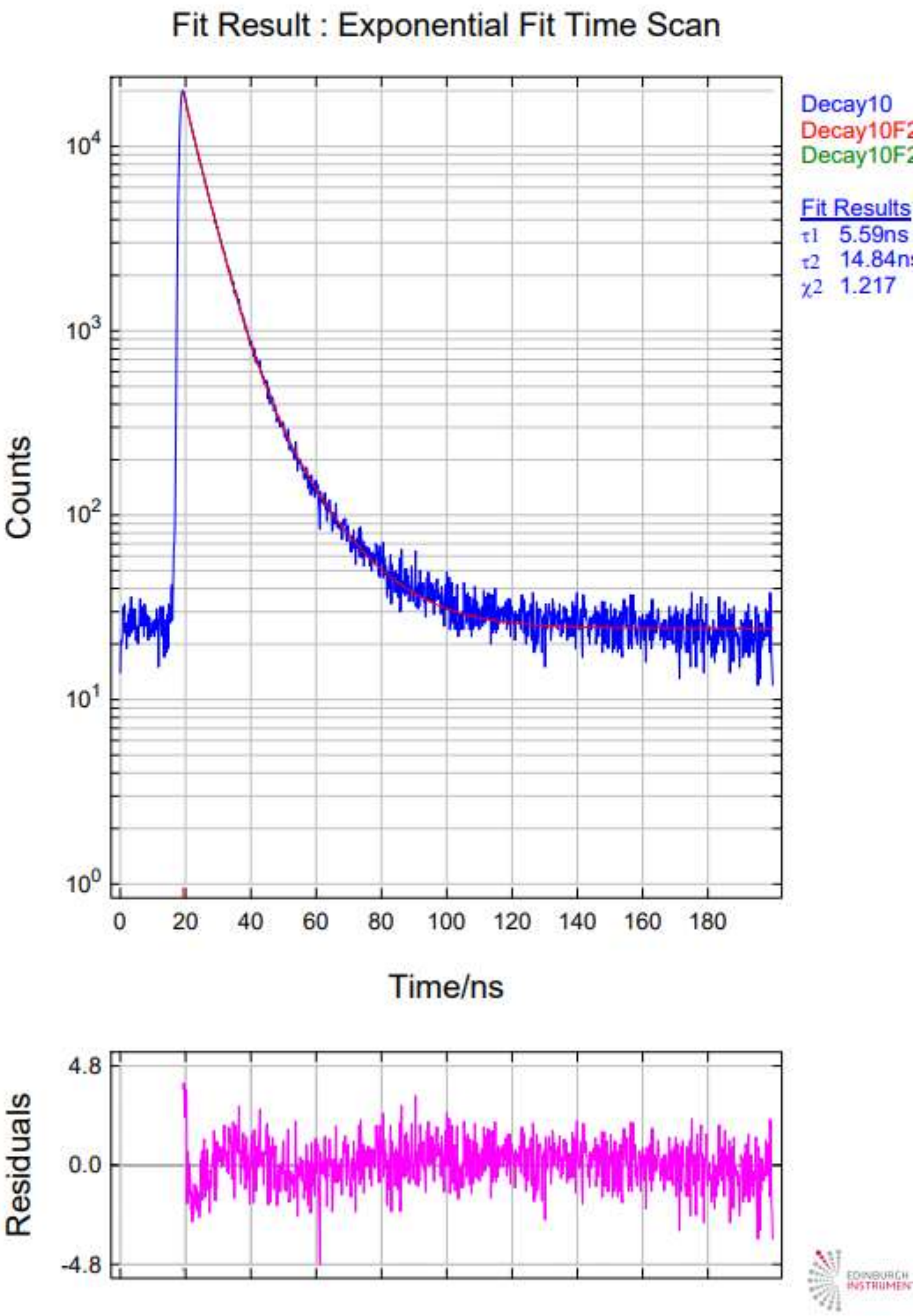

Fig. S85. TCSPC experiments in dichloromethane solution for **2m**. [c] = 1 x 10^{-5} M. λ_{exc} = 365 nm.

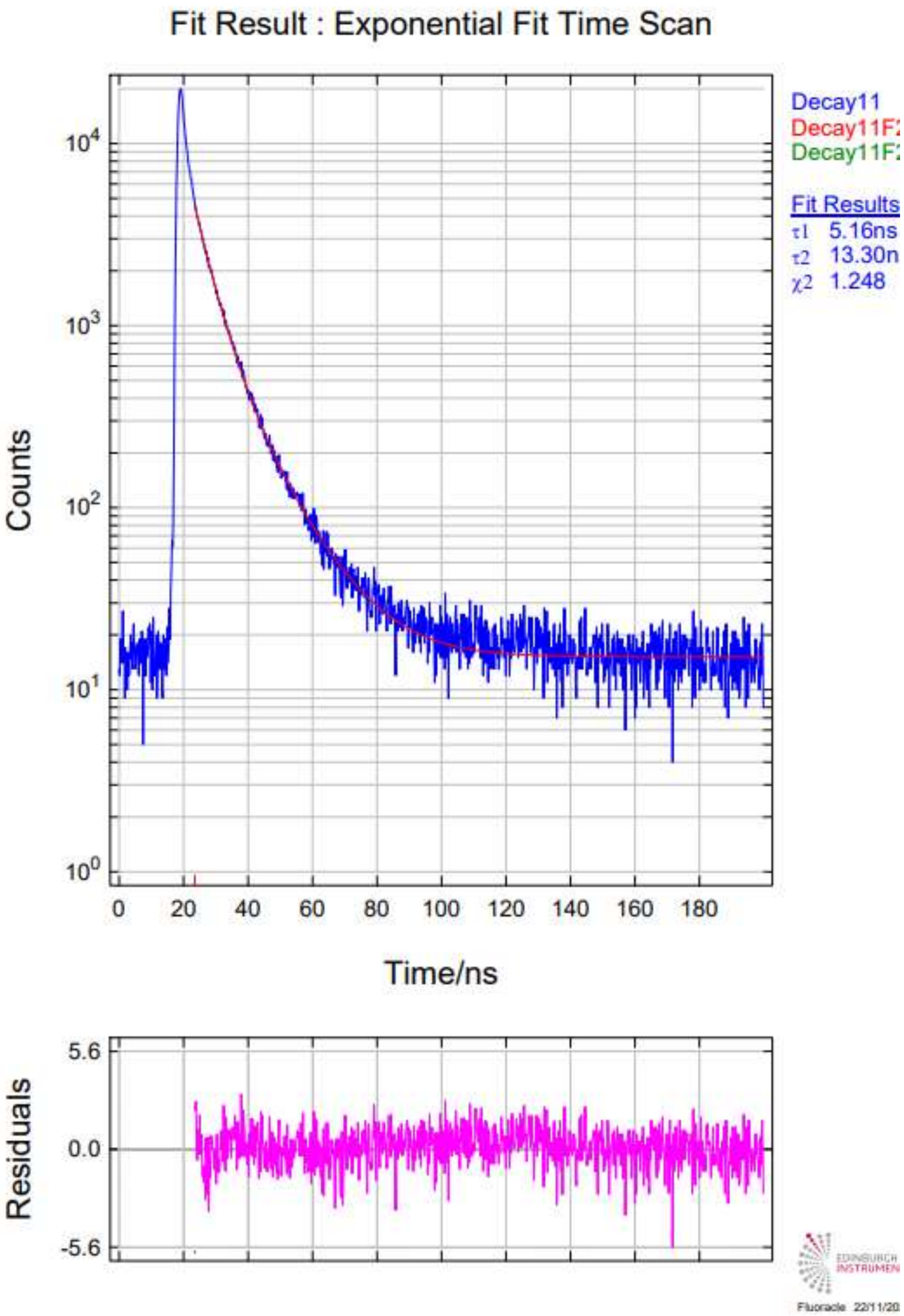

Fig. S86. TCSPC experiments in dichloromethane solution for **2n**. [c] = 1 x 10^{-5} M. λ_{exc} = 365 nm.

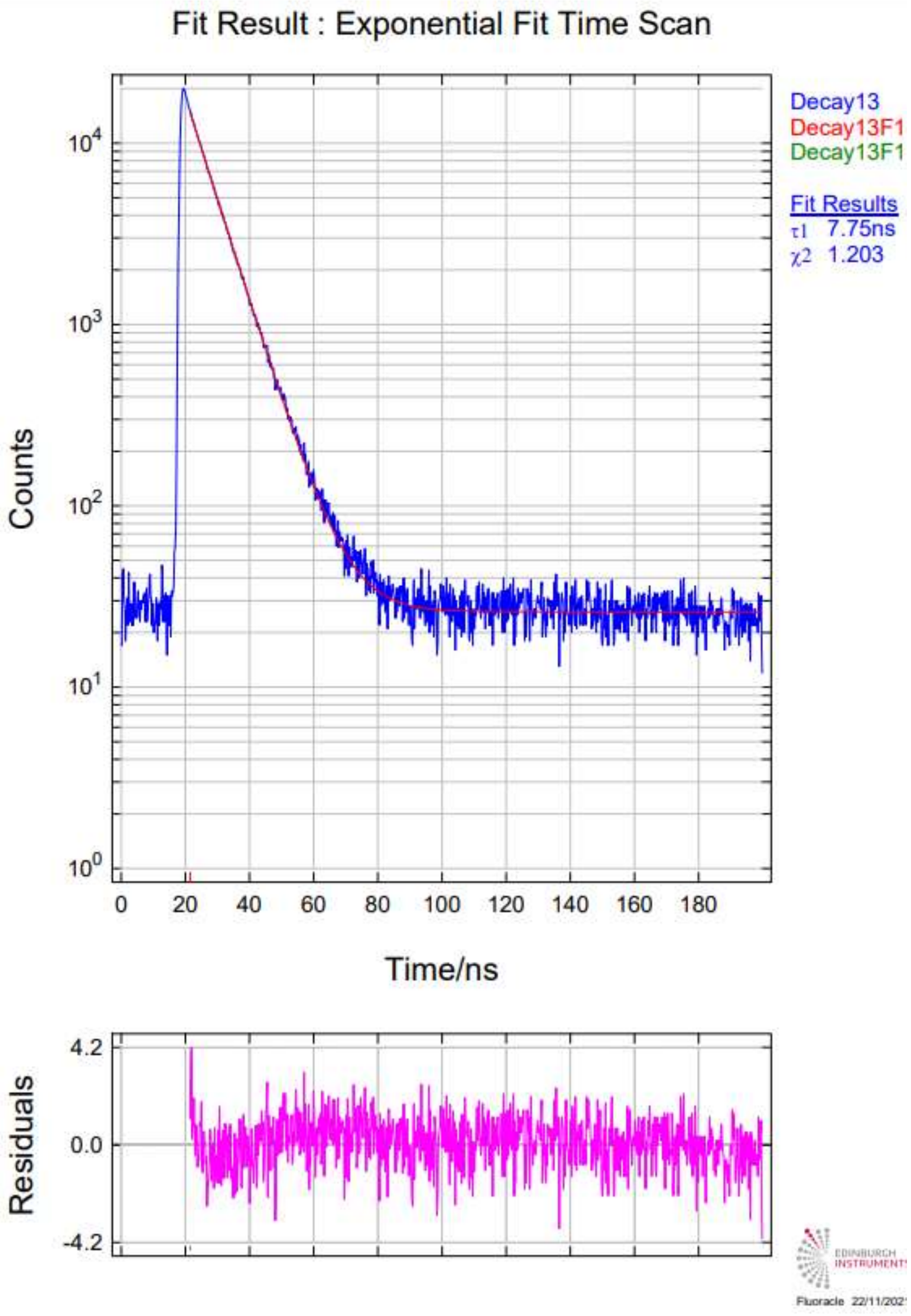

Fig. S87. TCSPC experiments in dichloromethane solution for **2o**. [c] = 1 x 10^{-5} M. λ_{exc} = 365 nm.

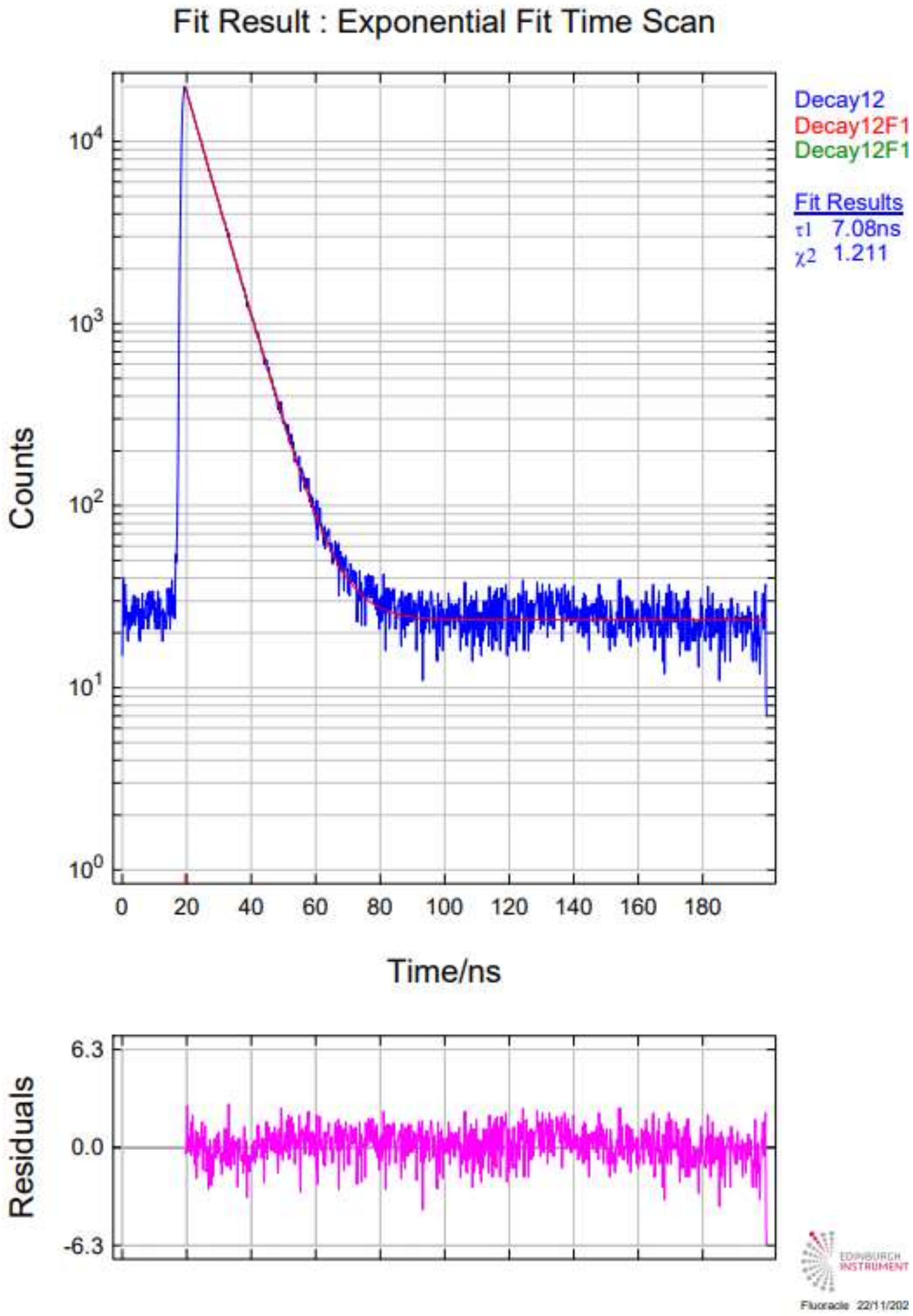

Fig. S88. TCSPC experiments in dichloromethane solution for **2p**. [c] = 1 x 10^{-5} M. λ_{exc} = 365 nm.

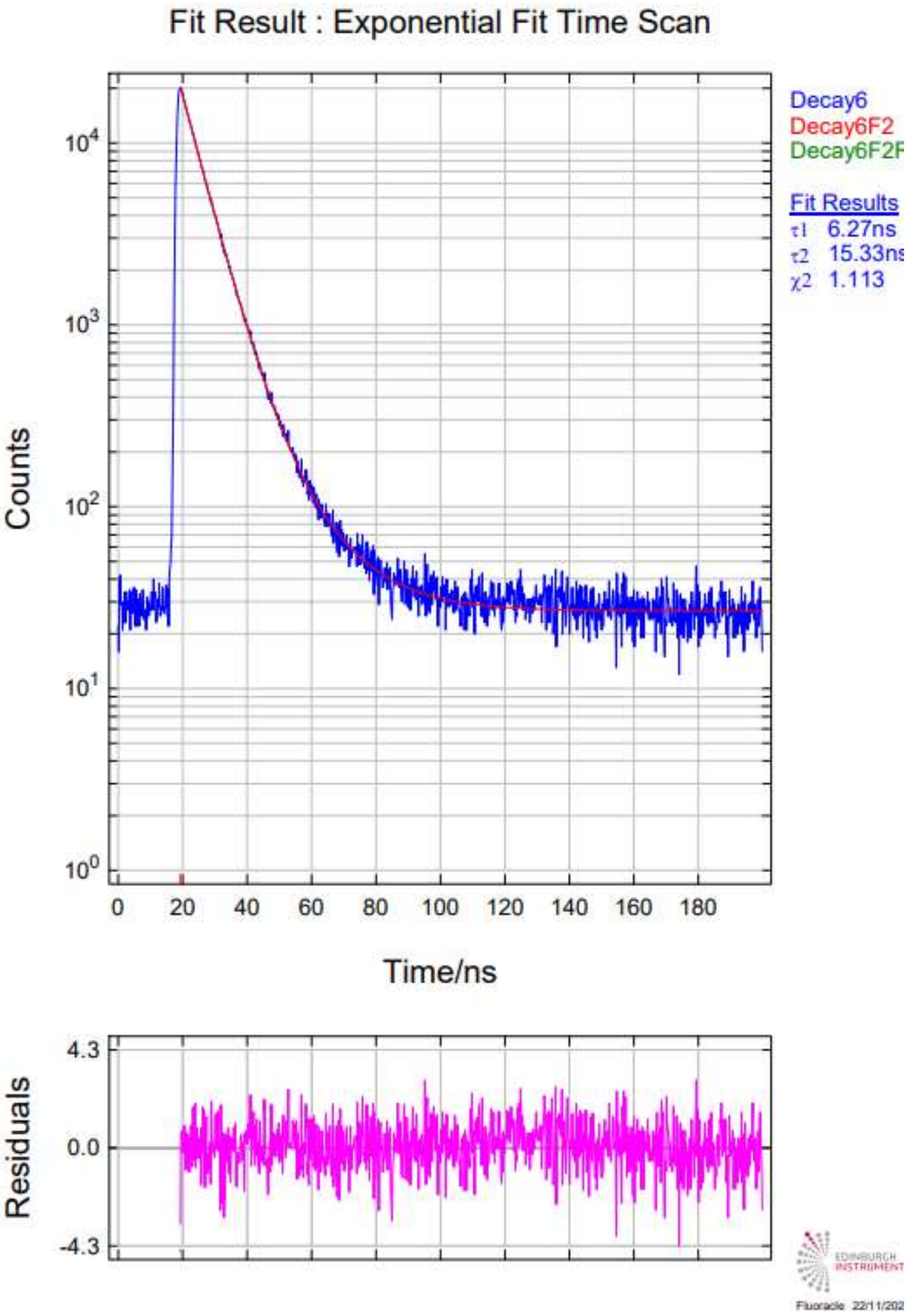

Fig. S89. TCSPC experiments in dichloromethane solution for **2q**. [c] = 1 x 10^{-5} M. λ_{exc} = 365 nm.

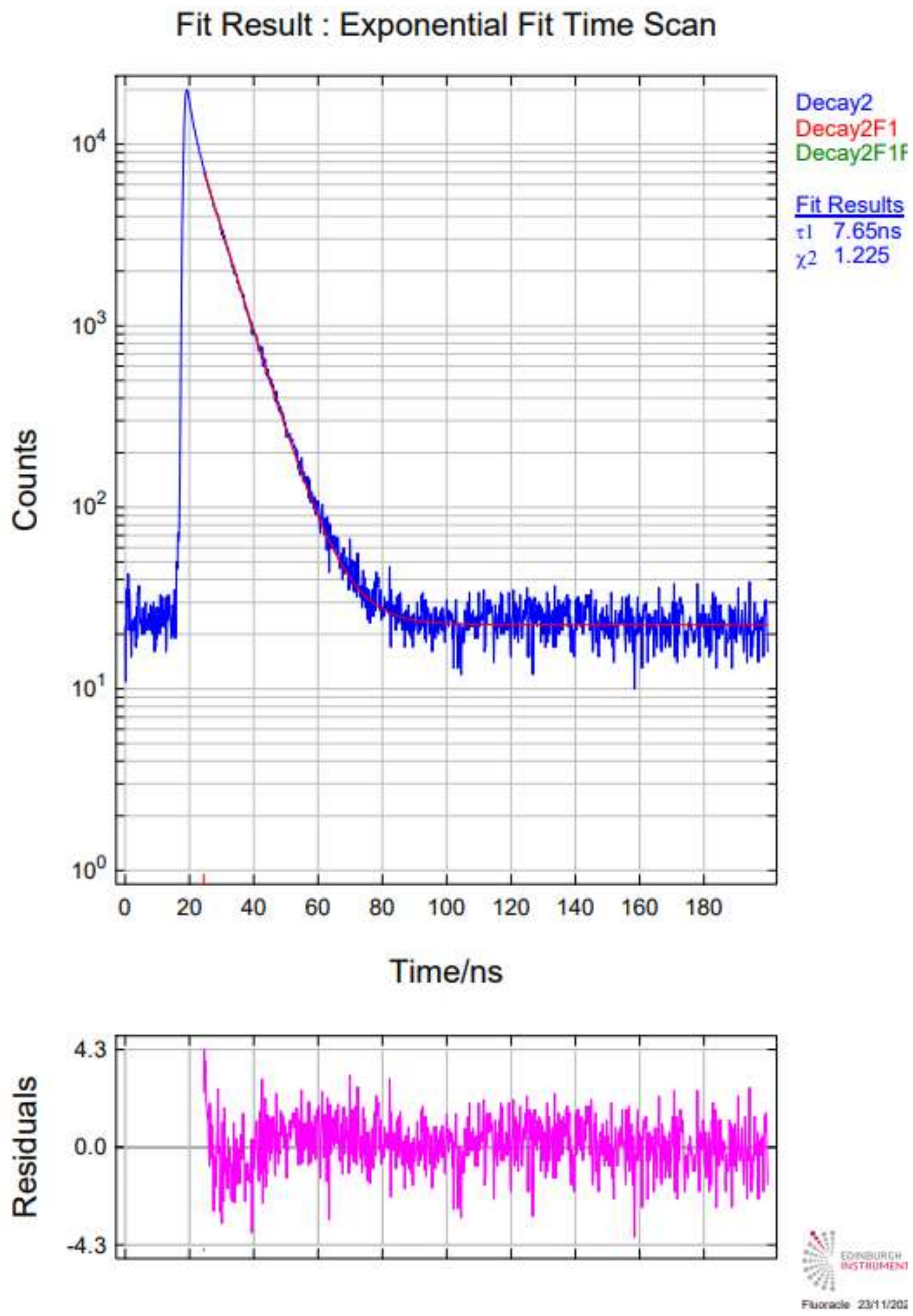

Fig. S90. TCSPC experiments in dichloromethane solution for **2s**. [c] = 1 x 10^{-5} M. λ_{exc} = 365 nm.

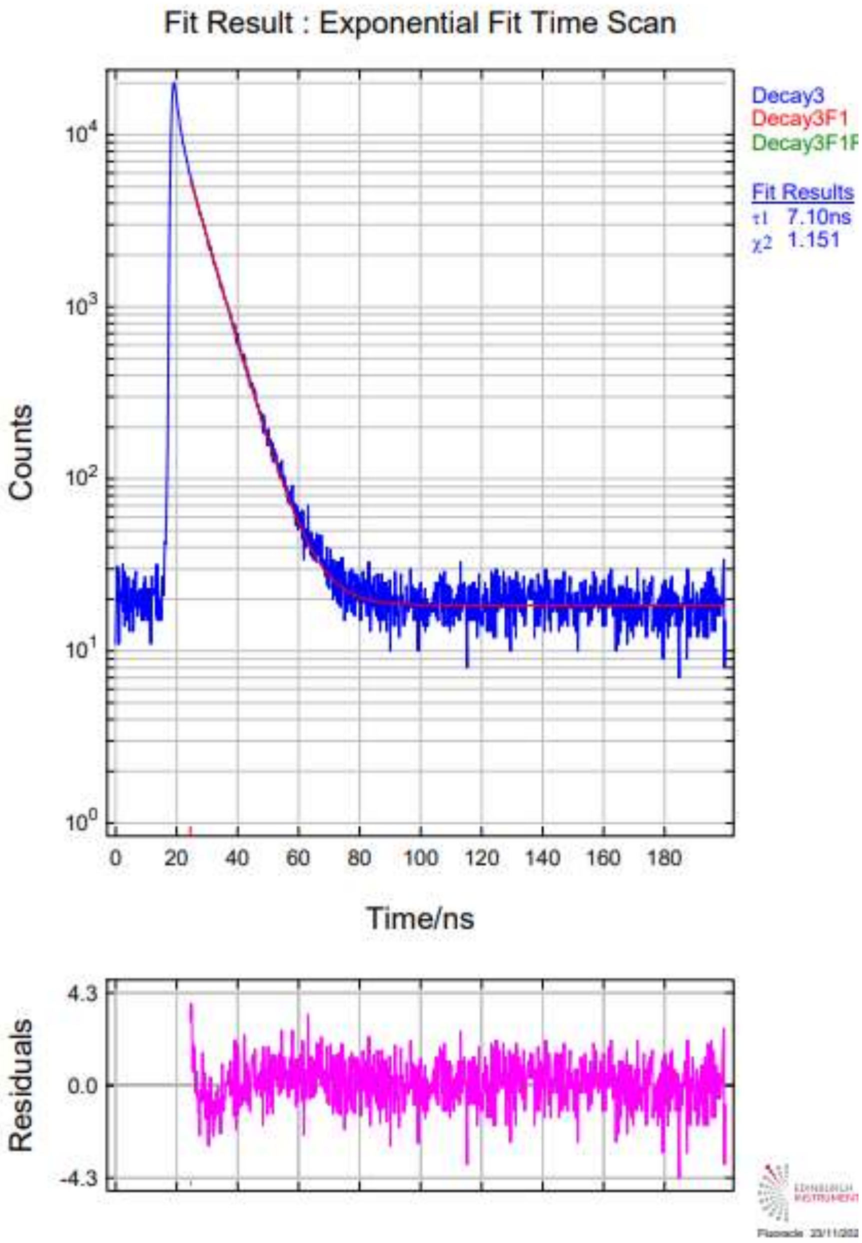

Fig. S91. TCSPC experiments in dichloromethane solution for **2t**. [c] = 1 x 10^{-5} M. λ_{exc} = 365 nm.

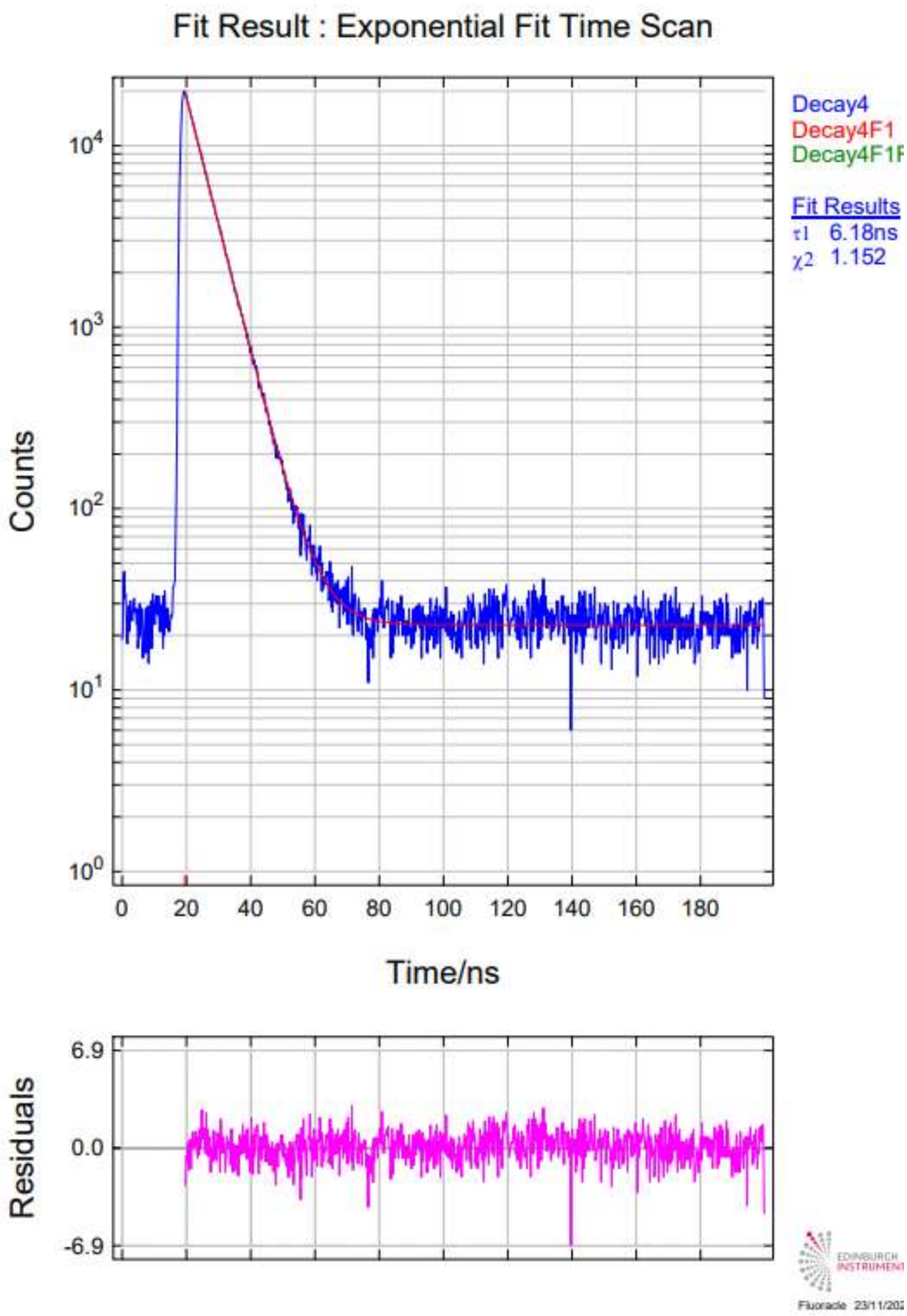

Fig. S92. TCSPC experiments in dichloromethane solution for **2u**. [c] = 1 x 10^{-5} M. λ_{exc} = 365 nm.

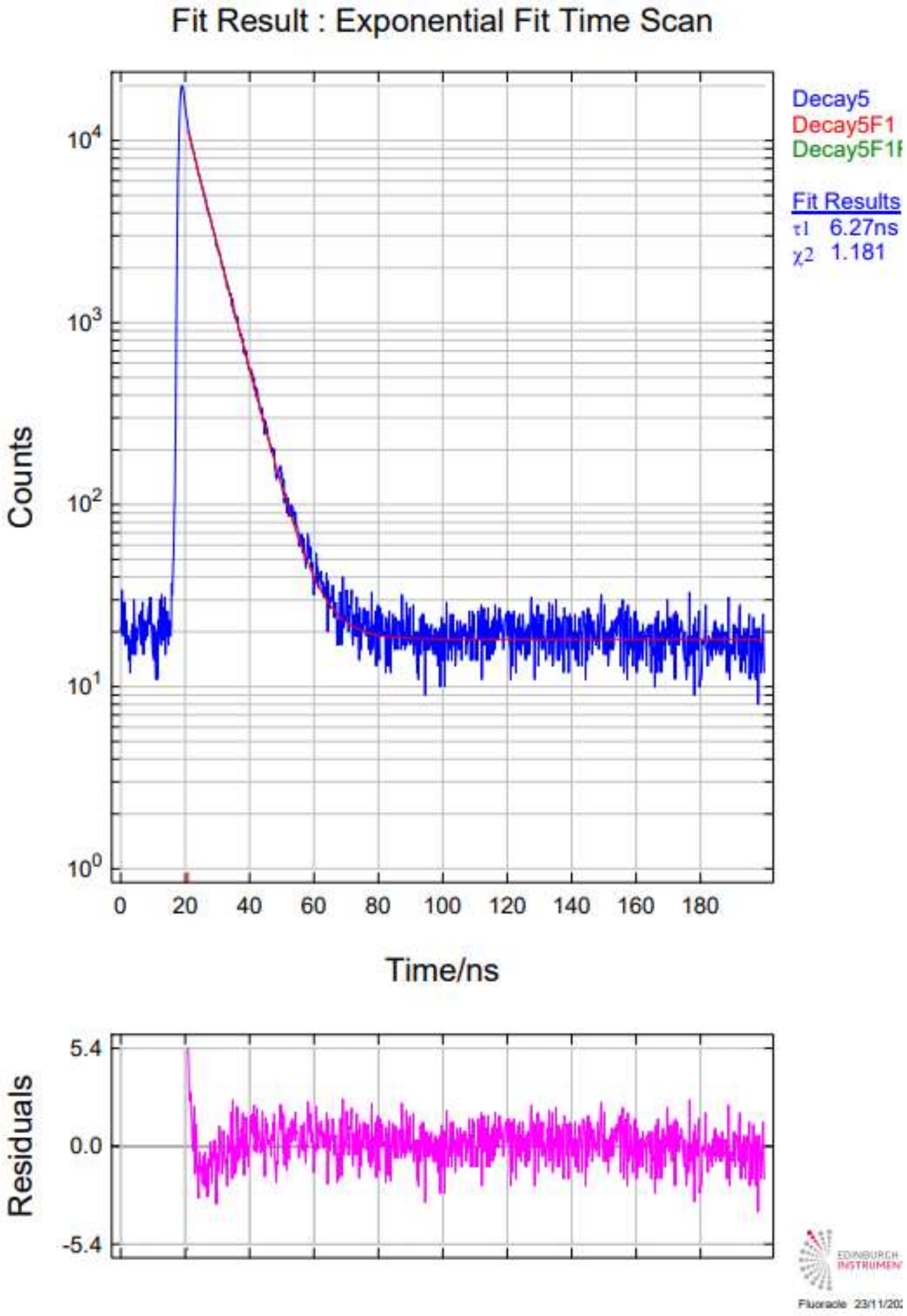

Fig. S93. TCSPC experiments in dichloromethane solution for **2v**. [c] = 1 x 10^{-5} M. λ_{exc} = 365 nm.

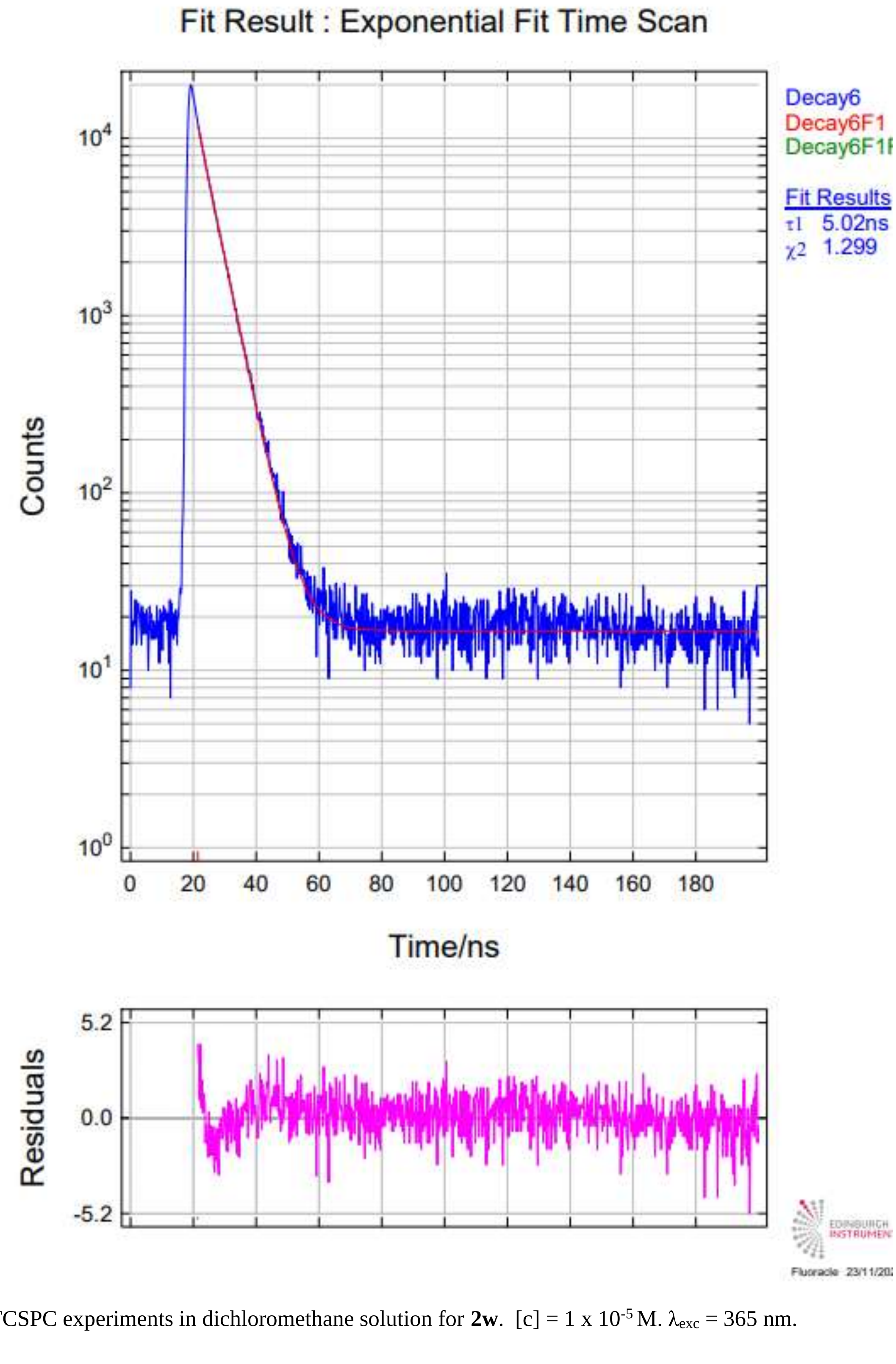

Fig. S94. TCSPC experiments in dichloromethane solution for **2w**. [c] = 1 x 10^{-5} M. λ_{exc} = 365 nm.

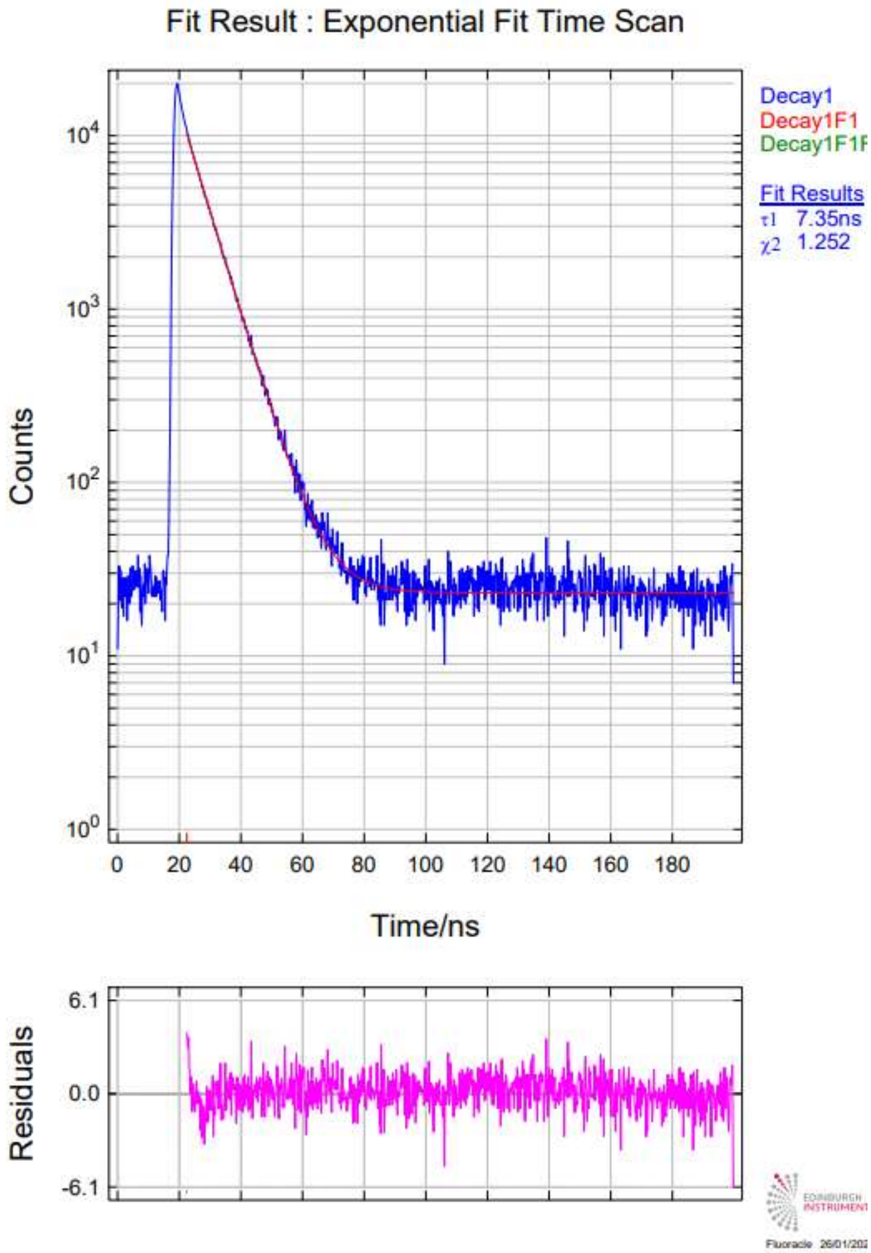

Fig. S95. TCSPC experiments in dichloromethane solution for **2x**. [c] = 1 x 10^{-5} M. λ_{exc} = 365 nm.

Quantum yield measurements:

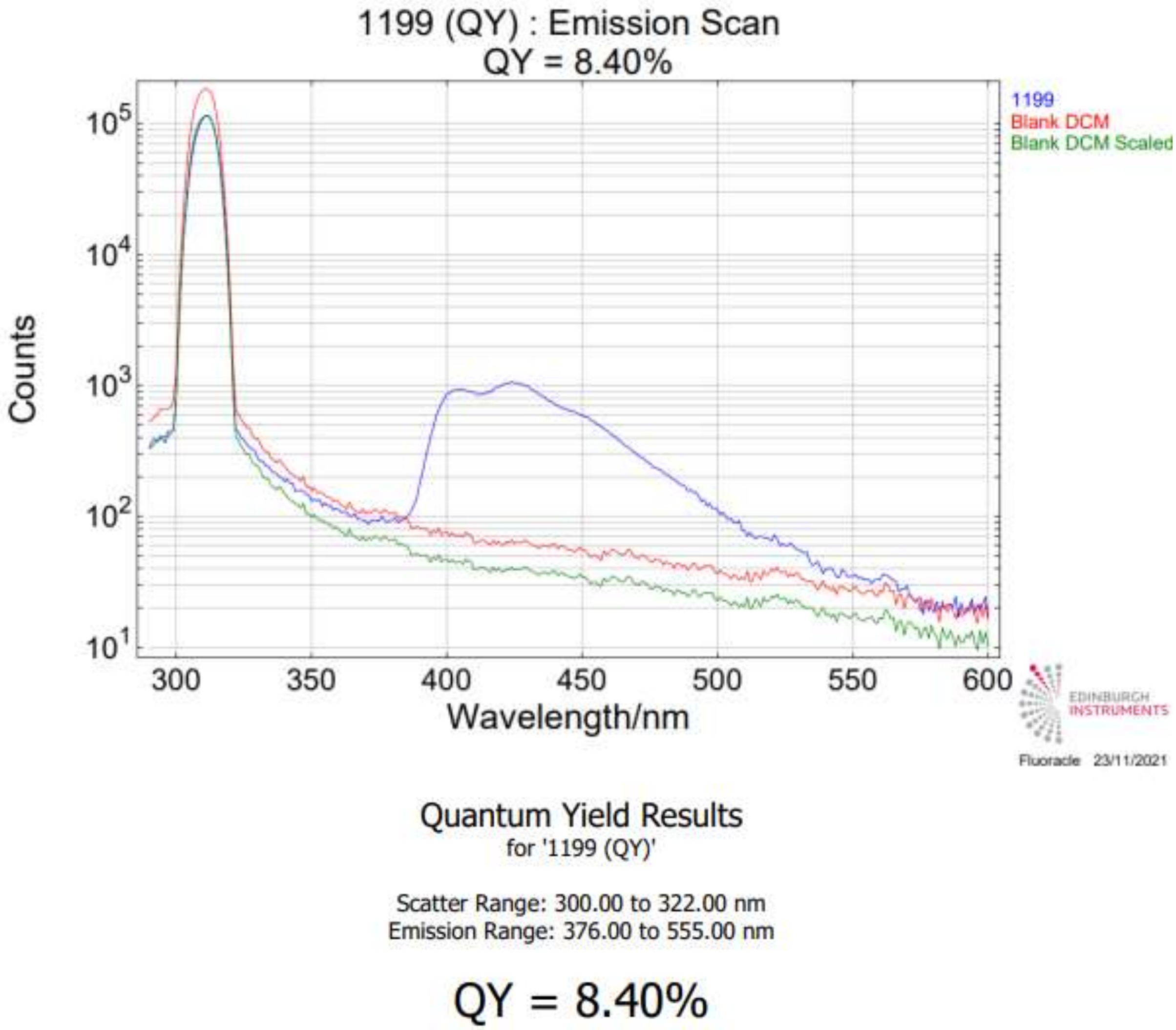

Fig. S96. Quantum yield measurement in dichloromethane solution for **2a**. [c] = 1 x 10^{-5} M.

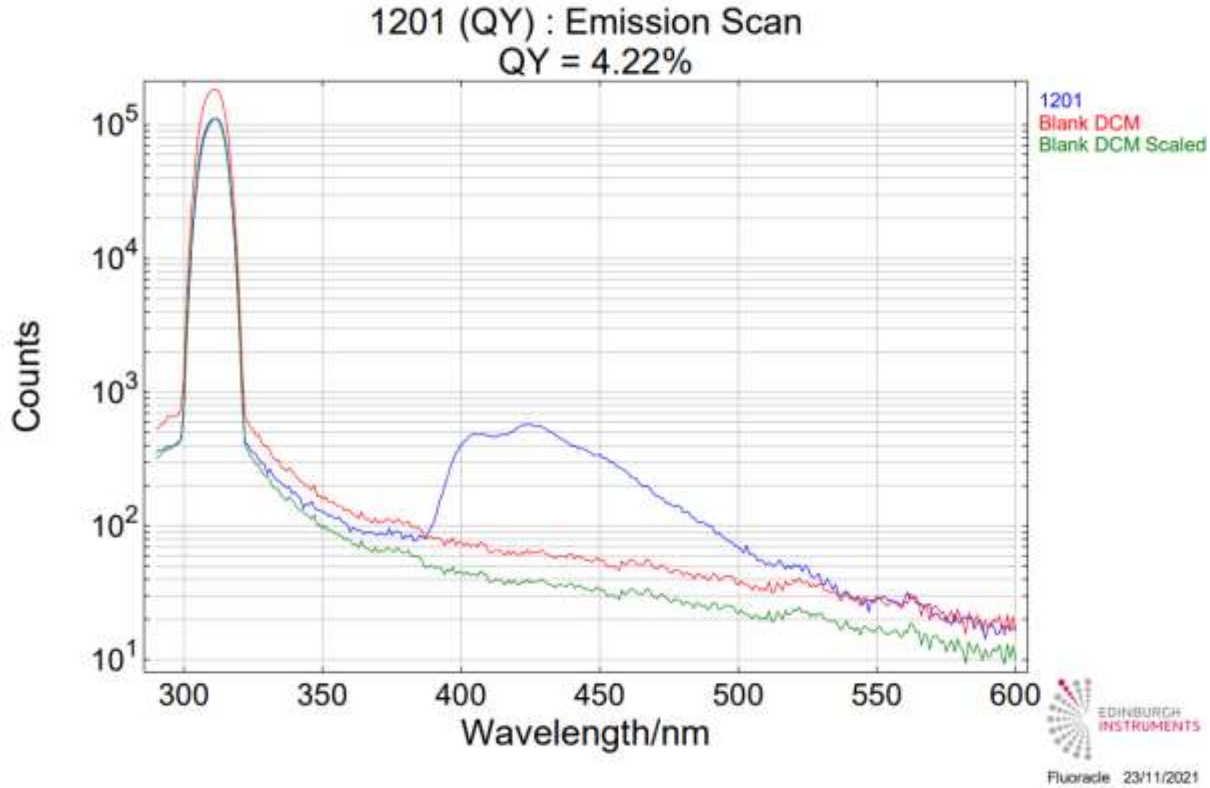

Quantum Yield Results
for '1201 (QY)'

Scatter Range: 299.00 to 321.00 nm
Emission Range: 377.00 to 566.00 nm

QY = 4.22%

Fig. S97. Quantum yield measurement in dichloromethane solution for **2b**. [c] = 1 x 10^{-5} M.

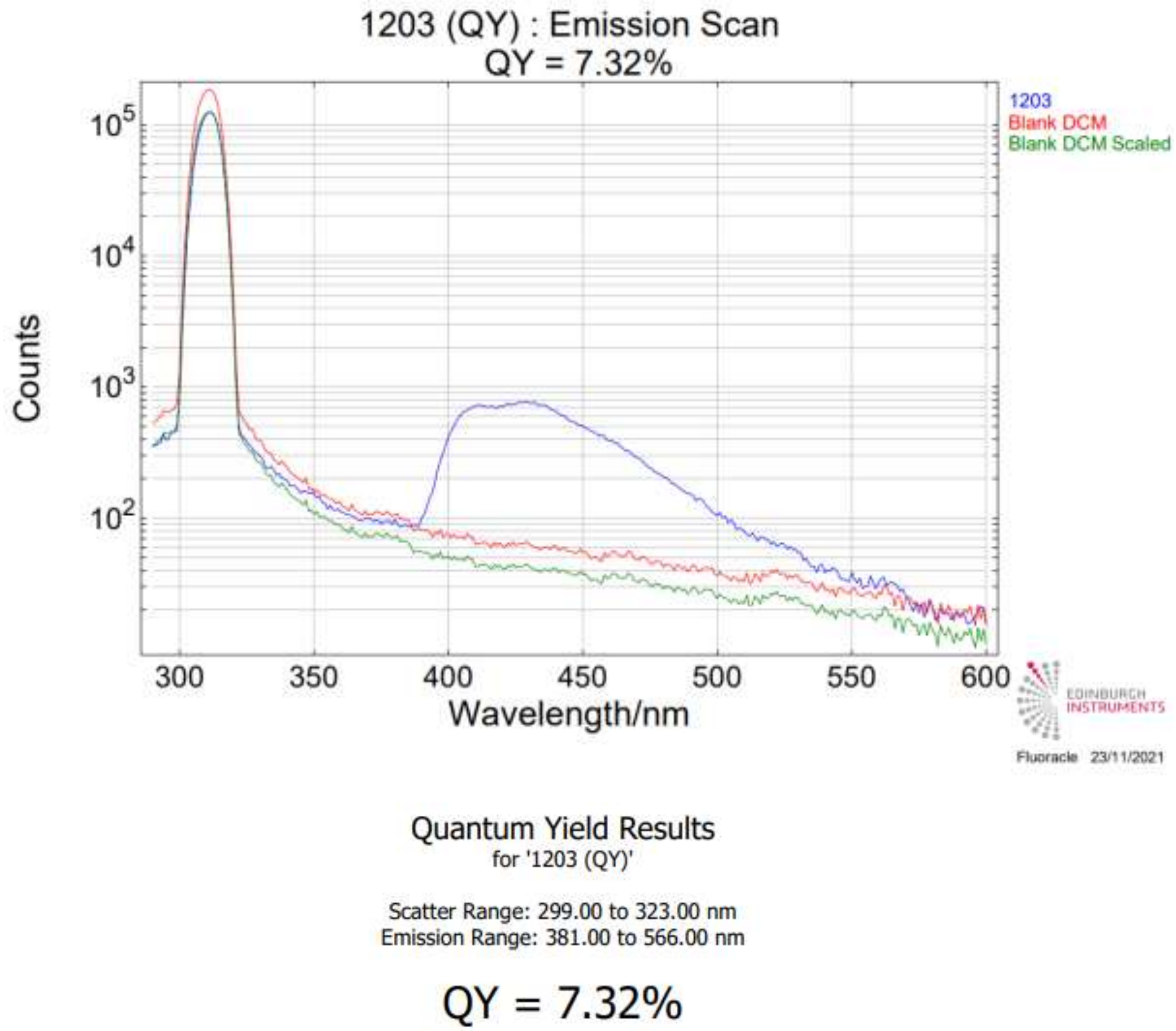

Fig. S98. Quantum yield measurement in dichloromethane solution for **2c**. [c] = 1 x 10^{-5} M.

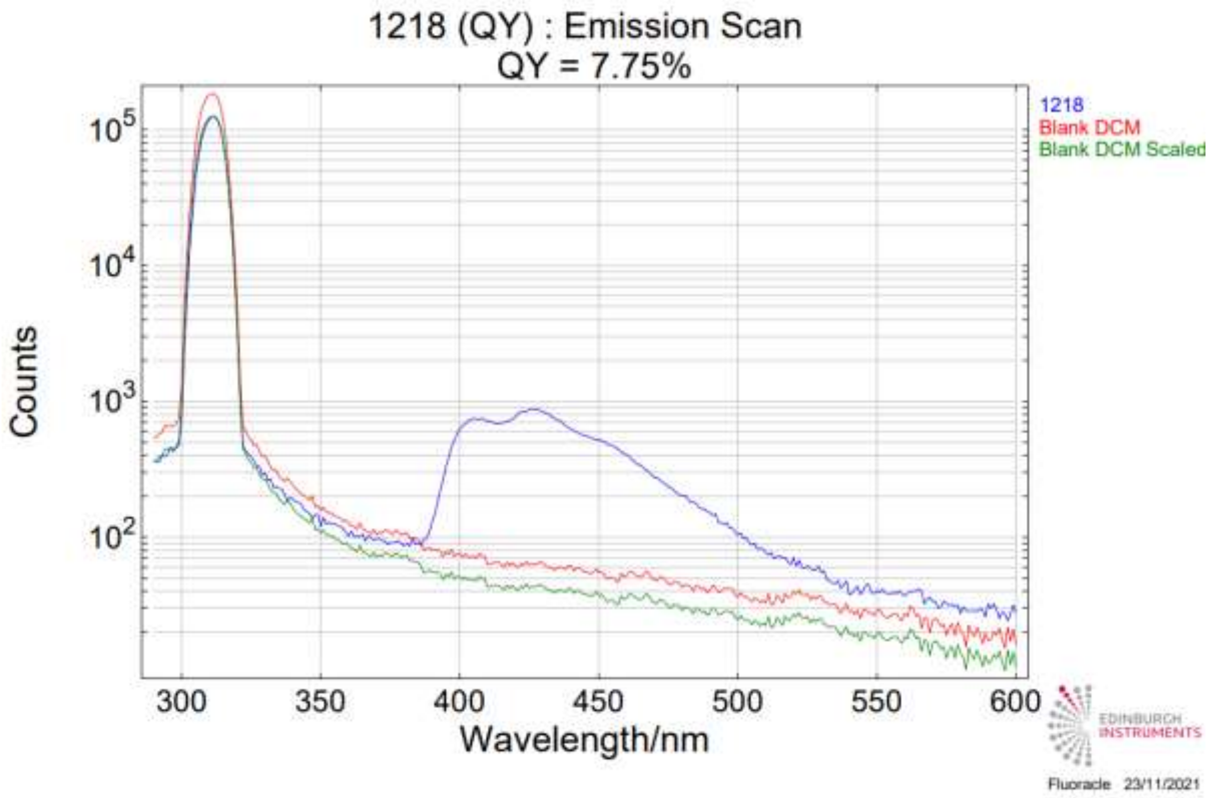

Quantum Yield Results
for '1218 (QY)'

Scatter Range: 300.00 to 323.00 nm
Emission Range: 381.00 to 544.00 nm

QY = 7.75%

Fig. S99. Quantum yield measurement in dichloromethane solution for **2d.** [c] = 1 x 10^{-5} M.

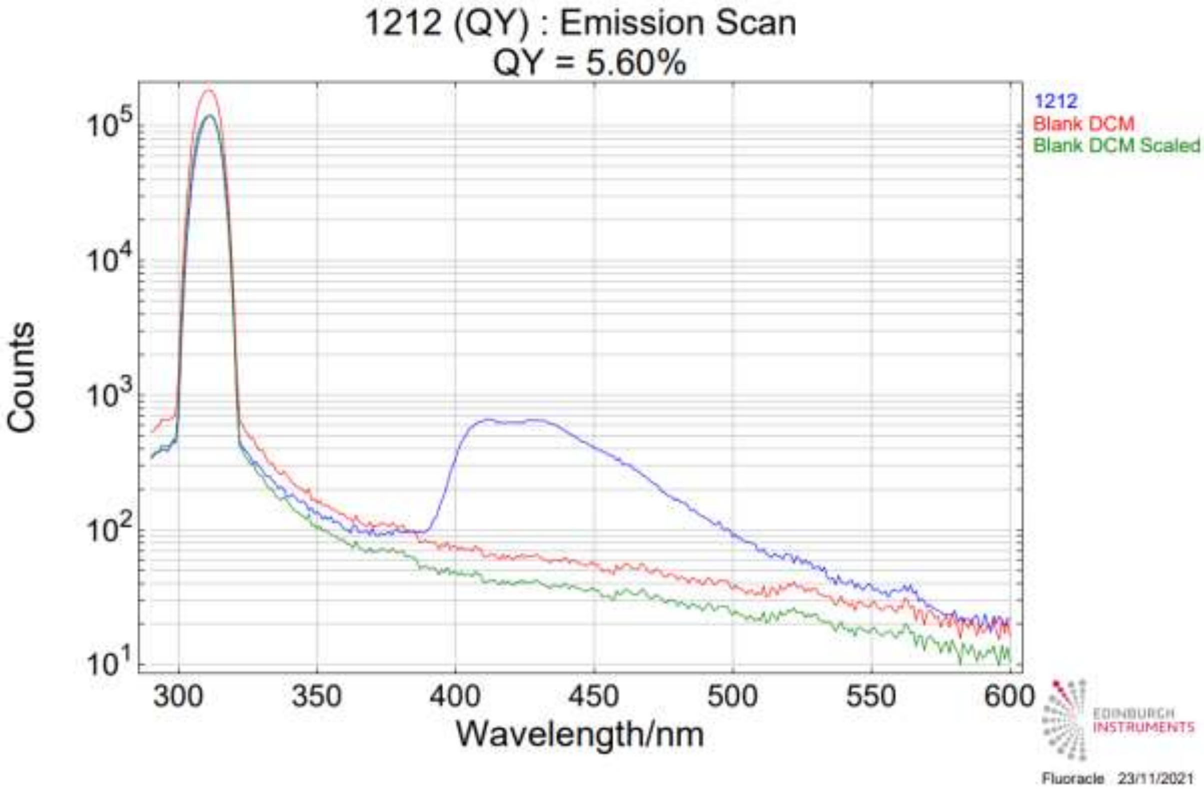

Quantum Yield Results
for '1212 (QY)'

Scatter Range: 300.00 to 321.00 nm
Emission Range: 382.00 to 563.00 nm

QY = 5.60%

Fig. S100. Quantum yield measurement in dichloromethane solution for **2e.** [c] = 1 x 10^{-5} M.

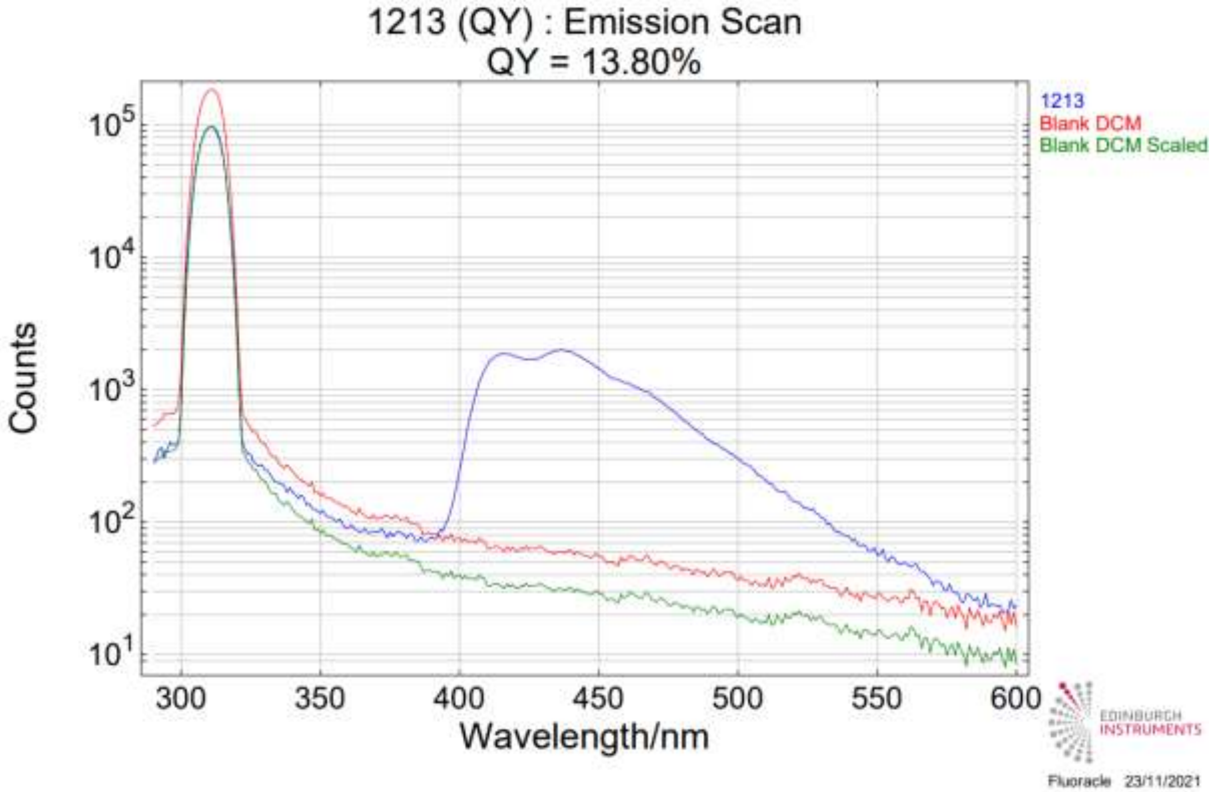

Quantum Yield Results

for '1213 (QY)'

Scatter Range: 300.00 to 322.00 nm
Emission Range: 390.00 to 576.00 nm

QY = 13.80%

Fig. S101. Quantum yield measurement in dichloromethane solution for **2f.** [c] = 1 x 10^{-5} M.

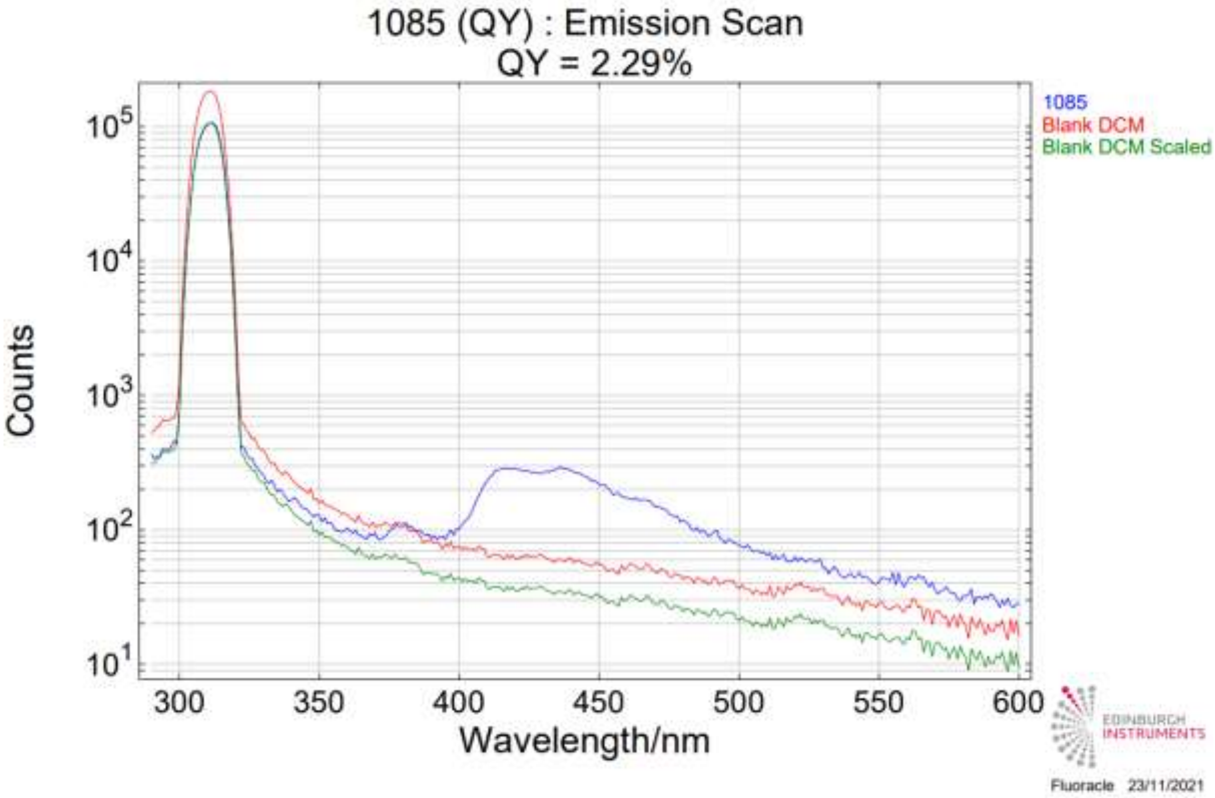

Quantum Yield Results
for '1085 (QY)'

Scatter Range: 299.00 to 321.00 nm
Emission Range: 394.00 to 542.00 nm

QY = 2.29%

Fig. S102. Quantum yield measurement in dichloromethane solution for **2h**. [c] = 1 x 10^{-5} M.

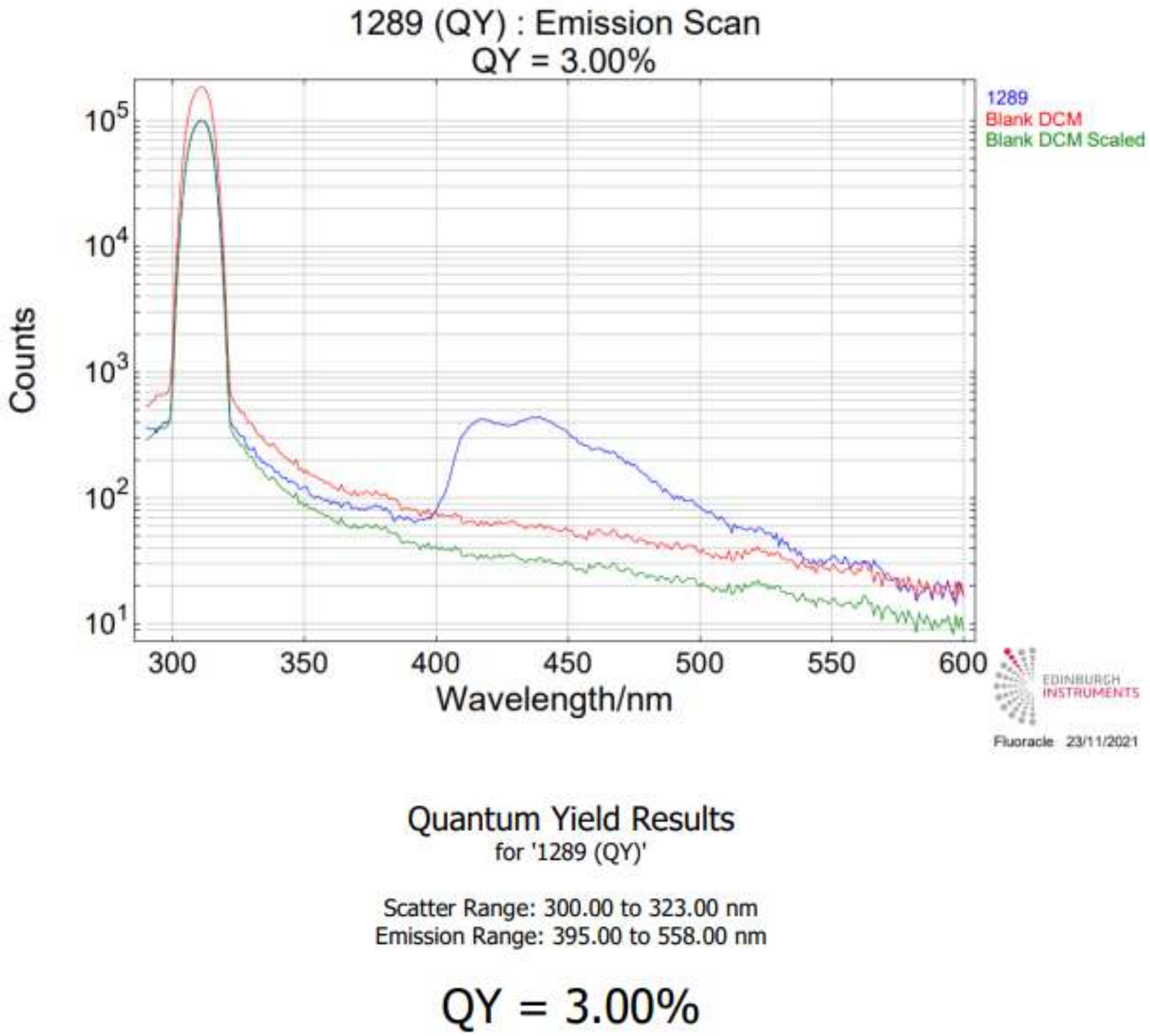

Fig. S103. Quantum yield measurement in dichloromethane solution for **2i.** [c] = 1 x 10^{-5} M.

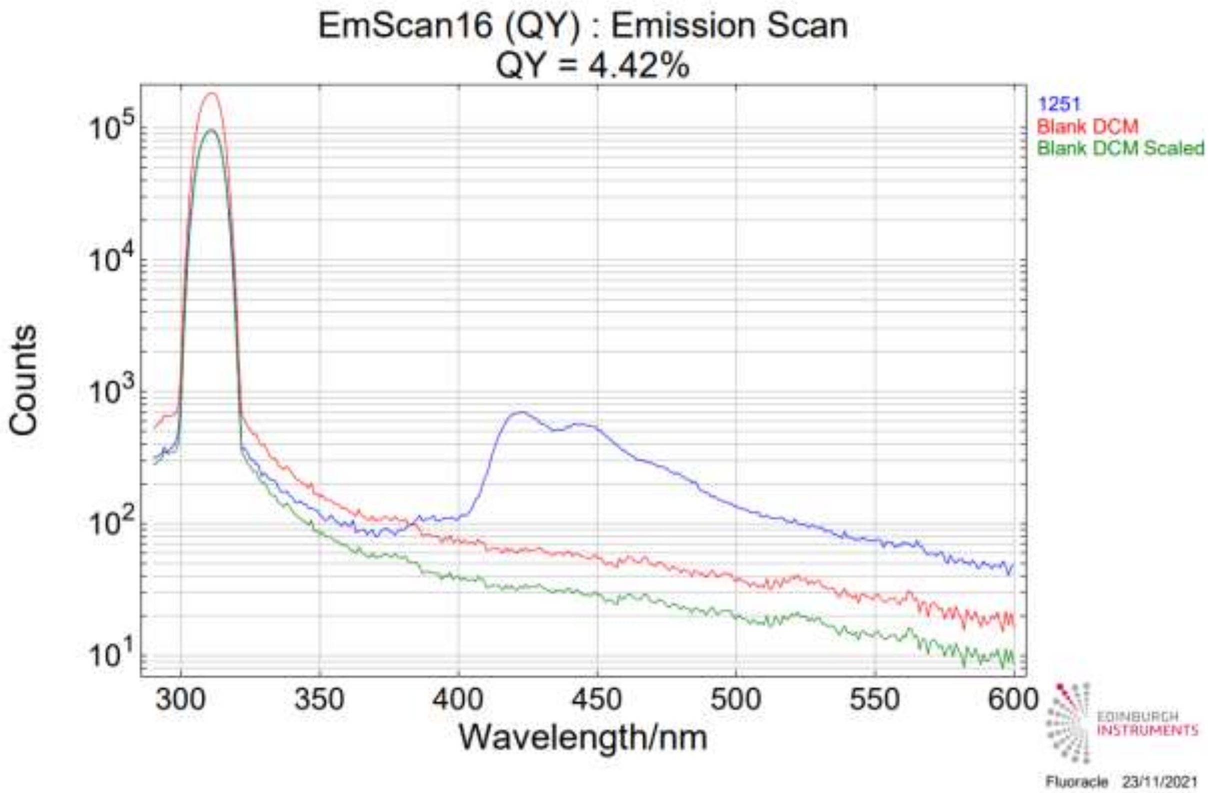

Quantum Yield Results
for 'EmScan16 (QY)'

Scatter Range: 300.00 to 321.00 nm
Emission Range: 396.00 to 559.00 nm

QY = 4.42%

Fig. S104. Quantum yield measurement in dichloromethane solution for **2j.** [c] = 1 x 10^{-5} M.

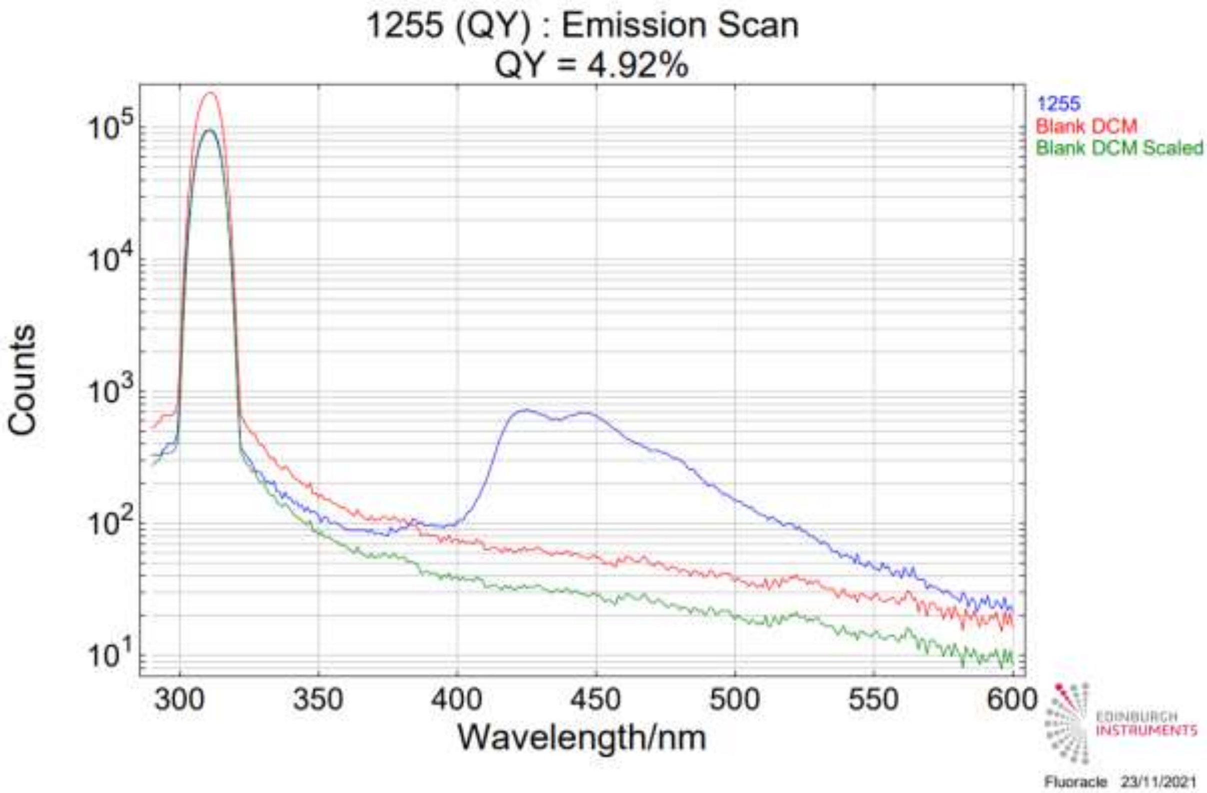

Quantum Yield Results
for '1255 (QY)'

Scatter Range: 298.00 to 321.00 nm
Emission Range: 394.00 to 564.00 nm

QY = 4.92%

Fig. S105. Quantum yield measurement in dichloromethane solution for **2k.** [c] = 1 x 10^{-5} M.

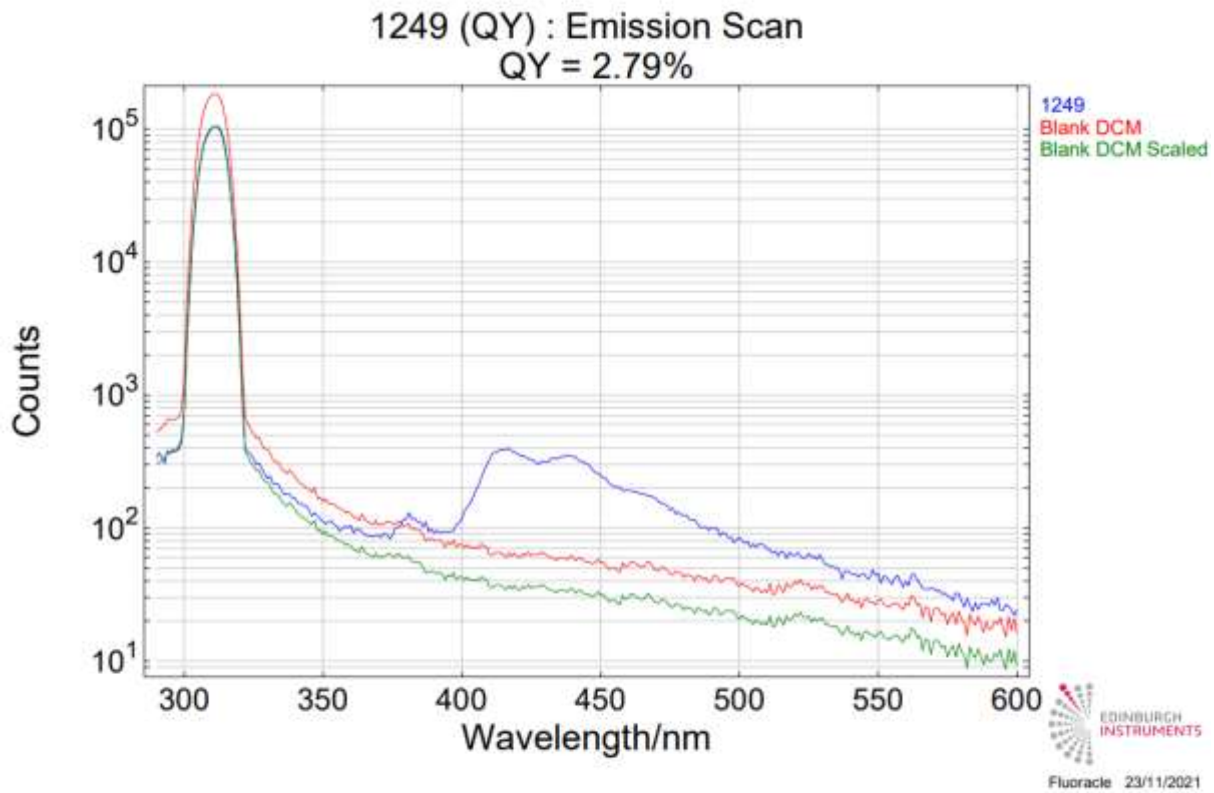

Quantum Yield Results
for '1249 (QY)'

Scatter Range: 299.00 to 321.00 nm
Emission Range: 377.00 to 542.00 nm

QY = 2.79%

Fig. S106. Quantum yield measurement in dichloromethane solution for **2l.** [c] = 1 x 10^{-5} M.

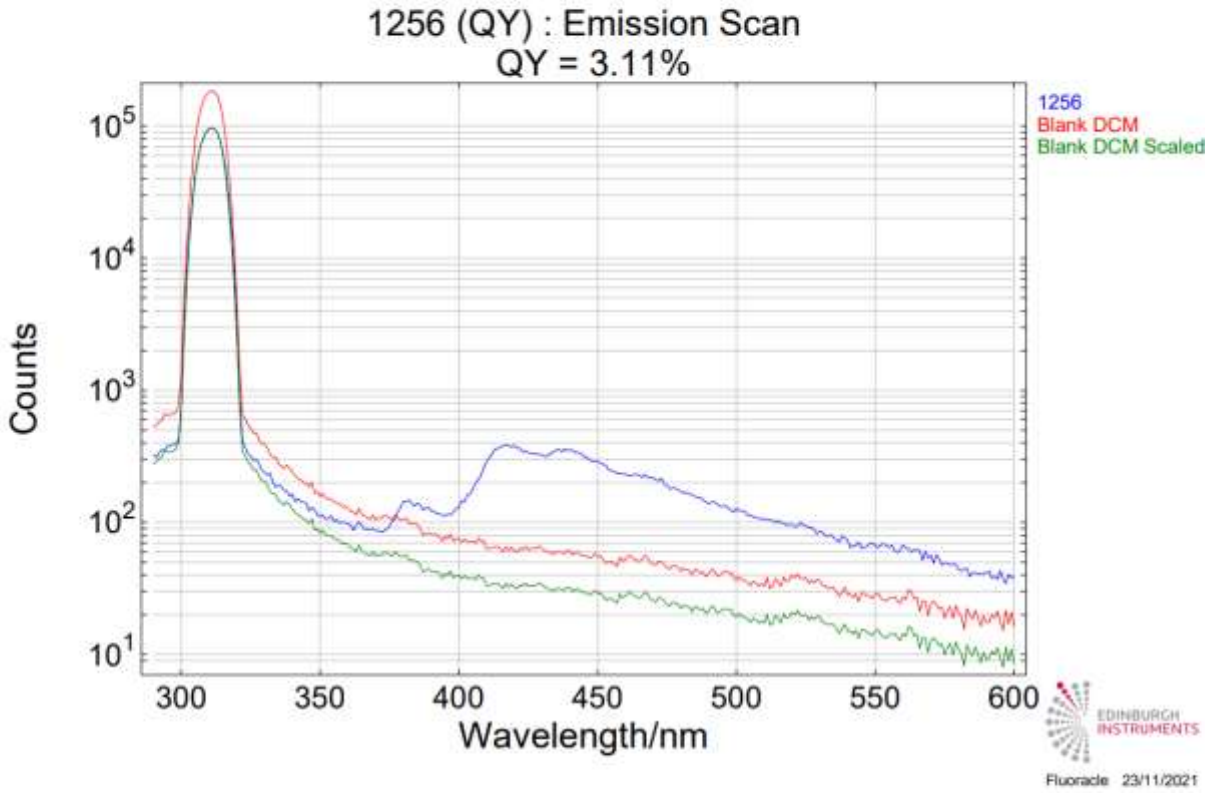

Quantum Yield Results
for '1256 (QY)'

Scatter Range: 300.00 to 322.00 nm
Emission Range: 368.00 to 532.00 nm

QY = 3.11%

Fig. S107. Quantum yield measurement in dichloromethane solution for **2m.** [c] = 1 x 10^{-5} M.

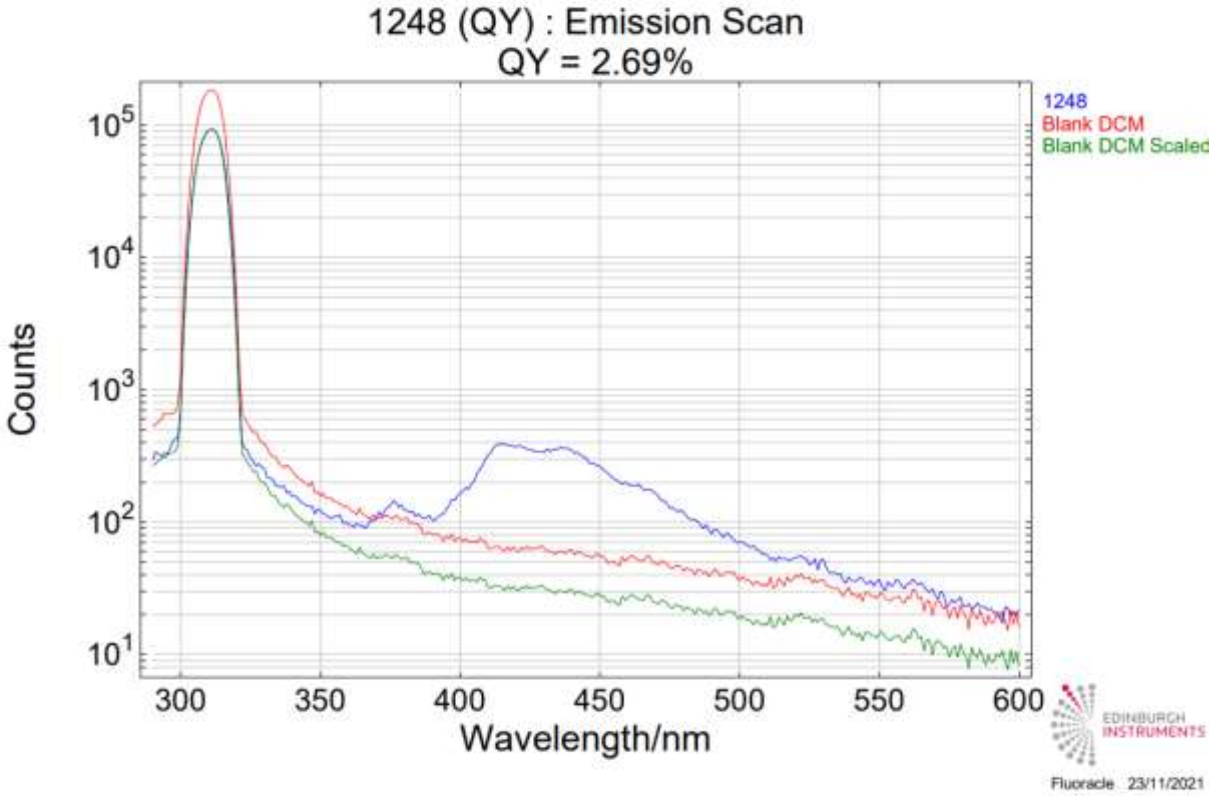

Quantum Yield Results
for '1248 (QY)'

Scatter Range: 300.00 to 321.00 nm
Emission Range: 359.00 to 537.00 nm

QY = 2.69%

Fig. S108. Quantum yield measurement in dichloromethane solution for **2n.** [c] = 1 x 10^{-5} M.

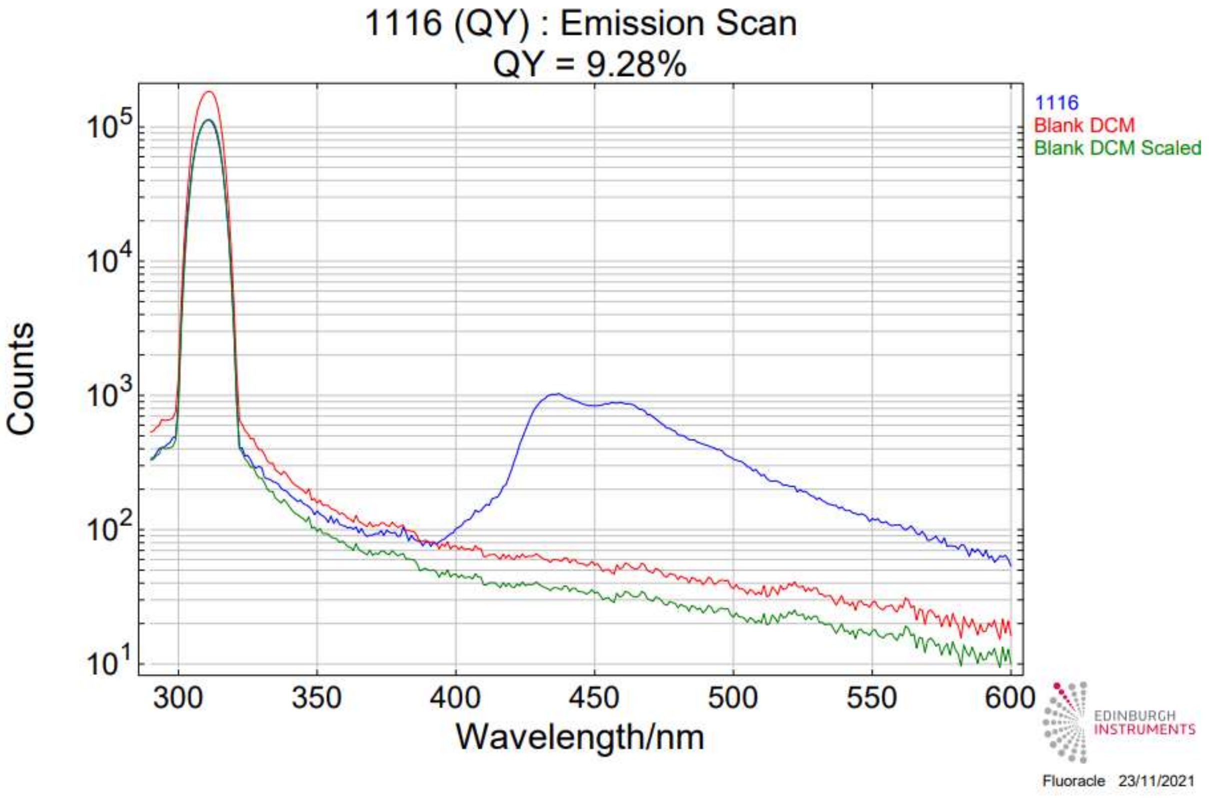

Quantum Yield Results
for '1116 (QY)'

Scatter Range: 300.00 to 321.00 nm
Emission Range: 392.00 to 586.00 nm

QY = 9.28%

Fig. S109. Quantum yield measurement in dichloromethane solution for **2o**. [c] = 1 x 10^{-5} M.

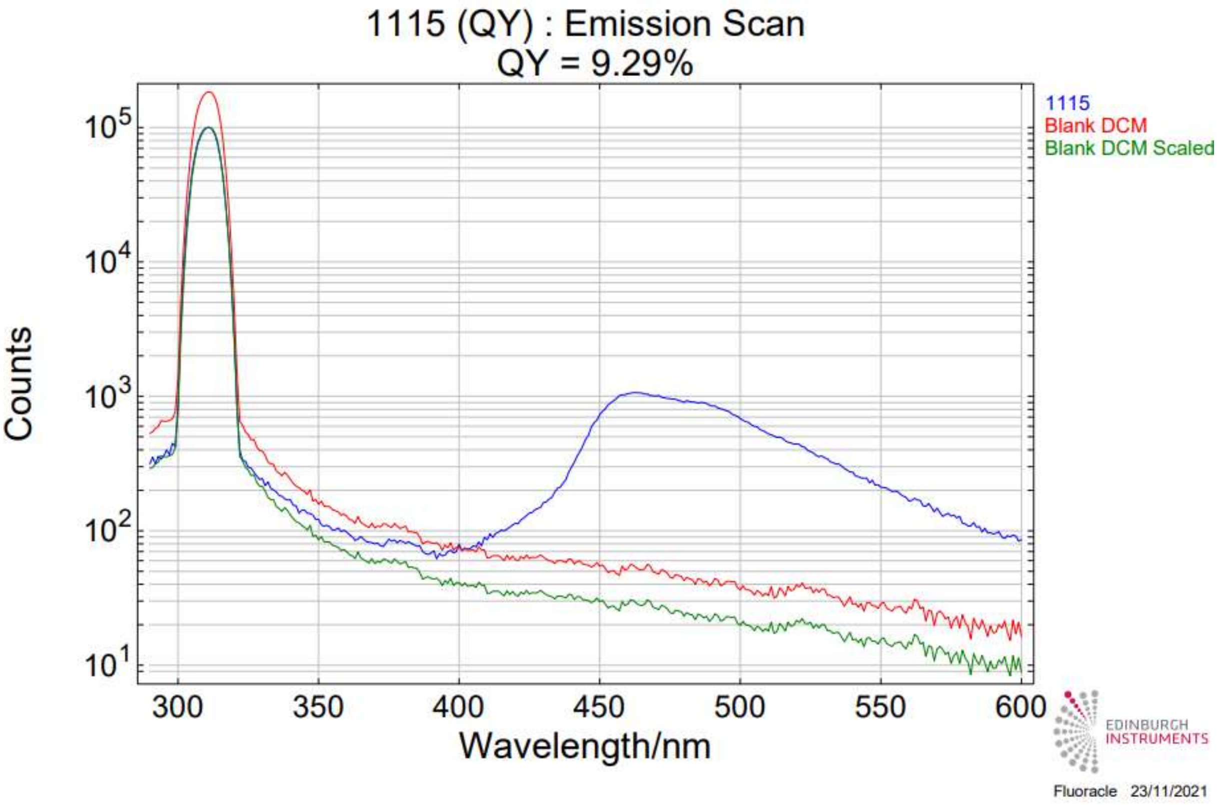

Quantum Yield Results
for '1115 (QY)'

Scatter Range: 300.00 to 322.00 nm
Emission Range: 394.00 to 600.00 nm

QY = 9.29%

Fig. S110. Quantum yield measurement in dichloromethane solution for **2p**. [c] = 1 x 10^{-5} M.

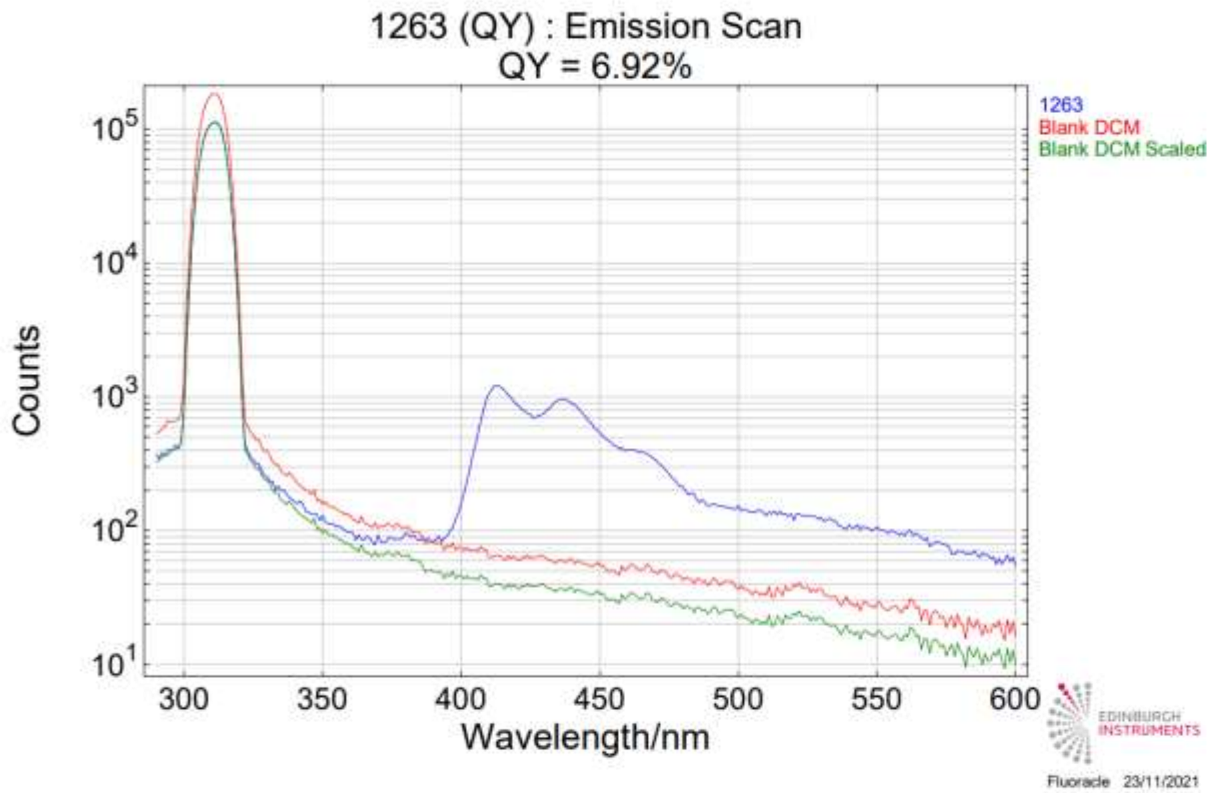

Quantum Yield Results
for '1263 (QY)'

Scatter Range: 300.00 to 322.00 nm
Emission Range: 388.00 to 501.00 nm

QY = 6.92%

Fig. S111. Quantum yield measurement in dichloromethane solution for **2q.** [c] = 1 x 10^{-5} M.

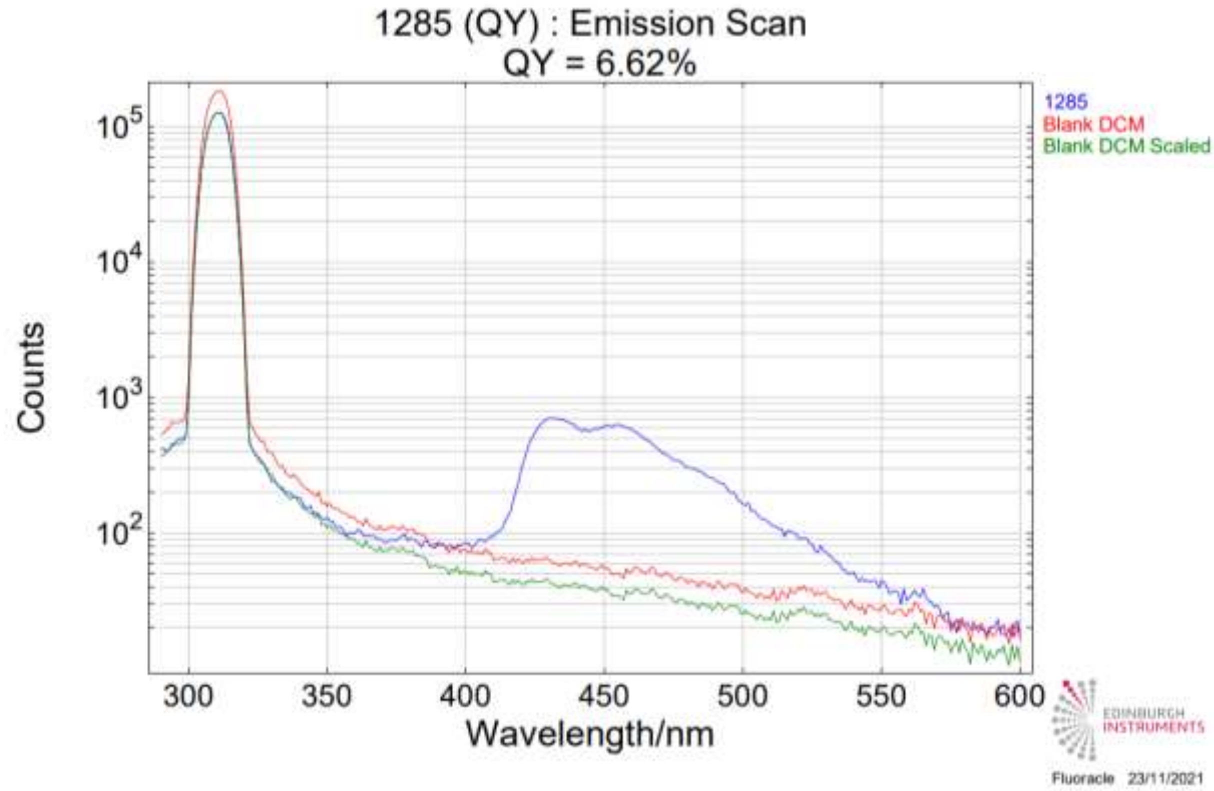

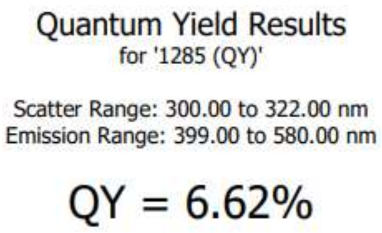

Fig. S112. Quantum yield measurement in dichloromethane solution for **2s.** [c] = 1 x 10^{-5} M.

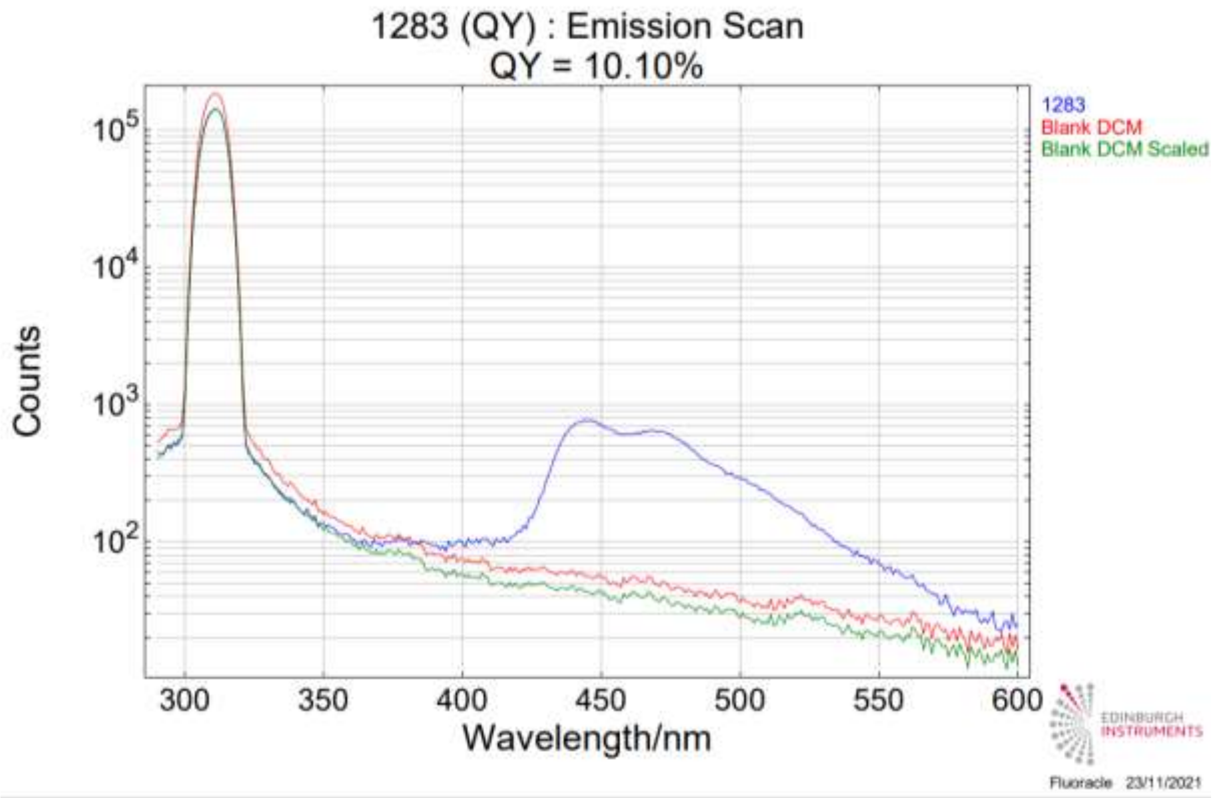

Quantum Yield Results
for '1283 (QY)'

Scatter Range: 300.00 to 322.00 nm
Emission Range: 379.00 to 597.00 nm

QY = 10.10%

Fig. S113. Quantum yield measurement in dichloromethane solution for **2t.** [c] = 1 x 10^{-5} M.

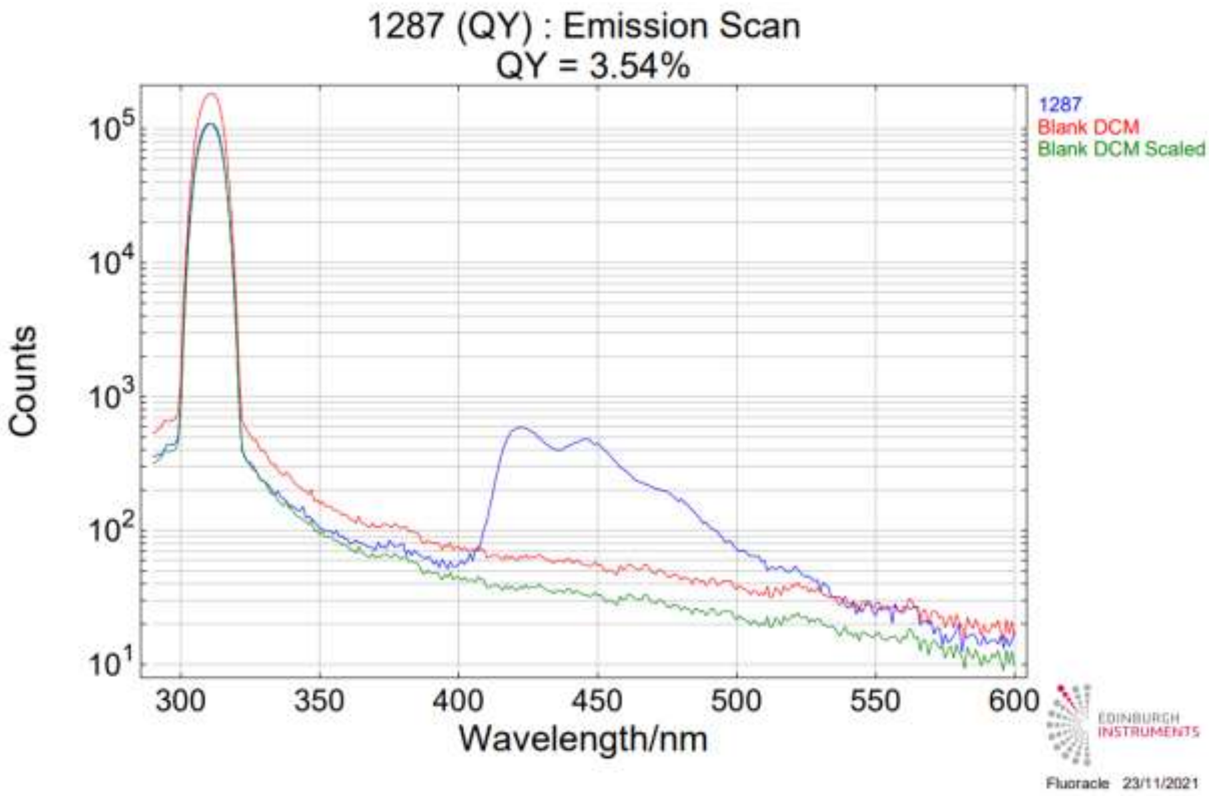

Quantum Yield Results
for '1287 (QY)'

Scatter Range: 298.00 to 322.00 nm
Emission Range: 401.00 to 565.00 nm

QY = 3.54%

Fig. S114. Quantum yield measurement in dichloromethane solution for **2u.** [c] = 1 x 10^{-5} M.

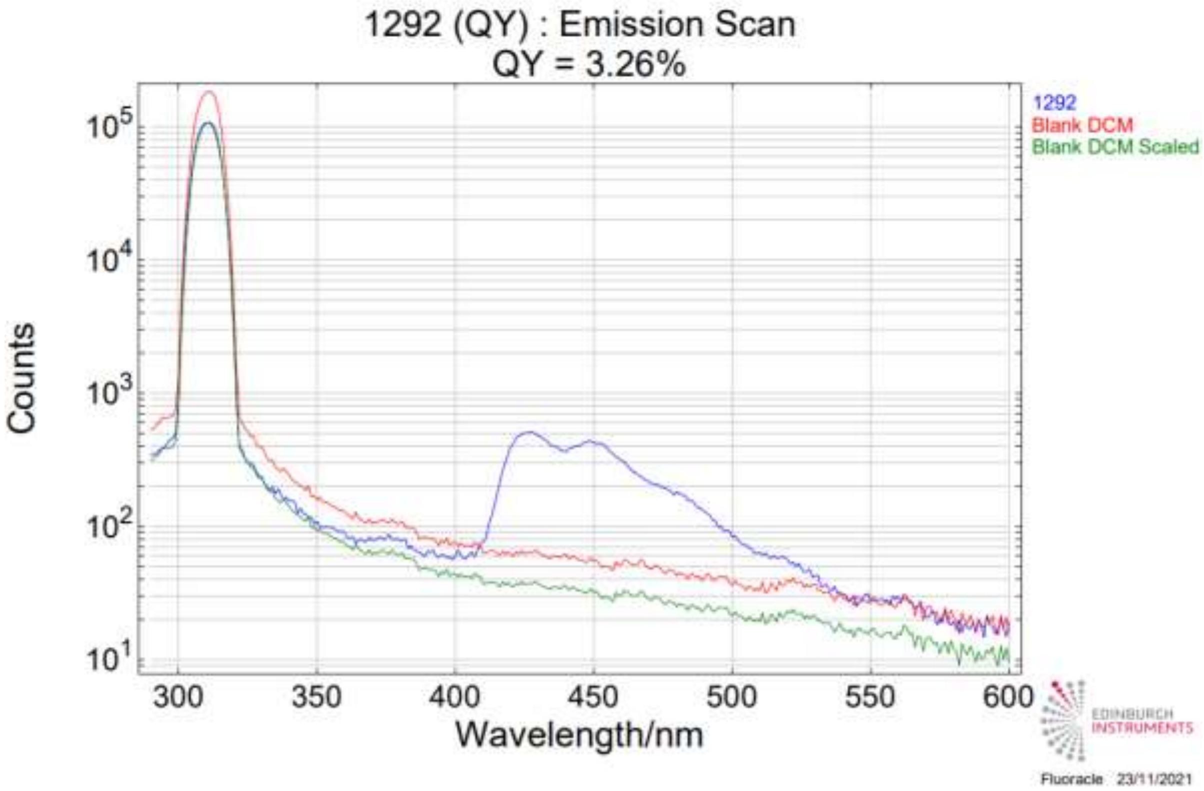

Quantum Yield Results
for '1292 (QY)'

Scatter Range: 300.00 to 321.00 nm
Emission Range: 397.00 to 571.00 nm

QY = 3.26%

Fig. S115. Quantum yield measurement in dichloromethane solution for **2v.** [c] = 1 x 10^{-5} M.

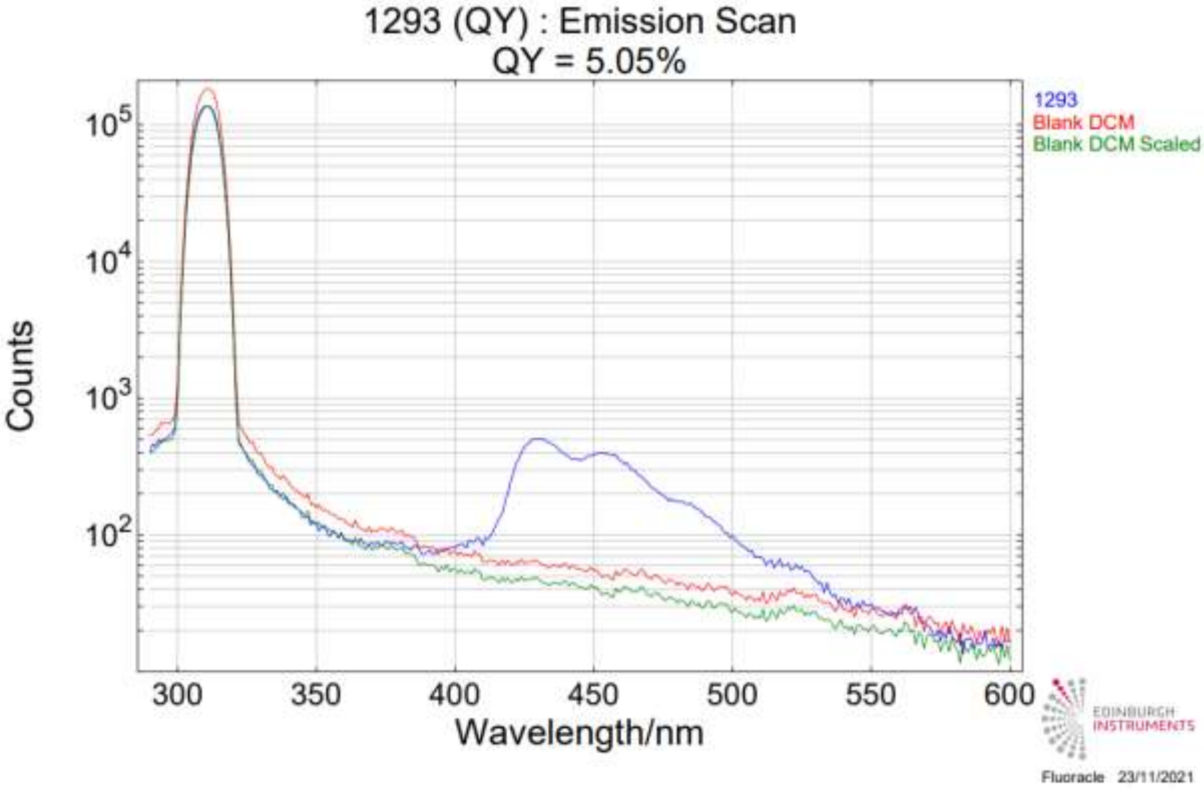

Quantum Yield Results

for '1293 (QY)'

Scatter Range: 300.00 to 322.00 nm
Emission Range: 395.00 to 570.00 nm

QY = 5.05%

Fig. S116. Quantum yield measurement in dichloromethane solution for **2w.** [c] = 1 x 10^{-5} M.

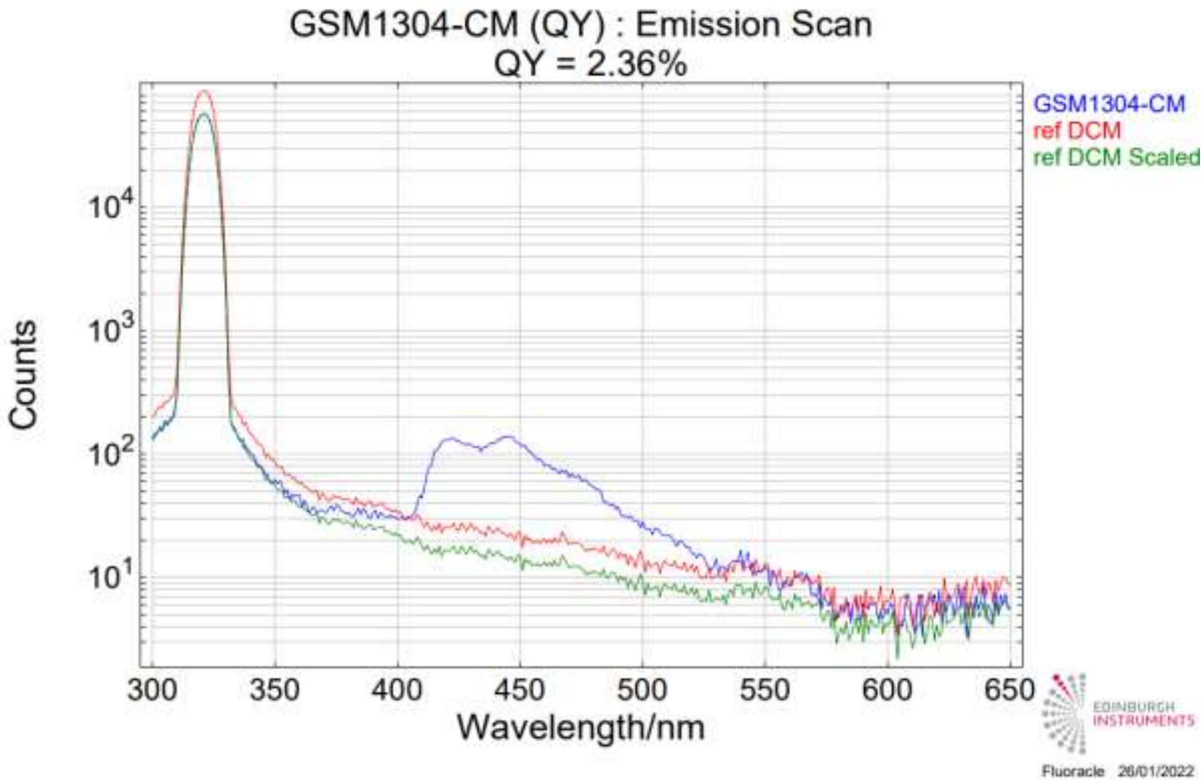

Quantum Yield Results
for 'GSM1304-CM (QY)'

Scatter Range: 310.00 to 332.00 nm
Emission Range: 381.00 to 600.00 nm

QY = 2.36%

Fig. S117. Quantum yield measurement in dichloromethane solution for **2x.** [c] = 1 x 10^{-5} M.

Photophysical properties of helicenes:

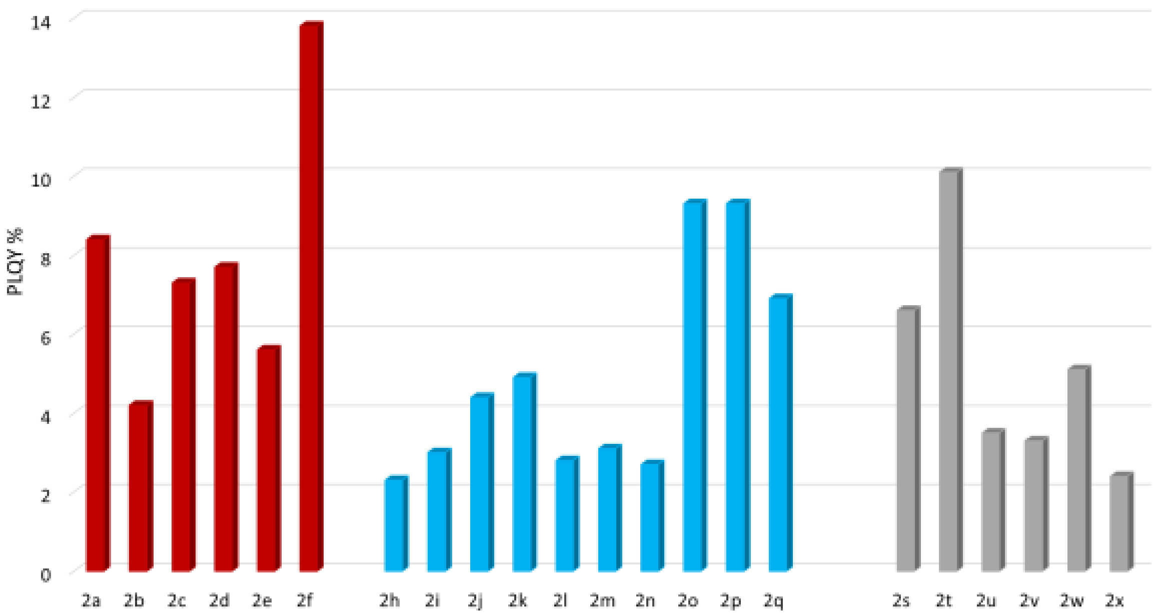

Fig. S118. Quantum yields in dichloromethane solution measured using an integration sphere [c] = 1 x 10^{-5} M.

Compound	$\lambda_{max,em\ 1}$ (nm)	$\lambda_{max,em\ 2}$ (nm)	PLQY (%)	g_{lum} x 10^3 at λ_{max}	τ (ns)
2a	404	424	8.4	1.2	8.0
2b	402	424	4.2	1.0	5.4
2c	406	428	7.3	3.1	5.3
2d	406	426	7.8	1.8	3.0
2e	410	432	5.6	3.6	3.9
2f	416	436	13.8	0.2	8.0
2h	418	440	2.3	0.3	5.6
2i	422	444	3.0	-1.8	5.4
2j	424	446	4.4	0.2	4.9
2k	426	448	4.9	0.3	10.1
2l	416	440	2.8	0.5	4.9
2m	418	442	3.1	N.D.	5.6
2n	418	438	2.7	+0.3	5.2
2o	438	460	9.3	2.7	7.8
2p	458	486	9.3	3.0	7.1
2q	412	438	6.9	2.2	6.3
2r	ND	ND	ND	ND	ND
2s	438	456	6.6	2.4	7.7
2t	444	470	10.1	3.3	7.1
2u	422	446	3.5	3.2	6.2
2v	426	450	3.3	2.7	6.3
2w	430	456	5.1	1.6	5.0
2x	423	447	2.4	-3.4	7.4

Table S1. Photophysical data of the helicenes at the excited state. [c] = 1 x 10^{-5} M.

DFT calculations:

Enantiomerisation barriers:

The geometries at the ground states ((*M*) enantiomer) and TS were carried out with Gaussian 16 program,[27] using the B3LYP functional, the 6-311+G(d,p) basis set, Grimme's D3 correction with Becke-Johnson (BJ) damping.[28]

Method: The enantiomerisation barriers were calculated by first approaching the geometry of the transition state by successive decrease of torsions like a–b–b'–a' (using modRedundant optimisation) followed by an optimization using the TS routine.

Compound 2a: Calculated $\Delta G^{\ddagger}_{en}$ = 35.3 kcalmol^{-1}

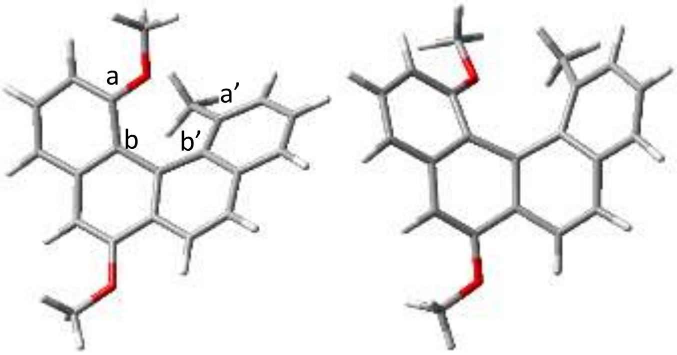

Fig. S119. Ground state (*M*-2a) and transition state of enantiomerisation

Compound 2h: Calculated $\Delta G^{\ddagger}_{en}$ = 40.1 kcalmol^{-1}

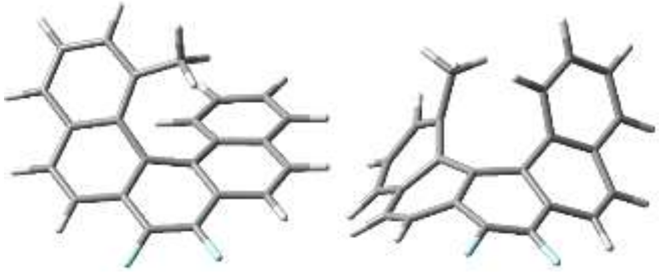

Fig. S120. Ground state (*M*-2h) and transition state of enantiomerisation

Compound 2r: Calculated $\Delta G^{\ddagger}_{en}$ = 38.0 kcalmol^{-1}

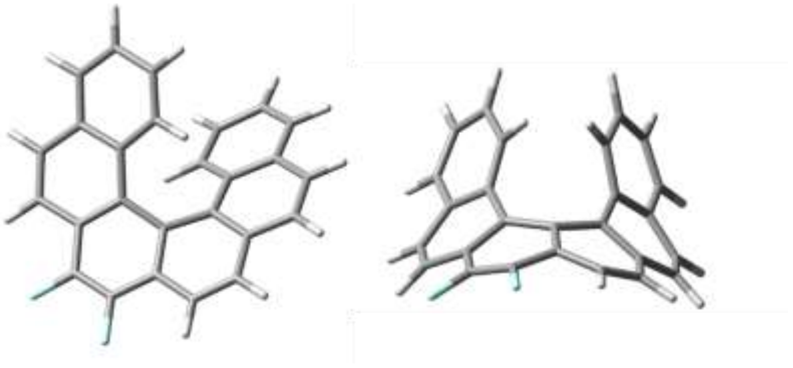

Fig. S121. Ground state (*M*-2h) and transition state of enantiomerisation

Optimization energies (with G_Grimme correction):

Compound	G (Hartrees)
2a (no imaginary frequency)	-961.556854
2a TS^{en} (one imaginary frequency: -74.02 cm^{-1})	-961.500537
2h (no imaginary frequency)	-1084.686633
2h TS^{en} (one imaginary frequency: -50.52 cm^{-1})	-1084.622776
2r (no imaginary frequency)	-1199.033769
2r TS^{en} (one imaginary frequency: -39.56 cm^{-1})	-1198.973258

Cartesian coordinates:

Cartesian Coordinates for **2a** (GS):

```
C 2.52776 -0.95820 0.03848
C 2.84779 0.33365 0.34365
C 1.85534 1.35443 0.26345
C 0.13075 -0.39638 0.00376
C 1.15843 -1.34967 -0.16405
C 0.84412 -2.68798 -0.53961
C -0.45069 -3.06191 -0.72063
C -1.51788 -2.19921 -0.33270
C -2.84844 -2.67248 -0.34186
C -3.85169 -1.93088 0.23293
C -2.24293 -0.22206 0.91156
C -3.52611 -0.74237 0.90556
C -0.36574 2.05361 -0.43428
C 2.22717 2.71498 0.37057
C 1.31485 3.70575 0.10191
C 0.02225 3.37961 -0.34275
C 0.50228 1.00324 -0.00768
C -1.22306 -0.89319 0.16779
C -1.96701 0.93827 1.84098
O -1.54853 1.66382 -0.97857
C -2.47293 2.64984 -1.39994
O 3.42616 -1.97598 -0.06102
C 4.79922 -1.67450 0.13212
H 3.86517 0.63005 0.55684
H 1.65842 -3.37554 -0.71838
H -0.69410 -4.05441 -1.08337
H -3.05176 -3.64749 -0.77070
H -4.87338 -2.29290 0.23697
H -4.29374 -0.23559 1.48129
H 3.25128 2.95947 0.62766
H 1.60037 4.74848 0.17862
H -0.64869 4.16985 -0.64694
H -0.93296 0.94599 2.18393
H -2.16125 1.90924 1.38186
H -2.61345 0.85558 2.71724
H -3.33556 2.10172 -1.77367
H -2.78293 3.29112 -0.56661
H -2.05804 3.27269 -2.19990
H 5.33431 -2.61079 -0.01472
H 5.14873 -0.93357 -0.59496
H 4.98566 -1.30375 1.14586
```

Cartesian Coordinates **2a** (TS^{en}):

```
C -2.50179 -0.84248 -0.31293
C -2.77356 0.27892 0.39931
C -1.73319 1.21659 0.65253
C -0.04465 -0.35710 -0.28532
C -1.12912 -1.25184 -0.48857
C -0.95551 -2.66353 -0.59953
C 0.18954 -3.21345 -0.14385
C 1.28431 -2.38901 0.23518
C 2.35669 -3.01278 0.90920
C 3.48970 -2.30715 1.21601
C 2.62325 -0.41656 -0.09341
C 3.62577 -1.04238 0.63758
C 0.29175 2.24626 -0.06811
C -2.05291 2.38223 1.38768
C -1.16204 3.42130 1.46775
C -0.00529 3.37764 0.68037
C -0.43336 1.02374 0.08396
C 1.29467 -0.98419 -0.09421
C 3.17027 0.63563 -1.03934
O 1.18902 2.28972 -1.07958
C 1.82374 3.51804 -1.38752
O -3.43560 -1.73438 -0.74227
C -4.79844 -1.45071 -0.46543
H -3.77117 0.52239 0.73587
H -1.80431 -3.26910 -0.87761
H 0.29279 -4.28738 -0.03724
H 2.25246 -4.05816 1.17654
H 4.30153 -2.75045 1.78004
H 4.59989 -0.56403 0.65911
H -3.02536 2.44069 1.86213
H -1.38361 4.30462 2.05480
H 0.61284 4.25910 0.59742
H 2.52321 0.78758 -1.89455
H 3.35394 1.59641 -0.55973
H 4.13194 0.26555 -1.40340
H 2.43916 3.32154 -2.26382
H 1.08560 4.29148 -1.62207
H 2.46418 3.86475 -0.56890
H -5.37031 -2.25327 -0.92728
H -4.99069 -1.43924 0.61294
H -5.09845 -0.49023 -0.89756
```

Cartesian Coordinates **2h** (GS):

C 0.00824 2.96697 -0.13739
C -1.27362 2.67418 0.21803
C -1.72501 1.33463 0.25991
C -0.80119 0.28935 0.00036
C 0.60323 0.60133 -0.02501
C 0.97789 1.94663 -0.27714
C 2.30471 2.26553 -0.69565
C 3.22862 1.27927 -0.82725
C 2.96446 -0.04180 -0.34917
C 4.00314 -0.99453 -0.30467
C 3.82197 -2.18764 0.35495
C 1.56827 -1.51475 1.01900
C 2.62871 -2.40748 1.05696
C -1.35553 -1.00891 -0.33721
C -3.09859 1.04746 0.52256
C -3.56445 -0.22550 0.43170
C -2.72755 -1.27433 -0.05674
C -0.62329 -1.99957 -1.03202
C -3.26357 -2.55090 -0.34066
C -2.50174 -3.51944 -0.95255
C -1.17916 -3.22377 -1.33024
C 1.67845 -0.35484 0.19231
C 0.41246 -1.77079 1.95882
H 2.53681 3.29224 -0.94384
H 4.21689 1.49836 -1.21521
H 4.95601 -0.74953 -0.76016
H 4.61833 -2.92131 0.39726
H 2.54134 -3.28618 1.68693
H -3.75269 1.86518 0.79349
H -4.60117 -0.44925 0.65631
H 0.38978 -1.79154 -1.34098
H -4.30211 -2.74246 -0.09387
H -2.92656 -4.49116 -1.17492
H -0.59353 -3.95977 -1.86835
H 0.79557 -2.22448 2.87523
H -0.10533 -0.85043 2.22681
H -0.32910 -2.45139 1.53669
F 0.37812 4.24946 -0.31934
F -2.15178 3.66999 0.44502

Cartesian Coordinates **2h** (TS^{en}):

C 1.38994 2.53756 0.58591
C 0.21066 2.91624 0.03262
C -0.75921 1.94834 -0.28998
C -0.57924 0.60276 0.13744
C 0.77845 0.14120 0.42536
C 1.75269 1.16998 0.61476
C 3.16182 0.93109 0.53560
C 3.57908 -0.18607 -0.09492
C 2.65958 -1.23071 -0.41661
C 3.12708 -2.30477 -1.19957
C 2.34308 -3.41570 -1.39430
C 0.73314 -2.51812 0.22448
C 1.19606 -3.53346 -0.60737
C -1.83022 -0.14745 0.16814
C -1.87315 2.33210 -1.10564
C -2.83507 1.43475 -1.43169
C -2.89472 0.19733 -0.72067
C -2.14714 -1.02788 1.21191
C -4.07999 -0.56589 -0.71715
C -4.29954 -1.52984 0.24507
C -3.34926 -1.70848 1.25952
C 1.32715 -1.21465 0.12627
C -0.12034 -3.02756 1.37064
H 3.84455 1.72585 0.79823
H 4.62115 -0.32184 -0.36066
H 4.11972 -2.23373 -1.62923
H 2.66328 -4.22988 -2.03291
H 0.69752 -4.49577 -0.54993
H -1.88815 3.34061 -1.49591
H -3.63400 1.69283 -2.11693
H -1.45485 -1.10946 2.03075
H -4.85367 -0.32290 -1.43713
H -5.22880 -2.08686 0.26079
H -3.56224 -2.36495 2.09528
H 0.31611 -3.97520 1.69560
H -0.08248 -2.36111 2.22825
H -1.15820 -3.21673 1.10768
F 2.31645 3.47226 0.87711
F -0.02144 4.21157 -0.24963

Cartesian Coordinates **2r** (GS):

```
C 3.08909 -1.33358 0.13121
C 3.32985 -0.03936 0.46924
C 2.32226 0.94749 0.33013
C 1.02848 0.55953 -0.09697
C 0.70085 -0.84990 -0.11232
C 1.77914 -1.77753 -0.17570
C 1.55488 -3.13023 -0.54840
C 0.29196 -3.55232 -0.83475
C -0.82833 -2.71717 -0.57069
C -2.15204 -3.22022 -0.76456
C -3.23992 -2.49587 -0.39592
C -1.77405 -0.74770 0.54218
C -3.08162 -1.26805 0.31569
C -4.05076 0.50584 1.65993
C -2.75711 0.96141 1.97080
C -1.65135 0.34929 1.42584
C 0.15458 1.61168 -0.58393
C 2.63335 2.31816 0.58167
C 1.71489 3.28746 0.33340
C 0.47697 2.97137 -0.30294
C -0.97384 1.36252 -1.39853
C -0.39872 3.99922 -0.72098
C -1.52657 3.71424 -1.45454
C -1.79361 2.38350 -1.82268
F 4.09686 -2.22721 0.12858
F 4.56460 0.33694 0.85394
C -0.63416 -1.40630 -0.07039
H 2.40433 -3.79377 -0.63512
H 0.11560 -4.55898 -1.19607
H -2.26528 -4.19834 -1.21852
H -4.24225 -2.87012 -0.57174
H -4.91783 1.00476 2.07646
H -2.62617 1.79740 2.64749
H -0.66885 0.70854 1.69141
H 3.61142 2.56001 0.97476
H 1.93775 4.32582 0.55145
H -1.19179 0.35180 -1.70743
H -0.14687 5.02481 -0.47419
H -2.18745 4.51064 -1.77563
H -2.64810 2.15688 -2.44910
C -4.20362 -0.60140 0.85841
H -5.19119 -0.99957 0.65232
```

Cartesian Coordinates **2r** (TS^{en}):

```
C 3.19909 -0.94796 -0.58553
C 3.37195 0.26272 -0.00358
C 2.25515 1.06810 0.31193
C 0.96473 0.69679 -0.15255
C 0.73112 -0.72417 -0.44825
C 1.90543 -1.52509 -0.64542
C 1.87634 -2.94759 -0.61977
C 0.80831 -3.56190 -0.05119
C -0.34575 -2.81385 0.29446
C -1.32878 -3.40895 1.14939
C -2.45562 -2.74027 1.49965
C -1.88662 -1.02608 -0.17732
C -2.81243 -1.56712 0.76794
C -4.60076 -0.21429 -0.16023
C -3.76169 0.12223 -1.23233
C -2.44147 -0.27862 -1.23143
C 0.02743 1.81487 -0.18141
C 2.44602 2.21117 1.15220
C 1.39963 3.00958 1.47962
C 0.18022 2.88986 0.74782
C -0.87513 2.03032 -1.23766
C -0.77202 3.93140 0.77352
C -1.74654 4.02328 -0.19647
C -1.74404 3.10189 -1.25366
F 4.27220 -1.70817 -0.87949
F 4.60748 0.69651 0.30825
C -0.49829 -1.47700 -0.15962
H 2.77314 -3.49400 -0.87380
H 0.82911 -4.62247 0.17189
H -1.11002 -4.38827 1.55983
H -3.15547 -3.14929 2.21910
H -5.62656 0.13403 -0.13753
H -4.15167 0.68397 -2.07265
H -1.83394 -0.09701 -2.10032
H 3.43239 2.37799 1.56288
H 1.51485 3.82130 2.18839
H -0.81466 1.38503 -2.09587
H -0.67134 4.70458 1.52716
H -2.45519 4.84286 -0.18620
H -2.41295 3.23779 -2.09499
C -4.13493 -1.07552 0.80956
H -4.80286 -1.45448 1.57526
```

TD-DFT, calculated UV/ECD spectra:

The geometries at the ground states ((*M*) enantiomers) were carried out with Gaussian 16 program,[27] using the B3LYP functional, the 6-311+G(2d,2p) basis set, Grimme's D3 correction with Becke-Johnson (BJ) damping,[28] and the IEF-PCM solvation model for dichloromethane.[29] The UV-vis absorption and CD spectra were simulated by time-dependent DFT (TD-DFT) calculations at the same level of theory and solvent model (the oscillator and rotatory strengths of their first 60 vertical transitions were calculated). Oscillator and rotatory strengths were fitted to Gaussian functions with a half-width at half-height of 0.2 eV to obtain the predicted spectra (carried out using GaussSum software[30] to generate the data).

Compound 2a:

Cartesian Coordinates for **2a** (ground state):

```
C 2.52376 -0.96247 0.03454
C 2.84205 0.32676 0.34377
C 1.85067 1.34633 0.26620
C 0.12881 -0.39842 -0.00236
C 1.15496 -1.35162 -0.16993
C 0.83655 -2.68729 -0.54785
C -0.45749 -3.06082 -0.72654
C -1.52128 -2.19858 -0.33378
C -2.85006 -2.67287 -0.33676
C -3.84888 -1.93348 0.24591
C -2.23898 -0.22449 0.91502
C -3.51998 -0.74676 0.91734
C -0.36370 2.05225 -0.43933
C 2.22445 2.70390 0.38409
C 1.31409 3.69630 0.11952
C 0.02556 3.37543 -0.33592
C 0.49953 0.99957 -0.01286
C -1.22399 -0.89435 0.16614
C -1.96254 0.93901 1.83692
O -1.53863 1.67295 -1.00708
C -2.46465 2.67771 -1.40202
O 3.42438 -1.97599 -0.06723
C 4.80072 -1.66625 0.13766
H 3.85662 0.62070 0.55912
H 1.64327 -3.37864 -0.73180
H -0.70070 -4.04998 -1.09007
H -3.05524 -3.64523 -0.76368
H -4.86771 -2.29495 0.25601
H -4.28329 -0.24216 1.49513
H 3.24506 2.94490 0.64668
H 1.59916 4.73562 0.20462
H -0.63969 4.16700 -0.63860
H -0.93745 0.93209 2.19878
H -2.13215 1.90528 1.36589
H -2.62629 0.87652 2.69787
H -3.32700 2.14526 -1.78981
H -2.76704 3.29133 -0.55148
H -2.04701 3.31697 -2.18121
H 5.33945 -2.59646 -0.00960
H 5.14443 -0.92283 -0.58279
H 4.97202 -1.30016 1.15084
```

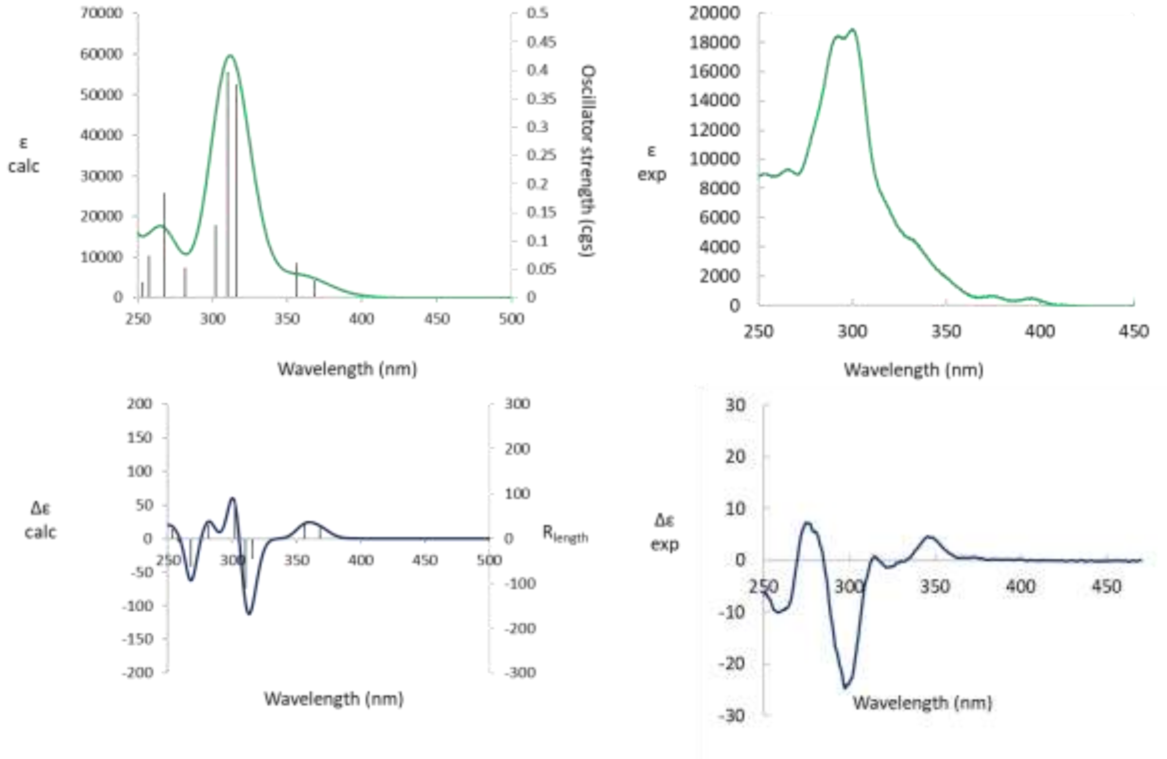

Fig. S122. ε = f(λ) (green) and Δε = f(λ) (blue) for **2a** (left: calculated; right experimental). Excitations/oscillator strengths and rotatory strengths (R (length)) are depicted in black in the calculated UV and ECD spectrum respectively. Units: ε and Δε ($M^{-1}.cm^{-1}$ or 10^{-40} esu^2 cm^2);

Excitation energies and oscillator strengths (from S_0 state):

Excited State 1: Singlet-A 3.3665 eV 368.29 nm f=0.0228 <S**2>=0.000

79 -> 81 0.38114

79 -> 82 -0.16510

80 -> 81 0.49987

80 -> 82 0.26811

This state for optimization and/or second-order correction.

Total Energy, E(TD-HF/TD-DFT) = -961.776110983

Copying the excited state density for this state as the 1-particle RhoCI density.

Excited State 2: Singlet-A 3.4787 eV 356.41 nm f=0.0546 <S**2>=0.000

78 -> 81 -0.10102

79 -> 81 -0.43758

80 -> 81 0.45798

80 -> 82 -0.27117

Excited State 3: Singlet-A 3.9236 eV 315.99 nm f=0.3674 <S**2>=0.000

77 -> 81 -0.12759

78 -> 81 0.32678

79 -> 81 -0.33198

79 -> 82 -0.31455

80 -> 82 0.38602

Excited State 4: Singlet-A 3.9940 eV 310.43 nm f=0.3902 <S**2>=0.000

78 -> 81 -0.36828

79 -> 81 -0.19754

79 -> 82 0.32486

80 -> 82 0.44246

Excited State 5: Singlet-A 4.1045 eV 302.07 nm f=0.1209 <S**2>=0.000

77 -> 81 0.18050

78 -> 81 0.48604

79 -> 82 0.43937

80 -> 81 0.14276

Excited State 6: Singlet-A 4.4029 eV 281.59 nm f=0.0464 <S**2>=0.000

77 -> 81 -0.30195

78 -> 82 0.53966

79 -> 82 0.16661

79 -> 83 -0.11430

80 -> 83 -0.17347

80 -> 84 0.12635

Excited State 7: Singlet-A 4.6323 eV 267.65 nm f=0.1779 <S**2>=0.000

77 -> 81 0.41767

78 -> 82 0.38829

79 -> 82 -0.10790

79 -> 83 -0.12287

80 -> 83 0.22286

80 -> 84 -0.28289

Excited State 8: Singlet-A 4.8107 eV 257.73 nm f=0.0671 <S**2>=0.000

77 -> 81 -0.22170

79 -> 84 0.10447

80 -> 83 0.61045

80 -> 84 0.15783

Excited State 9: Singlet-A 4.8908 eV 253.50 nm f=0.0208 <S**2>=0.000

77 -> 81 -0.16472

79 -> 83 0.53804

80 -> 84 -0.37843

Excited State 10: Singlet-A 5.0190 eV 247.03 nm f=0.0331 <S**2>=0.000

77 -> 82 0.44986

79 -> 84 0.26105

80 -> 85 0.43490

Excited State 11: Singlet-A 5.0260 eV 246.69 nm f=0.0315 <S**2>=0.000

77 -> 82 -0.32946

79 -> 84 -0.31138

80 -> 85 0.50591

Excited State 12: Singlet-A 5.1295 eV 241.71 nm f=0.0890 <S**2>=0.000

76 -> 81 0.48859

77 -> 81 -0.10189

78 -> 82 -0.10199

79 -> 83 -0.22196

79 -> 84 -0.10057

79 -> 88 -0.11506

80 -> 84 -0.28555

80 -> 88 0.17175

Excited State 13: Singlet-A 5.1822 eV 239.25 nm f=0.0010 <S**2>=0.000

76 -> 81 0.14701

79 -> 85 0.61220

80 -> 86 -0.17936

80 -> 87 -0.15833

Excited State 14: Singlet-A 5.2730 eV 235.13 nm f=0.0350 <S**2>=0.000

76 -> 82 0.14686

77 -> 82 -0.27829

78 -> 83 0.10941

79 -> 83 -0.13007

79 -> 84 0.40325

80 -> 83 -0.12935

80 -> 86 -0.19459

80 -> 87 0.30234

Excited State 15: Singlet-A 5.3141 eV 233.31 nm f=0.1226 <S**2>=0.000

76 -> 81 0.27551

77 -> 81 0.14610

77 -> 82 0.13317

79 -> 83 0.21373

79 -> 84 -0.17331

80 -> 84 0.23479

80 -> 87 0.43679

Excited State 16: Singlet-A 5.3516 eV 231.68 nm f=0.0260 <S**2>=0.000

79 -> 84 0.13772

79 -> 85 0.18393

80 -> 86 0.61892

80 -> 87 0.11498

Excited State 17: Singlet-A 5.3742 eV 230.70 nm f=0.2922 <S**2>=0.000

75 -> 81 0.13061

76 -> 81 -0.31388

77 -> 81 -0.12352

78 -> 83 -0.11541

78 -> 84 0.15009

79 -> 83 -0.12487

79 -> 84 -0.20827

79 -> 85 0.14836

79 -> 86 -0.14195

79 -> 87 0.12410

79 -> 88 -0.10899

80 -> 84 -0.20524

80 -> 87 0.32274

Excited State 18: Singlet-A 5.4135 eV 229.03 nm f=0.0431 <S**2>=0.000

75 -> 82 0.10974

76 -> 82 -0.26724

77 -> 83 -0.13452

78 -> 83 0.16075

78 -> 84 -0.23791

79 -> 88 0.27173

80 -> 84 -0.11685

80 -> 88 0.36004

Excited State 19: Singlet-A 5.4987 eV 225.48 nm f=0.0078 <S**2>=0.000

79 -> 86 0.47764

79 -> 87 0.40665

80 -> 89 -0.20311

80 -> 90 0.11838

Excited State 20: Singlet-A 5.5164 eV 224.76 nm f=0.0624 <S**2>=0.000

75 -> 81 -0.12508

78 -> 83 -0.17502

79 -> 85 -0.17268

79 -> 86 -0.32873

79 -> 87 0.43198

80 -> 84 0.10539

80 -> 88 0.21658

Excited State 21: Singlet-A 5.5200 eV 224.61 nm f=0.0385 <S**2>=0.000

76 -> 82 0.16763

77 -> 82 -0.10441

78 -> 83 -0.34545

79 -> 86 0.25403

79 -> 87 -0.21118

79 -> 88 -0.11470

80 -> 88 0.39124

Excited State 22: Singlet-A 5.6435 eV 219.69 nm f=0.0361 <S**2>=0.000

75 -> 81 -0.11658

76 -> 82 0.34628

78 -> 83 0.37725

78 -> 84 -0.14065

79 -> 88 -0.14856

80 -> 88 0.10110

80 -> 90 0.30705

Excited State 23: Singlet-A 5.6558 eV 219.22 nm f=0.0345 <S**2>=0.000

78 -> 83 0.11130

78 -> 85 0.10926

79 -> 86 0.16736

79 -> 87 0.16599

80 -> 88 0.14413

80 -> 89 0.57550

80 -> 92 -0.10561

Excited State 24: Singlet-A 5.6871 eV 218.01 nm f=0.0434 <S**2>=0.000

75 -> 81 0.10523

76 -> 82 -0.20427

78 -> 83 -0.12168

78 -> 84 0.19203

79 -> 87 -0.10241

80 -> 90 0.56317

80 -> 91 0.11589

Excited State 25: Singlet-A 5.6974 eV 217.62 nm f=0.0367 <S**2>=0.000

75 -> 81 0.30620

76 -> 82 0.32532

78 -> 83 -0.10571

78 -> 84 -0.16219

79 -> 88 0.44331

Excited State 26: Singlet-A 5.7561 eV 215.40 nm f=0.1016 <S**2>=0.000

78 -> 84 -0.31106

79 -> 88 -0.13599

79 -> 89 -0.14542

79 -> 90 0.10727

80 -> 88 -0.12898

80 -> 91 0.50840

Excited State 27: Singlet-A 5.7722 eV 214.80 nm f=0.0712 <S**2>=0.000

77 -> 83 -0.12111

78 -> 83 -0.11370

78 -> 84 -0.25616

78 -> 85 -0.30485

79 -> 88 -0.12861

79 -> 89 0.30523

79 -> 90 -0.13229

80 -> 89 0.18765

80 -> 91 -0.18640

80 -> 92 0.22845

Excited State 28: Singlet-A 5.8066 eV 213.52 nm f=0.0520 <S**2>=0.000

75 -> 81 -0.19282

77 -> 83 0.13035

78 -> 84 0.21814

78 -> 85 -0.15198

79 -> 88 0.26601

79 -> 89 0.33323

80 -> 91   0.31782

80 -> 92   0.14152

Excited State 29:   Singlet-A   5.8191 eV 213.06 nm f=0.0042 <S**2>=0.000

79 -> 89   -0.22314

79 -> 90   0.15569

79 -> 91   0.11294

80 -> 92   0.59268

Excited State 30:   Singlet-A   5.8439 eV 212.16 nm f=0.0138 <S**2>=0.000

75 -> 81   0.10550

78 -> 85   0.51208

79 -> 89   0.28373

79 -> 90   -0.21621

80 -> 91   0.11390

80 -> 92   0.11786

Excited State 31:   Singlet-A   5.8660 eV 211.36 nm f=0.0169 <S**2>=0.000

78 -> 84   -0.10252

78 -> 85   0.13993

79 -> 89   0.20812

79 -> 90   0.58582

80 -> 91   -0.13780

Excited State 32:   Singlet-A   5.9416 eV 208.67 nm f=0.2833 <S**2>=0.000

75 -> 81   0.43843

78 -> 83   0.18446

78 -> 85   -0.11954

79 -> 88 -0.14251

79 -> 89 0.13512

79 -> 91 0.14688

79 -> 92 -0.13952

80 -> 88 0.18490

80 -> 89 -0.11884

80 -> 90 -0.10518

80 -> 93 0.16354

80 -> 94 -0.10983

Excited State 33: Singlet-A 5.9505 eV 208.36 nm f=0.0256 <S**2>=0.000

75 -> 81 -0.14231

77 -> 83 0.11519

79 -> 89 0.11242

79 -> 91 0.63091

Excited State 34: Singlet-A 5.9895 eV 207.00 nm f=0.0138 <S**2>=0.000

75 -> 81 0.11810

79 -> 92 0.62291

80 -> 93 -0.15203

80 -> 94 0.10134

Excited State 35: Singlet-A 6.0300 eV 205.61 nm f=0.0777 <S**2>=0.000

75 -> 82 -0.16840

76 -> 82 -0.11547

77 -> 83 0.31040

78 -> 84 -0.16280

78 -> 87 -0.10724

79 -> 92 0.19850

80 -> 93 0.41624

80 -> 94 -0.21297

Excited State 36: Singlet-A 6.0862 eV 203.71 nm f=0.1917 <S**2>=0.000

74 -> 81 0.14688

75 -> 82 -0.13459

77 -> 83 0.42631

78 -> 84 -0.10399

78 -> 86 0.21872

78 -> 88 0.22317

80 -> 93 -0.28460

Excited State 37: Singlet-A 6.1139 eV 202.79 nm f=0.0044 <S**2>=0.000

78 -> 86 0.22307

78 -> 87 0.47773

80 -> 94 -0.36625

Excited State 38: Singlet-A 6.1372 eV 202.02 nm f=0.0009 <S**2>=0.000

74 -> 81 0.34294

75 -> 82 -0.11325

78 -> 86 -0.31330

78 -> 87 0.33561

79 -> 93 0.12972

79 -> 94 -0.10048

80 -> 94 0.23423

Excited State 39: Singlet-A 6.1447 eV 201.77 nm f=0.0051 <S**2>=0.000

74 -> 81 -0.16952

75 -> 82 -0.11551

78 -> 86 0.35001

78 -> 87 0.12432

80 -> 93 0.27657

80 -> 94 0.42082

Excited State 40: Singlet-A 6.1587 eV 201.32 nm f=0.0004 <S**2>=0.000

74 -> 81 0.43920

75 -> 82 0.20734

78 -> 86 0.31371

78 -> 87 -0.11600

78 -> 88 -0.18787

80 -> 93 0.15956

Excited State 41: Singlet-A 6.1897 eV 200.31 nm f=0.0483 <S**2>=0.000

75 -> 82 0.44947

77 -> 83 0.19842

78 -> 84 -0.10683

78 -> 87 0.19672

79 -> 93 -0.31638

79 -> 94 0.20472

Excited State 42: Singlet-A 6.2283 eV 199.06 nm f=0.0900 <S**2>=0.000

73 -> 81 0.11981

75 -> 82 0.10215

78 -> 88 0.39898

79 -> 93 0.22286

80 -> 93 0.18128

80 -> 95 -0.17134

80 -> 96 -0.34342

Excited State 43: Singlet-A 6.2378 eV 198.76 nm f=0.0023 <S**2>=0.000

77 -> 85 -0.11372

78 -> 89 0.16791

79 -> 93 0.32691

79 -> 94 0.37289

79 -> 97 0.10032

80 -> 95 -0.27020

80 -> 96 0.20016

80 -> 98 0.10982

Excited State 44: Singlet-A 6.2761 eV 197.55 nm f=0.0972 <S**2>=0.000

73 -> 81 -0.10393

75 -> 82 0.16175

77 -> 84 0.46632

77 -> 85 0.21423

79 -> 93 0.21734

79 -> 94 -0.25483

80 -> 96 0.14518

Excited State 45: Singlet-A 6.2831 eV 197.33 nm f=0.0077 <S**2>=0.000

77 -> 84 0.13904

78 -> 89 -0.12266

79 -> 93 0.20267

79 -> 94 0.33239

80 -> 95 0.46878

80 -> 97 0.11398

80 -> 98 -0.12200

Excited State 46: Singlet-A 6.3170 eV 196.27 nm f=0.0381 <S**2>=0.000

73 -> 81 0.25592

77 -> 84 -0.14824

78 -> 88 0.29362

79 -> 94 -0.10995

79 -> 95 -0.15424

79 -> 96 -0.15335

80 -> 95 0.16998

80 -> 96 0.41266

Excited State 47: Singlet-A 6.3205 eV 196.16 nm f=0.0356 <S**2>=0.000

77 -> 84 -0.19199

77 -> 85 0.55316

77 -> 87 0.14574

78 -> 90 0.11403

79 -> 94 0.20683

80 -> 95 -0.10032

80 -> 97 0.11590

Excited State 48: Singlet-A 6.3376 eV 195.63 nm f=0.0277 <S**2>=0.000

73 -> 81 -0.13755

77 -> 84 -0.15980

77 -> 85 -0.19745

78 -> 88 -0.14006

78 -> 90 0.31880

80 -> 97 0.46999

Excited State 49: Singlet-A 6.3564 eV 195.05 nm f=0.0266 <S**2>=0.000

73 -> 81 0.17423

75 -> 82 -0.11877

77 -> 84 0.28990

78 -> 89 -0.10389

78 -> 92 0.10279

79 -> 93 -0.18276

79 -> 96 0.19654

80 -> 95 -0.20336

80 -> 96 0.16706

80 -> 97 0.19449

80 -> 98 -0.26416

Excited State 50: Singlet-A 6.3898 eV 194.03 nm f=0.0088 <S**2>=0.000

71 -> 81 -0.17257

72 -> 81 0.12082

73 -> 81 0.38851

77 -> 84 0.12176

78 -> 89 0.18298

78 -> 90 0.17430

78 -> 92 -0.10336

79 -> 93 -0.10104

80 -> 96 -0.11088

80 -> 98 0.35460

Excited State 51: Singlet-A 6.4420 eV 192.46 nm f=0.0007 <S**2>=0.000

78 -> 88 0.14901

78 -> 89 0.43680

78 -> 92 -0.18857

79 -> 95 0.29997

80 -> 95 0.13901

80 -> 97 0.20162

80 -> 98 -0.15020

Excited State 52: Singlet-A 6.4516 eV 192.18 nm f=0.0132 <S**2>=0.000

71 -> 81 0.12240

73 -> 81 -0.14287

78 -> 88 0.15162

78 -> 89 -0.13000

78 -> 90 0.35532

79 -> 95 0.27061

79 -> 96 0.19698

80 -> 96 0.16939

80 -> 97 -0.24294

80 -> 98 0.15579

Excited State 53: Singlet-A 6.4677 eV 191.70 nm f=0.0280 <S**2>=0.000

73 -> 81 0.19002

74 -> 82 0.15905

75 -> 82 0.12956

77 -> 84 -0.11848

78 -> 88 -0.13341

78 -> 89 -0.24677

78 -> 90 -0.23578
79 -> 93 0.11605
79 -> 95 0.33046
79 -> 96 0.20641
80 -> 98 0.10324

Excited State 54: Singlet-A 6.4813 eV 191.30 nm f=0.0304 <S**2>=0.000
71 -> 81 0.15001
73 -> 81 -0.15165
78 -> 88 0.12604
78 -> 90 -0.31955
79 -> 95 -0.15294
79 -> 96 0.11353
80 -> 97 0.18962
80 -> 98 0.38214

Excited State 55: Singlet-A 6.4883 eV 191.09 nm f=0.0064 <S**2>=0.000
74 -> 82 0.25308
78 -> 89 0.13351
79 -> 95 -0.34015
79 -> 96 0.40483
79 -> 97 -0.16554
80 -> 97 -0.11720

Excited State 56: Singlet-A 6.5373 eV 189.66 nm f=0.0190 <S**2>=0.000
74 -> 82 -0.18681
77 -> 88 -0.11999
78 -> 92 -0.21582

79 -> 96 0.28416

79 -> 97 0.42081

79 -> 98 -0.19510

80 ->100 -0.10931

Excited State 57: Singlet-A 6.5490 eV 189.32 nm f=0.0116 <S**2>=0.000

73 -> 82 0.12360

74 -> 82 0.31063

76 -> 83 -0.18522

77 -> 86 0.16139

77 -> 88 0.19732

78 -> 91 0.12165

79 -> 97 0.39288

79 -> 98 0.14500

Excited State 58: Singlet-A 6.5605 eV 188.99 nm f=0.0011 <S**2>=0.000

71 -> 81 -0.12054

74 -> 82 -0.12557

76 -> 83 0.12284

77 -> 88 -0.14687

78 -> 89 0.21842

78 -> 91 -0.13814

78 -> 92 0.33870

79 -> 96 0.12164

79 -> 97 0.21907

79 -> 98 0.26082

80 -> 99 -0.14688

80 ->100 0.15073

Excited State 59:   Singlet-A   6.5832 eV 188.33 nm f=0.0055 <S**2>=0.000

74 -> 82   -0.13026

78 -> 91   0.57671

78 -> 92   0.14271

80 -> 96   0.10932

80 -> 99   -0.11794

Excited State 60:   Singlet-A   6.6005 eV 187.84 nm f=0.0064 <S**2>=0.000

77 -> 88   -0.11210

78 -> 91   0.17371

78 -> 92   -0.24610

79 -> 96   -0.11546

79 -> 98   0.51800

80 -> 99   0.17495

80 ->100   -0.11010

Compound 2c:

Cartesian Coordinates for **2c** (ground state):

```
C 2.51048 -1.44257 0.04265
C 3.10269 -0.25667 0.36214
C 2.36180 0.95696 0.28150
C 0.29993 -0.36231 -0.01042
C 1.09164 -1.51780 -0.17538
C 0.48836 -2.74677 -0.56516
C -0.85433 -2.82663 -0.75716
C -1.70389 -1.75170 -0.36779
C -3.10432 -1.92680 -0.38920
C -3.88355 -0.96993 0.18959
C -1.97725 0.33241 0.88308
C -3.34293 0.11656 0.87327
C 0.36441 2.13839 -0.43845
C 3.02572 2.19782 0.40845
C 2.36036 3.36762 0.13979
C 1.03687 3.34169 -0.32757
C 0.96985 0.91936 -0.01040
C -1.12978 -0.54706 0.14101
C -1.46114 1.40006 1.81705
O -0.86223 2.03109 -1.01384
C -1.53395 3.21705 -1.42199
O 3.16472 -2.62967 -0.05751
C 4.57360 -2.63383 0.16174
H 4.15509 -0.19507 0.58746
H 1.12350 -3.59873 -0.74800
H -1.30550 -3.73622 -1.12871
H -3.54129 -2.82065 -0.80896
H -4.00268 0.75808 1.43987
H 4.07174 2.20638 0.68098
H 2.86804 4.31765 0.23121
H 0.56684 4.26190 -0.63259
H -0.45828 1.17544 2.16942
H -1.43123 2.38468 1.35511
H -2.11917 1.46564 2.68181
H -2.48974 2.89038 -1.81850
H -1.69945 3.88745 -0.57671
H -0.97427 3.74132 -2.19778
H 4.89369 -3.65998 0.01445
H 5.08083 -1.98296 -0.55159
H 4.81106 -2.31818 1.17848
F -5.23740 -1.11373 0.18603
```

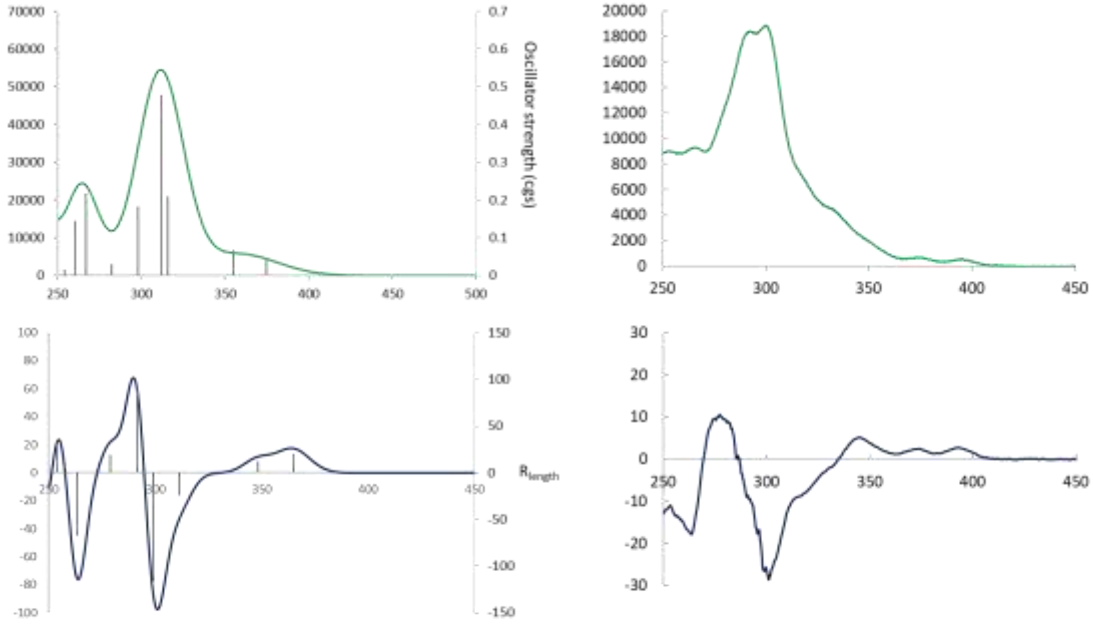

Fig. S123. ε = f(λ) (green),Δε = f(λ) (blue) for **2c** (left: calculated; right experimental). Excitations/oscillator strengths and rotatory strengths (R (length)) are depicted in black in the calculated UV and ECD spectrum respectively. Units: ε and Δε ($M^{-1}.cm^{-1}$ or 10^{-40} esu^2 cm^2);

Excitation energies and oscillator strengths (from S_0):

Excited State 1: Singlet-A 3.3080 eV 374.80 nm f=0.0316 <S**2>=0.000

83 -> 85 0.21937

83 -> 86 0.18797

84 -> 85 0.62110

84 -> 86 -0.16306

Excited State 2: Singlet-A 3.4935 eV 354.90 nm f=0.0580 <S**2>=0.000

82 -> 85 -0.12232

83 -> 85 0.55031

84 -> 85 -0.27622

84 -> 86 -0.31597

Excited State 3: Singlet-A 3.9276 eV 315.67 nm f=0.2017 <S**2>=0.000

81 -> 85 -0.12643

82 -> 85 0.45731

83 -> 85 0.26637

83 -> 86 -0.35669

84 -> 86 0.23583

Excited State 4: Singlet-A 3.9726 eV 312.10 nm f=0.4698 <S**2>=0.000

82 -> 85 -0.33353

83 -> 85 0.24652

83 -> 86 0.12425

84 -> 86 0.54782

Excited State 5: Singlet-A 4.1596 eV 298.07 nm f=0.1735 <S**2>=0.000

81 -> 85 0.18889

82 -> 85 0.38910

83 -> 86 0.51038

84 -> 85 -0.14951

Excited State 6: Singlet-A 4.3935 eV 282.20 nm f=0.0219 <S**2>=0.000

81 -> 85 0.34877

81 -> 86 0.10057

82 -> 86 0.50413

83 -> 86 -0.13044

83 -> 88 0.12151

84 -> 87 -0.24544

Excited State 7: Singlet-A 4.6448 eV 266.93 nm f=0.2090 <S**2>=0.000

81 -> 85 -0.31746

82 -> 86 0.43371

83 -> 87 -0.13922

84 -> 87 0.34989

84 -> 88 0.21319

Excited State 8: Singlet-A 4.7596 eV 260.49 nm f=0.1368 <S**2>=0.000

81 -> 85 0.35791

83 -> 86 -0.14315

83 -> 87 0.11294

84 -> 87 0.51988

84 -> 88 -0.18022

Excited State 9: Singlet-A 4.8798 eV 254.08 nm f=0.0071 <S**2>=0.000

81 -> 86 -0.11711

83 -> 87 0.55501

84 -> 88 0.37706

Excited State 10: Singlet-A 5.0338 eV 246.30 nm f=0.0129 <S**2>=0.000

80 -> 85 -0.11202

84 -> 88 0.12886

84 -> 89 0.64913

Excited State 11: Singlet-A 5.0849 eV 243.83 nm f=0.0414 <S**2>=0.000

81 -> 86 0.57481

82 -> 87 0.13013

83 -> 87 0.10083

83 -> 88 -0.34854

Excited State 12: Singlet-A 5.1031 eV 242.96 nm f=0.0711 <S**2>=0.000

80 -> 85 0.49810

81 -> 85 -0.10653

82 -> 86 0.10067

83 -> 87 0.17528

83 -> 92 -0.12908

84 -> 88 -0.32104

84 -> 89 0.19073

84 -> 92 0.11640

Excited State 13: Singlet-A 5.2482 eV 236.24 nm f=0.0171 <S**2>=0.000

80 -> 86 -0.10587

81 -> 86 0.17873

83 -> 88 0.33176

83 -> 89 0.42237

84 -> 90 -0.29762

84 -> 92 -0.13941

Excited State 14: Singlet-A 5.2875 eV 234.48 nm f=0.0191 <S**2>=0.000

80 -> 86 0.14565

81 -> 86 -0.16178

83 -> 87 -0.12087

83 -> 88 -0.26252

83 -> 89 0.44635

84 -> 91 -0.23454

84 -> 92 0.23595

Excited State 15: Singlet-A 5.3353 eV 232.39 nm f=0.1329 <S**2>=0.000

80 -> 85 0.28883

80 -> 86 0.11161

81 -> 85 0.11603

82 -> 87 0.17205

83 -> 87 -0.16068

83 -> 88 -0.10204

84 -> 88 0.21669

84 -> 90 -0.26167

84 -> 91 0.37734

84 -> 92 0.10757

Excited State 16: Singlet-A 5.3735 eV 230.73 nm f=0.1660 <S**2>=0.000

80 -> 85 0.21258

83 -> 88 0.16381

83 -> 89 0.11148

84 -> 88 0.14823

84 -> 90 0.54263

84 -> 91 0.11797

84 -> 92 -0.12220

Excited State 17: Singlet-A 5.3996 eV 229.62 nm f=0.1472 <S**2>=0.000

80 -> 85 -0.22666

82 -> 87 -0.15123

83 -> 89 0.22322

83 -> 90 -0.11306

84 -> 88 -0.14965

84 -> 91 0.48412

84 -> 92 0.10591

Excited State 18: Singlet-A 5.4151 eV 228.96 nm f=0.0133 <S**2>=0.000

79 -> 86 0.12991

80 -> 85 -0.10444

80 -> 86 0.12476

81 -> 85 -0.11277

81 -> 87 -0.11777

82 -> 87 0.35096

82 -> 88 0.17904

83 -> 87 0.14142

83 -> 88 0.27696

83 -> 92 0.18383

84 -> 88 -0.11380

84 -> 92 0.29395

Excited State 19: Singlet-A 5.4981 eV 225.50 nm f=0.1252 <S**2>=0.000

79 -> 85 0.14502

80 -> 86 -0.10160

81 -> 86 0.17523

82 -> 87 -0.37378

83 -> 88 0.16584

83 -> 89 -0.10821

84 -> 88 0.10148

84 -> 92 0.44430

Excited State 20: Singlet-A 5.5899 eV 221.80 nm f=0.0264 <S**2>=0.000

79 -> 85 -0.10791

83 -> 89 0.15316

83 -> 90 0.58995

83 -> 91 -0.25141

Excited State 21: Singlet-A 5.6173 eV 220.72 nm f=0.0082 <S**2>=0.000

80 -> 86 0.10059

82 -> 89 0.11001

83 -> 90 0.25176

83 -> 91 0.56891

84 -> 94 0.12932

84 -> 95 -0.12961

Excited State 22: Singlet-A 5.6563 eV 219.20 nm f=0.0136 <S**2>=0.000

79 -> 85 -0.24690

80 -> 85 0.10450

80 -> 86 0.27736

82 -> 87 -0.26845

83 -> 90 -0.11821

83 -> 91 -0.12257

83 -> 92 0.38231

84 -> 93 -0.19877

Excited State 23: Singlet-A 5.6930 eV 217.78 nm f=0.0124 <S**2>=0.000

79 -> 85 -0.23448

83 -> 92 0.20572

84 -> 93 0.58098

Excited State 24: Singlet-A 5.7115 eV 217.08 nm f=0.0629 <S**2>=0.000

79 -> 85 0.18236

80 -> 86 0.48646

82 -> 88 -0.21602

83 -> 92 -0.26844

84 -> 93 0.17467

84 -> 95 0.13189

Excited State 25: Singlet-A 5.7552 eV 215.43 nm f=0.0125 <S**2>=0.000

82 -> 88 0.15050

82 -> 89 0.17642

83 -> 91 -0.21490

84 -> 94 0.45602

84 -> 95 -0.31402

84 -> 96 0.17616

Excited State 26: Singlet-A 5.7796 eV 214.52 nm f=0.0096 <S**2>=0.000

82 -> 89 -0.15519

82 -> 91 -0.12207

84 -> 94 0.44553

84 -> 95 0.27125

84 -> 96 -0.32848

Excited State 27: Singlet-A 5.8034 eV 213.64 nm f=0.1055 <S**2>=0.000

79 -> 85 -0.11386

81 -> 87 -0.20217

82 -> 88 0.38859

82 -> 89 0.10345

83 -> 92 -0.12335

84 -> 95 0.41115

84 -> 96 0.20550

Excited State 28: Singlet-A 5.8293 eV 212.69 nm f=0.0270 <S**2>=0.000

79 -> 85 0.12657

81 -> 87 0.16057

82 -> 88 -0.26747

83 -> 92 0.19688

84 -> 94 0.11220

84 -> 95 0.21843

84 -> 96 0.48857

Excited State 29: Singlet-A 5.8573 eV 211.67 nm f=0.0187 <S**2>=0.000

79 -> 85 0.25396

81 -> 87 0.17852

82 -> 89 0.46713

83 -> 92 0.17509

83 -> 93 0.16804

83 -> 94 -0.10124

84 -> 91 0.12211

84 -> 95 0.11577

84 -> 96 -0.15570

Excited State 30: Singlet-A 5.9083 eV 209.85 nm f=0.1351 <S**2>=0.000

79 -> 85 0.22309

82 -> 87 -0.11587

82 -> 88 0.17091

82 -> 89 -0.33663

83 -> 92 0.15699

83 -> 93 0.41776

84 -> 92 -0.14265

84 -> 95 -0.10249

Excited State 31: Singlet-A 5.9477 eV 208.46 nm f=0.2278 <S**2>=0.000

79 -> 85 -0.27028

81 -> 87 -0.15724

82 -> 87 0.11903

82 -> 88 -0.16878

82 -> 89 0.15102

83 -> 92 -0.10158

83 -> 93 0.43593

83 -> 95 -0.20892

83 -> 96 0.13420

84 -> 92 0.12646

Excited State 32: Singlet-A 5.9804 eV 207.32 nm f=0.0560 <S**2>=0.000

79 -> 85 -0.10123

81 -> 87 -0.11288

83 -> 94 -0.42861

83 -> 95 0.43438

83 -> 96 -0.15061

84 -> 94 0.10595

Excited State 33: Singlet-A 6.0026 eV 206.55 nm f=0.0137 <S**2>=0.000

81 -> 87 0.11700

83 -> 93 0.24282

83 -> 94 0.43042

83 -> 95 0.29283

83 -> 96 -0.29583

84 -> 98 0.11911

Excited State 34: Singlet-A 6.0412 eV 205.23 nm f=0.0890 <S**2>=0.000

79 -> 85 -0.13277

79 -> 86 -0.16711

80 -> 86 0.15300

81 -> 87 0.39494

82 -> 88 0.16209

83 -> 94 -0.13798

83 -> 96 0.19149

84 -> 97 -0.18901

84 -> 98 0.25536

84 ->100 0.12220

Excited State 35: Singlet-A 6.0605 eV 204.58 nm f=0.0033 <S**2>=0.000

83 -> 94 0.18297

83 -> 95 0.35030

83 -> 96 0.52197

84 -> 98 -0.18480

Excited State 36: Singlet-A 6.1081 eV 202.98 nm f=0.1679 <S**2>=0.000

78 -> 85 0.11425

81 -> 87 0.22686

82 -> 90 -0.19398

82 -> 92 -0.29112

83 -> 94 -0.10544

84 -> 97 0.38828

84 -> 98 -0.26234

84 ->101 -0.11401

Excited State 37: Singlet-A 6.1240 eV 202.46 nm f=0.0100 <S**2>=0.000

78 -> 85 -0.14293

81 -> 87 -0.10977

82 -> 91 -0.14575

84 -> 97 0.39446

84 -> 98 0.43789

Excited State 38: Singlet-A 6.1635 eV 201.16 nm f=0.0027 <S**2>=0.000

77 -> 85 0.16742

78 -> 85 0.49811

79 -> 86 0.16205

82 -> 90 0.27951

82 -> 92 0.20167

Excited State 39: Singlet-A 6.1872 eV 200.39 nm f=0.0012 <S**2>=0.000

78 -> 85 -0.14669

79 -> 86 -0.20301

82 -> 90 0.48087

82 -> 91 -0.21924

82 -> 92 -0.12573

84 -> 98 -0.21427

84 -> 99 -0.15282

Excited State 40: Singlet-A 6.2100 eV 199.65 nm f=0.0028 <S**2>=0.000

82 -> 89 -0.10734

82 -> 90 0.27841

82 -> 91 0.52537

84 -> 97 0.20690

Excited State 41: Singlet-A 6.2192 eV 199.36 nm f=0.0861 <S**2>=0.000

79 -> 86 0.45826

82 -> 92 -0.30989

83 -> 98 -0.11217

84 -> 97 -0.15871

84 -> 98 0.13249

84 -> 99 -0.11036

84 ->101 -0.15627

Excited State 42: Singlet-A 6.2459 eV 198.50 nm f=0.0182 <S**2>=0.000

77 -> 85 -0.19692

78 -> 85 -0.11892

79 -> 86 0.20860

81 -> 87 0.13529

82 -> 91 -0.20707

82 -> 92 0.26282

83 -> 97   0.10713

83 -> 98   -0.15767

84 -> 98   -0.12329

84 -> 99   0.31751

84 ->100   0.22960

Excited State 43:   Singlet-A   6.2839 eV 197.30 nm f=0.0217 <S**2>=0.000

77 -> 85   0.15422

79 -> 86   -0.11637

82 -> 92   -0.21362

82 -> 94   -0.11281

83 -> 97   0.15536

83 -> 98   0.12711

84 -> 97   -0.11409

84 -> 99   0.50216

84 ->100   -0.18324

84 ->101   -0.10090

Excited State 44:   Singlet-A   6.3182 eV 196.23 nm f=0.0109 <S**2>=0.000

77 -> 85   0.13343

82 -> 93   -0.14042

83 -> 97   0.16256

83 -> 98   0.24553

84 ->100   0.49920

84 ->101   -0.16573

Excited State 45:   Singlet-A   6.3280 eV 195.93 nm f=0.0406 <S**2>=0.000

77 -> 85   0.16412

81 -> 88   0.28872

81 -> 89   0.10474

82 -> 93 0.11692

83 -> 97 0.45413

84 -> 99 -0.11933

84 ->101 0.26566

Excited State 46: Singlet-A 6.3387 eV 195.60 nm f=0.0639 <S**2>=0.000

77 -> 85 0.23317

81 -> 88 0.40760

81 -> 89 0.12037

83 -> 97 -0.31120

83 -> 98 -0.30199

84 -> 99 0.11760

Excited State 47: Singlet-A 6.3521 eV 195.19 nm f=0.0511 <S**2>=0.000

77 -> 85 0.24682

81 -> 88 -0.26680

82 -> 92 -0.13112

83 -> 97 -0.17817

84 ->100 0.19747

84 ->101 0.44419

Excited State 48: Singlet-A 6.3861 eV 194.15 nm f=0.0178 <S**2>=0.000

81 -> 89 0.61226

81 -> 91 0.16346

83 -> 98 0.13977

Excited State 49: Singlet-A 6.4142 eV 193.30 nm f=0.0419 <S**2>=0.000

76 -> 85 0.10364

77 -> 85 0.31613

81 -> 88 -0.30494

82 -> 92 0.14429

83 -> 97 0.14706

83 -> 98 -0.27178

83 -> 99 0.10029

84 ->101 -0.25000

84 ->102 0.11671

Excited State 50: Singlet-A 6.4295 eV 192.84 nm f=0.0006 <S**2>=0.000

81 -> 89 -0.10796

82 -> 94 0.13826

82 -> 95 -0.21928

83 -> 98 0.14256

84 ->102 0.55078

Excited State 51: Singlet-A 6.4556 eV 192.06 nm f=0.0128 <S**2>=0.000

77 -> 85 0.22519

78 -> 85 -0.20917

80 -> 86 0.10105

82 -> 92 0.18798

82 -> 94 -0.14037

82 -> 96 0.11390

83 -> 98 0.26539

83 -> 99 -0.23984

83 ->100 -0.15861

84 ->100 -0.19273

Excited State 52: Singlet-A 6.4848 eV 191.19 nm f=0.0068 <S**2>=0.000

82 -> 92 0.11225

82 -> 93 0.56426

82 -> 96 -0.11707

83 ->100 0.11327

84 ->101 -0.17438

84 ->102 -0.18923

Excited State 53: Singlet-A 6.5037 eV 190.64 nm f=0.0186 <S**2>=0.000

78 -> 86 -0.20217

79 -> 86 0.10274

80 -> 87 -0.15926

82 -> 93 -0.11567

83 -> 98 0.11186

83 -> 99 0.49865

83 ->100 0.16237

84 ->101 0.10340

Excited State 54: Singlet-A 6.5439 eV 189.47 nm f=0.0010 <S**2>=0.000

82 -> 93 0.11047

82 -> 95 -0.29390

82 -> 96 0.40004

83 -> 98 -0.11038

83 ->100 -0.15794

83 ->101 0.20925

84 ->102 -0.19827

84 ->104 0.18799

Excited State 55: Singlet-A 6.5542 eV 189.17 nm f=0.0006 <S**2>=0.000

78 -> 86 0.17844

80 -> 87 0.11484

82 -> 93 -0.16001

82 -> 94 0.33857

82 -> 95 -0.22634

82 -> 96 -0.10260

83 -> 99 0.14515

83 ->100 -0.27966

83 ->101 -0.14588

84 ->102 -0.21354

Excited State 56: Singlet-A 6.5674 eV 188.79 nm f=0.0575 <S**2>=0.000

77 -> 86 0.11531

78 -> 86 0.22060

80 -> 87 0.27133

81 -> 92 0.13140

82 -> 93 0.14773

82 -> 94 -0.28439

82 -> 95 0.19418

83 -> 99 0.29514

83 ->100 -0.21700

Excited State 57: Singlet-A 6.5868 eV 188.23 nm f=0.0320 <S**2>=0.000

76 -> 85 -0.12244

80 -> 87 0.26308

81 -> 92 0.24912

82 -> 95 -0.16055

82 -> 96 0.15307

83 ->100 0.43522

Excited State 58: Singlet-A 6.5952 eV 187.99 nm f=0.0132 <S**2>=0.000

76 -> 85 -0.20295

80 -> 87 0.12383

82 -> 94 0.14077

82 -> 96 -0.16548

83 ->101 0.56327

83 ->102 0.12792

Excited State 59: Singlet-A 6.6251 eV 187.14 nm f=0.0008 <S**2>=0.000

82 -> 94 0.36645

82 -> 95 0.38776

82 -> 96 0.26176

83 ->102 0.13412

84 ->104 0.21556

Excited State 60: Singlet-A 6.6449 eV 186.59 nm f=0.1467 <S**2>=0.000

76 -> 85 0.14698

76 -> 86 -0.11211

77 -> 86 -0.26699

78 -> 86 -0.36284

80 -> 87 0.40315

84 ->104 -0.11189

Compound 2p:

Cartesian Coordinates for **2p** (ground state):

```
C 2.61536 -0.85088 -0.15670
C 2.71026 0.46084 0.18731
C 1.59072 1.30015 0.24725
C 0.31989 0.69970 0.00291
C 0.21184 -0.73441 -0.04452
C 1.38557 -1.50351 -0.30475
C 1.29079 -2.85839 -0.73838
C 0.07459 -3.44267 -0.87190
C -1.10170 -2.80004 -0.37504
C -2.31472 -3.51357 -0.32247
C -3.39133 -3.00207 0.36194
C -2.07674 -1.05621 1.03024
C -3.23902 -1.80898 1.07854
C -0.76688 1.60563 -0.32272
C 1.70922 2.69839 0.50042
C 0.62170 3.50543 0.42319
C -0.62692 2.99608 -0.04575
C -1.93038 1.19420 -1.01045
C -1.69775 3.87492 -0.31785
C -2.84914 3.42579 -0.91994
C -2.94574 2.07649 -1.29995
O 3.87186 -1.38666 -0.33953
C 4.76184 -0.40273 0.21618
O 4.03218 0.83639 0.27422
C -1.01411 -1.48601 0.18023
C -1.97020 0.11008 1.98284
H 2.19732 -3.38607 -0.99577
H -0.01577 -4.44412 -1.26961
H -2.36656 -4.48775 -0.78938
H -4.32288 -3.54803 0.41140
H -4.04059 -1.47903 1.72614
H 2.68008 3.09582 0.75767
H 0.70246 4.56120 0.64263
H -2.02588 0.16827 -1.32282
H -1.58126 4.92177 -0.07111
H -3.65825 4.11006 -1.13263
H -3.82002 1.72748 -1.83136
H 5.62393 -0.28533 -0.43123
H 5.04015 -0.70262 1.22893
H -2.52102 -0.12399 2.89267
H -0.93784 0.31848 2.25209
H -2.38923 1.02672 1.57108
```

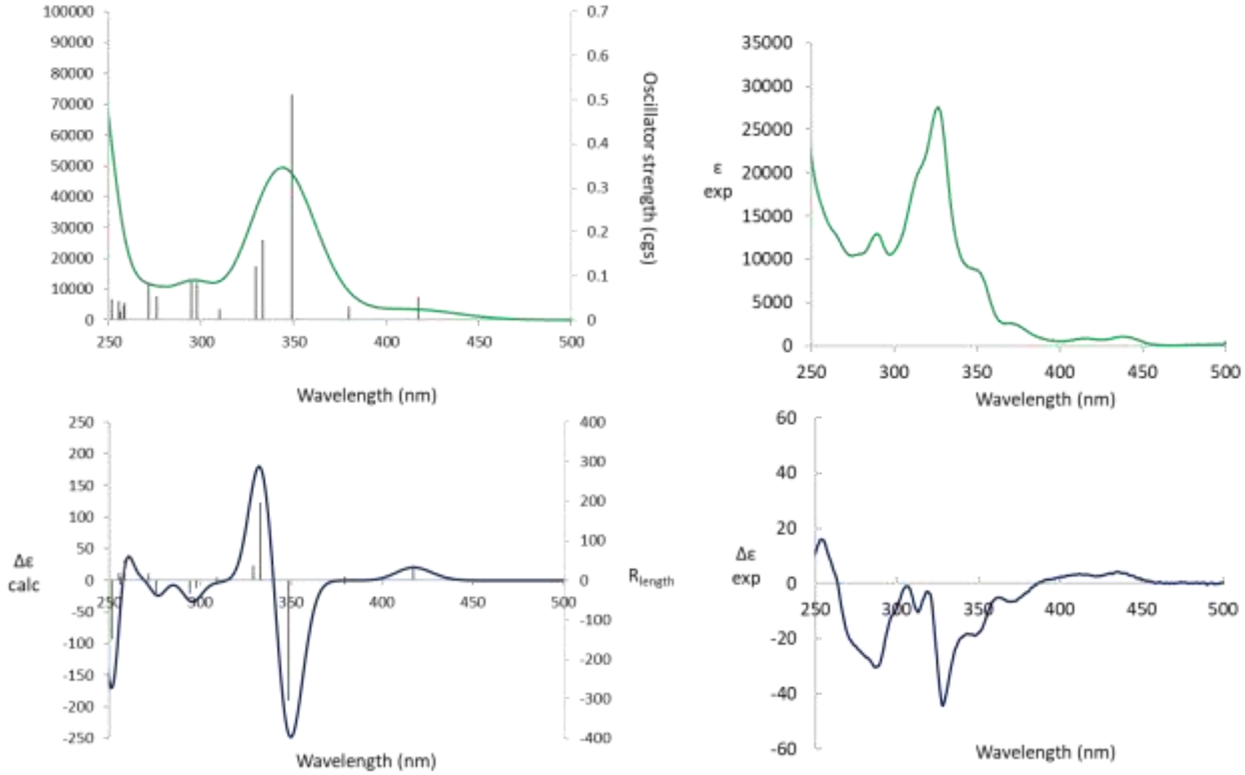

Fig. S124. ε = f(λ) (green),Δε = f(λ) (blue) for **2p** (left: calculated; right experimental). Excitations/oscillator strengths and rotatory strengths (R (length)) are depicted in black in the calculated UV and ECD spectrum respectively. Units: ε and Δε ($M^{-1}.cm^{-1}$ or 10^{-40} esu^2 cm^2);

Excitation energies and oscillator strengths (from S_0):

Excited State 1: Singlet-A 2.9722 eV 417.14 nm f=0.0432 <S**2>=0.000

87 -> 90 0.25404

88 -> 89 0.65605

Excited State 2: Singlet-A 3.2651 eV 379.73 nm f=0.0219 <S**2>=0.000

87 -> 89 -0.35788

88 -> 90 0.60681

Excited State 3: Singlet-A 3.5537 eV 348.89 nm f=0.5026 <S**2>=0.000

87 -> 89 0.57871

88 -> 90 0.34352

88 -> 91 -0.20072

Excited State 4: Singlet-A 3.7189 eV 333.39 nm f=0.1732 <S**2>=0.000

87 -> 90 0.64501

88 -> 89 -0.24638

Excited State 5: Singlet-A 3.7633 eV 329.46 nm f=0.1130 <S**2>=0.000

86 -> 90 0.20314

87 -> 89 0.15291

88 -> 91 0.64312

Excited State 6: Singlet-A 4.0048 eV 309.59 nm f=0.0157 <S**2>=0.000

86 -> 89 0.64639

87 -> 91 -0.26714

Excited State 7: Singlet-A 4.1640 eV 297.75 nm f=0.0771 <S**2>=0.000

84 -> 89 0.10397

85 -> 90 -0.13385

86 -> 89 0.20204

86 -> 90 -0.20194

87 -> 91 0.58153

88 -> 92 -0.20408

Excited State 8: Singlet-A 4.2108 eV 294.44 nm f=0.0785 <S**2>=0.000

85 -> 89 0.23135

86 -> 90 0.59279

87 -> 91 0.16095

88 -> 91 -0.18448

Excited State 9: Singlet-A 4.4946 eV 275.85 nm f=0.0454 <S**2>=0.000

85 -> 89 0.36277

85 -> 90 -0.23411

86 -> 90 -0.12425

87 -> 92 0.19217

88 -> 92 0.48110

Excited State 10: Singlet-A 4.5630 eV 271.72 nm f=0.0761 <S**2>=0.000

84 -> 89 -0.10771

85 -> 89 0.40128

85 -> 90 0.24378

86 -> 90 -0.18664

87 -> 92 0.29444

88 -> 92 -0.32600

Excited State 11: Singlet-A 4.7932 eV 258.67 nm f=0.0302 <S**2>=0.000

83 -> 90 0.10212

84 -> 89 0.57813

85 -> 90 0.18363

86 -> 92 0.12959

88 -> 93 -0.23177

Excited State 12: Singlet-A 4.8081 eV 257.87 nm f=0.0227 <S**2>=0.000

84 -> 89 0.22281

88 -> 93 0.56831

88 -> 94 0.24224

88 -> 95 0.15402

88 -> 96 -0.14072

Excited State 13: Singlet-A 4.8391 eV 256.21 nm f=0.0110 <S**2>=0.000

83 -> 90 0.18209

84 -> 89 -0.16128

85 -> 89 -0.11191

85 -> 90 -0.11707

87 -> 92 0.20029

88 -> 93 -0.21949

88 -> 94 0.53503

Excited State 14: Singlet-A 4.8558 eV 255.33 nm f=0.0340 <S**2>=0.000

84 -> 90 -0.22701

86 -> 91 0.60249

87 -> 92 -0.25083

Excited State 15: Singlet-A 4.9287 eV 251.56 nm f=0.0377 <S**2>=0.000

83 -> 89 -0.19734

84 -> 89 0.13761

84 -> 90 -0.21822

85 -> 89 -0.30473

86 -> 91 0.11474

87 -> 92 0.46134

88 -> 94 -0.19156

Excited State 16: Singlet-A 5.0147 eV 247.24 nm f=0.1649 <S**2>=0.000

83 -> 89 0.45843

84 -> 90 -0.44413

85 -> 90 -0.17699

86 -> 91 -0.14807

Excited State 17: Singlet-A 5.0457 eV 245.72 nm f=0.2572 <S**2>=0.000

83 -> 89 0.33315

84 -> 90 0.20595

85 -> 90 0.31266

86 -> 91 0.12394

87 -> 91 0.10467

87 -> 92 0.11182

87 -> 93 0.18714

87 -> 94 -0.29436

88 -> 92 0.15897

Excited State 18: Singlet-A 5.1154 eV 242.37 nm f=0.5349 <S**2>=0.000

84 -> 90 -0.28541

85 -> 90 0.30634

85 -> 91 -0.12375

86 -> 91 -0.11784

86 -> 92 -0.13605

87 -> 91 0.12228

87 -> 93 -0.27561

88 -> 92 0.18561

88 -> 94 0.19588

88 -> 95 -0.20514

Excited State 19: Singlet-A 5.1810 eV 239.31 nm f=0.0182 <S**2>=0.000

87 -> 93 -0.34372

87 -> 94 -0.17975

88 -> 93 -0.15585

88 -> 95 0.37748

88 -> 96 -0.37726

Excited State 20: Singlet-A 5.1936 eV 238.73 nm f=0.1657 <S**2>=0.000

83 -> 90 0.14050

84 -> 90 -0.12053

85 -> 90 0.15416

87 -> 93 0.36328

87 -> 94 0.29682

88 -> 92 0.10546

88 -> 95 0.3855

Excited State 21: Singlet-A 5.2193 eV 237.55 nm f=0.0040 <S**2>=0.000

83 -> 90 0.16753

85 -> 91 0.14349

87 -> 93 -0.26615

88 -> 95 0.28215

88 -> 96 0.48827

88 -> 97 0.10895

Excited State 22: Singlet-A 5.2787 eV 234.88 nm f=0.0019 <S**2>=0.000

83 -> 90 0.31724

85 -> 91 0.40513

86 -> 92 0.18009

87 -> 94 0.17908

88 -> 94 -0.13620

88 -> 95 -0.13912

88 -> 96 -0.24830

88 -> 97 0.14681

Excited State 23: Singlet-A 5.3044 eV 233.74 nm f=0.0036 <S**2>=0.000

82 -> 90 -0.11536

83 -> 89 0.15317

83 -> 90 -0.12307

84 -> 91 0.16537

85 -> 91 -0.18295

87 -> 93 -0.13287

87 -> 94 0.30630

88 -> 97 0.43485

88 -> 98 -0.16058

Excited State 24: Singlet-A 5.3505 eV 231.72 nm f=0.1897 <S**2>=0.000

83 -> 90 0.47352

85 -> 90 -0.16725

85 -> 91 -0.22491

86 -> 92 -0.34246

87 -> 94 -0.13345

Excited State 25: Singlet-A 5.3919 eV 229.95 nm f=0.0476 <S**2>=0.000

83 -> 89 -0.21791

84 -> 90 -0.15895

84 -> 91 -0.13461

85 -> 89 0.11325

86 -> 91 -0.13180

87 -> 93 0.13031

87 -> 94 -0.28739

88 -> 97 0.41494

88 -> 98 -0.13388

88 ->102 -0.13003

Excited State 26: Singlet-A 5.4863 eV 225.99 nm f=0.0038 <S**2>=0.000

84 -> 91 0.11592

86 -> 93 -0.15077

87 -> 95 0.57199

87 -> 96 0.22151

88 -> 97 -0.12268

88 -> 98 -0.20396

Excited State 27: Singlet-A 5.5064 eV 225.17 nm f=0.0130 <S**2>=0.000

87 -> 95 0.21506

88 -> 95 -0.10282

88 -> 97 0.16463

88 -> 98 0.57651

88 -> 99 -0.11240

88 ->101 -0.13093

88 ->103 -0.11861

Excited State 28: Singlet-A 5.5495 eV 223.41 nm f=0.0036 <S**2>=0.000

87 -> 95 -0.18095

87 -> 96 0.52638

88 -> 99 0.31051

88 ->100 0.21347

Excited State 29: Singlet-A 5.5607 eV 222.96 nm f=0.0093 <S**2>=0.000

87 -> 95 0.18528

87 -> 96 -0.34259

88 -> 99 0.47450

88 ->100 0.27614

Excited State 30: Singlet-A 5.6019 eV 221.33 nm f=0.0840 <S**2>=0.000

82 -> 89 -0.11062

84 -> 91 0.46632

85 -> 91 0.21238

86 -> 92 -0.22041

87 -> 97 -0.17329

88 -> 99 -0.11749

88 ->100 0.23141

88 ->102 -0.10102

Excited State 31: Singlet-A 5.6238 eV 220.46 nm f=0.0302 <S**2>=0.000

82 -> 89 0.27670

84 -> 91 0.27645

86 -> 92 0.17607

87 -> 95 -0.10077

87 -> 97 0.46490

87 -> 98 -0.14384

Excited State 32: Singlet-A 5.6422 eV 219.74 nm f=0.0141 <S**2>=0.000

84 -> 91 -0.14231

88 -> 98 -0.12462

88 -> 99 -0.26628

88 ->100 0.46414

88 ->101 -0.36524

Excited State 33: Singlet-A 5.6686 eV 218.72 nm f=0.0479 <S**2>=0.000

84 -> 91 -0.10448

85 -> 91 -0.10701

88 -> 99 -0.18324

88 ->100 0.25110

88 ->101 0.52724

88 ->102 0.11628

88 ->103 -0.10862

88 ->105 -0.10623

Excited State 34: Singlet-A 5.7413 eV 215.95 nm f=0.2976 <S**2>=0.000

83 -> 90 0.12762

83 -> 91 0.17180

84 -> 91 0.17243

85 -> 91 -0.31601

86 -> 92 0.34688

87 -> 97 -0.17896

87 -> 98 0.14222

88 ->101 -0.11618

88 ->102 -0.14291

88 ->103 -0.13050

Excited State 35: Singlet-A 5.7873 eV 214.23 nm f=0.0280 <S**2>=0.000

83 -> 91 0.27426

86 -> 93 0.36648

87 -> 97 0.28982

87 -> 98 0.36826

Excited State 36: Singlet-A 5.8172 eV 213.13 nm f=0.0026 <S**2>=0.000

82 -> 89 0.14211

83 -> 91 0.40671

86 -> 94 -0.21429

86 -> 96 -0.10004

87 -> 98 -0.37101

87 -> 99 -0.16052

88 ->103 0.22451

Excited State 37: Singlet-A 5.8312 eV 212.62 nm f=0.0239 <S**2>=0.000

83 -> 91 -0.11133

86 -> 92 0.11594

87 -> 98 0.13425

87 -> 99 0.12537

88 -> 98 0.10858

88 ->103 0.57757

88 ->104 -0.13195

88 ->105 0.10985

Excited State 38: Singlet-A 5.8629 eV 211.47 nm f=0.0129 <S**2>=0.000

82 -> 89 0.18481

83 -> 91 0.11528

86 -> 94 -0.11322

86 -> 95 0.14204

87 -> 97 -0.10366

87 -> 99 0.40559

87 ->100 -0.13205

88 ->102 0.35426

88 ->103 -0.10074

88 ->104 -0.13010

Excited State 39: Singlet-A 5.8792 eV 210.89 nm f=0.0248 <S**2>=0.000

83 -> 91 -0.11987

84 -> 91 0.13025

86 -> 93 0.19227

86 -> 95 -0.12562

87 -> 98 -0.11670

87 -> 99 -0.37606

88 ->102 0.40535

Excited State 40: Singlet-A 5.8999 eV 210.15 nm f=0.0085 <S**2>=0.000

83 -> 91 -0.11142

86 -> 93 0.45474

86 -> 94 0.28620

87 -> 95 0.12917

87 -> 96 0.13139

87 -> 97 -0.14151

87 -> 98 -0.17751

87 -> 99 0.12719

87 ->100 -0.12637

88 ->102 -0.18139

Excited State 41: Singlet-A 5.9173 eV 209.53 nm f=0.0716 <S**2>=0.000

82 -> 89 0.40613

83 -> 91 0.11103

86 -> 93 -0.12241

86 -> 94 0.19538

87 -> 97 -0.18106

87 -> 98 0.24354

87 -> 99 -0.23442

87 ->100 -0.18391

87 ->102 -0.19522

Excited State 42: Singlet-A 5.9453 eV 208.54 nm f=0.0639 <S**2>=0.000

82 -> 90 -0.22926

86 -> 94 0.25142

87 -> 99 0.14425

87 ->100 0.50738

87 ->101 0.15253

Excited State 43: Singlet-A 5.9911 eV 206.95 nm f=0.0055 <S**2>=0.000

82 -> 89 0.10020

85 -> 92 0.11684

88 ->102 0.11291

88 ->103 0.12073

88 ->104 0.60581

88 ->106 0.11695

Excited State 44: Singlet-A 5.9993 eV 206.66 nm f=0.1068 <S**2>=0.000

81 -> 89 -0.10451

82 -> 90 0.49090

83 -> 91 0.14923

86 -> 94 0.28441

87 ->100 0.12985

87 ->101 -0.11170

88 ->104 -0.10400

Excited State 45: Singlet-A 6.0466 eV 205.05 nm f=0.0216 <S**2>=0.000

82 -> 89 -0.22058

83 -> 91 0.23622

86 -> 94 0.26992

87 ->100 -0.30018

87 ->101 0.36452

88 ->104 0.10790

Excited State 46: Singlet-A 6.0610 eV 204.56 nm f=0.0134 <S**2>=0.000

82 -> 89 0.17309

82 -> 90 0.21903

83 -> 91 -0.17709

86 -> 94 -0.15363

87 -> 97 -0.10149

87 ->101 0.51269

Excited State 47: Singlet-A 6.0836 eV 203.80 nm f=0.0066 <S**2>=0.000

85 -> 92 -0.10426

88 ->105 0.45062

88 ->106 0.45675

Excited State 48: Singlet-A 6.1221 eV 202.52 nm f=0.0036 <S**2>=0.000

85 -> 92 0.12775

87 ->102 0.15005

88 ->105 0.47720

88 ->106 -0.42422

Excited State 49: Singlet-A 6.1662 eV 201.07 nm f=0.0146 <S**2>=0.000

80 -> 89 0.12599

81 -> 89 -0.30667

86 -> 95 -0.15297

87 ->102 0.48502

88 ->106 0.15737

88 ->107 0.10279

Excited State 50: Singlet-A 6.1926 eV 200.21 nm f=0.0189 <S**2>=0.000

85 -> 92 0.21070

86 -> 95 0.10306

87 ->103 -0.10775

88 ->107 0.60926

Excited State 51: Singlet-A 6.1943 eV 200.16 nm f=0.0044 <S**2>=0.000

86 -> 95 0.53105

86 -> 96 -0.16348

86 -> 97 0.12492

87 -> 99 -0.14676

87 ->102 0.13298

87 ->103 -0.21377

88 ->106 0.10173

88 ->107 -0.15371

Excited State 52: Singlet-A 6.2099 eV 199.66 nm f=0.0385 <S**2>=0.000

85 -> 92 -0.28436

86 -> 95 0.27209

86 -> 96 0.43169

86 -> 97 -0.10031

87 ->102 0.11211

87 ->103 0.17332

Excited State 53: Singlet-A 6.2173 eV 199.42 nm f=0.0329 <S**2>=0.000

81 -> 89 -0.13518

82 -> 90 -0.19138

85 -> 92 0.44903

86 -> 96 0.31805

87 ->103 0.16017

88 ->107 -0.16471

Excited State 54: Singlet-A 6.2596 eV 198.07 nm f=0.0311 <S**2>=0.000

79 -> 89 0.22305

80 -> 89 0.16072

81 -> 89 0.48654

82 -> 89 0.10168

87 ->102 0.24703

87 ->103 0.14273

Excited State 55: Singlet-A 6.2664 eV 197.86 nm f=0.0045 <S**2>=0.000

86 -> 95 0.10644

86 -> 96 -0.27447

86 -> 97 0.21724

87 ->103 0.53458

Excited State 56: Singlet-A 6.3039 eV 196.68 nm f=0.0224 <S**2>=0.000

83 -> 92 -0.13413

85 -> 92 -0.15438

85 -> 93 0.13000

85 -> 94 -0.10591

86 -> 95 -0.14249

86 -> 96 0.12194

86 -> 97 0.45281

86 -> 98 -0.16406

87 ->103 -0.17628

87 ->104 -0.15781

88 ->108 -0.18708

Excited State 57: Singlet-A 6.3358 eV 195.69 nm f=0.0185 <S**2>=0.000

80 -> 89 0.10629

84 -> 92 -0.11381

85 -> 93 0.11240

85 -> 94 -0.12877

86 -> 96 0.11715

86 -> 97 0.15999

86 -> 98 0.10337

86 -> 99 0.13334

87 ->104 0.51092

87 ->107 -0.14591

88 ->108 0.19771

Excited State 58: Singlet-A 6.3501 eV 195.25 nm f=0.0557 <S**2>=0.000

80 -> 89 0.15125

83 -> 92 0.33994

85 -> 93 -0.23465

85 -> 94 0.32806

86 -> 97 0.26123

87 ->104 0.14614

88 ->108 -0.11900

Excited State 59: Singlet-A 6.3541 eV 195.12 nm f=0.0027 <S**2>=0.000

81 -> 90 -0.11607

86 -> 97 0.13243

87 ->103 -0.10768

87 ->104 -0.21407

88 ->108 0.56515

Excited State 60: Singlet-A 6.3872 eV 194.11 nm f=0.0125 <S**2>=0.000

84 -> 92 0.22370

85 -> 93 0.23586

86 -> 99 -0.23115

87 ->105 0.45184

87 ->106 -0.19476

87 ->107 0.14558

88 ->109 0.11331

Compound 2u:

Cartesian Coordinates for **2u** (ground state):

```
C 3.37839 -0.92002 -0.00081
C 3.44268 0.37545 0.39371
C 2.29781 1.20611 0.36273
C 1.05736 0.64182 -0.01815
C 0.94335 -0.79715 -0.10053
C 2.13941 -1.54654 -0.27183
C 2.09691 -2.89428 -0.71405
C 0.89657 -3.48566 -0.96166
C -0.31490 -2.84494 -0.58965
C -1.55621 -3.53383 -0.74316
C -2.71333 -3.00655 -0.27141
C -1.47153 -1.10644 0.68937
C -2.69522 -1.81022 0.50544
C -3.83153 -0.28734 2.01200
C -2.60561 0.34660 2.27683
C -1.45957 -0.05366 1.63083
C 0.01277 1.56815 -0.40717
C 2.41380 2.59327 0.67133
C 1.34991 3.42091 0.51566
C 0.14413 2.94707 -0.07683
C -1.10703 1.17933 -1.17319
C -0.89168 3.84917 -0.40548
C -2.00377 3.43866 -1.09878
C -2.06429 2.10010 -1.49116
F 4.51243 -1.64261 -0.09952
F 4.63293 0.90945 0.73593
C -0.28782 -1.54943 -0.02247
H 3.02318 -3.42022 -0.88488
H 0.85052 -4.48339 -1.37518
H -1.55035 -4.48983 -1.24836
H -3.65452 -3.51889 -0.41600
H -4.73271 0.04573 2.50698
H -0.53111 0.43973 1.86353
H 3.35940 2.96801 1.03075
H 1.42579 4.46744 0.77573
H -1.22142 0.16980 -1.52656
H -0.78503 4.88699 -0.12279
H -2.79822 4.11887 -1.36584
C -3.86655 -1.35784 1.15131
H -4.79149 -1.89038 0.97568
H -2.55914 1.15102 2.99721
F -3.12971 1.70190 -2.23502
```

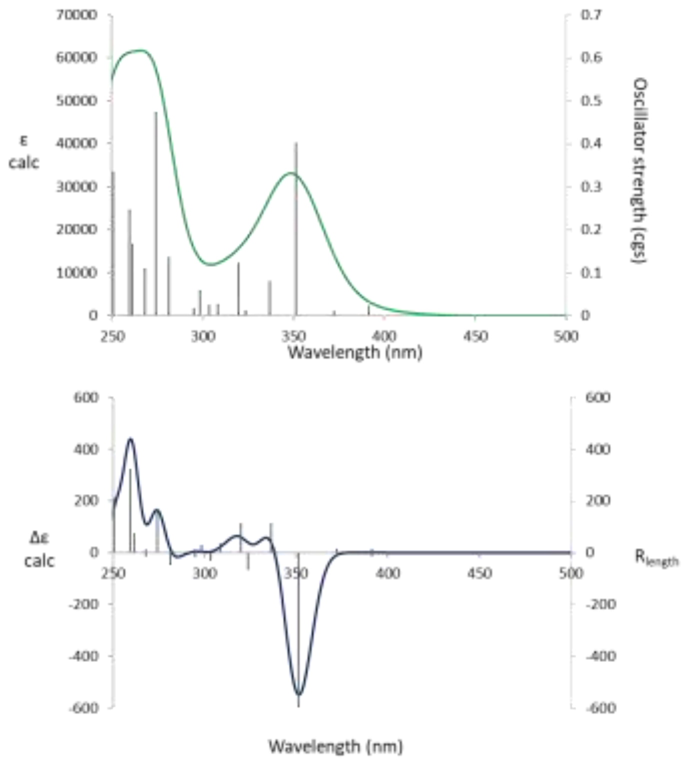

Fig. S125. ε = f(λ) (green),Δε = f(λ) (blue) for **2u** (left: calculated; right experimental). Excitations/oscillator strengths and rotatory strengths (R (length)) are depicted in black in the calculated UV and ECD spectrum respectively. Units: ε and Δε ($M^{-1}.cm^{-1}$ or 10^{-40} esu^2 cm^2);

Excitation energies and oscillator strengths (from S_0):

Excited State 1: Singlet-A 3.1663 eV 391.57 nm f=0.0159 <S**2>=0.000

97 -> 99 -0.33857

97 ->100 0.27304

98 -> 99 0.46846

98 ->100 0.28276

Excited State 2: Singlet-A 3.3309 eV 372.23 nm f=0.0017 <S**2>=0.000

97 -> 99 0.42971

97 ->100 0.16288

98 -> 99 0.41940

98 ->100 -0.32601

Excited State 3: Singlet-A 3.5298 eV 351.25 nm f=0.3928 <S**2>=0.000

97 -> 99 0.41306

97 ->100 0.20660

98 -> 99 -0.11253

98 ->100 0.50076

Excited State 4: Singlet-A 3.6835 eV 336.59 nm f=0.0728 <S**2>=0.000

95 -> 99 -0.24938

96 -> 99 0.18836

96 ->100 0.11968

97 ->100 0.50892

97 ->101 -0.10320

98 -> 99 -0.26247

98 ->100 -0.17277

Excited State 5: Singlet-A 3.8303 eV 323.69 nm f=0.0051 <S**2>=0.000

95 -> 99 0.27704

96 -> 99 0.60683

96 ->100 -0.14543

Excited State 6: Singlet-A 3.8754 eV 319.93 nm f=0.1136 <S**2>=0.000

95 -> 99 0.38030

95 ->100 -0.10745

96 -> 99 -0.23480

96 ->100 -0.25732

97 ->100 0.27252

97 ->101 0.12839

97 ->102 0.15396

98 -> 99 -0.11239

98 ->100 -0.13844

98 ->102 0.22353

Excited State 7: Singlet-A 4.0200 eV 308.42 nm f=0.0179 <S**2>=0.000

95 -> 99 0.33568

95 ->100 0.25485

96 ->100 0.26320

97 ->101 -0.17318

98 ->101 0.46012

Excited State 8: Singlet-A 4.0876 eV 303.32 nm f=0.0162 <S**2>=0.000

95 -> 99 -0.15438

95 ->100 0.39785

96 ->100 -0.29788

97 ->101 0.41290

98 ->101 0.22001

Excited State 9: Singlet-A 4.1567 eV 298.28 nm f=0.0503 <S**2>=0.000

95 ->100 0.37736

96 -> 99 -0.11044

97 ->101 -0.28658

97 ->102 0.21568

98 ->101 -0.29462

98 ->102 -0.31075

Excited State 10: Singlet-A 4.2025 eV 295.02 nm f=0.0102 <S**2>=0.000

95 ->100 0.21859

96 ->100 0.39862

97 ->101 0.22893

97 ->102 0.24117

98 ->101 -0.24557

98 ->102 0.32539

Excited State 11: Singlet-A 4.4064 eV 281.37 nm f=0.1281 <S**2>=0.000

94 -> 99 0.29396

95 ->100 -0.12699

95 ->101 0.18037

97 ->101 0.18742

97 ->102 0.42172

98 ->101 0.11619

98 ->102 -0.30851

Excited State 12: Singlet-A 4.5203 eV 274.28 nm f=0.4658 <S**2>=0.000

94 ->100 -0.16836

95 -> 99 -0.21185

96 ->100 -0.20758

96 ->101 -0.19337

97 ->101 -0.26925

97 ->102 0.34503

98 ->101 0.18386

98 ->102 0.28166

Excited State 13: Singlet-A 4.6256 eV 268.04 nm f=0.1007 <S**2>=0.000

95 ->100 0.13992

95 ->101 0.42305

96 ->101 -0.11344

96 ->102 0.38474

97 ->102 -0.14163

97 ->103 0.10263

98 ->102 0.12980

98 ->103 0.20197

Excited State 14: Singlet-A 4.7411 eV 261.51 nm f=0.1596 <S**2>=0.000

94 -> 99 0.51318

95 ->101 -0.30275

96 ->101 0.22405

97 ->103 0.10634

98 ->103 0.18982

Excited State 15: Singlet-A 4.7780 eV 259.49 nm f=0.2392 <S**2>=0.000

94 -> 99 -0.14026

94 ->100 -0.11722

95 ->101 0.20515

95 ->102 -0.16614

96 ->101 0.58198

97 ->102 0.11223

Excited State 16: Singlet-A 4.9467 eV 250.64 nm f=0.3270 <S**2>=0.000

94 ->100 0.57270

95 ->101 0.11250

95 ->102 0.15122

96 ->101 0.10269

96 ->102 -0.12152

97 ->103 0.20324

Excited State 17: Singlet-A 4.9959 eV 248.17 nm f=0.1611 <S**2>=0.000

94 ->100 0.10546

95 ->101 -0.27199

96 ->102 0.52642

97 ->103 -0.10468

98 ->103 -0.29812

Excited State 18: Singlet-A 5.0610 eV 244.98 nm f=0.0233 <S**2>=0.000

93 -> 99 0.15588

95 ->102 0.59194

96 ->101 0.11556

97 ->103 -0.24204

98 ->103 0.15545

Excited State 19: Singlet-A 5.1654 eV 240.03 nm f=0.0390 <S**2>=0.000

93 -> 99 0.50281

97 ->103 0.17690

98 ->103 -0.31788

98 ->105 -0.21177

Excited State 20: Singlet-A 5.2171 eV 237.65 nm f=0.0055 <S**2>=0.000

93 -> 99 0.20232

97 ->103 -0.13206

98 ->104 0.59315

98 ->105 0.18726

Excited State 21: Singlet-A 5.2528 eV 236.03 nm f=0.1127 <S**2>=0.000

92 -> 99 0.20886

93 -> 99 0.12132

93 ->100 0.11163

94 -> 99 -0.13937

95 ->101 -0.10102

97 ->103 0.24468

97 ->104 0.28826

98 ->103 0.12269

98 ->104 -0.18720

98 ->105 0.41698

Excited State 22: Singlet-A 5.2901 eV 234.37 nm f=0.1037 <S**2>=0.000

91 -> 99 -0.13259

93 ->100 0.10993

94 ->100 0.17035

94 ->101 -0.12420

95 ->102 -0.12254

97 ->103 -0.23028

97 ->104 0.32222

97 ->105 -0.25896

98 ->103 0.18759

98 ->104 -0.10929

98 ->105 -0.30172

Excited State 23: Singlet-A 5.3103 eV 233.48 nm f=0.0565 <S**2>=0.000

93 ->100 0.33712

94 -> 99 0.12869

96 ->102 -0.10338

97 ->103 -0.30387

97 ->104 -0.19535

97 ->105 -0.28033

98 ->103 -0.20446

98 ->105 0.23459

Excited State 24: Singlet-A 5.3608 eV 231.28 nm f=0.0681 <S**2>=0.000

92 -> 99 0.19942

93 -> 99 -0.16223

93 ->100 0.21380

94 -> 99 0.10727

94 ->101 0.10223

97 ->103 -0.15040

97 ->104 0.29493

97 ->105 0.42494

98 ->103 -0.15406

Excited State 25: Singlet-A 5.3923 eV 229.93 nm f=0.0184 <S**2>=0.000

92 -> 99 0.57808

97 ->104 -0.27188

98 ->105 -0.15365

98 ->108 -0.11559

Excited State 26: Singlet-A 5.4324 eV 228.23 nm f=0.0041 <S**2>=0.000

92 ->100 0.35982

93 -> 99 -0.12012

94 ->100 0.12266

94 ->101 0.38882

96 ->103 0.21934

97 ->105 -0.17145

98 ->108 0.19854

Excited State 27: Singlet-A 5.5047 eV 225.23 nm f=0.0343 <S**2>=0.000

92 ->100 -0.21926

93 -> 99 -0.22448

94 ->100 -0.12802

94 ->101 0.24321

95 ->102 0.12859

97 ->103 0.12117

97 ->104 0.20023

97 ->105 -0.25138

98 ->103 -0.14596

98 ->104 0.10838

98 ->106 0.28159

98 ->108 -0.14857

Excited State 28: Singlet-A 5.5185 eV 224.67 nm f=0.0197 <S**2>=0.000

93 -> 99 0.11848

97 ->105 0.12607

98 ->104 -0.12265

98 ->106 0.58171

98 ->107 0.12284

98 ->108 0.11999

Excited State 29: Singlet-A 5.5775 eV 222.29 nm f=0.1338 <S**2>=0.000

92 ->100 -0.18630

93 ->100 0.45410

94 -> 99 -0.14041

94 ->101 0.13998

95 ->101 -0.10998

97 ->103 0.12973

97 ->104 -0.16002

97 ->108 0.21307

98 ->104 0.10068

98 ->105 -0.12859

Excited State 30: Singlet-A 5.6385 eV 219.89 nm f=0.0069 <S**2>=0.000

92 ->100 0.34875

93 -> 99 -0.13574

93 ->100 0.20318

94 ->100 -0.11515

94 ->101 -0.24158

95 ->102 0.14029

96 ->103 -0.20184

97 ->103 0.14406

97 ->105 -0.10494

97 ->106 0.20917

97 ->108 -0.11914

98 ->104 0.10403

98 ->111 -0.11148

Excited State 31: Singlet-A 5.6682 eV 218.74 nm f=0.0026 <S**2>=0.000

92 ->100 -0.15673

97 ->106 0.58612

98 ->107 -0.27188

Excited State 32: Singlet-A 5.6974 eV 217.61 nm f=0.0034 <S**2>=0.000

94 ->102 -0.12709

97 ->106 0.24966

97 ->107 -0.12937

98 ->106 -0.12980

98 ->107 0.58000

98 ->109 0.11448

Excited State 33: Singlet-A 5.7233 eV 216.63 nm f=0.0302 <S**2>=0.000

94 ->102 0.52073

95 ->103 -0.33799

96 ->103 0.19157

98 ->107 0.1488

Excited State 34: Singlet-A 5.7322 eV 216.30 nm f=0.0267 <S**2>=0.000

91 -> 99 0.28035

94 ->102 -0.12551

95 ->103 0.12777

95 ->104 0.12214

97 ->106 0.11019

97 ->107 0.52139

97 ->108 0.16850

Excited State 35: Singlet-A 5.7499 eV 215.63 nm f=0.0900 <S**2>=0.000

91 -> 99 0.45260

97 ->107 -0.36763

97 ->108 0.23749

98 ->108 0.12436

Excited State 36: Singlet-A 5.7889 eV 214.18 nm f=0.0215 <S**2>=0.000

91 -> 99 -0.17084

91 ->100 -0.14615

92 -> 99 0.10023

92 ->100 -0.17432

95 ->103 -0.10097

96 ->104 0.13185

97 ->108 -0.10778

98 ->108 0.54402

Excited State 37: Singlet-A 5.8002 eV 213.76 nm f=0.3009 <S**2>=0.000

91 ->100 -0.10341

94 ->101 -0.28014

94 ->102 -0.15555

95 ->104 0.21813

95 ->105 -0.24258

96 ->103 0.41505

96 ->104 -0.13817

97 ->108 -0.14105

98 ->108 -0.12442

Excited State 38: Singlet-A 5.8492 eV 211.97 nm f=0.0222 <S**2>=0.000

90 -> 99 -0.10788

91 ->100 0.33802

94 ->102 -0.13536

95 ->103 -0.25945

95 ->105 -0.11891

96 ->103 0.12860

96 ->104 0.32979

96 ->105 -0.17925

97 ->108 0.21907

98 ->111 -0.11852

Excited State 39: Singlet-A 5.8947 eV 210.33 nm f=0.0575 <S**2>=0.000

91 ->100 0.31618

94 ->102 0.24067

95 ->103 0.41168

96 ->103 0.14863

96 ->105 0.18967

98 ->108 0.14465

98 ->109 -0.13798

Excited State 40: Singlet-A 5.9216 eV 209.38 nm f=0.0668 <S**2>=0.000

91 ->100 -0.14692

93 ->101 -0.20063

94 ->102 -0.14035

95 ->103 -0.19454

95 ->104 -0.28700

96 ->103 0.11496

96 ->104 0.14422

96 ->105 0.40802

98 ->109 -0.15417

Excited State 41: Singlet-A 5.9372 eV 208.83 nm f=0.0337 <S**2>=0.000

93 ->101 -0.12754

95 ->104 -0.19393

96 ->103 0.10601

96 ->106 0.19066

98 ->109 0.54658

Excited State 42: Singlet-A 5.9535 eV 208.25 nm f=0.0939 <S**2>=0.000

90 -> 99 0.13516

91 -> 99 0.11028

91 ->100 -0.17094

93 ->101 0.38570

95 ->103 0.12561

96 ->104 0.41031

97 ->108 -0.16557

Excited State 43: Singlet-A 5.9756 eV 207.48 nm f=0.1155 <S**2>=0.000

93 ->102 -0.12957

95 ->104 0.32836

96 ->103 -0.11471

96 ->104 0.13092

96 ->105 0.31899

97 ->108 0.21704

97 ->109 -0.22227

98 ->109 0.23724

Excited State 44: Singlet-A 5.9908 eV 206.96 nm f=0.0359 <S**2>=0.000

91 ->100 -0.15946

92 ->100 0.10656

93 ->102 0.17311

95 ->105 0.46312

96 ->103 0.19838

96 ->105 -0.18104

97 ->109 -0.23939

Excited State 45:   Singlet-A   6.0116 eV 206.24 nm f=0.0106 <S**2>=0.000

95 ->104   0.17445

95 ->105   0.18602

95 ->107   -0.14888

97 ->109   0.54886

97 ->111   -0.15221

Excited State 46:   Singlet-A   6.0409 eV 205.24 nm f=0.0051 <S**2>=0.000

90 -> 99   0.19556

91 -> 99   -0.14780

91 ->100   -0.19498

92 ->100   0.10353

95 ->104   -0.18821

95 ->105   -0.21905

96 ->105   -0.11807

97 ->108   0.30249

97 ->110   0.10739

97 ->111   -0.19426

98 ->110   0.15737

98 ->111   -0.13464

Excited State 47:   Singlet-A   6.0792 eV 203.95 nm f=0.0016 <S**2>=0.000

95 ->104   0.15534

95 ->105   0.13042

96 ->106   0.15507

97 ->109   -0.10309

98 ->109   -0.14088

98 ->110   0.48764

98 ->112   -0.15617

98 ->113   0.19015

Excited State 48:   Singlet-A   6.0861 eV 203.72 nm f=0.0143 <S**2>=0.000

92 ->101   0.11407

95 ->104 -0.15327

97 ->110 -0.25713

98 ->110 0.19936

98 ->111 0.50560

Excited State 49: Singlet-A 6.1132 eV 202.81 nm f=0.0145 <S**2>=0.000

93 ->101 -0.10005

95 ->106 0.21194

96 ->104 0.12343

96 ->106 0.17268

97 ->110 0.38649

98 ->110 0.12560

98 ->111 0.25313

98 ->112 0.33041

Excited State 50: Singlet-A 6.1243 eV 202.45 nm f=0.0294 <S**2>=0.000

89 -> 99 0.15899

90 -> 99 0.49875

92 ->101 0.13864

97 ->110 -0.15865

97 ->111 0.21465

97 ->112 -0.10432

98 ->112 0.22622

Excited State 51: Singlet-A 6.1753 eV 200.78 nm f=0.0194 <S**2>=0.000

91 -> 99 0.10041

93 ->101 -0.10400

95 ->106 -0.27486

97 ->110 -0.20184

97 ->111 -0.29997

97 ->112 0.17247

98 ->112 0.34227

98 ->113 0.19581

Excited State 52:   Singlet-A   6.1806 eV 200.60 nm f=0.0471 <S**2>=0.000

89 -> 99   -0.10182

92 ->101   0.26922

93 ->101   0.16897

96 ->106   0.30401

97 ->112   -0.17179

98 ->109   -0.12458

98 ->110   -0.20680

98 ->113   0.36669

Excited State 53:   Singlet-A   6.1979 eV 200.04 nm f=0.0270 <S**2>=0.000

89 -> 99   0.27218

90 -> 99   0.18301

92 ->101   -0.20171

93 ->101   -0.17229

97 ->110   0.14016

97 ->111   -0.14240

97 ->112   -0.19915

98 ->110   -0.13267

98 ->111   0.16170

98 ->112   -0.19308

98 ->113   0.26001

Excited State 54:   Singlet-A   6.2132 eV 199.55 nm f=0.0015 <S**2>=0.000

90 ->100   -0.11116

92 ->101   -0.12231

96 ->106   -0.29535

96 ->108   0.11439

97 ->111   0.32061

97 ->112   0.22899

98 ->113   0.34554

Excited State 55:   Singlet-A   6.2275 eV 199.09 nm f=0.0310 <S**2>=0.000

92 ->101   -0.16535

95 ->106   -0.13593

96 ->104   0.10354

96 ->106   0.40211

97 ->111   0.20792

97 ->112   0.26202

97 ->113   -0.11551

98 ->110   -0.15399

98 ->113   -0.13776

Excited State 56:   Singlet-A   6.2407 eV 198.67 nm f=0.0169 <S**2>=0.000

88 -> 99   0.17763

90 ->100   0.17408

92 ->101   0.37610

96 ->108   -0.10899

97 ->110   0.10417

97 ->112   0.34221

98 ->112   -0.21089

Excited State 57:   Singlet-A   6.2695 eV 197.76 nm f=0.0181 <S**2>=0.000

88 -> 99   0.16629

89 -> 99   -0.12196

92 ->102   -0.12665

93 ->101   -0.12218

95 ->106   0.45915

95 ->108   -0.11935

97 ->110   -0.31991

97 ->112   0.11985

97 ->113   0.10116

Excited State 58:   Singlet-A   6.2770 eV 197.52 nm f=0.0552 <S**2>=0.000

88 -> 99   0.24577

89 -> 99 -0.33232

93 ->102 0.14800

95 ->106 -0.18146

97 ->111 0.13520

97 ->113 0.38042

Excited State 59: Singlet-A 6.2871 eV 197.20 nm f=0.0123 <S**2>=0.000

88 -> 99 -0.28218

89 -> 99 0.21834

90 ->100 -0.10628

92 ->101 0.10790

96 ->108 0.12146

97 ->112 0.16021

97 ->113 0.48868

Excited State 60: Singlet-A 6.3002 eV 196.80 nm f=0.0774 <S**2>=0.000

88 -> 99 -0.25165

91 ->100 -0.19194

92 ->101 0.12356

92 ->102 0.32744

93 ->101 -0.13807

93 ->102 0.15199

95 ->106 0.14568

96 ->108 -0.25195

Optimization energies (with G_Grimme correction):

Compound	G (Hartrees)
2a	-961.556854
2c	-1060.831636
2p	-1074.714049
2u	-1298.310030

TD-DFT at the excited state, calculated g_{lum}, TEDM and TMDM:

The ground state (S_0) and first excited state (S_1) geometries were carried out with Gaussian 16 program,[27] using the B3LYP functional, the 6-311+G(2d,2p) basis set, Grimme's D3 correction with Becke-Johnson (BJ) damping,[28] and the IEF-PCM solvation model for dichloromethane.[29]

Compound 2a:

Cartesian Coordinates for **2a** (ground state):

```
C 2.52376 -0.96247 0.03454
C 2.84205 0.32676 0.34377
C 1.85067 1.34633 0.26620
C 0.12881 -0.39842 -0.00236
C 1.15496 -1.35162 -0.16993
C 0.83655 -2.68729 -0.54785
C -0.45749 -3.06082 -0.72654
C -1.52128 -2.19858 -0.33378
C -2.85006 -2.67287 -0.33676
C -3.84888 -1.93348 0.24591
C -2.23898 -0.22449 0.91502
C -3.51998 -0.74676 0.91734
C -0.36370 2.05225 -0.43933
C 2.22445 2.70390 0.38409
C 1.31409 3.69630 0.11952
C 0.02556 3.37543 -0.33592
C 0.49953 0.99957 -0.01286
C -1.22399 -0.89435 0.16614
C -1.96254 0.93901 1.83692
O -1.53863 1.67295 -1.00708
C -2.46465 2.67771 -1.40202
O 3.42438 -1.97599 -0.06723
C 4.80072 -1.66625 0.13766
H 3.85662 0.62070 0.55912
H 1.64327 -3.37864 -0.73180
H -0.70070 -4.04998 -1.09007
H -3.05524 -3.64523 -0.76368
H -4.86771 -2.29495 0.25601
H -4.28329 -0.24216 1.49513
H 3.24506 2.94490 0.64668
H 1.59916 4.73562 0.20462
H -0.63969 4.16700 -0.63860
H -0.93745 0.93209 2.19878
H -2.13215 1.90528 1.36589
H -2.62629 0.87652 2.69787
H -3.32700 2.14526 -1.78981
H -2.76704 3.29133 -0.55148
H -2.04701 3.31697 -2.18121
H 5.33945 -2.59646 -0.00960
H 5.14443 -0.92283 -0.58279
H 4.97202 -1.30016 1.15084
```

Cartesian Coordinates for **2a** (excited state):

```
C 2.54701 -0.95726 0.07224
C 2.84755 0.35817 0.31458
C 1.84095 1.36928 0.17285
C 0.13223 -0.38811 0.00399
C 1.20260 -1.36366 -0.19183
C 0.88549 -2.63534 -0.66575
C -0.43994 -2.98723 -0.88857
C -1.50900 -2.18216 -0.40596
C -2.83896 -2.64430 -0.42502
C -3.83595 -1.95214 0.25876
C -2.19618 -0.31443 1.01495
C -3.51050 -0.83196 1.00477
C -0.40678 2.04592 -0.43778
C 2.19082 2.71694 0.25855
C 1.24827 3.73852 0.04241
C -0.03563 3.40332 -0.33043
C 0.48295 0.98659 -0.05790
C -1.21337 -0.91710 0.20937
C -1.90756 0.83190 1.94606
O -1.57338 1.66853 -0.96496
C -2.55857 2.64202 -1.32448
O 3.46685 -1.95302 0.04162
C 4.83851 -1.61302 0.24191
H 3.85779 0.67548 0.51568
H 1.67911 -3.33945 -0.86207
H -0.67726 -3.95714 -1.30473
H -3.06848 -3.56239 -0.94897
H -4.85496 -2.31495 0.24134
H -4.26872 -0.33839 1.59809
H 3.21258 2.97572 0.49874
H 1.53790 4.77468 0.12985
H -0.74685 4.17565 -0.57250
H -0.85623 0.87420 2.22291
H -2.16839 1.80191 1.51829
H -2.49726 0.72034 2.85595
H -3.40996 2.06974 -1.67437
H -2.84394 3.23795 -0.45826
H -2.19097 3.28808 -2.12029
H 5.38890 -2.54395 0.15589
H 5.18121 -0.91065 -0.51864
H 4.99409 -1.18648 1.23343
```

Excitation energies and oscillator strengths (from S_1):

Excited State 1: Singlet-A 2.9027 eV 427.14 nm f=0.1423 <S**2>=0.000

80 -> 81 0.69145

Excited State 2: Singlet-A 3.1301 eV 396.10 nm f=0.1268 <S**2>=0.000

79 -> 81 -0.62086

80 -> 81 -0.10005

80 -> 82 0.29923

Excited State 3: Singlet-A 3.5692 eV 347.38 nm f=0.5813 <S**2>=0.000

78 -> 81 -0.28538

79 -> 81 0.31278

80 -> 82 0.55164

Compound 2c:

Cartesian Coordinates for **2c** (ground state):

```
C 2.51048 -1.44257 0.04265
C 3.10269 -0.25667 0.36214
C 2.36180 0.95696 0.28150
C 0.29993 -0.36231 -0.01042
C 1.09164 -1.51780 -0.17538
C 0.48836 -2.74677 -0.56516
C -0.85433 -2.82663 -0.75716
C -1.70389 -1.75170 -0.36779
C -3.10432 -1.92680 -0.38920
C -3.88355 -0.96993 0.18959
C -1.97725 0.33241 0.88308
C -3.34293 0.11656 0.87327
C 0.36441 2.13839 -0.43845
C 3.02572 2.19782 0.40845
C 2.36036 3.36762 0.13979
C 1.03687 3.34169 -0.32757
C 0.96985 0.91936 -0.01040
C -1.12978 -0.54706 0.14101
C -1.46114 1.40006 1.81705
O -0.86223 2.03109 -1.01384
C -1.53395 3.21705 -1.42199
O 3.16472 -2.62967 -0.05751
C 4.57360 -2.63383 0.16174
H 4.15509 -0.19507 0.58746
H 1.12350 -3.59873 -0.74800
H -1.30550 -3.73622 -1.12871
H -3.54129 -2.82065 -0.80896
H -4.00268 0.75808 1.43987
H 4.07174 2.20638 0.68098
H 2.86804 4.31765 0.23121
H 0.56684 4.26190 -0.63259
H -0.45828 1.17544 2.16942
H -1.43123 2.38468 1.35511
H -2.11917 1.46564 2.68181
H -2.48974 2.89038 -1.81850
H -1.69945 3.88745 -0.57671
H -0.97427 3.74132 -2.19778
H 4.89369 -3.65998 0.01445
H 5.08083 -1.98296 -0.55159
H 4.81106 -2.31818 1.17848
F -5.23740 -1.11373 0.18603
```

Cartesian Coordinates for **2c** (excited state):

```
C 2.52184 -1.44094 0.05976
C 3.10691 -0.21763 0.32106
C 2.35223 0.98315 0.19813
C 0.30209 -0.36439 0.00190
C 1.12340 -1.54411 -0.20605
C 0.53716 -2.72851 -0.66095
C -0.83138 -2.78336 -0.88322
C -1.68994 -1.75124 -0.42230
C -3.09413 -1.89282 -0.47485
C -3.87361 -0.97497 0.19711
C -1.95566 0.24074 0.98619
C -3.35332 0.05114 0.95700
C 0.32630 2.13072 -0.43142
C 3.00620 2.22237 0.28091
C 2.32380 3.42390 0.05130
C 0.99940 3.37446 -0.32926
C 0.94737 0.90941 -0.03939
C -1.12515 -0.57965 0.19928
C -1.42635 1.28554 1.93134
O -0.89436 2.03609 -0.96336
C -1.61906 3.20990 -1.34600
O 3.20699 -2.60197 0.02495
C 4.61621 -2.58062 0.26760
H 4.16252 -0.13776 0.52360
H 1.15919 -3.59024 -0.84473
H -1.27860 -3.67756 -1.29516
H -3.54933 -2.72666 -0.98831
H -4.01140 0.68667 1.53055
H 4.05856 2.23869 0.52659
H 2.83333 4.37110 0.13756
H 0.47629 4.28420 -0.57341
H -0.39572 1.08769 2.21632
H -1.46169 2.29331 1.51275
H -2.03436 1.29862 2.83592
H -2.57488 2.84796 -1.70670
H -1.77233 3.86407 -0.48848
H -1.09986 3.74434 -2.13995
H 4.94269 -3.61128 0.18258
H 5.12855 -1.96901 -0.47487
H 4.83132 -2.20863 1.26913
F -5.23125 -1.12299 0.14834
```

Excitation energies and oscillator strengths (from S_1):

Excited State 1: Singlet-A 2.8690 eV 432.15 nm f=0.0794 <S**2>=0.000

83 -> 85 0.10971

83 -> 86 -0.10232

84 -> 85 0.68526

Excited State 2: Singlet-A 3.1546 eV 393.03 nm f=0.1748 <S**2>=0.000

82 -> 85 -0.11520

83 -> 85 0.63199

84 -> 85 -0.12900

84 -> 86 0.25386

Excited State 3: Singlet-A 3.5894 eV 345.41 nm f=0.6063 <S**2>=0.000

82 -> 85 0.27494

83 -> 85 0.28564

83 -> 86 0.12558

84 -> 86 -0.56052

Compound 2d:

Cartesian Coordinates for **2d** (ground state):

C 2.51171 -1.47205 0.04012
C 3.11972 -0.29486 0.36201
C 2.39482 0.92888 0.28325
C 0.31496 -0.36367 -0.01146
C 1.09199 -1.52972 -0.17843
C 0.47214 -2.75174 -0.56393
C -0.87323 -2.81225 -0.74948
C -1.70802 -1.72571 -0.36130
C -3.11166 -1.87463 -0.37785
C -3.93442 -0.93133 0.19020
C -1.95967 0.35487 0.88167
C -3.32382 0.14169 0.86954
C 0.41473 2.13612 -0.43840
C 3.07442 2.16089 0.41329
C 2.42436 3.33994 0.14640
C 1.10163 3.33128 -0.32348
C 1.00293 0.90917 -0.00996
C -1.11609 -0.52994 0.14189
C -1.43222 1.42049 1.81294
O -0.81027 2.04488 -1.02044
C -1.47024 3.24004 -1.41830
O 3.15165 -2.66781 -0.06087
C 4.55935 -2.69002 0.16171
H 4.17272 -0.24762 0.58798
H 1.09516 -3.61281 -0.74596
H -1.33831 -3.71730 -1.11627
H -3.53035 -2.77400 -0.81102
H -3.95230 0.80770 1.44850
H 4.12017 2.15557 0.68727
H 2.94383 4.28332 0.24088
H 0.64349 4.25775 -0.62781
H -0.44236 1.17292 2.18826
H -1.36366 2.39984 1.34362
H -2.10418 1.51455 2.66455
H -2.42921 2.92624 -1.81767
H -1.62958 3.90490 -0.56736
H -0.90601 3.76625 -2.18969
H 4.86714 -3.71998 0.01424
H 5.07704 -2.04500 -0.54953
H 4.79885 -2.37833 1.17930
C -5.43303 -1.06170 0.16840
H -5.74105 -2.01597 -0.25520
H -5.85082 -0.98211 1.17314
H -5.88377 -0.26707 -0.42969

Cartesian Coordinates for **2d** (excited state):

C 2.52720 -1.47528 0.07641
C 3.12618 -0.26936 0.33724
C 2.39106 0.95343 0.19520
C 0.31715 -0.35029 -0.00784
C 1.12965 -1.55094 -0.20516
C 0.52667 -2.71239 -0.68577
C -0.84312 -2.74625 -0.91187
C -1.69474 -1.71277 -0.42906
C -3.09195 -1.84983 -0.45688
C -3.92823 -0.94842 0.21429
C -1.94053 0.26132 0.98124
C -3.33907 0.06112 0.95902
C 0.37625 2.14645 -0.43363
C 3.05491 2.17673 0.28947
C 2.38714 3.39233 0.06521
C 1.06322 3.37302 -0.32205
C 0.98334 0.90606 -0.05339
C -1.11206 -0.55144 0.18787
C -1.41130 1.31192 1.91851
O -0.84998 2.06535 -0.96269
C -1.55476 3.25027 -1.34060
O 3.18829 -2.65940 0.04469
C 4.59820 -2.65303 0.26542
H 4.18031 -0.20213 0.55246
H 1.13561 -3.58146 -0.88095
H -1.29983 -3.63323 -1.33000
H -3.52277 -2.69224 -0.98348
H -3.96401 0.71953 1.54906
H 4.10654 2.18174 0.53987
H 2.91384 4.33001 0.15869
H 0.56069 4.29423 -0.56682
H -0.37288 1.13207 2.18722
H -1.46834 2.31711 1.49624
H -2.00671 1.31931 2.83170
H -2.51737 2.90699 -1.70230
H -1.69612 3.90596 -0.48177
H -1.02729 3.77997 -2.13288
H 4.91565 -3.68657 0.17560
H 5.10779 -2.04493 -0.48282
H 4.83521 -2.28329 1.26359
C -5.42288 -1.10606 0.15122
H -5.72236 -2.13126 0.37533
H -5.92154 -0.44235 0.85558
H -5.79694 -0.87671 -0.84940

Excitation energies and oscillator strengths (from S1):

Excited State 1: Singlet-A 2.8976 eV 427.89 nm f=0.1401 <S**2>=0.000

84 -> 85 -0.69134

Excited State 2: Singlet-A 3.1336 eV 395.66 nm f=0.1314 <S**2>=0.000

82 -> 85 0.10364

83 -> 85 -0.62118

84 -> 85 0.10248

84 -> 86 -0.29674

Excited State 3: Singlet-A 3.5599 eV 348.28 nm f=0.3226 <S**2>=0.000

82 -> 85 0.48638

83 -> 85 0.27756

84 -> 86 -0.41141

Compound 2e:

Cartesian Coordinates for **2e** (ground state):

```
C 2.55629 -1.74632 0.03140
C 3.32797 -0.66306 0.33159
C 2.77794 0.64877 0.25315
C 0.53254 -0.34441 0.00914
C 1.13870 -1.60794 -0.16123
C 0.34710 -2.73334 -0.52121
C -0.99760 -2.60849 -0.68051
C -1.66652 -1.41275 -0.29222
C -3.07944 -1.37479 -0.29497
C -3.72969 -0.30481 0.27283
C -1.60350 0.69768 0.92547
C -2.97987 0.68552 0.93567
C 0.97201 2.11377 -0.44755
C 3.62443 1.77545 0.35741
C 3.14003 3.03113 0.08807
C 1.82051 3.20271 -0.35879
C 1.39138 0.82068 -0.01521
C -0.90313 -0.30812 0.18544
C -0.91719 1.68403 1.84059
O -0.26503 2.18975 -1.00611
C -0.75819 3.46173 -1.40795
O 3.02194 -3.02018 -0.06996
C 4.41753 -3.23608 0.12314
H 4.38140 -0.76068 0.53842
H 0.83937 -3.67514 -0.70448
H -1.58997 -3.44478 -1.02616
H -3.61606 -2.21639 -0.70318
H -3.52046 1.42539 1.51014
H 4.66440 1.62693 0.61260
H 3.78753 3.89367 0.16264
H 1.48987 4.18101 -0.66598
H 0.03683 1.30407 2.19666
H -0.72617 2.64269 1.36308
H -1.55233 1.87143 2.70472
H -1.75942 3.28196 -1.78580
H -0.80566 4.15128 -0.56310
H -0.14072 3.89507 -2.19614
H 4.57599 -4.29996 -0.01968
H 5.00362 -2.67490 -0.60589
H 4.72102 -2.95289 1.13195
O -5.08237 -0.14584 0.32373
C -5.88837 -1.15451 -0.28131
H -6.91639 -0.83030 -0.15660
H -5.66050 -1.25187 -1.34383
H -5.74637 -2.11746 0.21163
```

Cartesian Coordinates for **2e** (excited state):

```
C 2.55843 -1.74116 0.03895
C 3.32501 -0.61320 0.28028
C 2.76726 0.68906 0.16096
C 0.53243 -0.34206 0.00361
C 1.16110 -1.63692 -0.21135
C 0.39442 -2.72790 -0.63643
C -0.97335 -2.58724 -0.81734
C -1.65014 -1.43358 -0.33642
C -3.05583 -1.36114 -0.35878
C -3.70973 -0.32865 0.30982
C -1.58459 0.59040 1.04733
C -2.98045 0.60790 1.05125
C 0.92079 2.12054 -0.44809
C 3.60869 1.81147 0.21533
C 3.11071 3.09213 -0.02453
C 1.77943 3.23714 -0.38395
C 1.35950 0.82638 -0.05921
C -0.89380 -0.35261 0.24749
C -0.87773 1.54491 1.97226
O -0.33282 2.21435 -0.93264
C -0.86059 3.48648 -1.30414
O 3.06555 -2.99183 0.01899
C 4.46489 -3.17795 0.24791
H 4.38284 -0.69626 0.47070
H 0.88223 -3.67036 -0.83052
H -1.56101 -3.40603 -1.20877
H -3.60640 -2.13824 -0.86449
H -3.51852 1.33172 1.64618
H 4.65670 1.66670 0.43769
H 3.75496 3.95608 0.03455
H 1.40035 4.21483 -0.63302
H 0.10948 1.18195 2.24826
H -0.74959 2.53552 1.53386
H -1.46481 1.67139 2.88165
H -1.87540 3.29206 -1.63368
H -0.87798 4.16585 -0.45123
H -0.28685 3.92842 -2.11876
H 4.63167 -4.24738 0.17882
H 5.05473 -2.66364 -0.51063
H 4.74648 -2.82627 1.24024
O -5.06082 -0.18254 0.34007
C -5.86398 -1.12838 -0.36516
H -6.89114 -0.80924 -0.22187
H -5.62482 -1.12830 -1.42895
H -5.73287 -2.13228 0.03996
```

Excitation energies and oscillator strengths (from S_1):

Excited State 1: Singlet-A 2.9353 eV 422.39 nm f=0.0832 <S**2>=0.000

87 -> 89 0.14365

87 -> 90 -0.13503

88 -> 89 -0.66988

Excited State 2: Singlet-A 3.2199 eV 385.06 nm f=0.1299 <S**2>=0.000

87 -> 89 -0.60906

88 -> 89 -0.17778

88 -> 90 0.28895

Excited State 3: Singlet-A 3.5734 eV 346.96 nm f=0.0393 <S**2>=0.000

86 -> 89 -0.66668

87 -> 89 -0.15071

87 -> 90 -0.12017

Compound 2f:

Cartesian Coordinates for **2f** (ground state):

C 2.59736 -1.56851 0.04664
C 3.25759 -0.41722 0.35948
C 2.59135 0.83869 0.27543
C 0.45733 -0.35638 -0.01681
C 1.17693 -1.55950 -0.17424
C 0.50438 -2.75344 -0.56266
C -0.83882 -2.75392 -0.76034
C -1.62123 -1.62789 -0.37220
C -3.02350 -1.71093 -0.40300
C -3.78223 -0.71133 0.17174
C -1.77255 0.46530 0.88725
C -3.14470 0.33566 0.86957
C 0.66356 2.13498 -0.43829
C 3.32834 2.03754 0.39769
C 2.73138 3.24417 0.13002
C 1.40651 3.29644 -0.33053
C 1.19980 0.88344 -0.01435
C -0.97896 -0.45810 0.13692
C -1.18962 1.48993 1.83006
O -0.57073 2.09796 -1.00447
C -1.17947 3.32101 -1.40441
O 3.17901 -2.79216 -0.04600
C 4.58610 -2.87920 0.17183
H 4.31175 -0.41773 0.58474
H 1.09042 -3.64039 -0.74189
H -1.34386 -3.63278 -1.13581
H -3.49611 -2.57628 -0.84374
H -3.75014 1.02673 1.43805
H 4.37408 1.98489 0.66608
H 3.29445 4.16267 0.21810
H 0.98960 4.24294 -0.63230
H -0.21060 1.18896 2.19292
H -1.08033 2.47059 1.37243
H -1.85071 1.60145 2.68753
H -2.15570 3.04875 -1.79191
H -1.29868 3.99730 -0.55624
H -0.59858 3.81398 -2.18492
H 4.84366 -3.92342 0.02994
H 5.12944 -2.26378 -0.54617
H 4.84301 -2.57253 1.18652
C -5.20810 -0.77981 0.14144
N -6.36095 -0.82621 0.11615

Cartesian Coordinates for **2f** (excited state):

C 2.59418 -1.57234 0.05158
C 3.24965 -0.40283 0.41410
C 2.56724 0.83643 0.30386
C 0.43407 -0.37272 0.02034
C 1.20301 -1.58027 -0.21588
C 0.53200 -2.74763 -0.64446
C -0.82364 -2.75534 -0.78290
C -1.63116 -1.65130 -0.33545
C -3.01979 -1.73037 -0.37686
C -3.81066 -0.70332 0.18894
C -1.79027 0.44931 0.93351
C -3.16503 0.33326 0.88604
C 0.66033 2.10776 -0.44275
C 3.29239 2.04189 0.41158
C 2.70307 3.27802 0.12102
C 1.40909 3.31742 -0.33337
C 1.17803 0.86737 0.01686
C -0.97807 -0.48398 0.19676
C -1.20680 1.47740 1.87209
O -0.52733 2.08209 -1.03325
C -1.14793 3.28529 -1.51309
O 3.21918 -2.76256 -0.06456
C 4.62803 -2.83213 0.17205
H 4.30206 -0.39454 0.64137
H 1.10994 -3.63280 -0.86151
H -1.32909 -3.63967 -1.14632
H -3.49174 -2.59490 -0.81998
H -3.76919 1.04278 1.43560
H 4.32713 1.99978 0.72002
H 3.27282 4.18887 0.22354
H 0.95828 4.25409 -0.61394
H -0.25086 1.14964 2.27734
H -1.04485 2.44939 1.40718
H -1.88830 1.63660 2.70635
H -2.09908 2.96534 -1.92115
H -1.31194 3.98302 -0.69420
H -0.54223 3.74483 -2.29112
H 4.89993 -3.86895 0.00683
H 5.17050 -2.19295 -0.52403
H 4.86380 -2.55038 1.19791
C -5.21981 -0.75091 0.13255
N -6.37991 -0.79463 0.08279

Excitation energies and oscillator strengths (from S_1):

Excited State 1: Singlet-A 2.7178 eV 456.19 nm f=0.1684 <S**2>=0.000

86 -> 87 -0.70034

Excited State 2: Singlet-A 3.0259 eV 409.75 nm f=0.0315 <S**2>=0.000

85 -> 87 0.58787

86 -> 88 0.37476

Excited State 3: Singlet-A 3.3757 eV 367.28 nm f=0.6143 <S**2>=0.000

84 -> 87 -0.12396

85 -> 87 -0.35830

86 -> 88 0.58910

Compound 2p:

Cartesian Coordinates for **2p** (ground state):

```
C 2.61536 -0.85088 -0.15670
C 2.71026 0.46084 0.18731
C 1.59072 1.30015 0.24725
C 0.31989 0.69970 0.00291
C 0.21184 -0.73441 -0.04452
C 1.38557 -1.50351 -0.30475
C 1.29079 -2.85839 -0.73838
C 0.07459 -3.44267 -0.87190
C -1.10170 -2.80004 -0.37504
C -2.31472 -3.51357 -0.32247
C -3.39133 -3.00207 0.36194
C -2.07674 -1.05621 1.03024
C -3.23902 -1.80898 1.07854
C -0.76688 1.60563 -0.32272
C 1.70922 2.69839 0.50042
C 0.62170 3.50543 0.42319
C -0.62692 2.99608 -0.04575
C -1.93038 1.19420 -1.01045
C -1.69775 3.87492 -0.31785
C -2.84914 3.42579 -0.91994
C -2.94574 2.07649 -1.29995
O 3.87186 -1.38666 -0.33953
C 4.76184 -0.40273 0.21618
O 4.03218 0.83639 0.27422
C -1.01411 -1.48601 0.18023
C -1.97020 0.11008 1.98284
H 2.19732 -3.38607 -0.99577
H -0.01577 -4.44412 -1.26961
H -2.36656 -4.48775 -0.78938
H -4.32288 -3.54803 0.41140
H -4.04059 -1.47903 1.72614
H 2.68008 3.09582 0.75767
H 0.70246 4.56120 0.64263
H -2.02588 0.16827 -1.32282
H -1.58126 4.92177 -0.07111
H -3.65825 4.11006 -1.13263
H -3.82002 1.72748 -1.83136
H 5.62393 -0.28533 -0.43123
H 5.04015 -0.70262 1.22893
H -2.52102 -0.12399 2.89267
H -0.93784 0.31848 2.25209
H -2.38923 1.02672 1.57108
```

Cartesian Coordinates for **2p** (excited state):

```
C 2.62498 -0.82750 -0.15913
C 2.69546 0.51207 0.19176
C 1.57663 1.35966 0.24677
C 0.32763 0.70258 -0.03776
C 0.24427 -0.74000 -0.03787
C 1.41860 -1.52259 -0.34894
C 1.32923 -2.85034 -0.77789
C 0.08250 -3.44811 -0.89009
C -1.06310 -2.81369 -0.35380
C -2.31095 -3.47614 -0.31527
C -3.36169 -2.94964 0.40378
C -1.99939 -1.05490 1.10108
C -3.18698 -1.77743 1.15045
C -0.77096 1.56910 -0.38825
C 1.65537 2.73854 0.49776
C 0.52416 3.52542 0.41019
C -0.68492 2.97303 -0.07951
C -1.91583 1.10682 -1.07485
C -1.81473 3.78630 -0.32367
C -2.94206 3.28230 -0.93354
C -2.98039 1.93809 -1.34553
O 3.87094 -1.34799 -0.23554
C 4.78282 -0.29106 0.10145
O 3.98823 0.87751 0.35476
C -0.95748 -1.49702 0.23946
C -1.85628 0.11869 2.03687
H 2.23068 -3.38286 -1.04272
H -0.01652 -4.44388 -1.29744
H -2.41129 -4.42706 -0.82068
H -4.30826 -3.47182 0.44020
H -3.98138 -1.43593 1.79990
H 2.61160 3.16845 0.75768
H 0.56600 4.58062 0.63794
H -1.95714 0.07852 -1.39363
H -1.76677 4.83126 -0.04795
H -3.78845 3.92833 -1.12172
H -3.84324 1.55658 -1.87203
H 5.44267 -0.10503 -0.74234
H 5.32841 -0.55687 1.00384
H -2.38050 -0.10008 2.96656
H -0.81532 0.32657 2.27235
H -2.28517 1.03215 1.62547
```

Excitation energies and oscillator strengths (from S_1):

Excited State 1: Singlet-A 2.5796 eV 480.63 nm f=0.0858 <S**2>=0.000

87 -> 90 0.16656

88 -> 89 0.68527

Excited State 2: Singlet-A 2.9949 eV 413.98 nm f=0.0782 <S**2>=0.000

87 -> 89 -0.26439

88 -> 90 0.65398

Excited State 3: Singlet-A 3.2305 eV 383.79 nm f=0.6342 <S**2>=0.000

87 -> 89 0.63390

88 -> 90 0.25679

88 -> 91 -0.16788

Compound 2x:

Cartesian Coordinates for **2x** (ground state):

```
C 3.05387 -1.37666 0.12066
C 3.31501 -0.09215 0.46799
C 2.32908 0.91479 0.34189
C 1.03313 0.54971 -0.09187
C 0.68409 -0.85226 -0.12445
C 1.74151 -1.79998 -0.19376
C 1.49082 -3.14367 -0.57651
C 0.22035 -3.54201 -0.85741
C -0.88026 -2.68871 -0.57983
C -2.21350 -3.17197 -0.75132
C -3.28682 -2.43905 -0.36353
C -1.78069 -0.71065 0.53574
C -3.09637 -1.21253 0.33763
N -4.20132 -0.60160 0.84021
C -4.03539 0.45998 1.60433
C -2.77324 0.96009 1.96214
C -1.65374 0.37483 1.42608
C 0.17858 1.61726 -0.57474
C 2.65780 2.27652 0.60969
C 1.75538 3.26015 0.36638
C 0.51967 2.96775 -0.28133
C -0.94256 1.39281 -1.40386
C -0.33308 4.01245 -0.70033
C -1.45447 3.75182 -1.45003
C -1.73813 2.42946 -1.83182
F 4.05323 -2.28184 0.11496
F 4.55919 0.25206 0.85712
C -0.65791 -1.38412 -0.08363
H 2.32179 -3.82472 -0.67372
H 0.02523 -4.54037 -1.22274
H -2.34711 -4.14636 -1.20077
H -4.29840 -2.78842 -0.50889
H -4.93463 0.93249 1.98158
H -2.69294 1.78783 2.65095
H -0.67745 0.73645 1.70170
H 3.63281 2.50590 1.01061
H 1.99101 4.28983 0.59687
H -1.17311 0.39160 -1.72586
H -0.06859 5.02918 -0.44324
H -2.09604 4.55922 -1.77306
H -2.58387 2.22309 -2.47225
```

Cartesian Coordinates for **2x** (excited state):

```
C 3.14240 -1.18690 0.12189
C 3.31928 0.15022 0.43216
C 2.28469 1.08712 0.29902
C 1.00294 0.60380 -0.14662
C 0.74980 -0.81129 -0.12496
C 1.88884 -1.71405 -0.19643
C 1.72452 -3.06735 -0.55963
C 0.46204 -3.55578 -0.81102
C -0.67911 -2.77425 -0.52888
C -1.97208 -3.30350 -0.71963
C -3.09434 -2.61520 -0.33215
C -1.69489 -0.80962 0.58165
C -2.98985 -1.37866 0.36219
N -4.13377 -0.81853 0.81766
C -4.04288 0.28341 1.55520
C -2.82097 0.86180 1.91277
C -1.64914 0.31831 1.42709
C 0.06884 1.59121 -0.62156
C 2.49522 2.45567 0.56471
C 1.50115 3.37228 0.32506
C 0.29445 2.97818 -0.30826
C -1.05311 1.27405 -1.42056
C -0.67564 3.93289 -0.67623
C -1.79301 3.57140 -1.39740
C -1.96295 2.23504 -1.80005
F 4.21463 -1.99684 0.16909
F 4.53838 0.55254 0.81062
C -0.53789 -1.43093 -0.01871
H 2.59641 -3.69273 -0.66780
H 0.32524 -4.56485 -1.17237
H -2.06696 -4.28055 -1.17334
H -4.08217 -3.02429 -0.48567
H -4.97479 0.71921 1.89330
H -2.80138 1.72998 2.55525
H -0.70194 0.74834 1.70580
H 3.44884 2.77120 0.95970
H 1.65329 4.41646 0.55696
H -1.19715 0.25701 -1.74488
H -0.51267 4.96632 -0.40271
H -2.52347 4.31700 -1.67741
H -2.81368 1.95813 -2.40576
```

Excitation energies and oscillator strengths (from S_1):

Excited State 1: Singlet-A 2.9126 eV 425.68 nm f=0.0302 <S**2>=0.000

93 -> 95 0.37149

93 -> 96 -0.17776

94 -> 95 0.50580

94 -> 96 0.25353

Excited State 2: Singlet-A 3.0700 eV 403.86 nm f=0.0091 <S**2>=0.000

93 -> 95 0.43384

93 -> 96 0.10229

94 -> 95 -0.44163

94 -> 96 0.32036

Excited State 3: Singlet-A 3.2525 eV 381.19 nm f=0.5041 <S**2>=0.000

93 -> 95 0.39197

93 -> 96 0.22447

94 -> 96 -0.52583

Transition electric dipole moments (*μ*) of S_1-S_0 in optimized S_1 geometry:

Compound	***μ*** in atomic units (a.u.)			
	x	y	z	***μ***
**2a**	-0.1552	1.4057	0.0368	1.41
**2c**	-0.2673	1.028	0.0289	1.4
**2d**	0.1031	1.3987	0.0831	1.03
**2e**	-0.423	0.9822	0.1132	1.07
**2f**	1.5894	-0.0436	-0.0273	1.59
**2p**	1.164	0.028	-0.039	1.16
**2x**	0.1393	0.621	-0.1326	0.65

Transition magnetic dipole moments (*m*) of S_1-S_0 in optimized S_1 geometry:

Compound	***m*** in atomic units (a.u.)			
	x	y	z	***m***
**2a**	0.2385	-0.1526	-0.7668	0.82
**2c**	0.1722	-0.2234	-0.6941	0.75
**2d**	0.2128	-0.1913	-0.8143	0.86
**2e**	0.1391	-0.2368	-0.6303	0.69
**2f**	-0.0546	0.2632	-0.2958	0.40
**2p**	-0.2475	0.1430	-0.1142	0.31
**2x**	0.0515	-0.0024	-0.8517	0.85

Summary of the calculated photophysical and chiroptical properties of S_1-S_0 in optimized S_1 geometry:

	λ	f	***μ***	***m***	θ	cosθ	R	g_{lum}
**2a**	427.14	0.1423	359.6	0.75	76.0	0.24	66.3824	0.0020
**2c**	432.15	0.0794	270.12	0.70	68.2	0.37	70.2483	0.0038
**2d**	427.89	0.1401	357.1	0.80	75.0	0.25	74.2847	0.0023
**2e**	422.39	0.0832	273.5	0.63	60.6	0.49	85.9738	0.0045
**2f**	456.19	0.1684	404.2	0.37	81.8	0.14	21.4821	0.0005
**2p**	480.63	0.0858	296.1	0.3	38.7	0.77	66.4975	0.0030
**2x**	425.68	0.0302	165.2	0.80	102.3	-0.21	-28.1756	-0.0041

Units[31]: λ: nm; **μ**: 10^{-20} esu·cm (conversion from atomic units (a.u.) to CGS Units: μ in esu·cm = (254.17 × 10^{-20}) × μ in a.u.); **m** = 10^{-20} erg/Gauss (conversion from atomic units (a.u.) to CGS Units: m in erg·G^{-1} = (−1) × (0.92847643 × 10^{-20}) × |m| in a.u.); θ: degrees; R: 10^{-40} erg.esu.cm.G^{-1}

DFT Comparison between the experimental and theoretical glum values (x 1000):

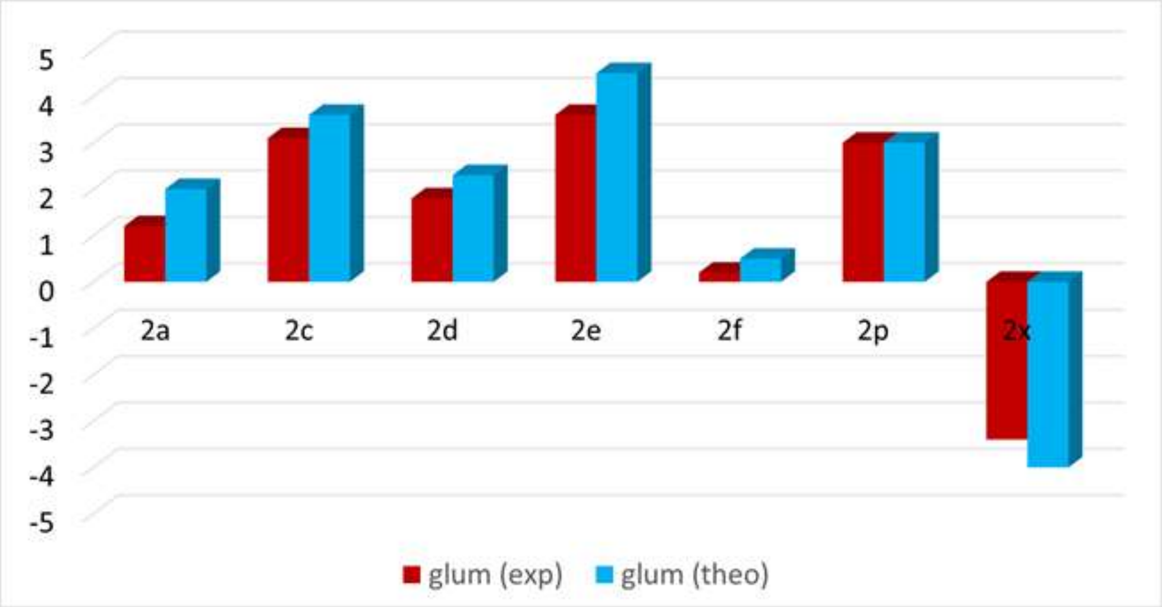

References

1. Savary, D. & Baudoin, O. Enantioselective Pd^0-catalyzed $C(sp^2)$–H arylation for the synthesis of chiral warped molecules. *Angew. Chem. Int. Ed*. **60**, 5136–5140 (2021).
2. Li, Y.-W., Zheng, H.-X., Yang, B., Shan, X.-H., Qu, J.-P. & Kang, Y.-B. *t*BuOK-promoted cyclization of imines with aryl halides. *Org. Lett.* **22**, 4553−4556 (2020).
3. Ishida, N., Okumura, S. & Murakami., M. Site- and regio-selective incorporation of carbon dioxide into the $C(sp^2)$-Si bond of benzosilacyclobutenes. *Chem. Lett.* **47**, 570–572 (2018).
4. Johnson, K., Schmidt, A. & Stanley, L. Rhodium-catalyzed, enantioselective hydroacylation of ortho-allylbenzaldehydes. *Org. Lett.* **17**, 4654−4657 (2015).
5. Partridge, B., Thomas, S. & Aggarwal, V. Enantioenriched synthesis of escitalopram using lithiationeborylation methodology. *Tetrahedron* **67**, 10082−10088 (2011).
6. Chen, X., Li, M., Liu, Z., Yang, C., Xie, H., Hu, X., Su, S.-J., Jiang, H. & Zeng, W. Bimetal cooperatively catalyzed arylalkynylation of alkynylsilanes. *Org. Lett.* **23**, 6724−6728 (2021).
7. Stegbauer, S, Jandl, C. & Bach, T. Enantioselective Lewis acid catalyzed ortho photocycloaddition of olefins to phenanthrene-9-carboxaldehydes. *Angew. Chem. Int. Ed.* **57**, 14593–14596 (2018).
8. Flores-Gaspar, A. & Martin, R. Mechanistic switch via subtle ligand modulation: palladium-catalyzed synthesis of a,b-substituted styrenes via C–H bond functionalization. *Adv. Synth. Catal.* **353**, 1223–1228 (2011).
9. Holzwarth, R., Bartsch, R., Cherkaoui, Z. & Solladié, G. New 2,2’-substituted 4,4’-dimethoxy-6,6’-dimethyl[1,1’-biphenyls], inducing a strong helical twisting power in liquid crystals. *Chem. Eur. J.* **10**, 3931–3935 (2004).
10. Rubial, B., Collins, B., Bigler, R., Aichhorn, S., Noble, A. & Aggarwal, V. K. Enantiospecific synthesis of ortho-substituted 1,1-diaryl alkanes by a 1,2-metalate rearrangement/anti-S_N2'

elimination/rearomatizing allylic Suzuki–Miyaura reaction sequence. *Angew. Chem. Int. Ed.* **58**, 1366–1370 (2019).

11. Wang, J., Chen, M.-W., Ji, Y., Hu, S.-B. & Zhou, Y.-G. Kinetic resolution of axially chiral 5- or 8-Substituted quinolines via asymmetric transfer hydrogenation. *J. Am. Chem. Soc.* **138**, 10413−10416 (2016).
12. Kamikawa, K., Takemoto, I., Takemoto, S. & Matsuzaka, H. Synthesis of helicenes utilizing Palladium-catalyzed double C-H arylation reaction. *J. Org. Chem.* **72**, 7406−7408 (2007).
13. Singh, I. & Seitz, O. Diastereoselective synthesis of *β*-aryl-*C*-nucleosides from 1,2-anhydrosugars. *Org. Lett*. **8**, 4319−4322 (2006).
14. Nagel, D. L., Kupper, R., Antonson, K. & Wallcave, L. Synthesis of alkyl-substituted benzo[c]phenanthrenes and chrysenes by photocyclization. *J. Org. Chem.* **42**, 3626−3628 (1977).
15. Yang, Z., Chen, P., Hao, W., Xie, Z., Feng, Y., Xing, G. & Chen, L. Sulfonated 2D covalent organic frameworks for efficient proton conduction. *Chem. Eur. J.* **27**, 3817–3822 (2021).
16. Bachmann, W. E. & Safir, S. R. 7-Methylcholanthrene and 1,5-dimethyl-1,2-benzanthracene. *J. Am. Chem. Soc*. **63**, 855–857 (1941).
17. Gaussian 09, revision D.01, Frisch, M. J. et al. Gaussian, Inc., Wallingford, CT (2013).
18. Becke, A. D. Density-functional thermochemistry. III. The role of exact exchange *J. Chem. Phys*. **98**, 5648–5652 (1993).
19. Lee, C., Yang, W. & Parr, R. G. Development of the Colle-Salvetti correlation-energy formula into a functional of the electron density. *Phys. Rev. B* **37**, 785–789 (1988).

20. Grimme, S., Antony, J., Ehrlich, S. & Krieg, H. A consistent and accurate ab initio parametrization of density functional dispersion correction (DFT-D) for the 94 elements H-Pu. *J. Chem. Phys.* **132**, 154104–154119 (2010).

21. Andrae, D., Haussermann, U., Dolg, M., Stoll, H., Preuss, H. Energy-adjustedab initio pseudopotentials for the second and third row transition elements. *Theor. Chim. Acta.* **77**,123–125 (1990).

22. Weigend, F. & Ahlrichs, R. Balanced basis sets of split valence, triple zeta valence and quadruple zeta valence quality for H to Rn: Design and assessment of accuracy. *Phys. Chem. Chem. Phys*. **7**, 3297–3305 (2005).

23. Cancès, E., Mennucci, B. & Tomasi, J. A new integral equation formalism for the polarizable continuum model: Theoretical background and applications to isotropic and anisotropic dielectrics. *J. Chem. Phys.* **107**, 3032–3041 (1997).

24. Lu, T. & Chen, F. Multiwfn: a multifunctional wavefnction analyzer. *J. Comput. Chem.* **33**, 580–592 (2012).

25. Humphrey, W., Dalke, A. & Schulten, K. VMD: Visual molecular dynamics. *J. Mol. Graphics* **14**, 33–38 (1996).

26. Legault, C. Y. CYLview, 1.0b. Université de Sherbrooke. (2009) (http://www.cylview.org).

27. Gaussian 16. Revision B.01. Frisch, M. J. et al. Gaussian, Inc., Wallingford, CT (2016).

28. Grimme, S.; Ehrlich, S.; Goerigk, L. Effect of the damping function in dispersion corrected density functional theory *J. Comput. Chem.* **32**, 1456–1465 (2011).

29. Tomasi, J.; Mennucci, B.; Cammi, R. Quantum mechanical continuum solvation models *Chem. Rev.* **105**, 2999–3093 (2005).

30. O'Boyle, N.M., Tenderholt, A.L., Langner, K.M. A Library for Package-Independent Computational Chemistry Algorithms *J. Comp. Chem.* **29**, 839-845 (2008).

31. For units conversion please see: https://gaussian.com/constants/ and Mohr, P. J. Taylor, B. N., Newell, D. B. CODATA Recommended Values of the Fundamental Physical Constants: 2010, *J. Phys. Chem. Ref. Data*, 41 043109-043184 (2012).

NMR Spectra

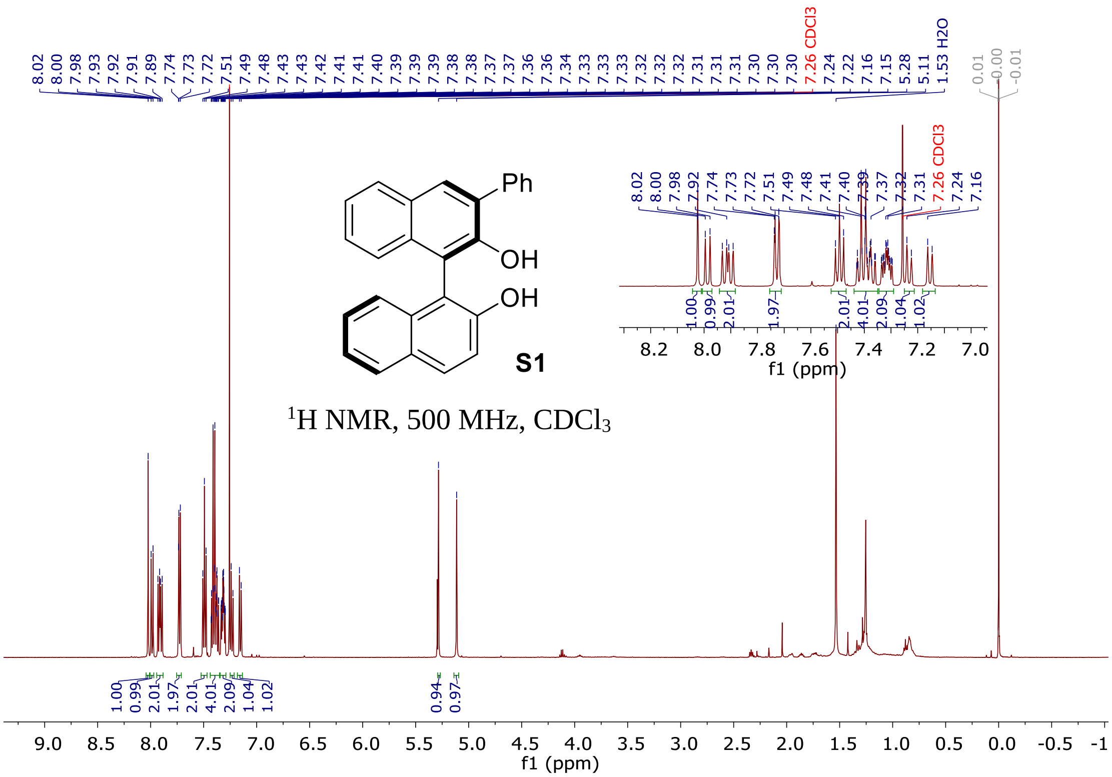

Ph
OH
OH
S1
¹H NMR, 500 MHz, CDCl₃
7.26 CDCl3
1.53 H2O
f1 (ppm)

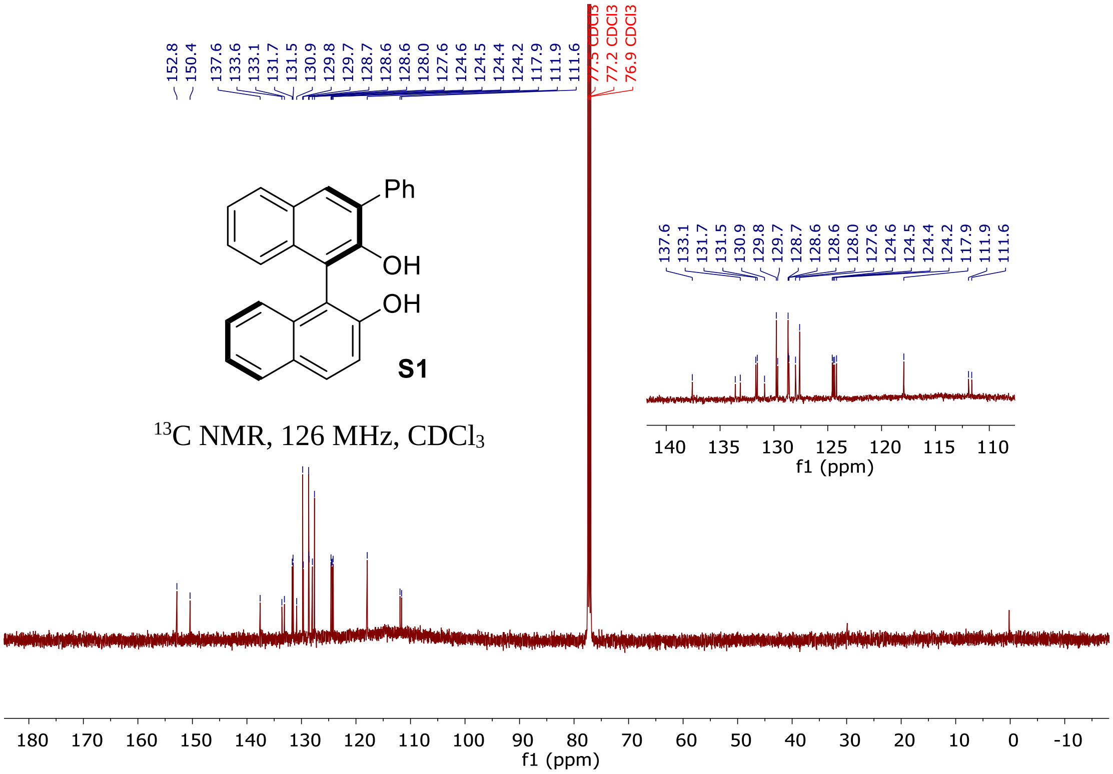

Ph
OH
OH
S1
¹³C NMR, 126 MHz, CDCl₃
77.2 CDCl3
76.9 CDCl3
f1 (ppm)

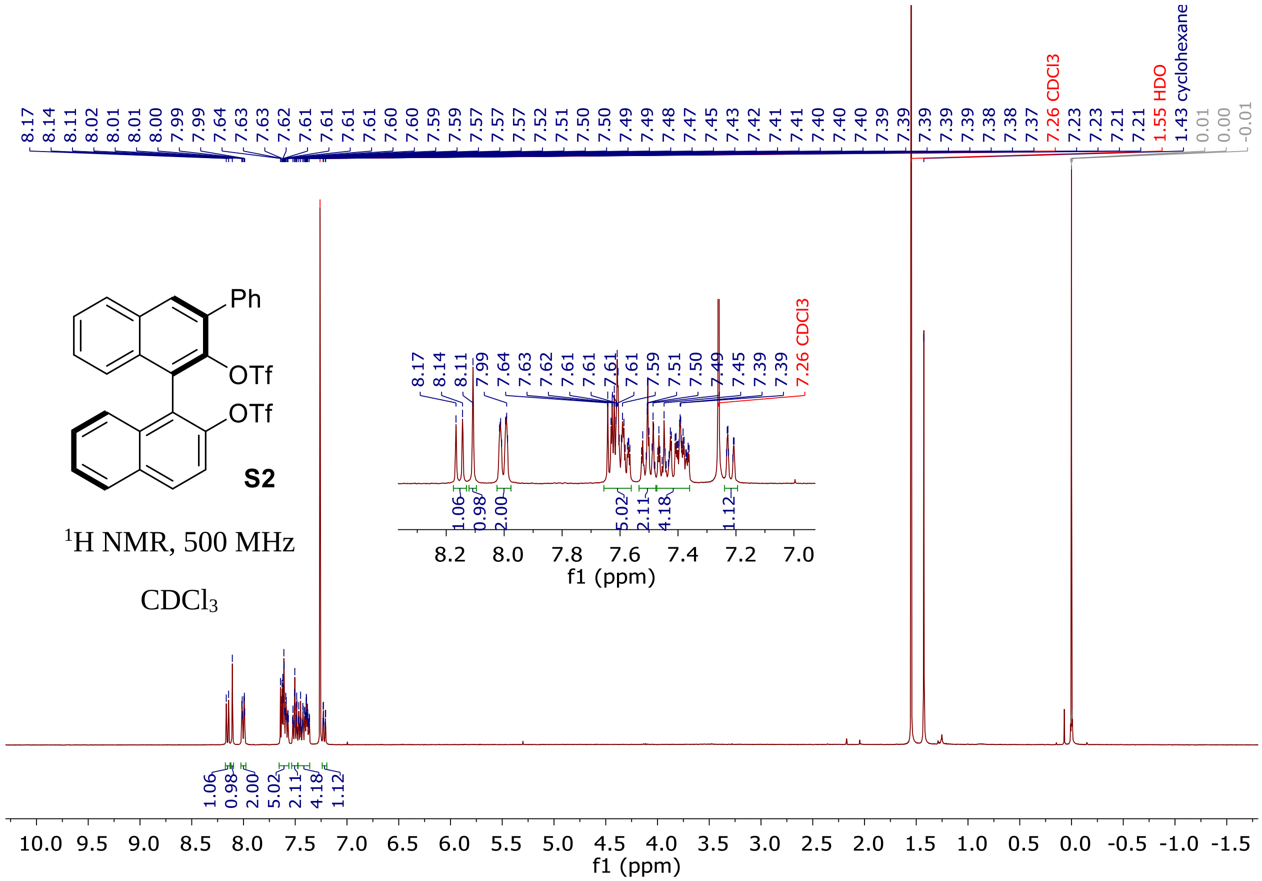
Ph
OTf
OTf
S2
^{1}H NMR, 500 MHz
CDCl3
7.26 CDCl3
1.55 HDO
1.43 cyclohexane
f1 (ppm)

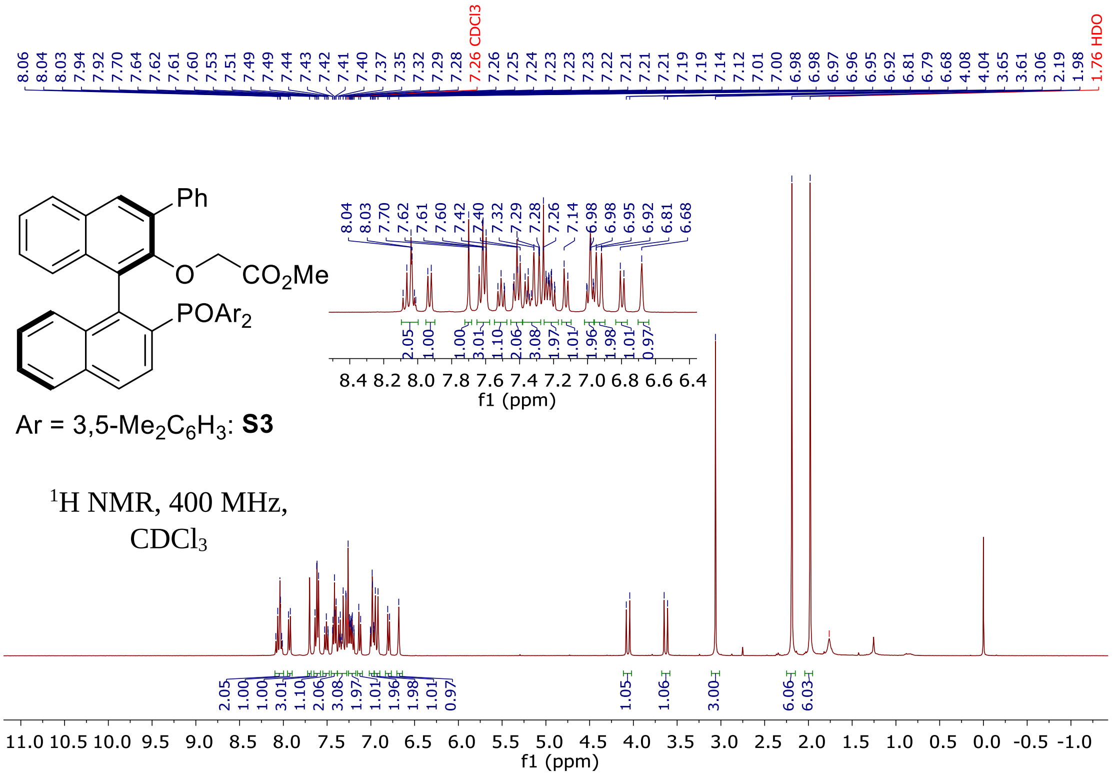
8.06 8.04 8.03 7.94 7.92 7.70 7.64 7.62 7.61 7.60 7.53 7.51 7.49 7.49 7.44 7.43 7.42 7.41 7.40 7.37 7.35 7.32 7.29 7.28 7.26 CDCl3 7.26 7.25 7.24 7.23 7.23 7.22 7.21 7.21 7.21 7.19 7.19 7.14 7.12 7.01 7.00 6.98 6.98 6.97 6.96 6.95 6.92 6.81 6.79 6.68 4.08 4.04 3.65 3.61 3.06 2.19 1.98 1.76 HDO
Ph
O
CO2Me
POAr2
Ar = 3,5-Me2C6H3: S3
1H NMR, 400 MHz, CDCl3
8.04 8.03 7.70 7.62 7.61 7.60 7.42 7.40 7.32 7.29 7.28 7.26 7.14 6.98 6.98 6.95 6.92 6.81 6.68
2.05 1.00 1.00 3.01 1.10 2.06 3.08 1.97 1.01 1.96 1.98 1.01 0.97
8.4 8.2 8.0 7.8 7.6 7.4 7.2 7.0 6.8 6.6 6.4
f1 (ppm)
2.05 1.00 1.00 3.01 1.10 2.06 3.08 1.97 1.01 1.96 1.98 1.01 0.97 1.05 1.06 3.00 6.06 6.03
11.0 10.5 10.0 9.5 9.0 8.5 8.0 7.5 7.0 6.5 6.0 5.5 5.0 4.5 4.0 3.5 3.0 2.5 2.0 1.5 1.0 0.5 0.0 -0.5 -1.0
f1 (ppm)

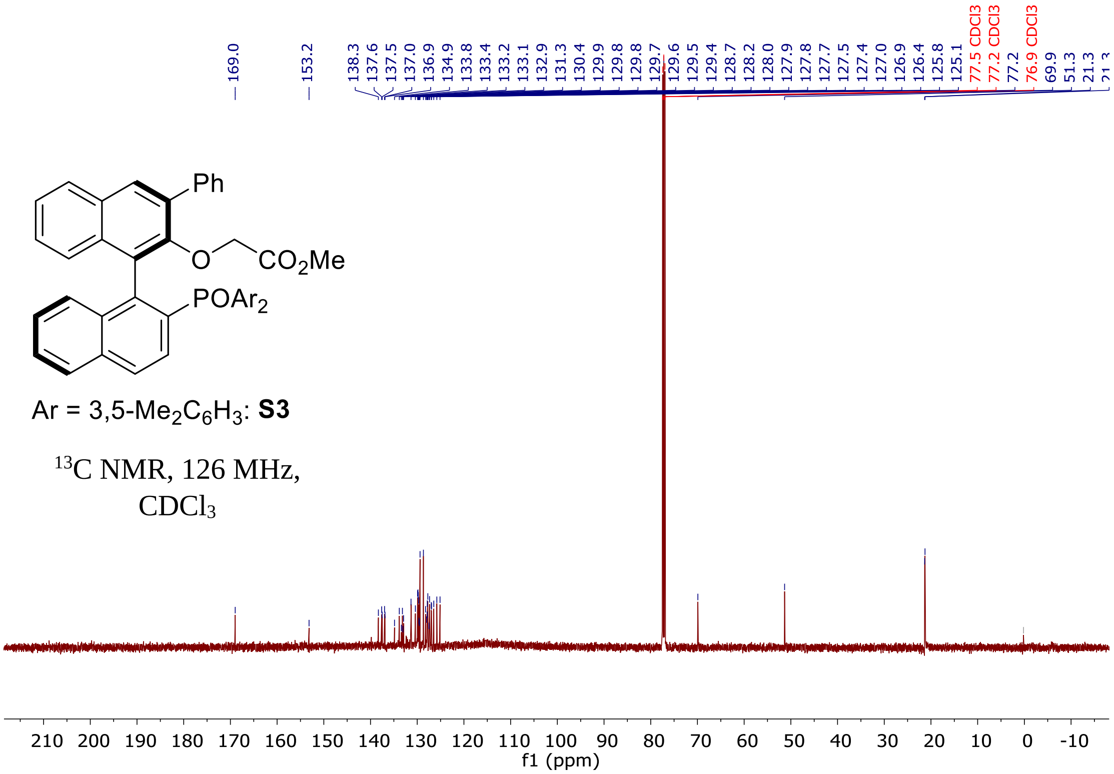
169.0 153.2 138.3 137.6 137.5 137.0 136.9 134.9 133.8 133.4 133.2 133.1 132.9 131.3 130.4 129.9 129.8 129.8 129.7 129.6 129.5 129.4 128.7 128.2 128.0 127.9 127.8 127.7 127.5 127.4 127.0 126.9 126.4 125.8 125.1 77.5 CDCl3 77.2 CDCl3 77.2 76.9 CDCl3 69.9 51.3 21.3
Ph
O
CO2Me
POAr2
Ar = 3,5-Me2C6H3: S3
13C NMR, 126 MHz, CDCl3
210 200 190 180 170 160 150 140 130 120 110 100 90 80 70 60 50 40 30 20 10 0 -10
f1 (ppm)

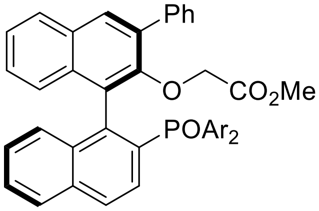

Ar = 3,5-$Me_2C_6H_3$: **S3**

^{31}P NMR, 162 MHz, $CDCl_3$

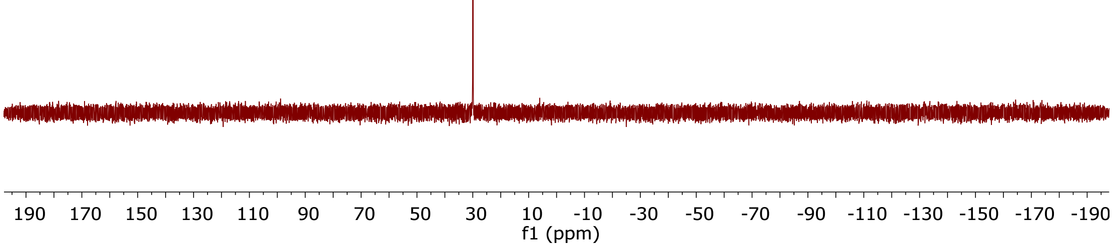

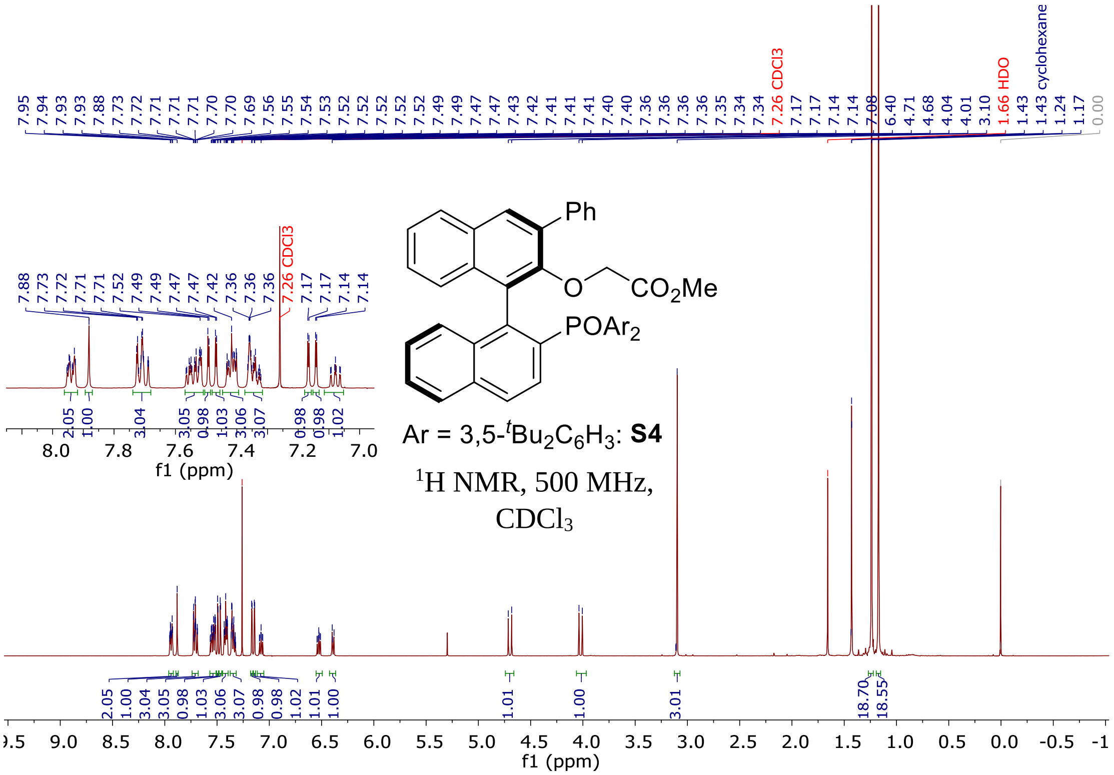
Ph
O
CO2Me
POAr2
Ar = 3,5-tBu2C6H3: S4
1H NMR, 500 MHz, CDCl3
f1 (ppm)

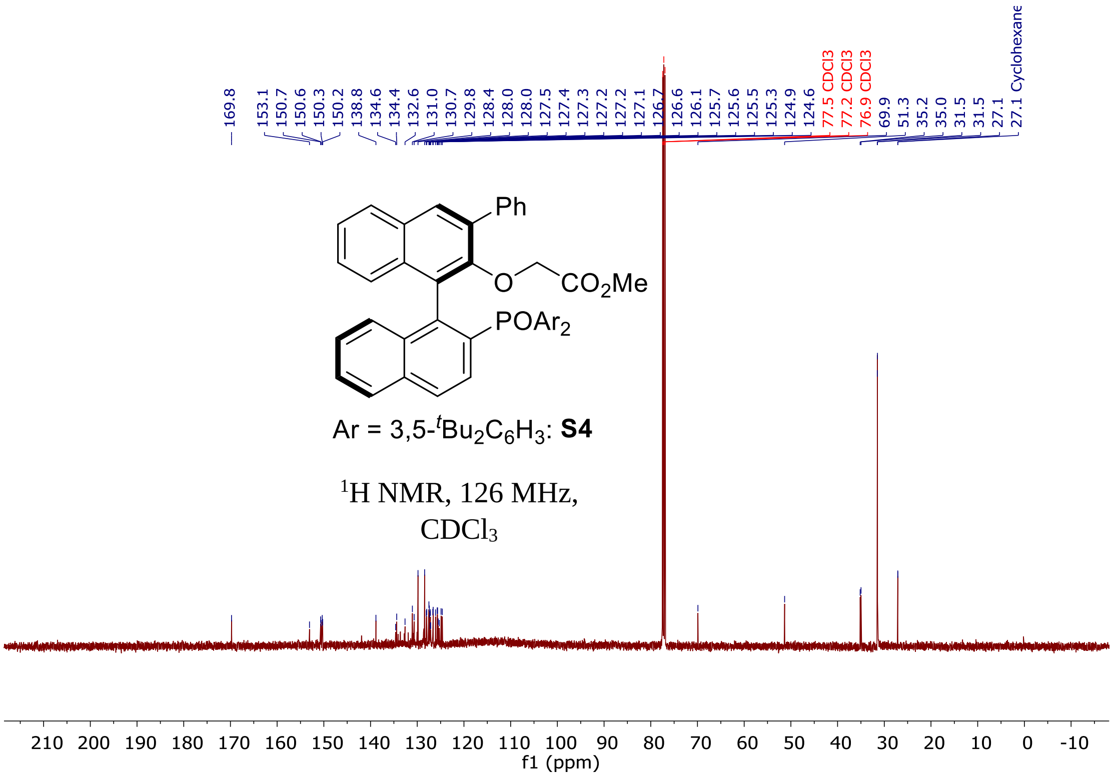
Ph
O
CO2Me
POAr2
Ar = 3,5-tBu2C6H3: S4
1H NMR, 126 MHz, CDCl3
f1 (ppm)

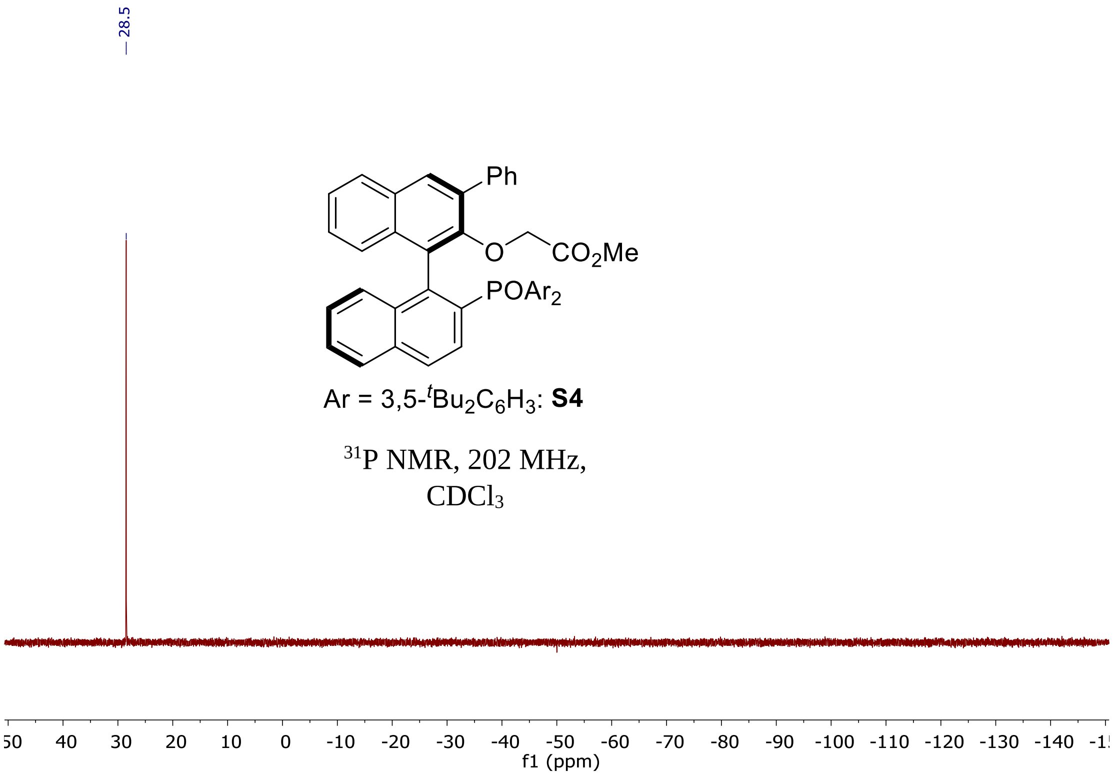
28.5
Ph
O
CO2Me
POAr2
Ar = 3,5-tBu2C6H3: S4
31P NMR, 202 MHz,
CDCl3
50 40 30 20 10 0 -10 -20 -30 -40 -50 -60 -70 -80 -90 -100 -110 -120 -130 -140
f1 (ppm)

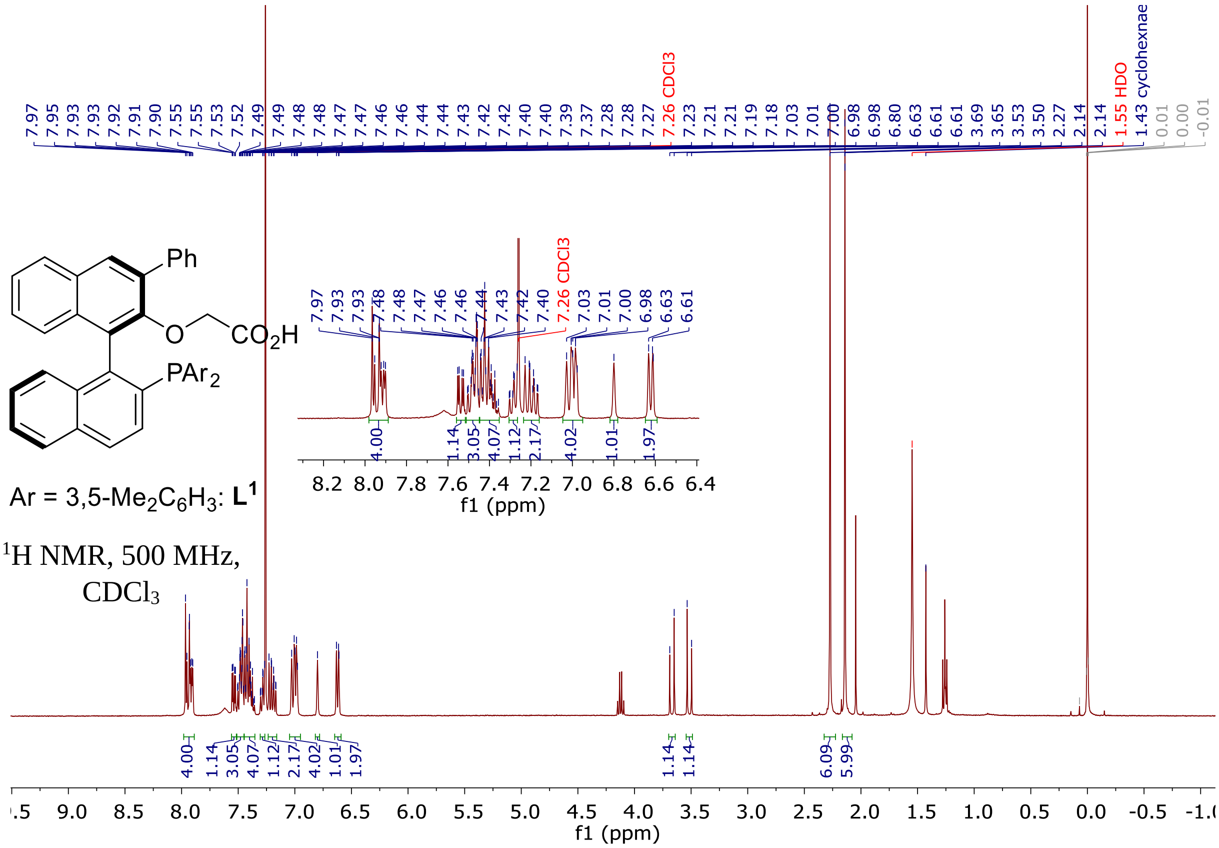
Ph
O
CO2H
PAr2
Ar = 3,5-Me2C6H3: L1
1H NMR, 500 MHz,
CDCl3
7.26 CDCl3
1.55 HDO
1.43 cyclohexnae
f1 (ppm)

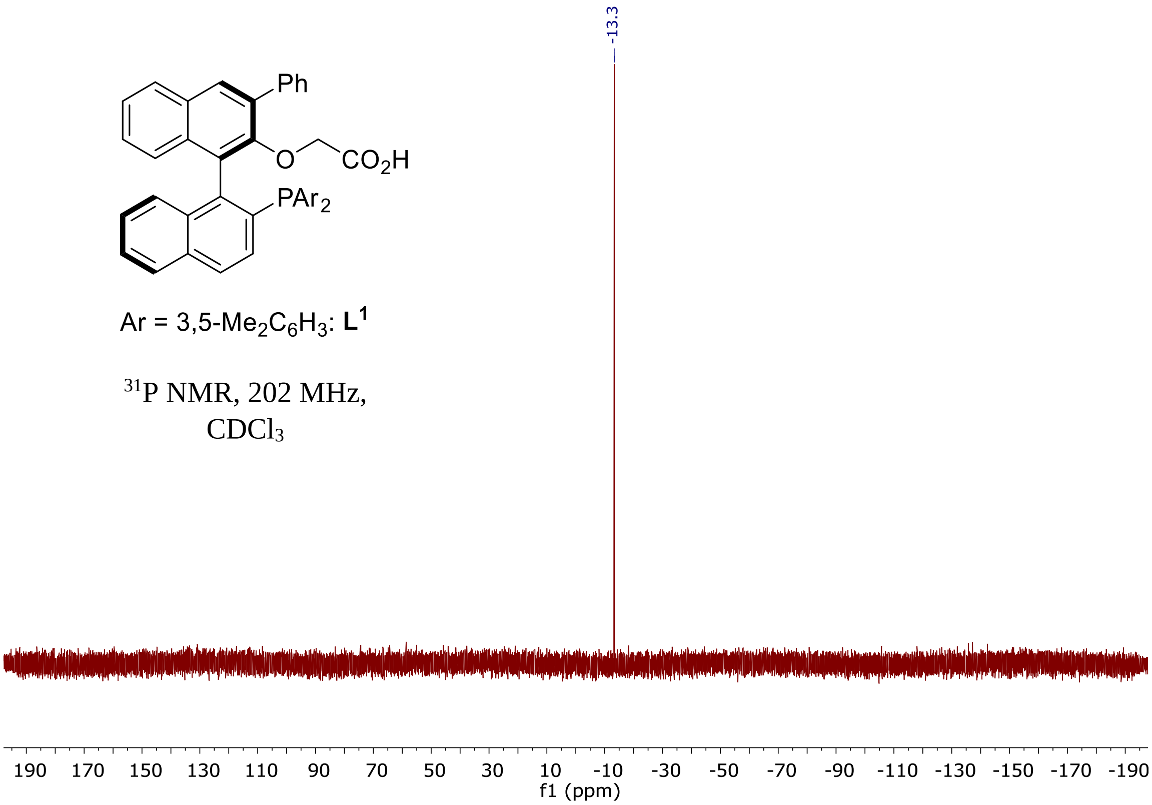
-13.3
Ph
O
CO2H
PAr2
Ar = 3,5-Me2C6H3: L1
31P NMR, 202 MHz,
CDCl3
f1 (ppm)

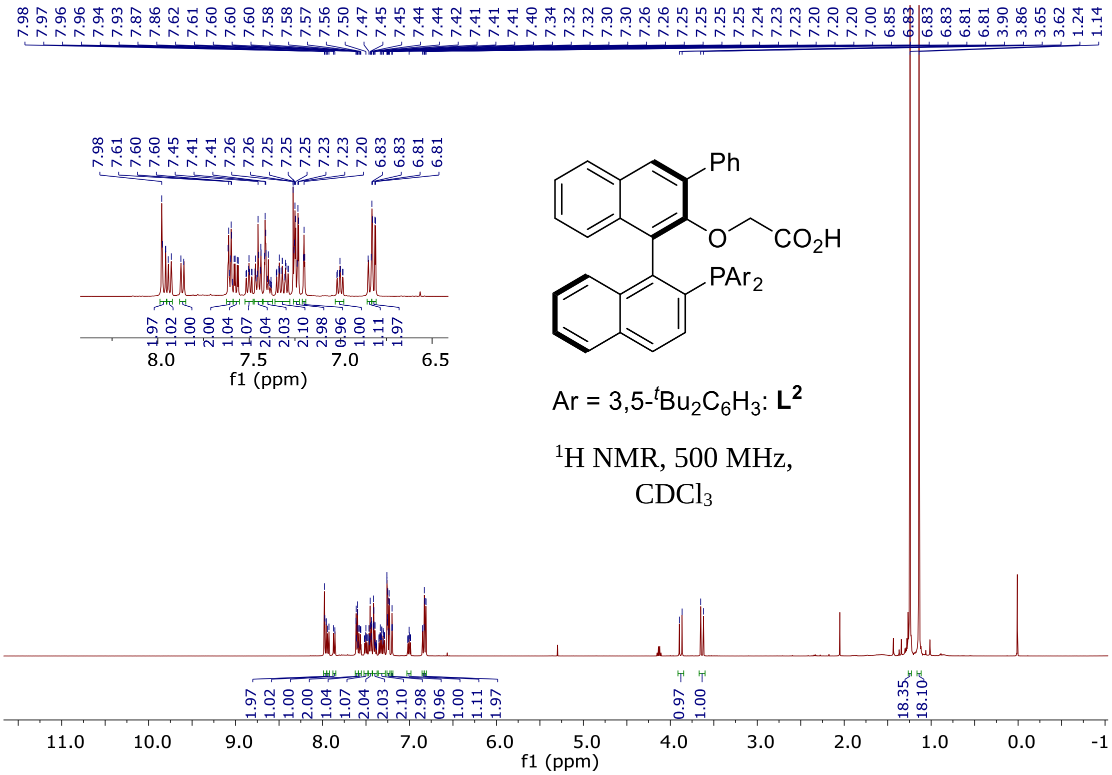
7.98 7.97 7.96 7.96 7.94 7.93 7.87 7.86 7.62 7.61 7.60 7.60 7.60 7.58 7.58 7.57 7.56 7.50 7.47 7.45 7.45 7.44 7.44 7.42 7.41 7.41 7.41 7.40 7.34 7.32 7.32 7.30 7.30 7.26 7.26 7.25 7.25 7.25 7.24 7.23 7.23 7.20 7.20 7.20 7.00 6.85 6.83 6.83 6.83 6.81 6.81 3.90 3.86 3.65 3.62 1.24 1.14
7.98 7.61 7.60 7.60 7.45 7.41 7.41 7.26 7.26 7.25 7.25 7.25 7.23 7.23 7.20 6.83 6.83 6.81 6.81
1.97 1.02 1.00 2.00 1.04 1.07 2.04 2.03 2.10 2.98 0.96 1.00 1.11 1.97
8.0 7.5 7.0 6.5
f1 (ppm)
Ph
O
CO2H
PAr2
Ar = 3,5-tBu2C6H3: L2
1H NMR, 500 MHz, CDCl3
1.97 1.02 1.00 2.00 1.04 1.07 2.04 2.03 2.10 2.98 0.96 1.00 1.11 1.97
0.97 1.00
18.35 18.10
11.0 10.0 9.0 8.0 7.0 6.0 5.0 4.0 3.0 2.0 1.0 0.0 -1
f1 (ppm)

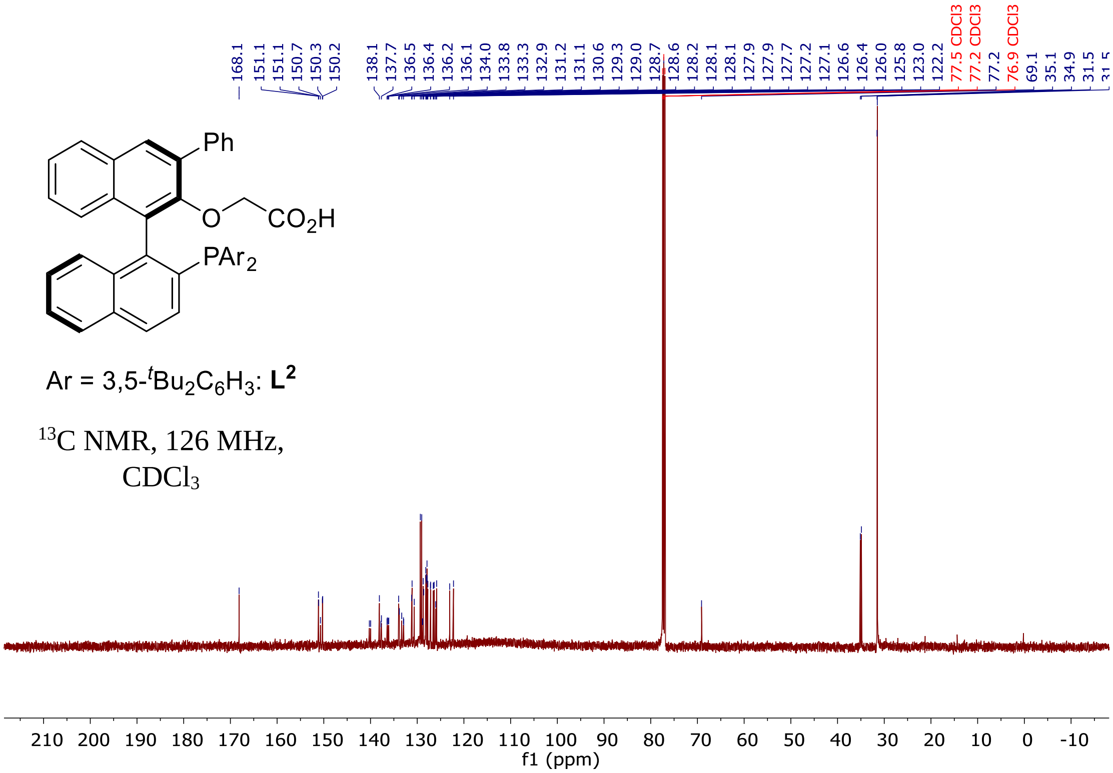
168.1 151.1 151.1 150.7 150.3 150.2 138.1 137.7 136.5 136.4 136.2 136.1 134.0 133.8 133.3 132.9 131.2 131.1 130.6 129.3 129.0 128.7 128.6 128.2 128.1 128.1 127.9 127.9 127.7 127.2 127.1 126.6 126.4 126.0 125.8 123.0 122.2 77.5 CDCl3 77.2 CDCl3 77.2 76.9 CDCl3 69.1 35.1 34.9 31.5
Ph
O
CO2H
PAr2
Ar = 3,5-tBu2C6H3: L2
13C NMR, 126 MHz, CDCl3
210 200 190 180 170 160 150 140 130 120 110 100 90 80 70 60 50 40 30 20 10 0 -10
f1 (ppm)

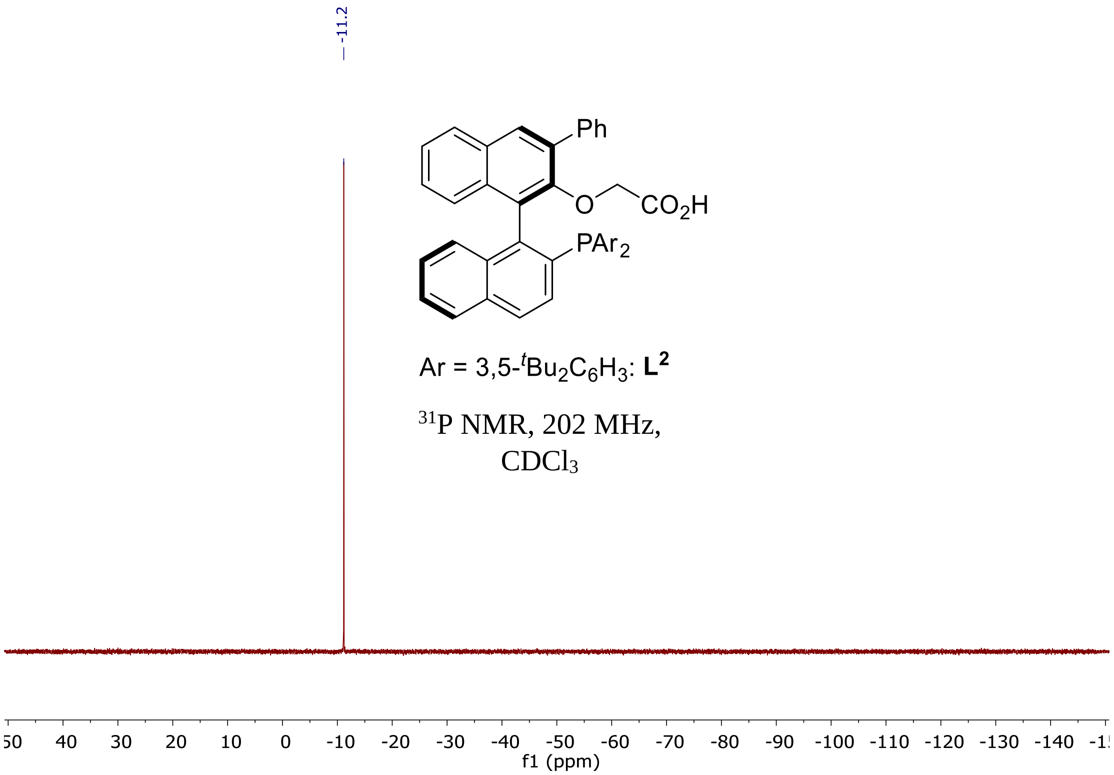
-11.2
Ph
O
CO_2H
PAr_2
Ar = 3,5-$^{t}Bu_2C_6H_3$: **L[2]**
^{31}P NMR, 202 MHz,
$CDCl_3$
50 40 30 20 10 0 -10 -20 -30 -40 -50 -60 -70 -80 -90 -100 -110 -120 -130 -140 -1
f1 (ppm)

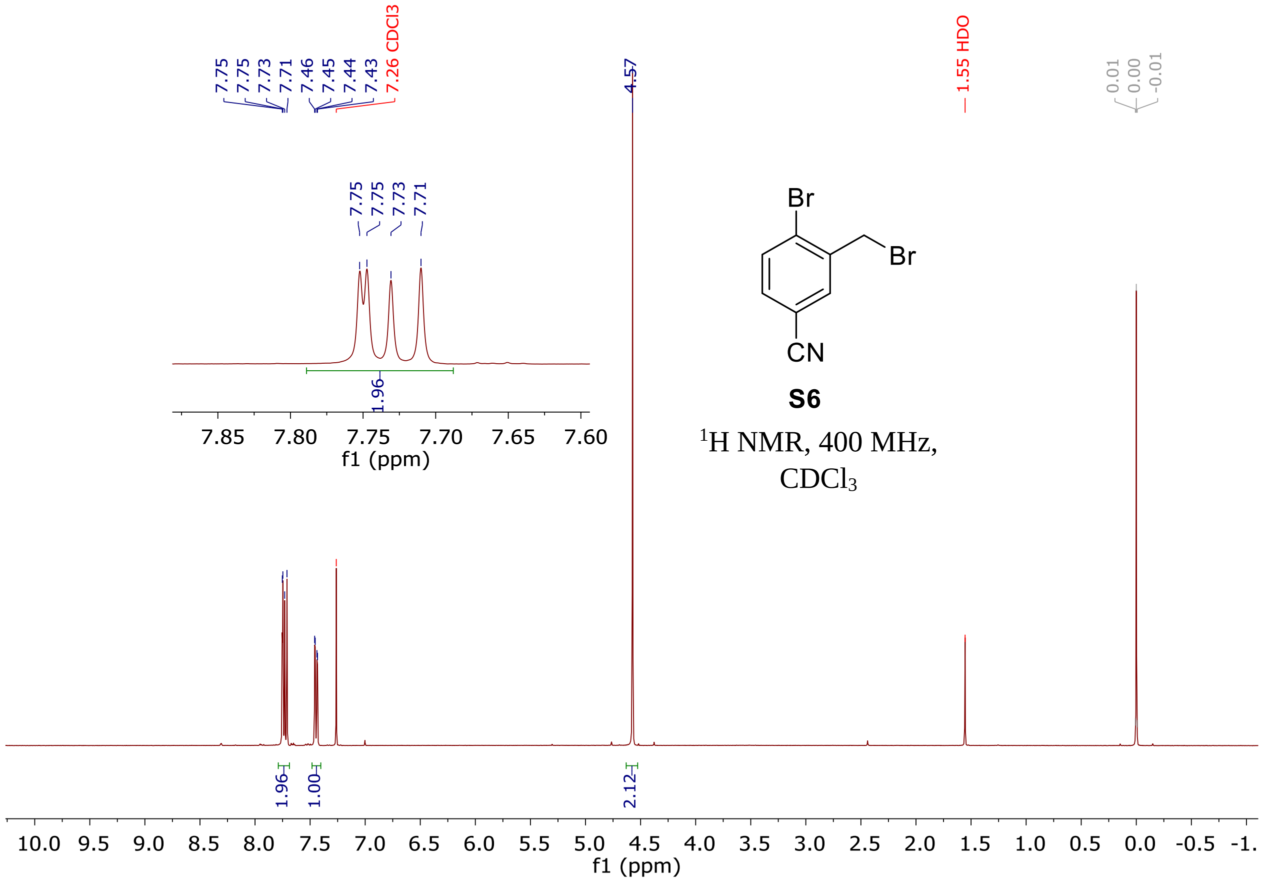

7.75
7.75
7.73
7.71
7.46
7.45
7.44
7.43
7.26 CDCl3
4.57
1.55 HDO
0.01
0.00
-0.01
Br
Br
CN
S6
^{1}H NMR, 400 MHz,
$CDCl_3$
1.96
1.00
2.12
7.85 7.80 7.75 7.70 7.65 7.60
f1 (ppm)
10.0 9.5 9.0 8.5 8.0 7.5 7.0 6.5 6.0 5.5 5.0 4.5 4.0 3.5 3.0 2.5 2.0 1.5 1.0 0.5 0.0 -0.5 -1.
f1 (ppm)

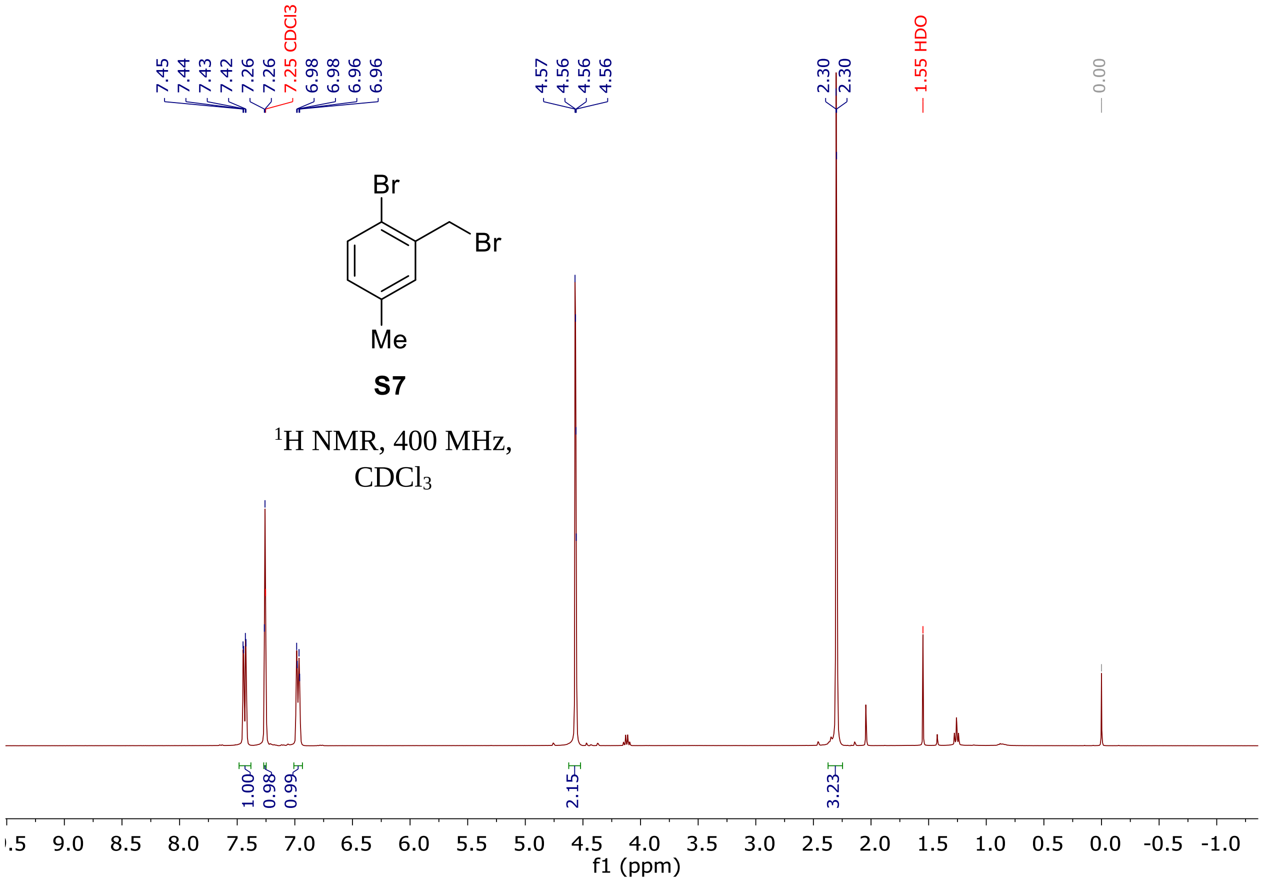
7.45
7.44
7.43
7.42
7.26
7.26
7.25 CDCl3
6.98
6.98
6.96
6.96
4.57
4.56
4.56
4.56
2.30
2.30
1.55 HDO
0.00
Br
Br
Me
S7
^{1}H NMR, 400 MHz,
$CDCl_3$
1.00
0.98
0.99
2.15
3.23
.5
9.0
8.5
8.0
7.5
7.0
6.5
6.0
5.5
5.0
4.5
4.0
3.5
3.0
2.5
2.0
1.5
1.0
0.5
0.0
-0.5
-1.0
f1 (ppm)

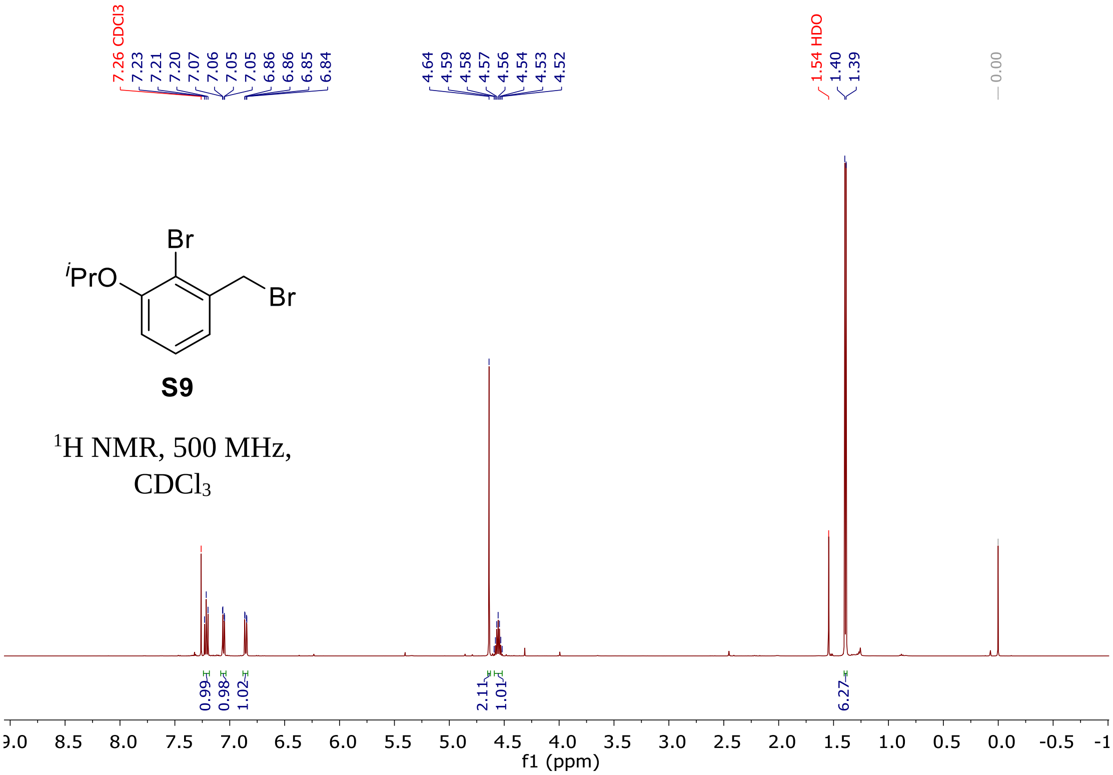

S9

^{1}H NMR, 500 MHz, $CDCl_3$

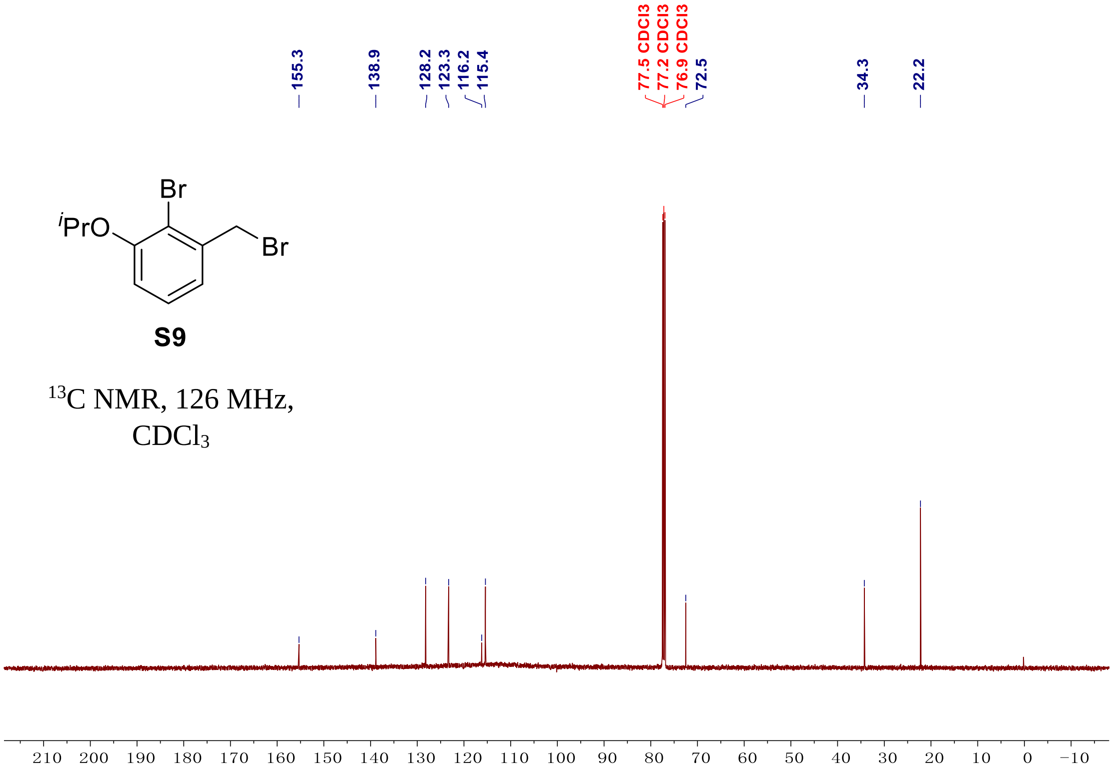

S9

^{13}C NMR, 126 MHz, $CDCl_3$

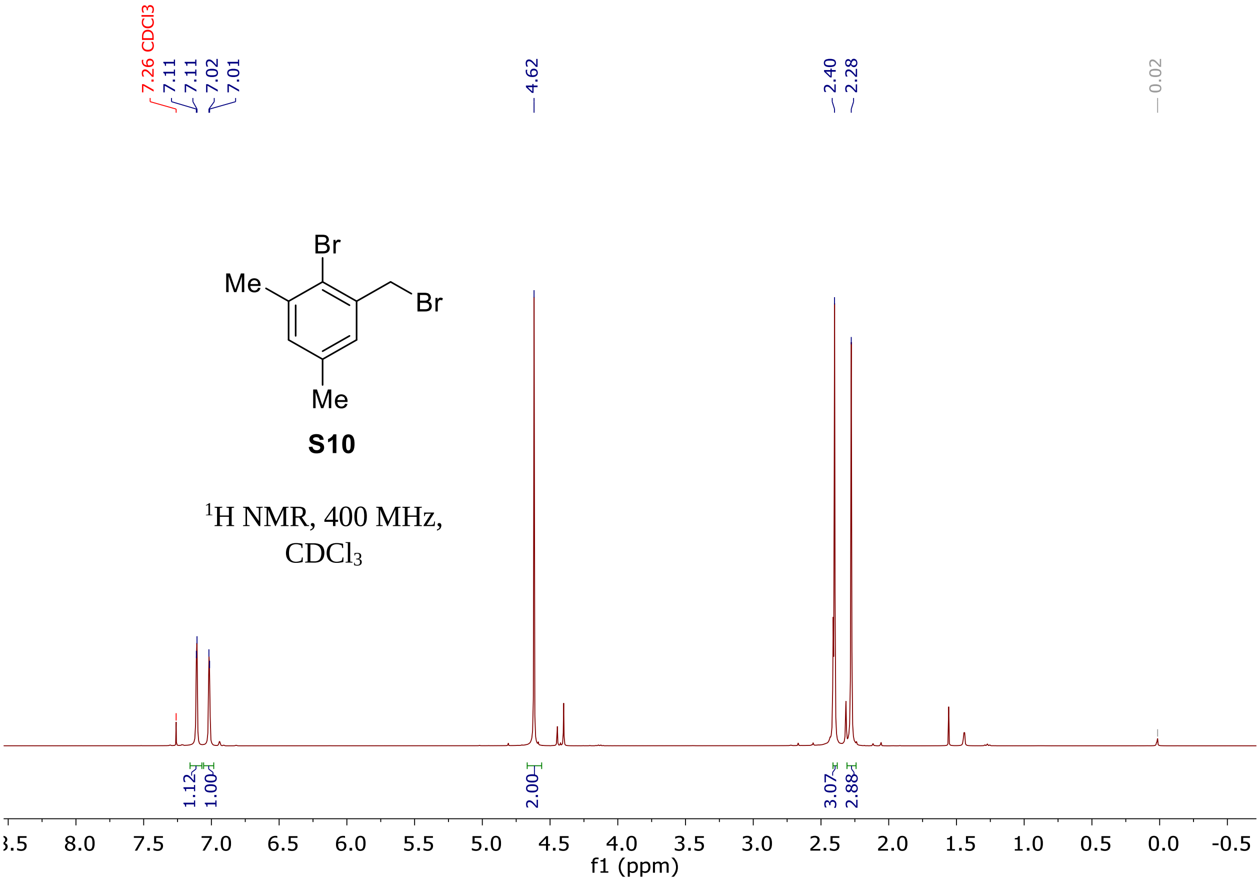
7.26 CDCl3
7.11
7.11
7.02
7.01
4.62
2.40
2.28
0.02
Br
Me
Br
Me
S10
^{1}H NMR, 400 MHz, $CDCl_3$
1.12
1.00
2.00
3.07
2.88
8.5 8.0 7.5 7.0 6.5 6.0 5.5 5.0 4.5 4.0 3.5 3.0 2.5 2.0 1.5 1.0 0.5 0.0 -0.5
f1 (ppm)

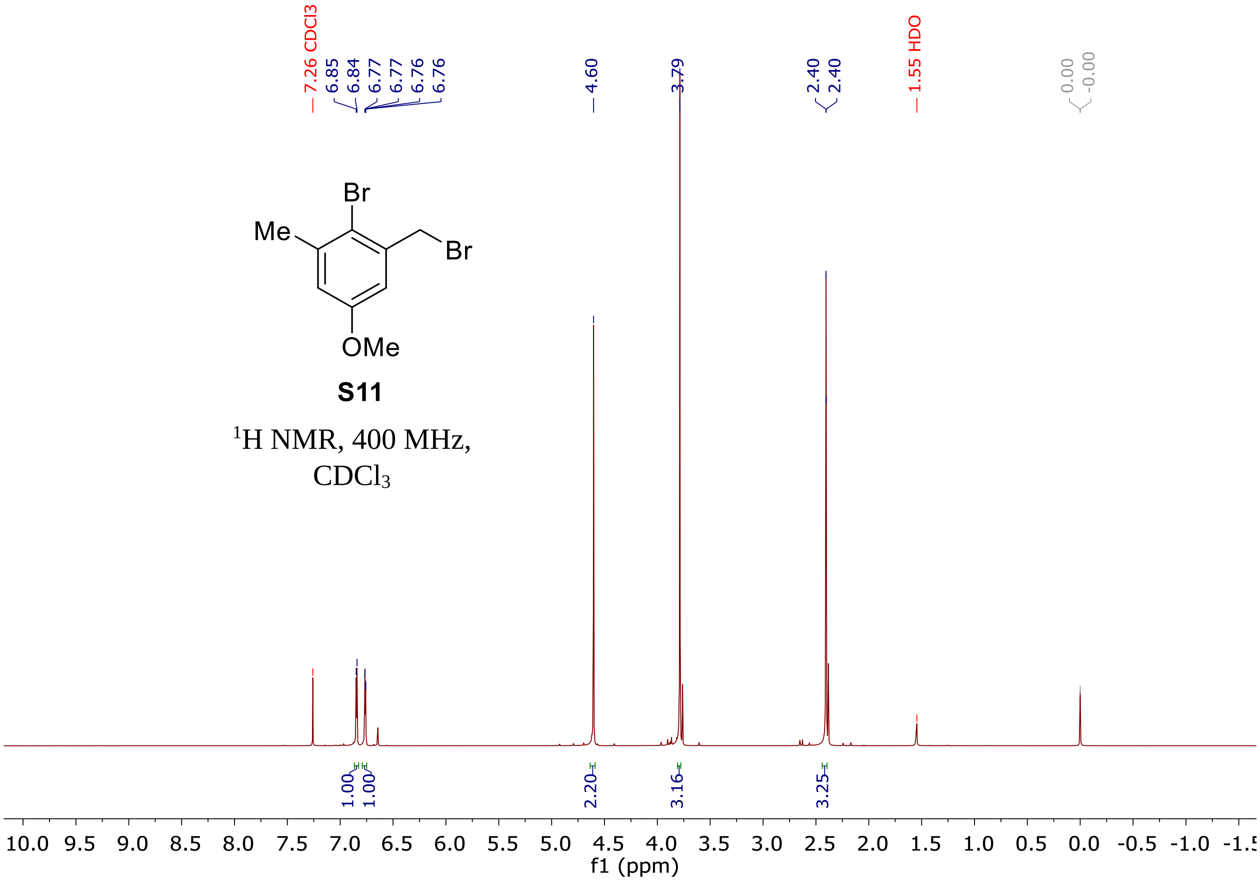

S11

^{1}H NMR, 400 MHz, $CDCl_3$

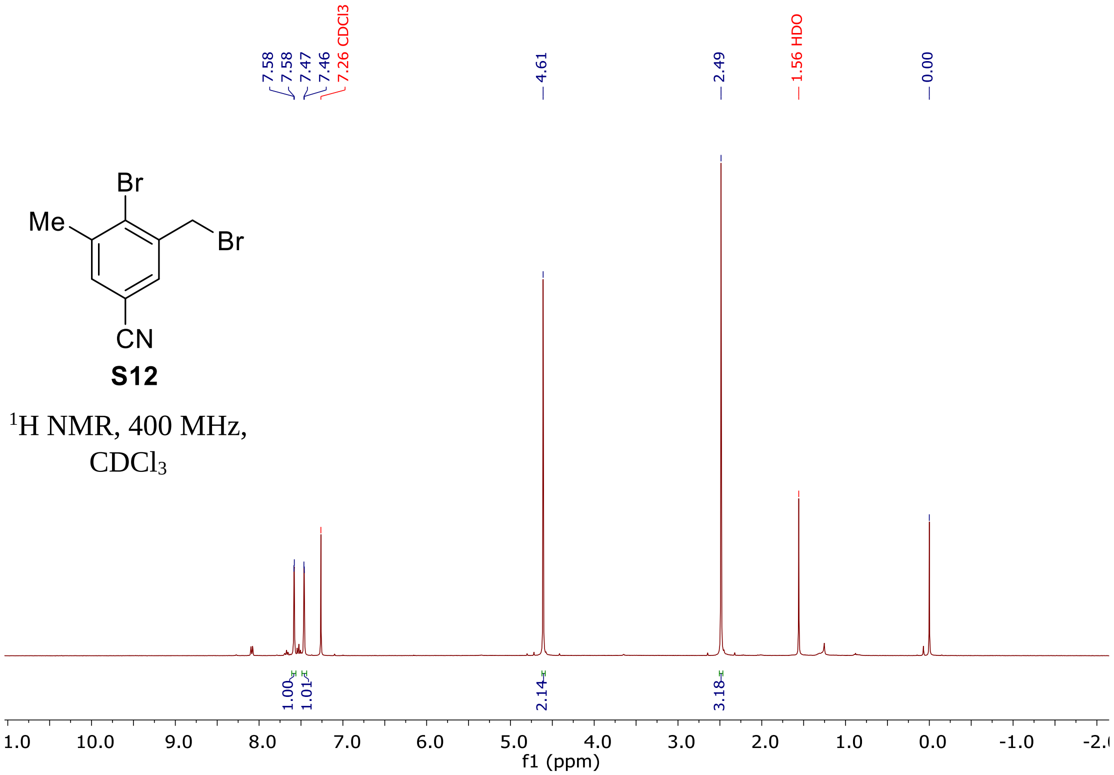
7.58
7.58
7.47
7.46
7.26 CDCl3
4.61
2.49
1.56 HDO
0.00
Br
Me
Br
CN
S12
^{1}H NMR, 400 MHz, $CDCl_3$
1.00
1.01
2.14
3.18
f1 (ppm)

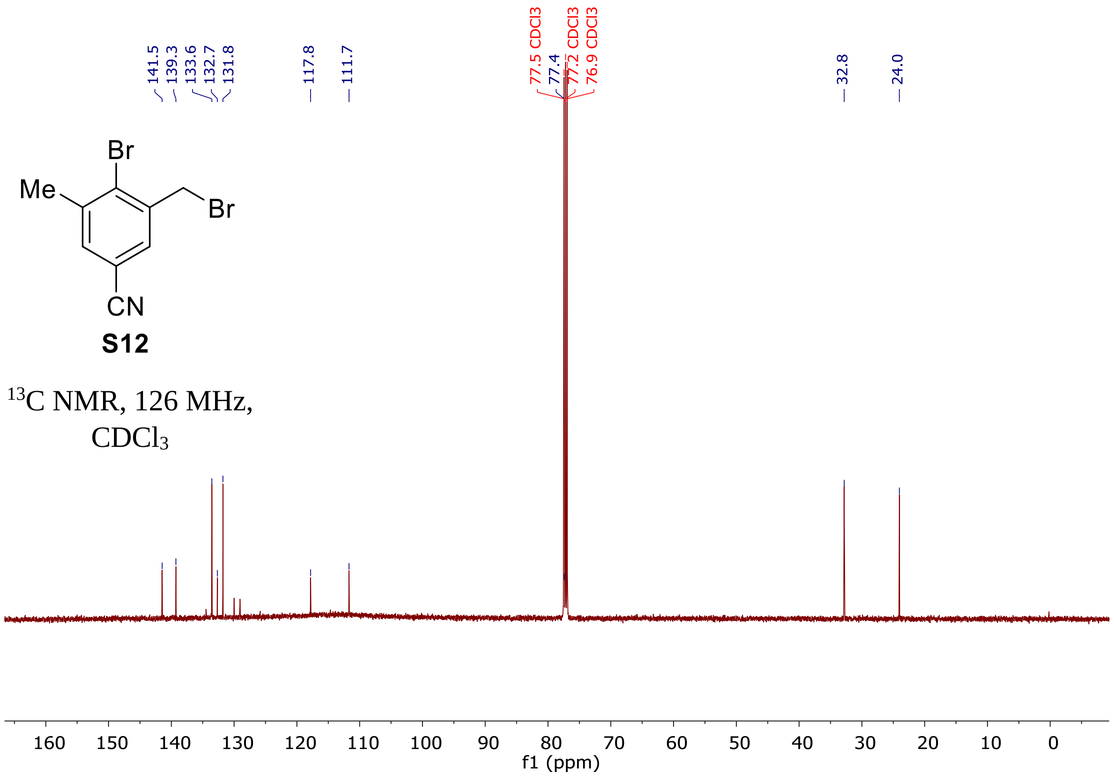
141.5
139.3
133.6
132.7
131.8
117.8
111.7
77.5 CDCl3
77.4
77.2 CDCl3
76.9 CDCl3
32.8
24.0
Br
Me
Br
CN
S12
^{13}C NMR, 126 MHz, $CDCl_3$
f1 (ppm)

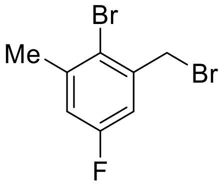

S13

^{1}H NMR, 400 MHz, $CDCl_3$

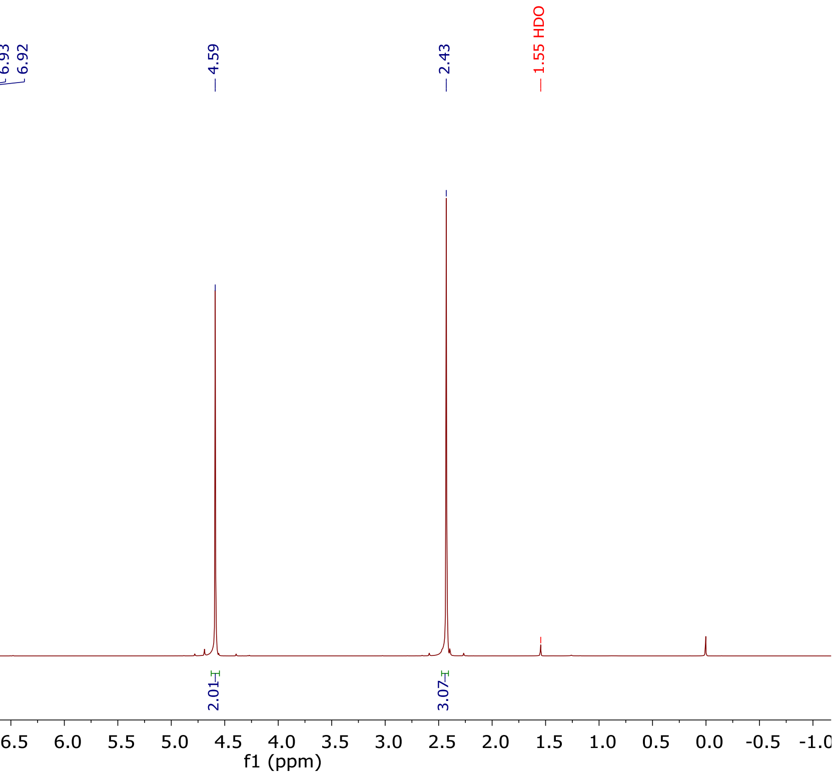

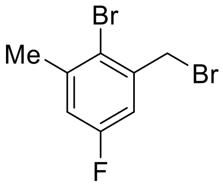

S13

^{13}C NMR, 126 MHz, $CDCl_3$

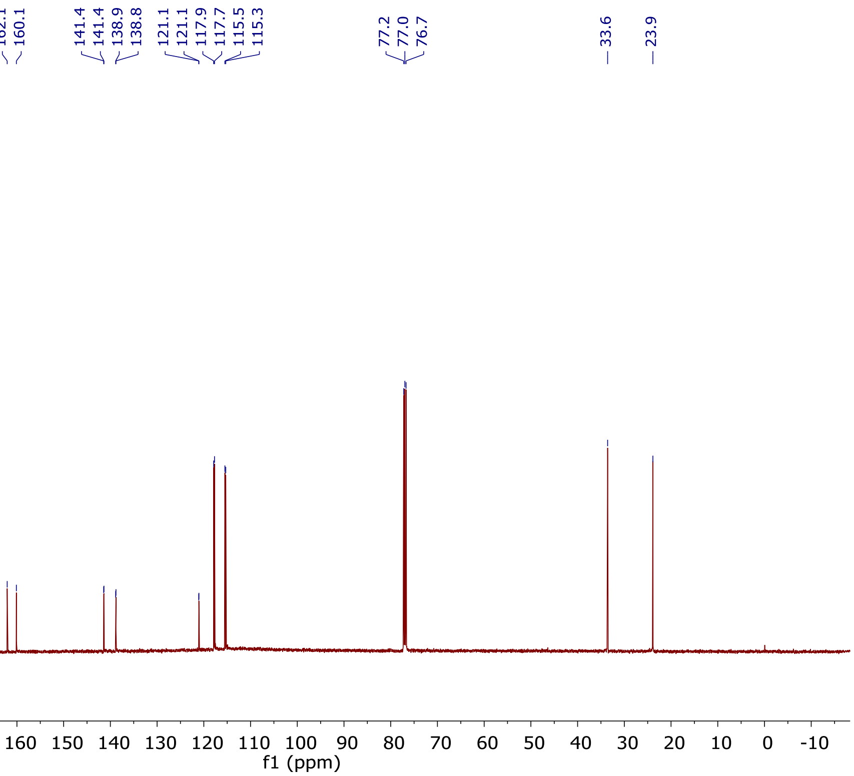

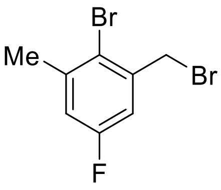

S13

^{19}F NMR, 376 MHz, $CDCl_3$

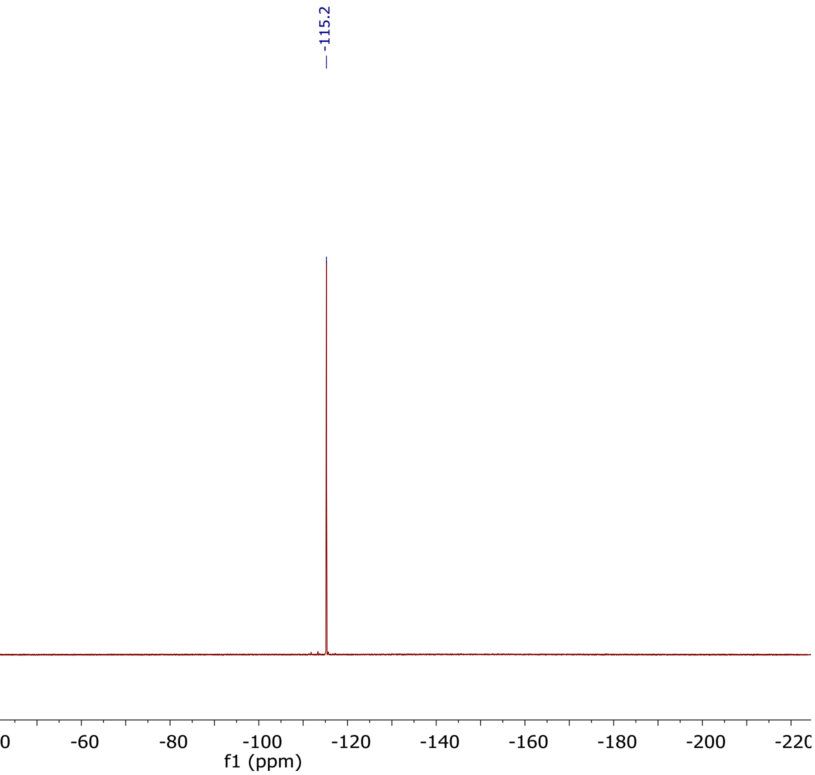

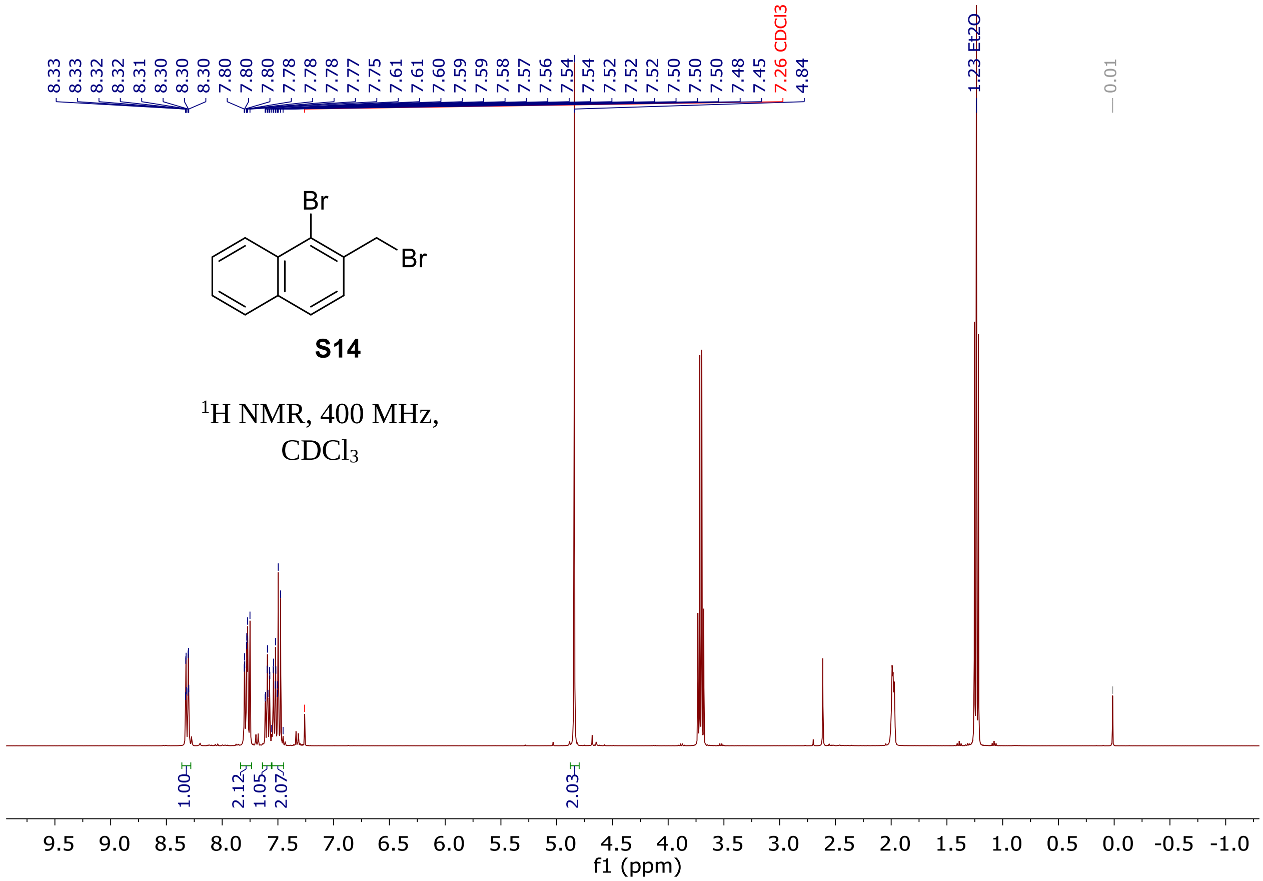
8.33
8.33
8.32
8.32
8.31
8.30
8.30
8.30
7.80
7.80
7.80
7.78
7.78
7.78
7.77
7.75
7.61
7.61
7.60
7.59
7.59
7.58
7.57
7.56
7.54
7.54
7.52
7.52
7.52
7.50
7.50
7.50
7.48
7.45
7.26 CDCl3
4.84
1.23 Et2O
0.01
Br
Br
S14
¹H NMR, 400 MHz,
CDCl3
1.00
2.12
1.05
2.07
2.03
9.5
9.0
8.5
8.0
7.5
7.0
6.5
6.0
5.5
5.0
4.5
4.0
3.5
3.0
2.5
2.0
1.5
1.0
0.5
0.0
-0.5
-1.0
f1 (ppm)

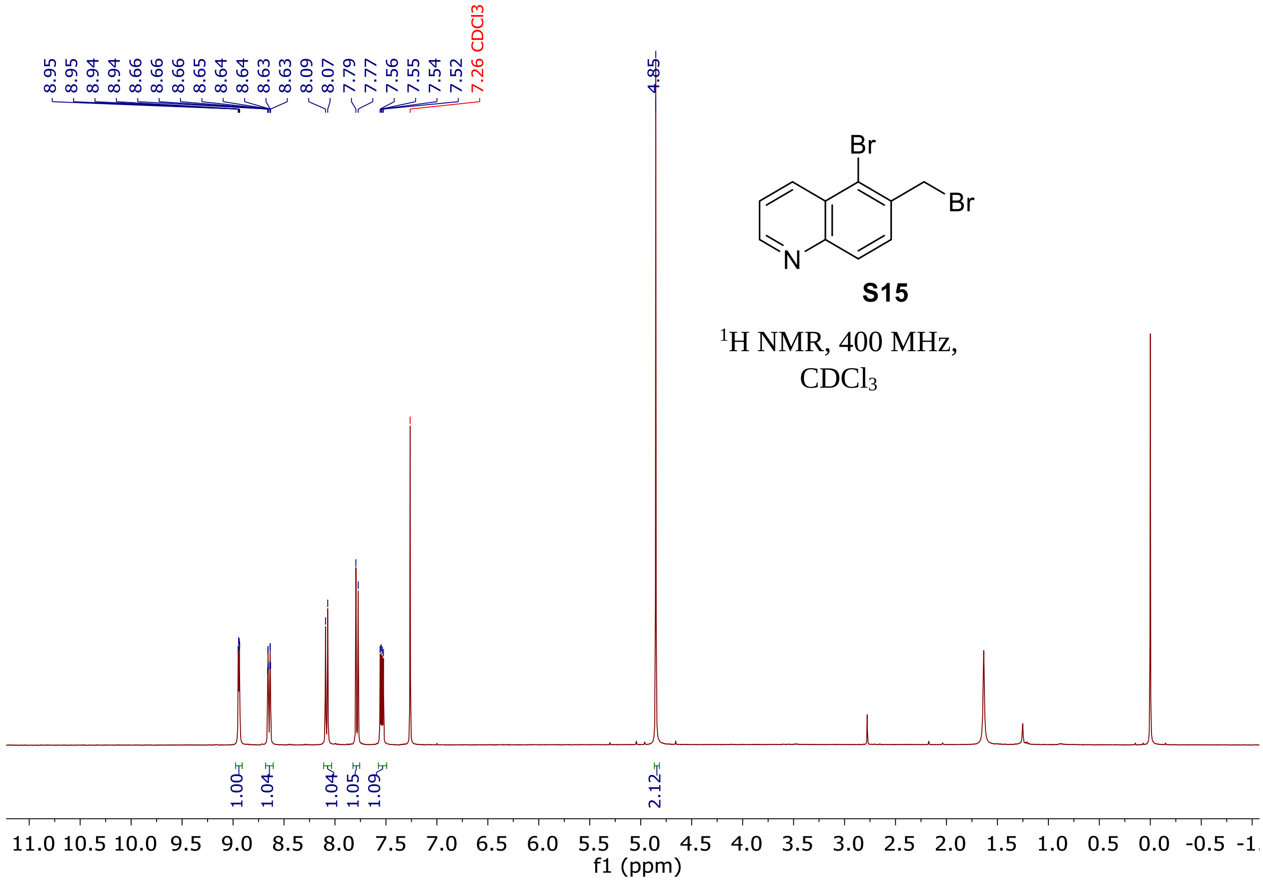

8.95
8.95
8.94
8.94
8.66
8.66
8.66
8.65
8.64
8.64
8.63
8.63
8.09
8.07
7.79
7.77
7.56
7.55
7.54
7.52
7.26 CDCl3
4.85
Br
Br
N
S15
1H NMR, 400 MHz, CDCl3
1.00
1.04
1.04
1.05
1.09
2.12
f1 (ppm)

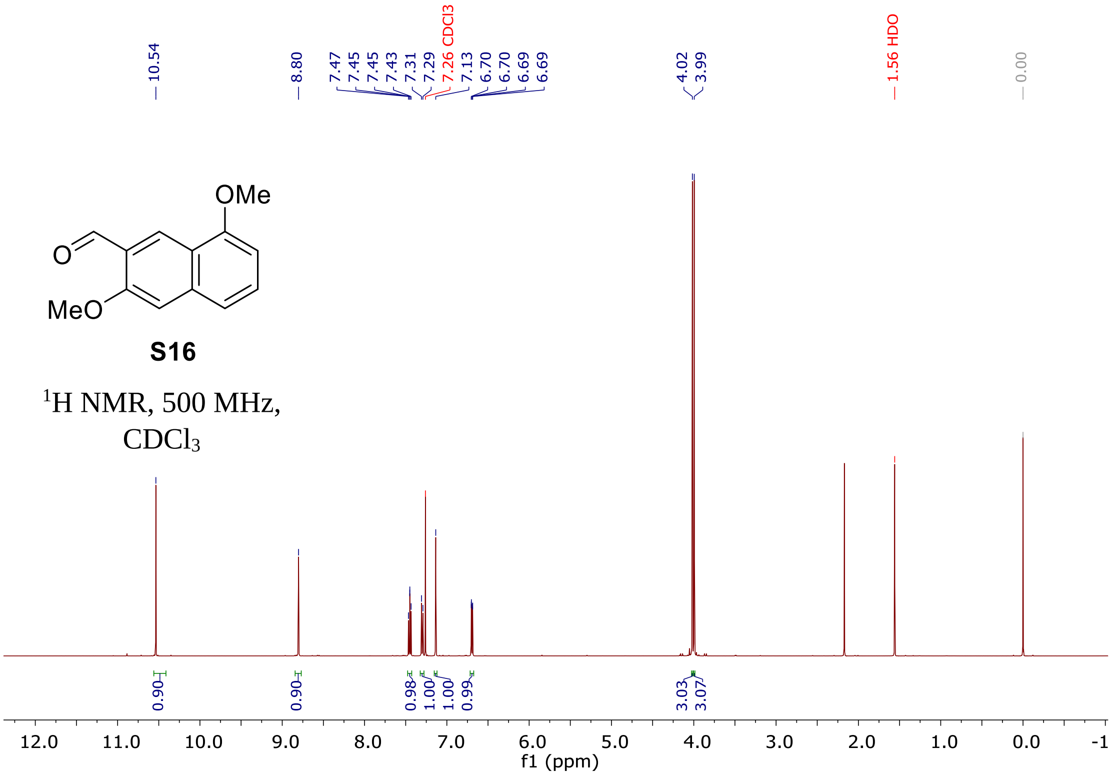
10.54
8.80
7.47
7.45
7.45
7.43
7.31
7.29
7.26 CDCl3
7.13
6.70
6.70
6.69
6.69
4.02
3.99
1.56 HDO
0.00
OMe
O
MeO
S16
1H NMR, 500 MHz,
CDCl3
0.90
0.90
0.98
1.00
1.00
0.99
3.03
3.07
12.0
11.0
10.0
9.0
8.0
7.0
6.0
5.0
4.0
3.0
2.0
1.0
0.0
-1
f1 (ppm)

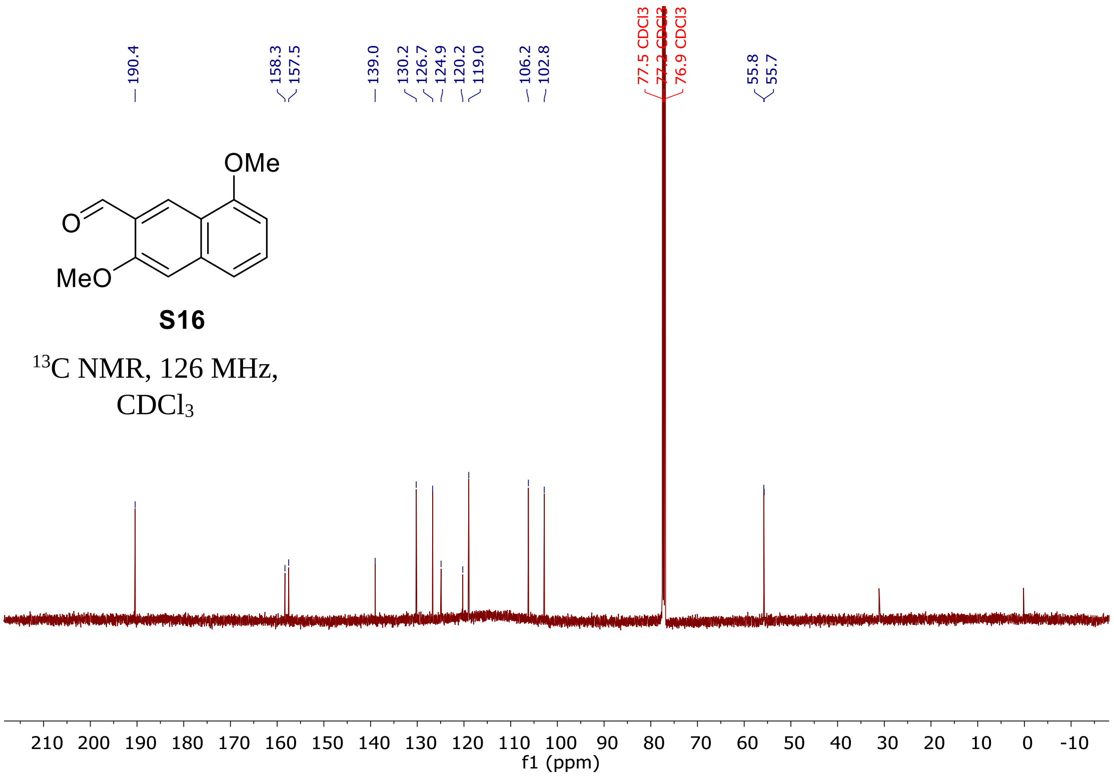
190.4
158.3
157.5
139.0
130.2
126.7
124.9
120.2
119.0
106.2
102.8
77.5 CDCl3
77.3 CDCl3
76.9 CDCl3
55.8
55.7
OMe
O
MeO
S16
13C NMR, 126 MHz,
CDCl3
210
200
190
180
170
160
150
140
130
120
110
100
90
80
70
60
50
40
30
20
10
0
-10
f1 (ppm)

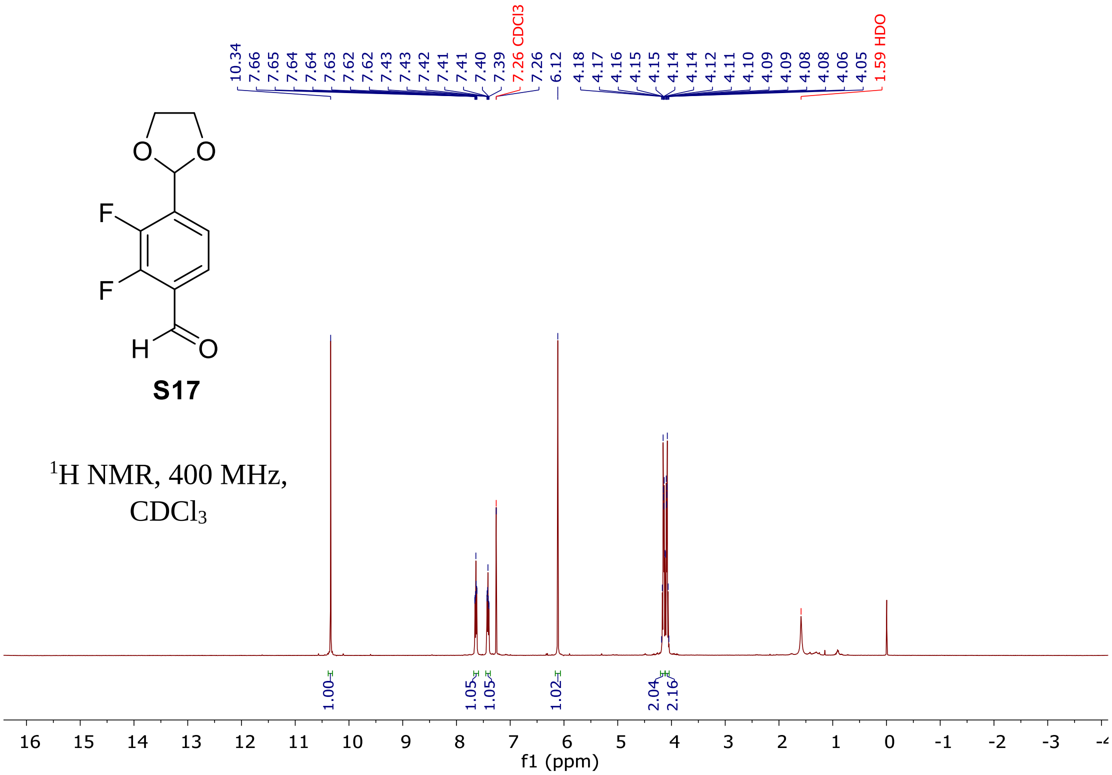
S17
1H NMR, 400 MHz, CDCl3
f1 (ppm)

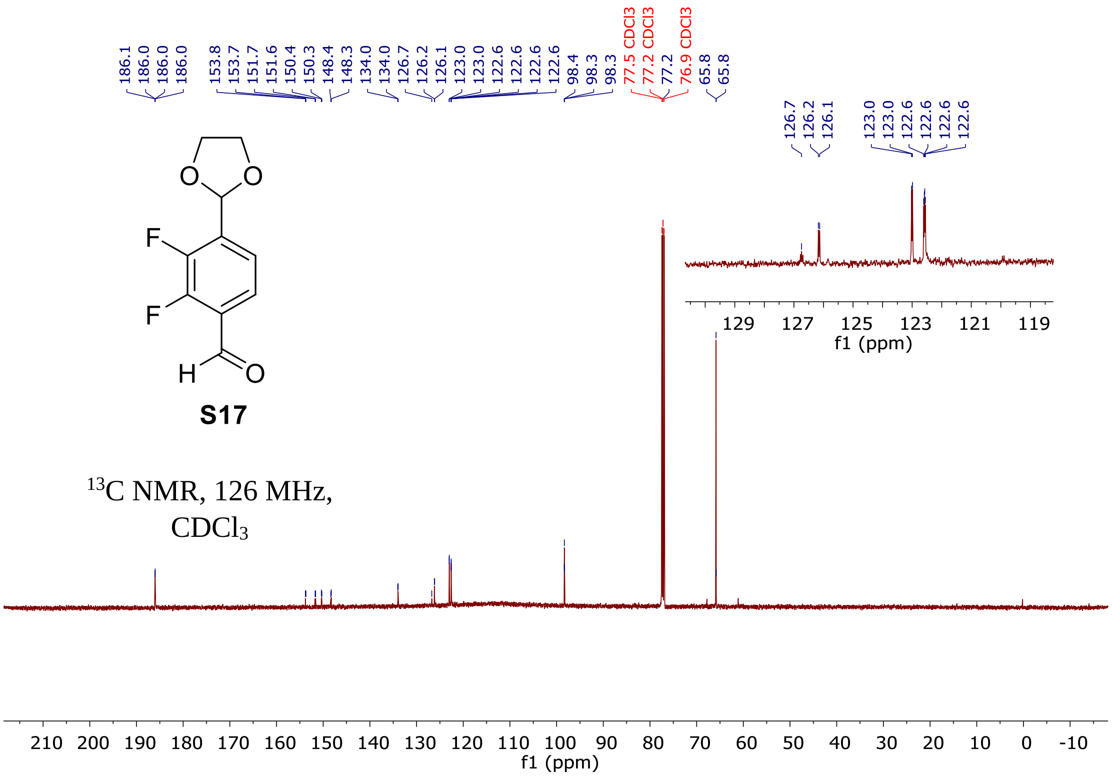
S17
13C NMR, 126 MHz, CDCl3
f1 (ppm)

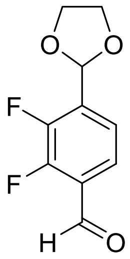

S17

^{19}F NMR, 376 MHz, $CDCl_3$

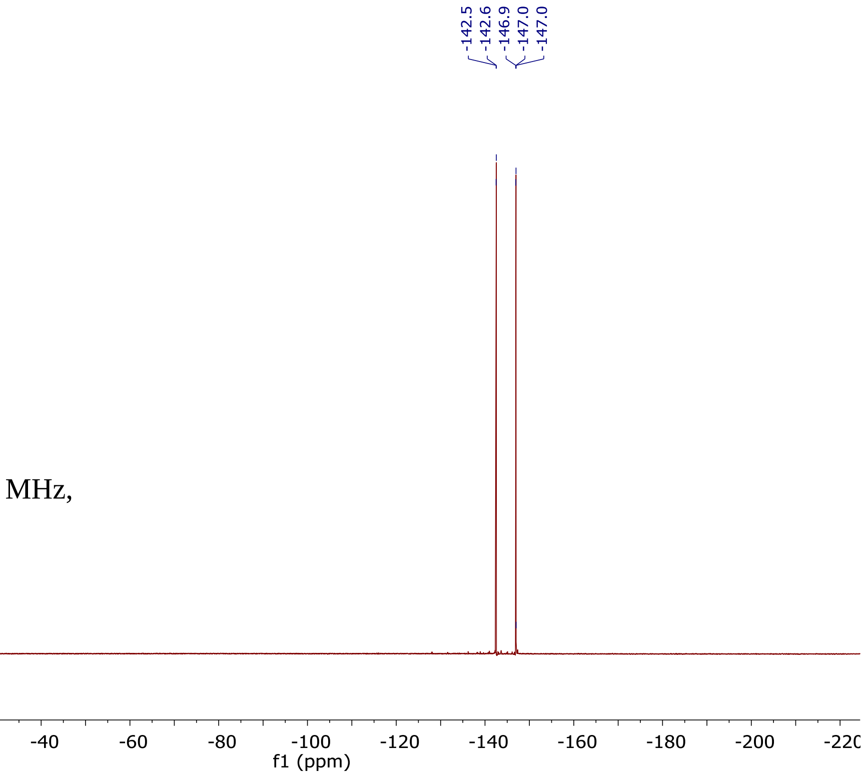

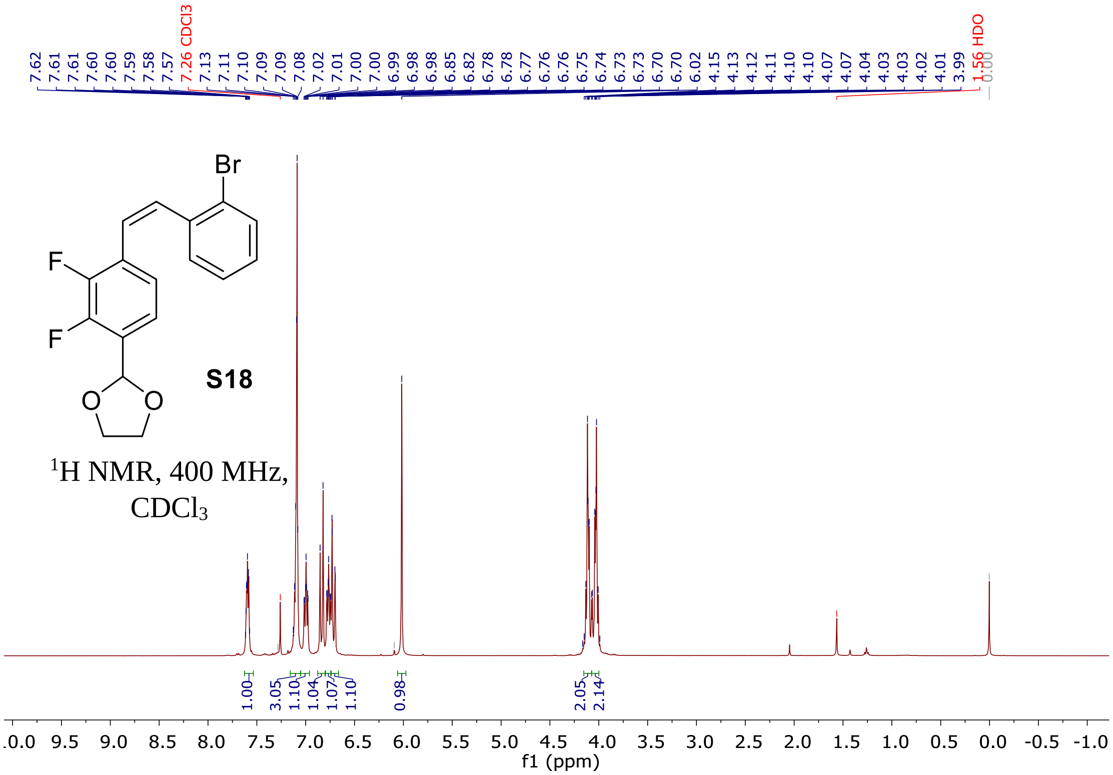
7.62 7.61 7.61 7.60 7.60 7.59 7.58 7.57 7.26 CDCl3 7.13 7.11 7.10 7.09 7.08 7.02 7.01 7.00 6.99 6.98 6.98 6.85 6.82 6.78 6.78 6.77 6.76 6.76 6.75 6.74 6.73 6.73 6.70 6.70 6.02 4.15 4.13 4.12 4.11 4.10 4.10 4.07 4.04 4.03 4.03 4.02 4.01 3.99 1.56 HDO 0.00
Br
F
F
O
O
S18
^{1}H NMR, 400 MHz, $CDCl_3$
1.00 3.05 1.10 1.04 1.07 1.10 0.98 2.05 2.14
f1 (ppm)

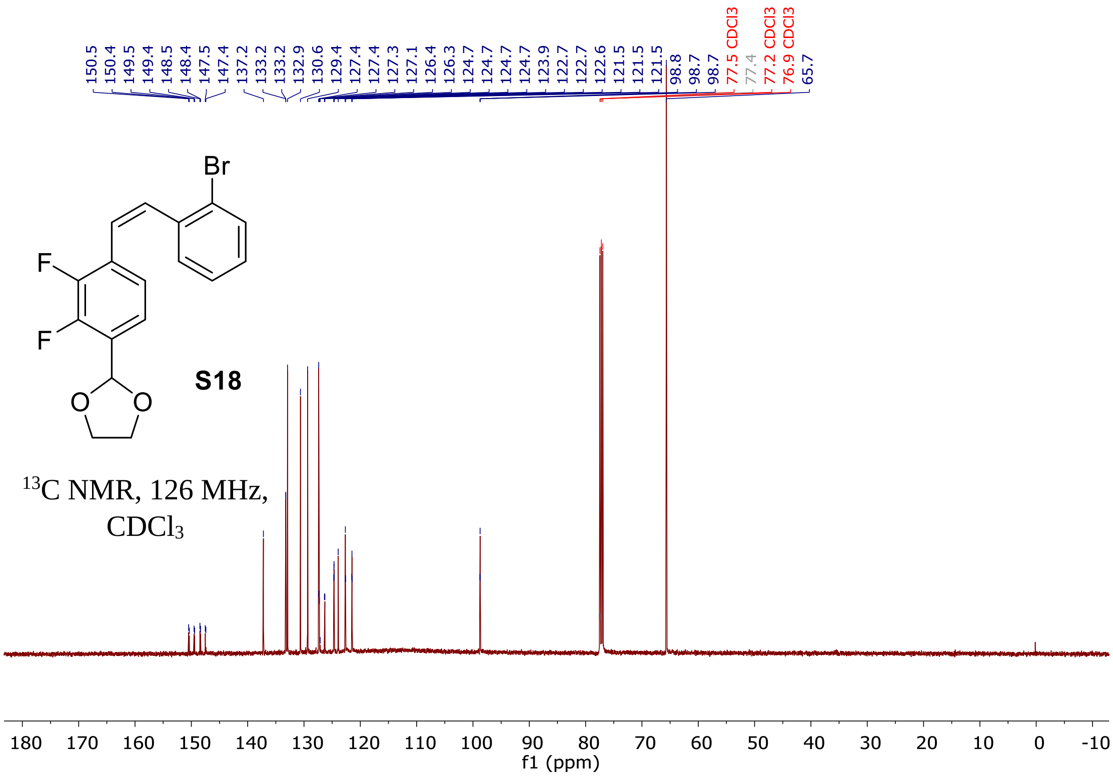
150.5 150.4 149.5 149.4 148.5 148.4 147.5 147.4 137.2 133.2 133.2 132.9 130.6 129.4 127.4 127.4 127.3 127.1 126.4 126.3 124.7 124.7 124.7 124.7 123.9 122.7 122.7 122.6 121.5 121.5 121.5 98.8 98.7 98.7 77.5 CDCl3 77.4 77.2 CDCl3 76.9 CDCl3 65.7
Br
F
F
O
O
S18
^{13}C NMR, 126 MHz, $CDCl_3$
f1 (ppm)

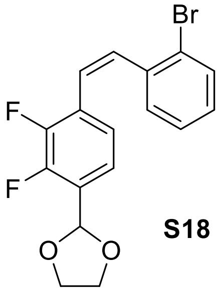

S18

^{19}F NMR, 376 MHz, $CDCl_3$

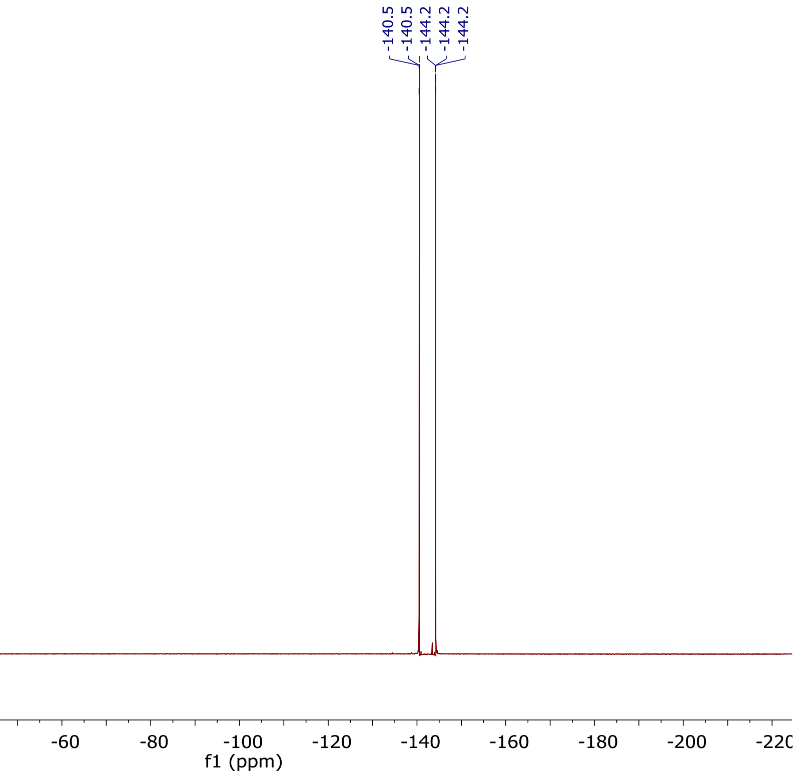

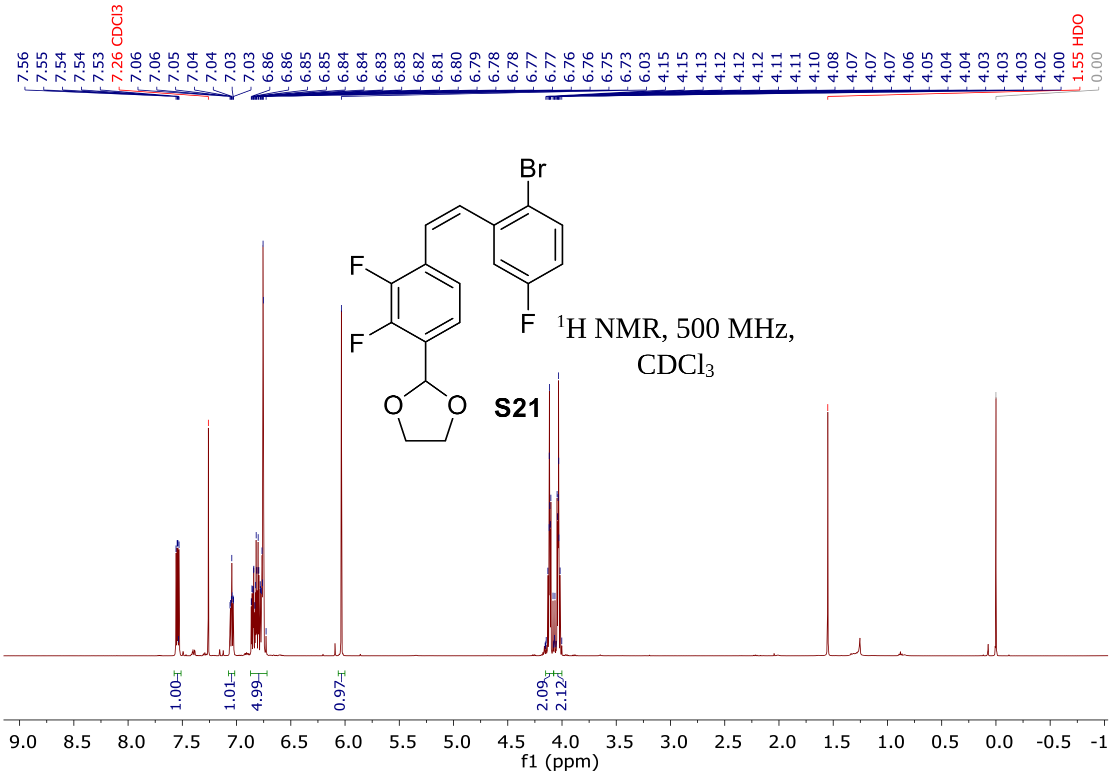
7.56
7.55
7.54
7.54
7.53
7.26 CDCl3
7.06
7.06
7.05
7.04
7.04
7.03
7.03
6.86
6.86
6.85
6.84
6.84
6.83
6.83
6.82
6.81
6.80
6.79
6.78
6.78
6.77
6.77
6.76
6.76
6.75
6.73
6.03
4.15
4.15
4.13
4.12
4.12
4.12
4.11
4.11
4.10
4.08
4.07
4.07
4.06
4.05
4.04
4.04
4.03
4.03
4.03
4.02
4.00
1.55 HDO
0.00
Br
F
F
F
O
O
S21
1H NMR, 500 MHz,
CDCl3
1.00
1.01
4.99
0.97
2.09
2.12
f1 (ppm)

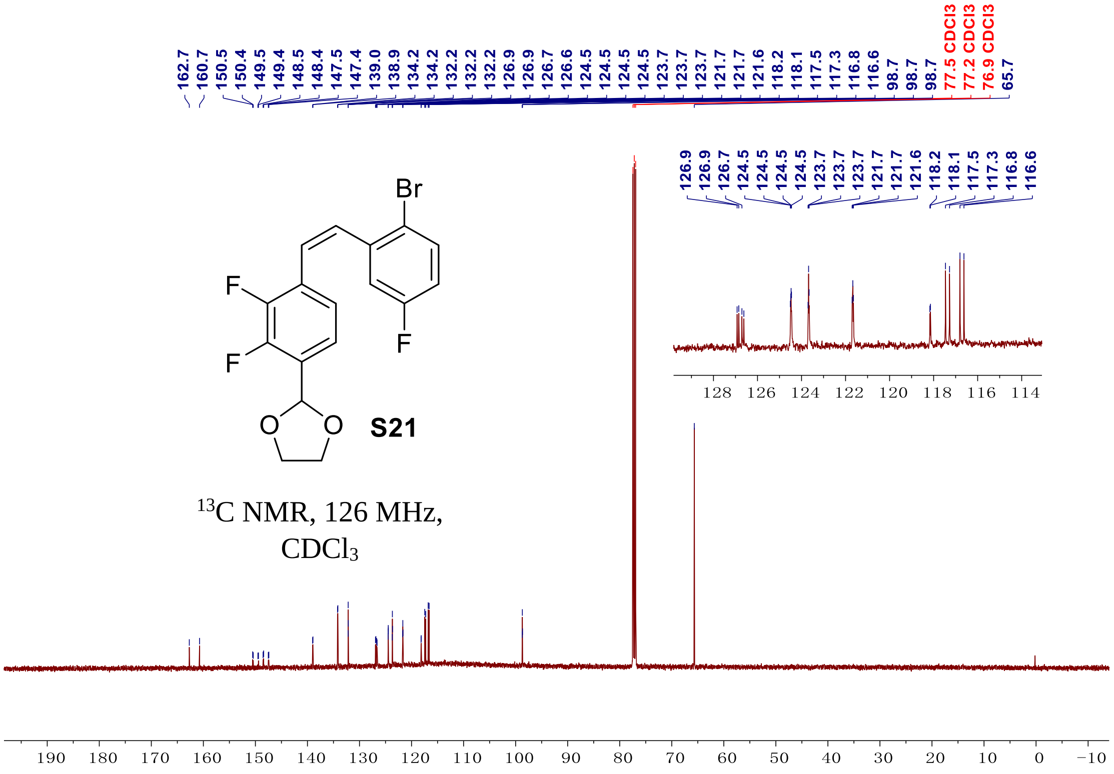
162.7
160.7
150.5
150.4
149.5
149.4
148.5
148.4
147.5
147.4
139.0
138.9
134.2
134.2
132.2
132.2
132.2
126.9
126.9
126.7
126.6
124.5
124.5
124.5
124.5
123.7
123.7
123.7
121.7
121.6
118.2
118.1
117.5
117.3
116.8
116.6
98.7
98.7
98.7
77.5 CDCl3
77.2 CDCl3
76.9 CDCl3
65.7
Br
F
F
F
O
O
S21
13C NMR, 126 MHz,
CDCl3

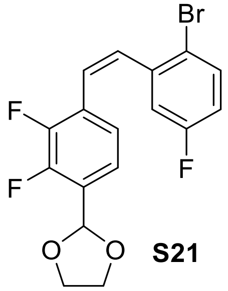

S21

^{19}F NMR, 376 MHz, $CDCl_3$

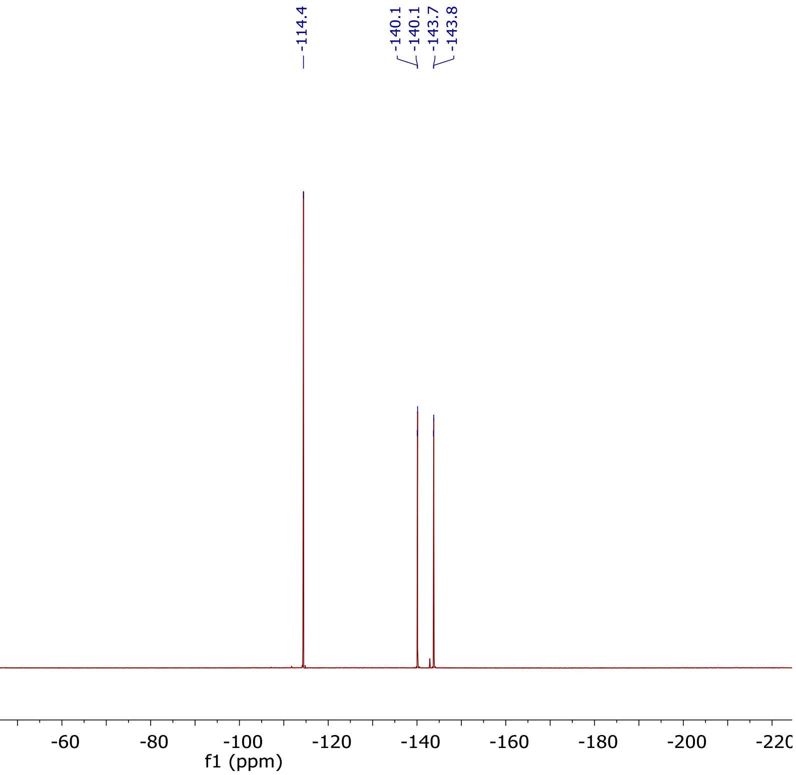

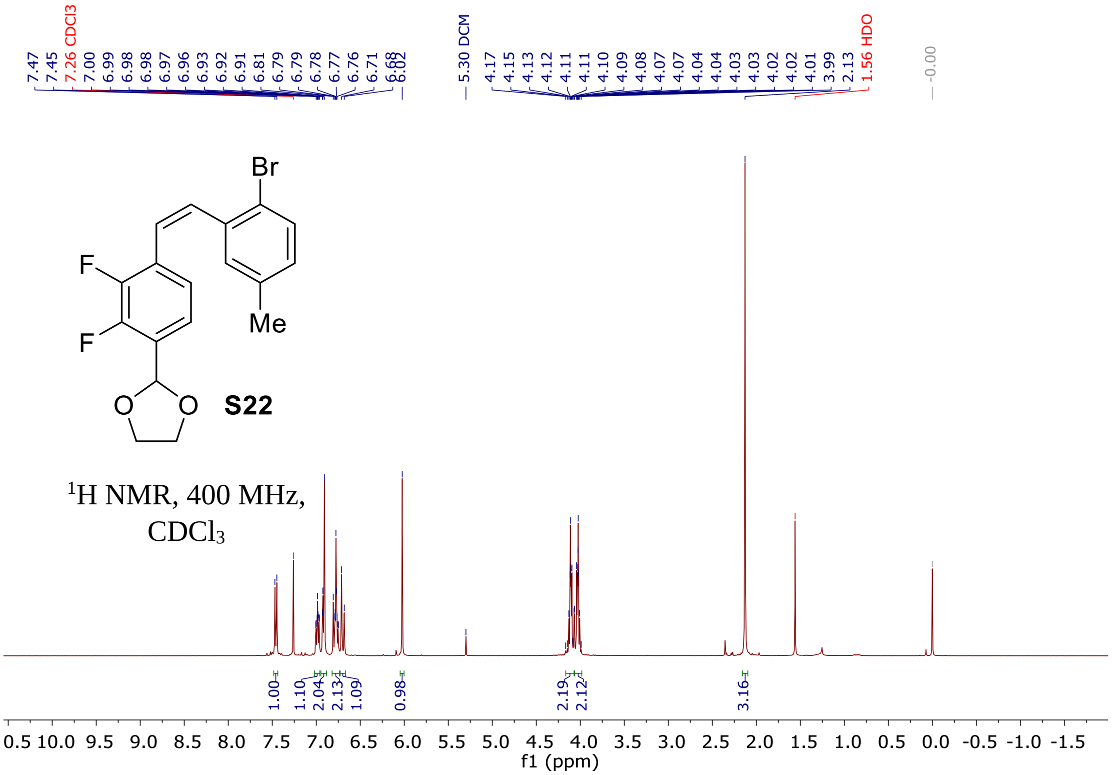
Br
F
F
Me
O
O
S22
^{1}H NMR, 400 MHz, $CDCl_3$
f1 (ppm)

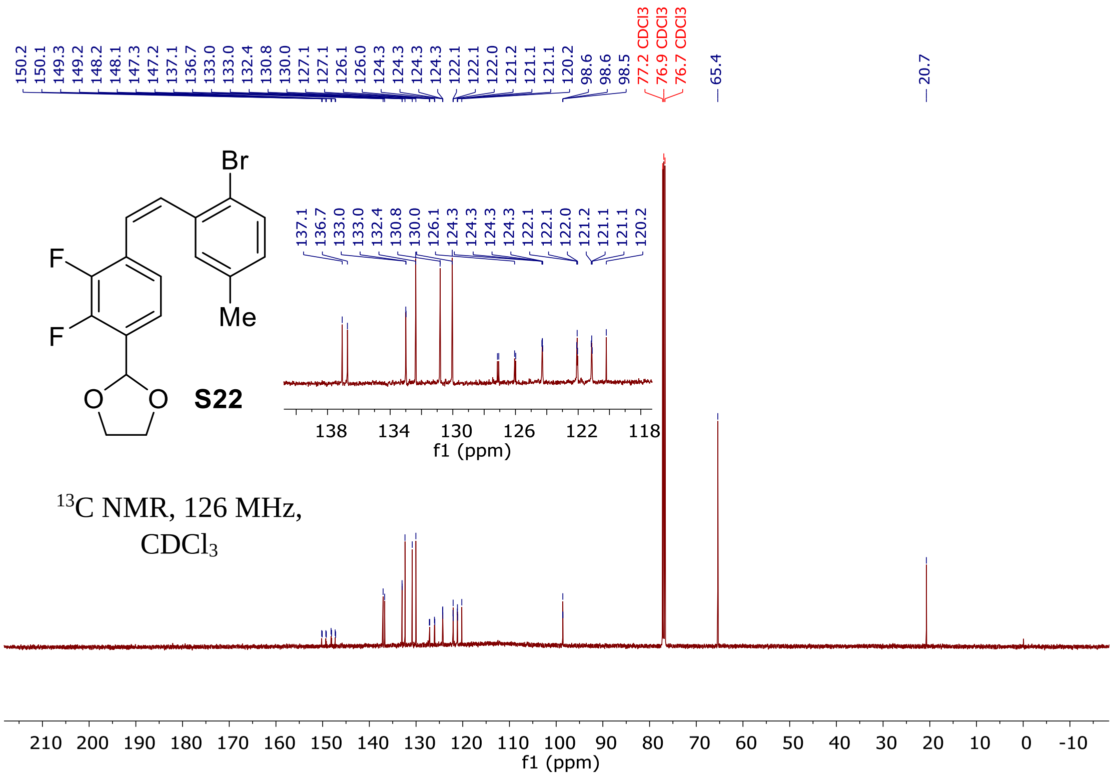
Br
F
F
Me
O
O
S22
^{13}C NMR, 126 MHz, $CDCl_3$
f1 (ppm)

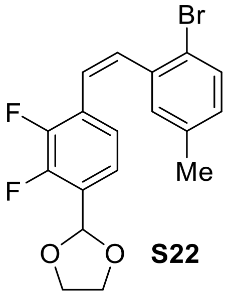

S22

^{19}F NMR, 376 MHz, $CDCl_3$

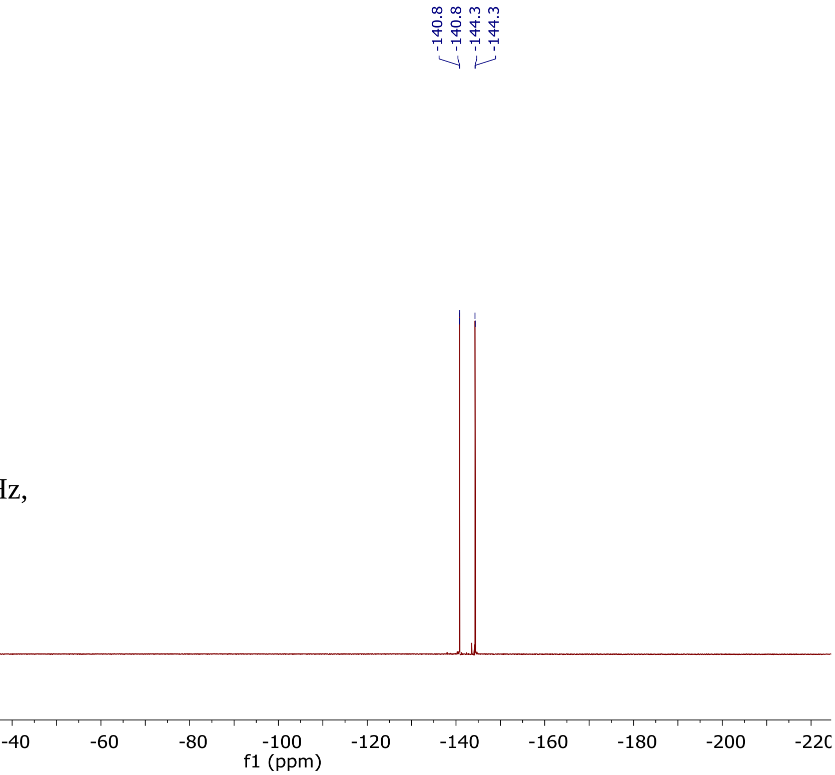

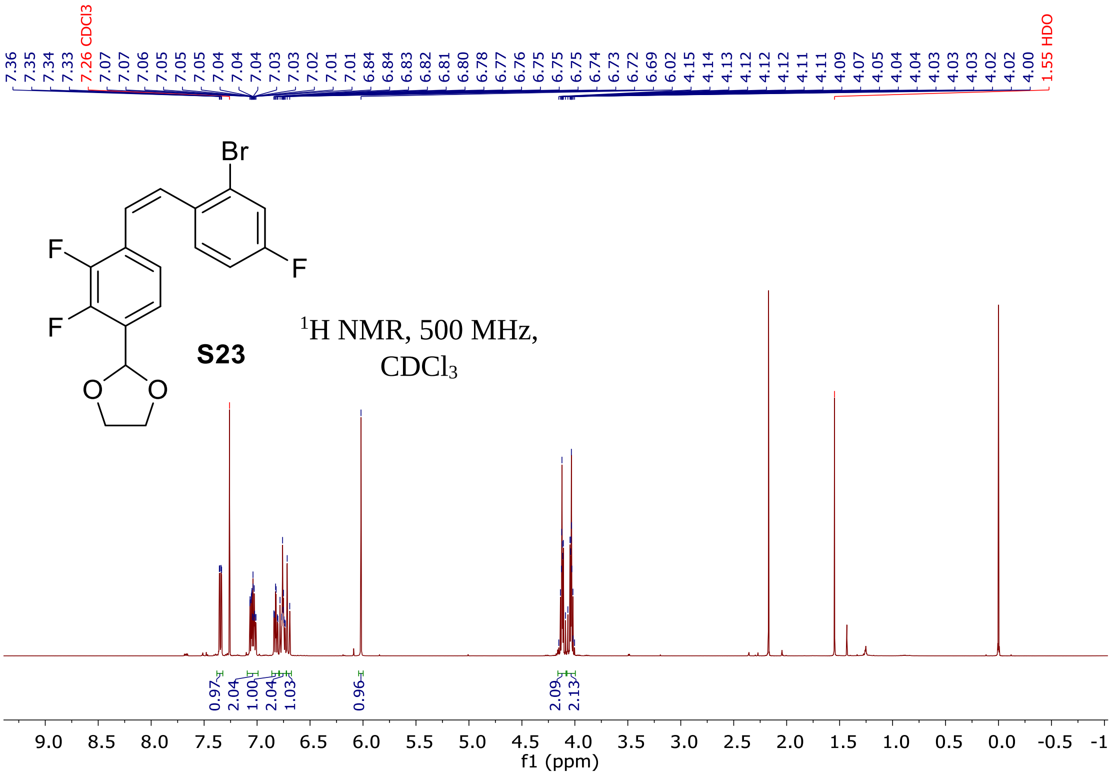
Br
F
F
F
O
O
S23
^{1}H NMR, 500 MHz,
$CDCl_3$
7.26 CDCl3
1.55 HDO
0.97
2.04
1.00
2.04
1.03
0.96
2.09
2.13
f1 (ppm)

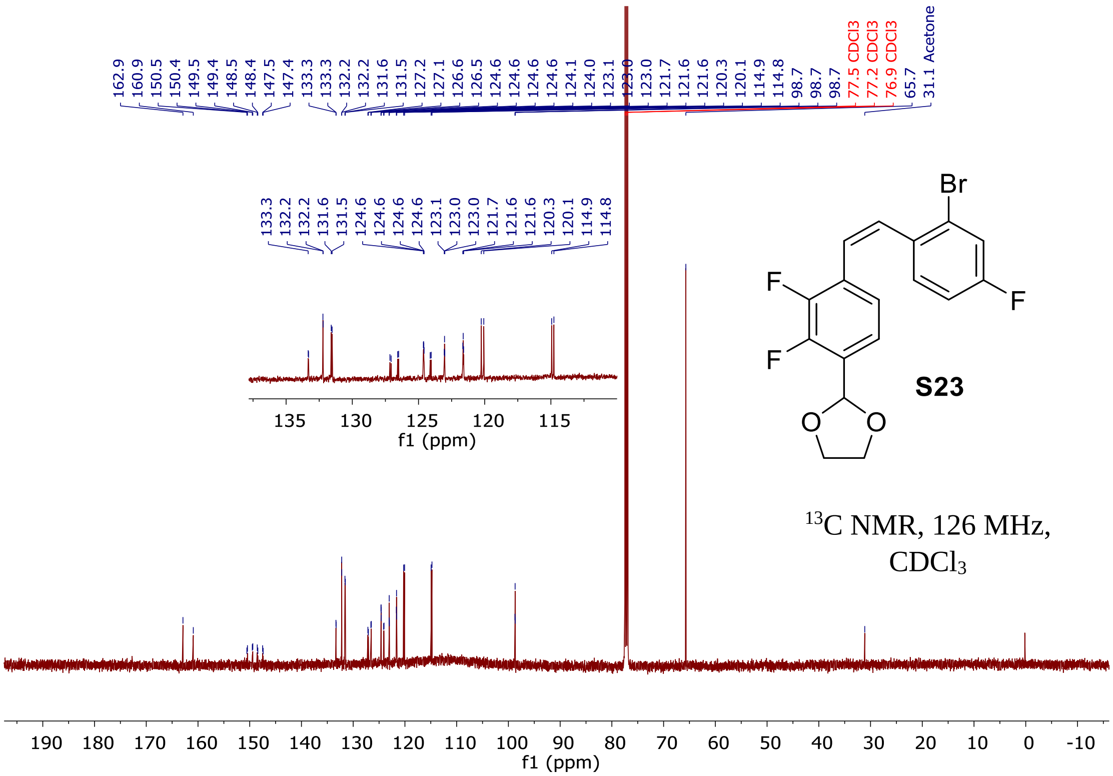
Br
F
F
F
O
O
S23
^{13}C NMR, 126 MHz,
$CDCl_3$
77.5 CDCl3
77.2 CDCl3
76.9 CDCl3
31.1 Acetone
f1 (ppm)

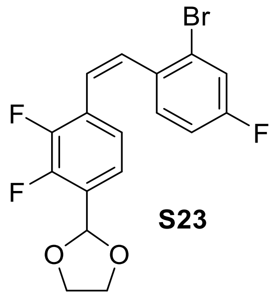

S23

^{19}F NMR, 376 MHz, $CDCl_3$

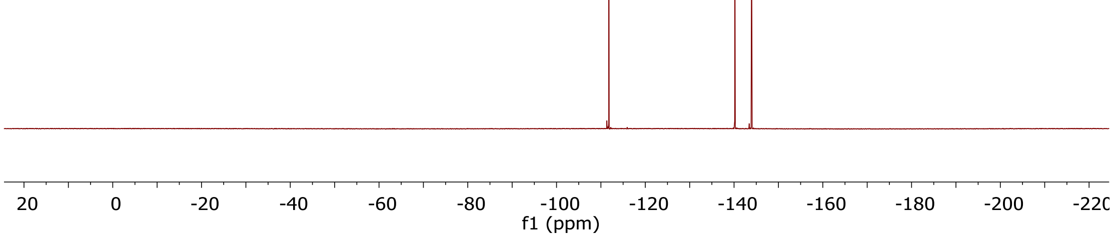

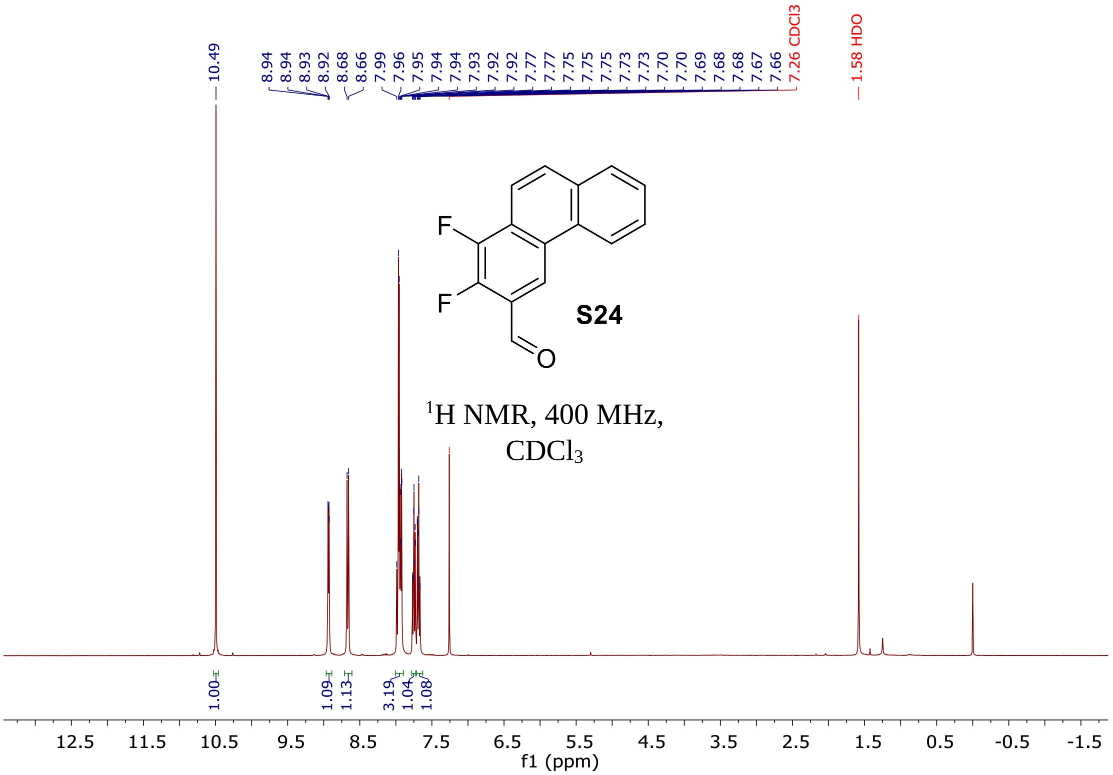

10.49
8.94
8.94
8.93
8.92
8.68
8.66
7.99
7.96
7.95
7.94
7.94
7.93
7.92
7.92
7.77
7.77
7.75
7.75
7.75
7.73
7.73
7.70
7.69
7.68
7.68
7.67
7.66
7.26 CDCl3
1.58 HDO
F
F
O
S24
1H NMR, 400 MHz, CDCl3
1.00
1.09
1.13
3.19
1.04
1.08
12.5
11.5
10.5
9.5
8.5
7.5
6.5
5.5
4.5
3.5
2.5
1.5
0.5
-0.5
-1.5
f1 (ppm)

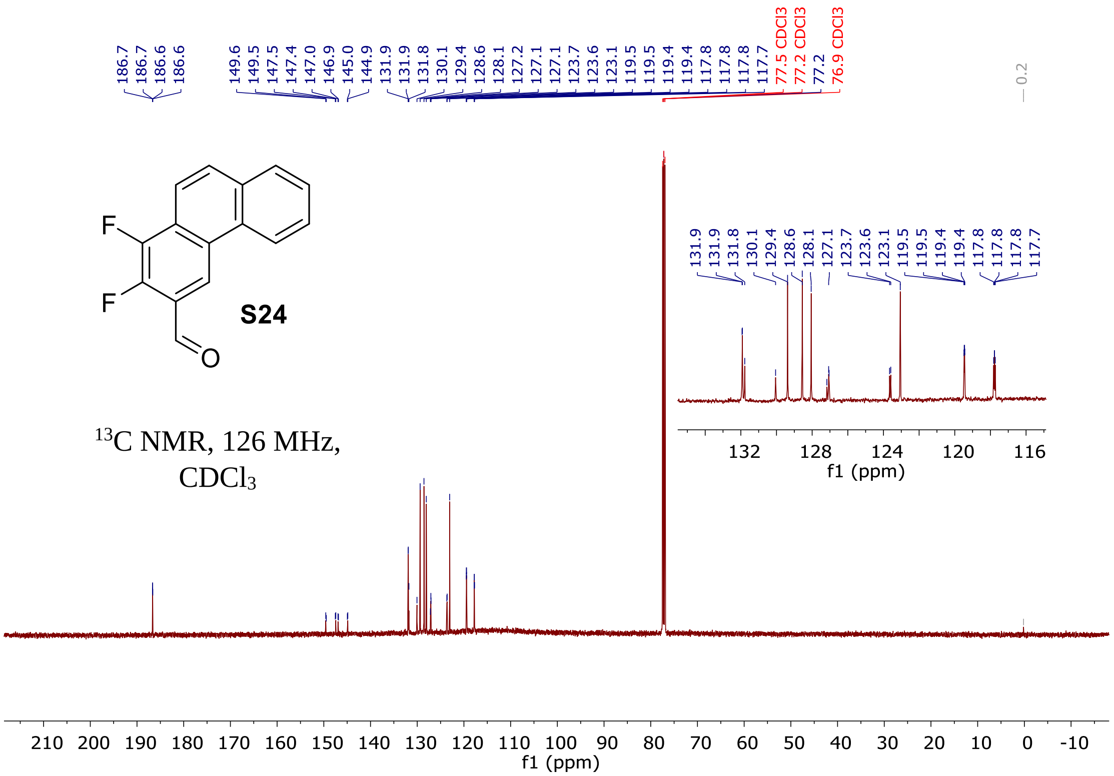

186.7
186.7
186.6
186.6
149.6
149.5
147.5
147.4
147.0
146.9
145.0
144.9
131.9
131.9
131.8
130.1
129.4
128.6
128.1
127.2
127.1
127.1
123.7
123.6
123.1
119.5
119.5
119.4
119.4
117.8
117.8
117.8
117.7
77.5 CDCl3
77.2 CDCl3
77.2
76.9 CDCl3
0.2
F
F
O
S24
13C NMR, 126 MHz, CDCl3
131.9
131.9
131.8
130.1
129.4
128.6
128.1
127.1
123.7
123.6
123.1
119.5
119.5
119.4
119.4
117.8
117.8
117.8
117.7
132
128
124
120
116
f1 (ppm)
210
200
190
180
170
160
150
140
130
120
110
100
90
80
70
60
50
40
30
20
10
0
-10
f1 (ppm)

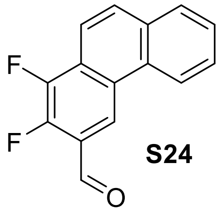

S24

^{19}F NMR, 376 MHz, $CDCl_3$

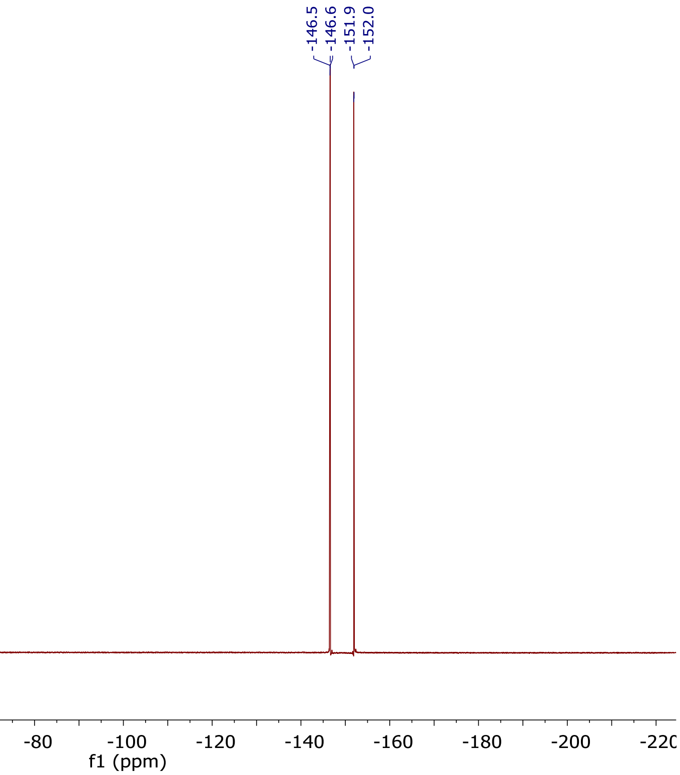

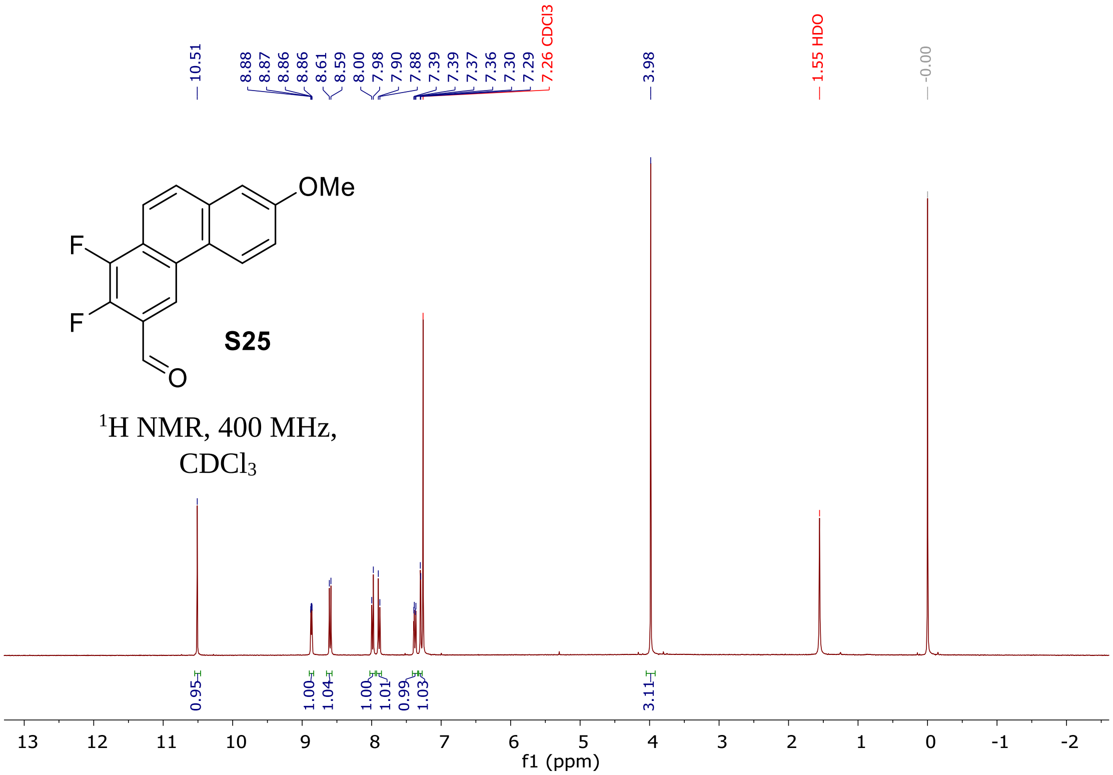
10.51
8.88
8.87
8.86
8.86
8.61
8.59
8.00
7.98
7.90
7.88
7.39
7.39
7.37
7.36
7.30
7.29
7.26 CDCl3
3.98
1.55 HDO
-0.00
OMe
F
F
O
S25
^{1}H NMR, 400 MHz, CDCl$_3$
0.95
1.00
1.04
1.00
1.01
0.99
1.03
3.11
f1 (ppm)

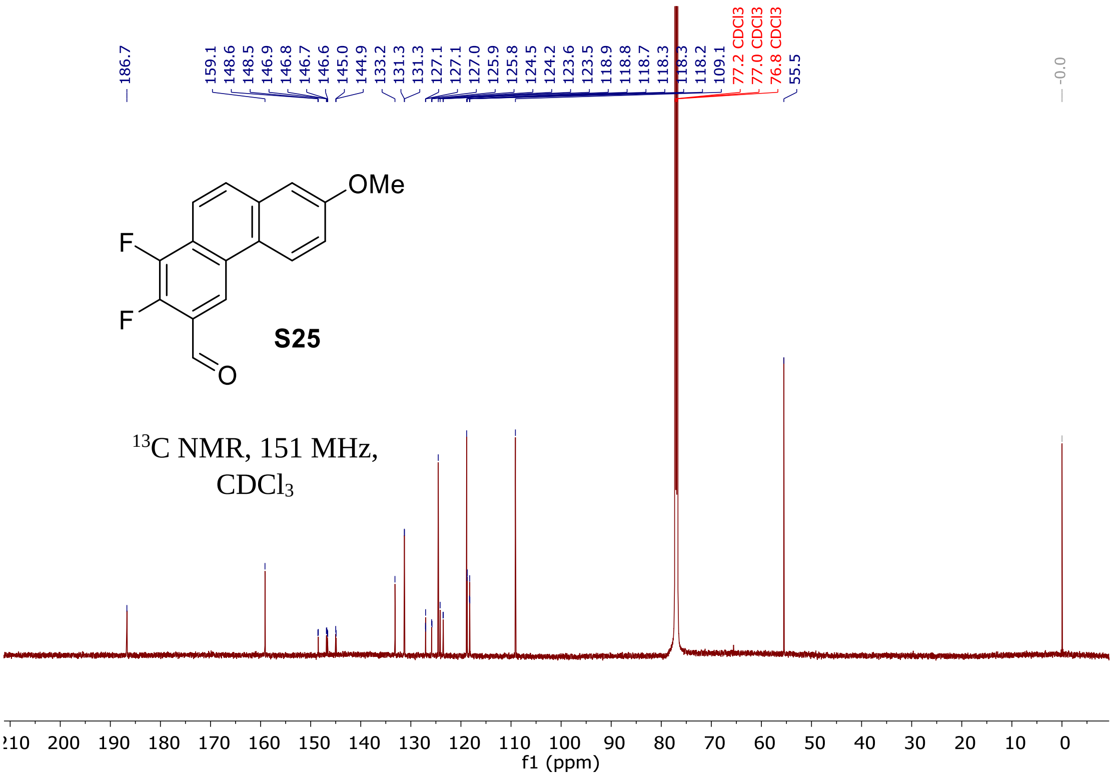
186.7
159.1
148.6
148.5
146.9
146.8
146.7
146.6
145.0
144.9
133.2
131.3
131.3
127.1
127.1
127.0
125.9
125.8
124.5
124.2
123.6
123.5
118.9
118.8
118.7
118.3
118.3
118.2
109.1
77.2 CDCl3
77.0 CDCl3
76.8 CDCl3
55.5
-0.0
OMe
F
F
O
S25
^{13}C NMR, 151 MHz, CDCl$_3$
f1 (ppm)

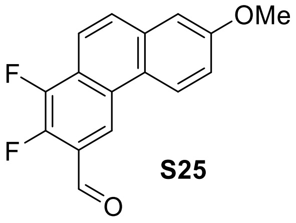

S25

^{19}F NMR, 376 MHz, $CDCl_3$

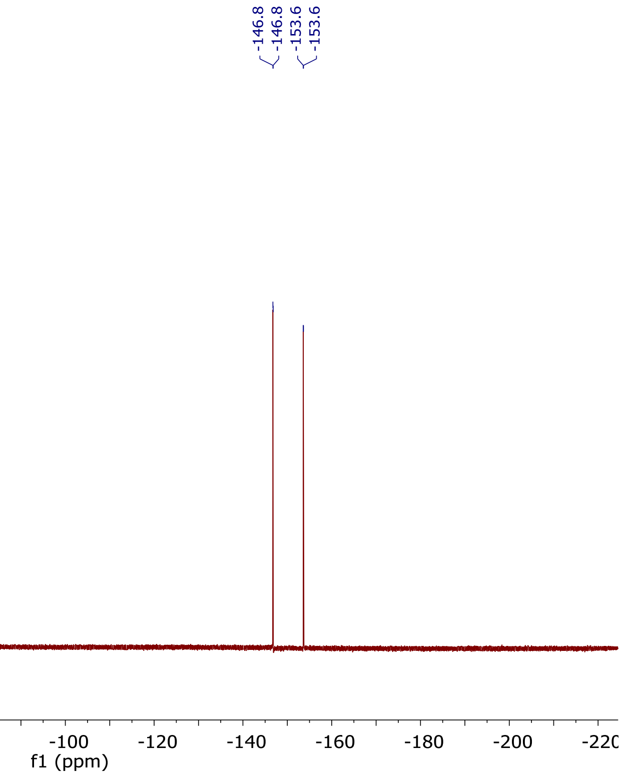

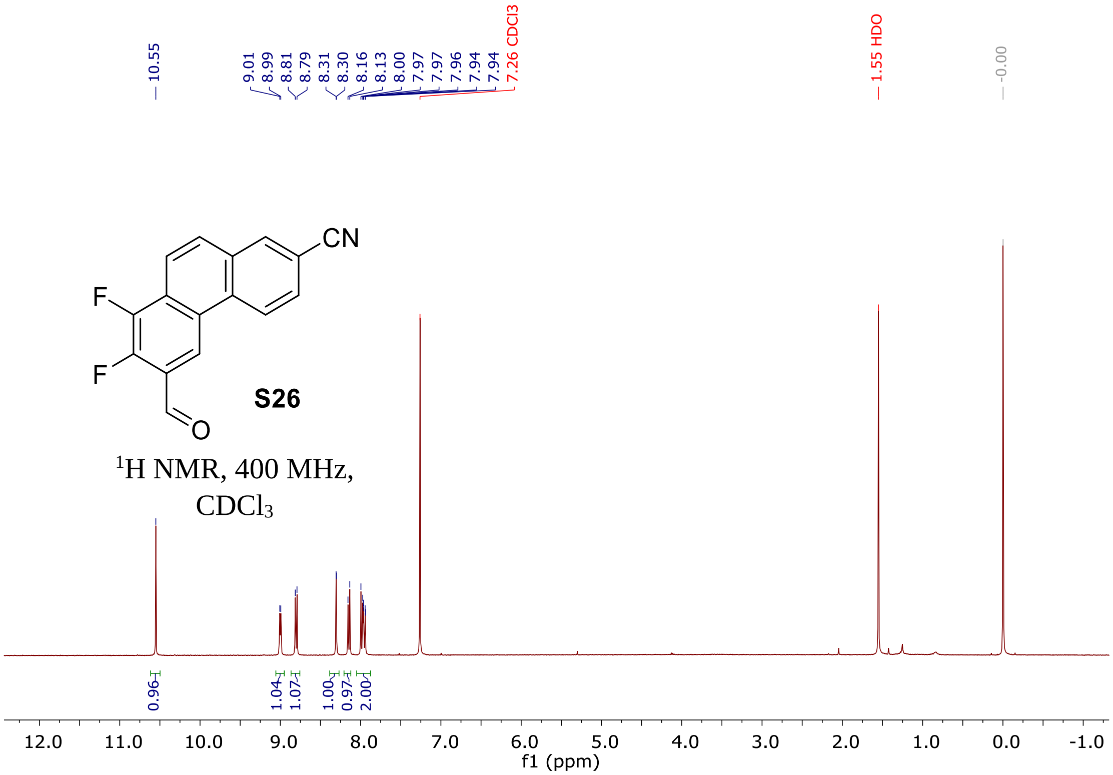
10.55
9.01
8.99
8.81
8.79
8.31
8.30
8.16
8.13
8.00
7.97
7.97
7.96
7.94
7.94
7.26 CDCl3
1.55 HDO
-0.00
CN
F
F
O
S26
¹H NMR, 400 MHz, CDCl₃
0.96
1.04
1.07
1.00
0.97
2.00
12.0 11.0 10.0 9.0 8.0 7.0 6.0 5.0 4.0 3.0 2.0 1.0 0.0 -1.0
f1 (ppm)

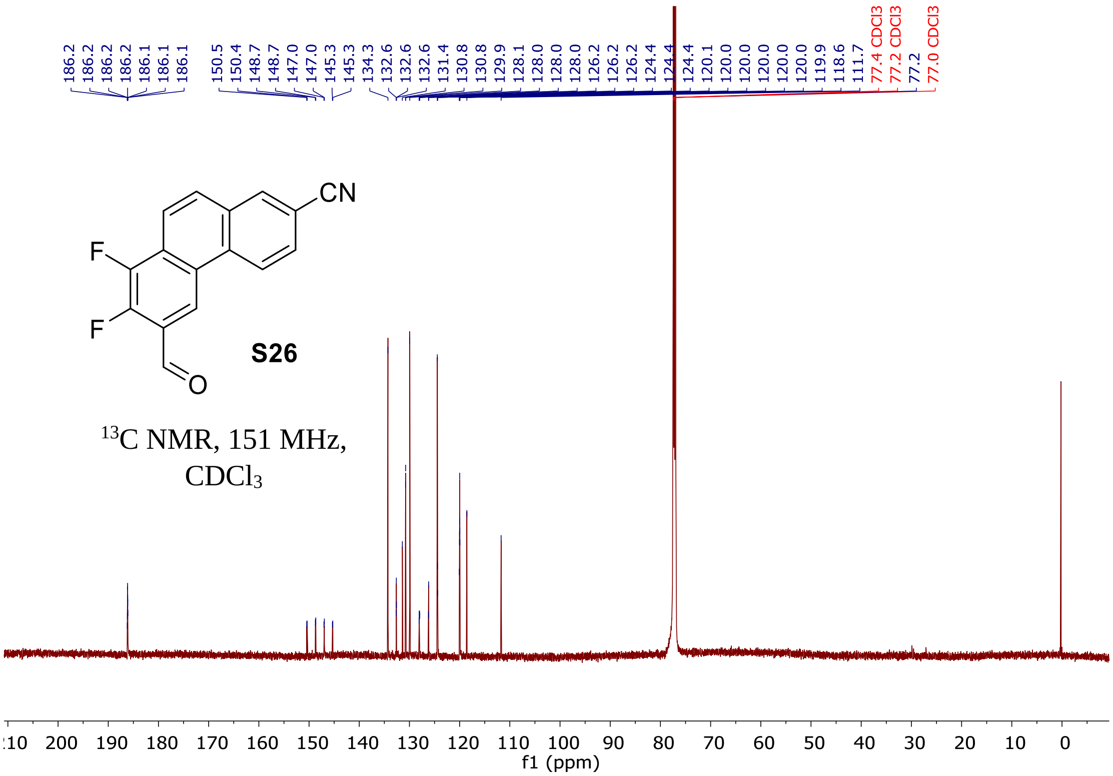
186.2
186.2
186.2
186.2
186.1
186.1
186.1
150.5
150.4
148.7
148.7
147.0
147.0
145.3
145.3
134.3
132.6
132.6
132.6
131.4
130.8
130.8
129.9
128.1
128.0
128.0
128.0
126.2
126.2
126.2
124.4
124.4
124.4
124.4
120.1
120.0
120.0
120.0
120.0
120.0
119.9
118.6
111.7
77.4 CDCl3
77.2 CDCl3
77.2
77.0 CDCl3
CN
F
F
O
S26
¹³C NMR, 151 MHz, CDCl₃
210 200 190 180 170 160 150 140 130 120 110 100 90 80 70 60 50 40 30 20 10 0
f1 (ppm)

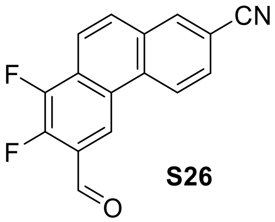

S26

^{19}F NMR, 376 MHz, $CDCl_3$

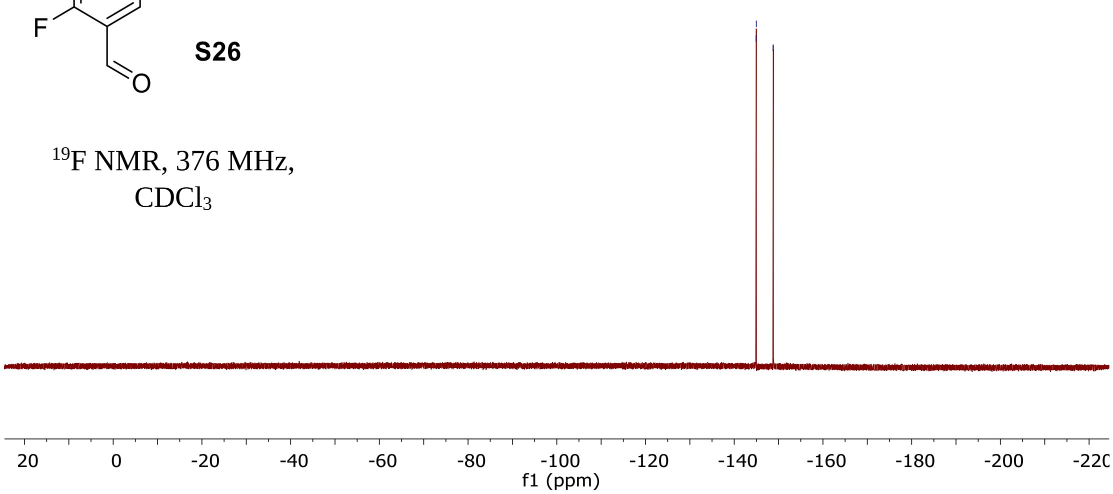

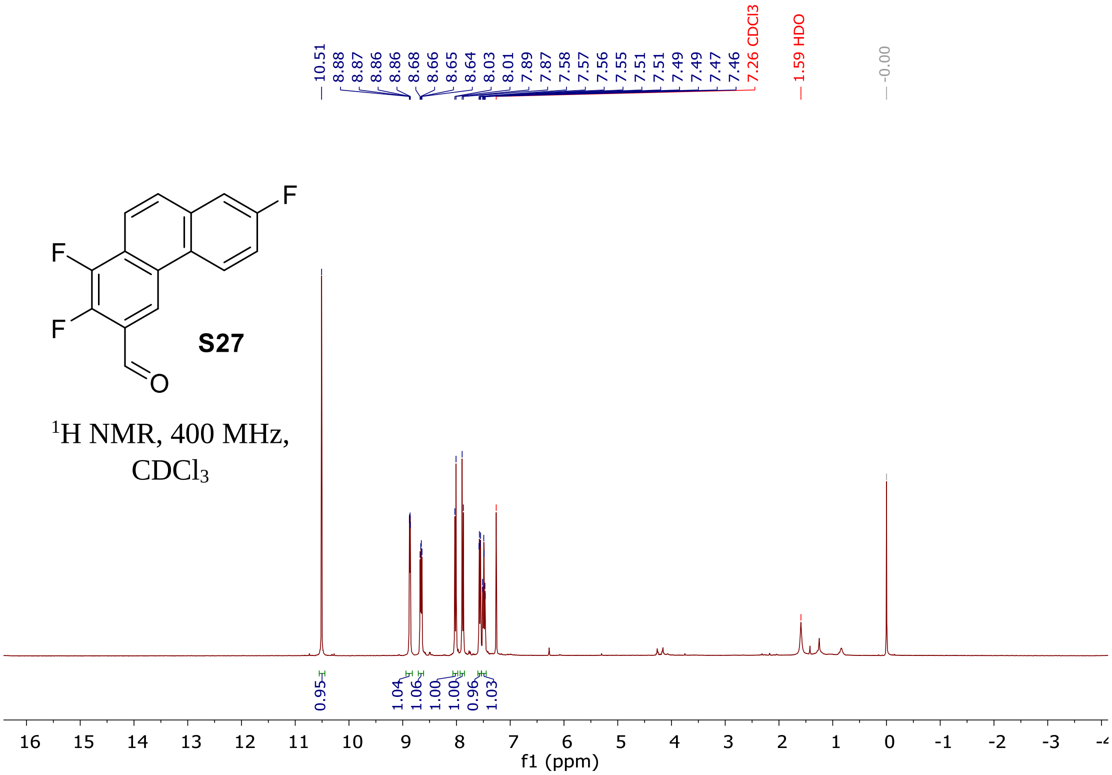

^{1}H NMR, 400 MHz, $CDCl_3$

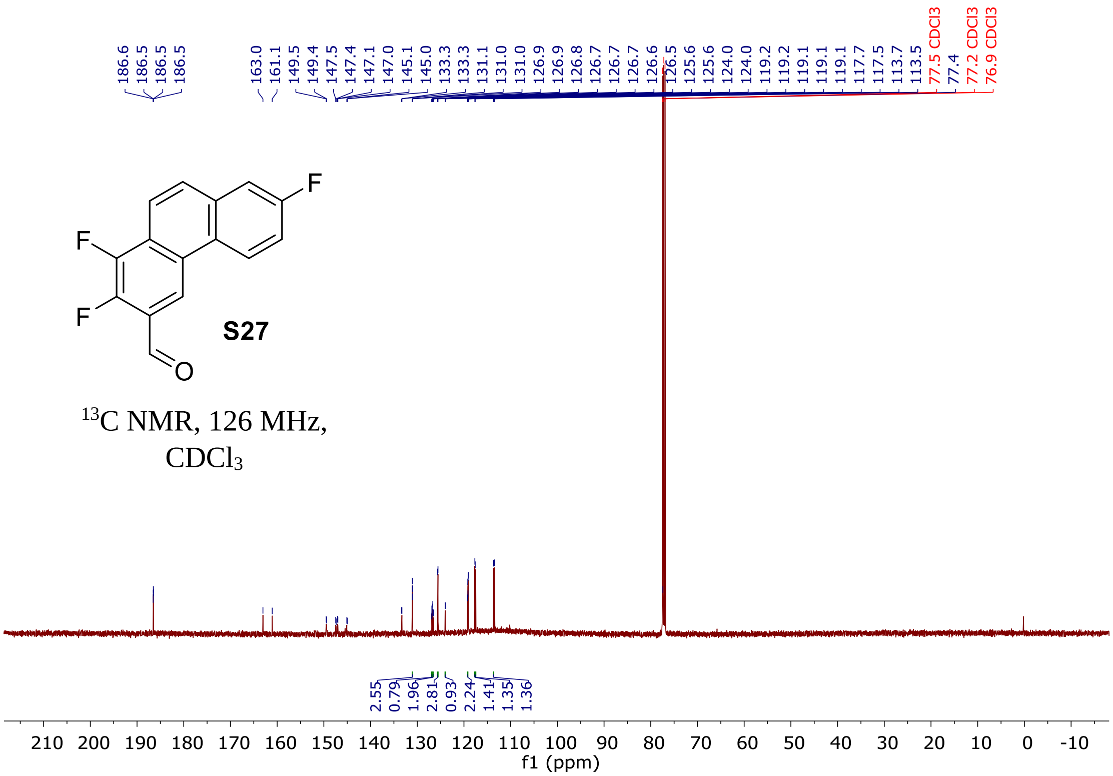

^{13}C NMR, 126 MHz, $CDCl_3$

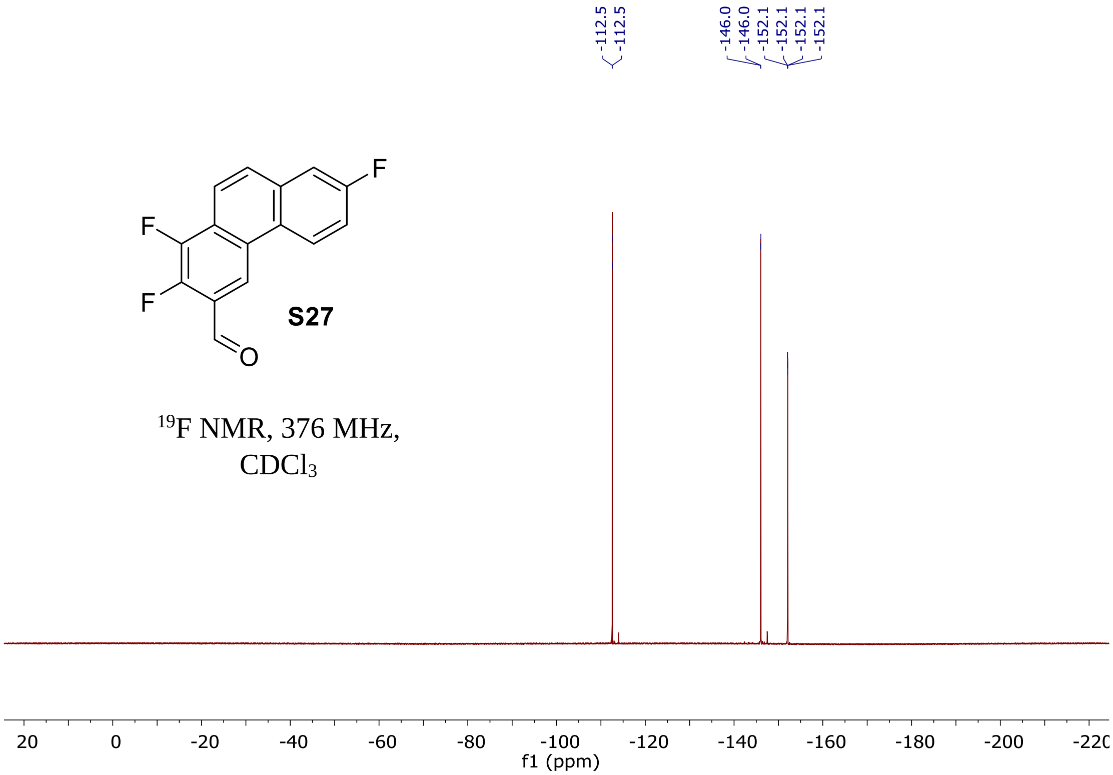

^{19}F NMR, 376 MHz, $CDCl_3$

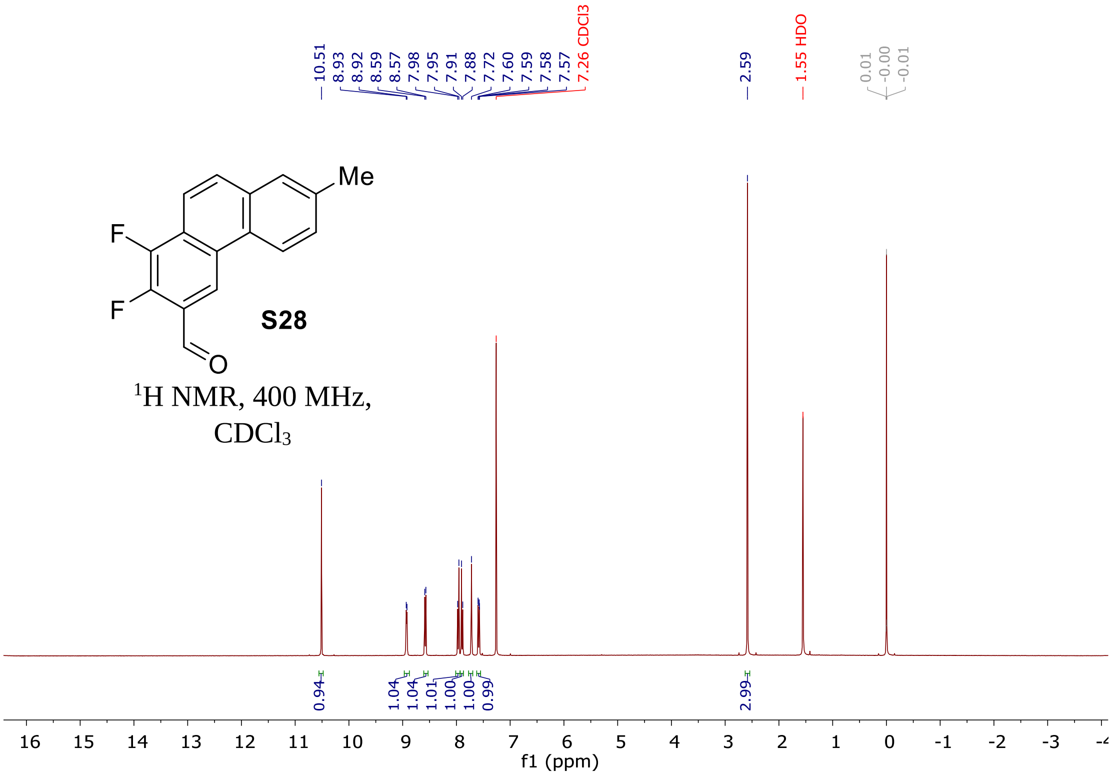
10.51
8.93
8.92
8.59
8.57
7.98
7.95
7.91
7.88
7.72
7.60
7.59
7.58
7.57
7.26 CDCl3
2.59
1.55 HDO
0.01
-0.00
-0.01
Me
F
F
O
S28
^{1}H NMR, 400 MHz, $CDCl_3$
0.94
1.04
1.04
1.01
1.00
1.00
0.99
2.99
f1 (ppm)

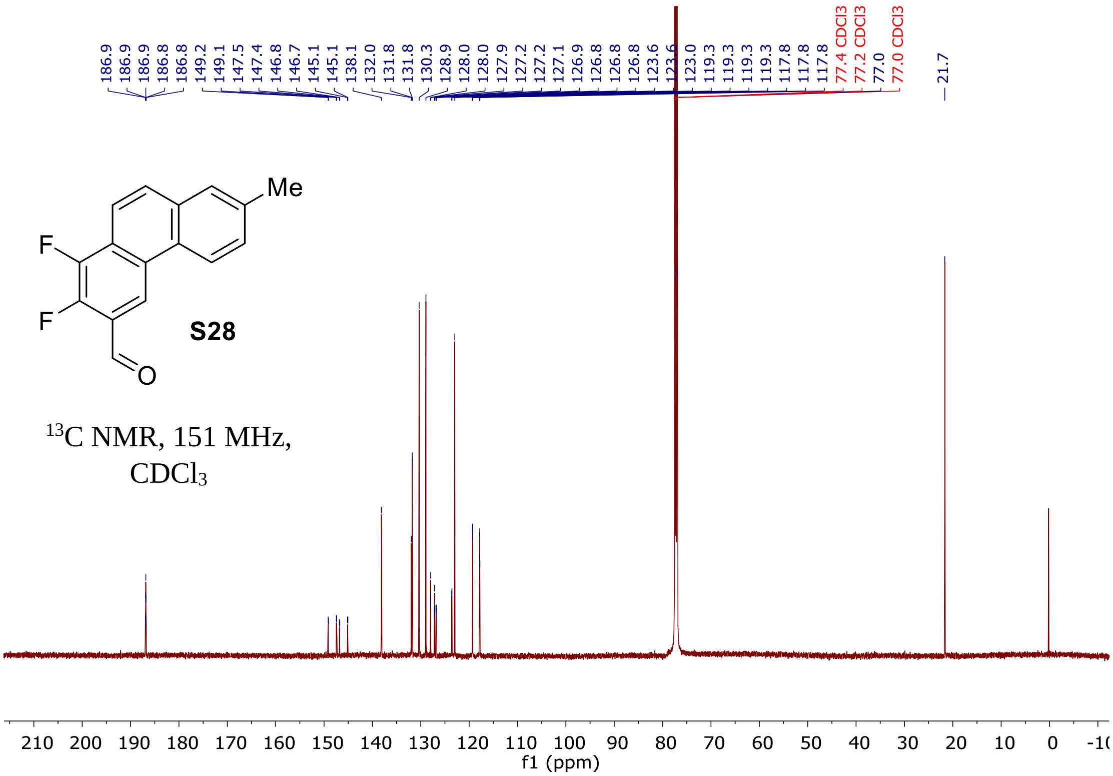
186.9
186.9
186.9
186.8
186.8
149.2
149.1
147.5
147.4
146.8
146.7
145.1
145.1
138.1
132.0
131.8
131.8
130.3
128.9
128.0
128.0
127.9
127.2
127.2
127.1
126.9
126.8
126.8
123.6
123.6
123.0
119.3
119.3
119.3
117.8
117.8
117.8
77.4 CDCl3
77.2 CDCl3
77.0
77.0 CDCl3
21.7
Me
F
F
O
S28
^{13}C NMR, 151 MHz, $CDCl_3$
f1 (ppm)

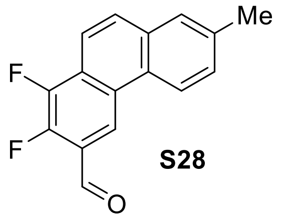

S28

^{19}F NMR, 376 MHz, $CDCl_3$

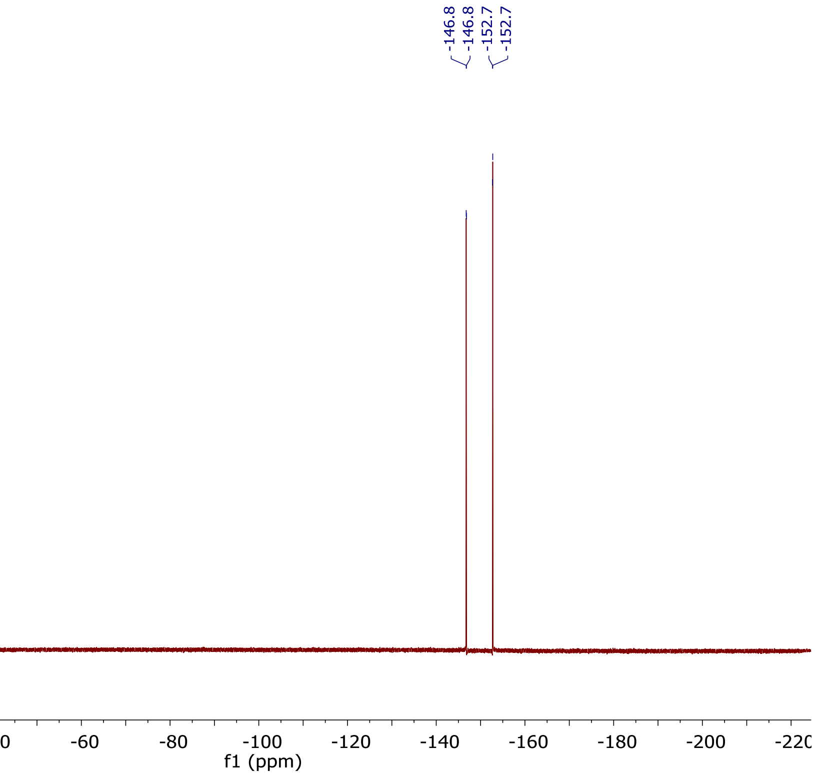

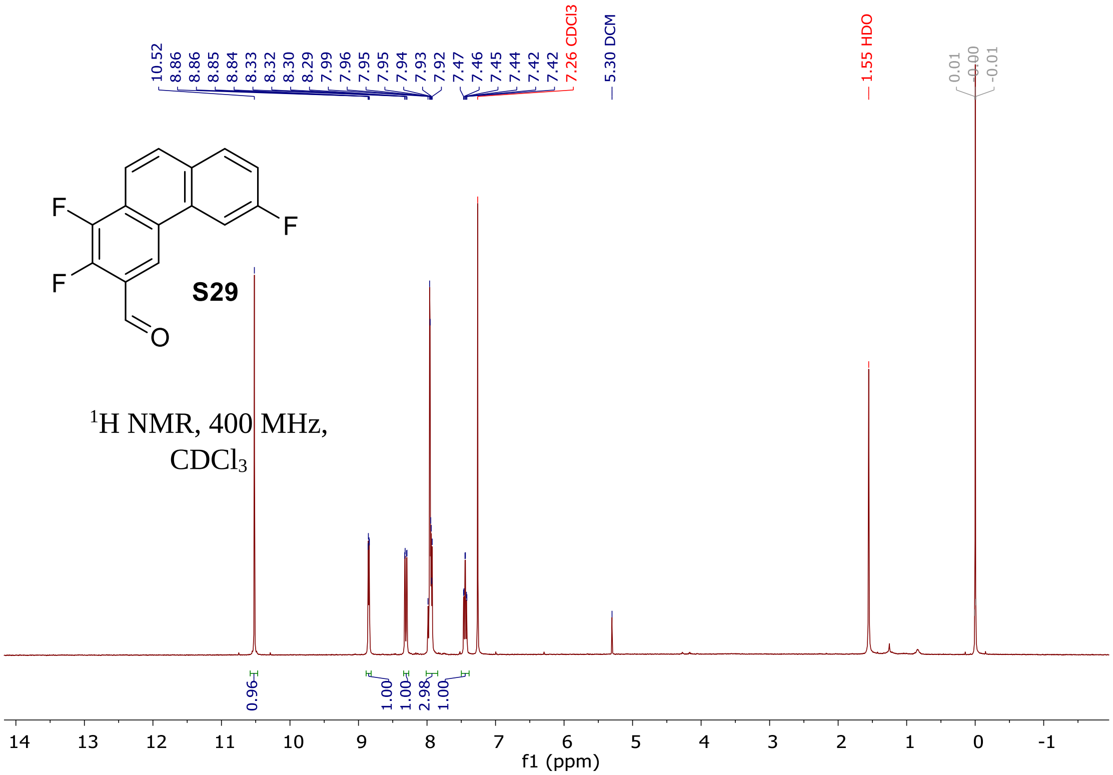
S29
^{1}H NMR, 400 MHz, CDCl$_3$
f1 (ppm)

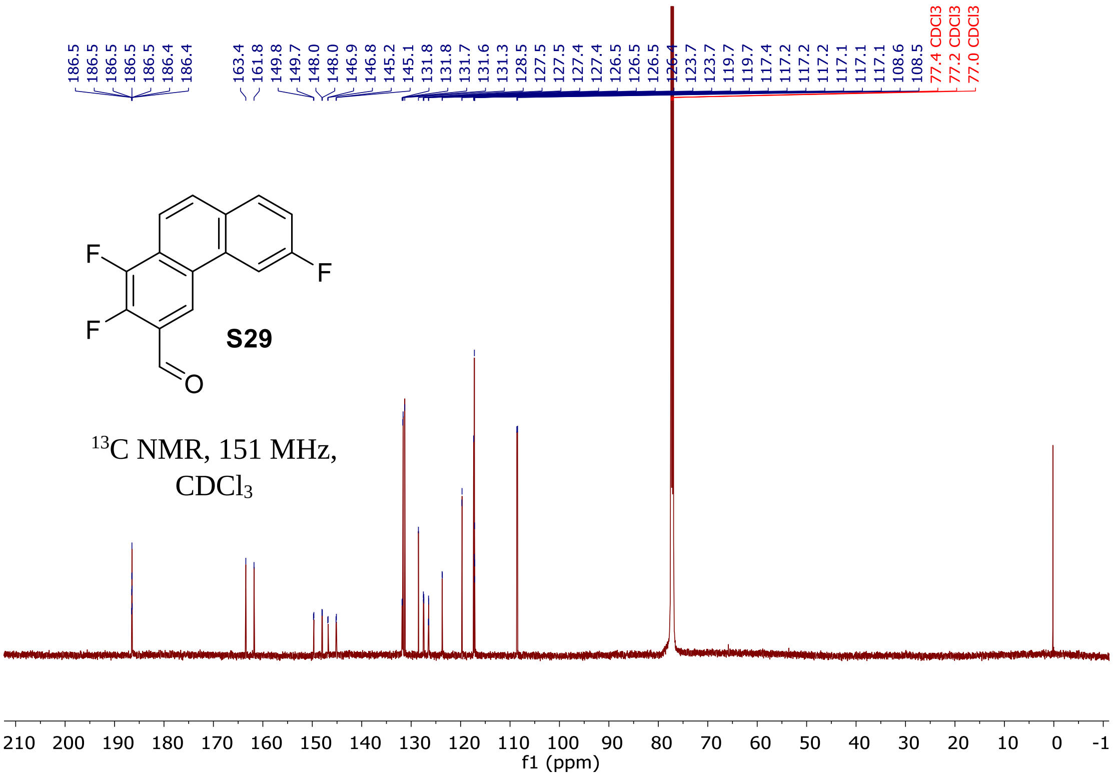
S29
^{13}C NMR, 151 MHz, CDCl$_3$
f1 (ppm)

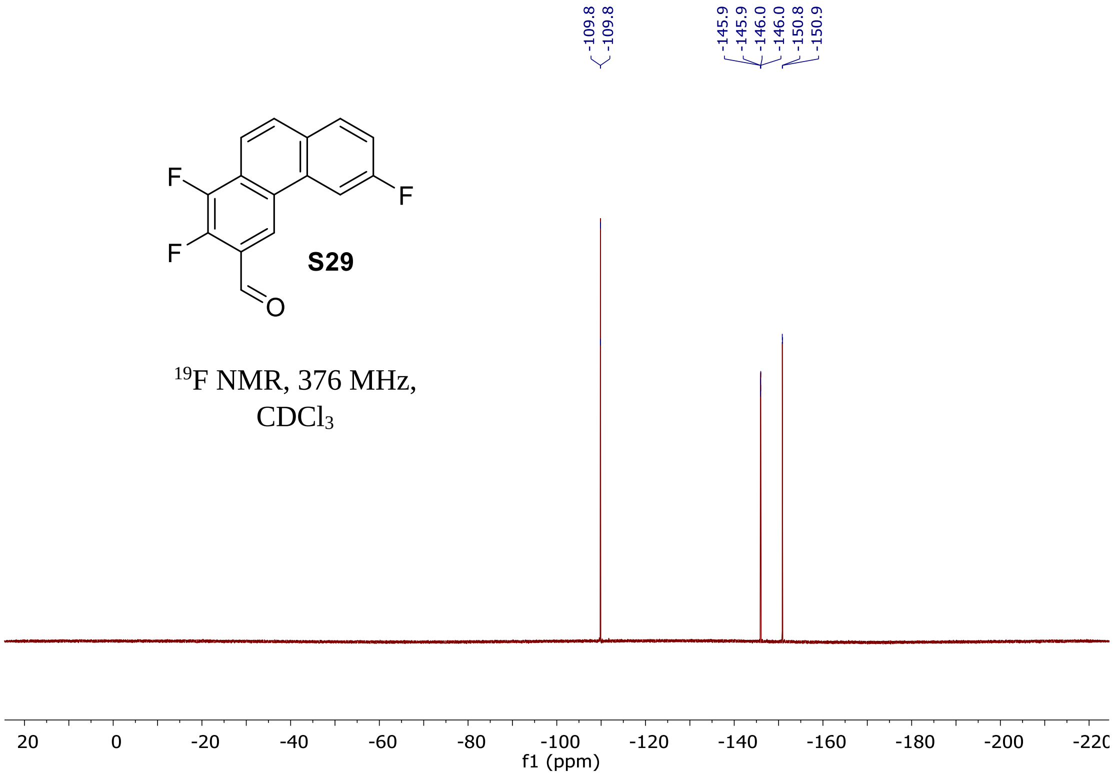
-109.8
-109.8
-145.9
-145.9
-146.0
-146.0
-150.8
-150.9
S29
19F NMR, 376 MHz,
CDCl3
f1 (ppm)

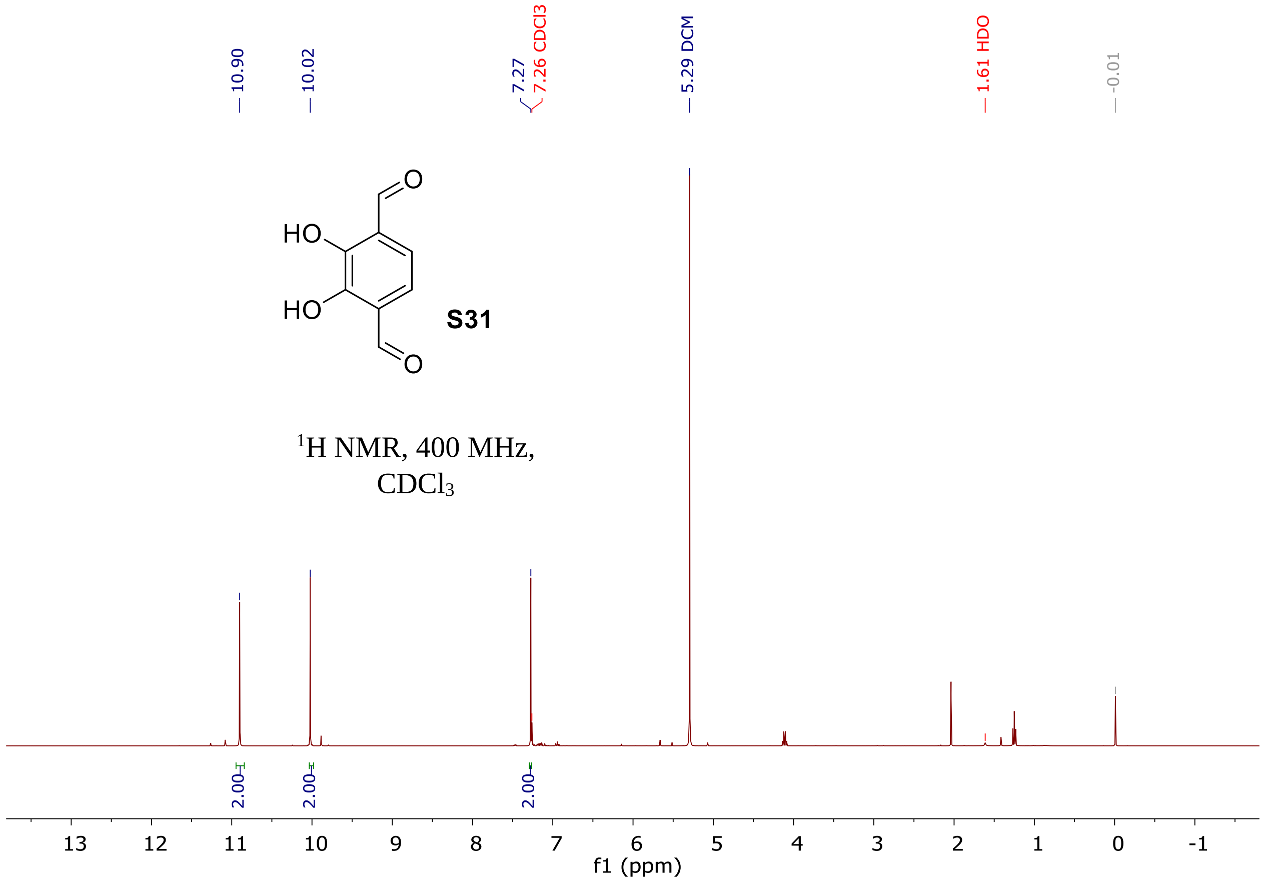
10.90
10.02
7.27
7.26 CDCl3
5.29 DCM
1.61 HDO
-0.01
HO
HO
O
O
S31
^{1}H NMR, 400 MHz, $CDCl_3$
2.00
2.00
2.00
13 12 11 10 9 8 7 6 5 4 3 2 1 0 -1
f1 (ppm)

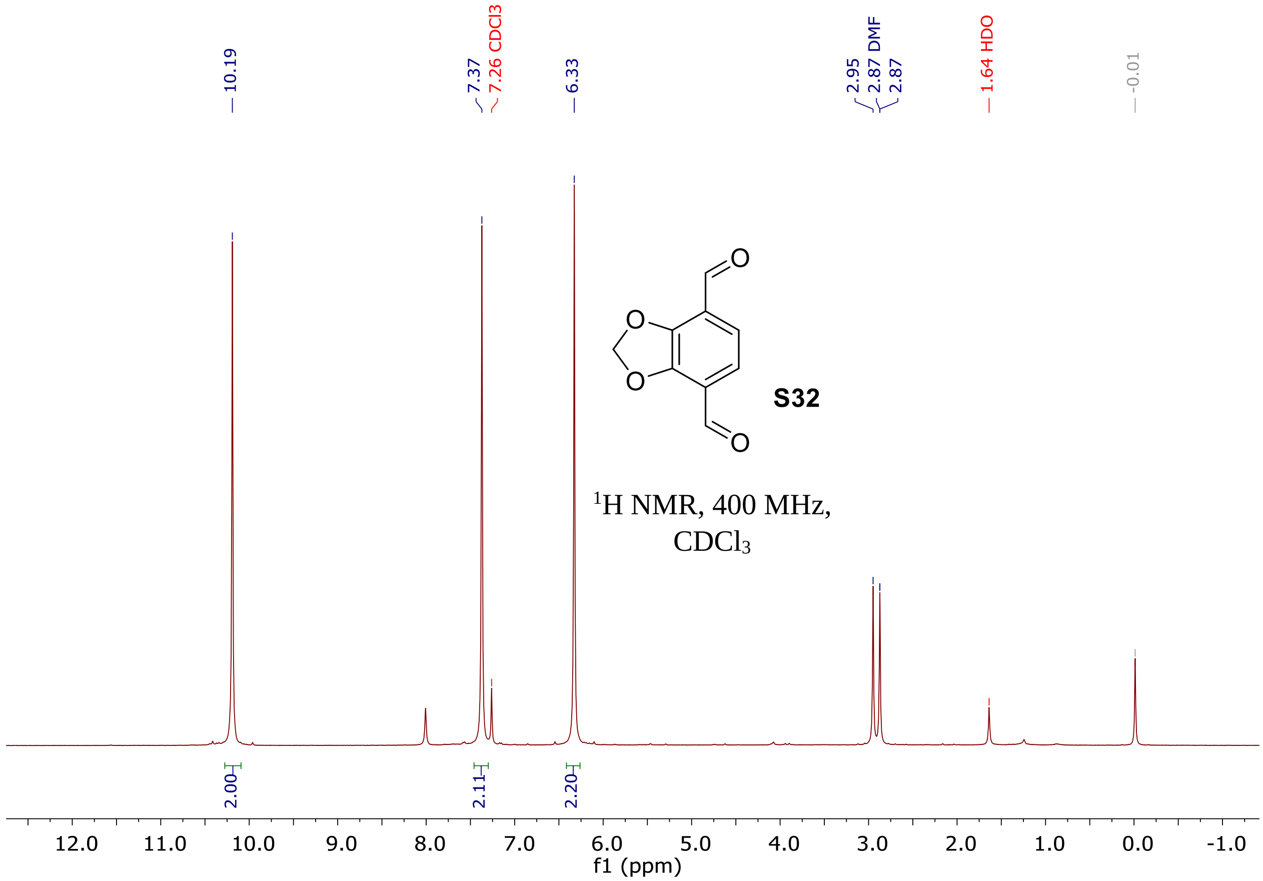
10.19
7.37
7.26 CDCl3
6.33
2.95
2.87 DMF
2.87
1.64 HDO
-0.01
S32
^{1}H NMR, 400 MHz, CDCl$_3$
2.00
2.11
2.20
12.0
11.0
10.0
9.0
8.0
7.0
6.0
5.0
4.0
3.0
2.0
1.0
0.0
-1.0
f1 (ppm)

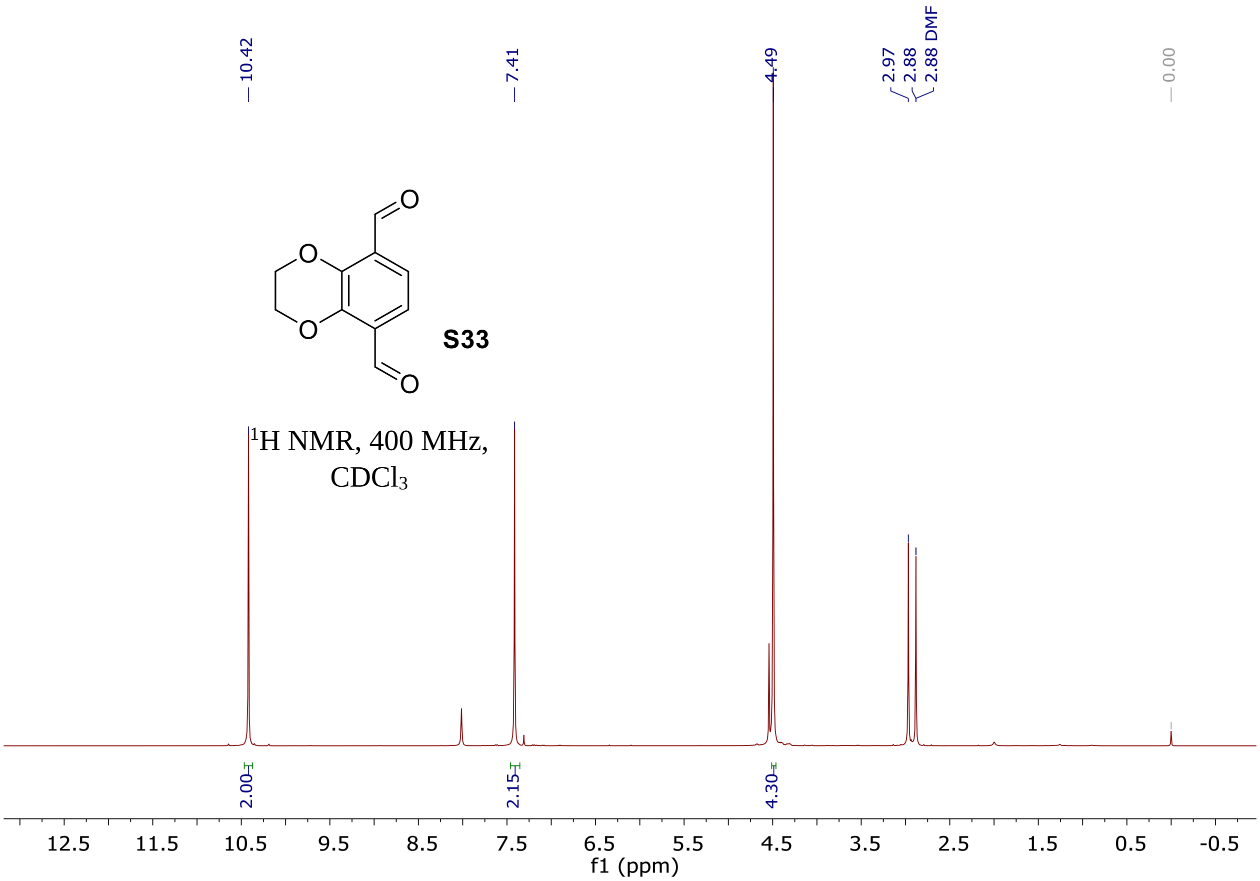

10.42
7.41
4.49
2.97
2.88
2.88 DMF
0.00
S33
1H NMR, 400 MHz,
CDCl3
2.00
2.15
4.30
12.5
11.5
10.5
9.5
8.5
7.5
6.5
5.5
4.5
3.5
2.5
1.5
0.5
-0.5
f1 (ppm)

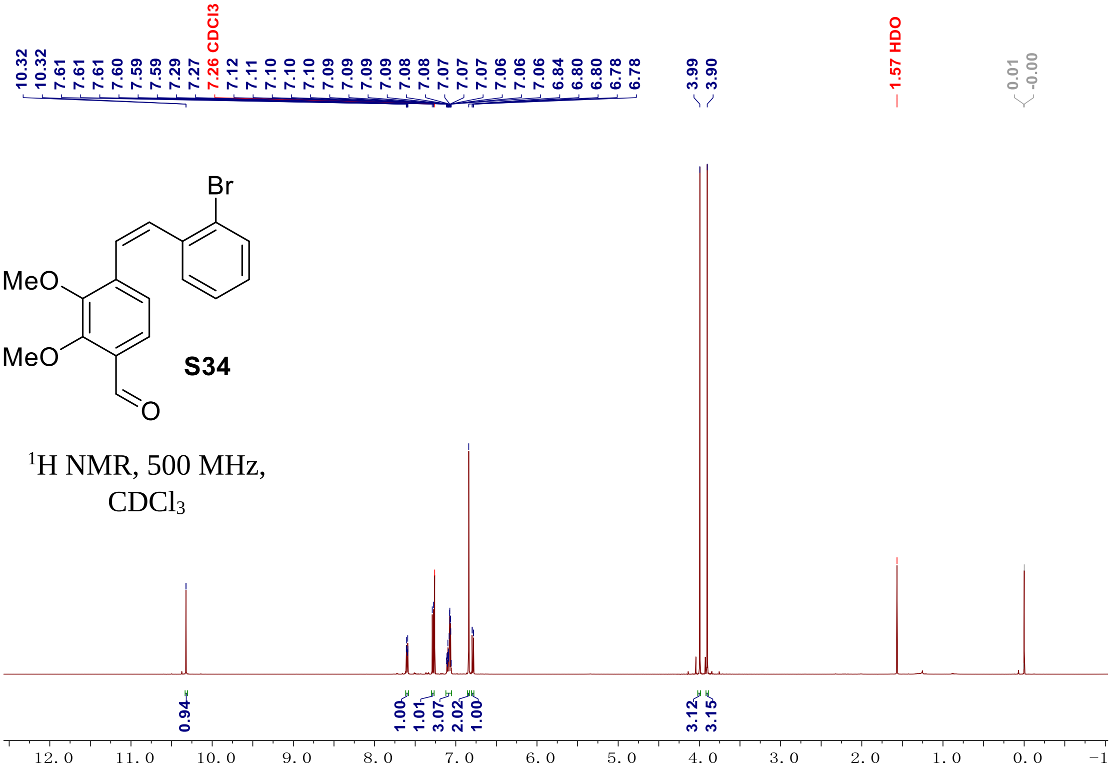
10.32
10.32
7.61
7.61
7.61
7.60
7.59
7.59
7.29
7.27
7.26 CDCl3
7.12
7.11
7.10
7.10
7.10
7.09
7.09
7.09
7.09
7.08
7.08
7.07
7.07
7.07
7.06
7.06
7.06
6.84
6.80
6.80
6.78
6.78
3.99
3.90
1.57 HDO
0.01
-0.00
Br
MeO
MeO
O
S34
¹H NMR, 500 MHz,
CDCl₃
0.94
1.00
1.01
3.07
2.02
1.00
3.12
3.15
12.0
11.0
10.0
9.0
8.0
7.0
6.0
5.0
4.0
3.0
2.0
1.0
0.0
-1

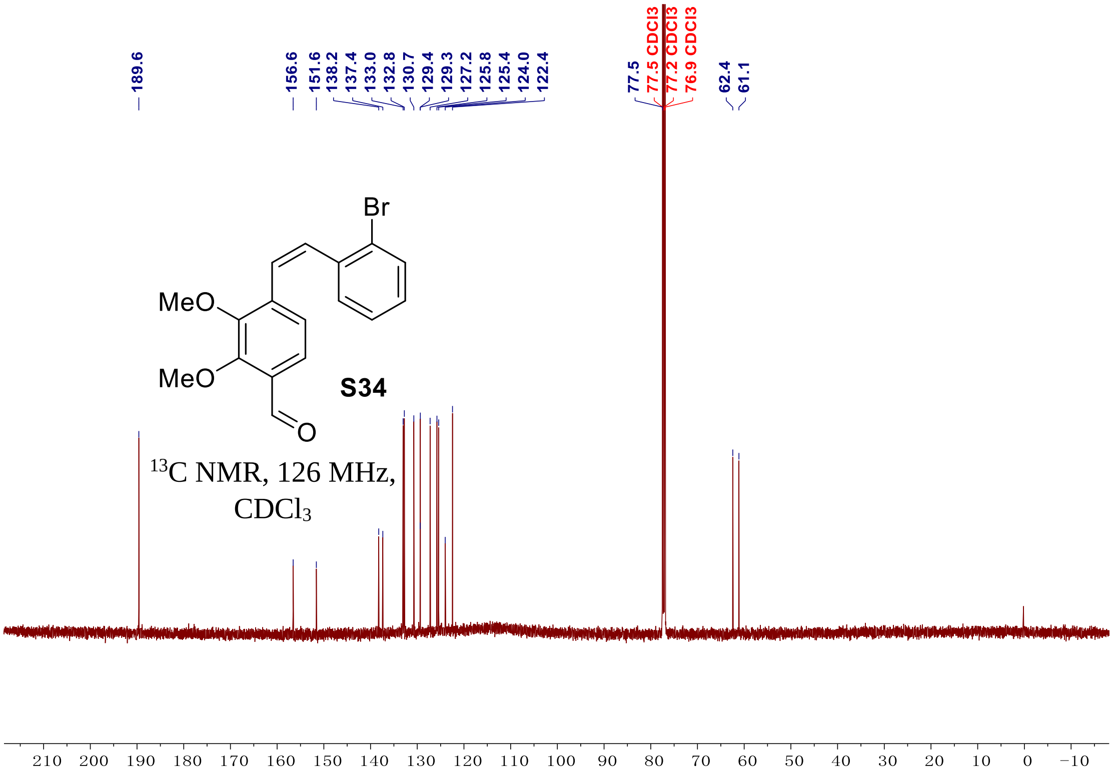
189.6
156.6
151.6
138.2
137.4
133.0
132.8
130.7
129.4
129.3
127.2
125.8
125.4
124.0
122.4
77.5
77.5 CDCl3
77.2 CDCl3
76.9 CDCl3
62.4
61.1
Br
MeO
MeO
O
S34
¹³C NMR, 126 MHz,
CDCl₃
210
200
190
180
170
160
150
140
130
120
110
100
90
80
70
60
50
40
30
20
10
0
-10

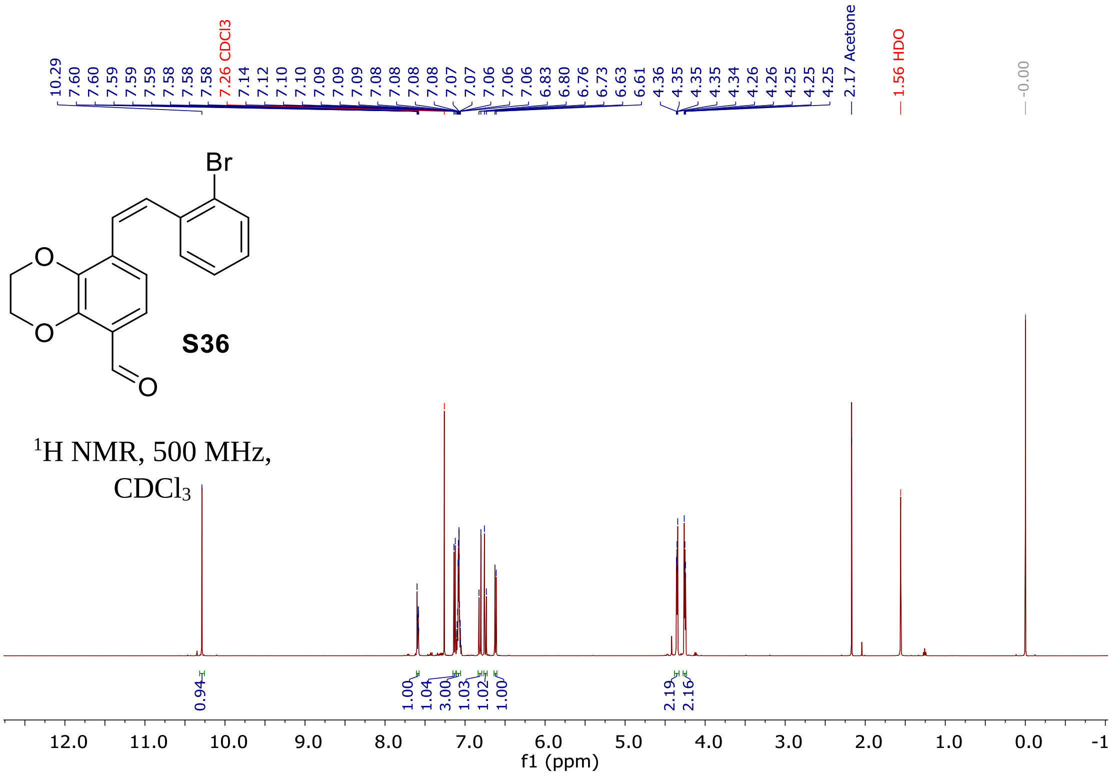
10.29
7.60
7.60
7.59
7.59
7.59
7.58
7.58
7.58
7.26 CDCl3
7.14
7.12
7.10
7.10
7.09
7.09
7.09
7.08
7.08
7.08
7.08
7.07
7.07
7.06
7.06
7.06
6.83
6.80
6.76
6.73
6.63
6.61
4.36
4.35
4.35
4.35
4.34
4.26
4.26
4.25
4.25
4.25
2.17 Acetone
1.56 HDO
-0.00
Br
O
O
O
S36
1H NMR, 500 MHz,
CDCl3
0.94
1.00
1.04
3.00
1.03
1.02
1.00
2.19
2.16
12.0
11.0
10.0
9.0
8.0
7.0
6.0
5.0
4.0
3.0
2.0
1.0
0.0
-1
f1 (ppm)

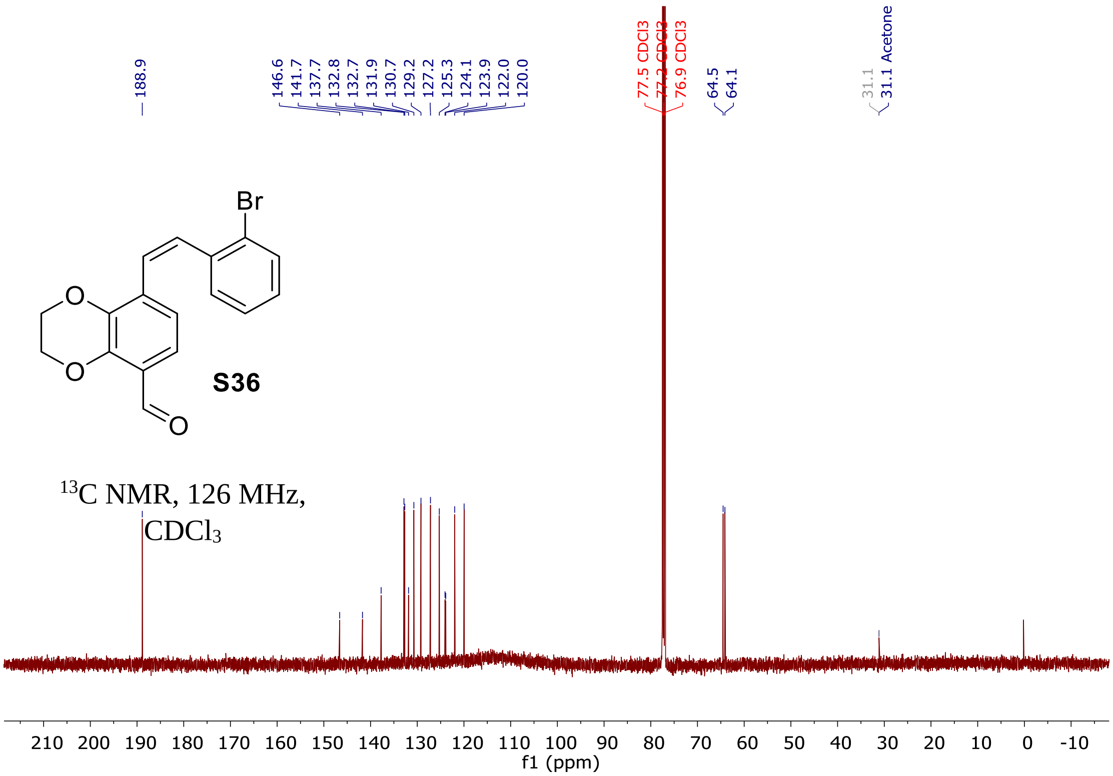
188.9
146.6
141.7
137.7
132.8
132.7
131.9
130.7
129.2
127.2
125.3
124.1
123.9
122.0
120.0
77.5 CDCl3
77.2 CDCl3
76.9 CDCl3
64.5
64.1
31.1
31.1 Acetone
Br
O
O
O
S36
13C NMR, 126 MHz,
CDCl3
210
200
190
180
170
160
150
140
130
120
110
100
90
80
70
60
50
40
30
20
10
0
-10
f1 (ppm)

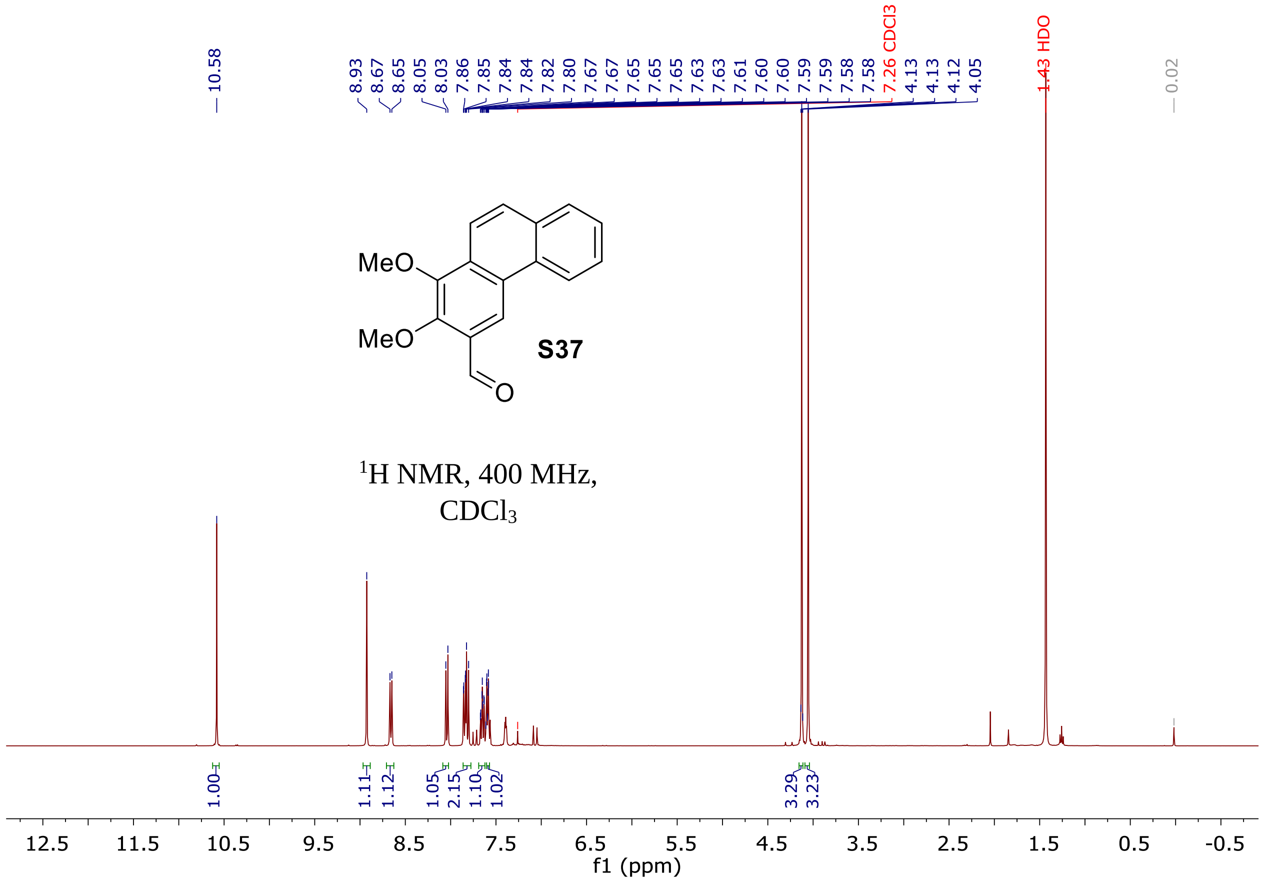
10.58
8.93
8.67
8.65
8.05
8.03
7.86
7.85
7.84
7.84
7.82
7.80
7.67
7.67
7.65
7.65
7.65
7.63
7.63
7.61
7.60
7.60
7.59
7.59
7.58
7.58
7.26 CDCl3
4.13
4.13
4.12
4.05
1.43 HDO
0.02
MeO
MeO
O
S37
^{1}H NMR, 400 MHz,
CDCl3
1.00
1.11
1.12
1.05
2.15
1.10
1.02
3.29
3.23
12.5
11.5
10.5
9.5
8.5
7.5
6.5
5.5
4.5
3.5
2.5
1.5
0.5
-0.5
f1 (ppm)

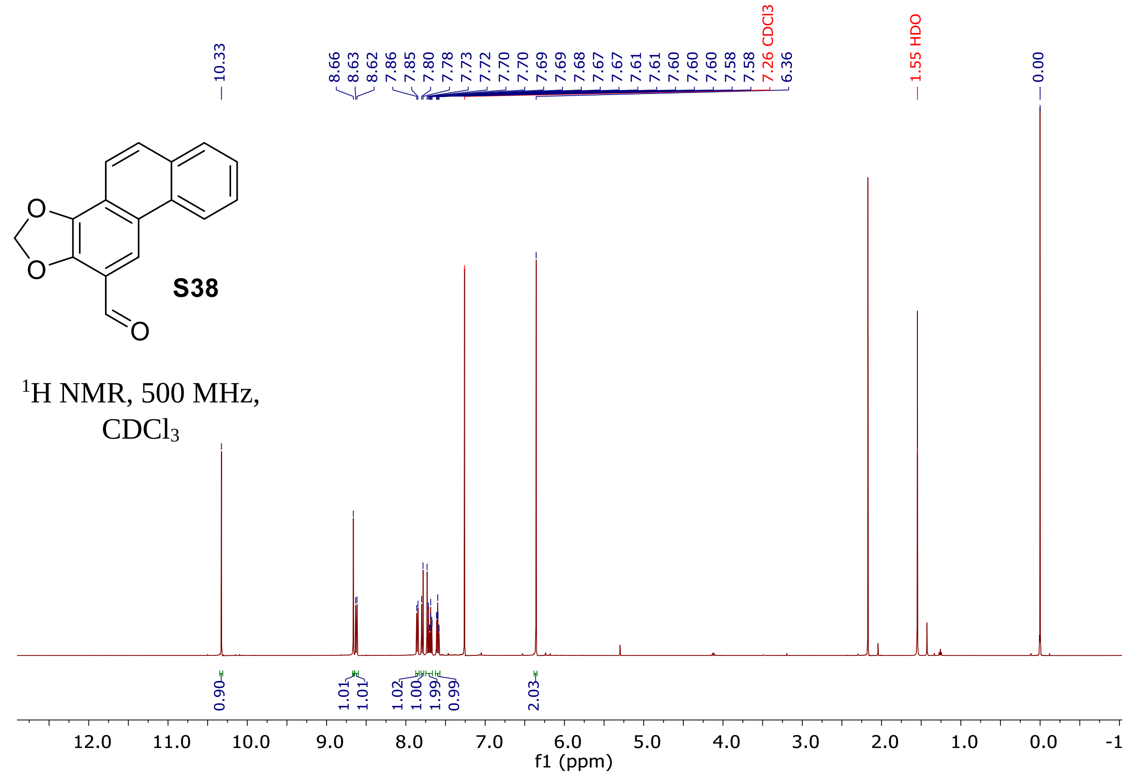

S38
^{1}H NMR, 500 MHz, $CDCl_3$
10.33
8.66
8.63
8.62
7.86
7.85
7.80
7.78
7.73
7.72
7.70
7.70
7.69
7.69
7.68
7.67
7.67
7.61
7.61
7.60
7.60
7.60
7.58
7.58
7.26 CDCl3
6.36
1.55 HDO
0.00
0.90
1.01
1.01
1.02
1.00
1.99
0.99
2.03
f1 (ppm)

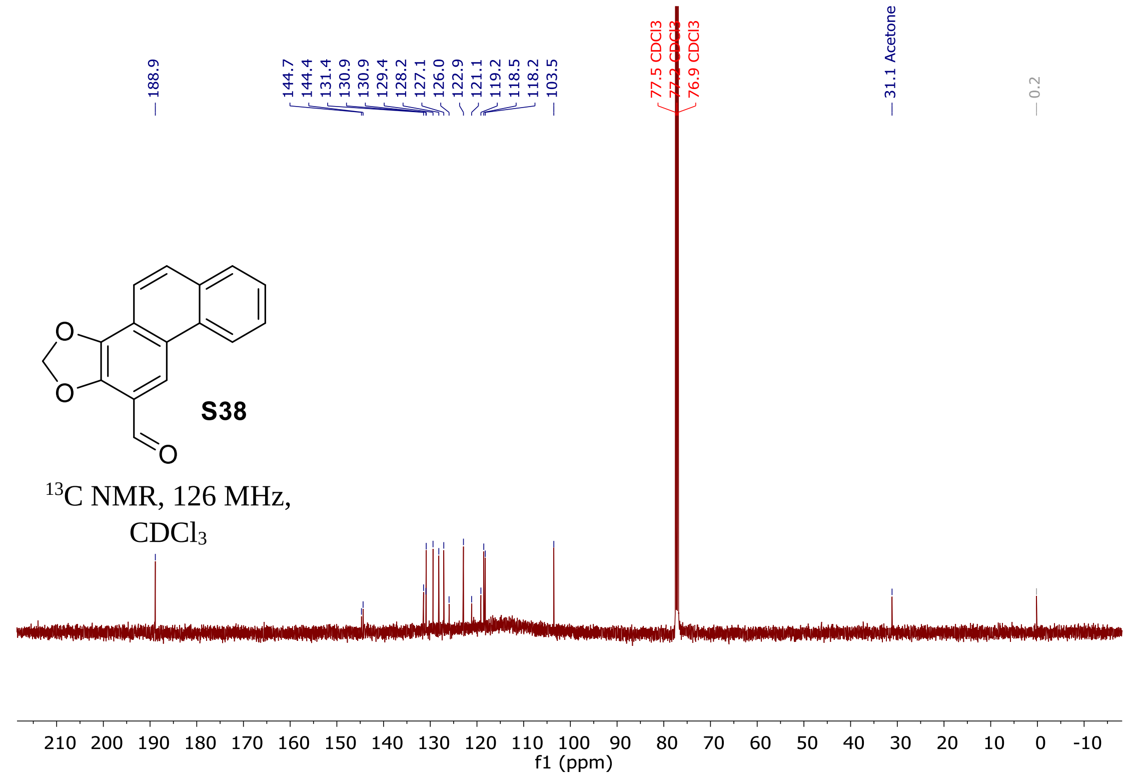

S38
^{13}C NMR, 126 MHz, $CDCl_3$
188.9
144.7
144.4
131.4
130.9
130.9
129.4
128.2
127.1
126.0
122.9
121.1
119.2
118.5
118.2
103.5
77.5 CDCl3
77.3 CDCl3
76.9 CDCl3
31.1 Acetone
0.2
f1 (ppm)

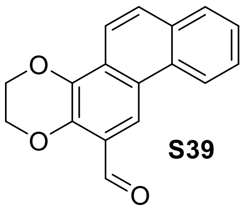

S39

^{1}H NMR, 500 MHz, $CDCl_3$

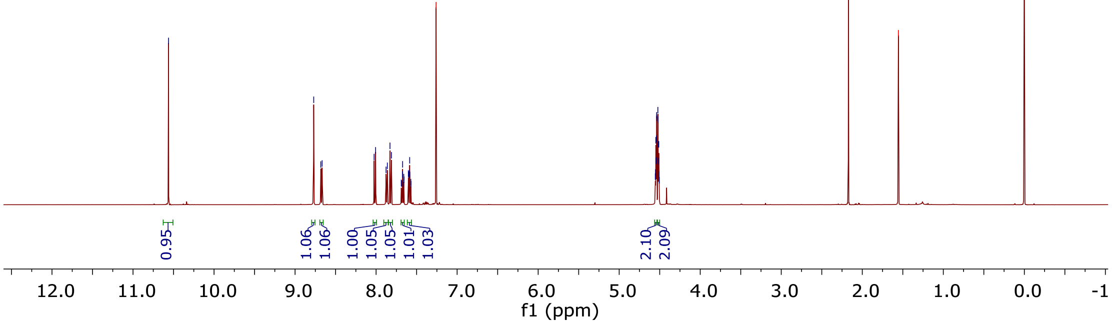

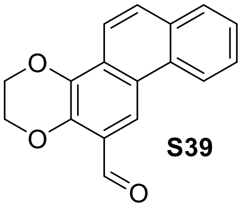

S39

^{13}C NMR, 126 MHz, $CDCl_3$

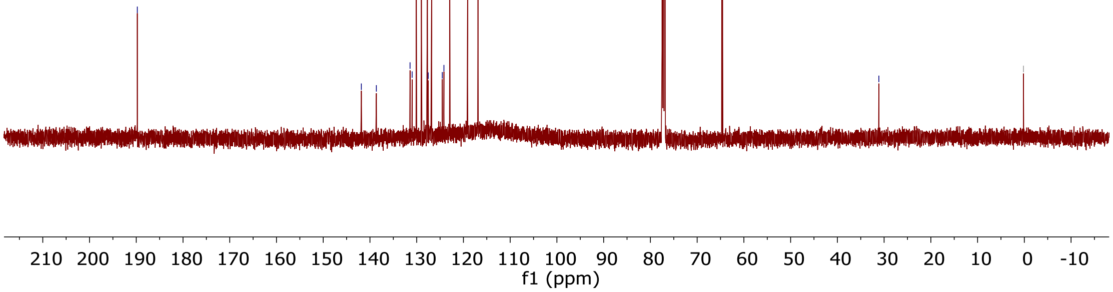

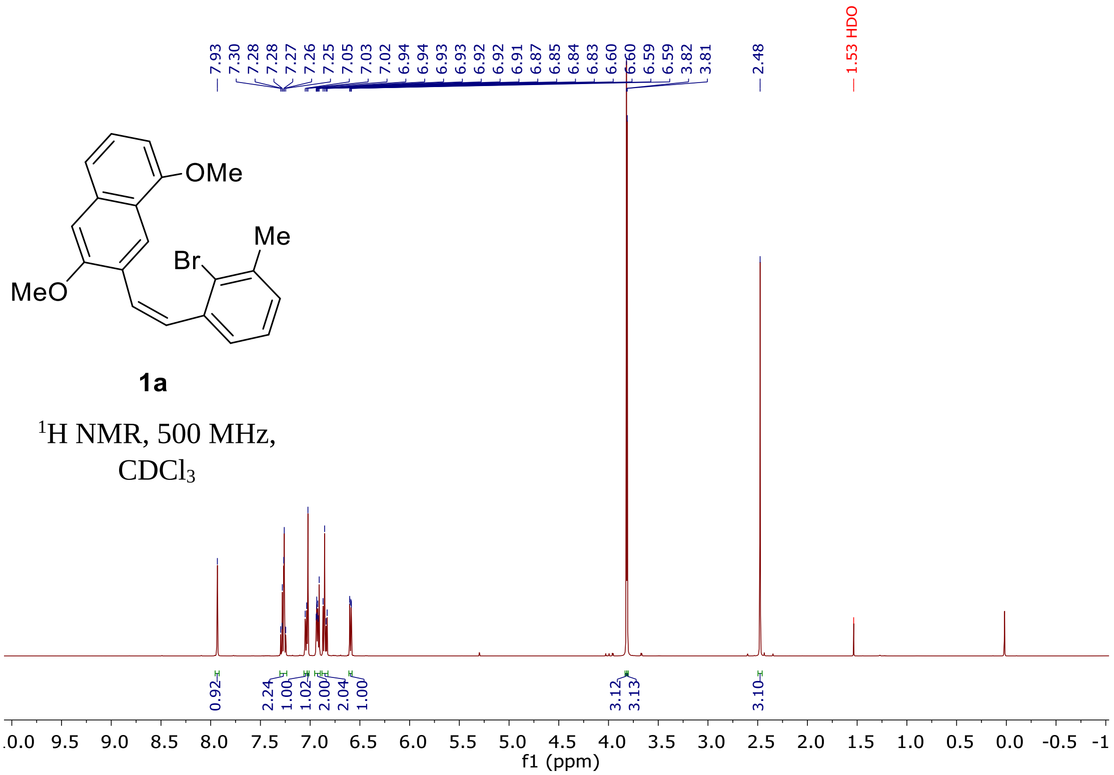
7.93 7.30 7.28 7.28 7.27 7.26 7.25 7.05 7.03 7.02 6.94 6.94 6.93 6.93 6.92 6.92 6.91 6.87 6.85 6.84 6.83 6.60 6.60 6.59 6.59 3.82 3.81
2.48
1.53 HDO
OMe
Me
Br
MeO
1a
^{1}H NMR, 500 MHz, $CDCl_3$
0.92 2.24 1.00 1.02 2.00 2.04 1.00 3.12 3.13 3.10
f1 (ppm)

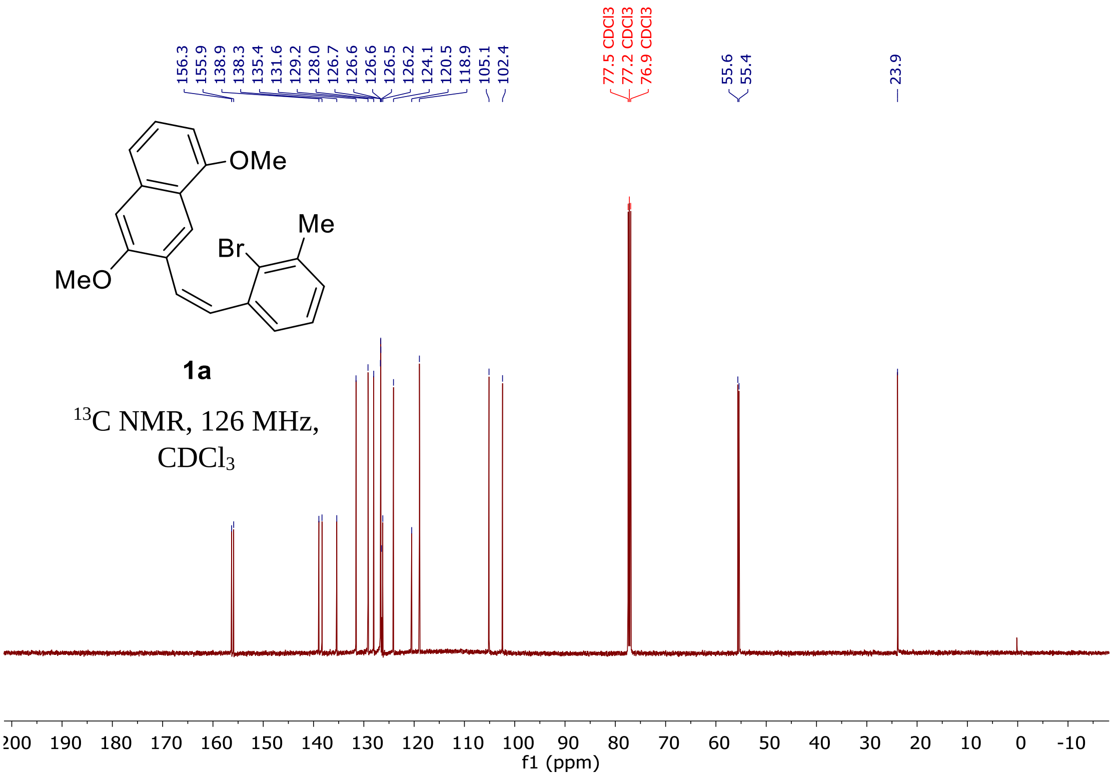
156.3 155.9 138.9 138.3 135.4 131.6 129.2 128.0 126.7 126.6 126.6 126.5 126.2 124.1 120.5 118.9 105.1 102.4
77.5 CDCl3 77.2 CDCl3 76.9 CDCl3
55.6 55.4
23.9
OMe
Me
Br
MeO
1a
^{13}C NMR, 126 MHz, $CDCl_3$
f1 (ppm)

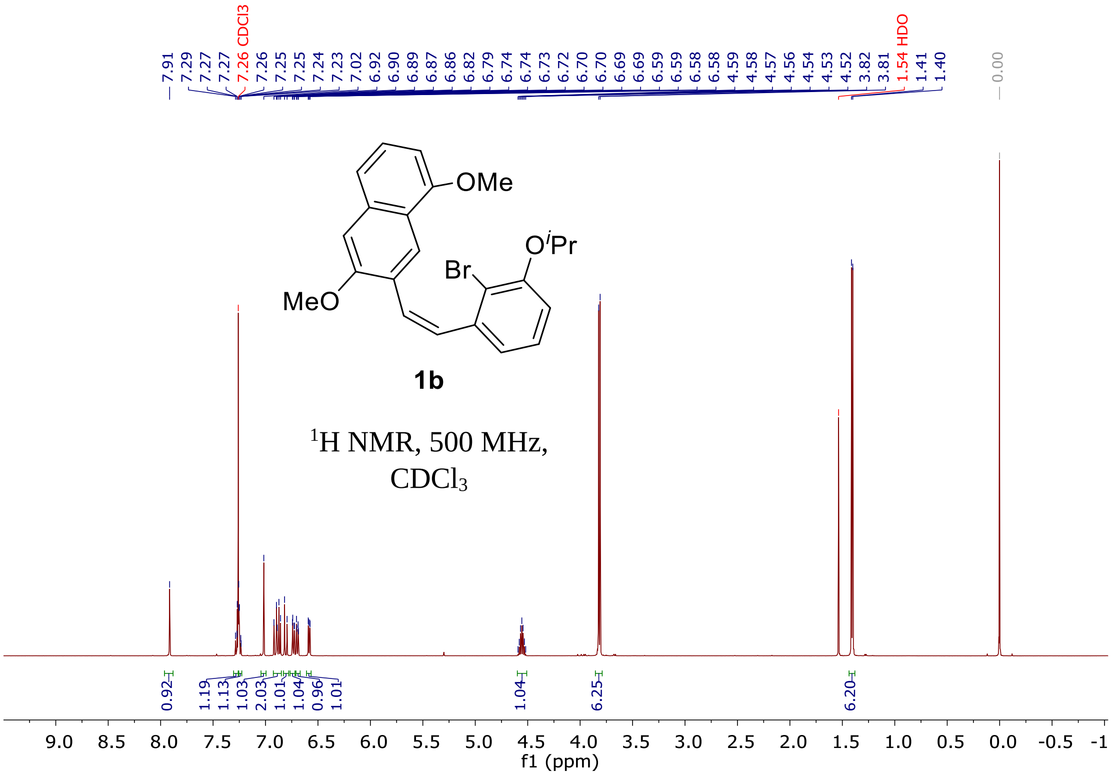
7.91
7.29
7.27
7.27
7.26 CDCl3
7.26
7.25
7.25
7.24
7.23
7.02
6.92
6.90
6.89
6.87
6.86
6.82
6.79
6.74
6.74
6.73
6.72
6.70
6.70
6.69
6.69
6.59
6.59
6.58
6.58
4.59
4.59
4.58
4.57
4.56
4.54
4.53
4.52
3.82
3.81
1.54 HDO
1.41
1.40
0.00
OMe
O^{i}Pr
Br
MeO
1b
^{1}H NMR, 500 MHz, $CDCl_3$
0.92
1.19
1.13
1.03
2.03
1.01
1.04
0.96
1.01
1.04
6.25
6.20
9.0 8.5 8.0 7.5 7.0 6.5 6.0 5.5 5.0 4.5 4.0 3.5 3.0 2.5 2.0 1.5 1.0 0.5 0.0 -0.5 -1
f1 (ppm)

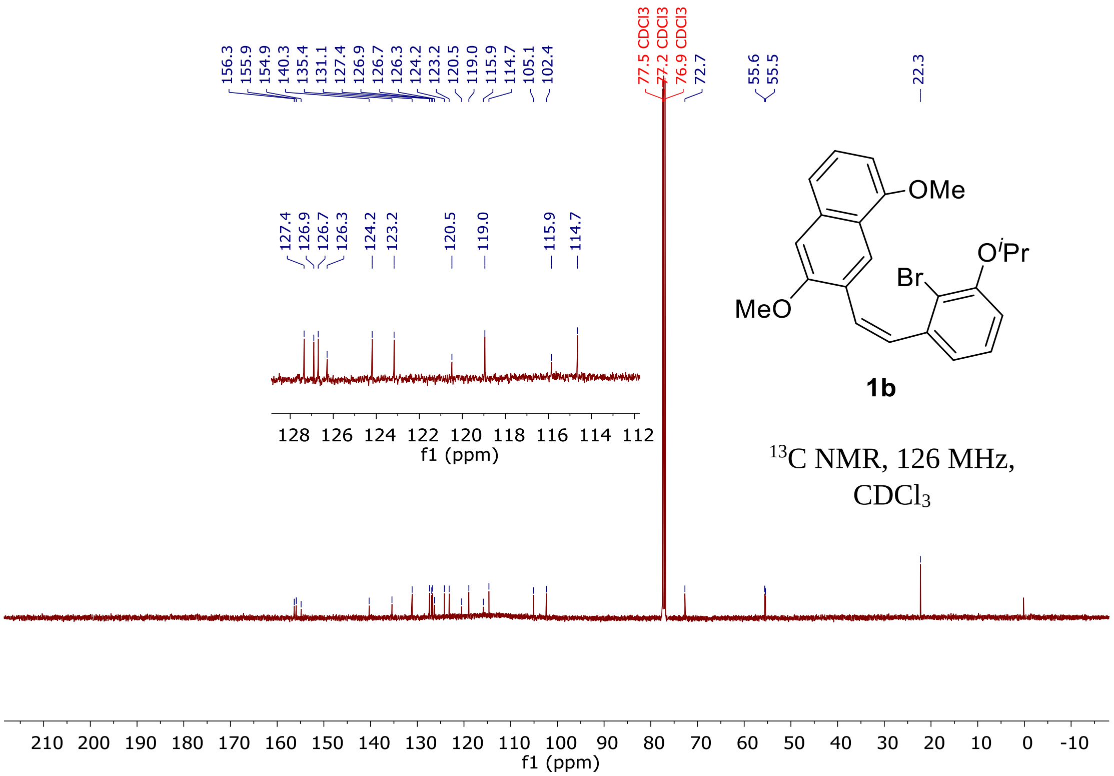
156.3
155.9
154.9
140.3
135.4
131.1
127.4
126.9
126.7
126.3
124.2
123.2
120.5
119.0
115.9
114.7
105.1
102.4
77.5 CDCl3
77.2 CDCl3
76.9 CDCl3
72.7
55.6
55.5
22.3
127.4
126.9
126.7
126.3
124.2
123.2
120.5
119.0
115.9
114.7
128 126 124 122 120 118 116 114 112
f1 (ppm)
OMe
O^{i}Pr
Br
MeO
1b
^{13}C NMR, 126 MHz, $CDCl_3$
210 200 190 180 170 160 150 140 130 120 110 100 90 80 70 60 50 40 30 20 10 0 -10
f1 (ppm)

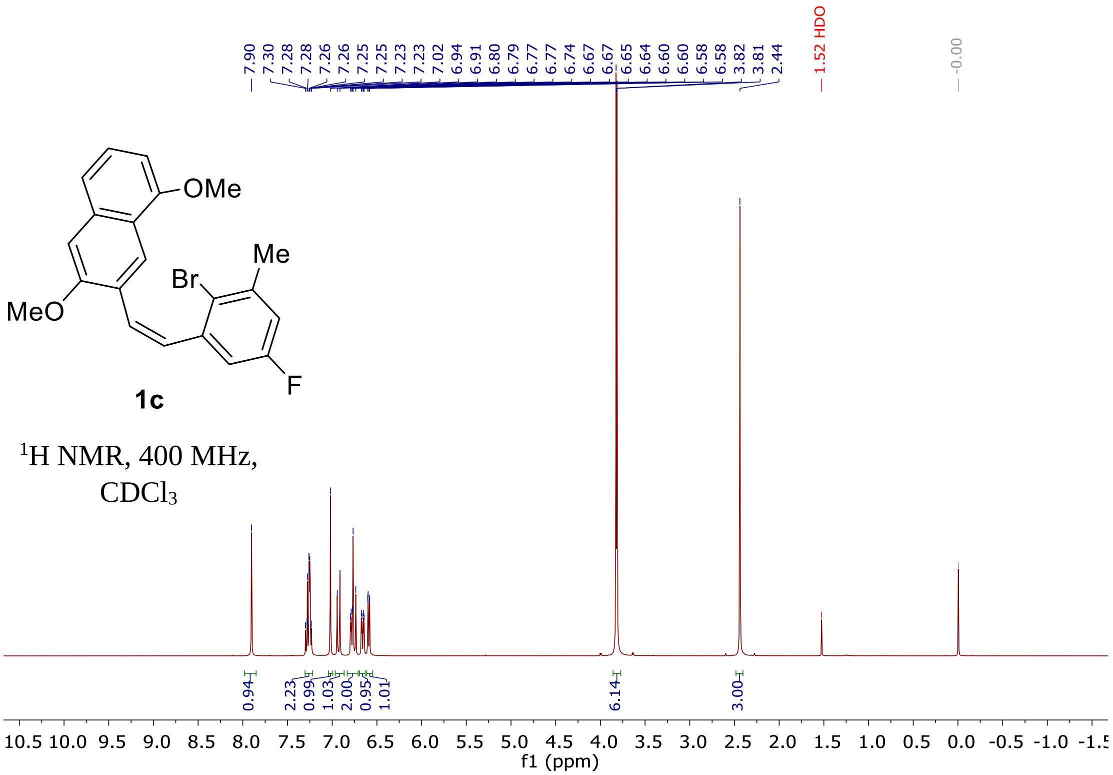
OMe
MeO
Br
Me
F
1c
^{1}H NMR, 400 MHz,
$CDCl_3$
7.90 7.30 7.28 7.28 7.26 7.26 7.25 7.25 7.23 7.23 7.23 7.02 6.94 6.91 6.80 6.79 6.77 6.77 6.74 6.67 6.67 6.65 6.64 6.60 6.60 6.58 6.58 3.82 3.81 2.44
1.52 HDO
-0.00
0.94 2.23 0.99 1.03 2.00 0.95 1.01 6.14 3.00
10.5 10.0 9.5 9.0 8.5 8.0 7.5 7.0 6.5 6.0 5.5 5.0 4.5 4.0 3.5 3.0 2.5 2.0 1.5 1.0 0.5 0.0 -0.5 -1.0 -1.5
f1 (ppm)

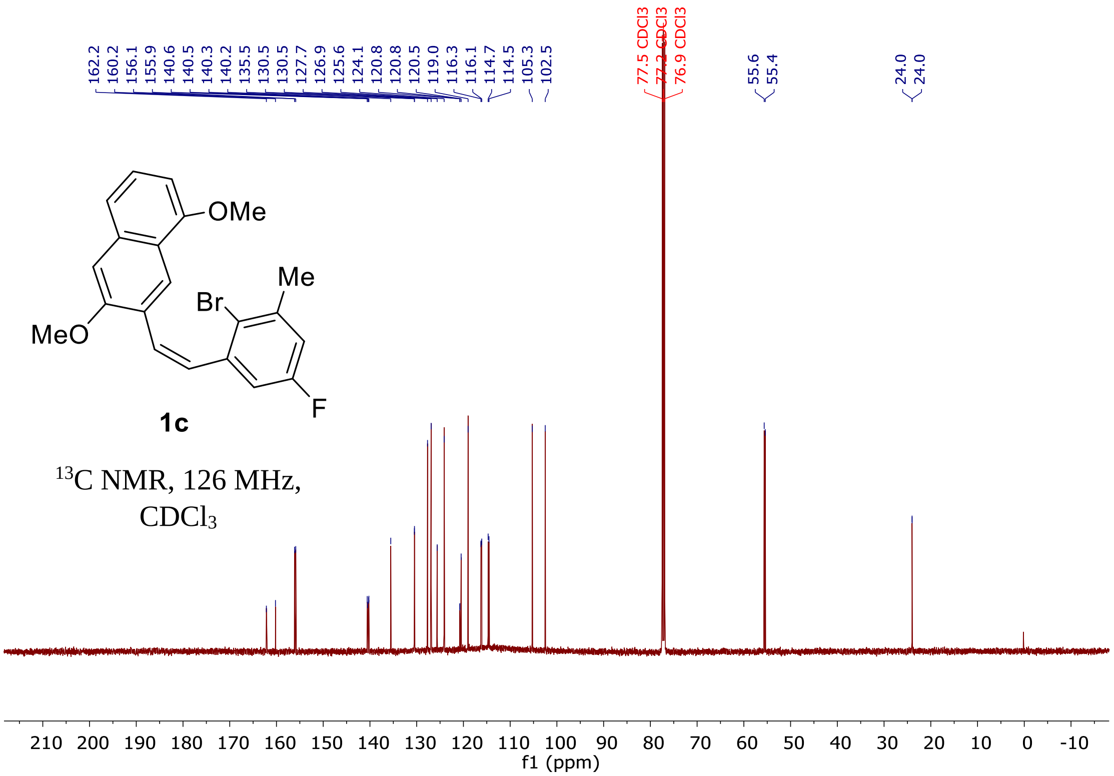
OMe
MeO
Br
Me
F
1c
^{13}C NMR, 126 MHz,
$CDCl_3$
162.2 160.2 156.1 155.9 140.6 140.5 140.3 140.2 135.5 130.5 130.5 127.7 126.9 125.6 124.1 120.8 120.8 120.5 119.0 116.3 116.1 114.7 114.5 105.3 102.5
77.5 CDCl3 77.2 CDCl3 76.9 CDCl3
55.6 55.4
24.0 24.0
210 200 190 180 170 160 150 140 130 120 110 100 90 80 70 60 50 40 30 20 10 0 -10
f1 (ppm)

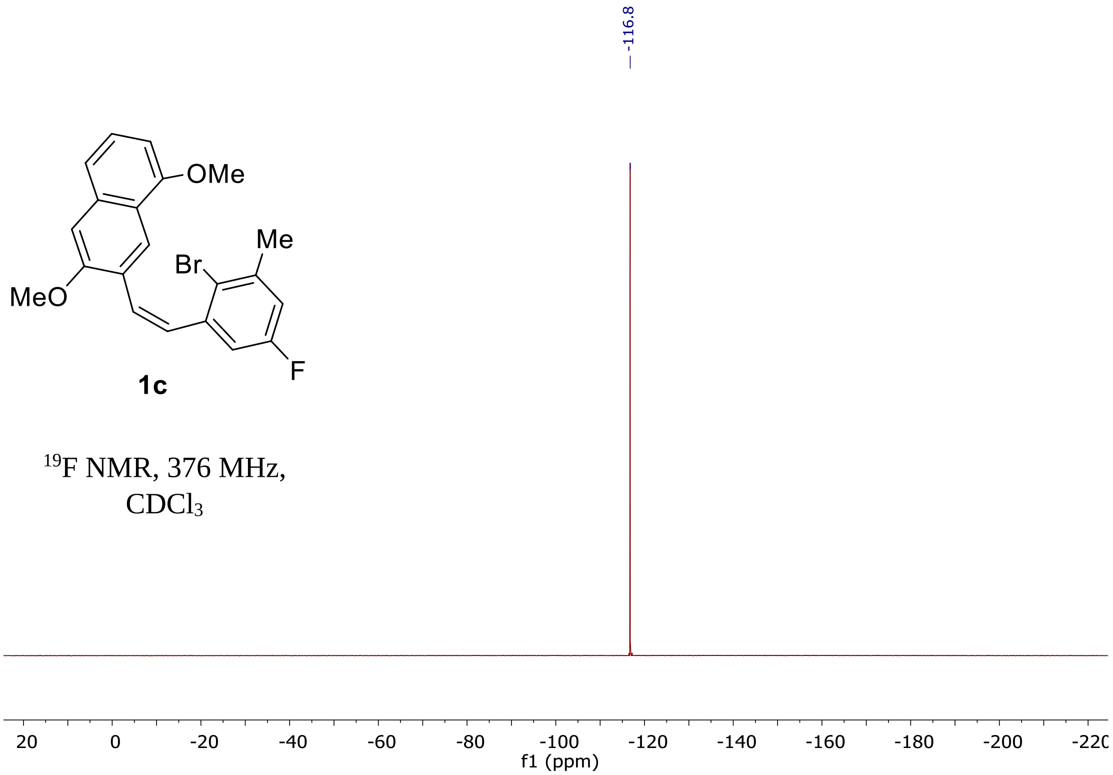
-116.8
OMe
Me
Br
MeO
F
1c
^{19}F NMR, 376 MHz,
$CDCl_3$
20
0
-20
-40
-60
-80
-100
-120
-140
-160
-180
-200
-220
f1 (ppm)

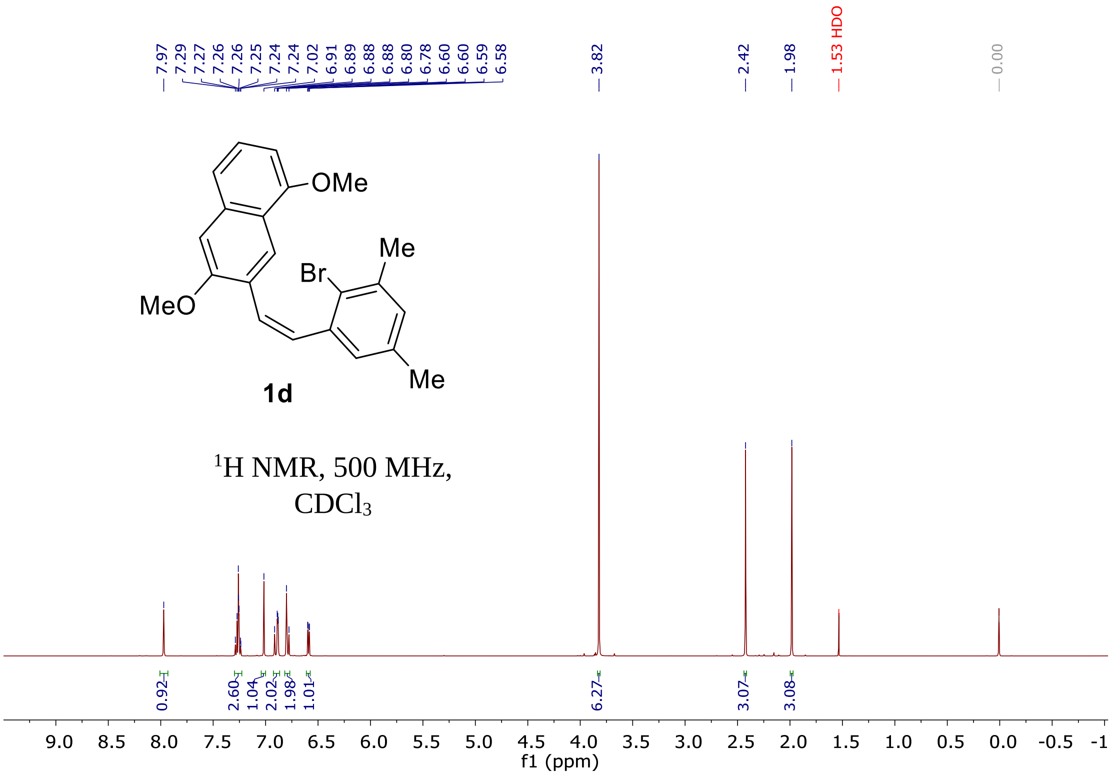
7.97
7.29
7.27
7.26
7.26
7.25
7.24
7.24
7.02
6.91
6.89
6.88
6.88
6.80
6.78
6.60
6.60
6.59
6.58
3.82
2.42
1.98
1.53 HDO
0.00
OMe
Me
Br
MeO
Me
1d
^{1}H NMR, 500 MHz, $CDCl_3$
0.92
2.60
1.04
2.02
1.98
1.01
6.27
3.07
3.08
f1 (ppm)

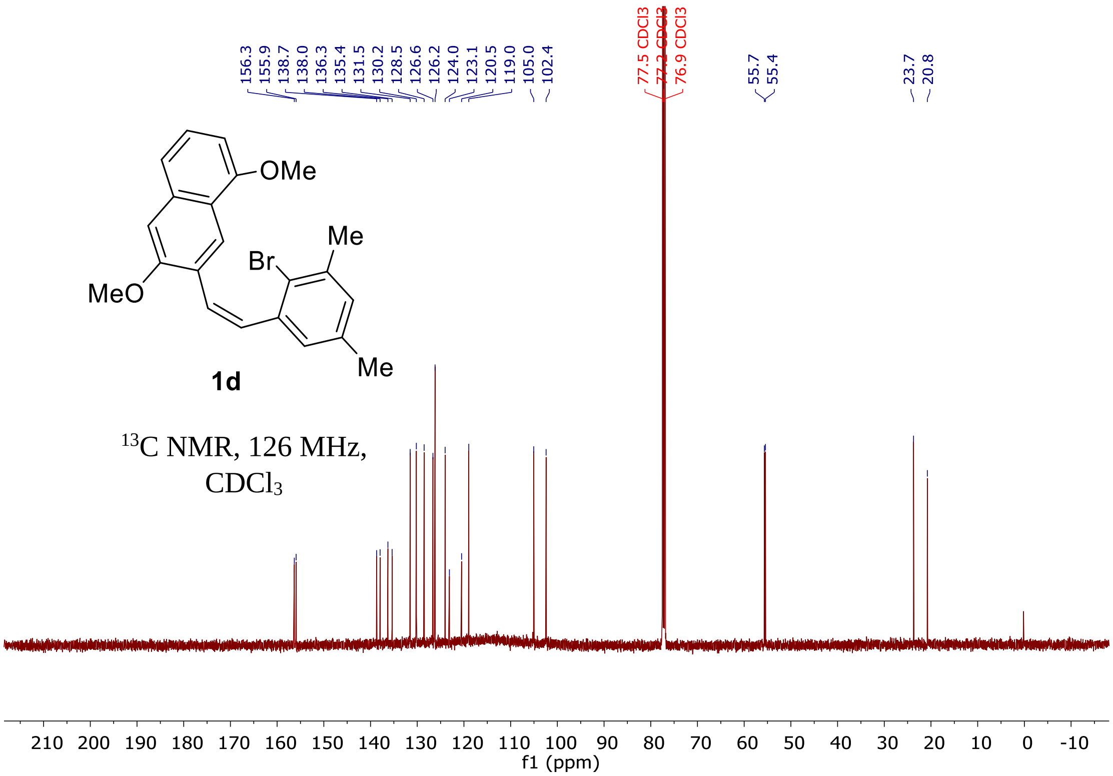
156.3
155.9
138.7
138.0
136.3
135.4
131.5
130.2
128.5
126.6
126.2
124.0
123.1
120.5
119.0
105.0
102.4
77.5 CDCl3
77.3 CDCl3
76.9 CDCl3
55.7
55.4
23.7
20.8
OMe
Me
Br
MeO
Me
1d
^{13}C NMR, 126 MHz, $CDCl_3$
f1 (ppm)

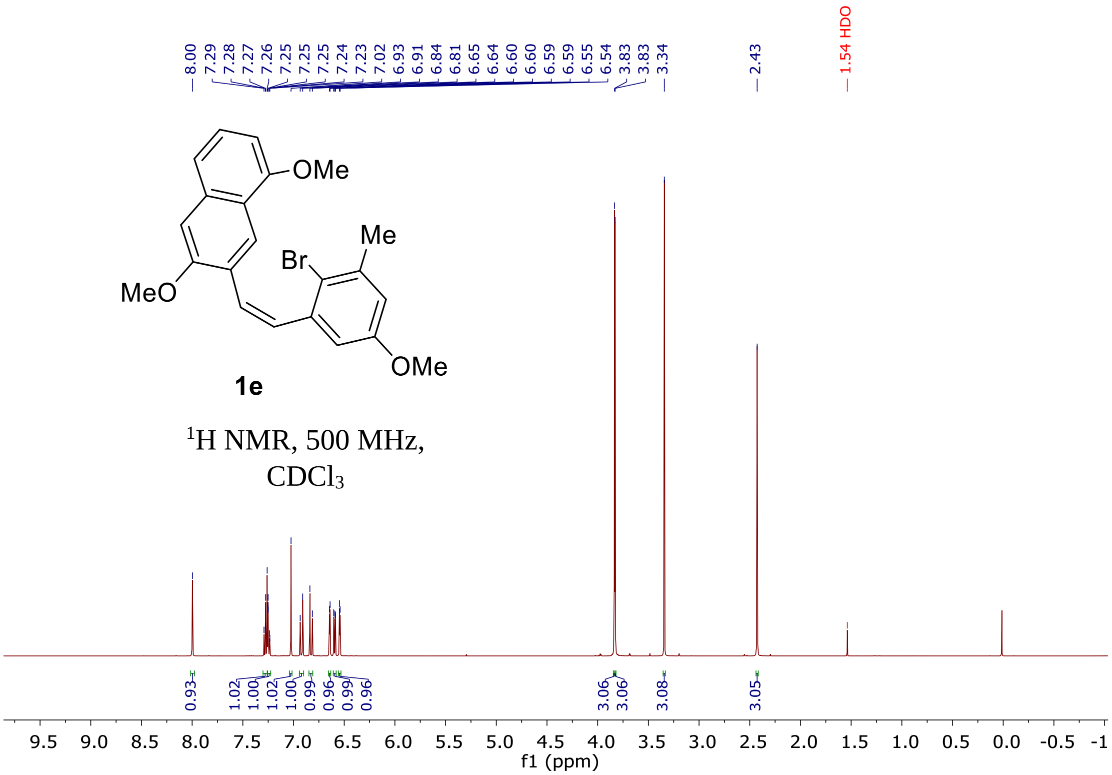
OMe
Me
Br
MeO
OMe
1e
^{1}H NMR, 500 MHz, CDCl$_3$
8.00
7.29
7.28
7.27
7.26
7.25
7.25
7.25
7.24
7.23
7.02
6.93
6.91
6.84
6.81
6.65
6.64
6.60
6.60
6.59
6.59
6.55
6.54
3.83
3.83
3.34
2.43
1.54 HDO
0.93
1.02
1.00
1.02
1.00
0.99
0.96
0.99
0.96
3.06
3.06
3.08
3.05
9.5 9.0 8.5 8.0 7.5 7.0 6.5 6.0 5.5 5.0 4.5 4.0 3.5 3.0 2.5 2.0 1.5 1.0 0.5 0.0 -0.5 -1
f1 (ppm)

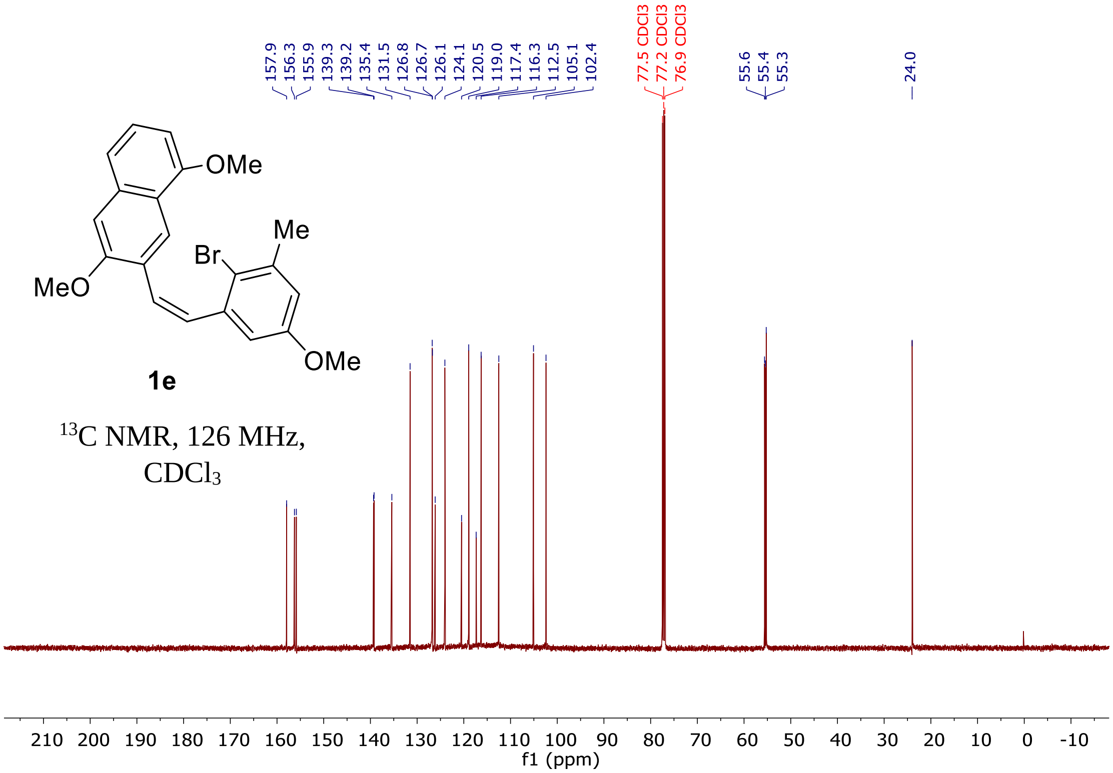
OMe
Me
Br
MeO
OMe
1e
^{13}C NMR, 126 MHz, CDCl$_3$
157.9
156.3
155.9
139.3
139.2
135.4
131.5
126.8
126.7
126.1
124.1
120.5
119.0
117.4
116.3
112.5
105.1
102.4
77.5 CDCl3
77.2 CDCl3
76.9 CDCl3
55.6
55.4
55.3
24.0
210 200 190 180 170 160 150 140 130 120 110 100 90 80 70 60 50 40 30 20 10 0 -10
f1 (ppm)

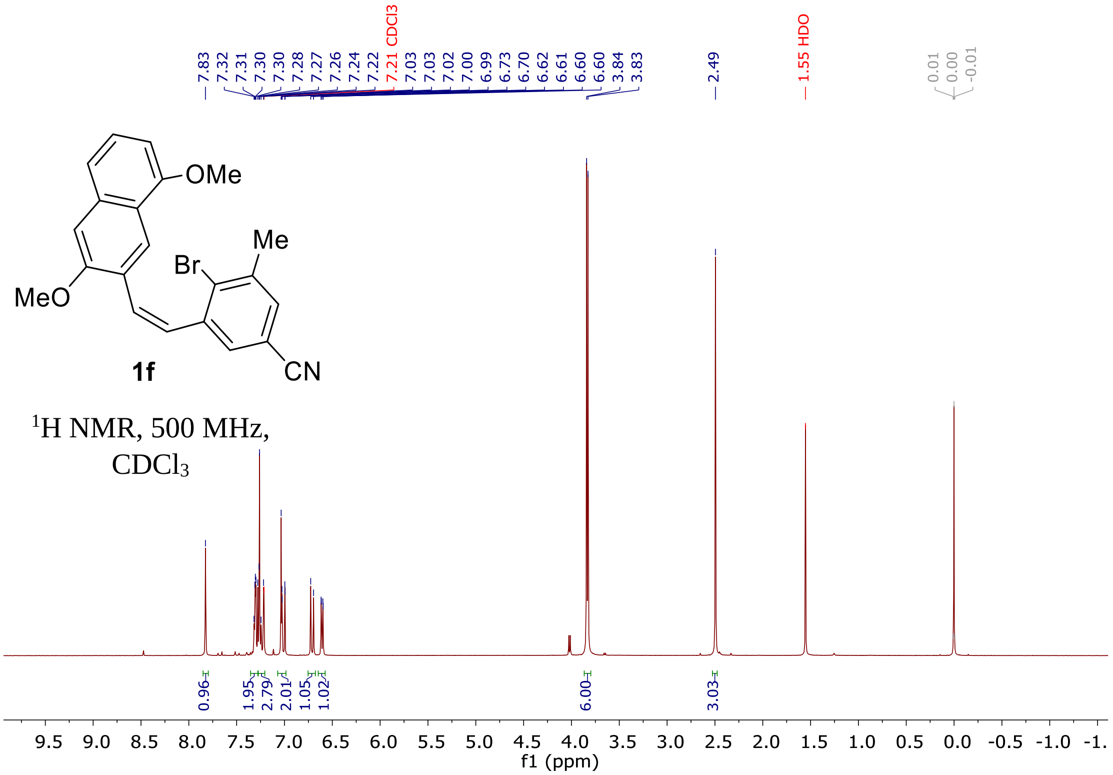
7.83
7.32
7.31
7.30
7.30
7.28
7.27
7.26
7.24
7.22
7.21 CDCl3
7.03
7.03
7.02
7.00
6.99
6.73
6.70
6.62
6.61
6.60
6.60
3.84
3.83
2.49
1.55 HDO
0.01
0.00
-0.01
OMe
Me
Br
MeO
1f
CN
1H NMR, 500 MHz,
CDCl3
0.96
1.95
2.79
2.01
1.05
1.02
6.00
3.03
f1 (ppm)

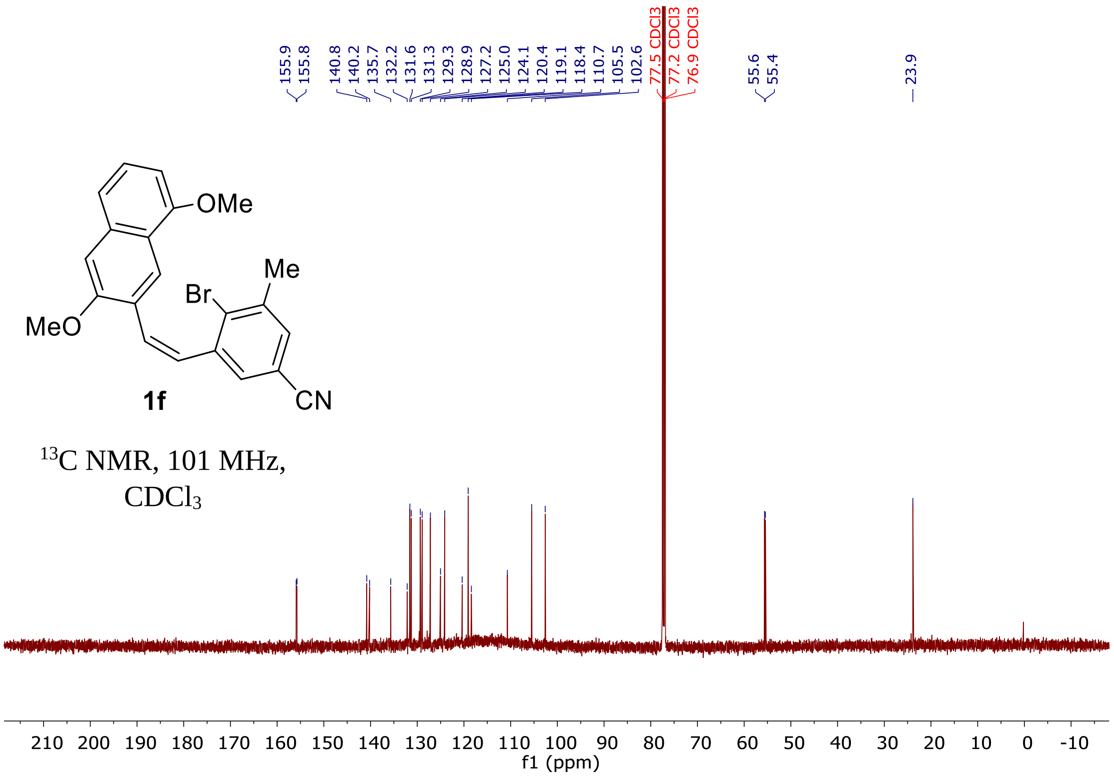
155.9
155.8
140.8
140.2
135.7
132.2
131.6
131.3
129.3
128.9
127.2
125.0
124.1
120.4
119.1
118.4
110.7
105.5
102.6
77.5 CDCl3
77.2 CDCl3
76.9 CDCl3
55.6
55.4
23.9
OMe
Me
Br
MeO
1f
CN
13C NMR, 101 MHz,
CDCl3
f1 (ppm)

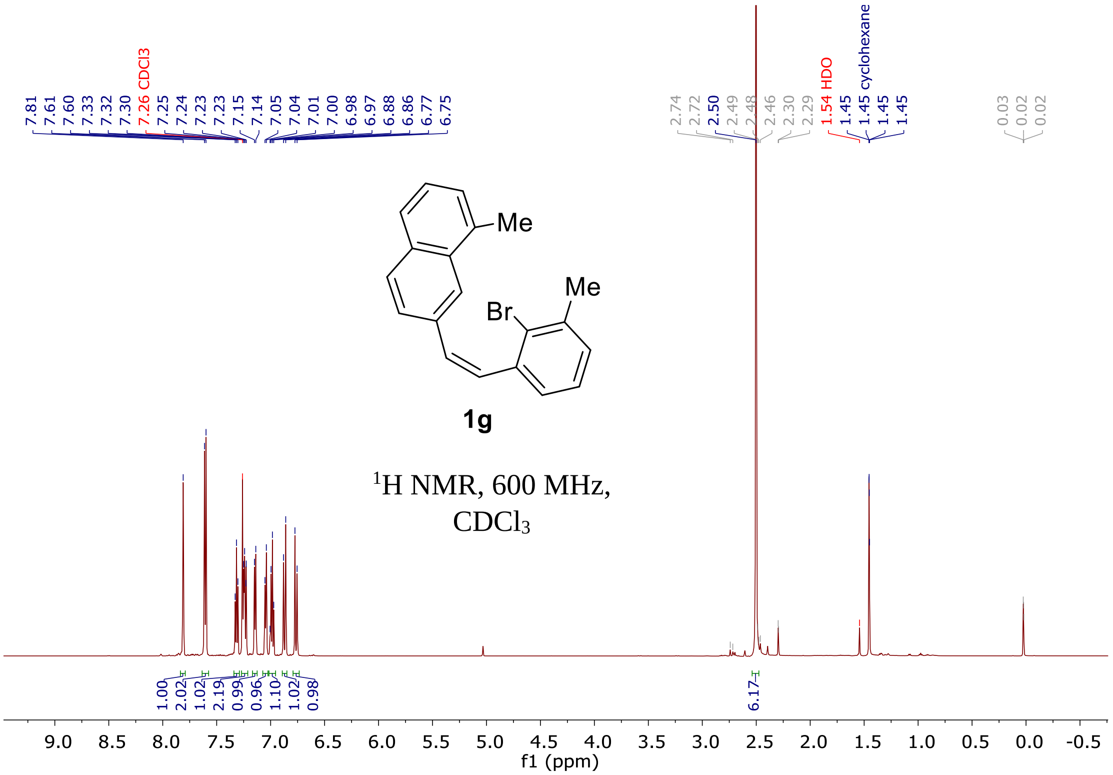
1g
¹H NMR, 600 MHz, CDCl3
f1 (ppm)

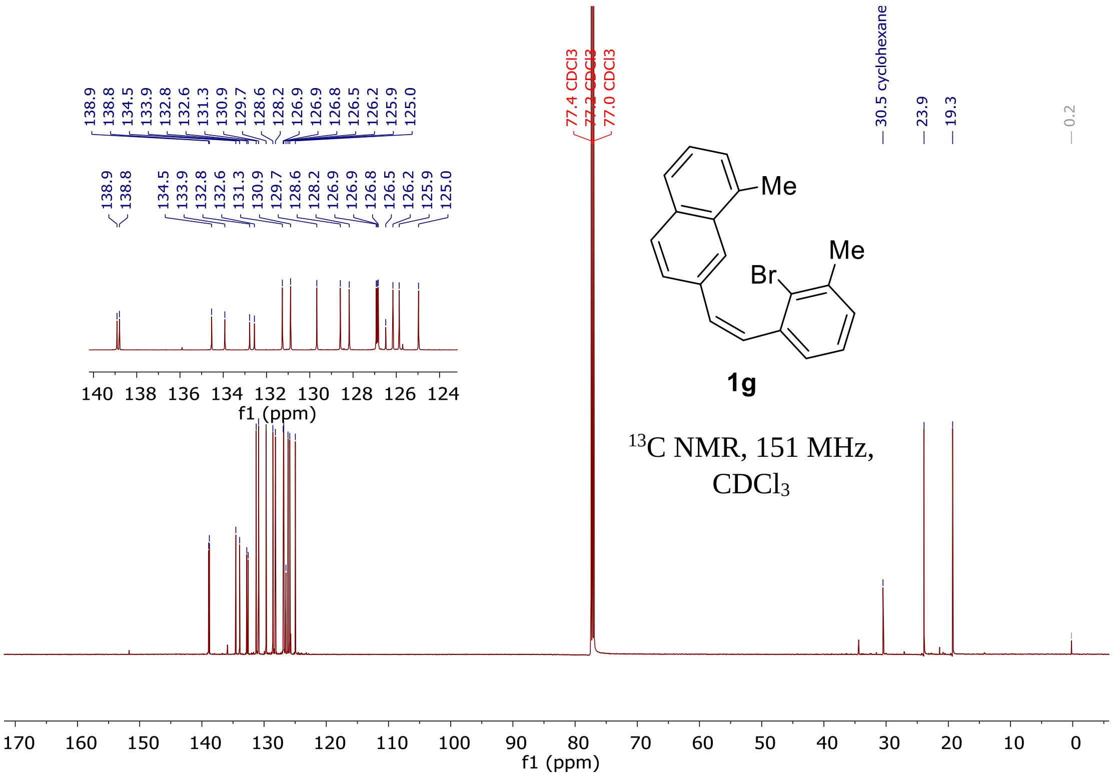
1g
¹³C NMR, 151 MHz, CDCl3
f1 (ppm)

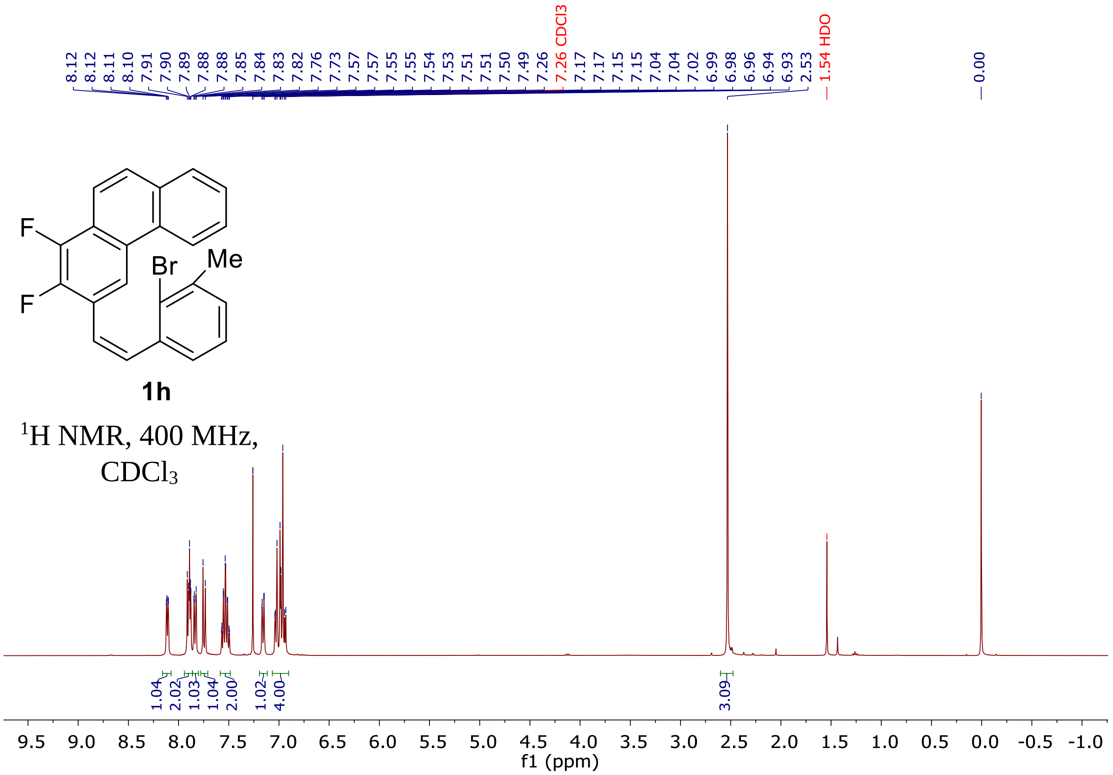
8.12 8.12 8.11 8.10 7.91 7.90 7.89 7.88 7.88 7.85 7.84 7.83 7.82 7.76 7.73 7.57 7.57 7.55 7.55 7.54 7.53 7.51 7.51 7.50 7.49 7.26 7.26 CDCl3 7.17 7.17 7.15 7.15 7.04 7.04 7.02 6.99 6.98 6.96 6.94 6.93 2.53 1.54 HDO 0.00
F
F
Br
Me
1h
^{1}H NMR, 400 MHz, $CDCl_3$
1.04 2.02 1.03 1.04 2.00 1.02 4.00 3.09
9.5 9.0 8.5 8.0 7.5 7.0 6.5 6.0 5.5 5.0 4.5 4.0 3.5 3.0 2.5 2.0 1.5 1.0 0.5 0.0 -0.5 -1.0
f1 (ppm)

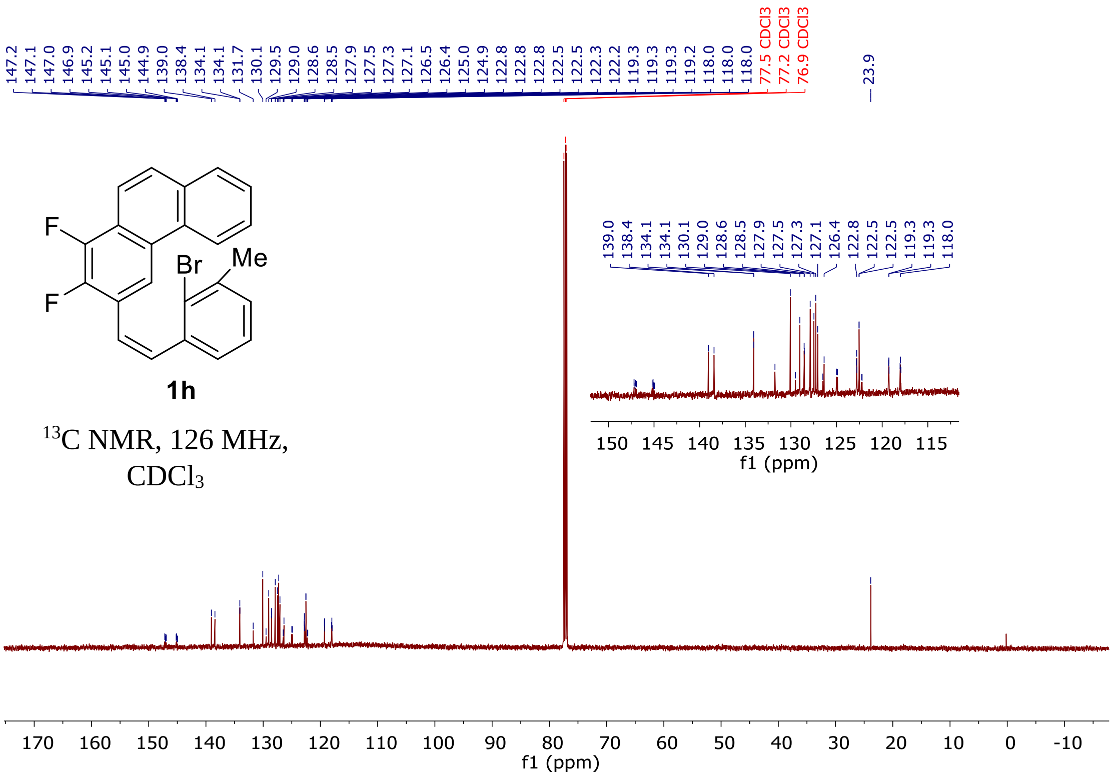
147.2 147.1 147.0 146.9 145.2 145.1 145.0 144.9 139.0 138.4 134.1 134.1 131.7 130.1 129.5 129.0 128.6 128.5 127.9 127.5 127.3 127.1 126.5 126.4 125.0 124.9 122.8 122.8 122.8 122.5 122.5 122.3 122.2 119.3 119.3 119.3 119.2 118.0 118.0 118.0 77.5 CDCl3 77.2 CDCl3 76.9 CDCl3 23.9
F
F
Br
Me
1h
^{13}C NMR, 126 MHz, $CDCl_3$
139.0 138.4 134.1 134.1 130.1 129.0 128.6 128.5 127.9 127.5 127.3 127.1 126.4 122.8 122.5 122.5 119.3 119.3 118.0
150 145 140 135 130 125 120 115
f1 (ppm)
170 160 150 140 130 120 110 100 90 80 70 60 50 40 30 20 10 0 -10
f1 (ppm)

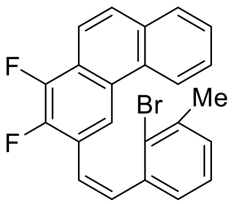

1h

^{19}F NMR, 376 MHz, $CDCl_3$

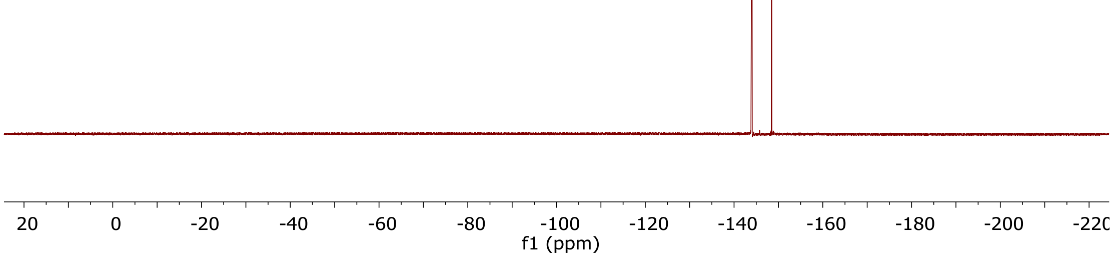

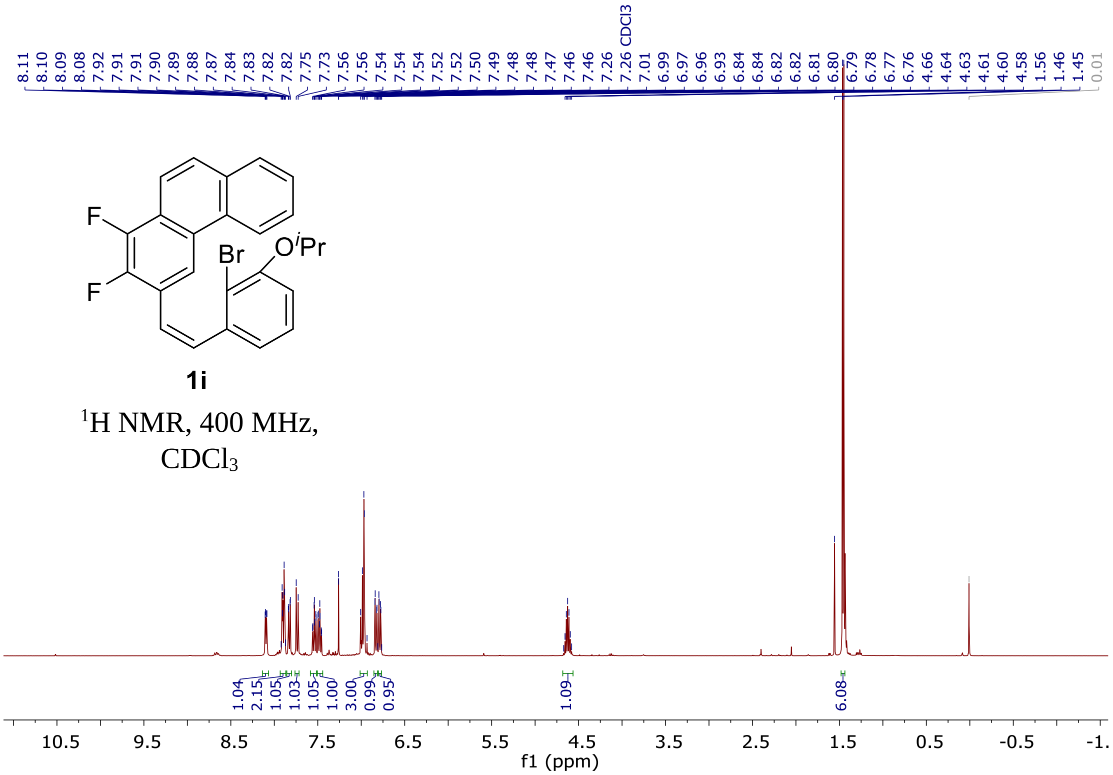
8.11 8.10 8.09 8.08 7.92 7.91 7.91 7.90 7.89 7.88 7.87 7.84 7.83 7.82 7.82 7.75 7.73 7.56 7.56 7.54 7.54 7.54 7.52 7.52 7.50 7.49 7.48 7.48 7.47 7.46 7.46 7.26 CDCl3 7.01 6.99 6.97 6.96 6.93 6.84 6.84 6.82 6.82 6.81 6.80 6.79 6.78 6.77 6.76 4.66 4.64 4.63 4.61 4.60 4.58 1.56 1.46 1.45 0.01
F
F
Br
O^{i}Pr
1i
^{1}H NMR, 400 MHz,
CDCl$_3$
1.04 2.15 1.05 1.03 1.05 1.00 3.00 0.99 0.95 1.09 6.08
10.5 9.5 8.5 7.5 6.5 5.5 4.5 3.5 2.5 1.5 0.5 -0.5 -1.
f1 (ppm)

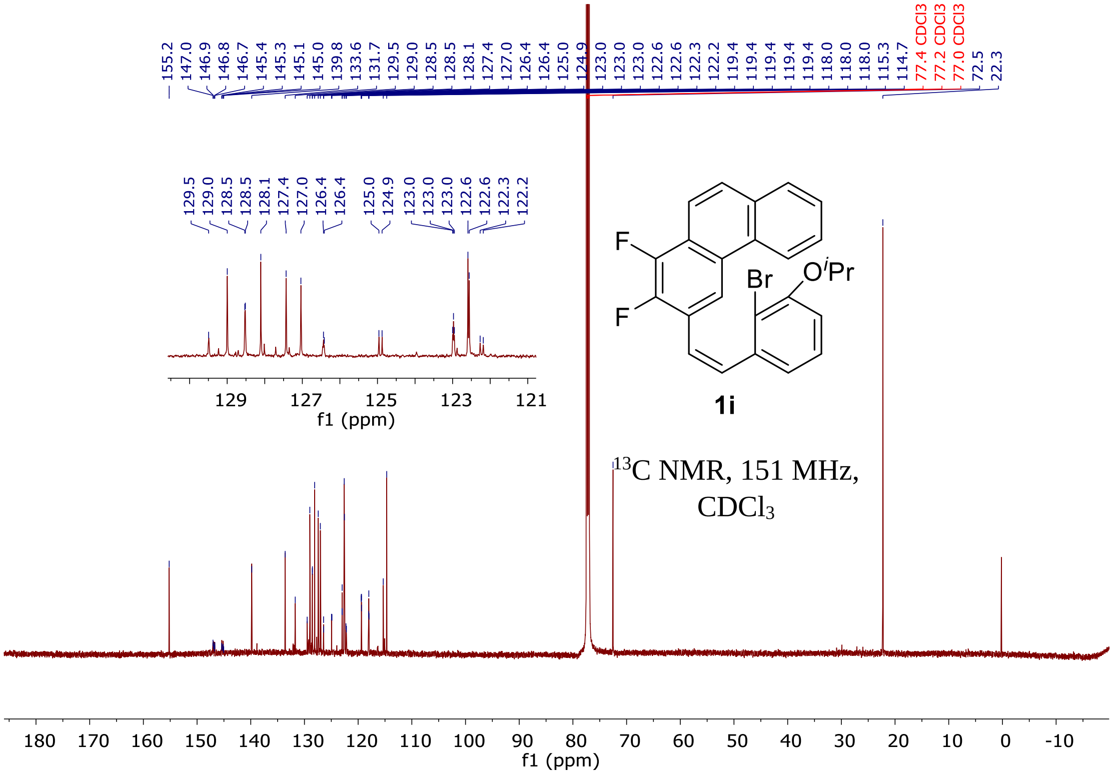
155.2 147.0 146.9 146.8 146.7 145.4 145.3 145.1 145.0 139.8 133.6 131.7 129.5 129.0 128.5 128.5 128.1 127.4 127.0 126.4 126.4 125.0 124.9 123.0 123.0 123.0 122.6 122.3 122.2 119.4 119.4 119.4 119.4 118.0 118.0 115.3 114.7 77.4 CDCl3 77.2 CDCl3 77.0 CDCl3 72.5 22.3
129.5 129.0 128.5 128.5 128.1 127.4 127.0 126.4 126.4 125.0 124.9 123.0 123.0 123.0 122.6 122.6 122.3 122.2
129 127 125 123 121
f1 (ppm)
F
F
Br
O^{i}Pr
1i
^{13}C NMR, 151 MHz,
CDCl$_3$
180 170 160 150 140 130 120 110 100 90 80 70 60 50 40 30 20 10 0 -10
f1 (ppm)

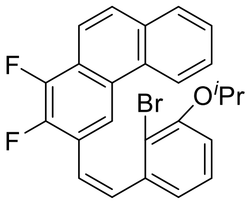

1i

^{19}F NMR, 376 MHz, $CDCl_3$

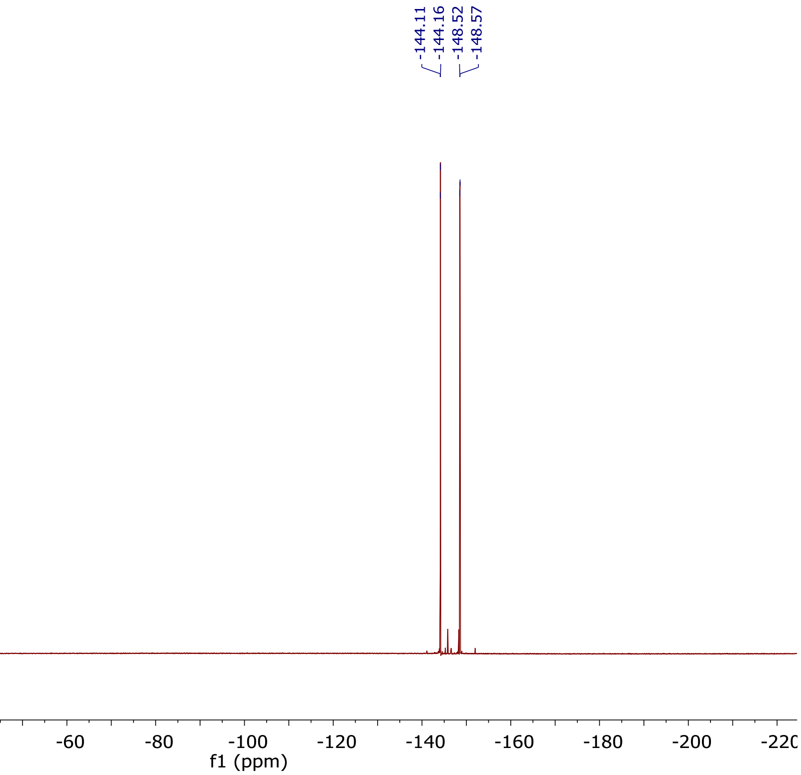

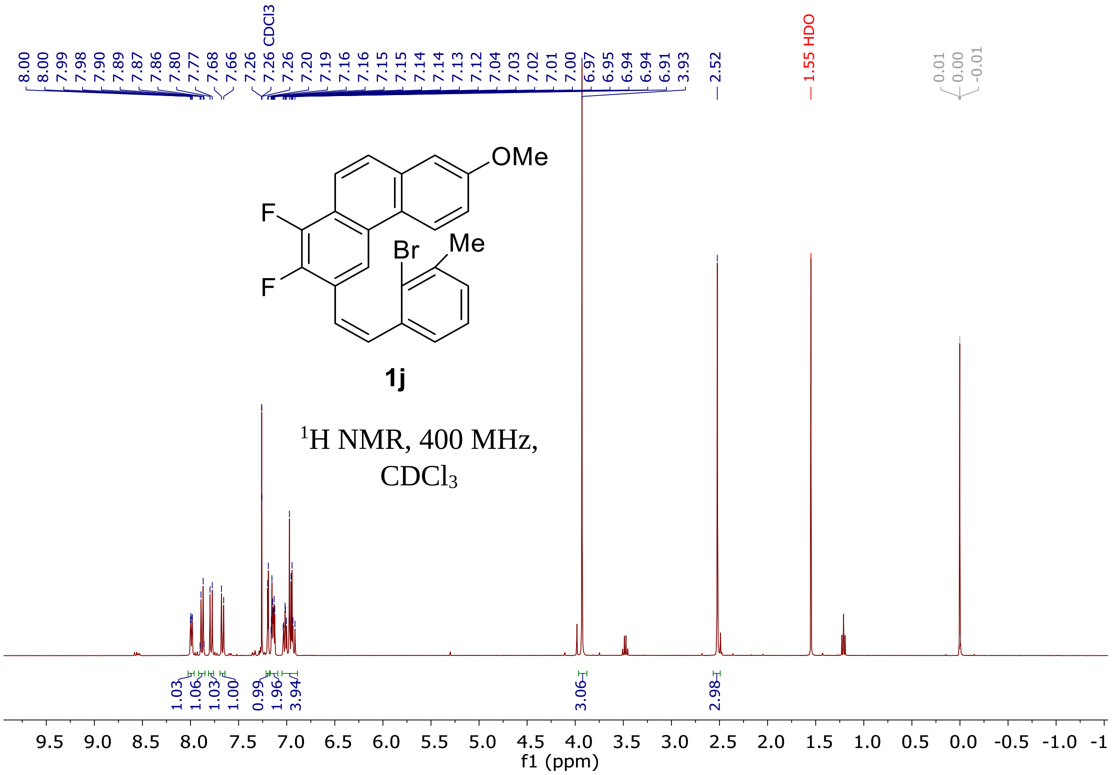

8.00
8.00
7.99
7.98
7.90
7.89
7.87
7.86
7.80
7.77
7.68
7.66
7.26
7.26 CDCl3
7.26
7.20
7.19
7.16
7.16
7.15
7.15
7.14
7.14
7.13
7.12
7.04
7.03
7.02
7.01
7.00
6.97
6.95
6.94
6.94
6.91
3.93
2.52
1.55 HDO
0.01
0.00
-0.01
OMe
F
F
Br
Me
1j
¹H NMR, 400 MHz, CDCl₃
1.03
1.06
1.03
1.00
0.99
1.96
3.94
3.06
2.98
f1 (ppm)

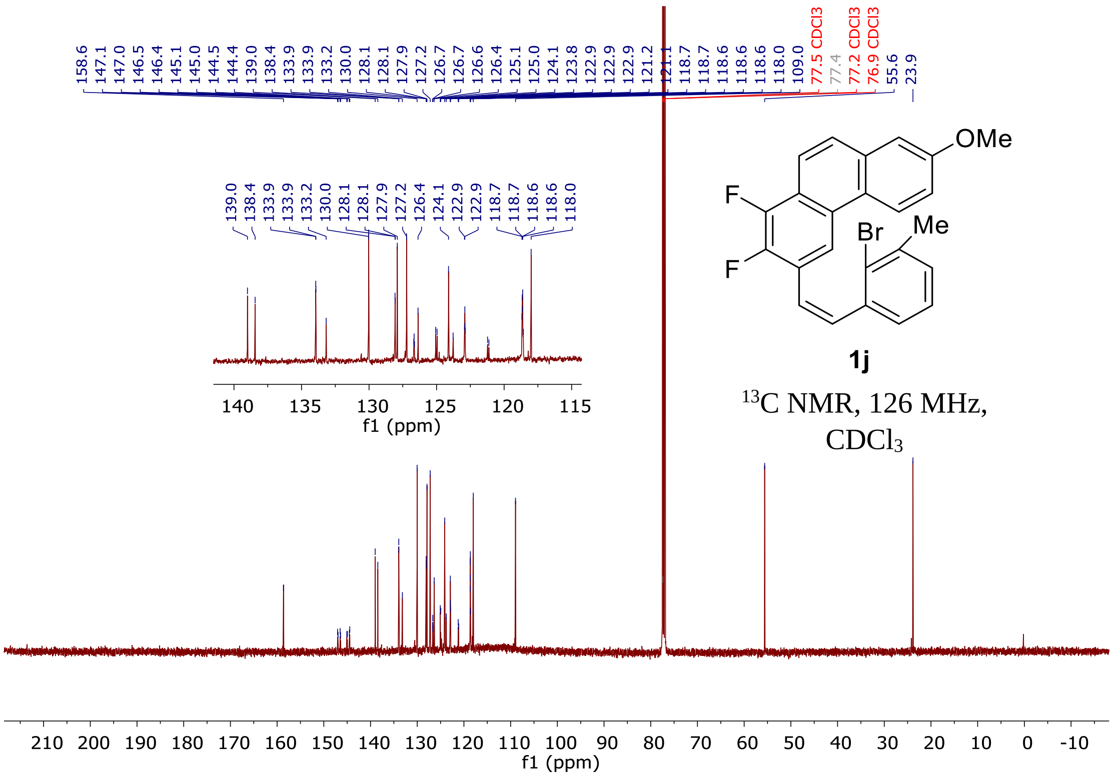

158.6
147.1
147.0
146.5
146.4
145.1
145.0
144.5
144.4
139.0
138.4
133.9
133.9
133.2
130.0
128.1
128.1
127.9
127.2
126.7
126.7
126.6
126.4
125.1
125.0
124.1
123.8
122.9
122.9
122.9
121.2
121.1
118.7
118.7
118.6
118.6
118.6
118.0
109.0
77.5 CDCl3
77.4
77.2 CDCl3
76.9 CDCl3
55.6
23.9
OMe
F
F
Br
Me
1j
¹³C NMR, 126 MHz, CDCl₃
f1 (ppm)

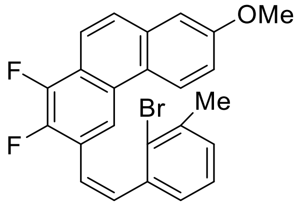

1j

^{19}F NMR, 376 MHz, $CDCl_3$

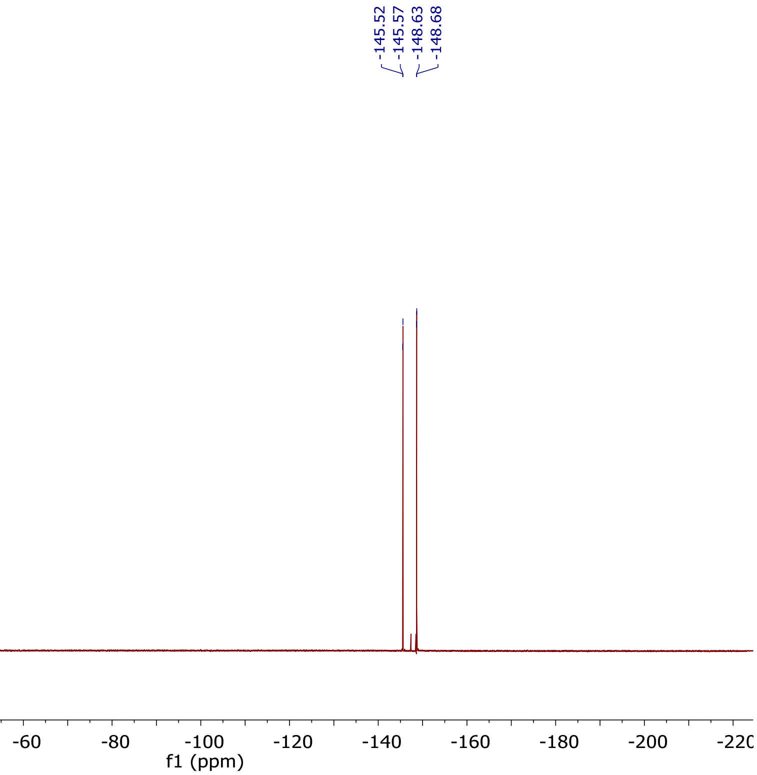

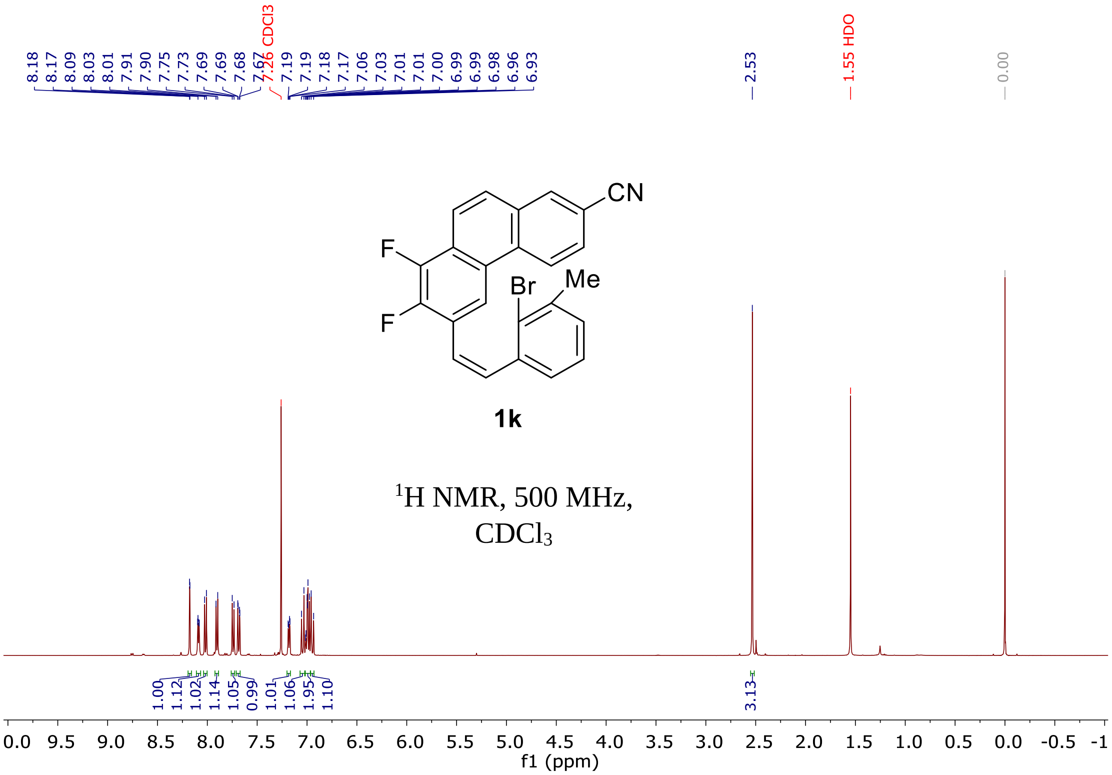
8.18 8.17 8.09 8.03 8.01 7.91 7.90 7.75 7.73 7.69 7.69 7.68 7.67 7.26 CDCl3 7.19 7.19 7.18 7.17 7.06 7.03 7.01 7.01 7.00 6.99 6.99 6.98 6.96 6.93
2.53
1.55 HDO
0.00
CN
F
F
Br
Me
1k
^{1}H NMR, 500 MHz, $CDCl_3$
1.00 1.12 1.02 1.14 1.05 0.99 1.01 1.06 1.95 1.10
3.13
f1 (ppm)

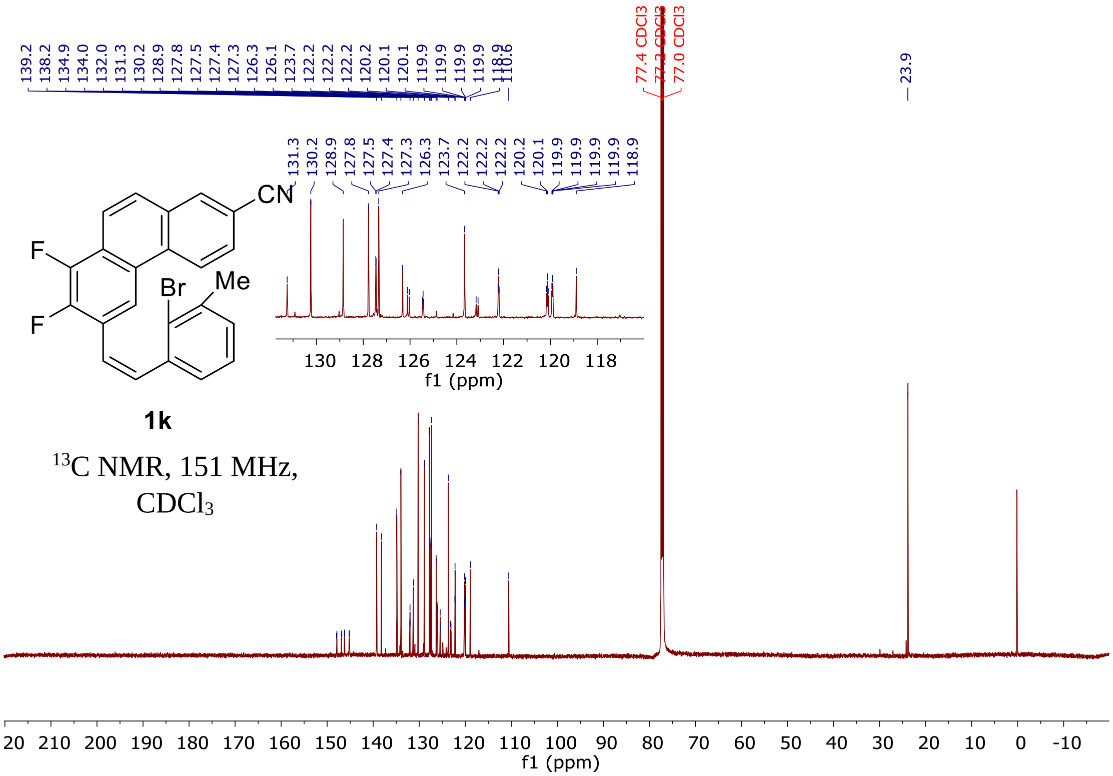
139.2 138.2 134.9 134.0 132.0 131.3 130.2 128.9 127.8 127.5 127.4 127.3 126.3 126.1 123.7 122.2 122.2 122.2 120.2 120.1 120.1 119.9 119.9 119.9 119.9 118.9 110.6
77.4 CDCl3 77.2 CDCl3 77.0 CDCl3
23.9
131.3 130.2 128.9 127.8 127.5 127.4 127.3 126.3 123.7 122.2 122.2 122.2 120.2 120.1 119.9 119.9 119.9 119.9 118.9
CN
F
F
Br
Me
1k
^{13}C NMR, 151 MHz, $CDCl_3$
f1 (ppm)

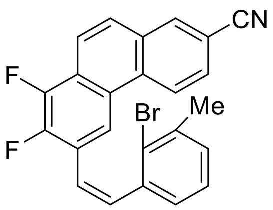

1k

^{19}F NMR, 376 MHz, $CDCl_3$

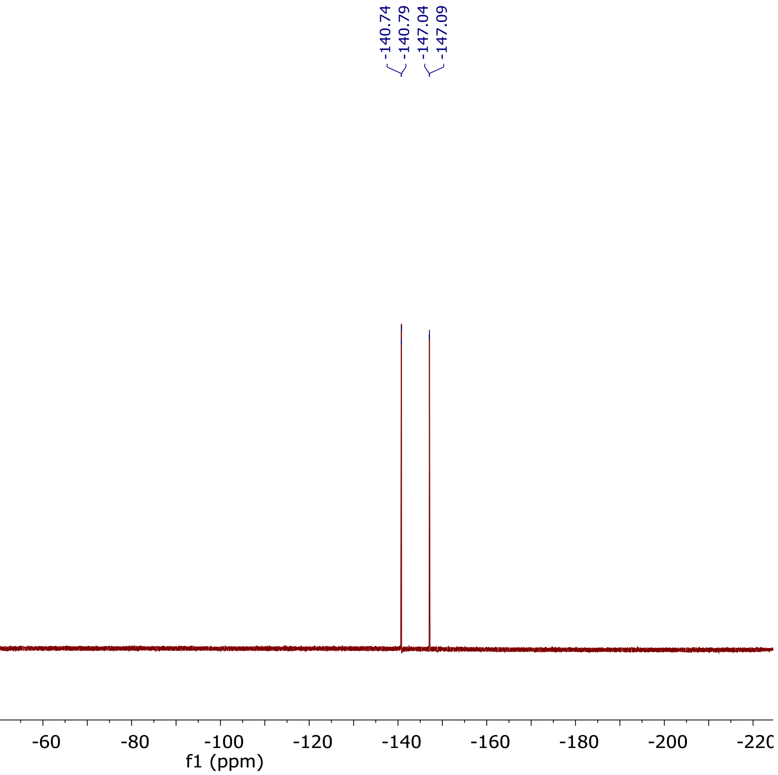

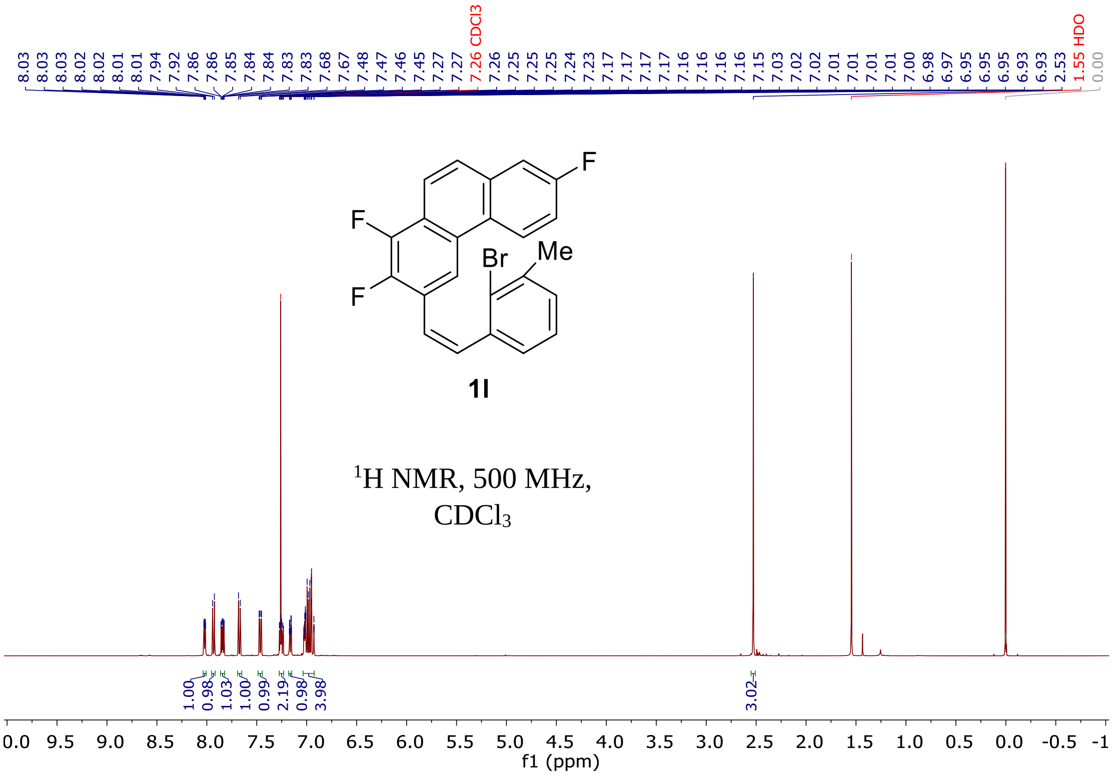
1l
^{1}H NMR, 500 MHz, CDCl3
f1 (ppm)

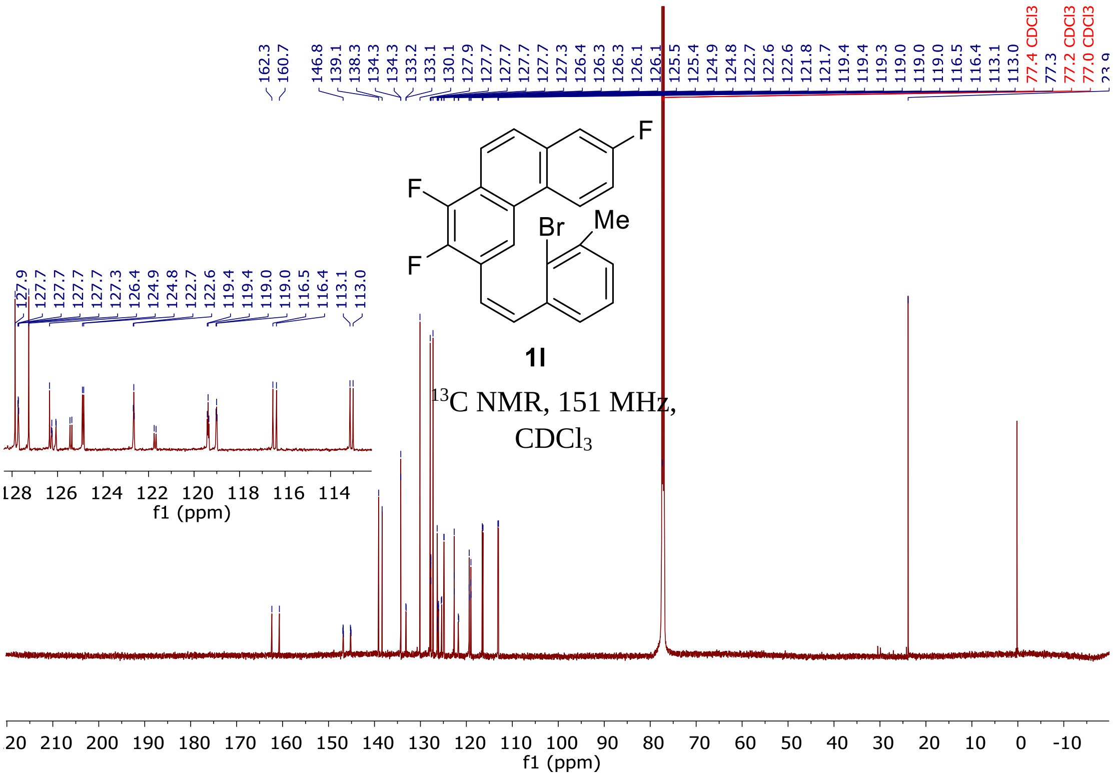
1l
^{13}C NMR, 151 MHz, CDCl3
f1 (ppm)

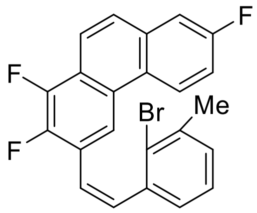

1l

^{19}F NMR, 376 MHz, $CDCl_3$

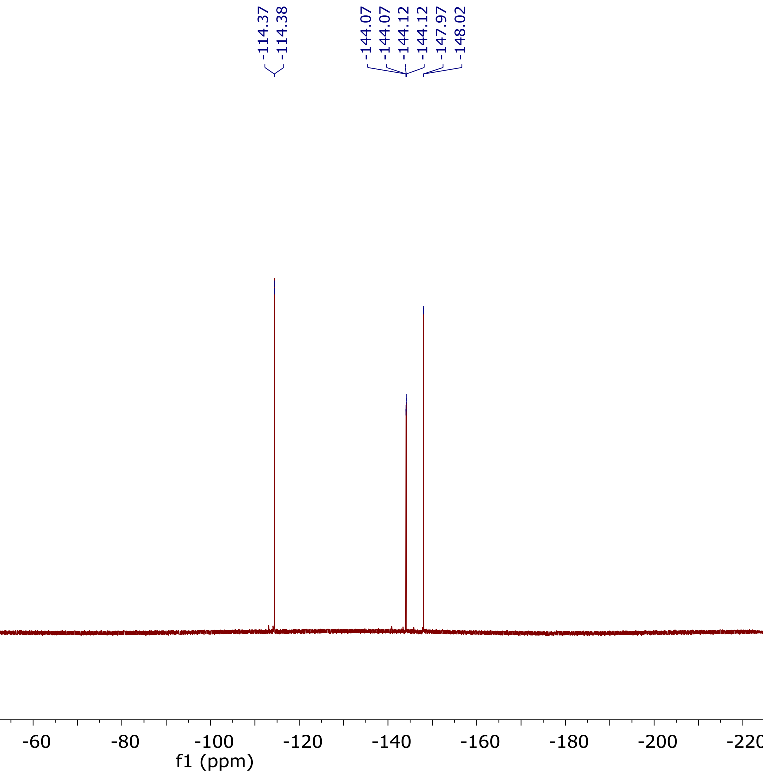

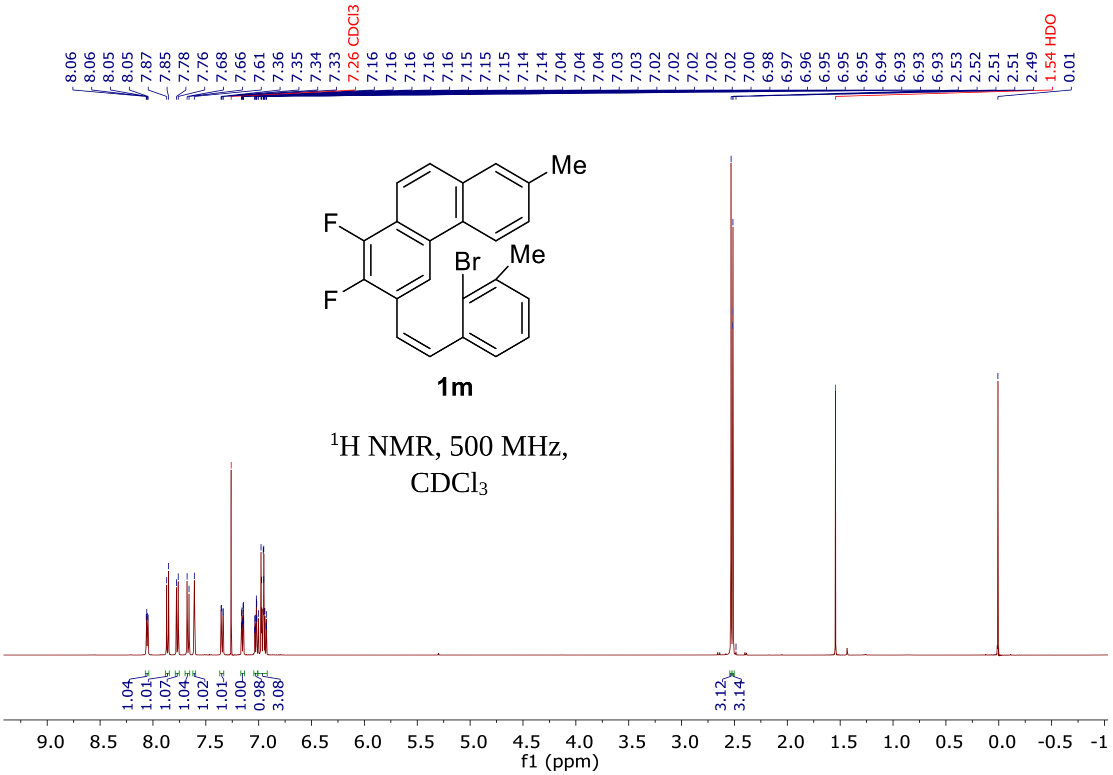
8.06
8.06
8.05
8.05
7.87
7.85
7.78
7.76
7.68
7.66
7.61
7.36
7.35
7.34
7.33
7.26 CDCl3
7.16
7.16
7.16
7.16
7.16
7.15
7.15
7.15
7.14
7.14
7.04
7.04
7.04
7.03
7.03
7.02
7.02
7.02
7.02
7.02
7.00
6.98
6.97
6.96
6.95
6.95
6.95
6.94
6.93
6.93
6.93
2.53
2.52
2.51
2.51
2.49
1.54 HDO
0.01
F
F
Br
Me
Me
1m
1H NMR, 500 MHz, CDCl3
1.04
1.01
1.07
1.04
1.02
1.01
1.00
0.98
3.08
3.12
3.14
f1 (ppm)

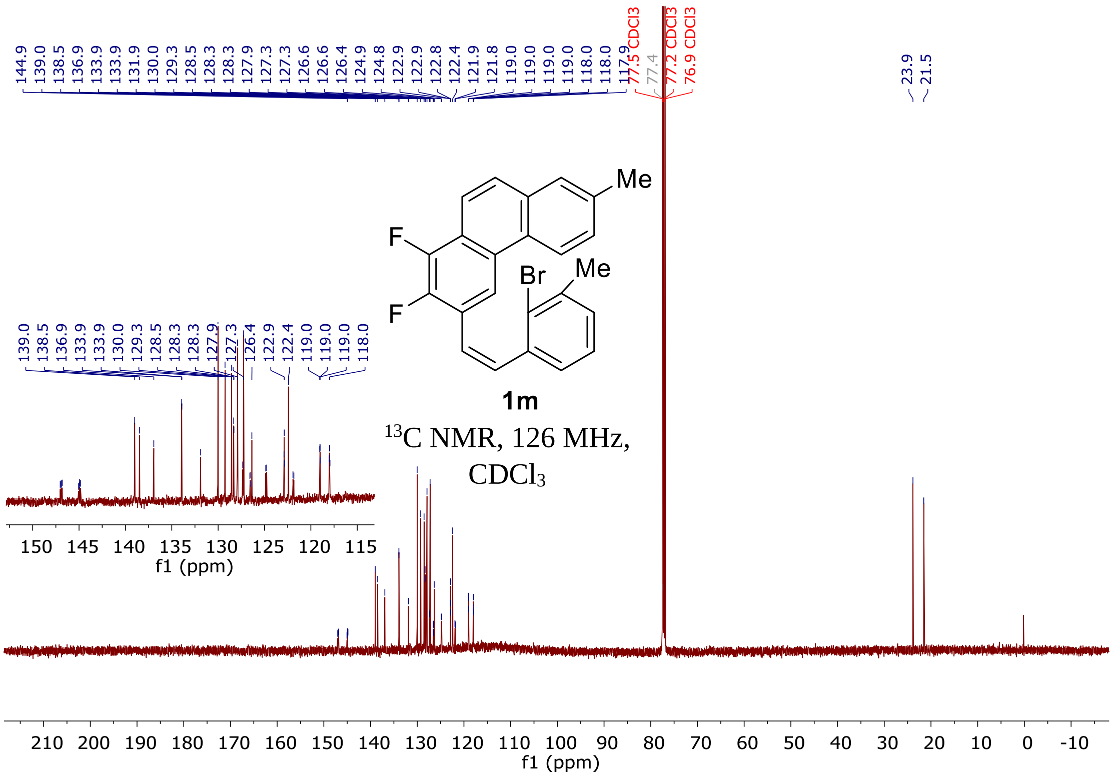
144.9
139.0
138.5
136.9
133.9
133.9
131.9
130.0
129.3
128.5
128.3
128.3
127.9
127.3
127.3
126.6
126.6
126.4
124.9
124.8
122.9
122.9
122.8
122.4
121.9
121.8
119.0
119.0
119.0
119.0
118.0
118.0
117.9
77.5 CDCl3
77.4
77.2 CDCl3
76.9 CDCl3
23.9
21.5
F
F
Br
Me
Me
1m
13C NMR, 126 MHz, CDCl3
f1 (ppm)

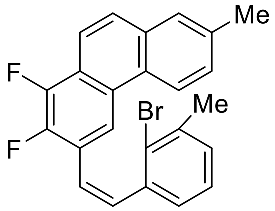

1m

^{19}F NMR, 376 MHz, $CDCl_3$

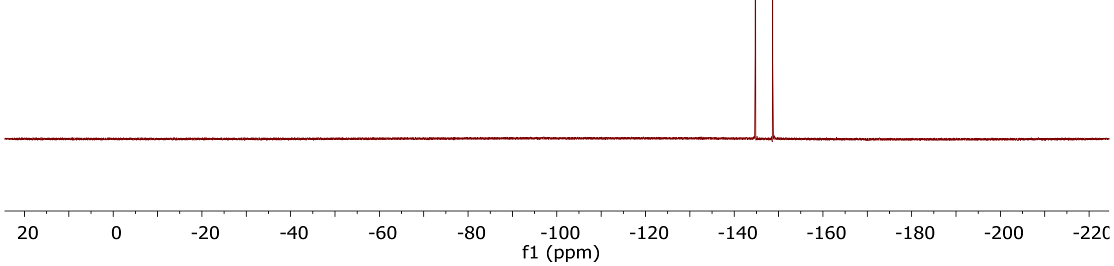

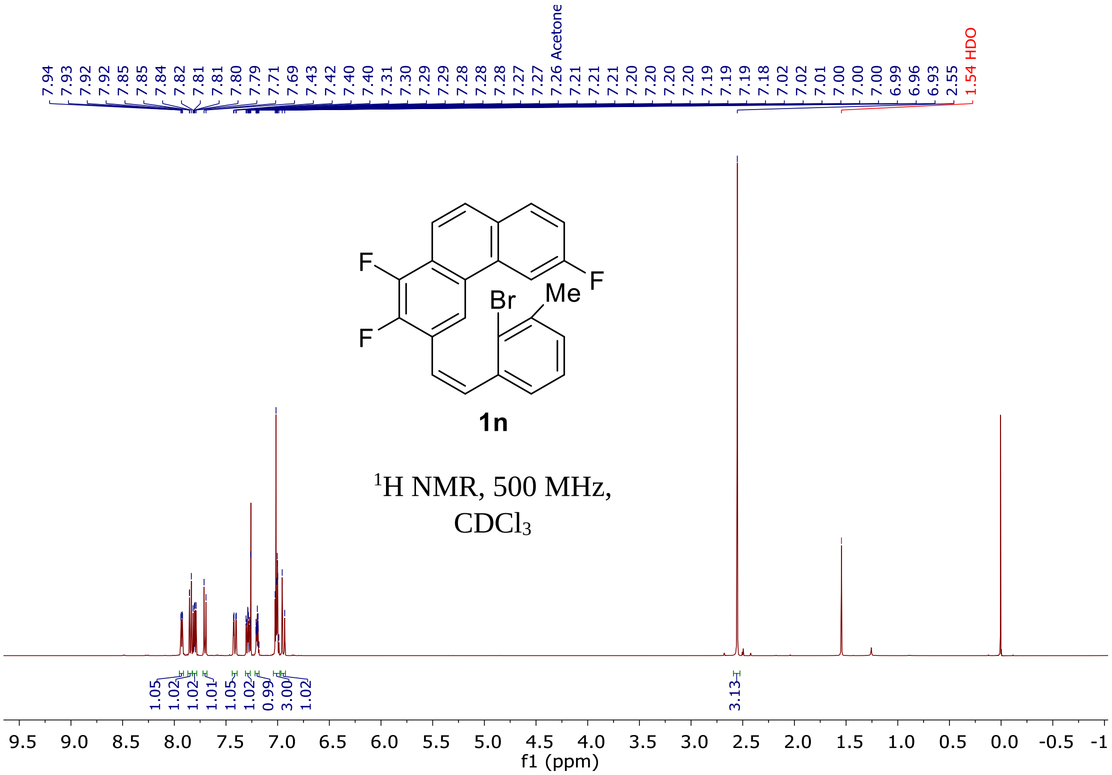
7.94 7.93 7.92 7.92 7.85 7.85 7.84 7.82 7.81 7.81 7.80 7.79 7.71 7.69 7.43 7.42 7.40 7.40 7.31 7.30 7.29 7.29 7.28 7.28 7.28 7.27 7.27 7.26 Acetone 7.21 7.21 7.21 7.20 7.20 7.20 7.20 7.19 7.19 7.19 7.18 7.02 7.02 7.01 7.00 7.00 6.99 6.96 6.93 2.55
1.54 HDO
F
F
Br
Me
F
1n
^{1}H NMR, 500 MHz, CDCl$_3$
1.05 1.02 1.02 1.01 1.05 1.02 0.99 3.00 1.02
3.13
f1 (ppm)

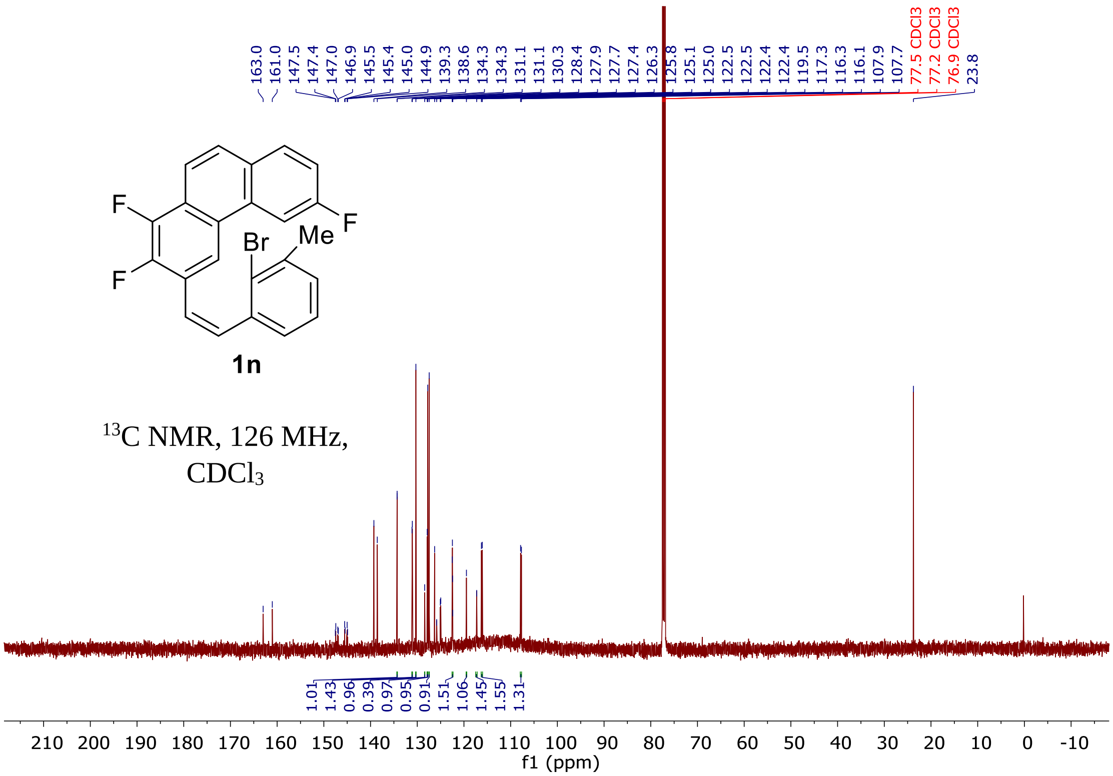
163.0 161.0 147.5 147.4 147.0 146.9 145.5 145.4 145.0 144.9 139.3 138.6 134.3 134.3 131.1 131.1 130.3 128.4 127.9 127.7 127.4 126.3 125.8 125.1 125.0 122.5 122.5 122.4 122.4 119.5 117.3 116.3 116.1 107.9 107.7
77.5 CDCl3 77.2 CDCl3 76.9 CDCl3
23.8
F
F
Br
Me
F
1n
^{13}C NMR, 126 MHz, CDCl$_3$
1.01 1.43 0.96 0.39 0.97 0.95 0.91 1.51 1.06 1.45 1.55 1.31
f1 (ppm)

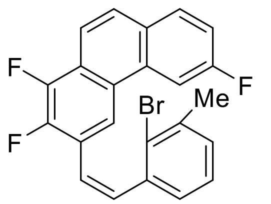

1n

^{19}F NMR, 376 MHz, $CDCl_3$

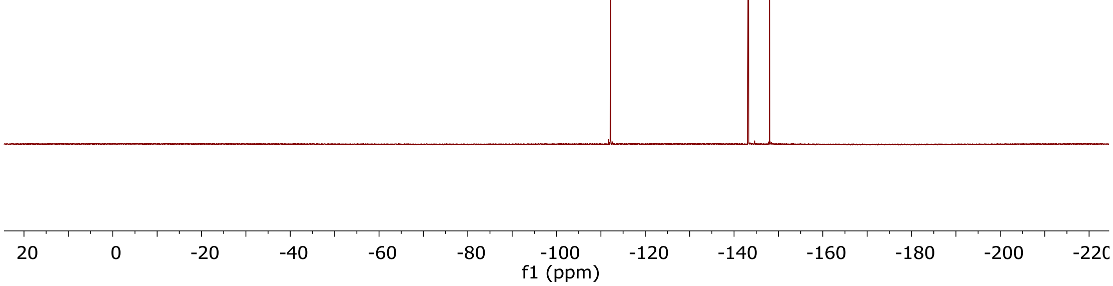

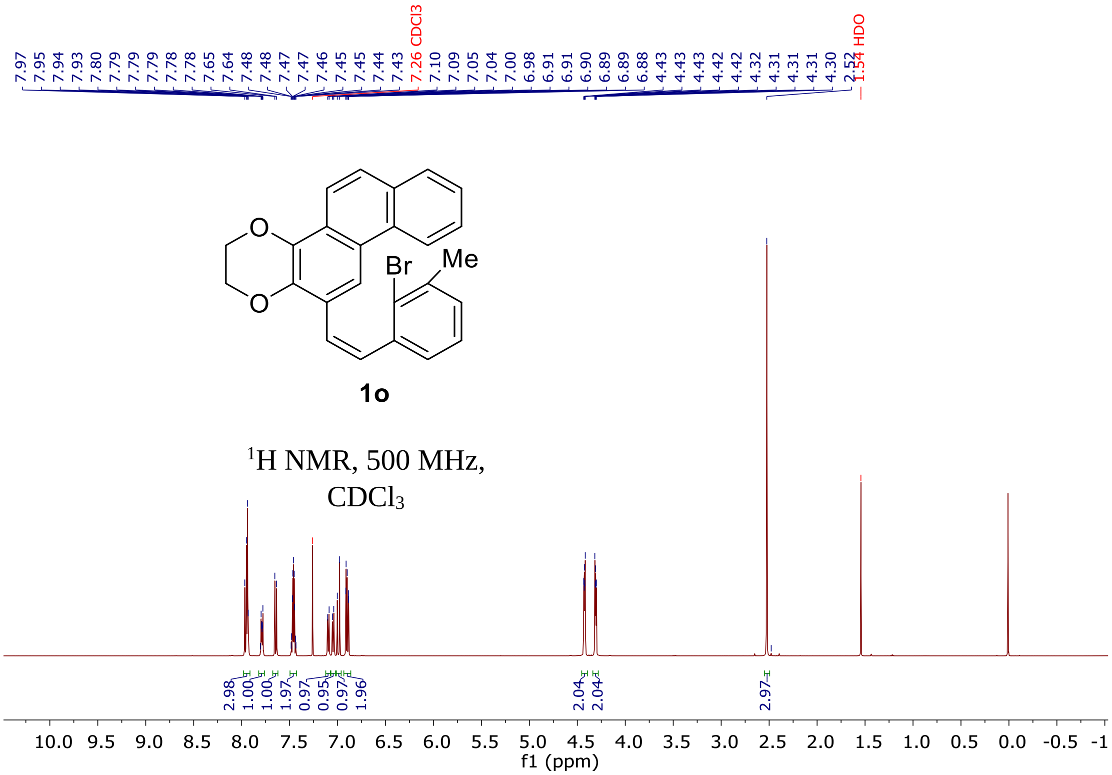
7.97 7.95 7.94 7.93 7.80 7.79 7.79 7.79 7.78 7.78 7.65 7.64 7.48 7.48 7.47 7.47 7.46 7.45 7.45 7.44 7.43
7.26 CDCl3
7.10 7.09 7.05 7.04 7.00 6.98 6.91 6.91 6.90 6.89 6.89 6.88 4.43 4.43 4.43 4.42 4.42 4.32 4.31 4.31 4.31 4.30 2.52
1.54 HDO
Br
Me
O
O
1o
¹H NMR, 500 MHz, CDCl3
2.98 1.00 1.00 1.97 0.97 0.95 0.97 1.96 2.04 2.04 2.97
f1 (ppm)

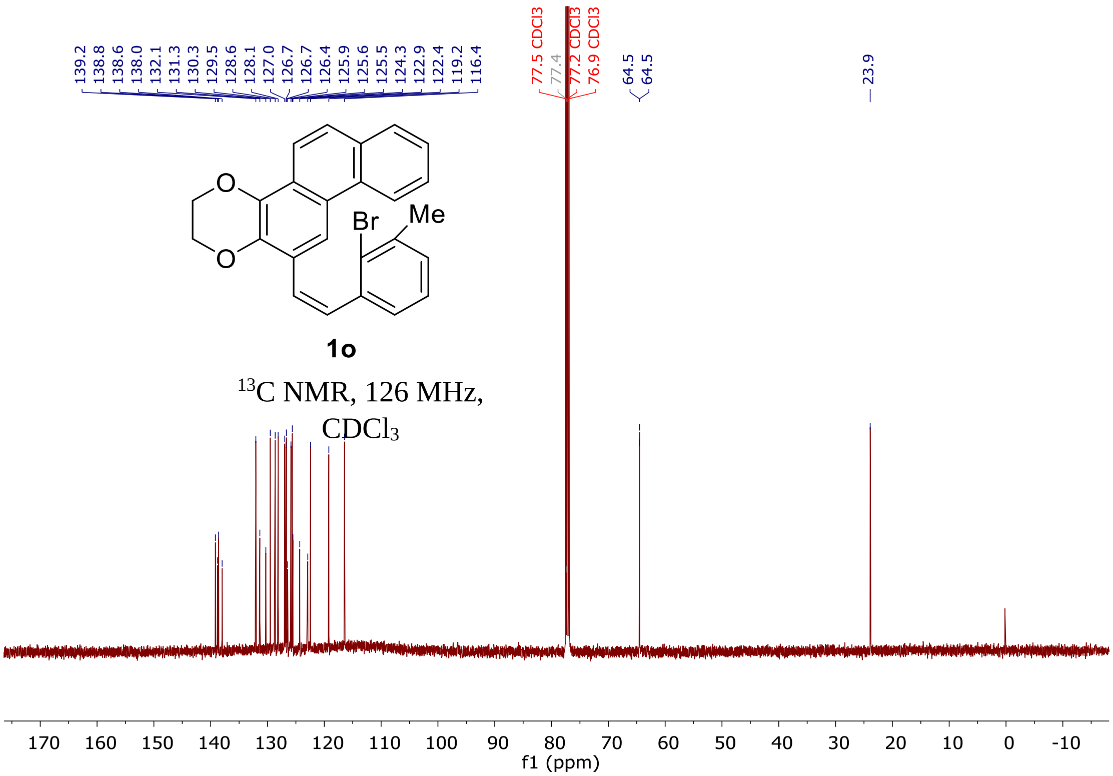
139.2 138.8 138.6 138.0 132.1 131.3 130.3 129.5 128.6 128.1 127.0 126.7 126.7 126.4 125.9 125.6 125.5 124.3 122.9 122.4 119.2 116.4
77.5 CDCl3
77.4
77.2 CDCl3
76.9 CDCl3
64.5 64.5
23.9
Br
Me
O
O
1o
¹³C NMR, 126 MHz, CDCl3
f1 (ppm)

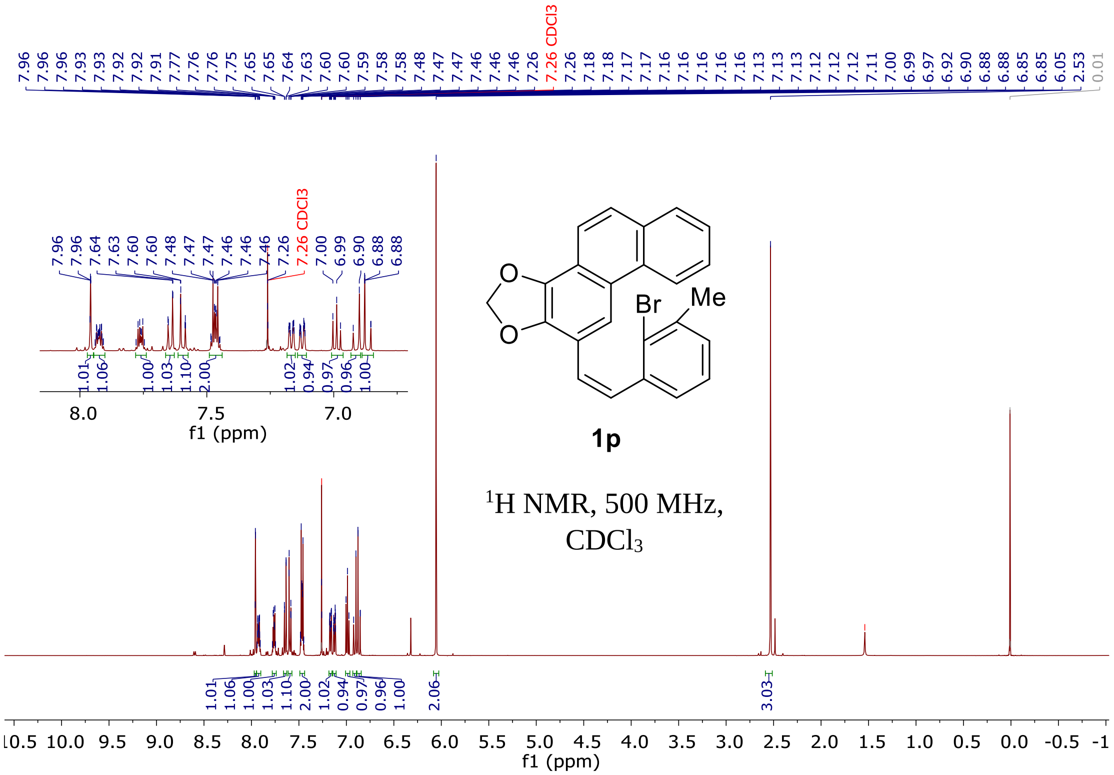
7.96 7.96 7.96 7.93 7.93 7.92 7.92 7.91 7.77 7.76 7.76 7.75 7.65 7.64 7.63 7.60 7.60 7.59 7.58 7.58 7.48 7.47 7.47 7.46 7.46 7.26 7.26 CDCl3 7.26 7.18 7.17 7.17 7.16 7.16 7.16 7.16 7.13 7.13 7.12 7.12 7.12 7.11 7.00 6.99 6.97 6.92 6.90 6.88 6.88 6.85 6.85 6.05 2.53 0.01
O
O
Br
Me
1p
^{1}H NMR, 500 MHz, $CDCl_3$
1.01 1.06 1.00 1.03 1.10 2.00 1.02 0.94 0.97 0.96 0.97 0.96 1.00 2.06 3.03
f1 (ppm)

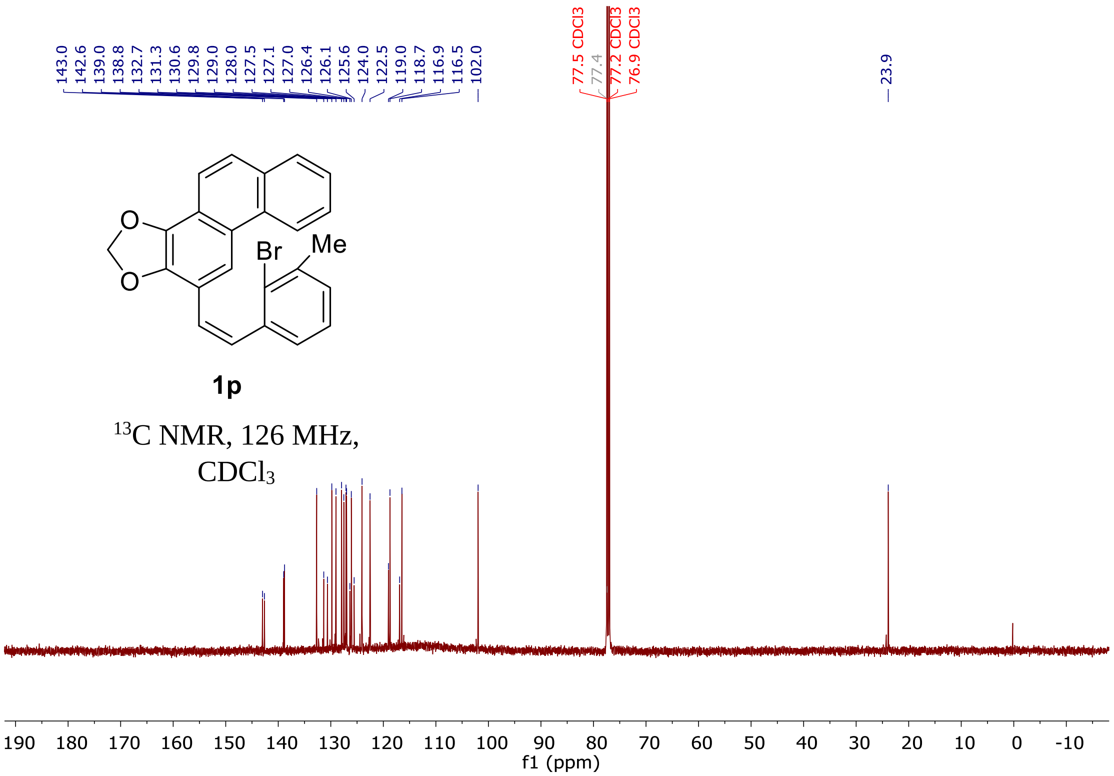
143.0 142.6 139.0 138.8 132.7 131.3 130.6 129.8 129.0 128.0 127.5 127.1 127.0 126.4 126.1 125.6 124.0 122.5 119.0 118.7 116.9 116.5 102.0
77.5 CDCl3 77.4 77.2 CDCl3 76.9 CDCl3
23.9
O
O
Br
Me
1p
^{13}C NMR, 126 MHz, $CDCl_3$
f1 (ppm)

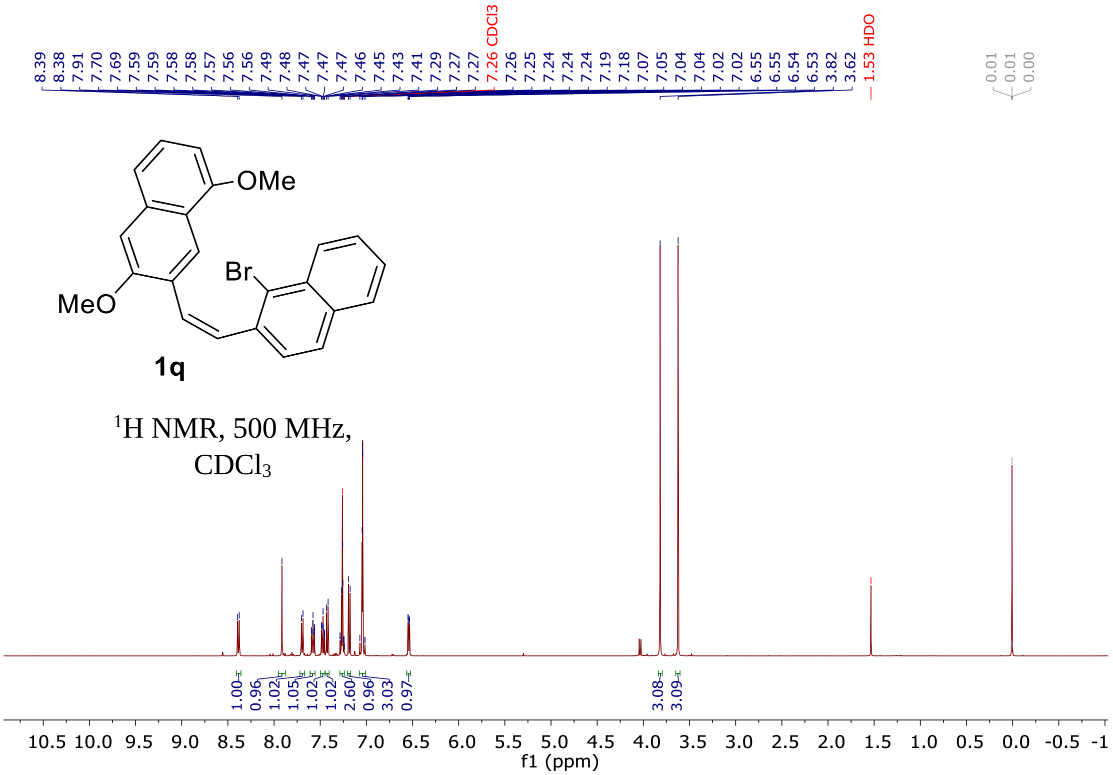
8.39 8.38 7.91 7.70 7.69 7.59 7.59 7.58 7.57 7.56 7.56 7.49 7.48 7.47 7.47 7.47 7.46 7.45 7.43 7.41 7.29 7.27 7.27 7.26 CDCl3 7.26 7.25 7.24 7.24 7.24 7.19 7.18 7.07 7.05 7.04 7.04 7.02 7.02 6.55 6.55 6.54 6.53 3.82 3.62 1.53 HDO
0.01 0.01 0.00
OMe
Br
MeO
1q
1H NMR, 500 MHz, CDCl3
1.00 0.96 1.02 1.05 1.02 1.02 2.60 0.96 3.03 0.97
3.08 3.09
10.5 10.0 9.5 9.0 8.5 8.0 7.5 7.0 6.5 6.0 5.5 5.0 4.5 4.0 3.5 3.0 2.5 2.0 1.5 1.0 0.5 0.0 -0.5 -1
f1 (ppm)

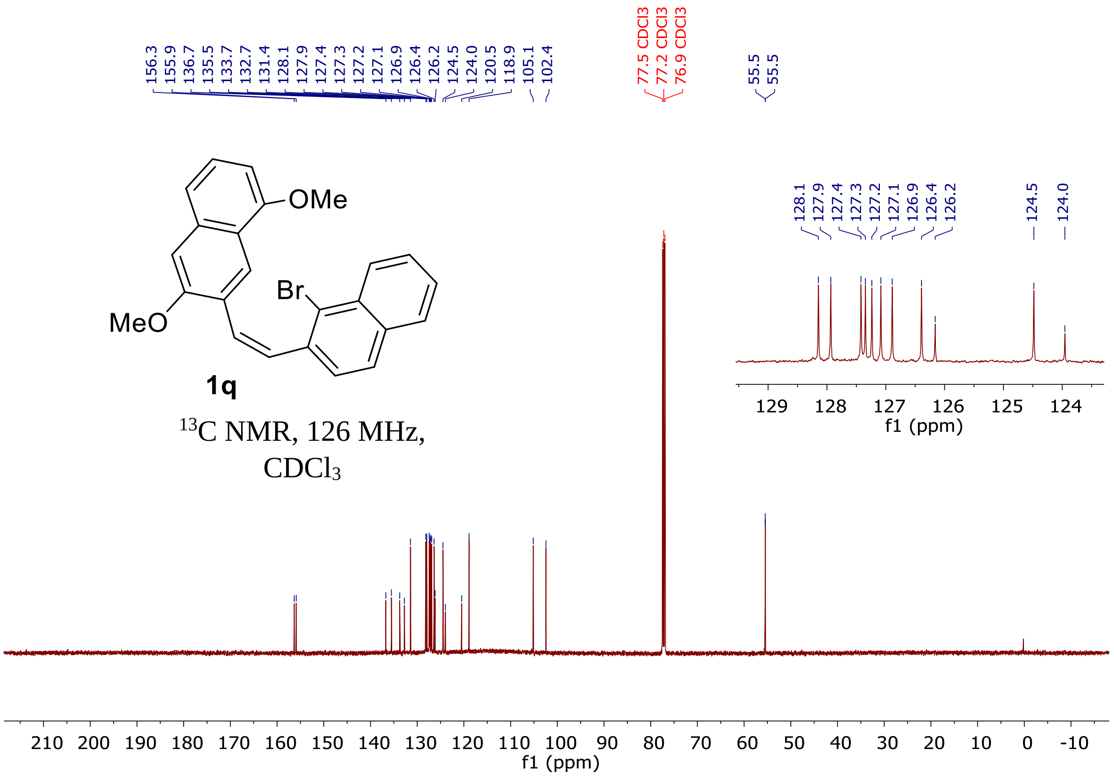
156.3 155.9 136.7 135.5 133.7 132.7 131.4 128.1 127.9 127.4 127.3 127.2 127.1 126.9 126.4 126.2 124.5 124.0 120.5 118.9 105.1 102.4
77.5 CDCl3 77.2 CDCl3 76.9 CDCl3
55.5 55.5
OMe
Br
MeO
1q
13C NMR, 126 MHz, CDCl3
128.1 127.9 127.4 127.3 127.2 127.1 126.9 126.4 126.2 124.5 124.0
129 128 127 126 125 124
f1 (ppm)
210 200 190 180 170 160 150 140 130 120 110 100 90 80 70 60 50 40 30 20 10 0 -10
f1 (ppm)

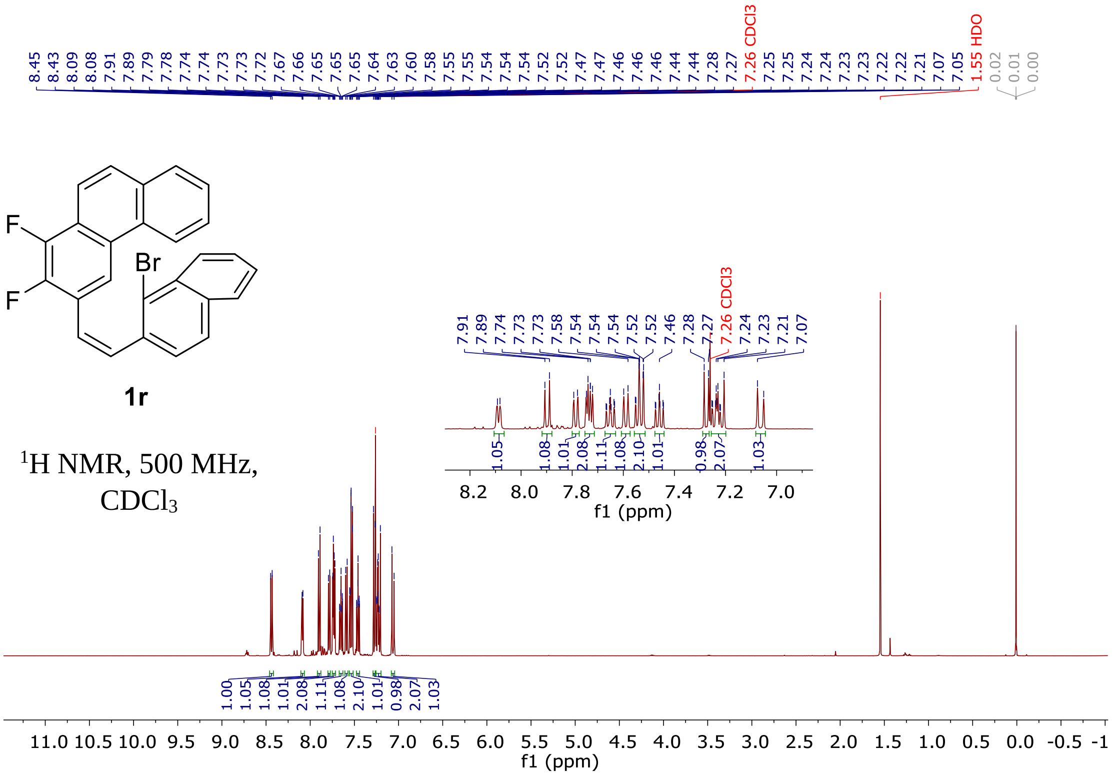
8.45 8.43 8.09 8.08 7.91 7.89 7.79 7.78 7.74 7.74 7.73 7.73 7.72 7.67 7.66 7.65 7.65 7.64 7.63 7.60 7.58 7.55 7.54 7.54 7.54 7.52 7.52 7.47 7.46 7.46 7.44 7.44 7.28 7.27 7.26 CDCl3 7.25 7.25 7.24 7.24 7.23 7.23 7.22 7.22 7.21 7.07 7.05 1.55 HDO 0.02 0.01 0.00
F
F
Br
1r
1H NMR, 500 MHz, CDCl3
f1 (ppm)

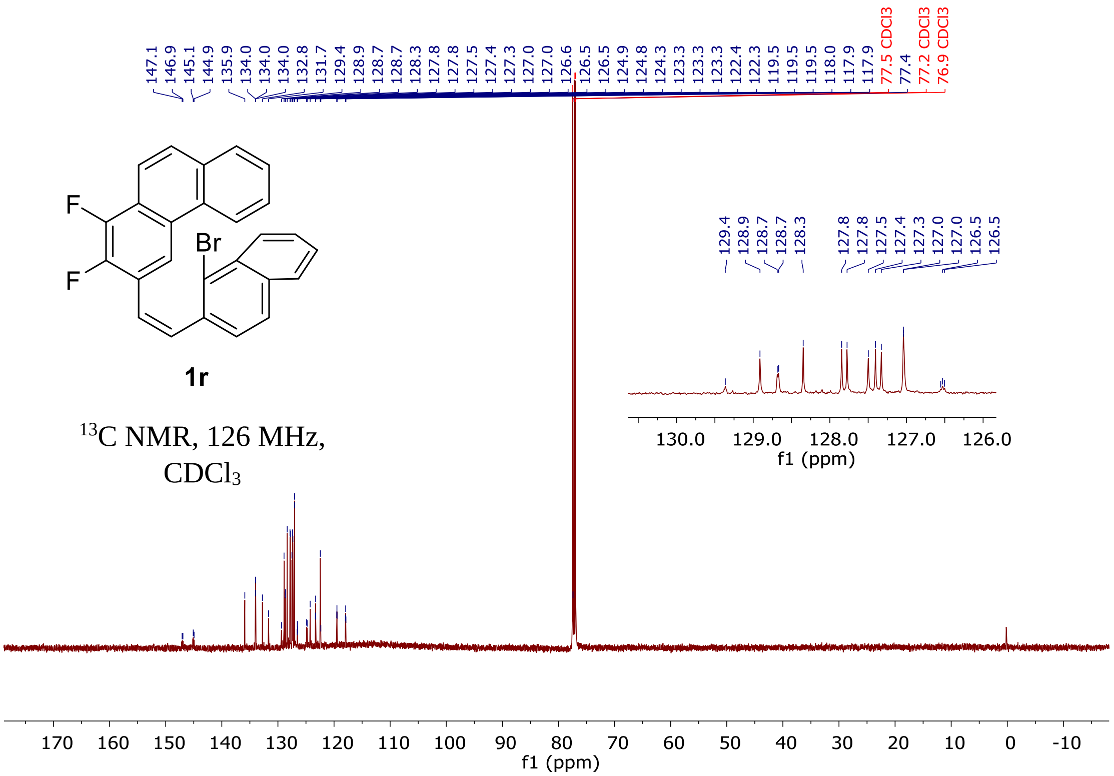
147.1 146.9 145.1 144.9 135.9 134.0 134.0 134.0 132.8 131.7 129.4 128.9 128.7 128.7 128.3 127.8 127.8 127.5 127.4 127.3 127.0 127.0 126.6 126.5 126.5 124.9 124.8 124.3 123.3 123.3 123.3 122.4 122.3 119.5 119.5 118.0 117.9 77.5 CDCl3 77.4 77.2 CDCl3 76.9 CDCl3
F
F
Br
1r
13C NMR, 126 MHz, CDCl3
f1 (ppm)

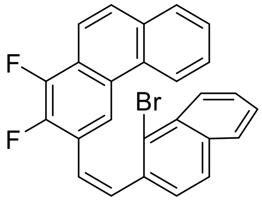

1r

^{19}F NMR, 376 MHz, $CDCl_3$

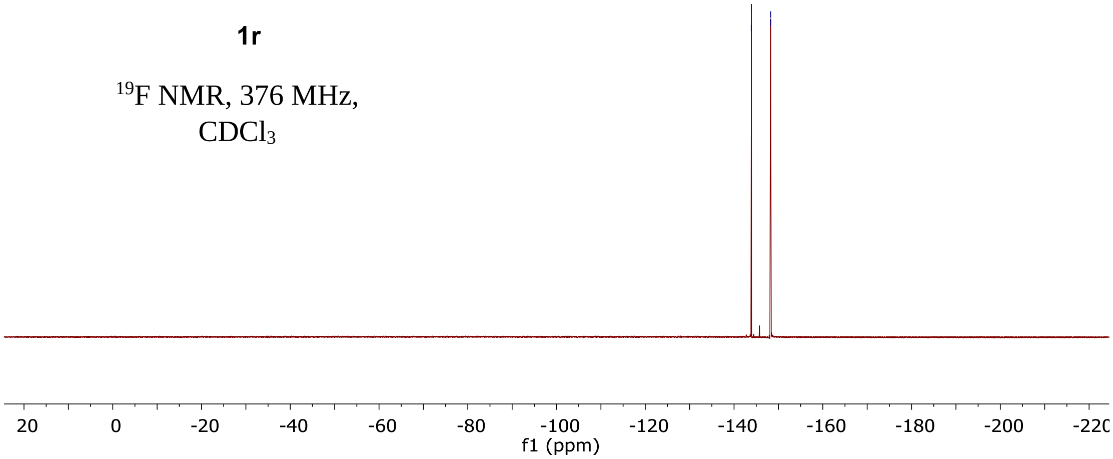

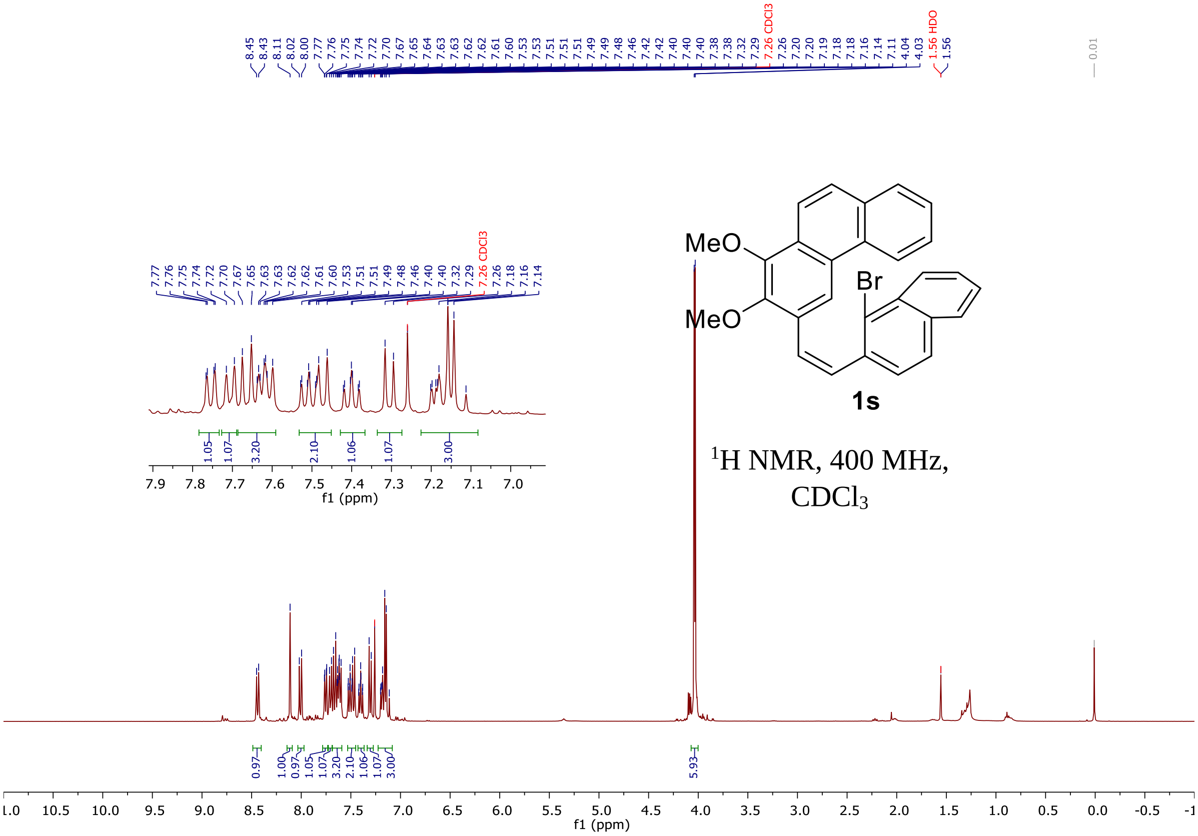
8.45 8.43 8.11 8.02 8.00 7.77 7.76 7.75 7.74 7.72 7.70 7.67 7.65 7.64 7.63 7.63 7.62 7.62 7.61 7.60 7.53 7.53 7.51 7.51 7.51 7.49 7.48 7.46 7.42 7.42 7.40 7.40 7.38 7.38 7.32 7.29 7.26 CDCl3 7.26 7.20 7.20 7.19 7.18 7.18 7.16 7.14 7.11 4.04 4.03 1.56 HDO 1.56
0.01
MeO
MeO
Br
1s
^{1}H NMR, 400 MHz, CDCl$_3$
0.97 1.00 0.97 1.05 1.07 3.20 2.10 1.06 1.07 3.00
5.93
f1 (ppm)

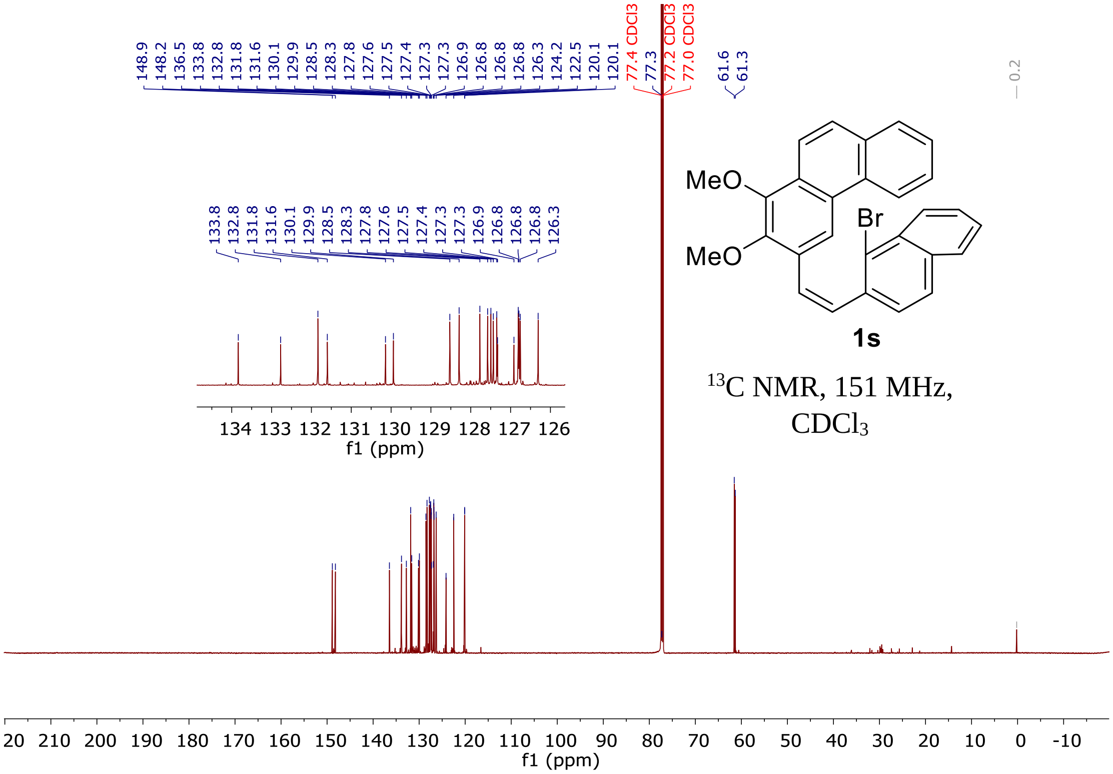
148.9 148.2 136.5 133.8 132.8 131.8 131.6 130.1 129.9 128.5 128.3 127.8 127.6 127.5 127.4 127.3 127.3 126.9 126.8 126.8 126.3 124.2 122.5 120.1 120.1
77.4 CDCl3 77.3 77.2 CDCl3 77.0 CDCl3
61.6 61.3
0.2
MeO
MeO
Br
1s
^{13}C NMR, 151 MHz, CDCl$_3$
f1 (ppm)

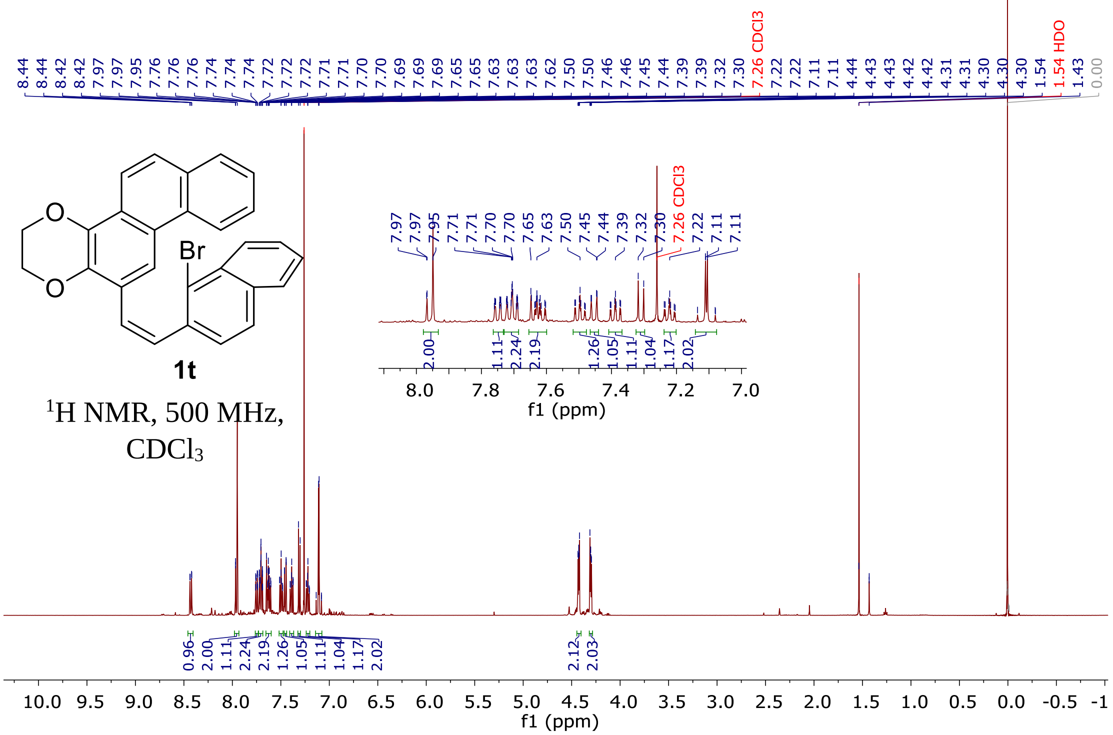
1t
^{1}H NMR, 500 MHz, $CDCl_3$
f1 (ppm)

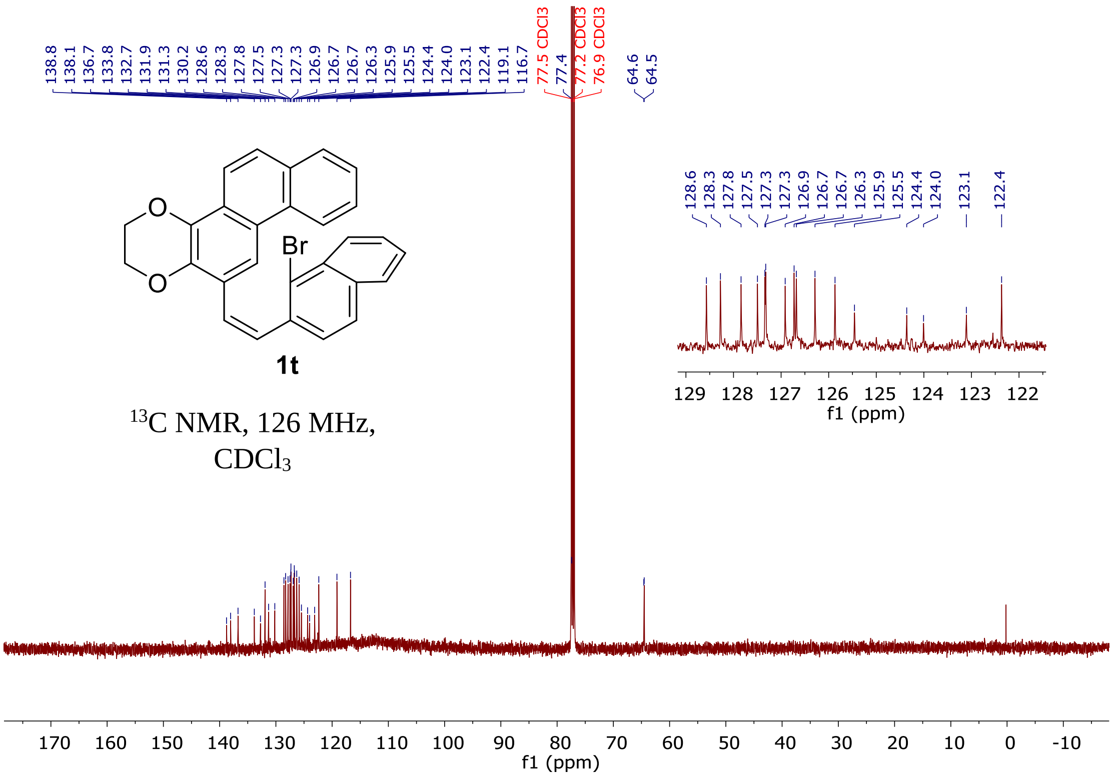
1t
^{13}C NMR, 126 MHz, $CDCl_3$
f1 (ppm)

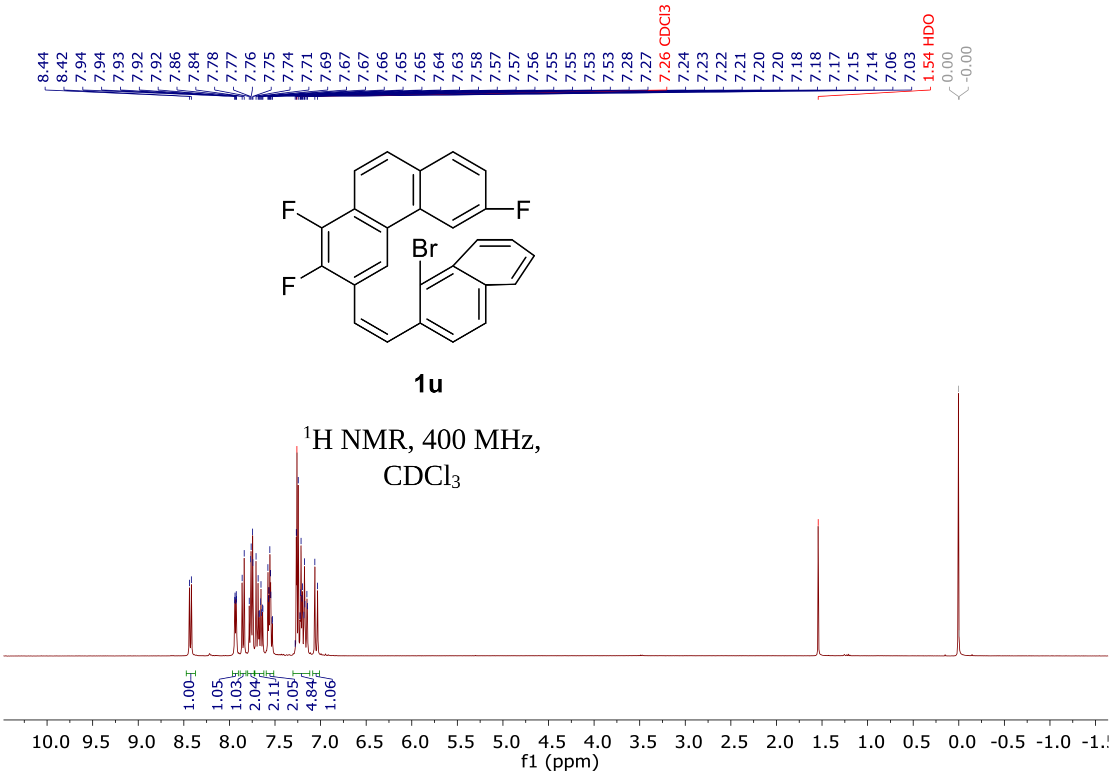
8.44
8.42
7.94
7.94
7.93
7.92
7.92
7.86
7.84
7.78
7.77
7.76
7.75
7.74
7.71
7.69
7.67
7.67
7.66
7.65
7.65
7.64
7.63
7.58
7.57
7.57
7.56
7.55
7.55
7.53
7.53
7.28
7.27
7.26 CDCl3
7.24
7.23
7.22
7.21
7.20
7.20
7.18
7.18
7.17
7.15
7.14
7.06
7.03
1.54 HDO
0.00
-0.00
F
F
F
Br
1u
1H NMR, 400 MHz, CDCl3
1.00
1.05
1.03
2.04
2.11
2.05
4.84
1.06
f1 (ppm)

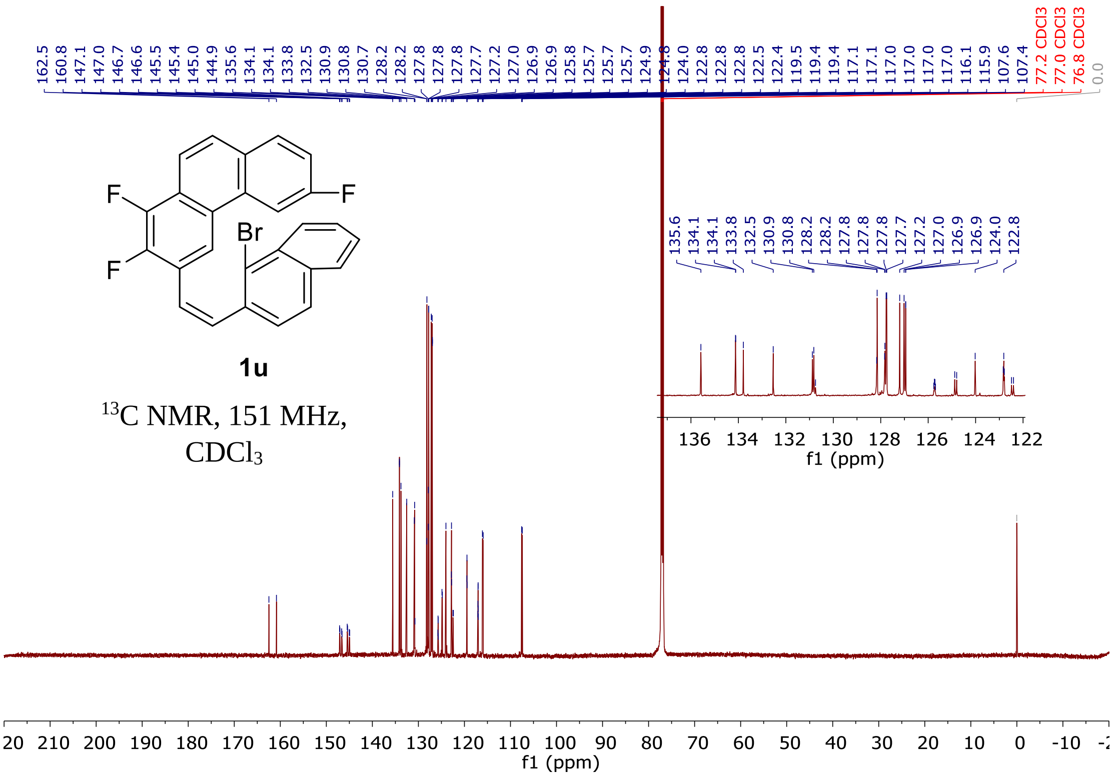
162.5
160.8
147.1
147.0
146.7
146.6
145.5
145.4
145.0
144.9
135.6
134.1
134.1
133.8
132.5
130.9
130.8
130.7
128.2
128.2
127.8
127.8
127.8
127.7
127.2
127.0
126.9
126.9
125.8
125.7
125.7
125.7
124.9
124.8
124.0
122.8
122.8
122.8
122.5
122.4
119.5
119.4
119.4
117.1
117.1
117.0
117.0
117.0
117.0
116.1
115.9
107.6
107.4
77.2 CDCl3
77.0 CDCl3
76.8 CDCl3
0.0
F
F
F
Br
1u
13C NMR, 151 MHz, CDCl3
f1 (ppm)

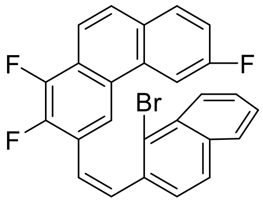

1u

^{19}F NMR, 376 MHz, $CDCl_3$

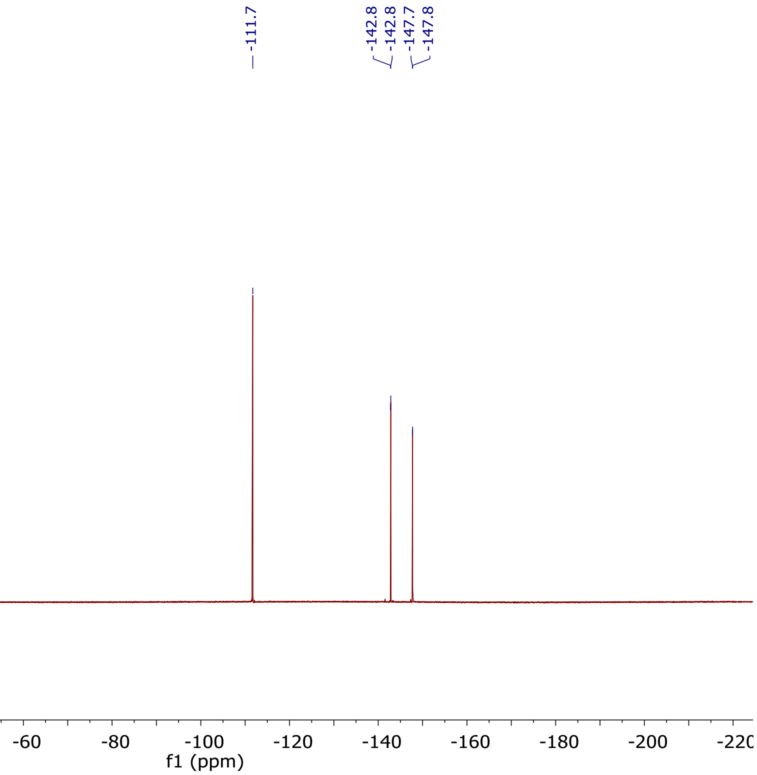

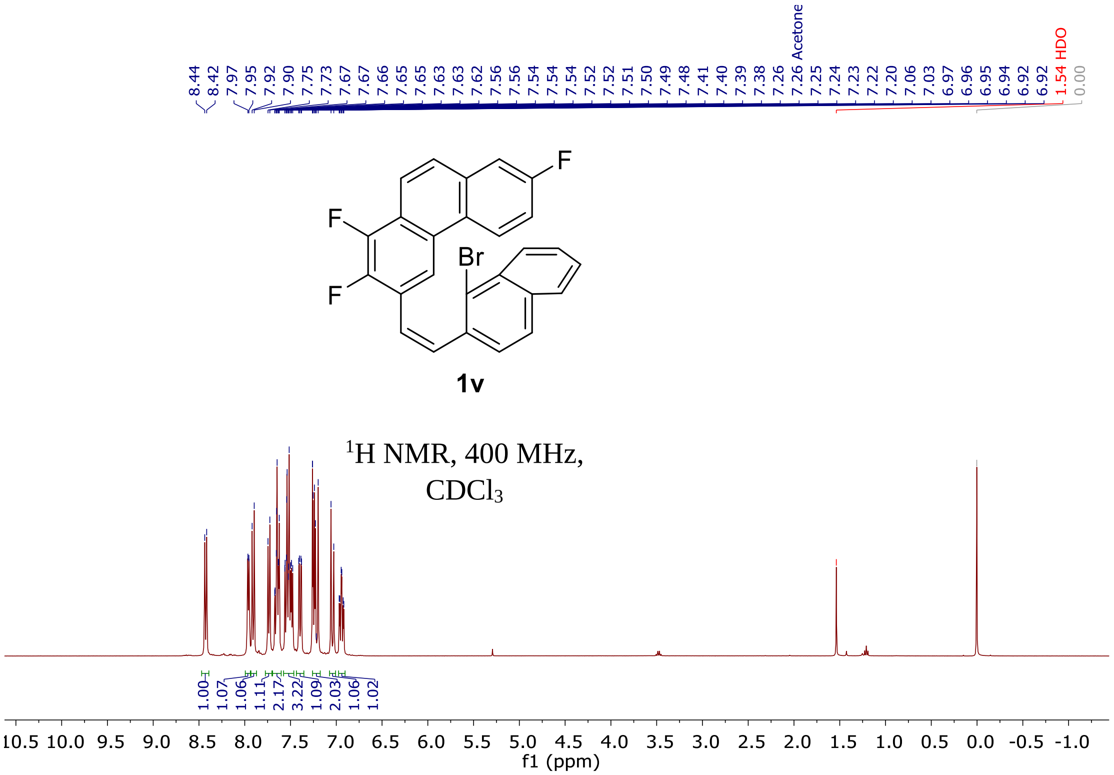
8.44
8.42
7.97
7.95
7.92
7.90
7.75
7.73
7.67
7.67
7.66
7.65
7.65
7.63
7.63
7.62
7.56
7.54
7.54
7.54
7.52
7.52
7.51
7.50
7.49
7.48
7.41
7.40
7.39
7.38
7.26
7.26 Acetone
7.25
7.24
7.23
7.22
7.20
7.06
7.03
6.97
6.96
6.95
6.94
6.92
6.92
1.54 HDO
0.00
F
F
F
Br
1v
1H NMR, 400 MHz,
CDCl3
1.00
1.07
1.06
1.11
2.17
3.22
1.09
2.03
1.06
1.02
10.5 10.0 9.5 9.0 8.5 8.0 7.5 7.0 6.5 6.0 5.5 5.0 4.5 4.0 3.5 3.0 2.5 2.0 1.5 1.0 0.5 0.0 -0.5 -1.0
f1 (ppm)

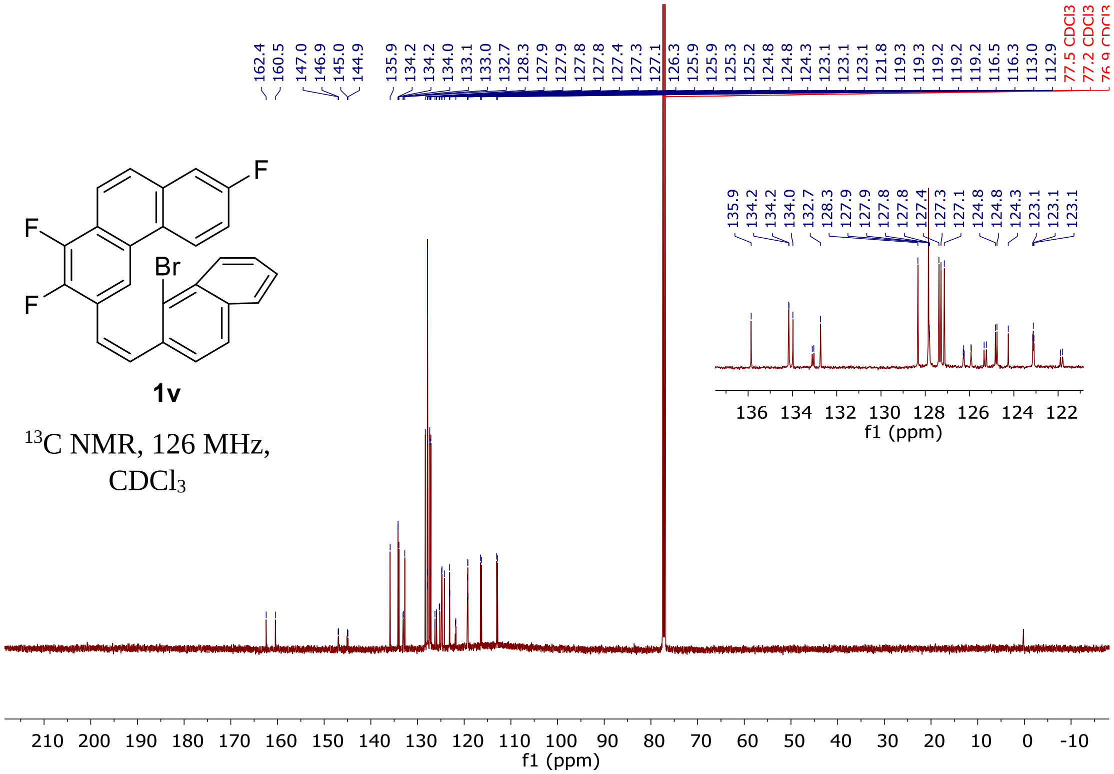
162.4
160.5
147.0
146.9
145.0
144.9
135.9
134.2
134.2
134.0
133.1
133.0
132.7
128.3
127.9
127.9
127.8
127.8
127.4
127.3
127.1
126.3
125.9
125.9
125.3
125.2
124.8
124.8
124.3
123.1
123.1
123.1
121.8
119.3
119.3
119.2
119.2
119.2
116.5
116.3
113.0
112.9
77.5 CDCl3
77.2 CDCl3
76.9 CDCl3
F
F
F
Br
1v
13C NMR, 126 MHz,
CDCl3
135.9
134.2
134.2
134.0
132.7
128.3
127.9
127.9
127.8
127.8
127.4
127.3
127.1
124.8
124.8
124.3
123.1
123.1
123.1
136 134 132 130 128 126 124 122
f1 (ppm)
210 200 190 180 170 160 150 140 130 120 110 100 90 80 70 60 50 40 30 20 10 0 -10
f1 (ppm)

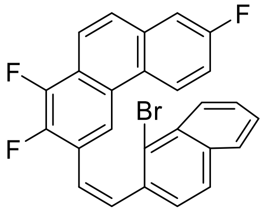

1v

^{19}F NMR, 376 MHz, $CDCl_3$

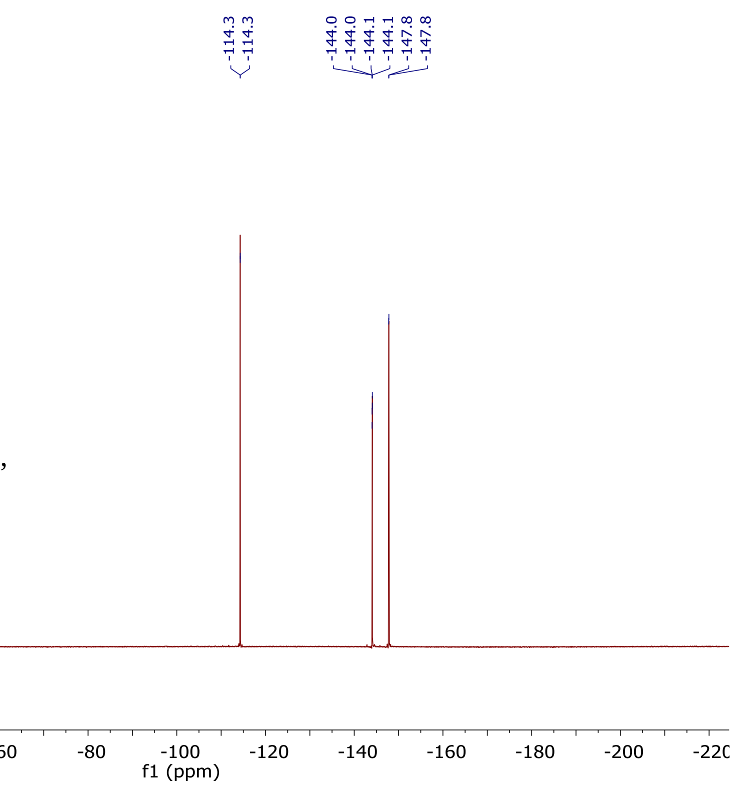

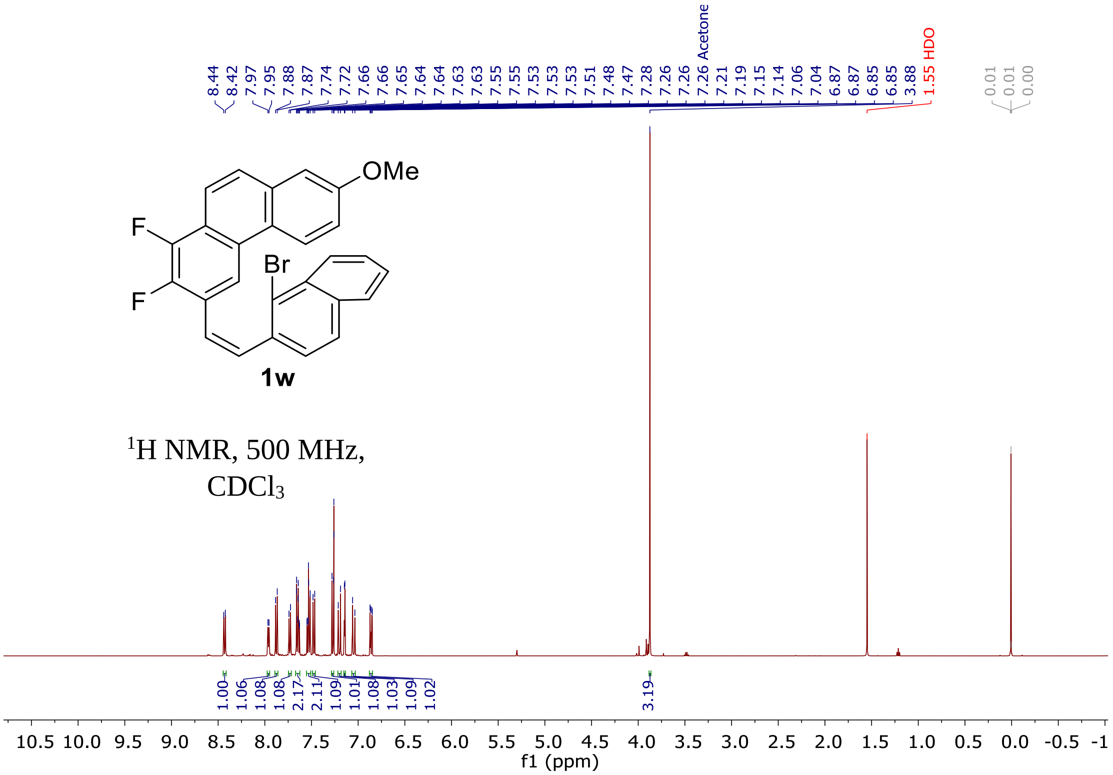
8.44 8.42 7.97 7.95 7.88 7.87 7.74 7.72 7.66 7.66 7.65 7.64 7.64 7.63 7.63 7.55 7.55 7.53 7.53 7.53 7.51 7.48 7.47 7.28 7.26 7.26 7.26 Acetone 7.21 7.19 7.15 7.14 7.06 7.04 6.87 6.87 6.85 6.85 3.88
1.55 HDO
0.01 0.01 0.00
OMe
F
F
Br
1w
^{1}H NMR, 500 MHz, $CDCl_3$
1.00 1.06 1.08 1.08 2.17 2.11 1.09 1.01 1.08 1.03 1.09 1.02
3.19
f1 (ppm)

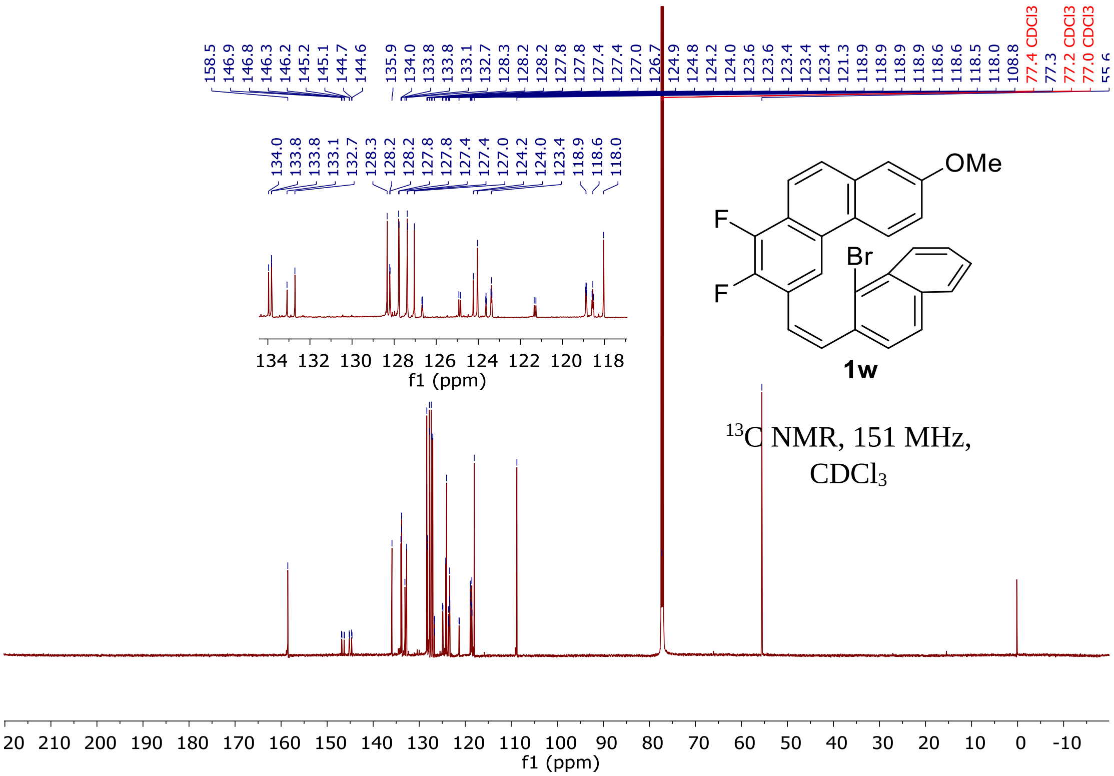
158.5 146.9 146.8 146.3 146.2 145.2 145.1 144.7 144.6 135.9 134.0 133.8 133.8 133.1 132.7 128.3 128.2 128.2 127.8 127.8 127.4 127.4 127.0 126.7 124.9 124.8 124.2 124.0 123.6 123.6 123.4 123.4 123.4 121.3 118.9 118.9 118.9 118.9 118.6 118.6 118.5 118.0 108.8
77.4 CDCl3 77.3 77.2 CDCl3 77.0 CDCl3
134.0 133.8 133.8 133.1 132.7 128.3 128.2 128.2 127.8 127.8 127.4 127.4 127.0 124.2 124.0 123.4 118.9 118.6 118.0
f1 (ppm)
OMe
F
F
Br
1w
^{13}C NMR, 151 MHz, $CDCl_3$
f1 (ppm)

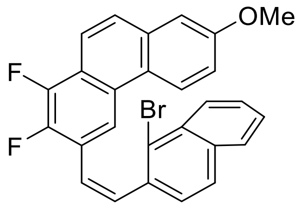

1w

^{19}F NMR, 376 MHz, $CDCl_3$

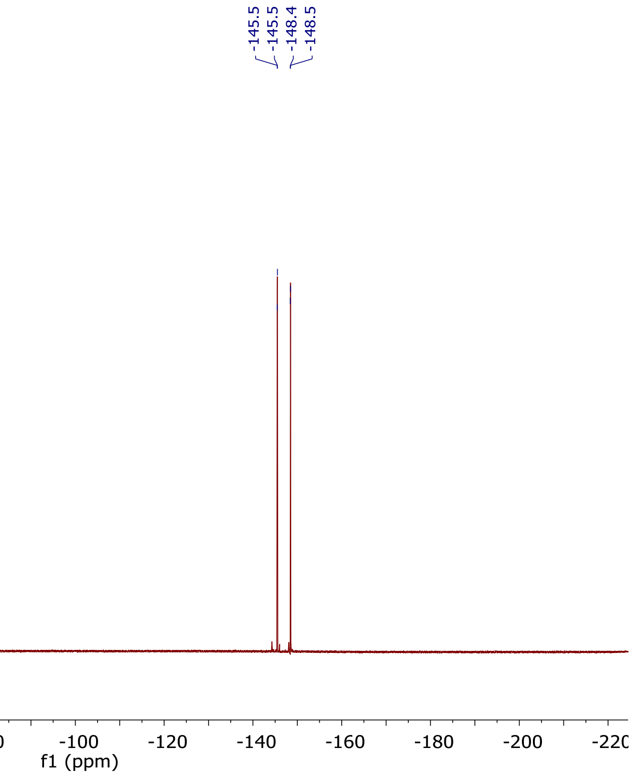

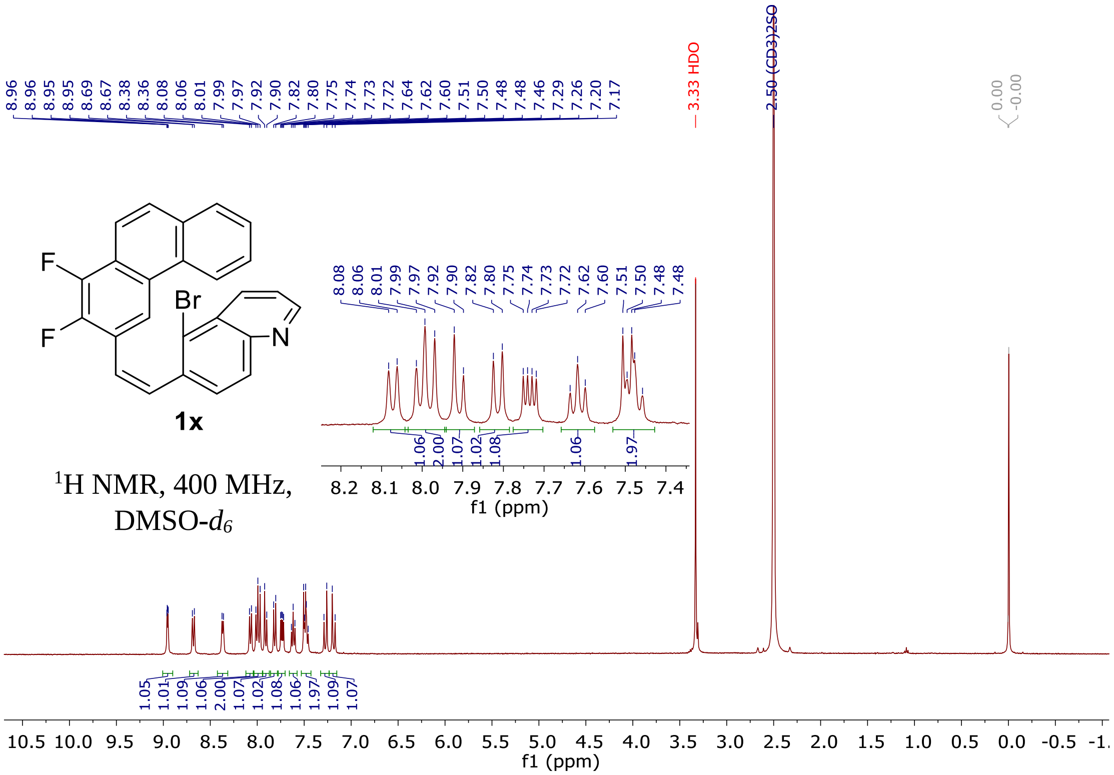
8.96 8.96 8.95 8.95 8.69 8.67 8.38 8.36 8.08 8.06 8.01 7.99 7.97 7.92 7.90 7.82 7.80 7.75 7.74 7.73 7.72 7.64 7.62 7.60 7.51 7.50 7.48 7.46 7.29 7.26 7.20 7.17
3.33 HDO
2.50 (CD3)2SO
0.00 -0.00
F
F
Br
N
1x
1H NMR, 400 MHz, DMSO-d6
8.08 8.06 8.01 7.99 7.97 7.92 7.90 7.82 7.80 7.75 7.74 7.73 7.72 7.62 7.60 7.51 7.50 7.48 7.48
1.06 2.00 1.07 1.02 1.08 1.06 1.97
8.2 8.1 8.0 7.9 7.8 7.7 7.6 7.5 7.4
f1 (ppm)
1.05 1.01 1.09 1.06 2.00 1.07 1.02 1.08 1.06 1.97 1.09 1.07
10.5 10.0 9.5 9.0 8.5 8.0 7.5 7.0 6.5 6.0 5.5 5.0 4.5 4.0 3.5 3.0 2.5 2.0 1.5 1.0 0.5 0.0 -0.5
f1 (ppm)

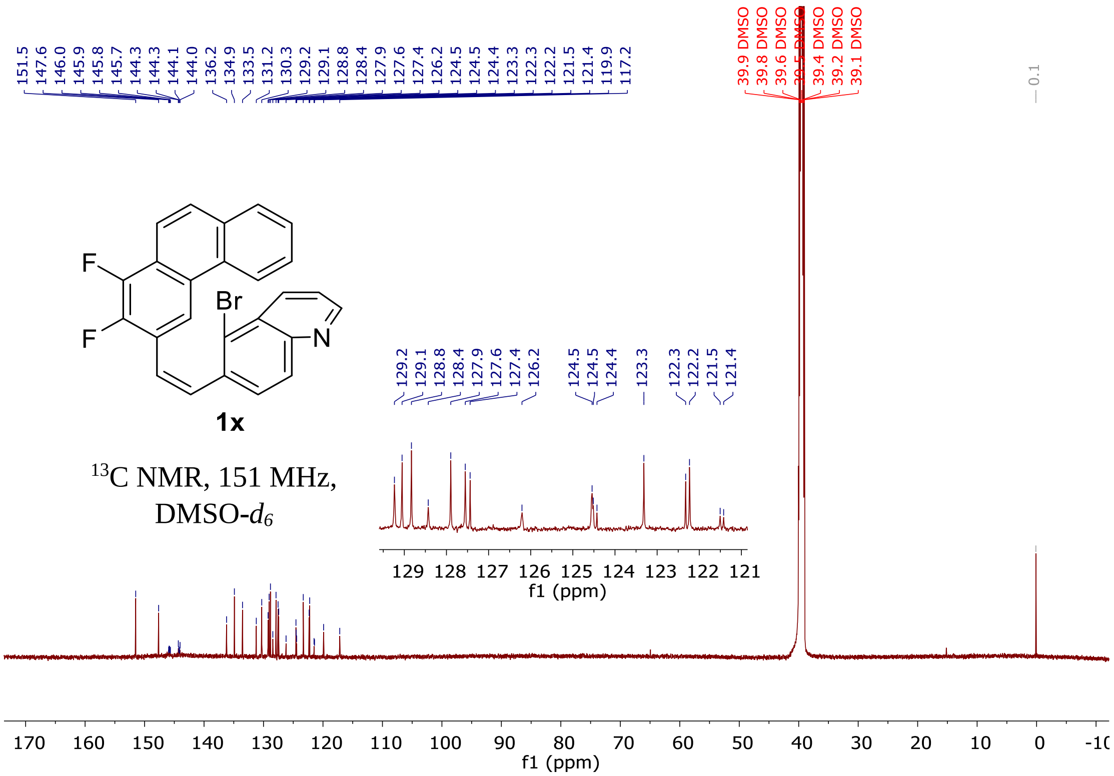
151.5 147.6 146.0 145.9 145.8 145.7 144.3 144.3 144.1 144.0 136.2 134.9 133.5 131.2 130.3 129.2 129.1 128.8 128.4 127.9 127.6 127.4 126.2 124.5 124.5 124.4 123.3 122.3 122.2 121.5 121.4 119.9 117.2
39.9 DMSO 39.8 DMSO 39.6 DMSO 39.5 DMSO 39.4 DMSO 39.2 DMSO 39.1 DMSO
0.1
F
F
Br
N
1x
13C NMR, 151 MHz, DMSO-d6
129.2 129.1 128.8 128.4 127.9 127.6 127.4 126.2 124.5 124.5 124.4 123.3 122.3 122.2 121.5 121.4
129 128 127 126 125 124 123 122 121
f1 (ppm)
170 160 150 140 130 120 110 100 90 80 70 60 50 40 30 20 10 0
f1 (ppm)

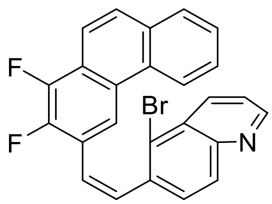

1x

^{19}F NMR, 376 MHz, DMSO-d_6

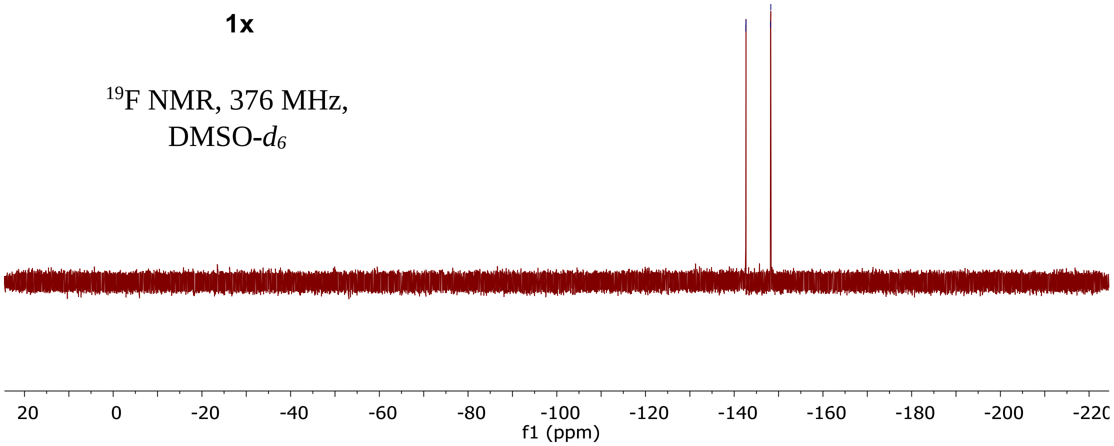

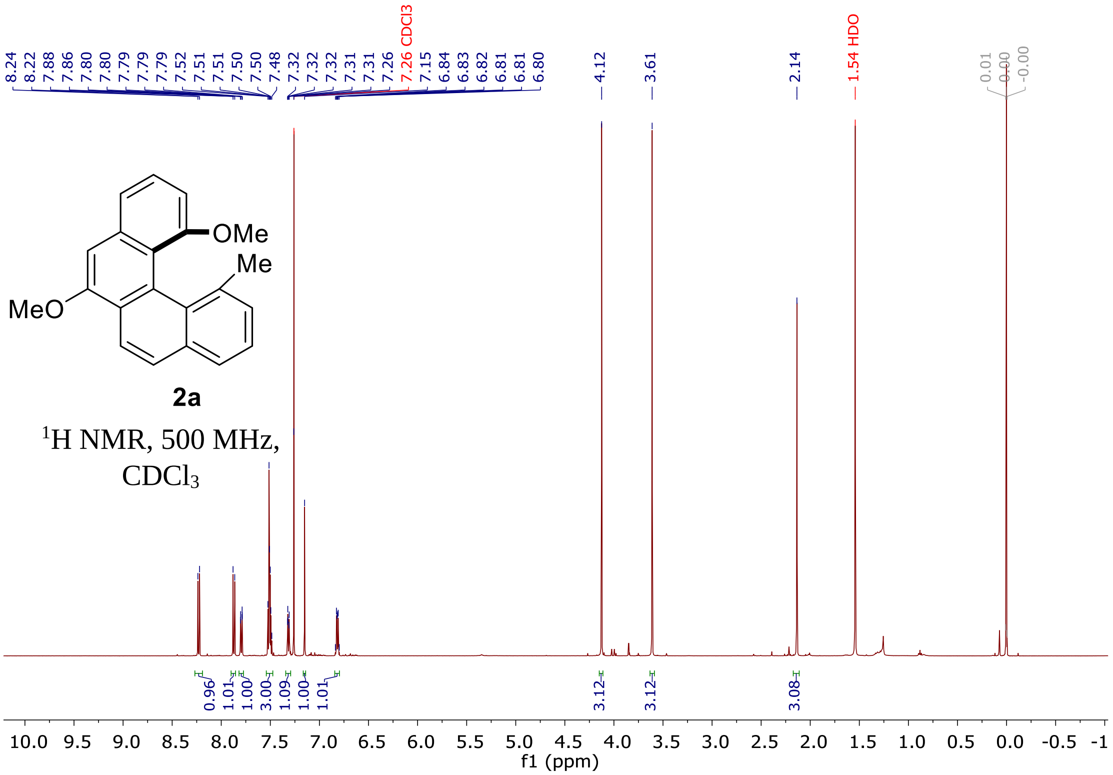
8.24 8.22 7.88 7.86 7.80 7.80 7.79 7.79 7.79 7.52 7.51 7.51 7.50 7.50 7.48 7.32 7.32 7.32 7.31 7.31 7.26 7.26 CDCl3 7.15 6.84 6.83 6.82 6.81 6.81 6.80
4.12
3.61
2.14
1.54 HDO
0.01 0.00 -0.00
OMe
Me
MeO
2a
^{1}H NMR, 500 MHz, $CDCl_3$
0.96 1.01 1.00 3.00 1.09 1.00 1.01
3.12 3.12 3.08
10.0 9.5 9.0 8.5 8.0 7.5 7.0 6.5 6.0 5.5 5.0 4.5 4.0 3.5 3.0 2.5 2.0 1.5 1.0 0.5 0.0 -0.5 -1
f1 (ppm)

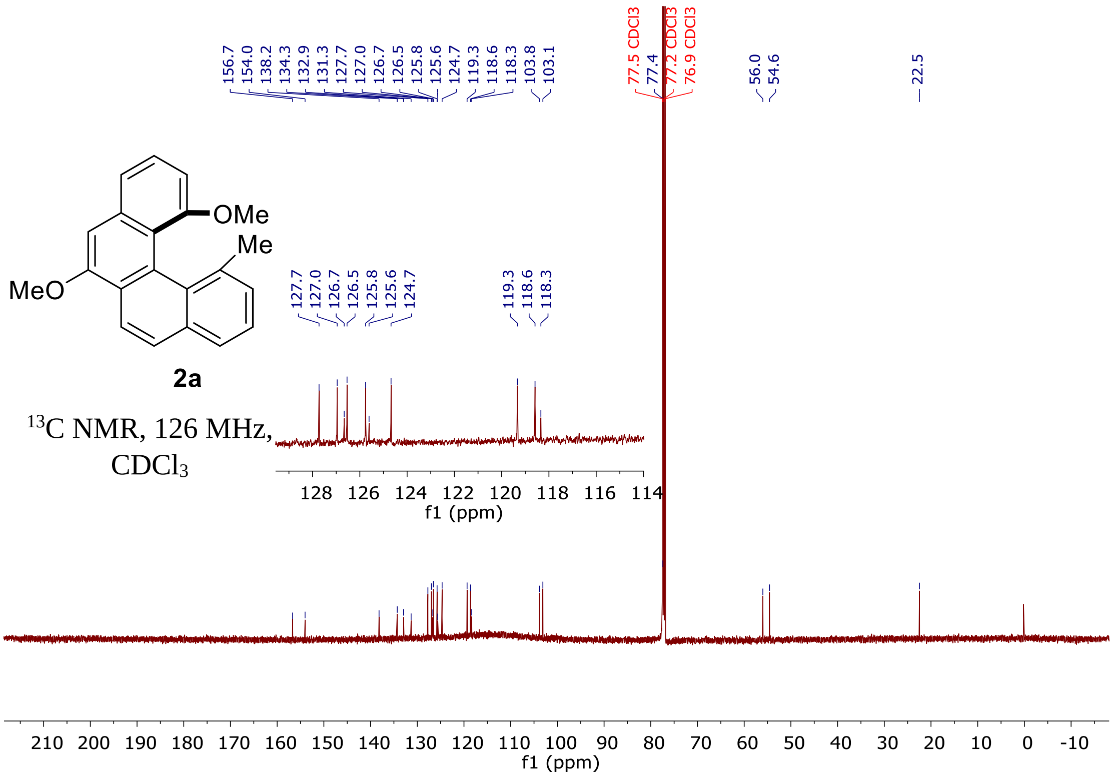
156.7 154.0 138.2 134.3 132.9 131.3 127.7 127.0 126.7 126.5 125.8 125.6 124.7 119.3 118.6 118.3 103.8 103.1
77.5 CDCl3 77.4 77.2 CDCl3 76.9 CDCl3
56.0 54.6
22.5
OMe
Me
MeO
2a
^{13}C NMR, 126 MHz, $CDCl_3$
127.7 127.0 126.7 126.5 125.8 125.6 124.7 119.3 118.6 118.3
128 126 124 122 120 118 116 114
f1 (ppm)
210 200 190 180 170 160 150 140 130 120 110 100 90 80 70 60 50 40 30 20 10 0 -10
f1 (ppm)

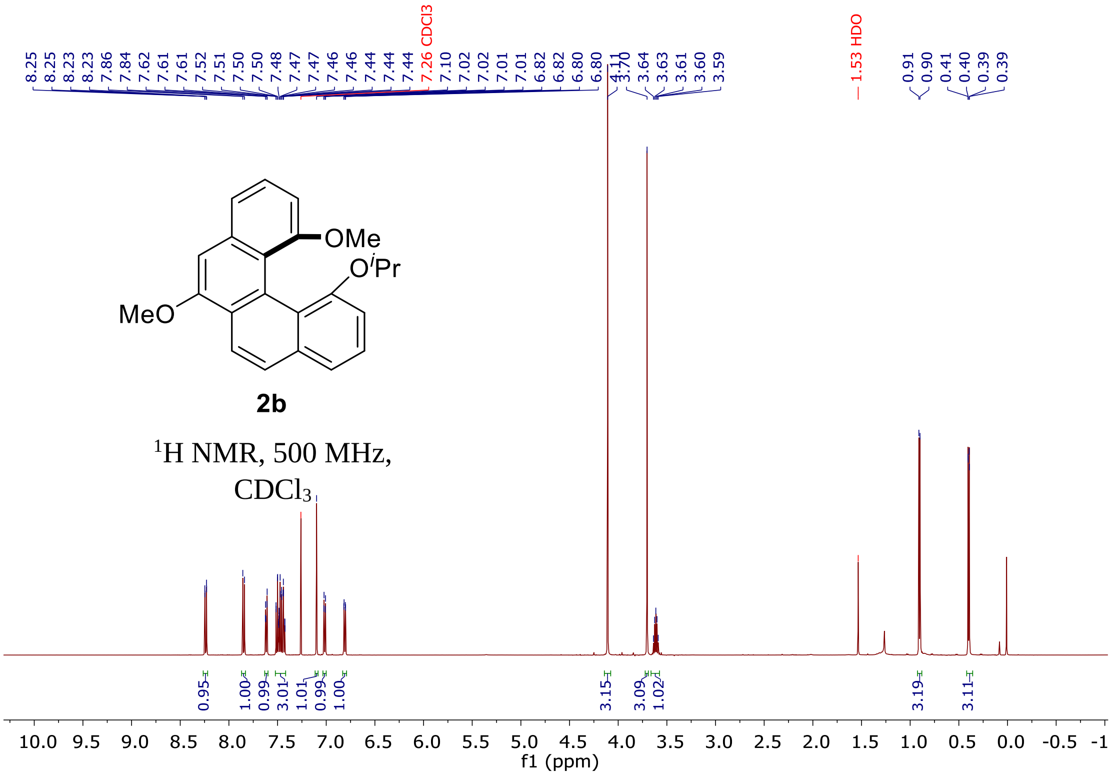

2b

^{1}H NMR, 500 MHz, $CDCl_3$

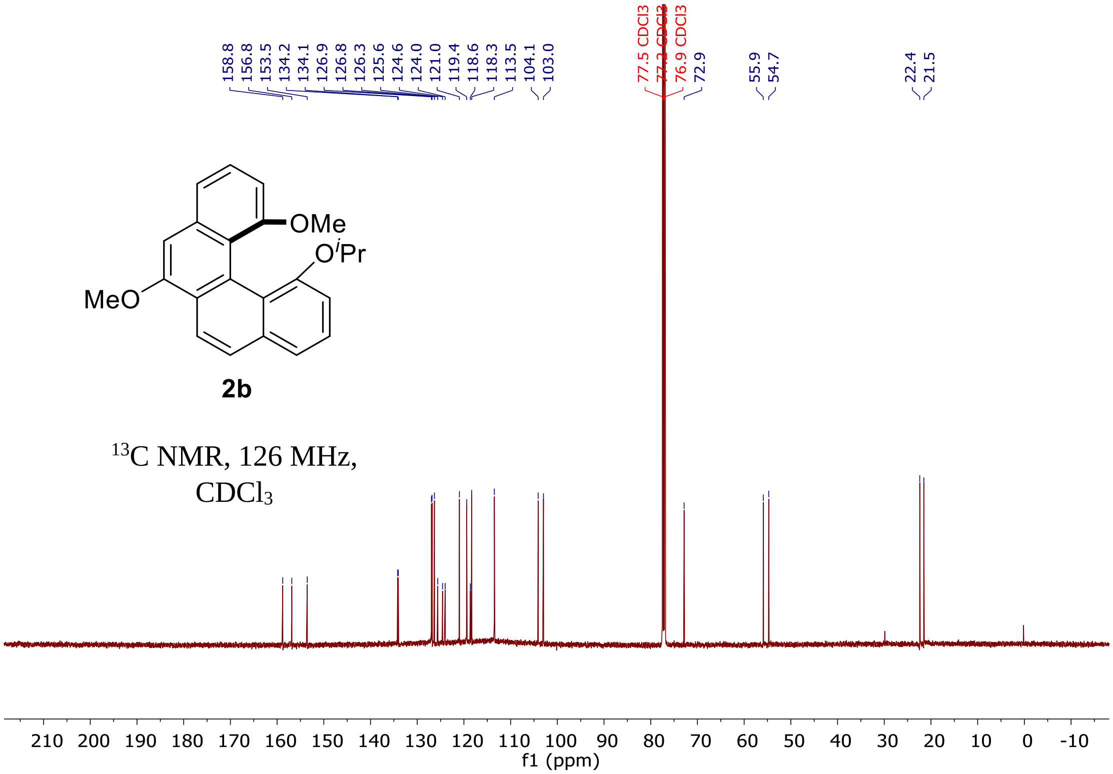

2b

^{13}C NMR, 126 MHz, $CDCl_3$

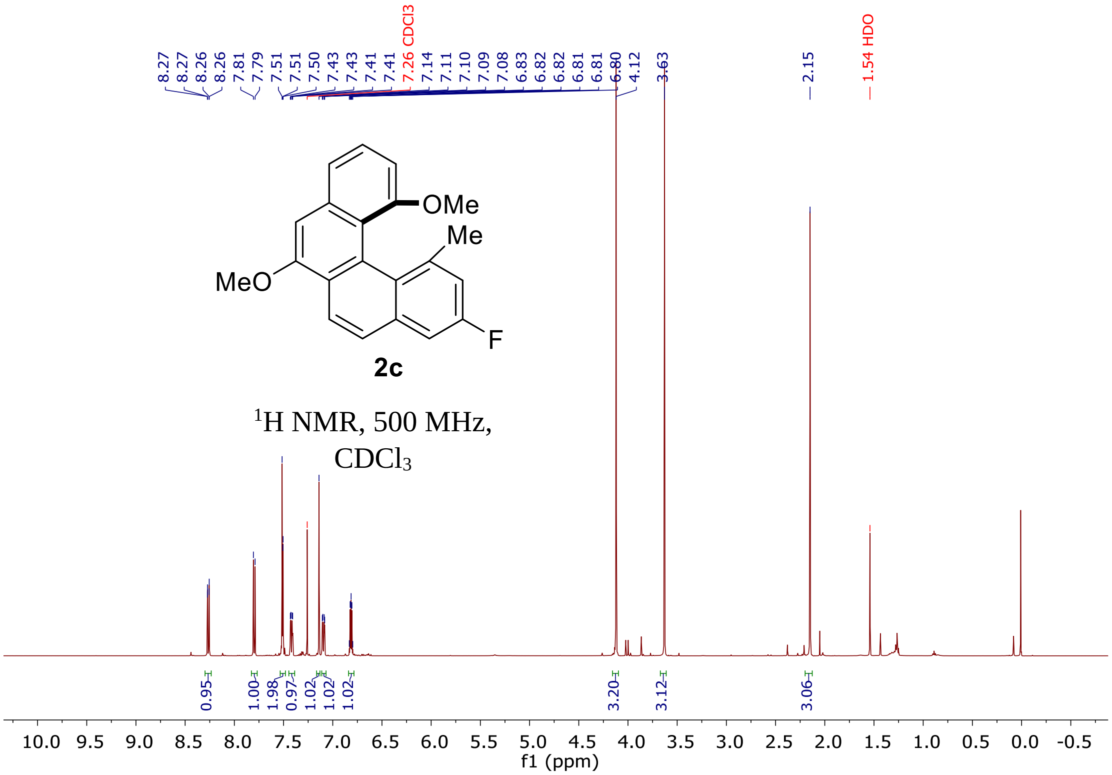
8.27
8.27
8.26
8.26
7.81
7.79
7.51
7.51
7.50
7.43
7.43
7.41
7.41
7.26 CDCl3
7.14
7.11
7.10
7.09
7.08
6.83
6.82
6.82
6.81
6.81
6.80
4.12
3.63
2.15
1.54 HDO
OMe
Me
MeO
F
2c
1H NMR, 500 MHz,
CDCl3
0.95
1.00
1.98
0.97
1.02
1.02
1.02
3.20
3.12
3.06
f1 (ppm)

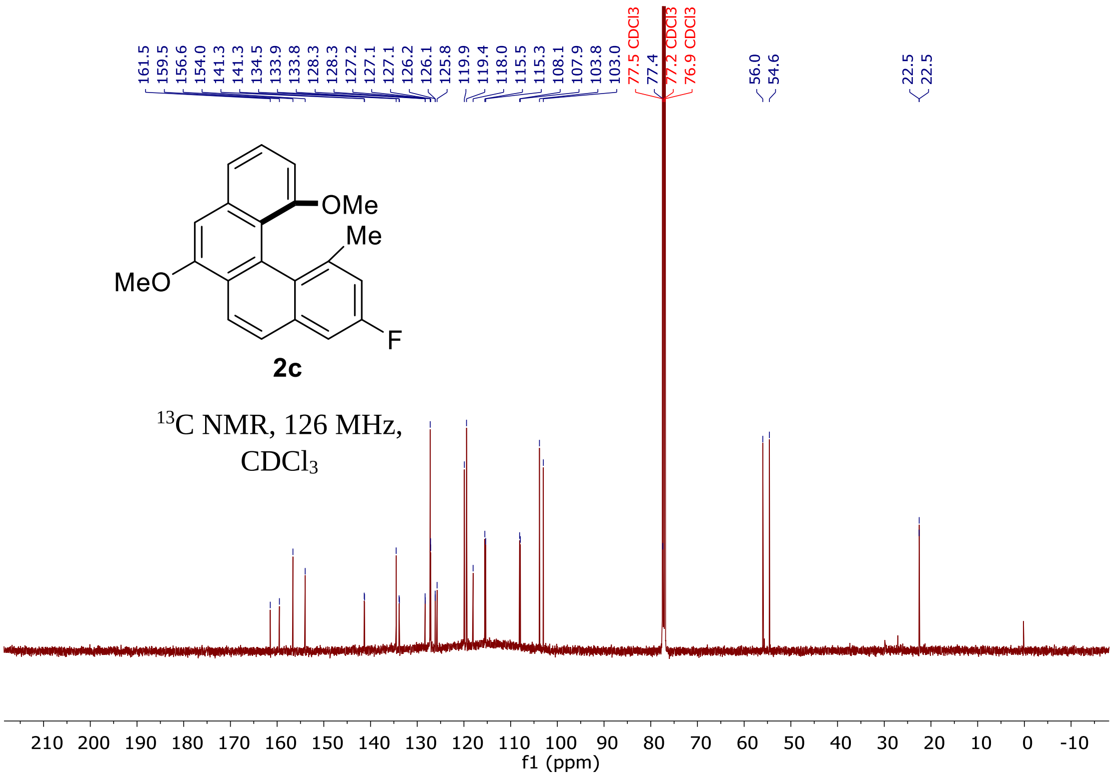
161.5
159.5
156.6
154.0
141.3
141.3
134.5
133.9
133.8
128.3
128.3
127.2
127.1
127.1
126.2
126.1
125.8
119.9
119.4
118.0
115.5
115.3
108.1
107.9
103.8
103.0
77.5 CDCl3
77.4
77.2 CDCl3
76.9 CDCl3
56.0
54.6
22.5
22.5
OMe
Me
MeO
F
2c
13C NMR, 126 MHz,
CDCl3
f1 (ppm)

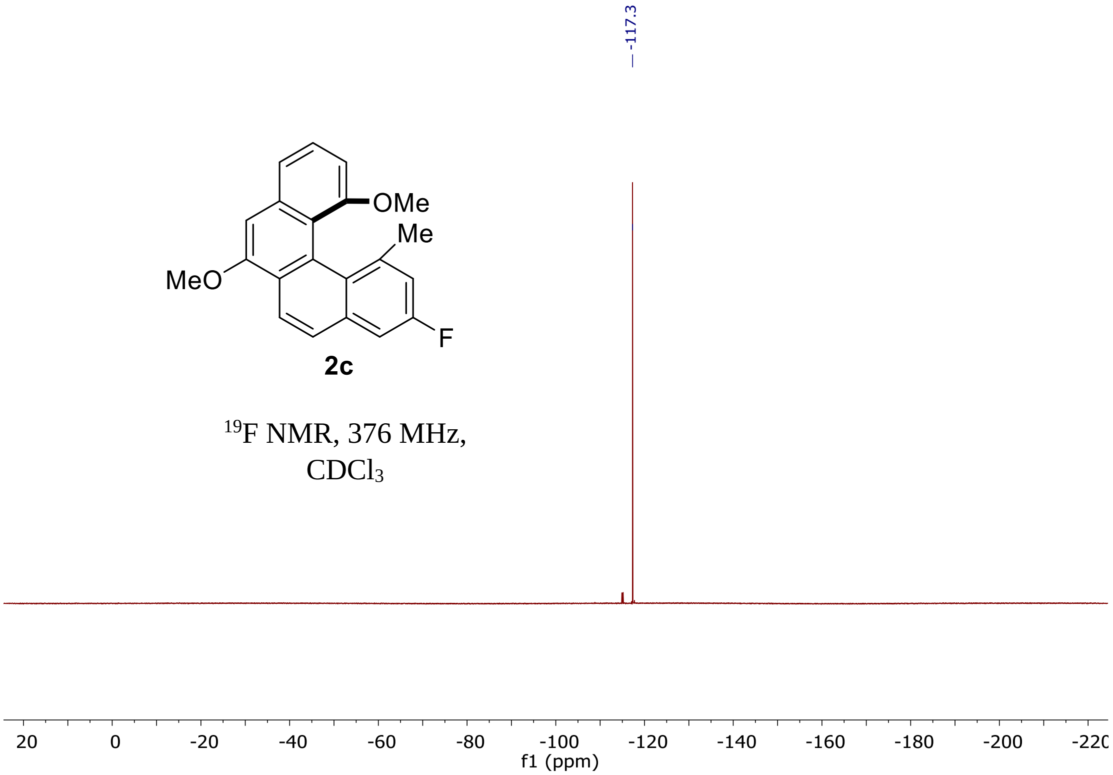
-117.3
OMe
Me
MeO
F
2c
19F NMR, 376 MHz,
CDCl3
20 0 -20 -40 -60 -80 -100 -120 -140 -160 -180 -200 -220
f1 (ppm)

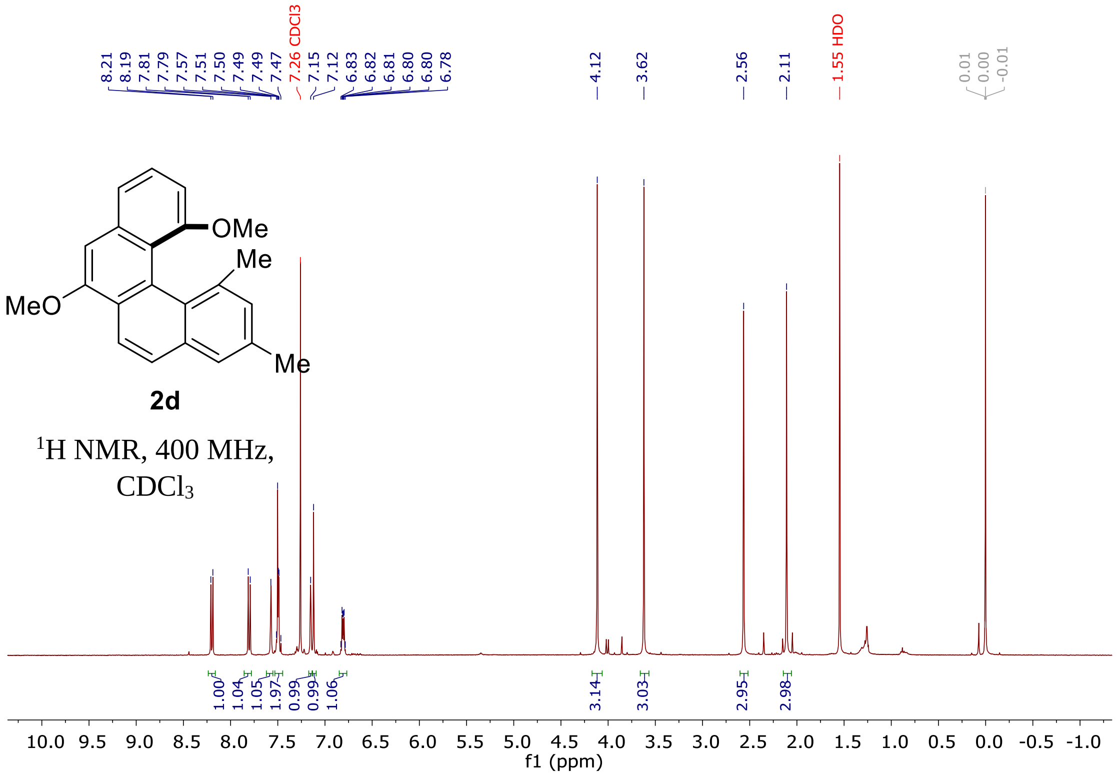
8.21
8.19
7.81
7.79
7.57
7.51
7.50
7.49
7.49
7.47
7.26 CDCl3
7.15
7.12
6.83
6.82
6.81
6.80
6.80
6.78
4.12
3.62
2.56
2.11
1.55 HDO
0.01
0.00
-0.01
OMe
Me
MeO
Me
2d
1H NMR, 400 MHz, CDCl3
1.00
1.04
1.05
1.97
0.99
0.99
1.06
3.14
3.03
2.95
2.98
10.0 9.5 9.0 8.5 8.0 7.5 7.0 6.5 6.0 5.5 5.0 4.5 4.0 3.5 3.0 2.5 2.0 1.5 1.0 0.5 0.0 -0.5 -1.0
f1 (ppm)

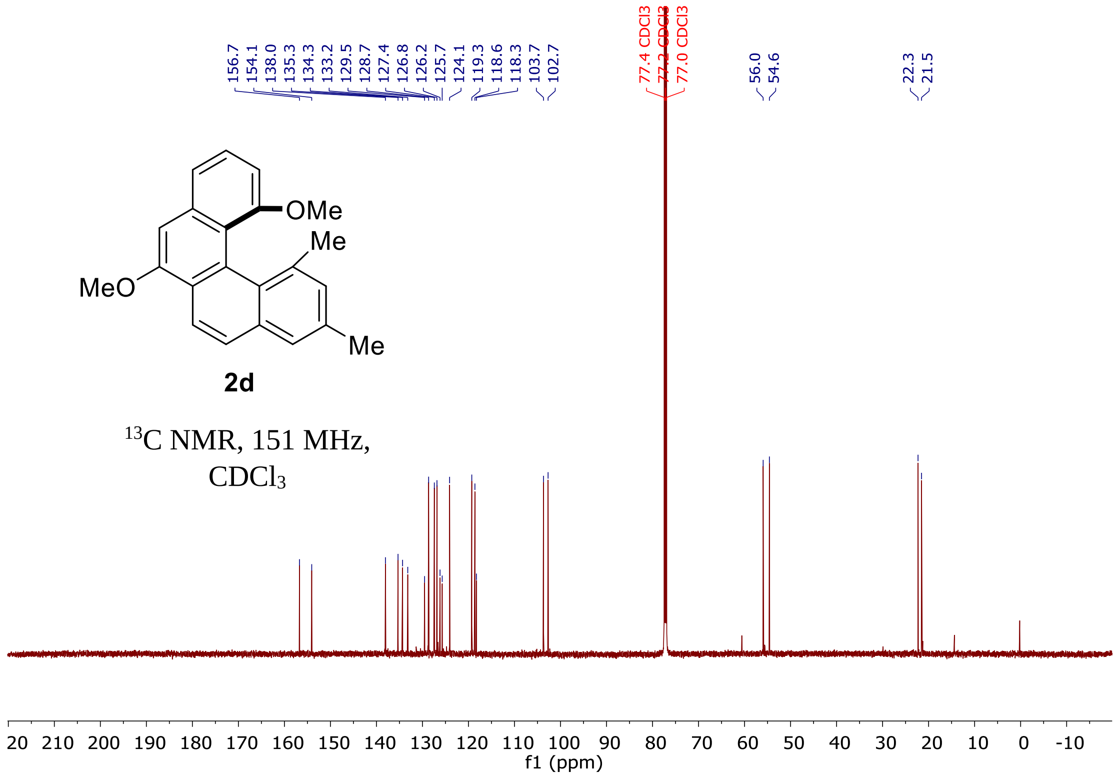
156.7
154.1
138.0
135.3
134.3
133.2
129.5
128.7
127.4
126.8
126.2
125.7
124.1
119.3
118.6
118.3
103.7
102.7
77.4 CDCl3
77.2 CDCl3
77.0 CDCl3
56.0
54.6
22.3
21.5
OMe
Me
MeO
Me
2d
13C NMR, 151 MHz, CDCl3
20 210 200 190 180 170 160 150 140 130 120 110 100 90 80 70 60 50 40 30 20 10 0 -10
f1 (ppm)

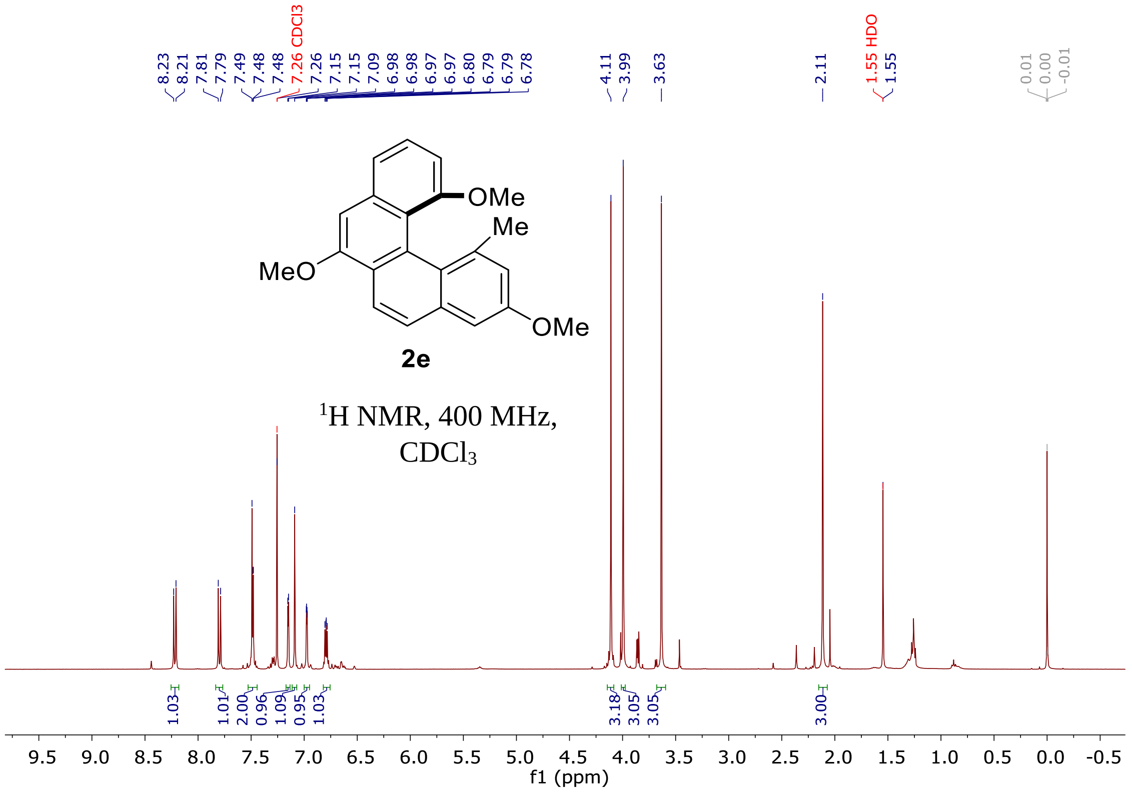
8.23
8.21
7.81
7.79
7.49
7.48
7.26 CDCl3
7.26
7.15
7.15
7.09
6.98
6.98
6.97
6.97
6.80
6.79
6.79
6.78
4.11
3.99
3.63
2.11
1.55 HDO
1.55
0.01
0.00
-0.01
OMe
Me
MeO
OMe
2e
^{1}H NMR, 400 MHz, $CDCl_3$
1.03
1.01
2.00
0.96
1.09
0.95
1.03
3.18
3.05
3.05
3.00
9.5 9.0 8.5 8.0 7.5 7.0 6.5 6.0 5.5 5.0 4.5 4.0 3.5 3.0 2.5 2.0 1.5 1.0 0.5 0.0 -0.5
f1 (ppm)

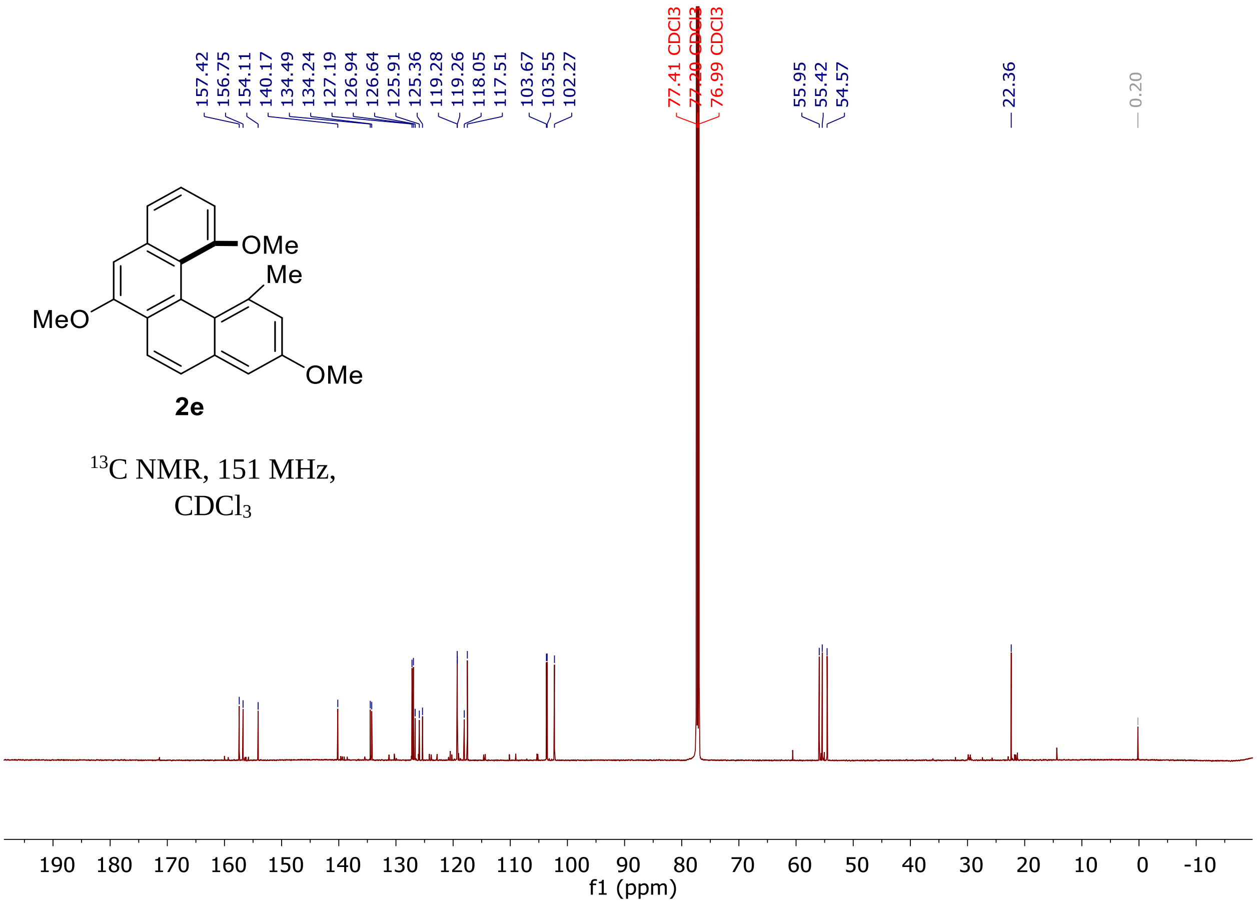
157.42
156.75
154.11
140.17
134.49
134.24
127.19
126.94
126.64
125.91
125.36
119.28
119.26
118.05
117.51
103.67
103.55
102.27
77.41 CDCl3
77.20 CDCl3
76.99 CDCl3
55.95
55.42
54.57
22.36
0.20
OMe
Me
MeO
OMe
2e
^{13}C NMR, 151 MHz, $CDCl_3$
190 180 170 160 150 140 130 120 110 100 90 80 70 60 50 40 30 20 10 0 -10
f1 (ppm)

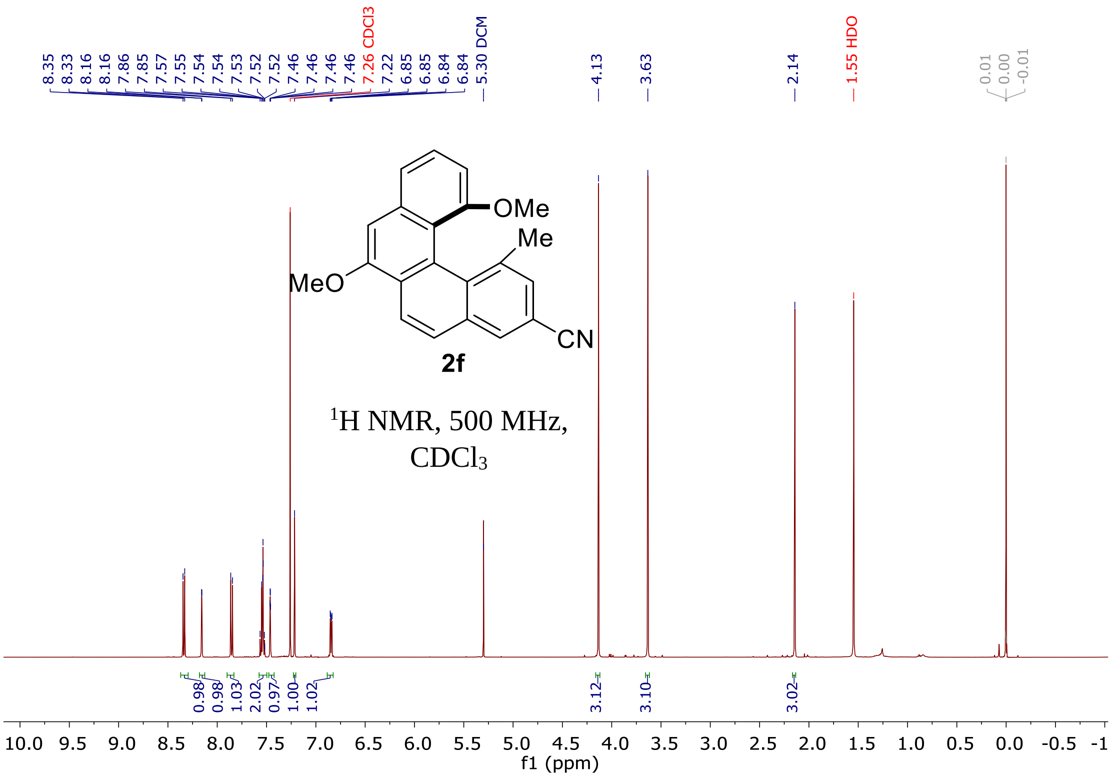
8.35 8.33 8.16 8.16 7.86 7.85 7.57 7.55 7.54 7.54 7.53 7.52 7.52 7.46 7.46 7.46 7.46 7.26 CDCl3 7.22 6.85 6.85 6.84 6.84
5.30 DCM
4.13
3.63
2.14
1.55 HDO
0.01 0.00 -0.01
OMe
Me
MeO
CN
2f
1H NMR, 500 MHz, CDCl3
0.98 0.98 1.03 2.02 0.97 1.00 1.02
3.12
3.10
3.02
f1 (ppm)

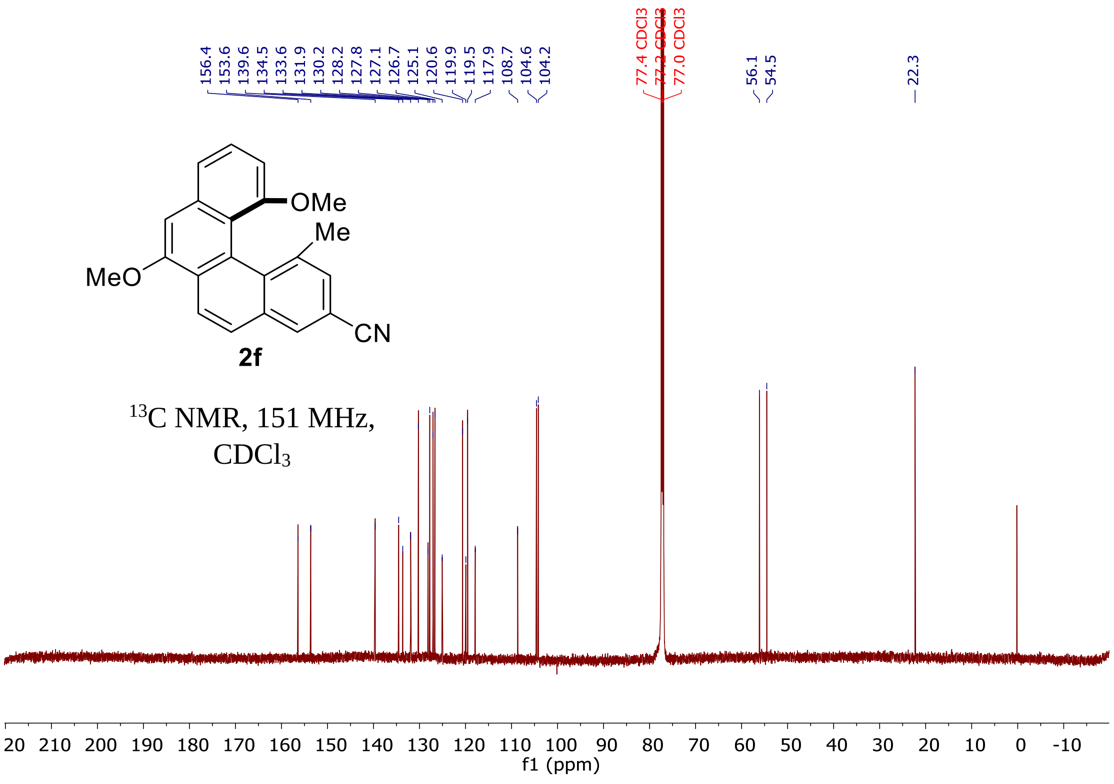
156.4 153.6 139.6 134.5 133.6 131.9 130.2 128.2 127.8 127.1 126.7 125.1 120.6 119.9 119.5 117.9 108.7 104.6 104.2
77.4 CDCl3 77.2 CDCl3 77.0 CDCl3
56.1 54.5
22.3
OMe
Me
MeO
CN
2f
13C NMR, 151 MHz, CDCl3
f1 (ppm)

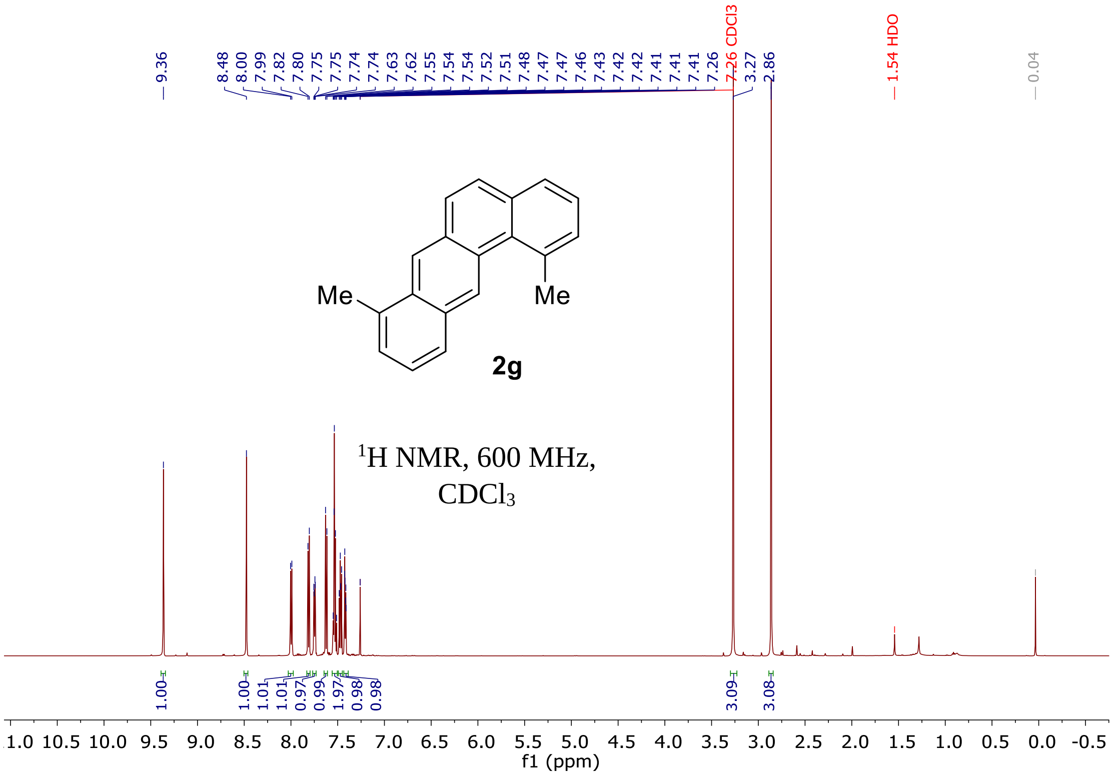
9.36
8.48
8.00
7.99
7.82
7.80
7.75
7.75
7.74
7.74
7.63
7.62
7.55
7.54
7.54
7.52
7.51
7.48
7.47
7.47
7.46
7.43
7.42
7.42
7.41
7.41
7.41
7.26
7.26 CDCl3
3.27
2.86
1.54 HDO
0.04
Me
Me
2g
^{1}H NMR, 600 MHz, $CDCl_3$
1.00
1.00
1.01
1.01
0.97
0.99
1.97
0.98
0.98
3.09
3.08
f1 (ppm)

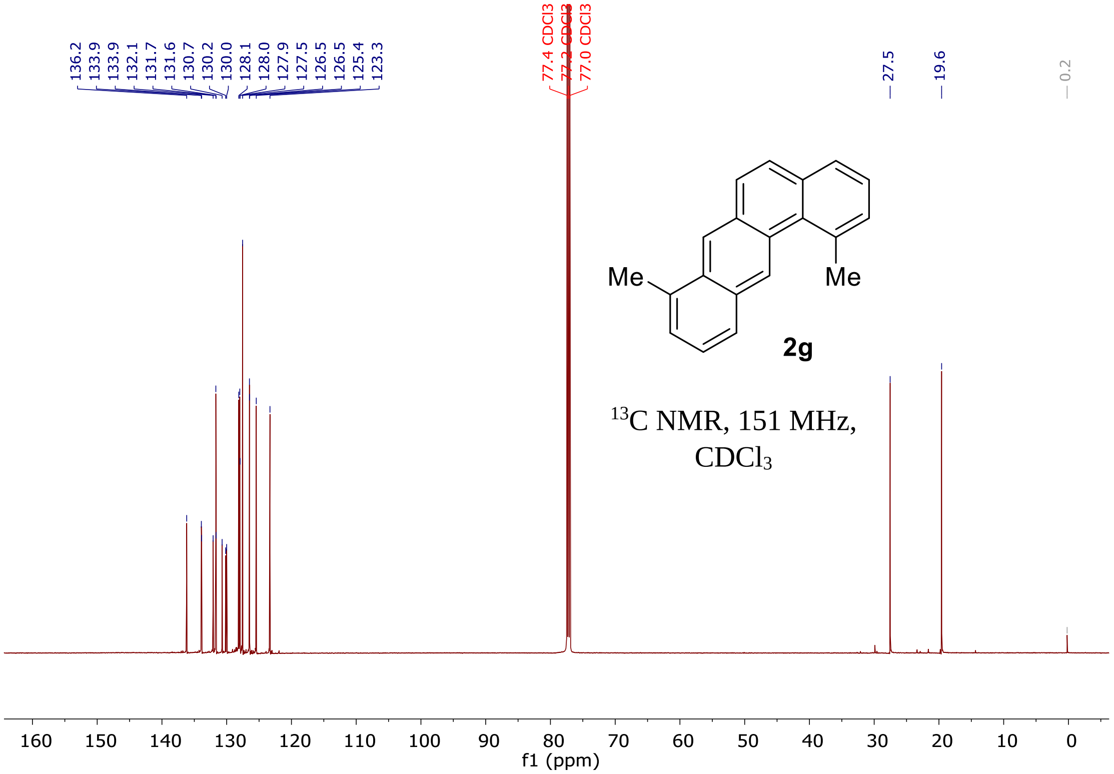
136.2
133.9
133.9
132.1
131.7
131.6
130.7
130.2
130.0
128.1
128.0
127.9
127.5
126.5
126.5
125.4
123.3
77.4 CDCl3
77.2 CDCl3
77.0 CDCl3
27.5
19.6
0.2
Me
Me
2g
^{13}C NMR, 151 MHz, $CDCl_3$
f1 (ppm)

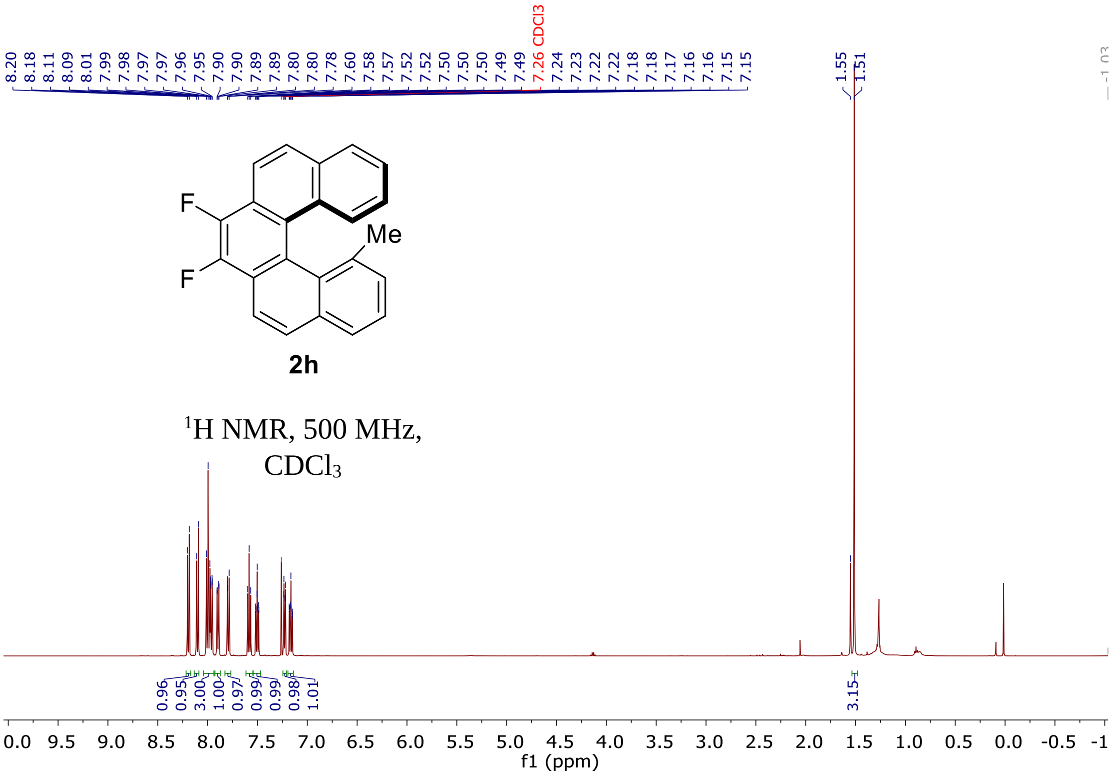
8.20 8.18 8.11 8.09 8.01 7.99 7.98 7.97 7.96 7.95 7.90 7.90 7.89 7.89 7.80 7.80 7.78 7.60 7.58 7.57 7.52 7.52 7.50 7.50 7.50 7.49 7.26 CDCl3 7.24 7.23 7.22 7.22 7.18 7.18 7.17 7.16 7.15 7.15
1.55 1.51
F
F
Me
2h
1H NMR, 500 MHz,
CDCl3
0.96 0.95 3.00 1.00 0.97 0.99 0.99 0.98 1.01
3.15
0.0 9.5 9.0 8.5 8.0 7.5 7.0 6.5 6.0 5.5 5.0 4.5 4.0 3.5 3.0 2.5 2.0 1.5 1.0 0.5 0.0 -0.5 -1
f1 (ppm)

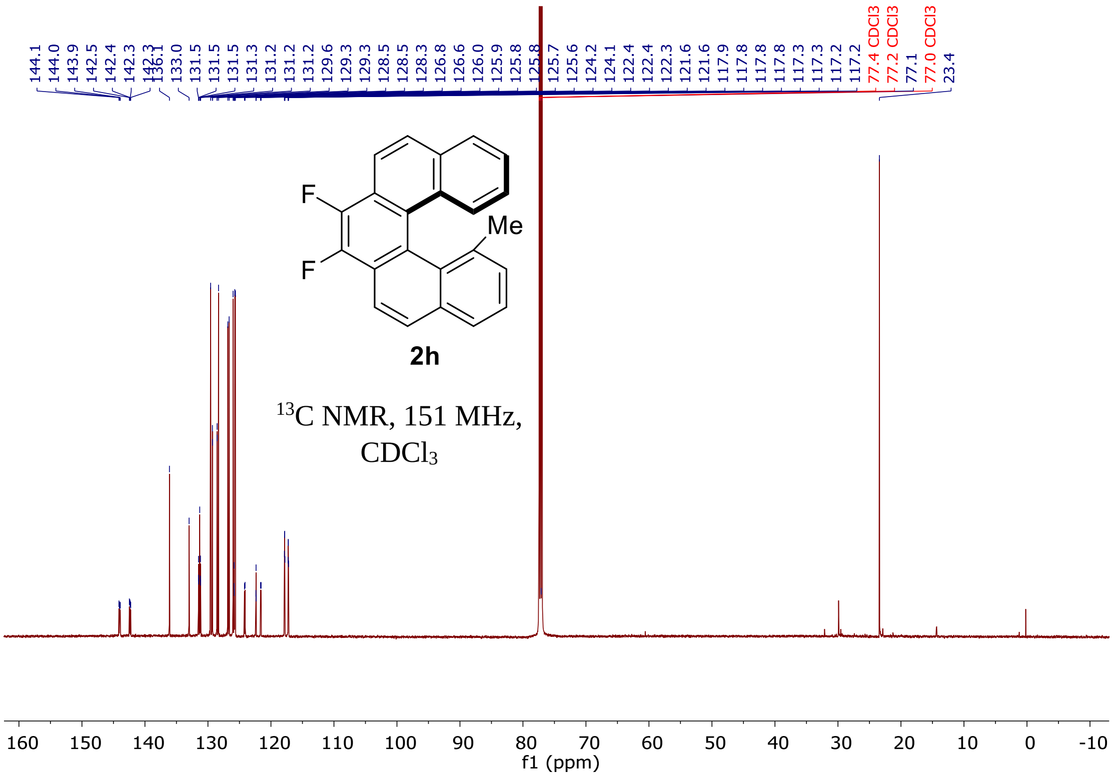
144.1 144.0 143.9 142.5 142.4 142.3 142.3 136.1 133.0 131.5 131.5 131.5 131.3 131.2 131.2 131.2 129.6 129.3 129.3 128.5 128.5 128.3 126.8 126.6 126.0 125.9 125.8 125.8 125.7 125.6 124.2 124.1 122.4 122.4 122.3 121.6 121.6 117.9 117.8 117.8 117.8 117.3 117.3 117.2 117.2 77.4 CDCl3 77.2 CDCl3 77.1 77.0 CDCl3 23.4
F
F
Me
2h
13C NMR, 151 MHz,
CDCl3
160 150 140 130 120 110 100 90 80 70 60 50 40 30 20 10 0 -10
f1 (ppm)

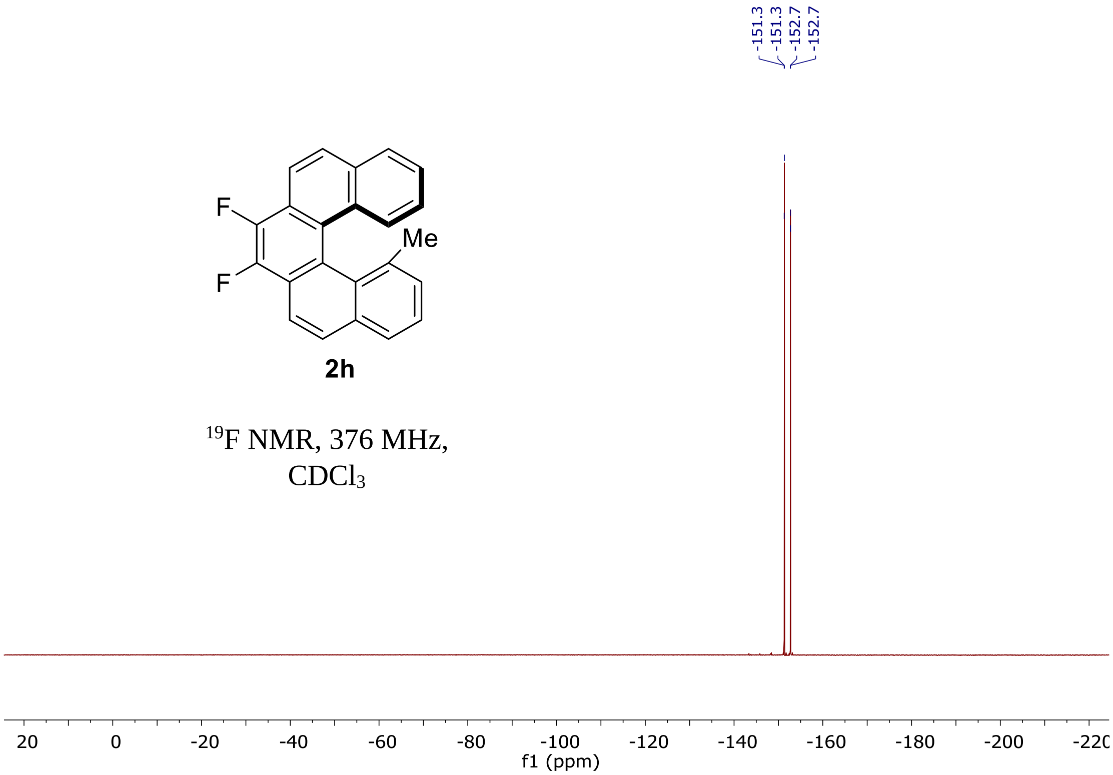

2h

^{19}F NMR, 376 MHz, $CDCl_3$

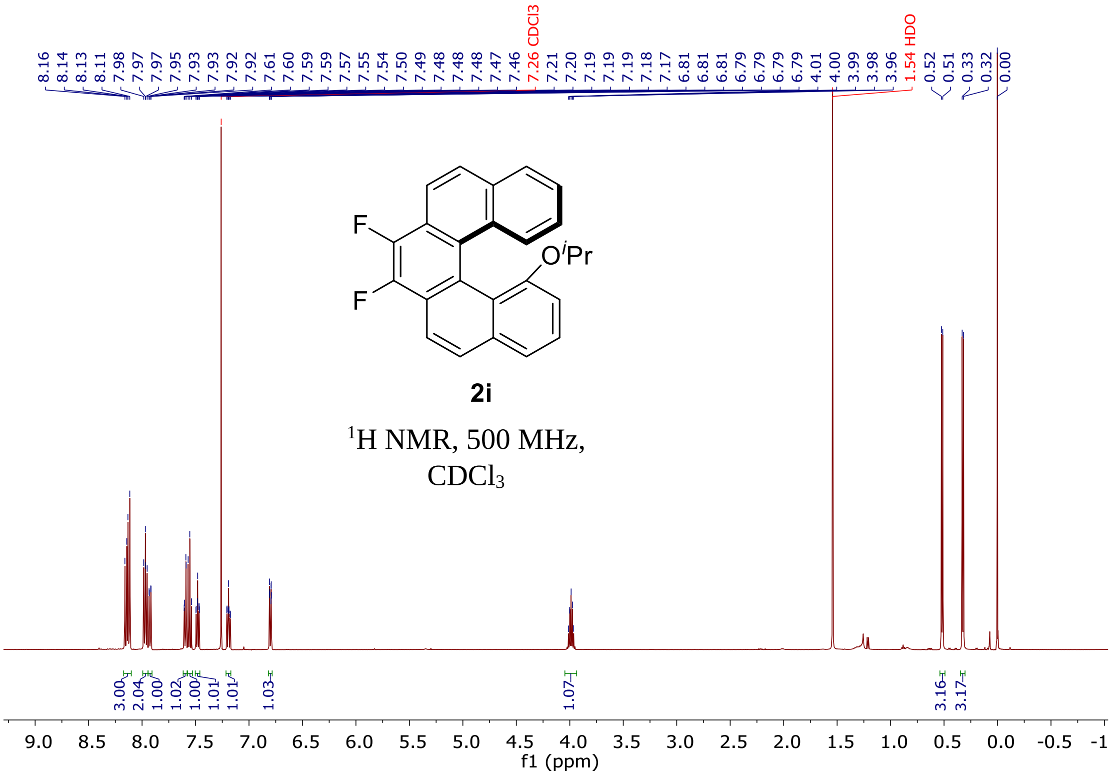

8.16 8.14 8.13 8.11 7.98 7.97 7.97 7.95 7.93 7.93 7.92 7.92 7.61 7.60 7.59 7.59 7.57 7.55 7.54 7.50 7.49 7.48 7.48 7.48 7.47 7.46 7.26 CDCl3 7.21 7.20 7.19 7.19 7.19 7.18 7.17 6.81 6.81 6.81 6.79 6.79 6.79 6.79 4.01 4.00 3.99 3.98 3.96 1.54 HDO 0.52 0.51 0.33 0.32 0.00
F
F
O^iPr
2i
1H NMR, 500 MHz,
CDCl3
3.00 2.04 1.00 1.02 1.00 1.01 1.01 1.03 1.07 3.16 3.17
9.0 8.5 8.0 7.5 7.0 6.5 6.0 5.5 5.0 4.5 4.0 3.5 3.0 2.5 2.0 1.5 1.0 0.5 0.0 -0.5 -1
f1 (ppm)

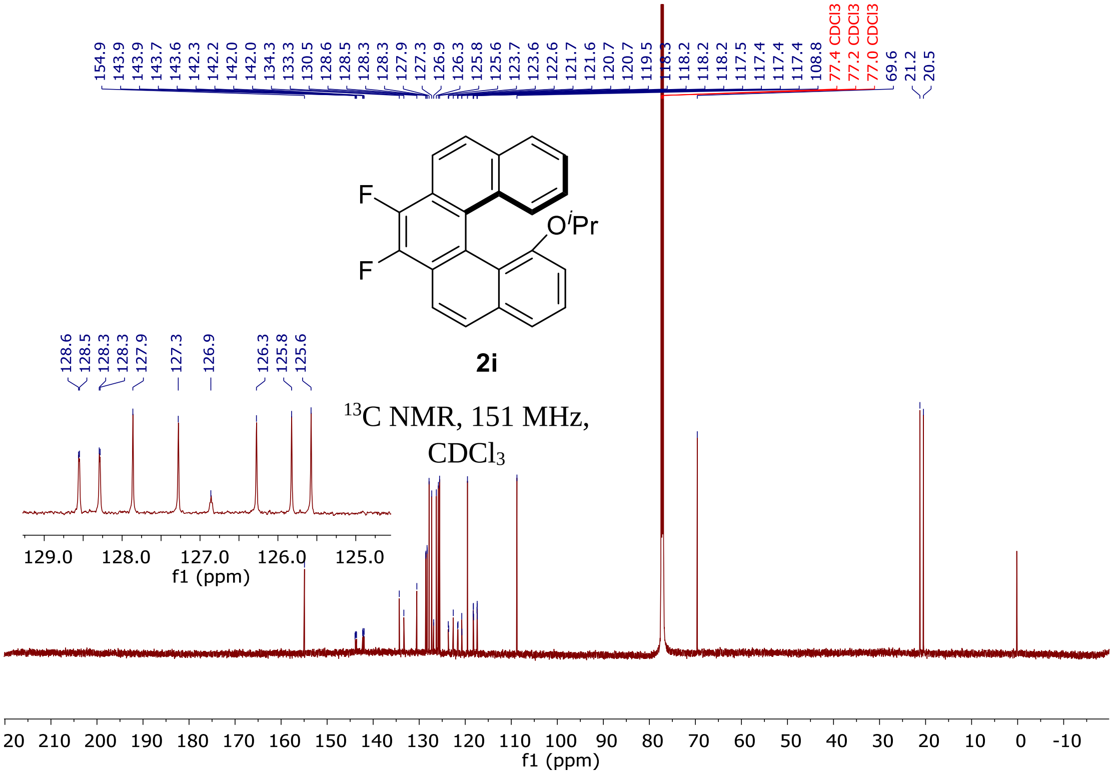

154.9 143.9 143.9 143.7 143.6 142.3 142.2 142.0 142.0 134.3 133.3 130.5 128.6 128.5 128.3 128.3 127.9 127.3 126.9 126.3 125.8 125.6 123.7 123.6 122.6 121.7 121.6 120.7 120.7 119.5 118.3 118.2 118.2 118.2 117.5 117.4 117.4 117.4 108.8 77.4 CDCl3 77.2 CDCl3 77.0 CDCl3 69.6 21.2 20.5
F
F
O^iPr
2i
13C NMR, 151 MHz,
CDCl3
128.6 128.5 128.3 128.3 127.9 127.3 126.9 126.3 125.8 125.6
129.0 128.0 127.0 126.0 125.0
f1 (ppm)
20 210 200 190 180 170 160 150 140 130 120 110 100 90 80 70 60 50 40 30 20 10 0 -10
f1 (ppm)

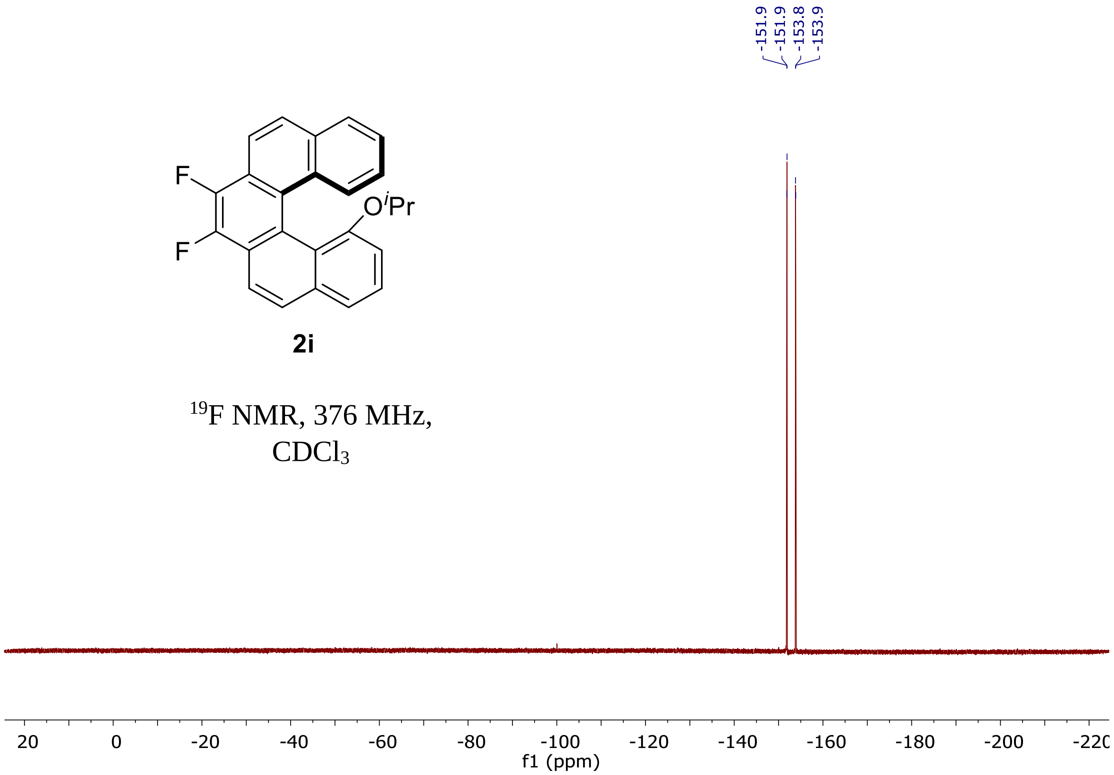

2i

^{19}F NMR, 376 MHz, $CDCl_3$

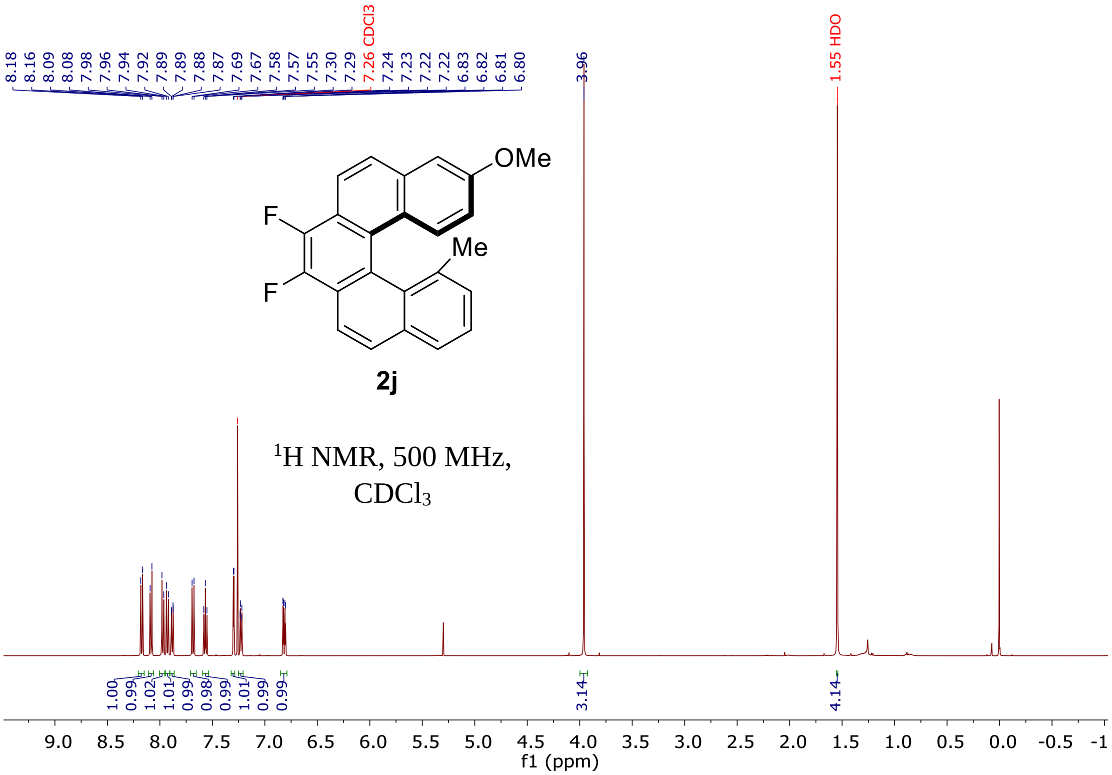
8.18 8.16 8.09 8.08 7.98 7.96 7.94 7.92 7.89 7.89 7.88 7.87 7.69 7.67 7.58 7.57 7.55 7.30 7.29 7.26 CDCl3 7.24 7.23 7.22 7.22 6.83 6.82 6.81 6.80
3.96
1.55 HDO
OMe
F
F
Me
2j
^{1}H NMR, 500 MHz, $CDCl_3$
1.00 0.99 1.02 1.01 0.99 0.98 0.99 1.01 0.99 0.99
3.14
4.14
f1 (ppm)

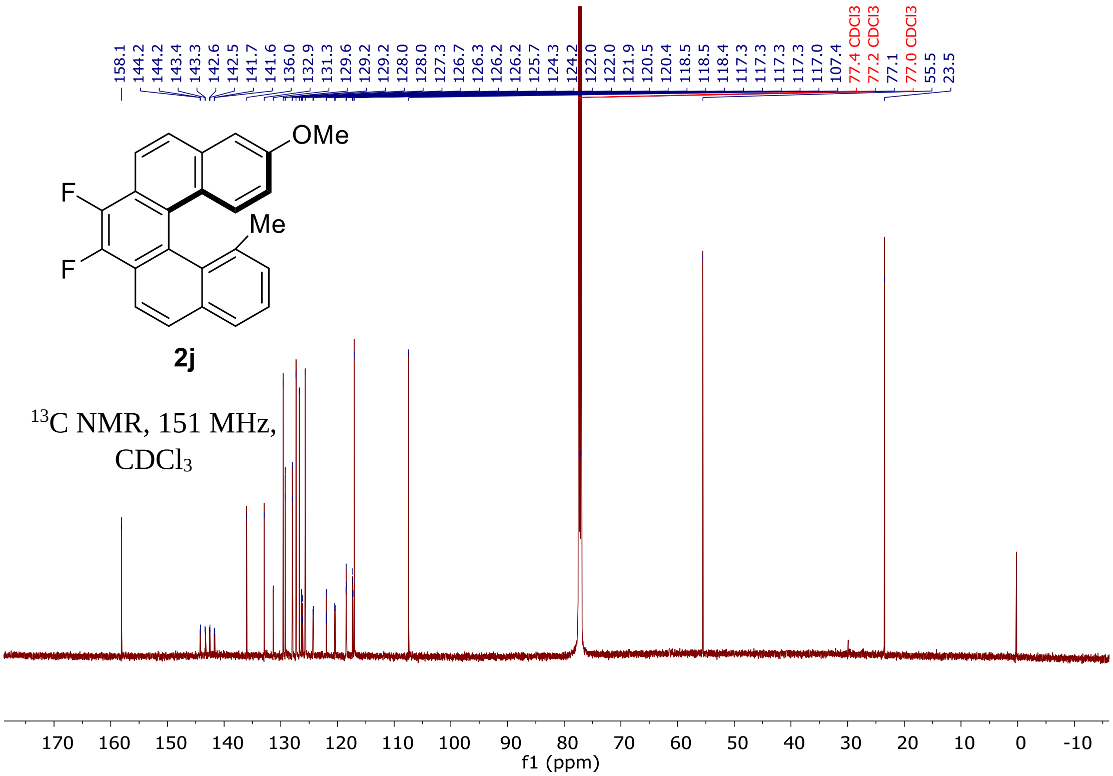
158.1 144.2 144.2 143.4 143.3 142.6 142.5 141.7 141.6 136.0 132.9 131.3 129.6 129.2 129.2 128.0 128.0 127.3 126.7 126.3 126.2 126.2 125.7 124.3 124.2 122.0 122.0 121.9 120.5 120.4 118.5 118.5 118.4 117.3 117.3 117.3 117.3 117.0 107.4 77.4 CDCl3 77.2 CDCl3 77.1 77.0 CDCl3 55.5 23.5
OMe
F
F
Me
2j
^{13}C NMR, 151 MHz, $CDCl_3$
f1 (ppm)

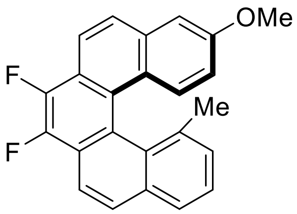

2j

^{19}F NMR, 376 MHz, $CDCl_3$

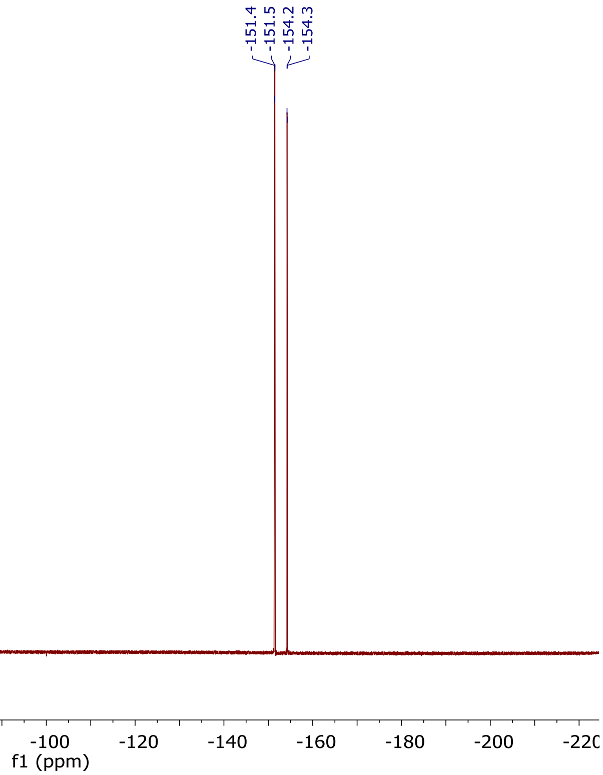

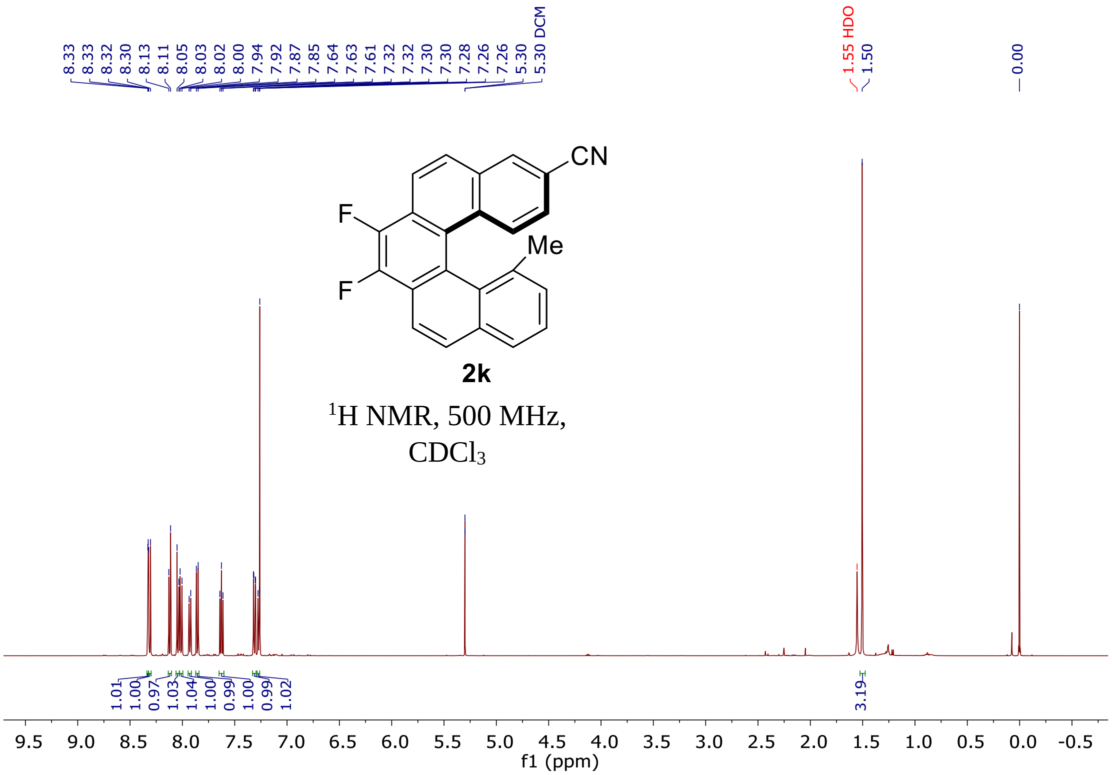
8.33
8.33
8.32
8.30
8.13
8.11
8.05
8.03
8.02
8.00
7.94
7.92
7.87
7.85
7.64
7.63
7.61
7.32
7.32
7.30
7.30
7.28
7.26
7.26
5.30
5.30 DCM
1.55 HDO
1.50
0.00
CN
F
F
Me
2k
^{1}H NMR, 500 MHz,
$CDCl_3$
1.01
1.00
0.97
1.03
1.04
1.00
0.99
1.00
0.99
1.02
3.19
9.5 9.0 8.5 8.0 7.5 7.0 6.5 6.0 5.5 5.0 4.5 4.0 3.5 3.0 2.5 2.0 1.5 1.0 0.5 0.0 -0.5
f1 (ppm)

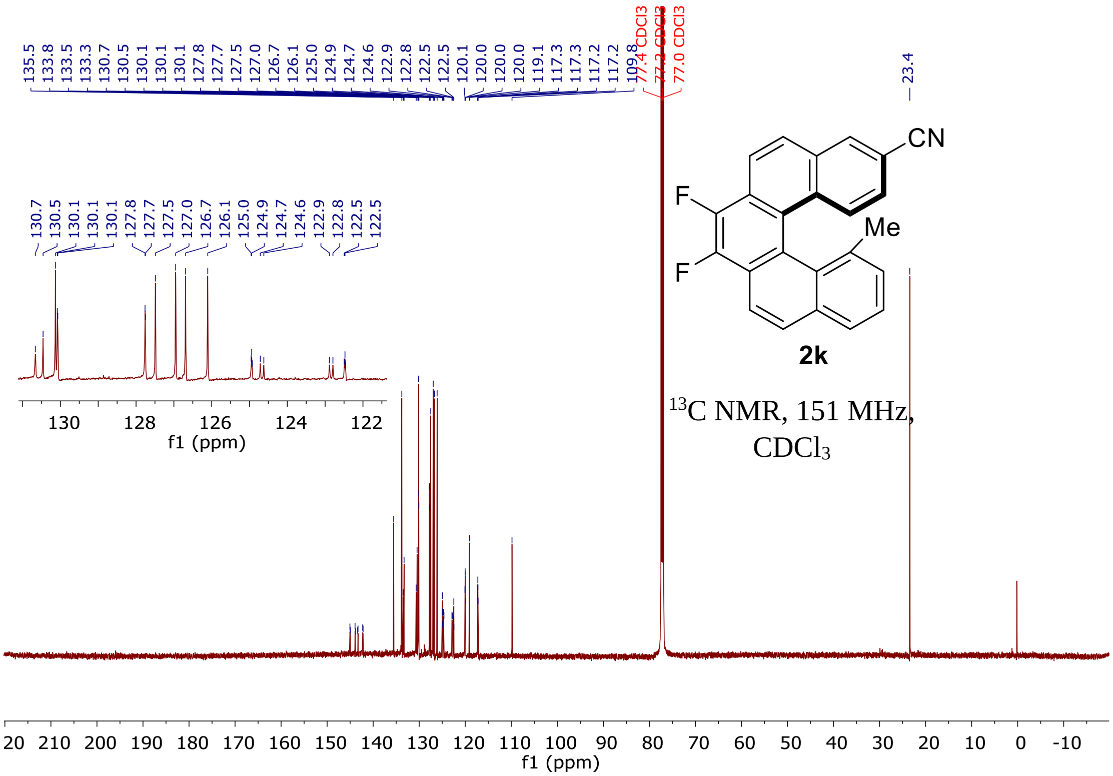
135.5
133.8
133.5
133.3
130.7
130.5
130.1
130.1
130.1
127.8
127.7
127.5
127.0
126.7
126.1
125.0
124.9
124.7
124.6
122.9
122.8
122.5
122.5
120.1
120.0
120.0
120.0
119.1
117.3
117.3
117.2
117.2
109.8
77.4 CDCl3
77.2 CDCl3
77.0 CDCl3
23.4
CN
F
F
Me
2k
^{13}C NMR, 151 MHz,
$CDCl_3$
130 128 126 124 122
f1 (ppm)
20 210 200 190 180 170 160 150 140 130 120 110 100 90 80 70 60 50 40 30 20 10 0 -10
f1 (ppm)

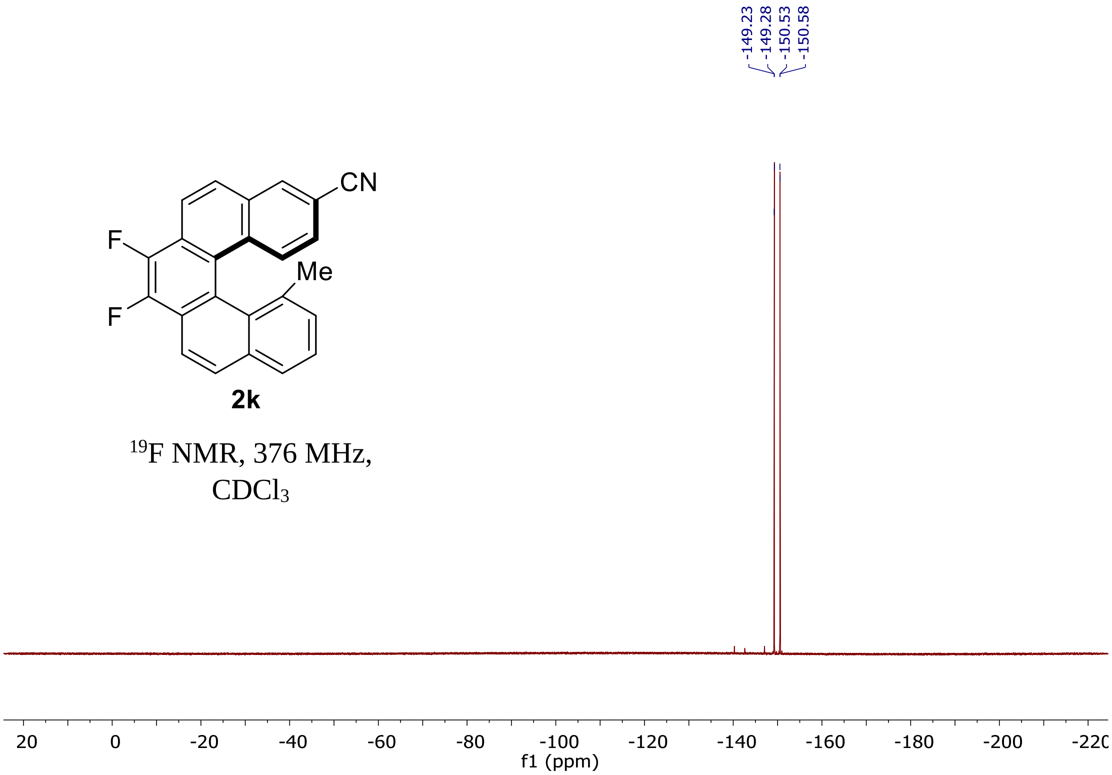

2k

^{19}F NMR, 376 MHz, $CDCl_3$

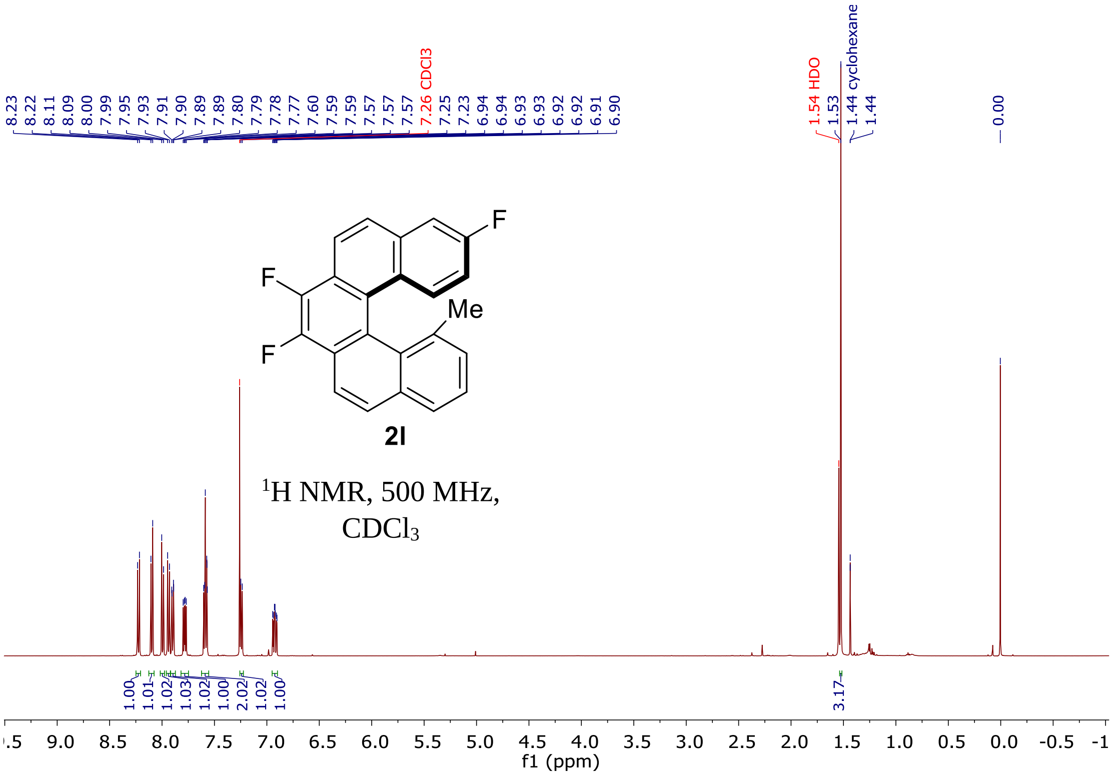
8.23 8.22 8.11 8.09 8.00 7.99 7.95 7.93 7.91 7.90 7.89 7.89 7.80 7.79 7.78 7.77 7.60 7.59 7.59 7.57 7.57 7.57 7.26 CDCl3 7.25 7.23 6.94 6.94 6.93 6.93 6.92 6.92 6.91 6.90
1.54 HDO
1.53
1.44 cyclohexane
1.44
0.00
F
F
F
Me
2l
^{1}H NMR, 500 MHz, CDCl$_3$
1.00 1.01 1.02 1.03 1.02 1.00 2.02 1.02 1.00
3.17
f1 (ppm)

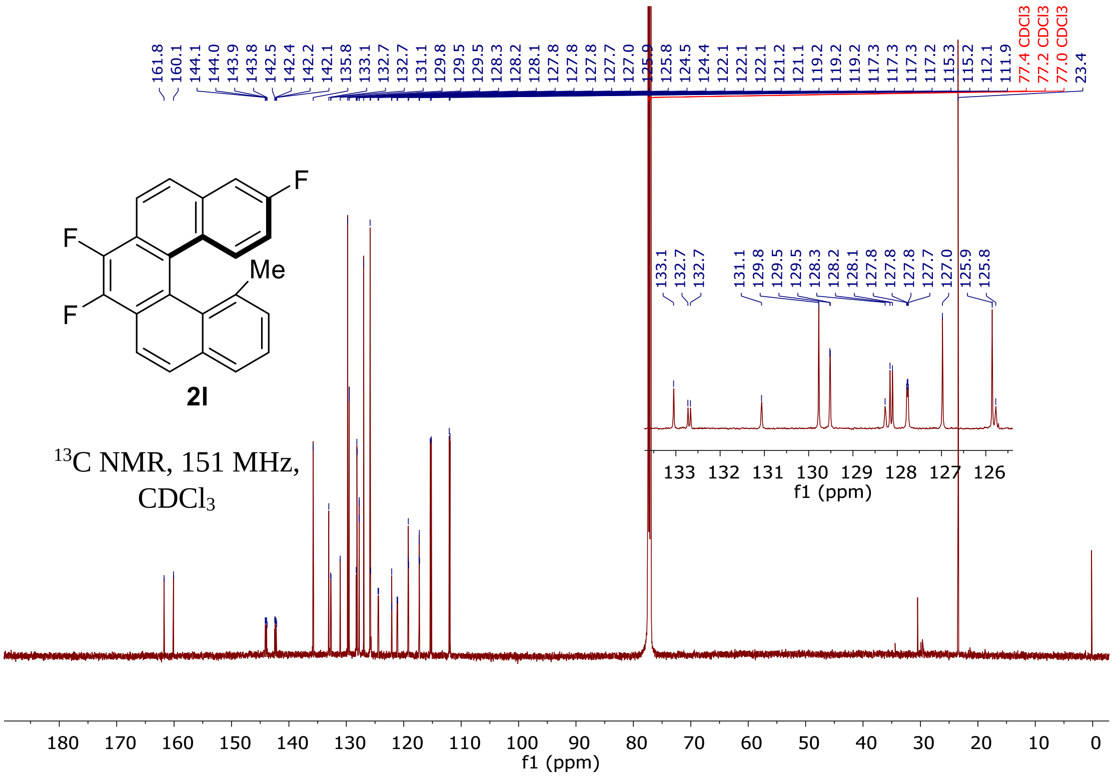
161.8 160.1 144.1 144.0 143.9 143.8 142.5 142.4 142.2 142.1 135.8 133.1 132.7 132.7 131.1 129.8 129.5 129.5 128.3 128.2 128.1 127.8 127.8 127.8 127.7 127.0 125.9 125.8 124.5 124.4 122.1 122.1 122.1 121.2 121.1 119.2 119.2 119.2 117.3 117.3 117.3 117.2 115.3 115.2 112.1 111.9
77.4 CDCl3 77.2 CDCl3 77.0 CDCl3
23.4
F
F
F
Me
2l
^{13}C NMR, 151 MHz, CDCl$_3$
f1 (ppm)

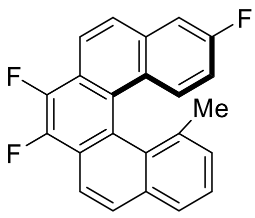

2l

^{19}F NMR, 376 MHz, $CDCl_3$

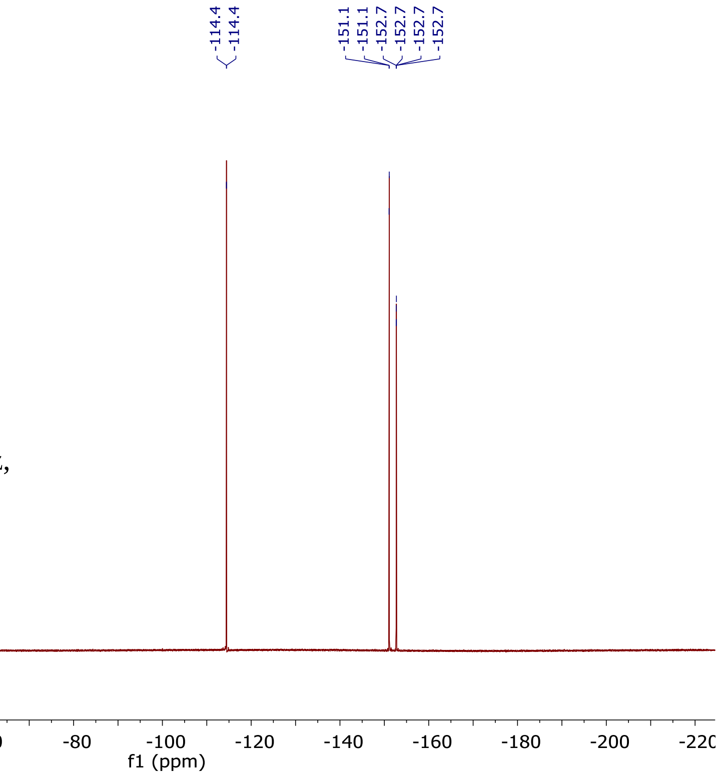

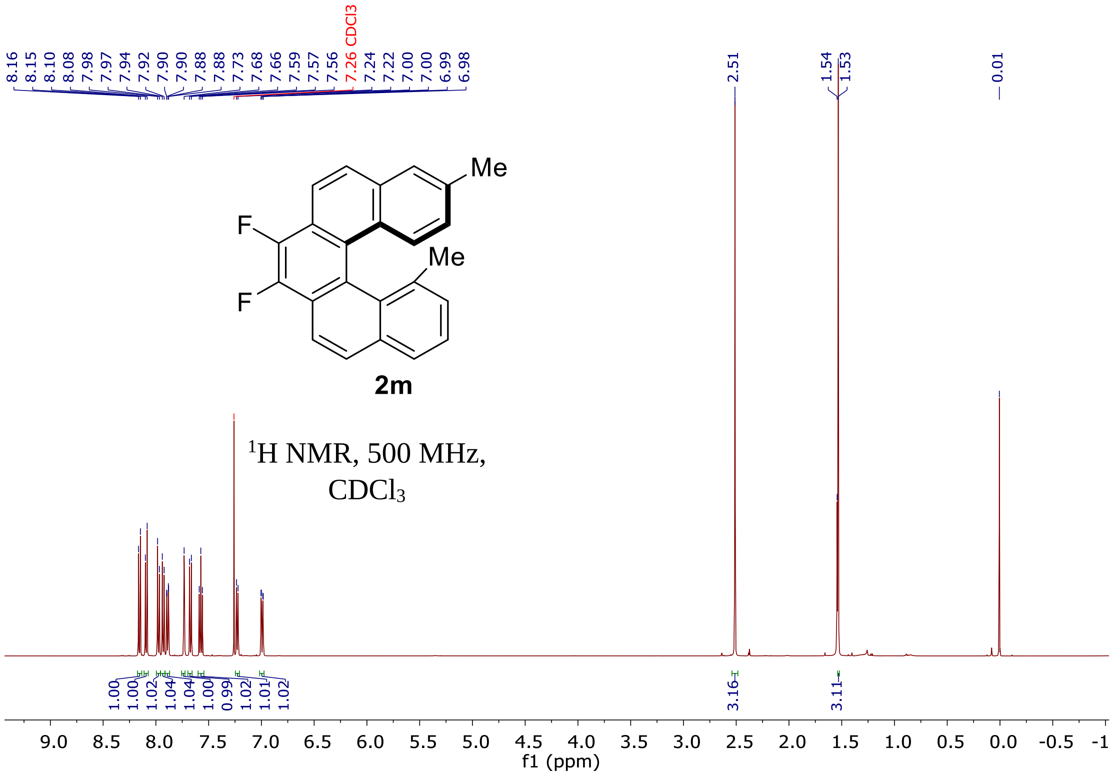

8.16
8.15
8.10
8.08
7.98
7.97
7.94
7.92
7.90
7.90
7.88
7.88
7.73
7.68
7.66
7.59
7.57
7.56
7.26 CDCl3
7.24
7.22
7.00
7.00
6.99
6.98
2.51
1.54
1.53
0.01
Me
F
Me
F
2m
¹H NMR, 500 MHz,
CDCl₃
1.00
1.00
1.02
1.04
1.04
1.00
0.99
1.02
1.01
1.02
3.16
3.11
9.0 8.5 8.0 7.5 7.0 6.5 6.0 5.5 5.0 4.5 4.0 3.5 3.0 2.5 2.0 1.5 1.0 0.5 0.0 -0.5 -1
f1 (ppm)

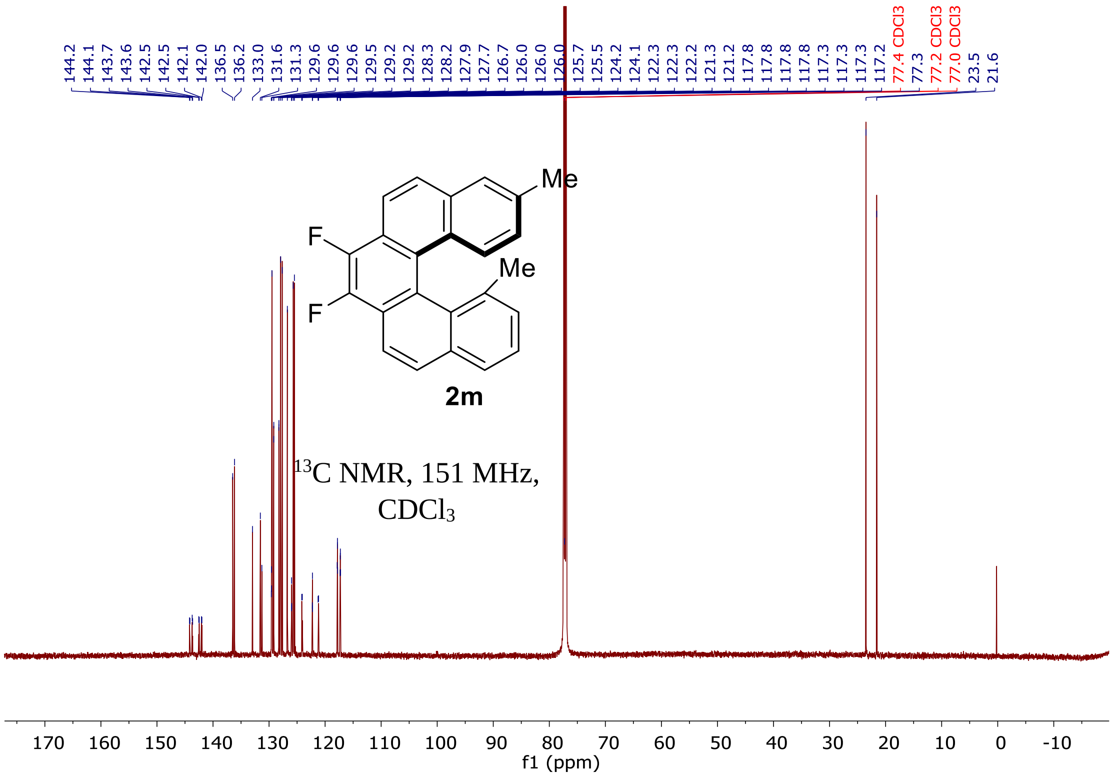

144.2
144.1
143.7
143.6
142.5
142.5
142.1
142.0
136.5
136.2
133.0
131.6
131.3
129.6
129.6
129.5
129.2
129.2
128.3
128.2
127.9
127.7
126.7
126.0
126.0
126.0
125.7
125.5
124.2
124.1
122.3
122.3
122.2
121.3
121.2
117.8
117.8
117.8
117.8
117.3
117.3
117.3
117.2
77.4 CDCl3
77.3
77.2 CDCl3
77.0 CDCl3
23.5
21.6
Me
F
Me
F
2m
¹³C NMR, 151 MHz,
CDCl₃
170 160 150 140 130 120 110 100 90 80 70 60 50 40 30 20 10 0 -10
f1 (ppm)

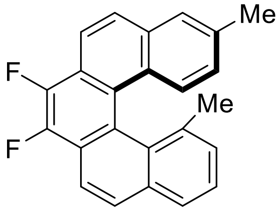

2m

^{19}F NMR, 376 MHz,
$CDCl_3$

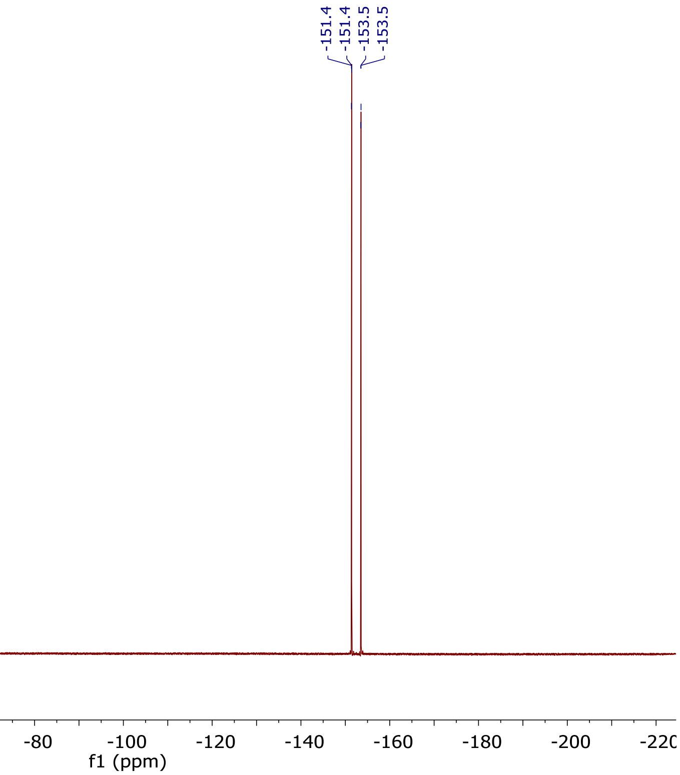

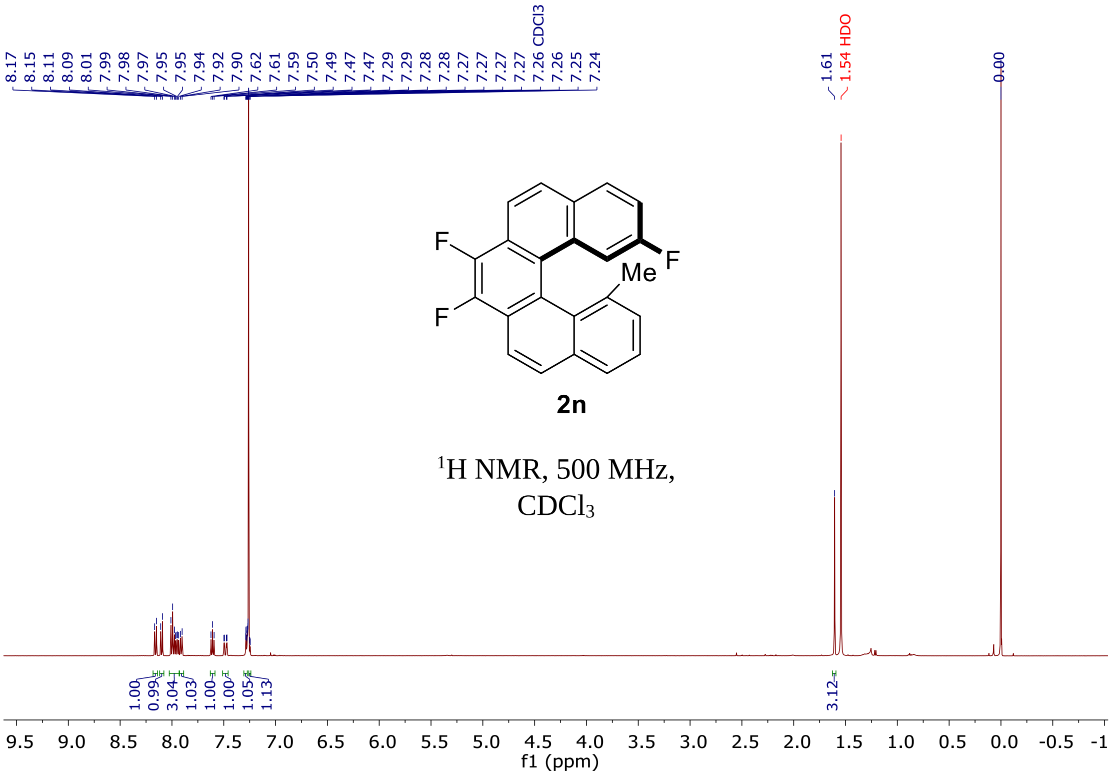
8.17 8.15 8.11 8.09 8.01 7.99 7.98 7.97 7.95 7.95 7.94 7.92 7.90 7.62 7.61 7.59 7.50 7.49 7.47 7.47 7.29 7.29 7.28 7.28 7.27 7.27 7.27 7.27 7.26 CDCl3 7.26 7.25 7.24
1.61 1.54 HDO
0.00
F
F
Me
F
2n
1H NMR, 500 MHz, CDCl3
1.00 0.99 3.04 1.03 1.00 1.00 1.05 1.13
3.12
f1 (ppm)

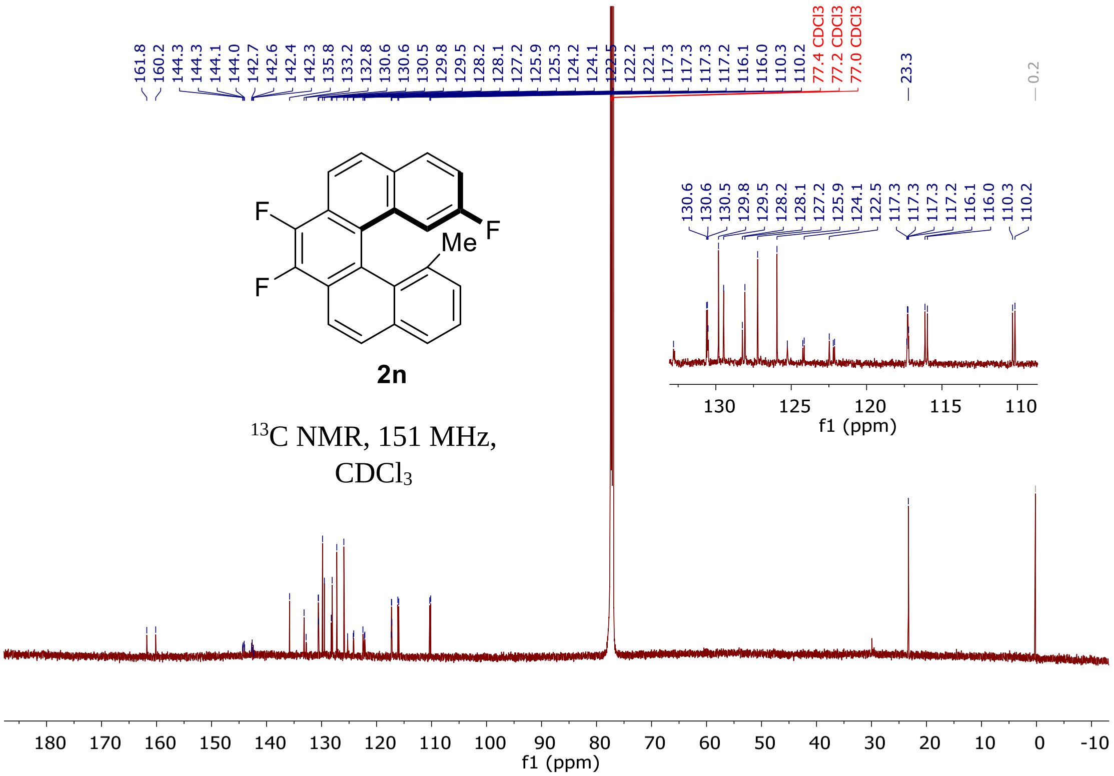
161.8 160.2 144.3 144.3 144.1 144.0 142.7 142.6 142.4 142.3 135.8 133.2 132.8 130.6 130.6 130.5 129.8 129.5 128.2 128.1 127.2 125.9 125.3 124.2 124.1 122.5 122.2 122.1 117.3 117.3 117.3 117.2 116.1 116.0 110.3 110.2 77.4 CDCl3 77.2 CDCl3 77.0 CDCl3
23.3
0.2
F
F
Me
F
2n
13C NMR, 151 MHz, CDCl3
130.6 130.6 130.5 129.8 129.5 128.2 128.1 127.2 125.9 124.1 122.5 117.3 117.3 117.3 117.2 116.1 116.0 110.3 110.2
f1 (ppm)

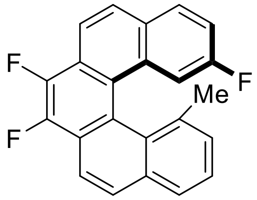

2n

^{19}F NMR, 376 MHz,
$CDCl_3$

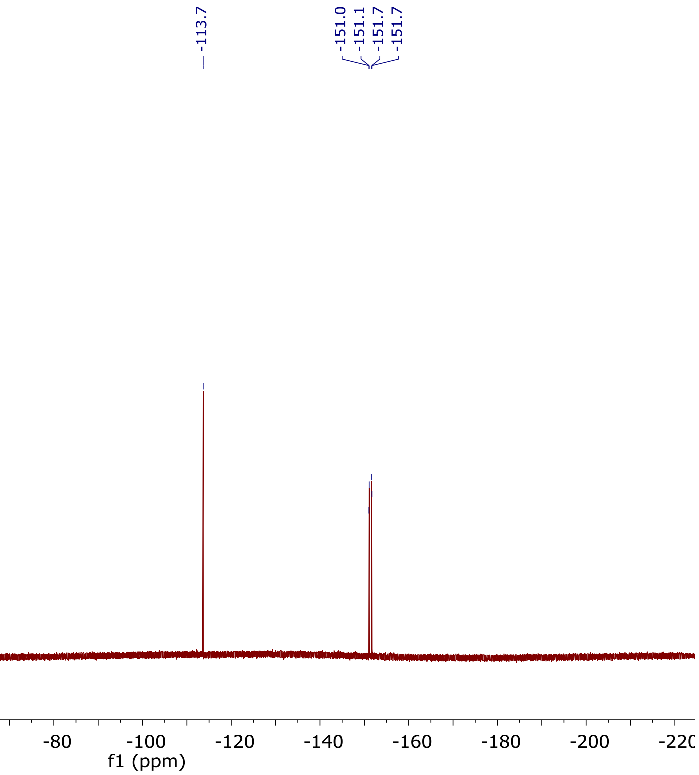

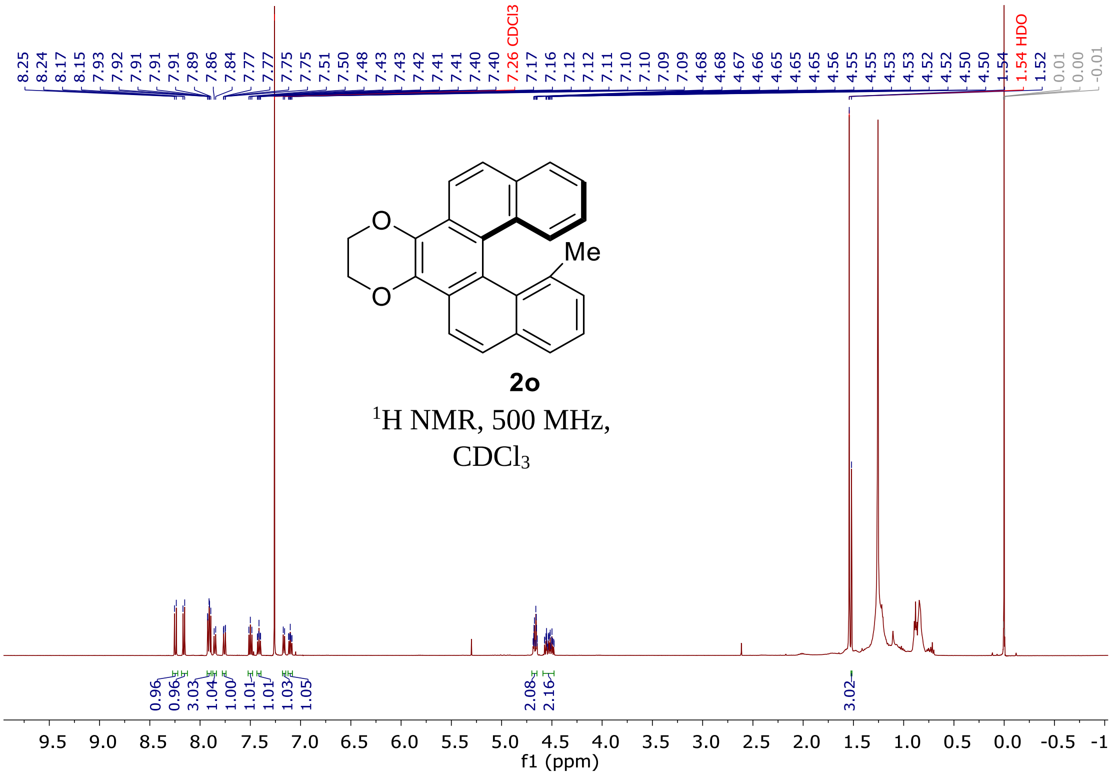
O
O
Me
2o
1H NMR, 500 MHz, CDCl3
7.26 CDCl3
1.54 HDO
0.96
0.96
3.03
1.04
1.00
1.01
1.01
1.03
1.05
2.08
2.16
3.02
f1 (ppm)

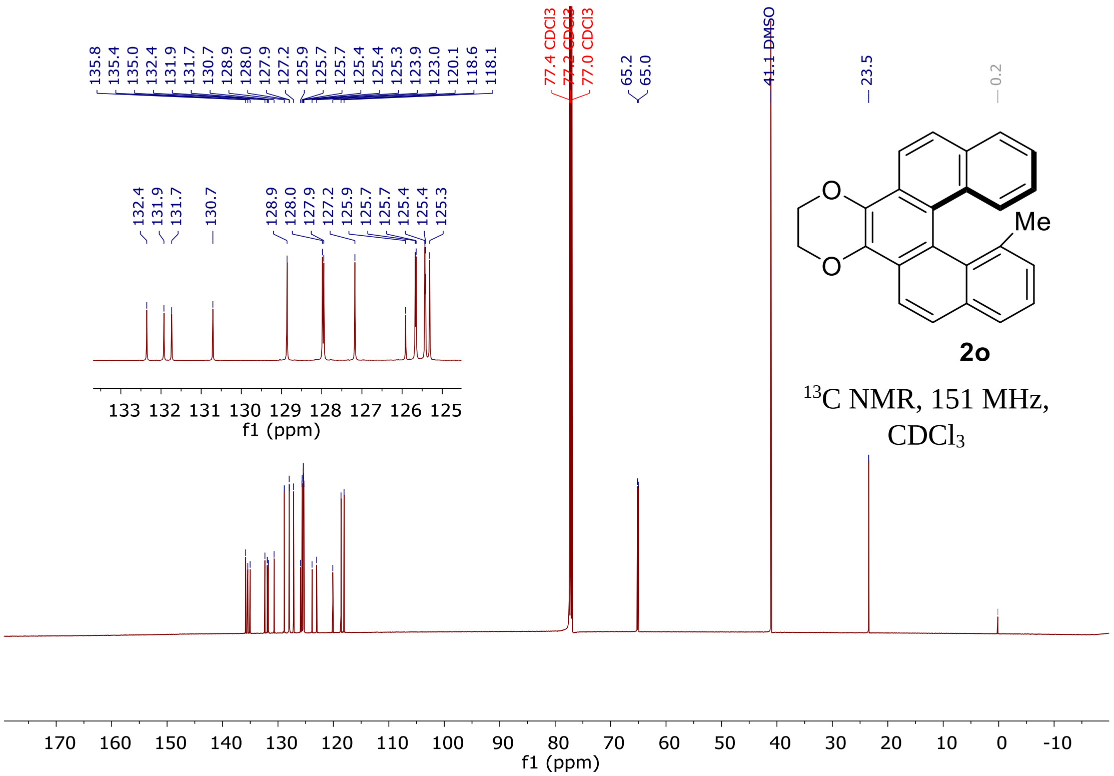
O
O
Me
2o
13C NMR, 151 MHz, CDCl3
77.4 CDCl3
77.2 CDCl3
77.0 CDCl3
65.2
65.0
41.1 DMSO
23.5
0.2
f1 (ppm)

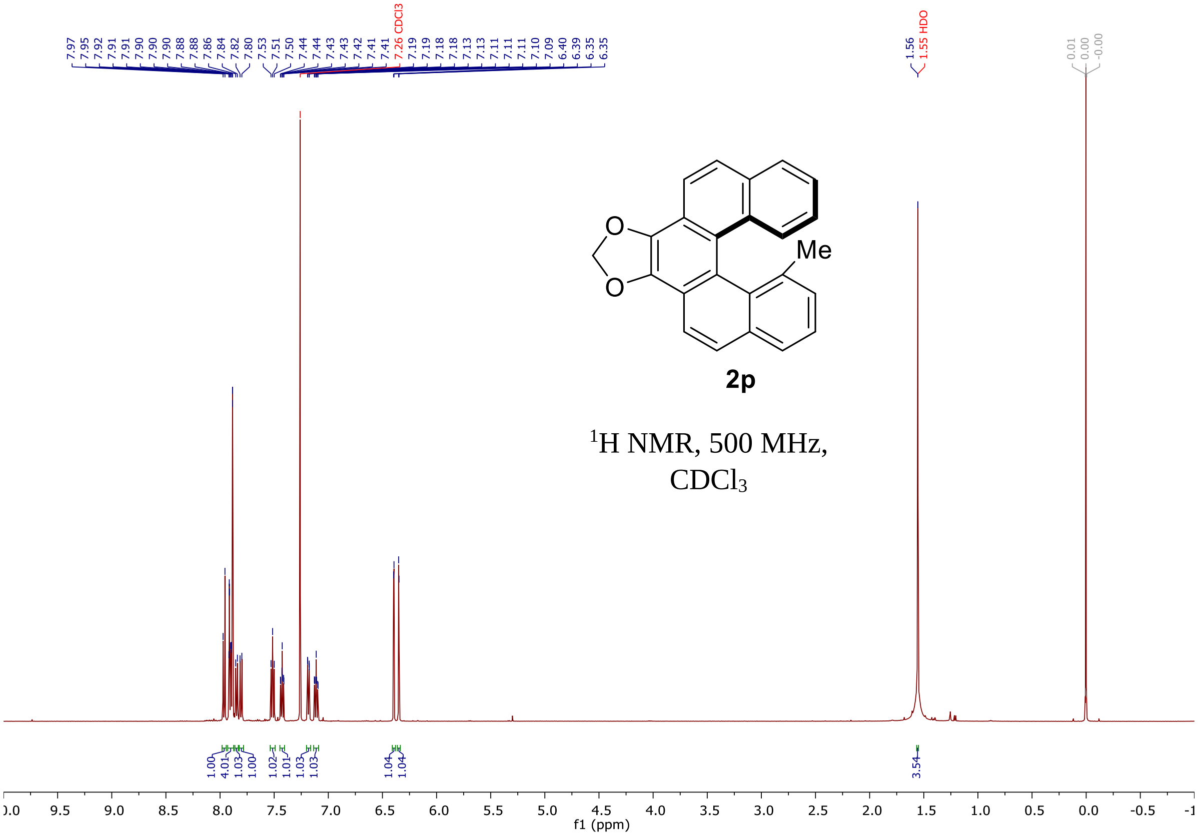
7.97 7.95 7.92 7.91 7.91 7.90 7.90 7.90 7.88 7.88 7.86 7.84 7.82 7.80 7.53 7.51 7.50 7.44 7.44 7.43 7.43 7.42 7.41 7.41 7.26 CDCl3 7.19 7.19 7.18 7.18 7.13 7.13 7.11 7.11 7.11 7.10 7.09 6.40 6.39 6.35 6.35
1.56 1.55 HDO
0.01 0.00 -0.00
Me
2p
^{1}H NMR, 500 MHz, $CDCl_3$
1.00 4.01 1.03 1.00 1.02 1.01 1.03 1.03 1.04 1.04 3.54
f1 (ppm)

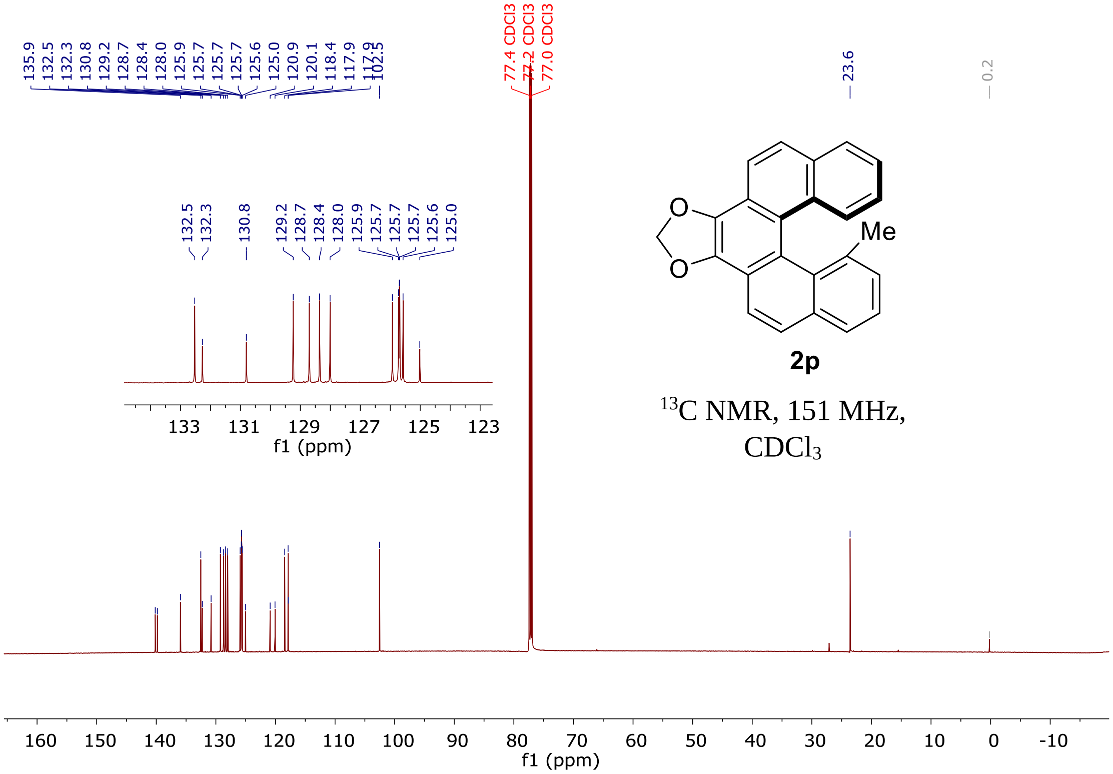
135.9 132.5 132.3 130.8 129.2 128.7 128.4 128.0 125.9 125.7 125.7 125.6 125.0 120.9 120.1 118.4 117.9 102.5
77.4 CDCl3 77.2 CDCl3 77.0 CDCl3
23.6
0.2
132.5 132.3 130.8 129.2 128.7 128.4 128.0 125.9 125.7 125.7 125.6 125.0
f1 (ppm)
Me
2p
^{13}C NMR, 151 MHz, $CDCl_3$
f1 (ppm)

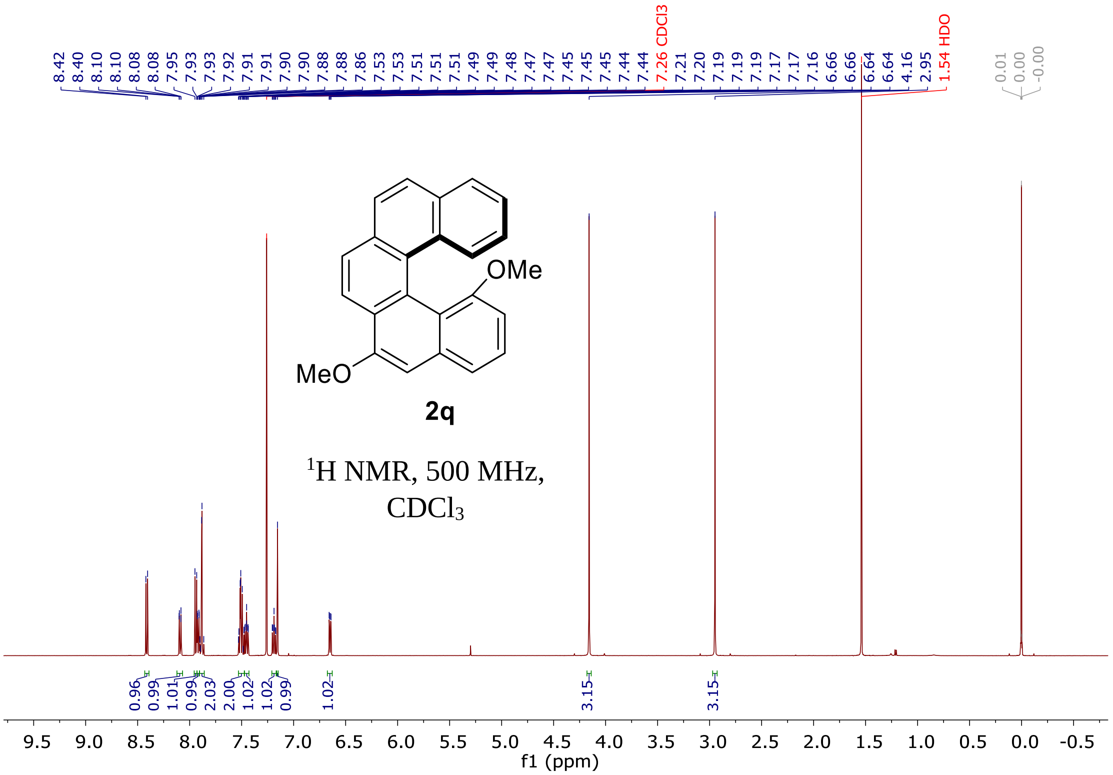
8.42
8.40
8.10
8.10
8.08
8.08
7.95
7.93
7.93
7.92
7.91
7.90
7.88
7.88
7.86
7.53
7.53
7.51
7.51
7.49
7.48
7.47
7.45
7.45
7.45
7.44
7.44
7.26 CDCl3
7.21
7.20
7.19
7.19
7.19
7.17
7.17
7.16
6.66
6.66
6.64
6.64
4.16
2.95
1.54 HDO
0.01
0.00
-0.00
OMe
MeO
2q
¹H NMR, 500 MHz, CDCl3
0.96
0.99
1.01
0.99
2.03
2.00
1.02
1.02
0.99
1.02
3.15
3.15
f1 (ppm)

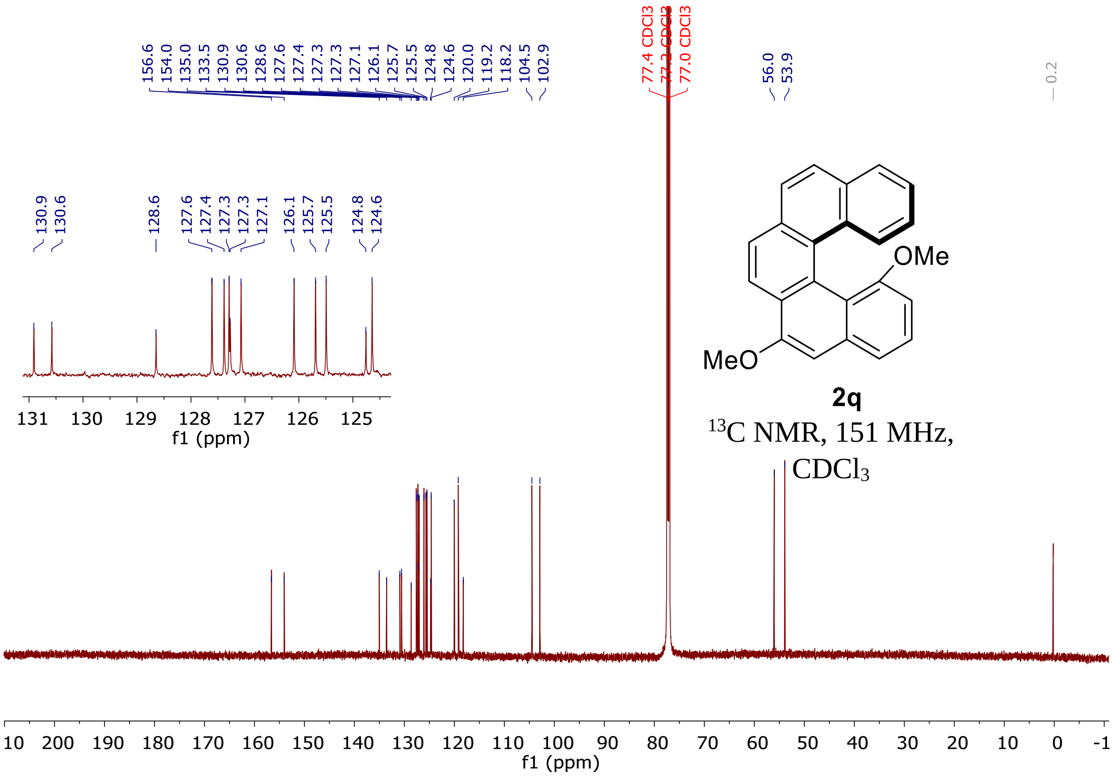
156.6
154.0
135.0
133.5
130.9
130.6
128.6
127.6
127.4
127.3
127.3
127.1
126.1
125.7
125.5
124.8
124.6
120.0
119.2
118.2
104.5
102.9
77.4 CDCl3
77.2 CDCl3
77.0 CDCl3
56.0
53.9
0.2
f1 (ppm)
OMe
MeO
2q
¹³C NMR, 151 MHz, CDCl3

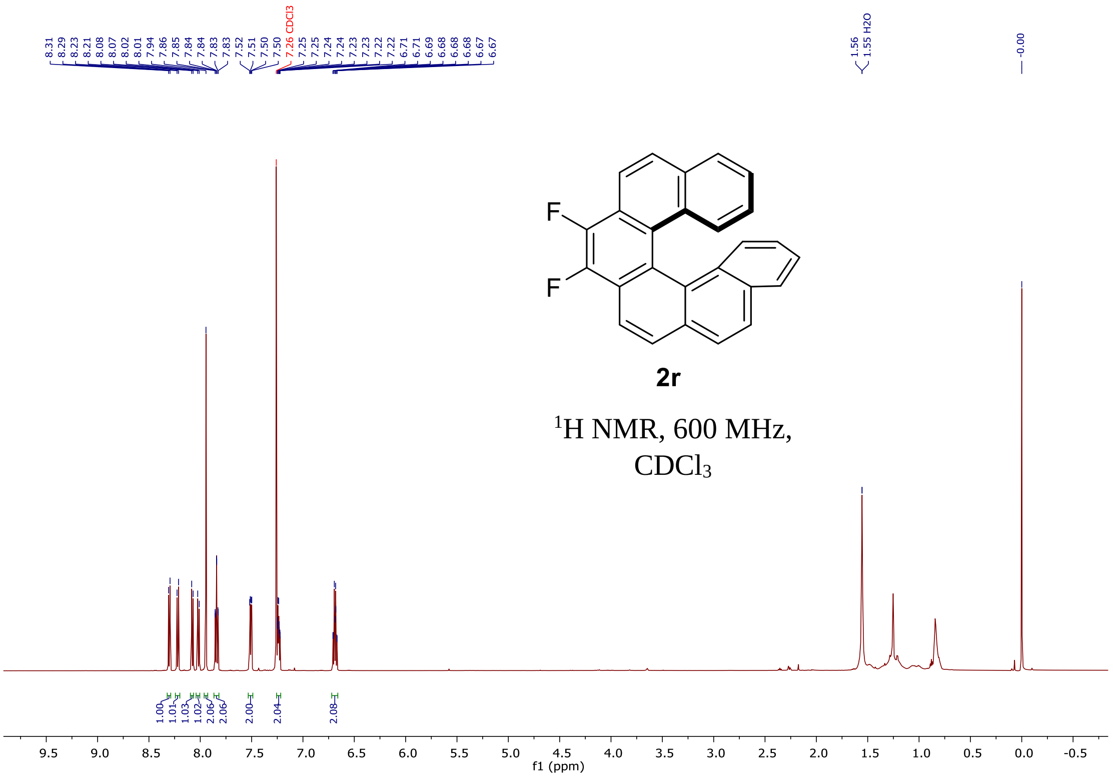
F
F
2r
^{1}H NMR, 600 MHz, CDCl3
f1 (ppm)

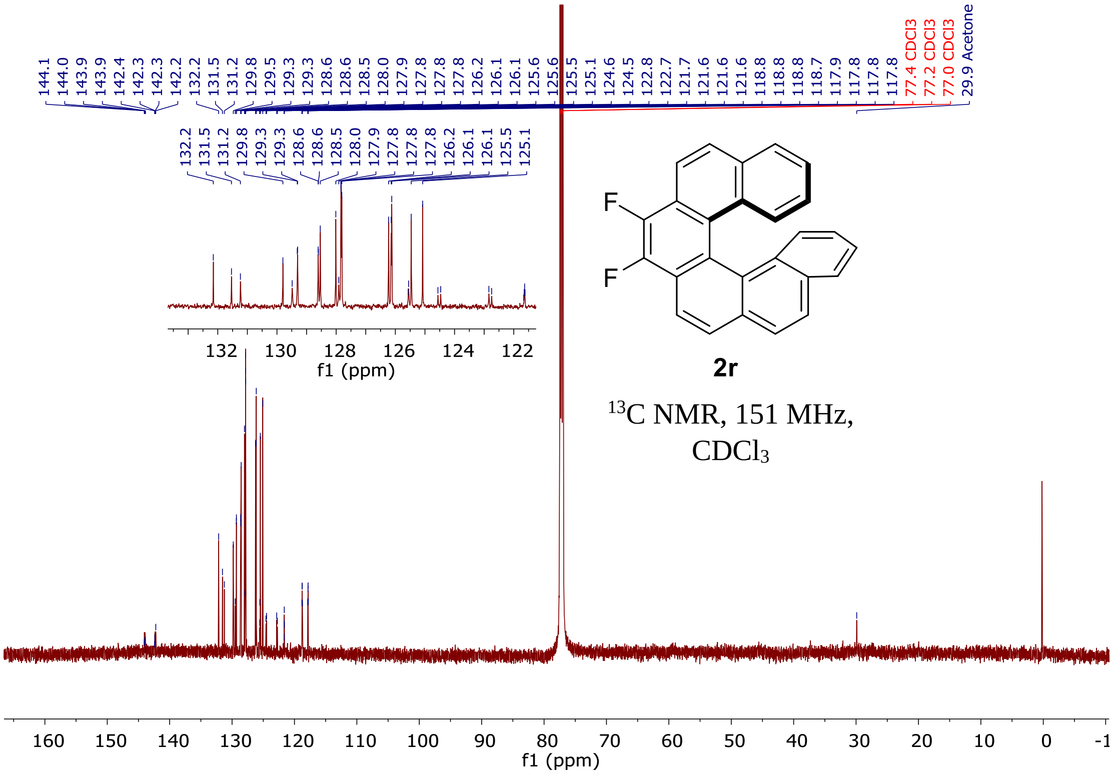
F
F
2r
^{13}C NMR, 151 MHz, CDCl3
f1 (ppm)

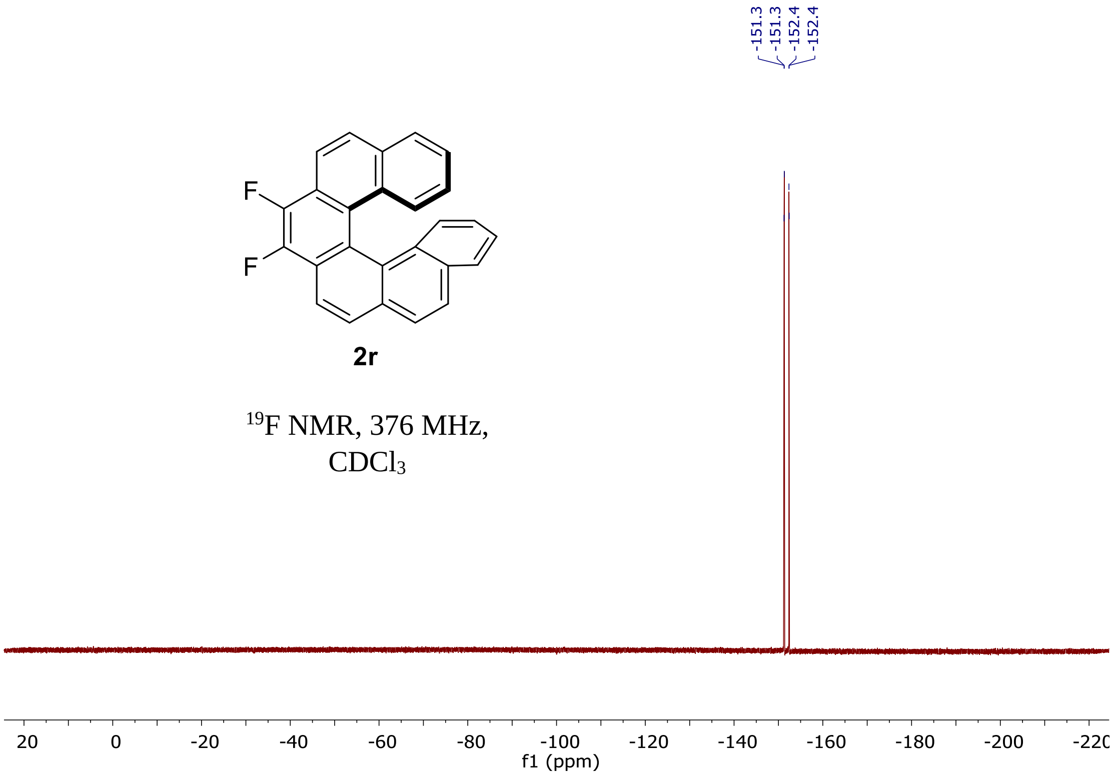

2r

^{19}F NMR, 376 MHz, $CDCl_3$

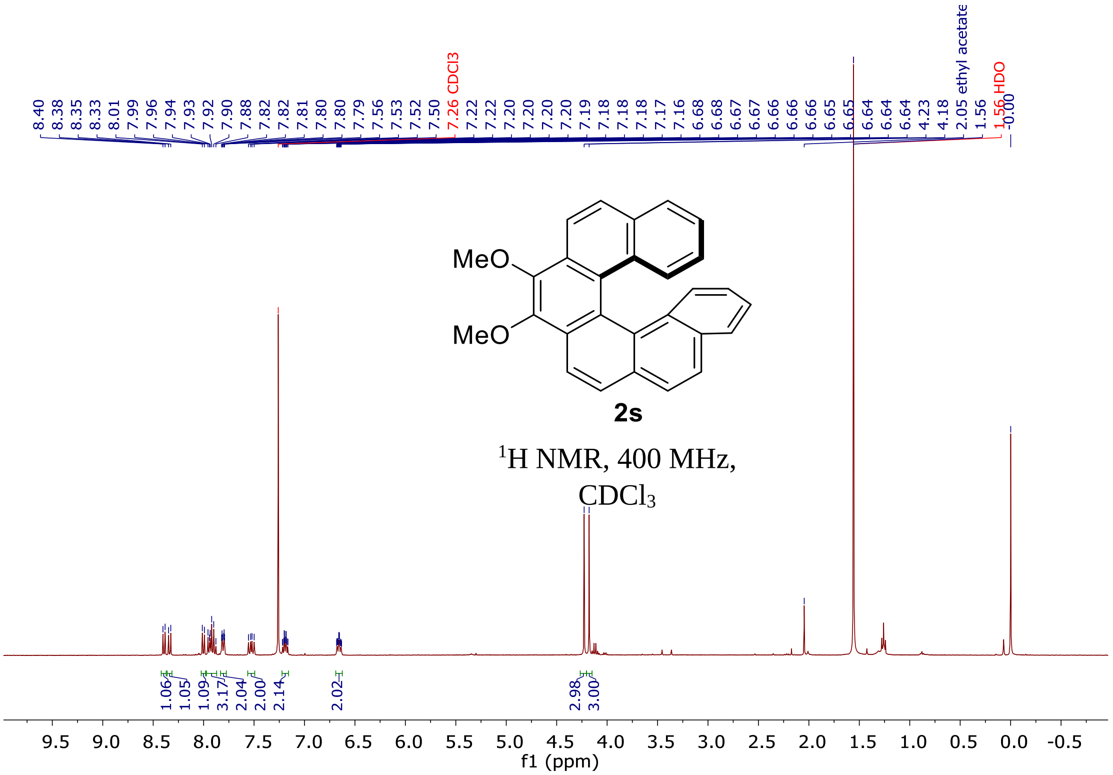
8.40 8.38 8.35 8.33 8.01 7.99 7.96 7.94 7.93 7.92 7.90 7.88 7.82 7.82 7.81 7.80 7.80 7.79 7.56 7.53 7.52 7.50 7.26 CDCl3 7.22 7.22 7.20 7.20 7.20 7.20 7.19 7.18 7.18 7.17 7.16 6.68 6.68 6.67 6.67 6.66 6.66 6.66 6.65 6.65 6.64 6.64 6.64 4.23 4.18 2.05 ethyl acetate 1.56 1.56 HDO -0.00
MeO
MeO
2s
1H NMR, 400 MHz, CDCl3
1.06 1.05 1.09 3.17 2.04 2.00 2.14 2.02 2.98 3.00
f1 (ppm)

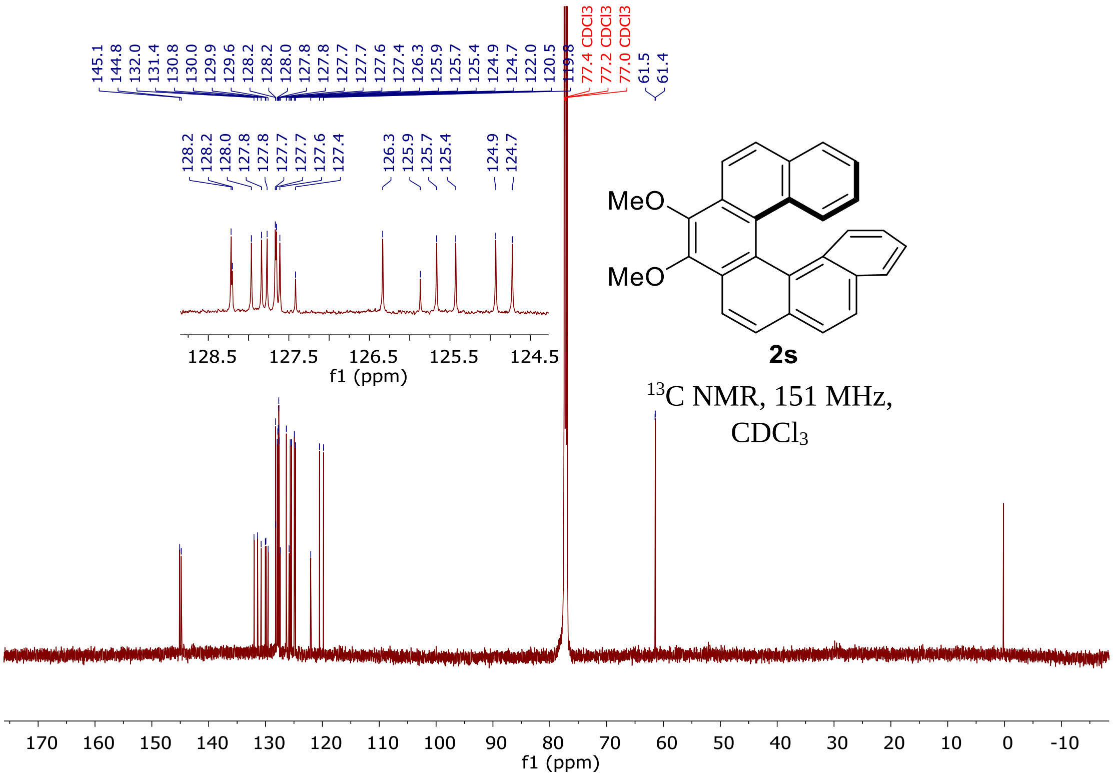
145.1 144.8 132.0 131.4 130.8 130.0 129.9 129.6 128.2 128.2 128.0 127.8 127.8 127.7 127.7 127.6 127.4 126.3 125.9 125.7 125.4 124.9 124.7 122.0 120.5 119.8 77.4 CDCl3 77.2 CDCl3 77.0 CDCl3 61.5 61.4
128.2 128.2 128.0 127.8 127.8 127.7 127.7 127.6 127.4 126.3 125.9 125.7 125.4 124.9 124.7
f1 (ppm)
MeO
MeO
2s
13C NMR, 151 MHz, CDCl3
f1 (ppm)

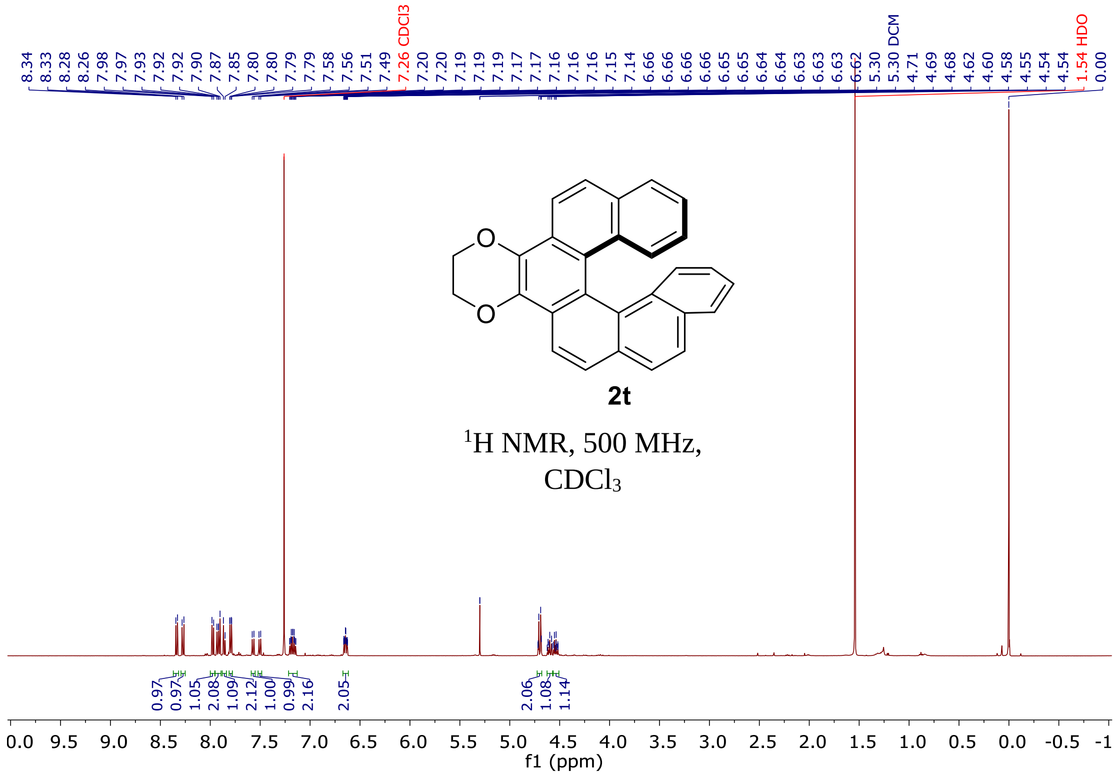
8.34 8.33 8.28 8.26 7.98 7.97 7.93 7.92 7.92 7.90 7.87 7.85 7.80 7.80 7.79 7.79 7.58 7.56 7.51 7.49 7.26 CDCl3 7.20 7.20 7.19 7.19 7.19 7.17 7.17 7.16 7.16 7.16 7.15 7.14 6.66 6.66 6.66 6.66 6.65 6.65 6.64 6.64 6.63 6.63 6.63 6.62 5.30 5.30 DCM 4.71 4.69 4.68 4.62 4.60 4.58 4.55 4.54 4.54 1.54 HDO 0.00
O
O
2t
^{1}H NMR, 500 MHz, CDCl$_3$
0.97 0.97 1.05 2.08 1.09 2.12 1.00 0.99 2.16 2.05 2.06 1.08 1.14
f1 (ppm)

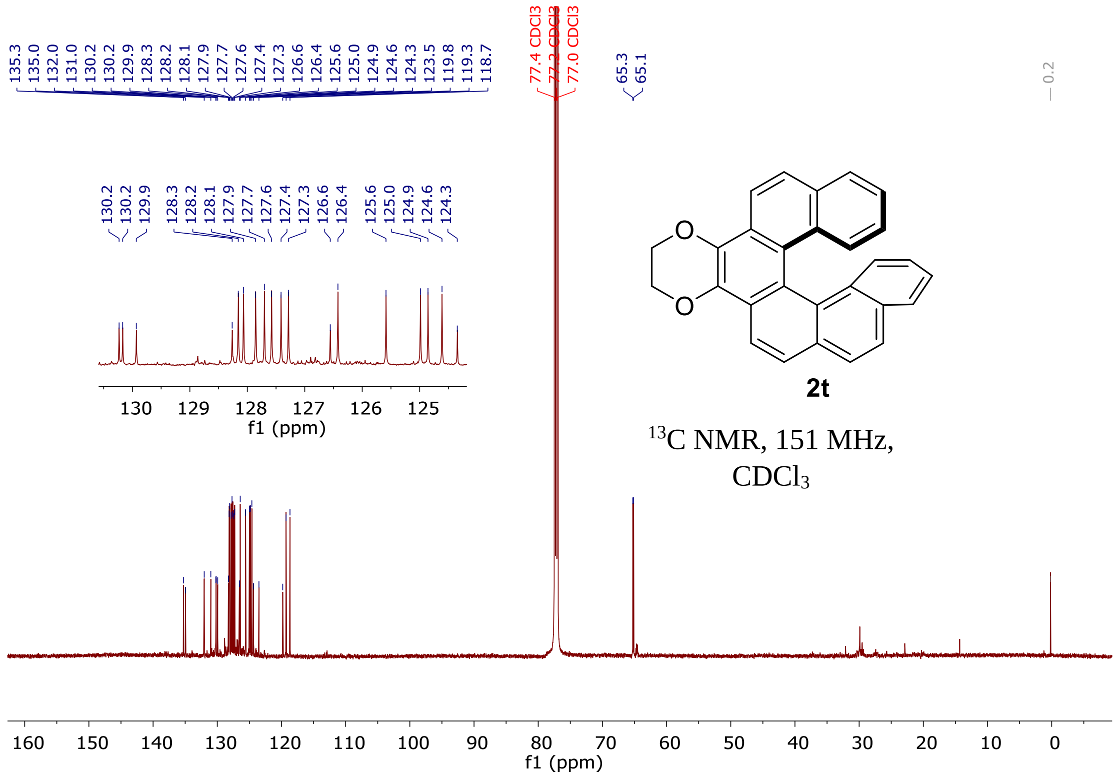
135.3 135.0 132.0 131.0 130.2 130.2 129.9 128.3 128.2 128.1 127.9 127.7 127.6 127.4 127.3 126.6 126.4 125.6 125.0 124.9 124.6 124.3 123.5 119.8 119.3 118.7
77.4 CDCl3 77.2 CDCl3 77.0 CDCl3
65.3 65.1
0.2
f1 (ppm)
O
O
2t
^{13}C NMR, 151 MHz, CDCl$_3$

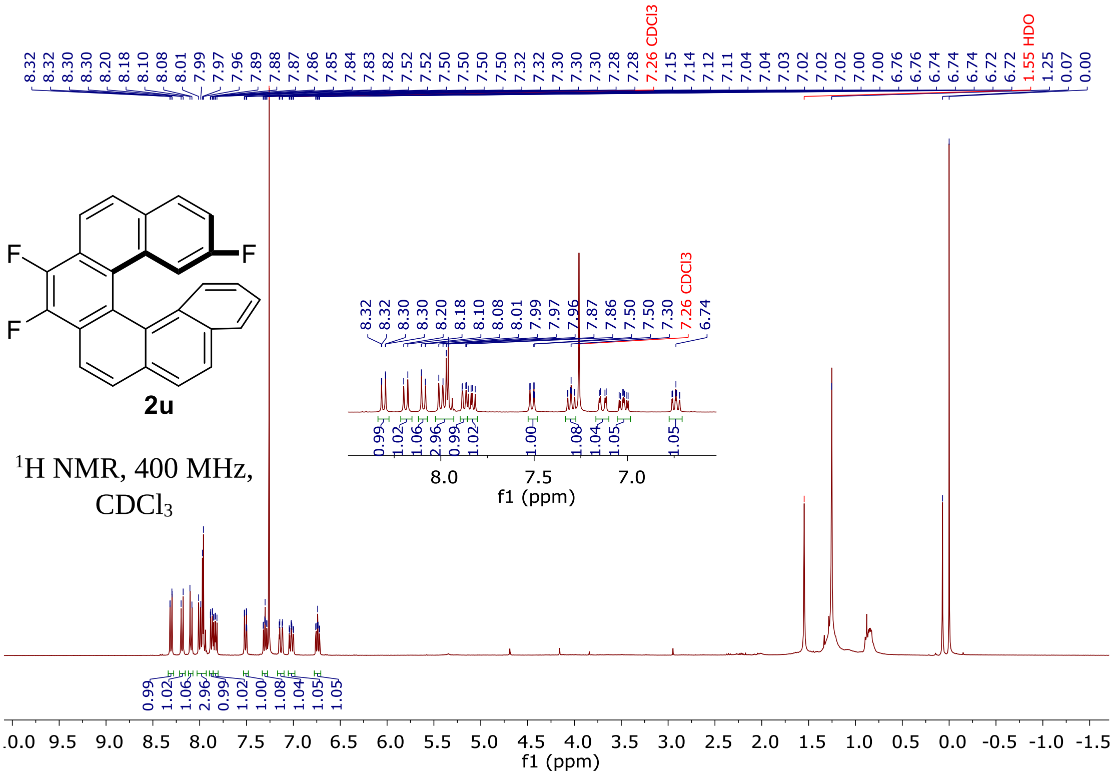
8.32 8.32 8.30 8.30 8.20 8.18 8.10 8.08 8.01 7.99 7.97 7.96 7.89 7.88 7.87 7.86 7.85 7.84 7.83 7.82 7.52 7.52 7.50 7.50 7.50 7.50 7.32 7.32 7.30 7.30 7.30 7.28 7.26 CDCl3 7.15 7.14 7.12 7.11 7.04 7.03 7.02 7.02 7.02 7.00 7.00 6.76 6.76 6.74 6.74 6.74 6.72 6.72 1.55 HDO 1.25 0.07 0.00
F
F
F
2u
^{1}H NMR, 400 MHz, CDCl$_3$
f1 (ppm)

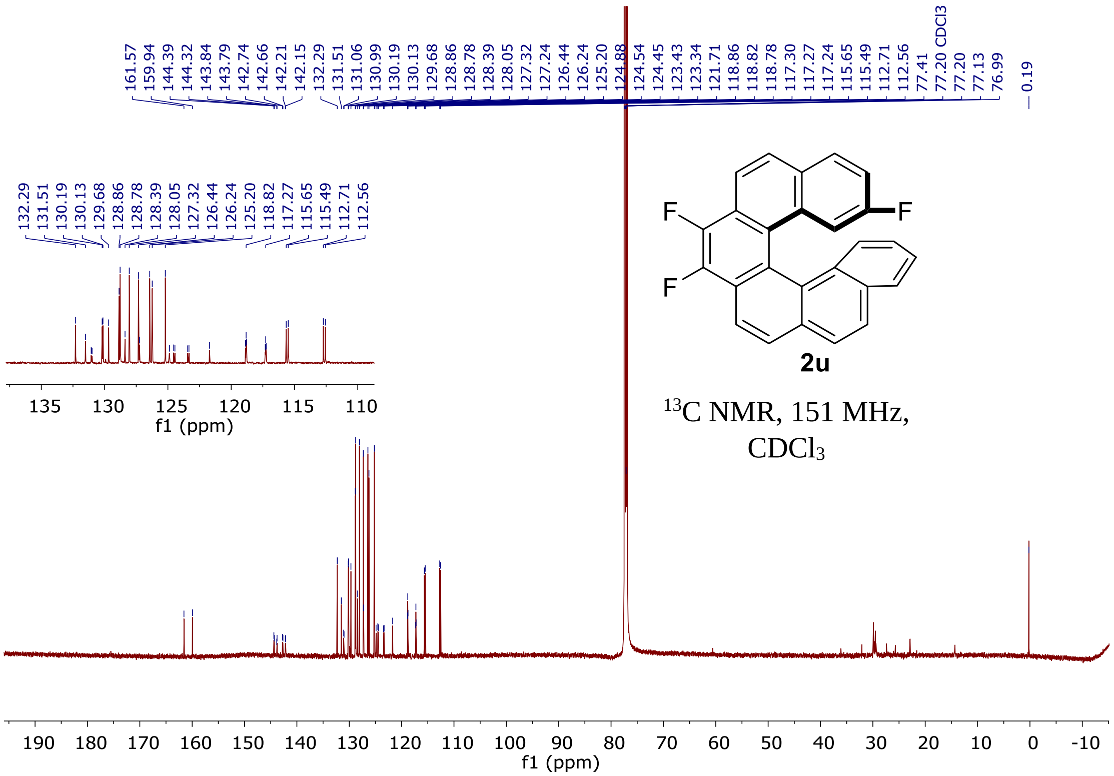
161.57 159.94 144.39 144.32 143.84 143.79 142.74 142.66 142.21 142.15 132.29 131.51 131.06 130.99 130.19 130.13 129.68 128.86 128.78 128.39 128.05 127.32 127.24 126.44 126.24 125.20 124.88 124.54 124.45 123.43 123.34 121.71 118.86 118.82 118.78 117.30 117.27 117.24 115.65 115.49 112.71 112.56 77.41 77.20 CDCl3 77.20 77.13 76.99 0.19
F
F
F
2u
^{13}C NMR, 151 MHz, CDCl$_3$
f1 (ppm)

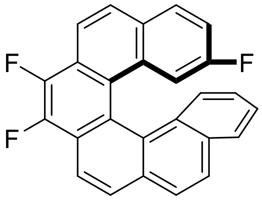

2u

^{19}F NMR, 376 MHz, $CDCl_3$

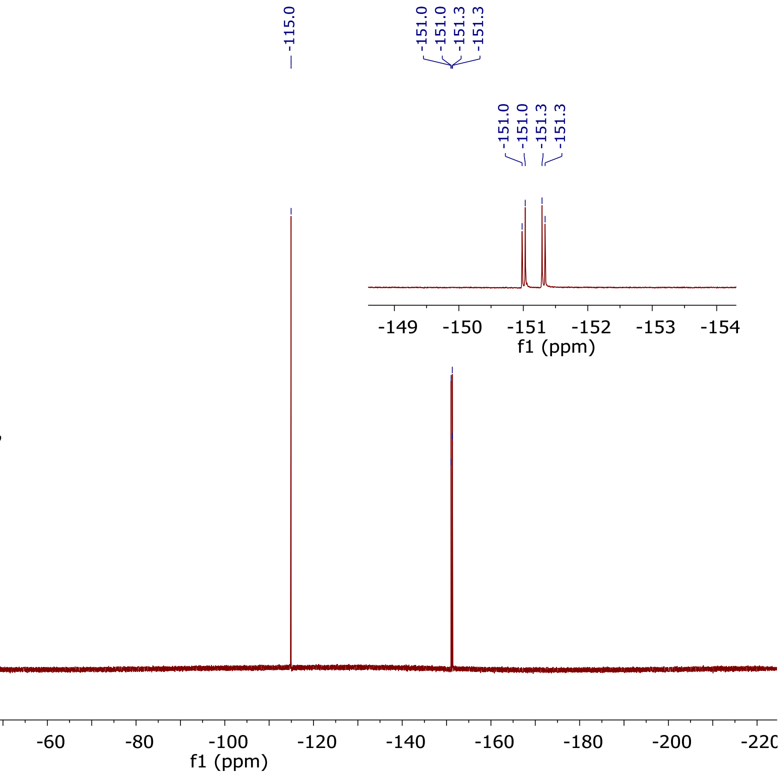

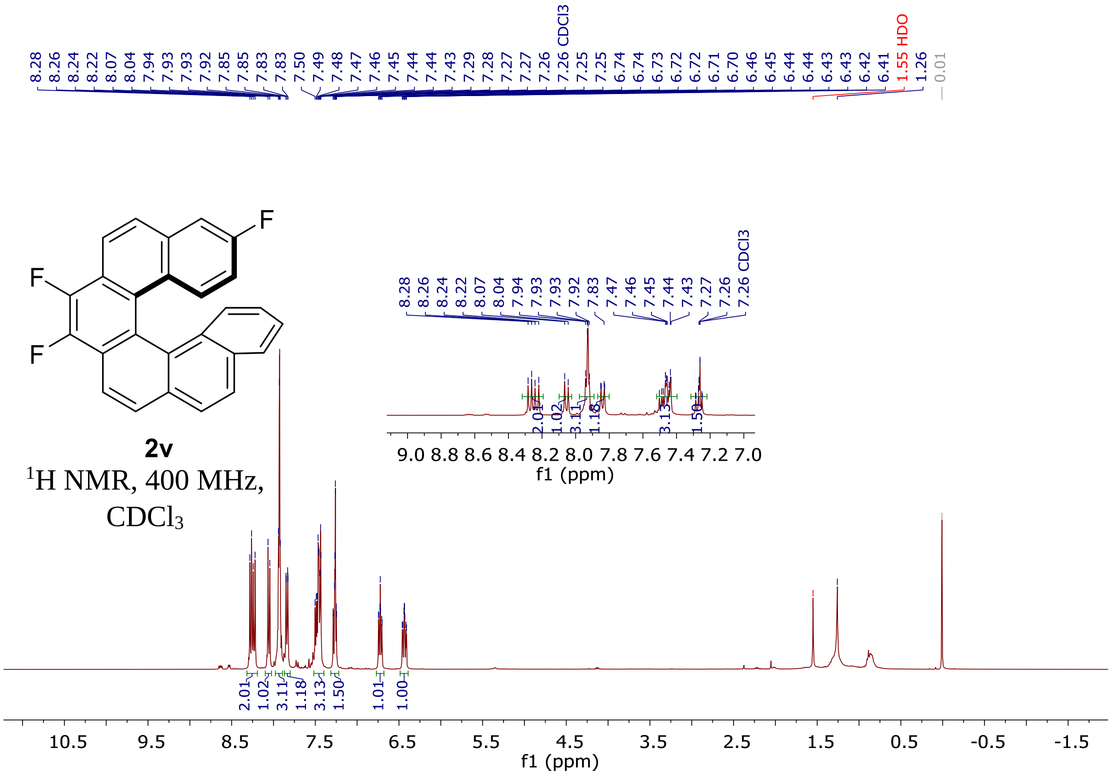
8.28 8.26 8.24 8.22 8.07 8.04 7.94 7.93 7.93 7.92 7.85 7.85 7.83 7.83 7.50 7.49 7.48 7.47 7.46 7.45 7.44 7.44 7.43 7.29 7.28 7.27 7.27 7.26 7.26 CDCl3 7.25 7.25 6.74 6.74 6.73 6.72 6.72 6.71 6.70 6.46 6.45 6.44 6.44 6.43 6.43 6.42 6.41 1.55 HDO 1.26 0.01
F
F
F
2v
^{1}H NMR, 400 MHz, CDCl$_3$
8.28 8.26 8.24 8.22 8.07 8.04 7.94 7.93 7.93 7.92 7.83 7.47 7.46 7.45 7.44 7.43 7.27 7.26 7.26 CDCl3
2.01 1.02 3.11 1.18 3.13 1.50
9.0 8.8 8.6 8.4 8.2 8.0 7.8 7.6 7.4 7.2 7.0
f1 (ppm)
2.01 1.02 3.11 1.18 3.13 1.50 1.01 1.00
10.5 9.5 8.5 7.5 6.5 5.5 4.5 3.5 2.5 1.5 0.5 -0.5 -1.5
f1 (ppm)

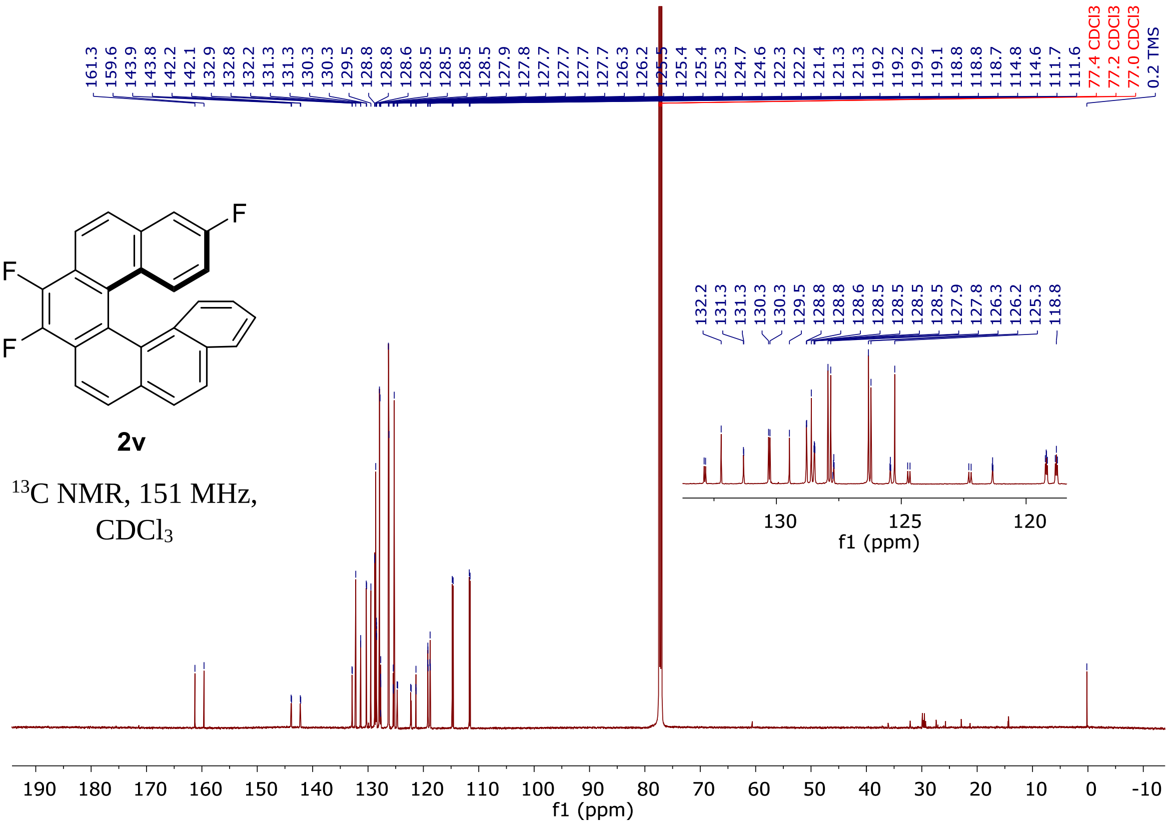
161.3 159.6 143.9 143.8 142.2 142.1 132.9 132.8 132.2 131.3 131.3 130.3 130.3 129.5 128.8 128.8 128.6 128.5 128.5 128.5 128.5 127.9 127.8 127.7 127.7 127.7 127.7 126.3 126.2 125.5 125.4 125.4 125.3 124.7 124.6 122.3 122.2 121.4 121.3 121.3 119.2 119.2 119.2 119.1 118.8 118.8 118.7 114.8 114.6 111.7 111.6 77.4 CDCl3 77.2 CDCl3 77.0 CDCl3 0.2 TMS
F
F
F
2v
^{13}C NMR, 151 MHz, CDCl$_3$
132.2 131.3 131.3 130.3 130.3 129.5 128.8 128.8 128.6 128.5 128.5 128.5 128.5 127.9 127.8 126.3 126.2 125.3 118.8
130 125 120
f1 (ppm)
190 180 170 160 150 140 130 120 110 100 90 80 70 60 50 40 30 20 10 0 -10
f1 (ppm)

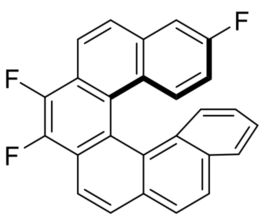

2v

^{19}F NMR, 376 MHz, $CDCl_3$

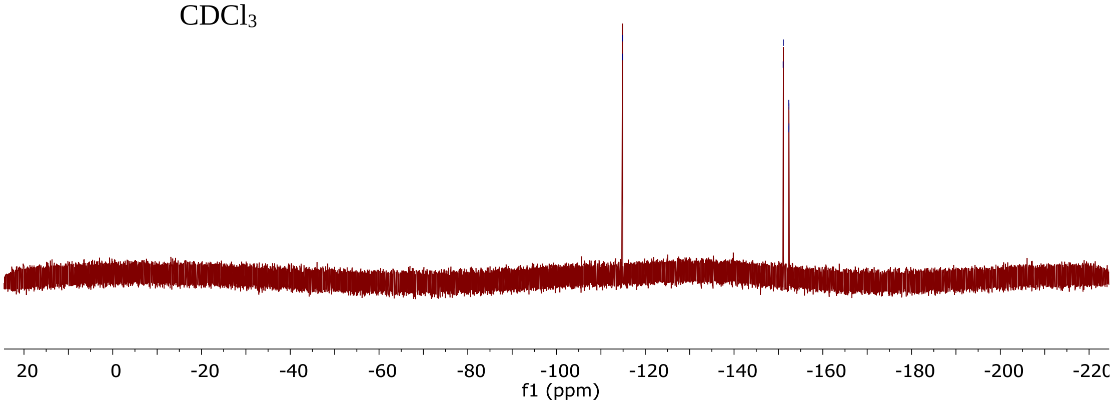

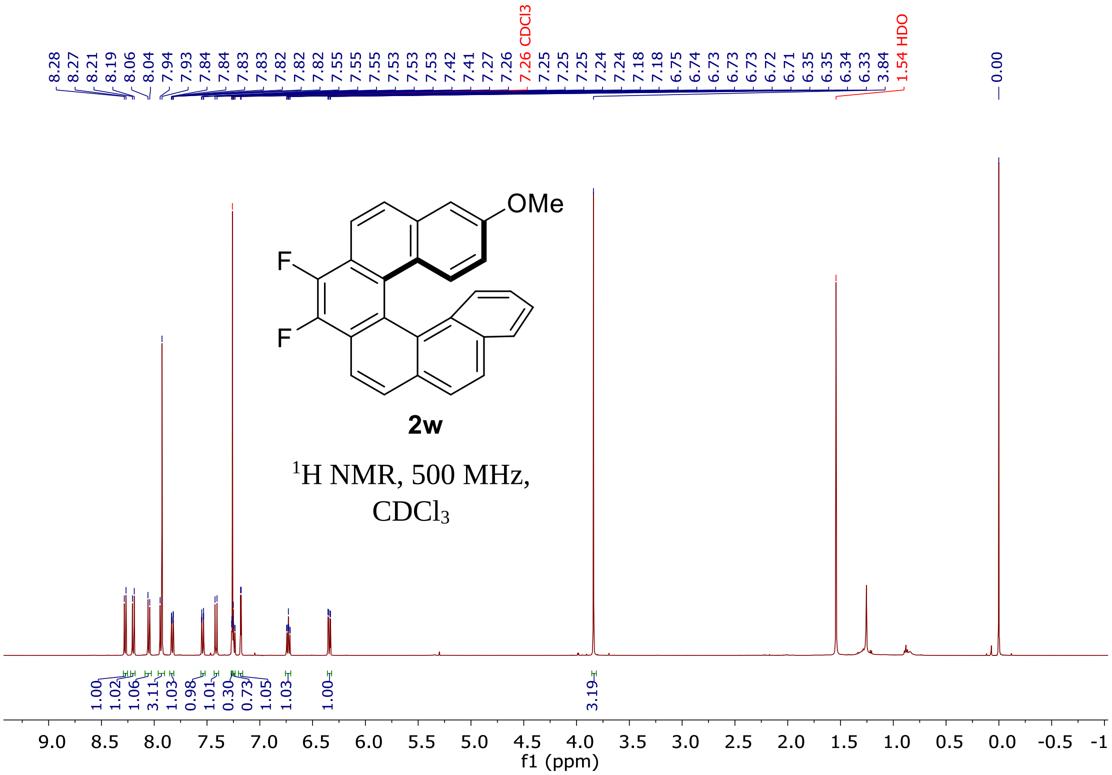
8.28 8.27 8.21 8.19 8.06 8.04 7.94 7.93 7.84 7.84 7.83 7.83 7.82 7.82 7.82 7.55 7.55 7.55 7.53 7.53 7.53 7.42 7.41 7.27 7.26 7.26 CDCl3 7.25 7.25 7.25 7.24 7.24 7.18 7.18 6.75 6.74 6.73 6.73 6.72 6.71 6.35 6.35 6.34 6.33 3.84 1.54 HDO 0.00
OMe
F
F
2w
^{1}H NMR, 500 MHz,
$CDCl_3$
1.00 1.02 1.06 3.11 1.03 0.98 1.01 0.30 0.73 1.05 1.03 1.00 3.19
9.0 8.5 8.0 7.5 7.0 6.5 6.0 5.5 5.0 4.5 4.0 3.5 3.0 2.5 2.0 1.5 1.0 0.5 0.0 -0.5 -1
f1 (ppm)

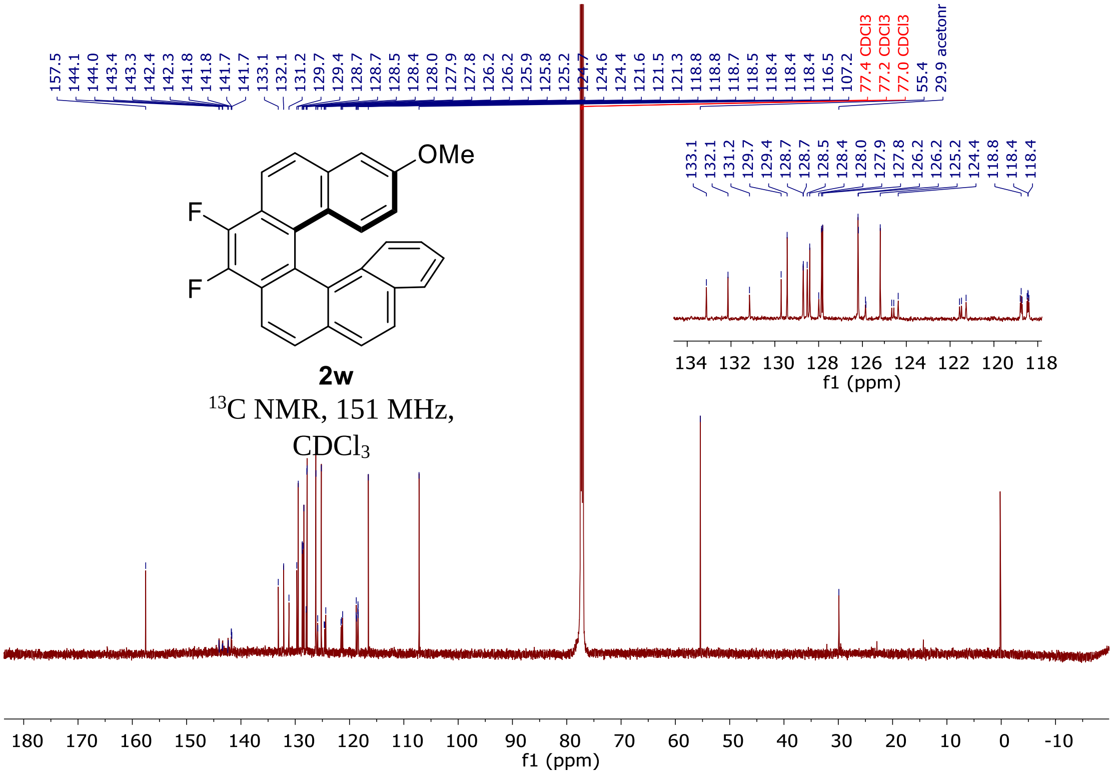
157.5 144.1 144.0 143.4 143.3 142.4 142.3 141.8 141.8 141.7 141.7 133.1 132.1 131.2 129.7 129.4 128.7 128.7 128.5 128.4 128.0 127.9 127.8 126.2 126.2 125.9 125.8 125.2 124.7 124.6 124.4 121.6 121.5 121.3 118.8 118.8 118.7 118.5 118.4 118.4 118.4 116.5 107.2 77.4 CDCl3 77.2 CDCl3 77.0 CDCl3 55.4 29.9 acetone
OMe
F
F
2w
^{13}C NMR, 151 MHz,
$CDCl_3$
133.1 132.1 131.2 129.7 129.4 128.7 128.7 128.5 128.4 128.0 127.9 127.8 126.2 126.2 125.2 124.4 118.8 118.4 118.4
134 132 130 128 126 124 122 120 118
f1 (ppm)
180 170 160 150 140 130 120 110 100 90 80 70 60 50 40 30 20 10 0 -10
f1 (ppm)

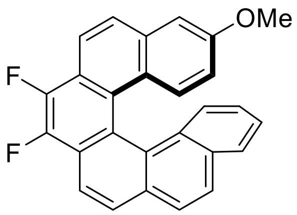

2w

^{19}F NMR, 376 MHz, $CDCl_3$

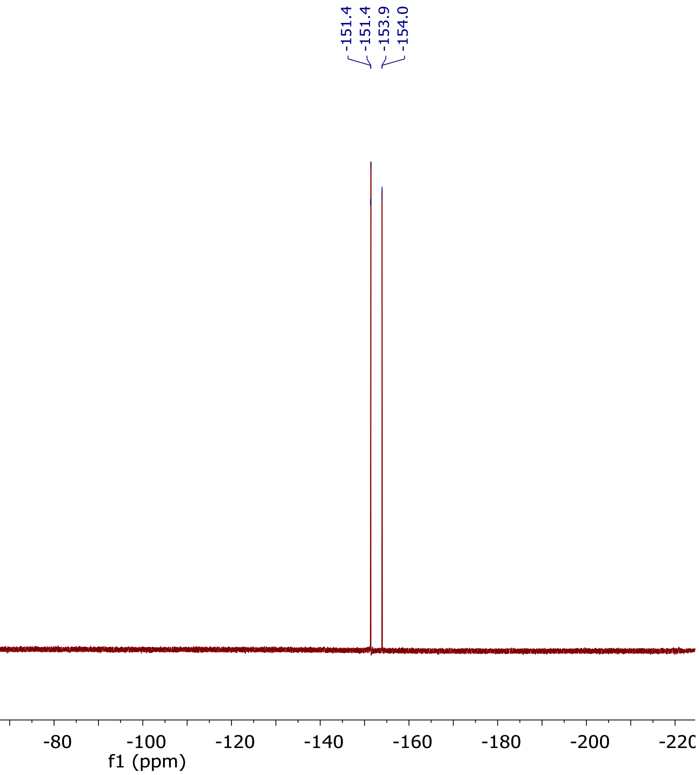

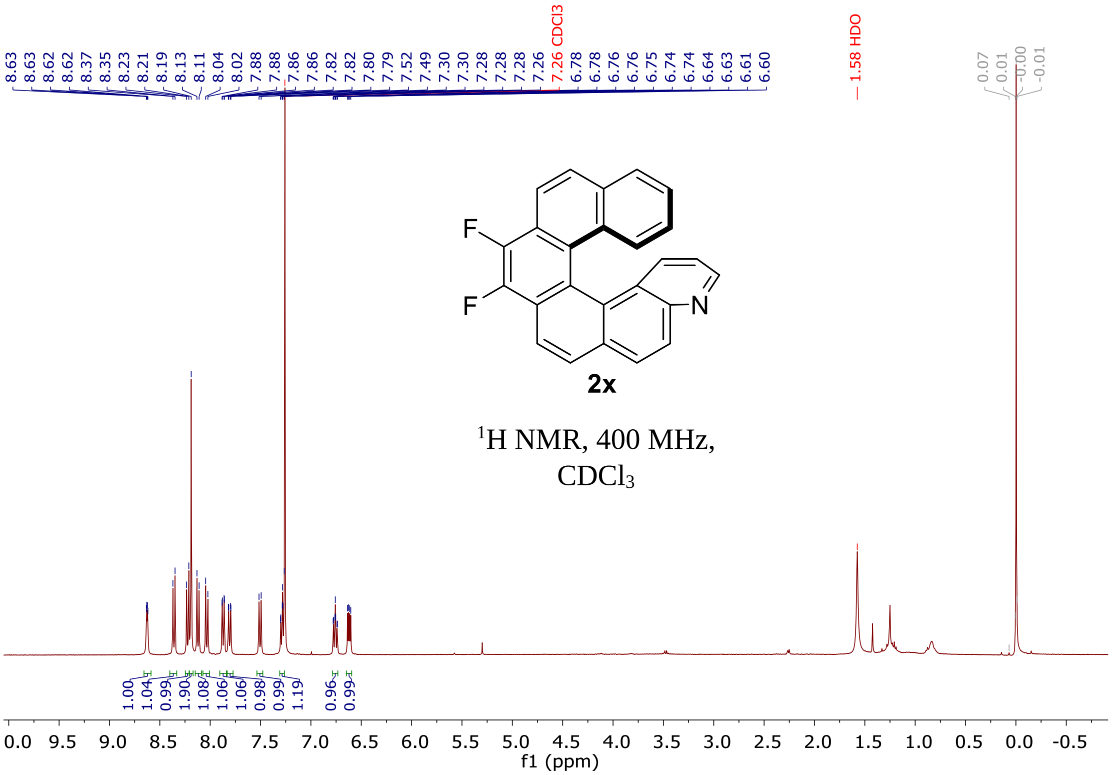
8.63 8.63 8.62 8.62 8.37 8.35 8.23 8.21 8.19 8.13 8.11 8.04 8.02 7.88 7.88 7.86 7.86 7.82 7.82 7.80 7.79 7.52 7.49 7.30 7.28 7.28 7.28 7.26
7.26 CDCl3
6.78 6.78 6.76 6.76 6.75 6.74 6.74 6.64 6.63 6.61 6.60
1.58 HDO
0.07 0.01 0.00 -0.01
F
F
N
2x
1H NMR, 400 MHz, CDCl3
1.00 1.04 0.99 1.90 1.08 1.06 1.06 0.98 0.99 1.19 0.96 0.99
f1 (ppm)

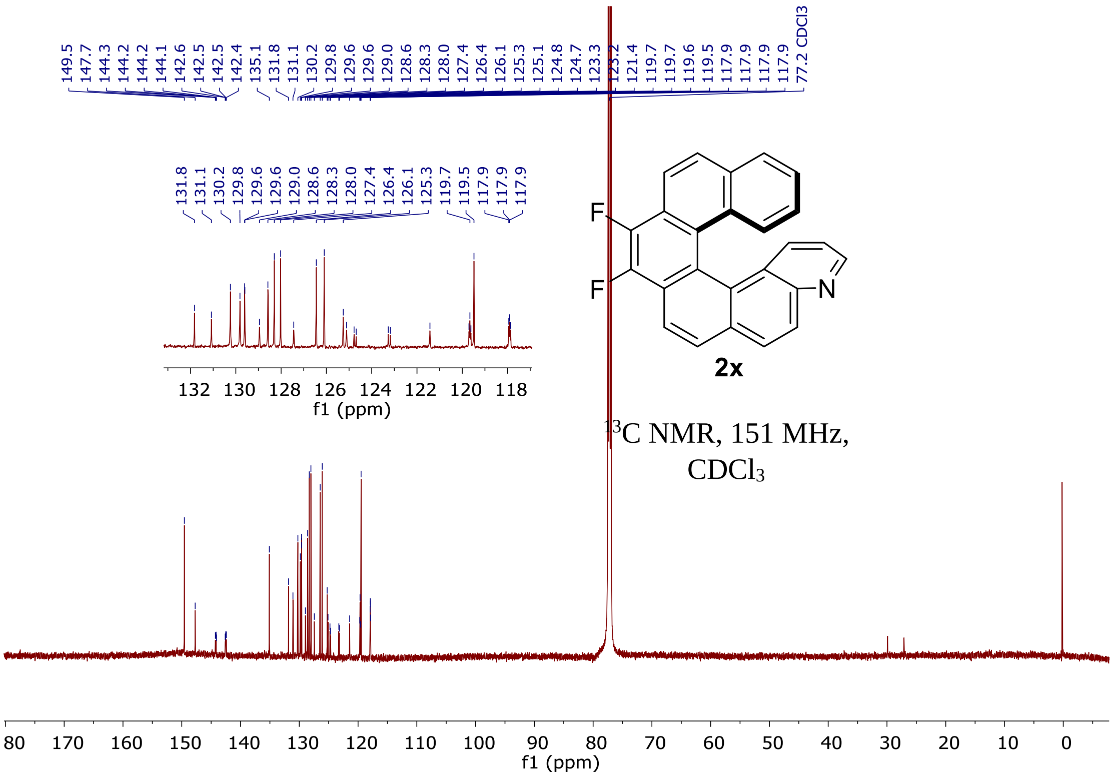
149.5 147.7 144.3 144.2 144.2 144.1 142.6 142.5 142.5 142.4 135.1 131.8 131.1 130.2 129.8 129.6 129.6 129.0 128.6 128.3 128.0 127.4 126.4 126.1 125.3 125.1 124.8 124.7 123.3 123.2 121.4 119.7 119.7 119.6 119.5 117.9 117.9 117.9 117.9
77.2 CDCl3
131.8 131.1 130.2 129.8 129.6 129.6 129.0 128.6 128.3 128.0 127.4 126.4 126.1 125.3 119.7 119.5 117.9 117.9 117.9
f1 (ppm)
F
F
N
2x
13C NMR, 151 MHz, CDCl3
f1 (ppm)

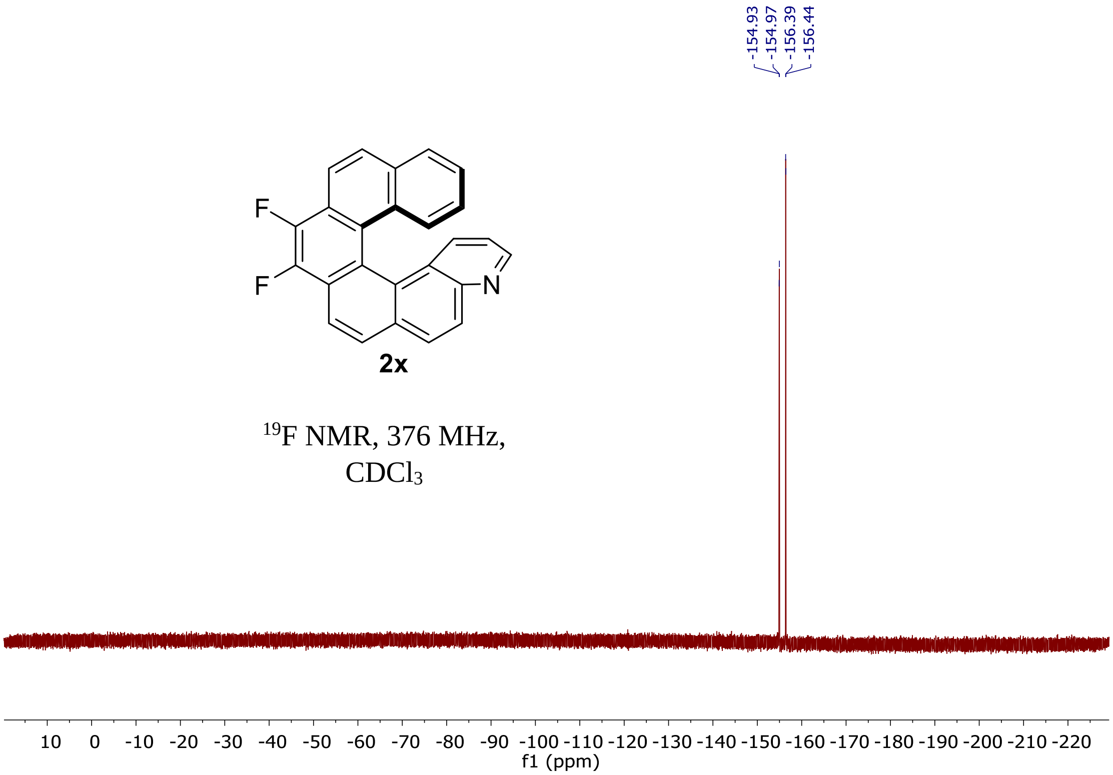

2x

^{19}F NMR, 376 MHz, $CDCl_3$

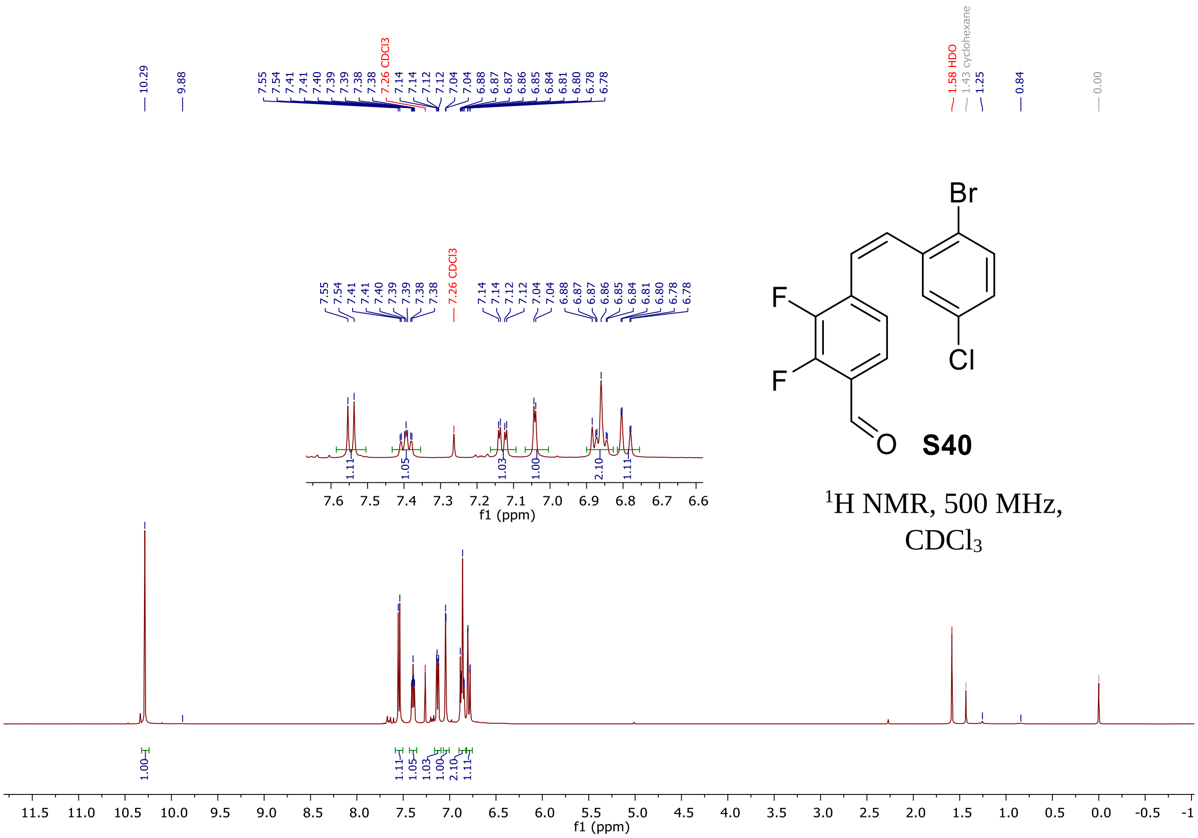

S40

^{1}H NMR, 500 MHz, $CDCl_3$

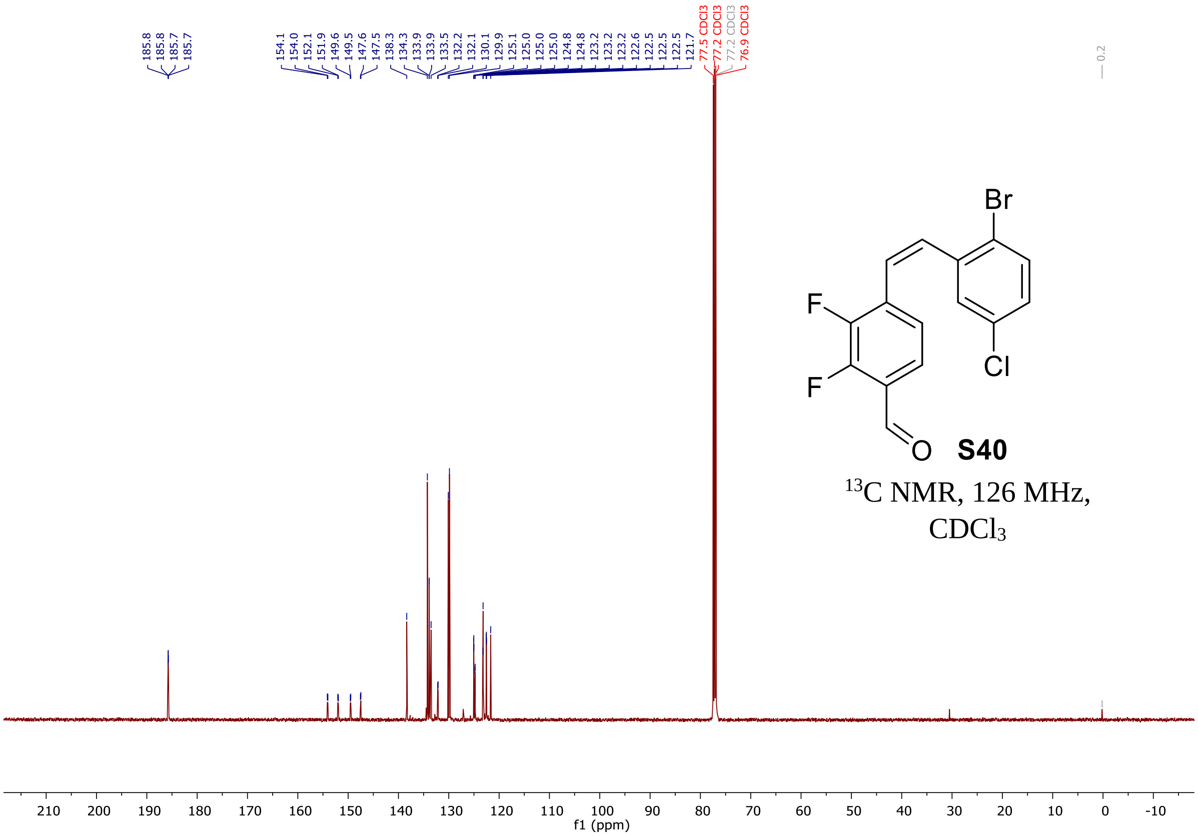

S40

^{13}C NMR, 126 MHz, $CDCl_3$

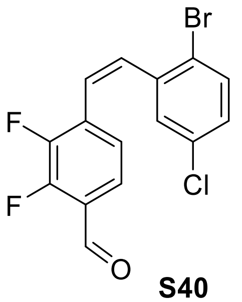

S40

^{19}F NMR, 376 MHz, $CDCl_3$

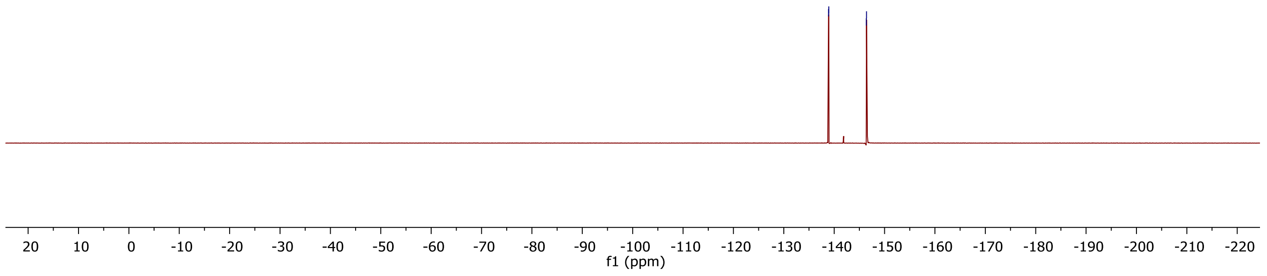

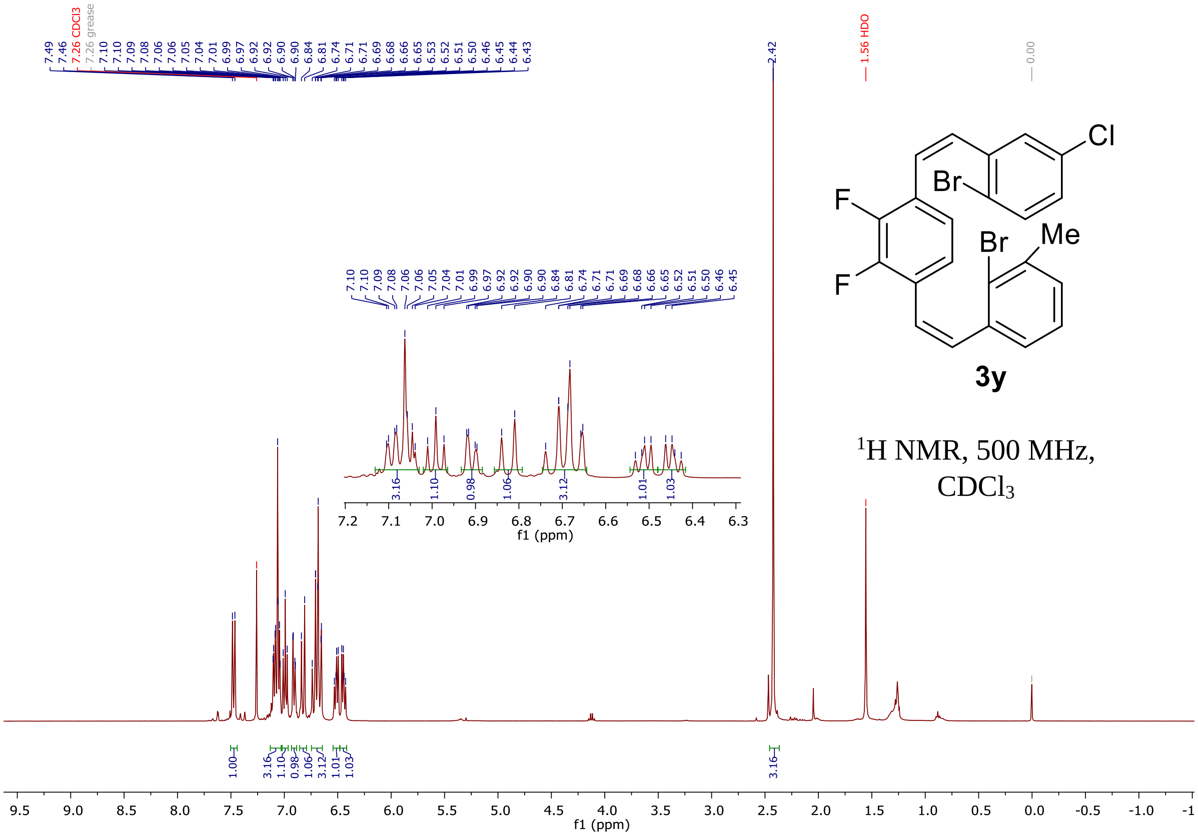
3y
^{1}H NMR, 500 MHz, $CDCl_3$
f1 (ppm)

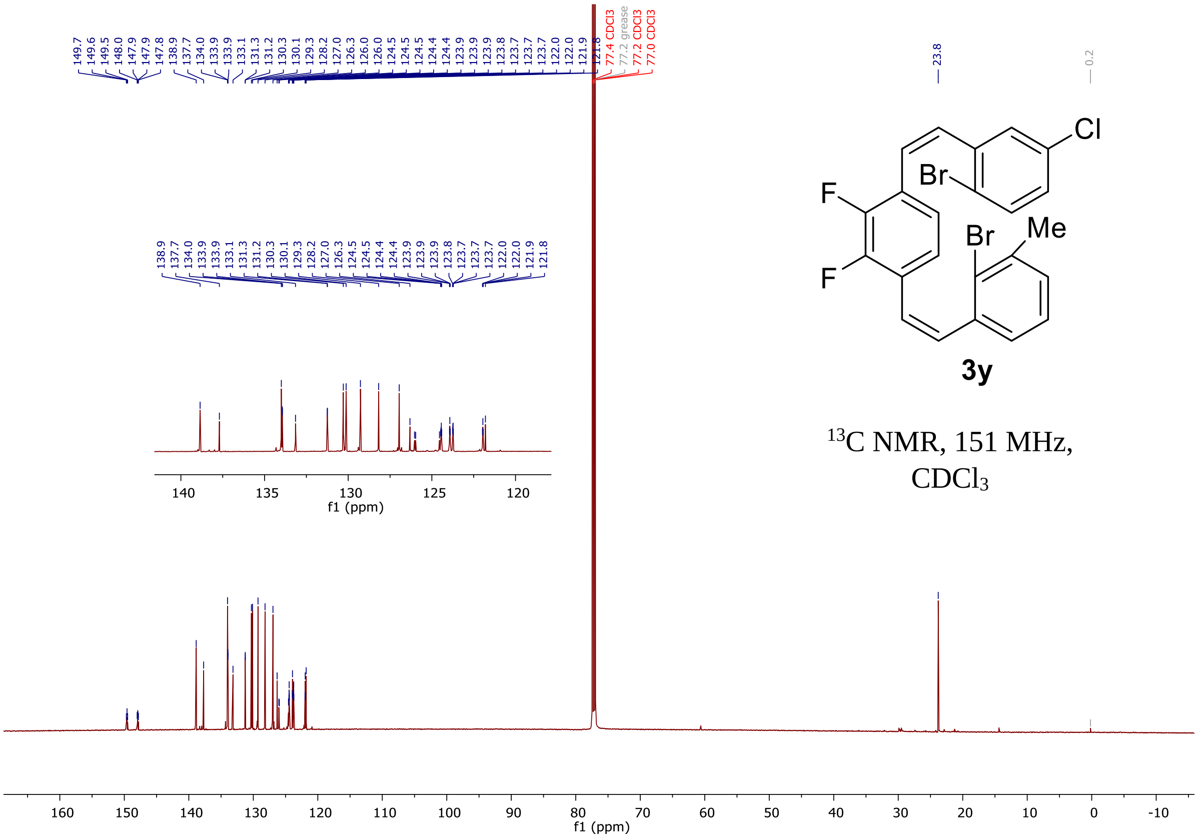
3y
^{13}C NMR, 151 MHz, $CDCl_3$
f1 (ppm)

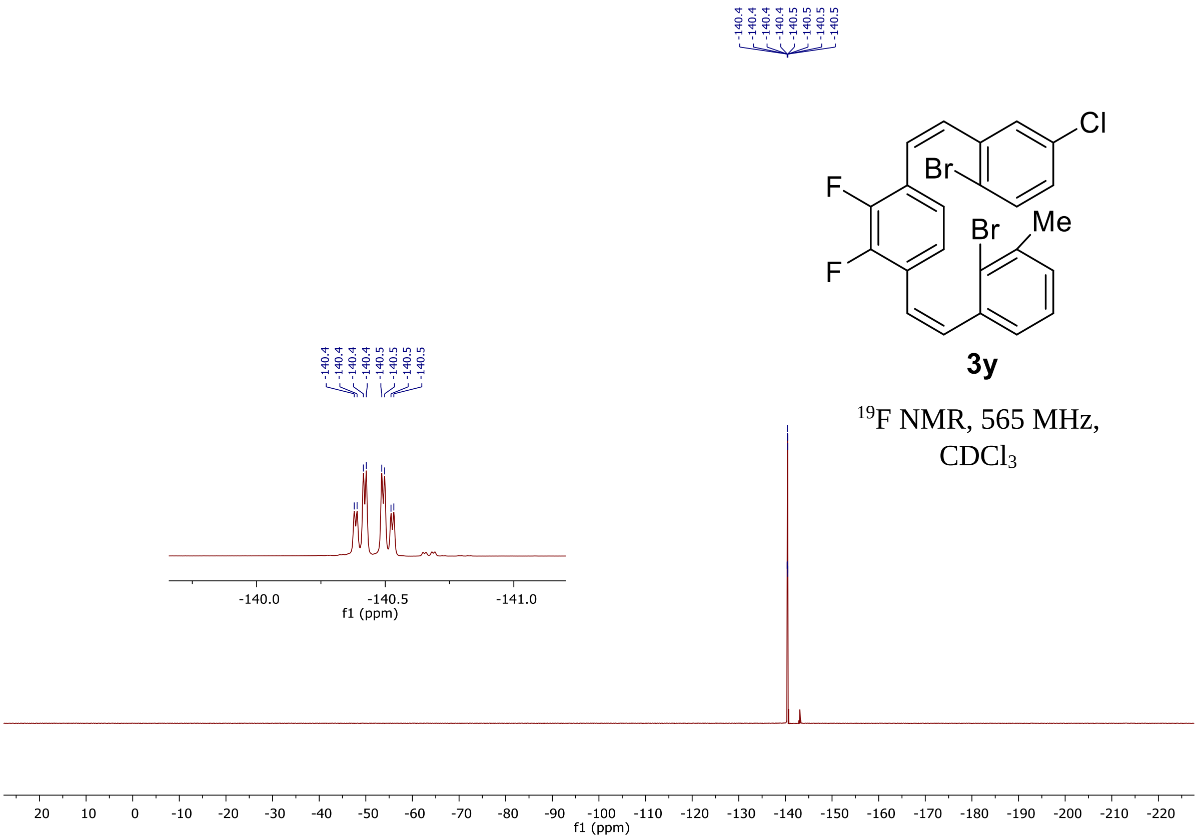

-140.4
-140.4
-140.4
-140.4
-140.5
-140.5
-140.5
-140.5
Cl
Br
F
F
Br
Me
3y
^{19}F NMR, 565 MHz, $CDCl_3$
-140.0
-140.5
-141.0
f1 (ppm)
20 10 0 -10 -20 -30 -40 -50 -60 -70 -80 -90 -100 -110 -120 -130 -140 -150 -160 -170 -180 -190 -200 -210 -220
f1 (ppm)

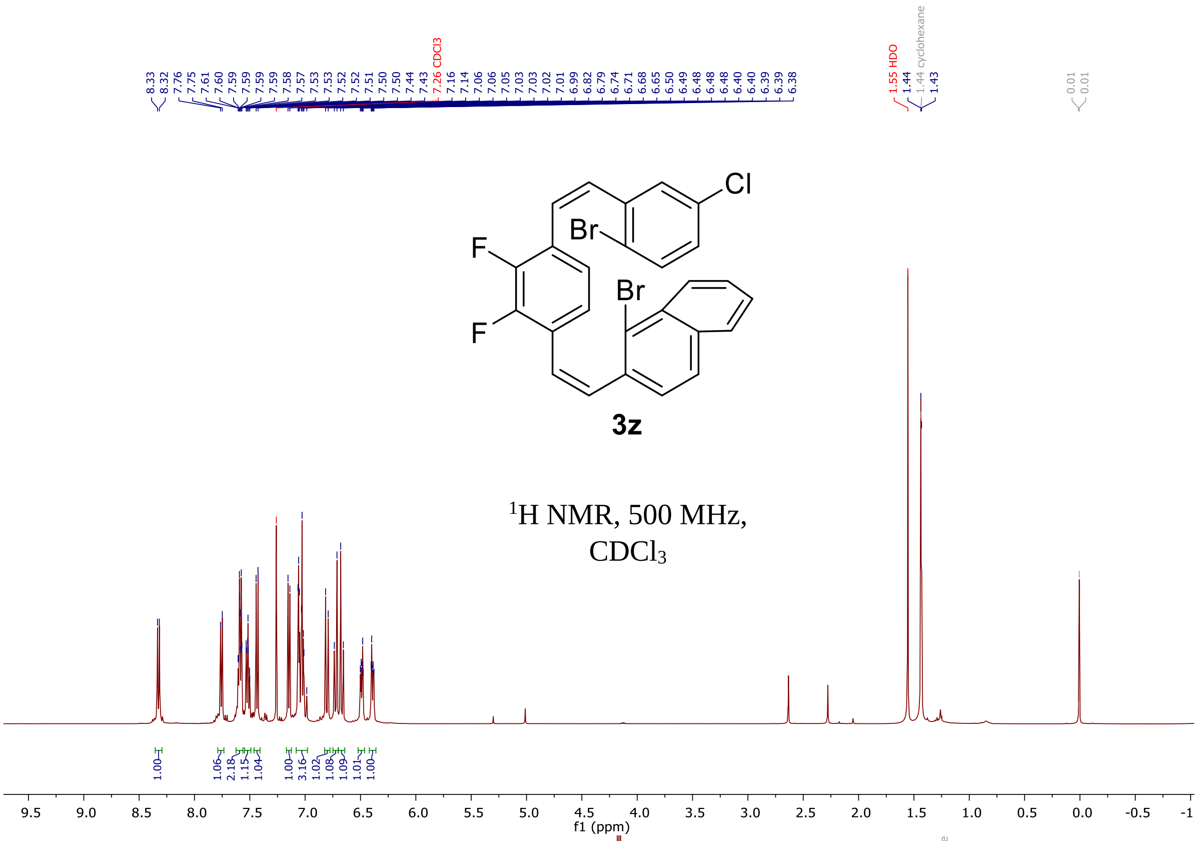

7.26 CDCl3
1.55 HDO
1.44 cyclohexane
Cl
Br
F
F
Br
3z
1H NMR, 500 MHz,
CDCl3
f1 (ppm)

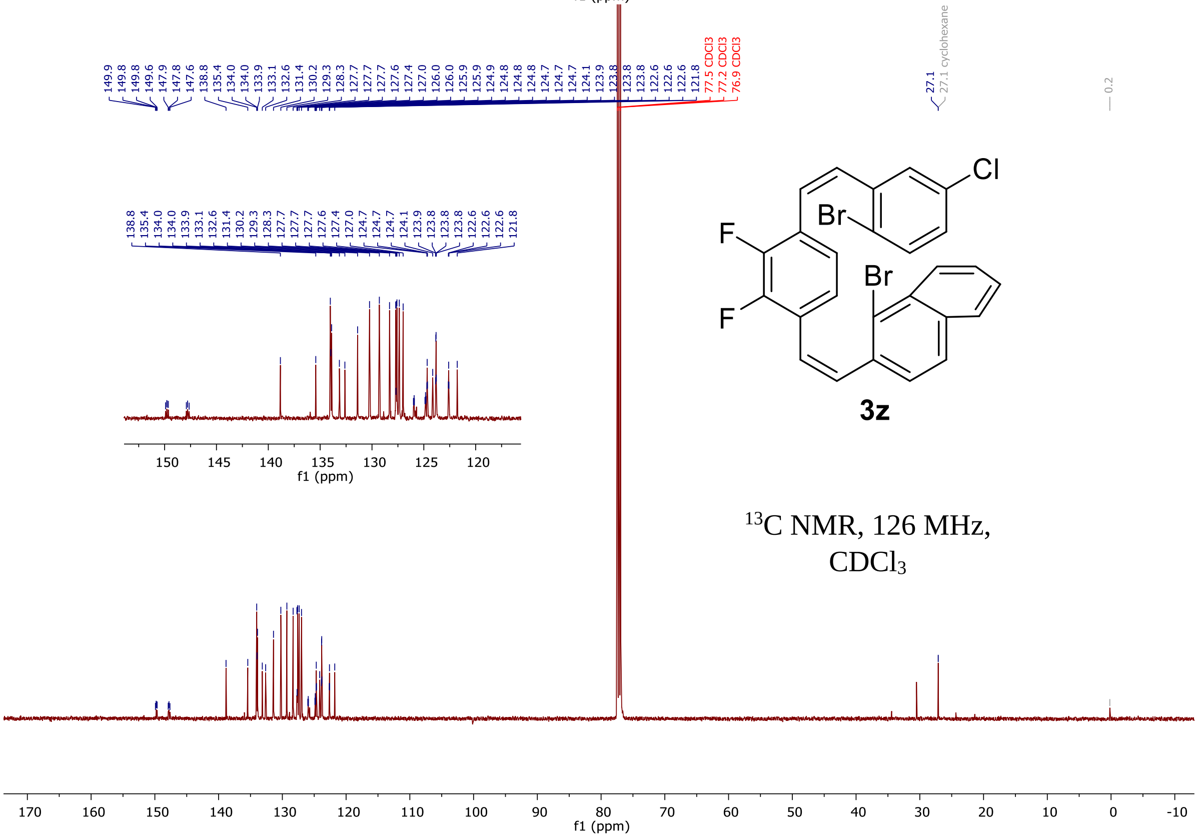

77.5 CDCl3
77.2 CDCl3
76.9 CDCl3
27.1 cyclohexane
Cl
Br
F
F
Br
3z
13C NMR, 126 MHz,
CDCl3
f1 (ppm)

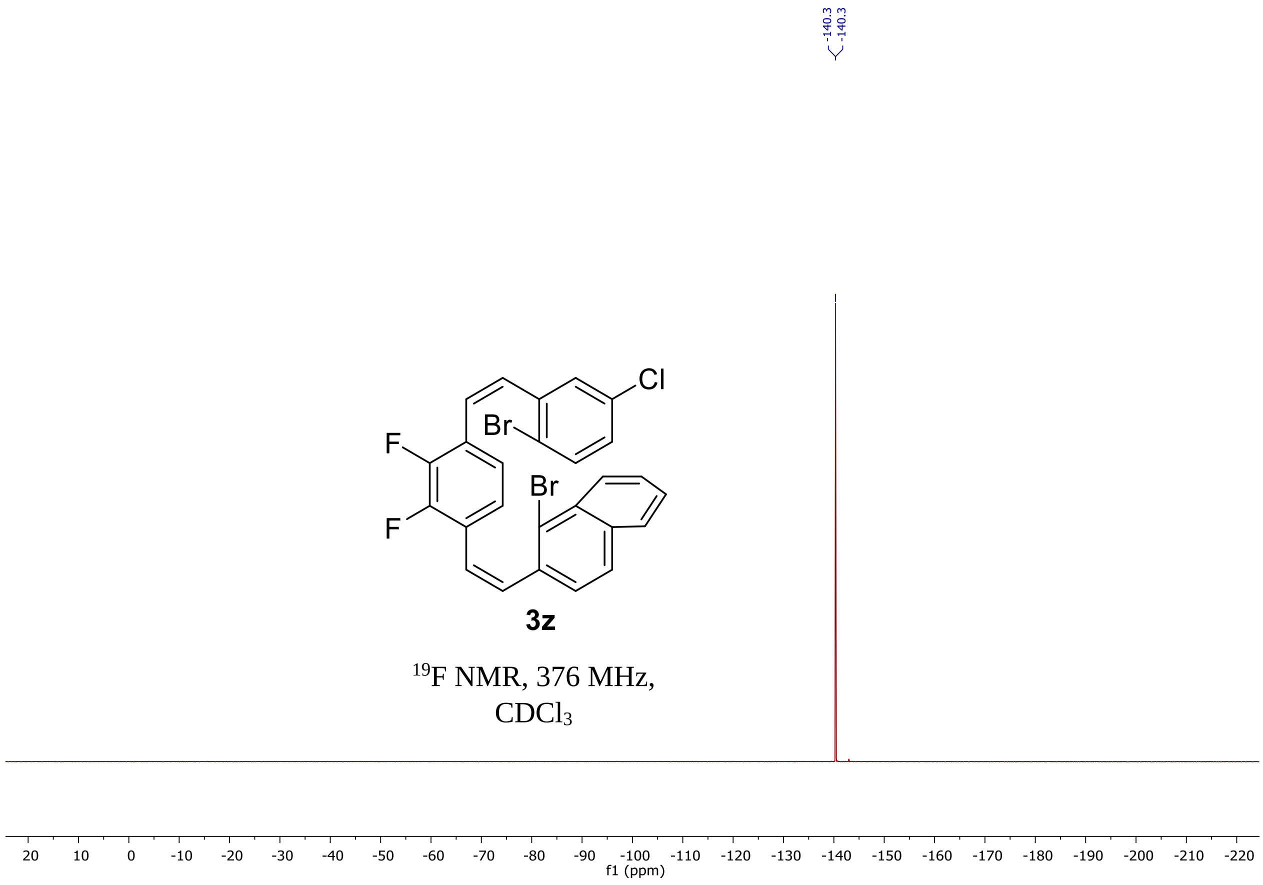
-140.3
-140.3
Cl
Br
F
F
Br
3z
19F NMR, 376 MHz,
CDCl3
20 10 0 -10 -20 -30 -40 -50 -60 -70 -80 -90 -100 -110 -120 -130 -140 -150 -160 -170 -180 -190 -200 -210 -220
f1 (ppm)

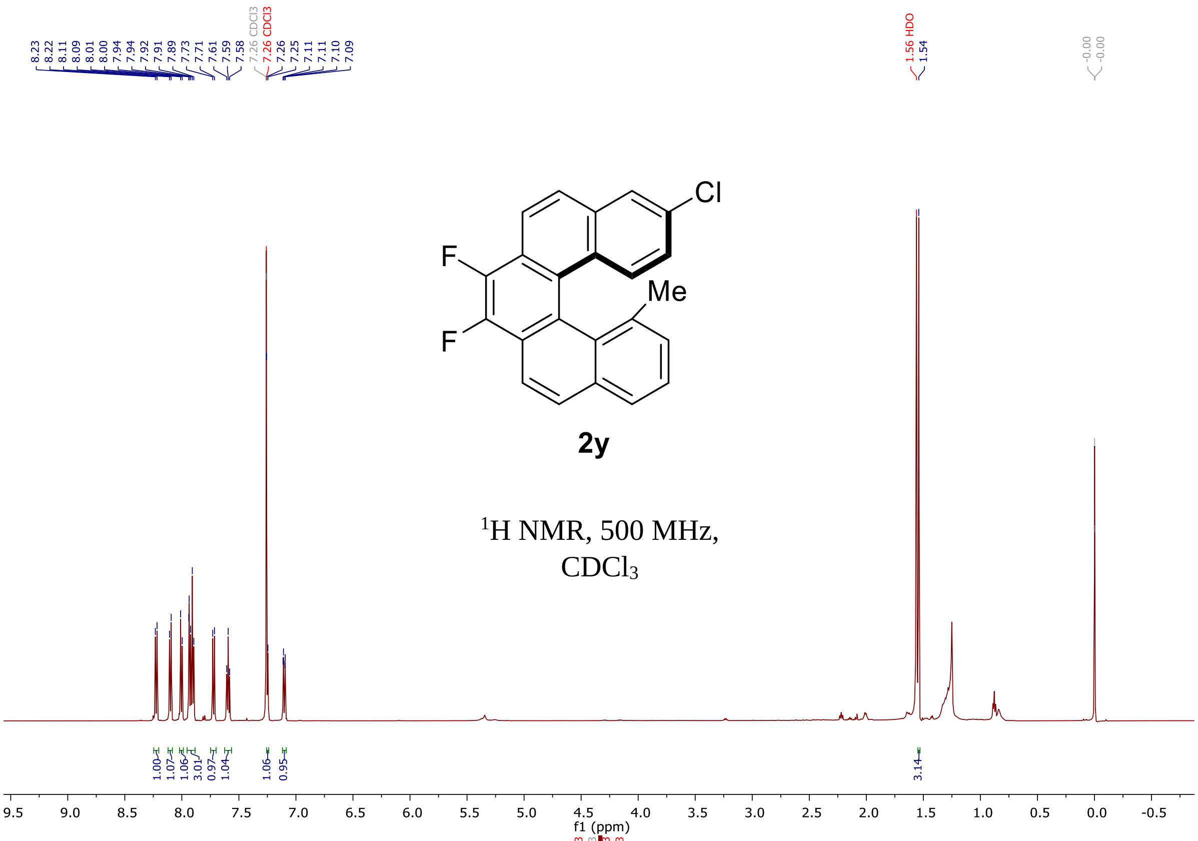
8.23
8.22
8.11
8.09
8.01
8.00
7.94
7.94
7.92
7.91
7.89
7.73
7.71
7.61
7.59
7.58
7.26 CDCl3
7.26 CDCl3
7.26
7.25
7.11
7.11
7.10
7.09
1.56 HDO
1.54
-0.00
-0.00
Cl
F
F
Me
2y
^{1}H NMR, 500 MHz,
$CDCl_3$
1.00
1.07
1.06
3.01
0.97
1.04
1.06
0.95
3.14
f1 (ppm)

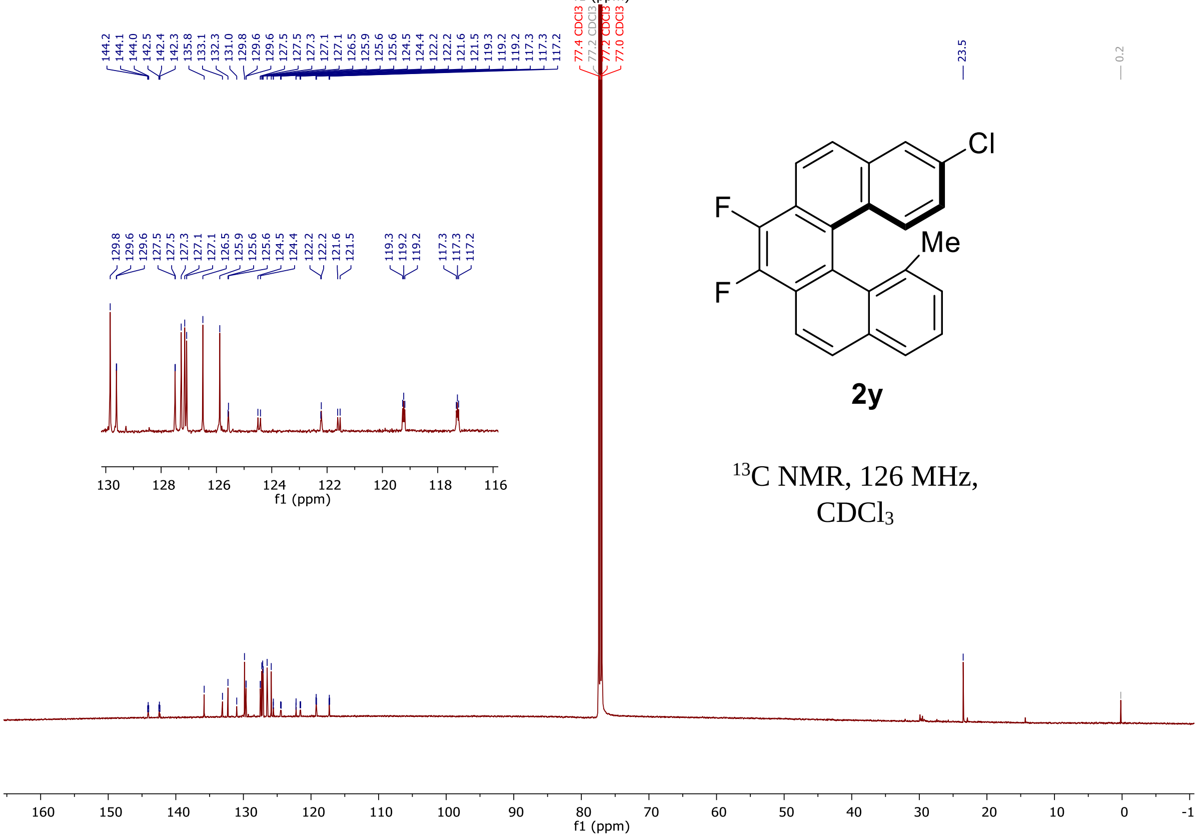
144.2
144.1
144.0
142.5
142.4
142.3
135.8
133.1
132.3
131.0
129.8
129.6
129.6
127.5
127.5
127.3
127.1
127.1
126.5
125.9
125.6
125.6
124.5
124.4
122.2
122.2
121.6
121.5
119.3
119.2
119.2
117.3
117.3
117.2
77.4 CDCl3
77.2 CDCl3
77.2 CDCl3
77.0 CDCl3
23.5
0.2
Cl
F
F
Me
2y
^{13}C NMR, 126 MHz,
$CDCl_3$
f1 (ppm)

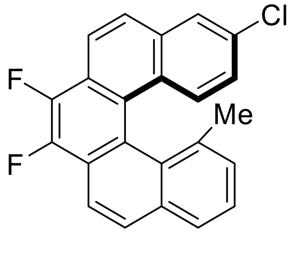

2y

^{19}F NMR, 376 MHz,
$CDCl_3$

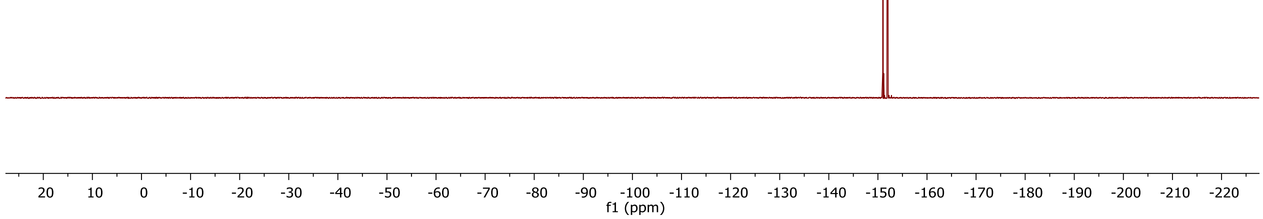

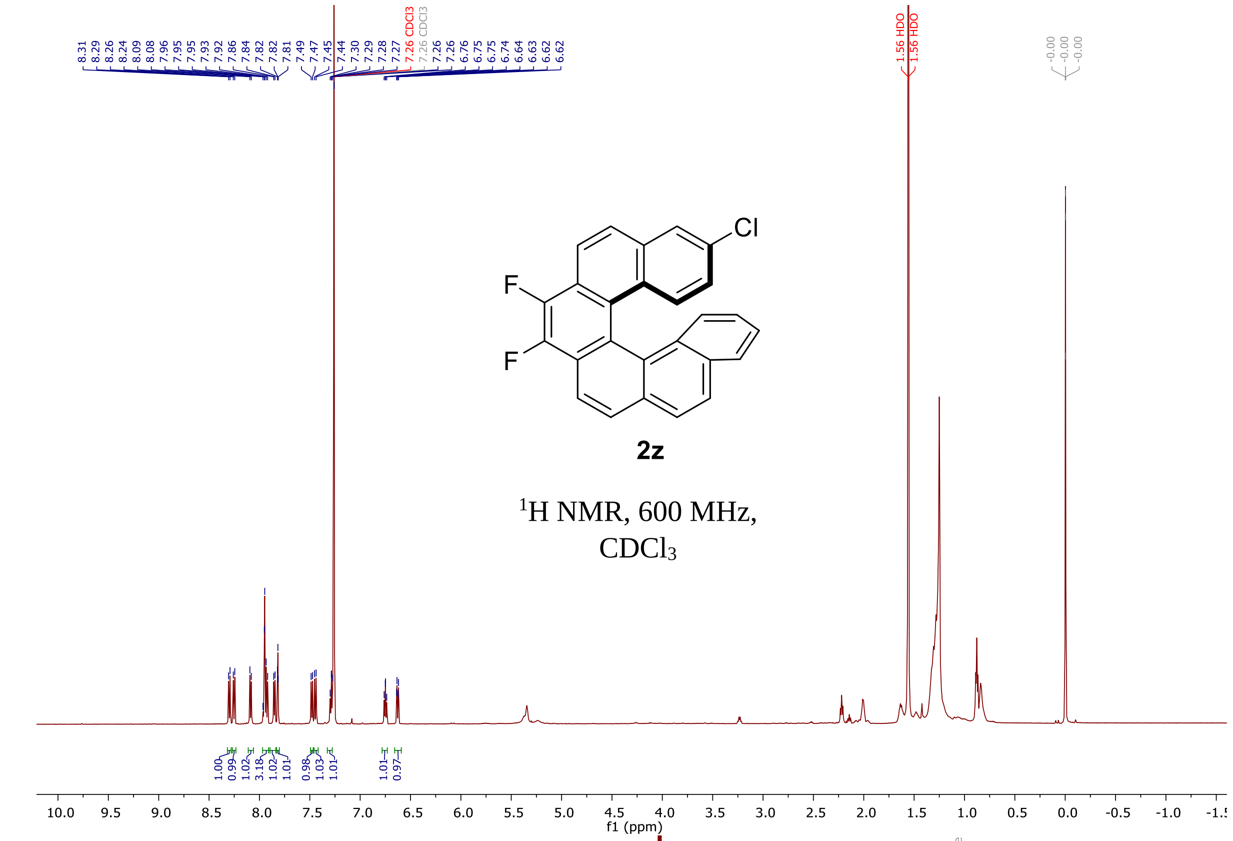

2z

^{1}H NMR, 600 MHz, $CDCl_3$

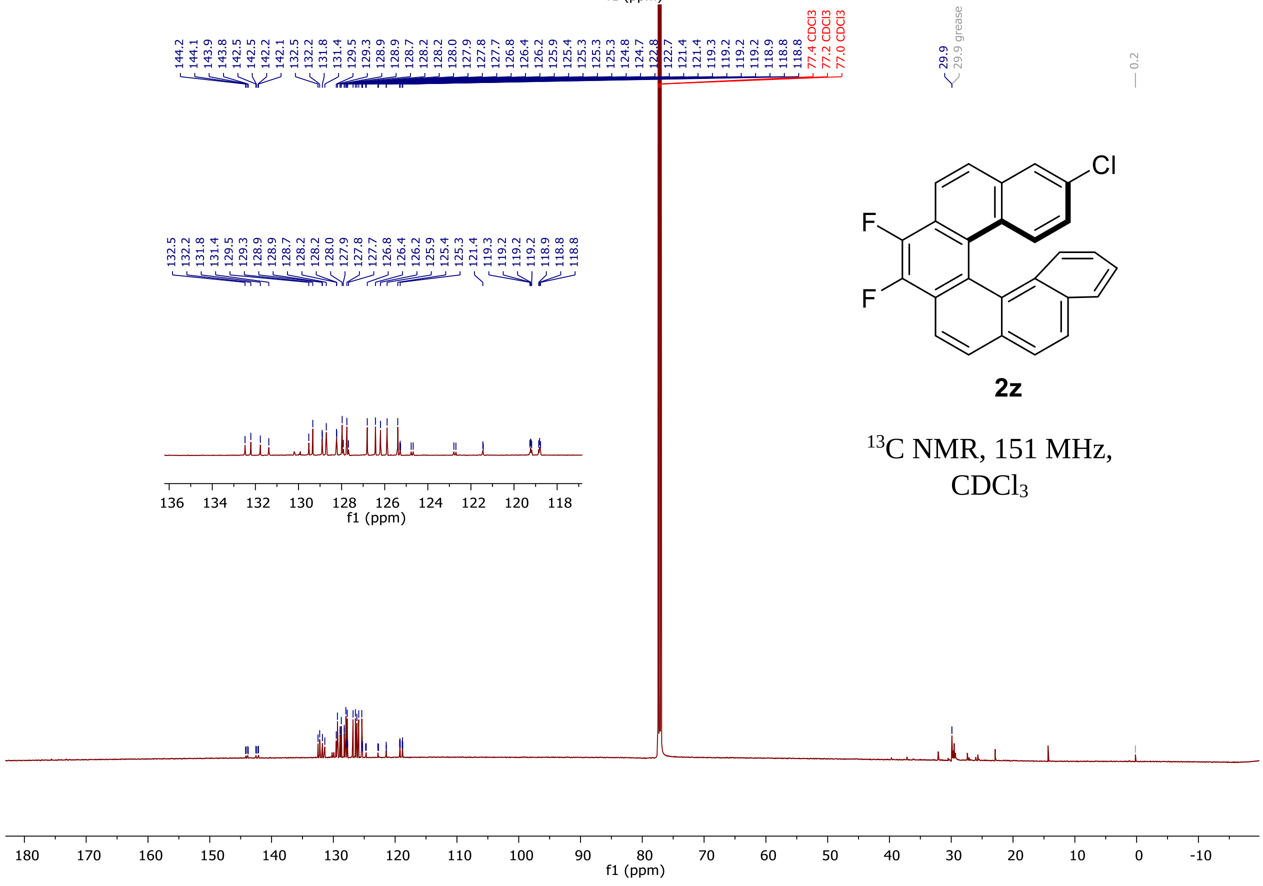

2z

^{13}C NMR, 151 MHz, $CDCl_3$

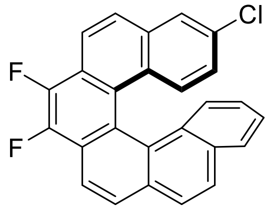

2z

^{19}F NMR, 565 MHz, $CDCl_3$

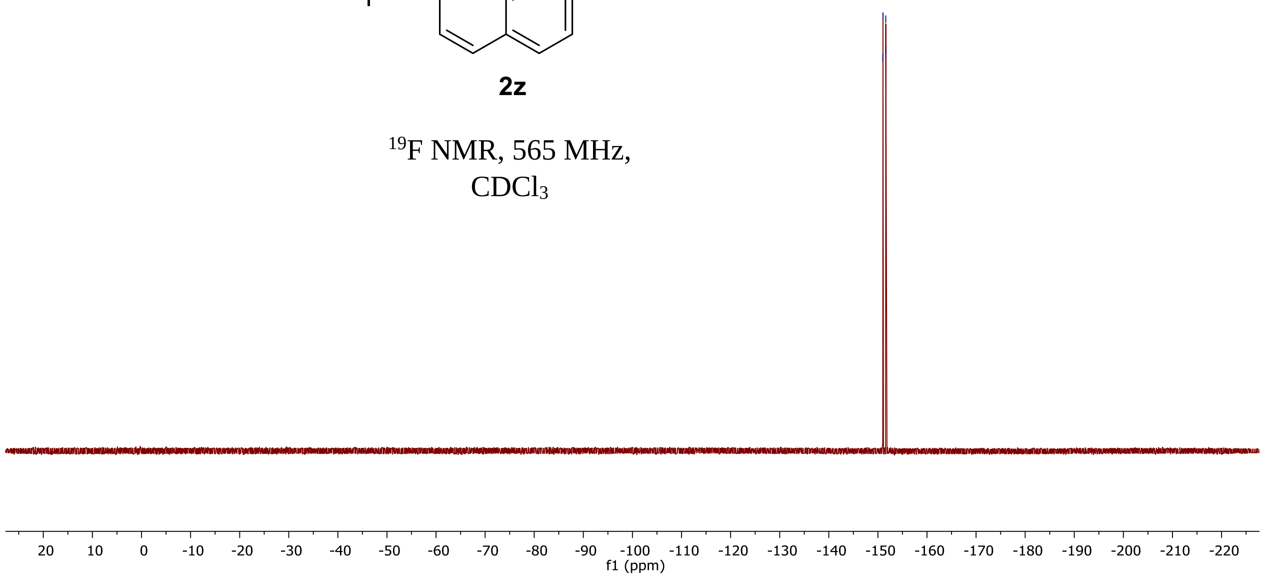

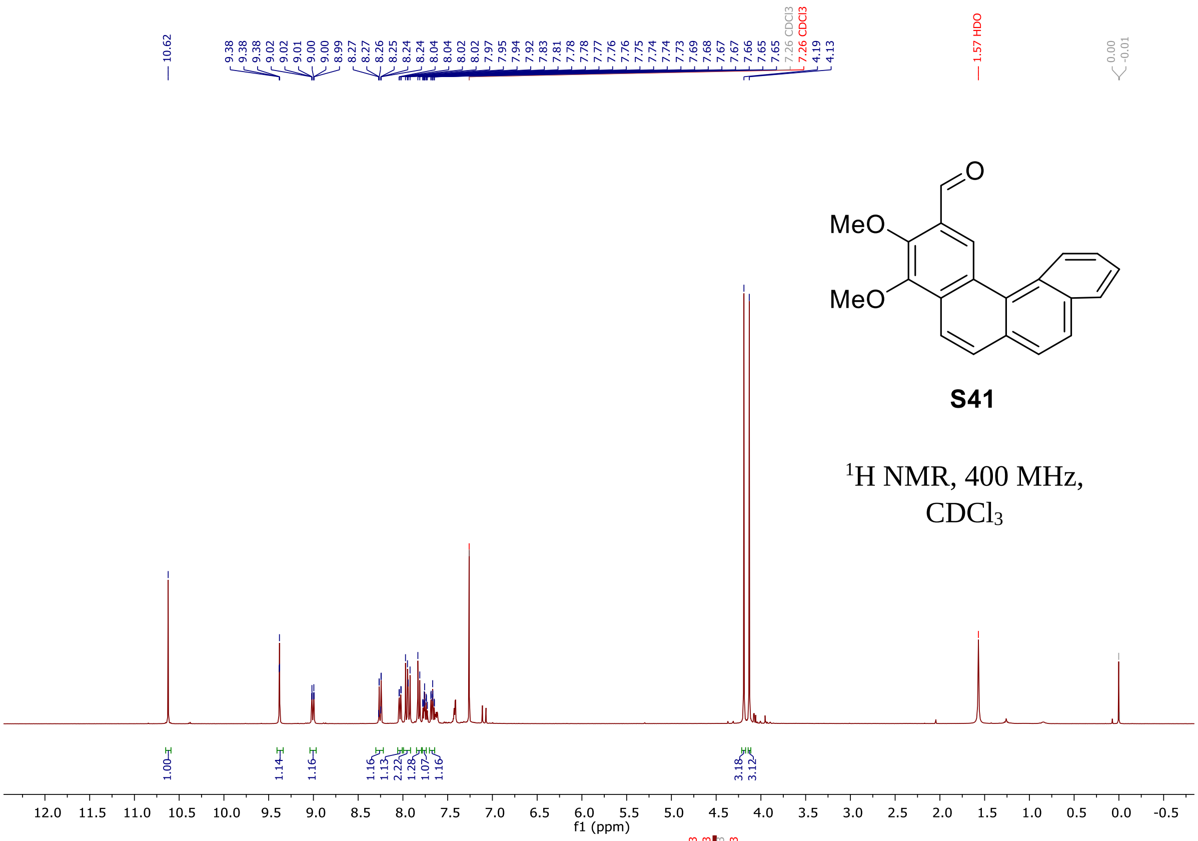

S41

^{1}H NMR, 400 MHz, $CDCl_3$

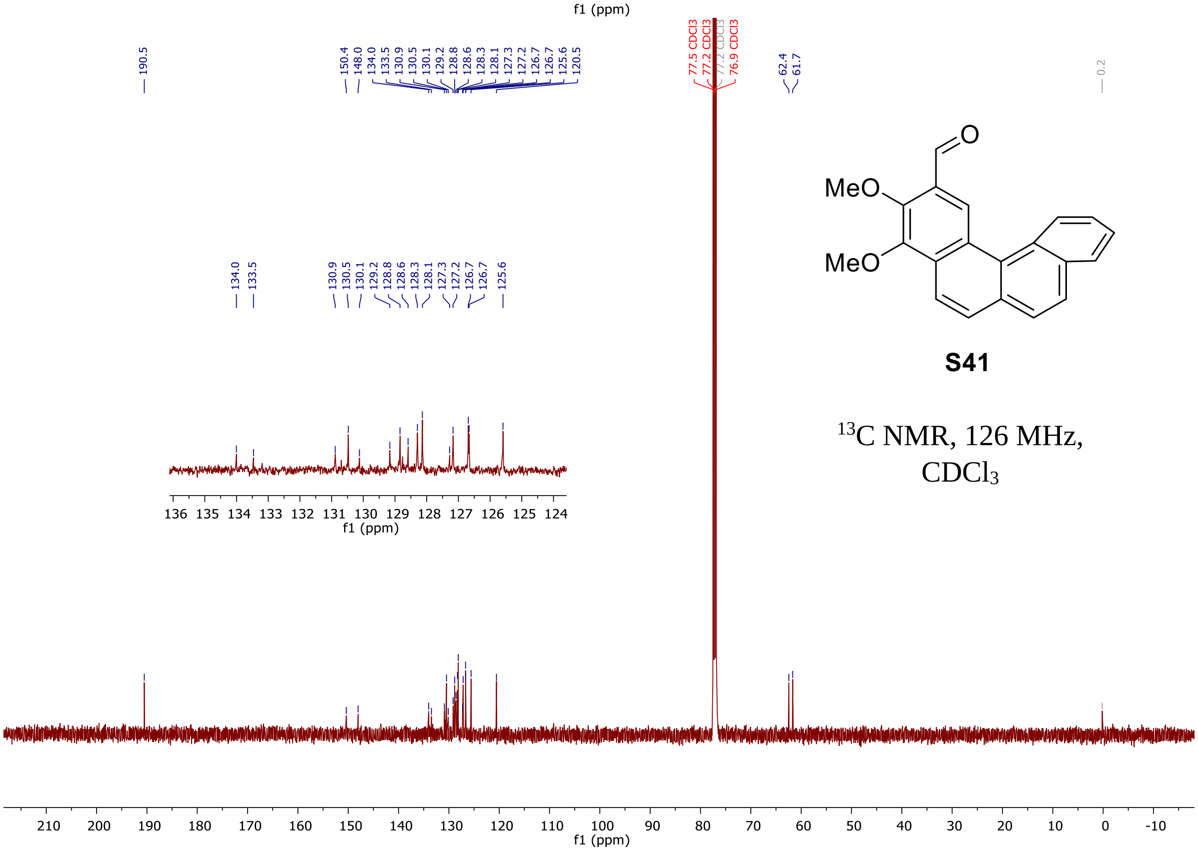

S41

^{13}C NMR, 126 MHz, $CDCl_3$

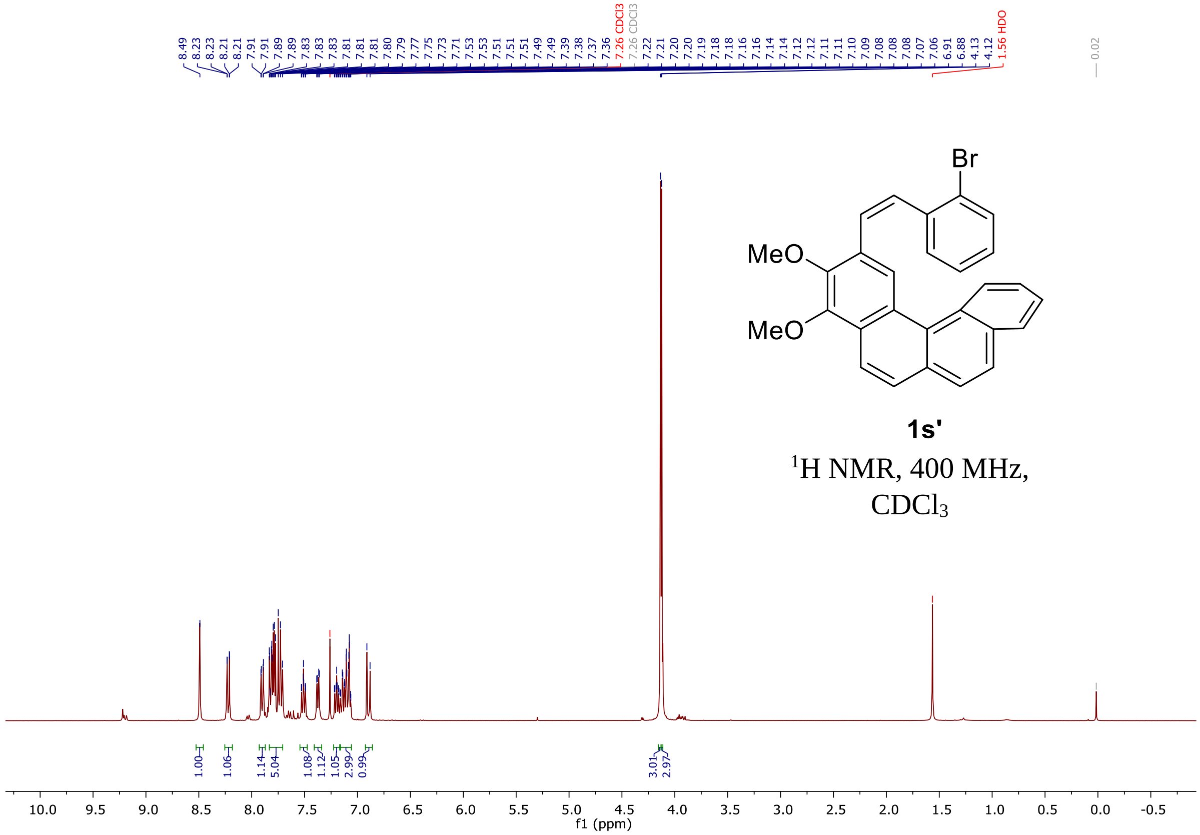

1s'

^{1}H NMR, 400 MHz, $CDCl_3$

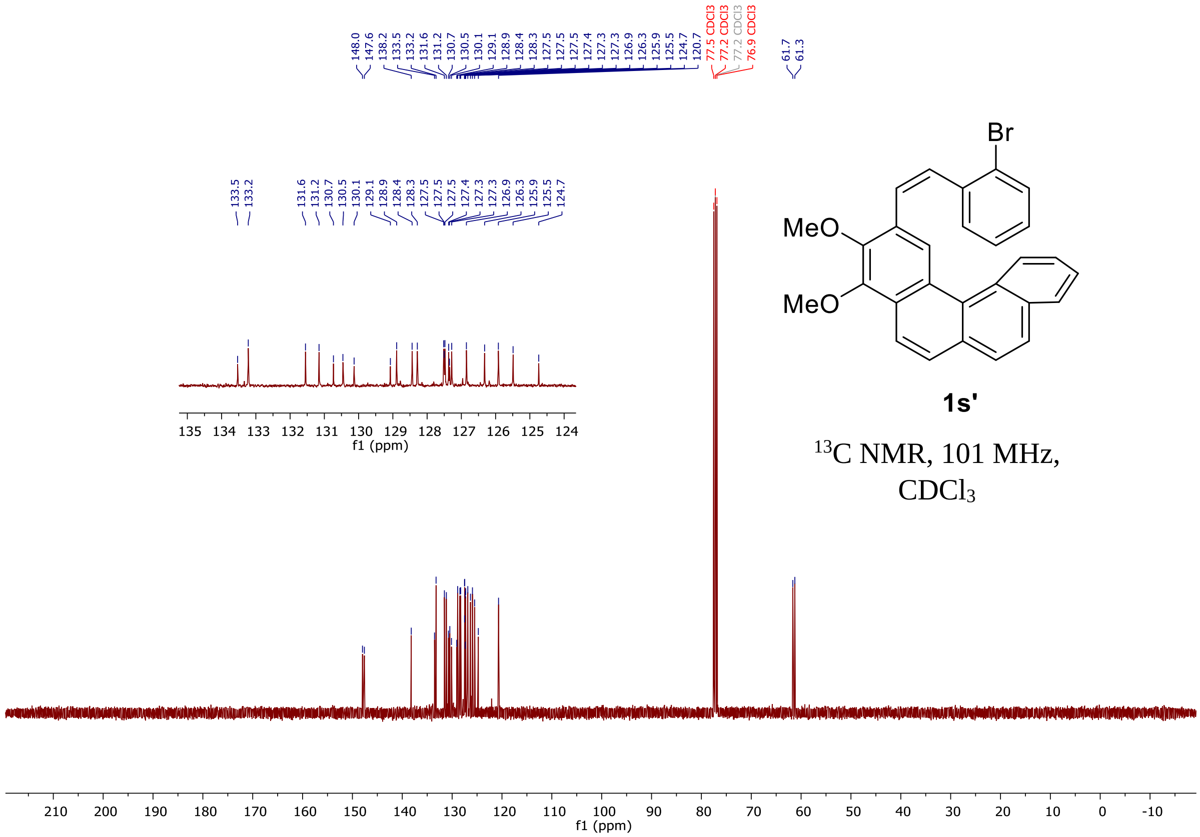

1s'

^{13}C NMR, 101 MHz, $CDCl_3$
